# Transcriptional Reprogramming in Nonhuman Primate (Rhesus Macaque) Tuberculosis Granulomas

**DOI:** 10.1371/journal.pone.0012266

**Published:** 2010-08-31

**Authors:** Smriti Mehra, Bapi Pahar, Noton K. Dutta, Cecily N. Conerly, Kathrine Philippi-Falkenstein, Xavier Alvarez, Deepak Kaushal

**Affiliations:** 1 Divisions of Bacteriology and Parasitology, Tulane National Primate Research Center, Covington, Louisiana, United States of America; 2 DNA Microarray and Expression Core, Tulane National Primate Research Center, Covington, Louisiana, United States of America; 3 Division of Comparative Pathology, Tulane National Primate Research Center, Covington, Louisiana, United States of America; 4 Division of Veterinary Medicine, Tulane National Primate Research Center, Covington, Louisiana, United States of America; 5 Department of Microbiology and Immunology, Tulane University Health Sciences Center, New Orleans, Louisiana, United States of America; Louisiana State University, United States of America

## Abstract

**Background:**

In response to *Mtb* infection, the host remodels the infection foci into a dense mass of cells known as the granuloma. The key objective of the granuloma is to contain the spread of *Mtb* into uninfected regions of the lung. However, it appears that *Mtb* has evolved mechanisms to resist killing in the granuloma. Profiling granuloma transcriptome will identify key immune signaling pathways active during TB infection. Such studies are not possible in human granulomas, due to various confounding factors. Nonhuman Primates (NHPs) infected with *Mtb* accurately reflect human TB in clinical and pathological contexts.

**Methodology/Principal Findings:**

We studied transcriptomics of granuloma lesions in the lungs of NHPs exhibiting active TB, during early and late stages of infection. Early TB lesions were characterized by a highly pro-inflammatory environment, expressing high levels of immune signaling pathways involving IFNγ, TNFα, JAK, STAT and C-C/C-X-C chemokines. Late TB lesions, while morphologically similar to the early ones, exhibited an overwhelming silencing of the inflammatory response. Reprogramming of the granuloma transcriptome was highly significant. The expression of ∼ two-thirds of all genes induced in early lesions was later repressed.

**Conclusions/Significance:**

The transcriptional characteristics of TB granulomas undergo drastic changes during the course of infection. The overwhelming reprogramming of the initial pro-inflammatory surge in late lesions may be a host strategy to limit immunopathology. We propose that these host profiles can predict changes in bacterial replication and physiology, perhaps serving as markers for latency and reactivation.

## Introduction

TB is a major public health problem of mankind, responsible for the death of over 1.7 million people every year [1]. A third of the world population is infected with *Mtb*, more than 8 million of whom develop and ∼1.8 million die of TB, each year [1]. This situation is exacerbated by the emergence of drug-resistant TB [2], co-infection with AIDS [3] and the failure of Bacille Calmette-Guerin vaccine [4]. A better understanding of the pathogenesis of TB is necessary in order to develop effective vaccines. This in turn requires the availability of robust animal models that accurately mimic infection [5].

Mouse [6], rabbit [7], guinea pig [8] and NHP models [9]–[15] have all contributed to our understanding of TB. Mice are inexpensive and have been extensively used to study TB from both the hosts and the pathogens perspective [6], [16]. However, mice do not sufficiently model critical aspects of human TB, such as latency and the spectrum of pathological lesions [17]. NHPs, such as the rhesus or the cynomolgus macaques most accurately reflect human TB in clinical and pathological contexts [9]–[15].

A hallmark of TB infections is a collection of activated immune cells, called the granuloma. It is believed that granulomas prevent the spread of *Mtb* to other pulmonary and extra-pulmonary sites [18]. A majority of TB granulomas in human TB exhibit central necrosis, with a peripheral rim of immune cells. As the disease is contained, the central necrosis in many lesions is mineralized. Changes in immune status of the host, e.g. AIDS cause granuloma failure and reactivation, during which the necrotic areas caseate [18]. Experimentally infected NHPs can be used to model and study these lesions. Characterization of TB granulomas could delineate the cascades of immune signaling patterns and immune cell recruitment. These studies are impossible to perform in human lesions due to a number of confounding factors. It is impossible to verify the dose and infectivity of *Mtb* strain, the time elapsed since infection and whether the lesions correspond to primary or post-primary infection, or re-infection(s). Differences in geographical location, genetics and the immune status of the host are also difficult to control. Therefore, NHPs infected with a defined dose and strain of *Mtb*, are ideal for the study of TB granulomas. In this report, we compare the transcriptome profiles of week 4 (early) vs. week 13 (late/mature) TB lung granulomas of rhesus macaques.

## Results

### Enumeration of lymphocytes and their activation status in the lungs of *Mtb*-infected NHPs

**Figure 1:**
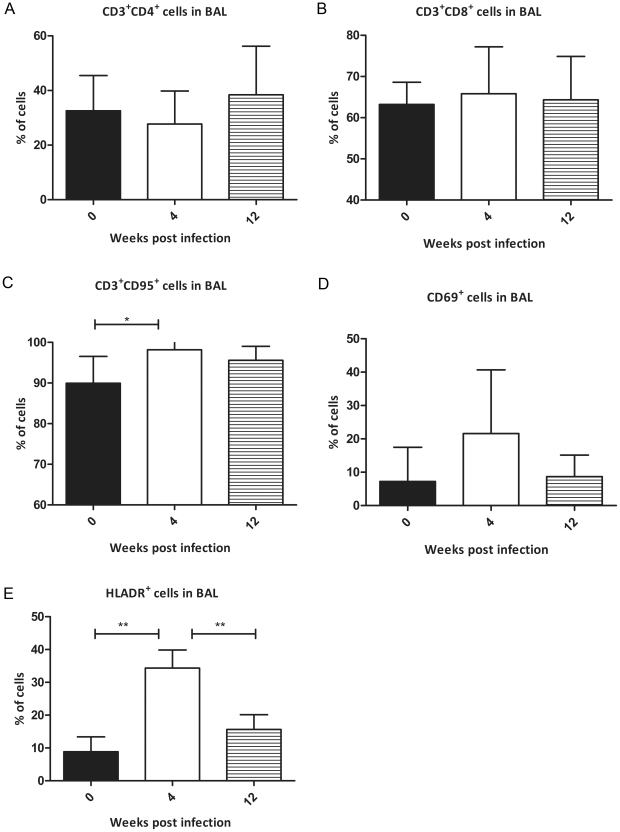
Comparison of lymphocyte recruitment and activation in “early” and “late” TB granulomas. Flow cytometry analysis of BAL samples from *Mtb* infected NHPs prior to infection (black bars), four weeks post-infection (clear bars) and 12 weeks post-infection (striped bars). Comparison is shown for CD4^+^ T cells (A), CD8^+^ T cells (B), CD95^+^ T cells (C), CD69^+^ cells (D) and HLA-DR^+^ cells (E). Results are expressed as percent of total BAL cells.

We studied the temporal recruitment of various lymphocyte subpopulations (the percentage of CD4+ and CD8+ T cells, and the percentage of T cells expressing activation [CD69 or HLA-DR] or apoptosis [CD95] markers) in broncho alveolar lavage (BAL) of the infected NHPs. Samples were derived from three different time points (pre-infection, 4 and 12 week post *Mtb*-infection) from three different NHPs. The levels of CD3^+^ cells remained relatively constant during the course of Mtb infection of NHPs (Fig. 1A & B). While the levels of CD4^+^ T cells decreased slightly at 4 weeks post-infection (Fig. 1A), those of CD8^+^ T cells increased slightly at this time, relative to pre-infection (Fig. 1B). These changes were not significant. The levels of both CD4^+^ and CD8^+^ T cells normalized to the pre-infection levels by 12 weeks post-infection. The relatively constant levels of T lymphocytes during *Mtb* infection of NHPs may represent a dynamic equilibrium between T cells being recruited to the lungs in response to infection and those being turned over due to tissue damage.

Despite the fact that the total number of T cells remained consistent, increased T cell activation was observed following *Mtb* exposure. A statistically significant increase in the number of CD3+CD95+ cells occurred at week 4, relative to pre-infection (Fig. 1C). CD95 is a member of an apoptotic pathway that plays an important role in peripheral T cell tolerance, homeostasis and clonal downsizing of an immune response. It is therefore considered to be a marker for memory T cells. Particularly in light of the fact that the total number of lymphocytes was unchanged, this indicates a transition from naïve to a memory phenotype (Fig. 1C). At week 12, the percentage of lymphocytes expressing CD95 declined slightly relative to week 4. However, this decline was statistically insignificant.

We also observed an increase in the number of CD69^+^ T cells in BAL at the week 4 time point (Fig. 1D). This increase was reversed during the chronic (12 weeks post infection) phase of Mtb infection (Fig. 1D). CD69, an early lymphocyte activation marker, acts as a co-stimulatory molecule for T cell activation and proliferation. CD69 is crucial for cytokine response because its stimulation leads to IL2 production. We also studied the expression of a different activation marker, HLA-DR on T cells in these samples. The fraction of HLA-DR^+^ T cells significantly increased during the active (4 wk) phase, relative to pre-infection. The percentage of these cells declined in the chronic (12 wk) phase, again significantly (Fig. 1E).

NHP TB lesions appear to be morphologically similar at both 4 and 13 weeks post infection. Moreover, the number of T-lymphocytes being recruited to the lungs of *Mtb* infected NHPs, are comparable between weeks 4 and 12. However, the observed qualitative differences in lymphocyte activation at these time points point to functional differences in “early” and “late” primate TB lung lesions. Therefore, we comprehensively studied gene-expression profiles of “early” and “late” lesions using rhesus macaque DNA microarrays.

### Global expression analysis in granuloma lesions at week 4 post-*Mtb* infection

The expression of 3,140 genes was significantly perturbed in early TB lesions relative to normal lung tissue. The expression of 1,584 genes was enhanced (Table S1), while the expression of 1,556 genes was reduced (Table S2) in early lesions. A short list of key immune function genes, whose expression was induced or repressed in early granuloma lesions, are shown in Tables S3 and S4, respectively.

### Genes with a higher level of expression in early TB granulomas

The expression of numerous chemokine ligands and receptors (CXCL1, 2, 3, 6, 9, 10, 11, 12, 13, 16; R4; CCL3, 4, 11, 13, 15, 18, 19, 20, SICA3; R1, R2); numerous cytokines and their cognate receptors (IL1α, 1R, 1RAP, 11, 13R, 4R, 6); the IFNγ network, (IFNγ; inducible proteins - IFI30, IFI35, IFIT1, IFIT2, IFIT3, IFITM1, IFITM3, ISG20; receptors - IFNGR1, IFNGR2, regulators - IRF1, IRF7a, IRF8; and IFN-dependent genes - INDO, STAT1, STAT5A, JAK); and various members of the TNF family (ligands - TNF, SF13A, SF13B, LITAF, LTB1; receptors - RSF1A, RSF4, RSF8; and related genes – AIP1, AIP3, C1QTNF1, TRAF3, TANK) was significantly induced in early lesions (Tables S1, S3).

Genes encoding innate immune (α-defensin, TLR4, TLR8, TICAM1), complement (C1R, C1QBP, C1QA, C1QB, C1S, C2, C3) and apoptotic factors (BAK1, BCL2L13, BCL2L14, BNIP3, CASP1, 3, 4, CARD4, GZMA, GZMB, IER3, IER3IP1, PDCD2, PDCD10), metallothioneins (MT1E, 1F, 1G, 1X, 4); metalloproteases (MMP1, MMP2, MMP7, MMP9, MMP14, MMP25, ADAM1, ADAM8, ADAM17, ADAM19), lymphocyte antigens (LAG3, CD83, LCP1, LCP2, LY6E, LY86, LY96), major histocompatibility complex (HLA-A, -B, -E, -F, -DMA, -DMB, -DPA1, - DQA1, -DRA, -DRB1, -DRB3, -DRB4, -DRB5), ubiquitination proteins (UBD, UBE2C, 2E, 2J, 2L3, 2L6, 3A, USP9X, 10, 18), adhesion molecules (ITLN1, ICAM1, ICAM2, LGALS3, LGALS9, PECAM1, VCAM1); signaling receptors (RARRES3, RARB1, SRA1, VDR1); growth factors/receptors (CSF1R, FGF1, IGF1, IGF2R, IGFBP3, IGFBP4, IGFBP7, PDGFB); transcription factors (CEBPB, CEBPD, CREB3, CREB3L2, CREB5) and G-protein signaling molecules (RGS1, S16, S19) exhibited increased expression in the early granulomas (Table S3).

### Genes with a lower level of expression in early TB granulomas

Genes which showed significantly reduced expression in the early TB granulomata, relative to normal lung tissues, included Th2 chemokines (CCL14, 24, 25), MAP kinases (MKNK2, MARK2, MAPK7, 11, 12, 15, 8IP3, 3K14, 3K6, BP1) calcium binding/signaling proteins (CABP2, CABP4, CACNA2D3, CAMK2A, CAMK2G, CAMKIIN, CALML3) and immune signaling repressors/negative regulators (BCOR, NFKBIL1, NFKBIL2, SOCS1, SOCS3) (Table S4).

### Global expression analysis in NHP TB granuloma lesions, 13 weeks post-*Mtb* infection

The expression of 2,226 genes was significantly perturbed in late (week 13) TB lesions relative to normal lung. Of these, 1259 genes showed an enhanced level of expression (Table S5), while 967 genes showed a reduced level of expression (Table S6) in late lesions. A short list of key immune genes whose expression was induced or repressed in early lesions is shown in Tables S7 and S8, respectively.

### Genes with higher expression in late TB granulomas

The expression of very few chemokines, only one cytokine (IL22RA1) and only one member of the interferon network (GIP3) was significantly induced in these late lesions (Table S7). This was in stark contrast to the early lesions, where the expression of numerous chemokines, cytokines and IFNγ and TNF networks was induced (Table S3). The expression of A2M, BCOR, BCL2L12, CD244, CD36, CD47, cyclins CCNL1, M1, T2, cyclin-dependent kinases CDC2, 3, 9, 10, laminins LAMA3, A5, B2 and B3, and immune regulators ARRB2, SOCS2, SOCS3, TGFBI14 and TGFBR3 was significantly elevated in these late lesions (Table S7).

### Genes with lower expression in late TB granulomas

The expression of a vast majority of pro-inflammatory genes, induced in early TB lesions, was repressed in late TB lesions, relative to normal lung. These included numerous chemokines and receptors (CCL2, 3, 5, 7, 11, 19; CXCL3, 6, 9, 10, 11, 12, 16, receptors CCR1, CXCR4); cytokines and receptors (1A, 4I1, 10RA, 10RB, 15RA, 2RG, 21R, 27RA, 4R); the IFN network (AR2, GR2, IRF1, IRF7, IRF8, ISG20, ISI30, IFI35, IFIT2, INDO, JAK2); and the TNF network (SF13B, SF5, RSF12A, RSF18, RSF1A, RSF4, RSF7, RSF8, AIP2, C1qTNF1, CD40, LITAF, LTA, LTB) (Table S8).

The expression of ubiquitinating (UBD, UBE2C, UBE2E2, UBE2G1, UBE2L6) and apoptotic proteins (AMID, BAK1, BAX, BCL2L14, BCL3, BNIP1, CARD11, GZMA, GZMB, IER3, PDCD2), cathepsins (CTSB, CTSC, CTSD, CTSS), transcriptional regulators (CITED4, CEBPB, CEBPD, STAT1, SPIB); complement components (C1R, C1QA, C1QB, C1S, C2, C4B); cyclins (CCNE1, CCNF, CDKN1A), growth factors/receptors (CSF1R, CSF2RA, CSF3R, ECGF1, FAP, FGF18, GDF15, GRB2, IGFBP2, IGFBP3, IGFBP4, PF4, PTAFR, PGF, SCGF, VGF), MHC antigens (HLA-A, -F, -DMA, -DPA1, -DQA1, -DRA, -DRB1, -DRB3, -DRB4), metalloproteases (MMP1, 2, 3, 9, 19, 25, MT1X), signaling molecules (GPSM3, HCST, LAG3, PRKCB1, RGS1, 16, 19, RARRES3, RIPK2, RIPK3, SLAMF1, TCIRG1, TLR2, VDR), adhesion molecules (ICAM1, 4), phospholipases (PLA1A, PLA2G4C, PLA2G7, PLCG2, PLD1); proteinase inhibitors (SERPINA1, A3, B1, B9, E1, F2, H2), MAP kinases (MAPK1, 2K1, 2K1IP1), RAS family members (RAB20, 31, 32, 5C, 7L1, RASSF4, RHOA, C, H, U, G3BP, RIS1) and immune cell surface markers (CD3E, 6, 14, 37, 53, 68, 86, 180, 274, 300A) was also down regulated in these late lesions (Table S8).

### Transcriptome comparison of the “early” and “late” TB lung lesions

**Figure 2:**
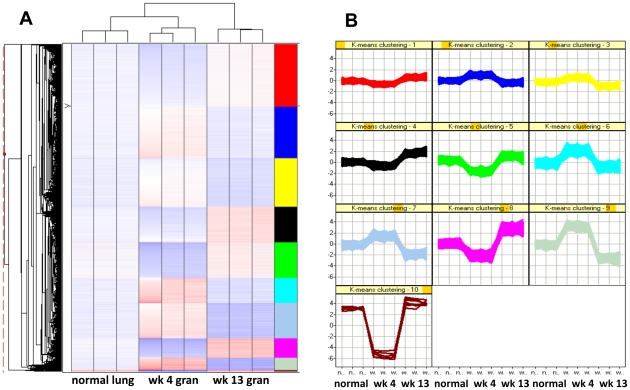
Hierarchical and profile cluster analysis of the transcriptome of early and late granulomas. Microarray analysis of genes expressed in a differential manner between normal lung, and granulomas from *Mtb* infected NHPs at week 4 and week 13, using hierarchical (A) as well as profile based (K-means) clustering (B). Clustering was performed using Spotfire DecisionSite for Functional Genomics, using data from triplicate biological samples. The coloring scheme for the hierarchical cluster is white -no change in expression, blue – lower expression in lesion samples relative to normal lung, and red – higher expression in lesion samples relative to normal lung. The hierarchical cluster is sorted by the results obtained by dividing the dataset into 10 distinct K-means clusters, with the right column showing the identical color scheme as the k-means clusters diagram.

**Figure 3:**
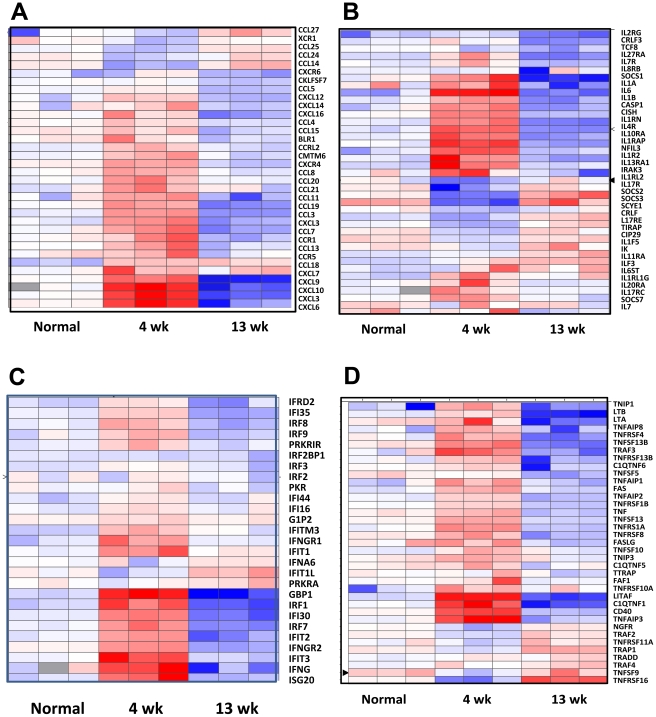
Clustering analysis of specific immune categories. Clustering analysis represents the differences in gene expression in early and late lesions between specific immune categories: chemokines and their receptors (A), cytokines and their receptors (B), IFNγ network (C) and TNF network (D). The coloring scheme is white -no change in expression, blue – lower expression in lesion samples relative to normal lung, and red – higher expression in lesion samples relative to normal lung. Each column represents results obtained from one distinct animal.

The expression of hundreds of genes belonging to key immune signaling pathways induced in early TB granulomas (Table S3) was silenced in late lesions (Table S8). We therefore directly compared the transcriptome of early and late TB granulomas in NHPs. We found that the expression of ∼1200 rhesus genes was altered in late, relative to early lesions, in a statistically significant manner. These differences are represented using a heat-map, sorted on the basis of profile (k-means) clustering (Fig. 2). The host genes contained in these ten distinct clusters are represented in Table S9. Figure 3 represents the differences in gene expression in early and late lesions between different immune specific categories, including chemokines and receptors (Fig. 3A), cytokines and receptors (Fig. 3B), and the IFNγ (Fig. 3C) and TNF networks (Fig. 3D).

While the expression of a majority of chemokine molecules was induced early, the expression of CCL14, 24, 25 and 27 was higher in late rather than early lesions (Fig 3A). Similarly, the expression of cytokines/receptors IL1RL2, 17R, 17RE, 1F5 and 11RA, IFN (PRKRA, IFIT1L, IFNA6) and TNF network (RSF11A, RSF16, SF9, TRAF2 and 4) genes increased in late relative to early lesions. The role played by these molecules needs to be studied in detail. The expression of cytokine inhibitors SOCS2 and SOCS3 was also induced in these late lesions, reflecting their role in controlling pro-inflammatory responses.

**Figure 4:**
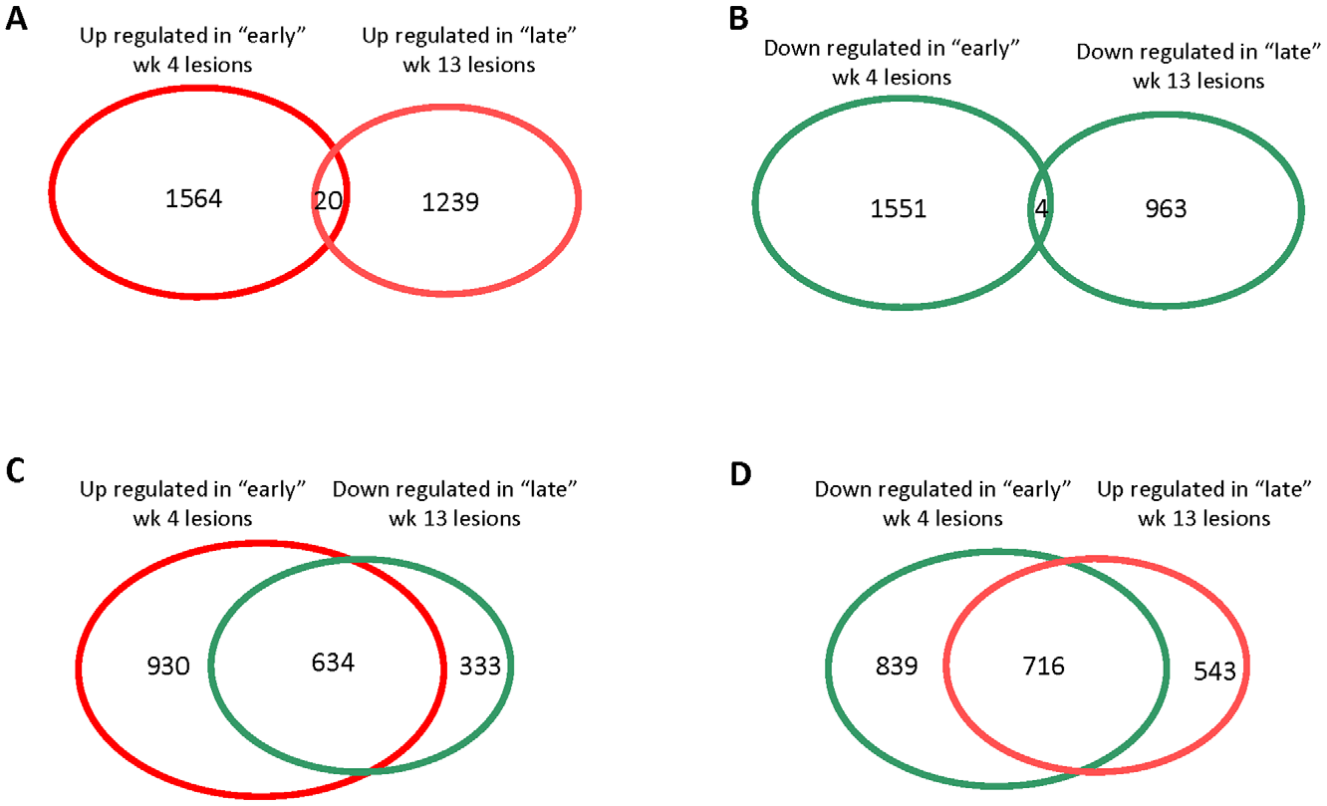
Reprogramming of lesion transcriptome occurs in late, relative to early granulomas. Venn diagrams show the degree of overlap between commonly up regulated (A) and commonly down regulated (B) genes between week 4 and week 13 granuloma lesions. Similarly, overlap is also shown between genes up regulated in week 4 granulomas and down regulated in week 13 granulomas. Genes up regulated in various analyses are represented in red while those down regulated in various analyses are represented in green. The analyses included genes induced or repressed more than 2-fold in a statistically significant manner in all three “early” relative to “late” animals.

Of the genes with significantly differential expression in either early or late lesions, relative to normal lung tissue, only 20 genes were commonly induced in both week 4 and week 13 lesions (Fig 4A, Table S10). There was no significant pair wise association between the up regulated transcriptome of early and late lesions. In fact, the overlap (1.59%) was significantly lower than what could be expected by chance. Similarly, of the potential 968 genes that could overlap between those significantly down regulated in early and late lesions, only four were commonly repressed in both (Fig 4B, Table S11). Again, there was a lack of pair-wise correlation in the down regulated transcriptome results from early and late lesions. The overlap (0.41%) was again significantly lower than what could be expected by chance. On the other hand, a highly significant pair-wise association was observed between the up regulated data set from early lesions and the down regulated data set from the late lesions (Fig. 4C, Table S12). Out of the 968 genes that could potentially overlap between these data sets, 634 genes were common to both (64.46% overlap, χ2 = 7458.18, df = 1, *P*<0.0000001). Similarly, a highly significant pair-wise association was apparent between the set of genes down regulated in week 4 lesions and the set of genes up regulated in the week 13 lesions (Fig. 4D, Table S13). Of the possible 1,259 genes that could overlap these two data sets, the expression of 715 was down regulated in week 4 lesions but up regulated in week 13 lesions (χ2 = 7109.56, df = 1, *P*<0.0000001). These results underline our assertion that a complete reprogramming of gene-expression occurs over a course of 8–10 weeks during the maturation of newly formed TB granulomas.

### Confirmation of transcriptome results from early and late TB lesions

**Figure 5:**
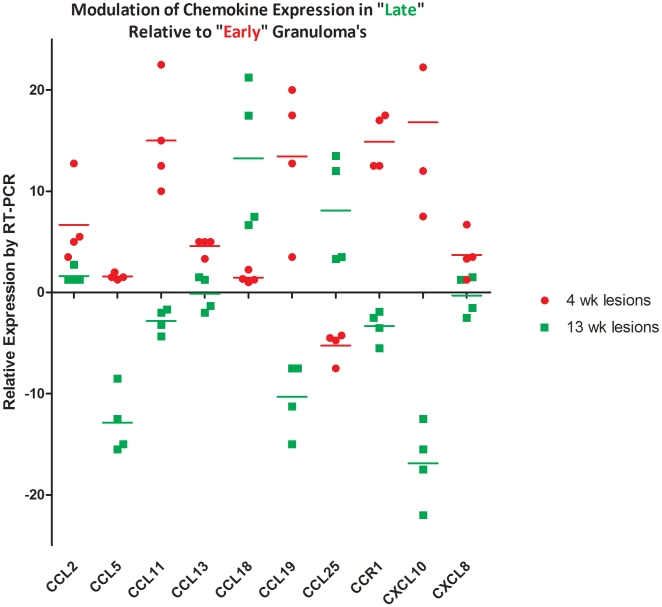
Confirmation of transcriptome results by RT-PCR. Quantitative real-time Reverse-Transcriptase (RT-PCR) arrays specific for chemokines and their receptors were used to confirm microarray results. Biologically independent samples from all three “early” and “late” animals (different from those used in microarray experiment) were pooled into two groups, and duplicate analysis performed using PCR arrays. GAPDH was used as the invariant housekeeping control for normalization. Numeric fold changes reflect the following difference: [Gene x lesion lung (week 4 or week 13) – 18S RNA lesion lung (week 4 or week 13)] – [Gene x normal lung (week 4 or week 13) – 18S RNA lesion lung (week 4 or week 13)]. The expression of CCL25 was checked by conventional SyBr green RT-PCR, using the Applied Biosystems (Foster City, CA) Fast SYBR Green kit since it was not included in the PCR array. Data was analyzed in a manner comparable to PCR-arrays.

**Figure 6:**
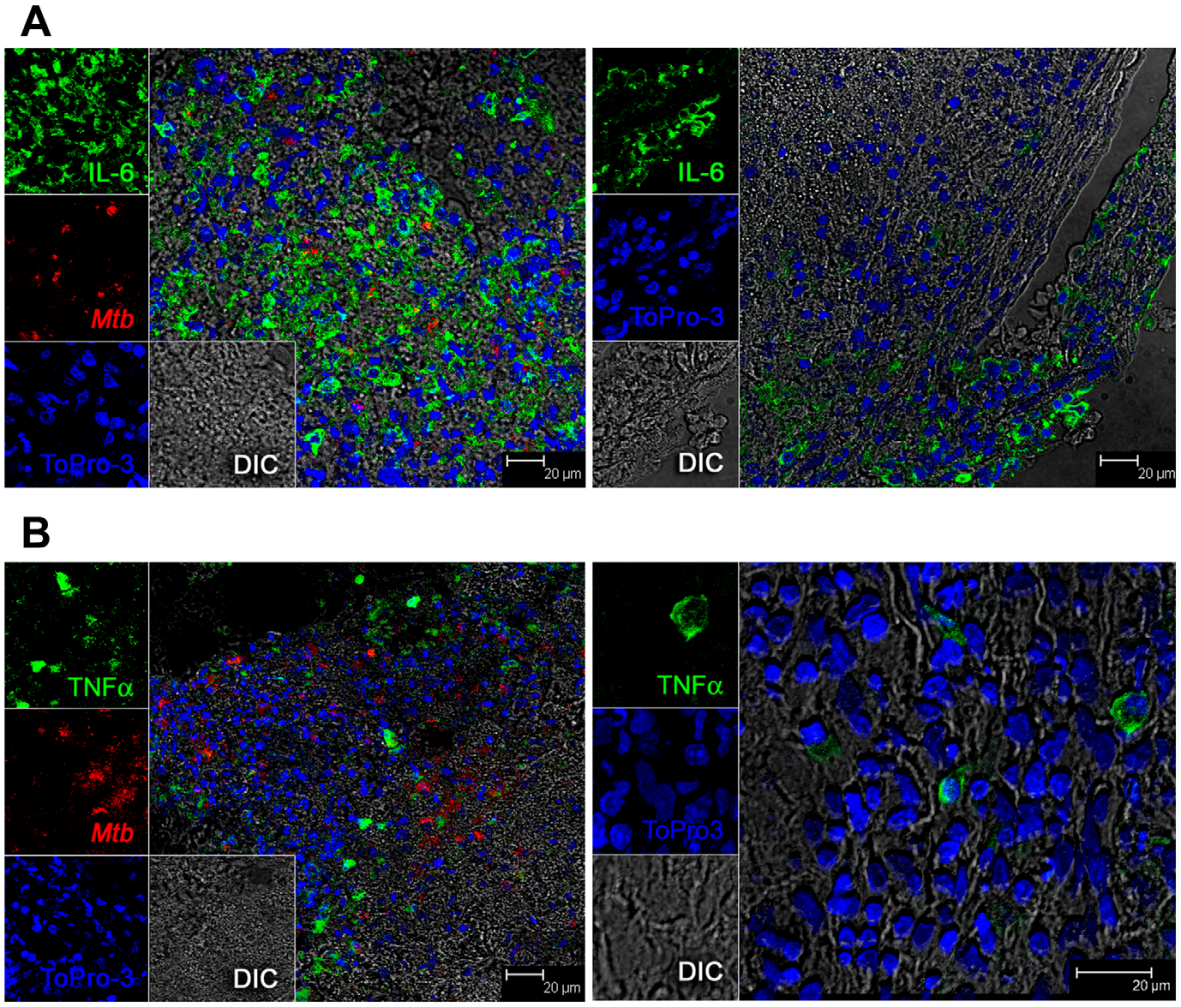
Confirmation of transcriptome results by confocal microscopy. Confocal microscopy shows differential expression of pro-inflammatory cytokines IL6 (A) and TNFα (B) in week 4 (left panels) and week 13 (right panels) lesions. Immunofluorescence was performed on tissues obtained from two animals – one “early” and one “late” animal.

We confirmed our microarray results by quantitatively analyzing the expression of numerous chemokine ligands and receptors, using RT-PCR arrays (Fig. 5). However, CCL25 was not included in this array. The expression of this ligand was confirmed by routine RT-PCR. The increased expression of all the chemokine ligands and receptors from early lesions was confirmed by RT-PCR. Similarly the expression of a majority of these genes was repressed in late lesions, with the exception of CCL18 and CCL25, which exhibited significantly more levels of expression in late, relative to early lesions (Fig. 5). The expression of many other related molecules that were present on the PCR array, including TNF, TREM1, MMP7 and CKLF was also in agreement with microarray results (not shown). To confirm the array results at the protein level, we performed confocal multi-label immune fluorescence using antibodies specific for IL6 (Fig 6A) and TNF (Fig. 6B). Sectioned granuloma samples from early and late stages were profiled for the expression of these two antigens and the presence of *Mtb*. Cells were detected by staining for TO-PRO-3, a nucleolar marker. The left panel in Figure 6A clearly shows an extremely high level of IL6 expression in early TB lesions, relative to the late TB lesions in the right panel of the same figure. Similarly, the left panel in Figure 6B shows an early TB lesion with cells expressing higher levels of TNF, relative to the right panel in the same figure, where very few cells from a late lesion express TNF. These results are consistent with our microarray observations. Not only was the expression of IL6 and TNF lower in late lesions, but it was confined to the outer edges of the generally circular granulomas. This was in contrast to early lesions, where the expression of IL6 and TNF would be seen central areas of the lesions, in the vicinity of *Mtb* infected macrophages. The load of *Mtb* in the BALs and the lungs of week 4 animals were not higher than those of the week 13 animals (not shown). Therefore, these results are not due to the clearance of *Mtb* from these animals. Rather, these results point to the amelioration of inflammation in the lungs of late stage animals, perhaps as a function of changes in bacterial physiology and the resulting changes in its antigen profile.

### Pathway analysis of “early” and “late” TB lung lesions

To visualize microarray data at a global level, we independently uploaded lists of genes which exhibited statistically significant differential gene expression in early as well as late lesions into Ingenuity Pathways. We sought to determine which of the established immune signaling pathways are perturbed to a level significantly higher than what could be expected just by chance. Pathway results mirror our assessment that the highly pro-inflammatory environment of the early TB lesions is replaced by an anti-inflammatory one in the late lesions. Pathways involved in chemokine (Fig. S1A [early] & S1B [late]) and interferon signaling (Fig. S2A & S2B), nitric oxide production (Fig. S3A & S3B), MHC antigen presentation (Fig S4A & S4B) differentiation of T helper cells (Fig. S5A & S5B) and maturation of DCs (Fig. S6A & S6B) were significantly perturbed in both early and late lesions. However, these pathways appeared to be turned on in early and turned off in late lesions. These results demonstrate the system-wide extent of transcriptional changes in these early and late animals.

## Discussion

The long-term control of TB requires a better understanding of virulence and pathogenesis in appropriate animal models. The architecture and organization of the NHP granulomas is highly similar to those seen in humans. Such hypoxic and necrotizing lesions are critical for containing *Mtb* replication [19]. Hypoxia and necrosis may have a profound influence on the physiology of *Mtb*, rendering a study of host-pathogen interactions in non-hypoxic models misleading [20]. Characterization of primate TB granulomas will allow a better understanding of latency and reactivation. The view that granuloma formation is exclusively mediated by the host immune system has recently been challenged [21]. It is thought that *Mtb* may promote the granulomatous response as it eventually allows the pathogen to survive in a chronic state [21]. The study of primate granulomas is also significant because of the potential to identify targets of host-specific therapy against TB.

We characterized the necrotic lung granulomas in NHPs, at an early, and a late stage of infection by transcriptomics and pathways analysis. Expression was confirmed by RT-PCR and confocal microscopy. There are two important results. Firstly, early TB granulomas in *Mtb* infected NHPs exhibited an immensely pro-inflammatory profile. This result was expected, although the breadth of this response, i.e. the comprehensive recruitment of multiple and diverse immune signaling pathways in these lesion tissues was surprising. Secondly, and more importantly, we found that the initial pro-inflammatory response in these TB lesions is almost entirely reversed within a matter of weeks. The breadth and the rapid nature of this transcriptional reprogramming were surprising. It is generally accepted that macaques develop various outcomes of TB depending upon the strain of *Mtb* as well as the initial inoculum. Low-dose infection is supposed to cause latent TB [11] while high-dose infection can lead to acute pulmonary TB [11], [15]. The accepted paradigm for the course of progression of TB in a large majority of humans (∼90%) and a large percentage of NHPs (∼50%) is that following initial infection, granulomatous lesions control the spread and multiplication of *Mtb*. This leads to fundamental changes in the pathogen's metabolism and physiology, establishing a dynamic where *Mtb* can be retained in a chronic, persistent state.

Based on our results, we postulate that TB lesions reprogram their physiological status over the course of time. Possibly, from the pathogen's standpoint, this reprogramming could involve the induction of the known markers for persistence e.g. the hypoxia induced DosRST regulon [22], and the repression of antigenic *Mtb* virulence factors. This could in turn force the host to reprogram its own response to the infection, by silencing pro-inflammatory immune signaling pathways dependent on chemokines, IFNγ and TNF.

### Genes with a higher expression in early granulomas

The induction of numerous chemokines in early TB lesions was not surprising, since these chemokine gradients guide the various immune effector cells to the site of infection. The induced chemokines include the monocyte chemotactic CXCL3, the lymphocyte chemotactic CXCL9, CXCL10 and CXCL11 [23] and CXCL13. The expression of these genes is also induced in the *Mtb* infected lungs of mice [24]–[26]. The expression of CXCL13 correlates with the recall response of the IL-17-producing CD4+ T cells in mice vaccinated against and infected with *Mtb* [27]. The expression of CCL3 and CCL4 was also significantly increased.

Increased expression of these two ligands has been observed following *Mtb* infections in vitro, in vivo and in HIV/TB co-infection [28]–[30]. These two CCR5 ligands may shape the migration of DCs to and from the lymph nodes during infection [31]. The role played by pro-inflammatory immune signaling pathways involving IFNγ and TNFα in protection against *Mtb* infection is well understood [16]. The increased levels of various immune cell surface markers and antigens presumably reflect the migration of these cells to the cell dense granuloma (Table S1). The expression of toll-receptor, defensin and complement genes is indicative of the involvement of innate immune response in early granulomatous response. Increased expression of a large number of apoptotic genes reflects the attempt by the innate immune system to contain infection by limiting the growth of *Mtb*, and reveals the role played by the various caspases in this process. The induction of metallothioneins and metalloproteases in early TB lesions was particularly interesting. Recent studies have shown the importance of MMP9 in interacting with *Mtb* secreted proteins and inducing the formation of granuloma lesions [32]. Recent reports have also suggested that the host ubiquitination machinery, involved in tagging proteins for degradation, may also recognize *Mtb* that escape from phagosomes [33]. This is reflected in the induction of numerous genes encoding ubiquitination proteins in early TB lesions.

### Genes with lower expression in early TB granulomas

The expression of numerous MAP kinases, calcium binding/signaling and immune signaling repressors/negative regulators was repressed in early lesions, relative to normal lung. These novel results likely reveal the immunomodulatory effects of *Mtb* on host lungs. Bacterial infections can mobilize intracellular calcium that favors pro-inflammatory responses [34]. It has been suggested that one of the ways in which *Mtb* represses the pro-inflammatory responses at the site of infection is by modulating the levels of Ca^2+^ [34]. Our results likely reflect this phenomenon. Similarly, studies have shown that MAP kinase signaling leads to reduced TNFα expression during infection [35]. We propose that the early repression of both calcium and MAPK signaling may, in part, be responsible for the silencing of pro-inflammatory responses observed at later stages.

### Genes with higher expression in late TB granulomas

The expression of CCL18 has been shown to be induced by *Mtb* infection of human macrophages [36]. Surprisingly, CCL18 was one of the few chemokine ligands, whose expression was not silenced in late lesions (Fig. 5, Tables S6, S8). The expression of chemokines CCL24, CCL25 and CCL27 was in fact higher in late rather than early lesions. While the expression of CCL25 was not induced in early lesions but strongly induced at week 13, the expression of CCL18 was induced at both stages. The role of these chemokines in response to *Mtb* infection remains to be clarified. It is possible that CCL18, CCL24, CCL25 and CCL27 negatively regulate the function of other chemokines by competing for common receptors. Alternatively, it is possible that they are responsible for the chemotactic inclusion of specific immune effector cell subtypes required to maintain granuloma structure. Finally, it is possible that these chemokines are induced in response to the production of alternative antigens by *Mtb* during persistent growth. Interestingly, CCL25 is also expressed in small intestine, and studies have shown that its expression, unlike other chemokine ligands, is not enhanced by inflammatory stimuli and is independent of signaling through the lymphotoxin α receptor [37]. These results are concordant with our results which show increased expression of CCL25 in late but not early lesions. CCL25 is an important lymphocyte recruiter, particularly those expressing α4β7, and its regulation is rather unique, involving caudal homeo-box transcription factors [37]. While further experiments are necessary, lung levels of CCL25 and its signaling components may be of interest as markers of *Mtb* growth restriction and latent TB.

Other genes with elevated expression in late TB lesions appear to modulate the pro-inflammatory immune response. A2M codes for alpha-2 macroglobulin, which interacts with cytokines to down-regulate acute responses [38]); SOCS2 and SOCS3 are suppressors of cytokine signaling; TGFBI14 and TGFBR3 belong to the TGFB family. TGFBR3, in particular, acts as a reservoir for the various members of the TGFB family of ligands at the cell surface. Thus, its induction is indicative of the higher expression/activity of these ligands. Other genes which were induced at this time point are important, directly or indirectly, for the perpetuation of *Mtb* virulence, including CD36 [39], CD47- an integrin associated molecule involved in the increase in intracellular Ca^2+^, laminins - basolateral membrane components and presumptive targets of the key *Mtb* virulence factor ESAT-6 [40], BCOR- a co-repressor for the pro-apoptotic BCL6; BCL2L12- which performs anti-apoptotic functions through G2/M arrest and ARRB2, which silences activated G-protein signaling. Consistent with the induction of ARRB2 in late lesions, the expression of numerous G protein signaling molecules, which was elevated in early lesions (Table S7), was silenced in late lesions (Table S8).

### Genes with a lower expression in late TB lesions

The transcriptome observations from week 13 lesions indicate a comprehensive silencing of the inflammatory response at the chronic stage of the disease. This is manifested in the complete reversal of the expression of chemokine, cytokine, IFN and TNF gene families. Further, the silencing of immune cell markers may reflect the progression of central necrosis at this time, causing the death of macrophages and lymphocytes. This further leads to the repression of key immune signaling pathways, involving growth factors/receptors and immune relevant transcription factors. Silencing of the apoptotic response in the lung lesions in this chronic phase of TB may either reflect a lower antigenic load, or an attempt by *Mtb* to actively silence the host pro-apoptotic response in lung tissues, thus perpetuating the survival program of the pathogen.

We show that the host mounts a massively inflammatory Th1-type immune response to aerosol *Mtb* infection. This is characterized by the recruitment of highly activated T-lymphocytes and macrophages to the lungs, and expression of IFNγ, TNF, cytokine and chemokine signaling pathways, four weeks post-infection. Simultaneously, a significant increase in apoptotic pathways was also observed. However, by week 13 post-infection, the pro-inflammatory response was largely silenced, even though the animals continued to exhibit clinical signs of active TB. Instead, these lesions are characterized by a Th2-type response. This suggests the existence of a complex balance between pro- and anti-inflammatory responses, which determine the progression of the granulomatous response.

While we are excited by our results, indeed these experiments represent only the first steps in the complete molecular characterization of primate TB granuloma lesions. A granuloma is a complex entity with multiple immune cell types participating. In future experiments, it would be important to completely characterize the cellular source of these observed gene-expression changes. By separating the various cell types in live granulomas, and the application of flow-cytometry, transcriptomics and confocal microscopy, we have recently begun to address this question. Another question to be answered is how co-infection with AIDS negatively impacts the transcriptional characteristics of these primate lesions. As an extension, one could study if prior protective vaccination, e.g. with BCG, could positively impact the lesion character. These studies could promote our understanding of the individual correlates of protection against TB.

## Methods

### Ethics Statement

The Tulane National Primate Research Center facilities are accredited by the American Association for Accreditation of Laboratory Animal Care and licensed by the US Department of Agriculture. All animals were routinely cared for according to the guidelines prescribed by the NIH Guide to Laboratory Animal Care. NHP studies were conducted following the recommendations of the institutional animal care and use committee. Humane endpoints were pre-defined in this protocol and applied as a measure of reduction of discomfort.

### NHP infection and veterinary procedures

*Mtb* infection, clinical management of TB and necropsy of NHPs have been described [15].

### Flow cytometry

BAL sample was collected in normal saline containing 10% (final) fetal bovine serum. The cells were washed and suspended in complete RPMI-10. All lymphocytes were >90% viable by trypan blue dye exclusion method. BAL cells from three NHPs were stained and analyzed using previously described protocols [41]. Polychromatic flow cytometry was performed on FACSAria (BD Biosciences) using PE-Texas Red, PE-Cy7, APC-Cy7, Pacific Blue and Qdot655 as the available fluorophores. Monoclonal antibodies CD3 (SP32-2), CD69 (FN50), HLA-DR (L243) were obtained from BD Biosciences. CD8 PE-Texas red (MHCD0817) and CD4 Qdot655 (T4/19Thy5D7) were obtained from Caltag Laboratories and the NIH Nonhuman Primate Reagent Resource courtesy of Dr. K. Reimann (Harvard) respectively. Data was analyzed using the FlowJo software (TreeStar) version 8.8.6.

### DNA Microarrays

Numerous surgically excised granulomatous lesion and non-granulomatous areas of lung tissue were obtained, 4 and 13 weeks post-*Mtb* infection. A total of three animals were profiled for each time point using microarrays. Lesions from the same animal were pooled and transcriptome profiled relative to the total pool of non-granulomatous “normal” lung. Pools contained lesion tissues from the same animal. As controls, we also obtained lung tissue sections from normal, uninfected animals. RNA was isolated from fresh lung using the RiboPure kit (Ambion), and profiled using macaque whole genome 4x44 k microarrays (Agilent Technologies). Raw array results from our experiments can be retrieved from the Gene Expression Omnibus (GPL10183). A stringent set of criteria applied to remove erroneous data from consideration [42]. Remaining data was log_2_ transformed and normalized using Locally Weighted Scatter-plot Smoothing in Spotfire S^+^. Genes were considered to be differentially expressed if they exhibited a 2-fold perturbation in gene expression magnitude in each of the three biological replicates, in a statistically significant manner (P<0.05). Statistical analysis of pair-wise association within different data sets was performed through a chi-squared test, using *STATISTICA* (Statsoft). Gene-sets from 4 and 13 week lesions were uploaded to Ingenuity Pathways Analysis (IPA) database (Ingenuity Systems, Redwood City, CA), and statistically significant changes in immune signaling pathways were catalogued.

### Confirmation of transcriptome results by RT-PCR and confocal microscopy

To validate transcriptome results, we performed quantitative RT-PCR on chemokine receptors and ligands using a pathway specific RT2Profiler PCR array (SABiosciences). Multilabel immune-fluorescence confocal microscopy has been described earlier [15]. In order to assay for the expression of specific antigens, antibodies against IL6 (ProSpec-Tany #mAHuIL6, made in mouse, 1∶2000 dilution) and TNFα (Becton Dickenson #558882, made in mouse, 1∶10 dilution) were labeled with Alexa Fluor 488 secondary antibodies. *Mtb* were detected using an antibody from Biocare Medical #CP140, made in rabbit and used at a 1∶100 dilution, and detected with an Alexa Fluor 568 secondary antibody. Host cells were revealed by labeling with a nuclolear marker (TO-PRO-3 iodide from Invitrogen #T-3605).

### Footnotes

All animal procedures were approved by the Tulane Institutional Animal Care and Use Committee (IACUC). All procedures related to *Mtb* were approved by the Tulane Institutional Biosafety Committee (IBC). DNA Microarray data associated with this manuscript can be obtained from Gene Expression Omnibus (accession number GPL10183).

## Supporting Information

Table S1 This table contains a comprehensive list of all NHP genes with a higher expression in a statistically significant manner, in early (week 4) TB lesions, relative to normal lungs.
(0.25 MB PDF)

Table S2 This table contains a comprehensive list of all NHP genes with a lower expression in a statistically significant manner, in early (week 4) TB lesions, relative to normal lungs.
(0.26 MB PDF)

Table S3 DNA Microarray Analysis: Immune function genes with significantly enhanced expression in Mtb granuloma's relative to non-granulomatous tissue four week's post-infection. Symbol  =  Official NCBI human gene symbol associated with that gene. P  =  p value of significance in a t-test.
(0.02 MB DOCX)

Table S4 DNA Microarray Analysis: Immune function genes with significantly reduced expression in Mtb granuloma's relative to non-granulomatous tissue four week's post-infection. Symbol  =  Official NCBI human gene symbol associated with that gene. P  =  p value of significance in a t-test.
(0.02 MB DOCX)

Table S5 This table contains a comprehensive list of all NHP genes with a higher expression in a statistically significant manner, in late (week 13) TB lesions, relative to normal lungs.
(0.21 MB PDF)

Table S6 This table contains a comprehensive list of all NHP genes with a lower expression in a statistically significant manner, in late (week 13) TB lesions, relative to normal lungs.
(0.20 MB PDF)

Table S7 DNA Microarray Analysis: Immune function genes with significantly enhanced expression in Mtb granuloma's relative to non-granulomatous tissue four week's post-infection. Symbol  =  Official NCBI gene symbol associated with that gene. P  =  p value of significance in a student's t-test.
(0.01 MB DOCX)

Table S8 DNA Microarray Analysis: Immune function genes with significantly reduced expression in Mtb granuloma's relative to non-granulomatous tissue four week's post-infection. Symbol  =  Official NCBI gene symbol associated with that gene. P  =  p value of significance in a student's t-test.
(0.02 MB DOCX)

Table S9 This table contains the detailed composition of all 10 K-means clusters defined in the statistically significant data set between early and late lesions.
(1.02 MB PDF)

Table S10 This table contains genes with a higher expression in both early and late lesions (i.e. the overlapping genes in Fig. 4A).
(0.05 MB PDF)

Table S11 This table contains genes with a lower expression in both early and late lesions (i.e. the overlapping genes in Fig. 4B).
(0.05 MB PDF)

Table S12 This table contains genes with a higher expression in early but a lower expression in late lesions (i.e. the overlapping genes in Fig. 4C).
(0.13 MB PDF)

Table S13 This table lists genes with a lower expression in early but a higher expression in late genes (i.e. the overlapping genes in Fig. 4D).
(0.11 MB PDF)

Figure S1 Comparison of the chemokine signaling pathway in early and late TB granulomas. Canonical pathways contained within the IPA algorithm were queried with the list of genes significantly up (red) or down (green) regulated in week 4 or week 13 lesions. Pathway illustrations are shown for week 4 (A) and week 13 (B) lesions.
(0.75 MB TIF)

Figure S2 Comparison of the interferon signaling pathway in early and late TB granulomas. Canonical pathways contained within the IPA algorithm were queried with the list of genes significantly up (red) or down (green) regulated in week 4 or week 13 lesions. Pathway illustrations are shown for week 4 (A) and week 13 (B) lesions.
(1.43 MB TIF)

Figure S3 Comparison of the nitric oxide production pathway in early and late TB granulomas. Canonical pathways contained within the IPA algorithm were queried with the list of genes significantly up (red) or down (green) regulated in week 4 or week 13 lesions. Pathway illustrations are shown for week 4 (A) and week 13 (B) lesions.
(0.96 MB TIF)

Figure S4 Comparison of the MHC antigen presentation pathway in early and late TB granulomas. Canonical pathways contained within the IPA algorithm were queried with the list of genes significantly up (red) or down (green) regulated in week 4 or week 13 lesions. Pathway illustrations are shown for week 4 (A) and week 13 (B) lesions.
(1.40 MB TIF)

Figure S5 Comparison of the T-helper cell differentiation pathway in early and late TB granulomas. Canonical pathways contained within the IPA algorithm were queried with the list of genes significantly up (red) or down (green) regulated in week 4 or week 13 lesions. Pathway illustrations are shown for week 4 (A) and week 13 (B) lesions.
(1.46 MB TIF)

Figure S6 Comparison of the DC maturation pathway in early and late TB granulomas. Canonical pathways contained within the IPA algorithm were queried with the list of genes significantly up (red) or down (green) regulated in week 4 or week 13 lesions. Pathway illustrations are shown for week 4 (A) and week 13 (B) lesions.
(3.22 MB TIF)
