# Supplementary material for: Transcriptional Reprogramming in Nonhuman Primate (Rhesus Macaque) Tuberculosis Granulomas
Source: PLoS One. 2010 Aug 31;5(8):e12266. doi: 10.1371/journal.pone.0012266 (PMC2930844; doi:10.1371/journal.pone.0012266)
Supplement: Table S1 — This table contains a comprehensive list of all NHP genes with a higher expression in a statistically significant manner, in early (week 4) TB lesions, relative to normal lungs. (0.25 MB PDF) [file pone.0012266.s001.pdf]

GeneName	Description	Symbol	Granuloma Av M	Granuloma Av Num# Ratio	Normal Av M	Normal Av Num# Ratio	P	Fold Change (Granuloma / Normal)
CO647377	Apolipoprotein L, 3	APOL3	2.969558985	7.832967568	-0.108891899	0.927300026	0.001081427	8.447069283
CO648453	General transcription factor IIH, polypeptide 5	GTF2H5	2.459941762	5.501945167	0.101241941	1.072696494	9.64E-05	5.1290791
CO649006	Outer dense fiber of sperm tails 1 isoform 1	C9orf123	1.15579344	2.228068272	-0.346894796	0.786274627	0.016954206	2.833702368
CN648464	24-dehydrocholesterol reductase	DHCR24	1.117078763	2.169073234	-1.524765241	0.347536102	0.004197939	6.241288947
CK230655		Hs.500464	1.084442805	2.120556327	-1.321155844	0.400214171	2.67E-05	5.298553828
DR774422		Hs.529672	2.78381345	6.886702951	-0.137628429	0.909012205	8.06E-06	7.576029133
CN806583	Interferon Induced Transmembrane Proteir	IFITM1	1.127723712	2.185136966	-0.50154275	0.706351039	0.00021833	3.093556668
CN641426	Mesoderm development candidate 2	MESDC2	1.515683993	2.859343595	-0.362102744	0.778029766	0.02638038	3.675108228
CO647336	anterior pharynx defective 1B	PSFL	2.417024092	5.340682399	-0.492904378	0.710593118	0.001535143	7.515809346
CO580929	Serum Amyloid A2	SAA2	3.514553518	11.42841573	0.249310027	1.188638509	2.24E-06	9.614710983
DR771539	antioxidant protein 1	ATOX1	1.988195488	3.967404479	-0.33794571	0.791167073	0.000105455	5.014622845
NM_001090	ATP-binding cassette, sub-family F	ABCF1 2	1.194331435	2.288387605	-0.329514352	0.795804327	0.000482828	2.875565673
NM_013375	activator of basal transcription 1	ABT1	1.106965384	2.153921071	-0.33021675	0.795416972	0.002635466	2.707914398
NM_014049	acyl-Coenzyme A dehydrogenase family, member 9	ACAD9	1.269256946	2.410373883	-0.055586504	0.962203196	0.008727031	2.505057034
NM_001610	acid phosphatase 2, lysosomal	ACP2	1.302909803	2.467260082	-0.887331595	0.540613111	0.000916951	4.563818435
NM_001611	acid phosphatase 5, tartrate resistant	ACP5	1.453588661	2.738884941	-0.35871667	0.779857983	0.001158897	3.512030395
CO647113		ACSL1	2.241933083	4.73030459	-0.985644019	0.505000241	2.60E-05	9.366935314
NM_004458	acyl-CoA synthetase long-chain family member 4	ACSL4 1	1.916207085	3.774294728	-0.571826033	0.672764725	0.000279687	5.610125784
NM_001615	actin, gamma 2, smooth muscle, enteric	ACTG2	1.519410692	2.86673926	-0.307875704	0.807830374	0.001215102	3.548689617
NM_001005386	ARP2 actin-related protein 2 homolog	ACTR2 1	1.762491678	3.392835957	-0.026230296	0.98198283	0.004027521	3.455086846
NM_005721	ARP3 actin-related protein 3 homolog	ACTR3	1.832777827	3.562222984	-0.686145601	0.621512107	0.007789307	5.731542388
NM_003183	a disintegrin and metalloproteinase domain 17 tumor necrosis factor, alpha, converting enzyme	ADAM17 1	1.601765719	3.035145586	-0.329902372	0.79559032	0.001091776	3.814960425
NM_033274	a disintegrin and metalloproteinase domain 19 meltrin beta	ADAM19 2	1.504004829	2.836289565	0.392428971	1.312601486	0.002141098	2.160815446
NM_001109	a disintegrin and metalloproteinase domain 8	ADAM8	1.488585598	2.806137296	-0.506249703	0.704050245	0.000893091	3.985706013
NM_014479	ADAM-like, decysin 1	ADAMDEC1	3.456507408	10.97772659	-1.170077423	0.444397491	0.03309358	24.70249453
NM_006988	a disintegrin-like and metalloprotease reprolysin type with thrombospondin type 1 motif, 1	ADAMTS1	1.375695624	2.594930005	-0.540963492	0.68731174	0.000112307	3.775477491
NM_014244	a disintegrin-like and metalloprotease reprolysin type with thrombospondin type 1 motif, 2	ADAMTS2 1	1.024269	2.033928551	0.049231298	1.034713457	0.017004033	1.965692565
NM_182920	a disintegrin-like and metalloprotease reprolysin type with thrombospondin type 1 motif, 9	ADAMTS9 1	1.247493848	2.374286196	0.003194259	1.002216545	0.010173561	2.369035124
NM_052853	aarF domain containing kinase 2	ADCK2	1.527966602	2.883790983	-1.271656716	0.414183873	0.016746418	6.962586362
NM_001122	adipose differentiation-related protein	ADFP	3.746960087	13.42602275	0.108445472	1.078065976	0.000416758	12.45380436
NM_001124	adrenomedullin	ADM	2.005520739	4.015336063	0.171111503	1.125925603	7.39E-05	3.566253447
NM_000676	adenosine A2b receptor	ADORA2B	1.912942839	3.765764652	-0.0818673	0.944833943	0.002291641	3.985636501
NM_006796	AFG3 ATPase family gene 3-like 2	AFG3L2	1.077231352	2.109982966	-0.016118797	0.988889484	1.56E-05	2.133689356
NM_020350	angiotensin II receptor-associated protein	AGTRAP	1.482306991	2.793951527	-0.038340472	0.973774434	0.000320019	2.869197866
NM_005161	angiotensin II receptor-like 1	AGTRL1	1.92452245	3.796111715	0.082005099	1.058488136	0.028300038	3.586352633
NM_000687	S-adenosylhomocysteine hydrolase	AHCY	1.170624286	2.251090855	-0.045410648	0.969013958	0.027466567	2.323073714
NM_004847	allograft inflammatory factor 1	AIF1 2	2.622316165	6.157378115	0.513994577	1.427998612	0.001080572	4.311893627

NM_006066	aldo-keto reductase family 1, member A1	AKR1A1 1	1.259203626	2.393635747	-0.346053302	0.786733378	0.000177322	3.042499294
NM_001628	aldo-keto reductase family 1, member B1	AKR1B1	1.121116465	2.175152368	-0.062091611	0.957874392	4.33E-05	2.270811693
NM_000689	aldehyde dehydrogenase 1 family, member A1	ALDH1A1	1.067571074	2.095901728	-0.499022466	0.707586061	0.001303318	2.962044961
NM_000382	aldehyde dehydrogenase 3 family, member A2	ALDH3A2	1.172338462	2.253767138	-0.569314539	0.673936917	0.001028457	3.344181156
NM_000034	aldolase A, fructose-bisphosphate	ALDOA 1	1.303969794	2.469073518	-0.65277354	0.636056338	8.16E-06	3.881847208
NM_005165	aldolase C, fructose-bisphosphate	ALDOC	3.068289799	8.387784515	0.419932076	1.337864565	0.003620158	6.269531863
NM_033087	asparagine-linked glycosylation 2 homolog	ALG2 1	1.512120395	2.852289453	0.496848619	1.411127767	0.047548638	2.021283629
NM_013338	asparagine-linked glycosylation 5 homolog	ALG5	1.014699792	2.020482411	-0.113613547	0.924270122	0.005655091	2.186030211
NM_001629	arachidonate 5-lipoxygenase-activating protein	ALOX5AP	2.127034061	4.368185313	-0.056898475	0.961328577	0.001225297	4.54390457
CO579644		ANAPC13	1.4085828	2.654762499	-0.621639842	0.649931762	0.000569912	4.084678816
NM_144590	ankyrin repeat domain 22	ANKRD22	5.050606123	33.14239884				
NM_152326	ankyrin repeat domain 9	ANKRD9	2.537489921	5.805780064	-0.438428389	0.737938049	0.012619033	7.867571092
NM_032208	anthrax toxin receptor 1	ANTXR1 1	2.121750005	4.352215535	0.092575066	1.066271673	0.001723611	4.081713548
NM_001153	annexin A4	ANXA4	1.108800277	2.15666228	-0.583479133	0.66735248	0.014004477	3.231668936
NM_001637	acyloxyacyl hydrolase	AOAH	2.620406269	6.14923213	-0.039663208	0.972882037	0.000343329	6.320634875
NM_001127	adaptor-related protein complex 1, beta 1 subunit	AP1B1-1	1.085264025	2.121763747	0.061571952	1.043602244	0.009291004	2.033115355
NM_032493	adaptor-related protein complex 1, mu 1 subunit	AP1M1	1.419865474	2.675605608	-0.690181503	0.619775872	0.003645979	4.317053517
NM_004069	adaptor-related protein complex 2, sigma 1 subunit	AP2S1	1.714279064	3.281326298	0.126818365	1.091883069	0.000176548	3.005199357
XR_013467	adaptor-related protein complex 3, beta 1 subunit	AP3B1	1.160904197	2.235975215	-0.266306531	0.831445422	0.005268058	2.689262767
NM_006803	adaptor-related protein complex 3, mu 2 subunit	AP3M2	2.825311869	7.087672137	-0.181656517	0.881690049	0.01927645	8.038734407
NM_019043	amyloid beta	APBB1IP	1.301258099	2.464436997	0.328438979	1.255654	0.001790587	1.96267204
NM_022488	APG3 autophagy 3-like	APG3L	1.663351717	3.167515584	-0.0027669	0.998083969	0.006669036	3.173596293
NM_021822	apolipoprotein B mRNA editing enzyme, catalytic polypeptide-like 3G	APOBEC3G	1.67186975	3.186272708	-2.251172409	0.210053334	0.000939719	15.16887471
NM_001645	apolipoprotein C-I	APOC1	1.988911694	3.969374529	0.089777569	1.064206093	8.23E-07	3.72989269
NM_003661	apolipoprotein L, 1	APOL1 1	3.600653078	12.13122284	-0.081848687	0.944846133	1.85E-05	12.83936338
NM_030643	apolipoprotein L, 4	APOL4 a	2.481223264	5.583707094	-0.125579542	0.916635749	0.012451692	6.091522284
NM_020979	adaptor protein with pleckstrin homology and src homology 2 domains	APS	1.227716496	2.341960099	-0.112282331	0.925123367	0.002126888	2.531511129
NM_001655	archain 1	ARCN1	1.252778043	2.382998499	0.171191921	1.125988366	0.02196403	2.116361564
NM_001659	ADP-ribosylation factor 3	ARF3	1.034902881	2.048975709	-0.127277252	0.915557721	0.00019668	2.237953612
NM_001660	ADP-ribosylation factor 4	ARF4	1.599638189	3.03067298	-0.350733582	0.784185254	0.005992696	3.864741099
NM_014570	ADP-ribosylation factor GTPase activating protein 3	ARFGAP3	1.549945375	2.928060524	-0.684882146	0.62205664	0.003427526	4.707064171
CN644332		ARHB	1.61880944	3.071214847	-0.532455106	0.691377182	0.015033846	4.442169815
NM_001007231	Rho GTPase activating protein 25	ARHGAP25	1.193240845	2.286658376	0.208127018	1.155187486	0.001895857	1.979469484
NM_004309	Rho GDP dissociation inhibitor	ARHGDIA	1.022076779	2.030840278	-0.692258427	0.618884276	0.000103932	3.281453992
NM_001011722	Rho guanine nucleotide exchange factor	ARHGEF10L 2	1.667585857	3.176825523	0.293013753	1.225197012	0.013410204	2.592909951
NM_001177	ADP-ribosylation factor-like 1	ARL1	2.349826095	5.097627994	-0.233305704	0.850683453	0.012652916	5.992391151
NM_012106	ADP-ribosylation factor-like 2 binding protein	ARL2BP	1.851973247	3.609935967	-1.434279383	0.37003166	0.008941701	9.75574893
NM_005738	ADP-ribosylation factor-like 4A	ARL4A 1	3.073630459	8.418892495	-1.061151734	0.479249312	0.009152749	17.56683272
NM_015161	ADP-ribosylation factor-like 6 interacting protein	ARL6IP	1.924361483	3.795688193	0.214958188	1.160670276	0.001978978	3.270255362
CN644516		ARL7	1.414757919	2.666149943	-0.55730172	0.679571983	0.002941653	3.923278195
CO725904		ARP10	1.245553258	2.371094657	0.033868417	1.023753523	0.028382649	2.316079606
NM_005720	actin related protein 2/3 complex, subunit 1B, 41kDa	ARPC1B	1.521639259	2.871171005	-0.203647159	0.868352575	0.000262557	3.306457639

NM_183376	arrestin domain containing 4	ARRDC4	1.266270832	2.405390011	-0.386673366	0.764891295	0.026806133	3.144747531
NM_024769	adipocyte-specific adhesion molecule	ASAM	1.253892763	2.38484047	-0.486340359	0.713833563	0.013333438	3.340891482
NM_005170	achaete-scute complex-like 2	ASCL2	1.729403158	3.31590611	0.331211007	1.258068962	0.002570446	2.635710928
NM_025080	asparaginase like 1	ASRGL1	1.341610608	2.534340918	-0.403841705	0.755842892	0.023374452	3.352999605
NM_004024	activating transcription factor 3	ATF3	2.136388802	4.396601581	-0.363230752	0.777421681	0.000704724	5.655362704
NM_007348	activating transcription factor 6	ATF6	1.584951244	2.999976593	-0.380658461	0.768086948	0.035309755	3.905777337
XM_087254	ATPase, Class VI, type 11B	ATP11B	1.589249646	3.008928126	-0.481433494	0.716265573	0.009763585	4.200855437
NM_001677	ATPase, Na+/K+ transporting, beta 1 polypeptide	ATP1B1	1.819686742	3.530045409	0.118867568	1.085882173	0.001948079	3.250854923
NM_001679	ATPase, Na+/K+ transporting, beta 3 polypeptide	ATP1B3	1.418062328	2.672263603	-1.20425128	0.433994517	0.003067309	6.157367202
NM_001001486	ATPase, Ca++ transporting, type 2C, member 1	ATP2C1 4	1.19010083	2.281686894	-0.088461846	0.940524972	0.009550454	2.425971625
NM_001686	ATP synthase, H+ transporting, mitochondrial F1 complex, beta polypeptide	ATP5B	1.242373721	2.365874786	-0.385156374	0.765696	2.12E-05	3.089835633
NM_001687	ATP synthase, H+ transporting, mitochondrial F1 complex, delta subunit	ATP5D	1.739422376	3.339014541	-1.254750854	0.419065932	0.003501449	7.967754688
NM_006886	ATP synthase, H+ transporting, mitochondrial F1 complex, epsilon subunit	ATP5E	1.155273638	2.227265645	-0.051586294	0.964874832	0.000919219	2.308346712
NM_005765	ATPase, H+ transporting, lysosomal accessory protein 2	ATP6AP2	1.639246023	3.115029926	0.157172286	1.115099375	0.002930693	2.793499839
NM_001694	ATPase, H+ transporting, lysosomal 16kDa, V0 subunit c	ATP6V0C	2.120853402	4.349511569	-0.441703449	0.736264757	4.54E-06	5.907537371
NM_004691	ATPase, H+ transporting, lysosomal 38kDa, V0 subunit d isoform 1	ATP6V0D1	2.465689725	5.52390966	-0.920704484	0.528251007	0.000355843	10.45697896
NM_001693	ATPase, H+ transporting, lysosomal 56/58kDa, V1 subunit B, isoform 2	ATP6V1B2	2.303725384	4.937310523	-0.515020827	0.69978283	0.001305091	7.055489659
NM_004231	ATPase, H+ transporting, lysosomal 14kDa, V1 subunit F	ATP6V1F	1.273546371	2.417551078	0.067235928	1.047707448	0.000239996	2.307467683
CN646039		ATP6V1G1	1.573816656	2.976912168	-0.734706251	0.600940371	0.000128108	4.953756334
NM_178191	ATPase inhibitory factor 1	ATPIF1	1.563535416	2.955772877	-0.384392603	0.766101471	0.00349977	3.858200236
NM_033027	AXIN1 up-regulated 1	AXUD1	1.506282792	2.840771501	0.001912476	1.001326506	0.001102241	2.837008192
NM_003781	UDP-Gal:betaGlcNAc beta 1,3-galactosyltransferase, polypeptide 3	B3GALT3	2.090876468	4.260068026	-0.338820193	0.790687656	0.005702408	5.387801357
NM_080605	UDP-Gal:betaGal beta 1,3-galactosyltransferase polypeptide 6	B3GALT6	1.389193987	2.619323021	-0.846856717	0.555994795	0.029471535	4.711056726
NM_001497	UDP-Gal:betaGlcNAc beta 1,4- galactosyltransferase, polypeptide 1	B4GALT1	2.316986109	4.982901683	-0.102416179	0.931471686	0.000361726	5.349493451
NM_001186	BTB and CNC homology 1, basic leucine zipper transcription factor 1	BACH1 2	1.587660102	3.005614753	-0.466262773	0.723837234	0.009528205	4.152335098
NM_004281	BCL2-associated athanogene 3	BAG3	1.173713752	2.255916629	-0.21315797	0.862646883	0.005141372	2.615110162
NM_014952	bromo adjacent homology domain containing 1	BAHD1	1.133464341	2.193849168	-1.299744103	0.406198241	0.002457004	5.400932221
NM_001188	BCL2-antagonist/killer 1	BAK1	1.097302578	2.139542857	0.425660676	1.343187469	0.006048658	1.592884766
CO646750		BANK1	2.366087047	5.155409571	-0.002729096	0.998110123	0.011988363	5.165171111
NM_006317	brain abundant, membrane attached signal protein 1	BASP1	1.564999705	2.958774413	0.41973306	1.337680023	0.003452621	2.211870076
NM_006399	basic leucine zipper transcription factor, ATF-like	BATF	3.294985081	9.814978322	0.188875101	1.139874586	0.000438605	8.610577378
NM_078468	BRCA2 and CDKN1A interacting protein	BCCIP B	1.12665152	2.183513605	0.097423098	1.069860799	0.002521719	2.040932434
NM_015367	BCL2-like 13 apoptosis facilitator	BCL2L13	1.42242789	2.680362058	-0.43102872	0.741732701	0.000374017	3.613649574
NM_030766	BCL2-like 14 apoptosis facilitator	BCL2L14 2	3.193509546	9.14833722	-0.054989366	0.962601538	0.025378606	9.503763351
NM_017745	BCL6 co-repressor	BCOR 1	1.731020499	3.319625511	-1.927855457	0.262819558	0.012429544	12.63081613
XR_012860	Trafficking protein particle complex subunit 3	BET3L	1.35853461	2.564245887	-0.042122674	0.971224908	0.000415478	2.640218415

NM_001710	B-factor, properdin	BF	3.538279696	11.61791839	-0.251915649	0.839780592	7.11E-05	13.8344688
CN648569		BHLHB2	1.814199053	3.516643411	-2.15959166	0.223819609	0.027238668	15.71195408
AK074898	cDNA FLJ90417 fis, clone NT2RP3000171, weakly Mus musculus partial mRNA for B-IND1 protein	B-IND1	2.448112269	5.457015981	-0.618004588	0.651571503	0.002249244	8.375160605
NM_001165	baculoviral IAP repeat-containing 3	BIRC3 1	2.214451437	4.641050632	0.118897407	1.085904632	0.000527225	4.2739026
NM_016252	baculoviral IAP repeat-containing 6	BIRC6	1.290117189	2.445479191	-0.188944981	0.877247003	0.00191988	2.787674603
NM_000386	bleomycin hydrolase	BLMH	1.220771123	2.330712609	-0.542126589	0.686757855	0.001147856	3.393790975
NM_078473	BBP-like protein 1	BLP1 1	2.364898314	5.151163433	-0.195371664	0.873347877	0.02329743	5.898180519
NM_001204	bone morphogenetic protein receptor, type II serine/threonine kinase	BMPR2 1	1.854380278	3.615963909	-0.417434296	0.748755035	7.65E-05	4.829301628
NM_004052	BCL2/adenovirus E1B 19kDa interacting protein 3	BNIP3	1.425638955	2.686334492	0.334538263	1.260973767	0.001866158	2.13036509
NM_006768	BRCA1 associated protein	BRAP	1.201395905	2.299620672	0.056156803	1.039692431	0.013288362	2.211827849
NM_015379	brain protein I3	BRI3	1.176414325	2.260143422	0.205243627	1.152881016	2.34E-05	1.960430773
NM_004335	bone marrow stromal cell antigen 2	BST2	1.998138663	3.994842606	0.410645392	1.329280337	4.96E-05	3.005267208
NM_152322	BTB POZ domain containing 11	BTBD11 1	2.30210361	4.931763475	0.292249737	1.224548349	0.026872068	4.027414254
CO645097	Kruppel-like factor 9	BTEB1	1.000927961	2.001286841	-0.263815961	0.832882012	0.000716047	2.402845556
NM_001731	B-cell translocation gene 1, anti-proliferative	BTG1	1.153991727	2.22528748	-0.289155582	0.818380922	0.002775672	2.719134111
NM_024018	butyrophilin, subfamily 2, member A3	BTN2A3	1.459895243	2.750883882	-0.059851268	0.959363018	0.005217354	2.867406634
NM_004725	BUB3 budding uninhibited by benzimidazoles 3 homolog	BUB3	1.608724978	3.049821861	-0.733486056	0.601448846	0.001396566	5.070791776
CN647641		BZRP	1.599060104	3.029458839	-0.32607567	0.797703399	0.000217585	3.797725876
NM_007311	benzodiazapine receptor	BZRP PBR-S	1.153096398	2.223906907	-0.410525338	0.752349366	0.008323534	2.955949731
NM_014038	basic leucine zipper and W2 domains 2	BZW2	1.276723464	2.422880858	0.303053495	1.233752917	0.027364201	1.963829892
NM_031453	chromosome 10 open reading frame 45	C10orf45	1.156972303	2.229889627	-0.203307578	0.868556992	0.009782166	2.567349809
NM_024789	chromosome 10 open reading frame 77	C10orf77	1.426770533	2.688442346	-0.479031733	0.717458986	0.00526005	3.747172171
NM_014206	chromosome 11 open reading frame 10	C11orf10	1.445541095	2.723649569	0.150054621	1.109611481	0.000861261	2.454597501
NM_022338	chromosome 11 open reading frame 24	C11orf24	1.075299173	2.107158991	-0.632913523	0.644872779	0.007251183	3.267557664
NM_006817	chromosome 12 open reading frame 8	C12orf8	1.739275193	3.338673913	-0.142399381	0.906011093	0.000864028	3.685025426
NM_017917	chromosome 14 open reading frame 10	C14orf10	1.347531776	2.544763839	0.121319962	1.087729602	0.002731345	2.339518788
NM_018229	chromosome 14 open reading frame 108	C14orf108	2.2141009	4.639923115	-0.909143709	0.532501056	0.003546051	8.713453364
NM_175748	chromosome 14 open reading frame 130	C14orf130	1.077569481	2.110477548	-0.424840488	0.744921089	0.021948505	2.833155858
CN802384	small subunit of serine palmitoyltransferase A	SSSPTA	1.659941904	3.160037994	-0.01880712	0.9870485	9.41E-05	3.201502252
CN644511	C-type lectin domain family 14, member A	CLEC14A	2.490224421	5.618653455	0.263603482	1.200473438	3.56E-05	4.680364661
NM_024496	chromosome 14 open reading frame 4	C14orf4	1.870638463	3.65694382	-0.892559656	0.538657574	0.007662308	6.788995456
XM_031561	chromosome 15 open reading frame 23	C15orf23	1.909940829	3.757936866	0.091492403	1.065471795	0.046975636	3.527016747
NM_020154	chromosome 15 open reading frame 24	C15orf24	1.92636238	3.800956147	-0.334129625	0.793262566	0.000198019	4.791548613
NM_023933	chromosome 16 open reading frame 24	C16orf24	1.20399638	2.303769504	-0.077651488	0.947598954	0.019850721	2.431165097
NM_152464	chromosome 17 open reading frame 32	C17orf32	1.258373818	2.392259373	-0.401490013	0.757075973	0.001585507	3.159866987
NM_024805	chromosome 18 open reading frame 22	C18orf22	1.230875701	2.347094128	0.36748711	1.290103766	0.003084491	1.819306469
NM_015609	chromosome 1 open reading frame 144	C1orf144	2.810678219	7.016143324	0.162726612	1.119400744	0.023347674	6.267767251
NM_198552	chromosome 1 open reading frame 153	C1orf153	2.931904509	7.631171284	-0.763839531	0.588926897	0.010189915	12.95775643
NM_174896	chromosome 1 open reading frame 162	C1orf162	1.14353124	2.209211036	-1.537790561	0.344412507	0.001422198	6.414433248
NM_138391	chromosome 1 open reading frame 37	C1orf37	1.495179851	2.818992914	-0.41424485	0.75041218	0.002784479	3.756592694
NM_015471	chromosome 1 open reading frame 48	C1orf48	1.148658343	2.21707618	0.396909194	1.316684045	0.017365926	1.683833102
NM_015991	complement component 1, q subcomponent, alpha polypeptide	C1QA	2.216478477	4.647576065	-0.064167198	0.956497301	0.045208195	4.858953665

NM_000491	complement component 1, q subcomponent, beta polypeptide	C1QB	2.02965279	4.083065723	-0.768452376	0.587046881	4.41E-06	6.955263468
NM_001212	complement component 1, q subcomponent binding protein	C1QBP	1.149552357	2.218450492	-0.136381865	0.909797979	0.005353524	2.43839901
CO580739		C1QG	1.731983005	3.321840964	0.166640642	1.1224418	0.002904649	2.959477242
NM_198594	C1q and tumor necrosis factor related protein 1	C1QTNF1	3.491966801	11.2508867	-0.167270523	0.8905259	0.006678319	12.63398031
NM_001734	complement component 1, s subcomponent	C1S 1	1.930918372	3.812978437	-0.095531681	0.935927264	1.83E-06	4.074011502
NM_000063	complement component 2	C2	1.65021381	3.138801532	-0.606782533	0.656659538	0.000582589	4.779952702
NM_080757	chromosome 20 open reading frame 127	C20orf127	1.651055691	3.140633707	0.41260528	1.331087379	0.000944993	2.359449691
NM_015600	chromosome 20 open reading frame 22	C20orf22	1.051735124	2.073021564	0.349213316	1.273865814	0.022311852	1.627346884
CN646485		C20orf3	2.837902901	7.149800073	-0.014626877	0.989912643	0.007174284	7.222657596
NM_018217	chromosome 20 open reading frame 31	C20orf31	1.027355766	2.038284969	-0.370779323	0.773364624	0.041744303	2.63560668
CN804671		C21orf91	1.319034772	2.494991277	-0.825140286	0.564427315	0.00642814	4.420394282
NM_213720	chromosome 22 open reading frame 16	C22orf16	1.823038121	3.538255232	-0.011914033	0.991775827	0.006388551	3.567595757
NM_024053	chromosome 22 open reading frame 18	C22orf18	1.252846553	2.383111664	0.236124427	1.177824368	0.001688579	2.023316658
NM_012264	chromosome 22 open reading frame 5	C22orf5	1.954972967	3.877086618	-0.624940173	0.648446667	0.001451367	5.979037004
NM_000064	complement component 3	C3	1.378461967	2.599910512	-0.579019567	0.669418549	4.03E-05	3.883833984
XR_014546	Complement factor I precursor	C3B	1.348441669	2.546369301	0.122969541	1.088974024	0.032911634	2.338319597
NM_020685	chromosome 3 open reading frame 14	C3orf14	1.718775963	3.291570191	-0.707137061	0.61253447	0.015232879	5.373689727
NM_020199	chromosome 5 open reading frame 15	C5orf15	3.322255349	10.00226861	-1.554069624	0.340548074	0.000205749	29.37109143
NM_018064	chromosome 6 open reading frame 166	C6orf166	1.511589755	2.851240541	0.174256355	1.128382624	0.005691553	2.526838398
NM_018368	chromosome 6 open reading frame 209	C6orf209	1.844923073	3.592337937	-1.62899475	0.323313409	0.010663768	11.11100818
NM_013397	chromosome 6 open reading frame 49	C6orf49	1.024242198	2.033890765	-0.181285486	0.881916831	0.00020547	2.306216067
NM_138408	chromosome 6 open reading frame 51	C6orf51	1.066058528	2.093705501	-0.041088457	0.971921394	0.007261493	2.154192215
NM_145169	chromosome 6 open reading frame 83	C6orf83	1.056210375	2.079462078	0.210713044	1.157260012	0.008153871	1.796884068
NM_138701	chromosome 7 open reading frame 11	C7orf11	1.348921388	2.547216149	0.307168815	1.237277249	0.016365748	2.058727058
NM_032936	chromosome 7 open reading frame 35	C7orf35	1.355287447	2.558480872	-0.166229099	0.891168967	0.000102198	2.8709268
NM_053279	chromosome 8 open reading frame 13	C8orf13	1.861526602	3.633919856	-2.173886918	0.221612795	0.020829224	16.39760854
NM_025232	chromosome 8 open reading frame 20	C8orf20	1.055110324	2.077877098	0.142894573	1.104118159	0.00091441	1.881933632
NM_020130	chromosome 8 open reading frame 4	C8orf4	1.170116946	2.250299374	-0.488819291	0.712608061	0.002573948	3.157835978
NM_001001790	chromosome 9 open reading frame 105	C9orf105	2.032423434	4.090914638	-0.150166506	0.901146452	0.007718164	4.539677904
CO578964		C9orf10OS	1.199468931	2.296551174	-0.44485209	0.734659633	0.005563717	3.126007027
NM_032342	chromosome 9 open reading frame 125	C9orf125	1.42272222	2.680908944	-1.368357521	0.387331967	0.015084682	6.92147608
NM_016481	chromosome 9 open reading frame 156	C9orf156	1.36258403	2.571453436	-0.06150077	0.95826676	0.000306638	2.683442174
NM_022343	chromosome 9 open reading frame 19	C9orf19	1.431760303	2.697756811	-0.003516851	0.997565274	0.008366794	2.704341142
NM_001218	carbonic anhydrase XII	CA12 1	1.294314703	2.452604662	0.192275774	1.142564629	0.028606071	2.146578495
NM_001216	carbonic anhydrase IX	CA9	2.198347777	4.58953432	-0.439077565	0.737606071	5.99E-05	6.222202475
NM_016289	calcium binding protein 39	CAB39	1.058869611	2.083298564	-0.151848146	0.900096667	0.002736163	2.314527584
NM_016547	calcium binding protein Cab45 precursor	Cab45	1.267554234	2.407530765	-0.948358182	0.518221874	0.022339528	4.645752882
NM_001219	calumenin	CALU	1.966187705	3.907342457	-0.47630711	0.718815234	8.93E-05	5.435809192
NM_172115	calcium/calmodulin-dependent protein kinase	CAMK2D 4	2.024253419	4.067813168	-0.110023643	0.926572877	0.031205835	4.390170779
NM_004345	cathelicidin antimicrobial peptide	CAMP	1.224866453	2.337338126	-0.286040122	0.820150102	0.003685739	2.849890672
NM_001746	calnexin	CANX	1.877115762	3.67339939	0.346307736	1.271302837	9.51E-05	2.889476279
NM_006367	CAP, adenylate cyclase-associated protein 1 CaM kinase II delta	CAP1	1.293219024	2.450742697	-0.068763255	0.953454994	0.001646549	2.570381101

NM_001747	capping protein actin filament, gelsolin-like	CAPG	1.947140483	3.856094701	-0.190604459	0.876238519	8.39E-06	4.400736351
NM_001003962	calpain, small subunit 1	CAPNS1 2	1.425228801	2.685570883	-0.425319227	0.744673938	0.004259761	3.606371521
NM_006136	capping protein actin filament muscle Z-line, alpha 2	CAPZA2	1.612961598	3.058791134	-0.036942608	0.974718406	0.004213008	3.138128013
NM_006092	caspase recruitment domain family, member 4	CARD4	1.614955809	3.06302217	-0.522419584	0.696203232	6.99E-05	4.39960924
NM_001223	caspase 1, apoptosis-related cysteine protease interleukin 1, beta, convertase	CASP1	1.520290501	2.868488037	-0.141412665	0.906630962	0.001352279	3.163898167
NM_004346	caspase 3, apoptosis-related cysteine protease	CASP3 alpha	2.108696445	4.313014138	0.344936418	1.270095006	0.000414819	3.395820091
NM_033306	caspase 4, apoptosis-related cysteine protease	CASP4 gamma	1.637067011	3.11032861	0.557902349	1.472127216	6.18E-07	2.112812382
NM_002986	chemokine C-C motif ligand 11	CCL11	2.913221206	7.532982646	0.470493796	1.385583635	0.005543828	5.436685637
NM_005408	chemokine C-C motif ligand 13	CCL13	1.859546377	3.628935406	0.075548268	1.053761421	0.013087537	3.443792241
NM_032965	chemokine C-C motif ligand 15	CCL15 3	1.371131767	2.58673411	0.525954622	1.439886028	0.032332453	1.796485318
NM_002988	chemokine C-C motif ligand 18 pulmonary and activation-regulated	CCL18	1.725592142	3.307158389	-1.618649636	0.325640121	0.000539855	10.15586894
NM_006274	chemokine C-C motif ligand 19	CCL19	2.760608151	6.776818583	0.016779913	1.011698852	0.002363396	6.698454359
NM_004591	chemokine C-C motif ligand 20	CCL20	2.955656604	7.757848507	-0.364812585	0.77656975	0.013194304	9.989892744
NM_002984	chemokine C-C motif ligand 4	CCL4	1.093116219	2.13334341	0.347038338	1.271946807	0.016948495	1.677226908
NM_001237	cyclin A2	CCNA2	2.957839402	7.76959502	-0.785955718	0.579967629	0.018972502	13.39660118
NM_001238	cyclin E1	CCNE1 1	2.830698628	7.114185673	0.073325337	1.052139016	0.000694619	6.761640394
NM_001761	cyclin F	CCNF	2.465368639	5.522680393	-0.035379857	0.975774809	0.007746682	5.659789887
NM_004060	cyclin G1	CCNG1 1	1.493799035	2.816296122	-0.461685693	0.72613732	0.002110183	3.878462167
NM_001239	cyclin H	CCNH	1.335799484	2.524153215	0.251935712	1.190803784	0.039026231	2.119705402
NM_001295	chemokine C-C motif receptor 1	CCR1	3.47801415	11.14260113	0.052278973	1.036901586	0.004645295	10.74605466
NM_003965	chemokine C-C motif receptor-like 2	CCRL2	1.797143376	3.475314102	-0.382374554	0.767173847	0.002176379	4.530021603
NM_000591	CD14 antigen	CD14	2.255046293	4.773496142	-0.29645093	0.814253019	2.58E-06	5.86242363
CK231776		CD164	2.745458356	6.7060273	-0.540618748	0.687475999	0.040823523	9.75456207
XR_010680	CD180 antigen	CD180	1.790098237	3.458384408	-0.767665445	0.587367178	0.016636933	5.887942905
NM_001767	CD2 antigen p50, sheep red blood cell receptor	CD2	1.04076525	2.05731863	0.219554495	1.164373972	0.001972864	1.766888199
NM_001004196	CD200 molecule	CD200 2	1.27419931	2.418645468	0.048488816	1.03418108	0.017932406	2.338705973
NM_014143	CD274 antigen	CD274	4.259645799	19.15495589	0.217138612	1.162425786	0.034456812	16.47843339
NM_012120	CD2-associated protein	CD2AP	1.251577953	2.381017052	-0.163786998	0.892678758	0.005937965	2.667271994
NM_007261	CD300A antigen	CD300A	2.149207876	4.435841682	-0.532246495	0.691477161	0.026136407	6.415022699
NM_016579	CD320 antigen	CD320	2.114837377	4.33141191	-0.524623543	0.695140476	0.000797085	6.23098792
CO726190		CD36	1.534224935	2.896327884	-0.348846838	0.785211475	0.00062031	3.688595972
NM_000732	CD3D antigen, delta polypeptide TiT3 complex	CD3D	1.163413562	2.239867764	-0.026020801	0.982125434	0.003688164	2.28063309
NM_001250	CD40 antigen TNF receptor superfamily member 5	CD40 1	2.936583526	7.655961242	0.14613756	1.106602862	3.69E-05	6.918436147
CO646433		CD44	2.02666991	4.074632401	-0.408358819	0.75348003	0.000266422	5.40775102
NM_001778	CD48 antigen B-cell membrane protein	CD48	2.034793633	4.097641113	-0.344161612	0.787765635	0.000725688	5.201599223
NM_000560	CD53 antigen	CD53	2.744784076	6.702893801	0.576765689	1.491501769	0.00281706	4.494056889
NM_001780	CD63 antigen melanoma 1 antigen	CD63	1.845251306	3.593155335	0.46917018	1.384313	3.11E-05	2.595623487
NM_001251	CD68 antigen	CD68	2.136591974	4.397220789	0.082854039	1.059111176	0.00056472	4.151802839
NM_004355	CD74 antigen invariant polypeptide of major histocompatibility complex, class II antigen-associated	CD74 2	2.382715246	5.215173488	-0.852851908	0.553689125	0.000112013	9.418955966

NM_004233	CD83 antigen activated B lymphocytes, immunoglobulin superfamily	CD83	2.22013954	4.659384989	0.54294723	1.456945822	0.001724348	3.198049591
NM_006889	CD86 antigen CD28 antigen ligand 2, B7-2 antigen	CD86 2	4.522711269	22.98644202	-0.289008972	0.818464092	0.010466534	28.08485095
NM_001768	CD8 antigen, alpha polypeptide p32	CD8A 1	1.472001877	2.774065548	-0.633968067	0.644401579	0.00148231	4.304870814
NM_001785	cytidine deaminase	CDA	1.69695823	3.242166608	-0.324040038	0.798829747	0.009434636	4.058645312
NM_020239	CDC42 small effector 1	CDC42SE1	1.50014904	2.828719335	-0.44412782	0.735028543	0.019714123	3.848448282
NM_152562	cell division cycle associated 2	CDCA2	2.053736128	4.15179764	-0.037729961	0.974186597	0.044242648	4.261809447
NM_031299	cell division cycle associated 3	CDCA3	2.412489772	5.323923244	0.527697449	1.441626513	0.002092662	3.692997594
NM_080668	cell division cycle associated 5	CDCA5	3.759730781	13.54539708	0.154205305	1.112808469	0.006965934	12.17226275
NM_018101	cell division cycle associated 8	CDCA8	1.386766353	2.614919173	-0.258126037	0.836173347	0.024086987	3.127245305
NM_004642	CDK2-associated protein 1	CDK2AP1	1.776005541	3.424766292	-0.932259497	0.524036971	0.003236005	6.535352429
NM_000389	cyclin-dependent kinase inhibitor 1A p21, Cip1	CDKN1A 1	3.063640355	8.360796303	-0.489136237	0.712451525	0.004658611	11.73524935
NM_005192	cyclin-dependent kinase inhibitor 3 CDK2-associated dual specificity phosphatase	CDKN3	3.354859521	10.23088839	-0.1956331	0.873189629	0.047447222	11.71668565
NM_001803	CDW52 antigen CAMPATH-1 antigen	CDW52	1.119273423	2.172375388	-0.328023937	0.796626879	0.000114041	2.726967223
NM_080546	CDW92 antigen	CDW92	1.92676606	3.802019839	-0.029344242	0.979865581	0.000103291	3.880144288
NM_004824	chromodomain protein, Y-like	CDYL 1	1.389037176	2.619038334	-0.20660532	0.866573894	0.004500598	3.022290832
NM_005194	CCAAT/enhancer binding protein C/EBP, beta	CEBPB	1.739765377	3.339808486	0.126646796	1.091753227	3.63E-05	3.059123987
NM_005195	CCAAT/enhancer binding protein C/EBP, delta	CEBPD	2.01311224	4.036520559	-0.936226711	0.52259792	9.92E-06	7.723950672
NM_016343	centromere protein F, 350/400ka mitosin	CENPF	1.490106453	2.809097021	-1.137021204	0.454697443	0.018442477	6.177947698
NM_018404	centaurin, alpha 2	CENTA2	3.336181811	10.0992889	0.119353997	1.086248358	0.000609849	9.297403147
NM_005507	cofilin 1	CFL1	1.316383294	2.490410033	-0.662467074	0.631796971	5.33E-05	3.941788498
NM_016056	CGI-119 protein	CGI-119	1.742465471	3.346065	0.255187661	1.193490978	0.018410192	2.803594717
NM_016033	CGI-90 protein	CGI-90	1.3608161	2.568304215	-0.224291984	0.85601502	0.029300416	3.000302747
NM_015703	CGI-96 protein	CGI-96	1.677526563	3.198790621	0.241527738	1.182243933	0.017144557	2.705694258
NM_203298	coiled-coil-helix-coiled-coil-helix domain containing 1	CHCHD1	1.442330207	2.717594506	-0.156136074	0.897425402	0.00855096	3.02821215
NM_016139	coiled-coil-helix-coiled-coil-helix domain containing 2	CHCHD2	2.06572616	4.186446418	-0.296248521	0.814367267	0.003377886	5.140735133
XR_010663	Mitochondrial intermembrane space import and assembly protein 40 Coiled-coil-helix-coiled-coil-helix domain-containing protein 4	CHCHD4	1.71805644	3.289928979	0.147217798	1.107431757	0.01555973	2.970773557
NM_001274	CHK1 checkpoint homolog	CHEK1	1.667957782	3.177644611	0.128477944	1.09313982	0.048912363	2.906896769
NM_004000	chitinase 3-like 2	CHI3L2	1.640067547	3.116804244	-0.448405372	0.732852431	0.012009616	4.252976605
NM_020412	chromatin modifying protein 1B	CHMP1B	3.623000773	12.32060139	-0.141856118	0.906352326	0.002296701	13.59361149
NM_176812	chromatin modifying protein 4B	CHMP4B	1.289195508	2.443917371	-0.192294491	0.875212658	0.002614863	2.792369772
NM_007236	calcium binding protein P22	CHP	1.204118499	2.303964518	0.10517358	1.075623798	0.000872801	2.141979866
NM_018413	carbohydrate chondroitin 4 sulfotransferase 11	CHST11	1.962802687	3.898185344	0.267549436	1.203761379	0.011088048	3.238337275
NM_014918	carbohydrate chondroitin synthase 1	CHSY1	2.495871566	5.640689665	-0.638288872	0.642474513	0.049118602	8.779631801
NM_004895	cold autoinflammatory syndrome 1	CIAS1 1	1.000319221	2.000442583	0.200172078	1.148835375	0.003389762	1.741278713
NM_006384	calcium and integrin binding 1 calmyrin	CIB1	1.163496462	2.239996475	-0.192219081	0.875258407	0.002723452	2.559240171
NM_133467	Cbp/p300-interacting transactivator, with Glu/Asp-rich carboxy-terminal domain, 4	CITED4	2.328792935	5.023848419	-0.175770262	0.88529473	0.010268502	5.674775021
NM_020990	creatine kinase, mitochondrial 1	CKMT1	1.21814486	2.326473671	-0.160457604	0.894741226	0.000640187	2.600163716
NM_001827	CDC28 protein kinase regulatory subunit 2	CKS2	2.520668502	5.738479416	-0.176537761	0.884823887	0.002058192	6.48544812
NM_001829	chloride channel 3	CLCN3	1.529709068	2.887276087	-1.252121156	0.419830488	0.025556906	6.877242526
NM_001002026	claudin 18	CLDN18 2	1.572115288	2.973403569	-0.451434053	0.731315552	0.002331441	4.065828442

NM_016511	C-type lectin-like receptor-1	CLEC1	2.220111198	4.659293456	0.260349897	1.197769165	0.000666458	3.889976126
NM_022570	C-type lectin domain family 7, member A	CLEC7A 2	2.579161947	5.975924607	-0.016521816	0.988613275	0.00915545	6.044754564
NM_016184	C-type calcium dependent, carbohydrate-recognition domain lectin, superfamily member 6	CLECSF6 1	2.120801349	4.349354641	0.330705684	1.257628384	0.027098781	3.458378243
NM_001288	chloride intracellular channel 1	CLIC1	1.740187094	3.340784895	-0.413200646	0.750955515	3.69E-05	4.44871211
NM_001289	chloride intracellular channel 2	CLIC2	2.412153586	5.322682775	0.335748126	1.26203168	0.001115158	4.217550841
NM_024734	calmin calponin-like, transmembrane	CLMN	1.304705128	2.470332312	0.466452855	1.381708091	0.041849532	1.787882931
NM_000391	ceroid-lipofuscinosis, neuronal 2, late infantile	CLN2	2.125102119	4.362339701	-0.135963334	0.910061953	1.29E-05	4.793453554
XR_013508	ClpX caseinolytic protease X homolog	CLPX	1.482010762	2.793377902	-0.335086863	0.792736405	0.008849011	3.523715933
NM_004859	clathrin, heavy polypeptide	CLTC	1.4699143	2.770054382	0.251406517	1.190367066	0.01028106	2.32705899
NM_018686	cytidine monophosphate N-acetylneuraminic acid synthetase	CMAS	1.392671854	2.625644971	-0.043938315	0.970003384	0.018747756	2.706841042
NM_198390	c-Maf-inducing protein	CMIP	1.083661102	2.119407646	-0.583970115	0.667125404	0.000359856	3.176925409
NM_018235	CNDP dipeptidase 2 metallopeptidase M20 family	CNDP2	1.917621583	3.77799707	0.301547673	1.232465851	9.22E-07	3.065396958
NM_005776	cornichon homolog	CNIH	2.618506343	6.141139364	-0.386941264	0.764749273	0.016757147	8.030265054
NM_000088	collagen, type I, alpha 1	COL1A1	3.302823727	9.868451524	-0.537809617	0.688815916	0.006613161	14.32668918
NM_000089	collagen, type I, alpha 2	COL1A2	1.193489729	2.287052889	-0.983331831	0.505810247	0.015890063	4.521562983
NM_000090	collagen, type III, alpha 1	COL3A1	2.165693284	4.486819925	-0.170141197	0.888755694	7.74E-06	5.048428894
CN644408		COL4A1	3.123957114	8.717757749	-0.679430889	0.624411542	0.000624487	13.96155766
XR_012719	alpha 2 type V collagen	COL5A2	1.354665125	2.557377482	-0.32267611	0.79958532	0.005942753	3.198379734
NM_004369	collagen, type VI, alpha 3	COL6A3 1	1.947607882	3.857344188	-0.237704083	0.848093903	0.0002379	4.54825129
NM_000094	collagen, type VII, alpha 1	COL7A1	1.421538418	2.67871003	-0.022974434	0.984201463	0.004858723	2.721709052
NM_024027	collectin sub-family member 11	COLEC11 1	2.142631414	4.41566711	0.050633859	1.035719876	0.000483312	4.263379717
NM_181789	collomin	COLM	1.089517854	2.128029062	-0.104992952	0.929809485	0.002021828	2.288672139
CO647278		COMT	1.698709002	3.246103505	-0.039943581	0.972692985	1.78E-05	3.337233387
NM_004766	coatomer protein complex, subunit beta 2	COPB2	1.808142936	3.501912256	0.194403432	1.144250904	0.002161345	3.060440891
NM_052889	CARD only protein	COPI 2	2.436321811	5.412600137	0.189194641	1.140127082	0.000147525	4.747365642
NM_016129	COP9 constitutive photomorphogenic homolog subunit 4	COPS4	1.257594017	2.390966667	-1.658292908	0.316813802	0.012357616	7.546914475
XR_013997	Ubiquinone biosynthesis protein COQ4 homolog Coenzyme Q biosynthesis protein 4 homolog	COQ4	1.096132968	2.137809007	-0.748975951	0.595025767	0.010566641	3.592800727
NM_021149	coactosin-like 1	COTL1	1.115578962	2.166819473	0.463126784	1.378526292	0.007039123	1.571837611
NM_004255	cytochrome c oxidase subunit Va	COX5A	1.612258147	3.057300047	-0.028416889	0.980495633	0.000139098	3.118116943
NM_004074	cytochrome c oxidase subunit 8A	COX8A	2.26417137	4.803784291	-0.462319234	0.725818516	2.55E-05	6.618437236
CN642215		CPE	1.450584119	2.733186904	0.138806744	1.100994105	0.006850298	2.482471879
NM_000097	coproporphyrinogen oxidase	CPOX	3.101350837	8.582219721	-0.331743759	0.794575514	0.032762013	10.80101207
NM_031311	carboxypeptidase, vitellogenic-like	CPVL 1	1.360137547	2.567096532	-0.472847985	0.720540793	0.000565854	3.562735875
NM_003652	carboxypeptidase Z	CPZ	2.654252917	6.295203071	0.131426973	1.095376605	0.006884586	5.747067301
NM_006368	cAMP responsive element binding protein 3	CREB3	1.088133154	2.125987558	0.334051571	1.26054845	0.004977449	1.686557591
NM_194071	cAMP responsive element binding protein 3-like 2	CREB3L2	1.236678917	2.356554288	-0.118044465	0.921435786	0.002246229	2.557480752
NM_182898	cAMP responsive element binding protein 5	CREB5 1	1.876753684	3.672477581	-0.407234132	0.754067652	0.025191801	4.870222944
NM_003851	cellular repressor of E1A-stimulated genes	CREG	2.527798712	5.766910818	-1.173650658	0.443298179	7.92E-05	13.00910107
NM_031476	cysteine-rich secretory protein LCCL domain containing 2	CRISPLD2	1.208629984	2.311180576	-0.223321335	0.856591143	0.000523202	2.698114025
NM_016507	CDC2-related protein kinase 7	CRK7	1.057407739	2.081188642	-0.498748353	0.707720516	0.008620822	2.940692824
NM_030782	cisplatin resistance related protein CRR9p	CRR9	1.652459373	3.143690899	-0.273165329	0.827501981	0.002618541	3.799013138

NM_005211	colony stimulating factor 1 receptor, formerly McDonough feline sarcoma viral	CSF1R	1.643109401	3.12338281	-0.315799816	0.803405467	0.003155701	3.887679307
NM_001892	casein kinase 1, alpha 1	CSNK1A1	1.514700371	2.857394773	-0.287194211	0.819494282	0.002594736	3.486778169
NM_004385	chondroitin sulfate proteoglycan 2	CSPG2	2.039267663	4.110368283	-0.371028127	0.773231262	8.97E-05	5.315833027
XR_010293	chondroitin sulfate proteoglycan 6	CSPG6	1.421739005	2.679082494	-0.386229118	0.765126863	0.000335655	3.501487952
NM_000100	cystatin B	CSTB	2.001198766	4.003325067	-0.266424441	0.831377471	1.43E-07	4.815291738
NM_001324	cleavage stimulation factor, 3' pre-RNA, subunit 1, 50kDa	CSTF1	2.174146425	4.513186587	-1.422550173	0.373052305	0.003216721	12.09799947
NM_138455	collagen triple helix repeat containing 1	CTHRC1	5.451666193	43.76380259				
NM_001903	catenin cadherin-associated protein, alpha 1, 102kDa	CTNNA1	1.160765271	2.23575991	-0.143269495	0.905464827	0.000517822	2.469184715
NM_001906	chymotrypsinogen B1	CTRB1	2.230760319	4.693812847	0.233162818	1.175408974	0.005249368	3.993344402
NM_147780	cathepsin B	CTSB 2	3.148726551	8.86872401	-0.214127652	0.862067264	1.07E-06	10.28774015
NM_001814	cathepsin C	CTSC 1	3.389958225	10.48284369	-0.254870468	0.838062376	5.56E-06	12.50842894
NM_148170	cathepsin C	CTSC 2	3.855508101	14.47516713	0.134205617	1.097488343	6.89E-05	13.18935843
CO646399		CTSD	1.815809262	3.52057057	-0.657784909	0.633850754	1.90E-06	5.554257915
NM_148979	cathepsin H	CTSH 2	1.648921861	3.135991961	-0.164658281	0.892139808	8.71E-06	3.515135108
NM_004079	cathepsin S	CTSS	3.177530717	9.047572212	0.733821304	1.663038196	0.027078539	5.440387499
XR_009750	Cathepsin Z precursor	CTSX	2.856521872	7.242671149	-0.4684181	0.722756659	0.000637439	10.02089855
NM_003592	cullin 1	CUL1	1.967955588	3.912133462	-0.40999121	0.752627959	0.033686849	5.197964563
NM_001511	chemokine C-X-C motif ligand 1 melanoma growth stimulating activity, alpha	CXCL1	2.487423237	5.607754676	0.201269525	1.149709617	0.038256215	4.877540025
NM_001565	chemokine C-X-C motif ligand 10	CXCL10	6.542988477	93.24719849	-0.146364109	0.903524666	0.001053314	103.2038217
CO646675		CXCL11	6.117735694	69.44195668	-1.045107246	0.484608883	0.000664625	143.294849
NM_000609	chemokine C-X-C motif ligand 12 stromal cell-derived factor 1	CXCL12	1.542532755	2.913054625	-1.24011637	0.423338508	0.042914655	6.881147293
NM_022059	chemokine C-X-C motif ligand 16	CXCL16	1.592034105	3.014741087	-0.060667777	0.95882021	0.021417062	3.144219382
CO647386		CXCL2	4.037359278	16.41973887	-1.051601838	0.482432218	0.02892919	34.0353282
NM_002090	chemokine C-X-C motif ligand 3	CXCL3	3.401071675	10.56390755	0.834011506	1.782635208	0.010544879	5.92600634
NM_002993	chemokine C-X-C motif ligand 6 granulocyte chemotactic protein 2	CXCL6	6.23390739	75.26500844	0.29144375	1.223864424	0.000302689	61.49783174
NM_002416	chemokine C-X-C motif ligand 9	CXCL9	4.349936749	20.39207598	0.077041534	1.054852685	0.000157812	19.33168136
NM_003467	chemokine C-X-C motif receptor 4	CXCR4	2.705911947	6.524701706	0.071557706	1.050850695	0.000837014	6.208971205
NM_007022	cytochrome b-561 domain containing 2	CYB561D2	1.874664528	3.667163344	-0.06047819	0.958946218	0.001299008	3.824159555
NM_000101	cytochrome b-245, alpha polypeptide	CYBA	2.415986181	5.33684156	-0.469604595	0.722162497	4.09E-06	7.390084067
NM_000397	cytochrome b-245, beta polypeptide chronic granulomatous disease	CYBB	3.55902919	11.78621995	-0.439785649	0.737244138	0.000391235	15.98686154
NM_018947	cytochrome c, somatic	CYCS	1.510401604	2.848893329	-0.268376482	0.830253335	0.000120759	3.431354273
NM_000104	cytochrome P450, family 1, subfamily B, polypeptide 1	CYP1B1	5.728635799	53.02628597	-0.16596459	0.891332373	0.024713356	59.49103567
NM_000772	cytochrome P450, family 2, subfamily C, polypeptide 18	CYP2C18	1.956875856	3.882203802	-0.206808243	0.866452014	0.025567044	4.480575656
AY635466	cytochrome P450 CYP3A66	CYP3A66	2.090582707	4.25920068	0.278353094	1.212809613	0.01838601	3.511846076
NM_004394	death-associated protein	DAP	1.071586963	2.101744013	-0.356083858	0.781282466	0.034090387	2.690120544
NM_018838	13kDa differentiation-associated protein	DAP13	1.970388799	3.918737127	0.073910168	1.052565613	0.004569181	3.723033585
NM_014395	dual adaptor of phosphotyrosine and 3-phosphoinositides	DAPP1	2.231677606	4.696798191	-0.124104684	0.9175733	0.017164131	5.118717156
NM_020548	diazepam binding inhibitor GABA receptor modulator, acyl-Coenzyme A binding protein	DBI	1.959554541	3.889418674	0.242946523	1.183407155	0.000240143	3.286627646

NM_020188	DC13 protein	DC13	1.250208272	2.37875761	0.246347096	1.186199851	0.001146148	2.005359896
NM_021227	DC2 protein	DC2	1.768665568	3.407386421	-0.66911115	0.628894032	0.017717851	5.418061309
NM_001919	dodecenoyl-Coenzyme A delta isomerase 3,2 trans-enoyl-Coenzyme A isomerase	DCI	1.797850387	3.477017642	-0.378606785	0.769180032	0.000181679	4.520421097
NM_005216	dolichyl-diphosphooligosaccharide-protein glycosyltransferase	DDOST	1.659596085	3.159280611	0.205843695	1.15336064	0.000292895	2.739195791
NM_001356	DEAD Asp-Glu-Ala-Asp box polypeptide 3, X-linked	DDX3X 2	1.106759537	2.153613766	-0.008462372	0.9941515	0.003086815	2.166283273
CN803456		DEGS	1.715478597	3.284055701	0.401774567	1.321131952	0.03600994	2.485789323
NM_015954	2-deoxyribose-5-phosphate aldolase homolog	DERA	1.236735828	2.356647251	0.41080909	1.329431175	0.014498573	1.772673378
NM_016041	Der1-like domain family, member 2	DERL2	1.537322213	2.902552603	0.09566429	1.068557313	0.000334266	2.716328425
NM_004753	dehydrogenase/reductase SDR family member 3	DHRS3	1.367432338	2.580109576	-0.360240307	0.779034807	0.000136084	3.311931064
NM_024612	DEAH Asp-Glu-Ala-His box polypeptide 40	DHX40	1.221162263	2.331344593	-0.455763911	0.729123997	0.011537888	3.197459696
NM_005219	diaphanous homolog 1	DIAPH1	1.188867775	2.279737595	-0.532846246	0.691189762	0.012958022	3.298280327
CN804369		DKC1	1.310102085	2.479590849	-0.317385436	0.802522955	0.008334738	3.089744453
NM_144566	hypothetical protein DKFZp434I1610	DKFZp434I1610	1.461648667	2.754229287	-0.005737079	0.996031256	0.029500506	2.765203672
NM_016613	hypothetical protein DKFZp434L142	DKFZp434L142	1.65206689	3.142835779	-0.238262398	0.847765759	0.000842276	3.7071983
CK231268		DKFZP564B167	1.019314547	2.026955684	0.056151736	1.039688779	0.001298538	1.949579263
NM_015544	DKFZP564K1964 protein	DKFZP564K1964	2.234290805	4.70531336	-0.851996129	0.554017661	0.005043411	8.49307467
NM_015393	DKFZP564O0823 protein	DKFZP564O0823	2.784779633	6.891316571	-0.265130775	0.832123303	0.001331453	8.281605082
NM_030800	hypothetical protein DKFZp564O1664	DKFZP564O1664	1.489411393	2.807743983	0.043199127	1.030396164	0.012029804	2.724916961
CN802671		DKFZp667B1218	2.019678868	4.054935224	-0.398303572	0.758749954	0.01173733	5.344231264
AL832403	mRNA; cDNA DKFZp667B1913	DKFZp667B1913	3.34594476	10.16786418	-0.228009155	0.853812296	0.000213152	11.90878162
NM_019045	rab11-binding protein	DKFZp686L20145	1.314795928	2.487671396	-1.055811941	0.481026426	6.72E-05	5.171589876
NM_018410	hypothetical protein DKFZp762E1312	DKFZp762E1312	2.285435206	4.875111469	-0.035367037	0.975783479	0.023253355	4.996099618
NM_080677	dynein light chain 2	Dlc2	1.520265549	2.868438426	0.357083845	1.280834297	0.016654947	2.239507821
NM_000108	dihydrolipoamide dehydrogenase E3 component of pyruvate dehydrogenase complex, 2-oxo-glutarate complex, branched chain keto acid dehydrogenase complex	DLD	1.023228358	2.03246197	-1.648673325	0.318933307	0.015095927	6.372686469
NM_001539	DnaJ Hsp40 homolog, subfamily A, member 1	DNAJA1	1.778749032	3.431285171	-0.199073628	0.871109734	0.000269114	3.938981551
NM_006145	DnaJ Hsp40 homolog, subfamily B, member 1	DNAJB1	1.29438274	2.452720329	-0.07770134	0.947566211	0.000274232	2.588442159
NM_016306	DnaJ Hsp40 homolog, subfamily B, member 11	DNAJB11	1.284032272	2.435186509	0.568728742	1.48321603	0.014394127	1.641828607
NM_173823	DnaJ Hsp40 homolog, subfamily C, member 13	DNAJC13	1.138710832	2.201841823	-0.553204003	0.681504929	0.002457675	3.230852381
NM_032364	DnaJ Hsp40 homolog, subfamily C, member 14	DNAJC14	1.068262162	2.09690596	-0.076788521	0.948165943	0.000441509	2.211538998
NM_006260	DnaJ Hsp40 homolog, subfamily C, member 3	DNAJC3	1.475514251	2.780827494	-0.005721925	0.996041718	0.005181479	2.791878536
NM_001376	dynein, cytoplasmic, heavy polypeptide 1	DNCH1	1.175055705	2.258015994	-0.031708654	0.978261009	0.000476677	2.308193798

NM_001005360	dynamin 2	DNM2 1	1.341342688	2.533870314	-0.230496626	0.852341435	0.003423088	2.97283484
NM_001379	DNA cytosine-5--methyltransferase 1	DNMT1	1.120174837	2.173733138	-0.17980159	0.882824401	0.000576197	2.462248593
NM_014890	downregulated in ovarian cancer 1	DOC1 2	3.149138632	8.87125757	-0.506341295	0.704005549	0.029318967	12.60111882
CN646906		DOCK1	1.174264663	2.256778244	-0.093128636	0.937487503	0.004658812	2.407262216
XR_013749	dedicator of cytokinesis 10	DOCK10	1.383629711	2.609240109	-0.167430312	0.890427273	0.011911202	2.930323663
NM_004946	dedicator of cytokinesis 2	DOCK2	2.045558034	4.128329245	-0.211183789	0.863828135	0.044343278	4.779109499
NM_003859	dolichyl-phosphate mannosyltransferase polypeptide 1, catalytic subunit	DPM1	1.442817857	2.718513245	-0.125044643	0.916975668	0.01555028	2.964651452
NM_003863	dolichyl-phosphate mannosyltransferase polypeptide 2, regulatory subunit	DPM2 1	1.43599647	2.705689842	-0.430320741	0.742096784	0.000320432	3.646006695
NM_152690	dolichyl-phosphate mannosyltransferase polypeptide 2, regulatory subunit	DPM2 2	1.278759671	2.426302905	-0.159440747	0.89537209	0.006267234	2.709826375
NM_018973	dolichyl-phosphate mannosyltransferase polypeptide 3	DPM3 1	1.339008694	2.529774329	-0.673945763	0.626790073	0.001550335	4.036079122
NM_005700	dipeptidylpeptidase 3	DPP3 1	1.526832863	2.881525652	-0.167597257	0.890324242	0.000317307	3.236490165
NM_000110	dihydropyrimidine dehydrogenase	DPYD	1.41633613	2.669068127	0.081749172	1.058300382	0.005558359	2.52203266
NM_016025	DORA reverse strand protein 1	DREV1	1.025452762	2.035598117	-0.944867585	0.519477229	0.026553819	3.918551199
NM_004416	deltex homolog 1	DTX1	1.640006326	3.116671985	0.146017325	1.106510641	0.045967045	2.816666981
NM_004418	dual specificity phosphatase 2	DUSP2	1.008397554	2.011675429	0.484475078	1.399076721	0.01330621	1.437859267
NM_006014	DNA segment on chromosome X	DXS9879E	2.450205942	5.464941083	-0.183379277	0.880637829	0.005504408	6.205662425
NM_017653	dymeclin	DYM	1.375133894	2.593919835	-0.005346424	0.996301	0.007093594	2.603550368
NM_001953	endothelial cell growth factor 1	ECGF1	2.002557036	4.007095895	0.71121835	1.63718613	8.77E-05	2.4475506
NM_001398	enoyl Coenzyme A hydratase 1, peroxisomal	ECH1	1.891158595	3.709329928	-0.660596672	0.632616604	0.001828586	5.863472286
NM_004092	enoyl Coenzyme A hydratase, short chain, 1, mitochondrial	ECHS1	1.466186157	2.762905382	-0.102955482	0.931123552	0.000140948	2.96728117
NM_001955	endothelin 1	EDN1	4.375792779	20.76083808	-0.303943588	0.810035147	0.001575852	25.6295522
NM_024329	EF hand domain containing 2	EFHD2	1.924768279	3.796758612	-1.039834348	0.486383318	0.001369776	7.806103691
NM_024580	elongation factor Tu GTP binding domain containing 1	EFTUD1	1.533997031	2.895870385	0.079581489	1.056711455	0.032383744	2.740455183
NM_003908	eukaryotic translation initiation factor 2, subunit 2 beta, 38kDa	EIF2S2	1.333697124	2.520477584	0.100164976	1.07189603	0.003380156	2.351419834
NM_003757	eukaryotic translation initiation factor 3, subunit 2 beta, 36kDa	EIF3S2	2.220847758	4.661672838	-1.182875802	0.440472605	0.048848345	10.58334341
NM_001416	eukaryotic translation initiation factor 4A, isoform 1	EIF4A1	1.601136991	3.033823154	-0.346409414	0.786539206	1.03E-05	3.857179818
NM_004096	eukaryotic translation initiation factor 4E binding protein 2	EIF4EBP2	1.477899349	2.785428626	-0.4376904	0.738315627	0.011374347	3.772680035
NM_001418	eukaryotic translation initiation factor 4 gamma, 2	EIF4G2	1.010842275	2.015087206	-0.175670824	0.885355751	0.000941648	2.276019785
NM_012081	elongation factor, RNA polymerase II, 2	ELL2	3.119940868	8.693522568	-0.586880975	0.665780732	0.014464731	13.05763616
NM_016242	endomucin	EMCN	2.665737586	6.345516445	0.137141682	1.099724144	0.042158626	5.770098328
XR_011598	Endoglin precursor CD105 antigen	ENG	1.262744614	2.399517965	0.019102873	1.013329154	0.014246635	2.367955126
NM_001428	enolase 1,	ENO1	2.449188359	5.461087827	-0.221411521	0.857725834	2.68E-05	6.366938724
NM_001975	enolase 2	ENO2	2.306654511	4.947345008	-0.519297385	0.697711547	0.000183283	7.090817156
NM_014936	ectonucleotide pyrophosphatase/phosphodiesterase 4	ENPP4	2.937426164	7.660434181	-0.185056159	0.879614835	0.022721405	8.708850597
NM_004436	endosulfine alpha	ENSA 3	1.430520656	2.695439738	-0.744480433	0.596882792	0.000706431	4.515861026
NM_001776	ectonucleoside triphosphate diphosphohydrolase 1	ENTPD1	1.90258602	3.738827784	0.056095436	1.039648207	0.000563402	3.596243191
NM_005239	v-ets erythroblastosis virus E26 oncogene homolog 2	ETS2	1.203139826	2.30240212	0.297496616	1.229009968	0.002672528	1.873379534
NM_016135	ets variant gene 7 TEL2 oncogene	ETV7	1.375782983	2.59508714	0.201154807	1.1496182	0.002137963	2.257346952

NM_001003927	ecotropic viral integration site 2A	EVI2A 1	2.378808674	5.201070791	0.381185693	1.302411815	0.016036045	3.993414932
NM_020158	exosome component 5	EXOSC5	1.624617718	3.083604457	-0.226209248	0.854878178	0.041293655	3.607068864
NM_001993	coagulation factor III thromboplastin, tissue factor	F3	1.11252839	2.162242585	0.337189084	1.263292822	0.013281944	1.711592552
NM_022763	FAD104	FAD104	1.360227085	2.567255857	-0.151088423	0.900570782	0.002501406	2.850698588
NM_031208	fumarylacetoacetate hydrolase domain containing 1	FAHD1	1.743689882	3.348906	-0.657597661	0.633933028	0.048002598	5.282744158
NM_020223	family with sequence similarity 20, member C	FAM20C	1.773054399	3.417767834	0.292365057	1.224646236	0.042811767	2.790820512
NM_182620	family with sequence similarity 33, member A	FAM33A	2.034833413	4.097754101	-0.200876232	0.870021988	0.007270232	4.709943146
NM_153690	family with sequence similarity 43, member A	FAM43A	1.491136181	2.811102737	-0.758522342	0.591101447	0.003256007	4.755702679
NM_017709	family with sequence similarity 46, member C	FAM46C	2.056073276	4.158528951	-0.467594078	0.723169592	0.001719688	5.750420089
NM_004460	fibroblast activation protein, alpha	FAP	1.229591475	2.345005773	0.157957729	1.115706632	0.005587426	2.101812167
NM_000043	Fas TNF receptor superfamily, member 6	FAS 1	1.302848973	2.467156054	-0.575660778	0.670978862	0.002099143	3.67695049
NM_004104	fatty acid synthase	FASN	1.796166827	3.472962486	-0.17118865	0.888110657	0.003630166	3.910506487
NM_001004019	fibulin 2	FBLN2 1	3.186016947	9.100948731	0.528141499	1.442070302	0.024625052	6.311029855
NM_015176	F-box protein 28	FBXO28	1.949425722	3.862207622	-0.017624632	0.987857854	0.00358081	3.909679522
NM_024735	F-box protein 31	FBXO31	1.281489633	2.430898461	-0.029071928	0.980050552	0.011348264	2.480380686
NM_012176	F-box protein 4	FBXO4 1	1.62588897	3.086322816	-0.064401078	0.956342252	0.004314797	3.22721579
NM_018438	F-box only protein 6	FBXO6	1.363785073	2.573595061	0.562306017	1.476627587	0.020517454	1.742887024
NM_012179	F-box protein 7	FBXO7	1.059722391	2.08453037	-0.514327201	0.700119356	0.000701249	2.977392856
NM_012180	F-box protein 8	FBXO8	1.239274114	2.360797199	-0.456697592	0.728652276	0.003540538	3.239950352
NM_021642	Fc fragment of IgG, low affinity IIa, receptor CD32	FCGR2A	1.462925394	2.756667748	-3.322570867	0.099955456	0.02975195	27.57896215
NM_004001	Fc fragment of IgG, low affinity IIb, receptor for CD32	FCGR2B	1.480491412	2.790437653	-3.024929876	0.122858546	0.021356867	22.71260515
NM_000569	Fc fragment of IgG, low affinity IIIa, receptor for CD16	FCGR3A	2.12618675	4.365620579	-0.272670718	0.827785729	0.00052592	5.273853396
NM_000570	Fc fragment of IgG, low affinity IIIb, receptor CD16b	FCGR3B	3.316627913	9.963329343	-0.525374069	0.694778941	0.007370694	14.3402869
CK231677		FGF1	1.520157384	2.868223374	-0.71402827	0.609615601	0.000166991	4.704970424
NM_006682	fibrinogen-like 2	FGL2	2.215886925	4.645670796	0.060665963	1.042947085	0.025121999	4.454368648
NM_005248	Gardner-Rasheed feline sarcoma viral	FGR	1.364458743	2.57479709	-0.101314028	0.932183558	0.002102884	2.762113822
NM_001450	four and a half LIM domains 2	FHL2 1	2.319185441	4.990503716	-1.001149188	0.49960188	0.046219533	9.988961036
NM_004468	four and a half LIM domains 3	FHL3	1.189744287	2.281123074	-0.598094397	0.660625976	0.013101717	3.452972115
NM_021939	FK506 binding protein 10, 65 kDa	FKBP10	1.4331208	2.700302062	-0.344782839	0.787426495	0.009653603	3.429275088
NM_016594	FK506 binding protein 11, 19 kDa	FKBP11	1.332487176	2.518364617	-0.069965911	0.952660508	2.77E-05	2.64350689
NM_000801	FK506 binding protein 1A, 12kDa	FKBP1A	1.688086618	3.222290622	-1.335997286	0.396118151	0.005851426	8.134670465
NM_054033	FK506 binding protein 1B, 12.6 kDa	FKBP1B 2	1.180839623	2.267086791	-0.428230411	0.743172791	0.010790694	3.050551388
NM_004117	FK506 binding protein 5	FKBP5	2.975260981	7.863987239	0.092396139	1.066139439	0.000960525	7.376133879
XM_375853	protein BAP28	FLJ10359	1.88867262	3.702943713	-0.692280473	0.618874819	0.004499061	5.983348488
NM_018075	hypothetical protein FLJ10375	FLJ10375	1.355392937	2.558667954	-0.298760376	0.812950618	0.030781221	3.14738423
NM_018128	hypothetical protein FLJ10534	FLJ10534	1.619419483	3.072513784	-0.786079497	0.579917871	0.042077903	5.29818779
NM_018145	hypothetical protein FLJ10579	FLJ10579	1.8073414	3.499967195	-0.431152244	0.741669196	0.001576181	4.719040796
NM_018149	hypothetical protein FLJ10587	FLJ10587	1.465380586	2.761363063	-0.024191265	0.983371695	0.012471467	2.80805628
NM_018193	hypothetical protein FLJ10719	FLJ10719	1.730002498	3.317283927	-0.783957273	0.580771566	0.007940835	5.711856644
XM_371236	hypothetical protein FLJ10747	FLJ10747	3.131802785	8.765295844	-0.619725958	0.650794535	0.005134995	13.46860702
NM_018295	hypothetical protein FLJ11000	FLJ11000	2.476915882	5.567060963	-0.087112624	0.941404971	0.001598078	5.913566567
NM_018370	hypothetical protein FLJ11259	FLJ11259	1.93818973	3.832244821	-0.091576094	0.938496914	3.38E-05	4.083385639
NM_022831	hypothetical protein FLJ12806	FLJ12806	1.226077647	2.339301227	-0.163416034	0.892908325	0.000429706	2.619867193
NM_024598	hypothetical protein FLJ13154	FLJ13154	1.206376282	2.30757299	-0.081012371	0.94539401	0.008373966	2.440858483

NM_025147	hypothetical protein FLJ13448	FLJ13448	1.069815411	2.099164767	0.235428352	1.177256225	0.047769053	1.783099313
NM_025108	hypothetical protein FLJ13909	FLJ13909	4.19138272	18.26972128	-0.52379346	0.695540554	0.047607149	26.26693896
NM_032558	hypothetical protein FLJ14753	FLJ14753	1.554508327	2.937336047	-0.575348269	0.671124221	0.000951028	4.376739737
NM_017785	hypothetical protein FLJ20364	FLJ20364	2.678460053	6.401722111	-0.135161299	0.910568023	0.049656266	7.030471035
NM_017813	hypothetical protein FLJ20421	FLJ20421	2.113601182	4.327702066	0.152884719	1.111790314	0.000514247	3.892552409
NM_017832	hypothetical protein FLJ20457	FLJ20457	1.421546278	2.678724624	-0.387340233	0.764537815	0.006648425	3.503717632
NM_024660	hypothetical protein FLJ22573	FLJ22573	1.41525285	2.66706475	-0.017491818	0.9879488	0.000330301	2.699598147
NM_024829	hypothetical protein FLJ22662	FLJ22662	1.828547094	3.551791992	-0.100687425	0.932588519	0.000203167	3.808530684
NM_025130	hypothetical protein FLJ22761	FLJ22761	2.073142186	4.208021821	-1.016407952	0.494345652	0.015509925	8.512306731
NM_032231	hypothetical protein FLJ22875	FLJ22875	1.310252031	2.479848579	-0.292404942	0.816539769	0.029462212	3.037021188
NM_024579	hypothetical protein FLJ23221	FLJ23221	1.089495765	2.12799648	0.10241455	1.073568726	8.64E-05	1.982170707
NM_024956	hypothetical protein FLJ23375	FLJ23375	2.753774796	6.74479594	-0.159739512	0.895186688	0.012144046	7.534513225
NM_144595	hypothetical protein FLJ30046	FLJ30046	1.336225735	2.524899099	0.066584503	1.04723448	0.038124202	2.411016012
NM_152515	hypothetical protein FLJ40629	FLJ40629	1.914495941	3.769820786	0.236355826	1.178013299	0.005475751	3.200151297
CN804242		FLJ42280	1.389319758	2.619551378	-0.036041594	0.975327341	0.000373706	2.68581764
NM_198446	FLJ45459 protein	FLJ45459	1.140643028	2.204792717	-0.733075903	0.60161986	5.76E-05	3.664760532
NM_005803	flotillin 1	FLOT1	1.362975222	2.57215079	-0.615471091	0.652716724	0.0072773	3.940684674
NM_013281	fibronectin leucine rich transmembrane protein 3	FLRT3 1	1.796832079	3.474564299	-0.636700153	0.643182406	0.016816509	5.402144504
NM_052905	formin-like 2	FMNL2	1.249847496	2.378162827	0.23892072	1.180109492	0.029035987	2.015205236
NM_212482	fibronectin 1	FN1 1	1.819044633	3.528474621	-0.786041701	0.579933064	6.62E-06	6.084279101
NM_000803	folate receptor 2	FOLR2	1.428505534	2.69167744	-0.497298924	0.708431897	0.003380418	3.799486517
NM_000804	folate receptor 3	FOLR3	3.526804675	11.52587741	0.497389188	1.411656607	0.013453809	8.164788341
NM_002029	formyl peptide receptor 1	FPR1	1.409966921	2.6573107	-0.304661798	0.809631991	0.000604196	3.282121666
NM_002030	formyl peptide receptor-like 2	FPRL2	4.200518969	18.38578626	-0.094523478	0.936581549	0.017016736	19.63073721
NM_007085	follistatin-like 1	FSTL1	1.754582705	3.374287042	0.235707447	1.177483992	0.00021774	2.865675512
NM_005860	follistatin-like 3	FSTL3	1.154656693	2.226313394	0.026020146	1.018199418	0.001587131	2.186520003
NM_017647	FtsJ homolog 3	FTSJ3	1.150471873	2.219864893	-0.766107012	0.588002008	0.005895672	3.775267535
NM_000147	fucosidase, alpha-L- 1, tissue	FUCA1	1.251168715	2.380341744	-0.299715273	0.812412716	6.72E-05	2.929966131
NM_001465	FYN binding protein FYB-120/130	FYB	1.539533745	2.907005388	-0.086479829	0.941817981	0.008514103	3.086589391
NM_005754	Ras-GTPase-activating protein SH3-domain-binding protein	G3BP	1.931540841	3.814623948	0.019591692	1.013672552	0.015312994	3.763171785
NM_000402	glucose-6-phosphate dehydrogenase	G6PD	2.569777726	5.937179479	-0.261264881	0.834356078	0.003060136	7.1158821
NM_031412	GABA A receptor-associated protein like 1	GABARAPL1	1.087533764	2.125104467	0.287187874	1.220259407	0.032750296	1.741518611
NM_052850	growth arrest and DNA-damage-inducible, gamma interacting protein 1	GADD45GIP1	1.084218019	2.12022595	-0.732574391	0.601829032	0.001007912	3.522970539
NM_015973	galanin	GAL	5.104151384	34.39558251	-1.227437178	0.427075435	0.024137594	80.53748756
NM_015892	B cell RAG associated protein	GALNAC4S	2.25865923	4.785465374	-0.914103101	0.530673679	0.02978813	9.017717598
NM_018590	chondroitin sulfate GalNAcT-2	GALNACT-2	1.776574245	3.426116586	-0.621493993	0.649997469	0.025525586	5.270969114
NM_020474	UDP-N-acetyl-alpha-D-galactosamine:polypeptide N-acetylgalactosaminyltransferase 1 GalNAc-T1	GALNT1	1.425900157	2.686820902	0.189058709	1.140019664	0.015890248	2.356819788
NM_002046	glyceraldehyde-3-phosphate dehydrogenase	GAPDH	1.308957406	2.477624245	-0.301174256	0.811591547	0.00156243	3.052797008
NM_152237	growth arrest-specific 2 like 1	GAS2L1 3	1.466764362	2.764012926	-0.623818241	0.648951137	0.003307182	4.259200376
NM_002053	guanylate binding protein 1, interferon-inducible, 67kDa	GBP1	4.855856574	28.95732787	-0.677270983	0.625347069	0.000459544	46.30601037
NM_198460	guanylate binding protein family, member 6	GBP6	3.149834465	8.87553734	0.305820257	1.236121245	0.008537878	7.180151117
NM_000161	GTP cyclohydrolase 1 dopa-responsive dystonia	GCH1	4.8280056	28.40367304	-0.248047688	0.84203512	0.013740842	33.73217148

NM_004864	growth differentiation factor 15	GDF15	3.245104767	9.481430674	0.122227083	1.088413747	0.002371467	8.711237525
NM_001494	GDP dissociation inhibitor 2	GDI2	1.120263897	2.173867331	0.111781967	1.080562085	0.004127023	2.011793085
NM_015465	gem nuclear organelle associated protein 5	GEMIN5	1.620445845	3.074700409	-0.214075704	0.862098306	0.004451339	3.566531086
CN802386		GFPT1	2.252924002	4.766479204	-1.370775945	0.386683217	0.003964724	12.32657378
NM_005110	glutamine-fructose-6-phosphate transaminase 2	GFPT2	1.479375535	2.788280175	0.030114752	1.02109334	0.019299932	2.730680992
NM_015044	golgi associated, gamma adaptin ear containing, ARF binding protein 2	GGA2 1	1.152962403	2.223700364	-0.423450327	0.745639231	0.009160848	2.982273828
NM_130759	GTPase, IMAP family member 1	GIMAP1	3.355902595	10.23828804	-1.400333059	0.378841672	0.000522646	27.02524243
NM_015660	GTPase, IMAP family member 2	GIMAP2	2.713734759	6.560177083	-0.071879293	0.951397874	0.000204439	6.895303492
NM_018326	GTPase, IMAP family member 4	GIMAP4	2.302135332	4.931871913	0.091082423	1.065169056	0.007422517	4.630130669
NM_175571	GTPase, IMAP family member 8	GIMAP8	3.212566455	9.26998147	-1.398090463	0.379431021	0.034519761	24.43126936
NM_000165	gap junction protein, alpha 1, 43kDa connexin 43	GJA1	1.838203117	3.575644028	0.140836049	1.10254386	0.002107453	3.243085519
NM_000166	gap junction protein, beta 1, 32kDa connexin 32, Charcot-Marie-Tooth neuropathy, X-linked	GJB1	1.35385838	2.555947813	0.142242809	1.103619466	0.000870956	2.315968403
NM_203391	glycerol kinase	GK 1	1.524123505	2.876119272	-1.282529774	0.411074055	0.01059254	6.996596436
NM_000169	galactosidase, alpha	GLA	2.174709639	4.514948835	0.098402279	1.070587178	0.000199866	4.217264068
CK230409		GLRX	2.847659726	7.198317443	-0.056917804	0.961315697	1.35E-05	7.487984924
NM_002065	glutamate-ammonia ligase glutamine synthase	GLUL	2.936904605	7.657665301	-0.896937026	0.537025677	0.008106986	14.25940254
XR_010040	Glycyl-tRNA synthetase	GlyRS	1.781791211	3.43852828	-0.873140162	0.545957229	1.56E-05	6.298164211
NM_000405	GM2 ganglioside activator	GM2A	1.409964543	2.657306319	0.171923245	1.126559291	0.000105859	2.358780705
NM_015895	geminin, DNA replication inhibitor	GMNN	1.084472836	2.120600469	-0.151643667	0.90022425	0.000216908	2.355635799
NM_013334	GDP-mannose pyrophosphorylase B	GMPPB 1	1.260117915	2.395153164	-1.141313883	0.453346521	0.029656874	5.283272409
NM_002068	guanine nucleotide binding protein G protein, alpha 15 Gq class	GNA15	1.282098762	2.431925043	0.101017926	1.072529944	0.015272131	2.267465871
NM_002074	guanine nucleotide binding protein G protein, beta polypeptide 1	GNB1	1.58105808	2.991891962	0.3172948	1.245991995	0.000101021	2.401212828
CO725743		GNG10	2.366004296	5.155113873	-0.23915632	0.847240629	0.000163554	6.084592375
NM_005274	guanine nucleotide binding protein G protein, gamma 5	GNG5	1.668041046	3.177828012	-0.320102398	0.801013022	0.00012221	3.967261359
NM_014498	golgi phosphoprotein 4	GOLPH4	2.227918263	4.684575308	-0.458313856	0.727836417	0.004356488	6.436302438
NM_000175	glucose phosphate isomerase	GPI	1.628289073	3.091461574	-0.503349731	0.705466885	0.000239161	4.382149806
NM_001005340	glycoprotein	GPNMB 1	3.531956324	11.56710817	-0.441329549	0.736455598	0.000256156	15.70645698
NM_177551	G protein-coupled receptor 109A	GPR109A	3.669214115	12.72165193	0.343519467	1.268848187	0.000368866	10.02614187
NM_020370	G protein-coupled receptor 84	GPR84	1.087989902	2.12577647	-0.139996593	0.907521298	0.002904179	2.342398435
AY966403	Callithrix jacchus cytosolic glutathione peroxidase	Gpx-1	1.899100305	3.729805256	-0.028144494	0.980680779	3.74E-07	3.803281697
NM_002086	growth factor receptor-bound protein 2	GRB2 1	1.350519125	2.55003867	-1.044859131	0.484692233	0.001881853	5.261150264
NM_013372	gremlin 1 homolog, cysteine knot superfamily	GREM1	2.264285424	4.804164075	-0.35832006	0.780072403	0.033374596	6.158613042
NM_002087	granulin	GRN	1.267530081	2.40749046	0.250171516	1.189348504	0.00027903	2.024209433
NM_025196	GrpE-like 1, mitochondrial	GRPEL1	1.727730172	3.312063131	-0.517052975	0.698797826	0.018783707	4.739658609
NM_002094	G1 to S phase transition 1	GSPT1	1.677368262	3.198439651	0.413417834	1.331837284	9.23E-05	2.401524337
NM_153699	glutathione S-transferase A5	GSTA5	1.176652126	2.260515995	-0.4656104	0.724164621	0.005078294	3.121549894
NM_001514	general transcription factor IIB	GTF2B	1.180497933	2.266549914	0.143131685	1.10429964	0.000790663	2.052477274
NM_002095	general transcription factor IIE, polypeptide 2, beta 34kDa	GTF2E2	1.442848741	2.718571441	0.039842066	1.028001284	0.000561231	2.644521446
NM_001521	general transcription factor IIIC, polypeptide 2, beta 110kDa	GTF3C2	1.110915915	2.159827234	-0.611946405	0.654313343	0.000335185	3.300906604

NM_013242	gene trap locus 3	GTL3	1.577413005	2.984342268	-1.138975857	0.454081808	0.010989168	6.572256846
NM_004130	glycogenin	GYG	1.669321969	3.180650756	-0.271654082	0.828369258	0.000225413	3.839653304
NM_002101	glycophorin C	GYPC 1	1.299959577	2.462219836	0.503194941	1.4173489	0.006597287	1.737200937
CN648306		GZMA	3.059172469	8.334943787	0.420937499	1.338797256	0.000488895	6.225695301
CN648055		GZMB	2.278031065	4.850155713	0.021381654	1.014931003	1.27E-05	4.778803385
NM_177925	H2A histone family, member J	H2AFJ 2	1.82102983	3.533333262	-0.524953011	0.694981745	0.009949764	5.084066288
NM_002105	H2A histone family, member X	H2AFX	1.77775592	3.428923981	-0.17033448	0.888636632	0.000105752	3.858634515
NM_002106	H2A histone family, member Z	H2AFZ	1.546011965	2.920088238	-0.025287052	0.982625067	0.002082846	2.971721705
CN646695		H41	1.189190617	2.280247805	-0.867281634	0.548178772	0.003443535	4.159679145
NM_002108	histidine ammonia-lyase	HAL	2.065598719	4.186076621	0.10298294	1.073991771	0.007057965	3.897680348
NM_178232	hyaluronan and proteoglycan link protein 3	HAPLN3	2.437889614	5.418485307	-0.158573697	0.895910364	0.002204857	6.048021681
NM_005328	hyaluronan synthase 2	HAS2	1.257095187	2.390140103	-0.159048627	0.895615482	0.000368867	2.668712356
NM_006118	HS1 binding protein	HAX1	1.230613769	2.346668034	-0.273758063	0.82716207	0.000745029	2.837011172
NM_006402	hepatitis B virus x interacting protein	HBXIP	1.135788959	2.197386972	-0.358605633	0.779918008	0.000213021	2.817458952
NM_005333	holocytochrome c synthase	HCCS	1.438810456	2.710972457	0.100339252	1.072025522	0.007458464	2.528832012
NM_005335	hematopoietic cell-specific Lyn substrate 1	HCLS1	1.261404368	2.397289877	-0.533635253	0.690811856	0.002371609	3.470250051
NM_014266	hematopoietic cell signal transducer	HCST	1.019438617	2.027130007	-0.925978592	0.526323387	6.26E-05	3.851491412
NM_002112	histidine decarboxylase	HDC	1.854081371	3.615214806	0.304165874	1.234704559	0.046981593	2.927999885
NM_004494	hepatoma-derived growth factor	HDGF	1.707460227	3.265853842	-1.00557776	0.498070628	0.000988604	6.55700951
NM_024711	human immune associated nucleotide 2	hIAN2	1.571686569	2.972520107	-0.865986926	0.548670941	0.018431019	5.417673665
NM_181054	hypoxia-inducible factor 1, alpha subunit	HIF1A 2	1.759421747	3.38562397	-0.046952587	0.967978838	5.85E-06	3.497621886
NM_014056	likely ortholog of mouse hypoxia induced gene 1	HIG1	1.336478034	2.525340692	-0.1763129	0.884961808	0.000326689	2.853615454
NM_005340	histidine triad nucleotide binding protein 1	HINT1	1.511269262	2.850607211	-0.006669522	0.995387709	0.000102127	2.863815963
NM_005319	histone 1, H1c	HIST1H1C	2.13426378	4.39013036	0.110847332	1.079862281	0.004540929	4.065453936
NM_021052	histone 1, H2ae	HIST1H2AE	1.744901218	3.351719037	-0.268954257	0.829920899	0.000320368	4.038600594
NM_138720	histone 1, H2bd	HIST1H2BD	1.986717218	3.963341325	-0.101599194	0.931999319	0.000316758	4.252515257
NM_003547	histone 1, H4g	HIST1H4G	2.202659194	4.603270413	-0.177741636	0.884085843	0.000906759	5.206813853
CN642065		HIST1H4H	2.00499657	4.013877452	-0.061863219	0.958026044	0.038336185	4.189737302
NM_003541	histone 1, H4k	HIST1H4K	2.119668791	4.345941609	-0.710695137	0.611025656	0.000204607	7.1125354
NM_003546	histone 1, H4l	HIST1H4L	1.855866408	3.619690657	0.15373248	1.11244382	0.045006588	3.253818836
NM_000188	hexokinase 1	HK1	1.48205501	2.793463578	-0.055900122	0.961994051	0.00080344	2.903826249
NM_000189	hexokinase 2	HK2	2.251659335	4.762302736	0.07696103	1.054793824	0.000206244	4.514913366
NM_002115	hexokinase 3	HK3	1.434346558	2.702597298	-0.303653062	0.810198286	0.015705214	3.335723297
NM_002116	major histocompatibility complex, class I, A	HLA-A	3.33190895	10.06942188	-0.550112528	0.682966856	9.82E-06	14.74364647
NM_005514	major histocompatibility complex, class I, B	HLA-B	4.085215579	16.97353999	-0.565044824	0.675934415	7.59E-06	25.11122324
NM_006120	major histocompatibility complex, class II, DM alpha	HLA-DMA	1.574064792	2.977424225	-0.05699009	0.961267532	5.56E-05	3.097393937
NM_002118	major histocompatibility complex, class II, DM beta	HLA-DMB	1.583684328	2.997343292	0.270493727	1.206220557	0.000271396	2.484904834
NM_033554	major histocompatibility complex, class II, DP alpha 1	HLA-DPA1	3.728139221	13.25200935	-0.782595226	0.581320131	5.07E-08	22.79640538
CO581942		HLA-DQA1	2.638058724	6.224934791	-0.772316911	0.585476467	9.84E-05	10.63225448
NM_019111	major histocompatibility complex, class II, DR alpha	HLA-DRA	1.9174185	3.777465293	-0.218529983	0.859440708	2.52E-05	4.395259917
DV770912		HLA-DRB1	2.017579719	4.049039505	-0.532291295	0.691455688	0.005931551	5.855819213
NM_022555	major histocompatibility complex, class II, DR beta 3	HLA-DRB3	2.441930794	5.43368448	-0.58624248	0.666075453	7.65E-05	8.157761192
NM_021983	major histocompatibility complex, class II, DR beta 4	HLA-DRB4	2.530745111	5.778700545	-0.71530189	0.609077666	4.89E-05	9.487625084
NM_002125	major histocompatibility complex, class II, DR beta 5	HLA-DRB5	2.544378055	5.833565987	-0.670000238	0.628506583	0.010760217	9.281630679
NM_005516	major histocompatibility complex, class I, E	HLA-E	3.151676574	8.88687733	-0.045021967	0.969275058	0.001129425	9.168581461
NM_018950	major histocompatibility complex, class I, F	HLA-F	2.296788998	4.913629225	-0.202814519	0.868853883	5.22E-05	5.655299842

NM_000859	3-hydroxy-3-methylglutaryl-Coenzyme A reductase	HMGCR	2.035014985	4.09826986	0.271661617	1.207197411	0.023716422	3.394863028
NM_006895	histamine N-methyltransferase	HNMT 1	1.703707488	3.257369741	-2.499300364	0.176862444	0.041619948	18.4175321
NM_006805	heterogeneous nuclear ribonucleoprotein A0	HNRPA0	1.414652902	2.665955875	-0.255326199	0.837797684	0.013502219	3.182099839
NM_194247	heterogeneous nuclear ribonucleoprotein A3	HNRPA3	1.402203208	2.643049066	-0.737962093	0.599585711	0.002518505	4.408125508
CO647754		HNRPF	1.563333054	2.95535831	-1.022714839	0.492189287	7.03E-05	6.004515718
NM_004838	homer homolog 3	HOMER3	1.081155183	2.115729493	-0.090321513	0.939313394	5.12E-05	2.252421297
NM_000860	hydroxyprostaglandin dehydrogenase 15-	HPGD	1.69826276	3.245099603	-0.646019783	0.639040919	0.007444114	5.078077958
NM_000194	hypoxanthine phosphoribosyltransferase 1	HPRT1	1.529711828	2.887281612	-0.089862692	0.939612172	0.000521127	3.072843985
CN645791		Hs.25892	1.331000811	2.515771357	-0.165480092	0.891631758	0.028308718	2.821536285
CO048892		Hs.406526	2.076034033	4.216465157	-0.241186446	0.846049251	0.015242495	4.983711234
CN648004		Hs.435390	1.537848388	2.903611404	-0.512079809	0.701210834	0.000443632	4.140853598
CO582842		Hs.512065	1.005989866	2.008320979	-0.436899169	0.73872066	0.000104472	2.718647371
CO648465		Hs.512323	1.604886264	3.0417177	-0.186938341	0.878468012	0.004161989	3.462525281
CO580643		Hs.515465	2.887859812	7.401716156	-0.265632005	0.831834251	0.000970723	8.898066108
CO647394		Hs.517602	1.274355645	2.418907573	-0.418459479	0.748223157	0.002597376	3.232869167
CN801916		Hs.518521	1.210826885	2.314702667	-0.160786713	0.89453714	0.001794668	2.587598172
CN641451		Hs.529772	1.181235627	2.267709167	-0.63177779	0.645380642	0.012938677	3.51375455
CN646671		Hs289044	3.381846729	10.42406969	-0.217014371	0.86034406	0.010261701	12.11616395
NM_181755	hydroxysteroid 11-beta dehydrogenase 1	HSD11B1 2	3.701554423	13.01004839	-0.268518165	0.830171802	0.007214201	15.67151324
NM_016142	hydroxysteroid 11-beta dehydrogenase 12	HSD17B12	1.87014716	3.655698676	-1.091553698	0.469255741	0.023762977	7.790418642
DQ266251	Macaca fascicularis 17-beta hydroxysteroid dehydrogenase 5	HSD17B5	1.212536107	2.317446619	-1.15500588	0.449064356	0.020912056	5.160611363
NM_017510	gp25L2 protein	HSGP25L2G	1.51306832	2.854164169	-0.20166289	0.869547721	0.000274889	3.282354839
NM_006597	heat shock 70kDa protein 8	HSPA8 1	2.269297065	4.820881824	-0.816568307	0.567790924	2.15E-05	8.49059332
NM_014306	hypothetical protein HSPC117	HSPC117	1.62542851	3.085337923	0.009576736	1.006660168	0.001120109	3.064925006
NM_014184	HSPC163 protein	HSPC163	1.218591448	2.327193945	0.416950781	1.335102754	0.000326772	1.743082274
NM_014187	HSPC171 protein	HSPC171	1.056074947	2.079266884	0.014850092	1.010346458	0.002441103	2.057974142
NM_016209	hematopoietic stem/progenitor cells 176	HSPC176	2.09712322	4.278553765	-0.221446161	0.857705239	0.001714617	4.988373125
NM_005348	heat shock 90kDa protein 1, alpha	HSPCA	1.224485415	2.33672088	-0.136539127	0.909698811	0.000885621	2.568675314
NM_002156	heat shock 60kDa protein 1	HSPD1	1.018424438	2.025705485	0.100276598	1.071978966	0.000770376	1.889687717
NM_014500	HIV TAT specific factor 1	HTATSF1	1.126160427	2.182770464	-0.3050152	0.809433689	0.002383251	2.696663723
NM_006389	hypoxia up-regulated 1	HYOU1	1.259528828	2.394175365	-0.339360007	0.79039186	0.008691147	3.029099219
CO646712		IAN4L1	2.168450566	4.495403346	0.025989951	1.018178108	0.017200007	4.415144376
NM_182757	IBR domain containing 2	IBRDC2	2.171318996	4.504350197	-0.322643253	0.799603531	0.022043189	5.6332295
NM_153341	IBR domain containing 3	IBRDC3	2.620833906	6.151055128	0.333437537	1.260012055	0.006617481	4.881743078
NM_000201	intercellular adhesion molecule 1	ICAM1	1.11513387	2.166151083	-0.014849866	0.989759651	0.016696145	2.18856273
NM_000873	intercellular adhesion molecule 2	ICAM2	1.070269888	2.099826149	-0.237413844	0.848264538	0.003940194	2.475437855
NM_170705	isoprenylcysteine carboxyl methyltransferase	ICMT 2	1.380517289	2.603617089	0.239987228	1.180982207	0.012936594	2.204620082
NM_004969	insulin-degrading enzyme	IDE	2.208757881	4.622770949	-0.109638829	0.926820058	0.019702518	4.987776118
NM_002168	isocitrate dehydrogenase 2 NADP+, mitochondrial	IDH2	1.719144554	3.292411256	0.164533956	1.120803958	1.55E-05	2.937544279
NM_052815	immediate early response 3	IER3 long	2.561339855	5.902556127	0.159060916	1.116560107	0.000592336	5.286375621
NM_016097	immediate early response 3 interacting protein 1	IER3IP1	1.57252154	2.974240974	-1.110370648	0.46317502	0.003125521	6.42141922
NM_006332	interferon, gamma-inducible protein 30	IFI30	2.532669091	5.786412168	-0.419891281	0.747480951	1.89E-06	7.741216899
NM_005533	interferon-induced protein 35	IFI35	1.014010483	2.019517269	-0.335443957	0.792540213	0.000186967	2.548157477
XM_498423	interferon-induced protein with tetratricopeptide repeats 1	IFIT1	2.616443651	6.132365349	-0.302588962	0.810796091	0.003043265	7.563387911

NM_001547	interferon-induced protein with tetratricopeptide repeats 2	IFIT2	2.025341782	4.070883067	-0.724404784	0.605246701	0.000943351	6.725989683
NM_001549	interferon-induced protein with tetratricopeptide repeats 3	IFIT3	3.918548646	15.12170223	-0.037351009	0.974442519	0.001256075	15.51831117
XR_013560	Interferon-induced transmembrane protein 3 Interferon-inducible protein 1-8U	IFITM3	1.380833148	2.60418718	-0.955285653	0.515739463	0.000412566	5.049423914
NM_000619	interferon, gamma	IFNG	4.364915094	20.60489339	0.044108611	1.031045936	0.03675358	19.98445721
NM_000416	interferon gamma receptor 1	IFNGR1	2.24427326	4.73798378	-0.33370816	0.793494341	0.004787803	5.971036634
NM_005534	interferon gamma receptor 2 interferon gamma transducer 1	IFNGR2	2.201450458	4.59941526	0.175428212	1.129299547	9.56E-05	4.072803601
NM_031943	IFP38	IFP38	1.54052076	2.908994886	-1.11277998	0.462402153	0.005761115	6.291049616
NM_000618	insulin-like growth factor 1 somatomedin C	IGF1	2.301691257	4.930354072	0.000396579	1.000274926	0.00855197	4.928998964
XR_012149	insulin-like growth factor 2 receptor	IGF2R	2.323321614	5.004831902	-0.556037732	0.680167637	0.008250324	7.358232925
DV768600		IGFBP3	2.945499852	7.703424097	-0.544839682	0.68546757	0.003963775	11.23820358
NM_001552	insulin-like growth factor binding protein 4	IGFBP4	1.636888994	3.109944844	-0.235159704	0.849590946	0.001546875	3.66052023
NM_001553	insulin-like growth factor binding protein 7	IGFBP7	2.856225831	7.241185109	-0.710064854	0.611292659	0.013049716	11.8456929
CN645487		IGHG1	2.086561061	4.247344299	-0.111198377	0.92581871	0.002281243	4.587663065
NM_144646	immunoglobulin J polypeptide, linker protein for immunoglobulin alpha and mu polypeptides	IGJ	1.227991348	2.342406314	-0.315367038	0.803646508	0.000804287	2.914722196
NM_201612	IKK interacting protein	IKIP2	1.892940173	3.713913394	-0.849378143	0.555023921	0.038205173	6.691447436
NM_001558	interleukin 10 receptor, alpha	IL10RA	2.322005051	5.000266717	-0.123280307	0.918097764	3.82E-05	5.446333616
NM_000628	interleukin 10 receptor, beta	IL10RB	2.263525512	4.801634241	-0.070879808	0.952057222	0.001025988	5.0434303
NM_000641	interleukin 11	IL11	2.848167233	7.200850093	-0.046082465	0.968562824	0.045333131	7.434572043
NM_001560	interleukin 13 receptor, alpha 1	IL13RA1	2.643167498	6.247017184	-0.502292887	0.705983863	0.007434898	8.848668517
NM_172200	interleukin 15 receptor, alpha	IL15RA 2	2.065633493	4.186177522	0.065557683	1.046489389	0.009131763	4.000210195
NM_000576	interleukin 1, beta	IL1B	3.471300923	11.09087222	-0.461363856	0.726299325	0.000871132	15.27038762
NM_004633	interleukin 1 receptor, type II	IL1R2 1	2.548693716	5.851042574	-0.296175767	0.814408336	0.040485349	7.184408998
NM_002182	interleukin 1 receptor accessory protein	IL1RAP 1	2.844243818	7.181293952	-0.236504024	0.848799656	5.15E-05	8.460528824
NM_000577	interleukin 1 receptor antagonist	IL1RN 3	1.928599906	3.806855754	0.246347316	1.186200032	3.09E-05	3.209286505
NM_004843	interleukin 27 receptor, alpha	IL27RA	2.341235283	5.06736336	-0.640245871	0.641603594	5.75E-05	7.897965982
NM_172374	interleukin 4 induced 1	IL4I1 2	2.39680143	5.266342788	-0.193173036	0.87467985	0.000784118	6.020880426
NM_000418	interleukin 4 receptor	IL4R 1	1.939859605	3.836683094	-0.17462865	0.885995545	0.000278774	4.330363865
NM_000600	interleukin 6	IL6	5.634289664	49.66954631	-0.84760096	0.555708048	0.007056147	89.38064952
NM_002185	interleukin 7 receptor	IL7R	1.118318143	2.170937426	-0.334769621	0.792910744	0.001704604	2.73793418
NM_002164	indoleamine-pyrrole 2,3 dioxygenase	INDO	5.469221031	44.29957739	-0.231842942	0.851546406	1.86E-05	52.02250527
NM_002193	inhibin, beta B	INHBB	1.834003367	3.565250303	0.102130338	1.073357253	0.035258531	3.32158775
XR_012012	IQ motif containing GTPase activating protein 1	IQGAP1	1.163208829	2.239549926	-0.762870376	0.589322651	0.000235919	3.80021016
NM_002198	interferon regulatory factor 1	IRF1	3.258271519	9.568358995	-0.746205127	0.596169665	0.001036519	16.0497247
NM_001572	interferon regulatory factor 7	IRF7 a	2.334317258	5.04312246	-0.421885344	0.746448512	0.001125398	6.756155822
NM_002163	interferon regulatory factor 8	IRF8	1.415971794	2.66839417	-0.41475757	0.750145538	0.003395217	3.557168621
NM_002201	interferon stimulated gene 20kDa	ISG20	4.2853467	19.49924961	-0.385017557	0.765769679	0.027753686	25.46359582
NM_002205	integrin, alpha 5 fibronectin receptor, alpha polypeptide	ITGA5	1.685808102	3.217205523	-0.184578945	0.879905842	0.000457789	3.656306584
NM_000632	integrin, alpha M complement component receptor 3, alpha; also known as CD11b p170, macrophage antigen alpha polypeptide	ITGAM	3.34873331	10.18753641	0.044007363	1.030973581	0.000131481	9.881471844

NM_000887	integrin, alpha X antigen CD11C p150, alpha polypeptide	ITGAX	1.502795996	2.833914036	0.349936673	1.274504682	0.016596902	2.223541488
NM_002211	integrin, beta 1 fibronectin receptor, beta polypeptide, antigen CD29 includes MDF2, MSK12	ITGB1	1.140908257	2.20519809	0.725930666	1.653967247	0.026896471	1.333277968
NM_033667	integrin, beta 1 fibronectin receptor, beta polypeptide, antigen CD29 includes MDF2, MSK12	ITGB1	1.324984317	2.505301643	-0.339191916	0.790483955	0.003947189	3.169326371
NM_133376	integrin, beta 1 fibronectin receptor, beta polypeptide, antigen CD29 includes MDF2, MSK12	ITGB1	1.471674911	2.773436917	-0.183791633	0.880386158	0.000241077	3.150250481
XR_012415	integrin, beta 2	ITGB2	1.595049986	3.021049841	0.122252109	1.088432627	1.91E-06	2.775596547
NM_017625	intelectin 1	ITLN1	1.988324662	3.96775972	0.578857337	1.493665746	0.001214909	2.656390648
NM_152713	integral membrane protein 1	ITM1	1.644836489	3.127124134	-0.002514559	0.998258559	0.005176735	3.132579337
NM_030926	integral membrane protein 2C	ITM2C	1.090500575	2.129479107	-0.353594797	0.782631565	0.004089524	2.72092157
NM_002225	isovaleryl Coenzyme A dehydrogenase	IVD	1.024769324	2.034634036	0.157010476	1.114974315	0.001028415	1.824825926
NM_004972	Janus kinase 2	JAK2	2.071928709	4.204483865	-0.554454082	0.680914668	0.000515219	6.174758835
NM_007213	JM4 protein	JM4	1.039252838	2.055163022	-0.102177525	0.931625785	0.012888175	2.205996287
NM_005548	lysyl-tRNA synthetase	KARS	1.065201866	2.092462643	0.439165168	1.35581954	0.003533059	1.543319433
NM_015483	kelch repeat and BTB POZ domain containing 2	KBTBD2	1.153146213	2.223983698	-0.260106405	0.835026331	0.000802588	2.663369546
NM_080671	potassium voltage-gated channel, Isk-related family, member 4	KCNE4	1.619825689	3.073379005	-0.265644384	0.831827114	0.007531303	3.694732901
NM_003740	potassium channel, subfamily K, member 5	KCNK5	1.53570429	2.899299335	-0.797262887	0.57543988	0.012265685	5.038405291
NM_004823	potassium channel, subfamily K, member 6	KCNK6	1.293376132	2.451009595	-0.03819233	0.97387443	0.007115249	2.516761422
NM_002247	potassium large conductance calcium-activated channel, subfamily M, alpha member 1	KCNMA1	1.230713674	2.346830544	0.026594187	1.018604635	0.004611661	2.303966095
NM_004137	potassium large conductance calcium-activated channel, subfamily M, beta member 1	KCNMB1	3.009456891	8.05261239	-0.159255481	0.895487078	0.009257665	8.992438406
NM_018992	potassium channel tetramerisation domain containing 5	KCTD5	1.659614773	3.159321535	-0.338721474	0.790741762	0.014740728	3.995389755
CN802611		KDELR2	2.081780526	4.233293528	-1.34114566	0.39470709	0.046106247	10.72515197
NM_006855	KDEL Lys-Asp-Glu-Leu endoplasmic reticulum protein retention receptor 3	KDELR3 1	1.609509779	3.051481363	0.655680367	1.575358708	0.018091916	1.937007329
NM_014752	KIAA0102 gene product	KIAA0102	2.22166261	4.664306551	0.519332753	1.433292196	0.000178982	3.25426076
XM_040265	KIAA0217	KIAA0217	1.347999412	2.545588832	-0.24433421	0.844205299	0.030588491	3.01536704
CN804590		KIAA0247	2.322800979	5.0030261	-0.072965723	0.950681688	0.000633584	5.262567022
NM_014861	KIAA0703 gene product	KIAA0703	2.333959097	5.041870619	0.482284883	1.396954356	0.01420337	3.6091878
XM_093895	KIAA0882 protein	KIAA0882	1.962393062	3.897078687	0.376211357	1.297928902	0.02016632	3.002536335
XM_375553	KIAA0963	KIAA0963	1.219064837	2.327957688	-0.673952427	0.626787178	0.002222741	3.714111853
NM_014939	KIAA1012	KIAA1012	1.206602921	2.307935525	-0.090954591	0.938901299	0.000288669	2.45812369
NM_018999	KIAA1128	KIAA1128	1.105428432	2.151627647	0.184061376	1.136077595	0.019477382	1.893909057
NM_020701	KIAA1160 protein	KIAA1160	1.188431536	2.279048356	-0.523234518	0.695810079	0.035701417	3.275388537
NM_020792	KIAA1363 protein	KIAA1363	1.618869554	3.071342822	-0.290883098	0.81740156	0.010503355	3.757446733
NM_021035	KIAA1404 protein	KIAA1404	1.277361863	2.42395323	0.001538685	1.001067104	0.007282614	2.421369377
NM_020895	KIAA1533	KIAA1533	1.148369609	2.216632509	-0.101037574	0.932362204	0.002831085	2.377437117
NM_012310	kinesin family member 4A	KIF4A	1.54755089	2.923204764	-0.377828283	0.769595206	0.028023142	3.79836665
CO725491		KNS2	1.287978622	2.441856841	-0.643847847	0.640003701	0.023501806	3.815379251
NM_002265	karyopherin	KPNB1	1.904997071	3.745081386	-1.035907172	0.487709111	0.041448579	7.678924391
NM_002275	keratin 15	KRT15	1.190669849	2.282587	-0.039167797	0.973216175	0.004480998	2.345405944

NM_005554	keratin 6A	KRT6A	3.41501288	10.66648464	-0.683264777	0.622754404	0.044356443	17.12791524
NM_173853	keratinocyte associated protein 3	KRTCAP3	2.476470397	5.565342195	0.269650199	1.205515499	0.014562989	4.616566275
NM_032857	lactamase, beta	LACTB	1.888808951	3.703293649	-0.381747293	0.767507475	0.002050051	4.825091308
NM_002286	lymphocyte-activation gene 3	LAG3	5.386856809	41.8413301	-1.215508996	0.430621126	0.026410737	97.16506597
NM_021708	leukocyte-associated Ig-like receptor 1	LAIR1	2.233623258	4.703136676	-0.296509264	0.814220097	0.000922013	5.776247348
NM_000228	laminin, beta 3	LAMB3	1.169102601	2.248717765	-0.226466245	0.854725906	3.22E-05	2.630922673
NM_005561	lysosomal-associated membrane protein 1	LAMP1	2.077401143	4.22046261	0.49237342	1.406757271	0.000866401	3.000135629
NM_013995	lysosomal-associated membrane protein 2	LAMP2	1.662595492	3.165855685	0.597985698	1.513601788	0.034280819	2.091604087
NM_015907	leucine aminopeptidase 3	LAP3	3.911300503	15.04592087	-1.169413125	0.444602164	0.006777353	33.84131272
NM_006762	Lysosomal-associated multispanning membrane protein-5	LAPTM5	2.818477927	7.054177739	-0.499607166	0.707299347	0.018635051	9.973397788
NM_203463	LAG1 longevity assurance homolog 6	LASS6	1.420208476	2.67624181	-0.035957222	0.975384383	0.00051345	2.74378169
NM_005564	lipocalin 2	LCN2	2.369376338	5.167177122	-0.21635452	0.86073765	0.000274889	6.003196356
NM_002298	lymphocyte cytosolic protein 1 L-plastin	LCP1	1.975441684	3.932486149	0.141356179	1.102941428	0.000135075	3.565453295
NM_005565	lymphocyte cytosolic protein 2 SH2 domain containing leukocyte protein of 76kDa	LCP2	1.830891407	3.557568183	-0.351580293	0.783725154	0.006860572	4.539305859
NM_005566	lactate dehydrogenase A	LDHA	4.525540081	23.03155765	-0.794792321	0.576426145	2.67E-06	39.95578245
XR_013661	lactate dehydrogenase B	LDHB	1.018683342	2.026069048	-0.151343441	0.900411606	0.001362596	2.250158742
NM_002306	lectin, galactoside-binding, soluble, 3	LGALS3	1.867584101	3.64920981	-0.414772453	0.7501378	2.28E-06	4.864719272
NM_009587	lectin, galactoside-binding, soluble, 9	LGALS9	1.131693329	2.191157713	-0.474426299	0.71975295	0.000632775	3.044319185
CN642140		LGMN	1.446090865	2.724687673	-0.72808312	0.603705512	4.23E-05	4.513272807
NM_024119	likely ortholog of mouse D11lgp2	LGP2	1.311705121	2.482347552	-0.912753782	0.531170238	0.000828436	4.673355875
NM_005779	lipoma HMGIC fusion partner-like 2	LHFPL2	2.846509337	7.192579874	1.05965423	2.084431888	0.004593896	3.450618808
NM_006866	leukocyte immunoglobulin-like receptor, subfamily A with TM domain, member 2	LILRA2	1.425015458	2.685173777	-0.156676199	0.897089482	0.016211559	2.993206176
NM_005874	leukocyte immunoglobulin-like receptor, subfamily B with TM and ITIM domains, member 2	LILRB2	1.826053127	3.545657363	-0.179107892	0.883248995	0.000812379	4.014335009
NM_002314	LIM domain kinase 1	LIMK1	2.910080963	7.51660381	-0.506528781	0.703914066	0.026665403	10.6782975
NM_016733	LIM domain kinase 2	LIMK2	2.165218415	4.485343315	0.04359564	1.030679398	3.10E-05	4.351831736
NM_004987	LIM and senescent cell antigen-like domains 1	LIMS1	1.548783262	2.925702877	0.066785322	1.047380261	0.04853632	2.793353078
CN806576		LITAF	2.581922811	5.987371596	-0.472029703	0.720949592	0.00880538	8.304840812
NM_004862	lipopolysaccharide-induced TNF factor	LITAF	3.661981976	12.65803868	-0.610164121	0.655122171	5.63E-08	19.32164601
NM_032737	lamin B2	LMNB2	1.089068059	2.127365702	-0.386164078	0.765161358	0.005121935	2.780283767
NM_005475	lymphocyte adaptor protein	LNK	1.070000755	2.099434466	-0.568563382	0.674287902	5.03E-05	3.113557963
XM_374879	hypothetical protein LOC114971	LOC114971	2.35312919	5.109312545	-0.061651711	0.958166507	0.001485219	5.332384829
NM_001002836	hypothetical protein LOC126208	LOC126208	1.188582431	2.279286741	-0.332438956	0.794192721	0.003110124	2.869941615
NM_145266	RIKEN cDNA 2700047N05	LOC134492	1.147442234	2.2152081	-0.92884036	0.525280393	0.009019329	4.217191672
NR_000030	peptidylprolyl isomerase A processed pseudogene	LOC134997	1.088486374	2.126508135	-0.320858979	0.800593063	7.69E-06	2.656166076
NM_207322	hypothetical LOC145741	LOC145741	1.219645677	2.32889513	-0.172305972	0.887423109	0.013920758	2.624334554
NM_194294	hypothetical protein LOC169355	LOC169355	1.602343279	3.0363609	-0.073580918	0.950276385	0.041150939	3.195239773
CK231449		LOC283241	1.562064616	2.952761057	-0.333983764	0.793342771	0.005745633	3.721923441
CN647374		LOC283951	1.585765927	3.001671143	-0.402954564	0.756307817	0.005654151	3.968848495
XM_208423	ribosomal protein S2; 40S ribosomal protein S2	LOC286444	1.033439298	2.046898122	-0.368350928	0.774667474	4.84E-06	2.642292583
NM_001012754	RIKEN cDNA 8030451K01	LOC387921	1.774406542	3.420972583	-0.835319725	0.560458818	0.000226414	6.103878594

NM_203434	RIKEN cDNA 2610524G09	LOC389792	1.717358619	3.288338049	0.225887153	1.169496183	0.000922859	2.81175612
XM_373301	peptidyl-Pro cis trans isomerase	LOC392352	1.162649128	2.238681251	-0.117651658	0.921686703	0.000306111	2.428896114
XM_375224	cervical cancer suppressor-1	LOC400410	4.617767178	24.55197502	-0.051722495	0.964783745	0.000347102	25.44816406
XM_376876	coiled-coil-helix-coiled-coil-helix domain containing 2; 16.7kD protein; chromosome 7 open reading frame 17	LOC401531	2.031679574	4.088805887	-0.475370323	0.719282135	0.000273111	5.684564774
XM_498527	LOC440068	LOC440068	1.677508828	3.198751299	-0.117777376	0.921606389	0.007218601	3.470843232
XM_496348	Ubiquinol-cytochrome C reductase complex 11 kDa protein, mitochondrial precursor	LOC440567	1.04803321	2.067709069	0.225631857	1.16928925	0.004477688	1.768346942
XM_496386	Fc gamma receptor type I	LOC440607	3.134354481	8.780812745	-0.000595931	0.999587017	0.004841751	8.784440568
NM_001007544	hypothetical gene supported by AK122631; BC071785	LOC440712	1.057645913	2.081532253	-0.052902526	0.963994937	0.039141103	2.159277163
XM_496823	RIKEN cDNA A630077B13 gene; RIKEN cDNA 2810048G17	LOC441168	4.572676189	23.79647851	-0.311032131	0.806064879	0.001233409	29.52179054
XM_496854	Heat shock cognate 71 kDa protein	LOC441198	1.443920279	2.720591366	-0.306996494	0.808322833	1.82E-06	3.36572376
XM_933457	tropomyosin 4 3	LOC643634	1.67642238	3.196343327	-1.116971389	0.461060703	0.00147247	6.932586771
NM_022733	hypothetical protein AL133206	LOC64744	1.80704108	3.499238697	-0.25254269	0.839415675	0.000534935	4.168660176
XR_009709	hypothetical protein LOC693502	LOC693502	1.715037908	3.283052698	-0.108787271	0.927367279	0.001199421	3.540186044
XR_009898	hypothetical protein LOC694682	LOC694682	1.208447038	2.310887517	0.206472077	1.153863109	0.003811815	2.002739752
XR_009904	Vitamin K-dependent protein S precursor	LOC694845	2.233945478	4.704187217	-0.195805847	0.87308508	0.035499787	5.388005503
XR_010283	aldehyde dehydrogenase 9A1	LOC695383	1.516553263	2.861066961	-0.320817874	0.800615874	0.000565419	3.573582605
XR_010049	transmembrane protein 109	LOC695517	3.29318527	9.802741439	-0.387356827	0.764529022	0.031201288	12.82193502
XR_010074	Histone acetyltransferase type B catalytic subunit	LOC695933	3.248322925	9.502604104	-0.208707336	0.86531221	0.02521951	10.98170579
XR_010166	Protein KIAA0143	LOC696036	1.383094798	2.608272852	-0.202763721	0.868884476	0.005068431	3.001863797
XR_010915	zinc finger protein 259	LOC696348	1.217807873	2.325930314	-0.392098729	0.762020265	0.005676288	3.052320809
XR_010654	acyl-CoA synthetase long-chain family member 5 isoform a	LOC696404	1.077825126	2.110851557	-0.160544523	0.894687321	0.005470204	2.359317615
XR_010210	hypothetical protein LOC696802	LOC696802	1.20584526	2.306723783	-0.66518823	0.630606423	0.008950162	3.657945272
XR_011062	hypothetical protein LOC697011	LOC697011	1.626831358	3.088339504	0.036311978	1.025488972	0.000119419	3.011577489
XR_010456	keratin 19	LOC698425	1.328300234	2.511066496	-0.475859208	0.719038433	3.43E-05	3.492256296
XR_010861	zinc finger protein 406 isoform ZFAT-1	LOC698512	1.748225352	3.359450683	-0.886469512	0.540936251	0.00304651	6.210437328
XR_011615	hypothetical protein LOC699195	LOC699195	1.698105476	3.244745839	-0.806461636	0.571782498	0.009259351	5.67479042
XR_010574	protein kinase Myt1 isoform 1	LOC699350	1.187589752	2.277718966	-0.000843559	0.99941546	0.047936528	2.279051162
XR_010577	hypothetical protein LOC699401	LOC699401	1.502726067	2.833776677	-0.41386683	0.750608832	0.040787549	3.775304202
XR_011344	Transmembrane protein 111	LOC699481	1.419236189	2.674438797	0.009590367	1.00666968	0.00011725	2.656719331
XR_011279	epithelial stromal interaction 1 isoform 1	LOC700208	1.224967715	2.337502187	-1.019286992	0.49336012	0.013542379	4.73792285
XR_011417	hypothetical protein LOC700304	LOC700304	1.383258363	2.608568581	-0.31026024	0.806496267	0.005183602	3.234445947
XR_011133	hypothetical protein LOC700625	LOC700625	1.604472829	3.040846157	-0.074076163	0.949950231	0.006938041	3.201058388
XR_010200	v-crk sarcoma virus CT10 oncogene homolog isoform a	LOC701185	1.942127191	3.842718223	-1.128889442	0.457267585	0.033055337	8.403653238
XR_010922	basic leucine zipper and W2 domains 1	LOC701731	2.024408889	4.068251556	-1.128266139	0.457465186	0.007249194	8.893029851
XR_011890	ribosomal protein L13a	LOC702871	1.191059804	2.283204057	0.39598808	1.315843653	0.003312781	1.735163636
XR_011197	hypothetical protein LOC703841	LOC703841	2.277398765	4.848030467	0.086352817	1.061682816	0.012912925	4.566364259
XR_012351	riboflavin kinase	LOC704540	1.688535187	3.223292667	0.450562434	1.366572911	0.021099358	2.358668638
XR_013774	hematopoietic protein 1	LOC705782	1.83583937	3.569790402	-0.275202529	0.826334307	0.003635758	4.320031702
XR_011517	hypothetical protein LOC706003	LOC706003	1.228118168	2.342612232	-0.816126701	0.56796475	0.000121991	4.124573279
XR_010481	hydroxyacyl dehydrogenase, subunit B	LOC707168	1.510487119	2.849062202	-0.366202174	0.775822126	0.000227711	3.672313674
XR_013105	hypothetical protein LOC707681	LOC707681	1.748022477	3.358978303	-0.246074644	0.843187483	0.020082567	3.98366718

XR_013529	golgi apparatus protein 1	LOC710037	1.110720268	2.159534355	0.038478027	1.027029789	6.50E-05	2.102698849
XR_013871	selenophosphate synthetase 2	LOC710575	2.187686822	4.555744448	-0.443881788	0.735153903	0.026906869	6.196994165
XR_012275	hypothetical protein LOC710725	LOC710725	1.686725531	3.219252038	-0.630523456	0.645942004	0.000197738	4.983809718
XR_012394	putative small membrane protein NID67	LOC711300	2.889144224	7.40830874	-0.563292514	0.67675591	0.009732103	10.94679578
XR_012352	rabaptin, RAB GTPase binding effector protein 1	LOC711646	2.060892799	4.172444323	-0.147241659	0.902975244	0.000652976	4.62077377
XR_012476	hypothetical protein LOC711693	LOC711693	3.108485883	8.624769394	-0.850560265	0.55456933	0.023030351	15.55219327
XR_012634	hypothetical protein LOC712466	LOC712466	1.338707436	2.529246128	-0.904079198	0.53437366	0.000114819	4.73310404
XR_014226	lactotransferrin	LOC713115	4.466716708	22.11137302	-2.307653415	0.201988713	0.032049784	109.4683592
XR_013476	procollagen-lysine, 2-oxoglutarate 5-dioxygenase 3 precursor	LOC714283	1.44592893	2.724381857	-0.350007761	0.784579877	0.000176976	3.472408529
XR_013163	Y45F10A.6b	LOC715258	1.351825252	2.552348362	-0.283429563	0.821635509	0.000981983	3.106424121
XR_014059	interferon-stimulated transcription factor 3, gamma 48kDa	LOC715335	1.36201154	2.570433235	-0.189084995	0.87716187	0.002441562	2.930397824
XR_013717	hypothetical protein LOC716802	LOC716802	2.580547944	5.981668434	-1.092095385	0.469079584	0.004497082	12.75192663
XR_013647	ubiquitin specific protease 10	LOC717518	1.336434737	2.525264904	-0.670486348	0.628294846	0.001205494	4.019235425
XR_013743	SH2 containing inositol phosphatase isoform b	LOC717832	1.596588063	3.024272341	-0.225327877	0.855400599	0.000671685	3.535504118
XR_014283	nucleobindin 1	LOC718380	1.363156032	2.572473173	-0.285059591	0.820707708	0.000423094	3.134457185
XR_014008	hypothetical protein LOC718777	LOC718777	1.721484145	3.297754826	-0.612235261	0.654182349	5.14E-05	5.041033024
XR_014077	hypothetical protein LOC719082	LOC719082	2.320002078	4.993329389	-1.162478269	0.446744456	0.000931516	11.17714909
XR_014088	glucocerebrosidase precursor	LOC719103	1.448079794	2.728446573	-0.033213281	0.977241285	0.005591761	2.791988646
XR_013248	heat shock 70kDa protein 1B	LOC720054	1.356097588	2.559917983	-0.182610916	0.88110697	4.16E-06	2.905343016
XR_014670	hypothetical protein LOC721870	LOC721870	1.766757233	3.402882257	-0.302351667	0.810929462	0.006552536	4.196274051
XR_014799	CG7289-PA	LOC722762	1.053140957	2.075042604	-0.285603594	0.820398299	0.000728688	2.529311196
XR_014800	lysyl hydroxylase precursor	LOC722763	2.659449551	6.317919482	-0.087404195	0.941214731	0.002236269	6.712516577
NM_138358	hypothetical protein BC011833	LOC90580	1.527270974	2.882400833	-0.222020931	0.857363598	0.007780492	3.36193517
CN804592		LOC93380	1.240853872	2.363383699	-0.737302913	0.59985973	0.001923821	3.939893916
CN804935		LOXL1	1.210427668	2.314062239	0.039693949	1.027895747	0.011015251	2.251261614
NM_002318	lysyl oxidase-like 2	LOXL2	2.002940636	4.008161488	-0.07541923	0.949066294	0.031334816	4.22326819
XR_012709	lipoprotein lipase	LPL	1.748489307	3.360065385	0.287313634	1.220365782	0.001473386	2.753326449
NM_004811	leupaxin	LPXN	1.483558576	2.796376422	-0.420766058	0.747027854	0.005723108	3.743336218
NM_130830	leucine rich repeat containing 15	LRRC15	4.085534949	16.97729785	-1.066742989	0.477395546	4.63E-05	35.56232982
NM_145256	leucine rich repeat containing 25	LRRC25	1.263426881	2.400652992	0.271847377	1.207352858	0.003963454	1.988360714
NM_019594	leucine rich repeat containing 8 family, member A	LRRC8A	1.331493287	2.516630285	-0.351273789	0.783891676	0.027837805	3.210431187
CO725352		LSM10	1.261395457	2.39727507	-0.219412389	0.858915202	0.002850654	2.791049761
NM_002341	lymphotoxin beta TNF superfamily, member 3	LTB 1	1.031600824	2.044291352	-0.504159772	0.705070892	0.007595008	2.89941249
NM_002345	lumican	LUM	2.12495715	4.361901374	-0.398049064	0.758883818	0.001911225	5.747785461
NM_020169	latexin	LXN	1.890747411	3.70827288	-0.860052206	0.550932621	0.003189551	6.730900909
NM_002346	lymphocyte antigen 6 complex, locus E	LY6E	2.433982596	5.403831148	-0.367402817	0.775176738	0.01635858	6.971095604
NM_004271	lymphocyte antigen 86	LY86	2.806570797	6.996196435	-0.114285171	0.923839943	0.006770279	7.572952965
NM_015364	lymphocyte antigen 96	LY96	4.238183798	18.87210961	0.17918748	1.132246029	0.03522352	16.66785232
NM_002350	v-yes-1 Yamaguchi sarcoma viral related oncogene homolog	LYN	2.561628875	5.903738726	-0.493985697	0.710060719	1.70E-05	8.314413924
CN643020		LYZ	3.959677122	15.55899663	-0.19591851	0.873016902	1.01E-05	17.82210242
NM_005907	mannosidase, alpha, class 1A, member 1	MAN1A1	1.546719294	2.92152026	-0.824493654	0.564680355	0.037008265	5.173759339
NM_002755	mitogen-activated protein kinase kinase 1	MAP2K1	1.815815968	3.520586936	0.43906936	1.355729504	0.001821387	2.596821067

NM_021970	mitogen-activated protein kinase kinase 1 interacting protein 1	MAP2K1IP1	2.003293503	4.009141959	-0.37553047	0.770821934	0.011079057	5.201125943
CO649191		MAPBPIP	1.197097884	2.292779926	-0.134748344	0.9108287	0.000710985	2.517246028
NM_002745	mitogen-activated protein kinase 1	MAPK1 1	1.250962233	2.380001088	-0.596199224	0.661494367	0.042555275	3.597915881
NM_138957	mitogen-activated protein kinase 1	MAPK1 2	2.716960486	6.574861415	-0.687149824	0.621079639	0.010550947	10.58618091
NM_002356	myristoylated alanine-rich protein kinase C substrate	MARCKS	2.057425028	4.162427165	0.397033946	1.316797906	7.48E-05	3.161022012
NM_006770	macrophage receptor with collagenous structure	MARCO	1.313027696	2.484624261	-0.143421071	0.905369699	3.82E-05	2.744320096
NM_182796	methionine adenosyltransferase II, beta	MAT2B 2	2.740334774	6.682253778	-0.268406907	0.830235826	0.000158971	8.04862133
NM_002388	MCM3 minichromosome maintenance deficient 3	MCM3	1.802024035	3.487091053	0.278260344	1.212731645	0.014712604	2.875402045
XR_014169	minichromosome maintenance deficient protein 5	MCM5	1.294128428	2.452288012	-0.416280169	0.749354263	0.037982046	3.272534944
NM_014060	malignant T cell amplified sequence 1	MCTS1	1.494719862	2.818094249	0.055016873	1.038871253	0.00075999	2.712650139
NM_005918	malate dehydrogenase 2, NAD	MDH2	1.009496936	2.013208977	0.553490392	1.467632128	0.000141429	1.371739511
NM_002396	malic enzyme 2, NAD+-dependent, mitochondrial	ME2	2.984895041	7.916677278	-0.087844113	0.940927772	0.006801756	8.413692861
NM_014791	maternal embryonic leucine zipper kinase	MELK	1.143378683	2.208977437				
NM_015143	methionyl aminopeptidase 1	METAP1	1.32765384	2.509941675	-0.240524802	0.846437352	0.041704493	2.965301177
NM_001004431	meteorin, glial cell differentiation regulator-like	METRNL	2.210971575	4.629869645	-0.140213087	0.907385124	0.000417265	5.102430626
NM_002403	microfibrillar-associated protein 2	MFAP2 2	1.493051208	2.81483666	0.515940069	1.429925585	0.024276105	1.968519684
NM_022736	major facilitator superfamily domain containing 1	MFSD1	3.998863975	15.98740604	-0.056479278	0.961607945	0.006796673	16.62570085
NM_002406	mannosyl alpha-1,3--glycoprotein beta-1,2-N-acetylglucosaminyltransferase	MGAT1	1.368638326	2.582267262	-0.130723138	0.913373515	0.001185892	2.827175542
NM_002408	mannosyl alpha-1,6--glycoprotein beta-1,2-N-acetylglucosaminyltransferase	MGAT2	2.315192582	4.976710898	-0.090532804	0.939175837	0.002143796	5.299019315
NM_032350	hypothetical protein MGC11257	MGC11257	1.050382851	2.071079381	-0.600231571	0.659648065	0.000296296	3.139673245
NM_032889	hypothetical protein MGC11308	MGC11308	1.045779849	2.064482013	0.316212395	1.245057521	0.033560175	1.658141875
NM_001001437	chemokine C-C motif ligand 3-like, centromeric	MGC12815	3.05733631	8.324342413	-0.506358896	0.70399696	0.000496879	11.82440108
NM_031465	hypothetical protein MGC13204	MGC13204	1.537647875	2.903207874	-0.222759097	0.856925033	0.022606345	3.387936823
NM_032906	hypothetical protein MGC14156	MGC14156	1.310761194	2.480723933	-0.376067666	0.770534967	0.024277833	3.219482617
NM_053045	hypothetical protein MGC14327	MGC14327	1.922019306	3.789530993	0.227490435	1.170796579	0.032279774	3.236711707
NM_032369	hypothetical protein MGC15619	MGC15619	3.264975618	9.612925936	-0.487459661	0.713279957	0.042484959	13.47707284
NM_020314	esophageal cancer associated protein	MGC16824	2.987367895	7.930258509	-1.159792792	0.447576814	0.000508589	17.71820669
NM_138417	hypothetical protein BC012173	MGC20419	1.101384841	2.145605501	-0.734117633	0.601185604	0.000918859	3.568956888
NM_052849	hypothetical protein MGC20481	MGC20481	1.888897406	3.703520712	0.65041818	1.569623101	0.007041023	2.359496818
NM_145045	hypothetical protein MGC20983	MGC20983	1.394376737	2.628749622	0.15157539	1.110781758	0.000613131	2.366576154
CO774990		MGC23909	1.921096824	3.787108681	-0.305126758	0.8093711	0.001072496	4.67907574
NM_024099	hypothetical protein MGC2477	MGC2477	1.369538881	2.583879662	0.020347655	1.014203849	0.007708312	2.54769262
NM_173824	hypothetical protein MGC26717	MGC26717	1.354950055	2.557882609	-0.110530615	0.926247331	0.021110522	2.761554635
NM_031298	hypothetical protein MGC2963	MGC2963	1.17568097	2.258994832	-0.024580277	0.983106572	0.006145992	2.297812766
NM_182565	hypothetical protein MGC29814	MGC29814	1.344887027	2.54010305	0.145423463	1.106055257	0.005222794	2.29654263
NM_144580	kidney predominant protein NCU-G1	MGC31963	1.335291115	2.523263924	0.159121274	1.116606821	0.000310313	2.259760442
NM_024028	hypothetical protein MGC3265	MGC3265	1.903065691	3.740071085	0.000279241	1.000193574	0.048283427	3.739347244
NM_152346	hypothetical protein MGC34680	MGC34680	1.177750862	2.262238227	-0.335140977	0.792706671	0.007863437	2.853815049
NM_152318	hypothetical protein MGC40397	MGC40397	1.049571145	2.069914454	-0.528229499	0.693405171	0.000397151	2.985144242
XM_373742	hypothetical protein MGC40489	MGC40489	1.737258097	3.334009227	-0.127327037	0.915526127	0.000650625	3.641631984
NM_016466	hypothetical protein MGC41816	MGC41816	1.317022764	2.491514143	-0.094166428	0.93681337	0.040143177	2.659562963

NM_032315	mitochondrial carrier protein	MGC4399	1.310642255	2.480519425	-1.259213096	0.417771767	0.012890381	5.937498941
NM_033309	hypothetical protein MGC4655	MGC4655	1.308681943	2.477151221	-0.268892872	0.829956212	0.035816561	2.984677004
NM_052871	hypothetical protein MGC4677	MGC4677	1.935097398	3.824039427	0.281382504	1.215358979	0.009357301	3.146427922
CO581027		MGC49942	2.206339269	4.615027577	-0.567630852	0.674723889	0.0047405	6.839875761
NM_145058	hypothetical protein MGC7036	MGC7036	1.711129195	3.274169919	-0.242339864	0.845373114	0.000444094	3.873047136
NM_016127	hypothetical protein MGC8721	MGC8721	1.349119473	2.547565911	-0.178766327	0.883458133	3.13E-06	2.883629473
DV769814		MGP	1.011650039	2.016215768	-0.234956713	0.849710494	0.000361389	2.372826724
NM_020300	microsomal glutathione S-transferase 1	MGST1	1.082890727	2.11827622	-0.10107393	0.932338708	0.005322714	2.272002869
NM_002413	microsomal glutathione S-transferase 2	MGST2	1.749643928	3.362755595	0.191689863	1.142100702	0.003773034	2.944359975
CO581416		MGST3	1.708508198	3.268227015	-0.312148874	0.805441172	0.008243005	4.057685562
NM_012216	midline 2	MID2 1	1.527905712	2.883669273	0.629206628	1.546714185	0.017477088	1.86438406
NM_021242	MID1 interacting G12-like protein	MIG12	1.444577702	2.721831396	-0.28428725	0.821147189	0.00124519	3.314669323
NM_004897	multiple inositol polyphosphate histidine phosphatase, 1	MINPP1	2.496328238	5.642475458	0.027629706	1.019336018	0.012451528	5.535442049
NM_170784	McKusick-Kaufman syndrome	MKKS 2	1.522016281	2.871921432	-0.203676172	0.868335112	0.003700119	3.307388347
NM_013446	makorin, ring finger protein, 1	MKRN1	1.498039542	2.824586225	-0.645277996	0.639369577	2.59E-05	4.417767633
NM_023009	MARCKS-like protein	MLP	1.844526003	3.591349362	-0.019156723	0.986809341	0.020065446	3.639354851
NM_002421	matrix metalloproteinase 1 interstitial collagenase	MMP1	5.490153635	44.94702225	0.161571464	1.118504813	0.004115795	40.18491627
NM_004995	matrix metalloproteinase 14 membrane-inserted	MMP14	1.67819072	3.20026355	-0.651089647	0.636799167	0.013987242	5.025546072
NM_004530	matrix metalloproteinase 2 gelatinase A, 72kDa gelatinase, 72kDa type IV collagenase	MMP2	1.438186137	2.709799552	-0.975784711	0.508463207	0.003098138	5.329391611
NM_022468	matrix metalloproteinase 25	MMP25	1.778416955	3.430495454	-0.680926676	0.623764488	0.020989099	5.499664573
NM_002423	matrix metalloproteinase 7 matrilysin, uterine	MMP7	1.748108225	3.359177953	-0.042934618	0.97067846	0.046832355	3.460649528
NM_004994	matrix metalloproteinase 9 gelatinase B, 92kDa gelatinase, 92kDa type IV collagenase	MMP9	4.752640851	26.9579867	0.429746402	1.34699678	1.37E-05	20.013401
AF184160	alpha-defensin 1A	MNP1A	4.318305729	19.94984634	-0.511742834	0.701374637	0.018986829	28.44392325
NM_002436	membrane protein, palmitoylated 1, 55kDa	MPP1	2.290468583	4.892149808	-0.174066216	0.886341017	0.000363523	5.519489353
NM_015488	myofibrillogenesis regulator 1	MR-1	2.491645446	5.624190439	-0.135831954	0.910144832	0.022387065	6.179445559
NM_002438	mannose receptor, C type 1	MRC1	2.264726654	4.805633594	-0.928243676	0.525497689	0.000652763	9.144918612
NM_033546	myosin regulatory light chain MRLC2	MRLC2	1.15690054	2.229778711	0.086130144	1.061518963	0.000216366	2.10055476
NM_002949	mitochondrial ribosomal protein L12	MRPL12	1.029611647	2.041474641	-0.200262027	0.870392466	0.008118177	2.345464514
NM_014078	mitochondrial ribosomal protein L13	MRPL13	1.119443153	2.172630978	0.338237276	1.264211002	0.025163447	1.718566738
NM_014175	mitochondrial ribosomal protein L15	MRPL15	1.534933177	2.897750088	-0.66179429	0.632091671	0.004062251	4.584382647
NM_014763	mitochondrial ribosomal protein L19	MRPL19	1.099460187	2.142745024	-0.020343771	0.985997729	0.004376252	2.173174401
NM_181512	mitochondrial ribosomal protein L21	MRPL21	1.898672424	3.728699217	0.165059282	1.121212149	0.013857393	3.325596518
NM_014180	mitochondrial ribosomal protein L22	MRPL22	1.178538487	2.263473611	0.260093399	1.197556231	0.026449083	1.890077103
NM_023937	mitochondrial ribosomal protein L34	MRPL34	2.233774015	4.703628164	-0.246951718	0.842675031	0.02160651	5.581781818
NM_032477	mitochondrial ribosomal protein L41	MRPL41	1.289253227	2.444015149	-0.209678182	0.864730103	2.19E-05	2.82633291
NM_019051	mitochondrial ribosomal protein L50	MRPL50	2.341182687	5.067178627	-0.399649213	0.758042576	0.006876235	6.684556758
NM_031420	mitochondrial ribosomal protein L9	MRPL9	1.018581694	2.025926303	-0.166634748	0.890918429	0.000303163	2.273975076
NM_018141	mitochondrial ribosomal protein S10	MRPS10	1.149571487	2.218479908	-0.346277238	0.78661127	0.001119661	2.82030018
NM_176805	mitochondrial ribosomal protein S11	MRPS11	1.412276234	2.661567647	-0.134786818	0.91080441	0.001740946	2.922216469
NM_021107	mitochondrial ribosomal protein S12	MRPS12	1.037890657	2.05322347	-0.41884227	0.748024656	0.001350181	2.744860683
NM_016034	mitochondrial ribosomal protein S2	MRPS2	1.644570971	3.12654866	-0.350670837	0.78421936	0.009204217	3.986829222
CO580437		MRPS23	1.691368712	3.229629595	-0.128016087	0.915088964	0.015425352	3.52930668
NM_016071	mitochondrial ribosomal protein S33	MRPS33	1.333486194	2.520109103	-0.056121383	0.961846525	0.000484358	2.620074031

NM_021821	mitochondrial ribosomal protein S35	MRPS35	3.036061152	8.202485579	0.56426654	1.478635586	0.015190474	5.54733408
NM_015971	mitochondrial ribosomal protein S7	MRPS7	1.391825614	2.624105301	-0.498349588	0.707916159	0.000370139	3.706802378
NM_152851	membrane-spanning 4-domains, subfamily A, member 6A	MS4A6A	3.248649598	9.504756043	0.208096428	1.155162991	0.013228861	8.228064884
NM_005823	mesothelin	MSLN 1	1.87083924	3.657452783	0.098546029	1.070693856	0.030169186	3.415965042
NM_012331	methionine sulfoxide reductase A	MSRA	2.113849321	4.32844648	0.264782615	1.201455001	0.019223366	3.602670492
NM_175617	metallothionein 1E	MT1E	2.128022521	4.371179192	-0.243982854	0.844410923	0.018790061	5.176601905
NM_005949	metallothionein 1F	MT1F	2.135305761	4.39330226	0.120750179	1.087300095	0.009593347	4.040560909
NM_005950	metallothionein 1G	MT1G	1.148213843	2.216393195	0.048266639	1.034021826	0.028596761	2.143468483
CN801994		MT1X	3.866478673	14.58565907	-0.101688229	0.931941803	3.50E-05	15.65082607
NM_032935	metallothionein IV	MT4	2.676199028	6.39169704	0.230509057	1.173248859	2.31E-05	5.447861289
NM_014342	mitochondrial carrier homolog 2	MTCH2	1.72909924	3.315207657	-0.240272819	0.846585205	0.011538714	3.915976371
NM_014221	mature T-cell proliferation 1	MTCP1	1.93104794	3.813320894	-0.460280061	0.726845147	0.032189541	5.246400707
NM_005956	methylenetetrahydrofolate dehydrogenase NADP+ dependent 1, methenyltetrahydrofolate cyclohydrolase, formyltetrahydrofolate synthetase	MTHFD1	1.476504724	2.782737308	-0.332659229	0.794071472	0.003646284	3.504391489
NM_015440	methylenetetrahydrofolate dehydrogenase NADP+ dependent 1-like	MTHFD1L	3.001057223	8.00586464	-0.121557692	0.919194651	0.02356207	8.709651029
NM_006636	methylenetetrahydrofolate dehydrogenase NADP+ dependent 2, methenyltetrahydrofolate cyclohydrolase	MTHFD2	3.336949829	10.10466669	-0.151898409	0.900065308	0.000629615	11.22659279
NM_016498	mitochondrial protein 18 kDa	MTP18	1.392595526	2.625506061	0.168149181	1.123616084	0.00438106	2.336657599
NM_002456	mucin 1, transmembrane	MUC1	1.12651435	2.183306008	-0.443328972	0.735435656	0.007967984	2.968724716
NM_152673	mucin 20	MUC20	1.865838412	3.644796867	0.009978208	1.00694034	0.00091893	3.619675091
NM_018406	mucin 4, tracheobronchial	MUC4	1.381283548	2.605000316	-0.246388816	0.843003884	0.006989118	3.090140348
NM_017458	major vault protein	MVP 1	1.559362992	2.947236823	-0.337581121	0.791367037	1.18E-05	3.724235008
NM_138373	myeloid-associated differentiation marker	MYADM	3.141723913	8.825780781	-0.25739132	0.836599291	0.0089758	10.54959152
NM_002466	v-myb myeloblastosis viral oncogene homolog	MYBL2	2.601390791	6.068713826	0.584960246	1.499997655	0.004128543	4.045815541
NM_002467	v-myc myelocytomatosis viral oncogene homolog	MYC	1.504407377	2.83708107	-0.288680533	0.818650442	0.004212659	3.46555859
NM_002468	myeloid differentiation primary response gene	MYD88	1.433986773	2.701923396	-0.04760679	0.967539999	0.00445814	2.792570229
NM_018946	N-acetylneuraminic acid synthase	NANS	1.574092468	2.977481343	-0.476885229	0.718527247	0.0002286	4.143866994
NM_005437	nuclear receptor coactivator 4	NCOA4	1.638081907	3.11251741	0.287452815	1.22048352	0.001975588	2.55023305
NM_181782	nuclear receptor coactivator 7	NCOA7	1.159985013	2.234551064	-0.352898887	0.783009173	0.000127618	2.853799344
NM_004544	NADH dehydrogenase ubiquinone 1 alpha subcomplex, 10, 42kDa	NDUFA10	2.666566232	6.349162188	-1.371310732	0.386539906	0.034402955	16.42563186
NM_002488	NADH dehydrogenase ubiquinone 1 alpha subcomplex, 2, 8kDa	NDUFA2	1.622756673	3.079629238	0.005900647	1.004098392	0.001709216	3.067059226
NM_005000	NADH dehydrogenase ubiquinone 1 alpha subcomplex, 5, 13kDa	NDUFA5	1.535528765	2.898946612	0.005679806	1.003944701	0.004661407	2.887556065
NM_005002	NADH dehydrogenase ubiquinone 1 alpha subcomplex, 9, 39kDa	NDUFA9	1.153551761	2.224608958	0.066303979	1.04703087	0.001390948	2.124683255
NM_002491	NADH dehydrogenase ubiquinone 1 beta subcomplex, 3, 12kDa	NDUFB3	1.652376925	3.143511247	0.422452221	1.340203631	0.023529921	2.345547478
NM_002492	NADH dehydrogenase ubiquinone 1 beta subcomplex, 5, 16kDa	NDUFB5	1.152755201	2.223381016	-0.269682076	0.829502321	0.009328341	2.680379498
CN647252		NDUFS6	1.335905085	2.524337983	-0.2942284	0.815508376	0.002213624	3.095416378
NM_004553	NADH dehydrogenase ubiquinone Fe-S protein 6, 13kDa	NDUFS6	2.076234779	4.217051904	-0.131651682	0.912785841	0.009065863	4.619979534

NM_021075	NADH dehydrogenase ubiquinone flavoprotein 3, 10kDa	NDUFV3	2.34813726	5.091664147	-0.861302441	0.550455392	0.00453274	9.249912388
CN643443	NECAP endocytosis associated	NECAP1	1.763106369	3.394281855	-0.910436783	0.532023994	0.002840221	6.379941308
NM_006164	nuclear factor erythroid-derived 2-like 2	NFE2L2	1.894079011	3.716846249	-0.722849502	0.605899533	9.41E-05	6.134426666
NM_004289	nuclear factor erythroid-derived 2-like 3	NFE2L3	2.36131381	5.138380797	-0.439541837	0.737368741	0.022426073	6.968536245
NM_005384	nuclear factor, interleukin 3 regulated	NFIL3	2.442412199	5.435497917	-0.972320602	0.509685564	0.003948679	10.6644141
NM_020529	nuclear factor of kappa light polypeptide gene enhancer in B-cells inhibitor, alpha	NFKBIA	1.287105319	2.440379165	0.348985579	1.273664744	0.001139627	1.916029455
NM_004556	nuclear factor of kappa light polypeptide gene enhancer in B-cells inhibitor, epsilon	NFKBIE	2.2850093	4.873672472	-0.05185849	0.964692804	0.003415757	5.052046051
NM_018297	N-glycanase 1	NGLY1	2.390633566	5.243875982	-1.229365156	0.426505084	0.032379956	12.29499056
CN801572		NH1	1.479188141	2.787918025	0.379378821	1.300781659	0.044518692	2.143263633
NM_004148	ninjurin 1	NINJ1	2.685244994	6.43190008	-0.054436549	0.962970462	3.31E-05	6.679228837
NM_001008860	non imprinted in Prader-Willi/Angelman syndrome 2	NIPA2	1.642720649	3.12254129	-0.196895753	0.872425744	0.013784664	3.579148498
CO644910		NMES1	3.803599143	13.9636011	-2.773799167	0.146218811	0.044695667	95.49797989
NM_004688	N-myc and STAT interactor	NMI	1.70576212	3.26201207	0.245064996	1.185146162	0.000846719	2.752413309
XR_013712	Alpha-enolase 2-phospho-D-glycerate hydro-lyase	NNE	2.297151681	4.914864631	-0.463068749	0.725441534	5.61E-06	6.774997576
NM_014062	nin one binding protein	NOB1P	1.42794562	2.690632996	-0.113152759	0.924565376	0.030881617	2.910159807
NM_018648	nucleolar protein family A, member 3 H/ACA small nucleolar RNPs	NOLA3	1.352372283	2.553316329	0.421343697	1.339174254	0.036658748	1.906634869
NM_004741	nucleolar and coiled-body phosphoprotein 1	NOLC1	1.782286107	3.439708021	-0.375363287	0.770911264	0.003944617	4.461872828
NM_005693	nuclear receptor subfamily 1, group H, member 3	NR1H3	1.857781713	3.624499307	-0.141114059	0.906818633	0.007200831	3.996939602
NM_002525	nardilysin N-arginine dibasic convertase	NRD1	1.060616313	2.085822386	-0.188779241	0.877347789	0.000252699	2.377417954
NM_003872	neuropilin 2	NRP2	1.342688258	2.536234703	0.040192715	1.028251171	0.004574996	2.466551727
NM_004559	nuclease sensitive element binding protein 1	NSEP1	1.046798287	2.065939902	-0.242686743	0.845169879	7.25E-05	2.444407868
CR936794	neurotrophic tyrosine kinase, receptor, type 1	NTRK1	1.997627519	3.993427491	0.099686217	1.07154038	0.01486431	3.726810082
NM_015332	NudC domain containing 3	NUDCD3	1.85722289	3.623095641	-0.578631966	0.669598422	0.023234459	5.410848532
NM_016320	nucleoporin 98kDa	NUP98	1.087273815	2.124721594	-0.58281607	0.667659266	0.04492167	3.182344199
CN647507		NXT2	1.276463337	2.422444036	0.31600459	1.244878196	0.003482896	1.945928561
NM_016816	2',5'-oligoadenylate synthetase 1, 40/46kDa	OAS1	2.325216809	5.011410817	-0.268577687	0.830137552	0.024618632	6.036843898
NM_000274	ornithine aminotransferase	OAT	1.133194483	2.193438844	-0.996862764	0.501088466	0.04905024	4.377348496
NM_015878	ornithine decarboxylase antizyme inhibitor	OAZIN	1.9295265	3.809301554	-0.091551377	0.938512993	0.008038074	4.058869279
NM_013370	pregnancy-induced growth inhibitor	OKL38	1.741081919	3.342857644	-0.396119873	0.759899286	0.002905675	4.399079859
NM_020190	olfactomedin-like 3	OLFML3	1.333250896	2.519698118	-0.036883343	0.974758447	0.00074363	2.584946173
NM_000607	orosomucoid 1	ORM1	3.8643475	14.5641288	-0.009916242	0.993150152	0.001243468	14.66457893
NM_005109	oxidative-stress responsive 1	OSR1	1.450558306	2.733138	-1.544563594	0.34279938	0.048033487	7.972995748
NM_012383	osteoclast stimulating factor 1	OSTF1	1.249467733	2.377536901	-0.378840403	0.769055487	0.004628886	3.091502422
NM_148962	oxoeicosanoid OXE receptor 1	OXER1	1.618621958	3.070815761	0.799638894	1.740665384	0.048540695	1.764162021
NM_002560	purinergic receptor P2X, ligand-gated ion channel, 4	P2RX4 1	1.709936229	3.271463624	-0.17785491	0.884016431	8.75E-05	3.70068192
NM_002561	purinergic receptor P2X, ligand-gated ion channel, 5	P2RX5 1	3.029327967	8.164293049	0.412332113	1.330835368	0.000105805	6.13471301
NM_176798	pyrimidinergic receptor P2Y, G-protein coupled, 6	P2RY6	2.929139095	7.616557572	-0.290818606	0.817438101	0.000112831	9.317595503
NM_000917	procollagen-proline, 2-oxoglutarate 4-dioxygenase proline 4-hydroxylase, alpha polypeptide I	P4HA1	2.924325797	7.591188608	-0.249218426	0.841352091	0.019822873	9.022606221
NM_002568	poly A binding protein, cytoplasmic 1	PABPC1	1.239133444	2.360567021	-0.492500586	0.710792032	0.001944416	3.321037539
XR_010782	peptidyl arginine deiminase, type IV	PADI4	1.946739935	3.855024248	-0.083789378	0.943575996	0.022056997	4.08554718
NM_002578	p21 CDKN1A-activated kinase 3	PAK3	1.274211228	2.418665447	-0.323774231	0.79897694	0.046615454	3.027203073

NM_153638	pantothenate kinase 2	PANK2 1	1.480575407	2.790600119	-0.562286119	0.677228166	0.017268662	4.120620287
NM_015368	pannexin 1	PANX1	1.672061367	3.186695933	0.184214406	1.136198108	0.02582334	2.804700968
NM_152911	polyamine oxidase exo-N4-amino	PAOX 1	1.998543519	3.995963814	-0.47383921	0.720045905	0.005473952	5.54959592
CN644296		PARK7	1.238381194	2.359336495	-0.19043077	0.876344018	0.000848078	2.692249217
NM_017554	poly ADP-ribose polymerase family, member 14	PARP14	2.101542861	4.291681048	0.204057327	1.151933415	0.000374373	3.725632917
NM_001003828	parvin, beta	PARVB	3.009103678	8.050641117	-0.513586475	0.700478912	0.015436088	11.49305279
NM_022141	parvin, gamma	PARVG	3.358732143	10.25838803	-0.565050994	0.675931525	0.001640937	15.17666754
NM_005746	pre-B-cell colony enhancing factor 1	PBEF1	1.351478821	2.551735546	-2.057422546	0.240244857	0.031750352	10.62139509
XR_014700	Pituitary tumor-transforming gene 1 protein-interacting protein precursor	PBF	1.278671387	2.426154434	-0.869401888	0.547373734	6.68E-05	4.432354506
NM_006196	poly	PCBP1	1.068288189	2.096943789	-0.790973698	0.57795389	0.002274583	3.628219872
NM_000282	propionyl Coenzyme A carboxylase, alpha polypeptide	PCCA	1.253026103	2.383408273	-0.640891182	0.641316672	0.000424912	3.716429614
NM_002592	proliferating cell nuclear antigen	PCNA	2.383740263	5.218880119	-0.071235417	0.951822579	0.000513938	5.48303879
NM_002593	procollagen C-endopeptidase enhancer	PCOLCE	1.716711458	3.286863303	-0.91746491	0.529438528	0.000105423	6.208205735
NM_007217	programmed cell death 10	PDCD10	1.565594529	2.959994569	-0.050852496	0.965365721	0.002990373	3.066189845
NM_002598	programmed cell death 2	PDCD2	1.676533122	3.196588689	-0.362878683	0.777611423	0.001683226	4.110778977
NM_002608	platelet-derived growth factor beta polypeptide	PDGFB	1.283892643	2.434950836	0.362461447	1.285617477	0.02271808	1.893993259
NM_003477	pyruvate dehydrogenase complex, component X	PDHX	2.052195934	4.147367625	-0.00427459	0.997041465	0.008950067	4.159674167
NM_213636	PDZ and LIM domain 7	PDLIM7	1.346843985	2.543550936	-0.081738779	0.944918117	0.009412494	2.691821536
NM_003768	phosphoprotein enriched in astrocytes 15	PEA15	1.992752609	3.979956353	-1.226019698	0.427495252	0.030564513	9.309942819
NM_000442	platelet/endothelial cell adhesion molecule CD31 antigen	PECAM1	1.244914972	2.370045855	-0.542992031	0.686346007	3.74E-05	3.453135635
NM_000285	peptidase D	PEPD	1.708805166	3.268899823	-0.173832279	0.886484752	0.026665875	3.687485675
NM_002620	platelet factor 4 variant 1	PF4V1	1.633723411	3.103128439	-0.017116974	0.988205524	0.024319639	3.140165039
NM_002626	phosphofructokinase, liver	PFKL	1.847850388	3.599634406	-0.22269004	0.856966052	0.006825086	4.200439908
NM_002627	phosphofructokinase, platelet	PFKP	2.814666213	7.035564648	0.207110917	1.154374164	0.00044575	6.094700372
NM_016095	DNA replication complex GINS protein PSF2	Pfs2	1.654819771	3.148838512	0.316826466	1.245587582	0.047314722	2.527994465
NM_002629	phosphoglycerate mutase 1	PGAM1	2.411164567	5.319035136	-1.269954527	0.414672843	5.72E-05	12.8270641
NM_002631	phosphogluconate dehydrogenase	PGD	2.451336715	5.469226139	-0.184688929	0.879838765	0.000236768	6.216168642
NM_012088	6-phosphogluconolactonase	PGLS	1.470138166	2.770484252	-0.267449092	0.830787208	0.036424376	3.334769992
NM_018290	phosphoglucomutase 2	PGM2	1.394668515	2.629281327	-0.04180751	0.9714371	0.020665907	2.706589369
NM_006667	progesterone receptor membrane component 1	PGRMC1	1.799828416	3.481788128	-0.539339002	0.688086096	0.010644909	5.060105337
NM_032758	PHD finger protein 5A	PHF5A	4.038842531	16.43662889	-0.644789642	0.639586041	0.020739778	25.69885493
NM_015886	protease inhibitor 15	PI15	1.689504739	3.225459584	-0.955653732	0.515607898	0.035784269	6.255644252
NM_002642	phosphatidylinositol glycan, class C	PIGC	1.122653746	2.177471364	-0.58885061	0.664872397	0.023484632	3.275021451
NM_152309	phosphoinositide-3-kinase adaptor protein 1	PIK3AP1	4.291414999	19.58144055	-1.071802039	0.475724411	0.04236017	41.16131127
NM_002647	phosphoinositide-3-kinase, class 3	PIK3C3	1.183497361	2.271267076	0.355675382	1.279584465	0.022118907	1.775003634
NM_013439	paired immunoglobin-like type 2 receptor alpha	PILRA	1.154812746	2.226554223	-0.411819367	0.751674847	0.001318377	2.962124159
NM_002652	prolactin-induced protein	PIP	4.070270498	16.7986163	0.376409666	1.298107324	0.011558344	12.94085319
XR_011777	pyruvate kinase 3	PKM2	1.519101716	2.866125368	-1.047864672	0.483683533	1.57E-05	5.925621136
NM_004572	plakophilin 2	PKP2	1.123446824	2.178668692	0.030919286	1.021662922	0.002094326	2.132473093
NM_015900	phospholipase A1 member A	PLA1A	2.360488948	5.135443762	-0.594668544	0.662196576	0.001794674	7.755165079
NM_003706	phospholipase A2, group IVC cytosolic, calcium-independent	PLA2G4C	2.76169494	6.781925519	-0.209551299	0.864806158	1.05E-05	7.842133705
NM_000930	plasminogen activator, tissue	PLAT	1.903155532	3.740303999	-0.902214748	0.535064698	0.012166496	6.990377077

NM_002658	plasminogen activator, urokinase	PLAU	4.135506598	17.57565557	-0.360389148	0.778954439	0.014171677	22.56313681
NM_002661	phospholipase C, gamma 2 phosphatidylinositol-specific	PLCG2	1.603624199	3.039057979	-0.324578275	0.798531777	0.004554777	3.805807192
NM_002664	pleckstrin	PLEK	2.391408563	5.24669368	-1.335089138	0.396367578	0.02788564	13.23693959
NM_017958	pleckstrin homology domain containing, family B evectins member 2	PLEKHB2	3.803800732	13.96555237	-5.549720041	0.021348521	0.006459953	654.169539
NM_021105	phospholipid scramblase 1	PLSCR1	2.326045934	5.014291728	-0.308053623	0.807730755	0.000895831	6.207875207
NM_000303	phosphomannomutase 2	PMM2	1.731074905	3.319750701	-0.451703483	0.731178988	0.003926937	4.540270926
NM_006029	paraneoplastic antigen MA1	PNMA1	1.403705411	2.645802569	-0.889298618	0.539876521	0.02094028	4.900755014
NM_021173	polymerase DNA-directed, delta 4	POLD4	1.063643736	2.090203974	-0.349038715	0.78510705	7.69E-05	2.662317163
NM_032311	polymerase DNA-directed, delta interacting protein 3	POLDIP3	1.881328784	3.684142293	-0.367298756	0.775232653	0.008456775	4.75230536
NM_006596	polymerase DNA directed, theta	POLQ 1	4.364645633	20.60104525	-1.797807272	0.287611393	0.027021346	71.6280571
NM_032940	polymerase RNA II DNA directed polypeptide C, 33kDa	POLR2C	1.080110833	2.114198496	-0.814206765	0.5687211	0.029810751	3.717460978
NM_002696	polymerase RNA II DNA directed polypeptide G	POLR2G	2.349484041	5.096419523	0.25834428	1.196105197	0.023385191	4.260845564
CN802953		POLR2K	1.240619678	2.363000079	-0.229712995	0.852804529	0.005818526	2.770857797
NM_006627	processing of precursor 4, ribonuclease P/MRP subunit	POP4	2.392410228	5.25033773	-0.65439169	0.635343326	0.00794462	8.263780403
NM_006475	periostin, osteoblast specific factor	POSTN	1.457198653	2.745746915	-1.647594921	0.319171796	0.014672828	8.602724131
NM_006235	POU domain, class 2, associating factor 1	POU2AF1	2.489925938	5.617491117	-0.086170179	0.942020148	0.015950233	5.963238824
NM_021129	pyrophosphatase	PP	2.803719598	6.982383501	0.054658278	1.038613064	8.79E-05	6.722795756
NM_022152	PP1201 protein	PP1201	1.510021448	2.848142733	-0.476762261	0.718588493	0.004706962	3.963523991
CN644776		PP2135	1.204988629	2.305354523	-0.370697333	0.773408576	0.001688811	2.980771865
NM_145201	CG3714 gene product	PP3856	1.224835145	2.337287403	-0.576509794	0.670584111	0.000366747	3.485450018
NM_176895	phosphatidic acid phosphatase type 2A	PPAP2A	1.269880919	2.411416608	0.472927288	1.387922765	0.018553067	1.737428528
NM_000308	protective protein for beta-galactosidase / galactosialidosis	PPGB	1.684506671	3.214304645	-0.283435241	0.821632275	7.15E-06	3.912096375
NM_000942	peptidylprolyl isomerase B cyclophilin B	PPIB	1.088017426	2.125817026	-0.165199224	0.89180536	3.32E-05	2.383723087
NM_005729	peptidylprolyl isomerase F cyclophilin F	PPIF	3.197131218	9.171331612	-0.132088237	0.912509677	0.005727185	10.05066778
NM_152329	peptidylprolyl isomerase cyclophilin-like 5	PPIL5 1	2.353526265	5.110718979	0.075267757	1.053556553	0.033920301	4.850920404
CN648627		PPP1R11	2.482997695	5.590578957	-1.345518953	0.393512412	0.007544303	14.20686815
NM_000310	palmitoyl-protein thioesterase 1	PPT1	2.453324295	5.476766204	-0.981527345	0.506443297	0.000801022	10.81417453
NM_199418	prolylcarboxypeptidase angiotensinase C	PRCP 2	1.916771734	3.775772218	0.003181648	1.002207784	0.000665899	3.767454492
NM_001198	PR domain containing 1, with ZNF domain	PRDM1	1.973002099	3.925841966	-0.037168531	0.974565779	0.021729322	4.028298604
NM_006793	peroxiredoxin 3	PRDX3	1.579384605	2.988423485	-0.186229037	0.878900019	0.000264843	3.400185938
NM_006406	peroxiredoxin 4	PRDX4	1.331251533	2.516208605	0.13052401	1.094691239	0.002334599	2.298555535
NM_005041	perforin 1	PRF1	1.368288176	2.581640608	0.037274002	1.026173022	0.005377304	2.51579466
NM_002727	proteoglycan 1, secretory granule	PRG1	3.469233999	11.0749939	0.453827887	1.369669569	0.000144777	8.08588739
NM_005044	protein kinase, X-linked	PRKX	1.943024298	3.845108474	-0.674743736	0.626443484	0.005577518	6.137997398
NM_000311	prion protein p27-30	PRNP 1	2.748098591	6.718311043	-0.488612562	0.712710181	0.025207161	9.426427776
NM_018509	hypothetical protein PRO1855	PRO1855	1.141521874	2.206136219	-0.211029082	0.863920772	0.011896345	2.553632566
NM_006404	protein C receptor, endothelial	PROCR	2.909484805	7.513498398	0.444833635	1.361157145	0.008719577	5.519934582
NM_016307	paired related homeobox 2	PRRX2	1.169879536	2.249929095	-0.779794436	0.582449778	0.012598145	3.862872264
NM_002775	protease, serine, 11	PRSS11	1.413437648	2.663711152	-0.123275253	0.91810098	0.000162665	2.901326988
CO579438		PSAP	1.535459977	2.898808395	-0.146869248	0.903208365	8.59E-06	3.209456985
CK232488		PSG4	3.250306108	9.515675722	0.287602658	1.22061029	0.000822341	7.795834425
CK230740		PSG5	1.331831709	2.517220695	0.138734414	1.100938907	0.000642525	2.286430862
CK231506		PSG5	1.037304328	2.052389185	-0.217216446	0.860223562	4.85E-06	2.385878828

CK231580		PSG5	1.184967945	2.27358343	-0.069781484	0.952782299	9.43E-06	2.386257
CK232214		PSG5	1.207778827	2.309817434	-0.74055697	0.598508246	0.000356222	3.859290908
CK230160		PSG5	1.628155068	3.091174436	-1.869251735	0.273715353	0.039541407	11.29339075
NM_148976	proteasome prosome, macropain subunit, alpha type, 1	PSMA1	2.089535868	4.256111269	-0.479124104	0.717413051	0.022346092	5.932581324
NM_002788	proteasome prosome, macropain subunit, alpha type, 3	PSMA3	1.373348164	2.590711136	0.810864179	1.754261937	0.014135863	1.476809752
CN646819		PSMA4	1.716713958	3.286869	-0.947898014	0.518387195	0.000150092	6.340567501
NM_002790	proteasome prosome, macropain subunit, alpha type, 5	PSMA5	1.775012461	3.422409664	0.013297773	1.009259924	0.000369213	3.391009177
NM_002791	proteasome prosome, macropain subunit, alpha type, 6	PSMA6	2.144486899	4.42134986	0.144502284	1.105349252	2.96E-05	3.999957344
NM_152255	proteasome prosome, macropain subunit, alpha type, 7	PSMA7	1.34374925	2.538100595	0.274111076	1.209248774	0.00377383	2.098906899
NM_002801	proteasome prosome, macropain subunit, beta type, 10	PSMB10	2.134404117	4.390557427	-0.141141721	0.906801246	0.000252624	4.841807887
NM_002794	proteasome prosome, macropain subunit, beta type, 2	PSMB2	1.950422789	3.864877772	0.386004376	1.306769218	0.001672702	2.957582501
NM_002797	proteasome prosome, macropain subunit, beta type, 5	PSMB5	3.665128656	12.68567742	0.108816271	1.078343094	0.010819097	11.76404568
NM_002798	proteasome prosome, macropain subunit, beta type, 6	PSMB6	1.450985264	2.733946979	-0.072818879	0.950778458	0.001438604	2.875482672
NM_004159	proteasome prosome, macropain subunit, beta type, 8	PSMB8	2.518099777	5.728271118	0.187098074	1.138471419	3.91E-07	5.031545827
NM_002800	proteasome prosome, macropain subunit, beta type, 9	PSMB9	2.922535355	7.581773486	-0.199271076	0.870990522	2.23E-05	8.70477152
NM_002803	proteasome prosome, macropain 26S subunit, ATPase, 2	PSMC2	1.074145853	2.105475158	-0.169424746	0.889197165	0.00032993	2.36783836
NM_002807	proteasome prosome, macropain 26S subunit, non-ATPase, 1	PSMD1	1.025174742	2.035205878	-0.389552903	0.763366138	0.005197603	2.666093997
NM_002814	proteasome prosome, macropain 26S subunit, non-ATPase, 10	PSMD10	2.285088116	4.873938734	-0.238013597	0.847911973	0.035813787	5.74816595
NM_005805	proteasome prosome, macropain 26S subunit, non-ATPase, 14	PSMD14	2.000711261	4.001972519	-0.059038576	0.959903594	0.041316709	4.169140053
CN646285		PSMD8	1.251752562	2.381305244	-0.211679193	0.863531558	0.006876497	2.757635459
NM_176783	proteasome prosome, macropain activator subunit 1	PSME1	1.214875798	2.321207987	-0.20199391	0.86934823	0.000343618	2.67005546
NM_002819	polypyrimidine tract binding protein 1	PTBP1	1.320909224	2.498235054	-0.649224568	0.637622937	0.005494599	3.918044522
NM_000958	prostaglandin E receptor 4	PTGER4	1.803157495	3.489831778	-0.259668293	0.835279946	0.006638989	4.178038504
NM_198797	prostaglandin E synthase	PTGES	1.634500437	3.104800212	0.097422023	1.069860002	0.003256874	2.902062146
NM_007284	PTK9L protein tyrosine kinase 9-like	PTK9L	1.180965802	2.267285081	0.165958944	1.121911553	0.003491887	2.0209125
NM_003463	protein tyrosine phosphatase type IVA, member 1	PTP4A1	1.601731739	3.035074099	-1.421769139	0.373254319	0.020031798	8.131383725
NM_002835	protein tyrosine phosphatase, non-receptor type 12	PTPN12	1.598047436	3.027333123	0.007675496	1.005334426	0.017209796	3.011269727
NM_014369	protein tyrosine phosphatase, non-receptor type 18	PTPN18	2.91896768	7.563047503	-1.102871824	0.465588773	0.001549535	16.24405041
NM_080422	protein tyrosine phosphatase, non-receptor type 2	PTPN2	1.030898961	2.043297059	-0.025614119	0.982402326	0.00483494	2.079898434
NM_080588	protein tyrosine phosphatase, non-receptor type 7	PTPN7	1.149509742	2.218384962	-0.136433386	0.909765489	0.006848667	2.438414062
NM_080792	protein tyrosine phosphatase, non-receptor type substrate 1	PTPNS1	1.463782343	2.758305672	-3.762674317	0.073675343	0.03751536	37.43865395
NM_002845	protein tyrosine phosphatase, receptor type, M	PTPRM	1.025736798	2.035998923	-0.839021266	0.559022685	0.006414148	3.642068518
NM_002852	pentaxin-related gene, rapidly induced by IL-1 beta	PTX3	2.427423756	5.379319788	-0.295256618	0.814927365	0.000364523	6.600980673
NM_013237	px19-like protein	PX19	1.769492255	3.409339465	0.021162369	1.014776749	0.001384584	3.35969411
NM_013258	PYD and CARD domain containing	PYCARD	1.64993779	3.138201067	0.232962449	1.175245738	0.000197574	2.670250965
NM_014402	low molecular mass ubiquinone-binding protein	QP-C	1.097671837	2.140090545	0.254169674	1.19264913	0.001224621	1.794400793
NM_004663	RAB11A, member RAS oncogene family	RAB11A	1.41952138	2.674967532	0.304567457	1.235048294	0.027235927	2.165880916
NM_021252	RAB18, member RAS oncogene family	RAB18	1.094036208	2.134704251	-0.088654392	0.940399455	0.038707272	2.269997328
NM_002865	RAB2, member RAS oncogene family	RAB2	2.122533766	4.35458057	-1.485491626	0.357126816	0.009300732	12.19337325
NM_017817	RAB20, member RAS oncogene family	RAB20	1.920300106	3.785017855	-1.356653234	0.390487093	0.002706946	9.693067778

NM_020387	RAB25, member RAS oncogene family	RAB25	1.274626642	2.419361985	-0.669561063	0.628697938	0.000260743	3.848210466
NM_004249	RAB28, member RAS oncogene family	RAB28	1.730898549	3.319344917	-0.912263391	0.53135082	0.013806519	6.246993118
NM_006868	RAB31, member RAS oncogene family	RAB31	1.263753595	2.401196707	-0.666682462	0.629953628	0.000464078	3.811703912
NM_006834	RAB32, member RAS oncogene family	RAB32	1.778604806	3.430942164	0.261833922	1.199001881	0.004911404	2.861498568
NM_022337	RAB38, member RAS oncogene family	RAB38	1.132088026	2.191757259	-1.440206964	0.368514435	0.009708352	5.947547919
NM_004251	RAB9A, member RAS oncogene family	RAB9A	1.2429834	2.36687481	-0.24016178	0.846650366	0.016428787	2.795575251
NM_013277	Rac GTPase activating protein 1	RACGAP1	1.593961925	3.018772266	-0.355638186	0.781523854	0.010140178	3.862674503
NM_002881	v-ral simian leukemia viral oncogene homolog B	RALB	1.861208095	3.633117676	-0.846662246	0.556069747	0.006058001	6.533564714
NM_016448	RA-regulated nuclear matrix-associated protein	RAMP	3.050598841	8.285557887	0.227181001	1.170545489	0.002508761	7.078373257
NM_021159	RAP1, GTP-GDP dissociation stimulator 1	RAP1GDS1	1.895236213	3.71982877	-0.187557499	0.878091083	0.000266681	4.23626756
NM_021033	RAP2A, member of RAS oncogene family	RAP2A	1.781510324	3.437858877	-0.054444278	0.962965303	0.005660711	3.570075541
NM_000965	retinoic acid receptor, beta	RARB 1	1.25111503	2.380253169	0.54575626	1.459785362	0.038216847	1.6305501
CO645773		RARRES3	1.658452717	3.156777803	-0.343826961	0.787948388	0.002140942	4.006325605
NM_002890	RAS p21 protein activator	RASA1 1	2.457289335	5.491839011	-0.168265857	0.889911727	0.014267531	6.171217711
NM_032023	Ras association	RASSF4 1	1.256815211	2.389676306	0.460232281	1.375763305	0.016962304	1.736982152
NM_002139	RNA binding motif protein, X-linked	RBMX	1.296116183	2.455669119	-1.51943257	0.348823086	0.002127692	7.039869829
NM_018191	regulator of chromosome condensation RCC1 and BTB POZ domain containing protein 1	RCBTB1	1.462468402	2.755794675	-0.465585098	0.724177321	0.006861035	3.805414219
NM_002901	reticulocalbin 1, EF-hand calcium binding domain	RCN1	1.830431335	3.556433863	0.016535085	1.011527179	0.000478206	3.515905392
XR_014069	Neutral amino acid transporter B Sodium-dependent neutral amino acid transporter type 2	RD114	1.265872498	2.404725964	0.143882878	1.104874785	0.02865976	2.176469223
NM_138412	retinol dehydrogenase 13	RDH13	1.034244233	2.048040483	-0.330462295	0.795281604	0.030672124	2.575239352
NM_002906	radixin	RDX	1.212580085	2.317517262	0.160363459	1.117568652	0.015586157	2.073713555
NM_006509	v-rel reticuloendotheliosis viral oncogene homolog B, nuclear factor of kappa light polypeptide gene enhancer in B-cells 3	RELB	1.269324648	2.410486999	0.094978568	1.068049541	0.00075788	2.256905607
NM_002910	renin binding protein	RENBP	1.827267125	3.548642216	-0.66731417	0.629677852	0.011196849	5.635647183
NM_007033	RER1 homolog	RER1	1.226168372	2.339448342	-0.294305182	0.815464975	0.005467215	2.868852022
NM_020415	resistin	RETN	1.215295401	2.3218832	0.027383174	1.019161846	0.006428129	2.278228143
NM_015523	small fragment nuclease	REXO2	1.296801831	2.456836466	0.418578762	1.336610175	0.001720861	1.838109952
NM_022457	ring finger and WD repeat domain 2	RFWD2	1.04214222	2.05928316	0.105414368	1.075803336	0.012800423	1.914181794
NM_002922	regulator of G-protein signalling 1	RGS1	3.393547275	10.50895473	-0.046958732	0.967974715	0.011525344	10.85664178
NM_002928	regulator of G-protein signalling 16	RGS16	2.589674758	6.019629769	-0.081266822	0.945227284	0.012563333	6.368446903
NM_005873	regulator of G-protein signalling 19	RGS19	2.088886717	4.254196632	0.031660938	1.022188268	0.000358562	4.161852338
NM_024599	rhomboid, veinlet-like 6	RHBDL6	2.052293529	4.147648193	-0.075406453	0.949074699	0.001214626	4.370202047
NM_001005498	rhomboid, veinlet-like 6	RHBDL6	2.420046324	5.351882062	0.048847724	1.034438391	0.000218983	5.173707885
NM_001664	ras homolog gene family, member A	RHOA	1.741609778	3.344080965	-1.030960818	0.489384116	0.000218479	6.833243775
NM_004310	ras homolog gene family, member H	RHOH	2.743515199	6.697001071	0.376406038	1.29810406	0.025134893	5.159063344
NM_012249	ras homolog gene family, member Q	RHOQ	1.490199343	2.809277893	-0.093643937	0.937152711	0.011138434	2.997673548
NM_021205	ras homolog gene family, member U	RHOU	3.898872983	14.91687042	-0.065909437	0.955342905	0.003325941	15.61415314
NM_018343	RIO kinase 2	RIOK2	1.582971698	2.995863096	-0.917334798	0.529486278	0.022582598	5.658056155
NM_003821	receptor-interacting serine-threonine kinase 2	RIPK2	2.167833334	4.493480478	-0.102509371	0.931411519	0.034329767	4.824377182
NM_006912	Ras-like without CAAX 1	RIT1	1.079020418	2.11260115	-0.209046344	0.8651089	0.005941168	2.442006029
NM_198232	ribonuclease, RNase A family, 1	RNASE1	1.86804729	3.650381607	-0.383801549	0.766415398	0.000198706	4.762928327
NM_002935	ribonuclease, RNase A family, 3	RNASE3	3.417989712	10.68851641	-0.298481199	0.813107948	0.036702385	13.14526126

NM_005615	ribonuclease, RNase A family, k6	RNASE6	2.067226339	4.190801935	-0.675103464	0.626287303	0.000279647	6.691500714
NM_007282	ring finger protein 13	RNF13 1	1.097352467	2.139616845	-0.268814718	0.830001174	0.00084184	2.577847975
NM_018434	ring finger protein 130	RNF130	1.428662524	2.691970358	-0.531607518	0.691783487	0.012090012	3.891348101
NM_173647	ring finger protein 149	RNF149	1.951044675	3.866544118	-0.009540681	0.993408722	0.000364216	3.892198682
DQ148160	clone ss1_g15_t7_375 ring finger protein 34	RNF34	1.128842575	2.186832278	0.265200659	1.201803192	0.024909309	1.819625952
CN806006		RNP24	1.671173788	3.184736007	-0.51223134	0.701137187	0.012882196	4.542243752
NM_005156	ROD1 regulator of differentiation 1	ROD1	1.152974623	2.223719199	-0.090638699	0.939106903	0.004643632	2.36790848
NM_002946	replication protein A2, 32kDa	RPA2	1.731781654	3.321377382	-0.203718981	0.868309347	0.01575108	3.825108406
NM_002947	replication protein A3, 14kDa	RPA3	1.325821055	2.506755096	-0.802323511	0.573424912	0.022596055	4.371548989
CO647725		RPE	1.178300443	2.263100171	-0.908258615	0.532827846	0.00711961	4.247338402
NM_001001	ribosomal protein L36a-like	RPL36AL	1.40600966	2.650031779	0.072696068	1.051680199	0.024021233	2.519807619
CK231337		RPL7	1.090115165	2.128910302	-0.527085181	0.693955385	0.000133226	3.067791312
CN641710		RPN2	1.4858776	2.800875002	-0.880184854	0.543297813	0.005414872	5.15532169
NM_007042	ribonuclease P 14kDa subunit	RPP14	1.656479657	3.152463479	-0.431706384	0.741384375	0.002847295	4.252130991
NM_003942	ribosomal protein S6 kinase, 90kDa, polypeptide 4	RPS6KA4	1.454803145	2.74119155	-0.206937317	0.866374498	0.000734451	3.16397996
CO649194		RRAGA	1.227307401	2.341296099	-0.067309766	0.954416066	0.002660126	2.453118909
NM_021244	Ras-related GTP binding D	RRAGD	2.938661848	7.666998236	0.393127538	1.313237214	0.046221266	5.838243201
NM_004587	ribosome binding protein 1 homolog 180kDa	RRBP1	1.590064604	3.010628308	-0.362329785	0.777907335	0.002275051	3.870163159
NM_001007279	RAS-related on chromosome 22	RRP22	1.243761802	2.368152195	0.475829346	1.390717456	0.032982551	1.702827691
NM_080388	S100 calcium binding protein A16	S100A16	1.382605179	2.607387812	-0.604938909	0.657499221	2.29E-05	3.965613536
NM_002964	S100 calcium binding protein A8 calgranulin A	S100A8	5.809318608	56.07627481	0.10892443	1.078423941	0.000765832	51.99835858
NM_002965	S100 calcium binding protein A9 calgranulin B	S100A9	2.115115917	4.332248253	-0.11233508	0.925089542	0.000441149	4.683058294
NM_005980	S100 calcium binding protein P	S100P	4.542154862	23.29833354	0.153497939	1.112262984	0.002387239	20.94678496
NM_000331	serum amyloid A1	SAA1	3.910305716	15.03554978	0.194362723	1.144218617	0.005087436	13.14045197
NM_152703	sterile alpha motif domain containing 9-like	SAMD9L	1.815982614	3.520993622	-0.503319127	0.70548185	9.85E-05	4.990906031
NM_022136	SAM domain, SH3 domain and nuclear localisation signals, 1	SAMSN1	1.551516026	2.931250017	-0.131804575	0.912689112	0.032875638	3.211663182
NM_003864	sin3-associated polypeptide, 30kDa	SAP30	2.896335629	7.445329145	-0.787031621	0.579535274	0.007543111	12.84706813
NM_013352	squamous cell carcinoma antigen recognized by T cells 2	SART2	1.741940671	3.344848043	-0.316340384	0.803104493	0.001267048	4.164897685
NM_005063	stearoyl-CoA desaturase delta-9-desaturase	SCD	2.162237025	4.476083735	-0.31016988	0.806546781	0.044704881	5.549688919
NM_054023	secretoglobin, family 3A, member 2	SCGB3A2	1.30019271	2.462617752	0.141253344	1.102862814	0.002634984	2.232932076
NM_002975	stem cell growth factor; lymphocyte secreted C-type lectin	SCGF	1.049106587	2.069248035	-0.007693981	0.994681135	0.010202999	2.08031294
NM_001037	sodium channel, voltage-gated, type I, beta	SCN1B a	1.640853249	3.118502141	-0.26564261	0.831828137	0.000725071	3.748974101
NM_005138	SCO cytochrome oxidase deficient homolog 2	SCO2	1.414043805	2.664830562	-0.07750314	0.947696398	0.004768438	2.811903229
NM_021626	serine carboxypeptidase 1	SCPEP1	1.138491708	2.201507422	-0.3998224	0.757951584	0.001890474	2.904548878
NM_001007067	syndecan binding protein	SDCBP	1.973307801	3.926673927	0.271825777	1.207334783	0.001502363	3.252348879
NM_005786	serologically defined colon cancer antigen 33	SDCCAG33	1.572781024	2.974775971	-0.28470367	0.820910207	1.52E-05	3.623753179
NM_022044	stromal cell-derived factor 2-like 1	SDF2L1	1.460177802	2.751422709	0.156937306	1.114917768	0.01176977	2.467825689
NM_003000	succinate dehydrogenase complex, subunit B, iron sulfur	SDHB	1.08273054	2.118041034	-0.012088091	0.991656178	0.002634709	2.135862289
NM_003002	succinate dehydrogenase complex, subunit D, integral membrane protein	SDHD	1.119798408	2.173166041	-0.485659765	0.714170395	0.00066343	3.042923728

NM_014300	SEC11-like 1	SEC11L1	1.3859383	2.613418737	-0.183964179	0.88028087	0.001975123	2.968846451
NM_003003	SEC14-like 1	SEC14L1	1.452653954	2.737111022	-0.234225411	0.850141321	0.001181772	3.219595323
NM_004892	SEC22 vesicle trafficking protein-like 1	SEC22L1	1.027078205	2.037892859	-0.659483759	0.633104801	0.029320379	3.218887072
NM_014822	SEC24 related gene family, member D	SEC24D	2.350710317	5.10075327	-3.299796433	0.101545877	0.029237386	50.23102295
NM_013336	Sec61 alpha 1 subunit	SEC61A1	1.281630272	2.431135444	0.206891699	1.15419877	0.000688285	2.106340352
NM_006808	Sec61 beta subunit	SEC61B	1.780445565	3.435322554	-0.170987373	0.88823457	0.025780109	3.867584837
NM_001012456	Sec61 gamma subunit	SEC61G	2.356191899	5.120170667	0.970646221	1.959718208	0.002166746	2.612707605
NM_007277	SEC6-like 1	SEC6L1	1.317412084	2.492186585	-0.304589122	0.809672778	0.003896899	3.078017013
NM_003004	secreted and transmembrane 1	SECTM1	3.985345345	15.83829734	-0.65540894	0.634895501	0.000350975	24.94630584
NM_021237	selenoprotein K	SELK	1.022390474	2.031281906	0.547194513	1.461241378	0.000117667	1.390107025
NM_003006	selectin P ligand	SELPLG	1.594676649	3.020268163	-0.364047342	0.776981772	0.002536434	3.887180205
NM_018445	selenoprotein S	SELS 2	1.809546981	3.505322011	-0.156594926	0.897140019	0.000203252	3.907218422
NM_022367	sema domain, immunoglobulin domain Ig, transmembrane domain TM and short cytoplasmic domain, semaphorin 4A	SEMA4A	1.26250244	2.39911521	-0.837380922	0.559658655	0.017188722	4.286747266
NM_017789	sema domain, immunoglobulin domain Ig, transmembrane domain TM and short cytoplasmic domain, semaphorin 4C	SEMA4C	1.002135791	2.002963028	-0.000981098	0.999320186	0.021200004	2.004325597
NM_006378	sema domain, immunoglobulin domain Ig, transmembrane domain TM and short cytoplasmic domain, semaphorin 4D	SEMA4D	1.581150923	2.992084508	0.148278662	1.108246389	1.92E-05	2.699836911
NM_004261	15 kDa selenoprotein	SEP15 1	1.497121824	2.822790038	0.079930126	1.056966848	8.69E-05	2.670651444
NM_000295	serine or cysteine proteinase inhibitor, clade A alpha-1 antiproteinase, antitrypsin, member 1	SERPINA1	3.399744133	10.5541913	-1.114100852	0.46197899	4.20E-06	22.84560882
NM_001085	serine or cysteine proteinase inhibitor, clade A alpha-1 antiproteinase, antitrypsin, member 3	SERPINA3	4.67565719	25.55718776	-0.048408548	0.967002452	3.15E-05	26.42928952
NM_030666	serine or cysteine proteinase inhibitor, clade B ovalbumin, member 1	SERPINB1	1.18860605	2.279324057	0.249189941	1.188539575	0.033471697	1.917751924
NM_002575	serine or cysteine proteinase inhibitor, clade B ovalbumin, member 2	SERPINB2	3.240638024	9.452120518	0.159803377	1.117134874	0.014981339	8.461037905
NM_004568	serine or cysteine proteinase inhibitor, clade B ovalbumin, member 6	SERPINB6	1.526106171	2.880074581	-0.758528292	0.591099009	0.001959983	4.872406374
NM_000602	serine or cysteine proteinase inhibitor, clade E nexin, plasminogen activator inhibitor type 1, member 1	SERPINE1	1.929271215	3.808627557	-0.628784455	0.646721082	0.00117	5.889134691
NM_002615	serine or cysteine proteinase inhibitor, clade F alpha-2 antiplasmin, pigment epithelium derived factor, member 1	SERPINF1	1.765976545	3.401041346	-0.429036543	0.742757646	1.58E-06	4.578938181
NM_000934	serine or cysteine proteinase inhibitor, clade F alpha-2 antiplasmin, pigment epithelium derived factor, member 2	SERPINF2	2.310396426	4.960193581	-0.484543692	0.714723093	0.017566434	6.940021427
NM_000062	serine or cysteine proteinase inhibitor, clade G C1 inhibitor, member 1, angioedema, hereditary	SERPING1	1.244727483	2.369737869	-0.209062353	0.8650993	7.32E-06	2.739266889
NM_001235	serine or cysteine proteinase inhibitor, clade H heat shock protein 47, member 1, collagen binding protein 1	SERPINH1	1.067015677	2.095095021	0.059762909	1.042294457	0.004733039	2.010079787
NM_013376	SERTA domain containing 1	SERTAD1	1.435646771	2.705034082	-0.72718947	0.604079582	0.000734484	4.477943239

XM_050625	secreted frizzled-related protein 2	SFRP2	4.305562859	19.77441155	-0.977936504	0.507705395	0.018803738	38.94859449
NM_003769	splicing factor, arginine/serine-rich 9	SFRS9	1.209109601	2.311949044	-0.184843452	0.879744533	0.001543587	2.627977735
NM_006926	surfactant, pulmonary-associated protein A2	SFTPA2	2.135025499	4.39244889	-1.068909715	0.476679103	1.52E-06	9.214687333
NM_178858	sideroflexin 2	SFXN2	1.453461961	2.738644419	0.007862146	1.0054645	0.020007863	2.72376043
NM_005627	serum/glucocorticoid regulated kinase	SGK	1.439279183	2.711853388	0.472067531	1.387095896	0.009126124	1.955058331
NM_003901	sphingosine-1-phosphate lyase 1	SGPL1	2.53946117	5.813718305	0.045019492	1.031697118	0.000135368	5.635101818
CN641646		SH3BGRL	1.515119077	2.858224182	-0.674818411	0.62641106	0.009298643	4.562857151
NM_031286	SH3 domain binding glutamic acid-rich protein like 3	SH3BGRL3	1.058540721	2.082823689	-0.649337255	0.637573135	2.19E-06	3.266799643
NM_004844	SH3-domain binding protein 5	SH3BP5	2.045386513	4.12783846	-0.004102685	0.997160275	0.006071478	4.139593767
NM_016009	SH3-domain GRB2-like endophilin B1	SH3GLB1	1.75033695	3.364371339	-0.040116025	0.972576727	0.00828648	3.459234881
NM_024745	SHC SH2-domain binding protein 1	SHCBP1	3.308919403	9.910235926	-0.153095496	0.899318782	0.005384649	11.01971417
NM_003031	seven in absentia homolog 1	SIAH1	1.905538737	3.746487758	-0.373592568	0.771858038	0.026617039	4.853855988
NM_005067	seven in absentia homolog 2	SIAH2	2.787088308	6.902353239	0.324865809	1.252547928	0.001283152	5.510649999
NM_052884	sialic acid binding Ig-like lectin 11	SIGLEC11	1.177167733	2.261324028	0.535171924	1.449114833	0.012350347	1.560486427
NM_014385	sialic acid binding Ig-like lectin 7	SIGLEC7	1.649823719	3.137952946	0.0407049	1.028616285	0.009394499	3.050654544
NM_014412	Siah-interacting protein	SIP	1.07034689	2.099938227	-0.188317289	0.877628761	0.030454218	2.392740894
NM_003037	signaling lymphocytic activation molecule family member 1	SLAMF1	3.027352461	8.153121197	-0.300878565	0.811757906	0.048361358	10.04378416
NM_020125	SLAM family member 8	SLAMF8	3.174907416	9.031135653	-0.27908722	0.82411226	0.000442335	10.9586231
NM_006527	stem-loop histone binding protein	SLBP	1.794538523	3.469044922	-1.20582468	0.433521461	0.009027459	8.002014275
NM_016582	solute carrier family 15, member 3	SLC15A3	1.130685843	2.189628083	-0.284634297	0.820949682	3.24E-05	2.667189148
XR_013400	solute carrier family 15, member 4	SLC15A4	1.53846941	2.904861562	0.079213141	1.05644169	1.02E-05	2.749665777
CO646894		SLC16A3	2.510723946	5.699059861	-0.555708421	0.680322911	0.000605979	8.376992414
NM_012434	solute carrier family 17 anion/sugar transporter, member 5	SLC17A5	1.097604521	2.139990691	-0.63151537	0.645498044	0.003412663	3.315255112
NM_004172	solute carrier family 1 high affinity glutamate transporter, member 3	SLC1A3	3.35522574	10.23348577	0.377709922	1.299277795	0.005393226	7.87628774
CN648722		SLC2A3	2.570211061	5.938963069	-0.529039307	0.693016061	0.009500654	8.569733664
NM_006931	solute carrier family 2 facilitated glucose transporter, member 3	SLC2A3	3.014396856	8.080232781	-3.021304804	0.123167642	0.007440095	65.60353542
NM_003039	solute carrier family 2 facilitated glucose/fructose transporter, member 5	SLC2A5	2.224311064	4.672877005	-0.50592178	0.704210293	6.81E-05	6.635627238
NM_017585	solute carrier family 2 facilitated glucose transporter, member 6	SLC2A6	1.47561916	2.781029715	0.089084334	1.06369485	0.005671964	2.614499557
NM_001860	solute carrier family 31 copper transporters, member 2	SLC31A2	2.106926374	4.30772566	0.230867495	1.173540389	0.032260058	3.670709334
NM_017945	solute carrier family 35, member A5	SLC35A5	1.709527539	3.270537006	-1.326251745	0.398803024	0.013098125	8.200883163
NM_015948	solute carrier family 35, member B3	SLC35B3	1.071101062	2.101036263	-0.510386614	0.702034281	0.000132451	2.992783
NM_018656	solute carrier family 35, member E3	SLC35E3	1.888073502	3.701406286	-1.377292026	0.384940661	0.008481331	9.615524322
NM_018976	solute carrier family 38, member 2	SLC38A2	1.911086643	3.760922671	-1.416433601	0.374637287	0.018643832	10.03883704
NM_153811	solute carrier family 38, member 6	SLC38A6	1.976292673	3.934806451	-0.452348438	0.730852188	0.000637279	5.383860804
NM_014437	solute carrier family 39 zinc transporter, member 1	SLC39A1	2.836813387	7.14440263	-0.863890535	0.549468799	0.007449695	13.00238093
NM_022154	solute carrier family 39 zinc transporter, member 8	SLC39A8	1.83981903	3.579651228	-0.542500376	0.686579946	0.000948437	5.213742767
NM_014096	solute carrier family 43, member 3	SLC43A3	1.900601705	3.733688852	-0.52603974	0.694458438	0.032123657	5.376403604
NM_003486	solute carrier family 7 cationic amino acid transporter, y+ system, member 5	SLC7A5	2.427810254	5.380761098	-0.004649628	0.996782312	0.03325213	5.398130601

NM_007256	solute carrier organic anion transporter family, member 2B1	SLCO2B1	1.674581346	3.19226705	0.383425705	1.304435586	0.002022309	2.447240081
NM_016354	solute carrier organic anion transporter family, member 4A1	SLCO4A1	3.35584174	10.23785618	-0.0447423	0.969462971	0.021091332	10.56033751
NM_138440	slit-like 2	SLITL2	1.014835994	2.020673169	0.075180447	1.053492795	0.017134721	1.918070232
NM_005585	SMAD, mothers against DPP homolog 6	SMAD6	1.27754727	2.424264763	-0.255046017	0.837960406	0.003780336	2.89305407
NM_003601	SWI/SNF related, matrix associated, actin dependent regulator of chromatin, subfamily a, member 5	SMARCA5	1.250993736	2.380053059	0.377770971	1.299332776	0.011421888	1.83175019
NM_000543	sphingomyelin phosphodiesterase 1, acid lysosomal acid sphingomyelinase	SMPD1	2.082260476	4.234702077	-0.598271628	0.660544825	0.003747881	6.410923101
NM_006049	small nuclear RNA activating complex, polypeptide 5, 19kDa	SNAPC5	1.756318043	3.378348227	-0.141925472	0.906308756	0.003759377	3.72759085
NM_014390	staphylococcal nuclease domain containing 1	SND1	1.549168223	2.92648366	-0.05436974	0.963015057	0.000114307	3.038876329
NM_003091	small nuclear ribonucleoprotein polypeptides B and B1	SNRPB	1.426775113	2.68845088	-0.520683979	0.697041289	7.90E-05	3.856946388
NM_013322	sorting nexin 10	SNX10	5.131852111	35.06238213	-0.771175313	0.585939936	0.016480841	59.8395501
DR774547		SNX17	1.738721119	3.337391925	-0.446580413	0.73378005	0.012321987	4.548218399
NM_021249	sorting nexin 6	SNX6 1	1.696499592	3.241136077	-0.301127572	0.811617809	0.002071148	3.993426511
NM_015976	sorting nexin 7	SNX7 1	1.410018439	2.657405593	-0.786701912	0.579667734	0.010862651	4.584360036
NM_003745	suppressor of cytokine signaling 1	SOCS1	2.153358134	4.448620817	0.568363642	1.482840724	0.038924238	3.000066525
NM_000636	superoxide dismutase 2, mitochondrial	SOD2	2.521352018	5.74119882	-0.031069032	0.978694819	0.049193955	5.86617882
NM_182513	spindle pole body component 24 homolog	SPBC24	2.188834907	4.559371315	0.414965881	1.333267145	0.04783596	3.419698244
NM_014041	signal peptidase 12kDa	SPC12	1.379902644	2.602508082	-0.409898823	0.752676157	0.001238598	3.457673072
NM_021972	sphingosine kinase 1	SPHK1	1.896164041	3.72222184	0.306541154	1.236739074	0.016509096	3.00970667
CN647492		SPI1	1.360913988	2.568478483	-1.331450654	0.39736848	0.00150708	6.463719722
NM_000582	secreted phosphoprotein 1	SPP1	4.376749287	20.77460709	-0.742723539	0.59761011	0.029934375	34.76281062
NM_021199	sulfide quinone reductase-like	SQRDL	2.517067454	5.724173709	-0.517917902	0.698379007	3.61E-05	8.196371387
CN645176		SRA1	1.141140845	2.205553636	-1.477044365	0.359223997	0.003093103	6.139772545
XR_000132	steroid receptor RNA activator 1	SRA1	1.081602117	2.116385029	-0.918722757	0.528977126	0.003829699	4.000900845
NM_003136	signal recognition particle 54kDa	SRP54	1.818826563	3.527941316	-0.961504773	0.513521017	0.005507589	6.870101131
NM_021203	signal recognition particle receptor, B subunit	SRPRB	1.652958951	3.144779688	-0.120411072	0.919925496	0.007942047	3.418515633
CN642598		SRPS28	1.348924832	2.54722223	-0.830009752	0.56252544	0.002699743	4.52819028
NM_006307	sushi-repeat-containing protein, X-linked	SRPX	2.383752908	5.218925862	0.115084632	1.083038577	0.010284224	4.818781132
NM_016305	synovial sarcoma translocation gene on chromosome 18-like 2	SS18L2	1.239518958	2.361197891	0.396982333	1.316750798	0.008283737	1.793200274
NM_003144	signal sequence receptor, alpha	SSR1	1.626449665	3.087522532	-0.063731907	0.956785939	0.000281028	3.226973146
NM_007107	signal sequence receptor, gamma	SSR3	1.347293454	2.544343498	-0.181263006	0.881930573	0.008558975	2.884970287
NM_006396	Sjogren's syndrome/scleroderma autoantigen 1	SSSCA1	1.301110454	2.4641848	-0.409548396	0.752859003	0.012199695	3.273102654
NM_003032	ST6 beta-galactosamide alpha-2,6-sialyltranferase 1	ST6GAL1	1.748785122	3.360754413	-0.177025822	0.884524604	0.000446964	3.799503596
NM_139266	signal transducer and activator of transcription 1, 91kDa	STAT1	2.295329908	4.908662269	0.338179738	1.264160584	0.000959322	3.882942033
NM_003152	signal transducer and activator of transcription 5A	STAT5A	2.792451347	6.92805963	-0.295751768	0.81464772	0.022819016	8.504362632
NM_152999	six transmembrane epithelial antigen of the prostate 2	STEAP2	1.155843153	2.228145049	-0.382882785	0.766903636	0.00961729	2.905378126
NM_003576	serine/threonine kinase 24	STK24	1.321174916	2.498695181	-0.210579201	0.864190214	6.10E-05	2.891371761
NM_004099	stomatin	STOM 1	1.574559093	2.978444534	0.226367648	1.169885753	8.53E-05	2.545927692
NM_198194	stomatin	STOM 2	1.366636675	2.578687008	0.360668908	1.284021098	0.002574444	2.008290216
NM_007178	serine/threonine kinase receptor associated protein	STRAP	1.651061692	3.14064677	-0.392424281	0.761848331	0.000986006	4.122404214

NM_003764	syntaxin 11	STX11	2.117620961	4.339777149	-0.997106727	0.501003738	0.000134413	8.662165206
NM_177424	syntaxin 12	STX12	1.884492766	3.692230872	-0.599272833	0.660086578	0.001105572	5.593555448
NM_004853	syntaxin 8	STX8	1.429744299	2.693989632	0.097705664	1.070070362	0.000668418	2.517581766
NM_033050	succinate receptor 1	SUCNR1	2.882235224	7.372915506	0.331334331	1.258176509	0.016067307	5.860000925
XR_012338	sulfatase modifying factor 1	SUMF1	1.92063487	3.785896236	-0.694303659	0.618007538	0.018601033	6.125970965
XM_497144	SMT3 suppressor of mif two 3 homolog 2	SUMO2	1.033639287	2.047181886	-0.519668338	0.697532171	0.000922815	2.934892427
NM_006936	SMT3 suppressor of mif two 3 homolog 3	SUMO3	1.10406219	2.149591005	-0.176038824	0.885129945	0.007813632	2.428559804
NM_033161	surfeit 4	SURF4	1.831607706	3.559334956	-0.395114722	0.760428905	0.014988272	4.680693923
NM_006753	surfeit 6	SURF6	1.873536273	3.664296571	0.13724461	1.099802606	0.007865481	3.331776587
NM_003177	spleen tyrosine kinase	SYK	1.149759491	2.218769026	-0.104410538	0.930184924	0.003811196	2.385298847
NM_006372	synaptotagmin binding, cytoplasmic RNA interacting protein	SYNCRIP	1.741986904	3.344955235	-0.545404021	0.685199488	0.011920418	4.881724654
CO648815		SYT11	1.759130297	3.384940083	0.317348305	1.246038206	0.017462663	2.716562033
NM_205848	synaptotagmin VI	SYT6	1.019153794	2.026729842	0.058806619	1.041603802	0.007520216	1.945778078
NM_006284	TAF10 RNA polymerase II, TATA box binding protein TBP-associated factor, 30kDa	TAF10	1.303749194	2.468696005	0.352783373	1.277021994	0.025002737	1.933166396
NM_005642	TAF7 RNA polymerase II, TATA box binding protein TBP-associated factor, 55kDa	TAF7	1.175430993	2.258603448	-0.028021287	0.980764533	0.010675281	2.302900821
NM_003187	TAF9 RNA polymerase II, TATA box binding protein TBP-associated factor, 32kDa	TAF9 1	1.371078793	2.58663913	0.694832754	1.618696773	0.006497812	1.597976331
NM_054114	T-cell activation GTPase activating protein	TAGAP	2.500147801	5.657433813	-0.164389256	0.892306184	0.006341443	6.340238264
NM_006755	transaldolase 1	TALDO1	1.486982276	2.803020461	-0.433805513	0.740306442	0.00712015	3.786297543
NM_004180	TRAF family member-associated NFKB activator	TANK 1	1.225559768	2.338461648	-0.57453722	0.671501618	7.91E-05	3.482436358
NM_016281	TAO kinase 3	TAOK3	2.037084118	4.104151871	-1.227604653	0.427025861	0.030504522	9.611014807
NM_000593	transporter 1, ATP-binding cassette, sub-family B	TAP1	2.426719224	5.376693466	-0.003206804	0.997779682	4.08E-05	5.388658003
CO646475		TAPBP	1.488404285	2.805784653	-0.121466387	0.919252827	1.58E-05	3.052244791
NM_152295	threonyl-tRNA synthetase	TARS	2.54792954	5.847944179	-0.466745431	0.723595113	0.00191467	8.0817906
NM_014604	Tax1 human T-cell leukemia virus type I binding protein 3	TAX1BP3	1.232899517	2.350388948	-0.594135228	0.662441413	0.000104437	3.548070669
NM_012453	transducin	TBL2 1	1.410357568	2.658030333	-0.154380962	0.89851783	0.029265541	2.958238828
NM_024863	transcription elongation factor A	TCEAL4 1	1.344460411	2.539352033	-0.225444349	0.855331544	0.001236608	2.968851145
NM_003198	transcription elongation factor B SIII, polypeptide 3 110kDa, elongin A	TCEB3	2.336404832	5.050425121	-0.018800497	0.987053031	0.03620602	5.116670497
NM_006019	T-cell, immune regulator 1, ATPase, H+ transporting, lysosomal V0 protein a isoform 3	TCIRG1	1.31682552	2.49117353	-0.669949949	0.628528492	0.000279715	3.963501354
NM_030752	t-complex 1	TCP1 1	1.670757995	3.18381828	-0.223090178	0.856728402	0.012407764	3.716251583
CN642847		TEBP	1.517884614	2.863708435	-0.245726219	0.843391146	0.001472414	3.395468934
NM_003217	testis enhanced gene transcript BAX inhibitor 1	TEGT	1.289855807	2.445036169	-0.395278008	0.760342844	0.000532031	3.215702216
NM_001063	transferrin	TF	1.872291761	3.661137002	0.217493617	1.16271186	7.72E-05	3.14879131
NM_006286	transcription factor Dp-2	TFDP2	2.275580002	4.841922547	-0.429596992	0.742469161	0.022237664	6.52137867
NM_012252	transcription factor EC	TFEC 1	4.668781232	25.43567071	-0.296914085	0.813991658	0.008298083	31.24807294
NM_006070	TRK-fused gene	TFG	1.390624669	2.621921822	-0.50871664	0.702847383	0.000213834	3.730428376
CK231327		TFPI2	2.372182392	5.177237105	-0.451344146	0.731361128	2.21E-05	7.078906586
CK231384		TFPI2	1.631182678	3.097668322	-0.012820274	0.99115303	0.000943415	3.125317916
CK232222		TFPI2	5.718584738	52.65814291	-1.578086212	0.334925886	0.011483257	157.2232698
CK230426		TFPI2	1.285999777	2.438509811	-0.098335143	0.934110325	0.000121279	2.610515853

CN644277		TFRC	2.184133041	4.544536124	-0.186602214	0.878672706	0.002140344	5.172046534
NM_000660	transforming growth factor, beta 1	TGFB1	1.277965082	2.424966945	-0.768950144	0.586844369	0.000238762	4.13221473
NM_000358	transforming growth factor, beta-induced, 68kDa	TGFBI	1.837947592	3.57501078	-0.074768218	0.949494653	0.000106632	3.765172102
NM_004612	transforming growth factor, beta receptor I activin A receptor type II-like kinase, 53kDa	TGFBR1	1.195144875	2.289678239	0.065951396	1.046775016	0.015857325	2.187364242
XR_009902	Trans-Golgi network integral membrane protein 2 precursor Trans-Golgi network protein	TGN51	1.764331087	3.397164519	0.14289597	1.104119228	0.003007848	3.07680949
NM_020457	THAP domain containing 11	THAP11	1.956558304	3.881349382	-0.42510456	0.744784751	0.005274276	5.211370637
NM_000361	thrombomodulin	THBD	3.61712446	12.2705198	-0.199823707	0.870656948	0.013918205	14.09340363
NM_003246	thrombospondin 1	THBS1	2.677358676	6.396836795	0.29102328	1.223507784	0.001252642	5.228276336
NM_032361	THO complex 3	THOC3	1.100318215	2.144019779	-0.30345252	0.810310916	0.003547271	2.645922371
NM_182919	toll-like receptor adaptor molecule 1	TICAM1	1.996747685	3.990992825	-0.609390798	0.655473428	0.02441683	6.088717947
NM_012456	translocase of inner mitochondrial membrane 10 homolog	TIMM10	2.176066714	4.519197833	-0.140502331	0.907203222	0.014198812	4.981461402
NM_006327	translocase of inner mitochondrial membrane 23 homolog	TIMM23	2.185039965	4.547393861	-0.744649858	0.5968127	0.003567433	7.619465634
NM_004085	translocase of inner mitochondrial membrane 8 homolog A	TIMM8A	1.809454208	3.505096607	-0.731115202	0.60243805	0.011645241	5.818185968
NM_003254	tissue inhibitor of metalloproteinase 1 erythroid potentiating activity, collagenase inhibitor	TIMP1	3.06162159	8.349105215	-0.115442549	0.923099105	0.002977169	9.044646635
NM_003255	tissue inhibitor of metalloproteinase 2	TIMP2	1.154521587	2.226104913	-1.112013811	0.462647785	0.000497123	4.811662318
NM_003258	thymidine kinase 1, soluble	TK1	3.639046014	12.4583924	-0.172727702	0.887163735	0.000682656	14.04294597
NM_015913	endoplasmic reticulum thioredoxin superfamily member, 18 kDa	TLP19	1.056860439	2.080399273	-0.02552563	0.982462584	0.000373445	2.117535372
NM_138557	toll-like receptor 4	TLR4 4	4.207483481	18.47475697	-1.024087262	0.491721294	0.026277299	37.57160243
NM_016610	toll-like receptor 8	TLR8 1	1.11660211	2.16835671	-0.587701264	0.66540229	0.001509583	3.258715432
NM_014220	transmembrane 4 L six family member 1	TM4SF1	2.969236861	7.831218824	-0.201030687	0.869928849	0.000478041	9.00213717
NM_003272	transmembrane 7 superfamily member 1	TM7SF1	2.054702447	4.154579452	0.00861972	1.005992619	0.001899023	4.129830949
NM_006405	transmembrane 9 superfamily member 1	TM9SF1	1.498839613	2.826153082	0.329369732	1.256464345	0.003238378	2.249290314
XR_011199	transmembrane 9 superfamily member 2	TM9SF2	2.675583944	6.388972563	0.182176342	1.134594159	0.006187239	5.63106421
NM_019026	transmembrane and coiled-coil domains 1	TMCO1	1.687667265	3.221354124	-0.341698662	0.789111647	0.005278425	4.082253934
NM_007364	transmembrane emp24 domain containing 3	TMED3	1.295532419	2.454675672	-0.005785354	0.995997928	0.001079051	2.464538935
NM_014051	transmembrane protein 14A	TMEM14A	1.532299884	2.892465765	0.426766606	1.344217514	0.038529864	2.15178402
NM_001007538	transmembrane protein 46	TMEM46	5.302568014	39.46681011	-0.243303571	0.844808601	0.003811553	46.71686586
NM_014313	transmembrane protein 50A	TMEM50A	1.406351569	2.650659892	-1.027580176	0.490532226	0.002798238	5.40364068
NM_018022	transmembrane protein 51	TMEM51	1.471480769	2.773063723	-0.610990965	0.654746812	0.007100742	4.235322222
NM_014547	tropomodulin 3	TMOD3	1.199524401	2.296639474	-0.041707519	0.971504431	0.021782854	2.364003087
XR_000287	Tmp21-II , transcribed pseudogene	Tmp21	2.684568906	6.428886612	-0.435493995	0.739440519	0.015374957	8.694257957
NM_000594	tumor necrosis factor TNF superfamily, member 2	TNF	1.164547046	2.241628255	0.342371743	1.267839167	0.02474095	1.768069888
NM_021137	tumor necrosis factor, alpha-induced protein 1	TNFAIP1	1.486521683	2.802125716	-0.63819733	0.64251528	0.013558119	4.36118144
NM_006290	tumor necrosis factor, alpha-induced protein 3	TNFAIP3	3.950890063	15.46451907	-0.434015993	0.740198443	0.002116201	20.8923961
NM_014350	tumor necrosis factor, alpha-induced protein 8	TNFAIP8	2.000350168	4.000970991	-1.236354654	0.42444377	0.032924425	9.426386412
NM_001065	tumor necrosis factor receptor superfamily, member 1A	TNFRSF1A	1.807695093	3.500825358	-0.195302667	0.873389646	0.002798636	4.008320198
NM_003327	tumor necrosis factor receptor superfamily, member 4	TNFRSF4	1.451205883	2.73436509	-0.039008242	0.973323814	0.043204735	2.809306678
NM_001243	tumor necrosis factor receptor superfamily, member 8	TNFRSF8	1.310780803	2.48075765	0.007721245	1.005366307	0.013130716	2.467516202

NM_003808	tumor necrosis factor ligand superfamily, member 13	TNFSF13A	1.513697273	2.855408734	0.009253323	1.006434528	5.26E-05	2.837152994
NM_006573	tumor necrosis factor ligand superfamily, member 13b	TNFSF13B	2.005975369	4.016601596	0.071178269	1.050574352	0.003441347	3.823243533
NM_006058	TNFAIP3 interacting protein 1	TNIP1	1.797478645	3.476121827	-1.915876997	0.265010789	0.041968449	13.1169068
NM_003282	troponin I, skeletal, fast	TNNI2	1.512686281	2.853408461	-0.046206465	0.96847958	0.000489002	2.946276329
NM_003286	topoisomerase DNA I	TOP1	3.504806758	11.35146627	-1.258814468	0.417887217	0.036351219	27.16394716
XR_011982	DNA topoisomerase II, alpha isozyme	TOP2A	2.385670235	5.225866374	-0.169547735	0.889121365	0.004444917	5.877562479
NM_004618	topoisomerase DNA III alpha	TOP3A	1.33657716	2.525514211	-0.180978961	0.882104229	0.000469573	2.86305646
NM_014506	torsin family 1, member B	TOR1B	2.463117001	5.51406778	-0.630860525	0.645791105	0.002611475	8.538469695
CK230208		TPBG	2.470939887	5.544048533	0.507877399	1.421956566	0.000127087	3.898887395
XR_011552	Triosephosphate isomerase TIM Triose-phosphate isomerase	TPI1	1.523642693	2.875160896	-0.345648611	0.786954096	2.70E-05	3.653530633
NM_153649	tropomyosin 3	TPM3	1.625770031	3.086068385	-0.921778815	0.527857781	9.41E-05	5.846401244
NM_012112	TPX2, microtubule-associated, homolog	TPX2	1.515357047	2.85869568	-0.229356646	0.8530152	0.004502275	3.351283402
NM_145725	TNF receptor-associated factor 3	TRAF3 1	2.45817106	5.495196459	-0.104960914	0.929830134	0.003237233	5.90989285
NM_006700	TRAF-type zinc finger domain containing 1	TRAFD1	2.036706519	4.103077825	-0.186027277	0.879022941	0.00223123	4.667771037
NM_018643	triggering receptor expressed on myeloid cells 1	TREM1	2.595956334	6.04589672	-0.289178255	0.81836806	0.008942573	7.387747657
NM_018965	triggering receptor expressed on myeloid cells 2	TREM2	2.849492625	7.207468494	-0.238414069	0.847676638	0.00157505	8.502615472
NM_016381	three prime repair exonuclease 1	TREX1	1.739758585	3.339792764	-0.337896011	0.791194328	1.00E-05	4.221204127
NM_007117	thyrotropin-releasing hormone	TRH	1.666237548	3.173857923	0.679775154	1.601890079	0.006192899	1.981320669
NM_014788	tripartite motif-containing 14	TRIM14 1	2.060438436	4.171130461	-0.023838539	0.98361215	0.049718682	4.240625191
NM_033452	tripartite motif-containing 47	TRIM47	1.581784382	2.993398562	-0.37001267	0.773775701	0.001244694	3.868561079
NM_004237	thyroid hormone receptor interactor 13	TRIP13	1.825111655	3.543344297	-0.207781917	0.865867443	0.005263368	4.092247982
NM_001001188	transient receptor potential cation channel, subfamily M, member 2	TRPM2 S	1.486045073	2.801200155	-0.163780201	0.892682964	0.002221067	3.137956328
NM_021625	transient receptor potential cation channel, subfamily V, member 4	TRPV4 1	1.241073876	2.363744131	-0.437527815	0.738398837	0.005763531	3.201175318
NM_033051	thymic stromal co-transporter	TSCOT	1.075374309	2.107268735	-0.512227058	0.701139268	0.001584644	3.00549239
NM_005726	Ts translation elongation factor, mitochondrial	TSFM	2.292488777	4.899005044	-0.456657741	0.728672404	0.00295908	6.723192782
NM_014399	tetraspanin 13	TSPAN13	1.587634453	3.005561318	-0.291744317	0.816913757	0.000799816	3.679165998
NM_001006616	tetraspanin 17	TSPAN17	1.286271597	2.438969297	0.116869708	1.084379472	0.000161979	2.249184311
CN647236		TTYH2	1.036605261	2.051394927	0.205512228	1.15309568	0.004350136	1.779032705
NM_006000	tubulin, alpha 1	TUBA1	1.10147337	2.145737167	-0.148485897	0.902196818	0.000334809	2.378347079
NM_025019	tubulin, alpha 4	TUBA4	2.355346942	5.117172766	0.282626466	1.216407373	0.001331329	4.206791968
NM_032704	tubulin alpha 6	TUBA6	1.209475363	2.312535259	-0.08111941	0.94532387	3.28E-05	2.446288867
NM_001069	tubulin, beta polypeptide	TUBB	1.381880291	2.606078049	-0.166657145	0.890904598	0.00069519	2.925204399
NM_001070	tubulin, gamma 1	TUBG1	1.071178902	2.101149626	-0.14931516	0.901678383	0.000579612	2.330265054
NM_022085	thioredoxin domain containing 5	TXNDC5	1.223838113	2.335672685	0.366297816	1.2890407	0.034991455	1.811946423
CN642567		TXNDC7	2.091365439	4.26151213	-1.216973124	0.430184329	0.002067706	9.906246792
NM_006472	thioredoxin interacting protein	TXNIP	1.70128646	3.251908039	-0.995822787	0.50144981	0.000205632	6.48501201
NM_003330	thioredoxin reductase 1	TXNRD1	1.767132133	3.403766647	-0.590847094	0.663952945	0.010771378	5.126517881
CN648808		TYROBP	1.865601553	3.64419852	0.203143658	1.151204118	0.000700009	3.165553755
NM_006398	ubiquitin D	UBD	3.812960444	14.05450214	0.264591217	1.201295618	0.00130935	11.69945343
NM_181803	ubiquitin-conjugating enzyme E2C	UBE2C 6	1.861536379	3.633944482	0.065243418	1.046261454	0.022065137	3.473266139
NM_152653	ubiquitin-conjugating enzyme E2E 2 UBC4/5 homolog, yeast	UBE2E2	1.156766198	2.229571086	-0.104973256	0.929822179	0.011311528	2.397846746

NM_016021	ubiquitin-conjugating enzyme E2, J1	UBE2J1	1.124424253	2.180145244	-1.006587201	0.497722254	0.002796457	4.380244657
NM_003347	ubiquitin-conjugating enzyme E2L 3	UBE2L3 1	1.245380207	2.370810262	0.192574216	1.142801009	0.003516969	2.074560875
XR_012726	ubiquitin-conjugating enzyme E2L 6 isoform 1	UBE2L6	2.863019885	7.27536629	-0.00756925	0.994767135	0.010838049	7.313637566
NM_130839	ubiquitin protein ligase E3A	UBE3A 3	1.103391091	2.148591312	-0.032579369	0.977670774	0.027467461	2.197663436
NM_006002	ubiquitin carboxyl-terminal esterase L3	UCHL3	1.834851393	3.567346598	-1.136339511	0.454912344	0.008942922	7.841832919
NM_003359	UDP-glucose dehydrogenase	UGDH	1.289458431	2.444362802	0.0952306	1.068236141	0.014621206	2.288223276
NM_013282	ubiquitin-like, containing PHD and RING finger domains, 1	UHRF1	3.194723701	9.156039588	-0.17096782	0.888246609	0.048706316	10.30799273
NM_016308	UMP-CMP kinase	UMP-CMPK	1.29615638	2.455737542	-1.135600777	0.455145342	0.001568932	5.39550186
CN804946		Hs.5250	1.163472229	2.239958851	-0.577050185	0.670332977	0.007458519	3.341561472
NM_198477	DMC	UNQ473	1.244445763	2.369275168	-0.507579904	0.703401393	0.003471918	3.368311739
NM_006830	ubiquinol-cytochrome c reductase, 6.4kDa subunit	UQCR	1.336702523	2.525733675	-0.055132994	0.962505711	0.008695174	2.624123313
XR_010024	Ubiquinol-cytochrome c reductase iron-sulfur subunit, mitochondrial precursor	UQCRFS1	1.59984508	3.031107629	-0.89536163	0.537612419	0.003440352	5.638090792
NM_178443	UNC-112 related protein 2	URP2	1.149317297	2.218089066	-0.043051619	0.970599742	0.000440957	2.285276795
NM_032747	upregulated during skeletal muscle growth 5	USMG5	1.264979507	2.403237962	0.435580766	1.352455163	0.02806185	1.776944647
NM_017414	ubiquitin specific protease 18	USP18	1.233628209	2.351576408	0.550227729	1.464316819	0.035339148	1.605920506
NM_004652	ubiquitin specific protease 9, X-linked fat facets-like, Drosophila	USP9X 1	1.076504421	2.108920076	-0.166294289	0.8911287	0.022307071	2.366571827
CN802199		VAMP3	1.998656552	3.996276905	-0.739259053	0.599046935	0.013737402	6.671058095
NM_006373	vesicle amine transport protein 1 homolog	VAT1	2.356077423	5.119764402	-0.299185367	0.812711173	0.008811374	6.299611194
NM_005428	vav 1 oncogene	VAV1	1.579226837	2.988096701	0.006097596	1.004235476	0.00042179	2.975494068
NM_001078	vascular cell adhesion molecule 1	VCAM1 1	2.306043764	4.945251054	0.521108123	1.435057081	0.043164952	3.446030905
NM_007126	valosin-containing protein	VCP	1.996145241	3.989326604	-0.493917518	0.710094276	0.000431035	5.618023887
NM_000376	vitamin D 1,25- dihydroxyvitamin D3 receptor	VDR 1	2.472388109	5.549616615	-0.361534296	0.778336384	0.00297144	7.130100466
NM_016226	vacuolar protein sorting 29	VPS29 1	1.154235322	2.225663246	-0.776734418	0.58368649	0.037036314	3.813114203
NM_007268	V-set and immunoglobulin domain containing 4	VSIG4	3.958791612	15.54944963	-0.386966663	0.76473581	5.56E-05	20.33309991
NM_000552	von Willebrand factor	VWF	3.931298265	15.25593043	0.143703991	1.104737794	1.08E-05	13.80954876
NM_004184	tryptophanyl-tRNA synthetase	WARS 1	3.78531246	13.78772439	-1.407105282	0.377067501	0.000206152	36.56566622
NM_032463	Williams-Beuren syndrome chromosome region 5	WBSCR5	1.945587525	3.851946125	0.234664238	1.176632864	0.000367018	3.273702649
NM_080733	WAP four-disulfide core domain 2	WFDC2	1.112705001	2.162507297	-0.251835936	0.839826993	0.00033076	2.574943786
NM_080614	WAP four-disulfide core domain 3	WFDC3	1.542310354	2.912605593	0.161787916	1.118672638	0.007674228	2.603626383
NM_004906	Wilms tumor 1 associated protein	WTAP	2.831680849	7.119030828	-0.598163087	0.660594523	0.017824745	10.77670278
NM_152858	Wilms tumor 1 associated protein	WTAP	1.789039737	3.455847936	0.37943438	1.300831754	0.015856066	2.656644816
NM_019001	5'-3' exoribonuclease 1	XRN1	1.123604763	2.178907215	-0.076147661	0.948587222	0.000620327	2.297002495
NM_024096	XTP3-transactivated protein A	XTP3TPA	1.821395391	3.534228679	-0.797567708	0.57531831	0.003379224	6.143083951
CN644462		YES1	2.319556867	4.991788699	-0.516575981	0.699028906	0.008281858	7.141033308
NM_012479	tyrosine 3-monooxygenase/tryptophan 5-monooxygenase activation protein, gamma polypeptide	YWHAG	1.172951234	2.254724609	0.273412643	1.208663499	0.000731813	1.865469266
NM_003405	tyrosine 3-monooxygenase/tryptophan 5-monooxygenase activation protein, eta polypeptide	YWHAH	1.226931018	2.340685361	-0.309536586	0.806900905	1.48E-05	2.900833728
NM_003403	YY1 transcription factor	YY1	1.407016739	2.651882289	0.244223319	1.184454943	0.001080415	2.238905164
NM_004729	zinc finger, BED-type containing 1	ZBED1	2.31287753	4.968731315	0.387747128	1.308348727	0.024642253	3.797711735
NM_025079	zinc finger CCCH-type containing 12A	ZC3H12A	1.646778633	3.131336677	0.464178611	1.379531702	0.000607648	2.269854815
NM_206831	zinc finger, CSL domain containing 2	ZCSL2	1.770234766	3.4110946	0.05968027	1.042234755	0.000845073	3.272865911
NM_032283	zinc finger, DHHC domain containing 18	ZDHHC18	1.310278053	2.479893308	-0.07014924	0.952539457	6.84E-06	2.603454679

XR_012666	zinc finger, DHHC domain containing 5	ZDHHC5	2.189133967	4.560316536	-0.691147811	0.619360889	0.033138394	7.362939144
XR_011110	zinc finger, DHHC domain containing 9	ZDHHC9	1.687784197	3.221615229	0.010336099	1.007190164	6.08E-06	3.198616651
NM_006963	zinc finger protein 22	ZNF22	1.763403305	3.39498054	-0.070282377	0.952451557	0.01680516	3.564465315
NM_007167	zinc finger protein 258	ZNF258	1.817217506	3.524008748	0.474009668	1.388964444	0.004968789	2.537148279
NM_018683	zinc finger protein 313	ZNF313	1.24445774	2.369294837	-0.370295272	0.773624145	0.000459882	3.062591637
NM_032997	ZW10 interactor	ZWINT	1.420850301	2.677432679	-0.253046645	0.839122506	0.004023346	3.19075303
XM_928629	peptidase prosome, macropain 26S subunit, ATPase 1 1	PSMC1	1.149905666	2.218993845	-0.546416688	0.684718697	0.001801351	3.240737921
XR_010082	neutrophil cytosolic factor 4	neutrophil cytosolic factor 4, 40kDa	1.796577711	3.473951736	-0.277496551	0.825021402	0.000129142	4.210741357
XR_012243	Actin, cytoplasmic 2	ACTG1	1.025822595	2.036120007	-0.964188225	0.512566741	0.000125935	3.972399772
XR_014443	hydroxysteroid 17-beta dehydrogenase 7	HSD17B10	1.338218773	2.528389578	-2.225805905	0.213779304	0.006350091	11.82710176
XR_014707	Complement C1r subcomponent precursor	C1R	2.71909386	6.584591135	-0.126637309	0.915963929	0.00015729	7.188701353
XR_011312	Coatomer subunit beta Beta-COP	COPB2	1.388855351	2.618708273	0.749421062	1.681118081	0.025402051	1.557718225
XR_014613	Low molecular weight phosphotyrosine protein phosphatase LMW-PTP Low molecular weight cytosolic acid phosphatase	ACP1	3.312093971	9.932066841	-0.125542377	0.916659363	0.009271643	10.83506834
XR_013405	Stress-70 protein, mitochondrial precursor 75 kDa glucose-regulated protein GRP 75	PBP74	1.551242201	2.930693715	-0.426853232	0.743882554	0.007341105	3.939726371
XR_013675	Glutathione peroxidase 3 precursor GSHPx-3 GPx-3 Plasma glutathione peroxidase GSHPx-P Extracellular glutathione peroxidase GPx-P	GPX3	1.06345726	2.089933823	-0.417064617	0.748946922	0.00075535	2.790496578
XR_010624	actin related protein 2	ARP2	1.909258001	3.756158653	-0.104423418	0.930176619	0.010746794	4.038113382
CB231011	AGENCOURT_11468692 NICHD_Rh_Ov1 cDNA clone IMAGE:6884016 5' sequence		1.182126225	2.269109491	0.004858153	1.003373091	0.000682308	2.261481308
CB230657	AGENCOURT_11469158 NICHD_Rh_Ov1 cDNA clone IMAGE:6883614 5' sequence		1.068586613	2.09737759	0.061422855	1.043494397	0.000791542	2.009955775
CB230042	AGENCOURT_11498869 NICHD_Rh_Ov1 cDNA clone IMAGE:6882817 5' sequence		2.855291961	7.236499338	-0.275295	0.826281344	0.001007347	8.757912045
CB229722	AGENCOURT_11500965 NICHD_Rh_Ov1 cDNA clone IMAGE:6882443 5' sequence		2.07849476	4.223663093	-0.238358628	0.847709213	0.012277253	4.982443302
CB311553	AGENCOURT_11823216 NICHD_Rh_Ov1 cDNA clone IMAGE:6912989 5' sequence		3.11658238	8.673308228	0.514519641	1.428518421	0.004790897	6.071541044
CB312177	AGENCOURT_11828698 NICHD_Rh_Ov1 cDNA clone IMAGE:6913202 5' sequence		2.346577148	5.08616106	-0.822011131	0.565652867	0.011347521	8.991664949
CB310604	AGENCOURT_11830389 NICHD_Rh_Ov1 cDNA clone IMAGE:6916324 5' sequence		1.095549313	2.136944312	-0.430922968	0.741787073	0.006857197	2.880805544
CB309570	AGENCOURT_11830463 NICHD_Rh_Ov1 cDNA clone IMAGE:6916521 5' sequence		1.390778545	2.622201488	-0.00196459	0.998639177	0.000188972	2.625774703
CB310061	AGENCOURT_11876913 NICHD_Rh_Ov1 cDNA clone IMAGE:6913844 5' sequence		1.789629176	3.457260171	-0.246343234	0.84303052	0.007462685	4.100990523
CD768052	AGENCOURT_14718943 NICHD_Rh_Test2 cDNA clone IMAGE:6973210 5' sequence		1.898890207	3.729262127	-0.430639607	0.741932782	0.000764323	5.026415082
BM918631	AGENCOURT_6635016 NIH_MGC_120 cDNA clone IMAGE:5747645 5' sequence		1.167704794	2.246540069	0.200280901	1.148922035	0.000937825	1.955345968

XR_010831	alpha/beta hydrolase domain containing protein 3; lung alpha/beta hydrolase 3	ABHD3	1.264479505	2.402405204	-1.591069786	0.331925233	0.002579098	7.23779021
AK057584	HD domain containing 3	HDDC3	1.522497018	2.872878577	-0.46292564	0.725513498	0.021038796	3.959786533
AK125512	cDNA FLJ43524 fis, clone PLACE5000297		1.588274447	3.00689491	-1.035532185	0.487835893	0.008777388	6.163742671
AK127395	cDNA FLJ45486 fis, clone BRTHA2002726		2.959506303	7.77857726	-0.16735544	0.890473486	6.89E-05	8.7353272
XR_012665	cytoplasmic beta-actin	ACTB	1.138538028	2.201578106	-0.518007563	0.698335605	4.62E-07	3.152607556
DB566244	DB566244 RIKEN full-length enriched human cDNA library, hypothalamus cDNA clone H033057I09 3' sequence		1.054571606	2.07710134	0.497173984	1.411446049	0.036996498	1.471612282
CK232102			1.961028813	3.893395253	0.092602747	1.066292132	0.042348499	3.651340133
CK230400			1.887856486	3.700849548	-0.916127435	0.52992958	0.000256349	6.983662895
CO645122	Hs.203697 sequence		3.990680742	15.89697922	-0.053767091	0.963417416	0.029073797	16.50061433
CK230616	Hs.417764 sequence		1.836380049	3.571128503	-0.812452888	0.569412911	0.034479513	6.271597344
CN644186	Hs.100543 sequence		1.468280431	2.76691904	-0.35357067	0.782644654	0.002466878	3.535345226
DQ155428	LILRAb mRNA, complete cds		2.096999595	4.278187151	-0.02024248	0.986066958	0.002144723	4.338637571
CB550573	MMPL0007_B09 MMPL cDNA sequence		1.314615509	2.487360315	-0.094639508	0.936506227	0.002754512	2.655999762
CB550386	MMPL0007_D06 MMPL cDNA sequence		1.355775146	2.559345905	0.174227641	1.128360166	0.000221213	2.268199448
CB550069	MMPL0008_B07 MMPL cDNA sequence		1.290650531	2.446383414	0.210537619	1.157119303	0.000471451	2.114201541
CB549988	MMPL0010_B10 MMPL cDNA sequence		2.208150829	4.620826206	-1.02788246	0.490429457	0.00321139	9.421999723
CB549220	MMPL0027_F12 MMPL cDNA sequence		2.061162779	4.173225213	-0.09794423	0.934363466	4.10E-05	4.466383122
CB555416	MMSP0026_H05 MMSP cDNA sequence		1.542387827	2.912762004	-0.083402016	0.943829378	0.008355687	3.086110764
CB553994	MMSP0053_A12 MMSP cDNA sequence		1.263330635	2.400492843	-0.056978302	0.961275386	0.007934363	2.497195786
CB551618	MMSP0057_A06 MMSP cDNA sequence		2.236529175	4.712619412	-0.768749228	0.586926101	0.0015727	8.029323293
AB021124	N-acetylglucosamine 6-O-sulfotransferase	CHST6	2.044504145	4.125314602	-3.899863792	0.066992166	0.047115942	61.57905988
AB209098	synaptotagmin binding, cytoplasmic RNA interacting protein	SYNCRIP	1.546973105	2.922034284	-1.116586613	0.461183687	0.039970469	6.335944587
BM423313	PLATE4_C07 Rhesus cDNA sequence		2.08889863	4.254231761	0.075462815	1.053699007	0.000598656	4.037425995
BC018929	pleckstrin homology-like domain, family A, member 1	PHLDB1	1.537651402	2.903214972	-0.0832372	0.94393721	0.006549066	3.075644166
XR_013663	Lymphocyte G0 /G1 switch protein 2 Small inducible cytokine A3 like 1/ C-C motif chemokine 3-like 1	SICA3	1.842958027	3.587448266	0.171180241	1.12597925	0.000205769	3.186069606
BU680450			2.005923622	4.01645753	-0.150532978	0.900917573	0.011946329	4.458185354
CN479040			1.664325032	3.16965327	0.116455342	1.084068065	3.60E-05	2.923850791
BM991538			1.064199601	2.091009477	0.52812013	1.442048943	0.014898702	1.450026705
A_01_P013097	Unknown		1.516383664	2.860730642	-0.390085241	0.763084517	0.004529196	3.74890406
A_01_P004730	Unknown		2.457902476	5.494173522	0.093439246	1.066910565	0.004935722	5.149610194
A_01_P005403	Unknown		2.229508409	4.68974152	-0.399084947	0.75833912	0.048111844	6.184227345
A_01_P010787	Unknown		1.248854856	2.376527105	-0.847463849	0.555760864	0.000559189	4.276168505
A_01_P007665	Unknown		1.69764417	3.243708488	-3.520926999	0.087115485	0.015996218	37.23457975
A_01_P014155	Unknown		4.359202998	20.52347325	-0.14609852	0.903691013	0.001898183	22.71071966
A_01_P007358	Unknown		1.365606155	2.576845705	-0.490019749	0.712015351	0.01602062	3.619087287
A_01_P003242	Unknown		1.503939801	2.836161725	-0.359112925	0.779643815	0.026749784	3.637765954
A_01_P020061	Unknown		1.49729074	2.823120561	-0.08937696	0.939928578	0.019400184	3.003547959
A_01_P013825	Unknown		1.374202554	2.592245855	-0.759797195	0.590579345	0.02314582	4.389326984
A_01_P002411	Unknown		2.406038047	5.300167843	-0.34199587	0.7889491	0.031380749	6.718009876
A_01_P018380	Unknown		1.118540602	2.171272203	-0.274624287	0.826665574	0.001518559	2.626542427
A_01_P003250	Unknown		1.748011559	3.358952884	-0.852751099	0.553727816	0.019745895	6.066072151

A_01_P018386	Unknown		1.573481628	2.976220937	-0.622572144	0.649511897	0.010157951	4.582242376
A_01_P007353	Unknown		1.316953627	2.491394748	0.106492378	1.076607498	0.000350744	2.314116104
A_01_P007045	Unknown		2.325951278	5.013962749	-0.174876221	0.885843519	0.027562118	5.660099829
A_01_P003292	Unknown		1.48281546	2.79493641	-0.412824575	0.751151294	0.012195698	3.720870125
A_01_P007942	Unknown		2.785694493	6.895687966	-0.676221143	0.625802296	0.006742733	11.018956
A_01_P000822	Unknown		3.034401141	8.193052961	-0.938076238	0.521928382	0.001537747	15.69765746
NM_138397	Unknown		2.872788707	7.324796642	-1.579054925	0.334701071	0.000564736	21.88459275
A_01_P006157	Unknown		1.162395783	2.238288161	-0.055459626	0.96228782	0.001158142	2.326006953
A_01_P018128	Unknown		1.789339618	3.456566349	-0.585587816	0.666377772	3.02E-05	5.187097309
A_01_P005239	Unknown		2.554005116	5.872623369	-0.46038794	0.726790799	0.00056633	8.080211499
A_01_P005341	Unknown		3.991184859	15.90253503	0.31834394	1.24689842	0.020133043	12.75367325
A_01_P012330	Unknown		3.455768129	10.97210272	-0.243356151	0.844777812	0.000118029	12.98815211
AI913343	wa11b04.x1 NCI_CGAP_Kid11 cDNA clone IMAGE:2297743 3' contains Alu repetitive element; sequence		1.067037532	2.095126758	-0.39988047	0.757921076	0.004397929	2.764307295
NM_002838	protein tyrosine phosphatase, receptor type, C	PTPRC	1.471895966	2.773861906		1		2.773861906
NM_001082	cytochrome P450, family 4, subfamily F, polypeptide 2	CYP4F2	1.950745726	3.865742993		1		3.865742993
NM_004733	solute carrier family 33 (acetyl-CoA transporter), member 1	SLC33A1	1.445943309	2.724409012		1		2.724409012
NM_007280	Opa interacting protein 5	OIP5	2.17985327	4.531074682		1		4.531074682
NM_001813	centromere protein E, 312kDa	CENPE	3.392971456	10.50476116		1		10.50476116
NM_024089	KDEL (Lys-Asp-Glu-Leu) containing 1	KDELC1	2.357716361	5.125583891		1		5.125583891
NM_000129	coagulation factor XIII, A1 polypeptide	F13A1	2.275542955	4.841798214		1		4.841798214
XR_012293	kelch-like 9	KLHL9	2.346987726	5.087608743		1		5.087608743
NM_173540	fucosyltransferase 11 (alpha (1,3) fucosyltransferase)	FUT11	1.577383179	2.98428057		1		2.98428057
NM_018685	anillin, actin binding protein	ANLN	2.052050057	4.146948287		1		4.146948287
NM_006419	chemokine (C-X-C motif) ligand 13	CXCL13	3.099121272	8.568966857		1		8.568966857
NM_004406	deleted in malignant brain tumors 1	DMBT1	2.02195739	4.06134445		1		4.06134445
NM_002445	macrophage scavenger receptor 1	MSR1	4.29586808	19.64197479		1		19.64197479
NM_004900	apolipoprotein B mRNA editing enzyme, catalytic polypeptide-like 3B	APOBEC3B	1.689169918	3.224711104		1		3.224711104
NM_138969	short chain dehydrogenase/reductase family 16C, member 5	SDR16C5	1.821118287	3.533549911		1		3.533549911
NM_001005738	formyl peptide receptor 2	FPR2	1.621408048	3.076751759		1		3.076751759
NM_001172	arginase, type II	ARG2	2.195406694	4.580187609		1		4.580187609
NM_207338	lactase-like	LCTL	3.66458186	12.68087033		1		12.68087033
NM_018131	centrosomal protein 55kDa	CEP55	1.931071224	3.813382437		1		3.813382437
NM_138715	macrophage scavenger receptor 1	MSR1	1.955293187	3.877947272		1		3.877947272
XM_370863			1.736948425	3.333293665		1		3.333293665
NM_152367	chromosome 1 open reading frame 161	C1orf161	0.953155379	1.936102562		1		1.936102562
NM_004004	gap junction protein, beta 2, 26kDa	GJB2	1.423437836	2.682239079		1		2.682239079
NM_032492	jagunal homolog 1	JAGN1	1.161849066	2.23744011		1		2.23744011
NM_007036	endothelial cell-specific molecule 1	ESM1	1.501506275	2.831381744		1		2.831381744
NM_001786	cyclin-dependent kinase 1	CDK1	0.829744168	1.777370155		1		1.777370155
NM_017839	lysophosphatidylcholine acyltransferase 2	LPCAT2	2.158400737	4.464197136		1		4.464197136

NM_000647			1.480312564	2.790091749	1	2.790091749
NM_005143	haptoglobin	HP	1.87225212	3.661036404	1	3.661036404
NM_003287	tumor protein D52-like 1	TPD52L1	2.161478168	4.473729936	1	4.473729936
XR_011794	poly(A)-specific ribonuclease (PARN)-like domain containing 1	PARNL1	1.807552799	3.500480085	1	3.500480085
NM_031415	gasdermin C	GSDMC	2.577733156	5.970009207	1	5.970009207
NM_018689			2.481034624	5.582977045	1	5.582977045
NM_000792	deiodinase, iodothyronine, type	DIO1	1.705887124	3.262294723	1	3.262294723
NM_016583	palate, lung and nasal epithelium associated	PLUNC	2.046757857	4.131764013	1	4.131764013
NM_024567	homeobox containing 1	HMBOX1	3.62955157	12.37667233	1	12.37667233
