# Supplementary material for: Transcriptional Reprogramming in Nonhuman Primate (Rhesus Macaque) Tuberculosis Granulomas
Source: PLoS One. 2010 Aug 31;5(8):e12266. doi: 10.1371/journal.pone.0012266 (PMC2930844; doi:10.1371/journal.pone.0012266)
Supplement: Table S2 — This table contains a comprehensive list of all NHP genes with a lower expression in a statistically significant manner, in early (week 4) TB lesions, relative to normal lungs. (0.26 MB PDF) [file pone.0012266.s002.pdf]

GeneName	Description	Symbol	Granuloma Av M	Granuloma Av Num. Ratio	Normal Av M	Normal Av Num. Ratio	*P*	Fold Change (Granuloma / Normal)
XR_011538	Homeobox protein Hox-D10	HOXD10	-5.41053652	-42.53376087	0.226315188	1.169843214	1.95E-05	36.358514
NM_021990	gamma-aminobutyric acid	GABRE	-4.621100344	-24.60876486	0.366017608	1.28879036	4.02E-07	19.09446689
NM_032405	transmembrane protease, serine 3	TMPRSS3	-4.094165408	-17.07916346	0.004758029	1.003303459	0.006810848	17.02292891
DQ159933	GluR5	GRIK1	-4.010438117	-16.11618222	0.125432105	1.090834402	0.001517741	14.77417854
NM_001937	dermatopontin	DPT	-3.833017962	-14.25126384	0.131938611	1.095765139	2.26E-05	13.00576495
NM_018218	ubiquitin specific protease 40	USP40	-4.507482344	-22.74507591	1.093001712	2.133174094	0.000694879	10.66255022
CO582652	glutamyl aminopeptidase (aminopeptidase A)	ENPEP	-3.966965376	-15.63779696	0.687492596	1.610482067	1.10E-06	9.71000999
NM_170699	G protein-coupled bile acid receptor 1	GPBAR1	-3.389205017	-10.47737219	0.147184572	1.107406252	0.000136763	9.461182081
NM_004636	sema domain, immunoglobulin domain	SEMA3B	-3.276737953	-9.691620765	0.151675931	1.110859171	1.08E-07	8.724436918
NM_016173	HemK methyltransferase family member 1	HEMK1	-3.334892029	-10.09026409	0.348146875	1.27292452	2.81E-05	7.926836144
NM_001034171	Pan troglodytes centromere protein J	CENPJ	-2.988764615	-7.937939764	0.008610726	1.005986347	9.66E-05	7.890703274
XM_379766	unc-84 homolog A	UNC84A	-3.089116984	-8.509751385	0.11138976	1.080268367	4.25E-06	7.87744198
NM_006925	splicing factor, arginine/serine-rich 5	SFRS5	-3.161155026	-8.945456	0.276590718	1.211328967	2.64E-05	7.384827941
NM_020639	receptor-interacting serine-threonine kinase 4	RIPK4	-2.961286351	-7.788180664	0.081161069	1.057869063	0.000171684	7.362140495
NM_025252	Ras association RalGDS/AF-6	RAPH1	-3.372176657	-10.35443305	0.511511096	1.425542544	1.64E-06	7.263503354
NM_000699	amylase, alpha 2A; pancreatic	AMY2A	-2.829907931	-7.110287675	0.068919389	1.048930717	7.10E-06	6.778605642
NM_133497	potassium channel, subfamily V, member 2	KCNV2	-3.513759793	-11.42212991	0.760910607	1.694559867	0.000244566	6.740469975
NM_001470	gamma-aminobutyric acid	GABBR1	-2.867698716	-7.298999467	0.165592724	1.121626798	3.63E-05	6.50751166
NM_024060	AHNAK nucleoprotein	AHNAK	-2.792130304	-6.926518096	0.120020585	1.086750368	4.17E-05	6.373605474
NM_012285	potassium voltage-gated channel, subfamily H	KCNH4	-3.6608596	-12.64819491	0.991755242	1.988602936	1.26E-05	6.360342069
NM_018964	solute carrier family 37 glycerol-3-phosphate transporter	SLC37A1	-2.688735166	-6.447478986	0.029797481	1.02086881	0.006482975	6.315678293
NM_002232	potassium voltage-gated channel, shaker-related subfamily, member 3	KCNA3	-3.065803617	-8.373342381	0.422891648	1.340611902	1.25E-06	6.245910816
NM_052870	sorting nexin associated golgi protein 1	SNAG1	-6.603084378	-97.21347362	3.974563322	15.72037058	0.00051607	6.18391743
NM_018281	hypothetical protein FLJ10948	FLJ10948	-2.706439937	-6.527090021	0.101560169	1.072933134	4.44E-05	6.083408011
NM_015100	pogo transposable element with ZNF domain	POGZ	-2.718986997	-6.584103418	0.148312417	1.108272319	6.75E-06	5.940871485
NM_003279	troponin C2, fast	TNNC2	-2.814361904	-7.034080783	0.24382797	1.184130405	0.008108486	5.940292347
NM_006766	MYST histone acetyltransferase monocytic leukemia	MYST3	-2.810701308	-7.016255611	0.261403265	1.198644022	2.84E-05	5.853494019
NM_001195	beaded filament structural protein 1, filensin	BFSP1	-2.608752457	-6.099759902	0.059982244	1.042452931	0.002837533	5.851352825
NM_173587	REST corepressor 2	RCOR2	-2.779286627	-6.865128034	0.233903048	1.176012217	0.000830607	5.83763326
U87259	oviductal glycoprotein	OVGP1	-2.768624069	-6.814576819	0.22917226	1.172162234	0.001390593	5.813680584
NM_018688	bridging integrator 3	BIN3	-2.892560279	-7.425871132	0.387918062	1.308503753	7.08E-05	5.675085849
NM_020734	KIAA1238 protein	KIAA1238	-2.931060807	-7.626709808	0.427545631	1.344943559	1.40E-06	5.670654173
XM_498519	hypothetical gene supported by AK124252	LOC440049	-2.91053318	-7.518960279	0.411895568	1.330432732	0.00017511	5.651514805
NM_021924	mucin and cadherin-like	MUCDHL	-5.820595402	-56.51631129	3.334050141	10.08437761	3.54E-07	5.604343023
NM_002152	histidine rich calcium binding protein	HRC	-6.1069784	-68.92609612	3.627010293	12.35489028	3.82E-05	5.578851335
NM_053017	ADP-ribosyltransferase 5	ART5	-2.601788018	-6.070384996	0.133787932	1.097170648	0.000653641	5.532762846
NM_145248	LOC122258	LOC122258	-3.527759505	-11.5335082	1.064402271	2.091303243	0.027770358	5.514986041
NM_000940	paraoxonase 3	PON3	-2.583315049	-5.993152351	0.138571022	1.100814228	0.000220386	5.444290415
NM_022844	myosin, heavy polypeptide 11, smooth muscle	MYH11	-2.733431188	-6.65035423	0.290154028	1.222770819	2.80E-06	5.438757719
NM_080861	SPRY domain-containing SOCS box protein SSB-3	SSB3	-2.456785288	-5.489920617	0.014102699	1.00982318	6.70E-06	5.436516735
NM_020795	neuroligin 2	NLGN2	-5.152155691	-35.55931656	2.725345487	6.613186037	0.000246737	5.377032547
NM_005876	aortic preferentially expressed protein 1	APEG1	-2.459088191	-5.498690903	0.041456298	1.029152159	9.57E-06	5.342932877

NM_147133	nuclear transcription factor, X-box binding 1	NFX1	-2.425850495	-5.373456827	0.017207056	1.011998433	0.00011288	5.30974817
NM_152459	hypothetical protein MGC45438	MGC45438	-2.45208372	-5.472058752	0.063423891	1.044942741	5.01E-06	5.236706792
AK097951	cDNA FLJ40632 fis, clone THYMU2015316		-3.040515809	-8.227851804	0.655988401	1.575695103	0.00758245	5.221728357
NM_134445	CD99 antigen-like 2	CD99L2	-3.483843772	-11.18771709	1.108484547	2.156190351	5.52E-06	5.18865001
NM_000526	keratin 14	KRT14	-5.936833788	-61.25831537	3.569168292	11.86934397	5.70E-07	5.161053176
NM_006843	serine dehydratase	SDS	-5.378760422	-41.60717472	3.031562184	8.176946403	7.78E-06	5.088351161
NM_030806	chromosome 1 open reading frame 21	C1orf21	-2.841804073	-7.169159912	0.494825778	1.409150574	0.003568485	5.087575483
NM_032607	cAMP responsive element binding protein 3-like 3	CREB3L3	-2.775554641	-6.847392165	0.441002311	1.357547154	0.000616099	5.043944252
NM_138934	palmitoyl-protein thioesterase 2	PPT2	-2.562069318	-5.905541361	0.229731733	1.172616882	0.000612325	5.036207009
NM_178354	late cornified envelope 1F	LCE1F	-2.729461261	-6.632079317	0.402704986	1.321984247	0.00119223	5.01676123
NM_014351	sulfotransferase family 4A, member 1	SULT4A1	-2.341710102	-5.069031403	0.016570783	1.011552209	0.010640748	5.01114165
NM_003970	myomesin	MYOM2	-2.58457931	-5.998406566	0.265013643	1.201647413	0.000250392	4.99181915
NM_004445	EphB6	EPHB6	-5.005106193	-32.11345965	2.688860179	6.448037699	1.86E-07	4.980346137
NM_019897	olfactory receptor, family 2, subfamily S, member 2	OR2S2	-2.79904704	-6.959805749	0.487174508	1.401696987	0.000375163	4.965271247
NM_001174	Rho GTPase activating protein 6	ARHGAP6	-2.78897632	-6.911392058	0.48288323	1.397533853	7.45E-06	4.945420138
NM_001630	annexin A8	ANXA8	-2.295024526	-4.907623337	0.011315342	1.007874036	2.96E-06	4.869282434
NM_207458	FLJ46026 protein	FLJ46026	-3.173643619	-9.023227866	0.892020926	1.855773871	0.000452132	4.862245346
NM_024007	early B-cell factor	EBF	-2.330559058	-5.030002295	0.061806012	1.04377157	0.029584193	4.819064287
NM_139025	a disintegrin-like and metalloprotease	ADAMTS13	-2.356189659	-5.120162716	0.093323956	1.066825308	0.00171171	4.799438742
NM_001519	BRF1 homolog, subunit of RNA polymerase III transcription initiation factor IIIB	BRF1	-2.95461556	-7.752252492	0.69863231	1.622965475	2.48E-06	4.776597291
NM_024980	G protein-coupled receptor 157	GPR157	-3.258914289	-9.572622974	1.004517803	2.006272821	0.002301082	4.771346585
NM_022450	rhomboid family 1	RHBDF1	-2.374443592	-5.185357982	0.133381406	1.096861528	2.03E-05	4.727449957
NM_001006657	WD repeat domain 35	WDR35	-2.359156289	-5.130702196	0.119335024	1.086234073	0.001778253	4.723385431
NM_144694	zinc finger protein 570	ZNF570	-2.954628463	-7.752321823	0.721003428	1.648328087	0.023982946	4.703142465
XR_013214	protein phosphatase 2, regulatory subunit B, beta isoform 1	LOC709587	-2.260127514	-4.7903382	0.029396349	1.020585004	2.65E-05	4.693717996
NM_024608	nei endonuclease VIII-like 1	NEIL1	-2.487604428	-5.608459009	0.280015047	1.214207549	5.21E-05	4.619028284
J04697	plasminogen	PLG	-2.653229188	-6.290737611	0.455223056	1.370994759	2.72E-05	4.588447598
NM_000495	collagen, type IV, alpha 5	COL4A5	-2.69122589	-6.458619775	0.497068467	1.411342821	2.50E-05	4.576223212
NM_004760	serine/threonine kinase 17a apoptosis-inducing	STK17A	-2.42774909	-5.380532985	0.234227339	1.176276593	0.044776664	4.574207306
NM_138703	melanoma antigen family E, 2	MAGEE2	-3.008865893	-8.049314322	0.81707454	1.761829774	5.60E-07	4.568724198
NM_016250	NDRG family member 2	NDRG2	-2.402651748	-5.287741858	0.218981086	1.163911275	3.78E-07	4.543079848
NM_018089	hypothetical protein FLJ10415	FLJ10415	-2.370718402	-5.171986115	0.187453691	1.138752081	1.20E-05	4.541801679
NM_032843	fibrinogen C domain containing 1	FIBCD1	-2.821408444	-7.06852131	0.644236005	1.56291141	0.00083025	4.52266281
NM_018011	hypothetical protein FLJ10154	FLJ10154	-2.635971636	-6.21593594	0.459517978	1.37508231	5.10E-06	4.520410085
NM_139290	angiopoietin 1	ANGPT1	-2.241296997	-4.728219452	0.069635834	1.049451747	0.000166009	4.505418631
NM_152526	amyotrophic lateral sclerosis 2	ALS2CR19	-5.322840901	-40.02531638	3.155544563	8.910735778	7.42E-06	4.491808238
NM_006772	synaptic Ras GTPase activating protein 1 homolog	SYNGAP1	-2.755515829	-6.752940419	0.601003596	1.516771326	0.000833459	4.452180959
NM_016290	receptor associated protein 80	RAP80	-2.588321229	-6.013984832	0.441203953	1.357736909	1.79E-05	4.42941839
CB550393	MMPL0003_B02 MMPL cDNA sequence		-2.219936554	-4.658729464	0.072956639	1.051870164	2.10E-05	4.42899668
NM_003317	thyroid transcription factor 1	TITF1	-2.284767541	-4.872855835	0.14298034	1.1041838	1.22E-06	4.413083978
NM_013271	proprotein convertase subtilisin/kexin type 1 inhibitor	PCSK1N	-2.721342912	-6.594864015	0.580761587	1.495638576	3.91E-05	4.409396843
NM_017797	BTB POZ	BTBD2	-2.515587055	-5.71830295	0.382697202	1.303777065	3.45E-05	4.385951481
NM_006226	phospholipase C-like 1	PLCL1	-6.291986379	-78.3567893	4.160277506	17.88003315	2.23E-07	4.382362641
NM_021827	hypothetical protein FLJ23514	FLJ23514	-2.218479117	-4.654025505	0.088682707	1.063398773	0.002740176	4.37655715
NM_004672	mitogen-activated protein kinase kinase kinase 6	MAP3K6	-2.44892732	-5.460099797	0.323918968	1.251726151	2.47E-05	4.362056184

NM_025008	thrombospondin repeat containing 1	TSRC1	-3.513339328	-11.41880148	1.389315241	2.619543175	0.011727671	4.359081229
NM_152683	hypothetical protein FLJ33167	FLJ33167	-2.356200506	-5.120201214	0.246318754	1.186176548	0.000166368	4.316559134
NM_182614	hypothetical protein MGC20579	MGC20579	-2.700527403	-6.500395071	0.599801216	1.515507735	0.000748137	4.289252321
NM_000023	sarcoglycan, alpha 50kDa dystrophin-associated glycoprotein	SGCA	-2.273145994	-4.833760504	0.175371825	1.12925541	0.006238723	4.2804847
NM_000691	aldehyde dehydrogenase 3 family, memberA1	ALDH3A1	-2.244457141	-4.738587707	0.149013908	1.108811333	0.000151183	4.273574382
NM_020824	Rho GTPase activating protein 21	ARHGAP21	-2.545472169	-5.837991736	0.465443861	1.380742088	0.000102636	4.228155125
NM_000518	hemoglobin, beta	HBB	-2.257360423	-4.781159128	0.192746016	1.142937106	1.58E-05	4.183221548
XR_012838	activating transcription factor 7 interacting protein 2	LOC710224	-2.130066742	-4.377377307	0.068205205	1.048411588	0.000337811	4.17524697
NM_198489	DLNB14	DLNB14	-2.132846321	-4.385819142	0.073876772	1.052541248	0.000483759	4.166885763
NM_001010972	zyxin	ZYX	-2.276286529	-4.84429435	0.222564057	1.166805472	1.12E-06	4.151758341
NM_203293	tripartite motif-containing 7	TRIM7	-2.211557503	-4.631750376	0.161585324	1.118515558	0.041618033	4.14097984
NM_007102	guanylate cyclase activator 2B	GUCA2B	-2.665681756	-6.345270888	0.616451755	1.533099949	0.000322598	4.138850108
NM_002062	glucagon-like peptide 1 receptor	GLP1R	-2.303585525	-4.936831912	0.257628219	1.195511675	0.024741181	4.129471936
NM_145297	zinc finger protein 626	ZNF626	-2.188485774	-4.558268077	0.145608347	1.10619701	0.000437968	4.120665702
NM_006533	melanoma inhibitory activity	MIA	-2.071960968	-4.20457788	0.034041324	1.023876227	3.95E-05	4.10652945
NM_017559	fibronectin type III domain containing 8	FNDC8	-2.786224203	-6.898220301	0.748560197	1.680115246	0.001784681	4.105801859
NM_001008218	amylase, alpha 1B; salivary	AMY1B	-2.584259181	-5.997075687	0.547850927	1.461906382	0.001268809	4.102229637
NM_003216	thyrotrophic embryonic factor	TEF	-2.537039591	-5.803968104	0.50666345	1.420760571	2.49E-05	4.085113439
NM_033312	CDC14 cell division cycle 14 homolog A	CDC14A	-2.815931511	-7.041737808	0.786335544	1.724688174	0.004343071	4.082904907
NM_005633	son of sevenless homolog 1	SOS1	-2.720628288	-6.591598128	0.701443103	1.626130569	0.000140708	4.053547883
NM_004991	myelodysplasia syndrome 1	MDS1	-2.043520437	-4.122502695	0.026176153	1.018309528	0.000408449	4.048378791
NM_020926	BCL6 co-repressor	BCOR	-2.378906896	-5.201424904	0.36686238	1.289545234	4.26E-07	4.033534278
NM_001004713	olfactory receptor, family 1, subfamily I, member 1	OR1I1	-2.708525305	-6.536531538	0.699531642	1.623977497	0.000332385	4.025013615
NM_147195	FLJ35740 protein	FLJ35740	-2.550864536	-5.859853254	0.546639903	1.460679747	0.000354739	4.011730337
XR_014601	olfactory receptor, family 7, subfamily D, member 4	LOC721322	-2.685128284	-6.431379778	0.696655654	1.620743351	0.000303194	3.968166691
NM_003325	HIR histone cell cycle regulation defective homolog A	HIRA	-2.473007446	-5.55199953	0.487040389	1.401566686	0.000581166	3.961281033
NM_002147	homeo box B5	HOXB5	-1.98393035	-3.955692688	0.003663671	1.00254269	0.012706811	3.945660097
NM_000422	keratin 17	KRT17	-2.583343959	-5.993272447	0.605305618	1.521300989	0.000473082	3.939570467
NM_144583	ATPase, H+ transporting, lysosomal 42kDa, V1 subunit C isoform 2	ATP6V1C2	-2.384295219	-5.220888034	0.418833596	1.336846291	0.000506594	3.905376459
NM_138286	hypothetical protein FLJ31526	LOC148213	-2.102848374	-4.295566401	0.141986881	1.103423706	0.002135647	3.892943734
NM_025158	RUN and FYVE domain containing 1	RUFY1	-2.050907922	-4.143666581	0.093952374	1.067290104	0.000219888	3.882418252
NM_144615	hypothetical protein MGC23244	MGC23244	-3.127827985	-8.741179623	1.171535431	2.252512997	2.66E-06	3.880634488
D86962	growth factor receptor-bound protein 10	GRB10	-1.973702535	-3.927748447	0.020176309	1.014083401	0.000124845	3.873200609
NM_032326	hypothetical protein MGC4618	MGC4618	-2.670663424	-6.367219167	0.72248554	1.65002232	1.77E-06	3.858868507
NM_025103	coiled-coil domain containing 2	CCDC2	-2.162258593	-4.47615065	0.214769878	1.160518787	2.20E-05	3.857025582
NM_031281	Fc receptor-like 5	FCRL5	-2.23671067	-4.71321231	0.291543859	1.223949352	0.005406935	3.850822996
NM_002630	progastricsin pepsinogen C	PGC	-2.010194238	-4.028364524	0.067923082	1.048206589	2.59E-06	3.843101701
NM_000847	glutathione S-transferase A3	GSTA3	-2.184045758	-4.544261188	0.250188325	1.189362361	1.67E-05	3.82075416
NM_024307	glycerophosphodiester phosphodiesterase domain containing 3	GDPD3	-2.072214899	-4.205318001	0.140583536	1.102350901	5.70E-06	3.814863306
NM_001007156	neurotrophic tyrosine kinase, receptor, type 3	NTRK3	-2.046928554	-4.132252905	0.115571996	1.083404506	0.000409897	3.814136718
NM_021211	transposon-derived Buster1 transposase-like protein	LOC58486	-2.059952584	-4.169725997	0.128950616	1.093498026	0.000124909	3.813199381
NM_178829	chromosome 7 open reading frame 34	C7orf34	-2.795635119	-6.943365509	0.868648955	1.825952145	8.50E-05	3.802599935
NM_024898	family with sequence similarity 31, member C	FAM31C	-3.953289128	-15.49025649	2.036366664	4.102111378	6.62E-05	3.776166726
NM_005909	microtubule-associated protein 1B	MAP1B	-2.285044035	-4.873789815	0.371886509	1.294043853	0.000492052	3.766325077

NM_020061	opsin 1	OPN1LW	-2.407100669	-5.304073139	0.495488325	1.409797865	0.003892864	3.762293356
NM_017619	U11/U12 snRNP 65K protein	FLJ25070	-2.030173401	-4.084539404	0.12376483	1.089574489	0.00038779	3.748747282
NM_006175	nebulin-related anchoring protein	NRAP	-2.300792727	-4.927284335	0.398298447	1.317952565	0.001090509	3.738590041
NM_152474	chromosome 19 open reading frame 18	C19orf18	-2.051452356	-4.145230585	0.151975521	1.111089876	0.010843182	3.730778827
NM_000758	colony stimulating factor 2 granulocyte-macrophage	CSF2	-2.289024145	-4.8872542	0.389845246	1.310252849	0.000146427	3.730008451
NM_005354	jun D proto-oncogene	JUND	-2.164025543	-4.481636204	0.265931122	1.202411841	9.61E-06	3.727205646
NM_012345	nuclear fragile X mental retardation protein interacting protein 1	NUFIP1	-1.917129283	-3.776708099	0.022611077	1.015796267	0.001612975	3.717977927
NM_032137	chromosome 3 open reading frame 20	C3orf20	-2.036488474	-4.102457744	0.147757528	1.107846138	0.009548263	3.703093421
NM_017820	hypothetical protein FLJ20433	FLJ20433	-2.405710057	-5.298963012	0.517872262	1.431841958	1.43E-05	3.700801601
NM_002971	special AT-rich sequence binding protein 1 binds to nuclear matrix/scaffold-associating DNA's	SATB1	-1.98271058	-3.952349642	0.095019366	1.068079746	0.000166581	3.700425608
NM_014871	ubiquitin specific protease 52	USP52	-2.046620276	-4.131370011	0.159977098	1.117269402	1.43E-05	3.697738436
NM_152479	hypothetical protein MGC33962	MGC33962	-2.997088553	-7.983871789	1.110987246	2.159934025	1.40E-05	3.69634984
NM_017451	BAI1-associated protein 2	BAIAP2	-1.91068474	-3.759875106	0.038478093	1.027029836	0.006736938	3.660921012
XR_014079	acid sphingomyelinase-like phosphodiesterase 3B isoform 1	LOC716655	-2.495666011	-5.639886037	0.631835507	1.549535178	0.000270063	3.639727655
NM_015662	selective LIM binding factor, rat homolog	SLB	-2.101667	-4.292050348	0.241456488	1.182185547	8.87E-07	3.630606344
NM_000033	ATP-binding cassette, sub-family D	ABCD1	-1.923813222	-3.794246008	0.066945533	1.04749658	0.006320779	3.622203721
NM_006385	zinc finger protein 211	ZNF211	-1.881488567	-3.684550346	0.025028188	1.017499572	0.000597821	3.621181223
NM_032293	GTPase activating Rap/RanGAP domain-like 3	GARNL3	-2.167183774	-4.491457786	0.317362193	1.246050201	0.000845662	3.604556045
NM_152314	hypothetical protein MGC34830	MGC34830	-2.540535047	-5.818047388	0.696939588	1.621062358	0.001624746	3.589033673
XM_166453	tau tubulin kinase 1	TTBK1	-2.026605314	-4.074449963	0.190380027	1.141064249	0.001207806	3.570745439
NM_005413	sine oculis homeobox homolog 3	SIX3	-2.429155355	-5.385780209	0.597581745	1.513178041	0.001146836	3.559250837
NM_152509	hypothetical protein FLJ31568	FLJ31568	-2.604010915	-6.079745423	0.772642194	1.708395726	0.000368206	3.558745395
NM_178565	hypothetical protein MGC35555	MGC35555	-2.28232334	-4.864607276	0.451088061	1.367070895	1.91E-05	3.558416242
NM_052949	RAS guanyl releasing protein 4	RASGRP4	-5.892754539	-59.4149688	4.074563852	16.8486822	4.34E-07	3.526386698
XM_376681	GTP-binding protein RAB19B	RAB19B	-2.173420385	-4.510915886	0.355990566	1.279864045	0.002134206	3.524527393
NM_001106	activin A receptor, type IIB	ACVR2B	-2.269341836	-4.821031433	0.459026055	1.374613521	4.43E-06	3.507190465
NM_031294	leucine rich repeat containing 48	LRRC48	-2.429085976	-5.385521214	0.619314541	1.536145149	0.000296083	3.50586741
NM_015490	SEC31-like 2	SEC31L2	-1.900700775	-3.733945253	0.091497326	1.065475431	9.55E-05	3.504487429
NM_181806	2-aminoadipic 6-semialdehyde dehydrogenase	NRPS998	-1.967336019	-3.910453746	0.159548913	1.116937851	0.000251449	3.501048643
NM_007148	zinc finger protein 179	ZNF179	-1.82909843	-3.553149593	0.026260669	1.018369184	7.70E-05	3.489058437
NM_080385	carboxypeptidase A5	CPA5	-2.762363152	-6.785067424	0.963925476	1.95061016	0.00026554	3.478433346
NM_013353	tropomodulin 4	TMOD4	-1.90378369	-3.741932904	0.110926519	1.079921555	0.000688439	3.465004368
NM_001008710	RNA binding protein with multiple splicing	RBPMS	-2.070533058	-4.200418452	0.278501891	1.212934707	0.000407608	3.463021074
NM_173674	discoidin, CUB and LCCL domain containing 1	DCBLD1	-2.507632389	-5.686860397	0.716943107	1.643695553	0.023149641	3.459801534
NM_058175	collagen, type VI, alpha 2	COL6A2	-2.305758461	-4.944273193	0.515912754	1.429898512	0.000343982	3.457779102
NM_005609	phosphorylase, glycogen; muscle	PYGM	-2.291612864	-4.89603158	0.508669012	1.422737014	0.018057946	3.441276589
NM_198488	FLJ46072 protein	FLJ46072	-2.186903896	-4.553272795	0.408701949	1.32749088	0.000478323	3.429984239
NM_022160	DMRT-like family A1	DMRTA1	-2.315663093	-4.978334236	0.537934765	1.451892627	0.000336054	3.428858404
NM_033033	keratin, hair, basic, 2	KRTHB2	-2.142786302	-4.416141204	0.365447172	1.288280878	0.006788758	3.427933519
NM_007112	thrombospondin 3	THBS3	-2.119023537	-4.343998294	0.347081289	1.271984675	7.68E-06	3.415134145
NM_015528	ring finger protein 167	RNF167	-1.865456596	-3.643832382	0.102138026	1.073362973	1.83E-06	3.394781145
NM_004349	core-binding factor, runt domain, alpha subunit 2; translocated to, 1; cyclin D-related	CBFA2T1	-2.093931878	-4.269099767	0.332316838	1.259033647	0.005388937	3.390774963
NM_014433	rhabdoid tumor deletion region gene 1	RTDR1	-5.804245801	-55.87944557	4.044849989	16.50521457	8.80E-06	3.385563109

NM_001002036	astacin-like metalloendopeptidase	ASTL	-2.095931546	-4.275021117	0.346235367	1.271239067	0.003443154	3.362877391
XR_010449	heat shock transcription factor 4	HSF4	-1.96714086	-3.9099248	0.220777607	1.165361543	0.003443669	3.355117409
NM_019002	ETAA16 protein	ETAA16	-1.769633833	-3.409674057	0.031652654	1.022182399	4.10E-05	3.335680659
NM_033208	tigger transposable element derived 7	TIGD7	-1.765271064	-3.399378636	0.028489345	1.019943577	0.019499596	3.332908519
XR_010179	short-chain dehydrogenase/reductase 10 isoform c		-1.750331322	-3.364358213	0.014286991	1.009952185	0.000174006	3.331205441
NM_014942	ankyrin repeat domain 6	ANKRD6	-1.780003334	-3.434269681	0.044435111	1.031279301	0.012625739	3.330106283
NM_005187	core-binding factor, runt domain, alpha subunit 2; translocated to, 3	CBFA2T3	-1.822581233	-3.537134878	0.087681801	1.062661269	0.016430153	3.328562903
NM_145056	thymus expressed gene 3-like	MGC15476	-2.068614981	-4.194837663	0.334945273	1.261329561	0.00060092	3.32572691
NM_145912	NFAT activating protein with ITAM motif 1	NFAM1	-2.404042076	-5.292840121	0.671933858	1.593207148	0.001935204	3.322129282
NM_024757	euchromatic histone methyltransferase 1	EHMT1	-1.940997099	-3.839709322	0.208939025	1.155837855	0.000331466	3.322013816
NM_018257	chromosome 20 open reading frame 36	C20orf36	-1.998271906	-3.995211574	0.273565818	1.208791833	0.002036528	3.305127868
NM_020747	zinc finger protein 608	ZNF608	-2.133579066	-4.388047266	0.409375475	1.328110767	0.000200838	3.303976878
NM_015404	deafness, autosomal recessive 31	DFNB31	-1.719862234	-3.294049499	0.000215532	1.000149407	0.003166935	3.29355742
XM_171060	zinc finger protein 620	ZNF620	-1.850784387	-3.606962408	0.136517882	1.099248742	0.002325429	3.281297735
NM_178026	gamma-glutamyltransferase-like 3	GGTL3	-1.886571377	-3.697554409	0.17323587	1.127584749	5.10E-05	3.279180933
NM_000937	polymerase RNA DNA directed	POLR2A	-2.330293715	-5.029077251	0.623037163	1.54011402	0.000582267	3.265392812
NM_002077	golgi autoantigen, golgin subfamily a, 1	GOLGA1	-1.741818322	-3.344564394	0.037828612	1.026567585	0.000791127	3.258007014
NM_003877	suppressor of cytokine signaling 2	SOCS2	-2.014484026	-4.04036051	0.312512548	1.241868613	2.31E-05	3.253452472
NM_177528	sulfotransferase family, cytosolic, 1A, phenol-preferring, member 2	SULT1A2	-1.8254104	-3.544078108	0.123466139	1.08934893	4.86E-06	3.253391095
NM_014935	pleckstrin homology domain containing, family A member 6	PLEKHA6	-2.041448233	-4.116585622	0.340254186	1.265979625	0.01158792	3.251699744
NM_145170	tetratricopeptide repeat domain 18	TTC18	-1.77114738	-3.413253058	0.077578431	1.05524532	0.007184525	3.23455882
XM_496422	cDNA sequence BC004853	LOC440699	-2.461512583	-5.507939004	0.768929606	1.704005044	0.000268659	3.232349002
NM_012433	splicing factor 3b, subunit 1, 155kDa	SF3B1	-1.700975318	-3.251206784	0.010203193	1.007097382	1.63E-05	3.228294345
NM_001454	forkhead box J1	FOXJ1	-2.24826582	-4.751113989	0.558654232	1.472894638	1.75E-05	3.225698477
NM_173462	papilin, proteoglycan-like sulfated glycoprotein	PAPLN	-1.709528623	-3.270539465	0.02120291	1.014805266	0.006018999	3.22282469
XR_013912	hypothetical protein LOC718426	LOC718426	-2.340332955	-5.064194989	0.654456982	1.574023393	0.004154481	3.217356878
NM_001002017	host cell factor C1 regulator 1 XPO1 dependant	HCFC1R1	-1.670857458	-3.184037789	0.003625318	1.002516039	0.004892741	3.176046733
NM_033438	SLAM family member 9	SLAMF9	-2.100566496	-4.288777572	0.441930706	1.358421037	0.00138776	3.157178412
NM_006105	Rap guanine nucleotide exchange factor	RAPGEF3	-1.665755404	-3.172797407	0.009307779	1.006472518	0.000131461	3.152393484
NM_033310	potassium channel, subfamily K, member 4	KCNK4	-2.789480396	-6.913807313	1.133191293	2.193433994	0.004023224	3.152047124
NM_182964	neuron navigator 2	NAV2	-1.845404008	-3.593535674	0.191351771	1.141833085	6.85E-06	3.147163733
NM_016828	8-oxoguanine DNA glycosylase	OGG1	-2.092252235	-4.264132403	0.440745871	1.357305871	0.002143667	3.141614941
XR_013013	Zinc finger CW-type PWWP domain protein 1 homolog	LOC711911	-1.662784475	-3.166270417	0.013232193	1.009214048	0.011969082	3.137362608
NM_024165	PHD finger protein 1	PHF1	-1.653471799	-3.145897792	0.007206946	1.005007972	0.000117367	3.130221728
NM_014380	nerve growth factor receptor TNFRSF16	NGFRAP1	-1.83613151	-3.570513343	0.191281131	1.141777178	0.016547518	3.127154239
NM_014555	transient receptor potential cation channel, subfamily M, member 5	TRPM5	-2.036200886	-4.101640038	0.392490741	1.312657687	1.68E-06	3.12468367
CN804253	PRO0659	PRO0659	-1.781401755	-3.437600173	0.137709924	1.100157384	1.68E-05	3.124644005
NM_006653	fibroblast growth factor receptor substrate 3	FRS3	-1.778023167	-3.429559218	0.135649748	1.098587474	0.000172244	3.121789843
NM_005808	CTD carboxy-terminal domain, RNA polymerase II, polypeptide A	CTDSPL	-1.818977426	-3.528310252	0.178606945	1.131790509	0.000138959	3.117458773
NM_178835	hypothetical protein LOC152485	LOC152485	-1.697554966	-3.243507929	0.063139509	1.044736784	0.001308795	3.104617333
NM_031264	mucin and cadherin-like	MUCDHL	-6.155715801	-71.29434787	4.522615818	22.98492125	1.83E-06	3.101787781
NM_152722	hypothetical protein FLJ25530	FLJ25530	-6.091482133	-68.18970949	4.459218982	21.9967577	5.02E-06	3.099989118

NM_025074	Fraser syndrome 1	FRAS1	-1.653005553	-3.144881274	0.022915491	1.016010626	0.010608325	3.095323211
NM_019591	zinc finger protein 26	ZNF26	-1.853679939	-3.614209009	0.225625274	1.169283914	0.001189127	3.090959317
NM_182528	complement component 1, q subcomponent-like 2	C1QL2	-1.99885085	-3.996815147	0.374437328	1.296333867	0.007841929	3.083168039
NM_032156	C1q domain containing 1	C1QDC1	-2.04130798	-4.116185441	0.417403249	1.335521544	9.94E-05	3.0820809
NM_005451	PDZ and LIM domain 7	PDLIM7	-1.703637377	-3.257211446	0.080371166	1.057290018	0.003863968	3.080717109
NM_000229	lecithin-cholesterol acyltransferase	LCAT	-1.721479595	-3.297744427	0.100571611	1.072198195	0.0004291	3.075685488
NM_000336	sodium channel, nonvoltage-gated 1, beta	SCNN1B	-3.252354459	-9.529195759	1.63326855	3.10215022	1.21E-06	3.071803454
NM_007351	multimerin 1	MMRN1	-1.962061059	-3.896181968	0.343855955	1.269144163	0.018872301	3.069928604
NM_006688	complement component 1, q subcomponent-like 1	C1QL1	-1.973876438	-3.928221928	0.358049934	1.281692285	1.85E-06	3.064871322
NM_003854	interleukin 1 receptor-like 2	IL1RL2	-1.730536555	-3.318512146	0.11748322	1.084840707	0.018474025	3.058985641
NM_207392	KIPV467	UNQ467	-5.855464618	-57.89892343	4.242989508	18.93507865	1.74E-05	3.05775986
NM_198537	FLJ44968 protein	FLJ44968	-2.321310176	-4.997858916	0.709554116	1.635298629	0.000162186	3.05623623
NM_007322	RAN binding protein 3	RANBP3	-1.633284023	-3.102183491	0.022541621	1.015747364	0.010651064	3.054089629
NM_020231	x 010 protein	MDS010	-1.706938249	-3.264672446	0.097670304	1.070044136	0.006175255	3.050969896
NM_001991	enhancer of zeste homolog 1	EZH1	-2.012056461	-4.033567673	0.402959505	1.322217492	5.09E-05	3.050608314
CN645828	ILLUMIGEN_MCQ_24955 Katze_MMBR cDNA clone IBIUW:10644 5' Bases 1 to 711 highly human LOC254531	Hs.352614	-2.321807756	-4.999582953	0.714184086	1.640555141	0.001206719	3.047494612
NM_017503	surfeit 2	SURF2	-1.634528992	-3.104861667	0.029552254	1.0206953	0.001018875	3.041908459
NM_032818	chromosome 9 open reading frame 100	C9orf100	-1.964082445	-3.901644806	0.364684619	1.287600122	0.002949545	3.030168093
NM_013957	neuregulin 1	NRG1	-1.879221547	-3.67876507	0.281157988	1.215169857	0.000960135	3.027366957
NM_153361	hypothetical protein MGC42105	MGC42105	-1.751875241	-3.367960554	0.15450626	1.113040632	0.000150653	3.025909797
NM_014964	epsin 2	EPN2	-1.629047883	-3.093088008	0.043938228	1.030924176	0.000405876	3.000306015
NM_213726	inhibitor of CDK interacting with cyclin A1	INCA1	-2.193501246	-4.574142292	0.608708364	1.52489337	0.00010208	2.999647308
NM_175850	DNA	DNMT3B	-1.664242756	-3.169472513	0.079890233	1.056937621	0.00339499	2.998731855
NM_017613	downstream neighbor of SON	DONSON	-1.67226149	-3.187138005	0.090184619	1.064506396	0.000155719	2.994005499
NM_177417	kinesin light chain 2-like	KLC2L	-1.712940687	-3.278283649	0.132356027	1.096082224	0.000307686	2.990910334
NM_012183	forkhead box D3	FOXD3	-1.742452745	-3.346035484	0.16278671	1.119447376	0.007787956	2.989006501
NM_003193	tubulin-specific chaperone e	TBCE	-1.865057052	-3.642823389	0.287910956	1.220871158	4.55E-05	2.983790195
NM_004924	actinin, alpha 4	ACTN4	-2.71159471	-6.550453133	1.136738164	2.198833195	0.000356087	2.979058688
NM_000778	cytochrome P450, family 4, subfamily A, polypeptide 11	CYP4A11	-6.768964459	-109.0589575	5.200926455	36.78196003	2.64E-06	2.965012125
NM_016408	CDK5 regulatory subunit associated protein 1	CDK5RAP1	-1.653953287	-3.146947886	0.088442666	1.063221856	8.11E-05	2.959822419
NM_005232	EphA1	EPHA1	-2.270621964	-4.825311116	0.708368265	1.633955015	0.00036576	2.95314808
NM_173558	FGD1 family, member 2	FGD2	-1.817530396	-3.524773115	0.257049574	1.195032268	0.002230542	2.949521288
NM_004714	dual-specificity tyrosine-	DYRK1B	-1.568828878	-2.966637966	0.010080668	1.007011855	0.002127486	2.945981173
NM_003283	troponin T1, skeletal, slow	TNNT1	-1.674564421	-3.192229602	0.116761694	1.084298289	0.018663952	2.94405113
NM_030955	a disintegrin-like and metalloprotease	ADAMTS12	-2.297279229	-4.91529917	0.741829921	1.672295642	1.56E-06	2.939252514
NM_016611	potassium channel, subfamily K, member 4	KCNK4	-4.57729876	-23.87284762	3.023061828	8.128909508	1.02E-08	2.936783537
NM_178448	chromosome 9 open reading frame 140	C9orf140	-1.967673715	-3.911369184	0.415493338	1.333754684	3.16E-05	2.932600148
NM_032205	CGI-72 protein	CGI-72	-1.679293139	-3.202709931	0.127908983	1.0927088	0.001312363	2.930982099
NM_005167	protein phosphatase 1J PP2C domain containing	PPM1J	-2.235749536	-4.71007338	0.684807975	1.607488008	0.00034235	2.93008306
XR_014158	Cytochrome P450 4F11	CYPIVF11	-1.974505394	-3.929934844	0.42419365	1.341822323	0.000827573	2.928804193
NM_016335	proline dehydrogenase	PRODH	-1.939984392	-3.837014967	0.392254017	1.312442317	0.005774199	2.923568462
NM_152343	hypothetical protein FLJ25414	FLJ25414	-2.5240013	-5.751751316	0.978671306	1.970649643	0.001367578	2.918708222
NM_019858	gene rich cluster, A gene	GRCA	-1.744287532	-3.350293603	0.20006135	1.148747204	0.000280387	2.916475958
NM_007054	kinesin family member 3A	KIF3A	-6.144437024	-70.73915221	4.601140815	24.27064955	1.14E-07	2.914596581
NM_020987	ankyrin 3, node of Ranvier	ANK3	-1.725103145	-3.306037628	0.182289476	1.134683136	0.005725765	2.913621895

NM_003427	zinc finger protein 76	ZNF76	-1.629678594	-3.094440525	0.089175654	1.063762182	0.00022932	2.908958954
NM_148888	chemokine C-C motif ligand 25	CCL25	-1.65501932	-3.149274081	0.115082813	1.083037212	0.000326456	2.90781706
NM_020982	claudin 9	CLDN9	-2.284249009	-4.871104755	0.746433551	1.677640448	2.50E-06	2.903545131
NM_024954	ubiquitin domain containing 1	UBTD1	-1.971792204	-3.922550998	0.435889971	1.352745058	0.000402121	2.899697156
NM_014067	LRP16 protein	LRP16	-2.001494989	-4.004147138	0.466177619	1.381444516	9.33E-05	2.898521867
NM_015023	WD and tetratricopeptide repeats 1	WDTC1	-1.539572543	-2.907083565	0.004307536	1.002990218	0.000455125	2.898416667
NM_004817	tight junction protein 2	TJP2	-1.773610203	-3.419084795	0.239011943	1.180184113	0.0002502	2.897077462
NM_001346	diacylglycerol kinase, gamma 90kDa	DGKG	-1.741765373	-3.344441645	0.207750895	1.154886358	0.0024729	2.895905405
NM_024333	fibronectin type III and SPRY domain containing 1	FSD1	-1.619168733	-3.071979807	0.085550024	1.061092203	0.000866851	2.895111092
NM_181353	inhibitor of DNA binding 1, dominant negative helix-loop-helix protein	ID1	-1.781629854	-3.438143722	0.253273519	1.191908526	0.002707779	2.884570122
NM_004209	synaptogyrin 3	SYNGR3	-1.583957883	-2.997911684	0.05636406	1.039841803	0.000279886	2.883045935
NM_001632	alkaline phosphatase, placental	ALPP	-1.57629678	-2.982034151	0.050266801	1.035456395	0.013344795	2.879922481
NM_001501	gonadotropin-releasing hormone 2	GNRH2	-1.857673501	-3.624227455	0.333003308	1.259632868	0.004035196	2.87720934
NM_014827	zinc finger CCCH-type containing 11A	ZC3H11A	-1.691597821	-3.230142521	0.167439577	1.123063558	0.000129024	2.876188528
NM_033513	chromosome 19 open reading frame 20	C19orf20	-2.388917266	-5.23764132	0.865365435	1.821801071	4.25E-05	2.874979823
NM_170692	RAS protein activator like 2	RASAL2	-1.793335876	-3.466154291	0.273431965	1.208679687	0.000524774	2.867719487
NM_147130	natural cytotoxicity triggering receptor 3	NCR3	-2.51938602	-5.733380471	1.000944468	2.001309739	0.028928568	2.864814156
NM_153488	melanoma antigen family A, 2B	MAGEA2B	-1.875512208	-3.669318681	0.358990556	1.282528207	0.000557694	2.861004272
XR_013910	meningioma expressed antigen 5 hyaluronidase	MGEA5	-1.755743564	-3.377003242	0.245094982	1.185170796	5.09E-05	2.84938108
NM_007346	opioid growth factor receptor	OGFR	-2.084744577	-4.241999871	0.574570098	1.489233627	6.26E-05	2.84844486
XR_013613	ZXD family zinc finger C isoform 2	LOC717391	-1.679069985	-3.202214577	0.168984708	1.124267007	2.75E-05	2.848268745
XR_014659	formiminotransferase cyclodeaminase	LOC721818	-1.575703602	-2.980808311	0.065951355	1.046774986	0.014215976	2.84761133
NM_001009598	retinoid X receptor, gamma	RXRG	-2.529736282	-5.774661106	1.025519461	2.03569223	0.031751114	2.836706365
NM_003243	transforming growth factor, beta receptor III betaglycan, 300kDa	TGFBR3	-1.586875572	-3.003980759	0.099947566	1.07173451	4.89E-05	2.802915023
NM_002043	gamma-aminobutyric acid	GABRR2	-2.01344783	-4.037459616	0.526680043	1.440610219	0.003807967	2.80260376
NM_018254	REST corepressor 3	RCOR3	-1.562512186	-2.95367724	0.07867759	1.056049595	0.018353159	2.796911483
NM_152901	pyrin-domain containing protein 1	PYC1	-2.04539349	-4.127858422	0.562139086	1.476456739	0.000213208	2.795786908
NM_203471	lectin, galactoside-binding, soluble, 14	LGALS14	-1.71282276	-3.278015691	0.236940542	1.178490837	0.006309506	2.78153685
NM_016368	myo-inositol 1-phosphate synthase A1	ISYNA1	-1.517876898	-2.863693119	0.042045198	1.029572338	0.000144165	2.781439451
NM_002380	matrilin 2	MATN2	-1.634971538	-3.105814228	0.159431361	1.116846846	0.007564622	2.780877467
NM_006321	ariadne homolog 2	ARIH2	-1.844807038	-3.59204902	0.371335695	1.293549888	0.001257696	2.77689253
XR_009782	hypothetical protein LOC694002	LOC694002	-1.567008115	-2.962896262	0.095461269	1.068406952	0.022273565	2.773190736
NM_015897	protein inhibitor of activated STAT, 4	PIAS4	-2.293459303	-4.902301798	0.826422645	1.773282815	0.000354242	2.764534657
NM_018687	hepatocellular carcinoma-associated gene TD26	LOC55908	-2.04118151	-4.115824624	0.577399224	1.492156881	0.006172256	2.758305562
NM_207324	hypothetical protein LOC147650	LOC147650	-1.822980723	-3.538114465	0.367957434	1.290524413	4.85E-05	2.741609867
NM_016484	hypothetical protein LOC51248	LOC51248	-1.704094679	-3.258244073	0.249276234	1.188610668	0.018633448	2.741220622
NM_014497	zinc finger protein 638	ZNF638	-1.721516204	-3.29782811	0.266871035	1.203195465	7.23E-05	2.740891405
NM_201402	deubiquitinating enzyme 3	DUB3	-2.650209434	-6.277584026	1.197245913	2.293015191	0.023360455	2.737698403
NM_003899	Rho guanine nucleotide exchange factor	ARHGEF7	-1.776677671	-3.42636221	0.327512407	1.254847814	0.000616912	2.730500202
NM_173156	chromosome 1 open reading frame 16	C1orf16	-1.49256881	-2.813895612	0.04409019	1.031032772	0.006025487	2.729200943
NM_033553	guanylate cyclase activator 2A	GUCA2A	-2.352669883	-5.107686164	0.905320228	1.87296018	0.003484874	2.727066073
NM_006779	CDC42 effector protein Rho GTPase binding	CDC42EP2	-1.57298351	-2.975193518	0.127125169	1.092115294	0.000577771	2.72424856
NM_177529	sulfotransferase family, cytosolic, 1A, phenol-preferring, member 1	SULT1A1	-1.643247388	-3.123681561	0.200590628	1.14916872	1.05E-07	2.7182097

XR_013358	Calretinin	CR	-1.676496385	-3.196507292	0.234073513	1.17615118	0.001563616	2.717769065
NM_144617	heat shock protein, alpha-crystallin-related, B6	HSPB6	-1.481236509	-2.791879179	0.040342637	1.02835803	0.008570112	2.714890239
NM_144990	hypothetical protein FLJ23878	FLJ23878	-1.505546523	-2.839322103	0.068920048	1.048931196	3.05E-05	2.706871637
XM_027330	RNA binding motif protein 25	RBM25	-1.718613978	-3.291200637	0.282148964	1.216004834	0.000497692	2.706568712
CO582642	Hs.521442		-1.521045224	-2.869989034	0.086964796	1.062133268	9.74E-05	2.702098803
NM_178822	immunoglobulin superfamily, member 10	IGSF10	-1.571913968	-2.972988675	0.141018671	1.102683434	0.00440243	2.696139785
NM_184244	RNA-binding region RNP1, RRM	RNPC2	-1.559237136	-2.946979727	0.128674156	1.093288502	0.000198371	2.695518815
NM_030655	DEAD/H Asp-Glu-Ala-Asp/His	DDX11	-1.912811976	-3.765423085	0.483373192	1.398008557	0.031380122	2.693419196
NM_205545	LY6/PLAUR domain containing 2	LYPDC2	-2.29718948	-4.914993401	0.870939103	1.82885298	5.91E-05	2.687473216
NM_032133	MYCBP associated protein	MYCBPAP	-1.507664861	-2.843494198	0.081692921	1.058259119	0.001353892	2.68695459
XR_010742	BTAF1 RNA polymerase II, B-TFIID transcription factor-associated, 170kDa	BTAF1	-1.531541376	-2.89094543	0.107595991	1.077431382	0.000165305	2.683182873
NM_016291	inositol hexaphosphate kinase 2	IHPK2	-1.444215077	-2.721147343	0.022312858	1.015586314	9.02E-05	2.679385598
NM_006671	solute carrier family 1 glutamate transporter	SLC1A7	-1.72009047	-3.294570661	0.298986849	1.23028013	0.015102055	2.677902846
NM_012418	fascin homolog 2, actin-bundling protein, retinal	FSCN2	-1.507510486	-2.843189948	0.087749247	1.06271095	0.005399697	2.675412302
NM_030895	zinc finger protein 696	ZNF696	-2.104563991	-4.300677621	0.686196355	1.609035722	0.004626154	2.672829175
NM_017877	chromosome 2 open reading frame 18	C2orf18	-1.80303392	-3.489532866	0.385605273	1.306407766	0.005278758	2.671090111
NM_024927	hypothetical protein FLJ21019	FLJ21019	-1.47204051	-2.774139833	0.055825364	1.039453603	0.002297393	2.668844309
CN643965	Hs.492201		-1.765210598	-3.399236165	0.349339874	1.273977567	0.002236295	2.668207237
NM_024895	PDZ domain containing 7	PDZK7	-1.628853354	-3.092670972	0.213011327	1.159105052	1.45E-05	2.668154164
NM_080632	UPF3 regulator of nonsense transcripts homolog B	UPF3B	-1.454663657	-2.74092653	0.04008591	1.028175051	0.012390767	2.665816999
CK231504	Hs.500464		-2.003926102	-4.010900291	0.590604713	1.505877812	0.005846119	2.663496507
NM_000207	insulin	INS	-2.028574524	-4.080015193	0.617846696	1.534583019	0.001102238	2.65871259
NM_173854	solute carrier family 41, member 1	SLC41A1	-1.639051701	-3.114610379	0.230245394	1.173034458	0.00044017	2.655173816
NM_002744	protein kinase C, zeta	PRKCZ	-1.44118654	-2.715441043	0.03295685	1.023106869	0.000109141	2.654112806
XR_013072	glutamate receptor, metabotropic 5	GRM5	-1.426453482	-2.68785159	0.018528319	1.012925675	0.042933704	2.65355263
NM_182982	G protein-coupled receptor kinase 4	GRK4	-1.468598046	-2.767528255	0.061232968	1.043357062	0.002242779	2.652522665
NM_147161	thioesterase, adipose associated	THEA	-3.075849943	-8.43185433	1.670403291	3.183035596	3.01E-06	2.648997812
NM_001012270	baculoviral IAP repeat-containing 5	BIRC5	-1.671288956	-3.184990249	0.266824232	1.203156433	0.012294723	2.647195462
XR_014528	insulin receptor-related receptor	INSRR	-2.301704522	-4.930399405	0.898346712	1.863928749	0.000660634	2.64516517
NM_178569	CEI protein	CEI	-1.486490595	-2.802065335	0.085700345	1.061202769	0.000535285	2.640461764
NM_016228	aminoadipate aminotransferase	AADAT	-1.668093244	-3.177942991	0.270862724	1.20652911	0.008038753	2.633954676
NM_152733	BTB POZ	BTBD2	-1.998080974	-3.994682868	0.602334114	1.518170805	0.000616746	2.631247323
NM_173596	solute carrier family 39 metal ion transporter	SLC39A5	-1.988024837	-3.966935217	0.59296358	1.508341999	0.002638569	2.629997189
NM_001882	corticotropin releasing hormone binding protein	CRHBP	-2.173282867	-4.510485925	0.780212001	1.71738322	9.01E-05	2.626371256
NM_001261	cyclin-dependent kinase 9	CDK9	-1.639551368	-3.115689288	0.251308222	1.190285965	6.15E-06	2.617597265
XM_375633	solute carrier family 8 sodium-calcium exchanger	SLC8A2	-2.255148276	-4.773833588	0.867373486	1.824338556	0.006797574	2.616747626
NM_015526	CLIP-170-related protein	CLIPR-59	-1.581663865	-2.993148515	0.194304424	1.14417238	0.003631126	2.615994379
NM_002224	inositol 1,4,5-triphosphate receptor, type 3	ITPR3	-1.420133404	-2.676102554	0.033585366	1.023552686	3.94E-05	2.614523501
NM_012384	glucocorticoid modulatory element binding protein 2	GMEB2	-1.495153849	-2.818942106	0.109134756	1.078581173	5.49E-05	2.613565095
NM_023004	reticulon 4 receptor	RTN4R	-1.786674887	-3.450187786	0.40156178	1.320937109	0.005184437	2.61192434
NM_018259	tetratricopeptide repeat domain 17	TTC17	-1.540942927	-2.909846252	0.156756077	1.114777722	0.001113897	2.610247939
NM_004907	immediate early response 2	IER2	-2.10840754	-4.312150528	0.726111742	1.654174853	0.000123923	2.606828727
NM_019099	hypothetical protein LOC55924	LOC55924	-1.889967792	-3.706269505	0.50817656	1.422251458	0.000104698	2.605917177
XR_011148	CG8841-PA, isoform A	LOC699954	-1.46583991	-2.762242365	0.085630425	1.061151339	0.038899802	2.603061659
NM_198698	keratin associated protein 12-4	KRTAP12-4	-1.665510939	-3.172259823	0.286577846	1.219743543	1.97E-05	2.600759677

NM_004790	solute carrier family 22 organic anion transporter	SLC22A6	-2.001604175	-4.00445019	0.624826782	1.54202567	0.004806636	2.596876477
NM_015665	achalasia, adrenocortical insufficiency, alacrimia	AAAS	-1.784903566	-3.445954294	0.408763086	1.327547136	0.000242666	2.595730276
NM_130434	dipeptidylpeptidase 8	DPP8	-1.612115242	-3.056997224	0.23820366	1.17952309	4.55E-05	2.591723088
NM_005586	MyoD family inhibitor	MDFI	-1.782621891	-3.440508698	0.410917214	1.329530813	0.013395312	2.587761535
NM_014419	dickkopf-like 1	DKKL1	-2.464439471	-5.519124656	1.09334884	2.133687419	1.56E-05	2.586660355
NM_058183	SON DNA binding protein	SON	-1.672127607	-3.18684225	0.301824739	1.232702566	0.001730417	2.58524833
NM_017784	oxysterol binding protein-like 10	OSBPL10	-1.874682611	-3.667209308	0.504720908	1.41884885	0.000262981	2.584637051
NM_017916	hypothetical protein FLJ20643	FLJ20643	-1.415783132	-2.668045245	0.048502086	1.034190592	1.84E-05	2.579839022
NM_006137	CD7 antigen	CD7	-2.307052969	-4.948711604	0.940172334	1.918757426	0.003326237	2.579123102
NM_013351	T-box 21	TBX21	-1.852081784	-3.610207561	0.488227714	1.402720637	1.14E-05	2.573718148
NM_002866	RAB3A, member RAS oncogene family	RAB3A	-2.204257319	-4.608372446	0.84068646	1.790902082	0.000362467	2.573212959
NM_001280	cold inducible RNA binding protein	CIRBP	-1.560301047	-2.949153771	0.198173077	1.147244649	6.29E-07	2.570640686
NM_173651	fibrous sheath interacting protein 2	FSIP2	-1.667593047	-3.176841355	0.305713935	1.23603015	0.016108886	2.5701973
NM_019590	KIAA1217	KIAA1217	-1.589146486	-3.008712982	0.227365115	1.170694882	0.000936151	2.570023179
NM_020899	zinc finger and BTB domain containing 4	ZBTB4	-1.632036806	-3.099502797	0.272793476	1.208144884	0.000173629	2.565505875
NM_017991	hypothetical protein FLJ10081	FLJ10081	-1.481040869	-2.791500605	0.124829213	1.090378646	2.99E-05	2.560120391
NM_015342	KIAA0073 protein	KIAA0073	-1.573307528	-2.975861799	0.218313917	1.163373153	8.56E-06	2.557959836
XR_011853	importin 13	IPO13	-2.411466792	-5.320149519	1.056537355	2.079933431	5.55E-05	2.557846054
NM_024558	chromosome 14 open reading frame 138	C14orf138	-1.372696652	-2.589541451	0.019827257	1.013838079	0.016013674	2.55419628
NM_000028	amylo-1, 6-glucosidase, 4-alpha-glucanotransferase	AGL	-1.357910988	-2.563137701	0.009114324	1.006337566	0.004384591	2.546995947
NM_147189	hypothetical protein MGC39325	MGC39325	-1.713447485	-3.279435465	0.36578409	1.28858177	0.000974148	2.544996011
NM_014359	opticin	OPTC	-1.61835202	-3.070241245	0.272348068	1.207771946	0.026495645	2.542070343
NM_152465	hypothetical protein MGC39650	MGC39650	-1.795714987	-3.471874952	0.451115984	1.367097354	0.03205608	2.539595986
NM_001684	ATPase, Ca++ transporting, plasma membrane 4	ATP2B4	-1.390890106	-2.622404265	0.049761666	1.035093911	5.11E-05	2.533494049
NM_001631	alkaline phosphatase, intestinal	ALPI	-1.406775365	-2.651438647	0.06851135	1.048634088	0.00053818	2.528468869
NM_173618	hypothetical protein FLJ90652	FLJ90652	-1.522507244	-2.87289894	0.185588799	1.137281031	1.84E-06	2.526111719
NM_024730	hypothetical protein FLJ22655	FLJ22655	-5.253217827	-38.1396005	3.918155693	15.11758403	3.53E-06	2.52286347
NM_182947	RAC/CDC42 exchange factor	GEFT	-1.669870496	-3.181860301	0.33707118	1.263189584	0.001119748	2.518909547
NM_032442	G protein pathway suppressor 2	GPS2	-1.498397932	-2.825287988	0.166820001	1.122581353	0.000119738	2.516777942
NM_018008	zinc finger protein 312	ZNF312	-6.180745423	-72.54204055	4.851214563	28.86430459	0.000225602	2.513209363
CN802687	ILLUMIGEN_MCQ_25727 Katze_MMBR cDNA clone IBIUW:13692 5' Bases 1 to 579 highly human FBXW7	Hs.312503	-1.343205874	-2.537144826	0.016404279	1.01143547	0.007914091	2.508459413
NM_005011	nuclear respiratory factor 1	NRF1	-1.335988112	-2.524483263	0.009507923	1.006612155	0.001438247	2.507900636
NM_182905	CXYorf1-related protein	WASH1	-1.480561343	-2.790572915	0.154981204	1.113407112	0.001634559	2.506336528
NM_001943	desmoglein 2	DSG2	-1.92349001	-3.793396064	0.598420413	1.514057937	0.018671354	2.505449739
NM_001001974	pleckstrin homology domain containing, family A phosphoinositide binding specific	PLEKHA1	-1.426763759	-2.688429723	0.102179744	1.073394011	0.006060132	2.504606598
XR_011047	RP11-506B15.1 protein isoform 1	LOC702661	-1.41393188	-2.664623831	0.090174961	1.06449927	5.54E-05	2.50317112
NM_016458	brain protein 16	LOC51236	-4.813497457	-28.11946921	3.493764292	11.2649132	0.00162868	2.496199368
XR_010623	SET binding protein 1	SETBP1	-1.338108979	-2.528197165	0.018576422	1.01295945	0.009897305	2.495852293
NM_019113	fibroblast growth factor 21	FGF21	-1.875480642	-3.669238397	0.557051134	1.471258891	0.002103152	2.493944757
NM_002498	NIMA never in mitosis gene a	NEK3	-1.623940266	-3.082156815	0.307939577	1.237938442	0.008572027	2.48974966
NM_000030	alanine-glyoxylate aminotransferase	AGXT	-2.280475878	-4.858381828	0.965501138	1.952741713	0.000215291	2.487979744
NM_152573	RAS and EF hand domain containing	RASEF	-1.443783131	-2.720332747	0.135298526	1.098320057	0.000231641	2.476812409
NM_153368	connexin40.1	CX40.1	-2.198650465	-4.590497339	0.893077839	1.857133901	2.45E-07	2.471818179
NM_207423	FLJ45983 protein	FLJ45983	-1.559263495	-2.947033571	0.256018985	1.194178902	0.010388051	2.467832556

NM_025141	BBP-like protein 2	BLP2	-1.403375683	-2.64519794	0.100479789	1.072129956	0.00881259	2.467236295
NM_144664	hypothetical protein MGC33371	MGC33371	-1.492583705	-2.813924666	0.195110543	1.144811876	0.003203217	2.457979975
NM_148916	abhydrolase domain containing 11	ABHD11	-1.714330501	-3.28144329	0.417702418	1.335798518	0.013836883	2.456540599
CN646702	ILLUMIGEN_MCQ_26875 Katze_MMBR cDNA clone IBIUW:8319 5' Bases 42 to 757 highly human FLJ37228	Hs.42834	-2.70393628	-6.515772714	1.408015504	2.653718799	0.000710259	2.455336533
NM_144622	DC-STAMP domain containing 2	DCST2	-1.900416674	-3.733210022	0.605836397	1.521860791	8.88E-05	2.453056182
NM_207481	FLJ34870 protein	FLJ34870	-1.424372107	-2.683976626	0.130878757	1.094960447	0.005436379	2.451208747
NM_021976	retinoid X receptor, beta	RXRB	-1.585424297	-3.000960432	0.294536329	1.226490729	4.07E-05	2.446786071
NM_015642	zinc finger and BTB domain containing 20	ZBTB20	-1.394286198	-2.628584655	0.105266787	1.075693292	1.63E-06	2.443619082
NM_013367	anaphase promoting complex subunit 4	ANAPC4	-1.834240922	-3.565837409	0.546778657	1.460820238	4.83E-07	2.440983029
NM_005185	calmodulin-like 3	CALML3	-1.425742868	-2.686527987	0.138773646	1.100968846	0.010268667	2.440148962
NM_024682	TBC1 domain family, member 17	TBC1D17	-1.693033505	-3.233358566	0.406089006	1.325088765	8.03E-05	2.440107147
NM_032432	actin binding LIM protein family, member 2	ABLIM2	-1.689675473	-3.225841318	0.404760045	1.3238687	0.000679674	2.436677683
NM_053051	LYST-interacting protein LIP8	LIP8	-1.540520456	-2.908994273	0.256383165	1.194480387	5.27E-05	2.435363783
XR_013530	RAR-related orphan receptor C isoform a	LOC717052	-1.359946528	-2.566756659	0.077557689	1.055230148	6.75E-05	2.432414069
CK231263	Hs.296141		-1.367272169	-2.579823147	0.086881768	1.062072144	0.000160056	2.429046992
NM_022105	death associated transcription factor 1	DATF1	-1.530757254	-2.889374593	0.25343527	1.192042167	0.001575255	2.423886229
NM_021079	N-myristoyltransferase 1	NMT1	-1.281059106	-2.430173144	0.004499065	1.003123382	0.00014801	2.422606418
NM_004166	chemokine C-C motif ligand 14	CCL14	-2.033980943	-4.095333505	0.758370201	1.691578585	0.000364537	2.421012859
NM_003628	plakophilin 4	PKP4	-1.514180006	-2.856364328	0.238823988	1.180030368	0.00050945	2.42058544
NM_020800	WD repeat domain 56	WDR56	-1.712163193	-3.2765174	0.438202533	1.354915174	6.50E-05	2.418245409
NM_020991	chorionic somatomammotropin hormone 2	CSH2	-1.431738843	-2.697716683	0.159077679	1.11657308	0.001249492	2.416068174
NM_144616	skeletal muscle sarcoplasmic reticulum protein JP-45	FLJ32416	-1.527189855	-2.882238768	0.255994514	1.194158646	0.027851035	2.41361462
NM_178353	late envelope protein 5	LEP5	-2.176020223	-4.519052205	0.905582424	1.873300603	0.016752595	2.412347595
NM_203370	RIKEN cDNA 6530418L21	LOC389119	-1.612355032	-3.057505369	0.346661924	1.271614986	1.06E-05	2.404426971
NM_080431	actin-related protein M2	ARPM2	-2.806317256	-6.994967021	1.542533859	2.913056855	1.45E-05	2.401246309
NM_015485	RWD domain containing 3	RWDD3	-1.319168045	-2.49522177	0.056094193	1.039647311	0.000399774	2.400065622
NM_006276	splicing factor, arginine/serine-rich 7, 35kDa	SFRS7	-1.311931529	-2.482737149	0.050248828	1.035443496	0.000376369	2.397752421
DR771278	FLJ14525	Hs.520494	-1.540934933	-2.909830127	0.280168817	1.214336972	0.000362399	2.396229543
NM_145645	Williams-Beuren Syndrome critical region protein 20 copy B	WBSCR20B	-1.539558754	-2.907055781	0.278879129	1.213251908	6.09E-05	2.396085892
NM_004689	metastasis associated 1	MTA1	-1.270404607	-2.412292093	0.010601121	1.007375201	1.02E-05	2.394631207
NM_002002	Fc fragment of IgE, low affinity II, receptor for	FCER2	-1.408039028	-2.65376207	0.148555492	1.108459065	0.004742459	2.394100202
NM_003040	solute carrier family 4, anion exchanger, member 2 erythrocyte membrane protein band 3-like 1	SLC4A2	-1.384171199	-2.610219622	0.125450983	1.090848676	3.91E-05	2.392833835
NM_003949	huntingtin-associated protein 1	HAP1	-1.832827873	-3.562346557	0.574150551	1.48880061	0.001287798	2.392762693
NM_013432	nuclear factor of kappa light polypeptide gene enhancer in B-cells inhibitor like 2	NFKBIL2	-1.962100633	-3.896288844	0.706255352	1.631563743	0.004326281	2.388070255
NM_018996	KIAA1582 protein	KIAA1582	-2.279194669	-4.854069178	1.025121577	2.035130879	5.68E-05	2.385138582
NM_145200	calcium binding protein 4	CABP4	-1.484169044	-2.797559944	0.235088818	1.176979195	0.024429734	2.376898382
NM_173659	hypothetical protein MGC29784	MGC29784	-1.367099385	-2.579514193	0.120721691	1.087278625	1.95E-05	2.372450018
NM_004313	arrestin, beta 2	ARRB2	-1.354810577	-2.557635327	0.108485689	1.078096029	1.08E-05	2.372363183
NM_016541	guanine nucleotide binding protein	GNG13	-2.083788749	-4.239190352	0.839702386	1.78968091	4.87E-07	2.368685015
NM_016333	serine/arginine repetitive matrix 2	SRRM2	-1.247556551	-2.37438939	0.004116061	1.00285711	0.000507315	2.367624827
NM_022039	split hand/foot malformation ectrodactyly	SHFM3	-1.536690758	-2.901282458	0.293274429	1.225418408	1.64E-05	2.367585176
CN646916	Down syndrome critical region gene 6	DSCR6	-1.624225911	-3.082767126	0.383081328	1.304124249	0.003103062	2.363859983
NM_001002252	ADP-ribosylation-like factor 6 interacting protein 4	ARL6IP4	-1.622982298	-3.080110903	0.382742999	1.303818453	0.000586426	2.362377137

NM_000894	luteinizing hormone beta polypeptide	LHB	-1.986402609	-3.962477132	0.746545486	1.677770618	1.56E-06	2.361751416
NM_006006	zinc finger and BTB domain containing 16	ZBTB16	-1.361901949	-2.570237986	0.122793491	1.088841146	6.06E-05	2.360526138
NM_017759	hypothetical protein FLJ20309	FLJ20309	-1.663583459	-3.168024426	0.428125527	1.345484272	0.000213609	2.354560727
NM_016423	zinc finger protein 219	ZNF219	-1.43803106	-2.709508287	0.203125668	1.151189763	1.33E-05	2.353659122
NM_003160	aurora kinase C	AURKC	-1.540037192	-2.908020002	0.305761112	1.23607057	0.00094449	2.352632667
NM_001567	inositol polyphosphate phosphatase-like 1	INPPL1	-1.724065214	-3.303659991	0.491255116	1.405667246	7.93E-07	2.350243274
NM_014801	pecanex-like 2	PCNXL2	-1.370261956	-2.585175019	0.138106621	1.100459935	0.014327816	2.349176864
NM_001001995	glycoprotein M6B	GPM6B	-1.308853912	-2.477446516	0.076811663	1.054684624	0.036426552	2.348992731
NM_004428	ephrin-A1	EFNA1	-1.46943519	-2.769134617	0.24210331	1.18271569	0.001013281	2.341335825
NM_014580	solute carrier family 2, facilitated glucose transporter	SLC2A8	-1.635600536	-3.107168621	0.408364368	1.327180292	0.000161458	2.341180501
NM_014515	CCR4-NOT transcription complex, subunit 2	CNOT2	-1.496260482	-2.821105233	0.269754429	1.205602596	0.000731482	2.339995983
NM_015245	ankyrin repeat and sterile alpha motif domain containing 1	ANKS1	-1.667474864	-3.176581126	0.441892307	1.358384882	0.00244506	2.338498587
BC043391	TAF7-like RNA polymerase II, TATA box binding protein	TBP	-4.847746995	-28.79501153	3.624609085	12.33434402	8.48E-06	2.334539356
NM_183240	voltage-dependent calcium channel gamma subunit-like protein	PR1	-1.561201006	-2.950994039	0.34040691	1.266113649	0.002886547	2.330749725
NM_018182	hypothetical protein FLJ10700	FLJ10700	-1.2720026	-2.414965535	0.051988431	1.036692788	0.014398434	2.329490051
NM_006784	WD repeat domain 3	WDR3	-1.288901907	-2.443420062	0.069067908	1.049038705	9.33E-05	2.329199152
NM_001190	branched chain aminotransferase 2, mitochondrial	BCAT2	-1.341819943	-2.534708677	0.123643376	1.089482766	0.000181235	2.326524802
NM_018261	SEC3-like 1	SEC3L1	-1.388830577	-2.618663305	0.171429731	1.126173986	0.000582087	2.325274191
NM_173197	Kv channel interacting protein 2	KCNIP2	-1.676042366	-3.195501504	0.45894567	1.374536932	0.000759934	2.324784027
NM_001011667	coiled-coil-helix-coiled-coil-helix domain containing 7	CHCHD7	-1.271638609	-2.414356317	0.055275487	1.039057495	0.013235274	2.323602234
NM_005515	homeo box HB9	HLXB9	-1.572837715	-2.974892868	0.357871866	1.281534099	0.000329471	2.321352878
NM_018093	WD repeat domain 74	WDR74	-1.216241712	-2.323406699	0.002220228	1.001540129	0.00179495	2.319833855
NM_005858	A kinase	AKAP8	-1.302361519	-2.466322599	0.089545165	1.064034674	0.000441226	2.317896831
NM_025211	G kinase anchoring protein 1	GKAP1	-1.286860059	-2.439964333	0.074683192	1.053129749	0.009157062	2.316869631
NM_138762	BCL2-associated X protein	BAX	-1.548556608	-2.925243271	0.337448081	1.263519633	0.000467614	2.315154585
NM_012231	PR domain containing 2, with ZNF domain	PRDM2	-1.38800917	-2.617172779	0.177632343	1.131026195	1.59E-05	2.313980693
NM_145288	zinc finger protein 342	ZNF342	-1.736317763	-3.331836862	0.527735898	1.441664934	0.000396128	2.311103492
NM_173640	roof plate-specific spondin	RSPONDIN	-1.237945735	-2.358624466	0.031109189	1.021797414	0.008103161	2.308309294
NM_000151	glucose-6-phosphatase, catalytic	G6PC	-1.35040014	-2.549828367	0.145106812	1.10581252	0.009828646	2.305841469
NM_003717	neuropeptide FF-amide peptide precursor	NPFF	-1.326088451	-2.507219753	0.122007275	1.088247929	0.001286113	2.303904915
NM_032269	coiled-coil domain containing 135	CCDC135	-1.733291126	-3.324854312	0.52976567	1.443694684	0.000986213	2.303017631
NM_001091	amiloride binding protein 1 amine oxidase copper-containing	ABP1	-1.336479875	-2.525343914	0.137935819	1.100329658	0.034931594	2.295079384
NM_001008568	tRNA 5-methylaminomethyl-2-thiouridylate	TRMT1	-1.341804222	-2.534681058	0.143627118	1.104678931	0.000340539	2.294495701
NM_033036	galactose-3-O-sulfotransferase 3	GAL3ST3	-1.792758211	-3.464766697	0.595454162	1.510948159	3.78E-05	2.293107595
NM_006271	S100 calcium binding protein A1	S100A1	-1.705119446	-3.260559273	0.511523609	1.425554908	0.000180131	2.287221105
NM_007180	trehalase	TREH	-1.256526249	-2.389197718	0.06312742	1.04472803	0.000873876	2.286908792
NM_152520	zinc finger protein 533	ZNF533	-1.471431297	-2.772968632	0.28076976	1.214842899	0.000469746	2.282573848
NM_021224	zinc finger protein 462	ZNF462	-1.631539679	-3.098434949	0.443948849	1.360322621	0.000863941	2.277720668
NM_176875	cholecystokinin B receptor	CCKBR	-2.042610581	-4.1199036	0.855605025	1.809517456	2.65E-06	2.276796826
NM_144645	hypothetical protein MGC26744	MGC26744	-2.339741632	-5.062119733	1.152925621	2.22364367	1.45E-05	2.276497715
XR_010245	hypothetical protein LOC697032	LOC697032	-1.398852822	-2.636918208	0.212110704	1.158381691	0.000404876	2.276381118
NM_002739	protein kinase C, gamma	PRKCG	-1.908236234	-3.753499351	0.722903138	1.6505	0.000332244	2.274158953
NM_022036	G protein-coupled receptor, family C, group 5, member C	GPRC5C	-1.50904271	-2.846211181	0.324188363	1.251959908	4.27E-05	2.273404414
NM_052844	WD repeat domain 34	WDR34	-1.318638635	-2.494306294	0.134849036	1.097977915	7.68E-06	2.2717272
NM_152312	glycosyltransferase-like 1B	GYLTL1B	-1.272611148	-2.415984414	0.089279126	1.06383848	0.001777564	2.271006793

NM_207477	FLJ27365 protein	FLJ27365	-1.473368437	-2.776694463	0.292773988	1.22499341	0.002943557	2.266701552
NM_144994	ankyrin repeat domain 23	ANKRD23	-1.229676041	-2.345143234	0.049166618	1.034667069	0.000139267	2.266567966
NM_152449	hypothetical protein FLJ33008	FLJ33008	-1.358043034	-2.563372309	0.177839337	1.131188483	0.00141759	2.266087701
NM_130900	retinoic acid early transcript 1L	RAET1L	-1.394529788	-2.629028512	0.214334597	1.160168696	0.001729031	2.26607434
NM_000309	protoporphyrinogen oxidase	PPOX	-1.380830547	-2.604182485	0.201176468	1.149635461	3.37E-05	2.265224564
NM_018010	estrogen-related receptor beta like 1	ESRRBL1	-1.725549272	-3.307060119	0.547896569	1.461952633	0.000655801	2.262084314
NM_175569	Xg blood group	XG	-1.544157257	-2.916336627	0.366928073	1.289603955	0.004548991	2.26142035
NM_015881	dickkopf homolog 3	DKK3	-1.640952503	-3.118716692	0.463935112	1.379298884	1.61E-05	2.26108839
NM_001920	decorin	DCN	-1.443499621	-2.719798216	0.266720938	1.203070292	2.84E-05	2.260714303
NM_004218	RAB11B, member RAS oncogene family	RAB11B	-1.95242348	-3.870241199	0.775721712	1.712046293	0.000601418	2.260593778
NM_001005492	olfactory receptor, family 5, subfamily J, member 2	OR5J2	-1.309526985	-2.47860261	0.136614602	1.09932244	0.003631612	2.254663892
NM_000352	ATP-binding cassette, sub-family C	ABCC8	-1.933393693	-3.819526213	0.760950081	1.694606233	3.28E-05	2.253931408
NM_016337	Enah/Vasp-like	EVL	-1.218206159	-2.326572524	0.046463497	1.032730269	6.72E-06	2.252836576
NM_020166	methylcrotonoyl-Coenzyme A carboxylase 1	MCCC1	-1.228694091	-2.343547588	0.060015762	1.04247715	0.004712324	2.248056553
NM_022447	PAP associated domain containing 5	PAPD5	-1.180846812	-2.267098088	0.012321041	1.008576867	0.000125513	2.247818844
BM423312	PLATE4_H11 Rhesus cDNA sequence		-2.139930689	-4.407408714	0.97163617	1.96106339	1.23E-05	2.247458566
NM_080731	HOM-TES-103 tumor antigen-like	HOM-TES-10:	-1.187189054	-2.277086433	0.022474626	1.015700197	0.003647998	2.241888345
NM_001012506	hypothetical protein LOC285331	LOC285331	-1.163011232	-2.23924321	0.000665901	1.000461674	0.005396974	2.238209886
NM_022096	ankyrin repeat domain 5	ANKRD5	-6.44867337	-87.34622029	5.287483895	39.05631402	1.74E-07	2.236417401
NM_198227	regulator of G-protein signalling 12	RGS12	-1.300483036	-2.463113376	0.139351463	1.101409886	0.028764262	2.236327645
XR_010160	myomesin 1	LOC695301	-1.603567412	-3.038938358	0.443487375	1.359887565	0.000154476	2.234698247
NM_139285	growth arrest-specific 2 like 2	GAS2L2	-1.179724222	-2.2653347	0.022215035	1.015517453	0.005120334	2.230719613
XR_011510	anaphase-promoting complex subunit 5	LOC699821	-1.178473674	-2.263371928	0.021618526	1.015097656	0.000114664	2.229708556
NM_173607	chromosome 14 open reading frame 24	C14orf24	-1.181778412	-2.268562508	0.025851034	1.018080072	0.013112715	2.228275133
NM_145652	WAP four-disulfide core domain 5	WFDC5	-1.694467894	-3.236574907	0.54194142	1.455930431	0.011984029	2.223028545
XR_012186	P3ECSL	LOC705660	-1.81393032	-3.515988423	0.661517009	1.581744971	0.000359957	2.222854181
NM_181489	zinc finger protein 445	ZNF445	-1.683498138	-3.212058432	0.532399206	1.446332452	0.000857551	2.220829954
NM_144621	zinc finger and BTB domain containing 8	ZBTB8	-1.285676201	-2.43796295	0.135198741	1.098244094	0.002801683	2.21987349
XM_035527	hypothetical protein FLJ10980	FLJ10980	-1.168956945	-2.248490743	0.018505004	1.012909306	0.000363227	2.219834224
CO725485	ILLUMIGEN_MCQ_35171 Katze_MMPL cDNA clone IBIUW:26108 5' Bases 1 to 543 highly human PPP1R14A	Hs.348037	-1.294536736	-2.45298215	0.144193548	1.105112734	0.002517955	2.219666895
NM_014972	KIAA1049 protein	KIAA1049	-1.342516381	-2.535932563	0.192634303	1.142848607	1.01E-05	2.218957566
NM_145733	septin 3	3-Sep	-1.631925453	-3.099263574	0.488309761	1.402800414	0.001663387	2.209340362
NM_080658	aspartoacylase	ACY3	-1.448364533	-2.728985129	0.305566992	1.235904263	0.000104668	2.208087803
NM_024841	hypothetical protein FLJ14213	FLJ14213	-1.435221998	-2.704237755	0.294540695	1.226494441	5.49E-05	2.204851213
NM_007059	kaptin actin binding protein	KPTN	-1.666887895	-3.175288978	0.526803432	1.440733435	3.58E-05	2.203939259
NM_017791	chromosome 14 open reading frame 58	C14orf58	-1.24732273	-2.374004599	0.109277985	1.078688258	0.001162646	2.200825476
NM_001928	D component of complement	DF	-1.176327379	-2.260007216	0.038584898	1.027105871	0.000544535	2.200364422
NM_020214	hypothetical protein from EUROIMAGE 1977056	LOC56965	-1.330561022	-2.515004571	0.19287786	1.14304156	2.66E-06	2.200273952
NM_203393	hypothetical gene supported by BC031661	LOC389458	-1.9846643	-3.957705601	0.848032804	1.800044788	0.001002974	2.198670626
NM_000106	cytochrome P450, family 2, subfamily D, polypeptide 6	CYP2D6	-1.314124586	-2.486514054	0.17816665	1.131445152	0.000974905	2.197644358
NM_134423	RAD52 homolog	RAD52	-1.250845415	-2.379808382	0.118117535	1.085317788	0.000437165	2.192729546
CN806534	chemokine (C-X-C motif) receptor 7	CXCR7	-1.156178063	-2.228662355	0.023930148	1.016725444	0.008371677	2.192000178
NM_014057	osteoglycin osteoinductive factor, mimecan	OGN	-1.217182442	-2.324922205	0.086084921	1.061485689	0.001586997	2.190252991
NM_002209	integrin, alpha L antigen CD11A	ITGAL	-1.558562401	-2.945601774	0.429239804	1.346523869	0.000326257	2.187560014
NM_201997	splicing factor 1	SF1	-1.335369184	-2.52340047	0.207166072	1.154418298	0.000326597	2.185863196

NM_014376	cytoplasmic FMR1 interacting protein 2	CYFIP2	-1.310623385	-2.480486981	0.184331016	1.136289948	1.36E-05	2.182970099
NM_022640	chorionic somatomammotropin hormone 1 placental lactogen	CSH1	-1.346433066	-2.542826565	0.222645204	1.166871103	0.00556128	2.179183767
NM_001847	collagen, type IV, alpha 6	COL4A6	-1.202208904	-2.300916939	0.078537905	1.055947351	0.001645842	2.179007255
NM_024561	NMDA receptor regulated 1-like	NARG1L	-1.280457543	-2.429160041	0.157398213	1.115274014	0.001741698	2.178083601
NM_033624	F-box protein 21	FBXO21	-1.168097765	-2.24715208	0.045360955	1.031941334	0.004972421	2.177596736
NM_183353	ring finger protein 12	RNF12	-1.454335377	-2.740302913	0.334545584	1.260980166	0.002079571	2.173153065
NM_203402	CG10671-like	LOC161247	-1.725410306	-3.306741585	0.609890627	1.526143505	6.85E-05	2.166730438
NM_021958	H2.0-like homeo box 1	HLX1	-1.276728329	-2.422889029	0.161215559	1.118228917	0.000229363	2.166720062
NM_032488	cornifelin	CNFN	-1.245712082	-2.371355702	0.134361911	1.097607246	0.000839936	2.160477447
NM_001798	cyclin-dependent kinase 2	CDK2	-1.138203514	-2.201067691	0.030631577	1.021459198	0.004869512	2.154826836
XM_496244	potassium channel tetramerisation domain containing 2	KCTD2	-1.34338875	-2.537466455	0.236758045	1.178341771	0.000244683	2.153421459
NM_138501	glycoprotein, synaptic 2	GPSN2	-1.271035888	-2.413347873	0.166117494	1.122034856	0.000562687	2.150867114
NM_175068	keratin 6 irs3	K6IRS3	-1.286355765	-2.439111593	0.182532796	1.134874524	0.001105286	2.149234601
NM_207414	FLJ43860 protein	FLJ43860	-1.547731405	-2.923570547	0.445820459	1.362088515	0.001688438	2.146388076
NM_005608	protein tyrosine phosphatase, receptor type, C-associated protein	PTPRCAP	-1.671545886	-3.185557516	0.570703675	1.485247824	0.00089265	2.144798641
NM_004227	pleckstrin homology, Sec7 and coiled-coil domains 3	PSCD3	-1.381725023	-2.605797587	0.281902609	1.215797207	0.000142721	2.143283084
NM_025215	pseudouridylate synthase 1	PUS1	-1.281361854	-2.430683167	0.184268516	1.136240723	0.000457859	2.139232573
NM_014328	RUN and SH3 domain containing 1	RUSC1	-1.291542352	-2.447896146	0.195511603	1.145130171	1.79E-05	2.137657542
NM_024897	progestin and adipoQ receptor family member VI	PAQR6	-1.126142656	-2.182743576	0.031718647	1.022229157	0.040170333	2.135278143
NM_016316	REV1-like	REV1L	-1.334758555	-2.522332653	0.243012549	1.183461316	0.000723005	2.131318209
NM_003597	TGFB inducible early growth response 2	TIEG2	-1.217038152	-2.32468969	0.125671529	1.091015448	0.001072304	2.130757813
NM_004296	regulator of G-protein signalling 6	RGS6	-1.633453038	-3.102546941	0.543762362	1.457769238	0.003216775	2.128283997
XR_013420	Keratin, type I cytoskeletal 16	LOC707236	-1.263977906	-2.401570076	0.174587255	1.128641462	0.00199432	2.127841441
NM_006545	tumor suppressor candidate 4	TUSC4	-1.093692565	-2.134195835	0.004596421	1.003191077	1.10E-05	2.127407115
NM_153635	copine family member	LOC151835	-1.266630444	-2.405989662	0.177590577	1.130993452	0.002901386	2.12732413
NM_014333	immunoglobulin superfamily, member 4	IGSF4	-1.190421764	-2.282194521	0.104778186	1.075329046	0.000396735	2.122322026
NM_017806	Lck interacting transmembrane adaptor 1	LIME1	-1.440610199	-2.71435647	0.356786259	1.280570126	0.001161416	2.119646878
NM_014634	protein phosphatase 1F PP2C domain containing	PPM1F	-1.085719693	-2.122434002	0.003404297	1.002362465	0.00624687	2.117431643
NM_033130	sialic acid binding Ig-like lectin 10	SIGLEC10	-1.084914989	-2.121250485	0.003823598	1.002653831	0.001915202	2.115635944
NM_030645	KIAA1720 protein	KIAA1720	-1.128824195	-2.186804418	0.04823153	1.033996663	0.001871793	2.114904715
NM_001001732	chromosome 10 open reading frame 130	C10orf130	-1.406063422	-2.650130533	0.326051965	1.253578172	0.00067566	2.11405287
NM_003315	DnaJ Hsp40	DNAJC7	-1.528718396	-2.885294129	0.449863473	1.36591099	0.000123112	2.112358822
NM_005089	U2 RNU2	U2AF1L2	-1.177850385	-2.262394291	0.099354619	1.071294119	3.24E-05	2.111833018
NM_002824	parathymosin	PTMS	-1.157334311	-2.230449232	0.081076256	1.057806874	0.004145579	2.108559971
NM_017822	hypothetical protein FLJ20436	FLJ20436	-1.354057891	-2.556301302	0.278006252	1.212518074	3.03E-05	2.108258308
NM_001680	FXYD domain containing ion transport regulator 2	FXYD2	-1.502374751	-2.833086696	0.426913559	1.344354443	0.000302148	2.107395643
NM_182765	HECT domain containing 2	HECTD2	-1.188406742	-2.27900919	0.113770068	1.082052177	0.002024183	2.106191586
NM_020321	amiloride-sensitive cation channel 3	ACCN3	-1.68081869	-3.206098368	0.606307203	1.522357513	0.000174301	2.106008833
CN643806	ubiquitin specific peptidase 32	USP32	-1.490591327	-2.810041286	0.416555591	1.334737086	0.003037282	2.105314459
NM_213653	hemochromatosis type 2	HFE2	-5.002520397	-32.05595306	3.928943569	15.23105077	2.10E-05	2.104644883
NM_024527	abhydrolase domain containing 8	ABHD8	-1.768511348	-3.4070222	0.696650844	1.620737947	2.42E-06	2.102142549
XM_375485	helicase with zinc finger domain	HELZ	-1.262668578	-2.399391503	0.190970308	1.141531213	0.000699636	2.101906173
NM_004984	kinesin family member 5A	KIF5A	-2.50108507	-5.661110445	1.431981328	2.698170147	0.003982426	2.098129524
NM_021817	hyaluronan and proteoglycan link protein 2	HAPLN2	-1.314523916	-2.487202404	0.245811745	1.185759761	0.009198931	2.097560134

XR_014513	regulator of G-protein signalling 9	RGS9	-1.119869846	-2.173273652	0.052571692	1.037111992	0.002736159	2.095505276
NM_018025	G patch domain containing 1	GPATC1	-1.220912349	-2.330940775	0.154584723	1.113101168	0.00027174	2.094096065
NM_183415	ubiquitin protein ligase E3B	UBE3B	-1.1626128	-2.238624879	0.097503297	1.069920274	0.00054437	2.092328685
NM_019092	hypothetical protein KIAA1164	KIAA1164	-1.501921814	-2.832197383	0.437034491	1.353818645	7.43E-05	2.092006484
NM_001586	chromosome X open reading frame 2	CXorf2	-1.363383069	-2.572878034	0.298852323	1.230165417	0.008008357	2.091489486
NM_032874	KIAA1984	KIAA1984	-1.465750182	-2.762070573	0.403914275	1.32309282	0.000259073	2.087586398
CN801832	prefoldin subunit 5	PFDN5	-1.062512072	-2.088565042	0.004648462	1.003227265	1.08E-05	2.081846372
NM_002991	chemokine C-C motif ligand 24	CCL24	-1.382749568	-2.60764878	0.325083427	1.252736878	0.006723106	2.081561439
NM_206891	chromosome 21 open reading frame 106	C21orf106	-1.332566525	-2.518503131	0.276053513	1.210877997	0.012783113	2.079898336
NM_018416	forkhead box J2	FOXJ2	-1.191449026	-2.283820122	0.136287573	1.099073274	0.001107817	2.077950739
NM_173527	hypothetical protein FLJ38964	FLJ38964	-1.237592762	-2.358047471	0.183087664	1.135311086	0.00688434	2.077005589
XR_014263	G protein-coupled receptor 123	LOC719701	-2.354808911	-5.11526475	1.30039516	2.46296335	6.74E-05	2.076874084
NM_015417	chromosome 20 open reading frame 28	C20orf28	-1.709675819	-3.27087317	0.655823821	1.575515361	0.000171855	2.076065553
U06694	surfactant protein C, SP-C	SFTPC	-1.383683319	-2.609337066	0.330029121	1.257038747	0.001100412	2.075780935
NM_004783	thousand and one amino acid protein kinase	TAO1	-1.149505385	-2.218378263	0.100317197	1.072009133	4.73E-05	2.069365078
NM_001004067	NODAL modulator 3	NOMO3	-2.117946794	-4.340757398	1.069634135	2.098901022	0.000287089	2.068109622
NM_005474	histone deacetylase 5	HDAC5	-1.173170976	-2.25506806	0.126091748	1.091333278	0.000154105	2.066342248
NM_013290	TBP-1 interacting protein	TBPIP	-1.199521841	-2.296635399	0.152525668	1.111513652	0.004109905	2.066223294
NM_014583	LIM and cysteine-rich domains 1	LMCD1	-1.118906469	-2.171822907	0.074312247	1.052859003	1.39E-05	2.06278609
NM_175605	tetratricopeptide repeat domain 10	TTC10	-1.095687132	-2.137148463	0.051737564	1.036512535	0.000721304	2.06186456
CO644967	ILLUMIGEN_MCQ_43063 Katze_MMPB2 cDNA clone IBIUW:23331 5' Bases 5 to 740 highly human RAB34	Hs.301853	-1.230967323	-2.34724319	0.193948408	1.143890066	0.000105923	2.051983193
NM_014315	kelch domain containing 2	KLHDC2	-1.198255419	-2.294620259	0.162156039	1.118958119	0.001258758	2.050675732
NM_000906	natriuretic peptide receptor A/guanylate cyclase A	NPR1	-1.173558635	-2.255674089	0.139793374	1.10174731	0.00010685	2.04736065
XM_376148	RIKEN cDNA 5830415L20	LOC401015	-1.084951615	-2.121304337	0.05228283	1.036904359	0.002608993	2.045805208
NM_005091	peptidoglycan recognition protein 1	PGLYRP1	-1.752792913	-3.370103532	0.72188761	1.649338604	0.000423176	2.04330604
NM_000903	NAD	NQO1	-1.278311109	-2.425548638	0.248798623	1.188217238	0.001867549	2.041334329
NM_024800	NIMA never in mitosis gene a	NEK11	-1.221081332	-2.331213815	0.192761542	1.142949406	0.003872664	2.039647427
NM_001976	enolase 3,	ENO3	-1.15533059	-2.22735357	0.129652623	1.094030245	0.00170529	2.035915898
NM_015981	calcium/calmodulin-dependent protein kinase	CAMK2A	-1.657808497	-3.155368493	0.636930475	1.555017132	0.000737179	2.029153525
NM_032255	zinc finger protein 541	ZNF541	-1.077031717	-2.109691014	0.056220278	1.039738175	0.035783466	2.029059877
NM_002660	phospholipase C, gamma 1	PLCG1	-1.139448863	-2.202968495	0.11940916	1.086289894	0.000102778	2.027974768
XR_012042	hypothetical protein LOC709438	LOC709438	-1.856157198	-3.620420317	0.839455372	1.789374512	0.001055683	2.02328819
NM_178477	chromosome 20 open reading frame 179	C20orf179	-1.173126124	-2.254997954	0.158161505	1.115864232	0.000237289	2.020853333
NM_021569	glutamate receptor, ionotropic, N-methyl D-aspartate 1	GRIN1	-1.828345513	-3.551295752	0.813643967	1.75764531	3.24E-06	2.020484867
NM_003220	transcription factor AP-2 alpha activating enhancer binding protein 2 alpha	TFAP2A	-1.806651105	-3.498292946	0.795745667	1.735974393	0.000790631	2.015175432
NM_013366	anaphase promoting complex subunit 2	ANAPC2	-1.229351713	-2.344616088	0.220654603	1.165262188	0.00104679	2.012093168
XR_014848	hypothetical protein LOC723278	LOC723278	-1.13128969	-2.190544754	0.123048159	1.089033368	2.22E-05	2.011457883
NM_016340	Rap guanine nucleotide exchange factor	RAPGEF6	-1.5426799	-2.913351752	0.53658973	1.450539648	2.21E-06	2.008460614
NM_152891	protease, serine, 33	PRSS33	-1.912796168	-3.765381826	0.90851636	1.877114118	5.78E-05	2.005941882
NM_023914	purinergic receptor P2Y, G-protein coupled, 13	P2RY13	-1.421498518	-2.678635947	0.417632295	1.335733591	0.016585753	2.005366912
NM_017490	MAP/microtubule affinity-regulating kinase 2	MARK2	-1.219297022	-2.328332376	0.2154415	1.161059173	1.04E-05	2.005352036
NM_017633	chromosome 6 open reading frame 37	C6orf37	-1.102852484	-2.147789319	0.099762103	1.071596744	0.002520271	2.004288769
NM_007029	stathmin-like 2	STMN2	-1.776214161	-3.425261564	0.773925899	1.709916528	8.31E-05	2.003174721
NM_002281	keratin, hair, basic, 1	KRTHB1	-1.330695501	-2.515239014	0.328809231	1.255976291	0.000116097	2.002616636

NM_021954	gap junction protein, alpha 3, 46kDa	GJA3	-1.422147662	-2.679841477	0.421843227	1.33963802	0.013812618	2.000422081
NM_003223	transcription factor AP-4 activating enhancer binding protein 4	TFAP4	-1.498179248	-2.824859762	0.501034828	1.415228325	0.005869738	1.996045241
NM_016589	chromosome 3 open reading frame 1	C3orf1	-1.230683915	-2.346782136	0.235396565	1.177230287	0.015607071	1.99347754
XR_011842	CDK5 regulatory subunit associated protein 2 isoform a	LOC701442	-1.014202076	-2.019785483	0.024303277	1.016988438	0.013950034	1.98604567
XR_014033	MYST histone acetyltransferase 1	MYST1	-1.154659664	-2.22631798	0.165237382	1.121350571	0.001022737	1.985389795
NM_003955	suppressor of cytokine signaling 3	SOCS3	-2.433033219	-5.400276284	1.448397909	2.729048264	8.52E-05	1.978813037
NM_152689	hypothetical protein MGC9712	MGC9712	-1.621738098	-3.077455719	0.637275209	1.55538875	0.003249712	1.978576558
NM_144674	hypothetical protein FLJ32871	FLJ32871	-1.398776393	-2.636778516	0.415274507	1.333552393	0.002703565	1.977259034
NM_000515	growth hormone 1	GH1	-1.715259624	-3.283557283	0.734457717	1.66377197	0.004526812	1.973562089
NM_001005367	tweety homolog 1	TTYH1	-2.123932253	-4.35880376	1.144107083	2.210093006	0.000929345	1.972226394
NM_198541	insulin growth factor-like family member 1	IGFL1	-1.464032634	-2.758784246	0.485470352	1.400042238	0.000211739	1.970500726
NM_018502	hypothetical protein PRO1580	PRO1580	-1.018740547	-2.026149386	0.04109737	1.028896147	5.90E-05	1.96924577
NM_133180	EPS8-like 1	EPS8L1	-1.141991179	-2.206853987	0.165244574	1.121356161	0.002858672	1.968022351
NM_002088	glutamate receptor, ionotropic, kainate 5	GRIK5	-1.349898979	-2.548942765	0.373294138	1.29530706	0.004374987	1.967828976
NM_001012505	forkhead box P1	FOXP1	-1.17589907	-2.259336362	0.202077753	1.150353889	0.006701244	1.964035923
NM_022819	phospholipase A2, group IIF	PLA2G2F	-1.479911494	-2.789316209	0.508153948	1.422229166	0.000713997	1.961228383
NM_145729	mitochondrial ribosomal protein L24	MRPL24	-1.361937354	-2.570301063	0.392443816	1.312614992	3.36E-05	1.95815306
NM_006093	proteoglycan 3	PRG3	-1.957232991	-3.883164947	0.987785997	1.983139269	3.92E-05	1.958089887
NM_021734	solute carrier family 25 mitochondrial deoxynucleotide carrier	SLC25A19	-1.413889334	-2.664545251	0.446199746	1.362446657	9.81E-05	1.95570611
NM_004959	nuclear receptor subfamily 5, group A, member 1	NR5A1	-1.515604582	-2.859186212	0.548710413	1.462777573	0.000962739	1.954628144
NM_016609	solute carrier family 22 organic cation transporter	SLC22A17	-1.068057059	-2.096607871	0.101454721	1.072854715	0.000487584	1.954232798
NM_001777	CD47 antigen	CD47	-1.175891481	-2.259324477	0.210523494	1.157107974	0.001734773	1.952561496
NM_012229	5'-nucleotidase, cytosolic II	NT5C2	-1.036833319	-2.051719233	0.071471932	1.05078822	0.000519764	1.952552564
NM_001003	ribosomal protein, large, P1	RPLP1	-1.076443466	-2.108830975	0.112559884	1.081144894	0.001960461	1.950553517
NM_025129	hypothetical protein FLJ22688	FLJ22688	-1.132005684	-2.191632167	0.169800026	1.12490255	0.00750571	1.948286247
NM_194281	chromosome 18 open reading frame 37	C18orf37	-1.209416962	-2.31244165	0.252618327	1.19136735	0.000718417	1.940998005
NM_013302	eukaryotic elongation factor-2 kinase	EEF2K	-1.413085744	-2.663061497	0.463515846	1.3788981	0.003456073	1.931296807
NM_002602	phosphodiesterase 6G, cGMP-specific, rod, gamma	PDE6G	-1.742184531	-3.345413475	0.792905409	1.732560113	0.001013033	1.930907591
NM_001010938	tyrosine kinase, non-receptor, 2	TNK2	-1.018541242	-2.025869498	0.070011732	1.04972522	0.00105756	1.929904568
NM_017711	glycerophosphodiester phosphodiesterase domain containing 2	GDPD2	-1.167484109	-2.246196449	0.223042566	1.167192539	0.001136064	1.924443804
NM_032620	GTP binding protein 3	GTPBP3	-1.085444998	-2.122029921	0.144216135	1.105130036	0.002990414	1.920163104
NM_005510	dom-3 homolog Z	DOM3Z	-1.547607341	-2.923319147	0.607693132	1.523820672	0.000758204	1.918414156
NM_138567	synaptotagmin VIII	SYT8	-1.342166441	-2.535317523	0.402867436	1.322133114	0.003239772	1.917596267
NM_005660	solute carrier family 35 UDP-galactose transporter	SLC35A2	-1.315634529	-2.489117835	0.377150676	1.298774241	0.000442527	1.916513091
NM_016111	KIAA0683 gene product	KIAA0683	-1.049994673	-2.070522202	0.112126033	1.080819818	0.000294125	1.915696001
NM_014716	centaurin, beta 1	CENTB1	-1.508127969	-2.844407113	0.571209366	1.485768522	1.25E-06	1.914434901
NM_001006636	glycosyltransferase-like domain containing 1	GTDC1	-1.016659002	-2.023228131	0.08304558	1.0592518	0.002601239	1.910053995
NM_001263	CDP-diacylglycerol synthase	CDS1	-1.419397906	-2.674738602	0.486039074	1.400594254	0.000821251	1.909716961
NM_003936	cyclin-dependent kinase 5, regulatory subunit 2	CDK5R2	-1.416580105	-2.669519533	0.483354728	1.397990666	0.001304456	1.909540312
NM_177401	midnolin	MIDN	-1.366603655	-2.578627989	0.435946007	1.352797601	0.00117738	1.906144708
NM_005332	hemoglobin, zeta	HBZ	-1.208058833	-2.31026578	0.282073562	1.215941282	0.000941531	1.899981368
NM_018697	LanC lantibiotic synthetase component C-like 2 bacterial	LANCL2	-1.461131495	-2.753242138	0.535512687	1.449457153	0.000378443	1.899498811
NM_001520	general transcription factor IIIC, polypeptide 1, alpha 220kDa	GTF3C1	-1.068187199	-2.096797007	0.145096595	1.10580469	0.00238833	1.89617301
XR_012522	spectrin repeat containing, nuclear envelope 2 isoform e	LOC706759	-1.363254727	-2.572649162	0.440956687	1.357504224	5.50E-05	1.895131607

NM_003954	mitogen-activated protein kinase kinase kinase 14	MAP3K14	-1.513729363	-2.855472247	0.593081773	1.508465576	0.000432041	1.892964807
NM_000737	chorionic gonadotropin, beta polypeptide	CGB	-1.147102583	-2.214686639	0.230187113	1.172987072	0.004521399	1.88807421
NM_032440	ligand-dependent corepressor	MLR2	-1.243795746	-2.368207914	0.326901312	1.254316399	0.000439114	1.888046681
NM_001291	CDC-like kinase 2	CLK2	-1.098949565	-2.141986763	0.186332217	1.13786722	0.00013923	1.882457571
NM_033105	DnaJ Hsp40	DNAJC5B	-1.583756332	-2.997492891	0.671160895	1.592353773	0.007264438	1.882428982
NM_207442	FLJ39779 protein	FLJ39779	-1.310568879	-2.480393267	0.398498124	1.31813499	0.000431667	1.8817445
NM_145275	kinesin light chain 2-like	KLC2L	-1.75205491	-3.368380016	0.844445291	1.795574224	6.98E-05	1.875934713
NM_001402	eukaryotic translation elongation factor 1 alpha 1	EEF1A1	-1.154559313	-2.226163127	0.247998585	1.187558501	0.003313889	1.874571337
NM_005777	RNA binding motif protein 6	RBM6	-1.24770601	-2.374635384	0.341823787	1.267357716	3.56E-06	1.873689925
NM_020690	MASK-4E-BP3 alternate reading frame gene	MASK-BP3	-1.087254164	-2.124692654	0.18183086	1.134322491	2.48E-05	1.873094002
XR_013836	smoothelin isoform a	LOC716424	-1.415763314	-2.668008595	0.512744926	1.426762226	0.002600652	1.86997423
NM_145270	hypothetical protein FLJ13841	LOC146325	-2.171413616	-4.504645627	1.269553249	2.41086898	1.93E-05	1.868473842
NM_020856	zinc finger protein 537	ZNF537	-1.143180107	-2.208673409	0.241540533	1.182254418	0.024820248	1.86818791
NM_014427	copine VII	CPNE7	-1.181802748	-2.268600775	0.281433172	1.215401664	0.00023853	1.866544075
NM_020145	SH3-domain GRB2-like endophilin B2	SH3GLB2	-1.089579008	-2.12811927	0.190288713	1.140992029	0.000270402	1.865148236
NM_025204	hypothetical protein PP2447	PP2447	-1.084434661	-2.120544356	0.186648113	1.138116398	0.000637875	1.863205169
NM_001783	CD79A antigen immunoglobulin-associated alpha	CD79A	-1.14213814	-2.2070788	0.246413354	1.186254331	0.004349425	1.860544357
NM_020897	hyperpolarization activated cyclic nucleotide-gated potassium channel 3	HCN3	-1.874967835	-3.667934395	0.982322114	1.97564278	0.000411518	1.856577734
NM_153634	copine VIII	CPNE8	-1.042740625	-2.060137492	0.150227592	1.109744525	0.01592196	1.856406988
NM_021045	zinc finger protein 248	ZNF248	-1.033270074	-2.04665804	0.140870791	1.102570411	0.000633524	1.856260624
NM_017570	5-oxoprolinase ATP-hydrolysing	OPLAH	-1.370845273	-2.586220481	0.480535736	1.395261692	0.00101506	1.853573775
NM_014343	claudin 15	CLDN15	-1.085152463	-2.12159968	0.196466549	1.145888404	0.000596635	1.851488917
XR_012239	ubiquitin-like 7 bone marrow stromal cell-derived	UBL7	-1.499576103	-2.82759619	0.611349022	1.527687036	2.71E-05	1.850900166
NM_153813	zinc finger protein, multitype 1	ZFPM1	-1.803780843	-3.491339961	0.915956871	1.886820095	0.000156139	1.850383071
NM_003695	lymphocyte antigen 6 complex, locus D	LY6D	-1.765084784	-3.398939739	0.877530568	1.837227862	0.002578992	1.850037118
NM_015503	SH2-B homolog	SH2B	-1.320091961	-2.496820246	0.432794912	1.349846083	0.001481822	1.849707369
XR_012428	ADAMTS-like 2	LOC713492	-1.326195916	-2.50740652	0.441299012	1.357826373	0.000648756	1.846632656
XR_013257	cyclin M1	LOC708335	-1.701631641	-3.252686187	0.817292257	1.762095672	0.000923258	1.845919174
NM_004448	v-erb-b2 erythroblastic leukemia viral oncogene homolog 2, neuro/glioblastoma derived oncogene homolog	ERBB2	-1.039660919	-2.055744428	0.156312447	1.11443498	0.000144044	1.844651743
NM_182554	chromosome 10 open reading frame 53	C10orf53	-1.617159532	-3.067704525	0.734855946	1.664231287	0.006249521	1.843316221
NM_021926	aristaless-like homeobox 4	ALX4	-1.609344877	-3.051132595	0.731333479	1.660172875	0.001674659	1.837840288
XR_014577	Insulin-like growth factor II precursor IGF-II Somatomedin A	IGF2	-1.680702348	-3.205839832	0.804542965	1.746592397	3.10E-05	1.83548253
NM_002297	lipocalin 1	LCN1	-1.484642306	-2.798477805	0.608943518	1.525141942	0.002165765	1.834896627
CO725791	peroxisomal membrane protein 2	PXMP2	-1.072305683	-2.102791317	0.197649606	1.146828455	0.000683822	1.83357093
NM_032951	Williams Beuren syndrome chromosome region 14	WBSCR14	-1.129920419	-2.18846668	0.2564127	1.19450484	0.00042209	1.832112024
NM_000898	monoamine oxidase B	MAOB	-1.194683511	-2.288946132	0.324034909	1.251826749	0.002363396	1.82848476
NM_145285	NK2 transcription factor related, locus 3	NKX2-3	-1.547852547	-2.923816047	0.678898941	1.600917475	0.005212542	1.826337768
NM_001715	B lymphoid tyrosine kinase	BLK	-1.481321607	-2.792043863	0.613337467	1.529794077	0.001028035	1.825110913
NM_152492	hypothetical protein FLJ32825	FLJ32825	-2.015609717	-4.04351431	1.149449546	2.218292403	6.22E-05	1.822804922
NM_006383	calcium and integrin binding family member 2	CIB2	-1.059192326	-2.083764626	0.193037808	1.143168293	0.001759137	1.822797779
NM_006783	gap junction protein, beta 6	GJB6	-1.565491321	-2.959782825	0.700865223	1.625479344	0.000647321	1.820867693
NM_000364	troponin T2, cardiac	TNNT2	-1.288277546	-2.442362842	0.423774021	1.341432091	0.000223371	1.820712997
NM_175062	RasGEF domain family, member 1C	RASGEF1C	-1.234909804	-2.35366632	0.370971738	1.293223598	0.001872926	1.819999515
NM_001012984	hypothetical gene supported by BC032064; BC041612	LOC388284	-1.90605704	-3.747833964	1.043152147	2.06072522	0.005185918	1.818696606

NM_015198	cordon-bleu homolog	COBL	-1.268716998	-2.409471937	0.406422881	1.325395459	1.33E-05	1.817926809
NM_138619	golgi associated, gamma adaptin ear containing, ARF binding protein 3	GGA3	-1.119800688	-2.173169475	0.257885926	1.195725247	0.000993066	1.81744885
NM_020672	S100 calcium binding protein A14	S100A14	-1.186753054	-2.276398374	0.325008739	1.252672027	0.000315387	1.817234141
NM_016153	LW-1	LW-1	-1.10259865	-2.147411461	0.243622315	1.183961621	0.006239724	1.813750905
NM_014977	apoptotic chromatin condensation inducer 1	ACIN1	-1.035277277	-2.049507511	0.180025269	1.132903728	4.62E-05	1.809074735
NM_054031	MAS-related GPR, member X3	MRGPRX3	-1.450661326	-2.733333176	0.595574763	1.51107447	0.000439762	1.808867286
NM_145274	hypothetical protein MGC21518	MGC21518	-1.074610126	-2.106152829	0.220805654	1.165384198	0.001769306	1.8072605
NM_025132	WD repeat domain 19	WDR19	-1.469086765	-2.768465925	0.617326207	1.534029479	2.56E-05	1.804701906
NM_152657	gametogenetin	GGN	-1.655536204	-3.150402594	0.803838716	1.745740009	0.001008521	1.804623013
NM_003180	synaptotagmin V	SYT5	-1.366416615	-2.578293702	0.516179062	1.430162482	0.000971444	1.802797748
NM_015833	adenosine deaminase, RNA-specific, B1	ADARB1	-1.39285226	-2.625973323	0.542615986	1.456611344	0.0001776	1.80279615
NM_203326	5-azacytidine induced 2	AZI2	-1.145293773	-2.211911668	0.295701308	1.227481523	0.002199025	1.801991824
NM_003564	transgelin 2	TAGLN2	-1.112964728	-2.162896646	0.264572401	1.201279951	3.56E-06	1.800493419
NM_030768	integrin-linked kinase-associated serine/threonine phosphatase 2C	ILKAP	-1.095055291	-2.136212684	0.251012752	1.190042215	0.000288357	1.795073029
NM_173525	hypothetical protein MGC34805	MGC34805	-2.029551835	-4.082780013	1.186159206	2.275461546	0.000406872	1.794264561
NM_015950	mitochondrial ribosomal protein L2	MRPL2	-1.490053575	-2.808994063	0.647103407	1.566020835	0.000349362	1.793714362
NM_007271	serine/threonine kinase 38	STK38	-1.174023974	-2.256401771	0.331796623	1.258579739	0.002294131	1.792815903
NM_173617	hypothetical protein FLJ36701	FLJ36701	-1.370252757	-2.585158536	0.529481504	1.443410349	0.003423892	1.791007344
NM_153340	hypothetical protein MGC46534	MGC46534	-1.337068565	-2.526374589	0.498424406	1.412669917	0.00075664	1.788368648
NM_152572	chromosome 9 open reading frame 98	C9orf98	-1.468068125	-2.766511891	0.630067402	1.547637297	2.81E-05	1.78757122
NM_020435	gap junction protein, alpha 12, 47kDa	GJA12	-1.118578692	-2.171329531	0.282455297	1.21626306	0.002318688	1.785246631
NM_032512	PDZ domain containing 4	PDZK4	-1.105103622	-2.151143281	0.269512413	1.20540037	0.000880215	1.784588204
NM_145245	ecotropic viral integration site 5-like	EVI5L	-1.003360945	-2.004664691	0.168982629	1.124265387	0.00237649	1.783088507
AB075502	neuroblastoma cDNA, clone:Nbla00237, full insert sequence		-1.036805096	-2.051679096	0.203543432	1.151523164	5.85E-05	1.781708923
BQ188851	UI-E-EJ1-ajx-d-17-0-UI.r1 UI-E-EJ1 cDNA clone UI-E-EJ1-ajx-d-17-0-UI 5' sequence		-1.740146077	-3.340689914	0.907536108	1.875839128	0.010232804	1.780904271
NM_198476	FLJ41131 protein	FLJ41131	-1.498865227	-2.826203259	0.668380811	1.589288251	0.001633489	1.778282358
NM_145272	LOC146853	LOC146853	-1.128747691	-2.186688457	0.300634326	1.231685844	0.00737855	1.775362174
NM_018263	additional sex combs like 2	ASXL2	-1.095648505	-2.137091242	0.273439442	1.208685951	5.88E-05	1.768111262
NM_001004450	olfactory receptor, family 1, subfamily B, member 1	OR1B1	-1.490593158	-2.810044853	0.674689858	1.596253583	0.003674655	1.760400029
NM_015190	DnaJ Hsp40	DNAJC9	-1.144129468	-2.210127298	0.336443628	1.262640234	6.93E-05	1.750401452
NM_003110	Sp2 transcription factor	SP2	-1.132430688	-2.192277896	0.324762964	1.252458642	0.012995029	1.750379472
XR_010104	protocadherin LKC precursor	LOC697249	-1.562693421	-2.954048313	0.755308557	1.687992573	0.000865073	1.750036321
XR_011748	phospholipase D family, member 4	LOC707527	-1.631041546	-3.097365306	0.823787009	1.77004619	0.00300869	1.749878237
NM_000272	nephronophthisis 1	NPHP1	-5.064034296	-33.45231843	4.259732938	19.1561129	5.01E-06	1.74629992
NM_013292	myosin light chain 2	HUMMLC2B	-1.937773929	-3.831140485	1.134567183	2.195526858	5.20E-06	1.744975458
NM_015626	WD repeat and SOCS box-containing 1	WSB1	-1.051166222	-2.072204265	0.250970569	1.19000742	3.07E-05	1.741337264
NM_014226	renal tumor antigen	RAGE	-1.258703173	-2.392805569	0.460203354	1.37573572	0.002077525	1.739291591
NM_002854	parvalbumin	PVALB	-1.168122336	-2.247190353	0.370537555	1.292834457	0.004718827	1.738188785
NM_033487	cell division cycle 2-like 1	CDC2L1	-1.219948013	-2.329383232	0.428868227	1.346177106	3.82E-06	1.730369073
XR_013555	ryanodine receptor 2	RYR2	-1.676388222	-3.19626765	0.885398966	1.8472754	0.000117377	1.730260496
NM_173474	N-terminal asparagine amidase	NTAN1	-1.153778149	-2.224958072	0.363127238	1.286210915	7.47E-05	1.729854758
NM_016633	erythroid associated factor	ERAF	-1.436553436	-2.7067346	0.64616763	1.565005394	0.000778307	1.729536914
NM_004127	G protein pathway suppressor 1	GPS1	-1.070809506	-2.100611704	0.285332058	1.218690731	0.000451277	1.723662657

XR_011624	tetratricopeptide repeat domain 16	LOC706784	-1.311922283	-2.482721237	0.531519939	1.445451236	0.000464126	1.717609819
NM_006062	SMYD family member 5	SMYD5	-1.290942292	-2.446878205	0.512499421	1.426519453	0.000597484	1.71527854
NM_153221	cartilage intermediate layer protein 2	CILP2	-1.204475964	-2.304535456	0.428670867	1.345992963	0.001576169	1.712145249
NM_015570	autism susceptibility candidate 2	AUTS2	-1.019658562	-2.027439076	0.244281815	1.184502969	0.001310202	1.711636973
NM_006001	tubulin, alpha 2	TUBA2	-2.000226321	-4.000627545	1.228934159	2.343937593	0.000796821	1.706797807
NM_152408	hypothetical protein FLJ35779	FLJ35779	-1.220523418	-2.330312471	0.449479793	1.365547779	0.000190606	1.706503798
NM_153746	zinc finger, DHHC domain containing 14	ZDHHC14	-2.167850826	-4.493534959	1.397381995	2.634231241	2.75E-05	1.705824033
NM_031429	retbindin	RTBDN	-1.896437696	-3.722927949	1.126347944	2.183054192	5.23E-05	1.705375873
NM_001001936	KIAA1914	KIAA1914	-1.196105587	-2.291203477	0.42623876	1.343725789	0.00096134	1.70511238
NM_017777	hypothetical protein FLJ20345	FLJ20345	-1.235607602	-2.354805009	0.468544039	1.383712327	0.000168522	1.701802436
NM_002762	protamine 2	PRM2	-1.27640852	-2.422351994	0.510775098	1.424815482	1.87E-05	1.700116278
NM_032019	histone deacetylase 10	HDAC10	-1.663005787	-3.166756166	0.898491417	1.864115714	0.000482885	1.698798064
NM_024557	RIC3 protein	RIC3	-4.848363313	-28.80731537	4.084217904	16.96180624	0.000125167	1.698363662
NM_014759	phytanoyl-CoA hydroxylase interacting protein	PHYHIP	-1.924844425	-3.796959011	1.16152687	2.23694048	0.000123143	1.697389379
NM_020927	KIAA1576 protein	KIAA1576	-1.362035422	-2.570475786	0.602007084	1.517826706	0.003206951	1.693523888
NM_013412	RAB, member of RAS oncogene family-like 2A	RABL2A	-1.162111016	-2.237846398	0.406133047	1.325129216	7.14E-05	1.688775986
NM_175885	hypothetical protein MGC33846	MGC33846	-1.49919906	-2.826857305	0.743941528	1.674745096	0.000372213	1.687932875
NM_171997	ubiquitin specific protease 2	USP2	-1.599747354	-3.030902313	0.845092602	1.796380046	0.000507186	1.687227777
NM_005220	distal-less homeo box 3	DLX3	-1.556528779	-2.941452581	0.803258379	1.745037911	2.83E-05	1.685609558
NM_000990	ribosomal protein L27a	RPL27A	-1.053110377	-2.074998621	0.29994389	1.231096532	0.000273897	1.685488154
XR_013017	exosome component 7	EXOSC7	-1.052623992	-2.074299181	0.300693551	1.231736407	0.00027097	1.68404471
NM_022753	hypothetical protein FLJ12903	FLJ12903	-1.09316831	-2.13342044	0.341270098	1.266871412	0.000319969	1.68400709
NM_001702	brain-specific angiogenesis inhibitor 1	BAI1	-1.519964562	-2.867840051	0.769829729	1.705068534	0.000867828	1.681950017
NM_005244	eyes absent homolog 2	EYA2	-1.114154019	-2.164680373	0.366851093	1.289535145	0.000323192	1.678651708
NM_003631	poly ADP-ribose	PARG	-1.281530752	-2.430967746	0.537153407	1.451106501	4.15E-05	1.675251089
CN641580	low density lipoprotein receptor	LDLR	-1.239513451	-2.361188877	0.49549019	1.409799686	3.72E-05	1.674839979
NM_182553	hypothetical protein MGC50896	MGC50896	-1.722535564	-3.300159067	0.978594595	1.970544862	0.00036248	1.674744448
NM_178500	phosphatase, orphan 1	PHOSPHO1	-1.328440038	-2.511309843	0.585745824	1.500814659	0.000146734	1.673297784
NM_003085	synuclein, beta	SNCB	-1.742030972	-3.34505741	0.999717042	1.999607775	0.004759106	1.672856773
NM_000264	patched homolog	PTCH	-1.066285912	-2.094035516	0.331473101	1.258297536	0.003649661	1.664181528
NM_080722	a disintegrin-like and metalloprotease	ADAMTS14	-1.37700879	-2.59729303	0.645731172	1.564532005	0.001416177	1.660108596
NM_054016	FUS interacting protein serine-arginine rich	FUSIP1	-1.504528032	-2.83731835	0.77356681	1.709490979	7.48E-05	1.659744559
NM_145283	chromosome 9 open reading frame 121	C9orf121	-1.602339696	-3.036353359	0.871626004	1.829723948	0.000499598	1.659459812
NM_001003792	RNA binding motif, single stranded interacting protein	RBMS3	-1.170880074	-2.251490006	0.441414425	1.357935001	0.019361125	1.658024873
NM_014447	ADP-ribosylation factor interacting protein 1	ARFIP1	-1.149188795	-2.217891507	0.419896135	1.337831236	0.000384188	1.657826075
NM_020693	Down syndrome cell adhesion molecule like 1	DSCAML1	-1.326510298	-2.507952976	0.598501653	1.514143198	0.000590503	1.656351248
NM_004599	sterol regulatory element binding transcription factor 2	SREBF2	-1.059382512	-2.084039341	0.332145946	1.258884519	0.000869704	1.655465064
NM_020219	carcinoembryonic antigen-like 1	CEAL1	-1.187362304	-2.2773599	0.465408108	1.380707872	0.000557145	1.649414729
NM_022821	elongation of very long chain fatty acids	ELOVL1	-1.093600461	-2.134059589	0.373161709	1.295188166	0.000142981	1.647683051
NM_023946	Ly6/neurotoxin 1	LYNX1	-1.3425529	-2.535996756	0.62393821	1.541076212	0.000467246	1.64560113
NM_013313	yippee-like 1	YPEL1	-1.053238051	-2.075182259	0.339630852	1.265432761	0.000725278	1.639899268
NM_152283	zinc finger protein 62 homolog	ZFP62	-1.08240319	-2.117560502	0.369675405	1.292062094	0.020714182	1.638899951
NM_177951	protein phosphatase 1A	PPM1A	-1.188562033	-2.279254514	0.479051642	1.393827131	0.001199913	1.635249066
XR_010258	rhophilin 1	LOC696895	-1.418807341	-2.673643926	0.710569327	1.636449778	0.000170923	1.633807503
NM_172006	WAP four-disulfide core domain 10B	WFDC10B	-1.483713329	-2.796676397	0.780871488	1.718168453	8.05E-05	1.627707919
XR_012837	hypothetical protein LOC706711	LOC706711	-1.204726308	-2.304935386	0.502116008	1.416289319	5.25E-05	1.627446706

NM_014619	glutamate receptor, ionotropic, kainate 4	GRIK4	-1.561274386	-2.951144139	0.866568877	1.823321383	0.001390055	1.618554011
NM_145291	zinc finger protein 509	ZNF509	-1.190801693	-2.282795609	0.498040768	1.412294313	0.000445635	1.616373859
NM_138360	hypothetical protein BC008134	LOC90668	-1.409914486	-2.65721412	0.72201241	1.649481286	0.003366259	1.610939234
NM_005613	regulator of G-protein signalling 4	RGS4	-1.201333919	-2.29952187	0.514827933	1.428823717	0.002488328	1.609381089
NM_004506	heat shock transcription factor 2	HSF2	-1.106382495	-2.153051002	0.420387523	1.338286984	0.000643398	1.608811135
NM_207373	chromosome 10 open reading frame 99	C10orf99	-1.402130532	-2.642915926	0.718251002	1.645186344	0.002945262	1.606453844
NM_030651	chromosome 6 open reading frame 31	C6orf31	-1.30512967	-2.471059364	0.622025849	1.539034795	0.003931623	1.605590317
NM_052863	secretoglobin, family 3A, member 1	SCGB3A1	-1.096107513	-2.137771288	0.413087752	1.331532602	0.001097002	1.605496767
NM_003635	N-deacetylase/N-sulfotransferase heparan glucosaminyl	NDST2	-1.336232228	-2.524910462	0.657005324	1.576806167	0.000672754	1.601281448
NM_024410	outer dense fiber of sperm tails 1	ODF1	-1.43887803	-2.71109944	0.762489152	1.696415008	0.001550196	1.598134552
NM_207429	FLJ45803 protein	FLJ45803	-1.287412855	-2.440899431	0.616045272	1.532668055	0.012317192	1.592581918
NM_004502	homeo box B7	HOXB7	-1.715065287	-3.283115004	1.044378929	2.062478283	0.000118342	1.591830096
NM_001008695	THAP domain containing 7	THAP7	-1.06532297	-2.092638297	0.395239536	1.315161103	0.000468989	1.591164985
NM_001005368	zinc finger protein 32	ZNF32	-1.312086063	-2.483003101	0.644334121	1.563017705	0.000296567	1.588595633
NM_002073	guanine nucleotide binding protein	GNAZ	-1.055652972	-2.078658807	0.395175889	1.315103084	0.003281601	1.580605226
NM_031206	hypothetical protein FLJ12525	FLJ12525	-1.003229863	-2.004482556	0.344055333	1.269319568	5.81E-05	1.579178803
NM_147193	GLIS family zinc finger 1	GLIS1	-1.033676044	-2.047234044	0.375755437	1.297518795	0.022726484	1.577806851
NM_148175	peptidylprolyl isomerase cyclophilin	PPIL2	-1.130594834	-2.189489959	0.472878065	1.387875413	0.001178632	1.577583938
NM_002769	protease, serine, 1	PRSS1	-1.698237532	-3.245042857	1.041947576	2.059005346	0.007709369	1.576024493
NM_018171	DIP13 beta	DIP13B	-1.11728226	-2.16937921	0.466017892	1.381291579	0.000351633	1.570544006
XR_009754	oxysterol-binding protein-like protein 7	OSBPL7	-1.400745969	-2.640380721	0.751763637	1.683850012	0.001528867	1.568061705
NM_016437	tubulin, gamma 2	TUBG2	-1.433841083	-2.701650558	0.785184101	1.723312215	0.00173822	1.567708123
NM_004669	chloride intracellular channel 3	CLIC3	-1.228080949	-2.342551799	0.579830557	1.494673691	7.82E-06	1.567266363
NM_022551	ribosomal protein S18	RPS18	-1.00428548	-2.00594977	0.356062428	1.279927797	4.25E-05	1.567236663
NM_032638	GATA binding protein 2	GATA2	-1.146632788	-2.213965572	0.499464441	1.413688674	0.000105961	1.566091328
XR_014428	Regulator of G-protein signaling 8	RGS8	-1.434338309	-2.702581844	0.791165947	1.730472419	0.000703436	1.561759561
NM_152296	ATPase, Na+/K+ transporting, alpha 3 polypeptide	ATP1A3	-1.696948021	-3.242143667	1.055892507	2.079003961	9.33E-05	1.559469692
NM_032467	aspartate beta-hydroxylase	ASPH	-1.14625929	-2.213392475	0.505785108	1.419895847	0.000202401	1.558841432
NM_014174	thymocyte protein thy28	THY28	-1.061778525	-2.087503368	0.42167739	1.339484039	0.000619686	1.558438405
XR_010002	hypothetical protein LOC695376	LOC695376	-1.180069546	-2.265876996	0.54112797	1.455109749	0.001779433	1.557186321
NM_005361	melanoma antigen family A, 2	MAGEA2	-1.263011224	-2.399961437	0.62559002	1.542841673	0.0033932	1.555546157
NM_015133	mitogen-activated protein kinase 8 interacting protein 3	MAPK8IP3	-1.49668702	-2.821939425	0.86205202	1.817621771	9.99E-06	1.552544908
NM_002150	4-hydroxyphenylpyruvate dioxygenase	HPD	-1.549929493	-2.928028291	0.915398056	1.886089394	7.77E-06	1.552433464
NM_003087	synuclein, gamma breast cancer-specific protein 1	SNCG	-1.013208855	-2.018395444	0.379994733	1.301337105	0.000737162	1.551016594
XM_376007	DEP domain containing 5	DEPDC5	-1.680782696	-3.206018378	1.048437907	2.068289173	0.001653716	1.550082271
NM_001967	eukaryotic translation initiation factor 4A, isoform 2	EIF4A2	-1.173673686	-2.25585398	0.544130076	1.458140842	1.51E-05	1.547075506
NM_000638	vitronectin serum spreading factor, somatomedin B, complement S-protein	VTN	-1.328344961	-2.511144347	0.700086434	1.624602122	0.000154668	1.545698059
NM_054028	acyl-malonyl condensing enzyme 1-like 2	AMAC1L2	-1.048352988	-2.068167435	0.421623623	1.339434119	0.001964164	1.54406059
NM_153381	pro-melanin-concentrating hormone-like 2	PMCHL2	-1.661277753	-3.162965354	1.036780438	2.051644029	1.90E-05	1.54167356
NM_002675	promyelocytic leukemia	PML	-1.181438686	-2.268028369	0.559694996	1.473957571	0.001483028	1.538733823
NM_014233	upstream binding transcription factor, RNA polymerase I	UBTF	-1.022109274	-2.030886022	0.40136586	1.320757736	0.000779554	1.53766733
NM_017767	solute carrier family 39 zinc transporter	SLC39A4	-2.994729736	-7.970828764	2.375349261	5.188614174	0.019816868	1.536215355
NM_014293	neuronal pentraxin receptor	NPTXR	-1.138649904	-2.201748837	0.520335349	1.434288604	0.001793631	1.535080758
NM_015401	histone deacetylase 7A	HDAC7A	-1.126693507	-2.183577153	0.511331505	1.425365099	7.65E-06	1.531942345
NM_144565	homolog of Drosophila Numb-interacting protein	NIP	-1.570256346	-2.969574744	0.960921177	1.946552394	0.003128453	1.525556031

NM_020974	signal peptide, CUB domain, EGF-like 2	SCUBE2	-1.059794397	-2.084634413	0.452752965	1.368649437	0.000629183	1.523132481
NM_022817	period homolog 2	PER2	-1.255213699	-2.387025038	0.649729142	1.568873621	7.21E-06	1.521489689
NM_005583	lymphoblastic leukemia derived sequence 1	LYL1	-1.915459019	-3.77233819	1.313662565	2.485717881	7.23E-05	1.517605123
NM_175918	hypothetical protein FLJ34443	FLJ34443	-1.266990758	-2.406590635	0.678116724	1.600049707	0.000129361	1.504072421
NM_006392	nucleolar protein 5A	NOL5A	-1.016179647	-2.022555998	0.428711318	1.346030702	4.98E-05	1.502607626
NM_004454	ets variant gene 5	ETV5	-1.036043233	-2.050595925	0.449769689	1.365822201	0.000255004	1.501363738
XR_014568	lymphocyte-specific protein 1 isoform 1	LOC721048	-1.499825816	-2.828085654	0.916045446	1.88693594	0.000260543	1.498771418
NM_006458	tripartite motif-containing 3	TRIM3	-1.54135538	-2.910678268	0.958057259	1.942692093	1.43E-06	1.498270508
NM_018052	hypothetical protein FLJ10305	FLJ10305	-1.058124706	-2.082223174	0.479682112	1.394436378	4.85E-05	1.493236412
NM_004435	endonuclease G	ENDOG	-1.100660726	-2.144528853	0.530977689	1.444908053	0.000830526	1.484197454
NM_005462	melanoma antigen family C, 1	MAGEC1	-1.381904284	-2.606121389	0.813081185	1.756959803	0.00146841	1.483313041
AB209405	receptor-like tyrosine kinase isoform 1 variant protein	RYK	-1.073872953	-2.105076923	0.505218859	1.419338656	0.000132021	1.483139288
NM_002749	mitogen-activated protein kinase 7	MAPK7	-1.070424368	-2.100051005	0.503632472	1.417778809	0.000246008	1.481226121
NM_031293	polyamine modulated factor 1 binding protein 1	PMFBP1	-1.475628196	-2.781047133	0.908846082	1.877543173	0.00010187	1.481216077
NM_000192	T-box 5	TBX5	-1.091750464	-2.131324796	0.525345833	1.439278553	0.000558399	1.480828566
NM_033290	midline 1	MID1	-1.372030934	-2.588346807	0.810485255	1.75380124	0.000255066	1.47584957
NM_002257	kallikrein 1, renal/pancreas/salivary	KLK1	-1.398693804	-2.636627575	0.837745537	1.78725506	0.000393413	1.475238557
NM_005007	nuclear factor of kappa light polypeptide gene enhancer in B-cells inhibitor like 1	NFKBIL1	-1.073416601	-2.104411153	0.515858942	1.429845178	4.45E-05	1.471775536
NM_016366	calcium binding protein 2	CABP2	-1.39460556	-2.629166596	0.837077373	1.786427509	0.000651589	1.471745471
NM_001003794	monoglyceride lipase	MGLL	-1.616873603	-3.067096594	1.070799149	2.100596625	8.87E-06	1.460107361
NM_005379	myosin IA	MYO1A	-1.148750156	-2.217217279	0.605802565	1.521825103	7.79E-05	1.456946186
NM_000537	renin	REN	-1.625976093	-3.086509203	1.085898256	2.122696712	5.12E-06	1.454050965
NM_001006607	c114 SLIT-like testicular protein	LOC474170	-1.258179551	-2.391937264	0.719752756	1.64689977	6.54E-05	1.452387879
NM_005481	thyroid hormone receptor associated protein 5	THRAP5	-1.34700943	-2.54384264	0.809332124	1.752400006	0.001116464	1.45163355
NM_015296	dedicator of cytokinesis 9	DOCK9	-1.1189064	-2.171822804	0.592156647	1.507498584	1.99E-05	1.44067983
NM_019071	inhibitor of growth family, member 3	ING3	-1.116965604	-2.168903107	0.596150849	1.511677982	0.003137501	1.434765295
NM_139215	TAF15 RNA polymerase II, TATA box binding protein	TAF15	-1.033080898	-2.046389685	0.520121932	1.434076446	0.000500291	1.426973918
NM_001003692	zinc finger, matrin type 5	ZMAT5	-1.005465958	-2.007591799	0.493845942	1.408193845	0.000323053	1.425650173
NM_025257	chromosome 6 open reading frame 29	C6orf29	-1.347949962	-2.545501582	0.838637426	1.788360301	0.00074427	1.423371778
NM_145178	atonal homolog 7	ATOH7	-1.779682718	-3.433506555	1.271687531	2.41443819	0.00116312	1.422072667
NM_000258	myosin, light polypeptide 3, alkali; ventricular, skeletal, slow	MYL3	-1.302410765	-2.466406788	0.79635104	1.736702982	0.004062442	1.42016615
NM_020695	transcription elongation factor B polypeptide 3 binding protein 1	TCEB3BP1	-1.311947873	-2.482765275	0.823122401	1.769230969	3.61E-07	1.403301954
NM_014578	ras homolog gene family, member D	RHOD	-1.043447662	-2.061147373	0.557359672	1.471573571	0.005138097	1.400641743
NM_033259	CaM-KII inhibitory protein	CAM-KIIN	-1.495908683	-2.820417394	1.01043827	2.01452299	4.86E-06	1.400042297
NM_024342	glucocorticoid receptor DNA binding factor 1	GRLF1	-1.103080555	-2.148128883	0.61826326	1.535026179	0.000159574	1.399408631
NM_206889	chromosome 21 open reading frame 106	C21orf106	-1.441582735	-2.716186863	0.958244555	1.942944317	0.00045152	1.39797463
NM_012467	tryptase gamma 1	TPSG1	-1.396482182	-2.632588776	0.913319423	1.883373876	0.018478073	1.397804658
NM_006032	copine VI	CPNE6	-1.162726413	-2.238801179	0.680633628	1.602843565	0.004012582	1.396768361
XR_010916	N-acetylgalactosamine-6-sulfatase precursor	LOC697850	-1.234027989	-2.352228135	0.758935841	1.692241935	2.67E-05	1.390007
NM_032637	S-phase kinase-associated protein 2	SKP2	-1.071069464	-2.100990246	0.597614461	1.513212355	0.001584768	1.388430539
NM_018944	chromosome 21 open reading frame 45	C21orf45	-1.138325238	-2.201253408	0.668516612	1.589437857	0.001296978	1.384925745
NM_002449	msh homeo box homolog 2	MSX2	-1.625439878	-3.085362236	1.157411487	2.230568551	0.000146694	1.38321785
NM_173846	chromosome 14 open reading frame 8	C14orf8	-2.304641821	-4.940447829	1.842495437	3.586298159	0.002788686	1.377589818
XR_014301	basic helix-loop-helix domain containing, class B, 4	LOC719866	-1.394404385	-2.628799999	0.933206371	1.909515156	0.001421444	1.376684543

NM_004626	wingless-type MMTV integration site family, member 11	WNT11	-1.620010396	-3.073772512	1.159632832	2.234005647	0.000388347	1.375901854
NM_006342	transforming, acidic coiled-coil containing protein 3	TACC3	-1.042837137	-2.060275314	0.588892988	1.504092181	0.000105333	1.36977995
NM_020064	BarH-like 1	BARHL1	-2.027743554	-4.077665844	1.589537978	3.009529542	0.000660771	1.354918032
NM_015015	jumonji domain containing 2B	JMJD2B	-1.428400195	-2.691480915	0.996801185	1.995570413	0.000863386	1.34872761
NM_198207	LAG1 longevity assurance homolog 1	LASS1	-1.174601595	-2.257305361	0.747773001	1.679198754	0.003403621	1.344275271
NM_004010	dystrophin	DMD	-1.153133375	-2.223963908	0.72686146	1.655034694	0.003224759	1.343756669
NM_173633	hypothetical protein FLJ90805	FLJ90805	-1.11305397	-2.163030443	0.689212396	1.612403026	0.000134211	1.341494904
NM_006048	ubiquitination factor E4B	UBE4B	-1.136366224	-2.198266388	0.715146713	1.641650154	0.00043251	1.339058985
NM_000805	gastrin	GAST	-1.338122286	-2.528220485	0.917321949	1.888606249	0.000666416	1.338669977
NM_001338	coxsackie virus and adenovirus receptor	CXADR	-1.118480962	-2.171182447	0.700839497	1.625450359	0.000693686	1.335742082
NM_018349	multiple C2-domains with two transmembrane regions 2	MCTP2	-1.11693014	-2.168849793	0.714013616	1.640361304	0.039976653	1.3221781
NM_152468	epidermodysplasia verruciformis 2	EVER2	-1.080566554	-2.114866437	0.682486156	1.604903058	9.44E-05	1.317753384
NM_033068	acid phosphatase, testicular	ACPT	-2.339826424	-5.062417262	1.942419627	3.843497225	4.06E-06	1.317138264
NM_006482	dual-specificity tyrosine-	DYRK2	-1.046008578	-2.064809348	0.667358126	1.588162049	7.68E-05	1.300125103
NM_014417	BCL2 binding component 3	BBC3	-1.177978633	-2.262595415	0.811358024	1.754862536	4.25E-05	1.289329146
NM_198534	FLJ35784 protein	FLJ35784	-1.234576643	-2.353122852	0.868167419	1.825342789	0.000793894	1.289140246
NM_003577	undifferentiated embryonic cell transcription factor 1	UTF1	-1.006241006	-2.008670613	0.644041648	1.562700872	0.000102874	1.285383946
NM_000554	cone-rod homeobox	CRX	-1.193232065	-2.28664446	0.833890595	1.782485814	0.003396479	1.282840201
NM_001003940	Bcl2 modifying factor	BMF	-1.069566921	-2.098803239	0.710413172	1.636272661	0.000437818	1.282673291
NM_002226	jagged 2	JAG2	-1.269304002	-2.410452503	0.91573506	1.886530023	6.03E-05	1.277717541
NM_022119	protease, serine, 22	PRSS22	-1.076947218	-2.109567453	0.73120927	1.660029949	0.000283326	1.27080084
NM_002383	MYC-associated zinc finger protein	MAZ	-1.349598959	-2.548412747	1.015394045	2.02145494	2.19E-05	1.26068244
NM_001118	adenylate cyclase activating polypeptide 1	ADCYAP1R1	-1.236135098	-2.355666159	0.909811205	1.878799618	4.75E-05	1.253814477
NM_022553	vacuolar protein sorting 52	VPS52	-1.688697499	-3.223655326	1.367413722	2.580076284	2.73E-05	1.249441865
NM_002230	junction plakoglobin	JUP	-1.226279576	-2.339628675	0.907729081	1.876090055	0.000157903	1.247076956
BC108680	cDNA clone MGC:131759 IMAGE:5416073, complete cds		-1.032733536	-2.04589703	0.714606837	1.641035942	0.000313953	1.246710677
NM_080647	T-box 1	TBX1	-1.162473291	-2.238408415	0.844550637	1.795705341	0.001146961	1.246534363
NM_052891	peptidoglycan recognition protein 3	PGLYRP3	-1.091942814	-2.131608977	0.774378303	1.710452811	3.77E-06	1.246224954
NM_025082	hypothetical protein FLJ13111	FLJ13111	-1.138425681	-2.201406669	0.82337505	1.769540829	0.000109082	1.244055312
NM_000426	laminin, alpha 2	LAMA2	-1.111478535	-2.160669685	0.801045586	1.742363438	0.01434704	1.240079789
NM_022834	von Willebrand factor A domain containing 1	VWA1	-1.066844009	-2.094845737	0.761427621	1.69516725	5.87E-05	1.235775253
NM_033405	peroxisomal proliferator-activated receptor A interacting complex 285	PRIC285	-1.776815611	-3.426689829	1.472883676	2.77576162	0.007974464	1.234504362
XR_011719	maestro	LOC701314	-1.087049756	-2.124391638	0.799280346	1.740232836	0.014229927	1.220751381
NM_012234	RING1 and YY1 binding protein	RYBP	-1.272896552	-2.416462409	1.006948047	2.009655269	1.93E-06	1.20242633
NM_032300	hypothetical protein MGC10854	MGC10854	-1.127546111	-2.184867985	0.863152268	1.81900848	0.003369082	1.201131281
NM_173637	hypothetical protein MGC34725	MGC34725	-1.251399821	-2.380723082	0.999150168	1.99882223	0.000814634	1.19106294
NM_199054	MAP kinase interacting serine/threonine kinase 2	MKNK2	-1.216950245	-2.324548045	0.997254732	1.996197869	9.89E-05	1.16448779
NM_005157	v-abl Abelson murine leukemia viral oncogene homolog 1	ABL1	-1.246070594	-2.37194506	1.027585698	2.038609849	6.41E-05	1.163511037
XR_014021	protein kinase N1 isoform 2	LOC718834	-1.026875674	-2.037606792	0.838277283	1.787913925	0.000349658	1.139655978
NM_022066	likely ortholog of mouse ubiquitin-conjugating enzyme E2-230K	E2-230K	-1.130256492	-2.188976539	0.946871174	1.927687474	0.001105849	1.135545346
NM_005568	LIM homeobox 1	LHX1	-1.035546554	-2.049890084	0.868886378	1.826252665	0.000215829	1.122456998
NM_152426	apolipoprotein B mRNA editing enzyme, catalytic polypeptide-like 3D	APOBEC3D	-1.148501742	-2.216835536	0.997662494	1.996762152	0.003168309	1.110215122
NM_004378	cellular retinoic acid binding protein 1	CRABP1	-1.437667627	-2.708825815	1.288688829	2.44305921	0.002818445	1.108784349

NM_031887	pro-melanin-concentrating hormone-like 1	PMCHL1	-1.159453937	-2.233728645	1.023832452	2.033313193	0.000878	1.098565953
XR_011854	thyroid hormone receptor-associated protein 4	THRAP4	-1.254001966	-2.385020995	1.123804791	2.179209339	7.55E-06	1.09444327
NM_198585	ectonucleoside triphosphate diphosphohydrolase 8	ENTPD8	-1.139442996	-2.202959536	1.011020833	2.015336623	4.07E-05	1.093097556
NM_181784	sprouty-related, EVH1 domain containing 2	SPRED2	-1.215174794	-2.321689103	1.097632088	2.140031582	0.000293629	1.08488544
NM_005809	peroxiredoxin 2	PRDX2	-1.050441546	-2.071163644	0.936144786	1.913408332	2.73E-05	1.08244728
NM_001035254	family with sequence similarity 102, member A	FAM102A	-4.54934768	-23.41478165	4.435783033	21.64231667	4.36E-06	1.081898117
NM_033118	myosin light chain kinase 2, skeletal muscle	MYLK2	-1.502676682	-2.833679674	1.400433941	2.639809717	0.000810069	1.073440883
NM_031945	tetraspanin 10	TSPAN10	-1.187649529	-2.277813343	1.095735823	2.137220593	0.005096919	1.065782985
NM_003867	fibroblast growth factor 17	FGF17	-1.354810747	-2.55763653	1.315906267	2.489586716	9.32E-06	1.027333418
NM_207474	FLJ42953 protein	FLJ42953	-1.90777449	-3.752298211	1.870224146	3.655893759	0.01092539	1.026369599
NM_173158	nuclear receptor subfamily 4, group A, member 1	NR4A1	-1.27436701	-2.418926629	1.256083476	2.388464569	5.69E-06	1.012753825
NM_001665	ras homolog gene family, member G	RHOG	-1.463483399	-2.757734175	1.465120371	2.76086505	9.04E-05	0.99886598
NM_024335	iroquois homeobox protein 6	IRX6	-1.270852777	-2.413041583	1.273965474	2.418253478	3.87E-05	0.997844769
NM_145003	hypothetical protein FLJ31164	FLJ31164	-1.609381113	-3.05120923	1.631787068	3.098966304	1.59E-08	0.984589353
NM_001286	chloride channel 6	CLCN6	-2.763350521	-6.78971266	2.786563028	6.899840577	4.47E-06	0.984039064
NM_001996	fibulin 1	FBLN1	-2.766393041	-6.804046689	2.806122606	6.994023318	6.50E-06	0.97283729
CN642591	synapse defective 1, Rho GTPase, homolog 1	SYDE1	-1.333612481	-2.520329712	1.377936583	2.59896388	0.000129924	0.969744032
NM_173479	hypothetical protein LOC126248	LOC126248	-1.320033274	-2.496718681	1.373247391	2.590530179	3.60E-05	0.963786758
NM_178466	chromosome 20 open reading frame 71	C20orf71	-1.020680733	-2.028876055	1.075964647	2.108131187	0.000533333	0.962405028
NM_033212	hypothetical protein LOC92922	MGC10992	-1.189860616	-2.281307016	1.269731822	2.411167409	0.001177035	0.946142109
NM_033342	tripartite motif-containing 7	TRIM7	-1.27805708	-2.425121585	1.363470873	2.573034627	0.001706727	0.942514166
NM_002740	protein kinase C, iota	PRKCI	-1.349236396	-2.547772388	1.474268417	2.778427156	0.002177414	0.916983691
NM_019612	hypothetical protein R30953_1	R30953_1	-1.336252098	-2.524945238	1.463168691	2.757132672	1.17E-05	0.91578663
NM_052919	KIAA1920 protein	KIAA1920	-1.127597014	-2.184945075	1.348698136	2.546822008	0.000156757	0.857910395
NM_003253	T-cell lymphoma invasion and metastasis 1	TIAM1	-2.751292143	-6.73319918	3.013101471	8.072980857	0.000126016	0.834041267
NM_139021	mitogen-activated protein kinase 15	MAPK15	-1.260428919	-2.395669546	1.536169172	2.90023373	4.07E-05	0.826026372
NM_012367	olfactory receptor, family 2, subfamily B, member 6	OR2B6	-3.529887416	-11.55053215	3.889431341	14.81956649	0.00015921	0.779410933
NM_013337	translocase of inner mitochondrial membrane 22 homolog	TIMM22	-1.073129754	-2.103992781	1.464170112	2.759047152	3.39E-06	0.762579494
NM_030931	defensin, beta 126	DEFB126	-1.400542719	-2.640008765	1.793365257	3.466224882	5.98E-05	0.761638051
NM_006887	zinc finger protein 36, C3H type-like 2	ZFP36L2	-1.117036732	-2.169010041	1.579172101	2.987983334	2.15E-06	0.725911024
NM_003479	protein tyrosine phosphatase type IVA, member 2	PTP4A2	-1.478807963	-2.787183451	2.907714302	7.504283344	1.31E-05	0.371412342
NM_002229	jun B proto-oncogene	JUNB	-1.00188941	-2.002620995	-2.060413047	-4.171057056	0.018295105	0.480123136
CR604926	full-length cDNA clone CS0DF038YH05 of Fetal brain of	human	-1.089461718	-2.127946261	-0.756473426	-1.689356053	0.011560751	1.259619757
NM_153265	hypothetical protein FLJ35827	FLJ35827	-1.079146774	-2.112786186	-0.721102737	-1.648441555	0.014603493	1.281687045
NM_002432	myeloid cell nuclear differentiation antigen	MNDA	-1.015491951	-2.021592128	-0.612828861	-1.52925486	0.007483203	1.321945857
NM_016115	ankyrin repeat and SOCS box-containing 3	ASB3	-1.048742302	-2.068725609	-0.637826534	-1.555983254	0.010008858	1.329529481
NM_006340	BAI1-associated protein 2	BAIAP2	-1.017706213	-2.024697269	-0.583262435	-1.498233448	0.008223338	1.351389713
NM_000954	prostaglandin D2 synthase 21kDa	PTGDS	-1.263367592	-2.400554336	-0.816611581	-1.761264496	0.028115708	1.362972082
XR_014061	WAP four-disulfide core domain 1 precursor	LOC715230	-1.029667179	-2.041553223	-0.570361189	-1.484895278	0.002714908	1.374880271
NM_001009996	DALR anticodon binding domain containing 3	DALRD3	-1.295915535	-2.455327612	-0.784601861	-1.722616865	0.033578717	1.425347482
NM_032890	dispatched homolog 1	DISP1	-1.494585633	-2.817832063	-0.978868835	-1.970919476	0.024399206	1.429704307
NM_006275	splicing factor, arginine/serine-rich 6	SFRS6	-1.139103945	-2.202441875	-0.617222008	-1.533918688	0.036259036	1.435827005
NM_002333	low density lipoprotein receptor-related protein 3	LRP3	-1.025061419	-2.035046019	-0.494347383	-1.408683379	0.001090992	1.444644019
NM_005600	nitrilase 1	NIT1	-1.010431643	-2.014513737	-0.455379732	-1.371143657	0.027950108	1.469221497
NM_000548	tuberous sclerosis 2	TSC2	-1.147331213	-2.215037638	-0.588968713	-1.50417113	0.006272327	1.472596829
XR_014317	splicing factor 1	SF1	-1.015960913	-2.022249373	-0.455911576	-1.371649217	0.018024537	1.474319635

NM_022118	chromosome 13 open reading frame 10	C13orf10	-1.317167554	-2.491764207	-0.749265906	-1.680937292	0.022642143	1.48236595
CN801728	hemoglobin, alpha 2	HBA2	-1.155951359	-2.228312173	-0.587258513	-1.502389112	0.000880624	1.483179128
NM_007144	ring finger protein 110	RNF110	-1.009312935	-2.012952229	-0.435387928	-1.352274399	0.005084916	1.488567875
NM_014572	LATS, large tumor suppressor, homolog 2	LATS2	-1.068560622	-2.097339805	-0.489974939	-1.404420479	0.025148423	1.493384521
NM_199341	hypothetical protein LOC374920	LOC374920	-1.046397891	-2.065366614	-0.455097314	-1.370875272	0.044753732	1.506604326
NM_025128	MUS81 endonuclease homolog	MUS81	-1.112577606	-2.162316349	-0.520716994	-1.434668076	0.013290253	1.507189283
NM_017732	hypoxia-inducible factor prolyl 4-hydroxylase	PH-4	-1.129607831	-2.187992558	-0.528128722	-1.442057531	0.017904745	1.517271337
NM_013382	protein-O-mannosyltransferase 2	POMT2	-1.115743262	-2.167066255	-0.5114647	-1.425496701	0.030108404	1.520218359
NM_006625	FUS interacting protein serine-arginine rich	FUSIP1	-1.003549543	-2.00492677	-0.393485305	-1.313562917	0.035549897	1.52632717
NM_000669	alcohol dehydrogenase 1C	ADH1C	-1.15290214	-2.223607479	-0.536401947	-1.450350857	0.015303666	1.533151422
NM_004584	RAD9 homolog A	RAD9A	-1.072300587	-2.102783889	-0.443795652	-1.360178178	0.009826039	1.545962082
CN645262	transcription elongation regulator 1	TCERG1	-1.144526335	-2.210735359	-0.508756884	-1.422823674	0.02374323	1.553766218
NM_001451	forkhead box F1	FOXF1	-1.011814416	-2.016445504	-0.374851117	-1.29670573	0.027435704	1.555052513
NM_144698	hypothetical protein FLJ25124	FLJ25124	-1.154711609	-2.22639814	-0.511376791	-1.425409842	0.016334502	1.561935434
NM_133175	amyloid beta	APBB3	-1.003473252	-2.004820751	-0.352787215	-1.277025395	0.008851004	1.569914551
NM_005605	protein phosphatase 3 formerly 2B calcineurin A gamma	PPP3CC	-1.029532025	-2.041361976	-0.374087063	-1.296019174	0.037480951	1.575101678
XM_042698	ubiquitin specific protease 22	USP22	-1.121477068	-2.175696118	-0.458950788	-1.374541808	0.001884529	1.582851904
NM_000187	homogentisate 1,2-dioxygenase homogentisate oxidase	HGD	-1.038871823	-2.054620327	-0.376324187	-1.298030414	0.023438095	1.582875336
NM_181526	myosin, light polypeptide 9, regulatory	MYL9	-1.020202909	-2.028204198	-0.332239148	-1.258965848	0.000243062	1.611008115
NM_020740	ankyrin repeat and FYVE domain containing 1	ANKFY1	-1.079798632	-2.11374103	-0.391403257	-1.311668596	0.016742959	1.611490155
NM_024729	myosin, heavy polypeptide 14	MYH14	-1.054794398	-2.077422127	-0.364242297	-1.287205412	0.002696595	1.61390102
NM_006702	neuropathy target esterase	NTE	-1.165818569	-2.243604791	-0.464410936	-1.379753874	0.002298976	1.62609059
NM_018113	lipocalin-interacting membrane receptor	LIMR	-1.226502406	-2.339990067	-0.523405727	-1.437344342	4.31E-05	1.627995463
NM_004192	acetylserotonin O-methyltransferase-like	ASMTL	-1.050427633	-2.07114367	-0.342284375	-1.267762391	0.030568193	1.633700198
NM_032105	protein phosphatase 1, regulatory inhibitor	PPP1R12B	-1.660407808	-3.161058661	-0.947436635	-1.928443175	0.001547481	1.639176462
NM_001354	aldo-keto reductase family 1, member C2	AKR1C2	-1.374918532	-2.593532651	-0.657751912	-1.577622368	0.029478569	1.643950227
NM_032147	ubiquitin specific protease 44	USP44	-1.207127651	-2.308775109	-0.488779927	-1.403257652	0.02217735	1.645296646
NM_000287	peroxisomal biogenesis factor 6	PEX6	-1.18826188	-2.278780365	-0.469370837	-1.38450555	0.000912476	1.645916382
NM_032118	hypothetical protein FLJ12953	FLJ12953	-1.062927106	-2.089165965	-0.341529756	-1.267099446	0.000208122	1.648778217
NM_001009820	small nuclear ribonucleoprotein 70kDa polypeptide RNP antigen	SNRP70	-1.117053315	-2.169034973	-0.395287215	-1.315204568	0.009413408	1.649199695
XM_498462	hypothetical protein LOC203522	LOC203522	-1.210623339	-2.314376115	-0.483176246	-1.397817725	0.025307666	1.655706658
NM_004242	high mobility group nucleosomal binding domain 3	HMGN3	-1.439645401	-2.712541859	-0.711411465	-1.637405294	0.031035525	1.656609924
NM_145798	oxysterol binding protein-like 7	OSBPL7	-1.123064474	-2.178091367	-0.393741655	-1.313796343	0.006294352	1.657860732
NM_013974	dimethylarginine dimethylaminohydrolase 2	DDAH2	-1.113134726	-2.163151523	-0.381747528	-1.302919119	0.006370489	1.660234693
NM_033396	tankyrase 1 binding protein 1, 182kDa	TNKS1BP1	-1.046458735	-2.065453721	-0.313428811	-1.242657581	0.014877157	1.662126199
NM_002217	inter-alpha	ITIH3	-1.488619938	-2.806204091	-0.752374934	-1.684563643	0.001151545	1.665834415
NM_002697	POU domain, class 2, transcription factor 1	POU2F1	-1.026806804	-2.037509525	-0.290271062	-1.222870016	0.013942461	1.666170155
NM_174922	aarF domain containing kinase 5	ADCK5	-1.189511015	-2.280754265	-0.451597163	-1.367553395	0.022113562	1.667762498
NM_016509	C-type lectin domain family 1, member B	CLEC1B	-1.398195207	-2.635716511	-0.655825248	-1.57551692	0.009927535	1.672921742
NM_198129	laminin, alpha 3	LAMA3	-1.196197508	-2.291349465	-0.452222588	-1.368146374	0.037691363	1.67478386
NM_006329	fibulin 5	FBLN5	-1.11720734	-2.169266556	-0.368363131	-1.29088737	0.010678693	1.68044603
NM_001277	choline kinase alpha	CHKA	-1.066072965	-2.093726452	-0.315014257	-1.244023947	0.013680903	1.68302745
NM_002547	oligophrenin 1	OPHN1	-1.341520419	-2.534182491	-0.590420982	-1.505686046	0.033168937	1.683074966
NM_024855	ARP5 actin-related protein 5 homolog	ACTR5	-1.06290819	-2.089138573	-0.309629761	-1.239389594	0.01136627	1.685618939
XR_000285	general transcription factor II, i, pseudogene 1	GTF2IP1	-1.04895432	-2.06902965	-0.290175546	-1.222789056	0.013172016	1.69205771

NM_016202	zinc finger protein 580	ZNF580	-1.245277028	-2.370640711	-0.486454299	-1.40099742	0.022551248	1.692109262
NM_000507	fructose-1,6-bisphosphatase 1	FBP1	-1.167857858	-2.246778431	-0.409032279	-1.327794866	0.000242378	1.692112606
NM_021190	polypyrimidine tract binding protein 2	PTBP2	-1.175813411	-2.259202219	-0.416597594	-1.334775947	0.039945579	1.69257037
NM_006647	NADPH oxidase activator 1	NOXA1	-1.254490805	-2.385829267	-0.491821283	-1.406218991	0.008407829	1.696627113
NM_004768	splicing factor, arginine/serine-rich 11	SFRS11	-1.620072632	-3.073905113	-0.857264642	-1.811600248	0.002494912	1.696789961
NM_138967	secretory carrier membrane protein 5	SCAMP5	-1.013593258	-2.018933312	-0.246048305	-1.185954207	0.003660783	1.702370378
NM_014016	SAC1 suppressor of actin mutations 1-like	SACM1L	-1.154845664	-2.226605027	-0.386611564	-1.307319314	0.00105744	1.703183762
NM_005855	receptor calcitonin	RAMP1	-1.312592274	-2.483874486	-0.542910594	-1.456908824	0.005937739	1.704893569
NM_207310	coiled-coil domain containing 74B	CCDC74B	-1.378841171	-2.600593973	-0.604324087	-1.520266331	0.048020995	1.710617357
NM_021933	hypothetical protein FLJ12438	FLJ12438	-1.254520584	-2.385878513	-0.478521485	-1.393315025	0.007601715	1.7123755
NM_015171	exportin 6	XPO6	-1.514297595	-2.856597149	-0.737650657	-1.66745827	0.002980021	1.713144611
NM_198969	amino-terminal enhancer of split	AES	-1.175107641	-2.258097283	-0.397593281	-1.317308529	0.001567877	1.714174951
NM_012207	heterogeneous nuclear ribonucleoprotein H3	HNRPH3	-1.275059363	-2.420087756	-0.496841476	-1.411120781	0.035957956	1.715011068
NM_003873	neuropilin 1	NRP1	-1.122445576	-2.177157194	-0.339780992	-1.265564461	0.000325137	1.720305257
NM_000123	excision repair cross-complementing rodent repair deficiency, complementation group 5	ERCC5	-1.170799955	-2.251364974	-0.387053084	-1.307719466	0.009920224	1.721596286
NM_213674	tropomyosin 2	TPM2	-1.217549803	-2.325514287	-0.429988598	-1.347222929	0.000190313	1.726154029
NM_138418	hypothetical protein MGC15416	MGC15416	-1.0054795	-2.007610643	-0.217513685	-1.162728034	0.005685558	1.726638204
NM_178502	deltex 3 homolog	DTX3	-1.391012913	-2.622627502	-0.602941816	-1.518810434	0.008363718	1.726764212
NM_017707	up-regulated in liver cancer 1	UPLC1	-1.008058458	-2.011202653	-0.21846295	-1.163493338	0.011934586	1.728589746
NM_018140	hypothetical protein FLJ10565	FLJ10565	-1.526225064	-2.880311938	-0.735493218	-1.664966578	0.026656185	1.729951805
NM_138783	zinc finger protein 653	ZNF653	-1.119780161	-2.173138555	-0.328270318	-1.255507214	0.044764341	1.730884961
XM_375812	KIAA0907 protein	KIAA0907	-1.065583838	-2.093016723	-0.272483492	-1.207885323	0.005194026	1.732794233
NM_021070	latent transforming growth factor beta binding protein 3	LTBP3	-1.315493497	-2.488874521	-0.520930113	-1.434880024	0.000476222	1.734552352
NM_004750	cytokine receptor-like factor 1	CRLF1	-1.320708665	-2.497887782	-0.524867906	-1.438801836	0.012569437	1.73608882
NM_030792	glycerophosphodiester phosphodiesterase domain containing 5	GDPD5	-1.222689959	-2.333814603	-0.425172994	-1.3427335	0.001183283	1.73810708
NM_032870	chromosome 6 open reading frame 111	C6orf111	-1.086490896	-2.123568867	-0.278467153	-1.212905502	0.005504711	1.750811473
NM_002075	guanine nucleotide binding protein	GNB3	-1.395314916	-2.630459642	-0.578651642	-1.4934528	0.007449488	1.761327604
NM_016174	cerebral endothelial cell adhesion molecule 1	CEECAM1	-1.212815325	-2.317895178	-0.394727072	-1.314694024	0.004409601	1.763068163
NM_032905	RNA binding motif protein 17	RBM17	-1.12890044	-2.18691999	-0.299973096	-1.231121455	0.00722073	1.776364129
NM_004058	calcyphosine	CAPS	-1.181314668	-2.267833412	-0.345765649	-1.27082524	0.000804598	1.784536016
NM_015885	pre-mRNA cleavage complex II protein Pcf11	PCF11	-1.45296547	-2.737702101	-0.61710541	-1.533794722	0.001032245	1.784920799
NM_015964	brain specific protein	CGI-38	-1.24238857	-2.365899136	-0.405946645	-1.324958015	0.007411367	1.785640835
XR_012668	CG5645-PA	LOC713047	-1.115232517	-2.166299202	-0.276496461	-1.211249828	0.004743113	1.788482568
NM_024330	solute carrier family 27 fatty acid transporter	SLC27A3	-1.130686184	-2.1896286	-0.291438093	-1.223859625	0.011978144	1.789117441
NM_007139	zinc finger protein 92	ZNF92	-1.325492353	-2.506184024	-0.481033206	-1.395742889	0.011306383	1.795591469
NM_023944	cytochrome P450, family 4, subfamily F, polypeptide 12	CYP4F12	-1.024401477	-2.034115326	-0.17814262	-1.131426306	0.010826708	1.797832802
NM_152743	chromosome 7 open reading frame 27	C7orf27	-1.123040452	-2.178055101	-0.272319306	-1.207747868	0.001016164	1.803402149
NM_030802	C/EBP-induced protein	LOC81558	-1.405560073	-2.649206077	-0.554680958	-1.468843772	0.030963642	1.803599625
NM_017999	ring finger protein 31	RNF31	-1.055925555	-2.079051585	-0.202904157	-1.151013023	0.001254996	1.80627981
XR_013919	pleckstrin homology domain containing, family H with MyTH4 domain	PLEKHH2	-1.242982535	-2.366873391	-0.378097423	-1.299626821	0.008864943	1.821194632
XM_371614	hypothetical protein FLJ10707	FLJ10707	-1.405033927	-2.648240097	-0.535477336	-1.449421636	0.003905312	1.827101259
NM_197960	dipeptidylpeptidase 8	DPP8	-1.260305186	-2.39546409	-0.390667026	-1.3109994	0.000148909	1.827204566
NM_032876	jub, ajuba homolog	JUB	-1.36180702	-2.57006887	-0.489361642	-1.40382358	0.018347187	1.830763429

XR_013922	hypothetical protein LOC718482	LOC718482	-1.223140516	-2.334543573	-0.348332514	-1.273088324	8.66E-05	1.833764028
NM_005595	nuclear factor I/A	NFIA	-1.13274903	-2.192761693	-0.257427786	-1.195345594	0.000379334	1.834416509
NM_013260	transcriptional regulator protein	HCNGP	-1.186760132	-2.276409543	-0.309743103	-1.239486968	6.03E-06	1.836574003
NM_031207	hypothetical protein HT036	HT036	-1.224039689	-2.335999052	-0.345101782	-1.270240594	0.00280747	1.83902094
NM_030915	likely ortholog of mouse limb-bud and heart gene	LBH	-1.362062855	-2.570524664	-0.481757058	-1.39644336	0.001701516	1.840765432
NM_000156	guanidinoacetate N-methyltransferase	GAMT	-1.015334423	-2.021371402	-0.128957883	-1.093503534	0.002579813	1.848527543
NM_007286	synaptopodin	SYNPO	-1.124252933	-2.179886368	-0.236786376	-1.178364911	0.036805415	1.849924711
NM_004424	E4F transcription factor 1	E4F1	-1.366963273	-2.579270839	-0.479357152	-1.394122324	0.01328808	1.850103679
NM_012469	chromosome 20 open reading frame 14	C20orf14	-1.215951131	-2.322938776	-0.327720724	-1.25502902	1.88E-06	1.850904433
NM_032709	chromosome 10 open reading frame 33	C10orf33	-1.314778282	-2.487640968	-0.425632445	-1.343161186	0.001307783	1.852079255
NM_198464	tryptophan/serine protease	UNQ9391	-1.126676375	-2.183551222	-0.233438731	-1.175633791	0.010071008	1.857339624
NM_000704	ATPase, H+/K+ exchanging, alpha polypeptide	ATP4A	-1.282425741	-2.432476288	-0.388801	-1.30930481	0.000189995	1.857838044
CN644139	ILLUMIGEN_MCQ_9651 Katze_MMBR cDNA clone IBIUW:8946 5' Bases 3 to 316 highly human RAMP1	Hs.32989	-1.135819777	-2.197433912	-0.241727088	-1.182407306	0.006021954	1.858440744
NM_004396	DEAD Asp-Glu-Ala-Asp	DDX5	-1.548186866	-2.924493669	-0.653767612	-1.573271449	0.001995918	1.858861464
NM_018719	transcription factor RAM2	RAM2	-1.064897965	-2.092021916	-0.170120758	-1.125152659	0.000212801	1.859322731
NM_000977	ribosomal protein L13	RPL13	-1.044578452	-2.062763541	-0.143919493	-1.104902826	0.00025807	1.866918513
NM_031477	yippee-like 3	YPEL3	-1.129985606	-2.188565566	-0.228489566	-1.171607688	0.005595297	1.868002052
NM_201627	tripartite motif-containing 41	TRIM41	-1.04944419	-2.069732313	-0.146131963	-1.106598569	0.000234039	1.870355133
NM_006225	phospholipase C, delta 1	PLCD1	-1.104905519	-2.150847919	-0.200065108	-1.148750196	9.05E-05	1.872337369
NM_003586	double C2-like domains, alpha	DOC2A	-1.107261076	-2.15436258	-0.199284685	-1.14812895	0.032076883	1.876411687
XR_014480	tubulin, gamma complex associated protein 6	TUBGCP6	-1.289588922	-2.444583903	-0.381435132	-1.30263702	0.000568414	1.876642431
NM_005520	heterogeneous nuclear ribonucleoprotein H1	HNRPH1	-1.056006309	-2.079167963	-0.147491082	-1.107641553	0.000120734	1.877112643
NM_018142	hypothetical protein FLJ10569	FLJ10569	-1.124546069	-2.180329337	-0.215157598	-1.160830715	0.001178217	1.878249178
NM_145260	odd-skipped homolog	ODD	-1.286351001	-2.439103539	-0.376152253	-1.29787573	0.044474659	1.879304377
NM_021168	RAB40C, member RAS oncogene family	RAB40C	-1.129894427	-2.188427253	-0.217036553	-1.162343557	0.000823763	1.88277144
XM_113678	nucleoporin 160kDa	NUP160	-1.25025629	-2.378836785	-0.335046279	-1.261417872	0.015787861	1.885843572
NM_015115	KIAA0276 protein	KIAA0276	-1.01559491	-2.021736405	-0.099175587	-1.071161184	9.15E-05	1.887425007
NM_015896	zinc finger, MYND-type containing 10	ZMYND10	-1.123688678	-2.179033955	-0.206857495	-1.154171407	0.011624618	1.887963904
NM_182774	hypothetical protein LOC259173	FLJ36525	-1.598324178	-3.027913891	-0.681054018	-1.60331069	0.008162679	1.888538454
NM_016223	protein kinase C and casein kinase substrate in neurons 3	PACSIN3	-1.068671596	-2.097501141	-0.146047673	-1.106533917	0.001119478	1.895559738
NM_003674	cyclin-dependent kinase	CDK10	-1.141415478	-2.205973527	-0.2181047	-1.163204455	0.003340085	1.896462412
NM_015555	zinc finger protein 451	ZNF451	-1.093781086	-2.13432679	-0.168056324	-1.123543766	0.001372174	1.899638318
NM_005842	sprouty homolog 2	SPRY2	-1.073530002	-2.104576573	-0.144100313	-1.105041318	0.012930532	1.904522971
NM_014234	hydroxysteroid 17-beta	HSD17B8	-1.04153134	-2.058411383	-0.111892781	-1.080645087	0.006125289	1.904798724
NM_016166	protein inhibitor of activated STAT, 1	PIAS1	-1.204533451	-2.304627286	-0.273756129	-1.208951299	6.52E-05	1.906302833
NM_015186	vacuolar protein sorting 13A	VPS13A	-1.526127274	-2.880116708	-0.591783956	-1.507109203	0.046612275	1.911020584
NM_152299	hypothetical protein 384D8_6	384D8-2	-1.124941967	-2.180927734	-0.190528818	-1.141181938	0.00230655	1.911113085
NM_024657	zinc finger, CW-type with coiled-coil domain 2	ZCWCC2	-1.081775745	-2.116639751	-0.146983336	-1.107251795	0.002348696	1.911615552
NM_016143	NSFL1 p97	NSFL1C	-1.206511196	-2.307788793	-0.270656604	-1.206356744	0.04802857	1.913023494
NM_004628	xeroderma pigmentosum, complementation group C	XPC	-1.198140483	-2.29443746	-0.257425526	-1.195343722	0.003198959	1.919479241
NM_002963	S100 calcium binding protein A7	S100A7	-1.100843667	-2.144800807	-0.158618985	-1.116218131	0.034749002	1.921488953
XR_010727	hypothetical protein LOC696654	LOC696654	-1.129339523	-2.18758568	-0.184409755	-1.136351966	0.003877846	1.925095168
XR_013095	phosphatidylethanolamine-binding protein 4	LOC709242	-1.073083427	-2.103925219	-0.12796251	-1.092749343	9.01E-05	1.925350249
NM_138769	ras homolog gene family, member T2	RHOT2	-1.1120925	-2.161589392	-0.160863113	-1.117955771	0.002666521	1.933519596
NM_004603	syntaxin 1A	STX1A	-1.104944279	-2.150905705	-0.145812198	-1.106353325	0.02479696	1.944139956

NM_018152	chromosome 20 open reading frame 12	C20orf12	-1.411778061	-2.660648747	-0.449029543	-1.365121672	0.027806363	1.949019491
NM_003202	transcription factor 7 T-cell specific, HMG-box	TCF7	-1.29894754	-2.460493218	-0.333404328	-1.259983052	0.030537991	1.952798661
XM_371246	hypothetical protein FLJ21156	FLJ21156	-1.120582421	-2.17434734	-0.15438905	-1.112950208	0.001421896	1.953678901
NM_001606	ATP-binding cassette, sub-family A	ABCA2	-1.543720333	-2.915453541	-0.576748803	-1.491484312	0.01448751	1.954732957
NM_001979	epoxide hydrolase 2, cytoplasmic	EPHX2	-1.249263443	-2.377200258	-0.279830502	-1.214052241	0.000699383	1.958070813
NM_002526	5'-nucleotidase, ecto	NT5E	-1.24605014	-2.371911432	-0.275645863	-1.210535898	0.004699199	1.959389585
XR_010398	histidyl-tRNA synthetase-like	HARSL	-1.1945174	-2.288682599	-0.222789207	-1.16698758	0.002195583	1.961188481
NM_014780	cullin 7	CUL7	-1.22939494	-2.344686342	-0.256238492	-1.19436061	0.000289061	1.963131002
NM_000155	galactose-1-phosphate uridylyltransferase	GALT	-1.326911329	-2.508650218	-0.352548223	-1.276813865	0.001623807	1.964773635
NM_002518	neuronal PAS domain protein 2	NPAS2	-1.074501574	-2.105994362	-0.09775173	-1.070104531	0.001224248	1.968026769
NM_022760	chromosome 20 open reading frame 81	C20orf81	-1.148852676	-2.217374843	-0.169673644	-1.124804011	0.00092484	1.971343294
NM_020225	storkhead box 2	STOX2	-1.092883046	-2.13299864	-0.112653697	-1.081215199	0.003285672	1.972779001
NM_173501	chromosome 16 open reading frame 52	C16orf52	-1.184612014	-2.273022577	-0.20343706	-1.151438263	0.044055968	1.97407247
NM_001011655	transmembrane protein 44	TMEM44	-1.232124899	-2.349127306	-0.248944983	-1.188337787	0.002540853	1.976817814
NM_014738	KIAA0195 gene product	KIAA0195	-1.116081069	-2.167573732	-0.130815265	-1.09491226	0.002747096	1.979678018
NM_005938	myeloid/lymphoid or mixed-lineage leukemia	MLLT7	-1.136585713	-2.198600854	-0.14954864	-1.109222388	0.003640593	1.98211006
NM_001692	ATPase, H+ transporting, lysosomal 56/58kDa, V1 subunit B, isoform 1	ATP6V1B1	-1.38274721	-2.607644517	-0.39270351	-1.312851292	0.021856763	1.986245154
NM_015949	chromosome 7 open reading frame 20	C7orf20	-1.284684373	-2.436287467	-0.290338647	-1.222927305	0.004320659	1.992176851
NM_007320	RAN binding protein 3	RANBP3	-1.07769242	-2.110657398	-0.080167629	-1.057140864	0.002171941	1.996571573
NM_003070	SWI/SNF related, matrix associated, actin dependent regulator of chromatin, subfamily a, member 2	SMARCA2	-1.147393424	-2.215133155	-0.149015526	-1.108812576	0.004331203	1.997752553
NM_130781	RAB24, member RAS oncogene family	RAB24	-1.051625186	-2.072863599	-0.052692545	-1.037198874	0.000131918	1.998520873
NM_014624	S100 calcium binding protein A6	S100A6	-1.084979966	-2.121346024	-0.083845713	-1.059839435	3.32E-05	2.001573026
NM_021830	progressive external ophthalmoplegia 1	PEO1	-1.091284159	-2.130636025	-0.088869127	-1.06353619	0.00276999	2.00335075
NM_153482	interleukin 17 receptor E	IL17RE	-1.05733054	-2.08107728	-0.049300581	-1.034763148	0.000535834	2.011162925
NM_206839	mortality factor 4 like 1	MORF4L1	-1.01848265	-2.025787223	-0.008935043	-1.006212518	0.004907873	2.013279687
NM_182983	hepsin transmembrane protease, serine 1	HPN	-1.12784035	-2.185313636	-0.117894868	-1.085150292	0.000204388	2.013834999
NM_138802	hypothetical protein BC018415	LOC130617	-1.221977565	-2.332662464	-0.210124651	-1.156788128	0.000151489	2.016499312
NM_003437	zinc finger protein 136	ZNF136	-1.285866533	-2.438284606	-0.272875227	-1.208213346	0.000435521	2.018091105
NM_024315	chromosome 7 open reading frame 23	C7orf23	-1.167463287	-2.24616403	-0.152879281	-1.111786124	0.000641721	2.020320259
NM_018262	WD repeat domain 10	WDR10	-1.070057034	-2.099516366	-0.054706894	-1.038648065	0.000228442	2.021393423
NM_005096	zinc finger protein 261	ZNF261	-1.101278244	-2.145446973	-0.08424485	-1.060132691	0.002091841	2.023753245
NM_004643	poly A	PABPN1	-1.16498978	-2.242316272	-0.147852884	-1.107919364	0.002162699	2.023898439
NM_178453	hypothetical protein MGC52282	MGC52282	-1.512434319	-2.852910165	-0.492352852	-1.406737215	0.045198094	2.028033476
XM_290517	KIAA0404 protein	KIAA0404	-1.225455679	-2.338292936	-0.205291482	-1.152919259	3.42E-05	2.028149775
NM_005688	ATP-binding cassette, sub-family C	ABCC5	-1.152866695	-2.223552849	-0.13204413	-1.095845287	0.010878827	2.029075524
NM_001031	ribosomal protein S28	RPS28	-1.125075178	-2.18112912	-0.100332233	-1.072020306	0.001749014	2.034596834
NM_025176	KIAA0980 protein	KIAA0980	-1.738600315	-3.337112482	-0.712654458	-1.638816653	0.012472585	2.036293978
NM_005550	kinesin family member C3	KIFC3	-1.064921553	-2.09205612	-0.034471266	-1.024181402	0.017144226	2.042661697
BC067766	MAD, mothers against decapentaplegic homolog 9	SMAD9	-1.327883644	-2.51034151	-0.295507111	-1.227316307	0.022308229	2.045390823
NM_170707	lamin A/C	LMNA	-1.310097738	-2.479583379	-0.27745864	-1.212057918	0.000788107	2.045763111
CN647337	Sin3A-associated protein, 18kDa	SAP18	-1.396508699	-2.632637164	-0.363779571	-1.286792624	0.016257887	2.045890779
NM_172027	ankyrin repeat and BTB	ABTB1	-1.57918007	-2.987999839	-0.543570879	-1.457575767	0.000124704	2.049979086
NM_033450	ATP-binding cassette, sub-family C	ABCC10	-1.437185757	-2.707921199	-0.400039234	-1.319543795	0.00254932	2.052164701
NM_198401	hypothetical protein LOC157567	LOC157567	-1.082679312	-2.117965827	-0.043139782	-1.030353779	0.025382767	2.055571465

NM_178837	hypothetical testis protein from macaque	LOC352909	-1.288882044	-2.443386422	-0.248558792	-1.188019727	0.01960361	2.056688426
NM_144679	hypothetical protein FLJ31528	FLJ31528	-1.406270626	-2.650511181	-0.363539075	-1.286578134	0.001233709	2.060124535
NM_181870	dishevelled, dsh homolog 1	DVL1	-1.070524199	-2.100196329	-0.027683048	-1.019373707	0.000569493	2.060281047
NM_149379	Williams Beuren syndrome chromosome region 20C	WBSCR20C	-1.278052476	-2.425113847	-0.23509516	-1.176984368	0.011825867	2.060446946
NM_014232	vesicle-associated membrane protein 2 synaptobrevin 2	VAMP2	-1.663658555	-3.168189334	-0.618924976	-1.535730406	0.000369099	2.062985354
NM_005072	solute carrier family 12 potassium/chloride transporters	SLC12A4	-1.057137932	-2.080799463	-0.0119023	-1.008284171	0.001345235	2.063703391
NM_002628	profilin 2	PFN2	-1.276096188	-2.421827631	-0.228596666	-1.171694667	0.014781229	2.066944315
NM_052902	serine/threonine kinase 11 interacting protein	STK11IP	-1.226888095	-2.340615723	-0.178152746	-1.131434248	0.002561173	2.068715639
NM_021167	GATA zinc finger domain containing 1	GATAD1	-1.334383281	-2.521676628	-0.284801879	-1.218242955	0.003247099	2.06992917
NM_014577	bromodomain containing 1	BRD1	-1.061221068	-2.086696913	-0.009759693	-1.006787837	0.002370699	2.07262825
NM_017810	zinc finger protein 434	ZNF434	-1.16982585	-2.249845371	-0.117605198	-1.084932433	0.026422941	2.073719342
NM_005052	ras-related C3 botulinum toxin substrate 3 rho family, small GTP binding protein Rac3	RAC3	-1.307360335	-2.474883019	-0.249947009	-1.189163436	0.027264463	2.081196701
NM_019052	coiled-coil alpha-helical rod protein 1	CCHCR1	-1.289026679	-2.443631392	-0.224387356	-1.16828103	0.007816764	2.091646898
NM_006454	MAX dimerization protein 4	MXD4	-1.27024407	-2.412023678	-0.202322377	-1.15054896	0.000259073	2.096411159
NM_002319	leucine-rich repeats and calponin homology	LRCH4	-1.686820712	-3.219464433	-0.618660421	-1.535448816	0.000716906	2.096757898
NM_013326	chromosome 18 open reading frame 8	C18orf8	-1.192725393	-2.285841535	-0.121307523	-1.087720223	0.012206245	2.101497689
NM_144626	hypothetical protein MGC17299	MGC17299	-1.136838378	-2.198985937	-0.06059938	-1.042898952	7.72E-05	2.108532119
NM_000355	transcobalamin II; macrocytic anemia	TCN2	-1.135002555	-2.196189518	-0.057447087	-1.040622702	0.004408731	2.110457049
NM_006615	calpain 9	CAPN9	-1.820681785	-3.532480961	-0.739967571	-1.670138297	0.014704715	2.115082906
NM_018347	chromosome 20 open reading frame 29	C20orf29	-1.091865465	-2.131494696	-0.008830009	-1.006139265	0.01119806	2.118488733
NM_001945	heparin-binding EGF-like growth factor	HBEGF	-1.229018815	-2.344075138	-0.145266838	-1.105935186	0.007106684	2.119541151
NM_145284	hypothetical protein MGC17347	LOC159090	-1.304169547	-2.469415405	-0.220230552	-1.164919734	0.005471315	2.119815927
NM_032179	cleavage and polyadenylation specific factor 3-like	CPSF3L	-1.290192983	-2.445607672	-0.204176991	-1.152028966	0.000209858	2.122869949
NM_001524	hypocretin	HCRT	-1.301169925	-2.464286381	-0.214364926	-1.160193085	0.026199684	2.124031261
NM_020944	glucosidase, beta	GBA2	-1.174654313	-2.257387847	-0.087697084	-1.062672527	0.005600325	2.124255395
NM_207514	hypothetical protein FLJ20186	FLJ20186	-1.17186316	-2.253024747	-0.084698987	-1.060466456	6.04E-05	2.124560126
NM_020796	sema domain, transmembrane domain	SEMA6A	-1.501630002	-2.831624576	-0.413791499	-1.332182282	0.009394534	2.125553398
NM_005650	transcription factor 20 AR1	TCF20	-1.28852983	-2.442789975	-0.199402423	-1.148222652	9.20E-05	2.127453217
NM_007182	Ras association RalGDS/AF-6	RASSF1	-1.228015196	-2.342445036	-0.138813607	-1.100999342	3.82E-06	2.127562611
NM_005920	MADS box transcription enhancer factor 2, polypeptide D	MEF2D	-1.318108432	-2.493389784	-0.22615262	-1.169711399	0.009186159	2.131628182
NM_024316	leukocyte receptor cluster	LENG1	-1.111514429	-2.160723443	-0.019376815	-1.013521586	0.001799474	2.131896817
XR_014196	pleckstrin homology domain interacting protein	LOC716810	-1.244868649	-2.369969756	-0.152163464	-1.11123463	0.01246668	2.132735691
NM_005919	MADS box transcription enhancer factor 2, polypeptide B	MEF2B	-1.605851985	-3.043754468	-0.512699038	-1.426716846	0.00118015	2.133397722
NM_176878	InaD-like	INADL	-2.109771342	-4.316228798	-1.013955977	-2.019440973	0.005074566	2.13733843
NM_016162	inhibitor of growth family, member 4	ING4	-1.227112748	-2.340980226	-0.130897122	-1.094974386	0.000449274	2.137931495
NM_000304	peripheral myelin protein 22	PMP22	-1.158445176	-2.232167323	-0.060743539	-1.043003167	0.001985256	2.140134751
NM_002805	proteasome prosome, macropain	PSMC5	-1.197623535	-2.293615462	-0.098904795	-1.070960147	0.000265525	2.141644082
XR_010913	calcineurin binding protein 1	CABIN1	-1.369160636	-2.583202309	-0.266876609	-1.203200114	0.001502771	2.146943205
BC043401		MGC2752	-1.828771922	-3.552345542	-0.724946392	-1.652839218	0.019156171	2.149238415
NM_014371	A kinase	AKAP8L	-1.690265747	-3.227161432	-0.586030442	-1.501110772	5.01E-05	2.14984896
NM_002773	protease, serine, 8	PRSS8	-1.310854413	-2.480884229	-0.206014975	-1.153497578	0.000146526	2.150749404
NM_052818	hypothetical gene CG018	CG018	-1.722269455	-3.299550401	-0.617293591	-1.533994798	0.003339013	2.150952797
NM_014270	solute carrier family 7 cationic amino acid transporter, y+ system	SLC7A9	-1.171106197	-2.251842924	-0.065449985	-1.046411271	0.019783315	2.151967383
NM_003019	surfactant, pulmonary-associated protein D	SFTPD	-1.280649049	-2.429482514	-0.173082026	-1.127464514	1.76E-05	2.154819495

NM_005407	sal-like 2	SALL2	-1.200751563	-2.298593836	-0.091291351	-1.065323323	0.013477741	2.157649032
NM_014779	KIAA0669 gene product	KIAA0669	-1.294046139	-2.452148141	-0.18449572	-1.136419679	0.002698548	2.157783947
NM_002292	laminin, beta 2	LAMB2	-1.479134574	-2.787814511	-0.368423502	-1.290941389	9.40E-05	2.15952059
NM_012477	WW domain binding protein 1	WBP1	-1.687958975	-3.22200554	-0.576972351	-1.491715438	0.001368576	2.159933094
NM_018282	paraspeckle component 1	PSPC1	-1.133671391	-2.194164044	-0.022188542	-1.015498805	0.000390126	2.160676146
AK125739	cDNA FLJ43751 fis, clone TESTI2034953, moderately 88-kDa Golgi protein	GM88	-1.335226713	-2.523151288	-0.217274681	-1.162535427	0.008565586	2.170386579
NM_198531	ATPase, Class II, type 9B	ATP9B	-1.286043047	-2.43858295	-0.166623026	-1.122428095	0.000122601	2.172596143
NM_014519	zinc finger protein 232	ZNF232	-1.139699126	-2.203350675	-0.012985346	-1.009041385	0.016165034	2.183607836
NM_005859	purine-rich element binding protein A	PURA	-1.150497572	-2.219904436	-0.023710395	-1.016570588	0.001511158	2.18371893
NM_019003	spindlin family, member 2	SPIN2	-1.780765562	-3.436084611	-0.652838484	-1.572258553	0.011888996	2.185445011
NM_022822	likely ortholog of kinesin light chain 2	KLC2	-1.257680533	-2.391110053	-0.128540816	-1.09318746	0.000307631	2.18728273
NM_002864	pregnancy-zone protein	PZP	-1.278241679	-2.42543191	-0.149020862	-1.108816677	0.000299092	2.187405691
NM_005341	GLI-Kruppel family member HKR3	HKR3	-1.238768467	-2.359969914	-0.107068354	-1.077037404	0.002769107	2.191168018
NM_017607	protein phosphatase 1, regulatory inhibitor	PPP1R12C	-1.181224659	-2.267691927	-0.047929126	-1.033779949	0.000396363	2.193592483
CN803406	insulin-like growth factor binding protein 6	IGFBP6	-1.46900219	-2.768303634	-0.331978924	-1.258738785	0.000162684	2.199267765
NM_199002	Rho guanine nucleotide exchange factor	ARHGEF1	-1.323278459	-2.502341098	-0.184797537	-1.136657447	0.00288577	2.201490963
NM_007020	U11/U12 snRNP 35K	U1SNRNPBP	-1.260679274	-2.39608531	-0.121886217	-1.088156617	5.27E-05	2.20196732
NM_000756	corticotropin releasing hormone	CRH	-1.7861522	-3.448938013	-0.644743843	-1.563461661	0.013603333	2.205962639
NM_004510	SP110 nuclear body protein	SP110	-1.184025551	-2.272098769	-0.041542574	-1.029213705	0.000661346	2.207606406
NM_000853	glutathione S-transferase theta 1	GSTT1	-1.553662648	-2.935614744	-0.410822133	-1.329443194	0.000596775	2.208153578
NM_003906	MCM3 minichromosome maintenance deficient 3	MCM3AP	-1.147840043	-2.215819005	-0.004245741	-1.002947258	0.001939141	2.209307605
NM_018069	centrosomal protein 192 kDa	Cep192	-1.480796845	-2.791028479	-0.335013018	-1.261388791	0.020934366	2.212663137
XM_496394	KIAA1693 protein	LOC440673	-1.264141035	-2.401841643	-0.11487874	-1.082884024	0.007869055	2.218004505
NM_019012	pleckstrin homology domain containing, family A member 5	PLEKHA5	-1.48694905	-2.802955908	-0.335857663	-1.262127504	0.000784088	2.22081834
NM_014494	trinucleotide repeat containing 6	TNRC6	-1.466608845	-2.763714992	-0.314494741	-1.243576054	0.000229667	2.222393221
NM_032329	inhibitor of growth family, member 5	ING5	-1.495725017	-2.820058356	-0.343120335	-1.2684972	0.001893548	2.223149058
NM_001481	growth arrest-specific 8	GAS8	-1.534220862	-2.896319708	-0.378322848	-1.299829907	0.008099589	2.22822978
NM_005131	THO complex 1	THOC1	-1.367551992	-2.580323574	-0.210259899	-1.156896578	0.013737669	2.230383963
NM_006924	splicing factor, arginine/serine-rich 1	SFRS1	-1.182182908	-2.269198645	-0.023307539	-1.016286762	5.84E-05	2.232833026
NM_000458	transcription factor 2, hepatic; LF-B3; variant hepatic nuclear factor	TCF2	-1.508907167	-2.845943788	-0.349734319	-1.274325931	0.001249322	2.233293477
NM_018467	uncharacterized hematopoietic stem/progenitor cells protein MDS032	MDS032	-1.298358698	-2.459489162	-0.138099682	-1.100454642	3.68E-05	2.234975499
NM_013293	transformer-2 alpha	TRA2A	-1.323245282	-2.502283553	-0.159359246	-1.11679102	0.000501449	2.240601427
NM_144689	zinc finger protein 420	ZNF420	-1.285181012	-2.437126289	-0.118582484	-1.085667619	0.002125401	2.244818071
NM_031421	tetratricopeptide repeat domain 25	TTC25	-1.582895828	-2.995705551	-0.410004782	-1.328690218	0.021587687	2.254630546
NM_000107	damage-specific DNA binding protein 2, 48kDa	DDB2	-1.180590427	-2.266695232	-0.006951848	-1.004830282	5.06E-05	2.255799085
NM_005676	RNA binding motif protein 10	RBM10	-1.323601965	-2.502902279	-0.149801349	-1.109416701	4.07E-05	2.256052461
NM_024056	hypothetical protein MGC5576	MGC5576	-1.599966836	-3.031363448	-0.423899348	-1.341548627	0.001772363	2.259600128
NM_032119	monogenic, audiogenic seizure susceptibility 1 homolog	MASS1	-1.434477538	-2.702842674	-0.257565013	-1.1954593	0.013939946	2.260924043
NM_007078	LIM domain binding 3	LDB3	-1.289659352	-2.444703246	-0.110895214	-1.079898122	0.003140089	2.263827667
NM_144999	hypothetical protein MGC20806	MGC20806	-1.366585709	-2.578595913	-0.187684467	-1.138934252	0.001005689	2.264042817
NM_003195	transcription elongation factor A	TCEA2	-1.18378469	-2.271719469	-0.004796908	-1.003330497	0.002450627	2.264178629
NM_017922	PRP39 pre-mRNA processing factor 39 homolog	PRPF39	-1.236921969	-2.356951332	-0.055009469	-1.038865922	0.008585675	2.268773364

NM_139067	SWI/SNF related, matrix associated, actin dependent regulator of chromatin, subfamily c, member 2	SMARCC2	-1.373129912	-2.590319241	-0.190638632	-1.141268805	2.93E-06	2.269683733
NM_144697	hypothetical protein BC017397	LOC148523	-1.391235975	-2.623033031	-0.208393562	-1.155400931	0.001242655	2.270236211
XR_011718	leucine rich repeat and sterile alpha motif containing 1	LOC700704	-1.546381881	-2.920837064	-0.362952534	-1.286055171	0.001902143	2.271160002
NM_012240	sirtuin silent mating type information regulation 2 homolog	SIRT4	-1.215370134	-2.322003479	-0.030823155	-1.021594848	0.009306698	2.272920114
NM_003611	oral-facial-digital syndrome 1	OFD1	-1.236937937	-2.35697742	-0.0520202	-1.036715617	8.72E-05	2.273504307
XR_011805	mitochondrial tumor suppressor 1 isoform 1	LOC702990	-1.370133352	-2.584944583	-0.183773464	-1.135850896	0.000312483	2.275778089
NM_012139	deafness locus associated putative guanine nucleotide exchange factor	DELGEF	-1.530910331	-2.889681186	-0.342906933	-1.268309579	0.000786723	2.278372121
NM_015205	ATPase, Class VI, type 11A	ATP11A	-1.259952055	-2.39487782	-0.070715959	-1.050237751	0.007096724	2.280319688
NM_024112	chromosome 9 open reading frame 16	C9orf16	-1.247695005	-2.37461727	-0.054613515	-1.03858084	0.002851732	2.286405814
NM_016262	tubulin, epsilon 1	TUBE1	-1.345610213	-2.541376657	-0.152078544	-1.111169222	0.017103395	2.287119376
NM_003565	unc-51-like kinase 1	ULK1	-1.235007768	-2.353826148	-0.040783378	-1.02867224	0.000266627	2.288217817
NM_145059	fucokinase	FUK	-1.651486262	-3.141571166	-0.455594778	-1.371348052	0.00026295	2.290863477
NM_201523	kinesin-like 8	KNSL8	-1.48017937	-2.789834171	-0.281753377	-1.215671451	3.08E-05	2.294891574
NM_002599	phosphodiesterase 2A, cGMP-stimulated	PDE2A	-1.2790063	-2.426717717	-0.078446615	-1.055880536	0.000111948	2.298288144
XR_010253	phosphatidylserine synthase 2	LOC698582	-1.205521764	-2.306206604	-8.73E-05	-1.000060503	0.007445875	2.306067081
NM_015113	zinc finger, ZZ-type with EF hand domain 1	ZZEF1	-1.475206685	-2.780234716	-0.269517099	-1.205404286	0.005130885	2.306474889
NM_144982	hypothetical protein MGC23401	MGC23401	-1.643782946	-3.124841352	-0.433834943	-1.35081953	0.000905683	2.313292991
NM_014786	Rho guanine nucleotide exchange factor	ARHGEF17	-1.565310443	-2.959411765	-0.354811747	-1.278818701	0.000843807	2.314176171
CN803179	ILLUMIGEN_MCQ_32437 Katze_MMBR cDNA clone IBIUW:12515 5' Bases 196 to 864 highly human BEX2	Hs.398989	-1.387188209	-2.615683907	-0.174569162	-1.128627308	0.001040158	2.317579851
NM_014994	mouse mitogen-activated protein kinase binding protein 1-like	MAPKBP1	-1.438419003	-2.710236977	-0.224182268	-1.168114963	0.005831607	2.320180002
NM_004484	glypican 3	GPC3	-1.502932972	-2.834183114	-0.288371849	-1.221261248	0.001482774	2.320701749
NM_003940	ubiquitin specific protease 13 isopeptidase T-3	USP13	-1.219418245	-2.328528023	-0.00472143	-1.003278007	0.001791133	2.320920032
NM_003922	hect homologous to the E6-AP	HERC1	-1.711882505	-3.275879988	-0.495979881	-1.410278293	0.00361237	2.322860675
NM_145755	tetratricopeptide repeat domain 21A	TTC21A	-1.677734133	-3.199250884	-0.456669964	-1.372370447	0.000897967	2.331186081
NM_001009881	zinc finger, CCHC domain containing 11	ZCCHC11	-1.249209556	-2.377111467	-0.028040377	-1.019626219	0.008916238	2.331355768
NM_181805	protein kinase cAMP-dependent, catalytic	PKIG	-1.304982658	-2.470807573	-0.083775558	-1.059787898	0.004040546	2.331417048
NM_004170	solute carrier family 1 neuronal/epithelial high affinity glutamate transporter, system Xag	SLC1A1	-1.512835769	-2.853704137	-0.288523634	-1.221389742	0.002518117	2.336440236
NM_002969	mitogen-activated protein kinase 12	MAPK12	-1.287851506	-2.441641699	-0.061689749	-1.043687458	0.000744359	2.339437615
NM_152360	zinc finger protein 573	ZNF573	-1.243043383	-2.36697322	-0.010677311	-1.007428402	0.004302374	2.349520039
NM_006676	ubiquitin specific protease 20	USP20	-1.251752077	-2.381304443	-0.018244336	-1.012726309	0.000460421	2.351380054
NM_003259	intercellular adhesion molecule 5, telencephalin	ICAM5	-1.31416377	-2.48658159	-0.080444683	-1.057343896	0.006295081	2.351724543
NM_024726	hypothetical protein FLJ22527	FLJ22527	-1.59721639	-3.025589768	-0.361282649	-1.284567453	0.018301797	2.355337403
NM_021932	likely ortholog of mouse synembryn	RIC8	-1.405711097	-2.649483416	-0.167528595	-1.123132856	0.000269336	2.359011582
NM_014466	tektin 2	TEKT2	-1.366616703	-2.578651311	-0.127940268	-1.092732496	0.01093597	2.359819371
NM_152856	RNA binding motif protein 10	RBM10	-1.292775166	-2.44998882	-0.053517409	-1.037792064	0.00018254	2.360770432
NM_015385	sorbin and SH3 domain containing 1	SORBS1	-1.564007011	-2.956739231	-0.322083597	-1.25013474	0.039392984	2.365136442
NM_004787	slit homolog 2	SLIT2	-1.365355839	-2.576398646	-0.122544544	-1.088653275	0.007411991	2.366592472
NM_017664	ankyrin repeat domain 10	ANKRD10	-1.591012524	-3.012607086	-0.348167191	-1.272942446	0.000334448	2.366648308
NM_025145	chromosome 10 open reading frame 79	C10orf79	-1.280322231	-2.428932219	-0.031405695	-1.022007437	0.010589609	2.376628711
NM_198278	hypothetical protein LOC255743	LOC255743	-1.637446274	-3.111146377	-0.388180522	-1.308741822	0.0012184	2.377204063
NM_017807	O-sialoglycoprotein endopeptidase	OSGEP	-1.333215864	-2.519636933	-0.083147076	-1.059326322	0.036562389	2.378527636

NM_015343	dullard homolog	DULLARD	-1.493482776	-2.815678818	-0.243126811	-1.18355505	0.002878351	2.379001144
NM_052897	methyl-CpG binding domain protein 6	MBD6	-1.365189008	-2.576100733	-0.114721264	-1.08276583	5.33E-05	2.379185474
XR_014164	prohibitin	LOC719376	-1.405563395	-2.649212178	-0.15416623	-1.112778329	0.046198811	2.3807187
NM_032328	hypothetical protein MGC12458	MGC12458	-1.442637859	-2.71817409	-0.189671173	-1.140503737	3.81E-05	2.383310114
XR_012588	myosin, heavy polypeptide 7B, cardiac muscle, beta	LOC712230	-1.611353254	-3.055383037	-0.358351119	-1.281959886	0.000299274	2.383368677
NM_003673	titin-cap	TCAP	-1.323880445	-2.503385456	-0.070337575	-1.049962334	0.00020329	2.384262153
NM_182571	hypothetical protein 284297	FLJ35258	-1.654992687	-3.149215944	-0.401028576	-1.320448995	0.011525701	2.384958416
NM_004830	cofactor required for Sp1 transcriptional activation, subunit 3, 130kDa	CRSP3	-1.390631371	-2.621934002	-0.13617377	-1.09898658	0.007397434	2.385774357
NM_017519	AT rich interactive domain 1B	ARID1B	-1.392659062	-2.62562169	-0.137946656	-1.100337923	0.002325386	2.386195763
NM_002687	pinin, desmosome associated protein	PNN	-1.663955836	-3.168842238	-0.408156959	-1.326989503	0.000452167	2.387993446
NM_152271	hypothetical protein FLJ23749	FLJ23749	-1.304889472	-2.470647985	-0.048063635	-1.033876338	0.000511416	2.389693907
NM_024517	PHD finger protein 2	PHF2	-1.415572334	-2.667655434	-0.155236203	-1.113603926	0.014086473	2.395515471
NM_001812	centromere protein C 1	CENPC1	-1.843086171	-3.587766926	-0.576135745	-1.490850656	0.004518612	2.406523357
NM_000481	aminomethyltransferase	AMT	-1.78976459	-3.457584693	-0.521500196	-1.435447131	0.000381979	2.408716153
NM_030818	hypothetical protein MGC10471	MGC10471	-1.489630368	-2.808170179	-0.220085592	-1.16480269	1.08E-05	2.41085482
NM_001703	brain-specific angiogenesis inhibitor 2	BAI2	-1.482099009	-2.793548774	-0.209350171	-1.156167297	0.007040478	2.416215006
NM_001299	calponin 1, basic, smooth muscle	CNN1	-1.529206985	-2.88627144	-0.255027743	-1.193358691	0.00188852	2.418611823
NM_172171	calcium/calmodulin-dependent protein kinase	CAMK2G	-1.701001184	-3.251265077	-0.42203416	-1.339815326	0.003843101	2.426651654
NM_023028	fibroblast growth factor receptor 2	FGFR2	-1.282760552	-2.433040865	-0.00192741	-1.001336872	0.003815147	2.429792545
NM_020770	cingulin	CGN	-1.374034159	-2.5919433	-0.092074457	-1.065901745	0.000411532	2.431690642
NM_018463	uncharacterized hematopoietic stem/progenitor cells protein MDS028	MDS028	-1.656990034	-3.153578912	-0.372454528	-1.294553446	0.001993626	2.436036089
NM_005741	zinc finger protein 263	ZNF263	-1.593352619	-3.01749759	-0.308491756	-1.238412344	0.000118102	2.436585525
NM_006696	bromodomain containing 8	BRD8	-1.42606692	-2.687131492	-0.141058205	-1.10271365	0.000176961	2.436835248
NM_198149	chromosome 1 open reading frame 40	C1orf40	-1.389873371	-2.620556784	-0.095842835	-1.068689564	0.000186426	2.45212162
NM_024042	meteorin, glial cell differentiation regulator	METRN	-2.211028326	-4.630051774	-0.912825794	-1.882729575	0.04785929	2.459222946
NM_021727	fatty acid desaturase 3	FADS3	-1.489003906	-2.806951052	-0.189284298	-1.140197938	9.94E-05	2.461810321
AB209485	vascular endothelial growth factor	VEGF	-1.790256026	-3.458762675	-0.488371215	-1.402860169	0.01173264	2.46550779
NM_003260	transducin-like enhancer of split 2	TLE2	-1.402226777	-2.643092246	-0.09712019	-1.069636195	0.000531287	2.471019827
NM_138355	secernin 2	SCRN2	-1.519962563	-2.867836076	-0.212201391	-1.158454508	0.000127636	2.475570734
NM_000116	tafazzin	TAZ	-1.46190442	-2.754717584	-0.153541879	-1.11229686	0.000195405	2.47660286
NM_005231	cortactin	CTTN	-1.522552027	-2.872988119	-0.212245611	-1.158490016	1.66E-05	2.479942062
NM_032867	hypothetical protein FLJ14966	FLJ14966	-1.408606486	-2.654806085	-0.095693698	-1.068579094	0.045238681	2.484426375
NM_018207	hypothetical protein FLJ10759	FLJ10759	-1.508027217	-2.844208478	-0.193288657	-1.143367079	0.01516351	2.487572477
XM_290835	zinc finger protein 181	ZNF181	-1.826881258	-3.547693214	-0.508257298	-1.422331054	0.007151958	2.494280923
CO646479	ATP-binding cassette, sub-family A	ABC1	-1.357807704	-2.56295421	-0.037129982	-1.026070587	0.000283752	2.497834206
NM_174940	hypothetical protein LOC283232	LOC283232	-1.599719694	-3.030844203	-0.27825862	-1.212730196	0.002246512	2.499190845
NM_015330	KIAA0376 protein	KIAA0376	-1.448974957	-2.730140045	-0.125715104	-1.091048401	0.00156712	2.502308827
NM_014683	unc-51-like kinase 2	ULK2	-1.816076292	-3.521222257	-0.49054874	-1.404979168	0.018740797	2.506245172
NM_138414	hypothetical protein BC011981	LOC112869	-1.438099542	-2.709636907	-0.106110166	-1.076322311	0.000590156	2.517495808
NM_005399	protein kinase, AMP-activated, beta 2 non-catalytic subunit	PRKAB2	-1.381871613	-2.606062371	-0.047207059	-1.033262673	0.002035807	2.522168311
NM_032687	cysteine and histidine rich 1	CYHR1	-1.585402377	-3.000914836	-0.248002295	-1.187561555	3.93E-05	2.526955191
NM_006594	adaptor-related protein complex 4, beta 1 subunit	AP4B1	-2.011009058	-4.030640349	-0.671012927	-1.592190464	0.015297712	2.5315064
NM_005385	natural killer-tumor recognition sequence	NKTR	-1.536103317	-2.900101345	-0.195969382	-1.145493587	0.000296041	2.531748216
NM_015037	KIAA0913	KIAA0913	-1.533988961	-2.895854186	-0.193554242	-1.143577581	6.20E-05	2.532276109

NM_002641	phosphatidylinositol glycan, class A paroxysmal nocturnal hemoglobinuria	PIGA	-1.589155854	-3.008732519	-0.247820163	-1.187411642	0.003404297	2.533858025
NM_032228	male sterility domain containing 2	MLSTD2	-1.400642525	-2.640191406	-0.058095164	-1.041090268	0.00050303	2.535987019
XR_012685	zinc finger protein 221	ZNF221	-1.833521472	-3.564059624	-0.490404924	-1.404839119	0.015539309	2.536987741
NM_002344	leukocyte tyrosine kinase	LTK	-1.390895231	-2.622413582	-0.04221899	-1.029696372	0.001078296	2.546783357
NM_153832	G protein-coupled receptor 161	GPR161	-1.538210321	-2.904339933	-0.187101091	-1.1384738	0.00425385	2.551081924
NM_004524	lethal giant larvae homolog 2	LLGL2	-1.570327087	-2.969720358	-0.212439561	-1.158645769	1.43E-05	2.563096018
NM_001266	carboxylesterase 1 monocyte/macrophage serine esterase 1	CES1	-1.75780718	-3.381837128	-0.397193122	-1.316943199	6.60E-05	2.567944564
NM_152775	KM-HN-1 protein	KM-HN-1	-1.44525458	-2.723108715	-0.082132159	-1.058581362	0.042546142	2.572413243
NM_020349	ankyrin repeat domain 2	ANKRD2	-1.426390401	-2.687734068	-0.062530653	-1.044295971	0.009862533	2.573728276
NM_015832	methyl-CpG binding domain protein 2	MBD2	-1.837967574	-3.575060295	-0.474006961	-1.388961837	0.003814874	2.573908223
NM_178493	hypothetical protein LOC147111	LOC147111	-1.545898608	-2.919858807	-0.179784822	-1.132714928	0.006615086	2.577752561
XR_013572	Connector enhancer of kinase suppressor of ras 1	CNKSR1	-1.574941979	-2.979235108	-0.205448467	-1.153044719	0.003195454	2.583798406
NM_005560	laminin, alpha 5	LAMA5	-1.91342253	-3.767016964	-0.542186487	-1.456177766	1.28E-05	2.586921082
NM_030781	collectin sub-family member 12	COLEC12	-1.505247482	-2.838733631	-0.133937091	-1.09728409	0.002691144	2.587054399
NM_000850	glutathione S-transferase M4	GSTM4	-2.00812778	-4.022598588	-0.634191078	-1.552067259	0.000163774	2.591768214
NM_020317	NPD014 protein	NPD014	-1.563382643	-2.955459894	-0.189356549	-1.140255042	8.34E-06	2.591928811
XM_498188	phosphoglycerate mutase 2	PGAM2	-2.283969524	-4.870161196	-0.908386573	-1.876945257	0.005813622	2.594727351
NM_018081	hypothetical protein FLJ10385	FLJ10385	-1.516300341	-2.860565425	-0.136019487	-1.09886906	0.00019813	2.603190434
NM_145753	pleckstrin homology-like domain, family B, member 2	PHLDB2	-1.982983845	-3.953098339	-0.599079997	-1.514750306	0.00023545	2.609735956
DR771020	protein phosphatase 1, regulatory (inhibitor) subunit 16A	PPP1R16A	-1.940621134	-3.838708829	-0.547616483	-1.461668836	0.009007577	2.626250718
NM_145296	immunoglobulin superfamily, member 4C	IGSF4C	-1.538471564	-2.904865899	-0.143777623	-1.104794179	0.01071785	2.629327665
NM_020246	solute carrier family 12 potassium/chloride transporters	SLC12A9	-1.594874017	-3.020681379	-0.199613988	-1.148391047	0.000211696	2.630359569
NM_015548	dystonin	DST	-1.694338759	-3.236285216	-0.289282007	-1.222031952	0.000158117	2.648281995
NM_018398	calcium channel, voltage-dependent, alpha 2/delta 3 subunit	CACNA2D3	-2.054010517	-4.152587354	-0.644271607	-1.562949979	0.022083573	2.656890757
XR_012635	cancer susceptibility candidate 1	LOC707753	-1.533695203	-2.8952646	-0.123952613	-1.089716319	0.010851485	2.656897534
XM_930351	zinc finger protein HIT-40, transcript variant 1	HIT-40	-1.887395299	-3.699666684	-0.473795582	-1.388758347	0.022740501	2.664010404
NM_004277	solute carrier family 25, member 27	SLC25A27	-1.906778424	-3.749708443	-0.488156746	-1.402651637	0.010363592	2.673299872
NM_018296	hypothetical protein FLJ11004	FLJ11004	-1.493659865	-2.81602446	-0.073375998	-1.052175964	0.000908322	2.676381668
NM_183380	dystonin	DST	-1.788575956	-3.454737167	-0.365307737	-1.288156373	6.89E-05	2.681923747
NM_013304	zinc finger, DHHC-type containing 1	ZDHHC1	-1.622585269	-3.079263374	-0.198687742	-1.147653988	0.00104177	2.683093866
XR_012933	centaurin, gamma 3	LOC714140	-1.46903418	-2.768365019	-0.042092258	-1.029605923	8.93E-07	2.688761746
NM_003573	latent transforming growth factor beta binding protein 4	LTBP4	-1.796623934	-3.474063042	-0.364946187	-1.287833592	2.44E-05	2.697602441
NM_015644	tubulin tyrosine ligase-like family, member 3	TTLL3	-1.571700498	-2.972548806	-0.137789512	-1.100218077	3.87E-05	2.701781463
NM_004670	3'-phosphoadenosine 5'-phosphosulfate synthase 2	PAPSS2	-1.716002225	-3.28524787	-0.280538029	-1.214647783	6.78E-05	2.704691777
NM_025099	hypothetical protein FLJ22170	FLJ22170	-1.539161683	-2.906255785	-0.1021123	-1.073343832	3.83E-05	2.707665239
NM_014587	SRY	SOX8	-1.608009495	-3.048309724	-0.168110797	-1.12358619	0.005682821	2.713018148
XR_011442	hypothetical protein LOC705419	LOC705419	-1.590336454	-3.01119566	-0.147001552	-1.107265775	0.006218828	2.719487703
NM_024650	hypothetical protein FLJ22531	FLJ22531	-1.738069285	-3.335884376	-0.292753903	-1.224976356	0.019400755	2.72322348
NM_002507	nerve growth factor receptor TNFR superfamily, member 1€	NGFR	-1.453935609	-2.739543684	-0.007281181	-1.005059687	0.00406269	2.72575223
NM_139205	Unknown		-1.529201709	-2.886260886	-0.069328652	-1.049228319	0.027037266	2.750841579
NM_007037	a disintegrin-like and metalloprotease	ADAMTS8	-1.621887061	-3.077773493	-0.157272886	-1.115177135	0.001047004	2.759896519
NM_004926	zinc finger protein 36, C3H type-like 1	ZFP36L1	-1.618262731	-3.070051233	-0.147500144	-1.10764851	0.003756096	2.771683621
NM_001554	cysteine-rich, angiogenic inducer, 61	CYR61	-1.935823563	-3.8259647	-0.464125111	-1.379480546	0.000254457	2.773482171
NM_152748	hypothetical protein FLJ31340	FLJ31340	-1.483273733	-2.795824365	-0.000647206	-1.00044871	3.96E-05	2.794570414
NM_152600	zinc finger protein 579	ZNF579	-1.704060721	-3.258167381	-0.217107341	-1.162400591	0.00021665	2.80296432

NM_004761	ral guanine nucleotide dissociation stimulator-like 2	RGL2	-1.703977248	-3.257978872	-0.208702383	-1.155648281	3.39E-06	2.819178574
NM_003500	acyl-Coenzyme A oxidase 2, branched chain	ACOX2	-1.698091859	-3.244715212	-0.201711453	-1.150061851	0.005881794	2.821339746
XR_013832	protein phosphatase 1, regulatory inhibitor	PPP1R14D	-1.551550099	-2.931319246	-0.055019418	-1.038873086	0.004365171	2.821633639
NM_001932	membrane protein, palmitoylated 3 MAGUK p55 subfamily member 3	MPP3	-1.529249008	-2.886355514	-0.031092681	-1.021785721	8.24E-05	2.824814884
XM_370946	endoplasmic reticulum ER	LOC388226	-1.655429453	-3.150169491	-0.155543901	-1.113841461	0.026260637	2.828202756
NM_020684	rhomboid, veinlet-like 7	RHBDL7	-1.579204577	-2.988050595	-0.079166	-1.056407171	5.11E-06	2.828502757
NM_176800	PRP4 pre-mRNA processing factor 4 homolog B	PRPF4B	-1.536247148	-2.900390489	-0.033460913	-1.023464394	0.001420347	2.833894862
NM_022095	zinc finger protein 335	ZNF335	-1.620982599	-3.075844563	-0.112927677	-1.08142055	0.001191312	2.844263097
NM_001001323	ATPase, Ca++ transporting, plasma membrane 1	ATP2B1	-1.517496495	-2.862938134	-0.005773889	-1.004010174	0.000717669	2.85150311
NM_152421	hypothetical protein MGC20262	MGC20262	-1.655966858	-3.151343151	-0.141390643	-1.102967777	0.004241278	2.857148883
NM_018062	Fanconi anemia, complementation group L	FANCL	-1.544274617	-2.916573874	-0.02150185	-1.015015564	0.028994767	2.873427735
NM_015065	SLAC2-B	SLAC2-B	-1.79789235	-3.477118777	-0.274020645	-1.209172979	0.007540511	2.875617333
NM_024581	chromosome 6 open reading frame 60	C6orf60	-2.616664042	-6.133302219	-1.078594424	-2.11197744	0.038621497	2.904056693
XR_011108	cleavage and polyadenylation specific factor 1, 160kDa	CPSF1	-1.841049853	-3.582706479	-0.300494202	-1.23156622	0.000746808	2.909065239
NM_001671	asialoglycoprotein receptor 1	ASGR1	-1.607023853	-3.046227845	-0.062924938	-1.044581413	0.002578982	2.916218696
NM_002766	phosphoribosyl pyrophosphate synthetase-associated protein 1	PRPSAP1	-1.594428759	-3.019749252	-0.047098012	-1.033184576	6.26E-05	2.922758741
NM_015308	formin binding protein 4	FNBP4	-1.844353914	-3.590921	-0.296495398	-1.228157341	0.001046713	2.923828146
NM_025179	plexin A2	PLXNA2	-1.617271591	-3.067942814	-0.068597981	-1.048697058	0.001617361	2.925480518
NM_138477	congenital dyserythropoietic anemia, type I	CDAN1	-1.670004185	-3.182155166	-0.118631749	-1.085704693	0.000244306	2.930958286
NM_000292	phosphorylase kinase, alpha 2	PHKA2	-1.612406914	-3.057615324	-0.060495792	-1.042824073	0.007876412	2.932052878
NM_003913	PRP4 pre-mRNA processing factor 4 homolog B	PRPF4B	-1.607017286	-3.046213977	-0.053357946	-1.037677362	0.002855936	2.935608011
NM_000070	calpain 3,	CAPN3	-1.634670806	-3.105166882	-0.07856368	-1.055966217	9.16E-05	2.940593015
NM_033419	per1-like domain containing 1	PERLD1	-1.587204692	-3.004666131	-0.028880306	-1.020220013	0.001684063	2.945115851
NM_019601	sushi domain containing 2	SUSD2	-1.813401818	-3.514700647	-0.241324333	-1.182077261	5.76E-06	2.973325656
NM_001835	clathrin, heavy polypeptide-like 1	CLTCL1	-2.050563408	-4.142677196	-0.471918395	-1.386952515	0.028777476	2.986891874
NM_021922	Fanconi anemia, complementation group E	FANCE	-1.687663845	-3.221346487	-0.10591715	-1.076178321	0.000302436	2.993320367
NM_013343	loss of heterozygosity, 3, chromosomal region 2, gene A	LOH3CR2A	-2.208072971	-4.620576839	-0.621476982	-1.538449387	0.023917964	3.003398667
NM_018607	hypothetical protein PRO1853	PRO1853	-1.647657566	-3.133244961	-0.061003843	-1.043191372	0.000113098	3.003518859
NM_002395	malic enzyme 1, NADP	ME1	-1.837866533	-3.574809921	-0.250243856	-1.189408141	0.004560185	3.005536786
NM_005657	tumor protein p53 binding protein, 1	TP53BP1	-1.659553953	-3.15918835	-0.070491142	-1.050074103	0.000338039	3.008538483
NM_022467	carbohydrate N-acetylgalactosamine 4-0	CHST8	-1.712864593	-3.278110743	-0.121828391	-1.088113003	0.008393633	3.012656529
NM_005964	myosin, heavy polypeptide 10, non-muscle	MYH10	-1.648644664	-3.135389475	-0.056056893	-1.039620432	4.29E-05	3.015898282
NM_198284	hypothetical protein LOC349114	LOC349114	-2.579550559	-5.977534531	-0.981394717	-1.974373201	0.004313084	3.027560609
NM_000828	glutamate receptor, ionotrophic, AMPA 3	GRIA3	-1.624670432	-3.083717129	-0.026497882	-1.018536642	0.005730714	3.027595672
NM_024874	KIAA0319-like	KIAA0319L	-1.630553455	-3.096317586	-0.013756803	-1.009581097	0.000254264	3.066933004
NM_002398	Meis1, myeloid ecotropic viral integration site 1 homolog	MEIS1	-1.632380645	-3.100241593	-0.009244926	-1.00642867	0.001197439	3.080438471
NM_002722	pancreatic polypeptide	PPY	-1.728706249	-3.314304715	-0.101495024	-1.072884686	0.002012312	3.089152783
NM_017514	plexin A3	PLXNA3	-1.66085133	-3.162030602	-0.030772643	-1.021559081	6.38E-05	3.095298805
NM_002513	non-metastatic cells 3, protein expressed in	NME3	-1.661792323	-3.164093698	-0.030655464	-1.02147611	8.07E-05	3.097569944
NM_139016	hypothetical gene LOC128439	LOC128439	-2.032466831	-4.091037698	-0.398657826	-1.318280912	0.001887042	3.10331255
NM_002528	nth endonuclease III-like 1	NTHL1	-1.687308394	-3.22055291	-0.049534614	-1.034931021	0.000188585	3.111852718
NM_032780	transmembrane protein 25	TMEM25	-2.084922626	-4.242523425	-0.445886415	-1.362150787	0.00030051	3.114576937
NM_003103	SON DNA binding protein	SON	-1.785779109	-3.448046209	-0.142114348	-1.103521201	2.53E-05	3.124585377
NM_016382	CD244 natural killer cell receptor 2B4	CD244	-2.0422119	-4.118765244	-0.39710254	-1.316860515	0.005943932	3.127715652

NM_003798	catenin cadherin-associated protein	CTNNAL1	-1.654499259	-3.148139037	-0.00843182	-1.005861605	0.020278079	3.129793425
NM_032207	hypothetical protein FLJ21742	FLJ21742	-1.881968136	-3.685775338	-0.222504694	-1.166757462	0.0008829	3.158990157
NM_002751	mitogen-activated protein kinase 11	MAPK11	-2.168882382	-4.496749076	-0.508114143	-1.422189927	0.002757188	3.161848491
NM_004816	chromosome 9 open reading frame 61	C9orf61	-2.561306441	-5.902419422	-0.900073187	-1.86616065	0.000168868	3.162867795
CN801688	ILLUMIGEN_MCQ_35906 Katze_MMPL1 cDNA clone IBIUW:15421 5' Bases 1 to 465 highly human ARVCF	Hs.326730	-1.691293373	-3.229460945	-0.026667317	-1.018656269	0.000249551	3.170314701
XR_009827	titin isoform N2-A	LOC694413	-1.932959187	-3.818376035	-0.267745984	-1.203925387	0.002877961	3.171605216
NM_024790	hypothetical protein FLJ22490	FLJ22490	-2.107406619	-4.309159858	-0.433336636	-1.350353037	0.003620644	3.191135753
XR_013084	F-box and WD-40 domain protein 7, archipelago homolog	LOC714887	-2.173775103	-4.51202513	-0.49827899	-1.412527534	0.008212123	3.194291807
NM_020307	cyclin L1	CCNL1	-1.821274522	-3.533932592	-0.13781541	-1.100237827	1.86E-05	3.211971543
NM_003605	O-linked N-acetylglucosamine	OGT	-2.027860876	-4.077997458	-0.342750914	-1.268172426	0.000187918	3.21564905
NM_007056	splicing factor, arginine/serine-rich 16	SFRS16	-2.004721465	-4.013112126	-0.316503125	-1.245308448	0.000574704	3.222584839
NM_023015	hypothetical protein FLJ21919	FLJ21919	-1.862644077	-3.636735688	-0.171992548	-1.126613409	4.85E-05	3.228024501
NM_006386	DEAD Asp-Glu-Ala-Asp	DDX17	-1.914271517	-3.769234404	-0.219284072	-1.164155739	3.61E-05	3.23774069
NM_005463	heterogeneous nuclear ribonucleoprotein D-like	HNRPDL	-2.045603311	-4.128458809	-0.350593065	-1.275084683	0.000382461	3.237791862
NM_005778	RNA binding motif protein 5	RBM5	-1.921558681	-3.788321262	-0.21803068	-1.163144776	4.66E-06	3.256964515
NM_001546	inhibitor of DNA binding 4, dominant negative helix-loop-helix protein	ID4	-1.955495528	-3.878491198	-0.239676456	-1.180727838	0.019632003	3.284830826
NM_005994	T-box 2	TBX2	-2.142993443	-4.416775313	-0.424369881	-1.341986242	3.26E-05	3.2912225
NM_001005912	inositol hexaphosphate kinase 2	IHPK2	-1.996647411	-3.990715441	-0.272587443	-1.207972359	0.001144223	3.303647978
XR_011927	modulator of estrogen induced transcription isoform a	LOC701841	-1.774087956	-3.420217223	-0.046254697	-1.032580814	0.000247781	3.312299799
NM_032140	chromosome 16 open reading frame 48	C16orf48	-1.814181885	-3.516601565	-0.076787531	-1.054666983	0.002694507	3.334324126
NM_020812	dedicator of cytokinesis 6	DOCK6	-1.907432405	-3.75140859	-0.154935142	-1.113371564	4.14E-06	3.36941297
NM_001009569	myeloid/lymphoid or mixed-lineage leukemia	MLLT10	-1.805958095	-3.496612916	-0.040973034	-1.028807478	0.00010878	3.398704804
NM_198943	CXYorf1-related protein	MGC52000	-2.31626978	-4.98042818	-0.54691814	-1.460961479	1.08E-06	3.409007185
NM_014859	KIAA0672 gene product	KIAA0672	-1.951360229	-3.867389922	-0.16888323	-1.12418793	7.61E-05	3.440163178
XR_010803	Y73F8A.5	LOC697670	-1.859751166	-3.629450564	-0.070644488	-1.050185723	0.003000415	3.45600829
XR_014054	leucine rich repeat containing 50	LOC715137	-1.837117999	-3.572955633	-0.047251193	-1.033294283	0.048311913	3.457829673
NM_033044	microtubule-actin crosslinking factor 1	MACF1	-1.828221245	-3.55098987	-0.025294893	-1.01768769	3.80E-05	3.489272695
NM_152221	casein kinase 1, epsilon	CSNK1E	-1.924014466	-3.794775312	-0.107097169	-1.077058916	7.87E-05	3.523275519
NM_057162	kelch-like 4	KLHL4	-1.925786658	-3.799439639	-0.106875081	-1.076893127	0.022828779	3.528149213
NM_001003679	leptin receptor	LEPR	-1.882319012	-3.686671861	-0.063111554	-1.04471654	0.005805604	3.528872874
BX647378	DKFZp686M24111		-2.100195868	-4.287675927	-0.267620394	-1.203820587	0.015331669	3.561723378
NM_005455	zinc finger protein 265	ZNF265	-2.146302972	-4.426918985	-0.311527367	-1.241020862	0.003993934	3.567159201
NM_030567	hypothetical protein MGC10772	MGC10772	-2.352846524	-5.108311579	-0.511084282	-1.425120867	0.019706761	3.584476025
NM_024631	hypothetical protein FLJ23342	FLJ23342	-1.962681629	-3.897858256	-0.119029709	-1.08600422	0.002593778	3.589174135
NM_005654	nuclear receptor subfamily 2, group F, member 1	NR2F1	-1.95537165	-3.878158183	-0.10557384	-1.075922259	6.53E-07	3.604496653
NM_006362	nuclear RNA export factor 1	NXF1	-1.883151939	-3.688800942	-0.030481809	-1.021353164	1.63E-05	3.611680143
NM_004177	syntaxin 3A	STX3A	-1.981426372	-3.948833044	-0.124056934	-1.089795119	0.000119354	3.623463692
CN646438	ILLUMIGEN_MCQ_26202 Katze_MMBR cDNA clone IBIUW:8578 5' Bases 1 to 503 highly human APBB1	Hs.378063	-2.010752534	-4.029923729	-0.151564708	-1.110773534	5.49E-05	3.628033624
NM_003456	zinc finger protein 205	ZNF205	-1.920770174	-3.786251314	-0.056741499	-1.040113883	8.10E-05	3.640227647
AW014767	UI-H-BI0-aae-f-12-0-UI.s1 NCI_CGAP_Sub1 cDNA clone IMAGE:2709262 3' sequence		-2.538154231	-5.808454038	-0.668410243	-1.589320673	9.86E-05	3.654677207
NM_000667	alcohol dehydrogenase 1A	ADH1A	-2.241983794	-4.730470864	-0.369343998	-1.291765323	7.36E-05	3.662020321
NM_032389	zinc finger protein 289, ID1 regulated	ZNF289	-1.95961581	-3.889583854	-0.08696698	-1.062134877	8.06E-05	3.662043248

NM_002673	plexin B1	PLXNB1	-2.160394256	-4.470370036	-0.285523181	-1.21885219	4.99E-05	3.667688397
NM_033520	chromosome 19 open reading frame 33	C19orf33	-2.214253402	-4.64041361	-0.337166	-1.263272609	0.000464359	3.67332718
NM_018584	calcium/calmodulin-dependent protein kinase II	CaMKIIN	-2.150962743	-4.441240639	-0.273409824	-1.208661137	1.10E-05	3.674512652
NM_014884	splicing factor, arginine/serine-rich 14	SFRS14	-2.176868311	-4.521709509	-0.294577831	-1.226526013	0.000208619	3.686598949
XM_113796	hypothetical protein LOC196996	LOC196996	-2.473374968	-5.553414064	-0.590331649	-1.505592815	0.00026854	3.688523223
NM_018032	LUC7-like	LUC7L	-2.072727018	-4.206811044	-0.184840353	-1.136691182	6.43E-06	3.700926964
XM_044461	KIAA1102 protein	KIAA1102	-2.314657597	-4.974865759	-0.415304819	-1.333580412	5.19E-05	3.730458032
NM_013241	formin homology 2 domain containing 1	FHOD1	-2.035326806	-4.099155747	-0.122520321	-1.088634997	9.12E-05	3.765408753
NM_004071	CDC-like kinase 1	CLK1	-2.058410159	-4.165270412	-0.141391424	-1.102968374	6.82E-06	3.776418717
NM_003320	tubby homolog	TUB	-1.948475603	-3.859664918	-0.023091005	-1.016134239	0.030733864	3.798380933
NM_152399	hypothetical protein FLJ30834	FLJ30834	-1.961818129	-3.895525959	-0.019722424	-1.013764412	0.001930193	3.842634357
NM_005252	v-fos FBJ murine osteosarcoma viral oncogene homolog	FOS	-2.770846841	-6.825084184	-0.815885254	-1.760378008	7.65E-06	3.877056036
NM_001136	advanced glycosylation end product-specific receptor	AGER	-2.050983806	-4.14388454	-0.094622108	-1.067785681	5.55E-05	3.880820481
XR_014590	hypothetical protein LOC721251	LOC721251	-1.996821632	-3.991197393	-0.038962977	-1.027375074	0.001066079	3.884849354
NM_015726	H326	H326	-2.076172166	-4.21686889	-0.109950173	-1.079190963	4.09E-05	3.907435323
NM_017980	LIM and senescent cell antigen-like domains 2	LIMS2	-2.216714671	-4.648337015	-0.249595905	-1.188874068	0.000102888	3.909864922
NM_016438	CLST 11240 protein	CLST11240	-2.010312675	-4.028695244	-0.041986199	-1.029530235	0.000453604	3.91313932
NM_025198	transcription termination factor-like protein	LOC80298	-2.16354633	-4.480147808	-0.189242263	-1.140164718	0.000481663	3.929386463
NM_024669	hypothetical protein FLJ11795	FLJ11795	-2.339292372	-5.060543618	-0.358912793	-1.28245908	0.001192754	3.94596888
NM_178525	hypothetical protein MGC33407	MGC33407	-2.552546352	-5.866688335	-0.548550293	-1.462615233	0.009614992	4.011094786
XR_012049	ankyrin repeat and sterile alpha motif domain containing 3	LOC706182	-2.150952335	-4.4412086	-0.141003619	-1.102671929	0.000263778	4.027679025
NM_152422	protein tyrosine phosphatase domain containing 1	PTPDC1	-2.012942936	-4.036046891	-0.00084862	-1.000588392	0.000570259	4.033673511
NM_001012973	placenta-specific 9	PLAC9	-2.104241286	-4.299715746	-0.084766436	-1.060516036	2.32E-06	4.054361838
NM_005490	SH2 domain containing 3A	SH2D3A	-2.648360213	-6.269542684	-0.624091538	-1.541240004	0.00110675	4.067856186
NM_021078	GCN5 general control of amino-acid synthesis 5-like 2	GCN5L2	-2.25495833	-4.773205105	-0.224387414	-1.168281076	4.19E-06	4.085665
NM_005251	forkhead box C2	FOXC2	-2.046503929	-4.131036849	-0.010491484	-1.007298649	0.041599697	4.101104328
NM_016569	T-box 3	TBX3	-2.192202714	-4.57002708	-0.149905914	-1.109497113	6.86E-05	4.119007634
NM_015319	tensin like C1 domain containing phosphatase	TENC1	-2.185064553	-4.547471364	-0.135251696	-1.098284406	2.14E-06	4.140522563
NM_000022	adenosine deaminase	ADA	-2.277651675	-4.848880418	-0.227002124	-1.170400365	3.58E-06	4.142924561
NM_004784	N-deacetylase/N-sulfotransferase heparan glucosaminyl	NDST3	-2.42512258	-5.370746321	-0.366884358	-1.289564879	0.000643692	4.164774032
NM_019057	hypothetical protein FLJ10404	FLJ10404	-2.235611009	-4.70962114	-0.160374968	-1.117577567	0.000651945	4.214133567
NM_203306	hypothetical protein MGC39606	MGC39606	-2.094211358	-4.26992686	-0.018741122	-1.013075097	0.003319805	4.214817708
NM_005049	PWP2 periodic tryptophan protein homolog	PWP2H	-2.15370433	-4.449688456	-0.072723832	-1.051700438	4.33E-05	4.23094666
NM_080825	chromosome 20 open reading frame 144	C20orf144	-2.128518379	-4.372681834	-0.029241828	-1.0204757	0.001353484	4.284944595
NM_175709	chromobox homolog 7	CBX7	-2.282637113	-4.865665399	-0.165489202	-1.121546318	7.83E-05	4.338354396
NM_005203	collagen, type XIII, alpha 1	COL13A1	-3.141140171	-8.822210423	-1.018243601	-2.025451586	0.013994425	4.355675784
NM_006715	mannosidase, alpha, class 2C, member 1	MAN2C1	-2.257959091	-4.783143556	-0.120616719	-1.087199516	5.37E-05	4.399508541
NM_014905	glutaminase	GLS	-2.526095844	-5.760107927	-0.387445543	-1.308075255	0.00617906	4.403498886
NM_007168	ATP-binding cassette, sub-family A	ABCA8	-2.626871848	-6.176852363	-0.480843635	-1.3955595	0.018198843	4.426075967
NM_005297	G protein-coupled receptor 24	GPR24	-2.629117153	-6.186473049	-0.461314339	-1.37679555	0.033217347	4.493385419
NM_003416	zinc finger protein 7	ZNF7	-2.555464986	-5.878568911	-0.378617619	-1.300095516	1.63E-05	4.521643864
NM_020039	amiloride-sensitive cation channel 2, neuronal	ACCN2	-2.477784882	-5.570415264	-0.298647668	-1.229990922	0.001497322	4.528826322
NM_004826	endothelin converting enzyme-like 1	ECEL1	-2.367194607	-5.15936891	-0.178550035	-1.131745865	0.000205337	4.558769836
NM_015689	KIAA1277	KIAA1277	-2.263964531	-4.803095624	-0.073146939	-1.052008921	0.001533368	4.565641533
NM_017673	chromosome 1 open reading frame 26	C1orf26	-2.221085071	-4.662439713	-0.024582039	-1.017184962	4.27E-05	4.583669526
NM_006107	acid-inducible phosphoprotein	OA48-18	-2.444987459	-5.445209132	-0.236784612	-1.17836347	2.57E-05	4.620992817

NM_019020	TBC1 domain family, member 16	TBC1D16	-2.251155807	-4.760640893	-0.033698919	-1.023633253	1.48E-06	4.650729038
NM_021805	single Ig IL-1R-related molecule	SIGIRR	-2.288100251	-4.884125432	-0.064032704	-1.045383797	5.18E-07	4.672088322
NM_014945	actin binding LIM protein family, member 3	ABLIM3	-2.312210187	-4.966433477	-0.0786945	-1.056061974	0.000225389	4.70278601
NM_000506	coagulation factor II	F2	-2.364804034	-5.150826817	-0.114138206	-1.082328323	0.006835231	4.759024323
NM_001323	cystatin E/M	CST6	-2.312484561	-4.967378088	-0.052946203	-1.037381253	3.14E-05	4.788382356
NM_024671	hypothetical protein FLJ23436	FLJ23436	-2.771631969	-6.828799467	-0.48085175	-1.39556735	0.00038345	4.893206673
XR_011978	vacuolar protein sorting 13C protein isoform 2A	LOC709080	-2.418779964	-5.347186382	-0.115162255	-1.083096851	0.003168456	4.936942044
NM_144650	alcohol dehydrogenase, iron containing, 1	ADHFE1	-2.378835892	-5.201168914	-0.073023476	-1.051918896	0.000290841	4.944458106
NM_014370	serine/threonine kinase 23	STK23	-2.31838146	-4.987723396	-0.008470461	-1.005888546	0.001293767	4.958524894
NM_201446	EGF-like-domain, multiple 7	EGFL7	-2.338172053	-5.0566154	-0.023288568	-1.016273398	4.32E-07	4.975644753
NM_024083	alveolar soft part sarcoma chromosome region, candidate 1	ASPSCR1	-2.715462138	-6.568036472	-0.363920552	-1.286918376	1.18E-05	5.103693127
NM_017934	pleckstrin homology domain interacting protein	PHIP	-2.567818746	-5.929123082	-0.208318653	-1.15534094	1.67E-05	5.131925022
NM_003638	integrin, alpha 8	ITGA8	-2.510070421	-5.696478833	-0.135123616	-1.098186907	1.67E-05	5.187166953
NM_020201	5',3'-nucleotidase, mitochondrial	NT5M	-2.60763476	-6.095036074	-0.221796512	-1.166184871	0.000560385	5.226474999
NM_012309	SH3 and multiple ankyrin repeat domains 2	SHANK2	-2.555206389	-5.877515298	-0.168665902	-1.124018594	0.000215789	5.229019634
NM_057091	artemin	ARTN	-2.498633361	-5.651498151	-0.109071237	-1.078533686	7.07E-05	5.239982974
NM_199242	unc-13 homolog D	UNC13D	-2.577288409	-5.968169085	-0.186761987	-1.138206235	2.82E-07	5.243486553
NM_024101	melanophilin	MLPH	-2.871768838	-7.319620432	-0.475454533	-1.390356194	8.72E-06	5.264564909
CB548968	MMPL0018_H05 MMPL cDNA sequence		-2.555881167	-5.880264974	-0.158924765	-1.116454739	1.83E-05	5.26690852
NM_025245	pre-B-cell leukemia transcription factor 4	PBX4	-2.442173742	-5.434599582	-0.024461832	-1.017100213	3.43E-05	5.343229225
NM_005090	phospholipase A2, group IVB	PLA2G4B	-2.770896696	-6.825320041	-0.348235189	-1.273002444	6.08E-06	5.361592251
NM_002404	microfibrillar-associated protein 4	MFAP4	-2.564011182	-5.913495558	-0.129940644	-1.09424868	5.91E-06	5.404160558
NM_000362	tissue inhibitor of metalloproteinase 3	TIMP3	-2.457899224	-5.494161139	-0.017717584	-1.012356613	1.78E-05	5.427100556
NM_012101	tripartite motif-containing 29	TRIM29	-2.788994826	-6.911480711	-0.319758731	-1.248121802	0.04059798	5.537504994
NM_005106	deleted in lung and esophageal cancer 1	DLEC1	-3.058353287	-8.330212435	-0.560683949	-1.4749683	0.003972061	5.647723028
NM_020353	phospholipid scramblase 4	PLSCR4	-2.554979715	-5.876591905	-0.051871003	-1.03660841	0.000136416	5.669056753
NM_000466	peroxisome biogenesis factor 1	PEX1	-2.704508861	-6.518359226	-0.171494851	-1.12622482	3.59E-05	5.787795748
NM_025092	hypothetical protein FLJ22635	FLJ22635	-2.887818454	-7.401503975	-0.353509267	-1.277664691	3.26E-06	5.792994065
NM_020890	KIAA1524 protein	KIAA1524	-2.603087351	-6.075854625	-0.060496023	-1.04282424	0.011358674	5.826345794
NM_001011656	zinc finger, matrin type 1	ZMAT1	-2.604645047	-6.082418343	-0.041891802	-1.029462874	0.002655296	5.90834162
NM_145307	pleckstrin homology domain containing, family K member 1	PLEKHK1	-2.766343684	-6.803813916	-0.176061614	-1.129795464	0.000177526	6.022164306
NM_004240	thyroid hormone receptor interactor 10	TRIP10	-2.731548352	-6.641680632	-0.138573082	-1.1008158	2.19E-05	6.033416885
XR_010759	protein kinase, cAMP-dependent, catalytic, gamma	LOC700652	-3.122365894	-8.708147798	-0.503144412	-1.417299259	0.01163141	6.144184258
NM_019593	hypothetical protein KIAA1434	KIAA1434	-2.652326883	-6.286804422	-0.012768846	-1.008889973	8.87E-05	6.231407382
NM_024552	LAG1 longevity assurance homolog 4	LASS4	-3.009939885	-8.055308742	-0.362294771	-1.285468957	0.001811977	6.266435839
NM_031456	F-box and WD-40 domain protein 10	FBXW10	-2.82931833	-7.107382433	-0.143293754	-1.104423701	0.000112549	6.435376589
NM_000779	cytochrome P450, family 4, subfamily B, polypeptide 1	CYP4B1	-3.535408108	-11.5948167	-0.77178262	-1.707378148	8.45E-07	6.791006851
XR_011250	phospholipase A2, group VI isoform a	LOC700184	-3.086363787	-8.493527114	-0.276773476	-1.211482425	9.23E-06	7.010854585
NM_024025	dual specificity phosphatase 26	DUSP26	-3.109333115	-8.629835831	-0.253786068	-1.192332053	1.52E-06	7.237778949
NM_004742	BAI1-associated protein 1	BAIAP1	-3.154118887	-8.901934511	-0.292988885	-1.225175893	0.004987835	7.265842043
NM_003812	a disintegrin and metalloproteinase domain 23	ADAM23	-2.926012323	-7.600067978	-0.053102929	-1.037493954	0.025104266	7.325409414
NM_000092	collagen, type IV, alpha 4	COL4A4	-3.39358485	-10.50922845	-0.509456329	-1.423513652	0.040694877	7.382597583
NM_001844	collagen, type II, alpha 1	COL2A1	-3.008386796	-8.046641713	-0.120782846	-1.087324715	0.009283067	7.400403581
NM_020978	amylase, alpha 2B; pancreatic	AMY2B	-2.911983124	-7.526520816	-0.004522639	-1.003139773	0.000319397	7.502963211
NM_000316	parathyroid hormone receptor 1	PTHR1	-3.011457649	-8.063787659	-0.068690335	-1.048764193	3.47E-07	7.688847226
NM_022161	baculoviral IAP repeat-containing 7	BIRC7	-3.540927161	-11.63925781	-0.581130272	-1.496020839	0.000152053	7.78014417

NM_007184	nischarin	NISCH	-3.045435188	-8.25595546	-0.05891172	-1.041679686	6.54E-06	7.925618184
NM_031921	ATPase family, AAA domain containing 3B	ATAD3B	-3.320481699	-9.989979374	-0.249180545	-1.188531834	0.001362957	8.405310729
NM_181710	zinc and ring finger 4	ZNRF4	-3.170271395	-9.002161175	-0.078858429	-1.056181977	0.005209557	8.523305044
NM_003166	sulfotransferase family, cytosolic, 1A, phenol-preferring, member 3	SULT1A3	-3.195154075	-9.158771361	-0.081034054	-1.057775931	0.00019501	8.658517452
NM_213647	fibroblast growth factor receptor 4	FGFR4	-3.466087959	-11.05086932	-0.130868904	-1.094952969	5.66E-06	10.09255158
NM_024875	synaptopodin 2-like	SYNPO2L	-3.469669025	-11.07833392	-0.10179588	-1.073108447	1.82E-05	10.32359214
CN647126	SH3-domain GRB2-like (endophilin) interacting protein 1	SGIP1	-3.436807043	-10.8288418	-0.064545897	-1.045755725	0.005436371	10.35503946
NM_032545	cripto, FRL-1, cryptic family 1	CFC1	-4.942628871	-30.75243772	-1.482817226	-2.794939833	0.012558139	11.00289794
NM_145249	family with sequence similarity 14, member B	FAM14B	-3.548214708	-11.69820043	-0.062508689	-1.044280072	0.000490065	11.20216764
AB038463	GC36	GC36	-3.771593934	-13.65723887	-0.178575139	-1.131765558	0.000347381	12.06719782
XR_013236	hypothetical protein LOC707467	LOC707467	-3.639411062	-12.46154517	-0.044075922	-1.031022575	0.000292514	12.08658809
XR_014213	tenascin XB isoform 1	LOC716998	-4.022709964	-16.25385432	-0.366988611	-1.289658069	3.42E-06	12.60322771
NM_182686	KIAA0319-like	KIAA0319L	-4.384189414	-20.88202063	-0.188803245	-1.139817814	0.041540592	18.3204898
XM_497045	ankyrin repeat domain 20A	LOC441425	-4.577418682	-23.87483209	-0.337415307	-1.263490929	7.99E-05	18.89592679
XR_011976	tumor suppressor candidate 5	LOC709072	-4.501679743	-22.65377765	-0.188173094	-1.139320064	0.000213245	19.88359405
