# Supplementary material for: Transcriptional Reprogramming in Nonhuman Primate (Rhesus Macaque) Tuberculosis Granulomas
Source: PLoS One. 2010 Aug 31;5(8):e12266. doi: 10.1371/journal.pone.0012266 (PMC2930844; doi:10.1371/journal.pone.0012266)
Supplement: Table S3 — DNA Microarray Analysis: Immune function genes with significantly enhanced expression in Mtb granuloma's relative to non-granulomatous tissue four week's post-infection. Symbol = Official NCBI human gene symbol associated with that gene. P = p value of significance in a t-test. (0.02 MB DOCX) [file pone.0012266.s003.docx]

| **Symbol** | **Gene** | **Av Fold Change (Lesion Lung /Normal Lung)** | ***P*** |
| --- | --- | --- | --- |
| MNP1A | α-defensin 1A | 19.94985 | 0.018986 |
| BAK1 | BCL2-antagonist/killer 1 | 2.139543 | 0.006048 |
| BCL2L13 | BCL2-like 13 apoptosis facilitator | 2.680362 | 0.000374 |
| BCL2L14 2 | BCL2-like 14 apoptosis facilitator | 9.148337 | 0.025378 |
| BCOR 1 | BCL6 co-repressor | 3.319626 | 0.012429 |
| C1QTNF1 | C1q and tumor necrosis factor related protein 1 | 11.25089 | 0.006678 |
| CREB3 | cAMP responsive element binding protein 3 | 2.125988 | 0.004977 |
| CREB3L2 | cAMP responsive element binding protein 3-like 2 | 2.356554 | 0.002246 |
| CREB5 | cAMP responsive element binding protein 5 | 3.672478 | 0.025191 |
| CASP1 | caspase 1, apoptosis-related cysteine protease interleukin 1, beta, convertase | 2.868488 | 0.001352 |
| CASP3 alpha | caspase 3, apoptosis-related cysteine protease | 4.313014 | 0.000414 |
| CASP4 gamma | caspase 4, apoptosis-related cysteine protease | 3.110329 | 6.18E-07 |
| CARD4 | caspase recruitment domain family, member 4 | 3.063022 | 6.99E-05 |
| CEBPB | CCAAT/enhancer binding protein C/EBP beta | 3.339808 | 3.63E-05 |
| CEBPD | CCAAT/enhancer binding protein C/EBP delta | 4.036521 | 9.92E-06 |
| CD14 | CD14 antigen | 4.773496 | 2.58E-06 |
| CD164 | CD164 antigen | 6.706027 | 0.040823 |
| CD180 | CD180 antigen | 3.458384 | 0.016636 |
| CD2 | CD2 antigen p50 | 2.057319 | 0.001972 |
| CD200 | CD200 molecule | 2.418645 | 0.017932 |
| CD274 | CD274 antigen | 19.15496 | 0.034456 |
| CD2AP | CD2-associated protein | 2.381017 | 0.005937 |
| CD300A | CD300A antigen | 4.435842 | 0.026136 |
| CD320 | CD320 antigen | 4.331412 | 0.000797 |
| CD36 | CD36 antigen | 2.896328 | 0.000620 |
| CD3D | CD3D antigen, delta polypeptide | 2.239868 | 0.003688 |
| CD40 | CD40 antigen TNF receptor superfamily - 5 | 7.655961 | 3.69E-05 |
| CD44 | CD44 antigen | 4.074632 | 0.000266 |
| CD48 | CD48 antigen B-cell membrane protein | 4.097641 | 0.000725 |
| CD53 | CD53 antigen | 6.702894 | 0.002817 |
| CD63 | CD63 antigen melanoma 1 antigen | 3.593155 | 3.11E-05 |
| CD74 | CD74 antigen invariant polypeptide of major histocompatibility complex, class II antigen-associated | 5.215173 | 0.000112 |
| CD8A | CD8 antigen, alpha polypeptide p32 | 2.774066 | 0.001482 |
| CD83 | CD83 antigen activated B lymphocytes, immunoglobulin superfamily | 4.659385 | 0.00172 |
| CD86 | CD86 antigen CD28 antigen ligand 2 | 22.98644 | 0.01046 |
| CKS2 | CDC28 protein kinase regulatory subunit 2 | 5.738479 | 0.00205 |
| CRK7 | CDC2-related protein kinase 7 | 2.081189 | 0.00862 |
| CXCL11 | chemokine (C-X-C motif) ligand 11 | 69.44196 | 0.000664 |
| CXCL13 | chemokine (C-X-C motif) ligand 13 | 8.568967 |  |
| CXCL2 | chemokine (C-X-C motif) ligand 2 | 16.41974 | 0.028929 |
| CCL11 | chemokine C-C motif ligand 11 | 7.532983 | 0.005543 |
| CCL13 | chemokine C-C motif ligand 13 | 3.628935 | 0.013087 |
| CCL15 | chemokine C-C motif ligand 15 | 2.586734 | 0.032332 |
| CCL18 | chemokine C-C motif ligand 18 | 3.307158 | 0.000539 |
| CCL19 | chemokine C-C motif ligand 19 | 6.776819 | 0.002363 |
| CCL20 | chemokine C-C motif ligand 20 | 7.757849 | 0.013194 |
| MGC12815 | chemokine C-C motif ligand 3-like | 8.324342 | 0.000496 |
| CCL4 | chemokine C-C motif ligand 4 | 2.133343 | 0.016948 |
| CCR1 | chemokine C-C motif receptor 1 | 11.1426 | 0.004645 |
| CCRL2 | chemokine C-C motif receptor-like 2 | 3.475314 | 0.002176 |
| CXCL1 | chemokine C-X-C motif ligand 1 | 5.607755 | 0.038256 |
| CXCL10 | chemokine C-X-C motif ligand 10 | 93.2472 | 0.001053 |
| CXCL12 | chemokine C-X-C motif ligand 12 | 2.913055 | 0.042914 |
| CXCL16 | chemokine C-X-C motif ligand 16 | 3.014741 | 0.021417 |
| CXCL3 | chemokine C-X-C motif ligand 3 | 10.56391 | 0.010544 |
| CXCL6 | chemokine C-X-C motif ligand 6 | 75.26501 | 0.000302 |
| CXCL9 | chemokine C-X-C motif ligand 9 | 20.39208 | 0.000157 |
| CXCR4 | chemokine C-X-C motif receptor 4 | 6.524702 | 0.000837 |
| GZMA | Granzyme A | 8.334944 | 0.000488 |
| GZMB | Granzyme B | 4.850156 | 1.27E-05 |
| GADD45GIP1 | growth arrest and DNA-damage-inducible interacting protein 1 | 2.120226 | 0.001007 |
| GAS2L1 | growth arrest-specific 2 like 1 | 2.764013 | 0.003307 |
| HYOU1 | hypoxia up-regulated 1 | 2.394175 | 0.008691 |
| HIF1A | hypoxia-inducible factor 1, alpha | 3.385624 | 5.85E-06 |
| ITLN1 | intelectin 1 | 3.96776 | 0.001214 |
| ICAM1 | intercellular adhesion molecule 1 | 2.166151 | 0.016696 |
| ICAM2 | intercellular adhesion molecule 2 | 2.099826 | 0.003940 |
| IFNGR1 | interferon gamma receptor 1 | 4.737984 | 0.004787 |
| IFNGR2 | interferon gamma receptor 2 | 4.599415 | 9.56E-05 |
| IFITM1 | Interferon induced transmembrane | 2.185137 | 0.000218 |
| IRF1 | interferon regulatory factor 1 | 9.568359 | 0.001036 |
| IRF7 a | interferon regulatory factor 7 | 5.043122 | 0.001125 |
| IRF8 | interferon regulatory factor 8 | 2.668394 | 0.003395 |
| ISG20 | interferon stimulated gene | 19.49925 | 0.027753 |
| IFNG | interferon, gamma | 20.60489 | 0.036753 |
| IFI30 | interferon, gamma-inducible protein | 5.786412 | 1.89E-06 |
| IFI35 | interferon-induced protein 35 | 2.019517 | 0.000186 |
| IFIT1 | interferon-induced protein | 6.132365 | 0.003043 |
| IFIT2 | interferon-induced protein wittetratricopeprepeats 2 | 4.070883 | 0.000943 |
| IFIT3 | interferon-induced protein | 15.1217 | 0.001256 |
| IFITM3 | Interferon-induced transmembrane | 2.604187 | 0.000412 |
| LOC715335 | interferon-stimulated transcription factor | 2.570433 | 0.002441 |
| IL1RAP | interleukin 1 receptor accessory protein | 7.181294 | 5.15E-05 |
| IL1RN | interleukin 1 receptor antagonist | 3.806856 | 3.09E-05 |
| IL1R2 | interleukin 1 receptor, type II | 5.851043 | 0.040485 |
| IL1B | interleukin 1, beta | 11.09087 | 0.000871 |
| IL10RA | interleukin 10 receptor, alpha | 5.000267 | 3.82E-05 |
| IL10RB | interleukin 10 receptor, beta | 4.801634 | 0.001025 |
| IL11 | interleukin 11 | 7.20085 | 0.045333 |
| IL13RA1 | interleukin 13 receptor, alpha 1 | 6.247017 | 0.007434 |
| IL4I1 | interleukin 4 induced 1 | 5.266343 | 0.000784 |
| IL4R | interleukin 4 receptor | 3.836683 | 0.000278 |
| IL6 | interleukin 6 | 49.66955 | 0.007056 |
| IL7R | interleukin 7 receptor | 2.170937 | 0.001704 |
| JAK2 | Janus kinase 2 | 4.204484 | 0.000515 |
| MAPK1 2 | mitogen-activated protein kinase 1 | 6.574861 | 0.010550 |
| MAPK1 1 | mitogen-activated protein kinase 1 | 2.380001 | 0.042555 |
| MAP2K1 | mitogen-activated protein kinase kinase | 3.520587 | 0.001821 |
| MAP2K1IP1 | mitogen-activated protein kinase kinase interacting protein 1 | 4.009142 | 0.011079 |
| MAPBPIP | mitogen-activated protein-binding | 2.29278 | 0.000710 |
| NFKBIA | nuclear factor of kappa light polypeptide | 2.440379 | 0.001139 |
| NFKBIE | nuclear factor of kappa light polypeptide | 4.873672 | 0.003415 |
| NFIL3 | nuclear factor, interleukin 3 regulated | 5.435498 | 0.003948 |
| PECAM1 | platelet/endothelial cell adhesion | 2.370046 | 3.74E-05 |
| RGS1 | regulator of G-protein signaling 1 | 10.50895 | 0.011525 |
| RGS16 | regulator of G-protein signaling 16 | 6.01963 | 0.012563 |
| RGS19 | regulator of G-protein signaling 19 | 4.254197 | 0.000358 |
| RARRES3 | retinoic acid receptor responder | 3.156778 | 0.002140 |
| RARB 1 | retinoic acid receptor, beta | 2.380253 | 0.038216 |
| SAA1 | serum amyloid A1 | 15.03555 | 0.005087 |
| SAA2 | serum amyloid A2 | 11.42842 | 2.24E-06 |
| STAT1 | signal transducer and activator of | 4.908662 | 0.000959 |
| STAT5A | signal transducer and activator of | 6.92806 | 0.022819 |
| SOD2 | superoxide dismutase 2 | 5.741199 | 0.049193 |
| TFPI2 | tissue factor pathway inhibitor | 5.177237 | 2.21E-05 |
| TRAF3 1 | TNF receptor-associated factor | 5.495196 | 0.003237 |
| TNIP1 | TNFAIP3 interacting protein | 3.476122 | 0.041968 |
| TLR4 4 | toll-like receptor 4 | 18.47476 | 0.026277 |
| TLR8 1 | toll-like receptor 8 | 2.168357 | 0.001509 |
| TICAM1 | toll-like receptor adaptor molecule | 3.990993 | 0.024416 |
| TANK 1 | TRAF family member- NFKB activator | 2.338462 | 7.91E-05 |
| TNFSF13A | tumor necrosis factor ligand superfamily | 2.855409 | 5.26E-05 |
| TNFSF13B | tumor necrosis factor ligand superfamily | 4.016602 | 0.003441 |
| TNFRSF1A | tumor necrosis factor receptor superfamily member 1A | 3.500825 | 0.002798 |
| TNFRSF4 | tumor necrosis factor receptor superfamily | 2.734365 | 0.043204 |
| TNFRSF8 | tumor necrosis factor receptor superfamily | 2.480758 | 0.013130 |
| TNF | tumor necrosis factor TNF superfamily | 2.241628 | 0.024740 |
| TNFAIP1 | tumor necrosis factor, alpha-induced | 2.802126 | 0.013558 |
| TNFAIP3 | tumor necrosis factor, alpha-induced | 15.46452 | 0.002116 |

**Table S3**. Rhesus macaque genes that exhibit enhanced expression in week four (early) granuloma lesions relative to normal lung.
