# Supplementary material for: Transcriptional Reprogramming in Nonhuman Primate (Rhesus Macaque) Tuberculosis Granulomas
Source: PLoS One. 2010 Aug 31;5(8):e12266. doi: 10.1371/journal.pone.0012266 (PMC2930844; doi:10.1371/journal.pone.0012266)
Supplement: Table S4 — DNA Microarray Analysis: Immune function genes with significantly reduced expression in Mtb granuloma's relative to non-granulomatous tissue four week's post-infection. Symbol = Official NCBI human gene symbol associated with that gene. P = p value of significance in a t-test. (0.02 MB DOCX) [file pone.0012266.s004.docx]

| **Symbol** | **Gene** | **Av Fold Change (Lesion Lung /Normal Lung)** | ***P*** |
| --- | --- | --- | --- |
| BCL2-associated X protein | BCL2-associated X protein | -2.925243 | 0.000467 |
| BCL6 co-repressor | BCL6 co-repressor | -5.201424 | 4.26E-07 |
| LOC81558 | C/EBP-induced protein | -2.649206 | 0.030963 |
| CACNA2D3 | calcium channel, voltage-dependent, alpha 2/delta 3 subunit | -4.152587 | 0.022083 |
| CAMK2A | calcium/calmodulin-dependent protein kinase | -3.155368 | 0.000737 |
| CAMK2G | calcium/calmodulin-dependent protein kinase | -3.251265 | 0.003843 |
| CaMKIIN | calcium/calmodulin-dependent protein kinase II | -4.441240 | 1.10E-05 |
| CALML3 | calmodulin-like 3 | -2.686527 | 0.010268 |
| CD244 | CD244 natural killer cell receptor 2B4 | -4.118765 | 0.005943 |
| CD47 | CD47 antigen | -2.259324 | 0.001734 |
| CD7 | CD7 antigen | -4.948711 | 0.003326 |
| CCL14 | chemokine C-C motif ligand 14 | -4.095333 | 0.000364 |
| CCL24 | chemokine C-C motif ligand 24 | -2.607648 | 0.006723 |
| CCL25 | chemokine C-C motif ligand 25 | -3.149274 | 0.000326 |
| CSF2 | colony stimulating factor 2 granulocyte-macrophage | -4.887254 | 0.000146 |
| C1QL1 | complement component 1, q subcomponent-like 1 | -3.928221 | 1.85E-06 |
| C1QL2 | complement component 1, q subcomponent-like 2 | -3.996815 | 0.007841 |
| DEFB126 | defensin, beta 126 | -2.640008 | 0.000005 |
| ICAM5 | intercellular adhesion molecule 5, telencephalin | -2.486581 | 0.00629 |
| IL1RL2 | interleukin 1 receptor-like 2 | -3.318512 | 0.018474 |
| IL17RE | interleukin 17 receptor E | -2.081077 | 0.000535 |
| JUNB | jun B proto-oncogene | -2.002621 | 0.018295 |
| JUND | jun D proto-oncogene | -4.481636 | 9.61E-06 |
| MKNK2 | MAP kinase interacting serine/threonine kinase 2 | -2.324548 | 9.89E-05 |
| MARK2 | MAP/microtubule affinity-regulating kinase 2 | -2.328332 | 1.04E-05 |
| MAPK11 | mitogen-activated protein kinase 11 | -4.496749 | 0.002751 |
| MAPK12 | mitogen-activated protein kinase 12 | -2.441641 | 0.000744 |
| MAPK15 | mitogen-activated protein kinase 15 | -2.395669 | 4.07E-05 |
| MAPK7 | mitogen-activated protein kinase 7 | -2.100051 | 0.000246 |
| MAPK8IP3 | mitogen-activated protein kinase 8 interacting protein 3 | -2.821939 | 9.99E-06 |
| MAP3K14 | mitogen-activated protein kinase kinase kinase 14 | -2.855472 | 0.000432 |
| MAP3K6 | mitogen-activated protein kinase kinase kinase 6 | -5.460099 | 2.47E-05 |
| MAPKBP1 | mouse mitogen-activated protein kinase binding protein 1-like | -2.710236 | 0.005831 |
| SOCS2 | suppressor of cytokine signaling 2 | -4.040360 | 2.31E-05 |
| SOCS3 | suppressor of cytokine signaling 3 | -5.400276 | 8.52E-05 |
| TGFBR3 | transforming growth factor, beta receptor III betaglycan, 300kDa | -3.003980 | 0.000048 |

**Table S4.** Rhesus macaque genes that exhibit a diminished expression in week four (early) granulomatous lesions relative to normal lung.
