# Supplementary material for: Transcriptional Reprogramming in Nonhuman Primate (Rhesus Macaque) Tuberculosis Granulomas
Source: PLoS One. 2010 Aug 31;5(8):e12266. doi: 10.1371/journal.pone.0012266 (PMC2930844; doi:10.1371/journal.pone.0012266)
Supplement: Table S5 — This table contains a comprehensive list of all NHP genes with a higher expression in a statistically significant manner, in late (week 13) TB lesions, relative to normal lungs. (0.21 MB PDF) [file pone.0012266.s005.pdf]

| Gene Name | Desccription | Symbol | Granuloma Av M | Granuloma Av Num# Ratio | Normal Av M | Normal Av Num# Ratio | P | Fold Change (Granuloma / Normal) |
|---|---|---|---|---|---|---|---|---|
| NM_181806 | 2-aminoadipic 6-semialdehyde dehydrogenase | NRPS998 | 0.038158073 | 1.026802044 | 2.403936494 | 5.292452786 | 0.021511223 | 5.154306828 |
| NM_002130 | 3-hydroxy-3-methylglutaryl-Coenzyme A synthase 1 | HMGCS1 | 0.49063611 | 1.405064257 | 1.425847182 | 2.686722244 | 0.008846001 | 1.912170373 |
| NM_004670 | 3'-phosphoadenosine 5'-phosphosulfate synthase 2 | PAPSS2 | -0.280538029 | 0.82328393 | 2.57407003 | 5.95487007 | 0.003129406 | 7.233069726 |
| NM_020201 | 5',3'-nucleotidase, mitochondrial | NT5M | -0.438955668 | 0.737668396 | 1.698981083 | 3.246715752 | 0.014685602 | 4.401321474 |
| NM_203326 | 5-azacytidine induced 2 | AZI2 | 0.295701308 | 1.227481523 | 1.554635251 | 2.937594475 | 0.004239594 | 2.393188345 |
| NM_000865 | 5-hydroxytryptamine | HTR1E | -0.272537434 | 0.827862208 | 1.144373954 | 2.210501869 | 0.031435013 | 2.670132599 |
| NM_213621 | 5-hydroxytryptamine | HTR3A | 0.197029083 | 1.146335295 | 1.457736985 | 2.746771662 | 0.007979642 | 2.396132854 |
| BC054816 | 5-methyltetrahydrofolate-homocysteine methyltransferase reductase | | -0.517525785 | 0.698568849 | 1.459685618 | 2.750484205 | 0.036453223 | 3.937312992 |
| NM_012229 | 5'-nucleotidase, cytosolic II | NT5C2 | 0.0861222 | 1.061513118 | 1.640135625 | 3.116951324 | 0.021050956 | 2.936328597 |
| NM_017570 | 5-oxoprolinase | OPLAH | 0.480535736 | 1.395261692 | 1.888086486 | 3.701439599 | 0.01964881 | 2.652864061 |
| NM_007037 | a disintegrin-like and metalloprotease | ADAMTS8 | -0.182340704 | 0.881272014 | 3.967566383 | 15.64431281 | 0.000281446 | 17.75196825 |
| NM_139025 | a disintegrin-like and metalloprotease | ADAMTS13 | 0.093323956 | 1.066825308 | 2.982176177 | 7.90177177 | 0.002065192 | 7.406809447 |
| NM_139057 | a disintegrin-like and metalloprotease | ADAMTS17 | 0.385545533 | 1.306353671 | 2.06858682 | 4.194755783 | 0.021517727 | 3.211041448 |
| NM_005858 | A kinase | AKAP8 | -0.145468404 | 0.904085798 | 1.254691104 | 2.38616053 | 0.019596994 | 2.639307614 |
| NM_007202 | A kinase | AKAP10 | -0.874422376 | 0.545472218 | 1.645276036 | 3.128077021 | 0.003333687 | 5.734622072 |
| NM_139275 | A kinase | AKAP1 | 0.339061228 | 1.264933225 | 1.396754095 | 2.633085 | 0.025525903 | 2.081599999 |
| NM_147150 | A kinase | AKAP2 | -0.224619527 | 0.855820696 | 1.659726758 | 3.159566779 | 0.008330215 | 3.691856009 |
| NM_023928 | acetoacetyl-CoA synthetase | AACS | 0.34492349 | 1.270083625 | 1.565153618 | 2.959090085 | 0.00155022 | 2.329838781 |
| NM_004192 | acetylserotonin O-methyltransferase-like | ASMTL | -0.336375489 | 0.792028644 | 1.162794324 | 2.238906568 | 0.007427506 | 2.826799996 |
| XR_014079 | acid sphingomyelinase-like phosphodiesterase 3B isoform 1 | SMPDL3B | 0.631835507 | 1.549535178 | 2.272164666 | 4.830473675 | 0.033605686 | 3.117369482 |
| NM_006107 | acid-inducible phosphoprotein | OA48-18 | -0.143699879 | 0.905194749 | 2.057716665 | 4.163268674 | 0.004255975 | 4.599307142 |
| NM_032432 | actin binding LIM protein family, member 2 | ABLIM2 | 0.404760045 | 1.3238687 | 1.908933646 | 3.755314267 | 0.04115241 | 2.836621386 |
| NM_014945 | actin binding LIM protein family, member 3 | ABLIM3 | -0.170478498 | 0.888547928 | 3.804217902 | 13.96959124 | 0.000178912 | 15.72182074 |
| NM_001613 | actin, alpha 2, smooth muscle, aorta | ACTA2 | -0.107082642 | 0.928463665 | 1.234877221 | 2.353613165 | 0.000938771 | 2.534954521 |
| NM_004924 | actinin, alpha 4 | ACTN4 | 1.294336514 | 2.452641741 | 2.611849335 | 6.112867666 | 0.038913387 | 2.49236061 |
| NM_001106 | activin A receptor, type IIB | ACVR2B | 0.459026055 | 1.374613521 | 1.569564147 | 2.968150297 | 0.006111641 | 2.159261677 |
| AF389338 | acyl-CoA-desaturase mRNA, complete cds | | -0.535091115 | 0.69011509 | 1.681127066 | 3.206783744 | 0.036793433 | 4.64673761 |
| NM_003500 | acyl-Coenzyme A oxidase 2, branched chain | ACOX2 | -0.16966305 | 0.8890503 | 2.614650587 | 6.124748429 | 0.012971791 | 6.88909101 |
| NM_003917 | adaptor-related protein complex 1, gamma 2 subunit | AP1G2 | -0.236303289 | 0.848917765 | 1.252623108 | 2.382742596 | 0.004053178 | 2.806800251 |
| NM_001282 | adaptor-related protein complex 2, beta 1 subunit | AP2B1 | 0.739723723 | 1.66985603 | 2.997640707 | 7.986927991 | 0.014326529 | 4.783003952 |
| NM_018263 | additional sex combs like 2 | ASXL2 | 0.502655542 | 1.416819076 | 1.50401427 | 2.836308125 | 0.0100986 | 2.001884484 |
| NM_000022 | adenosine deaminase | ADA | -0.227002124 | 0.854408483 | 1.773768569 | 3.419460132 | 0.001846712 | 4.002137385 |
| NM_015833 | adenosine deaminase, RNA-specific, B1 | ADARB1 | 0.279189658 | 1.21351308 | 2.829260136 | 7.10709575 | 0.001562595 | 5.85662888 |
| NM_139247 | adenylate cyclase 4 | ADCY4 | 0.048663519 | 1.034306321 | 1.633676035 | 3.103026538 | 0.021726087 | 3.000104007 |
| NM_001136 | advanced glycosylation end product-specific receptor | AGER | -0.094622108 | 0.936517522 | 3.569482107 | 11.87192606 | 0.000349186 | 12.67667265 |

| | | | | | | | | |
|---|---|---|---|---|---|---|---|---|
| BU933087 | AGENCOURT_10473089 NIH_MGC_127 cDNA clone IMAGE:6673792 5' sequence | | -0.211230483 | 0.863800177 | 2.505290535 | 5.677636691 | 0.00649162 | 6.572858911 |
| CB229350 | AGENCOURT_11571791 NICHD_Rh_Ov1 cDNA clone IMAGE:6885189 5' sequence | | -0.165456379 | 0.891646413 | 1.50005197 | 2.828529015 | 0.00342766 | 3.172254129 |
| NM_024060 | AHNAK nucleoprotein | AHNAK | 0.547389584 | 1.461438971 | 2.57802452 | 5.971215018 | 0.001411955 | 4.085846304 |
| NM_000667 | alcohol dehydrogenase 1A | ADH1A | -0.175638747 | 0.885375436 | 3.567366679 | 11.85453098 | 0.005109439 | 13.38927024 |
| NM_000669 | alcohol dehydrogenase 1C | ADH1C | -0.536401947 | 0.689488337 | 5.13718142 | 35.19214194 | 0.010374586 | 51.0409532 |
| NM_144650 | alcohol dehydrogenase, iron containing, 1 | ADHFE1 | -0.073023476 | 0.950643632 | 3.344992429 | 10.16115454 | 0.002300075 | 10.68871047 |
| NM_001354 | aldo-keto reductase family 1, member C2 | AKR1C2 | -0.657751912 | 0.633865252 | 6.197719623 | 73.40058331 | 0.0254142 | 115.7984021 |
| NM_012067 | aldo-keto reductase family 7, member A3 | AKR7A3 | -0.198357696 | 0.871542126 | 1.748624198 | 3.360379564 | 0.005873089 | 3.855670843 |
| NM_001631 | alkaline phosphatase, intestinal | ALPI | -0.024420797 | 0.983215254 | 1.926000954 | 3.800004045 | 0.020897256 | 3.86487499 |
| NM_000014 | alpha-2-macroglobulin | A2M | 0.209103547 | 1.155969671 | 2.179294796 | 4.529321021 | 0.007833582 | 3.918200568 |
| NM_015120 | Alstrom syndrome 1 | ALMS1 | 0.082616389 | 1.058936727 | 1.293699265 | 2.45155863 | 0.012974186 | 2.315113422 |
| NM_024083 | alveolar soft part sarcoma chromosome region, candidate 1 | ASPSCR1 | -0.308884119 | 0.807265914 | 2.755640595 | 6.753524448 | 0.004884019 | 8.365922964 |
| NM_016228 | aminoadipate aminotransferase | AADAT | -0.31880831 | 0.801731848 | 1.423029851 | 2.681480665 | 0.017282499 | 3.344610385 |
| NM_000031 | aminolevulinate, delta-, dehydratase | ALAD | 0.574767372 | 1.489437279 | 1.581894371 | 2.993626782 | 0.026760361 | 2.009904562 |
| NM_000481 | aminomethyltransferase | AMT | -0.6829911 | 0.62287255 | 2.392929872 | 5.25222919 | 0.002848864 | 8.432269475 |
| NM_001008218 | amylase, alpha 1B; salivary | AMY1B | 0.177825799 | 1.131177869 | 1.960214449 | 3.891198154 | 0.003218519 | 3.439952514 |
| NM_000699 | amylase, alpha 2A; pancreatic | AMY2A | 0.068919389 | 1.048930717 | 4.744324908 | 26.80304322 | 0.00148372 | 25.55272983 |
| NM_020978 | amylase, alpha 2B; pancreatic | AMY2B | -0.004522639 | 0.996870054 | 3.555498517 | 11.75741108 | 0.007219036 | 11.79432669 |
| NM_133175 | amyloid beta | APBB3 | -0.204608297 | 0.867774262 | 1.987916978 | 3.966638649 | 0.000317981 | 4.571048973 |
| NM_013367 | anaphase promoting complex subunit 4 | ANAPC4 | 0.500001628 | 1.414215158 | 2.148222427 | 4.432812767 | 0.018992996 | 3.134468431 |
| XR_011510 | anaphase-promoting complex subunit 5 | ANAPC5 | 0.033893727 | 1.023771484 | 2.040557589 | 4.114045043 | 0.000283124 | 4.018518886 |
| NM_012103 | ancient ubiquitous protein 1 | AUP1 | 0.10812469 | 1.077826296 | 1.446180358 | 2.724856695 | 0.001120769 | 2.528103745 |
| NM_139290 | angiopoietin 1 | ANGPT1 | 0.247652644 | 1.187273773 | 3.68598827 | 12.87042929 | 0.014516323 | 10.8403214 |
| NM_012098 | angiopoietin-like 2 | ANGPTL2 | 0.580790543 | 1.495668595 | 1.503756852 | 2.835802093 | 0.018535735 | 1.896009652 |
| NM_020987 | ankyrin 3, node of Ranvier | ankyrin G | 0.003391956 | 1.002353891 | 1.718192399 | 3.290239035 | 0.007559765 | 3.282512359 |
| NM_145804 | ankyrin repeat and BTB | ABTB2 | 0.109411888 | 1.07878838 | 2.45574996 | 5.48598227 | 0.045324685 | 5.085318281 |
| NM_015245 | ankyrin repeat and sterile alpha motif domain containing 1 | ANKS1 | 0.441892307 | 1.358384882 | 2.489501469 | 5.615838586 | 0.031188314 | 4.134202802 |
| XR_012049 | ankyrin repeat and sterile alpha motif domain containing 3 | ANKS3 | -0.059060077 | 0.959889288 | 1.943520602 | 3.846431463 | 0.000313598 | 4.007161567 |
| NM_017664 | ankyrin repeat domain 10 | ANKRD10 | -0.348167191 | 0.785581472 | 1.786785198 | 3.450451605 | 0.006300266 | 4.392226304 |
| NM_015158 | ankyrin repeat domain 15 | ANKRD15 | 0.261652552 | 1.198851156 | 1.483876775 | 2.796993256 | 0.002393541 | 2.333061316 |
| NM_020349 | ankyrin repeat domain 2 | ANKRD2 | 0.152897039 | 1.111799808 | 1.417198514 | 2.670664063 | 0.01946753 | 2.402108764 |
| NM_144994 | ankyrin repeat domain 23 | ANKRD23 | -0.316814296 | 0.802840724 | 1.723162628 | 3.301593786 | 0.00174948 | 4.112389528 |
| NM_022096 | ankyrin repeat domain 5 | ANKRD5 | 5.066733383 | 33.51496177 | 7.987050527 | 253.7124537 | 0.006060967 | 7.570125111 |
| NM_014942 | ankyrin repeat domain 6 | ANKRD6 | -0.105597367 | 0.929420024 | 1.348698869 | 2.546823301 | 0.023389006 | 2.740228568 |
| NM_005139 | annexin A3 | ANXA3 | 0.003824007 | 1.002654115 | 2.153499006 | 4.449055223 | 0.000771655 | 4.437278176 |
| NM_001153 | annexin A4 | ANXA4 | 0.361269339 | 1.284555602 | 2.600608387 | 6.065423525 | 1.33E-05 | 4.721806916 |
| NM_001630 | annexin A8 | ANXA8 | 0.011315342 | 1.007874036 | 3.919843936 | 15.13528499 | 0.00028402 | 15.01704027 |
| NM_005876 | aortic preferentially expressed protein 1 | APEG1 | 0.041456298 | 1.029152159 | 3.099983792 | 8.574091372 | 0.004450503 | 8.331218369 |
| CN646438 | APBB1 | APBB1 | 0.01293209 | 1.009004137 | 2.460868315 | 5.505479861 | 0.001275163 | 5.456350137 |
| NM_014977 | apoptotic chromatin condensation inducer 1 | ACIN1 | 0.238028566 | 1.179379944 | 1.939569 | 3.835910342 | 0.014886212 | 3.252480561 |

| | | | | | | | | |
|---|---|---|---|---|---|---|---|---|
| NM_003603 | Arg/Abl-interacting protein ArgBP2 | ARGBP2 | 0.43986098 | 1.35647361 | 2.840188269 | 7.161135021 | 0.036341253 | 5.279229148 |
| NM_000490 | arginine vasopressin | AVP | -0.250435142 | 0.840642824 | 1.643074942 | 3.123308209 | 0.028834632 | 3.715380801 |
| NM_000048 | argininosuccinate lyase | ASL | -0.304775745 | 0.809568047 | 1.269163971 | 2.410218551 | 0.001556687 | 2.977166107 |
| CO647185 | ARH | ARH | -0.478316936 | 0.717814546 | 1.087680714 | 2.125320938 | 0.004188162 | 2.960821775 |
| NM_006321 | ariadne homolog 2 | ARIH2 | 0.499976441 | 1.414190469 | 2.328343787 | 5.022284608 | 0.012344572 | 3.551349495 |
| NM_004313 | arrestin, beta 2 | ARRB2 | 0.153469603 | 1.112241138 | 1.542693255 | 2.913378722 | 0.002364468 | 2.61937688 |
| NM_057091 | artemin | ARTN | -0.157320877 | 0.8966887 | 3.137788069 | 8.80173581 | 0.012847291 | 9.815821042 |
| NM_001086 | arylacetamide deacetylase | AADAC | -0.451896766 | 0.731081036 | 3.801853329 | 13.94671387 | 0.013605775 | 19.07683715 |
| NM_020453 | ATPase, Class V, type 10D | ATP10D | -0.376149612 | 0.770491202 | 2.404094866 | 5.293033796 | 0.00943644 | 6.869687522 |
| NM_015205 | ATPase, Class VI, type 11A | ATP11A | -0.088378238 | 0.940579479 | 1.410188092 | 2.657718107 | 0.00032977 | 2.825617788 |
| NM_144583 | ATPase, H+ transporting, lysosomal 42kDa, V1 subunit C isoform 2 | ATP6V1C2 | 0.651165017 | 1.570435855 | 3.779842627 | 13.73554859 | 0.001046588 | 8.746328955 |
| NM_007168 | ATP-binding cassette, sub-family A | ABC1 | -0.480843635 | 0.716558484 | 2.160007738 | 4.469172523 | 0.044319814 | 6.236996174 |
| XR_012919 | ATP-binding cassette, sub-family A member 4 | ABCA4 | 0.605633072 | 1.521646324 | 2.499148253 | 5.653515507 | 0.043167229 | 3.715393924 |
| NM_000352 | ATP-binding cassette, sub-family C | CFTR/MRP | 0.778007798 | 1.714761341 | 3.047109191 | 8.265540657 | 0.006809348 | 4.820228016 |
| NM_003786 | ATP-binding cassette, sub-family C | CFTR/MRP | -0.46781505 | 0.723058836 | 2.347320463 | 5.088782261 | 0.006805744 | 7.03785364 |
| NM_005688 | ATP-binding cassette, sub-family C | CFTR/MRP | 0.163457684 | 1.119968134 | 1.702949338 | 3.255658413 | 0.012786929 | 2.906920576 |
| XR_012834 | ATP-binding cassette, sub-family D, member 3 | ABCD3 | -2.091506079 | 0.234635615 | 1.898025741 | 3.727028215 | 0.04037273 | 15.88432436 |
| NM_001698 | AU RNA binding protein/enoyl-Coenzyme A hydratase | AUH | 0.115002456 | 1.082976889 | 2.034479682 | 4.096749504 | 0.001736505 | 3.782859584 |
| XM_495839 | bA486O223 | RPS3A | 0.836420931 | 1.785614851 | 1.553114365 | 2.934499303 | 0.045383324 | 1.64341112 |
| NM_022161 | baculoviral IAP repeat-containing 7 | BIRC7 | -0.581130272 | 0.668439886 | 4.714609736 | 26.25662784 | 0.027209967 | 39.28046245 |
| NM_017451 | BAI1-associated protein 2 | BAIAP2 | 0.198105807 | 1.147191156 | 1.523534318 | 2.874944923 | 0.0174113 | 2.506073123 |
| NM_033028 | Bardet-Biedl syndrome 4 | BBS4 | -0.123518944 | 0.917945914 | 1.808858651 | 3.503649971 | 0.00513552 | 3.816837046 |
| NM_052842 | BCL2-like 12 | BCL2L12 | 0.080150587 | 1.057128377 | 1.190088265 | 2.281667021 | 1.02E-06 | 2.158363233 |
| NM_020926 | BCL6 co-repressor | BCOR | 0.36686238 | 1.289545234 | 1.670356955 | 3.182933367 | 0.015794119 | 2.468260347 |
| NM_001195 | beaded filament structural protein 1, filensin | BFSP1 | -0.397060897 | 0.759403789 | 1.994540333 | 3.984891195 | 0.019465019 | 5.247394407 |
| NM_005434 | BENE protein | BENE | -0.199452577 | 0.870880952 | 1.8932449 | 3.714697933 | 0.013116157 | 4.265448595 |
| CN803179 | BEX2 | BEX2 | 0.133176896 | 1.096706053 | 2.158195892 | 4.46356332 | 0.000601674 | 4.069972357 |
| NM_018476 | brain expressed, X-linked 1 | BEX1 | 0.037831597 | 1.026569709 | 1.306566066 | 2.473520859 | 0.000587633 | 2.409501116 |
| NM_015964 | brain specific protein | CGI-38 | -0.405946645 | 0.754740896 | 2.090205465 | 4.258087114 | 0.004603903 | 5.641786654 |
| NM_001190 | branched chain aminotransferase 2, mitochondrial | BCAT2 | 0.118767168 | 1.085806608 | 2.004348418 | 4.012074563 | 0.002517696 | 3.695017633 |
| NM_001519 | BRF1 homolog, subunit of RNA polymerase III transcription initiation factor IIIB | BRF1 | 0.611424944 | 1.527767434 | 2.312665113 | 4.96799979 | 0.014966243 | 3.251803697 |
| NM_018688 | bridging integrator 3 | BIN3 | 0.587771153 | 1.502923059 | 1.874620908 | 3.667052467 | 0.017739511 | 2.439946905 |
| NM_006696 | bromodomain containing 8 | BRD8 | -0.239890343 | 0.846809675 | 2.018658985 | 4.052069686 | 0.001236327 | 4.785100839 |
| NM_033254 | brother of CDO | BOC | -0.132983221 | 0.911943771 | 2.949463828 | 7.724619273 | 0.001743075 | 8.470499518 |
| XR_010742 | BTAF1 RNA polymerase II, B-TFIID transcription factor-associated, 170kDa | BTAF1 | 0.323036786 | 1.250960977 | 1.684788249 | 3.214932059 | 0.046942825 | 2.569969902 |
| NM_006994 | butyrophilin, subfamily 3, member A3 | BTN3A3 | 0.021994267 | 1.015362066 | 1.840894666 | 3.582321119 | 0.001730739 | 3.528121878 |
| NM_032156 | C1q domain containing 1 | C1QDC1 | 0.417403249 | 1.335521544 | 2.608399725 | 6.098268725 | 0.000216649 | 4.566207675 |
| CN805335 | C7orf28B | C7orf28B | 0.242558748 | 1.183089115 | 2.002254698 | 4.006256239 | 0.004921433 | 3.386267516 |
| NM_017954 | Ca2+-dependent activator protein for secretion 2 | CADPS2 | 0.280807443 | 1.214874632 | 1.992005184 | 3.977894969 | 0.003881626 | 3.274325485 |
| NM_198397 | calcium channel, voltage-dependent, alpha 1G subunit | CACNA1G | -1.844880256 | 0.278378509 | 2.756299915 | 6.756611553 | 0.037299311 | 24.27131167 |

| | | | | | | | | |
|---|---|---|---|---|---|---|---|---|
| XR_014749 | calcium channel, voltage-dependent, alpha 1H subunit | CACNA1H | -0.174061827 | 0.886343714 | 2.375004352 | 5.187373865 | 0.019338062 | 5.852553341 |
| NM_001003406 | calcium channel, voltage-dependent, alpha 1I subunit | CACNA1I | 0.471516474 | 1.386566177 | 1.326620692 | 2.50814489 | 0.018350717 | 1.808889422 |
| NM_199248 | calcium channel, voltage-dependent, beta 1 subunit | CACNB1 | -0.179730018 | 0.882868198 | 1.032654513 | 2.04578497 | 0.024751896 | 2.317203151 |
| NM_000725 | calcium channel, voltage-dependent, beta 3 subunit | CACNB3 | 0.394550386 | 1.314533024 | 2.634647215 | 6.210232212 | 0.005232628 | 4.724287712 |
| NM_018584 | calcium/calmodulin-dependent protein kinase II | CaMKIINalpha | -0.273250969 | 0.827452861 | 3.098220363 | 8.563617529 | 0.002369383 | 10.34937207 |
| NM_001748 | calpain 2, | CAPN2 | 0.128642955 | 1.093264857 | 2.05585366 | 4.157895962 | 0.003101739 | 3.803191821 |
| NM_000070 | calpain 3, | CAPN3 | -0.07856368 | 0.946999993 | 1.6801577 | 3.204629788 | 0.001252468 | 3.383980794 |
| NM_006615 | calpain 9 | CAPN9 | -0.739967571 | 0.598752811 | 1.722578847 | 3.300258079 | 0.003965738 | 5.511887407 |
| NM_032607 | cAMP responsive element binding protein 3-like 3 | CREB3L3 | 0.429234482 | 1.346518902 | 3.21957904 | 9.315150253 | 0.0016161 | 6.917949863 |
| NM_001266 | carboxylesterase 1 | CES1 | -0.382632771 | 0.76703655 | 2.949164109 | 7.723014653 | 9.97E-05 | 10.06863969 |
| NM_080385 | carboxypeptidase A5 | CPA5 | 0.963925476 | 1.95061016 | 1.694226438 | 3.236033265 | 0.020946214 | 1.658985138 |
| NM_194293 | cardiomyopathy associated 1 | CMYA1 | 0.47496149 | 1.389881119 | 2.140611967 | 4.409490497 | 0.038606552 | 3.172566658 |
| NM_014550 | caspase recruitment domain family, member 10 | CARD10 | -0.819538359 | 0.566623225 | 1.438129697 | 2.709693542 | 0.003073822 | 4.782178743 |
| NM_016382 | CD244 natural killer cell receptor 2B4 | CD244 | -0.28757332 | 0.819278965 | 3.308618438 | 9.908168735 | 0.003520039 | 12.09376679 |
| CO726190 | CD36 | CD36 | -0.42884665 | 0.742855417 | 1.441040477 | 2.715166136 | 0.009526981 | 3.655039829 |
| NM_001777 | CD47 antigen | CD47 | 0.208611758 | 1.155575689 | 1.973007794 | 3.925857464 | 0.026369276 | 3.397317459 |
| NM_134445 | CD99 antigen-like 2 | CD99L2 | 1.160314095 | 2.235060828 | 2.558356538 | 5.890362972 | 0.031870372 | 2.635437433 |
| NM_001826 | CDC28 protein kinase regulatory subunit 1B | CKS1B | 0.363602457 | 1.286634659 | 1.570889316 | 2.970877905 | 0.00506583 | 2.309029905 |
| NM_004071 | CDC-like kinase 1 | CLK1 | -0.30026843 | 0.812101282 | 2.56645392 | 5.923516635 | 0.001394413 | 7.294061426 |
| NM_001291 | CDC-like kinase 2 | CLK2 | -0.01390854 | 0.990405657 | 1.722398591 | 3.299845757 | 0.001073914 | 3.331812307 |
| NM_016408 | CDK5 regulatory subunit associated protein 1 | CDK5RAP1 | 0.03196805 | 1.022405888 | 1.897016896 | 3.724422897 | 0.019084601 | 3.642802668 |
| AK125739 | cDNA FLJ43751 fis, clone TESTI2034953, moderately 88-kDa Golgi protein | GM88 | 0.381800068 | 1.302966569 | 2.442472063 | 5.435723464 | 0.028160532 | 4.17180578 |
| NM_001263 | CDP-diacylglycerol synthase | CDS1 | 0.455533391 | 1.371289702 | 2.219113934 | 4.656073828 | 0.00937978 | 3.395397647 |
| NM_178569 | CEI protein | CEI | 0.019208296 | 1.013403205 | 1.475996702 | 2.781757585 | 0.014684505 | 2.74496624 |
| NM_022481 | centaurin, delta 3 | CENTD3 | -0.780931639 | 0.581990844 | 4.04913548 | 16.55431581 | 0.041140141 | 28.44428908 |
| XR_010491 | centaurin, gamma-like family, member 1 | CTGLF11P | 0.039119036 | 1.027486213 | 2.377428687 | 5.196098167 | 0.022735778 | 5.057097702 |
| NM_001812 | centromere protein C 1 | CENPC1 | -0.576135745 | 0.670757997 | 3.166995879 | 8.981745721 | 0.0395959 | 13.3904415 |
| NM_007018 | centrosomal protein 1 | CEP1 | -0.322302733 | 0.799792283 | 1.461243087 | 2.753455108 | 0.001551177 | 3.442712772 |
| NM_018069 | centrosomal protein 192 kDa | Cep192 | -0.265036318 | 0.832177785 | 2.785686497 | 6.895649745 | 0.002455313 | 8.286269916 |
| NM_016302 | cereblon | CRBN | 0.494751321 | 1.40907785 | 1.500769993 | 2.829937111 | 0.049271168 | 2.008361079 |
| CN801659 | CGI-115 | CGI-115 | -0.144281451 | 0.904829925 | 2.269428727 | 4.821321804 | 0.000947604 | 5.32842877 |
| NM_032205 | CGI-72 protein | CGI-72 | -0.107759264 | 0.928028319 | 1.716809489 | 3.287086654 | 0.01636821 | 3.542011151 |
| NM_002988 | chemokine | C-C motif ligand 18 | -1.418743397 | 0.374037962 | 1.509516458 | 2.847145965 | 0.001599602 | 7.611917116 |
| NM_148888 | chemokine | C-C motif ligand 25 | -0.137497071 | 0.909094975 | 1.343645153 | 2.537917465 | 0.002410203 | 2.791696726 |
| NM_178818 | chemokine-like factor super family 4 | CKLFSF4 | 0.849671069 | 1.802090006 | 1.873451994 | 3.664082516 | 0.011139505 | 2.033240573 |
| NM_001822 | chimerin | CHN1 | -0.08018659 | 0.945935297 | 1.768308441 | 3.406543056 | 0.011247418 | 3.6012432 |
| NM_004366 | chloride channel 2 | CLCN2 | 0.63445316 | 1.552349235 | 1.120357954 | 2.174009062 | 0.009586553 | 1.400463899 |

| | | | | | | | | | |
|---|---|---|---|---|---|---|---|---|---|
| NM_176875 | cholecystokinin B receptor | CCKBR | 0.855605025 | 1.809517456 | 1.799065398 | 3.479947156 | 0.025831087 | 1.923135444 |
| XR_014109 | Choline | Choline | -0.0241873 | 0.983374398 | 1.219816028 | 2.329170138 | 0.000440936 | 2.368548686 |
| NM_001277 | choline kinase alpha | CHKA | -0.315014257 | 0.803843047 | 1.237858873 | 2.358482462 | 0.010870805 | 2.934008661 |
| NM_020991 | chorionic somatomammotropin hormone 2 | CSH2 | 0.159077679 | 1.11657308 | 1.902163887 | 3.737733962 | 0.03179385 | 3.347504994 |
| NM_175709 | chromobox homolog 7 | CBX7 | -0.122865904 | 0.918361519 | 2.112580014 | 4.324639915 | 0.03234536 | 4.709082236 |
| NM_017673 | chromosome 1 open reading frame 26 | C1orf26 | 0.026315241 | 1.018407706 | 2.400979598 | 5.281616673 | 0.031127315 | 5.186151521 |
| NM_015997 | chromosome 1 open reading frame 66 | C1orf66 | 0.430724537 | 1.347910342 | 2.654131963 | 6.294675309 | 0.002340527 | 4.669951044 |
| NM_173554 | chromosome 10 open reading frame 107 | C10orf107 | 0.527618275 | 1.4415474 | 3.492300436 | 11.25348885 | 0.044356206 | 7.806534041 |
| XM_374765 | chromosome 10 open reading frame 18 | C10orf18 | -0.887116128 | 0.540693857 | 1.525043269 | 2.877953474 | 0.004418942 | 5.322704219 |
| NM_032709 | chromosome 10 open reading frame 33 | C10orf33 | -0.425632445 | 0.744512282 | 2.62588377 | 6.172623387 | 0.001347419 | 8.290828152 |
| NM_018185 | chromosome 13 open reading frame 17 | C13orf17 | -1.634870686 | 0.321999268 | 1.668248213 | 3.178284372 | 0.018984472 | 9.870470799 |
| NM_020195 | chromosome 14 open reading frame 124 | C14orf124 | -0.227479533 | 0.854125794 | 1.336994759 | 2.526245346 | 0.007464558 | 2.957697056 |
| NM_031427 | chromosome 14 open reading frame 168 | C14orf168 | 0.182101669 | 1.134535435 | 2.604210951 | 6.080588463 | 0.034833204 | 5.359540368 |
| NM_017791 | chromosome 14 open reading frame 58 | C14orf58 | 0.109277985 | 1.078688258 | 3.088592484 | 8.506658178 | 0.001170986 | 7.886113634 |
| NM_00100756<br>0 | chromosome 14 open reading frame 70 | C14orf70 | 0.674818494 | 1.596395917 | 1.336025471 | 2.524548636 | 0.000976375 | 1.581405095 |
| NM_017815 | chromosome 14 open reading frame 94 | C14orf94 | 0.476089052 | 1.390967828 | 1.447973121 | 2.728244839 | 0.020344027 | 1.961400389 |
| NM_032140 | chromosome 16 open reading frame 48 | C16orf48 | -0.128991254 | 0.914470633 | 2.383050146 | 5.216384251 | 2.82E-05 | 5.704266559 |
| NM_033520 | chromosome 19 open reading frame 33 | C19orf33 | -0.337166 | 0.791594778 | 1.907772962 | 3.752294238 | 0.00384039 | 4.74017053 |
| NM_080625 | chromosome 20 open reading frame 160 | C20orf160 | -0.739666977 | 0.598877577 | 2.071890206 | 4.204371657 | 0.011746949 | 7.020419228 |
| NM_006462 | chromosome 20 open reading frame 18 | C20orf18 | -0.042442172 | 0.971009845 | 1.287526939 | 2.441092457 | 0.000543503 | 2.513972922 |
| NM_018257 | chromosome 20 open reading frame 36 | C20orf36 | 0.273565818 | 1.208791833 | 1.773317292 | 3.418390687 | 0.009159549 | 2.827939926 |
| NM_080574 | chromosome 20 open reading frame 70 | C20orf70 | 0.627451753 | 1.544833926 | 1.942977627 | 3.844984085 | 0.023533507 | 2.488930376 |
| NM_022760 | chromosome 20 open reading frame 81 | C20orf81 | -0.169673644 | 0.889043772 | 1.823472243 | 3.539320092 | 0.000304456 | 3.981041433 |
| NM_016589 | chromosome 3 open reading frame 1 | C3orf1 | -0.013900244 | 0.990411352 | 1.779950661 | 3.434144299 | 0.016829651 | 3.467391899 |
| NM_032870 | chromosome 6 open reading frame 111 | C6orf111 | -0.374153156 | 0.771558175 | 2.274096138 | 4.836945015 | 0.006082597 | 6.269060677 |
| NM_138409 | chromosome 6 open reading frame 117 | C6orf117 | -0.058094935 | 0.960531655 | 2.137723055 | 4.400669588 | 0.000402608 | 4.581493556 |
| NM_025257 | chromosome 6 open reading frame 29 | C6orf29 | 0.617831033 | 1.534566359 | 1.934859593 | 3.823409149 | 0.008027909 | 2.491524154 |
| NM_017633 | chromosome 6 open reading frame 37 | C6orf37 | -0.056372057 | 0.961679415 | 1.244428451 | 2.369246737 | 0.00547364 | 2.463655455 |
| NM_024581 | chromosome 6 open reading frame 60 | C6orf60 | -1.078594424 | 0.473489906 | 2.575505285 | 5.960797177 | 0.009447115 | 12.58906917 |
| NM_015949 | chromosome 7 open reading frame 20 | C7orf20 | -0.483520821 | 0.715230012 | 1.714910089 | 3.282761841 | 0.004461214 | 4.589798786 |
| NM_024315 | chromosome 7 open reading frame 23 | C7orf23 | -0.152879281 | 0.899453571 | 2.263744973 | 4.802364716 | 0.001903417 | 5.339202457 |
| NM_178829 | chromosome 7 open reading frame 34 | C7orf34 | 0.795008885 | 1.73508806 | 2.08401575 | 4.239857421 | 0.008622877 | 2.443597832 |
| NM_025232 | chromosome 8 open reading frame 20 | C8orf20 | 0.142894573 | 1.104118159 | 1.120224458 | 2.173807905 | 0.000754332 | 1.968818181 |
| NM_032818 | chromosome 9 open reading frame 100 | C9orf100 | 0.691504371 | 1.614966648 | 1.935475181 | 3.825040917 | 0.000784184 | 2.368495301 |
| NM_016446 | chromosome 9 open reading frame 127 | C9orf127 | -1.048251026 | 0.48355402 | 1.357715112 | 2.562789726 | 0.033013595 | 5.299903672 |
| NM_178448 | chromosome 9 open reading frame 140 | C9orf140 | 0.506042267 | 1.420148964 | 2.335014786 | 5.045561346 | 0.005006891 | 3.552839508 |
| NM_032937 | chromosome 9 open reading frame 37 | C9orf37 | 0.850417307 | 1.803022383 | 2.040112389 | 4.112775687 | 0.000568143 | 2.281045275 |
| NM_032012 | chromosome 9 open reading frame 5 | C9orf5 | 0.273425916 | 1.208674618 | 1.566244594 | 2.961328617 | 0.049125724 | 2.450062716 |
| NM_004816 | chromosome 9 open reading frame 61 | C9orf61 | -0.735923348 | 0.600433615 | 3.424214666 | 10.73473496 | 0.00035168 | 17.87830443 |
| XM_371227 | ciliary rootlet coiled-coil, rootletin | CROCC | 0.716791893 | 1.643523282 | 2.308004807 | 4.951977664 | 0.009591707 | 3.013025564 |
| NM_020770 | cingulin | CGN | -0.113159812 | 0.924560856 | 2.584187651 | 5.996778356 | 0.00044976 | 6.486082898 |
| NM_006697 | cisplatin resistance associated | CRA | -0.278403243 | 0.824503062 | 2.365287578 | 5.152553495 | 0.003604991 | 6.249283638 |
| NM_001835 | clathrin, heavy polypeptide-like 1 | CLTCL1 | -0.471918395 | 0.721005218 | 2.314484659 | 4.974269452 | 0.018583241 | 6.899075524 |
| NM_014343 | claudin 15 | CLDN15 | -0.106442289 | 0.928875864 | 1.173853742 | 2.256135541 | 0.001427291 | 2.428888109 |

| | | | | | | | | |
|---|---|---|---|---|---|---|---|---|
| NM_001002026 | claudin 18 | CLDN18 | -0.451434053 | 0.731315552 | 1.611547589 | 3.055794632 | 0.007421724 | 4.178489878 |
| XR_011108 | cleavage and polyadenylation specific factor 1, 160kDa | CPSF1 | -0.300494202 | 0.811974203 | 1.760679076 | 3.388575875 | 0.007042525 | 4.173255582 |
| NM_032179 | cleavage and polyadenylation specific factor 3-like | CPSF3L | -0.204176991 | 0.86803373 | 1.128060968 | 2.185647842 | 0.002082284 | 2.517929622 |
| NM_016438 | CLST 11240 protein | CLST11240 | 0.012870615 | 1.008961143 | 2.089291645 | 4.255390844 | 0.000536281 | 4.21759636 |
| NM_001992 | coagulation factor II | F2R | -1.710151015 | 0.305628076 | 1.903767073 | 3.741889803 | 0.008041888 | 12.24327899 |
| NM_025103 | coiled-coil domain containing 2 | CCDC2 | 0.214769878 | 1.160518787 | 1.845989965 | 3.594995501 | 0.000641018 | 3.097748646 |
| NM_004645 | coilin | COIL | 0.260369772 | 1.197785665 | 1.486329464 | 2.801752396 | 0.02075543 | 2.339109974 |
| NM_001844 | collagen, type II, alpha 1 | COL2A1 | -0.23556457 | 0.849352557 | 1.646286234 | 3.130268118 | 0.004189762 | 3.685475592 |
| NM_000092 | collagen, type IV, alpha 4 | COL4A4 | 0.108042998 | 1.077765266 | 3.428190371 | 10.76435799 | 0.029503246 | 9.987664596 |
| NM_000495 | collagen, type IV, alpha 5 | COL4A5 | 0.578501011 | 1.493296877 | 4.08787478 | 17.00485478 | 0.003890587 | 11.38745754 |
| NM_001847 | collagen, type IV, alpha 6 | COL4A6 | -0.047314711 | 0.967735901 | 3.187985881 | 9.113377827 | 0.004801858 | 9.41721581 |
| NM_058175 | collagen, type VI, alpha 2 | COL6A2 | 0.571813822 | 1.486391155 | 2.111491408 | 4.321377928 | 0.011107375 | 2.907295239 |
| XR_014262 | collagen-like protein | LOC719698 | 0.25763657 | 1.195518595 | 1.438761734 | 2.710880905 | 0.016097124 | 2.267535542 |
| XR_014262 | collagen-like protein | LOC720707 | 0.199565794 | 1.148352685 | 1.746998433 | 3.356594903 | 0.004034244 | 2.922965172 |
| NM_030781 | collectin sub-family member 12 | COLEC12 | 0.043916756 | 1.030908833 | 2.015989241 | 4.044578162 | 0.004340573 | 3.923313132 |
| NM_000758 | colony stimulating factor 2 | CSF2 | 0.389845246 | 1.310252849 | 1.935530051 | 3.825186399 | 0.002795472 | 2.919426126 |
| XR_013448 | COMM domain containing 7 | COMM | 0.126477242 | 1.091624926 | 1.618213713 | 3.069946923 | 0.019337579 | 2.81227265 |
| NM_182528 | complement component 1, q subcomponent-like 2 | C1QL2 | 0.650798417 | 1.570036846 | 3.613107472 | 12.23640172 | 0.041103524 | 7.793703537 |
| NM_001735 | complement component 5 | C5 | -0.275882445 | 0.825944962 | 2.433396088 | 5.401634742 | 0.007506564 | 6.539945142 |
| XR_013572 | Connector enhancer of kinase suppressor of ras 1 | hCNK1 | -0.205448467 | 0.867269052 | 2.392870723 | 5.252013858 | 0.000851245 | 6.055806841 |
| NM_153368 | connexin401 | CX401 | 1.051548263 | 2.07275308 | 1.73258556 | 3.323228651 | 0.002519168 | 1.603292106 |
| NM_153634 | copine VIII | CPNE8 | 0.150227592 | 1.109744525 | 1.605146168 | 3.04226572 | 0.009097353 | 2.741410885 |
| NM_015198 | cordon-bleu homolog | COBL | 0.406422881 | 1.325395459 | 2.611203773 | 6.110132953 | 0.001121668 | 4.610045184 |
| NM_005231 | cortactin | CTTN | -0.212245611 | 0.863192592 | 1.817688542 | 3.525159515 | 0.002906007 | 4.083862104 |
| NM_001882 | corticotropin releasing hormone binding protein | CRHBP | 0.635171469 | 1.553122333 | 2.925337449 | 7.596513598 | 0.004875951 | 4.891123794 |
| CN647114 | COX7C | COX7C | -0.042235263 | 0.971149115 | 1.245407508 | 2.370855126 | 0.006297206 | 2.441288459 |
| NM_001338 | coxsackie virus and adenovirus receptor | CXADR | 0.700839497 | 1.625450359 | 2.059164736 | 4.167449554 | 0.024412491 | 2.563873779 |
| NM_139161 | crumbs homolog 3 | CRB3 | -0.122799414 | 0.918403844 | 1.349572921 | 2.548366754 | 0.002035898 | 2.77477797 |
| NM_004075 | cryptochrome 1 | CRY1 | 0.193576578 | 1.143595286 | 1.172525877 | 2.254059935 | 0.017303565 | 1.971029404 |
| NM_001885 | crystallin, alpha B | CRYAB | -0.402461627 | 0.756566275 | 1.993509781 | 3.982045708 | 0.012333936 | 5.26331379 |
| NM_144727 | crystallin, gamma N | CRYGN | -0.515485221 | 0.699557611 | 2.679145702 | 6.404765289 | 0.007206988 | 9.1554508 |
| CK231978 | CSHL1 | CSHL1 | 0.023576478 | 1.016476229 | 1.628406044 | 3.091712234 | 0.000684681 | 3.041598166 |
| NM_005808 | CTD | carboxy-terminal domain | 0.163744646 | 1.120190925 | 1.525748223 | 2.879360091 | 0.029725789 | 2.570419048 |
| CN803652 | CTGF | CTGF | -0.907266717 | 0.533194307 | 1.935745641 | 3.825758061 | 0.010870708 | 7.175166746 |
| NM_182905 | CXYorf1-related protein | DKFZp434K1323 | 0.154981204 | 1.113407112 | 1.746775671 | 3.356076661 | 0.040982851 | 3.014240366 |
| NM_198943 | CXYorf1-related protein | MGC52000 | -0.316834536 | 0.80282946 | 1.318808022 | 2.494599167 | 0.000255587 | 3.107259127 |
| NM_005190 | cyclin C | CCNC | -0.480807645 | 0.71657636 | 5.547947829 | 46.7841465 | 0.042265518 | 65.28843137 |

| | | | | | | | | |
|---|---|---|---|---|---|---|---|---|
| NM_021145 | cyclin D binding myb-like transcription factor 1 | cyclin D binding myb-like transcription factor 1 | -0.943908577 | 0.519822657 | 2.100138317 | 4.287504891 | 0.010370504 | 8.248014651 |
| NM_020307 | cyclin L1 | CCNL1 | -0.13781541 | 0.9088944 | 2.217763357 | 4.651717089 | 0.001644157 | 5.117995104 |
| XR_013257 | cyclin M1 | CCNM1 | 0.817292257 | 1.762095672 | 2.49361702 | 5.631881661 | 0.027252088 | 3.196127061 |
| NM_058241 | cyclin T2 | CCNT2 | -0.690231761 | 0.619754282 | 1.617096579 | 3.067570667 | 0.03543933 | 4.949656269 |
| NM_003674 | cyclin-dependent kinase | CDK10 | -0.131447202 | 0.912915224 | 1.167205486 | 2.24576269 | 0.000309653 | 2.459990403 |
| NM_001798 | cyclin-dependent kinase 2 | CDK2 | 0.030631577 | 1.021459198 | 1.150114297 | 2.219314761 | 0.022425322 | 2.172690564 |
| NM_001258 | cyclin-dependent kinase 3 | CDK3 | -0.534379034 | 0.690455799 | 2.658152283 | 6.312240977 | 0.038051322 | 9.142136233 |
| NM_001261 | cyclin-dependent kinase 9 | CDK9 | 0.372594353 | 1.294678919 | 1.740351983 | 3.341166743 | 0.00874806 | 2.580691393 |
| NM_001323 | cystatin E/M | CST6 | 0.108381607 | 1.078018253 | 2.799862916 | 6.963742784 | 9.24E-05 | 6.459763334 |
| NM_032687 | cysteine and histidine rich 1 | CYHR1 | -0.248002295 | 0.842061614 | 1.474477557 | 2.77882996 | 0.003478612 | 3.300031629 |
| NM_001554 | cysteine-rich, angiogenic inducer, 61 | CYR61 | -0.464125111 | 0.724910549 | 2.820629854 | 7.064707618 | 0.002996238 | 9.745626723 |
| NM_000492 | cystic fibrosis transmembrane conductance regulator, ATP-binding cassette | CFTR | 0.185716109 | 1.137381394 | 1.751678176 | 3.367500537 | 0.042589659 | 2.960748747 |
| NM_016593 | cytochrome P450, family 39, subfamily A, polypeptide 1 | CYP39A1 | -0.389333254 | 0.763482369 | 1.43586446 | 2.705442277 | 0.010615734 | 3.543555669 |
| NM_000778 | cytochrome P450, family 4, subfamily A, polypeptide 11 | CYP4A11 | 4.723556116 | 26.41995522 | 7.001190378 | 128.1056573 | 0.00191177 | 4.848821894 |
| NM_000779 | cytochrome P450, family 4, subfamily B, polypeptide 1 | CYP4B1 | -0.702764637 | 0.614393715 | 6.15304737 | 71.16260262 | 0.0007414 | 115.8257334 |
| NM_023944 | cytochrome P450, family 4, subfamily F, polypeptide 12 | CYP4F12 | -0.029091229 | 0.98003744 | 1.149226841 | 2.217949997 | 0.002508545 | 2.263127822 |
| NM_014608 | cytoplasmic FMR1 interacting protein 1 | CYFIP1 | 0.046918967 | 1.033056362 | 1.756888217 | 3.379683662 | 0.00979165 | 3.271538501 |
| NM_014376 | cytoplasmic FMR1 interacting protein 2 | CYFIP2 | 0.24284038 | 1.183320092 | 1.436732496 | 2.707070568 | 0.003375133 | 2.287690868 |
| NM_003388 | cytoplasmic linker 2 | CYLN2 | 0.32425399 | 1.25201686 | 2.917300824 | 7.554314377 | 6.53E-05 | 6.033716173 |
| NM_182485 | cytoplasmic polyadenylation element binding protein 2 | CPEB2 | -0.163634724 | 0.892772984 | 1.721569901 | 3.297950856 | 0.037269798 | 3.694053152 |
| NM_001928 | D component of complement | DF | 0.038584898 | 1.027105871 | 2.392547963 | 5.250839007 | 0.002061899 | 5.112266569 |
| NM_000107 | damage-specific DNA binding protein 2, 48kDa | DDB2 | 0.012941974 | 1.00901105 | 1.612260834 | 3.057305741 | 0.005034499 | 3.030002239 |
| NM_214462 | dapper, antagonist of beta-catenin, homolog 2 | DACT2 | 0.105729452 | 1.076038317 | 1.325499608 | 2.506196628 | 0.001066688 | 2.329096082 |
| NM_173543 | DAZ interacting protein 1-like | DZIP1L | -0.428522494 | 0.743022347 | 1.390286662 | 2.621307606 | 0.010953485 | 3.527898746 |
| CO581492 | DDT | DDT | -0.19217119 | 0.875287462 | 1.331500236 | 2.516642405 | 0.004686158 | 2.875218159 |
| NM_004396 | DEAD | DDX5 | -0.479783635 | 0.717085159 | 1.941562033 | 3.84121318 | 0.02357598 | 5.356704335 |
| NM_006386 | DEAD | DDX17 | -0.152717491 | 0.899554446 | 2.053514848 | 4.151160888 | 0.015227736 | 4.614685533 |
| NM_016355 | DEAD | DDX47 | 0.096553243 | 1.069215934 | 1.407755546 | 2.653240671 | 0.010913454 | 2.481482539 |
| NM_015404 | deafness, autosomal recessive 31 | DFNB31 | 0.152468479 | 1.111469592 | 1.301256376 | 2.464434054 | 0.000807922 | 2.217275283 |
| NM_022105 | death associated transcription factor 1 | DATF1 | 0.294786517 | 1.226703442 | 1.619666954 | 3.07304087 | 0.028143087 | 2.505121258 |
| NM_032998 | death effector domain containing | DEDD | -0.192785099 | 0.874915081 | 2.505382438 | 5.677998381 | 0.018304671 | 6.489770841 |
| NM_014326 | death-associated protein kinase 2 | DAPK2 | 0.299214331 | 1.230474134 | 3.659523349 | 12.63648534 | 0.013807714 | 10.26960664 |
| NM_152640 | decapping enzyme hDcp1b | DCP1B | -0.065715547 | 0.955471306 | 1.372816686 | 2.589756914 | 0.007248529 | 2.710449699 |
| NM_001920 | decorin | DCN | 0.244317619 | 1.184532366 | 2.045591003 | 4.128423588 | 0.002488013 | 3.485277149 |
| NM_020812 | dedicator of cytokinesis 6 | DOCK6 | -0.154935142 | 0.89817275 | 1.863806706 | 3.639667617 | 0.006371107 | 4.05230243 |
| NM_015296 | dedicator of cytokinesis 9 | DOCK9 | 0.592156647 | 1.507498584 | 2.479126155 | 5.575596487 | 0.029397099 | 3.698574941 |

| | | | | | | | | | |
|---|---|---|---|---|---|---|---|---|---|
| NM_005106 | deleted in lung and esophageal cancer 1 | DLEC1 | -0.329943854 | 0.795567445 | 1.749441305 | 3.362283338 | 0.010424323 | 4.226270644 |
| NM_178502 | deltex 3 homolog | DTX3 | -0.602941816 | 0.658410014 | 1.701090311 | 3.251465939 | 5.04E-05 | 4.938360398 |
| NM_022783 | DEP domain containing 6 | DEPDC6 | -0.298903581 | 0.812869926 | 1.404874381 | 2.647947247 | 0.024867863 | 3.257528864 |
| NM_001937 | dermatopontin | DPT | 0.131938611 | 1.095765139 | 3.595756256 | 12.09011663 | 0.00071767 | 11.03349267 |
| NM_001943 | desmoglein 2 | DSG2 | 0.598420413 | 1.514057937 | 3.745591542 | 13.41329282 | 0.00172654 | 8.859167468 |
| NM_152879 | diacylglycerol kinase, delta 130kDa | DGKD | -0.175027972 | 0.885750345 | 1.359670952 | 2.566266417 | 0.004695109 | 2.897279613 |
| NM_015881 | dickkopf homolog 3 | DKK3 | 0.541113603 | 1.455095259 | 3.578024493 | 11.94242982 | 0.002254763 | 8.207318212 |
| XR_014633 | Dipeptidyl-peptidase 2 precursor | DPP II | -0.221415024 | 0.857723751 | 1.200260058 | 2.297810873 | 0.0004948 | 2.678963793 |
| NM_004747 | discs, large homolog 5 | DLG5 | 0.226558089 | 1.170040192 | 1.993787283 | 3.982811726 | 0.004386886 | 3.403995652 |
| NM_181870 | dishevelled, dsh homolog 1 | DVL1 | 0.236205065 | 1.177890203 | 1.983374952 | 3.954170149 | 0.003584117 | 3.356993834 |
| NM_032890 | dispatched homolog 1 | DISP1 | -0.213192366 | 0.862626316 | 2.404486341 | 5.294470255 | 0.002391969 | 6.137617363 |
| NM_015497 | DKFZP564G2022 protein | DKFZP564G20 22 | -0.005165498 | 0.996425952 | 1.462032053 | 2.7549613 | 0.015227746 | 2.764842982 |
| NM_015462 | DKFZP586L0724 protein | DKFZP586L072 4 | 0.517875032 | 1.431844708 | 1.377922418 | 2.598938362 | 0.021884772 | 1.815097928 |
| NM_198489 | DLNB14 | DLNB14 | 0.073876772 | 1.052541248 | 1.962832427 | 3.898265702 | 0.007733756 | 3.703670245 |
| NM_022160 | DMRT-like family A1 | DMRTA1 | 0.679834002 | 1.601955422 | 5.021880925 | 32.48903359 | 0.008416682 | 20.28085997 |
| XR_009781 | DNA polymerase iota | LOC693999 | -0.806619939 | 0.571719761 | 3.083696077 | 8.47783611 | 0.017818243 | 14.82865678 |
| NM_007034 | DnaJ | DNAJB4 | -1.001127519 | 0.499609384 | 2.381355519 | 5.210260549 | 0.009208666 | 10.42866831 |
| NM_015190 | DnaJ | DNAJC9 | 0.403357153 | 1.322581982 | 1.412222498 | 2.661468512 | 0.02086505 | 2.012327817 |
| NM_033105 | DnaJ | DNAJC5B | 0.671160895 | 1.592353773 | 2.131850879 | 4.382794023 | 0.032006011 | 2.752399685 |
| NM_018110 | docking protein 4 | DOK4 | 0.187458019 | 1.138755498 | 1.849013517 | 3.602537672 | 0.002749802 | 3.163574339 |
| NM_017613 | downstream neighbor of SON | DONSON | 0.090184619 | 1.064506396 | 2.367369356 | 5.159993886 | 0.037937762 | 4.847311304 |
| CN646916 | DSCR6 | DSCR6 | 0.383081328 | 1.304124249 | 3.005019189 | 8.027880763 | 0.017099895 | 6.155763739 |
| NM_017434 | dual oxidase 1 | DUOX1 | 0.30566215 | 1.235985784 | 1.518210225 | 2.864354836 | 0.030329584 | 2.317465842 |
| NM_004417 | dual specificity phosphatase 1 | DUSP1 | -0.687064983 | 0.621116163 | 3.209228662 | 9.2485594 | 0.000215551 | 14.89022497 |
| NM_024025 | dual specificity phosphatase 26 | DUSP26 | -0.253786068 | 0.838692542 | 2.868495699 | 7.303032745 | 0.003529111 | 8.707640022 |
| NM_004420 | dual specificity phosphatase 8 | DUSP8 | -0.020333824 | 0.986004527 | 2.048625918 | 4.137117456 | 0.030233964 | 4.195840225 |
| NM_015548 | dystonin | DST | -0.212139677 | 0.863255977 | 2.965877397 | 7.813004241 | 0.008886267 | 9.050622815 |
| NM_183380 | dystonin | DST | -0.346395605 | 0.786546735 | 2.672789948 | 6.37661133 | 0.002861223 | 8.107097832 |
| NM_001390 | dystrobrevin, alpha | DTNA | 0.189203415 | 1.140134017 | 1.448542727 | 2.729322219 | 0.005241544 | 2.393860878 |
| NM_004424 | E4F transcription factor 1 | E4F1 | -0.273043088 | 0.827572099 | 1.930187406 | 3.811047016 | 0.002857481 | 4.605093648 |
| NM_005241 | ecotropic viral integration site 1 | EVI1 | 0.358314087 | 1.281926981 | 2.740190683 | 6.681586413 | 0.001082163 | 5.212142745 |
| NM_006209 | ectonucleotide pyrophosphatase/phosphodiesterase 2 | ENPP2 | -0.020243098 | 0.986066536 | 1.549049829 | 2.926243509 | 0.044052821 | 2.96759235 |
| NM_021572 | ectonucleotide pyrophosphatase/phosphodiesterase 5 | ENPP5 | -0.193894207 | 0.874242726 | 2.963471939 | 7.799988185 | 0.045860241 | 8.921993804 |
| NM_004105 | EGF-containing fibulin-like extracellular matrix protein 1 | EFEMP1 | -0.023997082 | 0.983504063 | 1.650613123 | 3.13967042 | 0.003515158 | 3.19233091 |
| NM_201446 | EGF-like-domain, multiple 7 | EGFL7 | -0.023288568 | 0.983987185 | 2.883963855 | 7.381754996 | 0.000142906 | 7.501881232 |
| NM_022821 | elongation of very long chain fatty acids | ELOVL1 | 0.347891937 | 1.272699602 | 1.964529557 | 3.902854168 | 0.008956454 | 3.066594947 |
| NM_016337 | Enah/Vasp-like | EVL | 0.046463497 | 1.032730269 | 1.868659044 | 3.651929828 | 0.000456681 | 3.536189398 |
| XM_370946 | endoplasmic reticulum | LOC388226 | -0.155543901 | 0.897793837 | 1.50120697 | 2.830794399 | 0.029036471 | 3.153056172 |
| NM_022464 | endoplasmic reticulum chaperone SIL1, homolog of yeast | SIL1 | 0.639924493 | 1.558247603 | 1.783518986 | 3.442648737 | 0.011542463 | 2.209307898 |
| NM_004826 | endothelin converting enzyme-like 1 | ECEL1 | -0.297201822 | 0.813829329 | 2.712231966 | 6.55334719 | 9.18E-05 | 8.052483434 |

| | | | | | | | | |
|---|---|---|---|---|---|---|---|---|
| NM_001991 | enhancer of zeste homolog 1 | EZH1 | 0.320177821 | 1.248484423 | 2.394010806 | 5.256165878 | 0.003133728 | 4.210037211 |
| NM_001976 | enolase 3, | ENO3 | 0.129652623 | 1.094030245 | 1.33582898 | 2.524204823 | 0.009362255 | 2.307253236 |
| NM_017512 | enolase superfamily member 1 | ENOSF1 | 0.275559174 | 1.210463162 | 1.839124451 | 3.577928236 | 0.009056684 | 2.955834055 |
| CO582652 | ENPEP | ENPEP | 0.468903161 | 1.38405681 | 4.850152103 | 28.84305557 | 0.017831574 | 20.83950266 |
| NM_005232 | EphA1 | EPHA1 | 0.708368265 | 1.633955015 | 1.946759246 | 3.855075851 | 0.032234832 | 2.359352501 |
| NM_004445 | EphB6 | EPHB6 | 2.688860179 | 6.448037699 | 3.619890292 | 12.29406652 | 0.00646745 | 1.906636886 |
| NM_004428 | ephrin-A1 | EFNA1 | 0.165803546 | 1.121790714 | 2.758716524 | 6.767938806 | 0.000155757 | 6.033156383 |
| NM_001424 | epithelial membrane protein 2 | EMP2 | -0.180476764 | 0.88241134 | 2.654353532 | 6.295642121 | 0.001488589 | 7.134588865 |
| NM_014805 | EPM2A | EPM2AIP1 | 0.074562012 | 1.053041295 | 1.446337094 | 2.725152742 | 0.026593952 | 2.587887821 |
| NM_000120 | epoxide hydrolase 1, microsomal | EPHX1 | -0.310586862 | 0.806313699 | 1.580487881 | 2.990709704 | 0.001591281 | 3.709114341 |
| NM_001979 | epoxide hydrolase 2, cytoplasmic | EPHX2 | -0.279830502 | 0.823687784 | 3.482780628 | 11.17947572 | 0.001262421 | 13.57246755 |
| NM_133180 | EPS8-like 1 | EPS8L1 | -0.04656161 | 0.9682412 | 2.161250328 | 4.473023469 | 0.000892671 | 4.619740896 |
| NM_014964 | epsin 2 | EPN2 | -0.041456013 | 0.971673809 | 1.760828598 | 3.388927087 | 0.003207135 | 3.487720936 |
| XR_010207 | erythroid differentiation-related factor 1 | EDRF | -0.348695354 | 0.785293927 | 2.181787132 | 4.537152439 | 0.005828604 | 5.777648703 |
| NM_019002 | ETAA16 protein | ETAA16 | -0.108717852 | 0.927411903 | 1.827208912 | 3.548499029 | 0.002751518 | 3.826238393 |
| NM_005240 | ets variant gene 3 | ETV3 | 0.079339594 | 1.056534292 | 3.259973173 | 9.579651499 | 0.007002605 | 9.067052127 |
| NM_004454 | ets variant gene 5 | ETV5 | 0.35662288 | 1.280425115 | 1.429372999 | 2.693296381 | 0.003309153 | 2.103439201 |
| NM_024757 | euchromatic histone methyltransferase 1 | EHMT1 | 0.33120992 | 1.258068014 | 1.733267332 | 3.324799476 | 0.015095051 | 2.642781979 |
| NM_001967 | eukaryotic translation initiation factor 4A, isoform 2 | EIF4A2 | 0.501291488 | 1.415480121 | 2.239986246 | 4.723925609 | 0.006404076 | 3.337330944 |
| NM_005243 | Ewing sarcoma breakpoint region 1 | EWSR1 | 0.102115713 | 1.073346372 | 1.339910569 | 2.531356267 | 0.011977517 | 2.358377811 |
| NM_000122 | excision repair cross-complementing rodent repair deficiency, complementation group 3 | ERCC3 | -0.225056153 | 0.855561725 | 1.46572363 | 2.762019738 | 0.015210429 | 3.228311479 |
| XR_013017 | exosome component 7 | EXOSC7 | 0.227132455 | 1.170506101 | 1.958218078 | 3.885817319 | 0.004566863 | 3.319775365 |
| NM_005244 | eyes absent homolog 2 | EYA2 | 0.402824781 | 1.322094024 | 1.838524029 | 3.576439482 | 0.008394496 | 2.705132477 |
| NM_145249 | family with sequence similarity 14, member B | FAM14B | -0.157265907 | 0.896722867 | 1.454213696 | 2.740071798 | 0.000403411 | 3.055650635 |
| NM_024898 | family with sequence similarity 31, member C | FAM31C | 2.080023894 | 4.228142188 | 3.499101224 | 11.30666243 | 0.032848977 | 2.674144323 |
| NM_004629 | Fanconi anemia, complementation group G | FANCG | 0.440662867 | 1.357227782 | 1.431383506 | 2.697052316 | 0.027031684 | 1.987177356 |
| NM_002004 | farnesyl diphosphate synthase | FDPS | -0.244758052 | 0.843957321 | 1.625993901 | 3.086547302 | 0.005322525 | 3.657231504 |
| NM_005103 | fasciculation and elongation protein zeta 1 | FEZ1 | 0.343870973 | 1.269157374 | 1.428973546 | 2.692550765 | 0.007610075 | 2.121526314 |
| NM_153342 | fasting-inducible integral membrane protein TM6P1 | FLJ90024 | 0.142534654 | 1.103842742 | 1.206003703 | 2.306977131 | 0.002449369 | 2.089950899 |
| NM_001442 | fatty acid binding protein 4, adipocyte | FABP4 | -0.107749678 | 0.928034486 | 3.185118433 | 9.095282399 | 0.000272735 | 9.800586655 |
| NM_001445 | fatty acid binding protein 6, ileal | FABP6 | 0.252553 | 1.191313405 | 2.025133934 | 4.070296621 | 0.042147249 | 3.41664637 |
| NM_021727 | fatty acid desaturase 3 | FADS3 | -0.189284298 | 0.877040702 | 2.241951457 | 4.730364834 | 0.001995299 | 5.39355223 |
| NM_031456 | F-box and WD-40 domain protein 10 | FBXW10 | -0.143293754 | 0.905449601 | 3.55193998 | 11.72844611 | 0.014810363 | 12.95317387 |
| XR_013084 | F-box and WD-40 domain protein 7, archipelago homolog | FBXW7 | -0.670024776 | 0.628495894 | 2.467668877 | 5.531492799 | 0.000661544 | 8.801159804 |
| XR_014794 | F-box only protein 2 | FBXO2 | -0.214025044 | 0.862128579 | 1.904303224 | 3.743280666 | 0.00235101 | 4.341905323 |
| CN802687 | FBXW7 | FBXW7 | 0.0200887 | 1.014021822 | 1.587929363 | 3.006175764 | 0.01721756 | 2.964606578 |
| NM_005766 | FERM, RhoGEF | FARP1 | -0.101777055 | 0.931884426 | 1.877977221 | 3.6755935 | 0.016021401 | 3.944258963 |
| NM_032843 | fibrinogen C domain containing 1 | FIBCD1 | 0.644236005 | 1.56291141 | 3.492135495 | 11.25220233 | 0.00322622 | 7.199513842 |
| NM_023028 | fibroblast growth factor receptor 2 | FGFR2 | -0.179160274 | 0.883216926 | 3.293161632 | 9.802580821 | 0.004593282 | 11.09872392 |
| NM_000142 | fibroblast growth factor receptor 3 | FGFR3 | -0.003643262 | 0.997477869 | 1.841475076 | 3.58376261 | 0.003451933 | 3.592824183 |
| NM_213647 | fibroblast growth factor receptor 4 | FGFR4 | -0.130868904 | 0.913281235 | 4.280403997 | 19.43255908 | 0.000121748 | 21.27773826 |
| NM_002023 | fibromodulin | FMOD | 0.49261438 | 1.406992248 | 1.472777371 | 2.775557095 | 0.007859213 | 1.972688264 |

| | | | | | | | | |
|---|---|---|---|---|---|---|---|---|
| NM_173651 | fibrous sheath interacting protein 2 | FSIP2 | 0.412952225 | 1.331407523 | 2.216877722 | 4.648862391 | 0.004010438 | 3.491690043 |
| NM_001996 | fibulin 1 | FBLN1 | 2.806122606 | 6.994023318 | 2.075737714 | 4.215599216 | 0.000357126 | 0.602743089 |
| NM_006329 | fibulin 5 | FBLN5 | -0.368363131 | 0.774660922 | 2.509388982 | 5.693788806 | 0.003145729 | 7.350040055 |
| NM_003665 | ficolin | FCN3 | -0.128745465 | 0.914626443 | 1.579131262 | 2.987898752 | 0.002204462 | 3.266796816 |
| CN646048 | FLJ10097 | FLJ10097 | -0.070958614 | 0.952005218 | 3.225944608 | 9.356342068 | 0.044445169 | 9.828036539 |
| CN645773 | FLJ10970 | FLJ10970 | -0.005058916 | 0.996499567 | 4.476365937 | 22.25975698 | 0.012885683 | 22.33794948 |
| DR771278 | FLJ14525 | FLJ14525 | 0.264352904 | 1.201097197 | 1.362679575 | 2.571623742 | 0.013327911 | 2.141062146 |
| CN644644 | FLJ20618 | FLJ20618 | -0.475141135 | 0.71939641 | 1.387776527 | 2.616750778 | 5.73E-05 | 3.637425405 |
| NM_022734 | FLJ20859 gene | FLJ20859 | -0.122636797 | 0.91850737 | 1.222419904 | 2.333377783 | 0.003329007 | 2.540401808 |
| NM_147195 | FLJ35740 protein | FLJ35740 | 0.475269652 | 1.390178032 | 3.24587351 | 9.48648422 | 0.001841761 | 6.823934778 |
| NM_207442 | FLJ39779 protein | FLJ39779 | 0.129786598 | 1.094131847 | 1.467253479 | 2.764950167 | 0.023524278 | 2.527072194 |
| NM_207414 | FLJ43860 protein | FLJ43860 | 0.445820459 | 1.362088515 | 1.810415316 | 3.507432443 | 0.041133189 | 2.575040024 |
| NM_198567 | FLJ44216 protein | FLJ44216 | -0.096958005 | 0.935002414 | 1.649516527 | 3.137284855 | 0.007496844 | 3.355376209 |
| NM_207395 | FLJ45850 protein | FLJ45850 | 0.2080864 | 1.155154963 | 1.205725774 | 2.306532745 | 0.028190934 | 1.996730152 |
| NM_207458 | FLJ46026 protein | FLJ46026 | 0.873895506 | 1.832604549 | 2.034652852 | 4.097241277 | 0.009609471 | 2.23574763 |
| NM_198471 | FLJ46061 protein | FLJ46061 | 0.020125986 | 1.01404803 | 1.717563882 | 3.288805938 | 0.000411802 | 3.243244739 |
| NM_144997 | folliculin | BHD | -0.098496628 | 0.934005773 | 1.193920093 | 2.287735232 | 0.030345103 | 2.449380184 |
| NM_006350 | follistatin | FST | 0.453322751 | 1.369190085 | 2.483753896 | 5.593510075 | 0.025020788 | 4.085269194 |
| NM_004496 | forkhead box A1 | FOXA1 | -0.636429606 | 0.643303032 | 2.322479965 | 5.001913003 | 0.018310712 | 7.775360529 |
| NM_001454 | forkhead box J1 | FOXJ1 | 0.928189306 | 1.902886227 | 2.833436679 | 7.127700309 | 0.007642144 | 3.745731199 |
| NM_015308 | formin binding protein 4 | FNBP4 | -0.296495398 | 0.814227922 | 1.666698756 | 3.174872721 | 0.003807364 | 3.89924324 |
| NM_013241 | formin homology 2 domain containing 1 | FHOD1 | -0.246591894 | 0.842885229 | 3.070811984 | 8.402461249 | 8.88E-05 | 9.968689639 |
| NM_001449 | four and a half LIM domains 1 | FHL1 | -0.916485129 | 0.529798209 | 1.92632428 | 3.800855768 | 0.01574948 | 7.17415745 |
| NM_003506 | frizzled homolog 6 | FZD6 | -0.367855308 | 0.774933647 | 2.117479742 | 4.339352369 | 0.020909119 | 5.599643771 |
| NM_000507 | fructose-1,6-bisphosphatase 1 | FBP1 | -0.585364011 | 0.666481155 | 2.14062763 | 4.409538372 | 0.001459285 | 6.616148617 |
| NM_145059 | fucokinase | FUK | -0.422531477 | 0.746114278 | 1.564693132 | 2.95814574 | 0.000647457 | 3.964735466 |
| CR604926 | full-length cDNA clone CS0DF038YH05 of Fetal brain of | full-length cDNA clone CS0DF038YH0 5 of Fetal brain of | -0.234583908 | 0.849930094 | 2.450838946 | 5.467339432 | 0.000484933 | 6.432693077 |
| NM_006625 | FUS interacting protein | FUSIP1 | -0.307121 | 0.808253077 | 1.222610241 | 2.333685649 | 2.17E-05 | 2.887320464 |
| NM_005971 | FXYD domain containing ion transport regulator 3 | FXYD3 | -0.553430453 | 0.681397966 | 2.029682237 | 4.083149063 | 0.012901386 | 5.992311787 |
| CN643246 | FXYD1 | FXYD1 | -1.218351757 | 0.429773443 | 2.332371504 | 5.036325416 | 0.000426923 | 11.71855892 |
| XM_371619 | FYVE, RhoGEF and PH domain containing 5 | FGD5 | 0.032285282 | 1.022630728 | 1.207494856 | 2.309362829 | 0.009915206 | 2.258256833 |
| NM_025211 | G kinase anchoring protein 1 | GKAP1 | 0.599264714 | 1.514944261 | 2.922865082 | 7.583506493 | 0.028162097 | 5.005799017 |
| NM_018025 | G patch domain containing 1 | GPATC1 | 0.154584723 | 1.113101168 | 1.367702048 | 2.580591971 | 0.00138479 | 2.318380436 |
| NM_032442 | G protein pathway suppressor 2 | GPS2 | 0.166820001 | 1.122581353 | 1.073870986 | 2.105074054 | 0.001673521 | 1.875208463 |
| NM_015234 | G protein-coupled receptor 116 | GPR116 | 0.142314982 | 1.103674678 | 2.240943164 | 4.727059957 | 0.000692592 | 4.283019309 |
| NM_020455 | G protein-coupled receptor 126 | GPR126 | -0.547805896 | 0.684059681 | 1.869118661 | 3.653093454 | 0.021244301 | 5.340313946 |
| NM_024980 | G protein-coupled receptor 157 | GPR157 | 0.419543431 | 1.337504208 | 1.356210003 | 2.560117458 | 0.006604704 | 1.914100489 |
| NM_182982 | G protein-coupled receptor kinase 4 | GRK4 | 0.061232968 | 1.043357062 | 1.198436265 | 2.294907914 | 0.008368864 | 2.199542225 |
| NM_005308 | G protein-coupled receptor kinase 5 | GRK5 | -0.474996053 | 0.719468758 | 1.437893849 | 2.709250605 | 0.004514905 | 3.765626478 |
| NM_022036 | G protein-coupled receptor, family C, group 5, member C | GPRC5C | 0.324188363 | 1.251959908 | 2.503038884 | 5.668782372 | 0.0026009 | 4.527926442 |
| NM_001470 | gamma-aminobutyric acid | GABBR1 | 0.121133374 | 1.087588931 | 2.56897932 | 5.933894677 | 0.00207571 | 5.456008707 |

| | | | | | | | | |
|---|---|---|---|---|---|---|---|---|
| NM_002043 | gamma-aminobutyric acid | GABRR2 | 0.419272154 | 1.337252734 | 1.648019638 | 3.134031408 | 0.017571204 | 2.343634325 |
| NM_021990 | gamma-aminobutyric acid | GABRE | 0.165051697 | 1.121206254 | 5.16922024 | 35.98241818 | 0.001468885 | 32.09259496 |
| NM_006783 | gap junction protein, beta 6 | GJB6 | 0.700865223 | 1.625479344 | 2.11784312 | 4.340445477 | 0.034684783 | 2.670255696 |
| NM_000805 | gastrin | GAST | 1.266761075 | 2.406207526 | 2.85795504 | 7.24986957 | 0.006999765 | 3.012985992 |
| NM_032638 | GATA binding protein 2 | GATA2 | 0.499464441 | 1.413688674 | 2.029298253 | 4.082062449 | 0.004863911 | 2.88752575 |
| NM_021167 | GATA zinc finger domain containing 1 | GATAD1 | -0.284801879 | 0.820854326 | 1.584833453 | 2.999731665 | 0.004968043 | 3.654401969 |
| AB038463 | GC36 mRNA, complete cds | GC36 | -0.068032539 | 0.953938035 | 2.756131813 | 6.755824321 | 0.00948847 | 7.082036855 |
| NM_021078 | GCN5 general control of amino-acid synthesis 5-like 2 | GCN5L2 | -0.181592469 | 0.881729193 | 2.973927599 | 7.856722466 | 0.000261701 | 8.910584484 |
| NM_019858 | gene rich cluster, A gene | GRCA | -0.034895449 | 0.976102495 | 1.568721625 | 2.966417428 | 0.011684769 | 3.039042973 |
| XR_000285 | general transcription factor II, i, pseudogene 1 | GTF2IP1 | -0.117578519 | 0.92173343 | 1.403855487 | 2.646077812 | 0.007298194 | 2.87076255 |
| CN805323 | GK001 | GK001 | 0.104072721 | 1.074803348 | 1.290023702 | 2.445320729 | 0.027186463 | 2.275133152 |
| XM_166529 | glucocorticoid induced transcript 1 | GLCCI1 | -0.48541502 | 0.71429156 | 1.76351815 | 3.395250806 | 0.028736971 | 4.753312227 |
| XR_010365 | glucocorticoid induced transcript 1 | GLCCI1 | -0.180235813 | 0.882558728 | 1.262126322 | 2.39848983 | 0.004701612 | 2.717654649 |
| NM_014905 | glutaminase | GLS | -0.387445543 | 0.76448201 | 1.889362003 | 3.704713564 | 0.004528834 | 4.846044139 |
| XR_013675 | Glutathione peroxidase 3 precursor | GPx-3 | -0.193168046 | 0.874682876 | 1.578316691 | 2.986212208 | 0.005371707 | 3.414051298 |
| NM_000846 | glutathione S-transferase A2 | GSTA2 | -0.037228351 | 0.97452537 | 1.3040153 | 2.469151399 | 0.026836372 | 2.533696377 |
| NM_000847 | glutathione S-transferase A3 | GSTA3 | 0.172100673 | 1.126697848 | 3.681418423 | 12.82972572 | 0.000191728 | 11.38701538 |
| NM_001512 | glutathione S-transferase A4 | GSTA4 | -0.073275671 | 0.950477466 | 1.939837855 | 3.836625253 | 0.006458404 | 4.036524158 |
| NM_000853 | glutathione S-transferase theta 1 | GSTT1 | -0.282859863 | 0.821960025 | 3.059060286 | 8.334295691 | 0.000184421 | 10.13953895 |
| NM_005276 | glycerol-3-phosphate dehydrogenase 1 | GPD1 | -0.564105558 | 0.676374625 | 2.618545494 | 6.141306018 | 0.040303861 | 9.079740415 |
| NM_024307 | glycerophosphodiester phosphodiesterase domain containing 3 | GDPD3 | 0.140583536 | 1.102350901 | 2.113911476 | 4.328632963 | 0.007097745 | 3.926728739 |
| NM_001001995 | glycoprotein M6B | GPM6B | 0.076811663 | 1.054684624 | 4.342966984 | 20.29379797 | 0.028795851 | 19.24157943 |
| NM_001010983 | glycosyltransferase 8 domain containing 1 | GLT8D1 | -0.016697418 | 0.98849295 | 1.325815713 | 2.506745814 | 0.001679389 | 2.535926851 |
| NM_152312 | glycosyltransferase-like 1B | GYLTL1B | 0.254897021 | 1.193250566 | 2.360559499 | 5.135694906 | 0.037294858 | 4.303953463 |
| NM_004484 | glypican 3 | GPC3 | -0.26073469 | 0.834662761 | 1.693436067 | 3.234260911 | 0.001855117 | 3.874931364 |
| NM_138619 | golgi associated, gamma adaptin ear containing, ARF binding protein 3 | GGA3 | 0.257885926 | 1.195725247 | 1.432477434 | 2.69909814 | 0.035029346 | 2.25728958 |
| NM_002077 | golgi autoantigen, golgin subfamily a, 1 | GOLGA1 | -0.043915112 | 0.970018985 | 1.835009943 | 3.567738665 | 0.011844121 | 3.678009111 |
| NM_018652 | golgi autoantigen, golgin subfamily a, member 6 | GOLGA6 | -0.635266544 | 0.643821855 | 1.386568577 | 2.614560723 | 0.009779486 | 4.061000264 |
| NM_018178 | golgi phosphoprotein 3-like | GOLPH3L | 0.311718499 | 1.241185286 | 1.495450997 | 2.819522777 | 0.023844504 | 2.271637288 |
| NM_001501 | gonadotropin-releasing hormone 2 | GNRH2 | 0.333003308 | 1.259632868 | 2.167329275 | 4.491910787 | 0.001055576 | 3.566047617 |
| NM_205854 | GSGL541 | UNQ541 | -0.108145717 | 0.927779763 | 1.794475508 | 3.468893403 | 0.001044628 | 3.738919021 |
| NM_019096 | GTP binding protein 2 | GTPBP2 | -0.417696836 | 0.74861879 | 1.28381452 | 2.434818985 | 0.002621195 | 3.252415004 |
| NM_032293 | GTPase activating Rap/RanGAP domain-like 3 | GARNL3 | 0.307755078 | 1.237780139 | 3.073161756 | 8.416157804 | 0.006149855 | 6.799396386 |
| NM_033258 | guanine nucleotide binding protein | GNG8 | -0.332610823 | 0.794098115 | 1.82590027 | 3.545281712 | 0.023761273 | 4.464538632 |
| NM_007102 | guanylate cyclase activator 2B | GUCA2B | 0.616451755 | 1.533099949 | 3.153177279 | 8.896126355 | 0.000954429 | 5.802704749 |
| NM_015726 | H326 | H326 | -0.045133886 | 0.969199868 | 1.363136931 | 2.572439114 | 0.001059223 | 2.654188469 |
| NM_005144 | hairless homolog | HR | 0.524082585 | 1.438018847 | 1.943484529 | 3.846335288 | 0.014158032 | 2.67474609 |
| NM_012258 | hairy/enhancer-of-split related with YRPW motif 1 | HEY1 | 0.928905106 | 1.903830587 | 2.362478453 | 5.142530529 | 0.015085726 | 2.701149233 |
| CN801728 | HBA2 | HBA2 | -0.446695339 | 0.733721599 | 2.297035623 | 4.914469268 | 2.00E-05 | 6.698002723 |
| NM_052970 | heat shock 70kD protein 12B | HSPA12B | 0.003500428 | 1.002429258 | 1.89942624 | 3.730647993 | 0.003823882 | 3.721607249 |

| | | | | | | | | |
|---|---|---|---|---|---|---|---|---|
| NM_016323 | hect domain and RLD 5 | HERC5 | 0.08549266 | 1.061050013 | 1.782122327 | 3.439317554 | 0.017732704 | 3.241428313 |
| NM_016173 | HemK methyltransferase family member 1 | HEMK1 | 0.348146875 | 1.27292452 | 3.034950064 | 8.196170887 | 0.008193797 | 6.438850661 |
| NM_000518 | hemoglobin, beta | HBB | 0.317571073 | 1.246230623 | 5.364313107 | 41.19259494 | 0.000713814 | 33.05374959 |
| NM_001945 | heparin-binding EGF-like growth factor | HBEGF | -0.190586976 | 0.876249137 | 1.480387945 | 2.790237536 | 0.001429913 | 3.184297043 |
| NM_014799 | hephaestin | HEPH | 0.625423746 | 1.542663868 | 1.800495845 | 3.483399269 | 0.040642855 | 2.258041652 |
| NM_182983 | hepsin | HPN | -0.117894868 | 0.921531338 | 1.895323377 | 3.720053518 | 0.001325658 | 4.036817159 |
| NM_005520 | heterogeneous nuclear ribonucleoprotein H1 | HNRPH1 | -0.147491082 | 0.902819146 | 2.486140646 | 5.602771461 | 0.012714992 | 6.205862476 |
| XR_010398 | histidyl-tRNA synthetase-like | HARSL | -0.222789207 | 0.856907149 | 1.940860879 | 3.839346792 | 0.000694643 | 4.480470021 |
| NM_032019 | histone deacetylase 10 | HDAC10 | 0.898491417 | 1.864115714 | 1.719497618 | 3.293217091 | 0.022635455 | 1.766637697 |
| NM_015401 | histone deacetylase 7A | HDAC7A | 0.511331505 | 1.425365099 | 1.495486133 | 2.819591445 | 0.009337785 | 1.978153841 |
| XM_496408 | histone H3 | H3 | -0.210578755 | 0.864190481 | 1.497879301 | 2.824272513 | 0.011885689 | 3.268113425 |
| NM_002141 | homeo box A4 | HOXA4 | -0.258739763 | 0.835817712 | 1.644736758 | 3.126907969 | 0.019748255 | 3.741136284 |
| NM_002147 | homeo box B5 | HOXB5 | -0.238075204 | 0.847875766 | 1.786952817 | 3.450852516 | 0.014859717 | 4.069997816 |
| NM_014620 | homeo box C4 | HOXC4 | 0.254167832 | 1.192647608 | 2.580618726 | 5.981961919 | 0.049661556 | 5.015699423 |
| XR_011538 | Homeobox protein Hox-D10 | Hox-4D | 0.226315188 | 1.169843214 | 5.858643257 | 58.02663071 | 0.004755016 | 49.60205779 |
| NM_032495 | homeodomain-only protein | HOP | -0.556610464 | 0.679897672 | 2.000468805 | 4.001300014 | 0.010836461 | 5.885150338 |
| NM_144565 | homolog of Drosophila Numb-interacting protein | NIP | 0.960921177 | 1.946552394 | 1.933096463 | 3.818739381 | 0.043119344 | 1.96179635 |
| NM_080731 | HOM-TES-103 tumor antigen-like | HOM-TES-103 | 0.087937367 | 1.062849531 | 1.631047749 | 3.097378623 | 0.012974412 | 2.914221188 |
| NM_015888 | hook homolog 1 | HOOK1 | 0.57066648 | 1.485209532 | 1.746772153 | 3.356068478 | 0.004569399 | 2.259659937 |
| NM_022727 | HpaII tiny fragments locus 9C | HTF9C | -0.010073297 | 0.993042042 | 1.208393193 | 2.31080127 | 0.00141718 | 2.326992385 |
| CN646542 | Hs159853 | Hs159853 | 0.33461822 | 1.261043655 | 2.484527493 | 5.596510209 | 0.010965342 | 4.437998785 |
| CN648422 | Hs294035 | Hs294035 | 0.124713973 | 1.090291551 | 1.152143214 | 2.222438064 | 0.002648785 | 2.03838878 |
| CK231263 | Hs296141 | Hs296141 | 0.128439575 | 1.093110748 | 1.765935935 | 3.400945612 | 0.003027979 | 3.111254389 |
| CK231501 | Hs417764 | Hs417764 | 0.603770731 | 1.519683333 | 1.937637059 | 3.830777038 | 0.043185134 | 2.520773213 |
| CN647387 | Hs420303 | Hs420303 | -0.473836263 | 0.720047376 | 1.468422806 | 2.767192111 | 0.000291843 | 3.843069502 |
| CN643612 | Hs4241 | Hs4241 | 0.064472943 | 1.045702845 | 1.578012536 | 2.985582708 | 0.013735171 | 2.855096668 |
| CO582642 | Hs521442 | Hs521442 | 0.086964796 | 1.062133268 | 1.842687007 | 3.586774403 | 0.001629336 | 3.376953261 |
| CN643290 | Hs528712 | Hs528712 | 0.502900461 | 1.417059623 | 1.477703919 | 2.785051332 | 0.005420554 | 1.96537343 |
| NM_003949 | huntingtin-associated protein 1 | HAP1 | 0.41506925 | 1.333362678 | 1.306200521 | 2.472894207 | 0.026776415 | 1.854629838 |
| NM_138612 | hyaluronan synthase 3 | HAS3 | 0.559569642 | 1.473829506 | 1.243540361 | 2.367788732 | 0.021616865 | 1.606555387 |
| NM_000196 | hydroxysteroid | HSD11B2 | 0.453473264 | 1.369332937 | 2.925383374 | 7.596755422 | 0.000202077 | 5.547778204 |
| NM_014234 | hydroxysteroid | HSD17B8 | -0.111892781 | 0.925373198 | 1.311540152 | 2.482063718 | 0.000161956 | 2.682229962 |
| NM_052818 | hypothetical gene CG018 | CG018 | -0.476196883 | 0.718870156 | 2.499594101 | 5.65526293 | 0.001962717 | 7.866876769 |
| NM_139016 | hypothetical gene LOC128439 | LOC128439 | -0.398657826 | 0.758563665 | 1.834102848 | 3.565496155 | 0.000388872 | 4.700325522 |
| NM_207446 | hypothetical gene supported by AK075564; BC060873 | LOC400451 | 0.345129958 | 1.270265403 | 1.213395064 | 2.318826798 | 0.000420284 | 1.825466389 |
| XM_496500 | hypothetical gene supported by AK094309 | LOC440798 | -0.511639295 | 0.701424975 | 2.290484011 | 4.892202123 | 0.018496657 | 6.974662006 |
| XM_498519 | hypothetical gene supported by AK124252 | LOC440049 | 0.891927672 | 1.85565392 | 3.437674686 | 10.83535627 | 0.001033635 | 5.839104023 |
| XM_498468 | hypothetical gene supported by AK128460; NM_198530 | LOC439921 | 0.138224591 | 1.100549924 | 1.724651516 | 3.30500285 | 0.000117526 | 3.00304673 |
| NM_203393 | hypothetical gene supported by BC031661 | LOC389458 | 0.84446312 | 1.795596414 | 1.728337112 | 3.313456805 | 0.004040838 | 1.845323804 |
| NM_001012982 | hypothetical gene supported by BC043530 | LOC345222 | 0.106620496 | 1.07670311 | 1.24345871 | 2.367654728 | 0.020883984 | 2.198985687 |
| NM_138414 | hypothetical protein BC011981 | LOC112869 | -0.106110166 | 0.929089725 | 2.095233927 | 4.272954417 | 0.00116944 | 4.599076173 |
| NM_144697 | hypothetical protein BC017397 | LOC148523 | -0.091115877 | 0.93879634 | 1.902069027 | 3.737488207 | 0.009298784 | 3.981149103 |

| | | | | | | | | |
|---|---|---|---|---|---|---|---|---|
| NM_023037 | hypothetical protein CG003 | 13CDNA73 | -0.979773086 | 0.507059486 | 2.465568559 | 5.523445749 | 0.031481976 | 10.89309223 |
| NM_207310 | hypothetical protein DKFZp434E2321 | DKFZp434E232 1 | -0.140806183 | 0.907012172 | 1.836074093 | 3.570371246 | 0.003957284 | 3.936409407 |
| NM_017559 | hypothetical protein DKFZp434H2215 | DKFZp434H22 15 | 0.706316611 | 1.631633023 | 2.506785717 | 5.683523937 | 0.00108105 | 3.483334706 |
| NM_032269 | hypothetical protein DKFZp434I099 | DKFZp434I099 | 0.52976567 | 1.443694684 | 2.066158765 | 4.18770195 | 0.015144424 | 2.900683916 |
| NM_022778 | hypothetical protein DKFZp434L0117 | DKFZP434L011 7 | -0.009282474 | 0.993586534 | 1.507396969 | 2.842966243 | 0.001837225 | 2.861317204 |
| NM_152793 | hypothetical protein Ells1 | Ells1 | -0.277620044 | 0.824950784 | 3.924672568 | 15.18602695 | 0.036299353 | 18.40840356 |
| NM_017991 | hypothetical protein FLJ10081 | FLJ10081 | 0.150699671 | 1.110107716 | 1.463803018 | 2.7583452 | 0.005947957 | 2.484754552 |
| NM_018011 | hypothetical protein FLJ10154 | FLJ10154 | 0.453500021 | 1.369358334 | 4.570842544 | 23.76625274 | 0.001331431 | 17.35575864 |
| NM_019057 | hypothetical protein FLJ10404 | FLJ10404 | -0.160374968 | 0.894792477 | 2.005202005 | 4.014449055 | 0.016848821 | 4.48645821 |
| NM_018089 | hypothetical protein FLJ10415 | FLJ10415 | 0.176438184 | 1.130090401 | 3.238741357 | 9.439702269 | 8.47E-05 | 8.353050571 |
| NM_018142 | hypothetical protein FLJ10569 | FLJ10569 | -0.215157598 | 0.861452051 | 1.288208077 | 2.44224524 | 0.01631417 | 2.835033287 |
| NM_018281 | hypothetical protein FLJ10948 | FLJ10948 | 0.107941461 | 1.077689416 | 2.213439251 | 4.637795643 | 0.002530334 | 4.303462179 |
| XM_035527 | hypothetical protein FLJ10980 | FLJ10980 | 0.191041178 | 1.141587291 | 2.130566181 | 4.378892951 | 0.002177044 | 3.83579336 |
| NM_024669 | hypothetical protein FLJ11795 | FLJ11795 | -0.358912793 | 0.779751975 | 1.313916415 | 2.486155293 | 0.006843091 | 3.188392428 |
| NM_024830 | hypothetical protein FLJ12443 | FLJ12443 | -0.606467089 | 0.656803131 | 2.496975998 | 5.645009457 | 0.00820365 | 8.594675011 |
| NM_031206 | hypothetical protein FLJ12525 | FLJ12525 | 0.399372172 | 1.318933816 | 1.762601966 | 3.393095336 | 0.005019381 | 2.572604701 |
| NM_022492 | hypothetical protein FLJ12788 | FLJ12788 | -0.880086683 | 0.543334785 | 1.500589835 | 2.829583743 | 0.004915323 | 5.207808926 |
| NM_022753 | hypothetical protein FLJ12903 | FLJ12903 | 0.341270098 | 1.266871412 | 1.475007885 | 2.779851633 | 0.018907026 | 2.194265027 |
| NM_032118 | hypothetical protein FLJ12953 Mus musculus D3Mm3e | FLJ12953 | -0.341529756 | 0.789204039 | 1.816269998 | 3.521695071 | 0.00049525 | 4.462337871 |
| NM_024841 | hypothetical protein FLJ14213 | FLJ14213 | 0.294540695 | 1.226494441 | 2.285325187 | 4.874739708 | 0.035094327 | 3.974530618 |
| NM_032793 | hypothetical protein FLJ14490 | FLJ14490 | -0.104585779 | 0.930071943 | 2.323315764 | 5.004811609 | 0.00359979 | 5.381101588 |
| NM_207514 | hypothetical protein FLJ20186 | FLJ20186 | -0.084698987 | 0.942981265 | 1.587195822 | 3.004647658 | 0.000548411 | 3.186328053 |
| NM_017786 | hypothetical protein FLJ20366 | FLJ20366 | -0.765154306 | 0.588390433 | 4.158547451 | 17.85860458 | 0.000537895 | 30.35162297 |
| NM_017820 | hypothetical protein FLJ20433 | FLJ20433 | 0.517872262 | 1.431841958 | 2.533434014 | 5.789480963 | 0.006132884 | 4.043379879 |
| NM_017822 | hypothetical protein FLJ20436 | FLJ20436 | 0.355932436 | 1.279812477 | 2.030303375 | 4.084907402 | 0.000347725 | 3.191801514 |
| NM_024927 | hypothetical protein FLJ21019 | FLJ21019 | 0.055825364 | 1.039453603 | 1.271369334 | 2.413905726 | 0.005749255 | 2.322283283 |
| NM_025137 | hypothetical protein FLJ21439 | FLJ21439 | -0.444832283 | 0.734669719 | 1.504301418 | 2.836872708 | 0.001120689 | 3.861425937 |
| NM_032207 | hypothetical protein FLJ21742 | FLJ21742 | -0.235827877 | 0.849197555 | 1.477148265 | 2.783978877 | 0.002996393 | 3.278364216 |
| NM_023015 | hypothetical protein FLJ21919 | FLJ21919 | -0.088845761 | 0.940274722 | 1.803050564 | 3.489573124 | 0.001857856 | 3.7112272 |
| NM_024790 | hypothetical protein FLJ22490 | FLJ22490 | -0.45661019 | 0.728696421 | 2.14846071 | 4.433544972 | 0.007239578 | 6.084214008 |
| NM_025092 | hypothetical protein FLJ22635 | FLJ22635 | -0.353509267 | 0.782677965 | 2.366637515 | 5.157377021 | 0.000527904 | 6.589398516 |
| NM_024730 | hypothetical protein FLJ22655 | FLJ22655 | 3.918155693 | 15.11758403 | 6.75424178 | 107.9516721 | 0.030310587 | 7.140801856 |
| NM_024574 | hypothetical protein FLJ23191 | FLJ23191 | 0.162910154 | 1.119543165 | 1.34204254 | 2.535099794 | 0.021056636 | 2.264405584 |
| NM_024631 | hypothetical protein FLJ23342 | FLJ23342 | -0.119029709 | 0.920806735 | 6.091033532 | 68.16850939 | 0.020017106 | 74.03128887 |
| NM_152343 | hypothetical protein FLJ25414 | FLJ25414 | 0.881435033 | 1.842206813 | 2.086382876 | 4.246819746 | 0.033236359 | 2.30528935 |
| NM_144595 | hypothetical protein FLJ30046 | FLJ30046 | 0.512877109 | 1.426892956 | 1.222325674 | 2.333225382 | 0.043686837 | 1.63517899 |
| NM_152378 | hypothetical protein FLJ31052 | FLJ31052 | 0.915333539 | 1.88600505 | 2.744025822 | 6.699371809 | 0.04140714 | 3.552149454 |
| NM_152748 | hypothetical protein FLJ31340 | FLJ31340 | -0.000647206 | 0.999551491 | 3.40861438 | 10.61928244 | 0.003717294 | 10.62404742 |
| NM_144679 | hypothetical protein FLJ31528 | FLJ31528 | -0.363539075 | 0.777255554 | 1.922694332 | 3.791304501 | 0.000204804 | 4.877809469 |
| NM_152509 | hypothetical protein FLJ31568 | FLJ31568 | 0.772642194 | 1.708395726 | 2.266674459 | 4.812126135 | 0.003173236 | 2.816751448 |
| NM_173795 | hypothetical protein FLJ32096 | FLJ32096 | -0.799983931 | 0.574355575 | 1.727878146 | 3.31240286 | 0.019541504 | 5.767164113 |

| | | | | | | | | | |
|---|---|---|---|---|---|---|---|---|---|
| NM_144674 | hypothetical protein FLJ32871 | FLJ32871 | 0.144899492 | 1.105653623 | 1.845868446 | 3.594692705 | 0.041406496 | 3.251192444 |
| NM_153041 | hypothetical protein FLJ32955 | FLJ32955 | 0.518548235 | 1.432513004 | 1.907809652 | 3.752389665 | 0.000655163 | 2.619445446 |
| NM_152449 | hypothetical protein FLJ33008 | FLJ33008 | 0.105641626 | 1.075972813 | 1.671508437 | 3.185474828 | 0.008631145 | 2.960553268 |
| NM_152683 | hypothetical protein FLJ33167 | FLJ33167 | 0.246318754 | 1.186176548 | 3.47305024 | 11.10432844 | 0.000747903 | 9.361446623 |
| NM_152544 | hypothetical protein FLJ35725 | FLJ35725 | -0.364054309 | 0.776978019 | 1.291217516 | 2.447345042 | 0.007242857 | 3.149825326 |
| NM_152598 | hypothetical protein FLJ35757 | FLJ35757 | 0.691682233 | 1.615165761 | 1.480227249 | 2.789926759 | 0.033298878 | 1.727331539 |
| NM_152408 | hypothetical protein FLJ35779 | FLJ35779 | 0.449479793 | 1.365547779 | 1.468056241 | 2.766489103 | 0.013459551 | 2.025918936 |
| NM_178314 | hypothetical protein FLJ39378 | FLJ39378 | 0.13194379 | 1.095769072 | 1.128307712 | 2.186021684 | 0.029343958 | 1.994965673 |
| NM_153365 | hypothetical protein FLJ90013 | FLJ90013 | 0.337855326 | 1.26387635 | 1.788406266 | 3.454330844 | 0.006447695 | 2.733124047 |
| NM_153345 | hypothetical protein FLJ90586 | FLJ90586 | -0.208756128 | 0.865282945 | 1.360836942 | 2.568341319 | 0.006897147 | 2.968209802 |
| NM_020467 | hypothetical protein from clone 643 | LOC57228 | -0.479798748 | 0.717077647 | 1.630663537 | 3.096553855 | 0.012580883 | 4.318296447 |
| NM_020214 | hypothetical protein from EUROIMAGE 1977056 | LOC56965 | 0.19287786 | 1.14304156 | 2.045684162 | 4.12869018 | 0.0003702 | 3.612021054 |
| NM_031207 | hypothetical protein HT036 | HT036 | -0.345101782 | 0.787252434 | 1.509593641 | 2.847298289 | 0.001380615 | 3.616753872 |
| NM_019092 | hypothetical protein KIAA1164 | KIAA1164 | 0.437034491 | 1.353818645 | 1.486495782 | 2.802075409 | 0.008123588 | 2.069756846 |
| NM_019593 | hypothetical protein KIAA1434 | KIAA1434 | -0.100182318 | 0.932915089 | 1.486735708 | 2.802541444 | 0.006036558 | 3.004069156 |
| NM_175873 | hypothetical protein LOC134548 | LOC134548 | 1.014756116 | 2.020561294 | 1.929886609 | 3.810252508 | 0.002243241 | 1.885739631 |
| NM_182507 | hypothetical protein LOC144501 | LOC144501 | 0.388735388 | 1.309245266 | 3.607145714 | 12.18594065 | 0.003597853 | 9.307607189 |
| NM_207324 | hypothetical protein LOC147650 | LOC147650 | 0.367957434 | 1.290524413 | 2.550716669 | 5.859252686 | 0.000812823 | 4.540210652 |
| NM_181712 | hypothetical protein LOC163782 | LOC163782 | 1.875881676 | 3.670258497 | 3.248484788 | 9.503670307 | 0.024486766 | 2.589373559 |
| XM_113796 | hypothetical protein LOC196996 | LOC196996 | -0.590331649 | 0.664190205 | 3.163161814 | 8.95790778 | 0.000752793 | 13.48696159 |
| NM_198278 | hypothetical protein LOC255743 | LOC255743 | -0.260463094 | 0.834819906 | 3.127790727 | 8.740953886 | 0.000314098 | 10.47046653 |
| NM_182774 | hypothetical protein LOC259173 | FLJ36525 | -0.681054018 | 0.623709432 | 2.371044761 | 5.173156226 | 0.003975127 | 8.29417668 |
| NM_174940 | hypothetical protein LOC283232 | LOC283232 | -0.343473325 | 0.788141555 | 2.280492696 | 4.858438463 | 0.001771072 | 6.164423678 |
| NM_207348 | hypothetical protein LOC284723 | LOC284723 | 0.068187116 | 1.048398443 | 3.089308009 | 8.510878226 | 0.021928986 | 8.117980604 |
| NM_00101250 6 | hypothetical protein LOC285331 | LOC285331 | -0.13852209 | 0.908449303 | 1.891935509 | 3.711328 | 0.005750826 | 4.0853441 |
| NM_198284 | hypothetical protein LOC349114 | LOC349114 | -0.981394717 | 0.506489857 | 1.785874033 | 3.448273085 | 0.004642538 | 6.808177967 |
| XR_010710 | hypothetical protein LOC700276 | LOC700276 | -0.672295674 | 0.627507377 | 1.397787616 | 2.634971972 | 0.000840237 | 4.199109156 |
| XR_013303 | hypothetical protein LOC707659 | LOC707659 | -0.322058489 | 0.799927697 | 1.308061558 | 2.47608623 | 0.012112484 | 3.095387545 |
| XR_012042 | hypothetical protein LOC709438 | LOC709438 | 0.944738767 | 1.924840318 | 2.408218983 | 5.308186216 | 0.033999178 | 2.757728091 |
| XR_014590 | hypothetical protein LOC721251 | LOC721251 | -0.038962977 | 0.973354352 | 1.225463977 | 2.338306385 | 0.000143932 | 2.402317697 |
| XR_014848 | hypothetical protein LOC723278 | LOC723278 | 0.125408039 | 1.090816206 | 1.426128118 | 2.687245481 | 0.011545102 | 2.463518113 |
| NM_178122 | hypothetical protein LOC90529 | LOC90529 | 0.535971578 | 1.449918268 | 3.464350321 | 11.03756723 | 0.03163105 | 7.612544427 |
| NM_030818 | hypothetical protein MGC10471 | MGC10471 | -0.429615852 | 0.742459454 | 1.445523011 | 2.723615429 | 0.001933467 | 3.668369248 |
| NM_032328 | hypothetical protein MGC12458 | MGC12458 | -0.234272506 | 0.850113569 | 2.09633199 | 4.276207883 | 0.005822335 | 5.030160721 |
| NM_032921 | hypothetical protein MGC15875 | MGC15875 | -0.007641186 | 0.994717535 | 1.38653574 | 2.614501214 | 0.009204978 | 2.628385569 |
| NM_144626 | hypothetical protein MGC17299 | MGC17299 | 0.079606455 | 1.056729741 | 1.916074769 | 3.773948586 | 0.004823502 | 3.571346996 |
| NM_152421 | hypothetical protein MGC20262 | MGC20262 | -0.141390643 | 0.906644801 | 3.802746343 | 13.95534942 | 0.015447849 | 15.39230072 |
| NM_182614 | hypothetical protein MGC20579 | MGC20579 | 0.599801216 | 1.515507735 | 1.887038144 | 3.698750905 | 0.037728576 | 2.440601799 |
| NM_145274 | hypothetical protein MGC21518 | MGC21518 | 0.186038686 | 1.137635734 | 1.430430688 | 2.695271654 | 0.016892911 | 2.369186879 |
| NM_145030 | hypothetical protein MGC22793 | MGC22793 | 0.036884035 | 1.02589568 | 1.653704235 | 3.146404675 | 0.00270891 | 3.066983063 |
| NM_144982 | hypothetical protein MGC23401 | MGC23401 | -0.433834943 | 0.74029134 | 1.668252482 | 3.178293776 | 0.015364326 | 4.293301305 |
| NM_025267 | hypothetical protein MGC2744 | MGC2744 | 0.313387416 | 1.242621926 | 1.291407823 | 2.447667895 | 0.003847399 | 1.969760748 |
| NM_174923 | hypothetical protein MGC31967 | MGC31967 | -0.248286587 | 0.841895697 | 1.691537778 | 3.230008089 | 0.001619082 | 3.836589379 |
| NM_144602 | hypothetical protein MGC32905 | MGC33367 | 0.41608121 | 1.334298276 | 2.051631717 | 4.145745966 | 0.019277946 | 3.107060873 |
| NM_144664 | hypothetical protein MGC33371 | MGC33371 | -0.076350229 | 0.94845404 | 1.569412132 | 2.967837563 | 0.02033891 | 3.129131658 |

| | | | | | | | | |
|---|---|---|---|---|---|---|---|---|
| NM_182539 | hypothetical protein MGC33600 | MGC33600 | 0.663833574 | 1.584286852 | 2.066605757 | 4.188999632 | 0.045082289 | 2.644091647 |
| NM_144668 | hypothetical protein MGC33630 | MGC33630 | 0.616207165 | 1.532840054 | 1.730250488 | 3.317854196 | 0.025571909 | 2.164514287 |
| NM_152390 | hypothetical protein MGC33926 | MGC33926 | -0.780567216 | 0.582137872 | 1.555568361 | 2.939495075 | 0.004774855 | 5.049482634 |
| NM_152479 | hypothetical protein MGC33962 | MGC33962 | 1.184330696 | 2.272579392 | 2.309830362 | 4.958247754 | 0.043471674 | 2.181770974 |
| NM_173525 | hypothetical protein MGC34805 | MGC34805 | 1.186159206 | 2.275461546 | 3.102065466 | 8.586471916 | 0.031069662 | 3.77350781 |
| NM_152314 | hypothetical protein MGC34830 | MGC34830 | 0.370799612 | 1.293069314 | 2.566004811 | 5.921672938 | 0.001991315 | 4.579547959 |
| NM_152453 | hypothetical protein MGC35118 | MGC35118 | 0.242132445 | 1.182739575 | 1.802329735 | 3.487830028 | 0.034661611 | 2.948941679 |
| NM_178565 | hypothetical protein MGC35555 | MGC35555 | 0.451088061 | 1.367070895 | 3.932922815 | 15.27311908 | 0.001432594 | 11.17214852 |
| NM_147189 | hypothetical protein MGC39325 | MGC39325 | 0.268003695 | 1.204140466 | 2.584318575 | 5.997322586 | 0.04380776 | 4.980583873 |
| NM_203306 | hypothetical protein MGC39606 | MGC39606 | -0.161638991 | 0.894008844 | 2.817445265 | 7.049130261 | 0.049237498 | 7.884855176 |
| NM_153361 | hypothetical protein MGC42105 | MGC42105 | 0.15450626 | 1.113040632 | 4.100449177 | 17.1537153 | 0.007339622 | 15.4115805 |
| NM_152459 | hypothetical protein MGC45438 | MGC45438 | 0.014513643 | 1.010110864 | 2.949508677 | 7.724859412 | 0.000580368 | 7.647536213 |
| NM_152499 | hypothetical protein MGC45441 | MGC45441 | -0.331740117 | 0.79457752 | 1.207119194 | 2.308761575 | 0.021881674 | 2.905646733 |
| NM_152689 | hypothetical protein MGC9712 | MGC9712 | 0.637275209 | 1.55538875 | 1.81317299 | 3.51414322 | 0.033660729 | 2.259334343 |
| NM_018607 | hypothetical protein PRO1853 | PRO1853 | -0.061003843 | 0.958596885 | 1.74643194 | 3.355277151 | 0.001989744 | 3.500196176 |
| NM_022462 | hypoxia inducible factor 3, alpha subunit | HIF3A | -0.077855141 | 0.947465199 | 1.877353194 | 3.674003992 | 0.002109608 | 3.877719198 |
| NM_152795 | hypoxia inducible factor 3, alpha subunit | HIF3A | -0.510300355 | 0.702076257 | 3.794683899 | 13.87757802 | 0.005961691 | 19.76648246 |
| CN803406 | IGFBP6 | IGFBP6 | -0.331978924 | 0.794446006 | 1.371012296 | 2.586519909 | 0.00243661 | 3.255752927 |
| NM_178822 | immunoglobulin superfamily, member 10 | IGSF10 | 0.141018671 | 1.102683434 | 4.705725774 | 26.09543913 | 0.035267524 | 23.66539509 |
| NM_176878 | InaD-like | INADL | -1.097965851 | 0.467174731 | 1.908798767 | 3.754963195 | 0.001093028 | 8.037599095 |
| NM_005799 | InaD-like protein | INADL | -0.404741999 | 0.755371366 | 2.53953841 | 5.814029574 | 0.002803438 | 7.696915498 |
| NM_002165 | inhibitor of DNA binding 1, dominant negative helix-loop-helix protein | ID1 | -0.062118081 | 0.957856817 | 1.619004753 | 3.071630658 | 0.012887669 | 3.206774336 |
| NM_181353 | inhibitor of DNA binding 1, dominant negative helix-loop-helix protein | ID1 | 0.120229597 | 1.086907824 | 1.718311942 | 3.290511677 | 0.048857303 | 3.027406376 |
| NM_002167 | inhibitor of DNA binding 3, dominant negative helix-loop-helix protein | ID3 | 0.170563151 | 1.125497733 | 1.73261264 | 3.323291031 | 0.025508403 | 2.952730098 |
| NM_001546 | inhibitor of DNA binding 4, dominant negative helix-loop-helix protein | ID4 | -0.239676456 | 0.846935228 | 1.527185729 | 2.882230526 | 0.049225062 | 3.403129815 |
| NM_032329 | inhibitor of growth family, member 5 | ING5 | -0.11655 | 0.922390781 | 1.879482121 | 3.679429573 | 0.019475774 | 3.98901382 |
| NM_002224 | inositol 1,4,5-triphosphate receptor, type 3 | ITPR3 | 0.033585366 | 1.023552686 | 1.941145258 | 3.840103666 | 0.001664117 | 3.751740109 |
| NM_001005912 | inositol hexaphosphate kinase 2 | IHPK2 | -0.272587443 | 0.827833512 | 2.710652756 | 6.546177654 | 0.005434762 | 7.90760166 |
| NM_016291 | inositol hexaphosphate kinase 2 | IHPK2 | 0.022312858 | 1.015586314 | 1.303669048 | 2.468558865 | 0.026487722 | 2.430673623 |
| NM_198541 | insulin growth factor-like family member 1 | IGFL1 | 0.489120001 | 1.403588469 | 1.947556334 | 3.857206364 | 0.007914472 | 2.748103486 |
| XR_014528 | insulin receptor-related receptor | INSRR | 0.443906087 | 1.360282301 | 2.167820626 | 4.493440897 | 0.049482152 | 3.303314976 |
| NM_000419 | integrin, alpha 2b | ITGA2B | -0.650178842 | 0.637201319 | 1.403975982 | 2.646298823 | 0.031064971 | 4.153002739 |
| NM_000210 | integrin, alpha 6 | ITGA6 | 0.397995176 | 1.317675546 | 1.304811993 | 2.470515304 | 0.00957645 | 1.874904115 |
| NM_003638 | integrin, alpha 8 | ITGA8 | -0.135123616 | 0.910591807 | 3.370043141 | 10.33913181 | 0.000342999 | 11.35429918 |
| NM_017625 | intelectin 1 | ITLN1 | 0.045336502 | 1.031923843 | 1.394009404 | 2.628080385 | 0.002890875 | 2.546777461 |
| NM_002217 | inter-alpha | ITIH3 | -0.483662615 | 0.715159719 | 1.936734802 | 3.828382029 | 0.003389931 | 5.353184649 |
| NM_002038 | interferon, alpha-inducible protein | G1P3 | 0.131395884 | 1.095353001 | 1.123557339 | 2.178835591 | 3.61E-06 | 1.989162936 |
| NM_021258 | interleukin 22 receptor, alpha 1 | IL22RA1 | -0.262899753 | 0.833411116 | 1.426421019 | 2.687791111 | 0.010387994 | 3.22504831 |
| NM_033261 | isopentenyl-diphosphate delta isomerase 2 | IDI2 | 0.523853375 | 1.437790399 | 2.625611647 | 6.171459206 | 0.014017584 | 4.292321892 |
| NM_016604 | jumonji domain containing 1B | JMJD1B | 0.346084123 | 1.271105805 | 1.789836329 | 3.457756627 | 0.002908843 | 2.720274436 |
| NM_020433 | junctophilin 2 | JPH2 | 0.199642216 | 1.148413517 | 1.714576128 | 3.282002023 | 0.010564916 | 2.857857361 |

| | | | | | | | | |
|---|---|---|---|---|---|---|---|---|
| NM_007059 | kaptin | KPTN | 0.408629267 | 1.327424003 | 2.435473478 | 5.409418356 | 0.000446233 | 4.075124711 |
| NM_014315 | kelch domain containing 2 | KLHDC2 | 0.162156039 | 1.118958119 | 2.829857026 | 7.110036797 | 0.011011002 | 6.354158101 |
| NM_000526 | keratin 14 | KRT14 | 3.21078552 | 9.258545201 | 4.906773105 | 29.99755696 | 0.000575789 | 3.239986014 |
| NM_000422 | keratin 17 | KRT17 | 0.841660974 | 1.792112212 | 2.618828473 | 6.142510732 | 0.040996775 | 3.427525738 |
| NM_000224 | keratin 18 | KRT18 | 0.050658704 | 1.035737712 | 1.598183777 | 3.027619234 | 0.000252703 | 2.923152453 |
| NM_015342 | KIAA0073 protein | KIAA0073 | 0.218313917 | 1.163373153 | 3.506329852 | 11.36345666 | 0.019258004 | 9.767679984 |
| NM_015115 | KIAA0276 protein | KIAA0276 | 0.064349809 | 1.045613599 | 1.143906754 | 2.20978614 | 0.006155587 | 2.113386955 |
| NM_024874 | KIAA0319-like | KIAA0319L | 0.02936171 | 1.0205605 | 1.450039022 | 2.732154411 | 0.016564872 | 2.677111657 |
| NM_015330 | KIAA0376 protein | KIAA0376 | -0.000378992 | 0.999737337 | 1.934423137 | 3.822252634 | 0.003625571 | 3.823256862 |
| NM_014802 | KIAA0528 gene product | KIAA0528 | 0.542313731 | 1.456306206 | 1.395869682 | 2.63147134 | 0.013901625 | 1.806949204 |
| NM_014859 | KIAA0672 gene product | KIAA0672 | 0.034007142 | 1.023851969 | 2.60493002 | 6.083619912 | 0.002662237 | 5.94189404 |
| NM_016111 | KIAA0683 gene product | KIAA0683 | 0.245038136 | 1.185124098 | 1.238883468 | 2.360158039 | 0.005176406 | 1.991485991 |
| NM_015037 | KIAA0913 | KIAA0913 | -0.063898654 | 0.95667536 | 1.211741778 | 2.316171013 | 0.001889427 | 2.421062682 |
| NM_025176 | KIAA0980 protein | KIAA0980 | -0.106770762 | 0.928664401 | 1.841544872 | 3.583935993 | 0.012657081 | 3.859236974 |
| NM_014972 | KIAA1049 protein | KIAA1049 | 0.192634303 | 1.142848607 | 1.352971261 | 2.554376634 | 0.009080603 | 2.235096248 |
| XM_044461 | KIAA1102 protein | KIAA1102 | -0.240855165 | 0.846243549 | 3.570382074 | 11.87933419 | 0.002536133 | 14.03772496 |
| NM_019590 | KIAA1217 | KIAA1217 | -0.081479188 | 0.945088155 | 1.714834672 | 3.28259024 | 0.000669582 | 3.473316455 |
| NM_020734 | KIAA1238 protein | KIAA1238 | 0.427545631 | 1.344943559 | 4.753408142 | 26.972328 | 0.013001439 | 20.05461703 |
| NM_015689 | KIAA1277 | KIAA1277 | -0.073146939 | 0.950562281 | 2.5567663 | 5.883873788 | 0.00484569 | 6.189887718 |
| XM_371146 | KIAA1683 | KIAA1683 | -0.486179041 | 0.713913387 | 1.76048076 | 3.388110106 | 0.010864372 | 4.74582795 |
| NM_032531 | kin of IRRE like 3 | KIRREL3 | 0.909454302 | 1.878334886 | 2.050461689 | 4.142385124 | 0.038471204 | 2.205349618 |
| NM_007054 | kinesin family member 3A | KIF3A | 4.644666662 | 25.01404836 | 7.410983089 | 170.187715 | 0.00185839 | 6.803685375 |
| NM_177417 | kinesin light chain 2-like | KLC2L | 0.132356027 | 1.096082224 | 2.157296863 | 4.460782675 | 0.000152983 | 4.069751865 |
| NM_014970 | kinesin-associated protein 3 | KIFAP3 | 0.756623827 | 1.689532177 | 1.292936214 | 2.450262327 | 0.004074104 | 1.450260824 |
| NM_201523 | kinesin-like 8 | KNSL8 | -0.281753377 | 0.822590675 | 1.743932863 | 3.349470075 | 0.008412066 | 4.071855148 |
| NM_152775 | KM-HN-1 protein | KM-HN-1 | -0.082132159 | 0.944660501 | 1.733669006 | 3.325725293 | 0.044720232 | 3.520550811 |
| NM_016270 | Kruppel-like factor 2 | KLF2 | -0.010044284 | 0.993062013 | 1.377632016 | 2.598415271 | 0.00445854 | 2.616568993 |
| NM_004235 | Kruppel-like factor 4 | KLF4 | -0.611657875 | 0.654444214 | 2.634575146 | 6.209921993 | 0.000446798 | 9.488848492 |
| NM_001300 | Kruppel-like factor 6 | KLF6 | -0.636963183 | 0.643065152 | 1.852945893 | 3.612370559 | 0.001388564 | 5.617425463 |
| XM_027074 | l | L3MBTL3 | 0.807673769 | 1.750386806 | 1.68530957 | 3.216093991 | 0.027381819 | 1.837361879 |
| NM_018357 | La ribonucleoprotein domain family, member 6 | LARP6 | -0.033669715 | 0.976932158 | 1.703478072 | 3.256851799 | 0.020283632 | 3.333754317 |
| NM_147190 | LAG1 longevity assurance homolog 5 | LASS5 | -0.230824095 | 0.852147989 | 1.2670648 | 2.406714149 | 0.029749826 | 2.824291297 |
| NM_198129 | laminin, alpha 3 | LAMA3 | -0.760174925 | 0.590424738 | 2.545631705 | 5.838637348 | 0.000557484 | 9.888876553 |
| NM_005560 | laminin, alpha 5 | LAMA5 | -0.542186487 | 0.686729342 | 2.626019463 | 6.173203981 | 0.000123256 | 8.98928239 |
| NM_002292 | laminin, beta 2 | LAMB2 | -0.368423502 | 0.774628506 | 1.97821654 | 3.940057106 | 0.000448954 | 5.086382796 |
| NM_000228 | laminin, beta 3 | LAMB3 | -0.226466245 | 0.854725906 | 1.555461593 | 2.939277544 | 0.00038543 | 3.438853934 |
| NM_018697 | LanC lantibiotic synthetase component C-like 2 | LANCL2 | 0.538114714 | 1.452073735 | 2.468538223 | 5.534826996 | 0.019821726 | 3.811670759 |
| NM_178354 | late cornified envelope 1F | LCE1F | 0.402704986 | 1.321984247 | 2.236716117 | 4.713230104 | 0.031741269 | 3.565269491 |
| NM_178356 | late cornified envelope 4A | LCE4A | 0.246787461 | 1.18656198 | 1.284988219 | 2.436800629 | 0.000789049 | 2.053664849 |
| NM_000627 | latent transforming growth factor beta binding protein 1 | LTBP1 | 0.663302377 | 1.583703628 | 2.131203397 | 4.380827465 | 0.02514819 | 2.766191469 |
| NM_000428 | latent transforming growth factor beta binding protein 2 | LTBP2 | -0.451853396 | 0.731103014 | 1.356968275 | 2.561463396 | 0.012290756 | 3.503560165 |
| XR_013086 | latrophilin 2 precursor | LPHN2P | 0.386977069 | 1.307650564 | 2.616983299 | 6.134659621 | 0.037784782 | 4.691360054 |
| CN641580 | LDLR | LDLR | 0.49549019 | 1.409799686 | 2.940010586 | 7.674169267 | 0.004994947 | 5.443446571 |
| NM_000229 | lecithin-cholesterol acyltransferase | LCAT | -0.199128704 | 0.871076479 | 1.927652453 | 3.804356521 | 0.011897386 | 4.367419638 |

| | | | | | | | | |
|---|---|---|---|---|---|---|---|---|
| NM_203471 | lectin, galactoside-binding, soluble, 14 | LGALS14 | 0.236940542 | 1.178490837 | 1.225227108 | 2.337922502 | 0.004333142 | 1.983827475 |
| NM_018192 | leprecan-like 1 | LEPREL1 | 0.015795951 | 1.011009078 | 2.822297847 | 7.072880308 | 0.003636713 | 6.995862314 |
| NM_001003679 | leptin receptor | LEPR | 0.011318995 | 1.007876588 | 3.606909318 | 12.18394406 | 0.01028892 | 12.08872614 |
| NM_004524 | lethal giant larvae homolog 2 | LLGL2 | -0.212439561 | 0.863076556 | 2.085166705 | 4.243241247 | 0.001051248 | 4.916413518 |
| XR_011718 | leucine rich repeat and sterile alpha motif containing 1 | LRSAM1 | -0.362952534 | 0.777571618 | 2.116829718 | 4.33739766 | 0.002909844 | 5.578132688 |
| NM_031294 | leucine rich repeat containing 48 | LRRC48 | 0.567288264 | 1.481735834 | 1.808351137 | 3.502417667 | 0.016991123 | 2.363726102 |
| NM_024652 | leucine-rich repeat kinase 1 | LRRK1 | -0.095747604 | 0.935787197 | 1.55399342 | 2.93628788 | 0.010013446 | 3.137773085 |
| NM_015541 | leucine-rich repeats and immunoglobulin-like domains 1 | LRIG1 | 0.020139142 | 1.014057277 | 2.705949299 | 6.524870636 | 0.0019316 | 6.434420209 |
| NM_005575 | leucyl/cystinyl aminopeptidase | LNPEP | 0.31364311 | 1.24284218 | 1.491404782 | 2.811626158 | 0.028453665 | 2.262255179 |
| NM_002344 | leukocyte tyrosine kinase | LTK | 0.047354483 | 1.033368265 | 1.91036338 | 3.759037687 | 0.018777937 | 3.637655436 |
| XR_013643 | LGP1 homolog | LGP1 | 0.230887 | 1.173556255 | 1.730418802 | 3.318241301 | 0.014133431 | 2.827509365 |
| NM_025265 | likely homolog of yeast SEN2 | SEN2L | -0.064431652 | 0.956321985 | 1.824288705 | 3.54132366 | 0.014440067 | 3.703066243 |
| NM_014583 | LIM and cysteine-rich domains 1 | LMCD1 | 0.074312247 | 1.052859003 | 2.146242363 | 4.42673301 | 0.003742952 | 4.204487968 |
| NM_017980 | LIM and senescent cell antigen-like domains 2 | LIMS2 | -0.108150524 | 0.927776672 | 2.031401221 | 4.088017071 | 0.0018237 | 4.406251196 |
| NM_007078 | LIM domain binding 3 | LDB3 | -0.110895214 | 0.926013278 | 1.870134772 | 3.655667285 | 0.006527652 | 3.947748236 |
| NM_006769 | LIM domain only 4 | LMO4 | 0.187121114 | 1.138489601 | 1.564258292 | 2.957254266 | 0.010082752 | 2.597524179 |
| NM_022363 | LIM homeobox 5 | LHX5 | 0.341998064 | 1.267510821 | 1.385422709 | 2.612484919 | 0.02267296 | 2.06111449 |
| NM_014647 | limkain b1 | LKAP | -0.371547239 | 0.772953087 | 1.250629848 | 2.379452819 | 0.025220383 | 3.078392284 |
| NM_005357 | lipase, hormone-sensitive | LIPE | 0.372466393 | 1.294564092 | 2.214842413 | 4.642308547 | 0.029207231 | 3.586001323 |
| NM_139248 | lipase, member H | LIPH | -0.016662556 | 0.988516837 | 2.530934308 | 5.779458424 | 0.005810443 | 5.846595837 |
| CN802260 | LOC283820 | LOC283820 | -0.131022098 | 0.913184263 | 1.370978132 | 2.586458659 | 0.003904009 | 2.832351327 |
| NM_002334 | low density lipoprotein receptor-related protein 4 | LRP4 | -0.446781914 | 0.73367757 | 3.114851492 | 8.662908577 | 0.007898816 | 11.80751454 |
| NM_002337 | low density lipoprotein receptor-related protein associated protein 1 | LRPAP1 | 0.029108577 | 1.02038145 | 1.385535133 | 2.612688508 | 0.001164798 | 2.560501769 |
| NM_018032 | LUC7-like | LUC7L | -0.230793001 | 0.852166356 | 2.261412126 | 4.794605536 | 0.006808653 | 5.626372717 |
| NM_016019 | LUC7-like 2 | LUC7L2 | -0.114028213 | 0.924004502 | 1.390207155 | 2.621163151 | 0.001514197 | 2.836742835 |
| NM_000894 | luteinizing hormone beta polypeptide | LHB | 0.794310038 | 1.734247781 | 2.052479541 | 4.148183 | 0.026881826 | 2.391920604 |
| NM_006152 | lymphoid-restricted membrane protein | LRMP | 0.517220755 | 1.431195498 | 1.596632714 | 3.024365944 | 0.004669022 | 2.113174579 |
| NM_053051 | LYST-interacting protein LIP8 | LIP8 | 0.265679624 | 1.202202249 | 1.403028417 | 2.6445613 | 0.010062682 | 2.19976406 |
| DQ159933 | Macaca fascicularis GluR5 | GRIK1 | 0.125432105 | 1.090834402 | 3.089114339 | 8.509735785 | 0.005783945 | 7.801125239 |
| NM_006770 | macrophage receptor with collagenous structure | MARCO | -0.076026739 | 0.948666732 | 2.915827834 | 7.546605367 | 0.000667956 | 7.954959431 |
| NM_002447 | macrophage stimulating 1 receptor | MST1R | -0.701630886 | 0.61487673 | 1.320266962 | 2.497123133 | 0.004066379 | 4.061176836 |
| BC067766 | MAD, mothers against decapentaplegic homolog 9 | MADH9 | -0.326565165 | 0.79743279 | 1.766403066 | 3.402046984 | 0.013737008 | 4.266249177 |
| NM_032228 | male sterility domain containing 2 | MLSTD2 | 0.249817823 | 1.189056957 | 1.4841601 | 2.797542599 | 0.013334591 | 2.352740617 |
| NM_002395 | malic enzyme 1, NADP | ME1 | -0.422582378 | 0.746087955 | 1.35808717 | 2.563450732 | 0.00655067 | 3.43585594 |
| NM_139208 | mannan-binding lectin serine protease 2 | MASP2 | 0.752827136 | 1.685091739 | 1.381203607 | 2.604855975 | 0.007806287 | 1.545824429 |
| XR_014258 | mannosidase, alpha, class 1C, member 1 | MAN1C1 | 0.157046415 | 1.11500209 | 1.298121247 | 2.459084392 | 0.006079211 | 2.20545272 |
| NM_006715 | mannosidase, alpha, class 2C, member 1 | MAN2C1 | -0.120616719 | 0.919794376 | 2.063437486 | 4.179810355 | 0.00078447 | 4.544287793 |
| XR_009857 | MAP | MAP | 0.138536907 | 1.100788197 | 1.687332119 | 3.220605872 | 0.019727272 | 2.925727112 |
| NM_016586 | MAP3K12 binding inhibitory protein 1 | MBIP | -0.927985724 | 0.525591656 | 3.421672271 | 10.71583428 | 0.002491715 | 20.38813622 |
| NM_020690 | MASK-4E-BP3 alternate reading frame gene | MASK-BP3 | 0.18183086 | 1.134322491 | 1.584645981 | 2.999341889 | 0.001639078 | 2.644170342 |

| | | | | | | | | |
|---|---|---|---|---|---|---|---|---|
| NM_145015 | MAS-related GPR, member F | MRGPRF | -0.535088529 | 0.690116328 | 1.421904228 | 2.679389331 | 6.72E-06 | 3.882518385 |
| NM_006454 | MAX dimerization protein 4 | MXD4 | -0.211586548 | 0.863587013 | 1.054591127 | 2.077129446 | 0.000220578 | 2.405234695 |
| NM_002392 | Mdm2, transformed 3T3 cell double minute 2, p53 binding protein | MDM2 | 3.472745782 | 11.10198529 | 1.319965944 | 2.496602163 | 0.01445395 | 0.224878893 |
| NM_002393 | Mdm4, transformed 3T3 cell double minute 4, p53 binding protein | MDM4 | 0.209715572 | 1.156460165 | 1.553514723 | 2.93531376 | 0.049578876 | 2.538188386 |
| NM_002398 | Meis1, myeloid ecotropic viral integration site 1 homolog | MEIS1 | -0.009244926 | 0.993612394 | 2.591970818 | 6.02921768 | 0.014274276 | 6.06797753 |
| NM_138703 | melanoma antigen family E, 2 | MAGEE2 | 0.81707454 | 1.761829774 | 4.356186115 | 20.48060055 | 0.008090424 | 11.62461939 |
| NM_201222 | melanoma antigen, family D, 2 | MAGED2 | 0.145429794 | 1.106060111 | 1.253480069 | 2.384158367 | 0.00389256 | 2.155541406 |
| NM_006533 | melanoma inhibitory activity | MIA | -0.163401744 | 0.892917169 | 1.34232824 | 2.535601875 | 0.003749908 | 2.83968319 |
| NM_024101 | melanophilin | MLPH | -0.475454533 | 0.719240152 | 3.471058519 | 11.08900887 | 0.000820032 | 15.41767216 |
| NM_002389 | membrane cofactor protein | CD46 | -0.318399013 | 0.801959334 | 2.612629014 | 6.116172147 | 0.002617341 | 7.626536518 |
| NM_001932 | membrane protein, palmitoylated 3 | MPP3 | -0.028638145 | 0.980345274 | 3.224229015 | 9.345222512 | 0.001128978 | 9.532582815 |
| NM_022474 | membrane protein, palmitoylated 5 | MPP5 | 0.540883233 | 1.454862927 | 2.57052384 | 5.940250784 | 5.69E-05 | 4.083031242 |
| NM_003791 | membrane-bound transcription factor protease, site 1 | MBTPS1 | 0.204255149 | 1.152091379 | 1.234964394 | 2.353755383 | 0.004343524 | 2.043028379 |
| XR_013910 | meningioma expressed antigen 5 | LOC712370 | 0.30671683 | 1.23688968 | 2.212342699 | 4.634271923 | 0.012460615 | 3.746714034 |
| NM_005588 | meprin A, alpha | MEP1A | 0.226929469 | 1.170341424 | 2.480477013 | 5.580819604 | 0.001054416 | 4.768539752 |
| NM_004689 | metastasis associated 1 | MTA1 | 0.010601121 | 1.007375201 | 1.564252741 | 2.957242887 | 0.000158091 | 2.935592304 |
| NM_052897 | methyl-CpG binding domain protein 6 | MBD6 | -0.002845517 | 0.998029582 | 1.625729298 | 3.085981254 | 0.000358671 | 3.092073932 |
| NM_020166 | methylcrotonoyl-Coenzyme A carboxylase 1 | alpha | -0.097008905 | 0.934969426 | 2.025925214 | 4.072529682 | 0.009692059 | 4.355789151 |
| NM_052845 | methylmalonic aciduria | MMAB | 0.29777559 | 1.229247644 | 1.127596956 | 2.184944987 | 0.026985429 | 1.777465263 |
| NM_019852 | methyltransferase like 3 | METTL3 | -0.092529329 | 0.937877023 | 2.03665999 | 4.102945498 | 0.000922986 | 4.374715871 |
| NM_00100367 6 | MGC4707 protein | MGC4707 | -0.670580421 | 0.628253879 | 2.289930758 | 4.890326394 | 0.018441052 | 7.783997135 |
| XM_376355 | microfibrillar-associated protein 3-like | MFAP3L | 0.760296196 | 1.693838346 | 2.391324588 | 5.246388295 | 0.000712609 | 3.097337067 |
| NM_002404 | microfibrillar-associated protein 4 | MFAP4 | 0.099142366 | 1.071136518 | 3.976279084 | 15.73907756 | 0.00013026 | 14.69381101 |
| NM_033044 | microtubule-actin crosslinking factor 1 | MACF1 | -0.023727396 | 0.983687929 | 2.18156136 | 4.536442461 | 0.003659102 | 4.611668322 |
| NM_020774 | mindbomb homolog 1 | MIB1 | -0.452041998 | 0.731007444 | 1.909843332 | 3.757682912 | 0.037010835 | 5.140416753 |
| NM_153255 | minichromosome maintenance deficient domain containing 1 | MCMDC1 | -0.44093602 | 0.736656511 | 2.39831957 | 5.271887447 | 0.032965156 | 7.156506959 |
| NM_014341 | mitochondrial carrier homolog 1 | MTCH1 | 0.150763423 | 1.110156772 | 1.345495885 | 2.541175272 | 0.0094365 | 2.289023799 |
| NM_145729 | mitochondrial ribosomal protein L24 | MRPL24 | 0.392443816 | 1.312614992 | 1.45708123 | 2.745523442 | 0.001714156 | 2.091644129 |
| NM_032476 | mitochondrial ribosomal protein S6 | MRPS6 | 0.299794764 | 1.230969284 | 1.291272445 | 2.447438224 | 0.002582909 | 1.988220385 |
| XR_011805 | mitochondrial tumor suppressor 1 isoform 1 | MTUS1 | -0.131472403 | 0.912899277 | 1.74043188 | 3.341351784 | 0.022704342 | 3.660153829 |
| NM_002746 | mitogen-activated protein kinase 3 | MAPK3 | -0.013868496 | 0.990433147 | 1.439552203 | 2.712366634 | 0.000494996 | 2.738566093 |
| NM_005922 | mitogen-activated protein kinase kinase kinase 4 | MAP3K4 | 0.092471892 | 1.066195422 | 1.521023019 | 2.869944861 | 0.022208027 | 2.691762506 |
| NM_004672 | mitogen-activated protein kinase kinase kinase 6 | MAP3K6 | 0.307557517 | 1.23761065 | 4.075757186 | 16.86262446 | 0.000467951 | 13.62514492 |
| CB550393 | MMPL0003_B02 MMPL cDNA sequence | | 0.072956639 | 1.051870164 | 3.663124481 | 12.66806685 | 0.008177347 | 12.04337501 |
| CB550080 | MMPL0011_H01 MMPL cDNA sequence | | -0.274174026 | 0.826923614 | 3.585462007 | 12.0041555 | 0.000923859 | 14.51664373 |
| CB549194 | MMPL0013_G06 MMPL cDNA sequence | | 0.241594068 | 1.182298289 | 2.335946352 | 5.048820379 | 0.009796928 | 4.270343978 |
| CB550361 | MMPL0014_E08 MMPL cDNA sequence | | 0.057387819 | 1.040579953 | 1.389817839 | 2.620455917 | 0.0054756 | 2.518264848 |
| CB548748 | MMPL0016_A07 MMPL cDNA sequence | | -0.335733518 | 0.792381159 | 1.544151974 | 2.916325948 | 0.015154078 | 3.68045847 |
| CB548968 | MMPL0018_H05 MMPL cDNA sequence | | -0.158924765 | 0.895692378 | 2.923428058 | 7.586466345 | 0.002145169 | 8.469946302 |
| CB554423 | MMSP0024_G06 MMSP cDNA sequence | | 0.438909066 | 1.355578881 | 1.161423209 | 2.236779756 | 0.004961849 | 1.650055034 |

| | | | | | | | | |
|---|---|---|---|---|---|---|---|---|
| NM_017898 | MOCO sulphurase C-terminal domain containing 2 | MOSC2 | -0.610741151 | 0.654860196 | 3.121969734 | 8.705756893 | 0.026028666 | 13.2940694 |
| NM_031419 | molecule possessing ankyrin repeats induced by lipopolysaccharide | MAIL | 0.011064827 | 1.00769904 | 1.992613068 | 3.979571419 | 0.005163378 | 3.949166627 |
| D86962 | mRNA for KIAA0207 gene, partial cds | mRNA for KIAA0207 gene, partial cds | -0.05321558 | 0.96378578 | 3.262906815 | 9.599151023 | 0.00064779 | 9.959838817 |
| AB209405 | mRNA for RYK receptor-like tyrosine kinase isoform 1 variant protein | RYKRLTK | 0.651342266 | 1.57062881 | 1.818129871 | 3.526238048 | 0.006632215 | 2.245112292 |
| AB209485 | mRNA for vascular endothelial growth factor variant protein | VEGF | -0.922947495 | 0.527430353 | 2.08065919 | 4.230004475 | 0.002201455 | 8.020024731 |
| BX537550 | mRNA; cDNA DKFZp686J2446 | DKFZp686J2446 | 0.328672742 | 1.255857473 | 3.242336179 | 9.463252884 | 0.025576135 | 7.535292091 |
| NM_021924 | mucin and cadherin-like | MUCDHL | 3.334050141 | 10.08437761 | 4.361573821 | 20.5572278 | 0.001391747 | 2.038522217 |
| NM_031264 | mucin and cadherin-like | MUCDHL | 4.066525196 | 16.75506289 | 5.712821116 | 52.44819128 | 0.002475417 | 3.130289133 |
| NM_130761 | mucosal vascular addressin cell adhesion molecule 1 | MADCAM1 | -0.440330379 | 0.736965823 | 1.530066793 | 2.887992094 | 0.008571214 | 3.918759872 |
| NM_007351 | multimerin 1 | MMRN1 | 0.532658123 | 1.446592045 | 2.495344104 | 5.638627755 | 0.030629975 | 3.897870014 |
| NM_205853 | musculoskeletal, embryonic nuclear protein 1 | MUSTN1 | -0.478881466 | 0.717533718 | 2.196328663 | 4.583115562 | 0.038718799 | 6.387317344 |
| NM_032133 | MYCBP associated protein | MYCBPAP | 0.081692921 | 1.058259119 | 2.542639373 | 5.826539826 | 0.006139877 | 5.505778048 |
| NM_004991 | myelodysplasia syndrome 1 | MDS1 | 0.026176153 | 1.018309528 | 1.257845655 | 2.391383741 | 0.002557935 | 2.348385904 |
| NM_022443 | myeloid leukemia factor 1 | MLF1 | 0.178403664 | 1.131631047 | 2.231653246 | 4.696718884 | 0.013374339 | 4.150397691 |
| NM_001009569 | myeloid/lymphoid or mixed-lineage leukemia | MLLT10 | 0.076236026 | 1.054263888 | 3.433194317 | 10.80175867 | 0.002910304 | 10.24578267 |
| NM_003970 | myomesin | MYOM2 | 0.265013643 | 1.201647413 | 2.651192542 | 6.281863271 | 0.00478976 | 5.227709229 |
| XR_010160 | myomesin 1 | MYOM1 | 0.268856444 | 1.204852419 | 1.819197277 | 3.52884797 | 0.000676166 | 2.928863249 |
| NM_001010985 | myosin binding protein H-like | MYBPHL | -0.339623193 | 0.790247684 | 1.621252601 | 3.076420264 | 0.00269856 | 3.892982322 |
| NM_025109 | myosin head domain containing 1 | MYOHD1 | -0.55038113 | 0.682839713 | 1.508792932 | 2.845718451 | 0.007499701 | 4.167476491 |
| NM_012223 | myosin IB | MYO1B | -0.497381666 | 0.708391268 | 2.093035628 | 4.266448483 | 0.033941355 | 6.022728788 |
| NM_015194 | myosin ID | MYO1D | -0.200215075 | 0.870420792 | 1.257382576 | 2.390616273 | 0.003936295 | 2.746506397 |
| NM_006471 | myosin regulatory light chain MRCL3 | MRCL3 | 0.60931157 | 1.525531078 | 1.635713893 | 3.107412769 | 0.006639716 | 2.036938358 |
| XR_013772 | myosin X | MYOX | 0.477086258 | 1.391929612 | 1.369325717 | 2.583497911 | 0.007285741 | 1.856054996 |
| NM_005964 | myosin, heavy polypeptide 10, non-muscle | MYH10 | -0.071142713 | 0.951883742 | 2.490335527 | 5.619086181 | 0.005338671 | 5.903122338 |
| NM_022844 | myosin, heavy polypeptide 11, smooth muscle | MYH11 | 0.290154028 | 1.222770819 | 3.954774312 | 15.50621117 | 3.40E-05 | 12.68120806 |
| XR_012588 | myosin, heavy polypeptide 7B, cardiac muscle, beta | MYH7 | -0.237277552 | 0.848344678 | 2.182095025 | 4.53812084 | 0.001034983 | 5.349383285 |
| NM_021223 | myosin, light polypeptide 7, regulatory | MYL7 | -0.295542447 | 0.814765927 | 1.753639653 | 3.372082079 | 0.003313394 | 4.138712687 |
| NM_181526 | myosin, light polypeptide 9, regulatory | MYL9 | -0.298656328 | 0.81300925 | 1.563336128 | 2.955364607 | 5.36E-06 | 3.635093459 |
| NM_053025 | myosin, light polypeptide kinase | MYLK | 0.109208436 | 1.078636259 | 1.718280387 | 3.290439708 | 0.006475355 | 3.05055544 |
| NM_006766 | MYST histone acetyltransferase | MYST3 | 0.261403265 | 1.198644022 | 1.640769336 | 3.11832076 | 0.008112145 | 2.601540326 |
| NM_017852 | NACHT, leucine rich repeat and PYD containing 2 | NALP2 | -0.571164716 | 0.673073184 | 1.862700543 | 3.63687803 | 0.014728156 | 5.403391661 |
| NM_006647 | NADPH oxidase activator 1 | NOXA1 | -0.197498377 | 0.872061401 | 1.853650909 | 3.614136283 | 0.004233279 | 4.14435988 |
| NM_000906 | natriuretic peptide receptor A/guanylate cyclase A | NPR1 | 0.154568508 | 1.113088658 | 2.626666444 | 6.175972994 | 0.000136267 | 5.548500517 |

| | | | | | | | | |
|---|---|---|---|---|---|---|---|---|
| NM_000907 | natriuretic peptide receptor B/guanylate cyclase B | NPR2 | -0.024319806 | 0.983284083 | 1.344213448 | 2.53891738 | 0.00701787 | 2.582079202 |
| NM_005385 | natural killer-tumor recognition sequence | NKTR | -0.343895048 | 0.787911202 | 2.528763266 | 5.770767734 | 8.97E-05 | 7.324134648 |
| NM_205842 | NCK-associated protein 1 | NCKAP1 | 0.161453964 | 1.11841372 | 1.199475737 | 2.296562008 | 0.010114587 | 2.053410082 |
| NM_003635 | N-deacetylase/N-sulfotransferase | NDST2 | 0.657005324 | 1.576806167 | 1.152598403 | 2.223139383 | 0.042570005 | 1.409900233 |
| NM_016250 | NDRG family member 2 | NDRG2 | 0.285136931 | 1.218525913 | 3.614011934 | 12.24407544 | 0.000972913 | 10.04826841 |
| NM_006175 | nebulin-related anchoring protein | NRAP | 0.542331755 | 1.4563244 | 2.70430856 | 6.517454291 | 0.011710089 | 4.47527645 |
| NM_024608 | nei endonuclease VIII-like 1 | NEIL1 | 0.274346421 | 1.209446054 | 3.614373415 | 12.24714369 | 0.000688556 | 10.12624222 |
| XR_014490 | nephroretinin | NPHP4 | -0.140798853 | 0.90701678 | 1.60411963 | 3.040101791 | 0.005058737 | 3.351759149 |
| NM_014380 | nerve growth factor receptor | NGFRAP1 | 0.245949811 | 1.185873244 | 2.91920145 | 7.564273094 | 0.001222226 | 6.378652299 |
| NM_181351 | neural cell adhesion molecule 1 | NCAM1 | -0.268777507 | 0.830022582 | 1.932648831 | 3.817554705 | 0.037136262 | 4.599338365 |
| NM_020795 | neuroligin 2 | NLGN2 | 3.027452922 | 8.153688955 | 3.318384808 | 9.97546994 | 0.021093724 | 1.223430277 |
| NM_182964 | neuron navigator 2 | NAV2 | 0.2343847 | 1.176404901 | 1.703472281 | 3.256838726 | 0.029891127 | 2.768467492 |
| NM_003717 | neuropeptide FF-amide peptide precursor | NPFF | 0.184803103 | 1.136661833 | 1.264289439 | 2.402088723 | 0.008002978 | 2.113283523 |
| NM_003873 | neuropilin 1 | NRP1 | -0.339780992 | 0.790161253 | 3.011244977 | 8.062599039 | 0.003901257 | 10.2037388 |
| NM_145912 | NFAT activating protein with ITAM motif 1 | NFAM1 | 0.671933858 | 1.593207148 | 1.576217023 | 2.981869299 | 0.032865474 | 1.871614311 |
| NM_002498 | NIMA | NEK3 | 0.17488595 | 1.12887516 | 2.703257015 | 6.512705611 | 0.016370714 | 5.769199148 |
| NM_007184 | nischarin | NISCH | -0.1577346 | 0.896431593 | 3.108293096 | 8.623616945 | 0.000413951 | 9.619938668 |
| NM_052946 | nitric oxide synthase trafficking | NOSTRIN | 0.248601398 | 1.188054812 | 1.951333358 | 3.867317891 | 0.011661717 | 3.255167903 |
| NM_145285 | NK2 transcription factor related, locus 3 | NKX2-3 | 0.678898941 | 1.600917475 | 4.109313789 | 17.25944047 | 0.012630667 | 10.78096825 |
| NM_018044 | NOL1/NOP2/Sun domain family, member 5 | NSUN5 | 0.340738565 | 1.266404744 | 1.586984245 | 3.004207047 | 0.030664527 | 2.372232938 |
| NM_002513 | non-metastatic cells 3, protein expressed in | NME3 | -0.030655464 | 0.978975416 | 2.174861936 | 4.515425477 | 6.88E-05 | 4.612399253 |
| NM_004557 | Notch homolog 4 | NOTCH4 | 0.024049268 | 1.016809396 | 2.315459081 | 4.977630296 | 0.02699421 | 4.895342545 |
| NM_020317 | NPD014 protein | NPD014 | -0.189356549 | 0.87699678 | 2.107860016 | 4.310514312 | 0.011886698 | 4.915085677 |
| NM_173474 | N-terminal asparagine amidase | NTAN1 | 0.207497509 | 1.154683538 | 1.502923811 | 2.834165116 | 0.001030616 | 2.454495126 |
| NM_002482 | nuclear autoantigenic sperm protein | NASP | 0.629420632 | 1.546943636 | 2.599927325 | 6.062560861 | 0.005232634 | 3.919057372 |
| NM_003297 | nuclear receptor subfamily 2, group C, member 1 | NR2C1 | -0.243679665 | 0.844588398 | 3.167293518 | 8.983598912 | 0.047195659 | 10.63665915 |
| NM_005654 | nuclear receptor subfamily 2, group F, member 1 | NR2F1 | -0.016367605 | 0.988718954 | 1.529682941 | 2.887223801 | 0.002118943 | 2.920166332 |
| NM_002135 | nuclear receptor subfamily 4, group A, member 1 | NR4A1 | 0.009358262 | 1.006507737 | 1.281423414 | 2.430786886 | 7.00E-05 | 2.415070244 |
| NM_006362 | nuclear RNA export factor 1 | NXF1 | -0.030481809 | 0.979093261 | 1.895036582 | 3.719314079 | 0.006131688 | 3.798733203 |
| NM_006392 | nucleolar protein 5A | NOL5A | 0.428711318 | 1.346030702 | 1.749835037 | 3.363201078 | 0.004285626 | 2.498606512 |
| NM_016122 | NY-REN-58 antigen | NY-REN-58 | -0.34841757 | 0.785445146 | 1.118059282 | 2.170547931 | 0.021074321 | 2.763462149 |
| NM_145260 | odd-skipped homolog | ODD | -0.376152253 | 0.770489791 | 2.529386145 | 5.773259783 | 0.017828029 | 7.492973756 |
| NM_014582 | odorant binding protein 2A | OBP2A | 0.355699834 | 1.279606152 | 1.579413721 | 2.988483797 | 0.029423074 | 2.335471576 |
| NM_198474 | olfactomedin-like 1 | OLFML1 | -0.645256533 | 0.639379089 | 1.703618641 | 3.257169145 | 0.007570899 | 5.094269114 |
| NM_001004713 | olfactory receptor, family 1, subfamily I, member 1 | OR1I1 | 0.737572931 | 1.667368438 | 4.845270065 | 28.74561647 | 0.012193967 | 17.24011071 |
| XR_014601 | olfactory receptor, family 7, subfamily D, member 4 | ORID2 | 0.696655654 | 1.620743351 | 3.609199604 | 12.20330149 | 0.035020909 | 7.529447202 |
| NM_003605 | O-linked N-acetylglucosamine | OGT | -0.288672933 | 0.818654754 | 2.947851037 | 7.715988733 | 0.001028267 | 9.42520482 |
| NM_007346 | opioid growth factor receptor | OGFR | 0.574570098 | 1.489233627 | 2.443116656 | 5.438152678 | 0.012887033 | 3.651645101 |
| NM_003611 | oral-facial-digital syndrome 1 | OFD1 | -0.123035905 | 0.918253309 | 1.863800773 | 3.63965265 | 0.006969096 | 3.963669517 |
| NM_002537 | ornithine decarboxylase antizyme 2 | OAZ2 | 0.578432124 | 1.493225576 | 1.571496694 | 2.972128917 | 0.011449885 | 1.990408526 |

| | | | | | | | | |
|---|---|---|---|---|---|---|---|---|
| U87259 | oviductal glycoprotein mRNA, complete cds | oviductal glycoprotein mRNA, complete cds | 0.22917226 | 1.172162234 | 1.590700602 | 3.011955807 | 0.006648134 | 2.56957247 |
| NM_181354 | oxidation resistance 1 | OXR1 | -0.282292356 | 0.822283419 | 1.576037036 | 2.981497312 | 0.025686892 | 3.625875511 |
| NM_002543 | oxidised low density lipoprotein | OLR1 | -1.012066995 | 0.49583534 | 1.492764863 | 2.814278029 | 0.005683563 | 5.675831878 |
| NM_145047 | oxidored-nitro domain-containing protein | NOR1 | 0.560380264 | 1.474657854 | 1.662020072 | 3.164593233 | 0.005462851 | 2.145984728 |
| NM_017784 | oxysterol binding protein-like 10 | OSBPL10 | 0.4532692 | 1.369139264 | 2.150752583 | 4.440593723 | 0.005845639 | 3.243346999 |
| NM_148909 | oxysterol binding protein-like 9 | OSBPL9 | -0.030422112 | 0.979133775 | 1.540384941 | 2.908721039 | 0.004930142 | 2.970708511 |
| NM_012385 | p8 protein | candidate of metastasis 1 | -0.002615605 | 0.998188643 | 1.784326944 | 3.444577276 | 0.001952071 | 3.450827957 |
| CO647467 | PAG | PAG | -0.194175139 | 0.874072504 | 1.887043298 | 3.698764119 | 0.004492035 | 4.231644517 |
| NM_017734 | palmdelphin | PALMD | 0.097876891 | 1.070197372 | 1.692922239 | 3.233109207 | 0.027509616 | 3.021040129 |
| NM_138934 | palmitoyl-protein thioesterase 2 | PPT2 | 0.231606633 | 1.174141784 | 2.889876744 | 7.412071223 | 0.004055251 | 6.312756538 |
| NM_001034171 | Pan troglodytes centromere protein J | CENPJ | -0.141081112 | 0.906839343 | 2.445248766 | 5.44619548 | 0.003710977 | 6.005689455 |
| NM_173462 | papilin, proteoglycan-like sulfated glycoprotein | PAPLN | -0.120319123 | 0.919984129 | 1.990755912 | 3.974451882 | 0.00068249 | 4.32013092 |
| NM_000940 | paraoxonase 3 | PON3 | 0.120712559 | 1.087271742 | 2.666958099 | 6.350886991 | 0.002922978 | 5.841122091 |
| NM_018282 | paraspeckle component 1 | PSPC1 | -0.022188542 | 0.984737742 | 1.930472651 | 3.811800597 | 0.012576378 | 3.87087895 |
| NM_000316 | parathyroid hormone receptor 1 | PTHR1 | -0.012770435 | 0.991187271 | 3.475703052 | 11.12476574 | 0.000253297 | 11.22367696 |
| NM_018222 | parvin, alpha | PARVA | 0.145574131 | 1.106170775 | 1.828736149 | 3.55225746 | 0.008018283 | 3.21131017 |
| NM_000264 | patched homolog | PTCH | 0.331473101 | 1.258297536 | 3.08093091 | 8.461602479 | 0.046767003 | 6.724643605 |
| NM_015022 | PDZ domain containing 3 | PDZK3 | 0.151765936 | 1.110928476 | 2.582008228 | 5.987726095 | 0.008320724 | 5.389839422 |
| NM_024895 | PDZ domain containing 7 | PDZK7 | 0.130057542 | 1.094337348 | 1.224659593 | 2.337003011 | 0.005528966 | 2.135541673 |
| NM_145065 | pellino 3 alpha | MGC35521 | 0.225180986 | 1.168923881 | 1.381142047 | 2.604744828 | 0.02306714 | 2.228327157 |
| NM_021255 | pellino homolog 2 | PELI2 | 0.605817404 | 1.521840755 | 1.460161684 | 2.75139197 | 0.045945271 | 1.807936843 |
| NM_052890 | peptidoglycan recognition protein 2 | PGLYRP2 | 0.483031435 | 1.397677426 | 1.183542351 | 2.271337905 | 0.044784622 | 1.625080196 |
| NM_020956 | periaxin | PRX | 0.123667634 | 1.089501086 | 1.251092218 | 2.380215533 | 0.000814701 | 2.184683947 |
| NM_002616 | period homolog 1 | PER1 | 0.256007351 | 1.194169272 | 1.319946189 | 2.496567976 | 0.003087 | 2.090631567 |
| NM_022817 | period homolog 2 | PER2 | 0.747459171 | 1.678833517 | 1.827471435 | 3.5491448 | 0.028117452 | 2.114054052 |
| NM_016831 | period homolog 3 | PER3 | -0.553991577 | 0.681132994 | 2.11853203 | 4.342518606 | 0.01209001 | 6.375434231 |
| NM_000304 | peripheral myelin protein 22 | PMP22 | -0.000521387 | 0.999638667 | 1.635023305 | 3.105925673 | 0.00954523 | 3.107048352 |
| NM_000466 | peroxisome biogenesis factor 1 | PEX1 | -0.26013934 | 0.835007268 | 2.751551351 | 6.734409038 | 0.004279829 | 8.065090324 |
| NM_015020 | PH domain and leucine rich repeat protein phosphatase-like | PHLPPL | -0.300038188 | 0.812230897 | 2.280921755 | 4.859883584 | 0.034312451 | 5.983376897 |
| NM_024165 | PHD finger protein 1 | PHF1 | 0.173245838 | 1.12759254 | 1.24698105 | 2.37344242 | 0.001014541 | 2.104875951 |
| NM_018288 | PHD finger protein 10 | PHF10 | -0.019177034 | 0.986795448 | 1.946075112 | 3.853248185 | 0.003699696 | 3.904809444 |
| NM_016119 | PHD finger protein 11 | PHF11 | 0.250817495 | 1.189881164 | 1.130917566 | 2.189979805 | 0.002362568 | 1.840502961 |
| NM_002686 | phenylethanolamine N-methyltransferase | PNMT | -0.585950217 | 0.666210401 | 2.604198248 | 6.080534924 | 0.000701848 | 9.127048925 |
| NM_003713 | phosphatidic acid phosphatase type 2B | PPAP2B | -0.265964065 | 0.831642813 | 1.750736805 | 3.36530393 | 0.000470266 | 4.046573694 |
| XR_013095 | phosphatidylethanolamine-binding protein 4 | PEBP4 | -0.252307706 | 0.839552409 | 2.151913837 | 4.444169486 | 0.000341696 | 5.293498581 |
| NM_002650 | phosphatidylinositol 4-kinase, catalytic, alpha polypeptide | PIK4CA | -0.337460191 | 0.791433374 | 1.216857331 | 2.324398343 | 1.78E-05 | 2.936947593 |
| NM_002641 | phosphatidylinositol glycan, class A | PIGA | -0.247820163 | 0.842167926 | 2.687752293 | 6.443087973 | 0.000923492 | 7.650597671 |

| | | | | | | | | |
|---|---|---|---|---|---|---|---|---|
| XR_010253 | phosphatidylserine synthase 2 | PSS2 | -0.000610005 | 0.999577266 | 1.146900412 | 2.214376307 | 0.011008504 | 2.215312795 |
| NM_000928 | phospholipase A2, group IB | pancreas | -0.247193323 | 0.842533921 | 1.828970063 | 3.552833459 | 1.83E-05 | 4.216843228 |
| NM_005090 | phospholipase A2, group IVB | PLA2G4B | -0.348235189 | 0.785544446 | 2.924739507 | 7.593365784 | 0.000320143 | 9.666373205 |
| XR_011250 | phospholipase A2, group VI isoform a | PLA2G6 | -0.331419727 | 0.794753997 | 2.039995525 | 4.112442551 | 0.003753017 | 5.174484893 |
| NM_006225 | phospholipase C, delta 1 | PLCD1 | -0.20051695 | 0.870238682 | 1.168027558 | 2.247042728 | 0.006048132 | 2.582099342 |
| NM_002660 | phospholipase C, gamma 1 | PLCG1 | 0.11940916 | 1.086289894 | 1.488222168 | 2.80543049 | 3.56E-05 | 2.582579941 |
| NM_006226 | phospholipase C-like 1 | PLCL1 | 4.160277506 | 17.88003315 | 6.052948402 | 66.39250137 | 0.00879238 | 3.713220262 |
| NM_002766 | phosphoribosyl pyrophosphate synthetase-associated protein 1 | PRPSAP1 | -0.047098012 | 0.96788127 | 2.875375736 | 7.337943192 | 0.006544083 | 7.581449729 |
| NM_000292 | phosphorylase kinase, alpha 2 | PHKA2 | 0.481843635 | 1.396527163 | 1.542254416 | 2.912492664 | 0.005767896 | 2.085525252 |
| NM_001012973 | placenta-specific 9 | PLAC9 | 0.101801548 | 1.073112662 | 2.965107771 | 7.808837395 | 0.000185172 | 7.276810415 |
| NM_003628 | plakophilin 4 | PKP4 | 0.298034427 | 1.229468205 | 2.274307403 | 4.83765338 | 2.62E-05 | 3.934752733 |
| BM423206 | PLATE3_F05 Rhesus cDNA sequence | | -0.145287229 | 0.904199341 | 1.709338189 | 3.270107786 | 0.004039417 | 3.616578378 |
| NM_025208 | platelet derived growth factor D | PDGFD | 0.120831798 | 1.087361609 | 2.465485511 | 5.523127804 | 0.018509315 | 5.079384593 |
| NM_019012 | pleckstrin homology domain containing, family A member 5 | PLEKHA5 | -0.335857663 | 0.792312977 | 1.807496279 | 3.50034295 | 0.023643106 | 4.41787911 |
| NM_014935 | pleckstrin homology domain containing, family A member 6 | PLEKHA6 | 0.340254186 | 1.265979625 | 3.029931026 | 8.167706505 | 0.005990737 | 6.451688751 |
| NM_006832 | pleckstrin homology domain containing, family C | PLEKHC1 | -0.089380639 | 0.939926181 | 1.748567526 | 3.360247564 | 0.01133187 | 3.5750122 |
| XR_013919 | pleckstrin homology domain containing, family H | LOC713488 | -0.380630207 | 0.76810199 | 1.588518384 | 3.007403371 | 0.023516084 | 3.915369847 |
| NM_145307 | pleckstrin homology domain containing, family K member 1 | PLEKHK1 | -0.112171185 | 0.925194641 | 2.973088168 | 7.852152366 | 0.005901437 | 8.487027505 |
| NM_017934 | pleckstrin homology domain interacting protein | PHIP | -0.208227579 | 0.86560001 | 2.354751101 | 5.115059781 | 0.009634681 | 5.909264928 |
| XR_014196 | pleckstrin homology domain interacting protein | PHDIP | -0.152163464 | 0.899899961 | 1.81941848 | 3.529389076 | 0.010679248 | 3.921979363 |
| NM_004227 | pleckstrin homology, Sec7 and coiled-coil domains 3 | PSCD3 | 0.281902609 | 1.215797207 | 1.578079866 | 2.985722048 | 0.000929773 | 2.455773077 |
| NM_145753 | pleckstrin homology-like domain, family B, member 2 | PHLDB2 | -0.599079997 | 0.660174813 | 3.052218866 | 8.294867095 | 0.001534968 | 12.56465247 |
| NM_025179 | plexin A2 | PLXNA2 | -0.068597981 | 0.953564227 | 2.497676842 | 5.6477524 | 0.001654697 | 5.922781329 |
| NM_017514 | plexin A3 | PLXNA3 | 0.249295624 | 1.188626642 | 1.665897248 | 3.173109369 | 0.001452507 | 2.669559353 |
| NM_002673 | plexin B1 | PLXNB1 | -0.259265137 | 0.835513395 | 2.471328105 | 5.545540595 | 0.000255286 | 6.637285085 |
| NM_015100 | pogo transposable element with ZNF domain | POGZ | 0.235305464 | 1.177155952 | 2.472818129 | 5.551271021 | 0.000337096 | 4.715833116 |
| NM_006505 | poliovirus receptor | PVR | 0.17493592 | 1.128914261 | 1.828388024 | 3.551400396 | 0.003646354 | 3.145854844 |
| NM_003631 | poly | PARG | 0.799085261 | 1.739997533 | 1.386622346 | 2.61465817 | 0.004274571 | 1.50267924 |
| NM_031293 | polyamine modulated factor 1 binding protein 1 | PMFBP1 | 0.699792112 | 1.624270724 | 1.941366052 | 3.84069141 | 0.007546871 | 2.364563587 |
| NM_000297 | polycystic kidney disease 2 | PKD2 | -0.68247897 | 0.623093698 | 1.333509822 | 2.520150378 | 0.001199589 | 4.044576901 |
| NM_000937 | polymerase | RNA II | 1.047783237 | 2.067350833 | 2.438754921 | 5.421736206 | 0.004689055 | 2.622552553 |
| NM_006999 | polymerase | POLS | 0.070781943 | 1.050285786 | 1.438731962 | 2.710824963 | 0.004197956 | 2.581035561 |
| NM_153331 | potassium channel tetramerisation domain containing 6 | KCTD6 | -0.260663199 | 0.834704122 | 1.763393596 | 3.394957693 | 0.01145165 | 4.067258809 |
| NM_021161 | potassium channel, subfamily K, member 10 | KCNK10 | -1.898862265 | 0.268154754 | 1.451320375 | 2.734582096 | 0.021398832 | 10.19777593 |
| NM_016611 | potassium channel, subfamily K, member 4 | KCNK4 | 3.023061828 | 8.128909508 | 3.862455982 | 14.54504628 | 0.007313586 | 1.789298585 |
| NM_133497 | potassium channel, subfamily V, member 2 | KCNV2 | 0.639057328 | 1.557311264 | 3.253432183 | 9.536316927 | 0.008005363 | 6.123577956 |
| NM_172108 | potassium voltage-gated channel, KQT-like subfamily, member 2 | KCNQ2 | -0.024322672 | 0.98328213 | 2.520655227 | 5.738426614 | 0.046900121 | 5.835991969 |

| | | | | | | | | |
|---|---|---|---|---|---|---|---|---|
| NM_002232 | potassium voltage-gated channel, shaker-related subfamily, member 3 | KCNA3 | 0.494055647 | 1.40839855 | 1.971442747 | 3.921600971 | 0.005472901 | 2.784439796 |
| NM_002235 | potassium voltage-gated channel, shaker-related subfamily, member 6 | KCNA6 | 0.497130657 | 1.411403661 | 1.647820136 | 3.133598052 | 0.049453004 | 2.220199748 |
| NM_012285 | potassium voltage-gated channel, subfamily H | KCNH4 | 0.991755242 | 1.988602936 | 3.444820698 | 10.88915951 | 0.017560746 | 5.475783684 |
| CO725485 | PPP1R114A | PPP1R114A | 0.039205938 | 1.027548106 | 1.891654962 | 3.710606364 | 0.013092358 | 3.611126664 |
| NM_012231 | PR domain containing 2, with ZNF domain | PRDM2 | 0.400039305 | 1.31954386 | 1.53198097 | 2.891826444 | 0.017480827 | 2.191534917 |
| NM_002585 | pre-B-cell leukemia transcription factor 1 | PBX1 | 0.227693835 | 1.170961656 | 1.172469856 | 2.25397241 | 0.012981157 | 1.924890024 |
| CN804253 | PRO0659 | PRO0659 | 0.111791873 | 1.080569505 | 2.544681809 | 5.834794354 | 0.00030695 | 5.399739977 |
| NM_004199 | procollagen-proline, 2-oxoglutarate 4-dioxygenase | P4HA2 | 0.09966697 | 1.071526084 | 1.678694607 | 3.201381494 | 0.003207083 | 2.987684147 |
| NM_002630 | progastricsin | PGC | 0.067923082 | 1.048206589 | 4.511774317 | 22.81284256 | 0.00029271 | 21.76368933 |
| NM_016335 | proline dehydrogenase | PRODH | 0.345255299 | 1.270375768 | 3.268754207 | 9.638136326 | 0.003403929 | 7.586838925 |
| NM_006813 | proline-rich nuclear receptor coactivator 1 | PNRC1 | -0.817098766 | 0.567582193 | 2.022438512 | 4.062699088 | 0.00154862 | 7.15790442 |
| NM_016223 | protein kinase C and casein kinase substrate in neurons 3 | PACSIN3 | -0.146047673 | 0.903722863 | 2.048728236 | 4.137410877 | 0.00046359 | 4.578185466 |
| NM_002742 | protein kinase C, mu | PRKCM | 0.140141031 | 1.102012838 | 2.433475701 | 5.401932829 | 0.008356088 | 4.901878311 |
| NM_006257 | protein kinase C, theta | PRKCQ | -0.293484698 | 0.815928875 | 1.539310318 | 2.90655522 | 8.18E-05 | 3.562265424 |
| NM_002744 | protein kinase C, zeta | PRKCZ | -0.067217882 | 0.954476854 | 2.088931762 | 4.254329462 | 0.000590754 | 4.457236909 |
| NM_153335 | protein kinase LYK5 | LYK5 | -0.109690444 | 0.926786899 | 1.224640855 | 2.336972658 | 0.005468072 | 2.521585772 |
| NM_006253 | protein kinase, AMP-activated, beta 1 non-catalytic subunit | PRKAB1 | 0.112216919 | 1.080887909 | 1.564210519 | 2.957156341 | 0.014695175 | 2.73585847 |
| NM_005399 | protein kinase, AMP-activated, beta 2 non-catalytic subunit | PRKAB2 | -1.946561482 | 0.259433829 | 1.880201923 | 3.681265806 | 0.022025796 | 14.18961367 |
| NM_032105 | protein phosphatase 1, regulatory | PPP1R12B | -0.947436635 | 0.518553003 | 1.39148759 | 2.623490545 | 0.009691313 | 5.059252439 |
| NM_021003 | protein phosphatase 1A | PPM1A | 0.474611838 | 1.389544308 | 1.265543842 | 2.404178213 | 0.013193076 | 1.730191833 |
| NM_177951 | protein phosphatase 1A | PPM1A | 0.371780418 | 1.293948697 | 1.627646988 | 3.090085995 | 0.014781625 | 2.388105496 |
| NM_005167 | protein phosphatase 1J | PPM1J | 0.561452566 | 1.475754321 | 2.001045688 | 4.002900315 | 0.011093228 | 2.712443567 |
| NM_002719 | protein phosphatase 2, regulatory subunit B | B56 | 0.076907985 | 1.054755043 | 1.441686079 | 2.716381437 | 0.016509902 | 2.575367101 |
| NM_006243 | protein phosphatase 2, regulatory subunit B | B56 | -0.387955404 | 0.764211883 | 1.338916611 | 2.529612866 | 0.003456344 | 3.310093604 |
| XR_013214 | protein phosphatase 2, regulatory subunit B, beta isoform 1 | PPP2R2B | 0.054111972 | 1.038219847 | 2.019386634 | 4.054113934 | 0.012269443 | 3.904870385 |
| NM_005134 | protein phosphatase 4, regulatory subunit 1 | PPP4R1 | 0.187249528 | 1.138590943 | 2.017339153 | 4.048364393 | 0.000432095 | 3.5555916 |
| NM_007039 | protein tyrosine phosphatase, non-receptor type 21 | PTPN21 | 0.155861253 | 1.114086502 | 2.943030023 | 7.690247464 | 0.017896885 | 6.902738208 |
| NM_000533 | proteolipid protein 1 | PLP1 | 0.820904443 | 1.766513094 | 3.521655028 | 11.48480955 | 0.003829465 | 6.501400748 |
| NM_000309 | protoporphyrinogen oxidase | PPOX | 0.201176468 | 1.149635461 | 1.866723657 | 3.647034018 | 0.001944397 | 3.17233953 |
| NM_017922 | PRP39 pre-mRNA processing factor 39 homolog | PRPF39 | -0.034270986 | 0.976525088 | 2.252043026 | 4.763569462 | 0.006808705 | 4.878082008 |
| NM_176800 | PRP4 pre-mRNA processing factor 4 homolog B | PRPF4B | -0.033460913 | 0.977073561 | 1.517511752 | 2.86296841 | 0.015231208 | 2.930146229 |
| NM_025215 | pseudouridylate synthase 1 | PUS1 | 0.164262425 | 1.120593031 | 1.696443784 | 3.241010701 | 1.67E-05 | 2.892228143 |
| CK230551 | PSG5 | PSG5 | -0.893406832 | 0.538341357 | 1.388204251 | 2.617526695 | 0.004558573 | 4.862206221 |
| NM_153831 | PTK2 protein tyrosine kinase 2 | PTK2 | -0.151530679 | 0.900294756 | 1.183678225 | 2.271551833 | 0.017038975 | 2.523120142 |
| NM_002558 | purinergic receptor P2X, ligand-gated ion channel, 1 | P2RX1 | 0.632641409 | 1.550401003 | 1.794983316 | 3.470114619 | 0.029878059 | 2.238204576 |
| NM_170683 | purinergic receptor P2X, ligand-gated ion channel, 2 | P2RX2 | -0.366654983 | 0.775578662 | 2.397601867 | 5.269265476 | 0.017315885 | 6.79397943 |

| | | | | | | | | |
|---|---|---|---|---|---|---|---|---|
| NM_014879 | purinergic receptor P2Y, G-protein coupled, 14 | P2RY14 | 0.613411593 | 1.52987268 | 2.397820676 | 5.270064708 | 0.005067028 | 3.444773396 |
| NM_005859 | purine-rich element binding protein A | PURA | 0.0960143 | 1.068816585 | 1.27057475 | 2.412576602 | 0.001673591 | 2.257240986 |
| XR_013663 | Putative lymphocyte G0 | Putative lymphocyte G0 | 0.061440689 | 1.043507297 | 1.573118232 | 2.975471361 | 0.002685062 | 2.851414043 |
| NM_004884 | putative neuronal cell adhesion molecule | PUNC | 0.909148061 | 1.877936214 | 3.278482611 | 9.703347978 | 0.001722785 | 5.167027456 |
| NM_005049 | PWP2 periodic tryptophan protein homolog | PWP2H | -0.072723832 | 0.950841099 | 2.416340002 | 5.338150579 | 0.005124283 | 5.6141353 |
| NM_000298 | pyruvate kinase, liver and RBC | PKLR | 0.731843786 | 1.660760212 | 2.984062169 | 7.912108274 | 0.039027052 | 4.764148501 |
| NM_130781 | RAB24, member RAS oncogene family | RAB24 | 0.008368594 | 1.005817524 | 1.356182772 | 2.560069138 | 0.000134723 | 2.545262015 |
| NM_012414 | rab3 GTPase-activating protein, non-catalytic subunit | RAB3-GAP150 | -0.128905139 | 0.91452522 | 1.697164726 | 3.242630701 | 0.006444657 | 3.5456985 |
| NM_002866 | RAB3A, member RAS oncogene family | RAB3A | 0.84068646 | 1.790902082 | 2.511256226 | 5.701162905 | 0.0348163 | 3.183402913 |
| NM_182947 | RAC/CDC42 exchange factor | GEFT | 0.181947791 | 1.134414431 | 1.882063303 | 3.686018478 | 0.026487575 | 3.249269735 |
| NM_004840 | Rac/Cdc42 guanine nucleotide exchange factor | GEF 6 | 0.180804742 | 1.13351599 | 1.167604202 | 2.246383435 | 0.004567792 | 1.981783631 |
| NM_021930 | Rad50-interacting protein 1 | RINT-1 | -0.596835272 | 0.661202795 | 1.866684241 | 3.64693438 | 0.041452085 | 5.515606418 |
| NM_031924 | radial spokehead-like 2 | RSHL2 | 0.031576364 | 1.022128347 | 1.215940501 | 2.322921661 | 0.018793296 | 2.27263207 |
| NM_018346 | radical S-adenosyl methionine domain containing 1 | RSAD1 | -0.62006478 | 0.650641712 | 1.968399019 | 3.91333609 | 0.021863742 | 6.014579173 |
| NM_004761 | ral guanine nucleotide dissociation stimulator-like 2 | RGL2 | -0.208702383 | 0.86531518 | 2.204418352 | 4.608886858 | 0.00305853 | 5.326252173 |
| NM_006105 | Rap guanine nucleotide exchange factor | GEF 3 | -0.097127692 | 0.934892447 | 1.913164667 | 3.766343718 | 0.000192031 | 4.028638514 |
| NM_012294 | Rap guanine nucleotide exchange factor | GEF 5 | -0.05376891 | 0.963416201 | 2.809749776 | 7.011629552 | 0.002447524 | 7.277882129 |
| NM_016339 | Rap guanine nucleotide exchange factor | GEF | -0.295648798 | 0.814705867 | 1.534816359 | 2.897515461 | 0.009268532 | 3.556517239 |
| NM_016340 | Rap guanine nucleotide exchange factor | GEF 6 | 0.450151665 | 1.366183871 | 1.754330467 | 3.373697138 | 0.003816741 | 2.469431245 |
| XR_013530 | RAR-related orphan receptor C isoform a | RORC | 0.118368849 | 1.085506864 | 1.521823953 | 2.871538597 | 0.005368386 | 2.645343565 |
| NM_152573 | RAS and EF hand domain containing | RASEF | 0.419276707 | 1.337256955 | 2.793966821 | 6.935341009 | 0.001049278 | 5.186244112 |
| NM_018993 | Ras and Rab interactor 2 | RIN2 | 0.139517709 | 1.101536812 | 1.360161835 | 2.567139748 | 0.011704383 | 2.330507451 |
| NM_025252 | Ras association | RAPH1 | 0.511511096 | 1.425542544 | 3.460616971 | 11.00904157 | 8.09E-05 | 7.722702922 |
| XR_010302 | Ras association domain-containing protein 7 | RASSF7 | 0.208764996 | 1.155698437 | 1.439865849 | 2.712956376 | 0.002127788 | 2.347460453 |
| NM_052949 | RAS guanyl releasing protein 4 | RASGRP4 | 4.128304721 | 17.48813721 | 5.435616225 | 43.27962894 | 0.026904875 | 2.474799255 |
| NM_016084 | RAS, dexamethasone-induced 1 | RASD1 | -0.469486305 | 0.722221711 | 1.357400974 | 2.562231755 | 0.019361202 | 3.547708018 |
| BC057815 | Ras-related associated with diabetes | RARRAD | 0.20534363 | 1.152960933 | 1.182812149 | 2.270188587 | 0.007437801 | 1.969007381 |
| NM_005856 | receptor | calcitonin activity modifying protein 3 | -0.09065172 | 0.939098427 | 2.733046286 | 6.648580197 | 0.000183976 | 7.079747986 |
| NM_016290 | receptor associated protein 80 | RAP80 | 0.441203953 | 1.357736909 | 3.570844388 | 11.88314156 | 0.003677324 | 8.752168024 |
| NM_005012 | receptor tyrosine kinase-like orphan receptor 1 | ROR1 | 0.409906636 | 1.328599831 | 1.70087461 | 3.250979841 | 0.009826858 | 2.446921763 |
| NM_020639 | receptor-interacting serine-threonine kinase 4 | RIPK4 | 0.194925843 | 1.144665321 | 2.748970105 | 6.722370718 | 0.001739949 | 5.872782721 |
| NM_004683 | regucalcin | RGN | 0.229901793 | 1.172755115 | 2.655629109 | 6.30121095 | 7.48E-05 | 5.372998053 |
| NM_005613 | regulator of G-protein signalling 4 | RGS4 | 0.469947066 | 1.385058648 | 2.566720572 | 5.924611574 | 0.002358816 | 4.277516755 |
| XR_014513 | regulator of G-protein signalling 9 | RGS9 | -0.132870675 | 0.912014916 | 1.486423484 | 2.801934991 | 0.021378987 | 3.072246892 |
| NM_014226 | renal tumor antigen | RAGE | 0.461577611 | 1.377046819 | 1.365505662 | 2.576666217 | 0.005156222 | 1.871153676 |
| XR_012426 | resistance to inhibitors of cholinesterase 8B isoform 2 | RIC8B | 0.38488637 | 1.305756939 | 1.751904274 | 3.36802833 | 0.00858502 | 2.57936851 |
| NM_020415 | resistin | RETN | 0.027383174 | 1.019161846 | 3.078741686 | 8.448772118 | 0.022168115 | 8.289921911 |

| | | | | | | | | | |
|---|---|---|---|---|---|---|---|---|---|
| NM_173587 | REST corepressor 2 | RCOR2 | 0.362567948 | 1.285712386 | 1.847705597 | 3.599273159 | 0.015879501 | 2.799438816 |
| NM_018254 | REST corepressor 3 | RCOR3 | -0.270891265 | 0.828807369 | 1.394897884 | 2.629699381 | 0.001597013 | 3.172871622 |
| NM_031429 | retbindin | RTBDN | 1.126347944 | 2.183054192 | 2.674982441 | 6.386309361 | 0.015993387 | 2.925401204 |
| NM_023004 | reticulon 4 receptor | RTN4R | 0.547692998 | 1.461746359 | 1.501467008 | 2.83130468 | 0.027485597 | 1.936932945 |
| NM_003979 | retinoic acid induced 3 | RAI3 | -0.272574204 | 0.827841108 | 1.413262984 | 2.663388682 | 0.008485098 | 3.217270387 |
| NM_021976 | retinoid X receptor, beta | RXRB | 0.226866606 | 1.170290429 | 1.654665158 | 3.14850107 | 0.002215718 | 2.690358728 |
| NM_006744 | retinol binding protein 4, plasma | RBP4 | -0.207817352 | 0.865846176 | 3.355453497 | 10.23510145 | 0.027708897 | 11.82092355 |
| NM_002905 | retinol dehydrogenase 5 | RDH5 | -0.867667785 | 0.548032066 | 1.608129868 | 3.048564074 | 0.011280111 | 5.562747626 |
| NM_016316 | REV1-like | REV1L | 0.3626123 | 1.285751913 | 2.450824429 | 5.467284416 | 0.003633842 | 4.25220788 |
| NM_173670 | RGM domain family, member B | RGMB | 0.323614687 | 1.251462175 | 1.568517216 | 2.96599716 | 0.042371505 | 2.370025414 |
| NM_014433 | rhabdoid tumor deletion region gene 1 | RTDR1 | 4.044849989 | 16.50521457 | 5.242165723 | 37.84853943 | 0.012756526 | 2.293126167 |
| J04697 | Rhesus monkey plasminogen mRNA, complete cds | Rhesus monkey plasminogen mRNA, complete cds | 0.455223056 | 1.370994759 | 2.100759036 | 4.289349986 | 0.003840924 | 3.128640688 |
| NM_001176 | Rho GDP dissociation inhibitor | ARHGDIG | -0.127515298 | 0.915406666 | 1.777159343 | 3.427506359 | 0.021257027 | 3.744244483 |
| NM_020824 | Rho GTPase activating protein 21 | ARHGAP21 | 0.465443861 | 1.380742088 | 3.258200345 | 9.567886963 | 0.009279175 | 6.929525105 |
| NM_001174 | Rho GTPase activating protein 6 | ARHGAP6 | 0.343391895 | 1.268735993 | 2.114141646 | 4.329323617 | 0.000581223 | 3.412312444 |
| NM_003899 | Rho guanine nucleotide exchange factor | GEF 7 | 0.327512407 | 1.254847814 | 1.872458889 | 3.661561149 | 0.008421946 | 2.917932444 |
| NM_014629 | Rho guanine nucleotide exchange factor | GEF 10 | 0.103060954 | 1.07404985 | 1.810601458 | 3.507885015 | 0.001393145 | 3.266035571 |
| NM_014786 | Rho guanine nucleotide exchange factor | GEF 17 | -0.340653129 | 0.789683729 | 2.005747574 | 4.015967445 | 0.000352402 | 5.085539055 |
| NM_153213 | Rho guanine nucleotide exchange factor | GEF 19 | 0.211308354 | 1.157737639 | 2.161406491 | 4.473507673 | 0.001922158 | 3.86400815 |
| NM_022450 | rhomboid family 1 | RHBDF1 | 0.133381406 | 1.096861528 | 3.006065599 | 8.03370563 | 0.002714535 | 7.324266031 |
| NM_014836 | Rho-related BTB domain containing 1 | RHOBTB1 | 0.368574244 | 1.291076283 | 1.570799014 | 2.970691956 | 0.032097048 | 2.300942241 |
| NM_194430 | ribonuclease, RNase A family, 4 | RNASE4 | -0.4248451 | 0.744918708 | 1.307111815 | 2.474456731 | 0.009798971 | 3.321780893 |
| NM_007104 | ribosomal protein L10a | RPL10A | 0.360222454 | 1.283623808 | 1.865182389 | 3.643139881 | 0.002034578 | 2.838167895 |
| NM_000977 | ribosomal protein L13 | RPL13 | -0.143919493 | 0.905056967 | 2.337084571 | 5.052805235 | 0.000905337 | 5.58285878 |
| NM_004586 | ribosomal protein S6 kinase, 90kDa, polypeptide 3 | RPS6KA3 | 0.300288758 | 1.231390854 | 1.419275775 | 2.674512183 | 0.012418542 | 2.171944167 |
| NM_024557 | RIC3 protein | RIC3 | 4.084217904 | 16.96180624 | 2.313923347 | 4.972334481 | 0.008096962 | 0.293148879 |
| NM_080669 | RIKEN cDNA 1110002C08 gene | MGC9564 | 0.296197672 | 1.227903915 | 1.638668028 | 3.113782184 | 0.012496852 | 2.535851662 |
| XM_376148 | RIKEN cDNA 5830415L20 | LOC401015 | 0.253970995 | 1.192484898 | 2.037217771 | 4.104532102 | 0.011316635 | 3.441999231 |
| NM_183353 | ring finger protein 12 | RNF12 | 0.238745997 | 1.179966579 | 1.95365672 | 3.873550962 | 0.028090679 | 3.282763284 |
| XM_027330 | RNA binding motif protein 25 | RBM25 | 0.261175476 | 1.198454781 | 2.155835343 | 4.456265974 | 0.001279545 | 3.718343024 |
| NM_005778 | RNA binding motif protein 5 | RBM5 | -0.21803068 | 0.859738203 | 2.089962421 | 4.257369834 | 0.007279725 | 4.951937484 |
| NM_005777 | RNA binding motif protein 6 | RBM6 | 0.307404802 | 1.237479651 | 1.481296154 | 2.791994605 | 0.001588986 | 2.256194357 |
| NM_001008710 | RNA binding protein with multiple splicing | RBPMS | 0.278501891 | 1.212934707 | 2.671076688 | 6.369043335 | 0.002832578 | 5.250936673 |
| NM_184244 | RNA-binding region | RNP1 | 0.072554971 | 1.051577348 | 2.640968726 | 6.237503527 | 0.001222024 | 5.931568932 |
| NM_173640 | roof plate-specific spondin | RSPONDIN | 0.031109189 | 1.021797414 | 1.589818068 | 3.010113879 | 0.004137095 | 2.945900858 |
| NM_019055 | roundabout homolog 4, magic roundabout | ROBO4 | 0.064655825 | 1.045835411 | 1.339792689 | 2.531149443 | 0.000625024 | 2.420217767 |
| XR_011047 | RP11-506B151 protein isoform 1 | LOC702661 | -0.014864357 | 0.989749709 | 1.704095646 | 3.258246257 | 0.006012852 | 3.291990114 |
| CN646799 | RPL19 | RPL19 | 0.000696033 | 1.00048257 | 1.208011622 | 2.310190179 | 0.007005139 | 2.309075888 |
| XM_496807 | Rps15a protein | RPS15A | 0.029837203 | 1.020896919 | 1.332462383 | 2.518321338 | 0.003941862 | 2.466773375 |

| | | | | | | | | | |
|---|---|---|---|---|---|---|---|---|---|
| NM_025158 | RUN and FYVE domain containing 1 | RUFY1 | 0.093952374 | 1.067290104 | 1.325301914 | 2.505853224 | 0.036047941 | 2.347865135 |
| NM_014328 | RUN and SH3 domain containing 1 | RUSC1 | 0.409984058 | 1.328671132 | 1.482863014 | 2.79502854 | 0.029852784 | 2.103627055 |
| NM_015705 | RUN and TBC1 domain containing 3 | RUTBC3 | -0.582780921 | 0.667675533 | 1.428127505 | 2.690972234 | 3.94E-05 | 4.03035921 |
| NM_020672 | S100 calcium binding protein A14 | S100A14 | 0.190484854 | 1.141147163 | 1.559313711 | 2.947136151 | 0.007085782 | 2.582608314 |
| NM_014624 | S100 calcium binding protein A6 | calcyclin | 0.054958431 | 1.03882917 | 1.312536486 | 2.483778439 | 0.004033075 | 2.390940215 |
| NM_002965 | S100 calcium binding protein A9 | calgranulin B | -0.016872741 | 0.988372831 | 1.197247249 | 2.293017315 | 0.002915688 | 2.319992257 |
| NM_005407 | sal-like 2 | SALL2 | -0.091291351 | 0.938682162 | 1.387416598 | 2.616098023 | 0.023863116 | 2.786990239 |
| NM_003919 | sarcoglycan, epsilon | SGCE | 0.26781729 | 1.203984893 | 1.764567114 | 3.397720345 | 0.017664602 | 2.822062274 |
| NM_007159 | sarcolemma associated protein | SLMAP | 0.312620955 | 1.241961933 | 1.508589166 | 2.845316551 | 0.001167241 | 2.290985316 |
| NM_178123 | SEC14 and spectrin domains 1 | SESTD1 | 0.283309587 | 1.216983482 | 1.343892245 | 2.538352176 | 0.016991488 | 2.08577373 |
| NM_004206 | SEC22 vesicle trafficking protein-like 3 | SEC22L3 | -0.644113563 | 0.639885836 | 1.784863972 | 3.445859722 | 0.015046169 | 5.385116419 |
| NM_015490 | SEC31-like 2 | SEC31L2 | 0.091497326 | 1.065475431 | 1.984543287 | 3.957373645 | 0.005811651 | 3.714185733 |
| NM_138355 | secernin 2 | SCRN2 | -0.260281802 | 0.834924818 | 2.361241834 | 5.138124451 | 0.002450435 | 6.153996552 |
| NM_003357 | secretoglobin, family 1A, member 1 | SCGB1A1 | 0.051170626 | 1.036105296 | 2.603216742 | 6.076399573 | 0.004206781 | 5.864654487 |
| NM_015662 | selective LIM binding factor, rat homolog | SLB | 0.241456488 | 1.182185547 | 2.308038386 | 4.952092923 | 0.003164287 | 4.188930355 |
| NM_005410 | selenoprotein P, plasma, 1 | SEPP1 | 0.918536363 | 1.890196686 | 1.747404802 | 3.3575405 | 0.003815703 | 1.776291602 |
| CN643169 | SELP | SELP | 0.564984986 | 1.479372114 | 2.129764865 | 4.376461457 | 0.007656854 | 2.958323613 |
| NM_004636 | sema domain, immunoglobulin domain | semaphorin 3B | -0.010577273 | 0.992695203 | 4.524043012 | 23.00767046 | 0.002154049 | 23.17697355 |
| NM_020796 | sema domain, transmembrane domain | TM | -0.073352749 | 0.950426687 | 1.680326855 | 3.20500555 | 0.002478913 | 3.37217546 |
| NM_153618 | sema domain, transmembrane domain | TM | -1.010452236 | 0.496390622 | 1.197650232 | 2.293657904 | 0.041087594 | 4.620671307 |
| NM_145733 | septin 3 | 3-Sep | 0.488309761 | 1.402800414 | 1.184136191 | 2.272273022 | 0.013894015 | 1.619812055 |
| NM_006843 | serine dehydratase | SDS | 3.031562184 | 8.176946403 | 3.999262494 | 15.99182289 | 0.045700524 | 1.955720645 |
| NM_016333 | serine/arginine repetitive matrix 2 | SRRM2 | 0.028152052 | 1.019705149 | 1.941123939 | 3.84004692 | 0.001292337 | 3.765840472 |
| NM_052902 | serine/threonine kinase 11 interacting protein | STK11IP | -0.084267441 | 0.943263377 | 1.400480443 | 2.639894807 | 0.008491674 | 2.798682607 |
| NM_014370 | serine/threonine kinase 23 | STK23 | 0.127410354 | 1.0923312 | 2.335707553 | 5.047984754 | 0.011249872 | 4.621295038 |
| NM_015690 | serine/threonine kinase 36 | STK36 | -0.334379304 | 0.793125293 | 2.102656074 | 4.294993876 | 0.006562488 | 5.415277906 |
| NM_019605 | SERTA domain containing 4 | SERTAD4 | 0.571590144 | 1.48616072 | 2.029104761 | 4.081515006 | 0.010719106 | 2.746348326 |
| NM_004657 | serum deprivation response | SDPR | -1.502266383 | 0.352998416 | 3.082116273 | 8.468557647 | 0.022251791 | 23.99035594 |
| NM_005490 | SH2 domain containing 3A | SH2D3A | -0.256457529 | 0.837140959 | 1.841006771 | 3.582599495 | 0.000531478 | 4.279565414 |
| NM_022071 | SH2 domain containing 4A | SH2D4A | -0.840701086 | 0.558372159 | 2.323793134 | 5.006467912 | 0.044347299 | 8.966184705 |
| NM_012309 | SH3 and multiple ankyrin repeat domains 2 | SHANK2 | -0.168665902 | 0.889664998 | 4.315134409 | 19.90604092 | 0.00031394 | 22.37476012 |
| NM_020870 | SH3 multiple domains 2 | SH3MD2 | -0.394778751 | 0.760606013 | 1.807770031 | 3.501007204 | 0.017571769 | 4.602918126 |
| NM_005413 | sine oculis homeobox homolog 3 | SIX3 | 0.821052687 | 1.766694621 | 3.244080714 | 9.474702958 | 0.000653174 | 5.362954551 |
| NM_021805 | single Ig IL-1R-related molecule | SIGIRR | -0.064032704 | 0.956586473 | 1.556122262 | 2.940623868 | 0.000531247 | 3.074080547 |
| NM_012240 | sirtuin | SIRT4 | -0.030823155 | 0.978861632 | 1.802525431 | 3.48830317 | 0.002606192 | 3.563632546 |
| NM_152999 | six transmembrane epithelial antigen of the prostate 2 | STEAP2 | -0.382882785 | 0.766903636 | 1.29782046 | 2.458571752 | 0.019188332 | 3.205841825 |
| NM_012245 | SKI interacting protein | SKIIP | -0.228410481 | 0.853574818 | 1.236155532 | 2.355699525 | 0.001001266 | 2.759804384 |
| CO725992 | SLA | SLA | -0.002577855 | 0.998214762 | 1.429721351 | 2.693946782 | 0.019208272 | 2.698764719 |
| NM_015065 | SLAC2-B | SLAC2-B | -0.22294898 | 0.856812255 | 1.278462097 | 2.425802501 | 0.003664065 | 2.831194917 |
| NM_033438 | SLAM family member 9 | SLAMF9 | 0.209388718 | 1.156198189 | 2.406318673 | 5.301198905 | 0.001777468 | 4.585026127 |
| NM_004787 | slit homolog 2 | SLIT2 | 0.152209362 | 1.111269983 | 1.625304745 | 3.085073252 | 0.037320175 | 2.776168977 |
| NM_001001419 | SMAD, mothers against DPP homolog 5 | SMAD5 | 0.182904776 | 1.135167174 | 1.6034026 | 3.038591213 | 0.013644654 | 2.676778613 |

| | | | | | | | | |
|---|---|---|---|---|---|---|---|---|
| NM_015001 | SMART/HDAC1 associated repressor protein | SHARP | 0.167378279 | 1.123015842 | 1.288633348 | 2.442965261 | 0.00450908 | 2.175361353 |
| NM_000336 | sodium channel, nonvoltage-gated 1, beta | SCNN1B | 1.63326855 | 3.10215022 | 1.253269801 | 2.383810908 | 0.001909007 | 0.768438257 |
| NM_000617 | solute carrier family 11 | SLC11A2 | 0.097718541 | 1.070079913 | 1.292367929 | 2.449297346 | 0.00487907 | 2.288891994 |
| NM_012450 | solute carrier family 13 | SLC13A4 | 0.45277484 | 1.36867019 | 1.209116069 | 2.311959409 | 0.009643149 | 1.68920126 |
| NM_004695 | solute carrier family 16 | SLC16A5 | -0.457877333 | 0.728056676 | 1.370433502 | 2.585482432 | 0.011345834 | 3.55121039 |
| NM_001042 | solute carrier family 2 | SLC2A4 | 0.435008583 | 1.351918876 | 2.573795307 | 5.953736232 | 0.047084901 | 4.403915307 |
| NM_003060 | solute carrier family 22 | SLC22A5 | -0.239954822 | 0.846771829 | 2.068167076 | 4.193535521 | 0.011418521 | 4.952379587 |
| NM_003951 | solute carrier family 25 | SLC25A14 | 0.290345033 | 1.222932718 | 1.33708655 | 2.526406083 | 0.002822123 | 2.065858608 |
| NM_004277 | solute carrier family 25, member 27 | SLC25A27 | -0.488156746 | 0.712935396 | 2.475534499 | 5.561733046 | 0.006649102 | 7.801173959 |
| NM_052934 | solute carrier family 26, member 9 | SLC26A9 | 0.145531451 | 1.10613805 | 2.77467958 | 6.843240165 | 0.001781499 | 6.186605881 |
| NM_024330 | solute carrier family 27 | SLC27A3 | -0.291438093 | 0.817087172 | 2.189735932 | 4.562219728 | 0.012451405 | 5.583516526 |
| NM_022127 | solute carrier family 28 | SLC28A3 | -0.374780499 | 0.771222742 | 1.248124848 | 2.375324879 | 0.014478945 | 3.079946622 |
| NM_018964 | solute carrier family 37 | SLC37A1 | 0.255221164 | 1.193518695 | 2.138287489 | 4.402391624 | 0.00172925 | 3.688582041 |
| NM_173854 | solute carrier family 41, member 1 | SLC41A1 | 0.230245394 | 1.173034458 | 2.749652704 | 6.725552104 | 0.023990655 | 5.733465081 |
| NM_014270 | solute carrier family 7 | cationic amino acid transporter | 0.089915696 | 1.064307988 | 2.132950579 | 4.386136101 | 0.041420447 | 4.121115458 |
| NM_004594 | solute carrier family 9 | SLC9A5 | -0.158729455 | 0.895813644 | 3.365827918 | 10.30896733 | 0.013461378 | 11.50793739 |
| NM_005630 | solute carrier organic anion transporter family, member 2A1 | SLCO2A1 | 0.236246025 | 1.177923646 | 2.069874198 | 4.198500613 | 0.004273859 | 3.564323229 |
| NM_003103 | SON DNA binding protein | SON | -0.101288469 | 0.932200073 | 2.142764095 | 4.416073228 | 0.019228171 | 4.737259045 |
| NM_032195 | SON DNA binding protein | SON | -0.543640949 | 0.686037361 | 1.763351302 | 3.394858168 | 0.001479538 | 4.948503334 |
| NM_058183 | SON DNA binding protein | SON | 0.192425998 | 1.142683608 | 2.375228863 | 5.188181184 | 0.002016861 | 4.540347956 |
| NM_005633 | son of sevenless homolog 1 | SOS1 | 0.701443103 | 1.626130569 | 3.251816176 | 9.525640986 | 0.007623343 | 5.857857399 |
| NM_000193 | sonic hedgehog homolog | SHH | 0.517565547 | 1.431537583 | 1.418237262 | 2.672587648 | 0.027118262 | 1.866935021 |
| NM_031953 | sorting nexin 25 | SNX25 | -1.069624441 | 0.476443009 | 2.389329664 | 5.239138728 | 0.015757141 | 10.99635975 |
| NM_004510 | SP110 nuclear body protein | SP110 | -0.041542574 | 0.971615511 | 1.280467151 | 2.42917622 | 0.00176217 | 2.500141458 |
| NM_004684 | SPARC-like 1 | mast9 | -0.376733276 | 0.770179551 | 2.458692821 | 5.497184195 | 0.000530157 | 7.1375359 |
| NM_002971 | special AT-rich sequence binding protein 1 | SATB1 | 0.095019366 | 1.068079746 | 3.1745823 | 9.029100684 | 0.021504561 | 8.453582907 |
| XR_012522 | spectrin repeat containing, nuclear envelope 2 isoform e | SYNE2 | 0.331354932 | 1.258194475 | 1.797618685 | 3.476459264 | 0.009604982 | 2.76305399 |
| XR_012522 | spectrin repeat containing, nuclear envelope 2 isoform e | | 0.440956687 | 1.357504224 | 1.852763594 | 3.611914128 | 0.010773925 | 2.660701944 |
| NM_032637 | S-phase kinase-associated protein 2 | SKP2 | 0.597614461 | 1.513212355 | 1.382414303 | 2.607042863 | 0.011708903 | 1.722853276 |
| NM_019003 | spindlin family, member 2 | SPIN2 | -0.652838484 | 0.636027705 | 1.675326127 | 3.193915461 | 0.01550404 | 5.021660905 |
| NM_001006682 | spindlin-like protein 2 | SPIN-2 | 0.017049955 | 1.011888238 | 2.372055306 | 5.176781066 | 0.001130345 | 5.1159613 |
| NM_201997 | splicing factor 1 | SF1 | 0.030535598 | 1.021391245 | 1.617039815 | 3.067449973 | 0.000952532 | 3.003207623 |
| NM_012433 | splicing factor 3b, subunit 1, 155kDa | SF3B1 | 0.077974756 | 1.055535248 | 2.090154502 | 4.257936699 | 0.009516112 | 4.033912375 |
| NM_004768 | splicing factor, arginine/serine-rich 11 | SFRS11 | -0.857264642 | 0.551998158 | 2.185406159 | 4.548548257 | 0.000481124 | 8.240151151 |
| NM_006925 | splicing factor, arginine/serine-rich 5 | SFRS5 | 0.258565512 | 1.19628863 | 3.471339113 | 11.09116581 | 0.000200271 | 9.271312568 |
| NM_006275 | splicing factor, arginine/serine-rich 6 | SFRS6 | -0.669790516 | 0.628597955 | 1.75853999 | 3.383555352 | 0.00087008 | 5.382701813 |
| NM_022039 | split hand/foot malformation | SHFM3 | 0.340293839 | 1.266014422 | 1.758342064 | 3.383091187 | 0.001021253 | 2.672237479 |
| NM_020172 | SPPL2b | SPPL2B | -0.758166188 | 0.591247389 | 1.297601685 | 2.458198955 | 0.006183389 | 4.157648728 |
| NM_005841 | sprouty homolog 1, antagonist of FGF signaling | SPRY1 | -0.602327082 | 0.658690623 | 1.892175086 | 3.711944361 | 0.00321241 | 5.635338096 |
| NM_080861 | SPRY domain-containing SOCS box protein SSB-3 | SSB3 | 0.014102699 | 1.00982318 | 1.707762309 | 3.266537741 | 5.99E-05 | 3.234762091 |

| | | | | | | | | | |
|---|---|---|---|---|---|---|---|---|---|
| NM_003129 | squalene epoxidase | SQLE | -0.299734451 | 0.812401917 | 2.481131716 | 5.583352787 | 0.001679433 | 6.872648464 |
| NM_005063 | stearoyl-CoA desaturase | SCD | -0.31016988 | 0.806546781 | 1.340840292 | 2.532988086 | 0.014309468 | 3.140534617 |
| CN647246 | STMN4 | STMN4 | 0.279278188 | 1.213587548 | 1.461445934 | 2.753842278 | 0.001317094 | 2.269174797 |
| NM_020225 | storkhead box 2 | STOX2 | -0.112653697 | 0.92488526 | 1.448915557 | 2.730027638 | 0.007552539 | 2.951747375 |
| NM_014351 | sulfotransferase family 4A, member 1 | SULT4A1 | -0.448169776 | 0.732972118 | 1.361063316 | 2.568744351 | 0.030123493 | 3.504559434 |
| NM_177529 | sulfotransferase family, cytosolic, 1A, phenol-preferring, member 1 | SULT1A1 | 0.200590628 | 1.14916872 | 2.964648693 | 7.806352951 | 0.0003511 | 6.793043367 |
| NM_177528 | sulfotransferase family, cytosolic, 1A, phenol-preferring, member 2 | SULT1A2 | 0.265007226 | 1.201642068 | 2.968545891 | 7.827469009 | 0.000111885 | 6.51397718 |
| NM_003166 | sulfotransferase family, cytosolic, 1A, phenol-preferring, member 3 | SULT1A3 | -0.220397937 | 0.858328652 | 3.744230826 | 13.40064768 | 0.000228719 | 15.61249021 |
| NM_021627 | SUMO1/sentrin/SMT3 specific protease 2 | SENP2 | -0.156308158 | 0.897318364 | 1.835916665 | 3.569981666 | 0.001266079 | 3.978500618 |
| NM_021738 | supervillin | SVIL | 0.501291554 | 1.415480185 | 2.448540789 | 5.458637106 | 0.007277232 | 3.856385391 |
| NM_003877 | suppressor of cytokine signaling 2 | SOCS2 | 0.503955089 | 1.418095889 | 1.503475029 | 2.835248187 | 0.029157837 | 1.999334607 |
| NM_003955 | suppressor of cytokine signaling 3 | SOCS3 | 1.428927361 | 2.692464571 | 2.291106929 | 4.894314906 | 0.035358123 | 1.817782473 |
| NM_005411 | surfactant, pulmonary-associated protein A1 | SFTPA1 | -0.4250919 | 0.744791287 | 2.344324455 | 5.078225485 | 0.002903999 | 6.818320211 |
| NM_006926 | surfactant, pulmonary-associated protein A2 | SFTPA2 | -1.068909715 | 0.476679103 | 2.577625178 | 5.969562397 | 5.66E-05 | 12.52323074 |
| NM_000542 | surfactant, pulmonary-associated protein B | SFTPB | 0.238856646 | 1.18005708 | 1.742114877 | 3.345251961 | 0.027637889 | 2.83482216 |
| NM_003019 | surfactant, pulmonary-associated protein D | SFTPD | -0.165724871 | 0.891480489 | 2.182467865 | 4.53929379 | 0.000220679 | 5.091859941 |
| NM_017503 | surfeit 2 | SURF2 | 0.029552254 | 1.0206953 | 1.26285686 | 2.399704662 | 0.008454465 | 2.351048998 |
| NM_019601 | sushi domain containing 2 | SUSD2 | -0.241324333 | 0.845968392 | 2.420070876 | 5.351973141 | 7.87E-05 | 6.326445753 |
| NM_015055 | SWAP-70 protein | SWAP70 | -0.354104105 | 0.782355325 | 2.499949782 | 5.656657345 | 0.012401181 | 7.230291869 |
| NM_003071 | SWI/SNF related, matrix associated, actin dependent regulator of chromatin, subfamily a, member 3 | SMARCA3 | -0.182442111 | 0.881210071 | 1.341310356 | 2.533813529 | 0.014952664 | 2.875379677 |
| NM_003072 | SWI/SNF related, matrix associated, actin dependent regulator of chromatin, subfamily a, member 4 | SMARCA4 | 0.319558648 | 1.247948716 | 1.805859011 | 3.496372777 | 0.003351408 | 2.801695881 |
| NM_139067 | SWI/SNF related, matrix associated, actin dependent regulator of chromatin, subfamily c, member 2 | SMARCC2 | -0.016747619 | 0.988458555 | 1.768952065 | 3.408063143 | 0.000692429 | 3.447856388 |
| NM_004209 | synaptogyrin 3 | SYNGR3 | -0.251852406 | 0.839817405 | 1.624248491 | 3.082815375 | 0.014113874 | 3.670816247 |
| NM_024875 | synaptopodin 2-like | SYNPO2L | 0.052032363 | 1.036724357 | 2.067113373 | 4.190473801 | 0.011827381 | 4.042032747 |
| NM_003180 | synaptotagmin V | SYT5 | 0.473613189 | 1.388582784 | 1.589979552 | 3.010450825 | 0.033413842 | 2.168002412 |
| NM_181519 | synaptotagmin XV | SYT15 | 0.313396747 | 1.242629963 | 1.460675675 | 2.752372387 | 0.002747467 | 2.214957364 |
| NM_206927 | synaptotagmin-like 2 | SYTL2 | -0.141283784 | 0.906711957 | 3.429061586 | 10.77086034 | 0.006100778 | 11.87903199 |
| NM_080737 | synaptotagmin-like 4 | SYTL4 | 0.613350494 | 1.52980789 | 1.804523969 | 3.493138798 | 0.010060485 | 2.28338396 |
| NM_198935 | synovial sarcoma translocation gene on chromosome 18-like 1 | SS18L1 | 0.401218287 | 1.320622643 | 1.5395642 | 2.907066753 | 0.001574117 | 2.201284953 |
| NM_001001433 | syntaxin 16 | STX16 | -0.279616244 | 0.823810121 | 1.255688779 | 2.387811215 | 0.006747329 | 2.898497062 |
| NM_004603 | syntaxin 1A | STX1A | -0.047518566 | 0.967599168 | 1.577541699 | 2.984608495 | 0.002175101 | 3.084550497 |
| NM_052874 | syntaxin 1B2 | STX1B2 | 0.42835596 | 1.345699196 | 1.362599974 | 2.571481855 | 0.009990231 | 1.910889048 |
| NM_004177 | syntaxin 3A | STX3A | -0.124056934 | 0.91760367 | 1.709474195 | 3.27041608 | 0.009601745 | 3.564083478 |
| NM_005486 | target of myb1-like 1 | TOM1L1 | 0.061242713 | 1.04336411 | 1.376811086 | 2.596937126 | 0.004464587 | 2.489003696 |
| NM_019020 | TBC1 domain family, member 16 | TBC1D16 | -0.017695003 | 0.98780967 | 2.115524393 | 4.333475033 | 0.001096836 | 4.386953443 |

| | | | | | | | | |
|---|---|---|---|---|---|---|---|---|
| XR_010229 | TBC1 domain family, member 4 | TBC1D4 | -0.258765971 | 0.835802529 | 1.909626133 | 3.757117233 | 0.001366629 | 4.495221183 |
| NM_005994 | T-box 2 | TBX2 | -0.298100573 | 0.813322498 | 3.09508604 | 8.545032859 | 0.00011467 | 10.50632791 |
| NM_016569 | T-box 3 | TBX3 | -0.068366404 | 0.953717302 | 1.952334869 | 3.870003494 | 0.030321639 | 4.057809883 |
| NM_018488 | T-box 4 | TBX4 | 0.050595831 | 1.035692575 | 3.182582547 | 9.079309283 | 0.017407503 | 8.766413415 |
| NM_000192 | T-box 5 | TBX5 | 0.525345833 | 1.439278553 | 1.95068507 | 3.865580469 | 0.008095644 | 2.685776468 |
| NM_005521 | T-cell leukemia, homeobox 1 | TLX1 | 0.37665717 | 1.298330042 | 2.245579943 | 4.742277029 | 0.016154411 | 3.652597472 |
| CN645262 | TCERG1 | TCERG1 | -0.633110794 | 0.644784606 | 1.480243387 | 2.789957968 | 0.001485152 | 4.326961193 |
| NM_021961 | TEA domain family member 1 | TEAD1 | -0.026533789 | 0.981776276 | 1.602207286 | 3.036074696 | 0.007512132 | 3.092430292 |
| XR_014213 | tenascin XB isoform 1 | TNXB | -0.366988611 | 0.775399328 | 5.29806036 | 39.34368978 | 0.000519717 | 50.73990699 |
| NM_022648 | tensin | TNS | -0.409264521 | 0.753007155 | 1.696544055 | 3.241235968 | 0.000579334 | 4.304389336 |
| NM_015319 | tensin like C1 domain containing phosphatase | TENC1 | -0.052057959 | 0.964559433 | 3.169020565 | 8.994359598 | 0.000570886 | 9.324837112 |
| NM_182911 | testis specific, 10 | TSGA10 | -0.757742577 | 0.591421019 | 4.228273966 | 18.74292182 | 0.011985046 | 31.69133531 |
| NM_001120 | tetracycline transporter-like protein | TETRAN | 0.087173518 | 1.062286944 | 1.621122697 | 3.076143268 | 0.002498875 | 2.895774334 |
| NM_175605 | tetratricopeptide repeat domain 10 | TTC10 | -0.103472425 | 0.930789973 | 1.625064848 | 3.084560297 | 0.044881956 | 3.313916551 |
| XR_011624 | tetratricopeptide repeat domain 16 | TTC16 | -0.154959112 | 0.898157828 | 1.146083171 | 2.213122289 | 0.003399013 | 2.464068363 |
| NM_018259 | tetratricopeptide repeat domain 17 | TTC17 | 0.055233006 | 1.0390269 | 1.25709798 | 2.39014473 | 0.001389377 | 2.300368479 |
| NM_145170 | tetratricopeptide repeat domain 18 | TTC18 | 0.326795588 | 1.254224484 | 1.549943209 | 2.928056129 | 0.029770943 | 2.334555071 |
| NM_003597 | TGFB inducible early growth response 2 | TIEG2 | 0.125671529 | 1.091015448 | 2.46641761 | 5.526697348 | 0.013230895 | 5.065645366 |
| NM_198976 | TH1-like | TH1L | -0.136883462 | 0.909481715 | 1.229348883 | 2.34461149 | 0.00099215 | 2.577964407 |
| NM_006440 | thioredoxin reductase 2 | TXNRD2 | -0.123781206 | 0.917779059 | 1.053766784 | 2.075942931 | 0.005094364 | 2.261920133 |
| NM_004783 | thousand and one amino acid protein kinase | TAO1 | 0.373018535 | 1.295059637 | 1.186160325 | 2.275463311 | 0.013108026 | 1.757033611 |
| NM_007112 | thrombospondin 3 | THBS3 | 0.288860482 | 1.221674952 | 2.707664204 | 6.532631242 | 0.002299195 | 5.347274438 |
| NM_025008 | thrombospondin repeat containing 1 | TSRC1 | 1.432156841 | 2.698498416 | 2.550462085 | 5.858218829 | 0.015060042 | 2.170918017 |
| NM_033051 | thymic stromal co-transporter | TSCOT | -0.498270018 | 0.707955204 | 1.892947696 | 3.71393276 | 0.008865204 | 5.245999661 |
| NM_183049 | thymosin-like 3 | TMSL3 | 0.621245989 | 1.538203083 | 1.233848193 | 2.351935006 | 0.042275343 | 1.529014622 |
| NM_145056 | thymus expressed gene 3-like | MGC15476 | 0.055227433 | 1.039022887 | 1.638479871 | 3.113376109 | 0.004723795 | 2.99644613 |
| NM_004240 | thyroid hormone receptor interactor 10 | TRIP10 | -0.094092244 | 0.936861543 | 2.806131639 | 6.994067109 | 0.001083811 | 7.465422358 |
| NM_003317 | thyroid transcription factor 1 | TITF1 | 0.14298034 | 1.1041838 | 3.185355054 | 9.096774271 | 0.002668909 | 8.238460183 |
| NM_003216 | thyrotrophic embryonic factor | TEF | 0.50666345 | 1.420760571 | 2.570315146 | 5.939391555 | 0.005459808 | 4.180431014 |
| NM_007117 | thyrotropin-releasing hormone | TRH | 0.679775154 | 1.601890079 | 1.270067712 | 2.411728845 | 0.031787264 | 1.50555202 |
| NM_033208 | tigger transposable element derived 7 | TIGD7 | 0.028489345 | 1.019943577 | 1.645298343 | 3.128125388 | 0.01535842 | 3.06695925 |
| NM_003257 | tight junction protein 1 | TJP1 | 0.199506142 | 1.148305204 | 1.668668514 | 3.179210437 | 0.021502137 | 2.768611016 |
| NM_004817 | tight junction protein 2 | TJP2 | 0.201236664 | 1.14968343 | 2.743389947 | 6.696419675 | 0.004904195 | 5.82457701 |
| NM_000362 | tissue inhibitor of metalloproteinase 3 | TIMP3 | 0.142126344 | 1.103530378 | 3.296916063 | 9.828124022 | 0.000595181 | 8.906074738 |
| NM_003319 | titin | TTN | -1.128324976 | 0.45744653 | 4.256663041 | 19.11539412 | 0.00332895 | 41.78716608 |
| NM_003673 | titin-cap | TCAP | -0.070337575 | 0.952415117 | 1.62880822 | 3.092574221 | 0.006076035 | 3.24708645 |
| NM_016292 | TNF receptor-associated protein 1 | TRAP1 | 0.280409359 | 1.214539457 | 1.374667509 | 2.593081426 | 0.007316409 | 2.135032675 |
| NM_000355 | transcobalamin II; macrocytic anemia | TCN2 | 0.119306473 | 1.086212577 | 2.030250366 | 4.084757311 | 0.002560792 | 3.760550557 |
| NM_018719 | transcription factor RAM2 | RAM2 | -0.170120758 | 0.888768285 | 1.431884666 | 2.697989374 | 0.012669039 | 3.035649921 |
| NM_025198 | transcription termination factor-like protein | LOC80298 | -0.189242263 | 0.877066256 | 1.692860808 | 3.232971541 | 0.001522264 | 3.686120084 |
| NM_005647 | transducin | TBL1X | -0.46234061 | 0.725807762 | 1.477704443 | 2.785052345 | 0.005825036 | 3.837176303 |
| NM_003260 | transducin-like enhancer of split 2 | TLE2 | 0.083849091 | 1.059841916 | 1.792265956 | 3.463584703 | 0.001539476 | 3.268020118 |
| NM_013293 | transformer-2 alpha | TRA2A | -0.159359246 | 0.895422673 | 1.469899772 | 2.770026489 | 0.011923027 | 3.093540707 |
| NM_183422 | transforming growth factor beta 1 induced transcript 4 | TGFB1I4 | 0.807028531 | 1.74960413 | 1.995538107 | 3.987648116 | 0.029062988 | 2.279171641 |
| NM_003243 | transforming growth factor, beta receptor III | TGFBR3 | 0.010632568 | 1.007397159 | 2.367365735 | 5.159980933 | 0.014145693 | 5.122092004 |

| | | | | | | | | |
|---|---|---|---|---|---|---|---|---|
| NM_006997 | transforming, acidic coiled-coil containing protein 2 | TACC2 | -0.159037268 | 0.895622534 | 2.685358534 | 6.432406289 | 0.003216989 | 7.182050523 |
| NM_017636 | transient receptor potential cation channel, subfamily M, member 4 | TRPM4 | 0.191740429 | 1.142140733 | 1.279800754 | 2.428054415 | 0.001378798 | 2.125880239 |
| NM_014555 | transient receptor potential cation channel, subfamily M, member 5 | TRPM5 | 0.392490741 | 1.312657687 | 1.85430969 | 3.615786992 | 0.001096263 | 2.754554388 |
| NM_144686 | transmembrane channel-like 4 | TMC4 | 0.152550078 | 1.111532458 | 1.319036382 | 2.494994063 | 0.000625708 | 2.244643461 |
| NM_005656 | transmembrane protease, serine 2 | TMPRSS2 | -0.212052557 | 0.863308108 | 2.466676441 | 5.527688971 | 0.018492715 | 6.402915622 |
| NM_032405 | transmembrane protease, serine 3 | TMPRSS3 | 0.004758029 | 1.003303459 | 4.135217485 | 17.57213381 | 0.005236149 | 17.51427612 |
| NM_032780 | transmembrane protein 25 | TMEM25 | -0.443797697 | 0.735196754 | 2.658609823 | 6.314243177 | 0.001865505 | 8.588507964 |
| NM_001011655 | transmembrane protein 44 | TMEM44 | -0.361080857 | 0.778581054 | 1.591541482 | 3.013711847 | 0.021996471 | 3.870774702 |
| CO725450 | TRIP6 | TRIP6 | -0.167736927 | 0.890238052 | 1.16019587 | 2.234877678 | 0.006850129 | 2.51042704 |
| NM_012101 | tripartite motif-containing 29 | TRIM29 | 0.047448917 | 1.033435907 | 1.620047921 | 3.073852464 | 0.00253107 | 2.974400679 |
| NM_033342 | tripartite motif-containing 7 | TRIM7 | 1.363470873 | 2.573034627 | 4.006515868 | 16.07242672 | 0.040064487 | 6.246486756 |
| NM_203293 | tripartite motif-containing 7 | TRIM7 | 0.222418659 | 1.166687885 | 1.97355521 | 3.927347374 | 0.000839571 | 3.366236527 |
| NM_013353 | tropomodulin 4 | muscle | 0.110926519 | 1.079921555 | 2.204311474 | 4.608545436 | 0.001683714 | 4.267481665 |
| NM_213674 | tropomyosin 2 | TPM2 | -0.331224614 | 0.794861489 | 2.633248027 | 6.204212182 | 0.000480102 | 7.805400397 |
| NM_003280 | troponin C, slow | TNNC1 | -0.275355805 | 0.82624652 | 2.081454396 | 4.232336675 | 0.000256031 | 5.122365508 |
| NM_003279 | troponin C2, fast | TNNC2 | 0.24382797 | 1.184130405 | 1.9075247 | 3.75164859 | 0.030466747 | 3.168273168 |
| NM_003283 | troponin T1, skeletal, slow | TNNT1 | 0.210090446 | 1.156760701 | 1.399822746 | 2.638691604 | 0.008424945 | 2.281104123 |
| DV769210 | TTC14 | TTC14 | -1.474916785 | 0.359754145 | 1.847843666 | 3.599617635 | 0.000637424 | 10.00577112 |
| NM_003320 | tubby homolog | TUB | -0.064637698 | 0.956185413 | 2.279153869 | 4.853931903 | 0.003184999 | 5.076350085 |
| NM_015644 | tubulin tyrosine ligase-like family, member 3 | TTLL3 | -0.064792547 | 0.956082788 | 1.290594987 | 2.44628923 | 0.000707027 | 2.558658372 |
| NM_016437 | tubulin, gamma 2 | TUBG2 | 0.785184101 | 1.723312215 | 1.740966819 | 3.342590958 | 0.021707322 | 1.939631675 |
| XR_014480 | tubulin, gamma complex associated protein 6 | TUBGCP6 | -0.381435132 | 0.767673561 | 1.467224602 | 2.764894824 | 0.001667569 | 3.601654355 |
| NM_003193 | tubulin-specific chaperone e | TBCE | 0.168799742 | 1.124122876 | 3.07092518 | 8.403120544 | 0.00053672 | 7.475268695 |
| NM_006545 | tumor suppressor candidate 4 | TUSC4 | -0.025029745 | 0.982800336 | 1.551373291 | 2.930960024 | 0.004394508 | 2.982253787 |
| XR_011976 | tumor suppressor candidate 5 | TUSC5 | -0.188173094 | 0.877716483 | 2.339490717 | 5.061239401 | 0.006210185 | 5.766371601 |
| NM_001010938 | tyrosine kinase, non-receptor, 2 | TNK2 | 0.205830631 | 1.153350197 | 1.698498296 | 3.245629444 | 0.000781085 | 2.814088429 |
| NM_017619 | U11/U12 snRNP 65K protein | FLJ25070 | 0.045115462 | 1.03176575 | 2.780779691 | 6.872236522 | 0.00283841 | 6.660655795 |
| NM_024954 | ubiquitin domain containing 1 | UBTD1 | 0.435889971 | 1.352745058 | 2.669926053 | 6.363965668 | 0.032931536 | 4.704482659 |
| NM_003940 | ubiquitin specific protease 13 | USP13 | -0.029026563 | 0.98008137 | 1.905326812 | 3.745937456 | 0.004740609 | 3.822067811 |
| NM_018218 | ubiquitin specific protease 40 | USP40 | 1.505464343 | 2.839160373 | 4.035634087 | 16.40011569 | 0.001025616 | 5.776396376 |
| NM_014871 | ubiquitin specific protease 52 | USP52 | 0.199287174 | 1.148130931 | 3.378673874 | 10.40116969 | 0.000329678 | 9.059219127 |
| NM_003968 | ubiquitin-activating enzyme E1C | UBE1C | -0.038773489 | 0.973482204 | 1.543893179 | 2.915802856 | 0.001487145 | 2.995229747 |
| NM_024642 | UDP-N-acetyl-alpha-D-galactosamine:polypeptide N-acetylgalactosaminyltransferase 12 | GALNT12 | -0.623955643 | 0.648889334 | 1.473002894 | 2.775991006 | 0.003565612 | 4.278065397 |
| NM_021808 | UDP-N-acetyl-alpha-D-galactosamine:polypeptide N-acetylgalactosaminyltransferase 9 | GALNT9 | 0.467752864 | 1.382953707 | 3.280713292 | 9.718362807 | 0.003031354 | 7.02725099 |
| AW014767 | UI-H-BI0-aae-f-12-0-UIs1 NCI_CGAP_Sub1 cDNA clone IMAGE:2709262 3' sequence | | -0.668410243 | 0.629199643 | 2.557408556 | 5.886493742 | 0.000129993 | 9.355526195 |
| NM_006377 | unc-13 homolog B | UNC13B | -0.517328202 | 0.698664527 | 1.670074237 | 3.182309683 | 0.00311214 | 4.554846511 |

| | | | | | | | | |
|---|---|---|---|---|---|---|---|---|
| NM_199242 | unc-13 homolog D | UNC13D | -0.096692718 | 0.935174361 | 2.71924564 | 6.58528391 | 6.83E-05 | 7.04177123 |
| NM_003565 | unc-51-like kinase 1 | ULK1 | -0.040783378 | 0.972126943 | 2.017367931 | 4.048445149 | 0.00083376 | 4.16452314 |
| XM_379766 | unc-84 homolog A | UNC84A | 0.230590616 | 1.173315187 | 1.352898483 | 2.554247779 | 0.00989838 | 2.176949389 |
| NM_018463 | uncharacterized hematopoietic stem/progenitor cells protein MDS028 | MDS028 | -0.393939776 | 0.761048459 | 1.813358054 | 3.514594031 | 0.000669486 | 4.618094931 |
| NM_020217 | Unknown | Unknown | -0.343939383 | 0.787886989 | 2.654137516 | 6.294699539 | 0.015992142 | 7.989343176 |
| NM_022907 | Unknown | Unknown | -0.329909067 | 0.795586628 | 2.319830469 | 4.992735463 | 0.038943732 | 6.275539693 |
| NM_139205 | Unknown | Unknown | -0.069328652 | 0.953081404 | 1.990037502 | 3.972473242 | 0.014438027 | 4.168031425 |
| NM_023011 | UPF3 regulator of nonsense transcripts homolog A | UPF3A | -0.764122794 | 0.588811277 | 2.48116028 | 5.583463333 | 0.006804521 | 9.482602578 |
| XR_012616 | upregulated in colorectal cancer gene 1 protein precursor | LOC705348 | 0.201370214 | 1.149789861 | 2.539098901 | 5.812258633 | 0.021813401 | 5.055061651 |
| NM_006760 | uroplakin 2 | UPK2 | 0.648147261 | 1.567154331 | 2.604898246 | 6.083485927 | 0.030576091 | 3.881867795 |
| CN643806 | USP32 | USP32 | 0.416555591 | 1.334737086 | 2.565980902 | 5.921574802 | 0.003292093 | 4.436510279 |
| XM_373419 | valyl-tRNA synthetase 2-like | VARS2L | -0.083231759 | 0.94394077 | 2.248745815 | 4.752694979 | 0.000415367 | 5.034950423 |
| NM_022913 | vasculin | DKFZp761C169 | 0.107675827 | 1.077491006 | 1.34418713 | 2.538871064 | 0.004031636 | 2.356280516 |
| NM_004448 | v-erb-b2 erythroblastic leukemia viral oncogene homolog 2, neuro/glioblastoma derived oncogene homolog | ERBB2 | 0.151845755 | 1.110989942 | 1.553024846 | 2.934317223 | 0.001481294 | 2.641173526 |
| NM_001982 | v-erb-b2 erythroblastic leukemia viral oncogene homolog 3 | ERBB3 | -0.727218387 | 0.604067474 | 1.683179792 | 3.211349737 | 0.031262592 | 5.316210316 |
| NM_003715 | vesicle docking protein p115 | VDP | 0.024060446 | 1.016817275 | 1.357780643 | 2.562906138 | 0.006629131 | 2.520517895 |
| NM_005239 | v-ets erythroblastosis virus E26 oncogene homolog 2 | ETS2 | -0.066493305 | 0.954956349 | 1.506139523 | 2.840489408 | 5.98E-05 | 2.974470415 |
| NM_005252 | v-fos FBJ murine osteosarcoma viral oncogene homolog | FOS | -0.678284172 | 0.624908049 | 4.085023014 | 16.97127459 | 6.66E-05 | 27.15803488 |
| NM_003385 | visinin-like 1 | VSNL1 | 0.020256643 | 1.01413987 | 2.536535668 | 5.801941174 | 0.024032119 | 5.721046323 |
| XR_014061 | WAP four-disulfide core domain 1 precursor | WFDC1 | -0.570361189 | 0.673448165 | 1.496169793 | 2.8209279 | 0.003836219 | 4.188782518 |
| NM_015626 | WD repeat and SOCS box-containing 1 | WSB1 | 0.069788957 | 1.049563138 | 2.037243758 | 4.104606037 | 0.002122578 | 3.91077572 |
| NM_018262 | WD repeat domain 10 | WDR10 | -0.054706894 | 0.962790029 | 1.377506388 | 2.598189013 | 0.00010781 | 2.698603989 |
| NM_025132 | WD repeat domain 19 | WDR19 | 0.519878299 | 1.433834289 | 1.503098996 | 2.834509286 | 0.011196959 | 1.976873693 |
| NM_006784 | WD repeat domain 3 | WDR3 | 0.208404767 | 1.155409904 | 1.979811827 | 3.944416307 | 0.000770422 | 3.413867488 |
| NM_001006623 | WD repeat domain 33 | WDR33 | -0.980510878 | 0.506800243 | 1.564008259 | 2.95674179 | 0.018963592 | 5.834136488 |
| NM_032951 | Williams Beuren syndrome chromosome region 14 | WBSCR14 | 0.462843007 | 1.378255164 | 2.346905563 | 5.087319007 | 0.002354596 | 3.691130017 |
| NM_149379 | Williams Beuren syndrome chromosome region 20C | WBSCR20C | 0.158081545 | 1.115802389 | 1.388460935 | 2.617992447 | 0.012393338 | 2.346286827 |
| NM_145645 | Williams-Beuren Syndrome critical region protein 20 copy B | WBSCR20B | 0.278879129 | 1.213251908 | 1.652592062 | 3.143980048 | 0.001356941 | 2.591366251 |
| NM_003881 | WNT1 inducible signaling pathway protein 2 | WISP2 | 0.214385873 | 1.16020993 | 1.410924207 | 2.659074516 | 0.011362228 | 2.291890844 |
| NM_012477 | WW domain binding protein 1 | WBP1 | -0.431923877 | 0.741272617 | 2.150374955 | 4.439431543 | 9.70E-05 | 5.988932332 |
| NM_020231 | x 010 protein | MDS010 | 0.480803776 | 1.395520944 | 2.145064239 | 4.423119559 | 0.007578351 | 3.169511413 |
| NM_000380 | xeroderma pigmentosum, complementation group A | XPA | -0.046163299 | 0.968508558 | 1.448982537 | 2.730154389 | 0.015569529 | 2.818926448 |

| | | | | | | | | |
|---|---|---|---|---|---|---|---|---|
| CN644462 | YES1 | YES1 | -0.516575981 | 0.699028906 | 1.705853616 | 3.262218952 | 0.021718769 | 4.666786915 |
| NM_031477 | yippee-like 3 | YPEL3 | -0.041855926 | 0.9714045 | 1.491242295 | 2.81130951 | 0.00158578 | 2.894066797 |
| NM_006006 | zinc finger and BTB domain containing 16 | ZBTB16 | 0.210034567 | 1.156715899 | 1.935439445 | 3.824946171 | 0.011781398 | 3.306729141 |
| NM_015642 | zinc finger and BTB domain containing 20 | ZBTB20 | 0.051877004 | 1.036612722 | 2.736939074 | 6.666544117 | 0.000469089 | 6.431084604 |
| NM_020899 | zinc finger and BTB domain containing 4 | ZBTB4 | 0.25139316 | 1.190356045 | 1.955720403 | 3.879095792 | 0.006013677 | 3.258769347 |
| NM_144621 | zinc finger and BTB domain containing 8 | ZBTB8 | 0.295743034 | 1.227517025 | 1.307896669 | 2.47580325 | 0.023298194 | 2.016919684 |
| XR_013013 | Zinc finger CW-type PWWP domain protein 1 homolog | ZCWPW2 | 0.341618405 | 1.267177307 | 2.085026671 | 4.242829402 | 0.008136365 | 3.348252355 |
| NM_007146 | zinc finger protein 161 | ZNF161 | 0.385347776 | 1.306174616 | 1.309215186 | 2.478066986 | 0.004811451 | 1.897194262 |
| XM_290835 | zinc finger protein 181 | HHZ181 | -0.483743655 | 0.715119548 | 1.939344035 | 3.835312241 | 0.014443908 | 5.363176341 |
| NM_006385 | zinc finger protein 211 | ZNF211 | 0.025028188 | 1.017499572 | 1.675576046 | 3.194468792 | 0.027926984 | 3.139528389 |
| NM_016423 | zinc finger protein 219 | ZNF219 | 0.077069525 | 1.054873151 | 1.579479284 | 2.988619611 | 0.000135203 | 2.833155444 |
| XR_012685 | zinc finger protein 221 | ZNF221 | -0.362986714 | 0.777553197 | 1.920212955 | 3.784789215 | 0.008259005 | 4.867563055 |
| NM_013360 | zinc finger protein 222 | ZNF222 | -0.915453643 | 0.530177135 | 1.669868287 | 3.18185543 | 0.00093268 | 6.00149501 |
| NM_013398 | zinc finger protein 224 | ZNF224 | -0.756250596 | 0.592032961 | 2.881635977 | 7.369853682 | 0.009755813 | 12.44838407 |
| NM_006630 | zinc finger protein 234 | ZNF234 | -0.443575876 | 0.735309803 | 1.947674957 | 3.857523529 | 0.0049768 | 5.246120089 |
| NM_019591 | zinc finger protein 26 | KOX 20 | 0.225625274 | 1.169283914 | 1.486409136 | 2.801907126 | 0.041844293 | 2.39625902 |
| NM_005455 | zinc finger protein 265 | ZNF265 | -0.311527367 | 0.805788227 | 1.817390839 | 3.524432167 | 0.043645132 | 4.373893846 |
| NM_152287 | zinc finger protein 276 homolog | ZFP276 | 0.018950311 | 1.013222003 | 1.521626678 | 2.871145968 | 0.013080613 | 2.833679055 |
| NM_00101087 7 | zinc finger protein 311 | ZNF311 | -0.658819165 | 0.633396515 | 1.796211404 | 3.473069795 | 0.002202457 | 5.483247402 |
| NM_00100536 8 | zinc finger protein 32 | KOX 30 | 0.376626563 | 1.298302497 | 1.736224938 | 3.331622493 | 0.008979655 | 2.566137322 |
| NM_018555 | zinc finger protein 331 | ZNF331 | -0.804154843 | 0.572697479 | 1.820321904 | 3.531599892 | 0.000322579 | 6.166606317 |
| NM_032433 | zinc finger protein 333 | ZNF333 | 0.099408993 | 1.071334496 | 2.316504434 | 4.981238311 | 0.046524774 | 4.649564006 |
| NM_022095 | zinc finger protein 335 | ZNF335 | -0.112927677 | 0.924709633 | 2.240024266 | 4.724050103 | 0.003399727 | 5.108684861 |
| NM_145914 | zinc finger protein 38 | ZNF38 | 0.311552043 | 1.241042088 | 1.732881578 | 3.323910596 | 0.0059926 | 2.678322217 |
| NM_133476 | zinc finger protein 384 | ZNF384 | 0.28335932 | 1.217025435 | 1.738546445 | 3.336987877 | 0.005656112 | 2.741921229 |
| NM_017810 | zinc finger protein 434 | ZNF434 | 0.231548067 | 1.174094121 | 1.451002234 | 2.733979138 | 0.0058234 | 2.328586004 |
| NM_181489 | zinc finger protein 445 | ZNF445 | 0.315689611 | 1.244606436 | 1.585377152 | 3.000862368 | 0.022844007 | 2.411093404 |
| XR_011954 | zinc finger protein 446 | ZNF446 | 0.390894971 | 1.311206554 | 1.777063333 | 3.42727827 | 0.001625195 | 2.613835524 |
| NM_145291 | zinc finger protein 509 | ZNF509 | 0.498040768 | 1.412294313 | 1.58739227 | 3.00505682 | 0.009609032 | 2.127783701 |
| NM_018181 | zinc finger protein 532 | ZNF532 | -0.017767292 | 0.987760175 | 2.064256196 | 4.182183012 | 0.02195904 | 4.234006511 |
| NM_152520 | zinc finger protein 533 | ZNF533 | 0.203032512 | 1.151115432 | 2.21011165 | 4.627110812 | 0.007872383 | 4.019675771 |
| NM_020747 | zinc finger protein 608 | ZNF608 | 0.256227159 | 1.194351228 | 2.497361362 | 5.64651752 | 0.004080022 | 4.72768595 |
| XM_171060 | zinc finger protein 620 | ZNF620 | 0.295366417 | 1.227196622 | 1.848242824 | 3.600613698 | 0.016877259 | 2.934015327 |
| NM_198484 | zinc finger protein 621 | ZNF621 | 0.311544309 | 1.241035435 | 1.671254625 | 3.18491446 | 0.022493136 | 2.56633644 |
| NM_003416 | zinc finger protein 7 | KOX 4 | -0.378617619 | 0.769174255 | 2.300202853 | 4.925270132 | 0.000841314 | 6.403321614 |
| NM_003427 | zinc finger protein 76 | ZNF76 | 0.089175654 | 1.063762182 | 1.510319159 | 2.848730529 | 0.007532836 | 2.67797688 |
| NM_021089 | zinc finger protein 8 | ZNF8 | 0.473156616 | 1.388143405 | 2.221634429 | 4.664215443 | 0.009468951 | 3.360038614 |
| XM_930351 | zinc finger protein HIT-40 | zinc finger protein HIT-40 | 0.093116901 | 1.066672209 | 1.782966261 | 3.441330042 | 0.012178943 | 3.226230151 |
| NM_016478 | zinc finger, C3HC-type containing 1 | ZC3HC1 | 0.341644339 | 1.267200086 | 1.614251469 | 3.061527132 | 0.005428582 | 2.415977686 |
| NM_017612 | zinc finger, CCHC domain containing 8 | ZCCHC8 | 0.061143631 | 1.043292456 | 1.649936247 | 3.138197711 | 0.032364819 | 3.007975082 |
| NM_024657 | zinc finger, CW-type with coiled-coil domain 2 | ZCWCC2 | -0.146983336 | 0.903136942 | 1.885897634 | 3.695828044 | 0.00134688 | 4.092212235 |

| | | | | | | | | |
|---|---|---|---|---|---|---|---|---|
| NM_013304 | zinc finger, DHHC-type containing 1 | ZDHHC1 | -0.167542284 | 0.890358168 | 1.429653585 | 2.693820245 | 0.008489798 | 3.025546731 |
| NM_014733 | zinc finger, FYVE domain containing 16 | ZFYVE16 | -0.494014981 | 0.710046306 | 1.462310649 | 2.755493357 | 0.013048959 | 3.880723459 |
| NM_015363 | zinc finger, imprinted 2 | ZIM2 | 1.189326514 | 2.280462606 | 2.565558399 | 5.919840885 | 0.018881032 | 2.595894741 |
| NM_006624 | zinc finger, MYND domain containing 11 | ZMYND11 | 0.471316794 | 1.386374279 | 1.449147536 | 2.730466651 | 0.004603979 | 1.969501809 |
| XM_372226 | zinc finger, X-linked, duplicated B | ZXDB | -0.313232213 | 0.804836582 | 1.422059639 | 2.679677977 | 0.027101601 | 3.329468413 |
| XR_013613 | ZXD family zinc finger C isoform 2 | ZXDC | -0.047441726 | 0.967650705 | 2.1942901 | 4.576644081 | 0.000739771 | 4.729644754 |
