# Supplementary material for: Transcriptional Reprogramming in Nonhuman Primate (Rhesus Macaque) Tuberculosis Granulomas
Source: PLoS One. 2010 Aug 31;5(8):e12266. doi: 10.1371/journal.pone.0012266 (PMC2930844; doi:10.1371/journal.pone.0012266)
Supplement: Table S6 — This table contains a comprehensive list of all NHP genes with a lower expression in a statistically significant manner, in late (week 13) TB lesions, relative to normal lungs. (0.20 MB PDF) [file pone.0012266.s006.pdf]

Gene Name	Description	Symbol	Av M Granuloma	Av Numeric Granuloma	Av M Normal	Av Numeric Normal	*P*	Ratio (Granuloma/Normal)
NM_016816	2',5'-oligoadenylate synthetase 1, 40/46kDa (OAS1), transcript variant E18.	OAS1	-1.135573135	-2.197058273	-0.09732872	0.934762186	0.017169632	2.350392757
NM_005721	ARP3 actin-related protein 3 homolog (yeast) (ACTR3)	ACTR3	-2.634217684	-6.208383527	-0.686145601	0.621512107	0.013797397	9.98915944
NM_005099	a disintegrin-like and metalloprotease (reprolysin type) with thrombospondin type 1 motif, 4 (ADAMTS4).	ADAMTS4	-1.51473964	-2.857472551	-0.169059777	0.88942214	0.028214071	3.212729277
NM_019859	5-hydroxytryptamine (serotonin) receptor 7 (adenylate cyclase-coupled) (HTR7), transcript variant d	HTR7	-2.13030968	-4.378114482	-0.219636638	0.858781705	0.024166863	5.098052808
NM_001628	aldo-keto reductase family 1, member B1 (aldose reductase) (AKR1B1)	AKR1B1	-1.915009547	-3.771163099	-0.062091611	0.957874392	0.001980559	3.937012131
NM_003689	aldo-keto reductase family 7, member A2 (aflatoxin aldehyde reductase) (AKR7A2).	AKR7A2	-1.277268474	-2.423796328	0.20737827	1.154588107	0.006397498	2.099273596
NM_000295	serine (or cysteine) proteinase inhibitor, clade A (alpha-1 antiproteinase, antitrypsin), member 1 (SERPINA1), transcript variant 1.	SERPINA1	-3.931267261	-15.25560259	-1.114100852	0.46197899	0.00817182	33.02228651
NM_001085	serine (or cysteine) proteinase inhibitor, clade A (alpha-1 antiproteinase, antitrypsin), member 3 (SERPINA3).	SERPINA3	-2.045918073	-4.12935964	-0.048408548	0.967002452	0.013569119	4.270268016
NM_000934	serine (or cysteine) proteinase inhibitor, clade F (alpha-2 antiplasmin, pigment epithelium derived factor), member 2 (SERPINF2)	SERPINF2	-3.059851621	-8.338868401	-0.206993158	0.866340965	0.007404545	9.625388543
NM_032797	apoptosis-inducing factor (AIF)-like mitochondrion-associated inducer of death (AMID)	AMID	-1.30973804	-2.478965236	-0.03059164	0.979018726	0.004217097	2.532091747
NM_000480	adenosine monophosphate deaminase (isoform E) (AMPD3), transcript variant 1.	AMPD3	-2.263007587	-4.799910773	0.160259701	1.11748828	0.009199358	4.295267215
NM_001637	acyloxyacyl hydrolase (neutrophil) (AOAH).	AOAH	-2.656383165	-6.304505277	0.074150741	1.052741145	8.58E-05	5.988656665
NM_022488	APG3 autophagy 3-like (S. cerevisiae) (APG3L).	APG3L	-2.128676951	-4.373162481	-0.569618342	0.673795014	0.00568738	6.490345562
NM_004309	Rho GDP dissociation inhibitor (GDI) alpha (ARHGDIA).	ARHGDIA	-1.486667112	-2.802408195	-0.692258427	0.618884276	0.003067945	4.528161894
NM_005170	achaete-scute complex-like 2 (Drosophila) (ASCL2)	ASCL2	-2.069530201	-4.197499639	0.331211007	1.258068962	0.008901653	3.336462281
NM_030766	BCL2-like 14 (apoptosis facilitator) (BCL2L14), transcript variant 2	BCL2L14	-1.426754385	-2.688412254	0.072275662	1.05137378	0.000148913	2.55704708
NM_005868	BET1 homolog (S. cerevisiae) (BET1).	BET1	-2.328401392	-5.022485146	-1.142057051	0.453113051	0.008389967	11.08439746
NM_199193	brain and reproductive organ-expressed (TNFRSF1A modulator) (BRE), transcript variant 4	BRE	-1.135427008	-2.196835749	-0.157978821	0.896279857	0.008753121	2.451060047
NM_152322	BTB (POZ) domain containing 11 (BTBD11), transcript variant 1.	BTBD11	-2.108225354	-4.311606016	0.285639058	1.218950092	0.007331449	3.537147291
NM_144653	BTB (POZ) domain containing 14A (BTBD14A)	BTBD14A	-1.771971477	-3.415203336	0.152130358	1.11120913	0.01875841	3.073411877
NM_052876	BTB (POZ) domain containing 14B (BTBD14B)	BTBD14B	-3.070206989	-8.398938412	-0.617173292	0.651947053	0.030058763	12.88285356
NM_007311	benzodiazapine receptor (peripheral) (BZRP), transcript variant PBR-S	BZRP	-1.662843658	-3.166400309	-0.410525338	0.752349366	0.001580943	4.208683428
NM_006367	CAP, adenylate cyclase-associated protein 1 (yeast) (CAP1)	CAP1	-1.298002864	-2.458882616	-0.085948985	0.942164589	0.000117419	2.609822789
NM_001747	capping protein (actin filament), gelsolin-like (CAPG).	CAPG	-1.687758081	-3.221556911	-0.190604459	0.876238519	0.019868411	3.676575318
XR_009750	Macaca mulatta Cathepsin Z precursor (Cathepsin X) (Cathepsin P) (LOC694157)	CTPP	-2.312828127	-4.968561172	-0.145954877	0.903780994	0.001741978	5.497527836

NM_012116	Cas-Br-M (murine) ecotropic retroviral transforming sequence c (CBLC).	CBLC	-1.127138764	-2.18425117	-0.078395516	0.947110384	0.025052953	2.30622661
NM_012117	chromobox homolog 5 (HP1 alpha homolog, Drosophila) (CBX5)	CBX5	-1.820429036	-3.531862151	-0.077668883	0.947587529	0.043471807	3.727214681
NM_002986	chemokine (C-C motif) ligand 11 (CCL11)	CCL11	-2.559218671	-5.893884017	-0.403978438	0.755771259	0.005802769	7.798502449
NM_006274	chemokine (C-C motif) ligand 19 (CCL19).	CCL19	-2.830621411	-7.113804914	0.016779913	1.011698852	0.001389972	7.031543924
NM_002982	chemokine (C-C motif) ligand 2 (CCL2)	CCL2	-2.494629099	-5.635833925	-0.104471495	0.930145622	0.019201845	6.05908773
NM_002985	chemokine (C-C motif) ligand 5 (CCL5).	CCL5	-1.601504038	-3.03459511	-0.14511124	0.904309647	0.002501796	3.355703568
NM_006273	chemokine (C-C motif) ligand 7 (CCL7).	CCL7	-2.169149889	-4.497582949	0.582494454	1.497436114	0.004600057	3.003522426
NM_001295	chemokine (C-C motif) receptor 1 (CCR1).	CCR1	-2.491349268	-5.623035939	0.052278973	1.036901586	0.010146012	5.422921531
NM_000733	CD3E antigen, epsilon polypeptide (TiT3 complex) (CD3E).	CD3E	-1.509016417	-2.84615931	-0.241362432	0.845946052	0.005365495	3.364469049
NM_001250	CD40 antigen (TNF receptor superfamily member 5) (CD40), transcript variant 1.	CD40	-1.306883025	-2.47406435	0.14613756	1.106602862	0.000563807	2.235729216
NM_006889	CD86 antigen (CD28 antigen ligand 2, B7-2 antigen) (CD86), transcript variant 2	CD86	-3.146637818	-8.855893167	-0.289008972	0.818464092	0.012616695	10.82013647
NM_000389	cyclin-dependent kinase inhibitor 1A (p21, Cip1) (CDKN1A), transcript variant 1.	CDKN1A	-2.791400855	-6.923016827	-0.612342451	0.654133746	0.000431614	10.58348826
NM_001803	CDW52 antigen (CAMPATH-1 antigen) (CDW52)	CDW52	-2.441728788	-5.432923709	-0.328023937	0.796626879	0.001149919	6.819910115
NM_005194	CCAAT/enhancer binding protein (C/EBP), beta (CEBPB)	CEBPB	-1.542170206	-2.912322667	0.178725701	1.131883677	0.00018275	2.572987601
NM_005195	CCAAT/enhancer binding protein (C/EBP), delta (CEBPD)	CEBPD	-1.810671763	-3.508055963	-1.017513583	0.493966948	0.000480602	7.101803023
NM_005507	cofilin 1 (non-muscle) (CFL1)	CFL1	-1.397478094	-2.634406714	-0.674539873	0.626532011	0.001957587	4.204744
XR_010663	Macaca mulatta Mitochondrial intermembrane space import and assembly protein 40 (Coiled-coil-helix-coiled-coil-helix domain-containing protein 4) (CHCHD4)	CHCHD4	-1.834702389	-3.566978176	0.147217798	1.107431757	0.011104968	3.220946261
NM_003465	chitinase 1 (chitotriosidase) (CHIT1)	CHIT1	-1.54745256	-2.923005533	-0.321122873	0.800446635	0.012738224	3.651718186
NM_018413	carbohydrate (chondroitin 4) sulfotransferase 11 (CHST11)	CHST11	-3.565311093	-11.83765239	0.267549436	1.203761379	0.031957579	9.833886179
NM_014918	carbohydrate (chondroitin) synthase 1 (CHSY1)	CHSY1	-1.841663055	-3.584229594	0.13935266	1.1014108	0.001918074	3.254216859
NM_020990	creatine kinase, mitochondrial 1 (ubiquitous) (CKMT1), nuclear gene encoding mitochondrial protein	CKMT1	-1.93312918	-3.818825982	-0.160457604	0.894741226	0.030238963	4.268078715
NM_018235	CNDP dipeptidase 2 (metallopeptidase M20 family) (CNDP2)	CNDP2	-1.458390534	-2.748016247	0.301547673	1.232465851	0.004226985	2.229689565
NM_000090	collagen, type III, alpha 1 (Ehlers-Danlos syndrome type IV, autosomal dominant) (COL3A1).	COL3A1	-1.559910211	-2.948354933	-0.170141197	0.888755694	0.005125988	3.317396392
NM_021149	coactosin-like 1 (Dictyostelium) (COTL1).	COTL1	-1.59169791	-3.014038634	0.29892422	1.230226723	0.000418023	2.449986314
NM_004074	cytochrome c oxidase subunit 8A (ubiquitous) (COX8A).	COX8A	-2.132940926	-4.386106754	-0.673828185	0.626841158	0.013839254	6.997158207
NM_001870	carboxypeptidase A3 (mast cell) (CPA3).	CPA3	-4.182811039	-18.16149473	-1.961628026	0.256738574	0.027004556	70.7392523
NM_019609	carboxypeptidase X (M14 family) (CPXM)	CPXM	-1.83948858	-3.578831402	0.162945443	1.11957055	0.003442009	3.196610881
NM_005211	colony stimulating factor 1 receptor, formerly McDonough feline sarcoma viral (v-fms) oncogene homolog (CSF1R).	CSF1R	-2.018654082	-4.052055914	0.011950088	1.00831757	0.003786995	4.018630672
NM_006140	colony stimulating factor 2 receptor, alpha, low-affinity (granulocyte-macrophage) (CSF2RA), transcript variant 1.	CSF2RA	-2.237873852	-4.717013898	0.281717003	1.215640801	0.003036605	3.880269478
NM_156039	colony stimulating factor 3 receptor (granulocyte) (CSF3R), transcript variant 3	CSF3R	-2.069371898	-4.197039083	0.233555719	1.175729127	0.018696768	3.569733018
NM_004385	chondroitin sulfate proteoglycan 2 (versican) (CSPG2)	CSPG2	-2.099781891	-4.28644577	-0.371028127	0.773231262	0.004819669	5.54354949

NM_000100	cystatin B (stefin B) (CSTB).	CSTB	-1.784672423	-3.445402239	-0.266424441	0.831377471	0.000220962	4.14420929
NM_001565	chemokine (C-X-C motif) ligand 10 (CXCL10)	CXCL10	-4.633536774	-24.82181617	0.005046517	1.003504104	0.007530597	24.73514166
NM_022059	chemokine (C-X-C motif) ligand 16 (CXCL16).	CXCL16	-1.907728587	-3.752178826	0.31005661	1.239756346	0.015086292	3.026545369
NM_002090	chemokine (C-X-C motif) ligand 3 (CXCL3).	CXCL3	-2.17236279	-4.507610287	-0.520678952	0.697043718	0.04959328	6.466754056
NM_002416	chemokine (C-X-C motif) ligand 9 (CXCL9)	CXCL9	-5.951113419	-61.86765383	0.077041534	1.054852685	0.007088675	58.65051559
NM_003467	chemokine (C-X-C motif) receptor 4 (CXCR4)	CXCR4	-1.33378342	-2.520628354	0.640759838	1.559150116	0.001202222	1.616668163
NM_000397	cytochrome b-245, beta polypeptide (chronic granulomatous disease) (CYBB).	CYBB	-3.213936306	-9.278787577	0.003272132	1.002270643	0.001282425	9.257766496
NM_001919	dodecenoyl-Coenzyme A delta isomerase (3,2 trans-enoyl-Coenzyme A isomerase) (DCI), nuclear gene encoding mitochondrial protein.	DCI	-2.067457478	-4.191473412	-0.617964933	0.651589412	0.001417175	6.432691099
XR_012642	Macaca mulatta ATP-dependent RNA helicase DDX19A (DEAD box protein 19A) (DDX19-like protein) (LOC712525)	DDX19L	-2.106800903	-4.307351035	-0.043411851	0.970357419	0.010136288	4.43893245
NM_022047	differentially expressed in FDCP 6 homolog (mouse) (DEF6).	DEF6	-1.271260729	-2.413724016	-0.386863096	0.76479071	0.023813434	3.156058233
NM_004753	dehydrogenase/reductase (SDR family) member 3 (DHRS3).	DHRS3	-1.275521605	-2.42086328	-0.360240307	0.779034807	0.00716739	3.107516198
NM_005771	dehydrogenase/reductase (SDR family) member 9 (DHRS9), transcript variant 1.	DHRS9	-1.769875436	-3.410245109	0.551684934	1.465796611	0.002005951	2.326547273
NM_006145	DnaJ (Hsp40) homolog, subfamily B, member 1 (DNAJB1)	DNAJB1	-1.476370737	-2.782478881	-0.07770134	0.947566211	0.010504903	2.936447975
NM_006260	DnaJ (Hsp40) homolog, subfamily C, member 3 (DNAJC3)	DNAJC3	-1.768407742	-3.406777536	0.403505943	1.322718392	0.001464901	2.575587938
NM_004413	dipeptidase 1 (renal) (DPEP1).	DPEP1	-2.433968187	-5.403777176	0.075898441	1.054017223	0.002380287	5.126839543
NM_004416	deltex homolog 1 (Drosophila) (DTX1)	DTX1	-3.847731858	-14.39735471	0.146017325	1.106510641	0.006457493	13.01149233
NM_006014	DNA segment on chromosome X (unique) 9879 expressed sequence (DXS9879E)	DXS9879E	-1.224551967	-2.336828676	-0.183379277	0.880637829	0.001117623	2.653563815
NM_001953	endothelial cell growth factor 1 (platelet-derived) (ECGF1)	ECGF1	-1.673601426	-3.190099507	0.71121835	1.63718613	0.023199686	1.948525857
NM_001428	enolase 1, (alpha) (ENO1)	ENO1	-2.027676356	-4.077475917	-0.200722296	0.870114825	0.000776665	4.686135439
NM_001975	enolase 2 (gamma, neuronal) (ENO2)	ENO2	-2.743789905	-6.698276381	-0.411357157	0.751915707	0.002745747	8.908280971
NM_016135	ets variant gene 7 (TEL2 oncogene) (ETV7).	ETV7	-1.858416084	-3.626093395	-0.256362816	0.837195919	0.002300792	4.331236347
NM_000569	Fc fragment of IgG, low affinity IIIa, receptor for (CD16) (FCGR3A)	FCGR3A	-1.880465034	-3.681937235	-0.272670718	0.827785729	0.006075203	4.447935142
NM_000570	Fc fragment of IgG, low affinity IIIb, receptor (CD16b) (FCGR3B).	FCGR3B	-2.035319311	-4.09913445	-0.333562868	0.793574257	0.005472221	5.165407539
NM_005248	Gardner-Rasheed feline sarcoma viral (v-fgr) oncogene homolog (FGR).	FGR	-1.28016437	-2.428666458	-0.101314028	0.932183558	0.005422727	2.605352172
NM_000803	folate receptor 2 (fetal) (FOLR2)	FOLR2	-1.417649774	-2.671499549	0.433800911	1.350787666	0.021260431	1.977734633
NM_000804	folate receptor 3 (gamma) (FOLR3)	FOLR3	-1.806699293	-3.498409795	0.212113716	1.158384108	0.004081609	3.020077511
NM_012418	fascin homolog 2, actin-bundling protein, retinal (Strongylocentrotus purpuratus) (FSCN2).	FSCN2	-1.620004996	-3.073761008	0.366815318	1.289503169	0.013778872	2.383678522
NM_001465	FYN binding protein (FYB-120/130) (FYB)	FYB	-1.575144942	-2.979654266	-0.238304784	0.847740852	0.016758778	3.514817365
NM_007210	UDP-N-acetyl-alpha-D-galactosamine:polypeptide N-acetylgalactosaminyltransferase 6 (GalNAc-T6) (GALNT6).	GALNT6	-1.614050852	-3.061101435	0.192958469	1.143105429	0.027884902	2.677881986
NM_000161	GTP cyclohydrolase 1 (dopa-responsive dystonia) (GCH1)	GCH1	-3.745667616	-13.41400013	-0.086390821	0.941876088	0.009075443	14.24178859
NM_002065	glutamate-ammonia ligase (glutamine synthase) (GLUL).	GLUL	-2.146467764	-4.42742468	-0.922509369	0.527590551	0.016857562	8.391781608

XR_010040	Macaca mulatta Glycyl-tRNA synthetase (Glycine--tRNA ligase) (GlyRS) (LOC696505)	GlyRS	-1.556581758	-2.941560599	-0.842849917	0.557541107	0.022688735	5.27595286
NM_002074	guanine nucleotide binding protein (G protein), beta polypeptide 1 (GNB1)	GNB1	-1.551969506	-2.932171538	-0.52571228	0.694616083	0.012172509	4.221283683
NM_005274	guanine nucleotide binding protein (G protein), gamma 5 (GNG5)	GNG5	-1.269344762	-2.410520606	-0.231174906	0.851940803	0.000476285	2.829446126
NM_001005340	glycoprotein (transmembrane) nmb (GPNMB), transcript variant 1.	GPNMB	-3.014093253	-8.078532545	-0.441329549	0.736455598	0.00048243	10.96947673
NM_022107	G-protein signalling modulator 3 (AGS3-like, C. elegans) (GPSM3).	GPSM3	-1.752534942	-3.369500973	-0.237439131	0.84824967	0.00759415	3.972298596
NM_002085	glutathione peroxidase 4 (phospholipid hydroperoxidase) (GPX4)	GPX4	-1.273992906	-2.41829946	-0.596191083	0.661498099	0.003459747	3.655792001
NM_002068	guanine nucleotide binding protein (G protein), alpha 15 (Gq class) (GNA15).	GNA15	-2.194534937	-4.577420839	-0.173336928	0.88678918	0.008663165	5.161791487
NM_002993	chemokine (C-X-C motif) ligand 6 (granulocyte chemotactic protein 2) (CXCL6).	CXCL6	-3.960212651	-15.56477321	0.034588834	1.024264867	0.001184469	15.19604324
XR_013405	Macaca mulatta Stress-70 protein, mitochondrial precursor (75 kDa glucose-regulated protein) (GRP 75) (Peptide-binding protein 74) (PBP74) (Mortalin) (MOT) (LOC705066)	GRP 75	-1.578585538	-2.986768743	-0.426853232	0.743882554	0.047398817	4.015107932
NM_002101	glycophorin C (Gerbich blood group) (GYPC), transcript variant 1	GYPC	-1.436852205	-2.707295199	0.503194941	1.4173489	0.001522344	1.91011204
NM_005333	holocytochrome c synthase (cytochrome c heme-lyase) (HCCS)	HCCS	-2.754861496	-6.749878325	0.100339252	1.072025522	0.024928657	6.296378387
NM_004494	hepatoma-derived growth factor (high-mobility group protein 1-like) (HDGF).	HDGF	-1.533187668	-2.894246235	-0.328558353	0.796331839	0.003782626	3.634472579
NM_001235	serine (or cysteine) proteinase inhibitor, clade H (heat shock protein 47), member 1, (collagen binding protein 1) (SERPINH1).	SERPINH1	-1.979668443	-3.944024306	0.059762909	1.042294457	0.00411957	3.783982807
NM_002115	hexokinase 3 (white cell) (HK3), nuclear gene encoding mitochondrial protein.	HK3	-2.397488337	-5.268850838	-0.066453197	0.954982899	9.69E-05	5.517220094
NM_178580	histocompatibility (minor) 13 (HM13), transcript variant 2	HM13	-1.595649143	-3.022304756	-0.151185378	0.900510262	0.000446361	3.356213564
NM_002133	heme oxygenase (decycling) 1 (HMOX1)	HMOX1	-1.670857325	-3.184037496	0.372934546	1.294984245	0.005732254	2.458746126
NM_004838	homer homolog 3 (Drosophila) (HOMER3).	HOMER3	-1.820180465	-3.531253677	0.124396864	1.090051928	0.000731159	3.239527939
NM_006042	heparan sulfate (glucosamine) 3-O-sulfotransferase 3A1 (HS3ST3A1)	HS3ST3A1	-1.671412064	-3.185262044	0.120012722	1.086744446	0.002607871	2.93101295
NM_181755	hydroxysteroid (11-beta) dehydrogenase 1 (HSD11B1), transcript variant 2	HSD11B1	-1.630623769	-3.096468499	-0.158170977	0.896160487	0.044906104	3.455261131
NM_017870	heat shock 70kDa protein 5 (glucose-regulated protein, 78kDa) binding protein 1 (HSPA5BP1), transcript variant 1	HSPA5BP1	-1.492013834	-2.812813371	0.108979937	1.078465434	0.00978203	2.60816275
NM_000074	tumor necrosis factor (ligand) superfamily, member 5 (hyper-IgM syndrome) (TNFSF5)	TNFSF5	-1.477450404	-2.784561978	0.520622951	1.434574559	0.01612831	1.941036777
NM_000201	intercellular adhesion molecule 1 (CD54), human rhinovirus receptor (ICAM1).	ICAM1	-1.983781304	-3.955284044	-0.266310314	0.831443241	0.00102094	4.757130551

NM_002168	isocitrate dehydrogenase 2 (NADP+), mitochondrial (IDH2)	IDH2	-1.115491478	-2.166688084	0.164533956	1.120803958	6.50E-05	1.933155275
NM_207585	interferon (alpha, beta and omega) receptor 2 (IFNAR2), transcript variant 1	IFNAR2	-1.816310071	-3.521792895	0.207041934	1.154318969	0.006098892	3.050970302
NM_005534	interferon gamma receptor 2 (interferon gamma transducer 1) (IFNGR2).	IFNGR2	-1.608234816	-3.048785846	0.175428212	1.129299547	0.002073214	2.699714044
NM_000597	insulin-like growth factor binding protein 2 (36kD) (IGFBP2)	IGFBP2	-1.921864769	-3.789125091	0.32400149	1.251797751	0.005026823	3.026946715
NM_000206	interleukin 2 receptor, gamma (severe combined immunodeficiency) (IL2RG)	IL2RG	-2.202490658	-4.602732689	-0.701735844	0.614831998	0.00844724	7.486163216
NM_033416	IMP4, U3 small nucleolar ribonucleoprotein, homolog (yeast) (IMP4).	IMP4	-1.633687496	-3.103051188	-0.613177651	0.653755167	0.017317117	4.746503499
NM_000883	IMP (inosine monophosphate) dehydrogenase 1 (IMPDH1), transcript variant 1	IMPDH1	-1.430791333	-2.695945503	-0.328919482	0.79613253	0.006282116	3.386302405
NM_002205	integrin, alpha 5 (fibronectin receptor, alpha polypeptide) (ITGA5)	ITGA5	-1.592319101	-3.015336689	-0.184578945	0.879905842	0.03847044	3.426885635
NM_004972	Janus kinase 2 (a protein tyrosine kinase) (JAK2).	JAK2	-1.975759154	-3.933351602	-0.116096121	0.922681016	0.041502167	4.262959281
NM_002800	proteasome (prosome, macropain) subunit, beta type, 9 (large multifunctional protease 2) (PSMB9), transcript variant 1	PSMB9	-2.732297411	-6.645129944	-0.199271076	0.870990522	0.002888124	7.629394098
NM_004159	proteasome (prosome, macropain) subunit, beta type, 8 (large multifunctional protease 7) (PSMB8), transcript variant 1	PSMB8	-2.235878573	-4.710494675	0.249095152	1.188461487	0.000267809	3.963523199
NM_024552	LAG1 longevity assurance homolog 4 (S. cerevisiae) (LASS4).	LASS4	-1.991883124	-3.977558432	-0.362294771	0.777926215	0.006559531	5.113027888
NM_203463	LAG1 longevity assurance homolog 6 (S. cerevisiae) (LASS6).	LASS6	-1.468283851	-2.766925598	-0.128773245	0.914608832	0.000200183	3.025255718
NM_005564	lipocalin 2 (oncogene 24p3) (LCN2).	LCN2	-1.716262131	-3.285839771	-0.176080567	0.885104335	0.00025034	3.712375641
NM_005565	lymphocyte cytosolic protein 2 (SH2 domain containing leukocyte protein of 76kDa) (LCP2).	LCP2	-2.174913358	-4.515586424	-0.351580293	0.783725154	0.02974645	5.761696433
NM_002306	lectin, galactoside-binding, soluble, 3 (galectin 3) (LGALS3).	LGALS3	-1.796283664	-3.473243755	-0.414772453	0.7501378	0.000903094	4.630140965
NM_009587	lectin, galactoside-binding, soluble, 9 (galectin 9) (LGALS9), transcript variant long	LGALS9	-1.47336782	-2.776693275	-0.478364852	0.717790706	0.018722934	3.868388449
NM_005874	leukocyte immunoglobulin-like receptor, subfamily B (with TM and ITIM domains), member 2 (LILRB2).	LILRB2	-1.596781021	-3.024676861	-0.179107892	0.883248995	0.013002812	3.424489444
NM_006864	leukocyte immunoglobulin-like receptor, subfamily B (with TM and ITIM domains), member 3 (LILRB3)	LILRB3	-1.622214719	-3.078472582	1.491273744	2.811370794	0.002288285	1.09500767
NM_020437	aspartate beta hydroxylase (ASPH) (LOC57168).	LOC57168	-1.601162405	-3.033876597	0.541844631	1.455832757	0.022334987	2.083945827
XR_010082	Macaca mulatta neutrophil cytosolic factor 4 (40kD) isoform 1 (LOC695612)	LOC695612	-2.2779725	-4.849958829	-0.010167725	0.992977047	8.49E-05	4.884260761
XR_010591	Macaca mulatta Putative eukaryotic translation initiation factor 3 subunit (eIF-3) (LOC704219)	LOC704219	-1.067591369	-2.095931212	0.227102775	1.170482022	0.012156251	1.790656476
XR_014443	Macaca mulatta hydroxysteroid (17-beta) dehydrogenase 7 (LOC720399)	LOC720399	-1.476210632	-2.782170108	-0.251349962	0.840109938	0.005162313	3.311673845

XR_014618	Macaca mulatta Mps one binder kinase activator-like 2A (Mob1 homolog 2A) (LOC721441)	LOC721441	-2.697624518	-6.487328605	0.474750314	1.389677688	0.042520211	4.668225344
XR_014707	Macaca mulatta Complement C1r subcomponent precursor (Complement component 1, r subcomponent) (LOC722131)	LOC722131	-1.432351625	-2.698862777	-0.126637309	0.915963929	0.001335082	2.946472772
NM_000595	lymphotoxin alpha (TNF superfamily, member 1) (LTA).	LTA	-2.64592757	-6.258980016	0.386207942	1.306953618	0.008827525	4.78898404
NM_002341	lymphotoxin beta (TNF superfamily, member 3) (LTB), transcript variant 1	LTB	-3.972322744	-15.69597501	-0.504159772	0.705070892	0.002368699	22.26155579
NM_005461	v-maf musculoaponeurotic fibrosarcoma oncogene homolog B (avian) (MAFB)	MAFB	-1.969157302	-3.915393487	-0.404263607	0.755621885	0.010728973	5.181683542
NM_001009813	Meis1, myeloid ecotropic viral integration site 1 homolog 3 (mouse) (MEIS3), transcript variant 2.	MEIS3	-1.483231425	-2.795742377	-0.514191307	0.700185307	0.010592116	3.992860676
NM_002406	mannosyl (alpha-1,3-)-glycoprotein beta-1,2-N-acetylglucosaminyltransferase (MGAT1).	MGAT1	-2.079050324	-4.225289885	-0.130723138	0.913373515	0.004730827	4.626026282
NM_001001437	chemokine (C-C motif) ligand 3-like, centromeric (MGC12815)	MGC12815	-2.490657442	-5.620340133	-0.433166208	0.740634568	0.006149542	7.588546869
NM_020831	megakaryoblastic leukemia (translocation) 1 (MKL1).	MKL1	-2.318670166	-4.988721618	-1.254792909	0.419053716	0.024210937	11.90473066
NM_002421	matrix metalloproteinase 1 (interstitial collagenase) (MMP1).	MMP1	-1.957680623	-3.884369984	-0.128097294	0.915037457	0.014790391	4.24503932
NM_004530	matrix metalloproteinase 2 (gelatinase A, 72kDa gelatinase, 72kDa type IV collagenase) (MMP2)	MMP2	-2.423627419	-5.365183145	-0.975784711	0.508463207	0.002836115	10.55176278
NM_002422	matrix metalloproteinase 3 (stromelysin 1, progelatinase) (MMP3)	MMP3	-2.892537216	-7.425752423	0.1403036	1.102137025	0.035970263	6.737594561
NM_004994	matrix metalloproteinase 9 (gelatinase B, 92kDa gelatinase, 92kDa type IV collagenase) (MMP9).	MMP9	-5.339938765	-40.50249185	0.582013181	1.496936663	0.000148469	27.05691753
NM_002430	meningioma (disrupted in balanced translocation) 1 (MN1)	MN1	-1.302346394	-2.466296742	-0.528871734	0.693096562	0.040720075	3.55837394
NM_005098	musculin (activated B-cell factor-1) (MSC)	MSC	-2.174718161	-4.514975507	0.65139125	1.570682139	0.0009309	2.874531641
NM_005947	metallothionein 1B (functional) (MT1B)	MT1B	-2.895163379	-7.439281962	-0.374526291	0.771358647	0.027385826	9.644387847
NM_175617	metallothionein 1E (functional) (MT1E)	MT1E	-2.998822863	-7.993475227	0.195086473	1.144792775	0.00144665	6.982464773
NM_005949	metallothionein 1F (functional) (MT1F)	MT1F	-2.721564231	-6.595875792	0.29247303	1.224737893	0.000776159	5.385540717
NM_015440	methylenetetrahydrofolate dehydrogenase (NADP+ dependent) 1-like (MTHFD1L).	MTHFD1L	-1.747444315	-3.357632458	-0.121557692	0.919194651	0.019490698	3.652798081
NM_006636	methylenetetrahydrofolate dehydrogenase (NADP+ dependent) 2, methenyltetrahydrofolate cyclohydrolase (MTHFD2), nuclear gene encoding mitochondrial protein.	MTHFD2	-2.137828417	-4.400990986	-0.151898409	0.900065308	0.024582829	4.889635173
NM_002466	v-myb myeloblastosis viral oncogene homolog (avian)-like 2 (MYBL2).	MYBL2	-2.263563182	-4.801759618	0.628472259	1.545927068	0.006328699	3.106071249
NM_018946	N-acetylneuraminic acid synthase (sialic acid synthase) (NANS)	NANS	-1.805092318	-3.494515191	-0.588156011	0.665192584	0.011558284	5.253388682
NM_020170	nicalin homolog (zebrafish) (NCLN).	NCLN	-1.111534038	-2.16075281	-0.260689512	0.834688898	0.007872516	2.588692403
NM_002488	NADH dehydrogenase (ubiquinone) 1 alpha subcomplex, 2, 8kDa (NDUFA2)	NDUFA2	-1.586931304	-3.004096807	-0.070001219	0.952637193	0.003260529	3.153453203
NM_021075	NADH dehydrogenase (ubiquinone) flavoprotein 3, 10kDa (NDUFV3)	NDUFV3	-1.572519377	-2.974236515	-0.724783182	0.605087975	0.041130293	4.915378657
NM_014397	NIMA (never in mitosis gene a)-related kinase 6 (NEK6).	NEK6	-2.322395371	-5.001619719	-0.54887339	0.683553712	0.001390038	7.317083695

NM_000602	serine (or cysteine) proteinase inhibitor, clade E (nexin, plasminogen activator inhibitor type 1), member 1 (SERPINE1)	SERPINE1	-3.455514904	-10.97017704	-0.628784455	0.646721082	0.001668885	16.96276394
NM_006164	nuclear factor (erythroid-derived 2)-like 2 (NFE2L2).	NFE2L2	-1.446790273	-2.726008902	-0.32222961	0.799832822	0.014319176	3.408223353
NM_004289	nuclear factor (erythroid-derived 2)-like 3 (NFE2L3).	NFE2L3	-1.476155516	-2.782063821	0.058965495	1.041718514	0.004318222	2.670648342
XR_013712	Macaca mulatta Alpha-enolase (2-phospho-D-glycerate hydro-lyase) (Non-neural enolase) (NNE) (Enolase 1) (LOC717728)	NNE	-2.694532399	-6.473439247	-0.428200147	0.743188382	0.00671142	8.710361205
NM_024815	nudix (nucleoside diphosphate linked moiety X)-type motif 18 (NUDT18).	NUDT18	-1.51483587	-2.857663155	0.302044511	1.232890363	0.006851632	2.317856673
NM_006703	nudix (nucleoside diphosphate linked moiety X)-type motif 3 (NUDT3)	NUDT3	-1.088601714	-2.126678151	0.512901113	1.426916697	0.001153412	1.490401056
NM_030666	serine (or cysteine) proteinase inhibitor, clade B (ovalbumin), member 1 (SERPINB1).	SERPINB1	-2.719058933	-6.584431724	0.249189941	1.188539575	0.006088207	5.539934778
NM_004155	serine (or cysteine) proteinase inhibitor, clade B (ovalbumin), member 9 (SERPINB9)	SERPINB9	-2.293449624	-4.902268909	0.705074372	1.630228702	0.00432137	3.007105017
NM_148962	oxoeicosanoid (OXE) receptor 1 (OXER1)	OXER1	-2.157420826	-4.461165981	0.200189134	1.148848957	0.006028201	3.88316145
NM_000632	integrin, alpha M (complement component receptor 3, alpha; also known as CD11b (p170), macrophage antigen alpha polypeptide) (ITGAM).	p170	-2.36456182	-5.149962119	0.044007363	1.030973581	0.00832586	4.9952416
NM_000917	procollagen-proline, 2-oxoglutarate 4-dioxygenase (proline 4-hydroxylase), alpha polypeptide I (P4HA1)	P4HA1	-1.558620578	-2.945720559	-0.249218426	0.841352091	5.84E-05	3.501174587
NM_002568	poly(A) binding protein, cytoplasmic 1 (PABPC1)	PABPC1	-1.401494819	-2.6417516	-0.492500586	0.710792032	0.01489261	3.716630859
NM_152911	polyamine oxidase (exo-N4-amino) (PAOX), transcript variant 1.	PAOX	-1.811506615	-3.510086576	-0.242591989	0.84522539	0.029018147	4.152840907
NM_014982	pecanex homolog (Drosophila) (PCNX)	PCNX	-1.860914615	-3.632378684	-0.1406048	0.907138789	0.0415689	4.004214932
NM_213636	PDZ and LIM domain 7 (enigma) (PDLIM7), transcript variant 4	PDLIM7	-1.568614221	-2.966196596	0.278444257	1.212886252	0.001389193	2.445568652
NM_002629	phosphoglycerate mutase 1 (brain) (PGAM1).	PGAM1	-2.128452188	-4.372481222	-1.176629066	0.442383945	0.009633802	9.883905755
XM_496526	phosphatidylinositol (4,5) bisphosphate 5-phosphatase, A (PIB5PA)	PIB5PA	-2.50521295	-5.677331369	0.135728821	1.098647689	0.003313708	5.167563202
NM_003706	phospholipase A2, group IVC (cytosolic, calcium-independent) (PLA2G4C).	PLA2G4C	-1.738433139	-3.336725808	-0.209551299	0.864806158	0.000221699	3.85835112
NM_005084	phospholipase A2, group VII (platelet-activating factor acetylhydrolase, plasma) (PLA2G7).	PLA2G7	-1.914653434	-3.770232346	0.089401522	1.063928738	0.006562794	3.543688793
NM_002661	phospholipase C, gamma 2 (phosphatidylinositol-specific) (PLCG2).	PLCG2	-1.371707461	-2.587766527	-0.047965425	0.967299511	0.001558983	2.675248459
NM_017958	pleckstrin homology domain containing, family B (evectins) member 2 (PLEKHB2)	PLEKHB2	-3.908507516	-15.01682088	0.383155673	1.304191456	0.004595932	11.51427639
NM_024310	pleckstrin homology domain containing, family F (with FYVE domain) member 1 (PLEKHF1).	PLEKHF1	-1.426173426	-2.687329876	-0.204137436	0.86805753	0.000843971	3.095796976
NM_024613	pleckstrin homology domain containing, family F (with FYVE domain) member 2 (PLEKHF2)	PLEKHF2	-1.671330275	-3.18508147	-0.066702047	0.954818188	0.04184043	3.335798911
NM_014798	pleckstrin homology domain containing, family M (with RUN domain) member 1 (PLEKHM1)	PLEKHM1	-1.644308657	-3.125980237	-0.905259806	0.533936542	0.039208022	5.854591307
NM_021173	polymerase (DNA-directed), delta 4 (POLD4)	POLD4	-1.204468998	-2.304524328	-0.349038715	0.78510705	0.012499896	2.935299496

NM_203290	polymerase (RNA) I polypeptide C, 30kDa (POLR1C), transcript variant 1.	POLR1C	-1.771737149	-3.41464867	-0.166351495	0.891093365	0.01345027	3.831976314
NM_006627	processing of precursor 4, ribonuclease P/MRP subunit (S. cerevisiae) (POP4).	POP4	-2.115902955	-4.334612283	-0.174093707	0.886324128	0.004581966	4.890549795
NM_021129	pyrophosphatase (inorganic) (PP)	PP	-2.420690121	-5.354270851	0.054658278	1.038613064	0.015862834	5.155212307
NM_005729	peptidylprolyl isomerase F (cyclophilin F) (PPIF), nuclear gene encoding mitochondrial protein	PPIF	-1.809828054	-3.506005001	0.357613096	1.281304256	0.005244786	2.736278277
NM_006347	peptidyl prolyl isomerase H (cyclophilin H) (PPIH)	PPIH	-1.443279363	-2.719383013	0.0221689	1.01548498	0.002537814	2.677915545
NM_000310	palmitoyl-protein thioesterase 1 (ceroid-lipofuscinosis, neuronal 1, infantile) (PPT1).	PPT1	-1.666457668	-3.174342214	-0.599558428	0.65995592	0.025429213	4.809930661
NM_199418	prolylcarboxypeptidase (angiotensinase C) (PRCP), transcript variant 2	PRCP	-2.682048451	-6.417664856	0.003181648	1.002207784	0.011384941	6.40352725
NM_006404	protein C receptor, endothelial (EPCR) (PROCR).	PROCR	-2.311909149	-4.96539727	0.444833635	1.361157145	0.015803163	3.647923597
NM_002775	protease, serine, 11 (IGF binding) (PRSS11)	PRSS11	-1.731637531	-3.321045597	0.110885725	1.079891019	0.0016324	3.075352549
NM_172341	presenilin enhancer 2 homolog (C. elegans) (PSENEN).	PSENEN	-1.712834804	-3.278043056	-0.384910024	0.765826759	0.007869582	4.280397645
NM_148976	proteasome (prosome, macropain) subunit, alpha type, 1 (PSMA1), transcript variant 1	PSMA1	-1.931084701	-3.813418062	-0.821352021	0.565911351	0.009440585	6.738543164
NM_002791	proteasome (prosome, macropain) subunit, alpha type, 6 (PSMA6)	PSMA6	-1.285568984	-2.437781774	0.144502284	1.105349252	0.001602912	2.205440289
NM_002801	proteasome (prosome, macropain) subunit, beta type, 10 (PSMB10).	PSMB10	-2.748723193	-6.721220306	-0.141141721	0.906801246	0.001107395	7.412010439
NM_002795	proteasome (prosome, macropain) subunit, beta type, 3 (PSMB3)	PSMB3	-1.671654856	-3.185798138	1.528175658	2.884208893	0.002085813	1.104565673
NM_002803	proteasome (prosome, macropain) 26S subunit, ATPase, 2 (PSMC2)	PSMC2	-1.274355068	-2.418906606	-0.169424746	0.889197165	0.014215247	2.720326493
NM_002815	proteasome (prosome, macropain) 26S subunit, non-ATPase, 11 (PSMD11)	PSMD11	-1.259823427	-2.394664305	0.023868324	1.016681875	0.001593627	2.355372278
NM_000958	prostaglandin E receptor 4 (subtype EP4) (PTGER4).	PTGER4	-1.522752471	-2.873387311	0.116490065	1.084094157	0.000338136	2.650496078
NM_007284	PTK9L protein tyrosine kinase 9-like (A6-related protein) (PTK9L)	PTK9L	-1.51450611	-2.857010048	0.03008527	1.021072474	0.000240615	2.798048248
NM_002863	phosphorylase, glycogen; liver (Hers disease, glycogen storage disease type VI) (PYGL)	PYGL	-1.947913627	-3.858161746	-0.260917772	0.834556846	2.11E-05	4.623006524
NM_198679	Rap guanine nucleotide exchange factor (GEF) 1 (RAPGEF1), transcript variant 2	RAPGEF1	-1.175547811	-2.258786339	-0.321585469	0.800190015	0.006627319	2.822812454
XM_376350	Rap guanine nucleotide exchange factor (GEF) 2 (RAPGEF2)	RAPGEF2	-1.896218428	-3.722362165	-0.199516728	0.870842228	0.001987052	4.274439209
NM_032023	Ras association (RalGDS/AF-6) domain family 4 (RASSF4), transcript variant 1.	RASSF4	-1.554793922	-2.937917578	0.561577129	1.475881744	0.000390786	1.990618551
NM_006509	v-rel reticuloendotheliosis viral oncogene homolog B, nuclear factor of kappa light polypeptide gene enhancer in B-cells 3 (avian) (RELB)	RELB	-1.7560759	-3.37778125	0.094978568	1.068049541	0.001590503	3.162569824
NM_024599	rhomboid, veinlet-like 6 (Drosophila) (RHBDL6)	RHBDL6	-2.381425563	-5.210513519	0.105446837	1.075827548	0.008716601	4.843260919
NM_001005498	rhomboid, veinlet-like 6 (Drosophila) (RHBDL6), transcript variant 2.	RHBDL6	-3.274701369	-9.677949219	-0.280804267	0.823132014	0.000875298	11.7574691
NM_198232	ribonuclease, RNase A family, 1 (pancreatic) (RNASE1), transcript variant 3	RNASE1	-1.32740543	-2.50950954	-0.064432919	0.956321146	0.002053124	2.624128465

NM_002935	ribonuclease, RNase A family, 3 (eosinophil cationic protein) (RNASE3).	RNASE3	-3.066068617	-8.37488057	-0.298481199	0.813107948	0.002501319	10.29983853
NM_144563	ribose 5-phosphate isomerase A (ribose 5-phosphate epimerase) (RPIA).	RPIA	-1.691724749	-3.23042672	-0.403033064	0.756266666	0.043365864	4.271544503
NM_016539	sirtuin (silent mating type information regulation 2 homolog) 6 (S. cerevisiae) (SIRT6).	SIRT6	-1.141092397	-2.205479571	-0.447675046	0.733223512	0.025179395	3.007922597
NM_001007067	syndecan binding protein (syntenin) (SDCBP), transcript variant 2.	SDCBP	-1.990238331	-3.973026265	0.433215733	1.350239878	0.005918482	2.942459581
NM_004892	SEC22 vesicle trafficking protein-like 1 (S. cerevisiae) (SEC22L1)	SEC22L1	-1.147724529	-2.215641596	0.176705376	1.130299716	0.023461444	1.960224853
NM_013336	Sec61 alpha 1 subunit (S. cerevisiae) (SEC61A1)	SEC61A1	-1.088156857	-2.126022488	0.183281087	1.135463308	0.000699144	1.872383258
NM_021807	SEC8-like 1 (S. cerevisiae) (SEC8L1)	SEC8L1	-1.474022608	-2.777953804	0.189206738	1.140136642	0.016500391	2.436509538
NM_004844	SH3-domain binding protein 5 (BTK-associated) (SH3BP5)	SH3BP5	-1.629837113	-3.094780551	-0.004102685	0.997160275	0.019953905	3.103593904
NM_005067	seven in absentia homolog 2 (Drosophila) (SIAH2)	SIAH2	-2.47935089	-5.576465088	0.380949131	1.302198273	0.000107834	4.282347168
NM_003051	solute carrier family 16 (monocarboxylic acid transporters), member 1 (SLC16A1).	SLC16A1	-3.085898723	-8.490789598	-0.062518344	0.957591105	0.049262628	8.866821711
NM_006516	solute carrier family 2 (facilitated glucose transporter), member 1 (SLC2A1).	SLC2A1	-2.943948935	-7.695147257	0.446537059	1.362765245	0.008198685	5.646715226
NM_017585	solute carrier family 2 (facilitated glucose transporter), member 6 (SLC2A6)	SLC2A6	-2.102000863	-4.293043713	0.178087229	1.131382867	0.008687811	3.794510097
NM_003486	solute carrier family 7 (cationic amino acid transporter, y+ system), member 5 (SLC7A5).	SLC7A5	-4.021712947	-16.24262549	0.368353239	1.290878519	0.000555646	12.58261351
NM_138440	slit-like 2 (Drosophila) (SLITL2).	SLITL2	-1.324973608	-2.505283046	0.062833184	1.044514981	0.007924876	2.398513274
NM_000543	sphingomyelin phosphodiesterase 1, acid lysosomal (acid sphingomyelinase) (SMPD1), transcript variant 1.	SMPD1	-2.005543202	-4.015398583	-0.598271628	0.660544825	0.003536023	6.078919146
NM_003121	Spi-B transcription factor (Spi-1/PU.1 related) (SPIB)	SPIB	-2.251736182	-4.762556413	-0.047193876	0.967816959	0.02121193	4.920926803
NM_000582	secreted phosphoprotein 1 (osteopontin, bone sialoprotein I, early T lymphocyte activation 1) (SPP1)	SPP1	-2.842299016	-7.171619846	-0.040807297	0.972110826	0.007214029	7.377368562
NM_003124	sepiapterin reductase (7,8-dihydrobiopterin:NADP+ oxidoreductase) (SPR).	SPR	-2.020698513	-4.057802118	0.144220251	1.105133189	0.034777597	3.671776541
NM_021199	sulfide quinone reductase-like (yeast) (SQRDL)	SQRDL	-2.189558486	-4.561658626	-0.485632865	0.714183711	0.002369892	6.387234202
NM_033389	slingshot homolog 2 (Drosophila) (SSH2)	SSH2	-1.382852388	-2.607834631	-0.036613777	0.974940596	0.005957812	2.674865157
NM_021978	suppression of tumorigenicity 14 (colon carcinoma, matriptase, epithin) (ST14).	ST14	-1.277594192	-2.424343612	-0.317991608	0.802185833	0.01052691	3.022172062
NM_000609	chemokine (C-X-C motif) ligand 12 (stromal cell-derived factor 1) (CXCL12).	CXCL12	-1.551078703	-2.930361604	0.008582247	1.00596649	0.01017099	2.912981331
NM_133439	transcriptional adaptor 2 (ADA2 homolog, yeast)-like (TADA2L), transcript variant 2.	TADA2L	-2.391872792	-5.248382226	-0.036196063	0.97522292	0.016418012	5.381725676
NM_004606	TAF1 RNA polymerase II, TATA box binding protein (TBP)-associated factor, 250kDa (TAF1), transcript variant 1	TAF1	-1.423988485	-2.683263034	-0.505960357	0.704191463	0.006878908	3.810416873
NM_000593	transporter 1, ATP-binding cassette, sub-family B (MDR/TAP) (TAP1).	TAP1	-1.888590909	-3.702733994	0.197112315	1.146401431	0.001897572	3.229875587
NM_014604	Tax1 (human T-cell leukemia virus type I) binding protein 3 (TAX1BP3)	TAX1BP3	-1.130117306	-2.188765365	-0.594135228	0.662441413	0.030752116	3.304088968
NM_001061	thromboxane A synthase 1 (platelet, cytochrome P450, family 5, subfamily A) (TBXAS1), transcript variant TXS-I.	TBXAS1	-1.474265014	-2.778420603	-0.127016266	0.915723362	0.049249803	3.034126593

NM_000660	transforming growth factor, beta 1 (Camurati-Engelmann disease) (TGFB1)	TGFB1	-2.232786587	-4.700409948	-0.50737696	0.703500347	0.010212522	6.681460739
XR_009902	Macaca mulatta Trans-Golgi network integral membrane protein 2 precursor (Trans-Golgi network protein TGN51) (TGN46) (TGN48) (TGN38 homolog) (LOC694942)	TGN46	-1.751717021	-3.367591209	0.14289597	1.104119228	0.000543678	3.050024964
NM_012456	translocase of inner mitochondrial membrane 10 homolog (yeast) (TIMM10)	TIMM10	-1.269579843	-2.410913421	-0.140502331	0.907203222	0.012127273	2.657522993
NM_012458	translocase of inner mitochondrial membrane 13 homolog (yeast) (TIMM13), nuclear gene encoding mitochondrial protein	TIMM13	-1.577099765	-2.983694374	-0.459499547	0.727238485	0.03556579	4.102772936
NM_003254	tissue inhibitor of metalloproteinase 1 (erythroid potentiating activity, collagenase inhibitor) (TIMP1)	TIMP1	-2.939836522	-7.673243415	-0.115442549	0.923099105	0.006392561	8.312480613
NM_022367	sema domain, immunoglobulin domain (Ig), transmembrane domain (TM) and short cytoplasmic domain, (semaphorin) 4A (SEMA4A).	SEMA4A	-2.799252172	-6.960795407	-0.837380922	0.559658655	0.017756569	12.43757305
NM_017789	sema domain, immunoglobulin domain (Ig), transmembrane domain (TM) and short cytoplasmic domain, (semaphorin) 4C (SEMA4C).	SEMA4C	-1.430651435	-2.69568409	-0.179593572	0.882951702	0.019019427	3.053036859
NM_006378	sema domain, immunoglobulin domain (Ig), transmembrane domain (TM) and short cytoplasmic domain, (semaphorin) 4D (SEMA4D)	SEMA4D	-1.774431833	-3.421032554	-0.088062695	0.940785223	0.003596153	3.636358726
NM_003272	transmembrane 7 superfamily member 1 (upregulated in kidney) (TM7SF1).	TM7SF1	-1.124217864	-2.17983338	0.00861972	1.005992619	0.011820291	2.166848284
NM_006573	tumor necrosis factor (ligand) superfamily, member 13b (TNFSF13B).	TNFSF13B	-2.396590236	-5.265571911	0.70091052	1.625530381	0.001678075	3.239294678
NM_000546	tumor protein p53 (Li-Fraumeni syndrome) (TP53)	TP53	-1.860096009	-3.630318205	-0.045354382	0.969051751	0.016007514	3.74625834
NM_012112	TPX2, microtubule-associated, homolog (Xenopus laevis) (TPX2).	TPX2	-1.231504154	-2.348116769	0.437739784	1.354480651	0.008073713	1.733591963
XR_011552	Macaca mulatta Triosephosphate isomerase (TIM) (Triose-phosphate isomerase) (TPI1)	TPI1	-1.769445994	-3.409230144	-0.345648611	0.786954096	0.002383575	4.332184256
NM_152653	ubiquitin-conjugating enzyme E2E 2 (UBC4/5 homolog, yeast) (UBE2E2)	UBE2E2	-2.381003421	-5.208989109	0.033318241	1.023363186	0.002619559	5.090068881
NM_003342	ubiquitin-conjugating enzyme E2G 1 (UBC7 homolog, C. elegans) (UBE2G1), transcript variant 1.	UBE2G1	-2.338608633	-5.058145834	-0.499201832	0.707498095	0.023881185	7.149341984
NM_006649	UTP14, U3 small nucleolar ribonucleoprotein, homolog A (yeast) (UTP14A)	UTP14A	-1.282094255	-2.431917445	1.117796772	2.170153019	0.011982864	1.120620262
NM_006634	vesicle-associated membrane protein 5 (myobrevin) (VAMP5)	VAMP5	-1.175691101	-2.259010696	-0.293441158	0.815953499	0.003628993	2.768553229
NM_000376	vitamin D (1,25- dihydroxyvitamin D3) receptor (VDR), transcript variant 1.	VDR	-4.400370607	-21.11755067	-0.361534296	0.778336384	0.027812776	27.1316504
NM_016226	vacuolar protein sorting 29 (yeast) (VPS29), transcript variant 1.	VPS29	-1.31479627	-2.487671985	-0.154869802	0.89821343	0.003659546	2.769577811
NM_022916	vacuolar protein sorting 33A (yeast) (VPS33A).	VPS33A	-2.108075989	-4.31115965	-0.255696922	0.837582426	0.004432745	5.147146734
NM_000377	Wiskott-Aldrich syndrome (eczema-thrombocytopenia) (WAS)	WAS	-1.557287587	-2.943000092	-0.432614769	0.740917714	0.017607074	3.972101135
NM_006963	zinc finger protein 22 (KOX 15) (ZNF22)	ZNF22	-1.328501153	-2.511416229	0.043008862	1.030260282	0.004380368	2.437652186

NM_016817	2'-5'-oligoadenylate synthetase 2, 69/71kDa (OAS2), transcript variant 1	OAS2	-1.443615849	-2.720017341	-0.01349749	0.990687882	0.001518661	2.745584549
NM_003733	2'-5'-oligoadenylate synthetase-like (OASL), transcript variant 1.	OASL	-1.93085243	-3.812804158	0.104212136	1.074907217	0.005568711	3.547100714
NM_005443	3'-phosphoadenosine 5'-phosphosulfate synthase 1 (PAPSS1).	PAPSS1	-1.703670881	-3.257287089	0.576662254	1.491394839	0.012013359	2.184054151
NM_004567	6-phosphofructo-2-kinase/fructose-2,6-biphosphatase 4 (PFKFB4).	PFKFB4	-2.086110453	-4.246017898	0.640178854	1.558522361	0.011753676	2.724386897
NM_012088	6-phosphogluconolactonase (PGLS)	PGLS	-2.246038164	-4.743783483	-0.267449092	0.830787208	0.020536295	5.709986187
NM_001109	a disintegrin and metalloproteinase domain 8 (ADAM8).	ADAM8	-1.964639813	-3.903152451	-0.641609256	0.640997548	0.003850639	6.089184681
NM_005470	abl-interactor 1 (ABI1), transcript variant 1	ABI1	-1.484802824	-2.798789189	-0.084605142	0.943042607	0.019178675	2.967828992
NM_001610	acid phosphatase 2, lysosomal (ACP2).	ACP2	-2.385878067	-5.226619255	-0.308717031	0.807359414	0.007905519	6.473720581
NM_001611	acid phosphatase 5, tartrate resistant (ACP5).	ACP5	-1.806572821	-3.498103124	-0.35871667	0.779857983	0.00734059	4.485564294
NM_005720	actin related protein 2/3 complex, subunit 1B, 41kDa (ARPC1B)	ARPC1B	-2.020059702	-4.056005762	-0.19079623	0.876122052	0.006379596	4.629498541
NM_005718	actin related protein 2/3 complex, subunit 4, 20kDa (ARPC4), transcript variant 1.	ARPC4	-2.161023358	-4.472319814	0.02296603	1.016046219	0.009976937	4.401689344
NM_001102	actinin, alpha 1 (ACTN1)	ACTN1	-1.325324179	-2.505891897	0.175967283	1.129721595	0.004127628	2.218149949
NM_004024	activating transcription factor 3 (ATF3)	ATF3	-1.446125834	-2.724753716	-0.363230752	0.777421681	0.001987317	3.504859437
NM_013375	activator of basal transcription 1 (ABT1).	ABT1	-1.558531426	-2.945538533	-0.33021675	0.795416972	0.007291528	3.703137647
NM_014049	acyl-Coenzyme A dehydrogenase family, member 9 (ACAD9).	ACAD9	-1.680170928	-3.204659171	0.432942155	1.349983856	0.032892097	2.373849995
NM_000017	acyl-Coenzyme A dehydrogenase, C-2 to C-3 short chain (ACADS), nuclear gene encoding mitochondrial protein.	ACADS	-1.10660704	-2.153386135	-0.356530217	0.781040781	0.011865003	2.757072599
NM_014479	ADAM-like, decysin 1 (ADAMDEC1).	ADAMDEC1	-3.559594111	-11.79083603	-0.078169252	0.947258935	0.023420623	12.44732099
NM_018090	adaptin-ear-binding coat-associated protein 2 (NECAP2).	NECAP2	-1.269441313	-2.410681933	-0.364853948	0.776547485	0.002205125	3.104358689
NM_020979	adaptor protein with pleckstrin homology and src homology 2 domains (APS)	APS	-1.324302462	-2.504117852	-0.112282331	0.925123367	0.004954676	2.706793431
NM_001127	adaptor-related protein complex 1, beta 1 subunit (AP1B1), transcript variant 1	AP1B1	-1.214556518	-2.320694342	-0.24721378	0.842521974	0.029767918	2.754461501
NM_057089	adaptor-related protein complex 1, sigma 1 subunit (AP1S1), transcript variant 2.	AP1S1	-1.97343916	-3.927031471	0.252677619	1.191416314	0.000154821	3.296103491
NM_004069	adaptor-related protein complex 2, sigma 1 subunit (AP2S1), transcript variant AP17.	AP2S1	-1.423435896	-2.682235472	0.126818365	1.091883069	0.001131028	2.456522632
NM_000676	adenosine A2b receptor (ADORA2B).	ADORA2B	-1.328066714	-2.51066008	0.324691646	1.25239673	0.033690746	2.004684315
NM_020683	adenosine A3 receptor (ADORA3), transcript variant 1.	ADORA3	-1.955489995	-3.878476325	0.943843879	1.923646731	0.005098974	2.016210286
NM_001122	adipose differentiation-related protein (ADFP)	ADFP	-2.105267006	-4.302773822	0.108445472	1.078065976	0.001688913	3.991197123
NM_015161	ADP-ribosylation factor-like 6 interacting protein (ARL6IP).	ARL6IP	-2.532484856	-5.785673281	0.214958188	1.160670276	0.020356224	4.984769061
NM_001124	adrenomedullin (ADM)	ADM	-1.930628415	-3.81221217	-0.351296159	0.783879521	0.021248198	4.863262871
CB229722	AGENCOURT_11500965 NICHD_Rh_Ov1 Macaca mulatta cDNA clone IMAGE:6882443 5' sequence		-1.478010046	-2.785642358	-0.238358628	0.847709213	0.002845034	3.286082437
CB311278	AGENCOURT_11616400 NICHD_Rh_Ov1 Macaca mulatta cDNA clone IMAGE:6915394 5' sequence		-1.214931015	-2.321296829	0.039872935	1.02802328	0.001343739	2.258019711
CB308778	AGENCOURT_11771972 NICHD_Rh_Ov1 Macaca mulatta cDNA clone IMAGE:6884883 5' sequence		-1.217527695	-2.325478651	0.039189626	1.027536489	0.000991865	2.263159194

CB311553	AGENCOURT_11823216 NICHD_Rh_Ov1 Macaca mulatta cDNA clone IMAGE:6912989 5' sequence		-1.449316897	-2.730787204	0.229713399	1.172601981	0.001247031	2.328827043
CB309708	AGENCOURT_11828410 NICHD_Rh_Ov1 Macaca mulatta cDNA clone IMAGE:6913568 5' sequence		-1.279624514	-2.427757821	-0.496261856	0.70894133	0.029616514	3.424483407
CB310604	AGENCOURT_11830389 NICHD_Rh_Ov1 Macaca mulatta cDNA clone IMAGE:6916324 5' sequence		-1.986812125	-3.96360206	-0.430922968	0.741787073	0.003199309	5.343315089
CB310061	AGENCOURT_11876913 NICHD_Rh_Ov1 Macaca mulatta cDNA clone IMAGE:6913844 5' sequence		-1.623124239	-3.080413957	-0.246343234	0.84303052	0.039446019	3.653976797
NM_000689	aldehyde dehydrogenase 1 family, member A1 (ALDH1A1)	ALDH1A1	-1.510852491	-2.849783835	-0.499022466	0.707586061	0.003633267	4.02747311
NM_000693	aldehyde dehydrogenase 1 family, member A3 (ALDH1A3).	ALDH1A3	-1.387698737	-2.616609687	0.217115723	1.162407344	0.009193673	2.251026459
NM_000034	aldolase A, fructose-bisphosphate (ALDOA), transcript variant 1	ALDOA	-1.399136607	-2.637436952	-0.65277354	0.636056338	0.014905358	4.146546142
NM_005165	aldolase C, fructose-bisphosphate (ALDOC)	ALDOC	-1.507966711	-2.844089195	0.221350591	1.165824472	0.001694392	2.439551805
NM_000478	alkaline phosphatase, liver/bone/kidney (ALPL)	ALPL	-1.269717632	-2.411143694	0.246332172	1.186187581	0.012834381	2.032683307
NM_004847	allograft inflammatory factor 1 (AIF1), transcript variant 2.	AIF1	-2.479574039	-5.577327695	-0.506209965	0.704069638	0.002714929	7.921556891
NM_020350	angiotensin II receptor-associated protein (AGTRAP).	AGTRAP	-1.406144535	-2.650279536	0.313790307	1.242968992	0.013605329	2.132216936
NM_005161	angiotensin II receptor-like 1 (AGTRL1)	AGTRL1	-2.367356574	-5.159948168	0.082005099	1.058488136	0.001681577	4.874828533
NM_152326	ankyrin repeat domain 9 (ANKRD9).	ANKRD9	-1.886858517	-3.698290408	0.150730758	1.110131636	0.000221189	3.331398085
NM_001645	apolipoprotein C-I (APOC1).	APOC1	-1.737694201	-3.335017198	0.089777569	1.064206093	0.002986759	3.133807651
NM_001646	apolipoprotein C-IV (APOC4).	APOC4	-1.685172728	-3.215788953	-0.503090491	0.705593663	0.025414119	4.557564957
NM_003661	apolipoprotein L, 1 (APOL1), transcript variant 1.	APOL1	-2.878387522	-7.353277965	-0.081848687	0.944846133	0.001173208	7.782513689
NM_030643	apolipoprotein L, 4 (APOL4), transcript variant a	APOL4	-1.777017412	-3.427169182	0.105470806	1.075845422	0.001137008	3.185559107
NM_006010	arginine-rich, mutated in early stage tumors (ARMET)	ARMET	-1.302703763	-2.466907744	-0.017083634	0.988228362	0.034329274	2.496293204
NM_054012	argininosuccinate synthetase (ASS), transcript variant 2	ASS	-2.61201828	-6.113583546	1.043653557	2.061441552	0.02931294	2.965683669
NM_183376	arrestin domain containing 4 (ARRDC4)	ARRDC4	-1.732495022	-3.323020107	-0.557693234	0.679387588	0.005365829	4.891199319
NM_025080	asparaginase like 1 (ASRGL1)	ASRGL1	-1.961481379	-3.894616784	-0.344763604	0.787436993	0.010981109	4.945940839
NM_014010	astrotactin 2 (ASTN2), transcript variant 1	ASTN2	-1.960919673	-3.893100729	0.224590888	1.16844586	0.002443912	3.331862316
NM_006886	ATP synthase, H+ transporting, mitochondrial F1 complex, epsilon subunit (ATP5E), nuclear gene encoding mitochondrial protein	ATP5E	-1.951373976	-3.867426774	-0.383117467	0.766778895	0.000497257	5.043731381
NM_004691	ATPase, H+ transporting, lysosomal 38kDa, V0 subunit d isoform 1 (ATP6V0D1).	ATP6V0D1	-2.185968913	-4.550322862	-0.870893432	0.546808118	0.004103002	8.321608089
NM_001693	ATPase, H+ transporting, lysosomal 56/58kDa, V1 subunit B, isoform 2 (ATP6V1B2).	ATP6V1B2	-2.095616642	-4.274088089	-0.563495483	0.676660705	0.008147699	6.316441986
NM_001690	ATPase, H+ transporting, lysosomal 70kDa, V1 subunit A (ATP6V1A)	ATP6V1A	-2.614570703	-6.124409301	0.13584508	1.098736226	0.001283518	5.574048762
NM_004217	aurora kinase B (AURKB)	AURKB	-1.251946883	-2.381626011	0.272121978	1.207582687	0.011929561	1.972226033
NM_015892	B cell RAG associated protein (GALNAC4S-6ST).	GALNAC4S-6ST	-2.868053268	-7.300793467	-0.914103101	0.530673679	0.007347991	13.75759484
NM_001165	baculoviral IAP repeat-containing 3 (BIRC3), transcript variant 1	BIRC3	-2.154423902	-4.45190838	0.118897407	1.085904632	0.004861786	4.099723168
NM_006399	basic leucine zipper transcription factor, ATF-like (BATF)	BATF	-2.632700507	-6.201858056	0.207342539	1.154559512	0.000909282	5.371622678
NM_005178	B-cell CLL/lymphoma 3 (BCL3)	BCL3	-1.951051647	-3.866562805	0.101857605	1.07315436	0.004208093	3.602988487

NM_201594	B-cell linker; B cell linker protein (LOC284948), transcript variant 2.	LOC284948	-1.509362262	-2.846841678	-0.524912222	0.695001394	0.042010189	4.096166857
NM_013979	BCL2/adenovirus E1B 19kDa interacting protein 1 (BNIP1), transcript variant BNIP1-b	BNIP1	-1.216593393	-2.323973138	0.094025354	1.067344095	0.00636809	2.17734201
NM_001188	BCL2-antagonist/killer 1 (BAK1).	BAK1	-1.64273709	-3.122576875	0.425660676	1.343187469	0.014318553	2.324751345
NM_138764	BCL2-associated X protein (BAX), transcript variant epsilon	BAX	-1.658988866	-3.157951176	0.053216586	1.037575692	0.01734191	3.043586314
NM_001710	B-factor, properdin (BF)	BF	-1.507432327	-2.843035921	-0.020312515	0.986019091	0.008327375	2.883347743
NM_004335	bone marrow stromal cell antigen 2 (BST2).	BST2	-2.63378405	-6.206517743	0.410645392	1.329280337	0.000410624	4.669081135
NM_015399	breast cancer metastasis suppressor 1 (BRMS1), transcript variant 1.	BRMS1	-1.253680531	-2.384489667	-0.79000481	0.578342164	0.004331255	4.122973934
NM_004053	bystin-like (BYSL)	BYSL	-1.291329083	-2.447534308	-0.112956839	0.924690942	0.004263626	2.646867399
NM_198594	C1q and tumor necrosis factor related protein 1 (C1QTNF1)	C1QTNF1	-3.295105943	-9.815800608	-0.167270523	0.8905259	0.002634257	11.02247627
AY966403	Callithrix jacchus cytosolic glutathione peroxidase (Gpx-1) mRNA, complete cds	Gpx-1 mRNA	-1.315762726	-2.489339027	-0.028144494	0.980680779	0.000181809	2.538378523
NM_001219	calumenin (CALU).	CALU	-1.262160415	-2.39854651	-0.18531987	0.879454065	0.008675658	2.727313008
NM_001216	carbonic anhydrase IX (CA9)	CA9	-2.679193152	-6.404975942	-0.439077565	0.737606071	0.005225579	8.683464244
NM_001218	carbonic anhydrase XII (CA12), transcript variant 1.	CA12	-2.034083713	-4.095625245	0.520434198	1.43438688	0.006599307	2.855314212
NM_003652	carboxypeptidase Z (CPZ)	CPZ	-2.053309574	-4.150570282	0.297929868	1.229379103	0.021641345	3.376151644
NM_052889	CARD only protein (COPl), transcript variant 2.	COPl	-1.298210158	-2.459235947	0.189194641	1.140127082	0.003079292	2.156984064
NM_032415	caspase recruitment domain family, member 11 (CARD11)	CARD11	-1.209044536	-2.311844779	0.301556902	1.232473735	0.012284975	1.875776102
NM_147780	cathepsin B (CTSB), transcript variant 2	CTSB	-3.192267055	-9.140461793	-0.214127652	0.862067264	3.39E-05	10.6029566
NM_001814	cathepsin C (CTSC), transcript variant 1	CTSC	-1.958369287	-3.886224614	-0.330837093	0.795075025	0.004524263	4.887871576
NM_148170	cathepsin C (CTSC), transcript variant 2.	CTSC	-2.788267869	-6.907998974	0.134205617	1.097488343	4.99E-05	6.294371158
NM_004079	cathepsin S (CTSS)	CTSS	-1.35910296	-2.565256272	-0.066436821	0.954993739	0.000241836	2.686149834
NM_133467	Cbp/p300-interacting transactivator, with Glu/Asp-rich carboxy-terminal domain, 4 (CITED4)	CITED4	-3.428903639	-10.7696812	0.141380265	1.102959842	0.001393635	9.7643457
NM_000591	CD14 antigen (CD14).	CD14	-2.54757578	-5.846510394	-0.206271445	0.866774463	0.001202048	6.745134567
NM_014143	CD274 antigen (CD274).	CD274	-3.694965793	-12.9507684	0.217138612	1.162425786	0.003791097	11.14115718
NM_007261	CD300A antigen (CD300A).	CD300A	-1.549000698	-2.926143856	-0.215449289	0.861277896	0.000921031	3.397444506
NM_001774	CD37 antigen (CD37).	CD37	-1.672478341	-3.187617099	-0.3310512	0.794957038	0.012598131	4.009797947
NM_000560	CD53 antigen (CD53)	CD53	-1.515804469	-2.859582382	-0.68249516	0.623086706	0.001177059	4.589381151
BC078669	CD6 antigen (cDNA clone MGC:88709 IMAGE:6302947), complete cds	cDNA clone MGC:88709 IMAGE:6302947	-1.851483286	-3.608710188	0.543215783	1.457217053	0.002099133	2.476439719
NM_001251	CD68 antigen (CD68).	CD68	-2.904250942	-7.486290033	0.082854039	1.059111176	0.002500447	7.068464765
NM_001827	CDC28 protein kinase regulatory subunit 2 (CKS2)	CKS2	-2.451312405	-5.469133979	-0.176537761	0.884823887	0.01496048	6.181042418
NM_020240	CDC42 small effector 2 (CDC42SE2).	CDC42SE2	-1.190675687	-2.282596237	0.125427778	1.09083113	0.000937009	2.092529424
AK094316	cDNA FLJ36997 fis, clone BRACE2007087, highly MAD2-like protein mRNA		-1.285235506	-2.437218347	-0.461089536	0.726437439	0.018548077	3.355028546
AK127395	cDNA FLJ45486 fis, clone BRTHA2002726		-1.481159236	-2.791729646	-0.16735544	0.890473486	0.020012792	3.135106986
NM_031299	cell division cycle associated 3 (CDCA3)	CDCA3	-1.407276964	-2.652360663	0.365869036	1.288657644	0.034719245	2.058235308
NM_080668	cell division cycle associated 5 (CDCA5)	CDCA5	-1.633092003	-3.101770624	0.154205305	1.112808469	0.002053756	2.787335565

NM_003851	cellular repressor of E1A-stimulated genes (CREG)	CREG	-3.150465496	-8.879420324	-1.173650658	0.443298179	0.021024873	20.03035596
NM_018404	centaurin, alpha 2 (CENTA2).	CENTA2	-2.341360175	-5.067802057	0.015583955	1.010860526	0.011571733	5.013354389
XM_375224	cervical cancer suppressor-1 (LOC400410)	LOC400410	-3.418484378	-10.69218187	0.13618358	1.098994053	0.029067401	9.729062541
NM_015703	CGI-96 protein (CGI-96).	CGI-96	-1.758780173	-3.384118701	0.241527738	1.182243933	0.002162288	2.862453853
NM_138410	chemokine-like factor super family 7 (CKLFSF7), transcript variant 1.	CKLFSF7	-1.681836561	-3.208361179	-0.101050258	0.932354006	0.020665967	3.441140551
NM_001289	chloride intracellular channel 2 (CLIC2)	CLIC2	-2.199780351	-4.594093921	0.335748126	1.26203168	0.004867485	3.640236606
NM_176812	chromatin modifying protein 4B (CHMP4B).	CHMP4B	-2.22354152	-4.670385122	-0.192294491	0.875212658	0.035318303	5.336286077
XM_370738	chromodomain helicase DNA binding protein 8 (CHD8)	CHD8	-1.242043003	-2.365332503	-0.5903508	0.664181388	0.021730347	3.561274897
NM_174896	chromosome 1 open reading frame 162 (C1orf162).	C1orf162	-2.326302828	-5.015184682	-0.513874816	0.700338927	0.008693541	7.161082285
NM_022338	chromosome 11 open reading frame 24 (C11orf24).	C11orf24	-1.832937106	-3.562616288	-0.078003791	0.947367581	0.009589409	3.760542748
NM_032490	chromosome 14 open reading frame 142 (C14orf142)	C14orf142	-1.25463984	-2.386075743	0.126843205	1.091901869	0.004935895	2.18524742
NM_004894	chromosome 14 open reading frame 2 (C14orf2).	C14orf2	-1.634998881	-3.105873091	0.090308179	1.064597571	0.009468243	2.917415159
NM_152260	chromosome 15 open reading frame 19 (C15orf19)	C15orf19	-1.553013477	-2.9342941	0.041247274	1.029003061	0.000525569	2.851589282
NM_023933	chromosome 16 open reading frame 24 (C16orf24).	C16orf24	-1.505729062	-2.839681376	-0.059609574	0.959523753	0.002327535	2.959469598
NM_032339	chromosome 17 open reading frame 37 (C17orf37).	C17orf37	-1.345894731	-2.541877898	-0.388646678	0.763845795	0.030018631	3.327736979
NM_019107	chromosome 19 open reading frame 10 (C19orf10)	C19orf10	-1.175669681	-2.258977156	0.102827656	1.073876179	0.001843419	2.103573205
NM_174983	chromosome 19 open reading frame 28 (C19orf28).	C19orf28	-1.646266663	-3.130225653	-0.062906853	0.957333266	0.002472383	3.269734547
NM_080757	chromosome 20 open reading frame 127 (C20orf127).	C20orf127	-2.508082876	-5.68863642	0.427799789	1.345180518	0.00147347	4.22890188
NM_213720	chromosome 22 open reading frame 16 (C22orf16)	C22orf16	-1.36183237	-2.57011403	0.118149277	1.085341667	0.00199283	2.368022999
NM_024053	chromosome 22 open reading frame 18 (C22orf18)	C22orf18	-1.256465738	-2.389097509	0.326278768	1.253775261	0.017219273	1.905522931
NM_178508	chromosome 6 open reading frame 1 (C6orf1), transcript variant 1	C6orf1	-1.261563455	-2.397554242	-0.180056783	0.882668255	0.005403748	2.716257472
NM_031452	chromosome 6 open reading frame 119 (C6orf119)	C6orf119	-2.161912433	-4.475076772	-0.132358921	0.912338484	0.029726586	4.905061938
NM_138408	chromosome 6 open reading frame 51 (C6orf51)	C6orf51	-1.794995604	-3.470144176	-0.041088457	0.971921394	0.006552329	3.570395915
NM_173562	chromosome 6 open reading frame 69 (C6orf69).	C6orf69	-1.461917735	-2.754743009	-0.101476386	0.932078658	0.001279408	2.955483408
NM_001001790	chromosome 9 open reading frame 105 (C9orf105).	C9orf105	-1.556365465	-2.941119625	0.22656037	1.170042042	0.003618106	2.513687132
NM_025159	chromosome X open reading frame 21 (CXorf21)	CXorf21	-2.92216856	-7.579846117	-0.366492321	0.775666113	0.009725432	9.772047527
NM_001906	chymotrypsinogen B1 (CTRB1).	CTRB1	-1.272446211	-2.415708222	0.233162818	1.175408974	0.007138364	2.05520655
NM_198390	c-Maf-inducing protein (CMIP), transcript variant C-mip	CMIP	-2.213033145	-4.636490327	-0.583970115	0.667125404	0.038381879	6.949953187
NM_016139	coiled-coil-helix-coiled-coil-helix domain containing 2 (CHCHD2)	CHCHD2	-1.439685327	-2.712616928	-0.296248521	0.814367267	0.013831994	3.330950346
XM_376876	coiled-coil-helix-coiled-coil-helix domain containing 2; 16.7kD protein; chromosome 7 open reading frame 17 (LOC401531)	LOC401531	-1.549872808	-2.927913248	-0.555220444	0.680553062	0.021376896	4.302255641
NM_000088	collagen, type I, alpha 1 (COL1A1)	COL1A1	-3.844235207	-14.36250218	-0.003100681	0.99785308	2.46E-05	14.39340367
NM_000089	collagen, type I, alpha 2 (COL1A2).	COL1A2	-2.196953849	-4.585102066	-0.33991626	0.79008717	0.000830118	5.803286319
NM_004369	collagen, type VI, alpha 3 (COL6A3), transcript variant 1	COL6A3	-1.730621601	-3.318707776	-0.237704083	0.848093903	0.000550289	3.913137171
NM_024027	collectin sub-family member 11 (COLEC11), transcript variant 1.	COLEC11	-1.614626698	-3.062323505	0.050633859	1.035719876	0.010302517	2.956710185
NM_015991	complement component 1, q subcomponent, alpha polypeptide (C1QA)	C1QA	-1.867338764	-3.648589298	-0.064167198	0.956497301	0.002207431	3.814531725
NM_000491	complement component 1, q subcomponent, beta polypeptide (C1QB)	C1QB	-3.309098302	-9.911464902	-0.768452376	0.587046881	0.003154079	16.88360032
NM_001734	complement component 1, s subcomponent (C1S), transcript variant 1	C1S	-1.502920152	-2.834157929	-0.095531681	0.935927264	0.000527019	3.028181825

NM_000063	complement component 2 (C2)	C2	-1.251186376	-2.380370883	-0.334538082	0.793038008	0.004594859	3.001584864
NM_000592	complement component 4B (C4B).	C4B	-2.046338489	-4.130563153	-0.028369119	0.9805281	0.00766739	4.212590289
NM_016511	C-type lectin-like receptor-1 (CLEC1)	CLEC1	-2.274043101	-4.8367672	0.260349897	1.197769165	0.00450534	4.038146366
NM_001238	cyclin E1 (CCNE1), transcript variant 1	CCNE1	-1.400962698	-2.640777402	0.073325337	1.052139016	0.007734545	2.509913007
NM_001761	cyclin F (CCNF).	CCNF	-2.728266637	-6.626589894	-0.035379857	0.975774809	0.019017165	6.79110573
NM_031476	cysteine-rich secretory protein LCCL domain containing 2 (CRISPLD2).	CRISPLD2	-1.473740999	-2.77741161	-0.243121652	0.844915136	0.017102107	3.287207784
NM_001785	cytidine deaminase (CDA)	CDA	-2.147414506	-4.430331051	-0.324040038	0.798829747	0.004758946	5.546026631
NM_153611	cytochrome b, ascorbate dependent 3 (CYBASC3).	CYBASC3	-2.133431537	-4.387598572	-0.389925706	0.763168904	0.007842425	5.749184154
NM_000101	cytochrome b-245, alpha polypeptide (CYBA).	CYBA	-2.034714217	-4.097415555	-0.469604595	0.722162497	0.00777944	5.673813822
NM_030579	cytochrome b5 outer mitochondrial membrane precursor (CYB5-M)	CYB5-M	-1.315431935	-2.488768319	-0.006042299	0.995820556	0.005053772	2.499213643
NM_004255	cytochrome c oxidase subunit Va (COX5A), nuclear gene encoding mitochondrial protein	COX5A	-1.802770045	-3.488894675	-0.049934879	0.965979931	0.009855516	3.611767246
NM_018947	cytochrome c, somatic (CYCS), nuclear gene encoding mitochondrial protein.	CYCS	-2.237040113	-4.714288708	-0.268376482	0.830253335	0.037914426	5.678132816
NM_000104	cytochrome P450, family 1, subfamily B, polypeptide 1 (CYP1B1).	CYP1B1	-4.555953131	-23.5222332	-0.16596459	0.891332373	0.000881099	26.38996846
NM_000784	cytochrome P450, family 27, subfamily A, polypeptide 1 (CYP27A1), nuclear gene encoding mitochondrial protein.	CYP27A1	-1.359577015	-2.566099329	-0.098314401	0.934123755	0.000565877	2.747065702
NM_015986	cytokine receptor-like factor 3 (CRLF3).	CRLF3	-2.185817968	-4.5498468	0.019819412	1.013832566	0.00337048	4.487769431
NM_025104	Dbf4-related factor 1 (DRF1), transcript variant 2.	DRF1	-3.167238083	-8.983253728	-0.391374111	0.762403099	0.045824347	11.78281376
NM_004946	dedicator of cytokinesis 2 (DOCK2)	DOCK2	-1.566465029	-2.961781124	-0.211183789	0.863828135	0.00700595	3.428669435
NM_000110	dihydropyrimidine dehydrogenase (DPYD).	DPYD	-2.356516021	-5.121321113	0.081749172	1.058300382	0.003319433	4.839194241
NM_015393	DKFZP564O0823 protein (DKFZP564O0823).	DKFZP564O0823	-1.636532241	-3.109175906	-0.265130775	0.832123303	0.001091741	3.736436531
NM_018973	dolichyl-phosphate mannosyltransferase polypeptide 3 (DPM3), transcript variant 1	DPM3	-2.013337979	-4.037152204	-0.673945763	0.626790073	0.004318097	6.440995759
NM_004414	Down syndrome critical region gene 1 (DSCR1), transcript variant 1	DSCR1	-1.783712768	-3.443111183	0.579141684	1.493960169	0.004296836	2.304687404
NM_014395	dual adaptor of phosphotyrosine and 3-phosphoinositides (DAPP1).	DAPP1	-1.540237223	-2.908423228	-0.124104684	0.9175733	0.016579722	3.169690344
NM_004418	dual specificity phosphatase 2 (DUSP2).	DUSP2	-1.371948385	-2.58819871	0.484475078	1.399076721	0.003552267	1.849933367
NM_001005360	dynamin 2 (DNM2), transcript variant 1.	DNM2	-1.447974472	-2.728247394	-0.451090856	0.731489542	0.007817166	3.72971483
NM_024329	EF hand domain containing 2 (EFHD2)	EFHD2	-4.970626255	-31.35505739	-1.039834348	0.486383318	0.009603302	64.4657336
NM_001974	egf-like module containing, mucin-like, hormone receptor-like 1 (EMR1).	EMR1	-1.506661878	-2.841518047	0.296831395	1.228443406	0.001761643	2.313104562
NM_030796	EGFR-coamplified and overexpressed protein (ECOP).	ECOP	-2.265534675	-4.808325873	-0.354671872	0.782047492	0.005828059	6.14838091
NM_012081	elongation factor, RNA polymerase II, 2 (ELL2)	ELL2	-2.214161784	-4.640118933	-0.586880975	0.665780732	0.040066449	6.969440098
NM_003775	endothelial differentiation, G-protein-coupled receptor 6 (EDG6).	EDG6	-1.340179424	-2.531828045	-0.115056567	0.923346107	0.025152843	2.742014102
NM_001398	enoyl Coenzyme A hydratase 1, peroxisomal (ECH1).	ECH1	-2.071138566	-4.202181762	-0.774080272	0.584761295	0.000928883	7.186148944
NM_001425	epithelial membrane protein 3 (EMP3).	EMP3	-2.48936745	-5.615316926	-0.699355629	0.61584721	0.028104637	9.118035832
NM_013333	epsin 1 (EPN1)	EPN1	-1.348040502	-2.545661337	-0.334289071	0.7931749	0.003784339	3.209457759
NM_014297	ethylmalonic encephalopathy 1 (ETHE1).	ETHE1	-1.840507983	-3.581361084	-0.461858304	0.726050446	0.014578054	4.9326615

NM_003757	eukaryotic translation initiation factor 3, subunit 2 beta, 36kDa (EIF3S2)	EIF3S2	-1.55046189	-2.929109018	-0.295499474	0.814790196	0.011665535	3.594924232
NM_003751	eukaryotic translation initiation factor 3, subunit 9 eta, 116kDa (EIF3S9), transcript variant 1	EIF3S9	-1.784055723	-3.44392977	0.135265277	1.098294745	0.013187126	3.135706317
NM_001416	eukaryotic translation initiation factor 4A, isoform 1 (EIF4A1)	EIF4A1	-1.292018877	-2.448704825	-0.346409414	0.786539206	0.003824686	3.1132648
NM_004096	eukaryotic translation initiation factor 4E binding protein 2 (EIF4EBP2)	EIF4EBP2	-2.391288651	-5.246257611	-0.251969758	0.839749096	0.009392352	6.247410847
NM_020158	exosome component 5 (EXOSC5).	EXOSC5	-1.829871094	-3.555053064	-0.226209248	0.854878178	0.010071106	4.15854932
NM_020223	family with sequence similarity 20, member C (FAM20C)	FAM20C	-2.106937344	-4.307758415	-0.03696584	0.974702709	0.000425328	4.419561344
NM_006712	FAST kinase (FASTK), transcript variant 1	FASTK	-1.204964331	-2.305315697	-0.577965695	0.66990773	0.027356081	3.441243611
NM_018438	F-box only protein 6 (FBXO6)	FBXO6	-1.81990206	-3.530572298	0.562306017	1.476627587	0.003438609	2.390970024
NM_024907	F-box protein 17 (FBXO17), transcript variant 2.	FBXO17	-1.261497594	-2.397444793	-0.658093836	0.633715042	0.001011425	3.783159047
NM_012179	F-box protein 7 (FBXO7).	FBXO7	-1.471521559	-2.773142129	-0.514327201	0.700119356	0.015246478	3.960956233
NM_004106	Fc fragment of IgE, high affinity I, receptor for; gamma polypeptide (FCER1G).	FCER1G	-2.512846563	-5.707450979	-0.68650173	0.621358705	0.032399713	9.185436578
XM_496386	Fc gamma receptor type I (LOC440607)	LOC440607	-2.79457465	-6.938263585	-0.634277463	0.644263397	0.016243441	10.76929656
NM_004460	fibroblast activation protein, alpha (FAP).	FAP	-1.769512679	-3.409387731	-0.243166221	0.844889035	0.007941825	4.035308297
NM_003862	fibroblast growth factor 18 (FGF18), transcript variant 1	FGF18	-1.886516553	-3.697413901	-1.233085035	0.42540679	0.049721456	8.691478344
NM_001004019	fibulin 2 (FBLN2), transcript variant 1.	FBLN2	-3.278935954	-9.706397573	0.31138742	1.240900483	0.004991863	7.822059631
NM_021939	FK506 binding protein 10, 65 kDa (FKBP10).	FKBP10	-1.822249238	-3.536321001	0.128552099	1.09319601	0.00887226	3.23484624
NM_054033	FK506 binding protein 1B, 12.6 kDa (FKBP1B), transcript variant 2.	FKBP1B	-2.396065715	-5.263657856	-0.428230411	0.743172791	0.006123796	7.082683755
NM_004117	FK506 binding protein 5 (FKBP5).	FKBP5	-2.540888026	-5.819471043	0.188759507	1.139783258	0.003981111	5.105769893
NM_207381	FLJ41287 protein (FLJ41287)	FLJ41287	-3.195873322	-9.163338548	-0.052180407	0.964477571	0.015022868	9.500831151
NM_198446	FLJ45459 protein (FLJ45459)	FLJ45459	-1.033916148	-2.047574789	-0.733075903	0.60161986	0.019534921	3.403436166
NM_002029	formyl peptide receptor 1 (FPR1)	FPR1	-1.308546058	-2.476917914	-0.43331336	0.740559029	0.014629755	3.344659667
NM_002030	formyl peptide receptor-like 2 (FPRL2)	FPRL2	-1.697775798	-3.244004449	-0.094523478	0.936581549	0.003277755	3.463664699
NM_004468	four and a half LIM domains 3 (FHL3)	FHL3	-2.505528647	-5.678573844	-0.547341708	0.684279813	0.049924308	8.29861372
CR601067	full-length cDNA clone CS0DC005YL10 of Neuroblastoma Cot 25-normalized of (human)	human	-1.742423558	-3.345967792	-0.097679703	0.934534802	0.003265853	3.58035654
NM_014164	FXYD domain containing ion transport regulator 5 (FXYD5), transcript variant 2.	FXYD5	-1.413770931	-2.664326579	-0.487722304	0.713150116	0.007117493	3.73599684
NM_022078	G patch domain containing 3 (GPATC3).	GPATC3	-1.426052454	-2.687104548	0.014618562	1.010184326	0.001641981	2.660014097
NM_177551	G protein-coupled receptor 109A (GPR109A)	GPR109A	-1.591024793	-3.012632706	-0.442634093	0.735789966	0.00150425	4.094419395
NM_013345	G protein-coupled receptor 132 (GPR132)	GPR132	-1.189454683	-2.280665211	0.035595668	1.024979935	0.019308586	2.225082788
NM_003608	G protein-coupled receptor 65 (GPR65).	GPR65	-2.818014711	-7.051913172	-0.045940127	0.968658389	0.003009946	7.280082694
NM_020370	G protein-coupled receptor 84 (GPR84)	GPR84	-1.854863199	-3.617174502	0.121636054	1.087967947	0.000521104	3.324706865
NM_016235	G protein-coupled receptor, family C, group 5, member B (GPRC5B)	GPRC5B	-3.178597916	-9.05426741	0.393473842	1.31355248	0.004007123	6.892962059
NM_002094	G1 to S phase transition 1 (GSPT1)	GSPT1	-1.276745198	-2.422917358	0.413417834	1.331837284	0.006250686	1.819229261
NM_000169	galactosidase, alpha (GLA)	GLA	-1.992708031	-3.979833378	0.098402279	1.070587178	0.001209891	3.717430455
NM_080839	gamma-glutamyltransferase-like 4 (GGTL4), transcript variant 2.	GGTL4	-1.437097033	-2.707754671	0.878647872	1.838651266	0.043519455	1.472685289
NM_001496	GDNF family receptor alpha 3 (GFRA3)	GFRA3	-1.748889058	-3.360996542	0.466703253	1.381947926	0.001387345	2.432071773

NM_013334	GDP-mannose pyrophosphorylase B (GMPPB), transcript variant 1	GMPPB	-1.300284096	-2.462773749	1.131260567	2.190500535	0.006137415	1.12429726
NM_016573	GEM interacting protein (GMIP).	GMIP	-2.137382892	-4.399632106	0.043718545	1.030767207	0.049810857	4.268308184
NM_138387	glucose 6 phosphatase, catalytic, 3 (G6PC3).	G6PC3	-1.462008588	-2.754916492	-0.626161592	0.64789791	0.004904461	4.252084242
NM_000175	glucose phosphate isomerase (GPI)	GPI	-1.29240992	-2.449368638	-0.503349731	0.705466885	0.021843068	3.47198244
NM_005313	glucose regulated protein, 58kDa (GRP58)	GRP58	-2.381479492	-5.210708295	-0.058251875	0.960427172	0.001588722	5.425406995
NM_000402	glucose-6-phosphate dehydrogenase (G6PD), nuclear gene encoding mitochondrial protein	G6PD	-1.527499714	-2.882857876	-0.261264881	0.834356078	0.014898152	3.455188921
NM_002046	glyceraldehyde-3-phosphate dehydrogenase (GAPDH).	GAPDH	-1.808577448	-3.502967124	-0.301174256	0.811591547	0.01358901	4.316170047
NM_004130	glycogenin (GYG).	GYG	-1.800586286	-3.483617646	-0.127680333	0.915301955	0.023136296	3.805976408
NM_000405	GM2 ganglioside activator (GM2A).	GM2A	-2.012134429	-4.033785667	0.171923245	1.126559291	5.57E-05	3.580624383
NM_015044	golgi associated, gamma adaptin ear containing, ARF binding protein 2 (GGA2), transcript variant 1	GGA2	-1.435590519	-2.704928611	-0.423450327	0.745639231	0.008087935	3.627664022
NM_004287	golgi SNAP receptor complex member 2 (GOSR2), transcript variant A.	GOSR2	-1.558087997	-2.944633326	-0.568467353	0.674332785	0.006251439	4.366736113
NM_017510	gp25L2 protein (HSGP25L2G)	HSGP25L2G	-1.438943307	-2.71122211	-0.20166289	0.869547721	0.000618762	3.117968164
NM_152237	growth arrest-specific 2 like 1 (GAS2L1), transcript variant 3	GAS2L1	-2.133121158	-4.386654731	-0.864795959	0.549124064	0.001081513	7.988458374
NM_004864	growth differentiation factor 15 (GDF15)	GDF15	-2.368774832	-5.165023208	-0.13739924	0.909156624	0.007710339	5.681114861
NM_002086	growth factor receptor-bound protein 2 (GRB2), transcript variant 1	GRB2	-2.994463394	-7.969357375	-0.908063837	0.532899788	0.018120513	14.95470173
NM_130759	GTPase, IMAP family member 1 (GIMAP1).	GIMAP1	-2.916716917	-7.551257516	-0.923145201	0.52735808	0.005327976	14.31903256
NM_015660	GTPase, IMAP family member 2 (GIMAP2).	GIMAP2	-1.437377897	-2.708281868	0.020511053	1.014318723	0.002291199	2.670050159
NM_018326	GTPase, IMAP family member 4 (GIMAP4).	GIMAP4	-2.048134775	-4.13570928	0.332599101	1.25928	0.00543018	3.28418563
NM_002053	guanylate binding protein 1, interferon-inducible, 67kDa (GBP1)	GBP1	-3.761850286	-13.56531164	-0.749553546	0.594787591	0.014513872	22.80698495
NM_198460	guanylate binding protein family, member 6 (GBP6).	GBP6	-3.43151	-10.7891552	0.305820257	1.236121245	0.005099508	8.728233775
NM_177925	H2A histone family, member J (H2AFJ), transcript variant 2	H2AFJ	-2.049541566	-4.139744031	-0.176222016	0.885017559	0.016145494	4.677584066
NM_002105	H2A histone family, member X (H2AFX)	H2AFX	-1.365633638	-2.576894794	-0.17033448	0.888636632	0.025818034	2.899829582
NM_014266	hematopoietic cell signal transducer (HCST)	HCST	-1.826228475	-3.546088334	-0.324784271	0.798417766	0.022811018	4.441394573
NM_005335	hematopoietic cell-specific Lyn substrate 1 (HCLS1)	HCLS1	-2.404290943	-5.293753223	-0.436529826	0.738909803	0.003799694	7.164275259
NM_016209	hematopoietic stem/progenitor cells 176 (HSPC176).	HSPC176	-1.717819174	-3.289387961	-0.221446161	0.857705239	0.027918536	3.835103028
NM_006848	hepatitis delta antigen-interacting protein A (DIPA)	DIPA	-1.804795599	-3.493796547	-0.505112311	0.704605523	0.001514201	4.958514277
NM_024747	Hermansky-Pudlak syndrome 6 (HPS6).	HPS6	-1.148018576	-2.21609323	-0.051624009	0.964849608	0.015071705	2.296827621
NM_000188	hexokinase 1 (HK1), nuclear gene encoding mitochondrial protein, transcript variant 1.	HK1	-1.513707227	-2.855428434	-0.055900122	0.961994051	0.00118028	2.96823918
NM_000189	hexokinase 2 (HK2).	HK2	-3.328921068	-10.04858926	0.07696103	1.054793824	0.000905303	9.526590909
NM_145904	high mobility group AT-hook 1 (HMGA1), transcript variant 6	HMGA1	-1.533473324	-2.894819358	-0.184441363	0.879989757	0.002713993	3.289605742
XM_375261	High-mobility group nucleosome binding domain 1 (LOC400452)	LOC400452	-1.255810517	-2.388012713	0.131115691	1.095140287	0.003920378	2.180554163
NM_002112	histidine decarboxylase (HDC).	HDC	-3.104894688	-8.603327084	0.304165874	1.234704559	0.001360196	6.967923641
NM_138720	histone 1, H2bd (HIST1H2BD), transcript variant 2	HIST1H2BD	-2.231244221	-4.695387486	-0.288402045	0.818808483	0.033026743	5.7344148
NM_080593	histone 1, H2bk (HIST1H2BK)	HIST1H2BK	-2.020617083	-4.05757309	-0.03271557	0.977578479	0.009262826	4.150636678
NM_003547	histone 1, H4g (HIST1H4G).	HIST1H4G	-2.425283652	-5.37134598	-0.051489646	0.964939472	0.000203117	5.566510789

NM_003541	histone 1, H4k (HIST1H4K).	HIST1H4K	-2.957342835	-7.766921234	-0.216029451	0.860931613	0.001272878	9.021530994
NM_003546	histone 1, H4l (HIST1H4L).	HIST1H4L	-1.863259941	-3.638288485	0.096066263	1.068855082	0.030119263	3.403911855
NM_003516	histone 2, H2aa (HIST2H2AA).	HIST2H2AA	-3.058625878	-8.331786538	0.296170285	1.227880606	0.004579164	6.78550219
NM_007069	HRAS-like suppressor 3 (HRASLS3).	HRASLS3	-1.859333743	-3.628400588	-0.427428434	0.743586027	0.014560865	4.879597592
NM_014187	HSPC171 protein (HSPC171).	HSPC171	-1.50551274	-2.839255617	0.014850092	1.010346458	0.001677673	2.810180206
NM_024711	human immune associated nucleotide 2 (hIAN2)	hIAN2	-1.697679659	-3.24378828	0.616479981	1.533129944	0.006714605	2.115794745
NM_178232	hyaluronan and proteoglycan link protein 3 (HAPLN3).	HAPLN3	-1.924229565	-3.795341136	0.168628947	1.123989803	0.004275187	3.376668656
NM_207322	hypothetical LOC145741 (LOC145741)	LOC145741	-1.667975754	-3.177684196	-0.172305972	0.887423109	0.024026186	3.580799466
NM_022733	hypothetical protein AL133206 (LOC64744)	LOC64744	-1.770075644	-3.410718395	-0.129618749	0.914072974	2.80E-05	3.73134147
NM_018982	hypothetical protein DJ167A19.1 (DJ167A19.1)	DJ167A191	-1.402864458	-2.644260768	-0.199311784	0.870965945	0.009822887	3.036009366
XM_290820	hypothetical protein FLJ10211 (FLJ10211)	FLJ10211	-1.804361231	-3.49274479	-0.296850976	0.814027266	0.03154922	4.290697542
NM_018166	hypothetical protein FLJ10647 (FLJ10647)	FLJ10647	-1.174995802	-2.257922239	0.021610618	1.015092092	0.008169793	2.224352113
NM_018295	hypothetical protein FLJ11000 (FLJ11000).	FLJ11000	-2.704171014	-6.516832952	0.480211432	1.394948086	0.000256196	4.671738696
NM_025147	hypothetical protein FLJ13448 (FLJ13448)	FLJ13448	-1.239468148	-2.361114733	0.235428352	1.177256225	0.007740099	2.005608195
NM_032790	hypothetical protein FLJ14466 (FLJ14466).	FLJ14466	-2.170593088	-4.502084353	-0.543761579	0.685980001	0.005871874	6.562996515
NM_017841	hypothetical protein FLJ20487 (FLJ20487).	FLJ20487	-1.119408982	-2.172579518	0.161322956	1.118312164	0.005595745	1.94273083
NM_017907	hypothetical protein FLJ20625 (FLJ20625)	FLJ20625	-1.530729405	-2.889318818	-0.383515192	0.766567537	0.016731498	3.769164071
NM_024660	hypothetical protein FLJ22573 (FLJ22573)	FLJ22573	-1.763114475	-3.394300926	-0.017491818	0.9879488	0.000662748	3.435705298
NM_024579	hypothetical protein FLJ23221 (FLJ23221)	FLJ23221	-2.410433294	-5.316339706	0.143956298	1.104931014	4.95E-05	4.811467541
NM_024671	hypothetical protein FLJ23436 (FLJ23436).	FLJ23436	-1.584652313	-2.999355053	-0.493956499	0.710075089	0.01721965	4.223997008
NM_024575	hypothetical protein FLJ23467 (FLJ23467)	FLJ23467	-3.142784625	-8.832272137	-0.439631034	0.737323153	0.003395386	11.97883465
NM_178518	hypothetical protein FLJ36878 (FLJ36878).	FLJ36878	-2.61718404	-6.135513275	0.059538053	1.04213202	0.048924791	5.887462586
NM_178830	hypothetical protein FLJ36888 (FLJ36888).	FLJ36888	-1.210508971	-2.314192652	-0.353991111	0.782416603	0.004824259	2.95774993
NM_001002836	hypothetical protein LOC126208 (LOC126208).	LOC126208	-1.515055761	-2.858098744	-0.317287273	0.802577561	0.003270654	3.561149578
NM_174925	hypothetical protein LOC205251 (LOC205251)	LOC205251	-1.420611319	-2.676989201	-0.345819679	0.786860788	0.022487317	3.402112853
NM_207340	hypothetical protein LOC254359 (LOC254359)	LOC254359	-1.417072764	-2.670431289	-0.197336941	0.872158989	0.005348627	3.061862943
NM_198282	hypothetical protein LOC340061 (LOC340061).	LOC340061	-3.178707941	-9.054957947	-0.7200907	0.607059276	0.006512117	14.91610178
NM_032350	hypothetical protein MGC11257 (MGC11257).	MGC11257	-1.182307724	-2.269394976	-0.600231571	0.659648065	0.018763402	3.44031173
NM_031465	hypothetical protein MGC13204 (MGC13204).	MGC13204	-2.031812023	-4.089181284	-0.222759097	0.856925033	0.040416511	4.771924178
NM_138418	hypothetical protein MGC15416 (MGC15416)	MGC15416	-1.346376248	-2.542726423	-0.479837122	0.717058574	0.02200791	3.546051208
NM_032369	hypothetical protein MGC15619 (MGC15619).	MGC15619	-3.314969905	-9.951885623	-0.487459661	0.713279957	0.017103271	13.95228554
NM_152637	hypothetical protein MGC17301 (MGC17301).	MGC17301	-1.767973001	-3.405751094	0.135197975	1.098243511	0.009772002	3.101089204
NM_032331	hypothetical protein MGC2408 (MGC2408)	MGC2408	-1.206809978	-2.308266786	-0.151609477	0.900245584	0.00155422	2.56404122
NM_182565	hypothetical protein MGC29814 (MGC29814)	MGC29814	-1.645873057	-3.129371761	0.145423463	1.106055257	0.004180086	2.829308698
NM_138346	hypothetical protein MGC33867 (MGC33867)	MGC33867	-1.221692084	-2.332200923	-0.271298597	0.828573395	0.026274508	2.81471857
NM_152456	hypothetical protein MGC34647 (MGC34647)	MGC34647	-1.552869571	-2.934001424	0.429957079	1.347193496	0.01614248	2.177861927
NM_152346	hypothetical protein MGC34680 (MGC34680)	MGC34680	-1.568114806	-2.965169972	-0.335140977	0.792706671	0.005784295	3.740563918
NM_152759	hypothetical protein MGC35140 (MGC35140)	MGC35140	-1.607861203	-3.047996408	0.151547867	1.110760568	0.02221717	2.744062489
NM_153374	hypothetical protein MGC35274 (MGC35274)	MGC35274	-2.160899069	-4.471934535	0.393577084	1.313646484	0.03825311	3.404214595
NM_152766	hypothetical protein MGC40107 (MGC40107).	MGC40107	-1.208679724	-2.31126026	0.040032985	1.028137333	0.002009677	2.248007329
NM_016466	hypothetical protein MGC41816 (MGC41816)	MGC41816	-1.100309098	-2.14400623	-0.044408778	0.969687117	0.007441056	2.21102889
NM_182705	hypothetical protein MGC45871 (MGC45871).	MGC45871	-2.892234489	-7.42419441	-0.051120157	0.965186635	0.001487928	7.691978051
NM_052871	hypothetical protein MGC4677 (MGC4677)	MGC4677	-3.38454932	-10.44361533	0.281382504	1.215358979	0.0017051	8.593029311
NM_145058	hypothetical protein MGC7036 (MGC7036)	MGC7036	-2.018513402	-4.051660811	-0.392842781	0.761627364	0.001003107	5.319741649
NM_153341	IBR domain containing 3 (IBRDC3).	IBRDC3	-3.07560642	-8.430431176	0.333437537	1.260012055	0.012456489	6.69075438

CN644277	ILLUMIGEN_MCQ_10044 Katze_MMPL2 Macaca mulatta cDNA clone IBIUW:9084 5' Bases 7 to 588 highly human TFRC (Hs.185726) sequence	Hs185726	-3.562936507	-11.81818438	-0.356676485	0.780961598	0.005114135	15.13286236
CK230655	ILLUMIGEN_MCQ_1009 Katze_MMPL2 Macaca mulatta cDNA 5' human Unigene Hs.500464 sequence		-2.81442523	-7.034389547	-0.473268736	0.720330683	0.000433609	9.765500357
CN644408	ILLUMIGEN_MCQ_10328 Katze_MMPL2 Macaca mulatta cDNA clone IBIUW:9224 5' Bases 70 to 978 highly human COL4A1 (Hs.437173) sequence	Hs437173	-2.451891362	-5.471329199	-0.618685473	0.651264064	0.003716785	8.401091821
CN644776	ILLUMIGEN_MCQ_10841 Katze_MMPL2 Macaca mulatta cDNA clone IBIUW:9592 5' Bases 1 to 695 highly human PP2135 (Hs.132569) sequence	Hs132569	-1.605669445	-3.043369375	-0.370697333	0.773408576	0.031724653	3.935008571
CK231327	ILLUMIGEN_MCQ_1845 Katze_MMLG Macaca mulatta cDNA 5' human TFPI2 (Hs.438231) sequence	Hs438231	-1.354607719	-2.557275723	-0.451344146	0.731361128	0.005128444	3.496597817
CK231337	ILLUMIGEN_MCQ_1868 Katze_MMLG Macaca mulatta cDNA 5' human RPL7 (Hs.421257) sequence	Hs421257	-1.209887968	-2.31319673	-0.560589922	0.678024861	0.015724178	3.41166949
CK231513	ILLUMIGEN_MCQ_2375 Katze_MMLG Macaca mulatta cDNA 5' human PSG5 (Hs.251850) sequence	Hs251850	-3.270697784	-9.651129428	-0.274551451	0.82670731	0.006937722	11.67417937
CN645487	ILLUMIGEN_MCQ_24069 Katze_MMSP Macaca mulatta cDNA clone IBIUW:10303 5' Bases 523 to 999 highly human IGHG1 (Hs.413826) sequence	Hs413826	-1.816233985	-3.521607164	-0.035781343	0.975503299	0.045393989	3.610041265
CN645791	ILLUMIGEN_MCQ_24884 Katze_MMBR Macaca mulatta cDNA clone IBIUW:10607 5' Bases 254 to 768 highly human Unigene Hs.25892 sequence		-1.548696157	-2.925526237	-0.108061888	0.927833674	0.014592678	3.153071847
CN647236	ILLUMIGEN_MCQ_28050 Katze_MMBR Macaca mulatta cDNA clone IBIUW:7788 5' Bases 196 to 727 highly human TTYH2 (Hs.27935) sequence	Hs27935	-1.947069225	-3.855904244	0.205512228	1.15309568	0.003953931	3.343958624
CN647305	ILLUMIGEN_MCQ_28193 Katze_MMBR Macaca mulatta cDNA clone IBIUW:7729 5' Bases 13 to 529 highly human RPS27L (Hs.108957) sequence	Hs108957	-1.399038763	-2.637258086	-0.470761273	0.721583736	0.013504377	3.65481919
CN647492	ILLUMIGEN_MCQ_28598 Katze_MMPB Macaca mulatta cDNA clone IBIUW:7532 5' Bases 1 to 534 highly human SPI1 (Hs.157441) sequence	Hs157441	-2.160955824	-4.472110464	-1.263326037	0.416582449	0.018509558	10.73523495
CN647641	ILLUMIGEN_MCQ_28871 Katze_MMPB Macaca mulatta cDNA clone IBIUW:7393 5' Bases 1 to 845 highly human BZRP (Hs.202) sequence	Hs202	-1.779106586	-3.432135679	-0.22454462	0.855865133	0.004513793	4.010136115
CN648004	ILLUMIGEN_MCQ_29540 Katze_MMPB Macaca mulatta cDNA clone IBIUW:7020 5' Bases 17 to 588 highly human Unigene Hs.435390 sequence		-1.996068628	-3.989114759	-0.31059093	0.806311425	0.001609899	4.947362315
CN648055	ILLUMIGEN_MCQ_29639 Katze_MMPB Macaca mulatta cDNA clone IBIUW:6975 5' Bases 1 to 889 highly human GZMB (Hs.1051) sequence	Hs1051	-1.413436373	-2.663708799	0.021381654	1.014931003	0.001957199	2.624522052
CO645773	ILLUMIGEN_MCQ_30118 Katze_MMPB Macaca mulatta cDNA clone IBIUW:22572 5' Bases 1 to 42 highly human RARRES3 (Hs.17466) sequence	Hs17466	-3.316417425	-9.961875805	-0.343826961	0.787948388	0.002165495	12.64280244

CN648306	ILLUMIGEN_MCQ_30203 Katze_MMPB Macaca mulatta cDNA clone IBIUW:6746 5' Bases 1 to 857 highly human GZMA (Hs.90708) sequence	Hs90708	-2.847369037	-7.196867197	0.420937499	1.338797256	0.000761791	5.375621412
CN648569	ILLUMIGEN_MCQ_30855 Katze_MMPB Macaca mulatta cDNA clone IBIUW:6483 5' Bases 1 to 1036 highly human BHLHB2 (Hs.171825) sequence	Hs171825	-1.76749445	-3.404621572	-0.524171099	0.695358514	0.013260714	4.896210379
CN648668	ILLUMIGEN_MCQ_31078 Katze_MMPB Macaca mulatta cDNA clone IBIUW:6390 5' Bases 1 to 798 highly human PFN1 (Hs.408943) sequence	Hs408943	-1.400230743	-2.639437936	-0.445119004	0.734523725	0.020255061	3.593400519
CO725352	ILLUMIGEN_MCQ_32983 Katze_MMBR Macaca mulatta cDNA clone IBIUW:27860 5' Bases 1 to 613 highly human LSM10 (Hs.3496) sequence	Hs3496	-2.172226102	-4.507183234	-0.219412389	0.858915202	0.018330217	5.247529934
CK232222	ILLUMIGEN_MCQ_3441 Katze_MMPL2 Macaca mulatta cDNA 5' human TFPI2 (Hs.438231) sequence	Hs438231	-4.091631836	-17.04919641	0.100873834	1.072422828	0.000110891	15.89783056
CN804935	ILLUMIGEN_MCQ_35237 Katze_MMPL1 Macaca mulatta cDNA clone IBIUW:12572 5' Bases 1 to 786 highly human LOXL1 (Hs.65436) sequence	Hs65436	-1.137788747	-2.200434987	0.039693949	1.027895747	0.013160935	2.140718058
CN805014	ILLUMIGEN_MCQ_35340 Katze_MMPL1 Macaca mulatta cDNA clone IBIUW:11710 5' Bases 1 to 303 highly human Unigene Hs.501487 sequence		-1.683384004	-3.211804331	-0.706231678	0.612918995	0.02384541	5.240177504
CN806576	ILLUMIGEN_MCQ_35604 Katze_MMPL1 Macaca mulatta cDNA clone IBIUW:15312 5' Bases 1 to 980 highly human LITAF (Hs.76507) sequence	Hs76507	-2.994416262	-7.969097021	-0.472029703	0.720949592	0.003800893	11.05361194
CN801572	ILLUMIGEN_MCQ_35741 Katze_MMPL1 Macaca mulatta cDNA clone IBIUW:14415 5' Bases 1 to 342 highly human HN1 (Hs.109706) sequence	Hs109706	-1.532731978	-2.893332202	0.29359953	1.225694579	0.002339317	2.360565391
CN801916	ILLUMIGEN_MCQ_37292 Katze_MMBR Macaca mulatta cDNA clone IBIUW:14420 5' Bases 166 to 772 highly human Unigene Hs.518521 sequence		-1.401254803	-2.641312138	-0.10726592	0.928345722	0.009880384	2.845181568
CN801994	ILLUMIGEN_MCQ_37433 Katze_MMBR Macaca mulatta cDNA clone IBIUW:16004 5' Bases 1 to 410 highly human MT1X (Hs.374950) sequence	Hs374950	-4.49067236	-22.48159299	-0.101688229	0.931941803	0.000854529	24.12338722
CO725743	ILLUMIGEN_MCQ_38118 Katze_MMBR Macaca mulatta cDNA clone IBIUW:27611 5' Bases 356 to 881 highly human GNG10 (Hs.433898) sequence	Hs433898	-2.246236197	-4.74443469	-0.327730745	0.79678879	0.001969712	5.954444577
CO646399	ILLUMIGEN_MCQ_39129 Katze_MMPB2 Macaca mulatta cDNA clone IBIUW:21744 5' Bases 393 to 753 highly human CTSD (Hs.343475) sequence	Hs343475	-2.962702724	-7.795830502	-0.657784909	0.633850754	0.000596416	12.29915788
CO646675	ILLUMIGEN_MCQ_39493 Katze_MMPB2 Macaca mulatta cDNA clone IBIUW:23645 5' Bases 32 to 976 highly human CXCL11 (Hs.103982) sequence	Hs103982	-7.086019161	-135.8639752	-1.045107246	0.484608883	0.020987959	280.357996
CO646710	ILLUMIGEN_MCQ_39534 Katze_MMPB2 Macaca mulatta cDNA clone IBIUW:22979 5' Bases 79 to 930 highly human APOBEC3F (Hs.337667) sequence	Hs337667	-3.724333793	-13.21710029	-0.021075728	0.985497606	0.011249437	13.4116006

CO646712	ILLUMIGEN_MCQ_39536 Katze_MMPB2 Macaca mulatta cDNA clone IBIUW:23033 5' Bases 5 to 869 highly human IAN4L1 (Hs.412331) sequence	Hs412331	-1.617241113	-3.067878001	0.025989951	1.018178108	0.021994045	3.013105446
CO646894	ILLUMIGEN_MCQ_39773 Katze_MMPB2 Macaca mulatta cDNA clone IBIUW:25891 5' Bases 5 to 696 highly human SLC16A3 (Hs.386678) sequence	Hs386678	-2.47974572	-5.577991438	-0.083199723	0.94396173	0.000105922	5.90912879
CO647278	ILLUMIGEN_MCQ_40268 Katze_MMPB2 Macaca mulatta cDNA clone IBIUW:23292 5' Bases 70 to 562 highly human COMT (Hs.240013) sequence	Hs240013	-1.992469048	-3.97917417	-0.039943581	0.972692985	0.001641676	4.090884
CO647377	ILLUMIGEN_MCQ_40406 Katze_MMPB2 Macaca mulatta cDNA clone IBIUW:21576 5' Bases 2 to 949 highly human APOL3 (Hs.241535) sequence	Hs241535	-3.617714	-12.27553502	-0.108891899	0.927300026	0.001028671	13.23793236
CO647394	ILLUMIGEN_MCQ_40429 Katze_MMPB2 Macaca mulatta cDNA clone IBIUW:24175 5' Bases 238 to 766 highly human Unigene Hs.517602 sequence		-1.230004443	-2.345677123	-0.418459479	0.748223157	0.024222076	3.134996695
CK232488	ILLUMIGEN_MCQ_4157 Katze_MMPL2 Macaca mulatta cDNA 5' human PSG4 (Hs.458318) sequence	Hs458318	-2.668663183	-6.35839738	0.263866011	1.200691909	0.003110683	5.295611084
CO648296	ILLUMIGEN_MCQ_41618 Katze_MMPB2 Macaca mulatta cDNA clone IBIUW:25660 5' Bases 4 to 498 highly human MGC50844 (Hs.27267) sequence	Hs27267	-2.948864071	-7.721408663	-0.329967029	0.795554665	0.000761998	9.705692145
CO648453	ILLUMIGEN_MCQ_41830 Katze_MMPB2 Macaca mulatta cDNA clone IBIUW:25190 5' Bases 185 to 962 highly human C6orf175 (Hs.356224) sequence	Hs356224	-2.205526168	-4.612427286	0.101241941	1.072696494	0.001224556	4.299843724
CO648815	ILLUMIGEN_MCQ_42308 Katze_MMPB2 Macaca mulatta cDNA clone IBIUW:25858 5' Bases 5 to 765 highly human SYT11 (Hs.380439) sequence	Hs380439	-1.410045013	-2.657454541	0.141297596	1.102896643	0.001425082	2.409522741
CO644910	ILLUMIGEN_MCQ_43164 Katze_MMJJ Macaca mulatta cDNA clone IBIUW:23286 5' Bases 7 to 752 highly human NMES1 (Hs.112242) sequence	Hs112242	-4.062771078	-16.71152027	-0.284911687	0.820791851	0.00577581	20.36024145
CO581942	ILLUMIGEN_MCQ_46474 Katze_MMJJ Macaca mulatta cDNA clone IBIUW:19887 5' Bases 82 to 1012 highly human HLA-DQA1 (Hs.387679) sequence	Hs387679	-2.721099946	-6.593753463	-0.788333884	0.579012386	0.003330134	11.38793162
CO581798	ILLUMIGEN_MCQ_46700 Katze_MMJJ Macaca mulatta cDNA clone IBIUW:19705 5' Bases 3 to 974 highly human ANPEP (Hs.1239) sequence	Hs1239	-2.060828686	-4.172258907	0.01259627	1.008769296	0.018928966	4.135989193
CO581027	ILLUMIGEN_MCQ_47873 Katze_MMOV Macaca mulatta cDNA clone IBIUW:17465 5' Bases 3 to 593 highly human MGC49942 (Hs.206824) sequence	Hs206824	-1.68054817	-3.205497248	-0.567630852	0.674723889	0.028874888	4.75082815
CO580929	ILLUMIGEN_MCQ_48009 Katze_MMLV Macaca mulatta cDNA clone IBIUW:18847 5' Bases 166 to 669 highly human SAA2 (Hs.1955) sequence	Hs1955	-4.09706731	-17.11355183	0.249310027	1.188638509	0.00059093	14.39760844
CO580739	ILLUMIGEN_MCQ_48269 Katze_MMTE Macaca mulatta cDNA clone IBIUW:20526 5' Bases 460 to 845 highly human C1QG (Hs.94953) sequence	Hs94953	-1.736369798	-3.331957036	0.166640642	1.1224418	0.014548399	2.9684898

CO580643	ILLUMIGEN_MCQ_48393 Katze_MMLV Macaca mulatta cDNA clone IBIUW:18409 5' Bases 755 to 975 highly human Unigene Hs.515465 sequence		-2.851437887	-7.217193256	0.034808409	1.02442077	0.012354004	7.045145379
CO580464	ILLUMIGEN_MCQ_48633 Katze_MMTE Macaca mulatta cDNA clone IBIUW:16662 5' Bases 400 to 718 highly human NDUFAB1 (Hs.5556) sequence	Hs5556	-1.747172186	-3.356999184	0.152922463	1.111819401	0.010190463	3.019374531
CO579438	ILLUMIGEN_MCQ_50129 Katze_MMOV Macaca mulatta cDNA clone IBIUW:19046 5' Bases 4 to 953 highly human PSAP (Hs.406455) sequence	Hs406455	-1.875945775	-3.670421571	-0.146869248	0.903208365	0.000912744	4.063759498
CN641710	ILLUMIGEN_MCQ_5231 Katze_MMBR Macaca mulatta cDNA clone IBIUW:6025 5' Bases 11 to 158 highly human RPN2 (Hs.406532) sequence	Hs406532	-2.514711322	-5.714832927	-0.726353318	0.604429794	0.01344759	9.454915994
DR774422	ILLUMIGEN_MCQ_58593 Katze_MMLV Macaca mulatta cDNA clone IBIUW:34887 5' Bases 9 to 306 highly human Unigene Hs.529672 sequence		-3.587654692	-12.02241393	-0.137628429	0.909012205	0.005722734	13.22580033
DR774547	ILLUMIGEN_MCQ_58787 Katze_MMTE Macaca mulatta cDNA clone IBIUW:32927 5' Bases 5 to 768 highly human SNX17 (Hs.278569) sequence	Hs278569	-1.367868286	-2.580889342	-0.446580413	0.73378005	0.01502591	3.517251991
CN642140	ILLUMIGEN_MCQ_5952 Katze_MMPL2 Macaca mulatta cDNA clone IBIUW:5117 5' Bases 1 to 669 highly human LGMN (Hs.18069) sequence	Hs18069	-1.488885461	-2.806720611	-0.538211494	0.688624066	0.020108281	4.075838691
DV768600	ILLUMIGEN_MCQ_62274 Katze_MMTE Macaca mulatta cDNA clone IBIUW:40854 5' Bases 5 to 527 highly human IGFBP3 (Hs.450230) sequence	Hs450230	-3.902005526	-14.94929481	0.067978792	1.048247066	0.000549136	14.2612322
DR771539	ILLUMIGEN_MCQ_66662 Katze_MMTE Macaca mulatta cDNA clone IBIUW:35638 5' Bases 6 to 389 highly human ATOX1 (Hs.125213) sequence	Hs125213	-2.403596044	-5.291204011	-0.471531802	0.721198449	0.001581563	7.336682465
DV770912	ILLUMIGEN_MCQ_70634 Katze_MMTE Macaca mulatta cDNA clone IBIUW:40195 5' Bases 4 to 751 highly human HLA-DRB1 (Hs.520049) sequence	Hs520049	-1.670439967	-3.183116517	-0.532291295	0.691455688	0.005265413	4.60350037
CN643020	ILLUMIGEN_MCQ_7125 Katze_MMPL2 Macaca mulatta cDNA clone IBIUW:4136 5' Bases 1 to 1118 highly human LYZ (Hs.234734) sequence	Hs234734	-3.261176508	-9.587645107	-0.360407623	0.778944464	0.001551883	12.30850921
CK230409	ILLUMIGEN_MCQ_731 Katze_MMPL2 Macaca mulatta cDNA 5' human GLRX (Hs.28988) sequence	Hs28988	-2.534136468	-5.792300573	-0.056917804	0.961315697	0.000565867	6.025388531
CO645122	ILLUMIGEN_MCQ_8291 Katze_MMBR Macaca mulatta cDNA clone IBIUW:23018 5' Bases 1 to 553 highly human Unigene Hs.203697 sequence		-2.043922283	-4.123651131	-0.053767091	0.963417416	0.016141984	4.280233119
NM_052815	immediate early response 3 (IER3), transcript variant long	IER3	-2.052708901	-4.148842531	0.159060916	1.116560107	0.001033882	3.715735953
NM_005849	immunoglobulin superfamily, member 6 (IGSF6).	IGSF6	-1.63603075	-3.108095322	-0.202877833	0.868815753	0.017559067	3.577392917
NM_002164	indoleamine-pyrrole 2,3 dioxygenase (INDO).	INDO	-4.963570307	-31.20208013	-0.231842942	0.851546406	0.000584447	36.64166735
NM_001552	insulin-like growth factor binding protein 4 (IGFBP4)	IGFBP4	-3.06905847	-8.39225474	-0.235159704	0.849590946	0.007985269	9.877994559
NM_152713	integral membrane protein 1 (ITM1).	ITM1	-1.603530525	-3.03886066	-0.002514559	0.998258559	0.021536203	3.044161889
NM_022377	intercellular adhesion molecule 4, Landsteiner-Wiener blood group (ICAM4), transcript variant 2.	ICAM4	-1.503532694	-2.835361515	-0.667931117	0.629408638	0.024688824	4.504802357

NM_002198	interferon regulatory factor 1 (IRF1).	IRF1	-3.241508663	-9.457826414	-0.746205127	0.596169665	0.001035001	15.86432013
NM_001572	interferon regulatory factor 7 (IRF7), transcript variant a.	IRF7	-2.043749435	-4.123157111	-0.093080176	0.937518993	0.004056373	4.397945151
NM_002163	interferon regulatory factor 8 (IRF8)	IRF8	-1.259592892	-2.394281682	-0.200747214	0.870099796	0.024111566	2.751732264
NM_002201	interferon stimulated gene 20kDa (ISG20)	ISG20	-1.439876045	-2.712975548	-0.385017557	0.765769679	0.005619433	3.542808786
NM_006332	interferon, gamma-inducible protein 30 (IFI30)	IFI30	-2.687714447	-6.442918952	-0.419891281	0.747480951	0.006016183	8.619509225
NM_005533	interferon-induced protein 35 (IFI35).	IFI35	-1.393877452	-2.627840027	-0.307383731	0.808105898	0.001918109	3.251851066
NM_001547	interferon-induced protein with tetratricopeptide repeats 2 (IFIT2).	IFIT2	-1.706510386	-3.263704379	0.108194765	1.077878649	0.012710166	3.027895933
NM_000575	interleukin 1, alpha (IL1A)	IL1A	-1.970084009	-3.917909325	0.635846029	1.553848695	0.035866927	2.521422669
NM_001558	interleukin 10 receptor, alpha (IL10RA)	IL10RA	-1.712697925	-3.277732059	-0.123280307	0.918097764	0.003058731	3.570134017
NM_000628	interleukin 10 receptor, beta (IL10RB).	IL10RB	-1.575810215	-2.981028596	-0.070879808	0.952057222	0.011564142	3.131144355
NM_172200	interleukin 15 receptor, alpha (IL15RA), transcript variant 2	IL15RA	-2.146699194	-4.428134963	0.065557683	1.046489389	0.01188695	4.231418885
NM_021798	interleukin 21 receptor (IL21R), transcript variant 1.	IL21R	-2.968553534	-7.827510475	0.471378906	1.386433968	0.027201583	5.645786715
NM_004843	interleukin 27 receptor, alpha (IL27RA).	IL27RA	-1.921992011	-3.789459299	-0.640245871	0.641603594	0.044893104	5.906231407
NM_172374	interleukin 4 induced 1 (IL4I1), transcript variant 2	IL4I1	-3.430365025	-10.78059593	-0.193173036	0.87467985	0.003048608	12.32519068
NM_000418	interleukin 4 receptor (IL4R), transcript variant 1.	IL4R	-1.869276573	-3.653493328	-0.164664694	0.892135843	0.000330522	4.095220877
NM_203500	kelch-like ECH-associated protein 1 (KEAP1), transcript variant 1	KEAP1	-1.522734638	-2.873351795	-0.156548228	0.897169059	0.045182789	3.202687125
NM_000420	Kell blood group (KEL).	KEL	-2.674242516	-6.383034813	-0.01962105	0.98649179	0.005145456	6.470438859
NM_002275	keratin 15 (KRT15).	KRT15	-1.21540198	-2.322054736	0.107779124	1.077568157	0.000280418	2.15490289
NM_015140	KIAA0153 protein (KIAA0153).	KIAA0153	-1.662575269	-3.165811309	0.005008994	1.003478004	0.006835165	3.154838766
NM_014699	KIAA0296 gene product (KIAA0296)	KIAA0296	-1.298601046	-2.459902349	-0.063297442	0.957074117	0.010930679	2.570231821
NM_014812	KIAA0470 (KIAA0470).	KIAA0470	-1.13792807	-2.200647495	-0.06814049	0.953866658	0.037212924	2.307080845
NM_014696	KIAA0514 (KIAA0514).	KIAA0514	-1.418663936	-2.673378177	-0.414138171	0.750467671	0.042059923	3.562282933
XM_093895	KIAA0882 protein (KIAA0882)	KIAA0882	-2.445232722	-5.446134913	0.376211357	1.297928902	0.002325024	4.196019448
XM_375553	KIAA0963 (KIAA0963)	KIAA0963	-1.875028675	-3.668089078	-0.673952427	0.626787178	0.012195681	5.852208222
NM_015079	KIAA1055 protein (KIAA1055).	KIAA1055	-1.09165004	-2.131176442	0.042173664	1.029664022	0.001523724	2.069778488
NM_020792	KIAA1363 protein (KIAA1363)	KIAA1363	-3.52883553	-11.5421136	-0.290883098	0.81740156	0.004838228	14.12049372
NM_021035	KIAA1404 protein (KIAA1404)	KIAA1404	-1.327143718	-2.509054342	-0.035062714	0.975989333	0.001136033	2.570780496
NM_032430	KIAA1811 protein (KIAA1811)	KIAA1811	-1.522696102	-2.873275046	-0.422184655	0.746293665	0.044282656	3.850059541
NM_144580	kidney predominant protein NCU-G1 (MGC31963)	MGC31963	-1.474734636	-2.779325173	0.159121274	1.116606821	0.001646827	2.489081314
NM_032857	lactamase, beta (LACTB), nuclear gene encoding mitochondrial protein, transcript variant 1	LACTB	-2.398657044	-5.273120787	-0.381747293	0.767507475	0.040050253	6.870448765
NM_005566	lactate dehydrogenase A (LDHA).	LDHA	-2.668979322	-6.359790853	-0.790440023	0.578167723	0.001099012	10.99990642
NM_020169	latexin (LXN)	LXN	-1.24934313	-2.377331567	-0.289792219	0.818019863	0.002137729	2.906202738
NM_015907	leucine aminopeptidase 3 (LAP3)	LAP3	-4.224995189	-18.70037361	-1.169413125	0.444602164	0.001017212	42.06091452
NM_024509	leucine rich repeat and fibronectin type III domain containing 3 (LRFN3).	LRFN3	-1.38183551	-2.605997158	-0.386086142	0.765202694	0.02664223	3.405629879
NM_130830	leucine rich repeat containing 15 (LRRC15).	LRRC15	-1.776472192	-3.425874238	-0.713365505	0.609895719	0.00265638	5.617147536
NM_145256	leucine rich repeat containing 25 (LRRC25).	LRRC25	-2.171905382	-4.506181371	0.271847377	1.207352858	0.002295608	3.732282025
NM_018103	leucine rich repeat containing 5 (LRRC5)	LRRC5	-1.546515956	-2.921108521	0.08612829	1.061517599	0.005259813	2.751822979
NM_007161	leukocyte specific transcript 1 (LST1), transcript variant 1	LST1	-1.503728617	-2.835746593	-0.208654434	0.86534394	0.004770009	3.277016759
NM_004811	leupaxin (LPXN).	LPXN	-1.289672684	-2.444725838	-0.274925435	0.826493034	0.011391451	2.957950929
NM_014056	likely ortholog of mouse hypoxia induced gene 1 (HIG1).	HIG1	-1.491623576	-2.812052591	-0.1763129	0.884961808	0.015336168	3.177597683
NM_002314	LIM domain kinase 1 (LIMK1), transcript variant 1	LIMK1	-2.429256364	-5.386157301	-0.506528781	0.703914066	0.007010168	7.651725636

NM_006033	lipase, endothelial (LIPG).	LIPG	-2.910017689	-7.516274149	0.003629061	1.00251864	0.016995342	7.497390919
NM_005779	lipoma HMGIC fusion partner-like 2 (LHFPL2)	LHFPL2	-1.344596441	-2.539591476	1.05965423	2.084431888	0.001514169	1.218361459
NM_004862	lipopolysaccharide-induced TNF factor (LITAF)	LITAF	-2.95894333	-7.775542469	-0.680475889	0.623959421	0.001362668	12.46161563
NM_014020	LR8 protein (LR8).	LR8	-2.104125998	-4.29937216	-0.478897741	0.717525624	0.024731511	5.991942332
NM_002345	lumican (LUM)	LUM	-1.541078717	-2.910120147	0.416353208	1.334549861	0.045978639	2.18060054
NM_002346	lymphocyte antigen 6 complex, locus E (LY6E).	LY6E	-2.131596125	-4.382020171	-0.367402817	0.775176738	0.001514994	5.652930433
NM_004271	lymphocyte antigen 86 (LY86)	LY86	-3.183352096	-9.084153574	-0.114285171	0.923839943	0.001777011	9.833038335
NM_015364	lymphocyte antigen 96 (LY96)	LY96	-2.006981723	-4.019404361	0.17918748	1.132246029	0.006426431	3.549939021
NM_002286	lymphocyte-activation gene 3 (LAG3)	LAG3	-3.356554156	-10.24291298	-1.215508996	0.430621126	0.002024202	23.78636894
NM_005561	lysosomal-associated membrane protein 1 (LAMP1)	LAMP1	-1.949423949	-3.862202875	0.49237342	1.406757271	0.001291456	2.745465017
NM_002318	lysyl oxidase-like 2 (LOXL2)	LOXL2	-1.656106097	-3.15164731	-0.07541923	0.949066294	0.0127496	3.320787315
XR_010199	Macaca mulatta 5-aminoimidazole-4-carboxamide ribonucleotide formyltransferase		-1.299046562	-2.460662104	0.100808693	1.072374406	0.008664229	2.294592346
XR_010624	Macaca mulatta actin related protein 2		-1.432072028	-2.698339783	-0.104423418	0.930176619	0.020045255	2.90088971
XR_013467	Macaca mulatta adaptor-related protein complex 3, beta 1 subunit (AP3B1)	AP3B1	-1.798313211	-3.478133267	0.131121483	1.095144684	0.013030609	3.175957767
XR_012912	Macaca mulatta block of proliferation 1 (LOC714024)	LOC714024	-1.242469412	-2.366031715	-0.333413907	0.793656199	0.008126971	2.981179656
XR_010680	Macaca mulatta CD180 antigen (CD180)	CD180	-1.485693145	-2.80051692	-0.016493697	0.988632544	0.03121176	2.832717714
XR_010248	Macaca mulatta CG17065-PA (LOC697051)	LOC697051	-1.339694696	-2.530977524	0.051557048	1.03638285	0.000171362	2.442126019
XR_014757	Macaca mulatta Crm, cramped-like (LOC722473)	LOC722473	-2.091834124	-4.262896782	-0.118674984	0.921033168	0.035361138	4.628385744
XR_012665	Macaca mulatta cytoplasmic beta-actin (LOC705671)	LOC705671	-1.19099776	-2.283105869	-0.518007563	0.698335605	0.000386712	3.269353376
XR_013330	Macaca mulatta deiodinase, iodothyronine, type III (DIO3)	DIO3	-2.255983472	-4.776598026	0.275770497	1.210640481	0.02761333	3.945513224
XR_014380	Macaca mulatta endothelial differentiation, lysophosphatidic acid G-protein-coupled receptor, 4 (EDG4)	EDG4	-1.291492815	-2.447812096	-0.097280057	0.934793717	0.011085207	2.618558566
XR_011279	Macaca mulatta epithelial stromal interaction 1 isoform 1 (LOC700208)	LOC700208	-1.528601475	-2.885060304	0.509286897	1.423346482	0.008487964	2.026955727
XR_012085	Macaca mulatta forkhead box M1 isoform 3 (LOC708805)	LOC708805	-1.504632989	-2.837524776	0.019363723	1.013512388	0.01119087	2.799694221
XR_014088	Macaca mulatta glucocerebrosidase precursor (LOC719103)	LOC719103	-2.168883373	-4.496752165	-0.040029101	0.972635328	0.000809088	4.623266333
XR_009675	Macaca mulatta glutamate dehydrogenase 1 (GLUD1)	GLUD1	-1.337635394	-2.527367386	-0.266967879	0.831064364	0.004351981	3.041121114
XR_013529	Macaca mulatta golgi apparatus protein 1 (LOC710037)	LOC710037	-1.169038833	-2.248618372	0.197079873	1.146375652	0.001527238	1.961502208
XR_013774	Macaca mulatta hematopoietic protein 1 (LOC705782)	LOC705782	-2.019138973	-4.053418043	-0.539207204	0.68814896	0.008238391	5.890320672
XR_010412	Macaca mulatta hypothetical protein LOC696459 (LOC696459)	LOC696459	-1.588851317	-3.008097475	-0.028009967	0.980772228	0.022488765	3.067070404
XR_011133	Macaca mulatta hypothetical protein LOC700625 (LOC700625)	LOC700625	-1.710287993	-3.272261384	-0.294417721	0.815401366	0.015574625	4.013068311
XR_012476	Macaca mulatta hypothetical protein LOC711693 (LOC711693)	LOC711693	-2.493889643	-5.632946005	-0.27222912	0.828039147	0.001746398	6.802753259
XR_012634	Macaca mulatta hypothetical protein LOC712466 (LOC712466)	LOC712466	-1.386132181	-2.613769973	-0.519473124	0.697626562	0.028303002	3.746660631
XR_012415	Macaca mulatta integrin, beta 2 (ITGB2)	ITGB2	-1.477049849	-2.78378897	0.146043671	1.106530848	0.000399027	2.515780717
XR_014226	Macaca mulatta lactotransferrin (LOC713115)	LOC713115	-3.52996698	-11.55116918	-0.45166788	0.731197032	0.017043329	15.79761496
DQ155428	Macaca mulatta LILRAb mRNA, complete cds		-1.105538899	-2.151792403	-0.02024248	0.986066958	0.040718436	2.182197046

XR_014800	Macaca mulatta lysyl hydroxylase precursor (LOC722763)	LOC722763	-2.280521878	-4.85853674	-0.087404195	0.941214731	0.007445221	5.161985444
XR_010708	Macaca mulatta macrophage expressed gene 1 (LOC700274)	LOC700274	-2.315926579	-4.979243534	-0.474643426	0.719644634	0.006455758	6.919031
XR_013439	Macaca mulatta mannosidase, alpha, class 2B, member 1 precursor (LOC716173)	LOC716173	-1.513218366	-2.854461029	-0.121990085	0.918919199	0.000908739	3.106324292
XR_014169	Macaca mulatta minichromosome maintenance deficient protein 5 (MCM5)	MCM5	-2.209410197	-4.624861613	-0.278498149	0.824448825	0.014854209	5.609640612
XR_013264	Macaca mulatta phospholipid transfer protein (PLTP)	PLTP	-1.864871602	-3.642355155	0.034736955	1.024370034	0.017137648	3.555702564
U46661	Macaca mulatta pineal arylalkylamine N-acetyltransferase (AANAT) mRNA, complete cds	AANAT mRNA	-2.917638367	-7.556082048	-0.035162136	0.975922076	0.000237229	7.742505505
XR_013476	Macaca mulatta procollagen-lysine, 2-oxoglutarate 5-dioxygenase 3 precursor (LOC714283)	LOC714283	-1.118552465	-2.171290057	-0.350007761	0.784579877	0.008582481	2.767455706
XR_012394	Macaca mulatta putative small membrane protein NID67 (LOC711300)	LOC711300	-1.574381292	-2.978077486	-0.563292514	0.67675591	0.020493999	4.400519364
XR_012351	Macaca mulatta riboflavin kinase (LOC704540)	LOC704540	-1.641796986	-3.120542771	0.212791346	1.158928326	0.031366816	2.692610665
XR_014267	Macaca mulatta selenoprotein N, 1 isoform 2 precursor (LOC719707)	LOC719707	-1.814641628	-3.517722376	0.20089908	1.149414441	0.028089771	3.060447348
XR_011741	Macaca mulatta serum		-1.987044354	-3.964240126	-0.076172043	0.94857119	0.001201559	4.179169857
XR_013743	Macaca mulatta SH2 containing inositol phosphatase isoform b (LOC717832)	LOC717832	-2.300917704	-4.927711194	-0.225327877	0.855400599	0.000877514	5.760705801
XR_010919	Macaca mulatta succinate dehydrogenase complex, subunit C isoform 1 precursor (LOC699251)	LOC699251	-1.742098802	-3.345214686	-0.015119484	0.989574696	0.043345935	3.380456976
XR_012726	Macaca mulatta ubiquitin-conjugating enzyme E2L 6 isoform 1 (LOC705561)	LOC705561	-3.372369472	-10.35581701	-0.00756925	0.994767135	0.003950135	10.41029266
XR_011110	Macaca mulatta zinc finger, DHHC domain containing 9 (ZDHHC9)	ZDHHC9	-1.112079996	-2.161570658	0.234328081	1.176358734	0.006416078	1.837509763
NM_022736	major facilitator superfamily domain containing 1 (MFSD1).	MFSD1	-3.312556261	-9.935249938	-0.056479278	0.961607945	0.001880296	10.33191332
NM_002116	major histocompatibility complex, class I, A (HLA-A).	HLA-A	-2.920465601	-7.570904139	-0.698173964	0.616351837	0.000852875	12.28341295
NM_018950	major histocompatibility complex, class I, F (HLA-F)	HLA-F	-1.429152068	-2.692883968	-0.147909258	0.902557494	0.000858162	2.983614878
NM_006120	major histocompatibility complex, class II, DM alpha (HLA-DMA)	HLA-DMA	-2.135298412	-4.393279883	-0.05699009	0.961267532	0.006313237	4.570298839
NM_033554	major histocompatibility complex, class II, DP alpha 1 (HLA-DPA1).	HLA-DPA1	-2.290191042	-4.891208764	-0.782595226	0.581320131	0.000795855	8.413967635
NM_019111	major histocompatibility complex, class II, DR alpha (HLA-DRA).	HLA-DRA	-1.752535809	-3.369502997	-0.218529983	0.859440708	0.002120542	3.920576449
NM_022555	major histocompatibility complex, class II, DR beta 3 (HLA-DRB3)	HLA-DRB3	-2.109308195	-4.314843385	-0.58624248	0.666075453	0.006166282	6.478009911
NM_021983	major histocompatibility complex, class II, DR beta 4 (HLA-DRB4).	HLA-DRB4	-2.055601608	-4.157169604	-0.818899829	0.566874065	0.000735676	7.333497616
NM_002125	major histocompatibility complex, class II, DR beta 5 (HLA-DRB5).	HLA-DRB5	-1.857738396	-3.624390482	-0.220982614	0.85798087	0.01691079	4.224325517
NM_017458	major vault protein (MVP), transcript variant 1	MVP	-2.061944287	-4.17548646	-0.337581121	0.791367037	0.013982482	5.276295656
NM_023009	MARCKS-like protein (MLP)	MLP	-2.575452285	-5.960578201	-0.031786205	0.978208425	0.008484955	6.093362163

NM_002429	matrix metalloproteinase 19 (MMP19), transcript variant rasi-1.	MMP19	-1.789715487	-3.457467013	-0.088302515	0.940628849	1.80E-05	3.675697398
NM_022468	matrix metalloproteinase 25 (MMP25), transcript variant 1	MMP25	-1.960608842	-3.892262043	-0.153027813	0.899360974	0.028276036	4.327808474
NM_145113	MAX protein (MAX), transcript variant 3	MAX	-1.390323784	-2.621375057	0.307403746	1.237478745	0.005608705	2.118319258
NM_002436	membrane protein, palmitoylated 1, 55kDa (MPP1)	MPP1	-1.835415947	-3.568742843	0.029845459	1.020902761	0.001894232	3.495673613
NM_152851	membrane-spanning 4-domains, subfamily A, member 6A (MS4A6A), transcript variant 3.	MS4A6A	-2.327689909	-5.020008856	0.916146753	1.887068447	0.000399467	2.660215566
NM_032935	metallothionein IV (MT4).	MT4	-3.212767071	-9.27127061	0.230509057	1.173248859	0.002260267	7.902220011
NM_005955	metal-regulatory transcription factor 1 (MTF1)	MTF1	-1.575649982	-2.980697528	-0.058008014	0.960589528	0.028513404	3.102987739
NM_001004431	meteorin, glial cell differentiation regulator-like (METRNL).	METRNL	-2.24270429	-4.732833896	-0.054893949	0.962665205	0.008720791	4.916386164
NM_182796	methionine adenosyltransferase II, beta (MAT2B), transcript variant 2	MAT2B	-2.230553989	-4.693141599	0.015722668	1.010957724	0.000325636	4.642272856
NM_012331	methionine sulfoxide reductase A (MSRA)	MSRA	-2.213854673	-4.639131282	-0.086585113	0.941749252	0.001276264	4.926079075
NM_002403	microfibrillar-associated protein 2 (MFAP2), transcript variant 2.	MFAP2	-2.168136417	-4.494424573	0.515940069	1.429925585	0.009376766	3.143117811
NM_016498	mitochondrial protein 18 kDa (MTP18)	MTP18	-1.59169246	-3.01402725	0.287808223	1.220784223	0.007888694	2.468927098
NM_002949	mitochondrial ribosomal protein L12 (MRPL12), nuclear gene encoding mitochondrial protein	MRPL12	-1.76759984	-3.404870291	0.108153538	1.077847848	0.007455488	3.158952627
NM_023937	mitochondrial ribosomal protein L34 (MRPL34), nuclear gene encoding mitochondrial protein.	MRPL34	-1.329153873	-2.512552728	-0.171508369	0.887913862	0.001320523	2.829725758
NM_032477	mitochondrial ribosomal protein L41 (MRPL41), nuclear gene encoding mitochondrial protein	MRPL41	-1.386432501	-2.614314128	-0.209678182	0.864730103	0.001033059	3.023271792
NM_018141	mitochondrial ribosomal protein S10 (MRPS10), nuclear gene encoding mitochondrial protein.	MRPS10	-1.650968839	-3.140444643	-0.069992526	0.952642933	0.01842559	3.296560057
NM_021107	mitochondrial ribosomal protein S12 (MRPS12), nuclear gene encoding mitochondrial protein, transcript variant 1	MRPS12	-1.603432963	-3.038655165	-0.206200049	0.86681736	0.001056637	3.505531046
NM_015971	mitochondrial ribosomal protein S7 (MRPS7), nuclear gene encoding mitochondrial protein.	MRPS7	-1.277576965	-2.424314663	-0.43328157	0.740575347	0.004109009	3.273555719
NM_002745	mitogen-activated protein kinase 1 (MAPK1), transcript variant 1.	MAPK1	-1.757546079	-3.381225134	-0.234362103	0.850060776	0.011142319	3.977627518
NM_002755	mitogen-activated protein kinase kinase 1 (MAP2K1).	MAP2K1	-1.242241735	-2.365658353	0.269888145	1.205714342	0.000949704	1.962038826
NM_021970	mitogen-activated protein kinase kinase 1 interacting protein 1 (MAP2K1IP1)	MAP2K1IP1	-1.379925322	-2.602548991	-0.37553047	0.770821934	0.04142221	3.376329702
CB549220	MMPL0027_F12 MMPL Macaca mulatta cDNA sequence		-1.827181953	-3.548432721	-0.082332359	0.944529421	0.00790584	3.756826037
CB553994	MMSP0053_A12 MMSP Macaca mulatta cDNA sequence		-2.267666987	-4.815437862	-0.056978302	0.961275386	0.012603311	5.009425949
CB551618	MMSP0057_A06 MMSP Macaca mulatta cDNA sequence		-1.449567901	-2.731262356	-0.768749228	0.586926101	0.017839905	4.653502975
AL832403	mRNA; cDNA DKFZp667B1913 (from clone DKFZp667B1913)	from clone DKFZp667B1913	-2.465104142	-5.521667984	-0.228009155	0.853812296	0.002858948	6.467074797
CR936794	mRNA; cDNA DKFZp781I14186 (from clone DKFZp781I14186)	from clone DKFZp781I14186	-2.491664905	-5.624266297	0.099686217	1.07154038	0.006538659	5.248767479
NM_153259	mucolipin 2 (MCOLN2).	MCOLN2	-5.417700711	-42.74550196	-0.233928706	0.850316179	0.002284242	50.27012658

NM_015488	myofibrillogenesis regulator 1 (MR-1)	MR-1	-1.796391905	-3.473504351	-0.135831954	0.910144832	0.010085151	3.816430341
NM_004998	myosin IE (MYO1E).	MYO1E	-2.288814769	-4.88654497	-0.585964773	0.666203679	0.009565933	7.334911412
NM_012335	myosin IF (MYO1F).	MYO1F	-2.007946893	-4.022094262	-0.157747718	0.896423442	0.002624571	4.486824053
NM_145808	myotrophin (MTPN)	MTPN	-1.537246978	-2.902401241	-0.77187244	0.585656871	0.040672629	4.955804987
NM_002356	myristoylated alanine-rich protein kinase C substrate (MARCKS)	MARCKS	-1.876127424	-3.670883741	0.397033946	1.316797906	0.001335545	2.787735099
NM_144603	NADPH oxidase organizer 1 (NOXO1), transcript variant a.	NOXO1	-3.471469807	-11.09217061	0.394536274	1.314520165	0.035074865	8.43818977
NM_006153	NCK adaptor protein 1 (NCK1)	NCK1	-1.426254965	-2.687481763	0.116122624	1.083818084	0.018720108	2.479642852
NM_016118	NEDD8 ultimate buster-1 (NYREN18).	NYREN18	-1.596515112	-3.024119422	-0.058240663	0.960434636	0.016452439	3.148698838
NM_014062	nin one binding protein (NOB1P).	NOB1P	-1.469162685	-2.768611616	-0.113152759	0.924565376	0.037984881	2.994500647
NM_004148	ninjurin 1 (NINJ1).	NINJ1	-2.357055002	-5.123234763	0.000495405	1.000343448	0.000351534	5.121475805
NM_001008860	non imprinted in Prader-Willi/Angelman syndrome 2 (NIPA2), transcript variant 2.	NIPA2	-1.456329105	-2.744092483	-0.461834435	0.726062459	0.044650294	3.779416563
NM_001001716	nuclear factor of kappa light polypeptide gene enhancer in B-cells inhibitor, beta (NFKBIB), transcript variant 2.	NFKBIB	-2.242155587	-4.73103419	0.046328535	1.032633663	0.022794458	4.581522332
NM_004556	nuclear factor of kappa light polypeptide gene enhancer in B-cells inhibitor, epsilon (NFKBIE).	NFKBIE	-2.889714382	-7.41123711	-0.05185849	0.964692804	0.002425669	7.682484079
NM_013392	nuclear receptor binding protein (NRBP)	NRBP	-1.218574988	-2.327167394	-0.674738154	0.626445907	0.002049542	3.714873649
NM_005437	nuclear receptor coactivator 4 (NCOA4).	NCOA4	-1.604598952	-3.041112004	0.175100658	1.129043176	0.016931152	2.693530299
NM_005693	nuclear receptor subfamily 1, group H, member 3 (NR1H3)	NR1H3	-2.748202921	-6.718796904	-0.307462171	0.808061963	0.001113706	8.314705075
NM_004741	nucleolar and coiled-body phosphoprotein 1 (NOLC1)	NOLC1	-1.526541705	-2.880944175	-0.375363287	0.770911264	0.012254603	3.737063279
NM_015441	olfactomedin-like 2B (OLFML2B).	OLFML2B	-2.067974499	-4.192975787	0.133205523	1.096727815	0.013521164	3.823169003
NM_015878	ornithine decarboxylase antizyme inhibitor (OAZIN), transcript variant 1	OAZIN	-1.507910275	-2.843977941	-0.091551377	0.938512993	0.00307241	3.030302151
NM_013439	paired immunoglobin-like type 2 receptor alpha (PILRA), transcript variant 1.	PILRA	-2.360563435	-5.135708916	-0.411819367	0.751674847	0.002610982	6.83235436
NM_016307	paired related homeobox 2 (PRRX2)	PRRX2	-1.778808371	-3.431426306	0.001230546	1.000853314	0.001354997	3.42850072
NM_001003828	parvin, beta (PARVB), transcript variant 1.	PARVB	-3.29168098	-9.792525498	-0.513586475	0.700478912	0.007367523	13.97975774
NM_022141	parvin, gamma (PARVG)	PARVG	-2.036044851	-4.101196449	-0.221788531	0.857501719	0.004294554	4.782726794
NM_002596	PCTAIRE protein kinase 3 (PCTK3), transcript variant 3.	PCTK3	-1.953163923	-3.872228056	0.289184721	1.221949549	0.018346341	3.16889356
NM_015545	pentatricopeptide repeat domain 1 (PTCD1).	PTCD1	-1.648416713	-3.13489411	-1.150000728	0.450625004	0.048679565	6.956769114
NM_004160	peptide YY (PYY)	PYY	-1.721368253	-3.297489929	0.096007991	1.068811911	0.025280155	3.085191974
NM_006475	periostin, osteoblast specific factor (POSTN).	POSTN	-2.508769677	-5.691345163	-0.370112616	0.773722098	0.013227529	7.355800198
NM_006793	peroxiredoxin 3 (PRDX3), nuclear gene encoding mitochondrial protein, transcript variant 1.	PRDX3	-1.350000787	-2.549122646	-0.373143757	0.772098194	0.028965411	3.301552402
NM_004461	phenylalanine-tRNA synthetase-like, alpha subunit (FARSLA)	FARSLA	-1.800198772	-3.482682057	-0.271066666	0.82870661	0.000224305	4.202551321
NM_177543	phosphatidic acid phosphatase type 2C (PPAP2C), transcript variant 3	PPAP2C	-1.974435793	-3.929745256	-0.455925215	0.72904248	0.037230892	5.39028296
NM_002627	phosphofructokinase, platelet (PFKP).	PFKP	-1.424348045	-2.683931861	0.207110917	1.154374164	0.00500983	2.325010334
NM_002631	phosphogluconate dehydrogenase (PGD).	PGD	-2.181194491	-4.535289015	-0.285817209	0.820276835	0.000487849	5.528973662
NM_015900	phospholipase A1 member A (PLA1A).	PLA1A	-2.212337557	-4.634255405	-0.149162931	0.901773531	0.011980347	5.13904572
NM_002662	phospholipase D1, phophatidylcholine-specific (PLD1).	PLD1	-1.88078277	-3.682748226	-0.151076793	0.900578042	0.023050276	4.089316035
NM_000303	phosphomannomutase 2 (PMM2).	PMM2	-2.674920348	-6.386034503	-0.451703483	0.731178988	0.002486846	8.733886789
NM_002764	phosphoribosyl pyrophosphate synthetase 1 (PRPS1)	PRPS1	-1.43146257	-2.697200125	0.345312434	1.27042608	0.024099052	2.123067346

NM_006452	phosphoribosylaminoimidazole carboxylase, phosphoribosylaminoimidazole succinocarboxamide synthetase (PAICS).	PAICS	-1.521594625	-2.871082178	-0.375655987	0.770754874	0.011561727	3.725026301
NM_002632	placental growth factor, vascular endothelial growth factor-related protein (PGF)	PGF	-1.574151818	-2.977603834	0.27726155	1.211892348	0.02067171	2.456987074
NM_000930	plasminogen activator, tissue (PLAT), transcript variant 1.	PLAT	-1.500185735	-2.828791285	-0.215431399	0.861288576	0.017487856	3.284371073
NM_002658	plasminogen activator, urokinase (PLAU).	PLAU	-2.717697884	-6.578222854	-0.360389148	0.778954439	0.031148211	8.444939173
BM423313	PLATE4_C07 Rhesus Macaca mulatta cDNA sequence		-1.304363945	-2.469748172	0.069242841	1.049165913	0.003381737	2.354011069
NM_002620	platelet factor 4 variant 1 (PF4V1).	PF4V1	-2.075279893	-4.214261662	-0.614459739	0.653174449	0.002500279	6.45196956
NM_000952	platelet-activating factor receptor (PTAFR).	PTAFR	-3.44675835	-10.90379433	-0.068217724	0.953815595	0.037862893	11.43176353
NM_002664	pleckstrin (PLEK).	PLEK	-3.529454723	-11.54706844	-0.51681683	0.698912217	0.004434112	16.52148605
NM_032242	plexin A1 (PLXNA1).	PLXNA1	-2.152976178	-4.447443194	-1.036509033	0.487505691	0.029652432	9.122853892
NM_007221	polyamine-modulated factor 1 (PMF1).	PMF1	-1.598834123	-3.028984348	0.080026803	1.057037679	0.014891091	2.865540566
NM_018992	potassium channel tetramerisation domain containing 5 (KCTD5)	KCTD5	-1.615141497	-3.063416435	0.042712701	1.030048809	0.005054829	2.974049782
NM_002250	potassium intermediate/small conductance calcium-activated channel, subfamily N, member 4 (KCNN4).	KCNN4	-1.777460697	-3.428222381	0.293839813	1.225898737	0.00045707	2.796497197
NM_002247	potassium large conductance calcium-activated channel, subfamily M, alpha member 1 (KCNMA1)	KCNMA1	-1.909730752	-3.757389696	0.441528745	1.358042609	0.00942713	2.766768635
NM_005472	potassium voltage-gated channel, Isk-related family, member 3 (KCNE3).	KCNE3	-1.699994767	-3.2489978	-0.13741611	0.909145993	0.002119408	3.573681043
NM_080671	potassium voltage-gated channel, Isk-related family, member 4 (KCNE4).	KCNE4	-1.833228775	-3.563336614	0.342541081	1.26798799	0.025753185	2.810228993
NM_006235	POU domain, class 2, associating factor 1 (POU2AF1).	POU2AF1	-2.920083491	-7.568899183	0.049340143	1.034791524	0.001249923	7.314419383
NM_001198	PR domain containing 1, with ZNF domain (PRDM1), transcript variant 1.	PRDM1	-1.4415328	-2.716092852	-0.281812511	0.822556959	0.043076802	3.302011882
NM_013370	pregnancy-induced growth inhibitor (OKL38)	OKL38	-1.51375057	-2.855514221	-0.276867388	0.825381274	0.003761838	3.459630492
NM_006228	prepronociceptin (PNOC).	PNOC	-2.366834233	-5.158080299	0.101834508	1.073137179	0.005311607	4.806543281
NM_014117	PRO0149 protein (PRO0149)	PRO0149	-1.314095994	-2.486464776	-0.035775211	0.975507445	0.00767288	2.548893694
NM_016459	proapoptotic caspase adaptor protein (PACAP).	PACAP	-2.406595752	-5.302217135	-0.025717463	0.982331956	0.016939283	5.39758185
NM_002593	procollagen C-endopeptidase enhancer (PCOLCE)	PCOLCE	-2.047009219	-4.132483957	-0.759391351	0.590745504	0.013986102	6.995370985
NM_002598	programmed cell death 2 (PDCD2), transcript variant 1	PDCD2	-1.288298669	-2.442398602	-0.219577959	0.858816635	0.000929129	2.843911613
NM_033102	prostate cancer associated protein 6 (PCANAP6).	PCANAP6	-1.769892219	-3.410284782	-0.392842514	0.761627505	0.005936038	4.477628182
NM_214710	protease, serine-like 1 (PRSSL1).	PRSSL1	-1.49825035	-2.824998987	-0.331315093	0.794811641	0.011336034	3.554299965
NM_212535	protein kinase C, beta 1 (PRKCB1), transcript variant 1	PRKCB1	-1.217983948	-2.326214201	-0.345274475	0.787158204	0.003019462	2.955205431
NM_002738	protein kinase C, beta 1 (PRKCB1), transcript variant 2	PRKCB1	-1.789849022	-3.45778705	0.594396437	1.509840799	0.005710518	2.290166653
NM_002827	protein tyrosine phosphatase, non-receptor type 1 (PTPN1)	PTPN1	-1.529170715	-2.88619888	-0.207613303	0.865968647	0.001135559	3.33291383
NM_080588	protein tyrosine phosphatase, non-receptor type 7 (PTPN7), transcript variant 2	PTPN7	-1.249853336	-2.378172454	-0.383502328	0.766574372	0.019354705	3.102337544
NM_080792	protein tyrosine phosphatase, non-receptor type substrate 1 (PTPNS1).	PTPNS1	-1.159179311	-2.233303482	0.054896919	1.038784879	0.026688511	2.149919128
NM_002727	proteoglycan 1, secretory granule (PRG1).	PRG1	-2.097802805	-4.280569663	0.405181535	1.324255531	8.34E-05	3.2324348
NM_002560	purinergic receptor P2X, ligand-gated ion channel, 4 (P2RX4), transcript variant 1.	P2RX4	-1.964153999	-3.901838321	-0.17785491	0.884016431	0.0278424	4.413762214

NM_002561	purinergic receptor P2X, ligand-gated ion channel, 5 (P2RX5), transcript variant 1.	P2RX5	-2.425807498	-5.373296684	0.458518716	1.374130209	0.001102057	3.91032571
NM_013237	px19-like protein (PX19)	PX19	-1.226437358	-2.339884564	-0.024852562	0.982921044	0.003357399	2.380541732
NM_013258	PYD and CARD domain containing (PYCARD), transcript variant 1.	PYCARD	-2.069947018	-4.198712538	0.232962449	1.175245738	0.006166086	3.572625197
NM_176798	pyrimidinergic receptor P2Y, G-protein coupled, 6 (P2RY6), transcript variant 2	P2RY6	-1.967279677	-3.910301033	-0.290818606	0.817438101	0.002777496	4.783605057
NM_017817	RAB20, member RAS oncogene family (RAB20).	RAB20	-2.992767307	-7.959993798	-1.356653234	0.390487093	0.043396655	20.38478083
NM_006868	RAB31, member RAS oncogene family (RAB31).	RAB31	-1.424199152	-2.68365488	-0.611513746	0.654509598	0.000247518	4.100252903
NM_006834	RAB32, member RAS oncogene family (RAB32).	RAB32	-1.463173833	-2.757142499	0.261833922	1.199001881	0.007377252	2.299531422
NM_201434	RAB5C, member RAS oncogene family (RAB5C), transcript variant 1	RAB5C	-1.705460334	-3.261329786	0.053041024	1.037449437	0.000962843	3.143603601
NM_003929	RAB7, member RAS oncogene family-like 1 (RAB7L1).	RAB7L1	-2.368369908	-5.163573735	0.253086883	1.191754343	0.031929563	4.332750089
NM_015263	rabconnectin-3 (RC3)	RC3	-1.594609035	-3.020126616	-0.28458393	0.820978343	0.017279616	3.678692188
NM_006266	ral guanine nucleotide dissociation stimulator (RALGDS)	RALGDS	-1.298945949	-2.460490505	-0.071132706	0.951890345	0.021288414	2.584846583
NM_001664	ras homolog gene family, member A (RHOA)	RHOA	-1.624758371	-3.083905101	-1.030960818	0.489384116	0.010344271	6.301604402
NM_175744	ras homolog gene family, member C (RHOC)	RHOC	-1.185919262	-2.27508313	-0.314862117	0.803927821	0.000267799	2.829959446
NM_004310	ras homolog gene family, member H (RHOH).	RHOH	-1.542872615	-2.913740943	-0.282597965	0.822109252	0.014685104	3.544225948
NM_021205	ras homolog gene family, member U (RHOU).	RHOU	-2.26996198	-4.823104203	-0.065909437	0.955342905	0.013919862	5.048558144
NM_005754	Ras-GTPase-activating protein SH3-domain-binding protein (G3BP), transcript variant 1	G3BP	-1.164050747	-2.240857249	0.186096837	1.137681589	0.001834915	1.96966996
NM_015444	Ras-induced senescence 1 (RIS1)	RIS1	-1.229669742	-2.345132995	0.173181825	1.127542509	0.000220048	2.079862156
NM_003821	receptor-interacting serine-threonine kinase 2 (RIPK2).	RIPK2	-1.667383884	-3.176380808	-0.102509371	0.931411519	0.000903877	3.410287231
NM_006871	receptor-interacting serine-threonine kinase 3 (RIPK3).	RIPK3	-1.516454672	-2.860871448	-1.208406914	0.432746209	0.049481041	6.610968253
NM_002922	regulator of G-protein signalling 1 (RGS1).	RGS1	-1.756193113	-3.378055692	0.43796152	1.354688844	0.018043622	2.493602651
NM_002928	regulator of G-protein signalling 16 (RGS16)	RGS16	-2.077726387	-4.221414187	-0.075655949	0.948910582	0.007932695	4.448695447
NM_005873	regulator of G-protein signalling 19 (RGS19)	RGS19	-1.333853494	-2.520750788	-0.619066903	0.6510919	0.005556366	3.871574483
NM_002910	renin binding protein (RENBP)	RENBP	-3.006882766	-8.038257345	-0.66731417	0.629677852	0.021053232	12.76566631
NM_005615	ribonuclease, RNase A family, k6 (RNASE6).	RNASE6	-2.305789059	-4.944378058	-0.675103464	0.626287303	0.029763968	7.894744207
NM_016093	ribosomal protein L26-like 1 (RPL26L1).	RPL26L1	-1.207786328	-2.309829444	0.093282374	1.066794561	0.002442311	2.165205494
NM_003942	ribosomal protein S6 kinase, 90kDa, polypeptide 4 (RPS6KA4)	RPS6KA4	-1.564327588	-2.957396313	0.221104666	1.16562576	0.002167745	2.537174806
NM_203434	RIKEN cDNA 2610524G09 (LOC389792)	LOC389792	-2.301487812	-4.929658854	0.225887153	1.169496183	0.004349176	4.215198756
NM_145266	RIKEN cDNA 2700047N05 (LOC134492)	LOC134492	-1.212836394	-2.317929028	-0.442117666	0.736053396	0.026029865	3.149131626
NM_001012754	RIKEN cDNA 8030451K01 (LOC387921), transcript variant 1.	LOC387921	-1.937155495	-3.829498558	-0.835319725	0.560458818	0.016381448	6.832792052
XM_496823	RIKEN cDNA A630077B13 gene; RIKEN cDNA 2810048G17 (LOC441168)	LOC441168	-4.226526541	-18.72023371	-0.207223034	0.866202935	0.013987397	21.61183361
NM_018434	ring finger protein 130 (RNF130)	RNF130	-2.80587324	-6.992814526	-0.136107921	0.909970751	0.004200682	7.684658563
NM_080388	S100 calcium binding protein A16 (S100A16).	S100A16	-1.315209849	-2.488385231	-0.604938909	0.657499221	0.019857136	3.784620803
NM_005978	S100 calcium binding protein A2 (S100A2)	S100A2	-1.956950603	-3.882404947	0.029982718	1.020999895	0.005700335	3.802551758
NM_005980	S100 calcium binding protein P (S100P).	S100P	-2.504879383	-5.676018858	0.201327787	1.149756049	0.000917721	4.936715806
NM_000687	S-adenosylhomocysteine hydrolase (AHCY)	AHCY	-1.542544244	-2.913077824	-0.045410648	0.969013958	0.028889117	3.00622896
NM_006808	Sec61 beta subunit (SEC61B)	SEC61B	-1.339781431	-2.531129691	-0.09317018	0.937460507	0.002594633	2.699985409
NM_001012456	Sec61 gamma subunit (SEC61G), transcript variant 2.	SEC61G	-1.995863669	-3.988548079	0.758816518	1.692101978	0.001136913	2.357155852
NM_003004	secreted and transmembrane 1 (SECTM1).	SECTM1	-5.413680982	-42.62656749	-0.65540894	0.634895501	0.000496843	67.13950157

NM_054023	secretoglobin, family 3A, member 2 (SCGB3A2).	SCGB3A2	-1.878302707	-3.676422843	0.141253344	1.102862814	0.007046495	3.333526888
NM_052837	secretory carrier membrane protein 3 (SCAMP3), transcript variant 2.	SCAMP3	-1.218743651	-2.327439474	-0.237111993	0.848442036	0.003284318	2.74319208
NM_182566	secretory protein LOC284013 (LOC284013)	LOC284013	-2.561378069	-5.902712474	-0.109571484	0.926863322	8.32E-06	6.368482098
NM_003006	selectin P ligand (SELPLG).	SELPLG	-2.691849495	-6.461412118	-0.194837906	0.873671052	0.00596021	7.395703572
NM_018445	selenoprotein S (SELS), transcript variant 2	SELS	-1.533936443	-2.895748772	-0.156594926	0.897140019	0.001019717	3.227755656
NM_013376	SERTA domain containing 1 (SERTAD1).	SERTAD1	-2.191350141	-4.56732718	-0.096190345	0.935500063	0.006608934	4.882230758
NM_000331	serum amyloid A1 (SAA1), transcript variant 1.	SAA1	-2.887059705	-7.397612364	0.194362723	1.144218617	0.024639566	6.465208882
NM_017827	seryl-tRNA synthetase 2 (SARS2).	SARS2	-2.302127549	-4.931845309	-1.040958702	0.486004406	0.028506922	10.14773786
NM_031459	sestrin 2 (SESN2)	SESN2	-1.897658139	-3.726078682	-0.300941436	0.811722531	0.001584371	4.590335415
NM_145064	SH3 and cysteine rich domain 3 (STAC3).	STAC3	-1.401734102	-2.642189793	-0.341529349	0.789204261	0.006354359	3.347916277
NM_031286	SH3 domain binding glutamic acid-rich protein like 3 (SH3BGRL3)	SH3BGRL3	-1.449985048	-2.732052198	-0.649337255	0.637573135	0.035736496	4.285080485
NM_138392	SH3KBP1 binding protein 1 (SHKBP1).	SHKBP1	-1.808815836	-3.503545995	-1.101860254	0.465915343	0.04420332	7.519705128
NM_014450	SHP2-interacting transmembrane adaptor protein (SIT)	SIT	-2.002265339	-4.006285788	-0.194129715	0.874100025	0.006130338	4.583326477
NM_178867	sideroflexin 4 (SFXN4), transcript variant 2	SFXN4	-1.505951142	-2.840118532	0.066528869	1.047194096	0.015284802	2.712122368
NM_021203	signal recognition particle receptor, B subunit (SRPRB)	SRPRB	-1.702043925	-3.253615852	0.197579891	1.146773038	0.015579462	2.837192491
NM_007315	signal transducer and activator of transcription 1, 91kDa (STAT1), transcript variant alpha	STAT1	-1.769171409	-3.408581336	0.334588209	1.261017423	0.010075334	2.703040635
NM_139266	signal transducer and activator of transcription 1, 91kDa (STAT1), transcript variant beta	STAT1	-3.275122971	-9.680777837	0.293529593	1.225635163	0.002159289	7.89858037
NM_003037	signaling lymphocytic activation molecule family member 1 (SLAMF1)	SLAMF1	-1.558330952	-2.945129254	0.303103964	1.233796077	0.001738061	2.387047025
NM_018556	signal-regulatory protein beta 2 (SIRPB2), transcript variant 1.	SIRPB2	-1.945949678	-3.852913183	-0.373061963	0.772141969	0.018758792	4.989902552
NM_003864	sin3-associated polypeptide, 30kDa (SAP30)	SAP30	-1.507775171	-2.843711624	-0.787031621	0.579535274	0.005892683	4.906882728
NM_006396	Sjogren's syndrome/scleroderma autoantigen 1 (SSSCA1)	SSSCA1	-2.226722369	-4.680693729	-0.409548396	0.752859003	0.004921003	6.217224888
NM_020125	SLAM family member 8 (SLAMF8).	SLAMF8	-4.076626306	-16.87278604	-0.560897344	0.677880397	0.004975323	24.89050594
NM_003093	small nuclear ribonucleoprotein polypeptide C (SNRPC)	SNRPC	-1.459562244	-2.750249004	-0.197826044	0.87186336	0.002795754	3.154449574
NM_006555	SNARE protein Ykt6 (YKT6)	YKT6	-1.263442884	-2.400679622	-0.241367181	0.845943268	0.00104791	2.837873074
NM_198857	sodium- and chloride-dependent creatine transporter (FLJ43855)	FLJ43855	-2.128449065	-4.372471756	-0.062303739	0.95773356	0.005612814	4.565436504
NM_001037	sodium channel, voltage-gated, type I, beta (SCN1B), transcript variant a	SCN1B	-2.380569892	-5.207424047	-0.26564261	0.831828137	8.16E-06	6.260216284
NM_016582	solute carrier family 15, member 3 (SLC15A3)	SLC15A3	-1.94683235	-3.855271199	-0.284634297	0.820949682	0.00984836	4.696111447
NM_153811	solute carrier family 38, member 6 (SLC38A6)	SLC38A6	-1.848534343	-3.601341331	-0.452348438	0.730852188	0.04075716	4.927591909
NM_014096	solute carrier family 43, member 3 (SLC43A3).	SLC43A3	-2.050005243	-4.141074746	-0.52603974	0.694458438	0.023079856	5.963027471
NM_016354	solute carrier organic anion transporter family, member 4A1 (SLCO4A1).	SLCO4A1	-2.376934345	-5.19431802	-0.0447423	0.969462971	0.020012123	5.357933388
NM_013322	sorting nexin 10 (SNX10).	SNX10	-4.892668383	-29.70571036	-0.771175313	0.585939936	0.021337584	50.69753495
NM_003901	sphingosine-1-phosphate lyase 1 (SGPL1)	SGPL1	-2.314842915	-4.975504836	0.045019492	1.031697118	0.035381222	4.822641014
NM_032038	spinster (SPIN1).	SPIN1	-2.032064435	-4.089896786	-0.780657317	0.582101517	0.009487575	7.026088519
NM_003177	spleen tyrosine kinase (SYK)	SYK	-3.137327935	-8.798929025	0.460643712	1.376155704	0.000542373	6.393847003
NM_014390	staphylococcal nuclease domain containing 1 (SND1)	SND1	-1.402294048	-2.643215493	-0.05436974	0.963015057	4.42E-05	2.744729145
NM_001008410	STEAP family member 3 (STEAP3), transcript variant 3.	STEAP3	-2.214716542	-4.641903535	-0.091765624	0.938373629	0.048644907	4.946754033

NM_002975	stem cell growth factor; lymphocyte secreted C-type lectin (SCGF)	SCGF	-1.594182859	-3.019234595	-0.381593083	0.767589519	0.015155877	3.93339737
NM_003578	sterol O-acyltransferase 2 (SOAT2).	SOAT2	-1.977064521	-3.936912154	-0.417673171	0.74863107	0.000743678	5.258814806
NM_012447	stromal antigen 3 (STAG3)	STAG3	-1.516431079	-2.860824663	-0.214238005	0.862001327	0.035687022	3.318817006
NM_022044	stromal cell-derived factor 2-like 1 (SDF2L1)	SDF2L1	-1.84579098	-3.594499691	0.156937306	1.114917768	0.001020736	3.224004312
NM_003002	succinate dehydrogenase complex, subunit D, integral membrane protein (SDHD), nuclear gene encoding mitochondrial protein.	SDHD	-1.771248712	-3.413492808	-0.485659765	0.714170395	0.024454781	4.779661593
NM_033050	succinate receptor 1 (SUCNR1).	SUCNR1	-2.13919471	-4.405160886	0.69233931	1.615901557	0.001760133	2.726131964
NM_018837	sulfatase 2 (SULF2), transcript variant 1	SULF2	-3.644709819	-12.50739832	-0.190259704	0.876447935	0.028931915	14.27055484
NM_003745	suppressor of cytokine signaling 1 (SOCS1).	SOCS1	-3.313242263	-9.939975274	0.568363642	1.482840724	0.011041865	6.703333079
NM_033161	surfeit 4 (SURF4)	SURF4	-1.598063293	-3.027366398	-0.395114722	0.760428905	0.035790246	3.981130092
NM_003073	SWI/SNF related, matrix associated, actin dependent regulator of chromatin, subfamily b, member 1 (SMARCB1)	SMARCB1	-1.265803722	-2.40461133	-0.267449489	0.830786979	0.020076239	2.894377728
NM_006455	synaptonemal complex protein SC65 (SC65)	SC65	-1.676702077	-3.196963065	-0.262578167	0.833596909	0.003055066	3.835142659
NM_205848	synaptotagmin VI (SYT6)	SYT6	-1.395011077	-2.629905713	0.058806619	1.041603802	0.026556344	2.524861862
NM_016305	synovial sarcoma translocation gene on chromosome 18-like 2 (SS18L2)	SS18L2	-1.477527935	-2.784711625	0.396982333	1.316750798	0.009645353	2.114835723
NM_003764	syntaxin 11 (STX11).	STX11	-2.142510583	-4.415297297	-0.997106727	0.501003738	0.010549855	8.8129029
NM_177424	syntaxin 12 (STX12).	STX12	-1.581720825	-2.993266693	-0.022010981	0.984858947	0.007636618	3.039284664
CO646475	TAPBP		-2.057143902	-4.161616148	-0.173189577	0.886879757	1.21E-05	4.692424327
NM_006019	T-cell, immune regulator 1, ATPase, H+ transporting, lysosomal V0 protein a isoform 3 (TCIRG1), transcript variant 1	TCIRG1	-1.637142615	-3.11049161	-0.564538004	0.676171913	0.002136976	4.60014909
NM_015926	testis expressed sequence 264 (TEX264)	TEX264	-1.297668123	-2.45831216	-0.59814654	0.660602099	0.03156774	3.721320538
NM_030927	tetraspanin 14 (TSPAN14).	TSPAN14	-1.861813725	-3.634643145	0.107165792	1.077110148	0.002364062	3.374439606
NM_003330	thioredoxin reductase 1 (TXNRD1), transcript variant 1	TXNRD1	-2.314489763	-4.974287048	-0.264327864	0.832586537	0.004035907	5.974498535
NM_005782	THO complex 4 (THOC4)	THOC4	-1.309236778	-2.478104074	0.282697137	1.216466961	0.005078414	2.037132248
NM_016381	three prime repair exonuclease 1 (TREX1), transcript variant 1	TREX1	-2.506656722	-5.683015783	-0.337896011	0.791194328	0.000965717	7.182831801
NM_003246	thrombospondin 1 (THBS1).	THBS1	-2.367174315	-5.159296343	0.083623545	1.059676237	0.000176484	4.868747796
NM_003247	thrombospondin 2 (THBS2).	THBS2	-2.752317172	-6.737984793	-0.525088434	0.694916512	0.014948826	9.696106908
NM_003258	thymidine kinase 1, soluble (TK1).	TK1	-2.062417086	-4.176855073	0.263206457	1.200143117	0.006150781	3.480297486
XR_000287	Tmp21-II , transcribed pseudogene (Tmp21-II), misc RNA	Tmp21-II	-2.156656147	-4.458802035	-0.512164164	0.701169835	0.027613695	6.359089928
NM_003264	toll-like receptor 2 (TLR2).	TLR2	-2.402846622	-5.288456156	-0.234213002	0.850148634	0.000795298	6.220625368
NM_022371	torsin family 3, member A (TOR3A).	TOR3A	-1.955169638	-3.877615186	-0.449029391	0.732535514	0.001488144	5.29341597
NM_006700	TRAF-type zinc finger domain containing 1 (TRAFD1).	TRAFD1	-1.161762	-2.237305086	-0.251676252	0.839919954	0.009662756	2.663712267
NM_006755	transaldolase 1 (TALDO1)	TALDO1	-2.212388121	-4.634417831	-0.345578158	0.786992527	0.013380398	5.888769805
NM_012252	transcription factor EC (TFEC), transcript variant 1.	TFEC	-3.112665621	-8.649793082	-0.465368263	0.724286172	0.016702289	11.94250756
NM_001063	transferrin (TF)	TF	-1.168209687	-2.247326417	0.217493617	1.16271186	0.001125347	1.932831765
NM_001001188	transient receptor potential cation channel, subfamily M, member 2 (TRPM2), transcript variant S.	TRPM2	-2.341234521	-5.067360686	-0.163780201	0.892682964	0.005475311	5.676551352
NM_014220	transmembrane 4 L six family member 1 (TM4SF1).	TM4SF1	-1.576691231	-2.982849589	-0.25959426	0.835322811	0.016294581	3.570894451
NM_014313	transmembrane protein 50A (TMEM50A).	TMEM50A	-1.606728777	-3.04560486	-0.467332079	0.723300934	0.013777273	4.210702234
NM_018022	transmembrane protein 51 (TMEM51).	TMEM51	-2.515516971	-5.718025172	-0.256557986	0.83708267	0.001253259	6.830896612

NM_018965	triggering receptor expressed on myeloid cells 2 (TREM2).	TREM2	-3.15222847	-8.890277612	-0.238414069	0.847676638	0.000484748	10.48781719
NM_014788	tripartite motif-containing 14 (TRIM14), transcript variant 1.	TRIM14	-1.296201538	-2.455814411	-0.028579931	0.980384832	0.007583632	2.504949414
NM_003449	tripartite motif-containing 26 (TRIM26).	TRIM26	-1.542117918	-2.912217117	-0.286615713	0.819822953	0.021908314	3.552251259
NM_006355	tripartite motif-containing 38 (TRIM38)	TRIM38	-1.64513923	-3.12778041	-0.139091358	0.908090911	0.017564176	3.444347224
NM_032681	tripartite motif-containing 51 (TRIM51).	TRIM51	-1.661495523	-3.163442828	0.232130075	1.174567867	0.012228597	2.693282285
NM_173547	tripartite motif-containing 65 (TRIM65).	TRIM65	-1.512870944	-2.853773716	0.146393458	1.106799163	0.003938386	2.578402488
NM_003282	troponin I, skeletal, fast (TNNI2)	TNNI2	-1.289161495	-2.443859753	-0.046206465	0.96847958	0.009151016	2.523398329
NM_004184	tryptophanyl-tRNA synthetase (WARS), transcript variant 1	WARS	-3.680062724	-12.81767531	-0.920415959	0.528356662	0.026920758	24.25951299
NM_005726	Ts translation elongation factor, mitochondrial (TSFM)	TSFM	-2.127908935	-4.370835053	-0.526787979	0.694098358	0.001317881	6.297140749
NM_153712	tubulin tyrosine ligase (TTL).	TTL	-2.885803323	-7.391172901	-0.326711646	0.797351829	0.017338072	9.269650657
NM_016639	tumor necrosis factor receptor superfamily, member 12A (TNFRSF12A)	TNFRSF12A	-1.748926537	-3.361083855	0.080506838	1.05738945	0.00944984	3.17866218
NM_004195	tumor necrosis factor receptor superfamily, member 18 (TNFRSF18), transcript variant 1.	TNFRSF18	-2.050031699	-4.141150683	0.147590397	1.107717805	0.008226651	3.738452757
NM_001065	tumor necrosis factor receptor superfamily, member 1A (TNFRSF1A).	TNFRSF1A	-1.07441724	-2.105871257	-0.195302667	0.873389646	0.041478678	2.411147496
NM_003327	tumor necrosis factor receptor superfamily, member 4 (TNFRSF4)	TNFRSF4	-3.092590007	-8.530261728	-0.039008242	0.973323814	0.029258927	8.764053243
NM_001242	tumor necrosis factor receptor superfamily, member 7 (TNFRSF7)	TNFRSF7	-1.285312547	-2.437348499	-0.162803674	0.893287405	0.007414285	2.728515464
NM_001243	tumor necrosis factor receptor superfamily, member 8 (TNFRSF8), transcript variant 1	TNFRSF8	-1.683238169	-3.211479683	0.612008538	1.528385566	0.004084046	2.10122351
NM_006291	tumor necrosis factor, alpha-induced protein 2 (TNFAIP2).	TNFAIP2	-1.257003511	-2.389988226	-0.30841274	0.807529719	0.000443701	2.959628816
NM_003405	tyrosine 3-monooxygenase/tryptophan 5-monooxygenase activation protein, eta polypeptide (YWHAH)	YWHAH	-1.683059173	-3.211081257	-0.308990203	0.807206556	0.015028377	3.978016819
NM_003596	tyrosylprotein sulfotransferase 1 (TPST1).	TPST1	-2.127842817	-4.370634745	-0.454057746	0.729986786	0.017029208	5.98727926
NM_006830	ubiquinol-cytochrome c reductase, 6.4kDa subunit (UQCR).	UQCR	-1.269691789	-2.411100504	-0.095322422	0.936063027	0.004921087	2.575788632
NM_006398	ubiquitin D (UBD).	UBD	-5.242250166	-37.85075483	-0.187114315	0.878360867	0.004663771	43.09248767
NM_181803	ubiquitin-conjugating enzyme E2C (UBE2C), transcript variant 6	UBE2C	-1.666505973	-3.174448501	-0.138767959	0.908294495	0.020760575	3.494955127
NM_014501	ubiquitin-conjugating enzyme E2S (UBE2S)	UBE2S	-1.38321848	-2.608496468	0.040607401	1.028546773	0.007697248	2.536099026
NM_003782	UDP-Gal:betaGlcNAc beta 1,3-galactosyltransferase, polypeptide 4 (B3GALT4)	B3GALT4	-2.40361829	-5.2912856	-0.196491198	0.87267042	0.006530562	6.063326404
NM_001497	UDP-Gal:betaGlcNAc beta 1,4- galactosyltransferase, polypeptide 1 (B4GALT1).	B4GALT1	-2.244153395	-4.737590145	-0.102416179	0.931471686	0.006359919	5.086134359
NM_003359	UDP-glucose dehydrogenase (UGDH).	UGDH	-1.849386748	-3.603469783	0.0952306	1.068236141	0.019863417	3.373289524
NM_178443	UNC-112 related protein 2 (URP2), transcript variant URP2LF.	URP2	-2.145252408	-4.423696496	-0.048523963	0.966925095	0.000554894	4.575014674
A_01_P012330	Unknown		-2.644678618	-6.253563915	-0.364415708	0.776783409	1.30E-05	8.050588931
A_01_P005239	Unknown		-1.594410131	-3.019710261	-0.46038794	0.726790799	0.001942973	4.154854831
A_01_P018128	Unknown		-1.408859768	-2.655272208	-0.147015293	0.903116937	0.004560247	2.940120043

A_01_P004143	Unknown		-2.053486438	-4.151079143	0.716445777	1.643129032	0.009612661	2.526325725
A_01_P011226	Unknown		-1.807582667	-3.500552556	-0.360448264	0.778922521	0.011265529	4.494095963
NM_138397	Unknown		-3.499172565	-11.30722156	-0.703299981	0.614165773	0.014869119	18.41069962
A_01_P016008	Unknown		-1.766820042	-3.403030406	-0.376995471	0.770039592	0.025954842	4.419292779
A_01_P000822	Unknown		-1.887030302	-3.698730798	-0.69898439	0.616005702	0.048867975	6.004377534
NM_012474	uridine-cytidine kinase 2 (UCK2)	UCK2	-1.566826156	-2.962522593	-0.188229251	0.877682319	0.018239053	3.375392815
NM_001078	vascular cell adhesion molecule 1 (VCAM1), transcript variant 1.	VCAM1	-1.893950494	-3.716515163	0.038269067	1.026881044	0.029052794	3.619226573
NM_003378	VGF nerve growth factor inducible (VGF)	VGF	-3.640222978	-12.46856023	-0.526675115	0.69415266	0.038494399	17.96227392
NM_000222	v-kit Hardy-Zuckerman 4 feline sarcoma viral oncogene homolog (KIT)	KIT	-1.257798846	-2.391306152	0.236698829	1.178293406	0.037884265	2.029465784
NM_007268	V-set and immunoglobulin domain containing 4 (VSIG4).	VSIG4	-3.781279338	-13.74923398	0.202478279	1.150673299	0.003403382	11.94885985
NM_002350	v-yes-1 Yamaguchi sarcoma viral related oncogene homolog (LYN).	LYN	-1.723168185	-3.301606504	-0.493985697	0.710060719	0.002697872	4.649752362
AI913343	wa11b04.x1 NCI_CGAP_Kid11 cDNA clone IMAGE:2297743 3' contains Alu repetitive element; sequence		-1.307317966	-2.474810338	-0.270330581	0.829129536	0.023165318	2.984829547
NM_017528	Williams Beuren syndrome chromosome region 22 (WBSCR22).	WBSCR22	-2.244184437	-4.737692086	-0.014927001	0.989706734	0.000235461	4.786965598
NM_032463	Williams-Beuren syndrome chromosome region 5 (WBSCR5), transcript variant 2	WBSCR5	-1.778777711	-3.431353382	0.234664238	1.176632864	9.59E-05	2.916248122
NM_152858	Wilms tumor 1 associated protein (WTAP), transcript variant 3	WTAP	-1.684296684	-3.213836831	0.37943438	1.300831754	0.004303757	2.47060146
NM_133264	WIRE protein (WIRE)	WIRE	-2.116053953	-4.335065983	-0.249726838	0.841055647	0.034394641	5.154315295
NM_024096	XTP3-transactivated protein A (XTP3TPA).	XTP3TPA	-1.79988048	-3.481913782	-0.582410298	0.667847079	0.014818605	5.21363931
NM_015898	zinc finger and BTB domain containing 7 (ZBTB7)	ZBTB7	-2.00774143	-4.021521491	-0.498774315	0.70770778	0.038724664	5.682460478
NM_024625	zinc finger CCCH type, antiviral 1 (ZC3HAV1), transcript variant 2	ZC3HAV1	-1.813118472	-3.514010426	0.08355821	1.059628249	0.001048414	3.31626722
NM_025079	zinc finger CCCH-type containing 12A (ZC3H12A).	ZC3H12A	-1.326601783	-2.508112017	0.464178611	1.379531702	0.004479884	1.818089438
NM_015655	zinc finger protein 337 (ZNF337).	ZNF337	-1.378779859	-2.600483455	-0.36586006	0.776006122	0.010437383	3.351112034
NM_153219	zinc finger protein 524 (ZNF524).	ZNF524	-1.803347804	-3.49029216	-0.436438656	0.738956499	0.002383404	4.723271483
NM_004729	zinc finger, BED-type containing 1 (ZBED1).	ZBED1	-1.678535617	-3.201028712	0.32198642	1.250050536	0.005168301	2.560719443
NM_024508	zinc finger, BED-type containing 2 (ZBED2).	ZBED2	-2.377977737	-5.198076036	0.091363159	1.065376349	0.013892531	4.879098397
NM_032283	zinc finger, DHHC domain containing 18 (ZDHHC18)	ZDHHC18	-1.548796598	-2.925729922	-0.07014924	0.952539457	0.000307488	3.071505226
NM_032850	zinc finger, FYVE domain containing 19 (ZFYVE19).	ZFYVE19	-1.451355168	-2.734648046	-0.676070098	0.625867819	0.010099497	4.369369961
NM_032265	zinc finger, MYND domain containing 15 (ZMYND15)	ZMYND15	-2.243223506	-4.734537518	-0.320721191	0.800669529	0.001429554	5.913223051
NM_080603	zinc finger, SWIM-type containing 1 (ZSWIM1).	ZSWIM1	-1.173526979	-2.255624595	-0.422055799	0.746360324	0.039936603	3.022165732
