# Supplementary material for: Transcriptional Reprogramming in Nonhuman Primate (Rhesus Macaque) Tuberculosis Granulomas
Source: PLoS One. 2010 Aug 31;5(8):e12266. doi: 10.1371/journal.pone.0012266 (PMC2930844; doi:10.1371/journal.pone.0012266)
Supplement: Table S7 — DNA Microarray Analysis: Immune function genes with significantly enhanced expression in Mtb granuloma's relative to non-granulomatous tissue four week's post-infection. Symbol = Official NCBI gene symbol associated with that gene. P = p value of significance in a student's t-test. (0.01 MB DOCX) [file pone.0012266.s007.docx]

| **Symbol** | **Gene** | **Av Fold Change (Lesion Lung /Normal Lung)** | ***P*** |
| --- | --- | --- | --- |
| ARRB2 | β-arrestin | 2.619377 | 0.002364 |
| CCL18 | chemokine (C-C motif) ligand 18 | 7.611917 | 0.0016 |
| CCL25 | chemokine (C-C motif) ligand 25 | 2.444154 | 0.010107 |
| FGFR3 | fibroblast growth factor receptor 3 | 3.592824 | 0.003452 |
| FGFR4 | fibroblast growth factor receptor 4 | 21.27774 | 0.000122 |
| HIF3A | hypoxia inducible factor 3, alpha | 6.437266 | 0.012085 |
| ITLN1 | intelectin 1 | 2.465053 | 0.002401 |
| IL22RA1 | interleukin 22 receptor, alpha 1 | 3.225048 | 0.010388 |
| LAMA3 | laminin, alpha 3 | 8.962433 | 0.009493 |
| LAMA5 | laminin, alpha 5 | 9.335793 | 0.000352 |
| LAMB2 | laminin, beta 2 | 5.086383 | 0.009493 |
| LAMB3 | laminin, beta 3 | 3.438854 | 0.000352 |
| MAPK3 | mitogen-activated protein kinase 3 | 2.893128 | 0.002367 |
| MAP3K4 | mitogen-activated protein kinase kinase kinase 4 | 2.691762 | 0.022208 |
| MAP3K6 | mitogen-activated protein kinase kinase kinase 6 | 11.10774 | 0.001497 |
| NCAM1 | neural cell adhesion molecule 1 | 4.599338 | 0.037136 |
| SOCS2 | suppressor of cytokine signaling 2 | 3.440873 | 0.028297 |
| TJP1 | tight junction protein 1 | 4.761958 | 0.030758 |
| TIMP3 | tissue inhibitor of metalloproteinase 3 | 8.906074 | 0.000595 |
| TGFB1I4 | transforming growth factor beta 1 induced transcript 4 | 2.381610 | 0.027687 |
| TGFBR3 | transforming growth factor, beta receptor III | 5.122092 | 0.014145 |

**Table S7**. Rhesus macaque genes that exhibit an enhanced expression in week 13 (late) granulomatous lung lesions relative to normal lung.
