# Supplementary material for: Transcriptional Reprogramming in Nonhuman Primate (Rhesus Macaque) Tuberculosis Granulomas
Source: PLoS One. 2010 Aug 31;5(8):e12266. doi: 10.1371/journal.pone.0012266 (PMC2930844; doi:10.1371/journal.pone.0012266)
Supplement: Table S8 — DNA Microarray Analysis: Immune function genes with significantly reduced expression in Mtb granuloma's relative to non-granulomatous tissue four week's post-infection. Symbol = Official NCBI gene symbol associated with that gene. P = p value of significance in a student's t-test. (0.02 MB DOCX) [file pone.0012266.s008.docx]

| **Symbol** | **Gene** | **Av Fold Change (Lesion Lung /Normal Lung)** | ***P*** |
| --- | --- | --- | --- |
| BCL3 | B-cell lymphoma | -2.53209 | 0.004217 |
| BAK1 | BCL2-antagonist/killer | -2.32475 | 0.014318 |
| BAX | BCL2-associated X protein | -3.04358 | 0.017341 |
| CARD11 | caspase recruitment domain family | -1.87577 | 0.012284 |
| CITED4 | Cbp/p300-interacting transactivator | -9.76434 | 0.001393 |
| CEBPB | CCAAT/enhancer binding protein C/EBP, beta | -3.21654 | 0.000316 |
| CEBPB | CCAAT/enhancer binding protein C/EBP, delta | -7.10180 | 0.000480 |
| CD14 | CD14 antigen | -6.74513 | 0.001202 |
| CD274 | CD274 antigen | -11.1411 | 0.003791 |
| CD300A | CD300A antigen | -3.39744 | 0.000921 |
| CD3E | CD3 antigen epsilon | -3.36447 | 0.005365 |
| CD40 | TNF receptor superfamily member | -2.23572 | 0.000563 |
| CD53 | CD53 antigen | -4.58938 | 0.001177 |
| CD68 | CD68 antigen | -7.59473 | 0.003037 |
| CD86 | CD86 antigen | -10.8201 | 0.012616 |
| CCL11 | chemokine C-C motif ligand 11 | -7.79850 | 0.005802 |
| CCL19 | chemokine C-C motif ligand 19 | -7.03154 | 0.001389 |
| CCL2 | chemokine C-C motif ligand 2 | -6.05908 | 0.019201 |
| CCL5 | chemokine C-C motif ligand 5 | -3.35570 | 0.002501 |
| CCL7 | chemokine C-C motif ligand 7 | -3.00033 | 0.004600 |
| CCR1 | chemokine C-C motif receptor 1 | -5.42292 | 0.010146 |
| CXCL10 | chemokine C-X-C motif ligand 10 | -24.7351 | 0.007530 |
| CXCL12 | chemokine C-X-C motif ligand 12 | -2.91298 | 0.010170 |
| CXCL16 | chemokine C-X-C motif ligand 16 | -9.42271 | 0.027922 |
| CXCL3 | chemokine C-X-C motif ligand 3 | -6.46675 | 0.049593 |
| CXCL6 | chemokine C-X-C motif ligand 6 | -15.1960 | 0.001184 |
| CXCL9 | chemokine C-X-C motif ligand 9 | -58.6505 | 0.007088 |
| CXCR4 | chemokine C-X-C motif receptor 4 | -1.61666 | 0.001202 |
| CXCL11 | chemokine (C-X-C motif) ligand 11 | -280.357 | 0.020987 |
| GZMA | Granzyme A | -5.37562 | 0.000761 |
| GZMB | Granzyme B | -2.62452 | 0.001957 |
| LITAF | lipopolysaccharide-induced TNF | -13.5891 | 0.018954 |
| MT1X | metallothionein 1X | -24.1233 | 0.000854 |
| SAA2 | Serum Amyloid A2 | -14.3976 | 0.000590 |
| INDO | indoleamine-pyrrole 2,3 dioxygenase | -41.6323 | 0.003848 |
| IFNGR2 | interferon gamma receptor 2 | -2.69971 | 0.002073 |
| IRF1 | interferon regulatory factor 1 | -15.8643 | 0.001035 |
| IRF7 | interferon regulatory factor 7 | -6.02871 | 0.007840 |
| IRF8 | interferon regulatory factor 8 | -3.65958 | 0.031799 |
| IFI30 | interferon, gamma-inducible protein 30 | -7.02698 | 0.00666 |
| IFI35 | interferon, gamma-inducible protein 35 | -4.57886 | 0.010378 |
| IL1A | interleukin 1, alpha | -2.52142 | 0.035867 |
| IL2RG | interleukin 2 receptor | -7.48616 | 0.008447 |
| IL21R | interleukin 21 receptor | -5.64579 | 0.027202 |
| IL27RA | interleukin 27 receptor, alpha | -5.90623 | 0.044893 |
| JAK2 | Janus kinase 2 | -4.26296 | 0.041502 |
| STAT1 | signal transducer and activator of transcription 1 | -7.359 | 0.005203 |
| SOCS1 | suppressor of cytokine signaling 1 | -6.70333 | 0.011042 |
| TLR2 | toll-like receptor 2 | -6.22063 | 0.000795 |
| TNFSF13B | tumor necrosis factor ligand superfamily | -4.41471 | 0.007926 |
| TNFRSF12 | tumor necrosis factor ligand superfamily | -3.17866 | 0.009449 |
| TNFRSF18 | tumor necrosis factor ligand superfamily | -3.73845 | 0.008226 |
| TNFRSF4 | tumor necrosis factor ligand superfamily | -8.764053 | 0.029258 |
| VGF | VGF nerve growth factor inducible | -17.9623 | 0.038494 |
| VDR | vitamin D 1,25- dihydroxyvitamin D3 receptor | -27.1317 | 0.027813 |

**Table S8**. Rhesus macaque genes that exhibit a diminished expression in week 13 (late) granulomatous lung lesions relative to normal lung.
