# Supplementary material for: Transcriptional Reprogramming in Nonhuman Primate (Rhesus Macaque) Tuberculosis Granulomas
Source: PLoS One. 2010 Aug 31;5(8):e12266. doi: 10.1371/journal.pone.0012266 (PMC2930844; doi:10.1371/journal.pone.0012266)
Supplement: Table S9 — This table contains the detailed composition of all 10 K-means clusters defined in the statistically significant data set between early and late lesions. (1.02 MB PDF) [file pone.0012266.s009.pdf]

Comprehensive list of the composition of all 10 K-means clusters
Cluster 10

Gene Name	Av Normal	Av M (w12)	Av M (w4)	*P*	Hierarchical Clustering (order)	K-means clustering	K-means clustering (rank)
NM_052870	3.97456332	2.55884029	-6.603084378	0.005025648	11413	10	42
NM_000778	4.72355612	7.00119038	-6.768964459	1.65E-06	11450	10	45
NM_002152	3.62701029	5.20858889	-6.1069784	0.000143105	11441	10	18
NM_152722	4.45921898	4.99949913	-6.091482133	9.00E-06	11442	10	21
NM_022096	5.06673338	7.98705053	-6.44867337	1.90E-06	11452	10	51
NM_031264	4.0665252	5.71282112	-6.155715801	1.04E-06	11445	10	27
NM_031264	4.52261582	6.84839314	-6.155715801	6.12E-05	11449	10	41
NM_014433	4.04484999	5.24216572	-5.804245801	8.26E-06	11444	10	13
NM_014433	3.8087349	5.42874008	-5.804245801	2.57E-05	11443	10	15
NM_007054	4.64466666	7.41098309	-6.144437024	5.30E-07	11451	10	46
NM_052949	4.07456385	6.21103755	-5.892754539	0.000240826	11448	10	37
NM_052949	4.12830472	5.43561623	-5.892754539	5.33E-06	11446	10	23
NM_000526	3.56916829	4.73683275	-5.936833788	2.71E-05	11440	10	11
NM_000526	3.21078552	4.90677311	-5.936833788	7.64E-07	11439	10	4
XR_012693	4.2756426	3.83594862	-6.284220597	8.85E-06	11431	10	19
XR_012693	4.19246522	3.47460042	-6.284220597	6.95E-05	11430	10	25
NM_021924	3.29390921	4.4915445	-5.820595402	5.49E-06	11438	10	3
NM_021924	3.33405014	4.36157382	-5.820595402	5.85E-07	11437	10	2
NM_006843	2.90093259	2.7875338	-5.378760422	0.000116697	11420	10	26
NM_006843	3.03156218	3.99926249	-5.378760422	1.13E-05	11424	10	1
NM_024730	3.91815569	6.75424178	-5.253217827	0.00011986	11453	10	39
NM_018218	1.09300171	3.90701084	-4.507482344	0.000380411	11396	10	38
NM_018218	1.50546434	4.03563409	-4.507482344	0.000160854	11397	10	31
NM_018008	5.00086042	4.63799832	-6.180745423	0.000558391	11447	10	35
NM_152526	3.33795062	3.22305685	-5.322840901	0.000167915	11421	10	16
NM_152526	3.15554456	3.54966117	-5.322840901	6.75E-05	11422	10	8
NM_020795	3.02745292	3.31838481	-5.152155691	4.02E-06	11425	10	10
NM_020795	2.72534549	3.74962568	-5.152155691	7.47E-06	11423	10	12
NM_207392	4.24298951	3.79902022	-5.855464618	0.00028192	11432	10	22
NM_207392	4.75996772	4.29564749	-5.855464618	0.001231896	11433	10	28
NM_000272	4.25973294	2.61431303	-5.064034296	0.001141935	11415	10	34
NM_000272	4.12727564	3.68037806	-5.064034296	0.000366179	11434	10	17
NM_004445	2.68886018	3.61989029	-5.005106193	3.71E-07	11426	10	9
NM_004445	2.86313118	3.67228741	-5.005106193	1.59E-07	11427	10	7
XR_011538	0.32726115	5.29725396	-5.41053652	1.20E-06	11387	10	43
XR_011538	0.22631519	5.85864326	-5.41053652	8.59E-05	11386	10	48
BC043391	3.62460909	2.30011235	-4.847746995	4.27E-05	11416	10	29
NM_016611	3.02306183	3.86245598	-4.57729876	3.34E-08	11428	10	6
NM_016611	3.05997211	3.84199225	-4.57729876	2.26E-08	11429	10	5
NM_024898	2.08002389	3.49910122	-3.953289128	0.000124398	11398	10	32

NM_024898	2.03636666	4.03952455	-3.953289128	0.000565105	11399	10	33
NM_001035254	4.07086459	3.13617892	-4.54934768	6.51E-05	11417	10	24
NM_001035254	4.43578303	3.90357804	-4.54934768	7.49E-05	11436	10	20
NM_021990	0.36601761	5.88656899	-4.621100344	9.42E-06	11389	10	47
NM_021990	0.1650517	5.16922024	-4.621100344	2.75E-06	11388	10	44
NM_213653	3.92894357	4.01105568	-5.002520397	0.000443437	11435	10	14
CO582652	0.6874926	5.85413704	-3.966965376	0.000587441	11394	10	49
NM_024557	4.0842179	2.31392335	-4.848363313	0.00058225	11418	10	36
NM_024557	4.44964291	2.87261801	-4.848363313	0.000638676	11419	10	30
NM_016458	3.378757	2.09922955	-4.813497457	0.00607869	11414	10	40
A_01_P001117	4.42725702	2.87163803	-2.292428095	0.039128673	11253	10	50

Cluster 9

Gene Name	Av Normal	Av M (w12)	Av M (w4)	*P*	Hierarchical Clustering (order)	K-means clustering	K-means clustering (rank)
CR936794	0.02900911	-2.6460049	2.164108547	0.045101419	5866	9	361
NM_172341	-0.6828238	-1.8493965	2.619369334	0.019503307	54	9	342
XR_013264	-0.0331776	-2.5154483	2.489115701	0.021147429	5287	9	322
NM_000602	-0.6287845	-3.4555149	3.184909984	0.005980494	5288	9	306
NM_000602	-0.2936283	-3.3562353	3.184909984	0.006200671	5289	9	297
NM_002250	0.11644242	-2.0548902	2.643176774	0.021046938	11308	9	352
XR_010708	-0.8597375	-1.924339	2.812118833	0.045635047	11304	9	379
XR_010708	-0.4746434	-2.3159266	2.812118833	0.034876313	11303	9	360
NM_023009	-0.0191567	-3.930176	1.844526003	0.011591708	11272	9	371
NM_006273	0.58249445	-2.1691499	3.290346891	0.009194482	5253	9	303
NM_003757	-0.4018589	-2.6115704	2.790707398	0.03944705	5868	9	367
NM_003757	-1.1828758	-1.8953055	2.790707398	0.046706635	5860	9	377
NM_006235	-0.0861702	-2.8227498	2.489925938	0.00131008	5491	9	147
NM_006235	0.04934014	-2.9200835	2.489925938	0.001321186	5492	9	163
NM_003486	0.36835324	-4.0217129	2.427810254	0.00101172	5284	9	311
NM_003486	0.37960026	-3.5766228	2.427810254	0.00136697	5285	9	279
NM_014822	-3.2997964	-3.1826514	2.350710317	0.009277479	3	9	431
NM_003516	-1.0565677	-2.5961733	2.521660173	0.012386519	52	9	357
NM_003516	0.29617029	-3.0586259	2.521660173	0.010611	5490	9	312
NM_005084	-0.3500493	-2.4845072	2.538215418	0.004801908	53	9	364
CN644277	-0.3566765	-3.5629365	2.184133041	4.23E-05	5523	9	232
CN644277	-0.6670939	-3.0607884	2.184133041	6.18E-05	5356	9	273
CN644277	0.41619997	-3.1175483	2.184133041	0.000533932	5454	9	256
NM_002314	-0.297198	-2.7411279	2.910080963	0.006321139	5497	9	223
NM_002314	-0.5065288	-2.4292564	2.910080963	0.006098351	5498	9	219
NM_018413	0.26754944	-3.5653111	1.962802687	0.003967374	88	9	323
NM_015364	0.17918748	-2.0069817	4.238183798	0.007892995	5254	9	331
NM_015364	0.29712997	-1.4668624	4.238183798	0.011757375	5255	9	353
NM_014397	-0.5488734	-2.3223954	3.375500334	0.022776021	11347	9	335

NM_014397	-0.0761405	-1.876495	3.375500334	0.030320014	11348	9	347
NM_001450	-1.0011492	-2.6839546	2.319185441	0.00138374	5361	9	326
NM_016613	-0.2382624	-3.1883139	2.450003848	0.031721923	5854	9	374
NM_198552	-0.7638395	-2.1778703	2.931904509	0.003562539	5364	9	275
NM_032369	-0.4874597	-3.3149699	3.264975618	0.008258795	11315	9	320
NM_032369	-0.7593969	-2.3617212	3.264975618	0.013360486	11305	9	329
NM_004271	-0.1142852	-3.1833521	2.806570797	0.000502928	5493	9	90
NM_004271	0.16206182	-3.0399732	2.806570797	0.000504143	5494	9	100
NM_003864	-0.5217251	-2.0896387	2.896335629	0.004615531	5485	9	231
NM_004691	-0.9207045	-2.7028879	2.465689725	0.000703777	5560	9	171
NM_004691	-0.8708934	-2.1859689	2.465689725	0.00025525	5559	9	185
NM_003547	-0.1777416	-2.7398529	2.202659194	8.80E-05	5602	9	135
NM_001186	0.16147637	-2.0799927	3.260892239	0.044042028	11311	9	389
NM_198460	0.30582026	-3.43151	3.149834465	0.000508158	5330	9	153
NM_198460	-0.8297398	-3.5319104	3.149834465	0.000831879	5318	9	210
NM_005949	0.12075018	-3.8575657	2.135305761	0.001249531	81	9	304
NM_005949	0.35425971	-3.0227526	2.135305761	0.000129522	5604	9	198
NM_001827	-0.1765378	-2.4513124	2.520668502	0.000130799	5695	9	101
NM_001827	-0.0071631	-2.2759559	2.520668502	0.000624597	5655	9	140
NM_133467	-0.1757703	-3.769515	2.328792935	0.005497767	11273	9	330
NM_133467	0.14138027	-3.4289036	2.328792935	1.06E-05	5527	9	158
NM_001216	0.0210262	-2.2553691	3.851061407	0.030252396	11318	9	391
NM_001216	-0.4390776	-2.6791932	3.851061407	0.023495437	11317	9	381
NM_052815	0.34561894	-2.1225643	2.561339855	0.000106172	5663	9	151
NM_052815	0.62371147	-2.2440698	2.561339855	4.67E-05	5665	9	165
NM_052815	0.15906092	-2.0527089	2.561339855	5.84E-05	5662	9	134
NM_052815	0.46464475	-2.1248194	2.561339855	3.83E-05	5664	9	154
NM_001251	0.08285404	-2.9042509	2.136591974	3.20E-08	5600	9	157
NM_001251	0.11588232	-3.0408817	2.136591974	5.57E-07	5530	9	166
NM_000491	-0.7684524	-3.3090983	2.02965279	2.37E-05	5412	9	236
NM_002065	-0.896937	-2.4777077	2.936904605	0.002424113	5483	9	184
NM_002065	-0.9225094	-2.1464678	2.936904605	0.00311157	5484	9	220
NM_001693	-0.5150208	-2.8901411	2.303725384	0.000828492	5548	9	182
NM_002422	0.1403036	-2.8925372	2.588116903	0.00133423	5496	9	139
NM_002422	0.16572017	-2.8467312	2.588116903	0.010078359	5851	9	290
XR_012476	-0.2722291	-2.4938896	3.108485883	0.000449949	5698	9	32
XR_012476	-0.3129148	-2.2526683	3.108485883	0.000537671	5699	9	60
XR_012476	-0.8505603	-2.2657812	3.108485883	0.009587205	5858	9	301
NM_000189	0.07696103	-3.3289211	2.251659335	8.72E-09	5528	9	159
NM_000189	-0.2574615	-3.7330564	2.251659335	0.000154684	5524	9	254
NM_003264	-0.234213	-2.4028466	2.374249216	0.001747957	5495	9	164
NM_002125	-0.8117844	-2.1120837	2.544378055	0.000526442	5363	9	267
NM_002125	-0.4496154	-2.0841825	2.544378055	0.000354417	5588	9	172

XR_013712	-0.4282001	-2.6945324	2.297151681	4.40E-06	5568	9	118
XR_014800	-0.0874042	-2.2805219	2.659449551	0.000152826	5696	9	79
NM_000591	-0.2964509	-2.7241253	2.255046293	7.83E-06	5569	9	120
NM_015892	-0.9141031	-2.8680533	2.25865923	0.003178074	5388	9	334
NM_001637	0.07415074	-2.6563832	2.620406269	4.79E-05	5700	9	69
NM_001637	-0.0396632	-2.5124652	2.620406269	5.67E-05	5701	9	64
NM_024329	-0.486057	-3.5290709	1.924768279	0.000527849	5410	9	284
NM_024329	-1.0398343	-4.9706263	1.924768279	4.48E-05	11278	9	385
XR_009750	-0.4684181	-2.7410958	2.856521872	0.000359964	5553	9	56
XR_009750	-0.1459549	-2.3128281	2.856521872	0.000108984	5697	9	41
NM_001012456	0.97064622	-2.7721998	2.356191899	0.000104169	5455	9	262
NM_002631	-0.1846889	-2.3287448	2.451336715	1.98E-06	5626	9	92
NM_005067	0.32486581	-2.5896604	2.787088308	7.29E-05	5707	9	57
NM_005067	0.38094913	-2.4793509	2.787088308	7.44E-05	5708	9	73
NM_002466	0.62847226	-2.2635632	2.601390791	0.00033107	5782	9	175
NM_002466	0.58496025	-2.0188846	2.601390791	0.000388084	5783	9	207
NM_002935	-0.6142388	-3.5155398	3.417989712	0.006937593	5298	9	310
NM_002935	-0.2984812	-3.0660686	3.417989712	0.006047542	5499	9	263
NM_001912	0.09133799	-1.9404018	3.175725579	0.003823471	5797	9	222
NM_000804	0.49738919	-1.9884118	3.526804675	0.002876974	5821	9	265
NM_000804	0.21211372	-1.8066993	3.526804675	0.001706745	5820	9	235
NM_002198	-0.8415147	-2.6511604	3.258271519	0.00048842	5519	9	129
NM_002198	-0.7462051	-3.2415087	3.258271519	0.000143385	5518	9	93
NM_002041	0.21352049	-2.8676513	2.513262119	0.033378244	5855	9	365
NM_005940	0.69259414	-1.7213565	3.517501911	0.031343037	11349	9	356
NM_005940	1.14079656	-2.1951658	3.517501911	0.018856339	11350	9	351
NM_003652	0.13142697	-3.4657121	2.654252917	0.004914807	11274	9	281
NM_003652	0.29792987	-2.0533096	2.654252917	0.003288184	5784	9	217
NM_052871	0.2813825	-3.3845493	1.935097398	5.27E-05	5525	9	269
NM_000088	-0.5378096	-4.3744179	3.302823727	0.000583935	5297	9	308
NM_000088	-0.0031007	-3.8442352	3.302823727	0.000387312	5328	9	192
NM_015393	0.0502336	-1.8868683	2.784779633	0.000550765	5785	9	137
NM_004159	0.18709807	-2.3818992	2.518099777	1.26E-07	5638	9	86
NM_004159	0.24909515	-2.2358786	2.518099777	2.57E-07	5639	9	116
NM_004847	-0.50621	-2.479574	2.622316165	2.20E-06	5551	9	51
NM_004847	-0.6437568	-2.520836	2.622316165	5.19E-05	5550	9	83
NM_139266	0.29352959	-3.275123	2.295329908	1.83E-05	5531	9	169
NM_139266	0.33817974	-3.2176898	2.295329908	1.27E-05	5532	9	178
DV768600	-0.5448397	-3.8755022	2.945499852	1.29E-05	5317	9	221
DV768600	0.06797879	-3.9020055	2.945499852	1.96E-05	5319	9	168
NM_003851	-1.1736507	-3.1504655	2.527798712	5.90E-05	5546	9	214
NM_003851	-0.9280481	-2.6536094	2.527798712	0.000298254	5547	9	180
NM_021199	-0.4856329	-2.1895585	2.517067454	1.09E-06	5580	9	107

NM_021199	-0.5179179	-2.7072201	2.517067454	0.00013114	5549	9	109
XR_000287	-0.5121642	-2.1566561	2.684568906	0.000899483	5508	9	177
CO648453	0.04855506	-2.3021356	2.459941762	2.55E-05	5636	9	117
NM_002922	0.43796152	-1.7561931	3.393547275	0.002692938	5798	9	234
NM_001005498	-0.2808043	-3.2747014	2.420046324	1.37E-05	5526	9	111
NM_001005498	0.04884772	-3.2194124	2.420046324	4.97E-06	5529	9	115
CN806576	-0.5303404	-3.2340385	2.581922811	0.00132932	5514	9	225
CN806576	-0.4720297	-2.9944163	2.581922811	0.001317829	5515	9	188
NM_033050	0.33133433	-1.9735022	2.882235224	0.000464653	5787	9	204
NM_033050	0.69233931	-2.1391947	2.882235224	0.000199903	5795	9	160
NM_176798	-0.2908186	-1.9672797	2.929139095	5.87E-05	5745	9	82
NM_000389	-0.4891362	-2.9446062	3.063640355	0.000747534	5516	9	119
NM_000389	-0.6123425	-2.7914009	3.063640355	0.000773084	5517	9	123
NM_016354	-0.0447423	-2.3769343	3.35584174	0.001783726	5500	9	174
NM_016354	0.10294998	-2.2406211	3.35584174	0.002278941	5502	9	138
NM_001975	-0.4113572	-2.7437899	2.306654511	1.01E-05	5567	9	108
NM_002346	-0.3674028	-2.1315961	2.433982596	0.004027973	5473	9	266
CB311553	0.51451964	-1.8584803	3.11658238	0.000874743	5796	9	205
NM_032935	0.23050906	-3.2127671	2.676199028	3.15E-06	5533	9	72
NM_032935	0.11271576	-2.8505453	2.676199028	4.41E-05	5687	9	42
NM_001295	0.63485519	-3.7755657	3.47801415	0.000261476	5329	9	218
NM_001295	0.05227897	-2.4913493	3.47801415	0.000666601	5501	9	87
NM_000376	0.4319032	-2.9273789	2.472388109	2.42E-05	5601	9	85
NM_000376	-0.3615343	-4.4003706	2.472388109	0.00593207	5293	9	376
NM_006404	0.44483364	-2.3119091	2.909484805	0.000571921	5792	9	136
CK230409	-0.0569178	-2.5341365	2.847659726	1.49E-06	5704	9	12
CK230409	-0.1163921	-2.3406877	2.847659726	6.11E-06	5705	9	22
NM_002986	-0.4039784	-2.5592187	2.913221206	0.003937476	5389	9	287
NM_006332	-0.4198913	-2.6877144	2.532669091	2.63E-05	5571	9	58
NM_006332	-0.2943395	-2.5185662	2.532669091	3.86E-05	5572	9	61
NM_004137	-0.1592555	-2.3185412	3.009456891	0.013549274	5852	9	299
NM_002800	-0.1992711	-2.7322974	2.922535355	2.25E-05	5703	9	14
NM_002800	-0.0497517	-2.3263417	2.922535355	1.98E-05	5706	9	27
NM_138557	-1.0240873	-3.3237787	4.207483481	0.004692067	11312	9	369
NM_138557	-1.1560311	-3.1743373	4.207483481	0.004831333	11313	9	344
NM_012118	-0.6577551	-1.5913242	4.191260298	0.006046155	11327	9	410
NM_001553	-1.1246242	-2.0475331	2.856225831	7.68E-05	5478	9	224
NM_004148	-0.0544365	-2.7292022	2.685244994	0.000134077	5686	9	49
NM_004148	0.00049541	-2.357055	2.685244994	6.14E-06	5702	9	52
NM_014437	-0.8638905	-1.9468277	2.836813387	0.000315767	5390	9	201
NM_006889	0.08183047	-3.2839447	4.522711269	0.002722197	11340	9	319
NM_006889	-0.289009	-3.1466378	4.522711269	0.002440322	11339	9	305
NM_152851	0.91614675	-2.3276899	3.248649598	0.000133365	5793	9	162

NM_152851	0.20809643	-2.117033	3.248649598	0.000275314	5794	9	91
NM_003901	0.04501949	-2.3148429	2.53946117	0.002945829	5512	9	212
DR774422	-0.1793984	-3.8980682	2.78381345	0.000346409	5307	9	226
DR774422	-0.1376284	-3.5876547	2.78381345	2.08E-05	5308	9	105
NM_002658	-0.3603891	-2.7176979	4.135506598	0.00315487	11316	9	294
NM_002658	-0.7099929	-2.4013029	4.135506598	0.003581853	11314	9	336
NM_018295	0.48021143	-2.704171	2.476915882	2.07E-05	5691	9	132
NM_000934	-0.2069932	-3.0598516	2.310396426	0.000464005	5543	9	173
NM_018965	-0.0780946	-2.5802411	2.849492625	0.000103406	5316	9	40
NM_018965	-0.2384141	-3.1522285	2.849492625	8.04E-05	5554	9	48
NM_014143	0.21713861	-3.6949658	4.259645799	0.003740651	11332	9	337
NM_014143	-0.4973922	-2.790606	4.259645799	0.010484002	11334	9	348
NM_000636	-0.031069	-2.1479207	2.521352018	0.001856972	5592	9	191
NM_000636	-0.3164399	-2.5317271	2.521352018	0.000151127	5545	9	113
NM_153341	0.33343754	-3.0756064	2.620833906	0.000568719	5522	9	181
NM_153341	-0.2787083	-2.5012106	2.620833906	0.000747797	5591	9	104
NM_001004019	0.31138742	-3.278936	3.186016947	0.000303556	5314	9	77
NM_001004019	0.5281415	-3.1858794	3.186016947	0.000165074	5503	9	179
NM_015907	-1.1694131	-4.2249952	3.911300503	0.001151795	5296	9	340
NM_015907	-0.3789695	-3.3575831	3.911300503	0.001677128	11335	9	259
CO646894	-0.0831997	-2.4797457	2.510723946	3.05E-05	5635	9	76
CO646894	-0.5557084	-2.2570666	2.510723946	3.62E-05	5579	9	124
CO647377	-0.1088919	-3.617714	2.969558985	3.13E-05	5306	9	127
CO647377	-0.206268	-2.7816982	2.969558985	4.21E-05	5688	9	8
NM_019859	-0.282899	-2.8251013	3.098849975	0.001197409	5290	9	317
NM_019859	-0.2196366	-2.1303097	3.098849975	0.002795696	1313	9	200
NM_019859	0.1757948	-1.7872216	3.098849975	0.002793242	5816	9	237
NM_004556	-0.0518585	-2.8897144	2.2850093	0.000153263	5539	9	144
NM_004556	0.08200214	-2.733914	2.2850093	0.000182444	5540	9	150
NM_182796	0.01572267	-2.230554	2.740334774	1.73E-05	5641	9	67
NM_182796	-0.2684069	-2.1051379	2.740334774	2.73E-05	5640	9	89
NM_002090	0.83401151	-4.3209751	3.401071675	0.016994403	11276	9	405
NM_002090	-0.520679	-2.1723628	3.401071675	0.000235815	5738	9	65
CO581942	-0.7723169	-2.8867689	2.638058724	6.50E-05	5562	9	88
CO581942	-0.8416188	-2.8790945	2.638058724	3.98E-05	5561	9	98
CO581942	-0.609668	-2.6512356	2.638058724	5.50E-05	5564	9	59
CO581942	-0.7883339	-2.7210999	2.638058724	2.45E-05	5563	9	75
CO580643	-0.265632	-3.0858454	2.887859812	0.000349694	5309	9	95
CO580643	0.03480841	-2.8514379	2.887859812	0.000178777	5689	9	34
NM_021129	0.05465828	-2.4206901	2.803719598	0.000422823	5709	9	99
NM_021129	0.12493776	-1.8415925	2.803719598	0.000216623	5653	9	156
NM_024508	2.8928483	-2.332161	3.201583999	0.00022125	11261	9	420
NM_024508	0.09136316	-2.3779777	3.201583999	0.000122334	5720	9	39

NM_001761	-0.0353799	-2.7282666	2.465368639	0.000496261	5521	9	126
NM_001761	-0.2065819	-2.1753209	2.465368639	0.000757243	5590	9	161
NM_001001437	-0.4331662	-2.4906574	3.05733631	3.33E-06	5714	9	2
NM_001001437	-0.5063589	-2.6926063	3.05733631	6.46E-06	5552	9	21
NM_002201	-0.3850176	-1.439876	4.2853467	0.014733038	11352	9	358
NM_002201	-0.8667179	-1.0513346	4.2853467	0.017911001	11351	9	382
XR_012454	-0.2017802	-2.7576198	2.751914802	0.023905219	5346	9	350
CN644408	-0.6186855	-2.4518914	3.123957114	1.58E-05	5734	9	38
CN644408	-0.6794309	-1.7845998	3.123957114	1.67E-06	5735	9	149
NM_000632	0.04400736	-2.3645618	3.34873331	9.43E-05	5804	9	26
NM_000632	0.23789431	-1.9755062	3.34873331	4.17E-05	5806	9	121
NM_003745	-0.1689871	-3.289889	2.153358134	0.002400632	5520	9	296
NM_003745	0.56836364	-3.3132423	2.153358134	0.001414936	5452	9	293
NM_002561	0.41233211	-2.9646431	3.029327967	1.03E-05	5692	9	47
NM_002561	0.45851872	-2.4258075	3.029327967	1.23E-05	5694	9	71
NM_138397	-1.5790549	-4.7645244	2.872788707	0.000141427	5291	9	372
NM_138397	-0.7033	-3.4991726	2.872788707	1.10E-05	5305	9	128
NM_003246	0.29102328	-2.6616439	4.8333619	0.015905747	11353	9	396
NM_003246	0.08362355	-2.3671743	4.8333619	0.018080185	11354	9	395
NM_014918	-0.6382889	-2.1954211	2.495871566	0.000218335	5380	9	229
NM_002727	0.45382789	-2.2931666	3.469233999	1.32E-05	5807	9	70
NM_002727	0.40518154	-2.0978028	3.469233999	1.67E-05	5808	9	94
CK231513	-3.4931845	-3.166945	4.918233225	0.015964881	11322	9	426
CK231513	-0.2745515	-3.2706978	4.918233225	0.015330099	11325	9	411
NM_004864	-0.1373992	-2.3687748	3.245104767	3.63E-06	5715	9	6
NM_004864	0.12222708	-1.8417184	3.245104767	4.76E-06	5743	9	130
NM_004117	0.09239614	-2.5493428	2.975260981	3.40E-06	5711	9	25
NM_004117	0.18875951	-2.540888	2.975260981	5.45E-05	5710	9	31
NM_002116	-0.5501125	-3.2710552	3.33190895	1.21E-06	5671	9	18
NM_002116	-0.698174	-2.9204656	3.33190895	2.65E-07	5669	9	7
NM_006931	0.60264909	-3.442193	3.014396856	0.003268583	11275	9	261
NM_006931	-3.0213048	-2.6269559	3.014396856	5.69E-05	5379	9	270
NM_152309	-1.5875548	-2.6517335	4.291414999	0.002168542	11337	9	285
NM_152309	-1.071802	-2.860864	4.291414999	0.001870196	11336	9	316
CK232222	0.10087383	-4.0916318	5.718584738	0.001450699	11297	9	407
CK232222	-0.5191511	-4.3137772	5.718584738	0.00164525	11296	9	413
CK232222	-2.4125262	-3.8839067	5.718584738	0.001888111	11295	9	415
NM_022141	-0.565051	-2.209555	3.358732143	0.000357449	5739	9	125
NM_022141	-0.2217885	-2.0360449	3.358732143	0.000201818	5802	9	97
NM_000295	-1.1141009	-3.9312673	3.399744133	2.69E-05	5299	9	238
NM_000295	-0.8336715	-2.6253532	3.399744133	5.79E-06	5670	9	33
NM_147780	-0.0237573	-2.8703223	3.148726551	0.000112264	5690	9	15
NM_147780	-0.2141277	-3.1922671	3.148726551	1.63E-07	5674	9	5

CN648306	-0.4455231	-2.7631324	3.059172469	1.24E-05	5303	9	183
CN648306	0.4209375	-2.847369	3.059172469	4.80E-06	5693	9	46
NM_020199	-1.5540696	-2.1209269	3.322255349	0.002842257	11329	9	307
NM_003247	1.58502661	-3.901325	2.962244465	0.001120738	11356	9	409
NM_003247	-0.5250884	-2.7523172	2.962244465	5.52E-05	5555	9	16
NM_002664	-1.3350891	-3.4261817	3.173631387	0.001228018	5311	9	194
NM_002664	-0.5168168	-3.5294547	3.173631387	0.00121097	5310	9	187
NM_005980	0.15349794	-2.6037527	4.542154862	0.000481171	11342	9	252
NM_005980	0.20132779	-2.5048794	4.542154862	0.000407198	11343	9	251
NM_014479	-1.1700774	-4.132288	3.139791754	0.000596071	5292	9	341
NM_014479	-0.0781693	-3.5595941	3.139791754	0.000240112	5313	9	106
NM_006274	0.07620568	-2.8957235	2.760608151	7.93E-05	5684	9	44
NM_006274	0.01677991	-2.8306214	2.760608151	0.000103816	5685	9	66
CO644910	-0.2849117	-4.0627711	3.803599143	5.98E-05	5321	9	248
CO644910	-2.7737992	-3.4605757	3.803599143	6.04E-05	11264	9	430
NM_001122	0.10844547	-2.105267	3.746960087	2.89E-05	5809	9	80
NM_001122	-1.4995317	-2.4046003	3.746960087	0.000129975	11330	9	333
NM_001814	-0.2548705	-2.3014011	3.389958225	2.54E-05	5719	9	19
NM_001814	-0.3308371	-1.9583693	3.389958225	3.88E-05	5721	9	78
NM_018404	0.119354	-2.866679	3.336181811	0.00457356	5849	9	240
NM_018404	0.01558396	-2.3413602	3.336181811	0.000235214	5803	9	62
NM_002652	0.13693844	-1.3788842	4.070270498	0.000345014	5260	9	280
NM_002652	0.37640967	-1.0478777	4.070270498	0.000364652	5261	9	318
NM_001710	-0.0203125	-1.5074323	3.538279696	1.59E-05	5815	9	196
NM_001710	-0.2519156	-1.44343	3.538279696	5.28E-05	5814	9	215
NM_004184	-1.4071053	-3.5179386	3.78531246	0.000979505	5301	9	302
NM_004184	-0.920416	-3.6800627	3.78531246	0.000404451	5300	9	247
NM_014220	-0.2010307	-2.1235245	2.969236861	0.002076724	5513	9	167
A_01_P005341	0.31834394	-4.7371808	3.991184859	5.76E-06	11287	9	343
A_01_P005341	0.07586246	-4.073678	3.991184859	2.34E-05	5326	9	246
CN646671	0.02406781	-1.4558222	3.381846729	0.00426263	5801	9	274
CN646671	-0.2170144	-1.7443921	3.381846729	0.008591173	5853	9	282
NM_006399	0.1888751	-2.6086719	3.294985081	6.56E-05	5723	9	10
NM_006399	0.20734254	-2.6327005	3.294985081	3.44E-05	5722	9	9
NM_000397	-0.4397856	-3.393249	3.55902919	2.37E-06	5667	9	68
NM_000397	0.00327213	-3.2139363	3.55902919	3.36E-06	5677	9	20
CO580929	-0.1967917	-3.7544316	3.514553518	7.00E-05	5324	9	133
CO580929	0.24931003	-4.0970673	3.514553518	1.98E-07	5320	9	216
NM_003661	-0.0818487	-2.8783875	3.600653078	4.28E-07	5678	9	1
NM_003661	0.13680414	-2.0184356	3.600653078	2.01E-05	5805	9	74
NM_000619	0.04410861	-2.4902885	4.364915094	0.003756634	11341	9	313
XR_012726	-0.0075693	-3.3723695	2.863019885	0.000632273	5312	9	131
XR_012726	0.00219572	-2.9949079	2.863019885	0.000701644	5315	9	110

NM_030643	0.21940453	-2.3548131	2.481223264	0.002043696	1068	9	203
NM_030643	-0.1255795	-2.8068729	2.481223264	0.000837342	5541	9	143
NM_030643	0.13176281	-2.1903591	2.481223264	0.00142607	1069	9	186
NM_020125	-0.2790872	-5.6027497	3.174907416	0.000837798	11279	9	403
NM_020125	-0.5608973	-4.0766263	3.174907416	4.17E-05	5304	9	227
NM_000104	-0.3976535	-4.7020329	5.728635799	0.003650905	11293	9	425
NM_000104	-0.1659646	-4.5559531	5.728635799	0.003600457	11294	9	422
NM_130759	-1.4003331	-3.0968514	3.355902595	1.08E-05	5302	9	190
NM_130759	-0.9231452	-2.9167169	3.355902595	2.86E-05	5668	9	43
NM_004862	-0.6101641	-3.2141934	3.661981976	1.33E-05	5672	9	45
NM_004862	-0.6804759	-2.9589433	3.661981976	5.03E-06	5673	9	24
NM_033554	-0.7825952	-2.290191	3.728139221	3.75E-07	5736	9	54
NM_033554	-0.7049159	-2.3360556	3.728139221	1.44E-08	5737	9	35
NM_002928	-0.0756559	-2.0777264	2.589674758	0.002326321	5511	9	208
NM_002928	-0.0812668	-2.1034712	2.589674758	0.003371463	5510	9	230
AL832403	-0.1731839	-2.3653069	3.34594476	1.42E-05	5718	9	13
AL832403	-0.2280092	-2.4651041	3.34594476	1.82E-05	5717	9	11
XR_013717	-1.0920954	-2.3962244	2.580547944	0.004260834	5381	9	277
NM_032758	-0.6447896	-1.3159534	4.038842531	0.012100116	11320	9	362
NM_032758	-0.6392219	-1.7863404	4.038842531	0.011648998	11319	9	354
CK232488	0.26386601	-2.6686632	3.250306108	7.01E-05	5712	9	28
CK232488	0.28760266	-2.6609865	3.250306108	0.000140683	5713	9	53
NM_000576	-1.0622143	-2.051178	3.471300923	3.19E-05	5733	9	189
NM_000576	-0.4613639	-2.5258165	3.471300923	0.000265438	5682	9	50
NM_006636	-0.0676178	-1.7112133	3.336949829	0.000218419	5822	9	239
NM_006636	-0.1518984	-2.1378284	3.336949829	0.000565126	5732	9	112
NM_003258	-0.1727277	-2.5764584	3.639046014	3.48E-05	5683	9	30
NM_003258	0.26320646	-2.0624171	3.639046014	4.58E-05	5812	9	122
A_01_P012330	-0.2433562	-2.9475335	3.455768129	9.38E-06	5676	9	3
A_01_P012330	-0.3644157	-2.6446786	3.455768129	1.41E-05	5716	9	4
CN643020	-0.1959185	-3.8371113	3.959677122	1.16E-05	5322	9	176
CN643020	-0.3604076	-3.2611765	3.959677122	4.25E-06	5679	9	55
NM_148170	0.13420562	-2.7882679	3.855508101	5.16E-06	5724	9	23
NM_148170	0.13671183	-2.4104869	3.855508101	3.99E-06	5725	9	29
NM_002421	-0.1280973	-1.9576806	5.490153635	0.001976562	11346	9	378
NM_181755	-0.158171	-1.6306238	3.701554423	0.00052376	5844	9	228
NM_181755	-0.2685182	-1.3598321	3.701554423	0.001033784	5845	9	288
NM_017958	0.38315567	-3.9085075	3.803800732	0.000206681	5327	9	245
NM_017958	-5.54972	-2.6131233	3.803800732	0.000111388	11265	9	404
NM_175571	-1.2663887	-1.5134212	3.212566455	0.002657882	5872	9	338
NM_175571	-1.3980905	-1.5707467	3.212566455	0.001947185	5871	9	373
NM_003254	-0.1154425	-2.9398365	3.06162159	0.000552472	5334	9	63
NM_003254	-0.1807523	-2.1470674	3.06162159	0.000952555	5730	9	114

A_01_P000822	-0.9380762	-6.4355743	3.034401141	0.020895619	1	9	432
A_01_P000822	-0.6989844	-1.8870303	3.034401141	0.001187586	5731	9	195
NM_177551	0.34351947	-2.5155143	3.669214115	0.000122557	5726	9	81
NM_177551	-0.4426341	-1.5910248	3.669214115	0.0001443	5823	9	213
NM_177551	0.27352096	-1.5960961	3.669214115	1.56E-05	5813	9	199
NM_177551	0.18237602	-2.0839222	3.669214115	7.48E-05	5811	9	102
CN801994	-0.1016882	-4.4906724	3.866478673	9.35E-07	11285	9	289
CN801994	-0.123346	-3.8277785	3.866478673	1.04E-05	5325	9	155
NM_012252	-0.2969141	-2.4659643	4.668781232	0.001677257	11338	9	315
NM_012252	-0.4653683	-3.1126656	4.668781232	0.001318057	11333	9	300
NM_004414	-0.1573546	-2.0230481	3.075839342	0.001914724	5829	9	170
NM_004414	0.57914168	-1.7837128	3.075839342	0.002090901	5837	9	253
NM_005729	-0.1320882	-2.4247882	3.197131218	0.000613642	5728	9	103
NM_005729	0.3576131	-1.8098281	3.197131218	0.001007733	5835	9	211
NM_006398	0.26459122	-5.3323607	3.812960444	3.54E-05	11284	9	383
NM_006398	-0.1871143	-5.2422502	3.812960444	3.28E-05	11283	9	370
XM_496386	-0.6342775	-2.7945747	3.134354481	0.000415162	5727	9	84
XM_496386	-0.3271429	-2.2188399	3.134354481	0.000709312	5729	9	96
NM_001005340	-0.4413295	-3.0140933	3.531956324	3.87E-05	5675	9	17
NM_001005340	-0.623297	-2.8811125	3.531956324	5.64E-05	5681	9	36
NM_006290	-0.434016	-1.4309553	3.950890063	0.001470308	5846	9	276
NM_007268	0.20247828	-3.7812793	3.958791612	1.63E-06	5323	9	209
NM_007268	-0.3869667	-3.0318239	3.958791612	2.18E-06	5680	9	37
NM_015440	-1.1582524	-2.0624236	3.001057223	0.005345563	5828	9	241
NM_015440	-0.1215577	-1.7474443	3.001057223	0.006353651	5831	9	260
NM_002923	-1.2951904	-1.1942226	3.817930226	0.003242081	5873	9	346
NM_002923	0.7004596	-1.4885538	3.817930226	0.002616309	11372	9	355
NM_005849	2.26160145	-2.2026668	3.466617462	0.000501263	5848	9	278
NM_005849	-0.2028778	-1.6360308	3.466617462	0.000722545	5810	9	206
NM_005514	-0.488722	-1.226656	4.085215579	0.000104724	5843	9	292
NM_005514	-0.5650448	-1.6519361	4.085215579	0.001035446	5842	9	272
NM_001511	0.20126953	-4.2168259	2.764069683	0.003274017	5294	9	339
NM_001511	-0.0002928	-2.1433171	2.764069683	0.014643942	5850	9	295
NM_000331	0.1639054	-3.2121942	3.910305716	0.000681427	5332	9	148
NM_000331	0.19436272	-2.8870597	3.910305716	0.000724187	5333	9	145
NM_000570	-0.5253741	-4.0193888	3.316627913	0.002411937	5295	9	314
NM_000570	-0.3335629	-2.0353193	3.316627913	0.001600892	5830	9	141
NM_002286	-1.215509	-3.3565542	5.386856809	0.010807785	11323	9	421
NM_002286	-0.6564361	-3.0074434	5.386856809	0.012257534	11324	9	417
NM_001085	-0.0484085	-2.0459181	4.67565719	3.25E-05	11345	9	249
NM_001085	-0.3799831	-2.0401674	4.67565719	4.24E-05	11344	9	258
NM_003004	-0.6554089	-5.413681	3.985345345	6.97E-06	11282	9	386
NM_003004	0.09074036	-4.7480521	3.985345345	1.08E-05	11286	9	328

NM_002416	0.07704153	-5.9511134	4.349936749	0.000187824	11280	9	416
NM_130830	-1.066743	-1.7487209	4.085534949	2.80E-05	5840	9	244
NM_130830	-0.7133655	-1.7764722	4.085534949	2.39E-05	5841	9	193
NM_021205	-0.0659094	-2.269962	3.898872983	0.000939952	5834	9	142
NM_021205	-2.2171638	-1.6578284	3.898872983	0.000758354	11331	9	368
NM_013322	-0.7711753	-4.8926684	5.131852111	0.000448969	11292	9	418
NM_002448	0.50627873	-2.7502007	2.461054255	0.04472985	5920	9	392
NM_080668	0.25809825	-1.6959215	3.759730781	0.003884407	5909	9	291
NM_080668	0.15420531	-1.633092	3.759730781	0.001717674	5908	9	271
NM_004994	0.4297464	-5.3263097	4.752640851	1.58E-05	11288	9	398
NM_004994	0.58201318	-5.3399388	4.752640851	6.18E-07	11289	9	399
NM_005566	-0.7947923	-2.9725341	4.525540081	1.12E-06	5344	9	202
NM_005566	-0.79044	-2.6689793	4.525540081	2.30E-06	5345	9	197
XM_496823	-0.3110321	-5.1563688	4.572676189	0.001477162	11281	9	402
XM_496823	-0.207223	-4.2265265	4.572676189	0.000336951	5331	9	327
XM_496526	0.13572882	-2.505213	2.520784824	0.012903012	5469	9	298
XM_496526	-0.1594609	-2.1761267	2.520784824	0.016616315	5884	9	325
NM_198594	-0.1283064	-3.1056624	3.491966801	0.001360795	5336	9	152
NM_198594	-0.1672705	-3.2951059	3.491966801	0.000805976	5335	9	146
NM_012081	-0.586881	-2.2141618	3.119940868	0.006304942	5827	9	250
NM_012081	-0.2423319	-2.5215109	3.119940868	0.014845249	5857	9	309
NM_005771	0.87796784	-1.7581873	3.50572552	0.00431402	11370	9	349
NM_005771	0.55168493	-1.7698754	3.50572552	0.003686264	5836	9	286
XR_014226	-0.4516679	-3.529967	4.466716708	7.04E-05	5342	9	257
XR_014226	-2.3076534	-3.496089	4.466716708	3.59E-05	11328	9	393
NM_080593	-0.010695	-2.2391174	3.483940199	0.007995532	5832	9	268
NM_080593	-0.0327156	-2.0206171	3.483940199	0.009621854	5833	9	283
NM_080593	1.05498994	-1.597907	3.483940199	0.015008263	11371	9	359
XM_375224	0.13618358	-3.4184844	4.617767178	9.96E-05	5343	9	264
CO645122	-0.0132563	-2.5241439	3.990680742	0.002447845	5337	9	242
CO645122	-0.0537671	-2.0439223	3.990680742	0.001960083	5905	9	255
NM_002993	0.29144375	-3.3480011	6.23390739	0.001424762	11301	9	414
NM_002993	0.03458883	-3.9602127	6.23390739	5.72E-05	11300	9	406
NM_022736	-0.261794	-3.4441006	3.998863975	0.000851846	5338	9	243
NM_022736	-0.0564793	-3.3125563	3.998863975	0.000818509	5339	9	233
NM_002164	-0.1990493	-5.1805808	5.469221031	1.45E-05	11290	9	408
NM_002164	-0.2318429	-4.9635703	5.469221031	2.22E-06	11291	9	401
NM_030769	-1.5948329	-4.1688827	2.995863263	0.008409557	11263	9	427
NM_002053	-0.677271	-4.0335291	4.855856574	0.000268051	5340	9	345
NM_002053	-0.7495535	-3.7618503	4.855856574	0.00030282	5341	9	332
NM_002030	-0.0945235	-1.6977758	4.200518969	0.005795516	5907	9	324
NM_002030	-0.0700063	-1.772008	4.200518969	0.005305996	5906	9	321
NM_001565	-0.1463641	-4.8138318	6.542988477	0.000484885	11298	9	429

NM_001565	0.00504652	-4.6335368	6.542988477	0.000357943	11299	9	428
NM_020792	-0.2908831	-3.5288355	2.676974695	0.029529735	11358	9	387
NM_020792	-0.8658011	-2.6444417	2.676974695	0.045517379	11359	9	384
NM_015973	-0.3133112	-1.3838979	5.104151384	0.023350798	11374	9	412
NM_015973	-1.2274372	-1.3265757	5.104151384	0.006571699	11373	9	400
NM_000600	-0.847601	-4.2589735	5.634289664	0.003242367	11302	9	419
NM_006169	0.23640984	-2.6310068	3.768865744	0.024304475	11363	9	375
NM_006169	-0.9295552	-1.2838797	3.768865744	0.034627096	11364	9	388
CO580739	0.54477147	-1.6755163	3.79449994	0.031082448	11367	9	380
CO580739	0.16664064	-1.7363698	3.79449994	0.026723791	11366	9	366
NM_000161	-0.0863908	-3.7456676	4.8280056	0.002105274	11360	9	363
NM_001776	0.05609544	-1.4863484	3.711262649	0.045449158	11365	9	390
NM_138455	0	-2.9793137	5.451666193	0.004647102	11361	9	394
NM_138455	-0.2687221	-2.0472468	5.451666193	0.006559195	11362	9	397
NM_005024	0.38427694	-1.3415704	4.971124857	0.044804907	11368	9	423
NM_005024	0.00661183	-1.0154249	4.971124857	0.049033165	11369	9	424

Cluster 8

Gene Name	Av Normal	Av M (w12)	Av M (w4)	*P*	Hierarchical Clustering (order)	K-means clustering	K-means clustering (rank)
NM_018697	0.53811471	2.46853822	-1.461131495	0.000313572	10583	8	327
NM_006001	1.22893416	2.62297294	-2.000226321	0.0035027	11154	8	543
NM_006001	1.23241733	2.72832356	-2.000226321	0.002520889	11155	8	525
NM_000853	-0.2828599	3.05906029	-1.553662648	1.16E-06	10306	8	181
NM_000853	-0.4108221	2.62927367	-1.553662648	0.000209073	10287	8	271
NM_001847	-0.0473147	3.18798588	-0.899661613	0.000275505	10704	8	507
NM_001932	-0.0310927	3.01348574	-1.529249008	3.49E-05	10316	8	133
NM_001932	-0.0286381	3.22422902	-1.529249008	1.41E-05	10318	8	193
NM_020217	-0.5313643	2.4305121	-1.621330257	0.001180862	10284	8	407
NM_020217	-0.3439394	2.65413752	-1.621330257	0.001735545	10286	8	361
XM_290835	-0.4837437	1.93934404	-1.826881258	0.001570225	9870	8	466
NM_022036	0.32418836	2.50303888	-1.50904271	3.90E-05	10580	8	192
NM_022036	0.56944538	2.5140106	-1.50904271	9.30E-05	10351	8	252
NM_174940	-0.3434733	2.2804927	-1.599719694	4.32E-05	10217	8	294
NM_174940	-0.2782586	2.32315403	-1.599719694	0.001298209	10285	8	371
AK125739	0.38180007	2.44247206	-1.335226713	0.001103141	10582	8	413
XR_013572	-0.2054485	2.39287072	-1.574941979	1.61E-05	10222	8	225
XR_012685	-0.3629867	1.92021296	-1.833521472	0.000343577	10168	8	414
NM_014935	-0.2682379	1.8646681	-2.041448233	0.001105499	9733	8	498
NM_014935	0.34025419	3.02993103	-2.041448233	0.000206672	11044	8	210
NM_032205	0.12790898	1.98204206	-1.679293139	0.008341606	9841	8	492
NM_147130	0.23313417	2.26157732	-2.51938602	0.027844487	6005	8	622
NM_017791	0.10927799	3.08859248	-1.24732273	0.000100356	10353	8	353
NM_004484	-0.2883718	3.59620063	-1.502932972	0.019847826	11170	8	625

NM_000805	1.26676108	2.85795504	-1.338122286	2.42E-05	11216	8	502
NM_015198	0.40642288	2.61120377	-1.268716998	5.03E-06	10346	8	314
NM_015198	0.33410212	3.19720963	-1.268716998	9.15E-05	10354	8	364
NM_016316	0.3626123	2.45082443	-1.334758555	8.89E-05	10343	8	324
NM_002334	-0.4467819	3.11485149	-1.102419845	0.001175704	10737	8	505
NM_001979	-0.1238381	2.72646889	-1.249263443	4.24E-05	10329	8	317
NM_001979	-0.2798305	3.48278063	-1.249263443	7.47E-06	11084	8	443
XM_027074	0.22517153	3.45941758	-0.717197453	0.014303305	11229	8	626
XR_011510	0.03389373	2.04055759	-1.762698507	0.014205637	10436	8	539
XR_011510	0.02161853	1.93697408	-1.762698507	0.017137715	10435	8	554
NM_014315	0.16215604	2.82985703	-1.198255419	0.001389609	10702	8	449
CN643806	0.45717524	2.31985377	-1.490591327	0.000750876	10349	8	358
CN643806	0.41655559	2.5659809	-1.490591327	0.000297218	10350	8	255
NM_024953	0.0182959	2.72259099	-1.263822563	0.048301088	11105	8	635
XR_009781	-0.8066199	3.08369608	-1.220330875	0.005285404	11181	8	570
NM_173525	1.18615921	3.10206547	-1.533447278	0.007370036	11219	8	587
NM_007351	0.53265812	2.4953441	-1.962061059	0.003054134	10439	8	396
NM_022481	-0.9088056	2.86080084	-1.18905091	4.52E-05	10688	8	520
NM_022481	-0.7809316	4.04913548	-1.18905091	0.016864939	11220	8	652
NM_020776	1.74557936	2.93234103	-1.110772146	0.008404695	11218	8	632
XM_376007	1.04843791	2.79808094	-1.258727345	0.010637202	11225	8	600
NM_022474	0.57506806	3.48966363	-0.803369322	0.003320944	10831	8	596
NM_033261	0.56363766	3.32263267	-0.803274326	0.030953107	11203	8	638
NM_001001995	0.07681166	4.34296698	-0.627334485	0.014692738	11403	8	656
NM_001282	0.73972372	2.99764071	-0.986109926	0.001255163	10780	8	535
NM_000466	-0.2601393	2.75155135	-1.314157745	0.006973914	10438	8	517
NM_000669	-0.5364019	5.13718142	-0.808623759	0.002168996	11221	8	666
CN645773	-0.0050589	4.47636594	-0.098033042	0.004674935	11232	8	663
NM_005613	0.51482793	3.42944889	-0.955575531	0.008072458	11211	8	597
CN803179	-0.1745692	3.46211824	-0.794787862	0.021372998	11210	8	633
NM_001003679	0.011319	3.60690932	-1.300351411	0.019839033	11209	8	630

Cluster 7

Gene Name	Av Normal	Av M (w12)	Av M (w4)	*P*	Hierarchical Clustering (order)	K-means clustering	K-means clustering (rank)
NM_052876	-1.3727574	-2.8902358	0.304710962	0.010973472	111	7	1198
NM_052876	-0.6171733	-3.070207	0.304710962	0.012827743	112	7	1172
NM_004998	-0.5859648	-2.2888148	1.095316167	0.03483611	241	7	1120
NM_004998	0.00026979	-1.8110151	1.095316167	0.048735786	242	7	1062
NM_153611	-0.0860007	-2.063194	0.622132367	0.025532151	1077	7	1010
NM_153611	-0.3899257	-2.1334315	0.622132367	0.015372098	1074	7	982
NM_016459	-1.962438	-2.9404496	0.010422596	0.000702191	15	7	1234
NM_000420	-0.1360827	-3.3690898	0.63515352	0.010337746	11269	7	1196
NM_000420	-0.0196211	-2.6742425	0.63515352	0.00215356	316	7	960

NM_012116	-0.0512501	-1.9210213	0.659334309	0.008419279	388	7	817
AB209098	-1.1165866	-2.0012191	1.086409364	0.011114203	192	7	1136
AB209098	0.00700723	-1.3416554	1.086409364	0.024522258	1108	7	907
AB209098	-0.0641713	-1.4985092	1.086409364	0.026825601	1107	7	838
NM_022107	-0.1948722	-1.5810155	0.938553069	0.049183746	1078	7	1025
NM_022107	-0.2374391	-1.7525349	0.938553069	0.02177703	1082	7	850
A_01_P004143	0.71644578	-2.0534864	1.229469092	0.014791296	5093	7	1060
A_01_P004143	-0.4727249	-1.605911	1.229469092	0.021821893	211	7	1130
NM_002429	-0.0883025	-1.7897155	1.190763002	0.032323753	1087	7	987
NM_170744	0.20116113	-2.4273299	0.640308809	0.035415999	129	7	1119
CO648874	-2.3468851	-3.7888553	0.060353232	0.03419172	11268	7	1228
CO648874	0.08446509	-2.5396315	0.060353232	0.000239434	143	7	1170
NM_004121	-0.1140193	-1.6979389	0.939140938	0.026812506	3924	7	914
NM_000074	-0.8116996	-2.7841831	0.120549144	0.004846775	93	7	1133
NM_022916	-0.2556969	-2.108076	0.531150528	0.001251458	333	7	800
NM_022916	-0.2374204	-2.2686648	0.531150528	0.002383482	332	7	804
NM_003733	-0.2018951	-3.0517767	1.734995037	0.004684342	5863	7	1142
NM_003733	0.10421214	-1.9308524	1.734995037	0.013232461	1109	7	916
NM_003121	-0.0471939	-2.2517362	1.147903051	0.00460248	1080	7	622
NM_003121	-0.2180255	-1.8591877	1.147903051	0.009680524	1079	7	814
XR_009675	-0.5163878	-1.2402937	1.275848156	0.016701178	271	7	869
XR_009675	-0.2669679	-1.3376354	1.275848156	0.013430094	1201	7	712
NM_012458	-0.4594995	-1.5770998	0.992123553	0.032939472	1083	7	942
NM_012458	-0.6761864	-1.9776483	0.992123553	0.032270963	1094	7	1003
NM_207322	-0.0428373	-1.758027	1.205279972	0.008845439	1081	7	756
NM_207322	-0.172306	-1.6679758	1.205279972	0.009622009	1088	7	660
NM_012117	-0.0776689	-1.820429	0.842821552	0.013821461	1684	7	725
NM_144603	0.39453627	-3.4714698	0.946061494	0.022575954	82	7	1208
XR_013330	-0.7674052	-4.6204092	0.282657069	0.006132876	76	7	1230
XR_013330	0.2757705	-2.2559835	0.282657069	0.018852213	1339	7	1092
XR_012642	-0.0434119	-2.1068009	0.465102099	0.00018656	341	7	839
A_01_P016008	-0.1237779	-2.4326494	0.595031846	0.033171861	96	7	1094
NM_153712	-0.3267116	-2.8858033	1.131833449	0.010907599	84	7	1078
NM_153712	-0.425567	-2.0766347	1.131833449	0.009491509	244	7	841
NM_021204	-0.2639299	-2.1605924	0.510140679	0.006650634	334	7	858
NM_000096	0	-3.2826947	1.664967821	0.011409153	11270	7	1184
XM_045423	-0.4864751	-2.4109244	0.967129885	0.013563768	301	7	939
XM_376350	-0.1995167	-1.8962184	0.980743056	0.002739104	394	7	370
XM_376350	-0.9693937	-2.249444	0.980743056	0.005038114	286	7	931
XM_376350	-0.9301802	-1.5437093	0.980743056	0.006967741	263	7	993
NM_138636	0	-2.9736874	2.034789629	0.017973147	5286	7	1191
NM_006840	-0.9515349	-2.0993343	2.095069341	0.045106678	5486	7	1195
NM_003740	-0.7972629	-1.8607941	1.779720114	0.014684728	5374	7	998

NM_015986	0.01981941	-2.185818	0.403303607	0.001499647	335	7	876
NM_000478	0.08552164	-1.9218448	0.873617187	0.002859696	378	7	576
NM_018556	-0.373062	-1.9459497	0.986426303	0.001418323	384	7	368
NM_016196	-0.467737	-1.9201116	0.908334822	0.001358821	472	7	798
XR_010574	-0.0008436	-1.2012988	1.236107016	0.008902548	4099	7	844
NM_004287	-0.5526993	-1.4689036	1.418240376	0.006197278	270	7	550
NM_004287	-0.5684674	-1.558088	1.418240376	0.005229975	269	7	537
NM_004287	-0.1514693	-1.5339318	1.418240376	0.02965453	548	7	929
NM_145266	-0.9288404	-1.3801774	1.155338752	0.010561227	201	7	932
CR936794	0.09968622	-2.4916649	2.164108547	0.04440209	5867	7	1209
NM_005470	-0.6327399	-2.4060109	0.515955413	0.002237454	1659	7	955
NM_005028	-0.2743113	-2.0246421	0.601106419	0.032682644	123	7	1067
NM_017828	-0.429522	-1.1184785	1.320512895	0.02972864	1137	7	962
NM_017828	-0.2639477	-1.3028158	1.320512895	0.037072429	1105	7	933
NM_054033	-0.4282304	-2.3960657	0.476138221	0.006714917	94	7	1011
NM_001001716	0.04632854	-2.2421556	0.822158794	0.01224291	1345	7	956
NM_002032	0.16129169	-1.9599403	0.674770358	0.001646255	375	7	663
A_01_P003225	-1.3922235	-1.9384632	1.076013482	0.016957973	174	7	1076
NM_139280	-0.3653154	-1.6621966	1.034639245	0.023593455	246	7	852
NM_005978	0.02998272	-1.9569506	0.99408219	0.004462232	395	7	555
NM_004887	-0.8672899	-1.4598018	1.708268354	0.017943575	51	7	1217
NM_032790	-0.5437616	-2.1705931	0.864136538	0.00129193	322	7	551
NM_032790	-0.5558902	-2.2078693	0.864136538	0.000364352	383	7	483
NM_002341	-0.1858166	-3.5739499	0.718963367	2.56E-05	5435	7	1154
NM_002341	-0.5041598	-3.9723227	0.718963367	0.000336393	77	7	1199
NM_021798	-1.3129249	-1.4310883	1.190795923	0.002894116	264	7	1023
NM_001421	-1.7792896	-1.803925	1.078017634	0.007648498	19	7	1200
NM_030574	-0.4243993	-1.6650089	0.830280235	0.002020776	1075	7	679
NM_153211	-0.1522379	-1.5755797	1.425415972	0.018061105	1089	7	818
NM_018982	-0.5935975	-1.6343418	0.900568383	0.002325863	1678	7	519
NM_013321	-0.0316317	-1.4627844	0.978969763	0.011075806	3920	7	731
A_01_P011226	-0.3463226	-1.5468645	1.542373188	0.03165076	261	7	1048
A_01_P011226	-0.3604483	-1.8075827	1.542373188	0.018304236	254	7	936
NM_005947	-0.1907441	-3.9449139	1.609314124	0.009515115	5282	7	1224
NM_005947	-0.3745263	-2.8951634	1.609314124	0.019273355	5283	7	1164
A_01_P018842	-0.0409473	-1.7445466	1.169688735	0.014064726	1076	7	740
NM_003006	-0.3640473	-2.9260852	0.737080352	0.00106653	314	7	1013
NM_003006	-0.1948379	-2.6918495	0.737080352	0.000659146	315	7	872
NM_016639	0.08050684	-1.7489265	0.796370319	0.00033243	379	7	459
NM_182566	-0.0155891	-2.1614769	0.81818635	0.000644927	377	7	491
NM_182566	-0.1095715	-2.5613781	0.81818635	3.27E-05	338	7	581
NM_024579	0.1439563	-2.4104333	0.849146878	0.000925017	337	7	742
NM_024579	0.10241455	-2.3746321	0.849146878	0.001534068	336	7	635

NM_032265	-0.0322718	-2.1208605	0.751882479	5.02E-05	340	7	464
NM_032265	-0.3207212	-2.2432235	0.751882479	0.000105191	339	7	480
NM_017528	-0.014927	-2.2441844	0.569698827	6.40E-06	343	7	667
NM_017528	-0.0709778	-2.0058055	0.569698827	2.65E-05	365	7	582
NM_005488	-0.6199937	-1.7245443	0.86707312	0.004370709	197	7	621
NM_032242	-0.1483084	-1.5314115	1.002270266	0.001266887	3779	7	375
NM_032242	-1.036509	-2.1529762	1.002270266	0.001154108	283	7	710
NM_004106	-0.6865017	-2.5128466	0.849788159	0.001461619	321	7	832
NM_004106	-0.8332313	-2.0256091	0.849788159	0.005076179	325	7	861
NM_016183	-0.0352938	-1.319964	1.330559213	0.012298298	4113	7	721
NM_017827	-0.6112677	-1.2493185	1.275770308	0.036518124	272	7	1018
NM_017827	-1.0409587	-2.3021275	1.275770308	0.008983403	243	7	988
NM_022371	-0.053933	-1.7875551	0.836735681	0.000820351	392	7	331
NM_022371	-0.4490294	-1.9551696	0.836735681	0.002944946	287	7	583
NM_014699	-0.0632974	-1.298601	1.406524123	0.010033969	1203	7	678
NM_005128	-0.1158885	-1.2682084	1.24530167	0.015228659	1202	7	748
A_01_P007044	-0.636388	-2.1910617	0.563815448	0.017302396	95	7	999
NM_024098	-1.0452812	-1.6222042	0.886934891	0.003757048	196	7	908
NM_006777	-0.0123069	-1.9197623	0.61158504	0.027693623	1685	7	965
NM_153219	-0.5854597	-1.9104207	0.671406494	9.48E-05	352	7	523
NM_153219	-0.4364387	-1.8033478	0.671406494	3.69E-06	353	7	436
XR_013439	-0.1219901	-1.5132184	0.95872697	0.00027291	3707	7	271
NM_152637	-2.3866352	-2.3953528	1.57439979	0.015196812	50	7	1225
NM_152637	0.13519798	-1.767973	1.57439979	0.024491211	1086	7	1015
NM_032681	-0.0653086	-1.7675837	1.543258832	0.008504846	1090	7	647
NM_032681	0.23213008	-1.6614955	1.543258832	0.006582146	1091	7	600
NM_023930	0	-1.976253	0.516515689	0.045176082	1344	7	1085
NM_004413	0.07589844	-2.4339682	0.470765505	5.73E-05	342	7	864
CN804671	-0.8251403	-1.577739	1.047274703	0.022676049	1699	7	961
NM_004843	-0.0510862	-1.750899	1.023989933	0.002697321	376	7	351
NM_004843	-0.6402459	-1.921992	1.023989933	0.007267277	303	7	585
NM_020683	0.94384388	-1.95549	1.188438528	0.004522418	1333	7	1084
NM_017580	0.03560396	-1.1958283	1.553871591	0.026379962	1140	7	991
AY635466	0.27835309	-2.9256616	1.539545141	0.00526192	5864	7	1072
NM_198857	-0.0623037	-2.1284491	0.843919887	0.000473233	374	7	388
NM_198857	-0.5056962	-1.8362374	0.843919887	0.000670632	355	7	352
NM_004461	-0.1616015	-1.8335011	0.650950747	8.54E-07	357	7	423
NM_004461	-0.2710667	-1.8001988	0.650950747	5.43E-05	3607	7	437
NM_016602	-0.0092415	-1.860909	0.909157162	0.025420434	1346	7	959
NM_015420	0.15955654	-1.2066919	1.416326347	0.025640844	1139	7	935
NM_015420	-0.5596436	-1.2220837	1.416326347	0.036422288	181	7	1040
NM_139279	-1.0450125	-1.9257222	1.071609131	0.022953879	194	7	1114
NM_002863	-0.2609178	-1.9479136	0.882933112	7.54E-05	360	7	201

NM_002863	-0.1697339	-1.6984957	0.882933112	0.012688357	307	7	651
NM_001496	0.46670325	-1.7488891	1.530902992	0.006040228	1092	7	802
NM_001496	-1.0980458	-1.4227896	1.530902992	0.008241916	4700	7	968
NM_178496	-0.0199769	-1.2942652	1.438391701	0.008773655	1204	7	754
NM_178496	0.43914822	-1.6344567	1.438391701	0.01796452	555	7	888
NM_145904	-0.038249	-1.745164	0.865151676	4.29E-05	3702	7	251
NM_002787	-1.9631841	-1.9672485	1.177533746	0.017385406	193	7	1153
NM_002787	-0.2888643	-1.6316713	1.177533746	0.023612703	1101	7	827
CN802860	-0.4162646	-1.6334354	1.048244282	0.00578729	473	7	532
NM_001870	-1.961628	-4.182811	1.391838241	0.015247042	16	7	1235
NM_001870	-0.7627812	-2.6755397	1.391838241	0.003168274	5353	7	995
CO580464	0.15292246	-1.7471722	1.180339015	0.002860745	549	7	473
CO580464	0.01243835	-2.0273514	1.180339015	0.012501229	309	7	755
NM_004436	-0.533654	-1.5216006	0.981699275	0.005685228	1679	7	516
NM_032038	-0.7806573	-2.0320644	0.638847161	0.000230673	351	7	711
BC044226	2.15316152	-1.7243138	1.23116051	0.007790629	5094	7	1093
BC044226	0.16497808	-1.2359609	1.23116051	0.005589158	4095	7	666
NM_001924	-1.4160483	-1.3227658	1.4860121	0.004101147	265	7	1065
NM_005700	-0.1675973	-1.7451153	1.562474306	0.01669362	1104	7	808
NM_174983	-0.3620743	-1.6831734	0.847889717	3.09E-05	350	7	339
NM_174983	-0.0629069	-1.6462667	0.847889717	5.93E-05	3704	7	289
NM_006396	-0.5663582	-2.4641963	1.192677921	0.001256412	295	7	594
NM_006396	-0.4095484	-2.2267224	1.192677921	0.000506843	296	7	380
NM_003051	-0.0625183	-3.0858987	1.42890618	0.015264748	86	7	1158
NM_000592	-0.2108284	-1.807787	0.706958736	0.000363132	367	7	471
NM_000592	-0.0283691	-2.0463385	0.706958736	6.05E-05	358	7	441
NM_003751	0.13526528	-1.7840557	0.71029223	0.008677027	1341	7	810
XR_010919	-0.0151195	-1.7420988	1.666807392	0.031689839	1106	7	1041
NM_024625	0.08355821	-1.8131185	1.171230787	0.002600149	396	7	357
NM_024625	0.22547326	-1.7944008	1.171230787	0.005376119	397	7	530
NM_006527	-1.2058247	-1.6412837	1.27946545	0.002887784	1416	7	970
NM_004837	0.6896462	-2.0408151	0.840962506	1.58E-05	574	7	717
NM_005333	0.10033925	-2.7548615	1.608299153	0.011238559	85	7	1091
NM_005950	-1.0492906	-1.9291746	1.321749857	0.009871993	210	7	821
NM_016059	0.18654891	-1.427809	1.122205436	0.00627215	553	7	719
NM_032490	0.12684321	-1.2546398	1.191989548	0.000877236	4094	7	498
NM_014450	-0.7788525	-1.9894357	1.071563797	0.000133735	471	7	952
NM_014450	-0.1941297	-2.0022653	1.071563797	0.00010108	364	7	72
NM_145808	-0.7718724	-1.537247	1.641471595	0.021714634	4701	7	1017
NM_145808	-0.82134	-1.4120915	1.641471595	0.028641318	180	7	1069
NM_001425	-0.6993556	-2.4893675	0.749757458	0.004049768	320	7	953
NM_001425	-0.1140051	-2.2093555	0.749757458	0.000685178	347	7	641
NM_014020	-0.3893694	-2.3180884	0.804670245	0.003184694	323	7	762

NM_014020	-0.4788977	-2.104126	0.804670245	0.002333983	324	7	623
NM_014982	-0.1406048	-1.8609146	1.532891871	0.006949587	1120	7	557
NM_005955	-0.058008	-1.57565	1.072568592	0.000451649	3780	7	298
NM_005955	-0.1237859	-1.8206451	1.072568592	0.000401169	572	7	274
NM_003596	-1.4821771	-1.6068371	1.287381669	0.003502117	65	7	1202
NM_003596	-0.4540577	-2.1278428	1.287381669	0.004103913	289	7	538
NM_183376	-0.3866734	-2.1469697	1.228003426	0.008893961	247	7	856
NM_183376	-0.5576932	-1.732495	1.228003426	0.013430436	245	7	790
NM_002827	-0.2076133	-1.5291707	1.383084886	0.008435383	1096	7	527
NM_002827	-0.4969403	-1.5105328	1.383084886	0.009114136	1095	7	595
XR_013467	0.13112148	-1.7983132	1.023266887	0.001362164	393	7	299
NM_030927	0.10716579	-1.8618137	0.865023694	0.000504853	391	7	346
NM_030927	-0.1634852	-1.9119273	0.865023694	0.001812108	390	7	415
NM_022338	-0.6329135	-1.5964355	0.88107133	0.000948288	486	7	625
NM_022338	-0.0780038	-1.8329371	0.88107133	0.000347578	359	7	261
XR_013264	0.03473696	-1.8648716	2.489115701	0.012416249	11309	7	1126
NM_178580	-0.3831242	-1.9981063	0.874715751	3.00E-06	361	7	166
NM_178580	-0.1511854	-1.5956491	0.874715751	2.69E-05	3703	7	286
NM_130446	-1.829243	-2.1203023	0.971271408	0.039285112	58	7	1221
NM_006509	0.46521636	-1.7497749	1.030376261	0.000165181	602	7	528
NM_006509	0.09497857	-1.7560759	1.030376261	2.48E-05	584	7	162
NM_006875	0.54744147	-1.4289739	1.129094476	0.001970578	607	7	799
NM_012335	-0.1577477	-2.0079469	0.744918568	6.55E-06	356	7	318
NM_006886	-0.0515863	-2.1915847	1.539271737	0.005770789	253	7	686
NM_006886	-0.30147	-1.9974151	1.539271737	0.00675953	252	7	587
NM_006886	-0.3831175	-1.951374	1.539271737	0.007036262	251	7	588
NM_006886	-0.3755659	-2.0445498	1.539271737	0.00773885	250	7	650
NM_004217	0.27212198	-1.2519469	1.529252975	0.004406454	4098	7	746
NM_004217	0.08378884	-2.0005902	1.529252975	0.008496369	5366	7	1124
NM_006461	-1.1630859	-1.8574555	0.825667213	0.041952859	100	7	1123
XR_012634	-0.5194731	-1.3861322	1.459075578	0.001405267	3968	7	324
XR_012634	-0.9040792	-1.991946	1.459075578	0.00189931	285	7	495
NM_175617	-0.0445996	-3.0888854	0.995871649	0.000188339	5437	7	978
NM_175617	0.19508647	-2.9988229	0.995871649	0.000249847	5438	7	972
NM_175617	-0.2439829	-1.5042071	0.995871649	0.003222842	3918	7	685
NM_175617	-0.0308323	-2.0916078	0.995871649	0.000570811	389	7	262
NM_182705	-0.0511202	-2.8922345	1.420184417	0.001827238	5439	7	930
NM_182705	0.18474104	-2.2637635	1.420184417	0.004984156	257	7	771
NM_014096	-0.5260397	-2.0500052	1.900601705	0.004242869	1118	7	834
NM_014096	-0.1823378	-2.1939229	1.900601705	0.004516895	1119	7	848
NM_004001	-3.0249299	-1.3911067	1.480491412	0.002487412	205	7	1134
NM_002318	-0.0754192	-1.6561061	2.002940636	0.005306471	1271	7	782
NM_001008211	0.69092839	-2.8352544	1.243622511	0.011356753	132	7	1146

NM_001008211	0.23308079	-1.6129876	1.243622511	0.004352052	550	7	567
NM_031459	0.24222337	-1.7529245	0.966329212	3.26E-05	586	7	332
NM_031459	-0.3009414	-1.8976581	0.966329212	6.57E-05	363	7	90
NM_004364	0	-3.1315894	0.981830383	0.046765306	120	7	1205
NM_004364	-0.1274367	-1.7644694	0.981830383	0.008850827	559	7	566
XM_375553	-0.3918328	-1.8613388	1.219064837	0.000508563	386	7	98
XM_375553	-0.6739524	-1.8750287	1.219064837	0.000236263	385	7	142
NM_001949	0.02943692	-1.6717312	0.760141066	0.011094817	1688	7	732
NM_015044	-0.3919972	-1.3669958	1.152962403	0.001967981	3840	7	409
NM_015044	-0.4234503	-1.4355905	1.152962403	0.001266025	3839	7	335
NM_025239	-0.1521742	-1.8328921	1.152735505	0.009896824	1348	7	744
NM_003975	-0.2091246	-2.2869446	0.812767837	0.00133127	346	7	761
NM_003975	-0.0503926	-1.73303	0.812767837	0.010743905	1350	7	880
NM_021149	0.29892422	-1.5916979	1.115578962	2.84E-05	589	7	306
NM_021149	0.46312678	-1.333984	1.115578962	0.000108762	625	7	630
NM_000399	-2.5245461	-2.0291616	1.288487435	0.007300317	49	7	1233
NM_000399	-1.8156764	-3.2254531	1.288487435	0.013297931	113	7	1212
NM_004096	-0.4376904	-3.3086158	1.477899349	0.000953288	277	7	1086
NM_004096	-0.2519698	-2.3912887	1.477899349	0.000256839	300	7	385
NM_012474	-1.878926	-1.4302161	1.226423794	0.000755064	37	7	1223
NM_012474	-0.1882293	-1.5668262	1.226423794	0.002344236	560	7	300
NM_015483	-0.2601064	-2.0708248	1.153146213	0.007437298	331	7	634
NM_013363	0.35689925	-2.3390512	1.143786284	0.004837645	1347	7	853
NM_013363	0.88935489	-1.5887408	1.143786284	0.00012851	150	7	1160
CO581798	0.01259627	-2.0608287	0.587977237	0.009248515	1340	7	917
NM_080605	-1.2777649	-1.9239208	1.389193987	0.000897488	64	7	1161
NM_080605	-0.8468567	-2.8035747	1.389193987	0.020164325	102	7	1174
NM_004416	0.14601733	-3.8477319	1.354060519	0.000294202	80	7	1190
NM_004416	-0.9714524	-2.4168008	1.354060519	0.00016186	5354	7	947
CO647394	-0.4184595	-1.2300044	1.274355645	0.000500156	3845	7	358
CN647236	0.20551223	-1.9470692	1.036605261	9.32E-06	583	7	189
CN647236	-0.0628668	-1.5136918	1.036605261	0.000525173	3849	7	328
NM_002275	-0.0391678	-1.3463052	1.190669849	0.000341748	3851	7	295
CN644516	-0.5573017	-1.3368186	1.414757919	0.001449259	3967	7	401
NM_016258	-0.4774589	-1.5063921	0.997416397	0.032713772	209	7	1043
NM_153236	0.2219225	-1.6752015	2.102771357	0.018906573	5869	7	1169
NM_138408	-0.0410885	-1.7949956	1.066058528	0.00135205	3809	7	231
NM_138408	-0.1715409	-1.6083101	1.066058528	0.003021893	3810	7	279
NM_007161	-0.2577492	-1.8920796	1.502280537	0.031618453	5471	7	1044
NM_007161	-1.3800965	-1.9158701	1.502280537	0.016616942	176	7	1139
NM_007161	-0.2900371	-1.8677752	1.502280537	0.017632232	1084	7	884
NM_007161	-0.2086544	-1.5037286	1.502280537	0.024304207	1085	7	906
NM_024575	-0.5233251	-2.9672103	0.893901201	5.89E-06	317	7	946

NM_024575	-0.439631	-3.1427846	0.893901201	2.67E-06	5436	7	996
NM_000952	-0.0682177	-3.4467584	0.512706923	0.011103762	78	7	1193
A_01_P007358	-0.4900197	-1.3786302	1.365606155	0.014644187	1098	7	735
NM_018000	-0.7224971	-2.5367202	0.962763186	0.00571223	114	7	1177
NM_018000	0	-1.4760948	0.962763186	0.001471	3848	7	404
NM_138392	-1.1018603	-1.8088158	0.763024908	8.95E-05	1433	7	866
NM_021625	-0.0518385	-1.9593496	0.917222437	0.004409953	306	7	591
NM_018973	-0.8678412	-1.6906868	1.339008694	0.002663373	3964	7	633
NM_018973	-0.6739458	-2.013338	1.339008694	0.000421172	446	7	174
NM_018973	-0.4012774	-1.6475659	1.339008694	0.000942182	3965	7	153
NM_018973	-0.5911115	-1.3853013	1.339008694	0.002587162	3966	7	485
NM_001188	0.21198275	-1.5730433	1.097302578	0.002296695	603	7	535
NM_001188	0.42566068	-1.6427371	1.097302578	0.000691359	604	7	569
A_01_P013825	-0.2341842	-1.2795651	1.374202554	0.010339255	4623	7	700
NM_005318	0.47694611	-1.3149028	1.385379637	0.013930195	1185	7	967
NM_198939	-1.0999202	-1.6634928	1.048196038	0.005748926	1374	7	1080
NM_012452	-0.2776794	-1.5422078	1.055590518	0.00053782	487	7	479
NM_016308	-1.1356008	-1.8077302	1.29615638	0.010240557	511	7	909
NM_016308	-0.9757448	-1.4804214	1.29615638	0.007143771	513	7	787
NM_017858	0.40075952	-1.3476564	1.175029017	0.037191304	131	7	1030
NM_000385	-0.8778651	-2.6738038	0.981858079	0.038543851	97	7	1167
NM_000385	-0.8185843	-1.6012745	0.981858079	0.007191814	1674	7	683
NM_002133	0.38725391	-2.1452725	1.27134254	5.87E-05	576	7	427
NM_002133	0.37293455	-1.6708573	1.27134254	0.000108269	592	7	341
NM_020861	-0.176175	-1.7741562	0.815678879	0.039429522	1686	7	966
NM_001198	-0.2818125	-1.4415328	1.973002099	0.004842785	1134	7	705
NM_002745	-0.2343621	-1.7575461	1.250962233	0.000158482	387	7	44
NM_002745	-0.5961992	-2.3191069	1.250962233	0.004056893	275	7	912
NM_006868	-0.6115137	-1.4241992	1.263753595	0.000139542	3945	7	221
NM_006868	-0.6666825	-1.4166642	1.263753595	0.000144705	3944	7	253
NM_014187	0.01485009	-1.5055127	1.056074947	2.56E-05	3852	7	211
NM_015969	0.52177973	-1.6193248	1.166505978	0.00728566	569	7	828
NM_014117	0.22063837	-2.3910357	0.796641787	0.012043607	1661	7	1000
NM_021107	-0.4188423	-1.530109	1.037890657	8.88E-05	3775	7	234
NM_021107	-0.2062	-1.603433	1.037890657	5.55E-05	3776	7	129
A_01_P003250	-0.9110066	-1.3041426	1.748011559	0.003085268	544	7	820
A_01_P003250	-0.8527511	-2.5466917	1.748011559	0.017904857	110	7	1157
CK230655	-1.3211558	-2.5886163	1.084442805	0.000595744	5398	7	1016
CK230655	-0.4732687	-2.8144252	1.084442805	5.21E-05	318	7	734
NM_178812	0.75500809	-1.5876992	0.86436869	0.01465353	558	7	1028
NM_177543	-0.7621559	-1.8570894	1.06736747	0.004033961	1414	7	1034
NM_177543	-0.4559252	-1.9744358	1.06736747	0.007213739	288	7	525
CN641710	-0.7263533	-2.5147113	1.4858776	0.001272508	282	7	632

CN641710	-0.8801849	-2.1361113	1.4858776	0.001319195	284	7	514
NM_007348	-0.3806585	-1.4304369	1.662150429	0.012310513	273	7	1014
NM_015140	0.00500899	-1.6625753	0.944162194	0.001568268	3773	7	419
NM_005335	-0.4365298	-2.4042909	1.261404368	5.39E-05	398	7	137
NM_005335	-0.5336353	-2.1846583	1.261404368	2.80E-05	399	7	104
NM_001218	0.19227577	-2.0414151	1.294314703	0.000151272	573	7	278
NM_001218	0.5204342	-2.0340837	1.294314703	0.000123467	577	7	558
NM_030969	-0.5812445	-2.2249357	0.956963451	0.043894594	124	7	1097
NM_005803	-0.6154711	-1.0799745	1.362975222	0.001546379	3977	7	722
NM_002949	0.10815354	-1.7675998	1.029611647	1.22E-05	587	7	218
NM_005533	-0.335444	-1.8595446	1.014010483	2.90E-05	362	7	61
NM_006818	-0.4365814	-1.45049	1.399125881	0.009326797	554	7	570
NM_004838	0.12439686	-1.8201805	1.081155183	5.57E-06	585	7	132
NM_004838	-0.0903215	-1.539424	1.081155183	2.75E-05	3777	7	134
NM_022917	-0.3323982	-1.6743624	0.864417694	0.001218212	373	7	435
NM_000089	-0.9833318	-2.3379084	1.193489729	8.12E-06	279	7	823
NM_000089	-0.3399163	-2.1969538	1.193489729	1.04E-05	402	7	31
NM_014297	-3.1194028	-2.0768509	1.31170172	0.007701806	2	7	1237
NM_014297	-0.4618583	-1.840508	1.31170172	0.000720687	450	7	120
NM_031286	-0.6493373	-1.449985	1.058540721	0.000232757	3943	7	378
NM_000314	0.2901099	-1.5971899	0.999839873	0.035786261	561	7	971
NM_013375	-0.3302168	-1.5585314	1.106965384	0.000305144	3818	7	159
NM_003358	-0.4172177	-2.3663556	1.245496485	0.026591679	118	7	1127
NM_017585	0.17808723	-2.1020009	1.47561916	0.000956639	802	7	342
NM_017585	0.08908433	-2.0118877	1.47561916	0.000811034	803	7	210
NM_002086	-1.0448591	-2.6244983	1.350519125	0.000309184	5399	7	903
NM_002086	-0.9080638	-2.9944634	1.350519125	0.000394275	276	7	974
NM_022044	0.15693731	-1.845791	1.460177802	0.000300277	806	7	144
NM_022044	-0.2343116	-2.2905391	1.460177802	0.006279741	297	7	706
NM_153374	0.39357708	-2.1608991	1.640951767	0.000856949	580	7	428
NM_014400	0.49098114	-1.57753	1.226439563	0.002631621	1358	7	944
NM_014400	0.76899428	-1.8395587	1.226439563	0.002282466	570	7	900
NM_001679	-1.1615718	-1.205979	1.418062328	0.000660066	3578	7	918
NM_001679	-1.2042513	-1.2981444	1.418062328	0.012660346	517	7	1032
NM_018438	0.00043527	-2.0390088	1.363785073	0.00179024	1349	7	379
NM_018438	0.56230602	-1.8199021	1.363785073	5.65E-05	593	7	490
NM_024734	0.36626262	-1.3524517	1.304705128	0.000342585	4096	7	482
NM_194247	-0.7379621	-1.7472554	1.402203208	0.011611108	292	7	791
NM_001610	-0.8873316	-2.5657219	1.302909803	6.22E-05	280	7	620
NM_001610	-0.308717	-2.3858781	1.302909803	0.000292601	403	7	197
NM_199203	-0.1815214	-1.4112453	1.595674183	0.005368375	1194	7	571
NM_199203	-0.3092665	-1.6571008	1.595674183	0.003808133	478	7	739
NM_013439	-0.4118194	-2.3605634	1.154812746	5.86E-05	319	7	244

NM_013439	-0.1182613	-1.8923277	1.154812746	1.97E-05	480	7	243
NM_031452	-0.6734535	-1.4779789	1.589052932	0.00297091	490	7	813
NM_031452	-0.603295	-3.3122642	1.589052932	0.028382919	121	7	1216
NM_031452	-0.1323589	-2.1619124	1.589052932	0.002655343	310	7	350
NM_031452	-0.235116	-1.966344	1.589052932	0.004885077	312	7	431
NM_031452	-0.5921084	-1.3229931	1.589052932	0.003744155	4702	7	680
NM_006848	-0.2554397	-1.8875231	0.877446898	0.000154288	372	7	285
NM_006848	-0.5051123	-1.8047956	0.877446898	4.76E-05	354	7	272
NM_032509	-0.6078833	-1.6368017	1.004982177	0.021122112	1702	7	847
NM_052850	-0.7325744	-1.4199873	1.084218019	0.004074696	3940	7	690
NM_003327	-0.0390082	-3.09259	1.451205883	0.001318279	83	7	1058
NM_003327	-0.0620849	-1.442932	1.451205883	0.004248136	3929	7	544
NM_021939	-0.3447828	-1.6943647	1.4331208	0.002789965	3928	7	407
NM_021939	0.1285521	-1.8222492	1.4331208	0.001017045	832	7	230
CN801916	-0.1072659	-1.4012548	1.210826885	0.000503641	3873	7	200
NM_031476	-0.2233213	-1.6176262	1.208629984	0.001051678	3926	7	208
NM_031476	-0.2431217	-1.473741	1.208629984	0.000119558	3778	7	131
NM_018141	-0.3462772	-1.6253888	1.149571487	0.000125499	3797	7	91
NM_018141	-0.0699925	-1.6509688	1.149571487	0.000403341	3847	7	94
NM_006367	-0.085949	-1.2980029	1.293219024	2.13E-05	3855	7	194
NM_006367	-0.0687633	-1.3699452	1.293219024	0.000184599	3863	7	177
NM_003002	-0.4856598	-1.7712487	1.119798408	4.77E-05	3796	7	65
NM_003002	-0.4769399	-1.9296631	1.119798408	0.000781019	3808	7	163
NM_145256	0.27184738	-2.1719054	1.263426881	3.78E-05	575	7	202
NM_145256	0.42057784	-1.8640413	1.263426881	3.18E-05	591	7	308
NM_014035	-0.8210653	-1.0968297	1.332972227	0.012575164	179	7	992
NM_003578	-0.4176732	-1.9770645	1.173031377	0.015213226	1093	7	904
NM_016817	-0.0134975	-1.4436158	1.819703161	0.003624459	1121	7	617
NM_020990	-0.1604576	-1.9331292	1.21814486	0.003487847	3925	7	406
NM_020990	-0.1314469	-1.5506109	1.21814486	0.004025967	3927	7	467
NM_020158	-0.2262092	-1.8298711	1.624617718	0.007280309	256	7	723
NM_024769	-0.4863404	-2.1234538	1.253892763	0.00338928	305	7	613
NM_024769	-0.1341671	-1.432447	1.253892763	0.000738153	3814	7	315
NM_006912	-0.5360317	-1.754953	1.079020418	0.000647074	3806	7	133
NM_002568	-0.4925006	-1.4014948	1.239133444	0.000429997	3844	7	232
NM_002568	-0.5984446	-1.3402469	1.239133444	0.000791888	3841	7	345
NM_022768	0.09264134	-1.8298341	0.822409775	0.028943979	1690	7	958
NM_002068	0.10101793	-2.0700965	1.282098762	0.000863385	578	7	277
NM_002068	-0.1733369	-2.1945349	1.282098762	0.000543848	479	7	440
NM_024775	0.12931557	-1.6706332	1.438953773	0.01840814	1111	7	868
NM_023009	-0.0317862	-2.5754523	1.844526003	0.000639706	5598	7	676
NM_021035	0.00153869	-1.4545645	1.277361863	0.000229344	3782	7	305
NM_021035	-0.0350627	-1.3271437	1.277361863	1.71E-05	3856	7	216

NM_006228	0.10183451	-2.3668342	0.826157548	0.000262661	345	7	597
NM_006228	-0.0477647	-2.3636045	0.826157548	0.000124872	344	7	497
NM_001628	-0.0620916	-1.9150095	1.121116465	2.56E-05	581	7	23
NM_001628	0.10118466	-1.5397279	1.121116465	6.04E-05	3864	7	168
NM_002247	0.02659419	-2.2900592	1.230713674	0.000109893	406	7	173
NM_002247	0.44152875	-1.9097308	1.230713674	0.000398224	590	7	403
NM_006153	-1.1968655	-1.5460218	1.124181444	1.61E-06	66	7	1187
NM_006153	0.11612262	-1.426255	1.124181444	0.000136958	3815	7	396
CB309708	-0.3017584	-1.5010127	1.245608099	0.00393769	3883	7	400
CB309708	-0.4962619	-1.2796245	1.245608099	0.003179954	3842	7	496
NM_007284	0.16595894	-1.5877347	1.180965802	2.52E-07	588	7	160
NM_007284	0.03008527	-1.5145061	1.180965802	2.98E-07	3854	7	106
NM_025080	-0.3447636	-1.9614814	1.341610608	0.002157069	475	7	245
NM_025080	-0.4038417	-1.9073636	1.341610608	0.004689601	474	7	515
NM_016582	-0.5360101	-1.744357	1.130685843	0.001199153	459	7	291
NM_016582	-0.2846343	-1.9468324	1.130685843	9.62E-05	380	7	45
NM_198390	-0.2653608	-1.7865997	1.083661102	0.000214541	3811	7	119
NM_198390	-0.5839701	-2.2130331	1.083661102	0.004331542	302	7	573
NM_002101	0.50319494	-1.4368522	1.299959577	0.000110602	626	7	546
NM_013314	-0.6433244	-1.3031348	1.192126199	0.000349131	3970	7	534
NM_016021	-1.0065872	-2.21802	1.124424253	0.003665411	290	7	759
XR_014088	-0.0332133	-1.9610081	1.448079794	0.000409591	801	7	111
XR_014088	-0.0400291	-2.1688834	1.448079794	9.73E-05	800	7	36
NM_080655	-1.6673002	-1.7168938	2.177432603	0.007988711	5362	7	1166
XM_934556	-0.358695	-2.2617989	0.819718739	0.009097928	1660	7	819
XR_014443	0.07616451	-2.0922659	0.976118462	0.00714969	298	7	699
XR_014443	-0.25135	-1.4762106	0.976118462	0.000117597	3819	7	241
NM_003782	-0.1964912	-2.4036183	1.902909013	0.000994371	5596	7	646
NM_003782	-1.6135779	-1.8193425	1.902909013	0.001843144	20	7	1211
NM_002738	0.64801051	-1.7529503	1.197627396	1.09E-05	613	7	776
NM_002738	0.59439644	-1.789849	1.197627396	0.001066018	567	7	639
NM_002795	1.52817566	-1.6716549	1.016282406	1.98E-05	5098	7	1152
NM_002795	1.43327532	-1.8272901	1.016282406	0.000341525	5097	7	1129
NM_001803	-0.3280239	-2.4417288	1.119273423	3.85E-05	400	7	213
NM_001803	-0.2741504	-2.372806	1.119273423	2.62E-05	401	7	146
CN647252	-0.2942284	-1.2258807	1.335905085	0.00180616	704	7	445
CK232214	-0.740557	-1.3269903	1.207778827	0.00168904	3959	7	579
XR_011615	-0.5561077	-1.4895785	1.698105476	0.003777038	1097	7	545
DQ266251	0.15371787	-1.342038	1.212536107	0.001022924	712	7	470
NM_019594	-0.3512738	-1.1859736	1.331493287	2.17E-05	488	7	638
NM_019594	0.3111705	-1.6049248	1.331493287	0.00650564	568	7	619
NM_014313	-0.4673321	-1.6067288	1.406351569	8.17E-05	753	7	51
NM_014313	-1.0275802	-1.2381659	1.406351569	0.001115827	3579	7	831

NM_016135	0.20115481	-2.6394661	1.375782983	0.000430313	329	7	636
NM_016135	-0.2563628	-1.8584161	1.375782983	4.07E-05	756	7	15
NM_024660	-0.0174918	-1.7631145	1.41525285	1.44E-05	3858	7	11
NM_024660	-0.0418114	-1.6235127	1.41525285	0.00019098	3860	7	40
NM_004460	-0.2431662	-1.7695127	2.48014541	0.018872405	5488	7	1145
NM_003000	-0.0120881	-1.5322934	1.08273054	0.003852988	3850	7	438
NM_213720	-0.011914	-1.639756	1.823038121	0.000986025	834	7	344
NM_030796	-0.2735862	-2.2732641	1.397313241	0.006571746	255	7	921
NM_030796	-0.3546719	-2.2655347	1.397313241	0.003964009	249	7	596
NM_013376	-0.0961903	-2.1913501	1.435646771	0.000229305	455	7	126
NM_013376	-0.7271895	-1.99978	1.435646771	0.000642629	449	7	312
NM_021203	0.19757989	-1.7020439	1.652958951	0.000386224	825	7	214
NM_021203	-0.1204111	-1.5129132	1.652958951	0.001527755	552	7	365
NM_022763	-0.1510884	-1.8151489	1.059490048	0.012252623	308	7	655
NM_015886	-0.9556537	-1.306845	1.689504739	0.003448169	268	7	926
NM_178443	-0.0430516	-2.2815543	1.149317297	6.44E-06	408	7	114
NM_178443	-0.048524	-2.1452524	1.149317297	9.27E-07	409	7	43
NM_178443	-0.0467121	-1.896764	1.149317297	2.31E-05	381	7	53
NM_178443	0.19243984	-1.8704159	1.149317297	1.50E-06	582	7	101
NM_016481	-0.5282288	-1.0747008	1.36258403	0.006912773	698	7	758
XM_375359	-0.4958595	-1.6681277	1.146360683	0.009455171	1100	7	559
NM_006516	0.44653706	-2.9439489	1.002460421	0.00296585	5446	7	1079
NM_006516	0.38807551	-1.4366582	1.002460421	0.000466281	619	7	709
NM_014056	-0.1763129	-1.4916236	1.336478034	0.000236631	3861	7	64
NM_005507	-0.6745399	-1.3974781	1.316383294	7.09E-05	3947	7	296
NM_005507	-0.6624671	-1.4461233	1.316383294	2.01E-06	3948	7	180
CN804946	-1.2018072	-1.710063	1.163472229	4.56E-05	1377	7	1049
NM_003821	-0.1025094	-1.6673839	2.167833334	0.005246964	1128	7	919
NM_078468	0.0974231	-1.5142944	1.12665152	0.018600942	562	7	780
NM_000732	-0.0260208	-1.4893675	1.163413562	0.009736486	3987	7	689
XR_011517	-0.8161267	-1.264619	1.228118168	0.005187148	658	7	698
NM_007364	0.01771054	-1.3876242	1.295532419	0.000701017	3876	7	239
NM_007364	-0.0057854	-1.2015264	1.295532419	0.001009466	3877	7	429
NM_003177	-0.1044105	-3.2153418	1.149759491	2.63E-06	5440	7	985
NM_003177	0.46064371	-3.1373279	1.149759491	3.66E-05	5441	7	1033
CN644186	-0.2088375	-1.2700682	1.468280431	0.000922704	705	7	330
NM_016507	-0.4987484	-2.4670203	1.057407739	0.010407437	304	7	951
NM_001235	0.20931223	-1.8871044	1.067015677	0.000650314	382	7	371
NM_001235	0.05976291	-1.9796684	1.067015677	3.48E-05	410	7	135
CN648004	-0.5120798	-2.268672	1.537848388	4.51E-05	452	7	95
CN648004	-0.3105909	-1.9960686	1.537848388	3.03E-05	763	7	6
CN648004	-0.0511677	-1.9227759	1.537848388	4.55E-05	764	7	16
CN648004	-0.0513555	-1.8913686	1.537848388	8.17E-05	776	7	9

NM_001002836	-0.332439	-1.4906892	1.188582431	9.91E-05		3822	7	167
NM_001002836	-0.3172873	-1.5150558	1.188582431	2.17E-05		3821	7	79
NM_013336	0.2068917	-1.4832954	1.281630272	2.19E-05		3865	7	205
NM_003405	-0.3095366	-2.0059613	1.226931018	0.001201385		3800	7	102
NM_003405	-0.3089902	-1.6830592	1.226931018	0.000524165		3803	7	47
NM_018683	-1.0957528	-1.6437481	1.24445774	0.002300484		512	7	729
NM_145201	-0.8127764	-1.504284	1.236711523	0.001332146		3933	7	518
NM_145201	-0.5765098	-1.4041701	1.236711523	0.002070565		3934	7	469
NM_000689	-0.4990225	-1.5108525	1.067571074	9.18E-05		3817	7	220
NM_000689	-0.343366	-1.6117245	1.067571074	0.000254925		3799	7	121
NM_006402	-0.5277326	-1.4803503	1.135788959	0.003065883		3795	7	391
NM_006402	-0.3586056	-1.3382753	1.135788959	0.002833417		3827	7	412
NM_004753	-0.5116401	-1.1916339	1.367432338	0.000124457		3992	7	413
NM_004753	-0.3602403	-1.2755216	1.367432338	6.65E-05		3846	7	188
NM_016454	0.08995011	-1.4337551	1.065918301	0.006692659		3794	7	648
CN803456	0.79227424	-1.4829117	1.847375777	0.029895792		1338	7	1137
NM_001611	-0.3367677	-1.783189	1.453588661	0.000188718		762	7	20
NM_001611	-0.3587167	-1.8065728	1.453588661	3.00E-05		752	7	12
XR_012020	0.82425847	-1.67533	1.713697866	0.013430699		133	7	1075
NM_015444	0.44405009	-2.3554657	2.362609137	0.003900178		5269	7	1206
NM_000034	-0.7952386	-1.3530109	1.303969794	0.000105348		3946	7	425
NM_000034	-0.6527735	-1.3991366	1.303969794	4.28E-05		659	7	206
NM_174896	-0.5138748	-2.3263028	1.515551895	0.002105996		248	7	542
NM_018101	-0.258126	-2.0940253	1.386766353	0.02521944		5470	7	1063
XR_014799	-0.9562601	-1.5430614	1.053140957	0.000292774		202	7	877
NM_005720	-0.1907962	-2.0200597	1.521639259	6.21E-05		774	7	5
NM_005720	-0.2036472	-1.9899815	1.521639259	0.000146219		775	7	7
NM_000147	-0.3460734	-1.5776271	1.251168715	0.000722895		3804	7	99
NM_000147	-0.2997153	-1.6150855	1.251168715	0.001283985		3805	7	112
NM_004468	-0.5473417	-2.5055286	1.189744287	0.010228492		125	7	948
NM_004468	-0.5980944	-2.2771756	1.189744287	0.000861537		281	7	526
NM_006145	-0.0777013	-1.4763707	1.29438274	0.000131102		3862	7	87
NM_016034	-0.3506708	-1.3740899	1.644570971	0.001406584		1192	7	424
XR_010624	-0.1044234	-1.432072	1.909258001	0.000704522		878	7	488
NM_002632	0.27726155	-1.5741518	1.660150962	0.001294419		833	7	510
NM_002632	0.03319624	-1.7060624	1.660150962	0.000556317		492	7	896
NM_144580	0.15912127	-1.4747346	1.335291115	0.000120813		3867	7	175
NM_144580	0.09269688	-1.1651337	1.335291115	9.19E-05		4236	7	446
CN647641	-0.3260757	-2.197245	1.599060104	0.00062755		453	7	207
CN647641	-0.2245446	-1.7791066	1.599060104	0.000152677		765	7	30
NM_005248	-0.101314	-1.2801644	1.364458743	0.000212069		3875	7	256
NM_005248	-0.27524	-1.4126197	1.364458743	0.000923815		3872	7	165
XR_009904	-0.1958058	-1.7606491	2.233945478	0.02229801		1116	7	1103

NM_012088	-0.2674491	-2.2460382	1.095112683	0.00176682	330	7	411
NM_012088	-0.4227755	-1.6136872	1.095112683	0.000152673	464	7	288
NM_005472	-0.1374161	-1.6999948	0.812276122	0.003633051	1025	7	628
NM_004618	-0.180979	-1.1412963	1.33657716	0.002588299	4031	7	606
NM_006327	-0.5228219	-1.6218388	2.185039965	0.004390633	1132	7	887
NM_003091	-0.6843085	-1.1427658	1.426775113	0.002135709	3963	7	691
NM_000405	0.29012043	-1.8991587	1.409964543	2.77E-06	811	7	108
NM_000405	0.17192325	-2.0121344	1.409964543	1.32E-06	810	7	34
NM_000480	0.24877092	-2.824837	1.768924247	0.004359894	5428	7	1057
NM_000480	0.1602597	-2.2630076	1.768924247	0.000437857	258	7	348
NM_015991	-0.182746	-1.7984524	2.216478477	0.017579887	1315	7	1099
NM_015991	-0.0641672	-1.8673388	2.216478477	0.007266164	1020	7	964
NM_144723	1.30359278	-1.5037893	0.971290688	0.000374602	151	7	1222
NM_144723	-0.1555957	-1.5265487	0.971290688	0.003993777	645	7	420
CB310604	-0.635132	-1.9252201	1.095549313	0.000218395	458	7	287
CB310604	-0.430923	-1.9868121	1.095549313	0.000117866	461	7	179
NM_016498	0.16814918	-1.4215296	1.392595526	0.000685765	610	7	418
NM_016498	0.28780822	-1.5916925	1.392595526	0.000295948	3868	7	240
NM_016305	0.39698233	-1.4775279	1.239518958	9.75E-05	623	7	460
NM_016305	0.34251895	-1.2968685	1.239518958	0.000185281	624	7	547
NM_024053	0.23612443	-1.606615	1.252846553	0.001491162	609	7	451
NM_024053	0.32627877	-1.2564657	1.252846553	0.000198136	611	7	603
CO579644	-0.8808496	-1.0844057	1.4085828	0.000309419	4765	7	779
NM_000064	-0.5434637	-1.2263106	1.378461967	0.004273876	3962	7	654
NM_000064	-0.5790196	-1.2362206	1.378461967	0.000546547	3961	7	455
NM_152346	-0.335141	-1.5681148	1.177750862	0.000182318	466	7	219
NM_152346	0.16110148	-1.5759594	1.177750862	0.000392792	596	7	248
NM_080671	0.34254108	-1.8332288	1.619825689	0.002332588	820	7	543
NM_080671	-0.2656444	-1.4675999	1.619825689	0.002086487	640	7	439
NM_003359	0.0952306	-1.8493867	1.289458431	0.000427605	3812	7	172
NM_003359	-0.2343595	-1.9198884	1.289458431	0.001545753	3801	7	122
NM_020701	-0.5232345	-2.1363359	1.188431536	4.55E-05	407	7	62
NM_002775	-0.1232753	-2.1416866	1.413437648	1.43E-05	411	7	14
NM_002775	0.11088573	-1.7316375	1.413437648	1.92E-05	3859	7	21
NM_030627	-0.3927201	-1.3531551	1.204433682	0.011626937	5370	7	1144
NM_024711	0.61647998	-1.6976797	1.571686569	0.000211515	1014	7	795
NM_024711	0.35113706	-1.2980835	1.571686569	5.31E-05	935	7	642
NM_032283	-0.0701492	-1.5487966	1.310278053	7.16E-07	3853	7	35
NM_032283	0.08699082	-1.3086199	1.310278053	2.33E-07	3857	7	268
NM_054023	0.14125334	-1.8783027	1.30019271	0.000225089	579	7	117
NM_054023	0.06430673	-1.6006587	1.30019271	0.000258297	3866	7	83
NM_000303	-0.4517035	-2.6749203	1.731074905	0.000913394	5417	7	857
NM_000303	-0.7138052	-2.4748958	1.731074905	5.22E-05	442	7	449

NM_001061	-0.4489849	-2.4487183	1.120749298	0.022572424	126	7	1050
NM_001061	-0.1270163	-1.474265	1.120749298	0.001044004	3813	7	422
NM_080388	-0.6049389	-1.3152098	1.382605179	6.72E-05	3949	7	281
NM_080388	-0.6072778	-1.3091314	1.382605179	4.74E-05	3950	7	297
NM_002403	0.51594007	-2.1681364	1.493051208	0.003040174	1161	7	855
NM_002403	-0.1802927	-1.6305902	1.493051208	0.010013854	3982	7	778
NM_176812	-0.1922945	-2.2235415	1.289195508	0.000659735	405	7	183
NM_176812	-0.0132579	-1.5828822	1.289195508	0.000695884	3807	7	138
XM_933457	-1.2862876	-1.4095244	1.67642238	0.002026363	4690	7	937
XM_933457	-1.1169714	-1.596041	1.67642238	0.002664897	4691	7	815
NM_002620	-0.6144597	-2.0752799	1.633723411	0.000124157	448	7	140
NM_002593	-0.9174649	-1.8336159	1.716711458	0.00103982	451	7	564
NM_002593	-0.7593914	-2.0470092	1.716711458	8.88E-05	447	7	252
XR_013647	-0.6704863	-1.1754845	1.336434737	0.000133703	3582	7	541
XR_013647	-1.0649011	-1.2345362	1.336434737	0.000502075	3969	7	803
NM_002975	-0.007694	-1.6739811	1.049106587	0.000415968	3784	7	247
NM_002975	-0.3815931	-1.5941829	1.049106587	0.000155127	3798	7	151
NM_006455	-0.320551	-1.7929293	1.959983102	0.032113494	262	7	1149
NM_006455	-0.2625782	-1.6767021	1.959983102	0.028768934	1321	7	1083
NM_012106	-1.4342794	-2.7974015	1.851973247	0.004839747	115	7	1171
NM_012106	-1.1866684	-2.2167088	1.851973247	0.005517528	5372	7	1140
NM_148976	-0.821352	-1.9310847	2.089535868	0.003294642	518	7	860
NM_148976	-0.4791241	-1.7715887	2.089535868	0.00438244	522	7	897
DR774547	-0.4465804	-1.3678683	1.738721119	0.005442625	1197	7	716
XR_010024	-0.75565	-2.5714738	1.59984508	0.001796548	278	7	845
NM_005443	0.57666225	-1.7036709	1.185853242	0.000142668	614	7	640
NM_003929	-0.0063001	-1.7180295	2.148097189	0.024221625	1316	7	1115
NM_003929	0.25308688	-2.3683699	2.148097189	0.022997642	5347	7	1168
NM_004155	0.70507437	-2.2934496	2.274626793	0.006634362	5865	7	1141
NM_004155	-1.7157593	-2.2058157	2.274626793	0.005757745	5482	7	1165
NM_006864	1.49127374	-1.6222147	1.354173273	0.000545692	5099	7	1135
NM_006864	0.95502876	-1.3911722	1.354173273	0.000788429	632	7	973
NM_144653	-0.4272986	-1.8831863	1.378952232	7.41E-05	476	7	259
NM_144653	0.15213036	-1.7719715	1.378952232	9.84E-05	595	7	48
NM_014164	-0.5929369	-1.4258674	1.286886641	0.006355046	3935	7	671
NM_014164	-0.4877223	-1.4137709	1.286886641	0.003412512	3936	7	487
NM_015971	-0.4983496	-1.0783008	1.391825614	9.13E-05	4140	7	524
NM_015971	-0.4332816	-1.277577	1.391825614	2.68E-05	4010	7	236
NM_003403	0.24422332	-1.2846044	1.407016739	0.003917254	713	7	618
NM_005084	0.08940152	-1.9146534	2.538215418	0.007687738	5489	7	1102
NM_006793	-0.3731438	-1.3500008	1.579384605	0.000131115	706	7	169
NM_006793	-0.186229	-1.7590882	1.579384605	0.004767074	313	7	347
NM_004053	-0.0987809	-1.5825214	2.069988579	0.009137177	1317	7	1002

XR_014267	0.20089908	-1.8146416	0.817547515	0.008316708	1342	7	829
NM_006120	-0.0569901	-2.1352984	1.574064792	0.000124047	804	7	63
NM_006120	0.10422572	-1.9121171	1.574064792	9.51E-05	805	7	70
XR_013405	0.62129147	-1.9670945	1.551242201	6.15E-06	812	7	103
XR_013405	-0.4268532	-1.5785855	1.551242201	2.73E-05	477	7	182
NM_014266	-0.9259786	-2.0749719	1.715458365	0.017430039	259	7	1068
NM_014266	-0.3247843	-1.8262285	1.715458365	0.024786658	260	7	1038
NM_016235	0.29619699	-3.0165228	1.611974744	5.97E-06	5448	7	950
NM_016235	0.39347384	-3.1785979	1.611974744	0.000118009	5447	7	1111
CB310061	-0.2463432	-1.6231242	1.789629176	0.000172585	797	7	82
NM_002205	-0.1845789	-1.5923191	1.685808102	0.002156873	836	7	398
NM_018075	-0.2987604	-1.2934423	1.355392937	0.000656537	3816	7	616
XR_010663	0.1472178	-1.8347024	1.71805644	0.002352879	1112	7	461
NM_025159	-0.2139718	-3.6578114	1.342390924	5.39E-06	5433	7	1173
NM_025159	-0.3664923	-2.9221686	1.342390924	4.61E-05	5442	7	730
NM_003136	-0.9615048	-1.606494	1.818826563	0.000355934	528	7	773
XR_013743	-0.2253279	-2.3009177	1.596588063	7.22E-05	417	7	73
XR_013743	-0.4131358	-1.9616395	1.596588063	1.64E-05	751	7	10
XM_928586	-0.0615139	-1.5340096	1.051374668	0.004515613	3793	7	508
CN804242	-0.0360416	-1.5649139	1.389319758	0.010227392	563	7	562
CN804242	0.13008192	-1.3596455	1.389319758	0.0058599	723	7	553
NM_016823	-0.3441827	-2.9352515	1.240592134	0.00119846	5434	7	1066
NM_016823	-1.1608084	-2.650421	1.240592134	0.008648775	116	7	1151
NM_022488	-0.5696183	-2.128677	1.663351717	0.001846767	293	7	504
CO646712	0.02598995	-1.6172411	2.168450566	0.002436663	1131	7	835
XR_011777	-1.0099414	-3.0789565	1.519101716	0.005337117	5407	7	1148
XR_011777	-1.0478647	-2.9919858	1.519101716	0.004177151	5408	7	1121
NM_015603	-0.8226316	-1.5656162	1.007361862	0.007975215	3930	7	796
NM_080757	0.41260528	-2.6407822	1.651055691	6.55E-05	5611	7	738
NM_080757	0.42779979	-2.5080829	1.651055691	2.11E-05	5612	7	586
NM_000660	-0.7689501	-2.4440757	1.277965082	0.001371129	5418	7	724
NM_000660	-0.507377	-2.2327866	1.277965082	0.001077438	5419	7	399
NM_022733	-0.2525427	-2.0071408	1.80704108	3.32E-05	781	7	22
NM_022733	-0.1296187	-1.7700756	1.80704108	3.58E-05	782	7	29
NM_012310	-0.3778283	-1.3455219	1.54755089	0.001053752	638	7	414
NM_032231	-0.2924049	-1.8481385	1.310252031	0.001328844	493	7	454
NM_032231	0.09226495	-1.4219867	1.310252031	0.001428804	711	7	336
CO725352	-0.2194124	-2.1722261	1.261395457	0.000104648	404	7	69
CO725352	-0.32051	-1.8577233	1.261395457	0.000317246	3802	7	27
NM_181803	-0.138768	-1.666506	1.861536379	0.001291012	837	7	366
NM_181803	0.06524342	-1.727097	1.861536379	0.000816165	1123	7	329
XM_051271	-0.5448555	-1.8024004	0.915748784	0.028901997	99	7	924
CN644464	-0.5589132	-1.2699198	1.231415059	0.005568967	3937	7	693

CN644464	-0.8740172	-1.2999535	1.231415059	0.004297113	3932	7	786
NM_022367	-0.8373809	-2.7992522	1.076936084	0.000539813	5429	7	1020
NM_022367	-0.4432753	-2.0896968	1.076936084	0.005460019	326	7	697
NM_000803	-0.4972989	-1.9358819	1.428505534	0.001785369	482	7	367
NM_000803	0.43380091	-1.4176498	1.428505534	0.001265243	612	7	658
NM_001008860	-0.1968958	-1.4896448	1.642720649	0.003879269	1193	7	492
NM_001008860	-0.4618344	-1.4563291	1.642720649	0.003209858	534	7	447
XR_009902	-0.0660043	-1.7530456	1.764331087	5.97E-05	783	7	32
XR_009902	0.14289597	-1.751717	1.764331087	0.000172591	784	7	156
NM_003942	-0.2069373	-1.3765839	1.454803145	0.000538293	3999	7	242
NM_003942	0.22110467	-1.5643276	1.454803145	9.72E-05	936	7	199
NM_004255	-0.0284169	-1.8059271	1.612258147	0.000505143	780	7	58
NM_004255	-0.0499349	-1.80277	1.612258147	0.00058726	779	7	56
NM_018482	0.35860366	-1.41238	1.711243789	0.000668929	5107	7	1042
NM_003144	-0.1526817	-1.6246023	1.626449665	0.000721089	788	7	76
NM_003144	-0.0637319	-1.3688772	1.626449665	0.000413083	716	7	235
NM_006136	-0.0369426	-2.0340281	1.612961598	0.002433442	311	7	322
NM_003872	0.41814864	-1.3797623	1.342688258	0.000250237	627	7	509
NM_017510	-0.0302882	-1.5167299	1.51306832	4.72E-05	3871	7	71
NM_017510	-0.2016629	-1.4389433	1.51306832	5.46E-05	3869	7	66
NM_017458	-0.3375811	-2.0619443	1.559362992	0.000191282	454	7	52
NM_017458	-0.1890052	-1.6422763	1.559362992	0.000101333	766	7	41
NM_001001188	0.49167117	-2.8589733	1.486045073	1.84E-05	5451	7	1051
NM_001001188	-0.1637802	-2.3412345	1.486045073	0.000821253	299	7	325
NM_012296	-1.4627178	-1.9386779	1.636078908	0.007607731	5373	7	1070
NM_012296	-0.6590913	-1.7119776	1.636078908	0.008581272	516	7	737
NM_004069	0.12681837	-1.4234359	1.714279064	2.30E-05	891	7	229
NM_004688	0.245065	-1.7740135	1.70576212	0.001491817	823	7	355
NM_004688	-0.052921	-1.3292665	1.70576212	0.000758142	721	7	316
NM_022978	-1.1521219	-1.3521417	1.593325879	0.011452191	5367	7	1104
NM_005868	-1.1420571	-2.3284014	1.054332563	0.003348577	274	7	969
NM_005868	-1.6108365	-2.2169721	1.054332563	0.029765101	127	7	1095
NM_206831	-0.4685339	-1.3478914	1.770234766	0.001333394	4703	7	777
NM_004268	-1.1031268	-1.4995896	1.284778101	0.001720785	515	7	789
NM_020979	-0.1122823	-1.3243025	1.227716496	0.000239664	3996	7	282
NM_016226	-0.1548698	-1.3147963	1.154235322	0.000649227	3788	7	450
NM_016226	-0.7767344	-2.0552163	1.154235322	0.021411796	103	7	1064
NM_015424	1.82887158	-2.0054704	1.975284448	0.002652197	153	7	1210
CO579438	-0.1468692	-1.8759458	1.535459977	1.37E-07	760	7	1
CO579438	-0.1001259	-1.2525536	1.535459977	7.68E-05	719	7	228
NM_016343	-1.1370212	-1.863246	1.490106453	0.011987559	175	7	1098
NM_016343	0.12355438	-1.1992723	1.490106453	0.001941716	714	7	602
XR_013476	-0.3500078	-1.1185525	1.44592893	1.48E-05	4011	7	394

NM_006475	-0.3701126	-2.5087697	1.457198653	4.92E-05	412	7	226
CO646475	-0.1731896	-2.0571439	1.488404285	1.51E-06	757	7	3
CO646475	-0.1214664	-1.8631983	1.488404285	1.46E-06	759	7	2
CB550386	0.17422764	-1.2047683	1.355775146	0.00242679	724	7	568
NM_001109	-0.5062497	-2.0419591	1.488585598	4.57E-05	437	7	33
NM_001109	-0.6416093	-1.9646398	1.488585598	3.75E-05	436	7	25
NM_000543	-0.4236627	-1.8201414	2.082260476	0.002046064	521	7	661
NM_000543	-0.5982716	-2.0055432	2.082260476	0.000807878	519	7	536
AK094316	-0.4610895	-1.2852355	1.146899966	0.000834765	3626	7	484
NM_022468	-0.6809267	-2.5277436	1.778416955	9.29E-05	5358	7	895
NM_022468	-0.1530278	-1.9606088	1.778416955	0.000126282	817	7	185
CN642140	-0.5382115	-1.4888855	1.446090865	0.000187901	470	7	161
CN642140	-0.7280831	-1.2967872	1.446090865	0.000275836	660	7	397
XR_010922	-0.9093063	-1.5243648	2.024408889	0.000986471	545	7	902
NM_002163	-0.4147576	-1.456921	1.415971794	0.000921555	3843	7	215
NM_002163	-0.2007472	-1.2595929	1.415971794	0.001606573	3884	7	503
NM_014049	0.43294216	-1.6801709	1.269256946	0.002237009	566	7	637
NM_018946	-0.4768852	-1.7219791	1.574092468	2.88E-05	754	7	17
NM_018946	-0.588156	-1.8050923	1.574092468	0.000715856	524	7	130
NM_001007067	0.27182578	-2.1249342	1.973307801	0.00030435	821	7	452
NM_001007067	0.43321573	-1.9902383	1.973307801	0.000220093	822	7	540
NM_002406	-0.1307231	-2.0790503	1.368638326	3.32E-05	429	7	18
NM_002406	-0.3877173	-1.5342523	1.368638326	0.000413588	484	7	270
NM_021642	-3.3225709	-1.8256983	1.462925394	0.000104701	62	7	1227
NM_021642	0.29763069	-1.6105648	1.462925394	2.26E-05	5106	7	923
NM_006019	-0.6699499	-2.083016	1.31682552	1.52E-05	435	7	80
NM_006019	-0.564538	-1.6371426	1.31682552	3.49E-05	468	7	68
NM_002074	-0.4676644	-1.6714015	1.58105808	6.18E-05	485	7	78
NM_002074	-0.5257123	-1.5519695	1.58105808	5.48E-06	469	7	113
XR_012415	0.12225211	-1.7604693	1.595049986	1.84E-05	814	7	50
XR_012415	0.14604367	-1.4770498	1.595049986	7.65E-08	890	7	127
XR_011279	0.5092869	-1.5286015	1.224967715	0.00012076	615	7	645
XR_011279	-1.019287	-1.9501884	1.224967715	0.001361765	291	7	846
XR_010861	-0.7246467	-1.5291742	1.748225352	0.003022497	639	7	849
XR_010861	-0.8864695	-1.6714662	1.748225352	0.005246495	294	7	867
CN645791	-0.3193019	-1.8183162	1.331000811	0.000503768	467	7	212
CN645791	-0.1080619	-1.5486962	1.331000811	0.000244943	3998	7	116
CN645791	-0.1654801	-1.2843892	1.331000811	0.000181449	489	7	529
CN645791	0.03527099	-1.7996522	1.331000811	0.003733131	501	7	317
NM_002910	-0.6673142	-3.0068828	1.827267125	0.003652921	5424	7	1112
NM_002910	-0.4807562	-2.809514	1.827267125	0.002052624	5425	7	986
NM_005718	-0.2737263	-1.925179	1.550429042	0.001841409	491	7	816
NM_005718	0.02296603	-2.1610234	1.550429042	0.00034687	799	7	263

NM_002764	0.34531243	-1.4314626	1.150093188	0.002238937	618	7	745
XR_011552	-0.2282188	-1.4471852	1.523642693	7.34E-05	4000	7	100
XR_011552	-0.3456486	-1.769446	1.523642693	1.80E-07	758	7	4
NM_182565	-0.0422327	-1.363455	1.344887027	0.000492512	3783	7	384
NM_182565	0.14542346	-1.6458731	1.344887027	0.000343334	597	7	139
NM_001005360	-0.2304966	-1.2754114	1.341342688	0.00090758	3990	7	430
NM_001005360	-0.4510909	-1.4479745	1.341342688	0.000228771	3989	7	293
NM_032023	0.46023228	-2.0287489	1.256815211	0.000148373	600	7	392
NM_032023	0.56157713	-1.5547939	1.256815211	0.000175264	616	7	592
NM_012249	0.46094693	-1.2867978	1.490199343	0.003135304	630	7	788
NM_005554	-0.6832648	-3.3129196	1.099450061	0.032371626	119	7	1215
NM_012112	0.43773978	-1.2315042	1.515357047	0.000163302	940	7	774
NM_001664	-1.2095431	-1.848523	1.741609778	0.000326394	4688	7	757
NM_001664	-1.0309608	-1.6247584	1.741609778	8.72E-05	4689	7	517
NM_033280	-0.1792915	-1.812417	1.384529331	0.012219856	503	7	675
NM_033280	-0.6533542	-2.3666395	1.384529331	0.026047051	104	7	1156
CO647278	-0.0399436	-1.992469	1.698709002	1.60E-05	777	7	13
NM_005627	0.47206753	-1.2697243	1.439279183	0.000184224	628	7	664
NM_152237	-0.6238182	-2.4041622	1.466764362	0.000387647	432	7	402
NM_152237	-0.864796	-2.1331212	1.466764362	0.000376561	433	7	381
NM_005437	0.28745282	-1.5951818	1.638081907	0.000173054	827	7	310
NM_005437	0.17510066	-1.604599	1.638081907	5.06E-05	826	7	145
NM_152911	-0.242592	-1.8115066	1.998543519	0.000643025	819	7	364
NM_001552	-0.2351597	-3.0690585	1.636888994	0.000922154	5416	7	1022
NM_001552	-0.4333364	-2.5363504	1.636888994	0.001196828	5420	7	681
CN642598	-0.8300098	-1.1146134	1.348924832	0.000203423	3972	7	766
CN642598	-0.7834299	-1.2806087	1.348924832	0.002154472	656	7	702
A_01_P018128	-0.5855878	-1.7351232	1.789339618	3.15E-05	536	7	60
A_01_P018128	-0.1470153	-1.4088598	1.789339618	1.19E-05	903	7	152
NM_004060	-0.5740283	-1.996356	1.493799035	0.003309931	498	7	395
NM_018445	-0.1565949	-1.5339364	1.809546981	1.33E-05	798	7	84
NM_031465	-0.2227591	-2.031812	1.537647875	3.60E-06	481	7	249
NM_031465	-1.1901345	-1.697416	1.537647875	3.83E-05	514	7	590
CN644277	-0.1866022	-2.4785094	2.184133041	0.000119879	5597	7	652
CN644277	-0.8533197	-2.8314026	2.184133041	0.000675654	5360	7	1053
CN644277	0.07253261	-2.1480655	2.184133041	0.001019027	5654	7	797
NM_013258	0.23296245	-2.069947	1.64993779	2.32E-05	808	7	149
NM_013258	-0.1847941	-1.7077647	1.64993779	4.35E-05	767	7	19
XR_014059	-0.189085	-1.179337	1.36201154	0.001909483	679	7	462
NM_000201	-0.0148499	-2.4772263	1.11513387	0.000437145	423	7	505
NM_000201	-0.2663103	-1.9837813	1.11513387	0.00084896	462	7	237
NM_014175	-0.6617943	-1.2489102	1.534933177	0.00316741	657	7	703
NM_018465	0.46852329	-1.5986793	1.478581225	0.023410856	1002	7	1021

NM_018465	-0.7522476	-1.3145203	1.478581225	0.026229338	1703	7	979
NM_001223	-0.1414127	-1.3447246	1.520290501	0.000404995	707	7	203
NM_001223	-0.1382424	-1.7141648	1.520290501	0.003506296	551	7	354
NM_018947	-0.2683765	-2.2370401	1.510401604	0.003564341	500	7	472
NM_018947	-0.6307465	-1.4658135	1.510401604	0.001267216	661	7	314
NM_014390	-0.0543697	-1.402294	1.549168223	9.44E-06	4004	7	118
NM_014390	-0.1491895	-1.2299145	1.549168223	3.14E-05	4005	7	292
NM_013300	-0.3944455	-1.3196858	1.835855617	0.030133002	1110	7	1055
NM_003965	-0.3823746	-1.4789457	1.797143376	0.006450743	535	7	624
NM_003930	0.14611754	-1.2987337	1.168899341	0.002197279	650	7	539
NM_015161	0.21495819	-2.5324849	1.924361483	0.002069025	5595	7	899
NM_021822	-2.2511724	-3.5666771	1.67186975	0.021285495	11266	7	1231
NM_006378	-0.0880627	-1.7744318	1.581150923	1.76E-06	761	7	8
NM_006378	0.14827866	-1.9512113	1.581150923	0.000112081	778	7	49
XR_012351	0.45056243	-2.1376907	1.688535187	0.002259804	1011	7	768
XR_012351	0.21279135	-1.641797	1.688535187	0.000686982	828	7	377
NM_023933	-0.0596096	-1.5057291	1.20399638	0.000392556	3787	7	148
NM_023933	-0.0776515	-1.4323044	1.20399638	0.000532101	3789	7	319
NM_002029	-0.4333134	-1.3085461	1.409966921	0.000152394	4007	7	246
NM_002029	-0.3046618	-2.1741707	1.409966921	0.005455337	499	7	565
NM_004741	-0.3753633	-1.5265417	1.782286107	0.000450507	1191	7	250
NM_002105	-0.1703345	-1.3656336	1.77775592	6.39E-05	717	7	190
NM_005194	0.1266468	-1.8121603	1.739765377	6.91E-07	815	7	54
NM_005194	0.1787257	-1.5421702	1.739765377	6.59E-06	892	7	187
CB553994	-0.0569783	-2.267667	1.263330635	0.000325163	428	7	223
CB553994	-0.4654511	-2.6326919	1.263330635	0.002063745	327	7	750
NM_015344	-0.2896794	-2.2615547	1.048552849	0.034926078	98	7	1059
NM_015344	-0.9657506	-2.1693337	1.048552849	0.024517096	117	7	1175
NM_000188	-0.0559001	-1.5137072	1.48205501	2.83E-05	4002	7	67
NM_000188	-0.0894761	-1.461216	1.48205501	3.60E-05	4003	7	85
NM_006049	-0.1419255	-1.9371323	1.756318043	0.004448485	504	7	580
NM_018992	0.0427127	-1.6151415	1.659614773	5.19E-07	816	7	57
NM_018992	-0.3387215	-1.4578993	1.659614773	1.58E-05	637	7	457
NM_001416	-0.3464094	-1.2920189	1.601136991	1.36E-06	4014	7	164
NM_031937	-0.0999885	-1.5342891	1.243332096	0.010376583	3986	7	687
NM_194294	-0.4561502	-3.3903807	1.602343279	0.031736473	11271	7	1226
NM_194294	-0.0735809	-3.884751	1.602343279	0.000692691	5394	7	1192
CN648569	-2.1595917	-2.0254248	1.814199053	0.000728915	63	7	1220
CN648569	-0.5241711	-1.7674945	1.814199053	4.39E-05	850	7	89
NM_002835	-0.4143714	-1.2076336	1.598047436	0.033280203	130	7	1001
NM_024599	0.10544684	-2.3814256	2.052293529	4.94E-05	5599	7	574
NM_024599	-0.0754065	-2.8677008	2.052293529	0.00044688	5593	7	915
NM_007311	-0.2157092	-1.9963037	1.153096398	0.000425524	463	7	143

NM_007311	-0.4105253	-1.6628437	1.153096398	0.000829569	465	7	227
NM_007311	-0.1973418	-1.5614135	1.153096398	0.000656836	3786	7	176
NM_007311	-0.2356726	-2.0147707	1.153096398	0.001663636	418	7	192
NM_015441	0.13320552	-2.0679745	1.838856899	0.001914627	1122	7	577
NM_015441	1.00660019	-1.933483	1.838856899	0.001406074	5103	7	1183
NM_152653	0.03331824	-2.3810034	1.070937951	0.000637286	424	7	493
NM_152653	-0.1049733	-2.3080306	1.070937951	0.000763943	427	7	463
NM_003282	-0.0462065	-1.2891615	1.512686281	4.31E-05	4006	7	233
NM_033128	0.04963568	-1.7624368	1.70612116	5.00E-05	813	7	39
NM_033128	0.97436413	-3.6049193	1.70612116	0.0165686	87	7	1218
NM_025079	0.46417861	-1.3266018	1.646778633	4.56E-05	981	7	589
NM_020350	-0.0383405	-1.3660434	1.482306991	0.000360547	4001	7	260
NM_020350	0.31379031	-1.4061445	1.482306991	0.000101017	938	7	456
NM_018487	-0.9966164	-2.5920529	1.202454808	0.009230632	5397	7	1071
NM_018487	-0.9617596	-2.8077908	1.202454808	0.005249694	5396	7	1108
NM_004811	-0.4207661	-1.7303638	1.483558576	0.000266273	483	7	157
NM_004811	-0.2749254	-1.2896727	1.483558576	0.000180099	3994	7	313
CO646399	-0.6236034	-2.7127581	1.815809262	0.000111204	5415	7	714
CO646399	-0.6577849	-2.9627027	1.815809262	1.87E-06	5414	7	836
NM_213636	0.27844426	-1.5686142	1.346843985	0.000129732	598	7	181
NM_213636	-0.0817388	-2.1477339	1.346843985	0.004936565	420	7	556
NM_004553	-0.1316517	-1.5230733	2.076234779	0.002843973	1114	7	662
NM_024760	-0.3176459	-1.2547209	1.270291892	0.001741595	636	7	910
NM_018413	-0.7862814	-2.4004656	1.962802687	0.000215804	5355	7	976
NM_002488	-0.0700012	-1.5869313	1.622756673	0.00020692	3870	7	59
NM_004130	-0.1276803	-1.8005863	1.669321969	0.000253597	785	7	55
NM_004130	-0.2716541	-1.5916156	1.669321969	0.001148285	786	7	150
NM_000687	-0.0454106	-1.5425442	1.170624286	0.002515884	646	7	373
NM_000687	-0.1102934	-1.4285251	1.170624286	0.005094961	647	7	465
NM_016381	-0.337896	-2.5066567	1.739758585	2.87E-06	415	7	198
NM_016381	-0.5987884	-2.2279597	1.739758585	5.66E-05	444	7	186
NM_016307	-0.7797944	-2.6970064	1.169879536	0.002368097	5431	7	954
NM_016307	0.00123055	-1.7788084	1.169879536	0.000760978	3785	7	155
NM_032857	-1.4529528	-2.4154086	1.888808951	0.000338933	5375	7	1096
NM_032857	-0.3817473	-2.398657	1.888808951	0.000522689	494	7	552
NM_148962	0.20018913	-2.1574208	1.618621958	9.06E-05	807	7	254
NM_148962	0.79963889	-1.7230077	1.618621958	0.001284039	1162	7	938
NM_002560	-0.1778549	-1.964154	1.709936229	0.002136962	502	7	290
NM_030666	0.24918994	-2.7190589	1.18860605	0.000841788	5444	7	809
NM_030666	0.2245565	-2.6169869	1.18860605	0.001706832	5445	7	792
NM_001465	-0.2383048	-1.5751449	1.539533745	0.000825809	691	7	123
NM_001465	-0.0864798	-1.1919423	1.539533745	0.002199058	715	7	607
NM_006058	-1.915877	-2.2576623	1.291617514	0.00216386	17	7	1214

NM_006058	-0.4857979	-2.4421016	1.291617514	0.000932696	328	7	474
NM_000308	-0.2834352	-1.5637527	1.684506671	0.000369451	787	7	74
NM_005274	-0.2311749	-1.2693448	1.668041046	4.28E-06	4016	7	204
NM_014306	0.07758748	-1.5677148	1.62542851	0.000847774	694	7	224
NM_014306	0.00957674	-1.3528193	1.62542851	0.000452948	718	7	284
XR_011110	0.0103361	-1.3165112	1.687784197	0.000456241	720	7	307
NM_016530	0.45322906	-2.0389449	2.137539354	0.001653563	5657	7	943
NM_003015	-0.0419669	-1.3755057	1.153988408	0.005503834	649	7	513
NM_001124	-0.3512962	-1.9306284	2.005520739	0.000914398	5623	7	338
NM_001124	0.1711115	-1.772942	2.005520739	0.001018782	824	7	481
NM_018022	-0.256558	-2.515517	1.471480769	4.61E-05	413	7	195
NM_018022	-0.610991	-2.2163971	1.471480769	5.27E-05	434	7	309
NM_181054	-0.0469526	-1.5633232	1.759421747	0.002140654	695	7	333
NM_005170	0.33121101	-2.0695302	1.729403158	5.20E-05	809	7	301
NM_005170	-0.39473	-1.8145534	1.729403158	0.000100396	755	7	26
NM_000569	-0.2726707	-1.880465	2.12618675	0.000272307	5625	7	321
NM_000569	-0.1097427	-1.8029469	2.12618675	0.00048187	5624	7	389
XR_011741	0.18211811	-3.0230123	1.865341669	0.002403113	5426	7	1107
XR_011741	-0.076172	-1.9870444	1.865341669	0.001810984	1017	7	442
NM_015589	-0.4272003	-2.9400772	1.466210988	0.006652228	5409	7	1089
NM_015589	2.21284319	-3.684847	1.466210988	0.000647404	11277	7	1229
CO581027	-0.5676309	-1.6805482	2.206339269	0.002577783	1124	7	825
CO648815	0.1412976	-1.410045	1.759130297	7.28E-06	893	7	265
CO648815	0.31734831	-1.970143	1.759130297	5.76E-05	1012	7	417
NM_181512	0.16505928	-1.717569	1.898672424	0.005855144	1021	7	765
NM_005219	-0.2139184	-1.2913336	1.188867775	0.006972547	3991	7	715
NM_006573	0.70091052	-2.3965902	2.005975369	1.79E-05	5615	7	873
NM_006573	0.07117827	-2.2134979	2.005975369	2.33E-05	818	7	374
CN647492	-1.3314507	-2.5677133	1.360913988	0.002074605	5400	7	1054
CN647492	-1.263326	-2.1609558	1.360913988	0.000865729	5402	7	871
NM_003330	-0.5908471	-2.7830373	1.767132133	0.000164825	5357	7	911
NM_003330	-0.2643279	-2.3144898	1.767132133	2.13E-05	416	7	81
NM_006164	-0.7228495	-1.5132779	1.894079011	0.000174602	537	7	349
NM_006164	-0.3222296	-1.4467903	1.894079011	0.000340942	696	7	343
NM_019111	-0.21853	-1.7525358	1.9174185	1.87E-05	793	7	46
NM_024496	-0.8925597	-1.7145019	1.870638463	0.007870445	5368	7	1004
NM_001243	0.61200854	-1.6832382	1.310780803	0.001668253	617	7	775
NM_001243	0.00772125	-1.1640651	1.310780803	0.006142318	4043	7	747
NM_006260	0.40350594	-1.7684077	1.475514251	0.000180017	601	7	363
NM_006260	-0.0057219	-1.6016593	1.475514251	0.000853153	594	7	196
NM_002638	0.95078694	-1.5749803	0.895324733	0.008192442	5086	7	1159
NM_004079	-0.6131742	-1.6784559	1.396737983	0.001780345	3931	7	302
NM_004079	-0.2953016	-1.6340106	1.396737983	0.002012758	1026	7	327

NM_004079	-0.0664368	-1.359103	1.396737983	0.002996801	741	7	421
NM_004079	-0.0327672	-1.1256762	1.396737983	0.003724874	742	7	643
NM_004530	-0.9757847	-2.4236274	1.438186137	0.000431199	5401	7	805
NM_004530	-0.5659439	-1.7197951	1.438186137	0.001317124	460	7	258
NM_001747	-0.1906045	-1.6877581	1.947140483	0.000411665	847	7	294
CN801572	0.29359953	-1.532732	1.479188141	0.000326061	599	7	340
CN801572	0.37937882	-1.1830395	1.479188141	0.000348531	622	7	770
NM_177424	-0.022011	-1.5817208	1.884492766	0.000159547	543	7	217
NM_177424	-0.5992728	-1.3890302	1.884492766	0.000366161	541	7	486
NM_001772	-0.9688043	-1.7122438	2.416646313	0.007591366	5509	7	1090
XR_010040	-0.8731402	-1.3427844	1.781791211	0.00011217	4766	7	548
XR_010040	-0.8428499	-1.5565818	1.781791211	0.000180567	527	7	359
NM_004385	-0.5117917	-1.871841	2.039267663	5.22E-05	851	7	225
NM_004385	-0.3710281	-2.0997819	2.039267663	2.07E-05	5620	7	158
NM_000801	-1.1871345	-1.6313185	1.688086618	0.034725517	5352	7	1143
NM_000801	-1.2275587	-1.6985359	1.688086618	0.004301283	5351	7	981
NM_000801	-1.3359973	-1.7846128	1.688086618	0.002856489	5350	7	1005
NM_000801	-0.7268757	-1.3873752	1.688086618	0.009169231	3938	7	875
NM_001001790	0.22656037	-1.5563655	2.032423434	1.60E-05	900	7	433
NM_001001790	-0.1501665	-2.0848227	2.032423434	0.006360383	510	7	949
NM_018235	0.08950473	-1.5558983	1.917621583	0.000103497	924	7	255
NM_018235	0.30154767	-1.4583905	1.917621583	4.28E-05	925	7	432
NM_000110	0.08174917	-2.356516	1.41633613	0.000122061	426	7	238
NM_000110	-0.217128	-1.9706374	1.41633613	0.00072428	419	7	92
NM_000609	-1.2401164	-1.9953469	1.542532755	0.000155753	5376	7	1105
NM_000609	0.00858225	-1.5510787	1.542532755	0.00031664	692	7	109
NM_006830	-0.055133	-1.6416771	1.336702523	0.001612534	648	7	171
NM_006830	-0.0953224	-1.2696918	1.336702523	0.001101341	3997	7	356
NM_001037	-0.3534273	-2.4175644	1.640853249	0.001681758	5421	7	614
NM_001037	-0.2656426	-2.3805699	1.640853249	4.00E-05	414	7	136
NM_024789	-0.4790317	-1.2649339	1.426770533	0.003296238	3983	7	575
NM_024789	-0.5123751	-1.5529003	1.426770533	0.004477981	4694	7	520
NM_005099	2.35076773	-1.9246214	2.31750334	0.002255568	5270	7	1163
NM_024310	-0.2041374	-1.4261734	1.172486408	0.010814596	1027	7	694
NM_024310	-0.7346627	-1.9545619	1.172486408	0.013338174	1701	7	826
NM_004494	-1.0055778	-1.8017447	1.707460227	0.000636542	4692	7	692
NM_004494	-0.3285584	-1.5331877	1.707460227	3.08E-05	838	7	141
NM_178232	0.16862895	-1.9242296	2.437889614	0.000328165	5661	7	833
NM_001919	-0.3786068	-2.4755474	1.797850387	0.000985467	5422	7	659
NM_001919	-0.6179649	-2.0674575	1.797850387	6.38E-05	445	7	170
NM_015703	0.24152774	-1.7587802	1.677526563	0.000220005	888	7	382
NM_015703	0.36020778	-1.4909389	1.677526563	0.000175965	937	7	475
NM_001687	-1.066543	-2.6817013	1.739422376	0.000773815	5406	7	1006

NM_001687	-1.2547509	-2.6709315	1.739422376	0.000227943	5405	7	1037
NM_004335	0.41064539	-2.6337841	1.998138663	1.64E-06	5613	7	784
NM_004335	0.38197036	-2.3897712	1.998138663	1.99E-06	5614	7	608
NM_001734	-0.0955317	-1.5029202	1.930918372	1.57E-06	904	7	147
NM_001734	-0.083864	-1.4576469	1.930918372	4.82E-06	905	7	193
NM_005211	-0.3157998	-2.0670572	1.643109401	6.44E-05	456	7	115
NM_005211	0.01195009	-2.0186541	1.643109401	3.35E-05	769	7	38
DR771539	-0.4715318	-2.403596	1.988195488	2.72E-05	5573	7	387
DR771539	-0.3379457	-1.9538081	1.988195488	2.14E-05	852	7	125
XR_010082	-0.2774966	-2.195637	1.796577711	3.63E-05	457	7	105
XR_010082	-0.0101677	-2.2779725	1.796577711	7.47E-07	768	7	93
NM_032965	0.69997192	-1.3324168	1.569120378	0.013540232	1166	7	1047
NM_032965	0.52595462	-1.2512826	1.569120378	0.005139685	1165	7	920
NM_144589	-1.3487872	-1.4111714	1.184198438	0.002419643	1378	7	1117
NM_144589	-0.6712053	-1.6168497	1.184198438	0.008937786	198	7	882
XM_093895	0.37621136	-2.4452327	1.962393062	0.000939375	509	7	905
NM_138720	-0.288402	-2.2312442	1.986717218	2.69E-05	5619	7	376
NM_138720	-0.1015992	-2.5255158	1.986717218	0.001246392	5594	7	741
NM_145058	-0.2423399	-2.0371955	1.711129195	5.32E-05	771	7	37
NM_145058	-0.3928428	-2.0185134	1.711129195	1.69E-05	770	7	24
NM_000063	-0.3345381	-1.2511864	1.65021381	8.42E-05	4013	7	276
NM_032463	0.23466424	-1.7787777	1.945587525	2.21E-05	921	7	264
NM_032463	0.37123124	-1.5671344	1.945587525	6.06E-06	927	7	477
NM_006002	-0.6034542	-1.4279777	1.834851393	0.002050289	4704	7	665
AW294925	-1.0589686	-1.2637827	1.576582723	0.035659877	182	7	1147
NM_203434	-0.040567	-2.3906737	1.717358619	0.000110349	5607	7	386
NM_203434	0.22588715	-2.3014878	1.717358619	5.92E-05	5609	7	323
NM_000175	-0.3518619	-1.3409737	1.628289073	0.000707656	4009	7	337
NM_000175	-0.5033497	-1.2924099	1.628289073	0.000172594	4008	7	320
NM_000169	0.09840228	-1.992708	2.174709639	9.81E-06	5644	7	369
NM_000169	0.3106259	-1.6401275	2.174709639	3.40E-05	932	7	593
NM_002306	-0.4147725	-1.7962837	1.867584101	5.85E-07	789	7	28
NM_002306	-0.2796876	-1.6843073	1.867584101	2.78E-05	790	7	42
NM_002491	-0.3967515	-1.2269003	1.652376925	0.003066993	655	7	767
NM_001860	-0.0994964	-1.7334264	2.106926374	0.005212377	1113	7	824
NM_001645	0.08977757	-1.7376942	1.988911694	1.27E-05	898	7	222
NM_001645	0.17330251	-1.6239298	1.988911694	1.85E-05	899	7	326
NM_004310	0.37640604	-1.9803444	2.125391853	8.11E-05	1016	7	927
NM_002755	0.43906936	-1.5607618	1.815815968	0.000174838	901	7	426
NM_006755	-0.4338055	-1.8723011	1.486982276	0.000566352	438	7	124
NM_006755	-0.3455782	-2.2123881	1.486982276	0.001347425	495	7	269
NM_001012754	-0.8353197	-1.9371555	1.774406542	0.000366295	523	7	360
CO725743	-0.3277307	-2.2462362	2.366004296	4.53E-05	5628	7	610

CO725743	-0.2391563	-2.0928478	2.366004296	5.51E-05	5629	7	563
NM_001785	-0.32404	-2.1474145	1.69695823	0.000548644	5423	7	500
NM_001785	-0.1156179	-1.7116001	1.69695823	0.000584875	844	7	178
NM_015900	-0.5946685	-1.905607	2.360488948	2.39E-05	5479	7	753
NM_015900	-0.1491629	-2.2123376	2.360488948	0.000178858	5632	7	718
NM_005461	-0.4042636	-1.9691573	2.088525979	0.000345502	520	7	416
NM_005461	-0.231118	-1.793102	2.088525979	0.00059864	542	7	444
NM_003547	-0.0514896	-2.4252837	2.202659194	4.46E-05	5603	7	599
NM_000100	-0.2664244	-1.7846724	2.001198766	7.28E-08	791	7	75
NM_000100	-0.204147	-1.801343	2.001198766	1.26E-06	792	7	77
NM_002356	0.39703395	-1.8761274	2.057425028	1.94E-05	922	7	489
NM_002356	0.33356834	-1.6227742	2.057425028	0.000125398	926	7	521
NM_001003962	-0.4253192	-1.3150849	1.425228801	0.019421224	3984	7	898
NM_001003962	-0.565326	-1.1293973	1.425228801	0.012868685	3985	7	885
NM_005195	-1.0175136	-1.8106718	2.01311224	4.39E-05	525	7	598
NM_005195	-0.9362267	-1.8503836	2.01311224	0.000155477	526	7	531
NM_001004431	-0.1402131	-2.1800583	2.210971575	0.000477794	5658	7	653
NM_001004431	-0.0548939	-2.2427043	2.210971575	0.000342885	5659	7	688
XM_376876	-0.4753703	-1.501125	2.031679574	0.000193326	539	7	362
XM_376876	-0.5552204	-1.5498728	2.031679574	0.000305181	538	7	390
NM_005949	0.29247303	-2.7215642	2.135305761	0.000264072	5606	7	940
NM_005949	0.06030855	-2.7380906	2.135305761	0.000188648	5605	7	879
NM_013370	-0.3961199	-1.6701751	1.741081919	0.000930624	841	7	283
NM_013370	-0.2768674	-1.5137506	1.741081919	0.000137357	839	7	257
NM_006834	0.13485524	-1.7896781	1.778604806	9.53E-05	829	7	128
NM_006834	0.26183392	-1.4631738	1.778604806	3.90E-05	889	7	408
NM_152858	0.37943438	-1.6842967	1.789039737	0.000108717	1013	7	507
NM_152858	0.35864239	-1.6537812	1.789039737	0.000293875	831	7	499
NM_001953	0.52515282	-1.6440821	2.002557036	0.00508041	1163	7	989
NM_001953	0.71121835	-1.6736014	2.002557036	0.003110447	1164	7	1007
NM_013324	-0.9157468	-1.386122	1.824825061	0.000943906	5371	7	1109
NM_014395	0.05766074	-2.0210579	2.231677606	0.003541938	1018	7	894
NM_004369	-0.2377041	-1.7306216	1.947607882	1.27E-05	794	7	88
NM_004369	-0.0986504	-1.7129556	1.947607882	3.04E-05	796	7	110
NM_018434	-0.1361079	-2.8058732	1.428662524	0.00035535	5443	7	704
NM_018434	-0.5316075	-2.8767408	1.428662524	0.013742605	105	7	1122
XR_010412	-0.02801	-1.5888513	1.882028703	0.001563183	1029	7	629
XR_010412	1.15240355	-1.6711316	1.882028703	0.002775053	1004	7	870
NM_016573	0.04371855	-2.1373829	1.176385753	0.01888717	128	7	984
NM_199418	0.00318165	-2.6820485	1.916771734	0.001327442	506	7	863
NM_199418	0.00448922	-2.1536323	1.916771734	0.000144771	5621	7	209
NM_001165	0.11889741	-2.1544239	2.214451437	0.000143101	5642	7	578
NM_001165	0.21419301	-2.0072307	2.214451437	6.35E-05	5645	7	560

NM_198232	-0.2352099	-1.7796597	1.86804729	6.38E-05	842	7	107
NM_198232	-0.3838015	-1.454701	1.86804729	0.000200683	840	7	304
NM_007207	-0.5232843	-1.5015811	1.058732752	0.022466385	1366	7	934
NM_015419	-0.2409976	-3.177659	1.142228137	0.005769844	5458	7	1128
NM_015419	-0.3365058	-3.6975986	1.142228137	0.00754919	79	7	1201
NM_001547	-0.7244048	-2.6502282	2.025341782	0.004573744	106	7	1061
NM_001547	0.10819477	-1.7065104	2.025341782	0.000230747	923	7	334
NM_024096	-0.7975677	-1.7255442	1.821395391	0.000984938	4693	7	549
NM_024096	-0.5824103	-1.7998805	1.821395391	0.000629204	441	7	273
NM_201612	-0.8493781	-1.4682525	1.892940173	0.004707061	5475	7	1077
NM_024027	0.05063386	-1.6146267	2.142631414	0.000117869	931	7	443
NM_005615	-0.6751035	-2.3057891	2.067226339	0.001012524	5565	7	783
NM_005615	-1.1449014	-2.00278	2.067226339	0.002389686	5476	7	1008
NM_002629	-1.2699545	-2.2152508	2.411164567	6.04E-05	5557	7	1031
NM_002629	-1.1766291	-2.1284522	2.411164567	1.74E-05	5558	7	963
CO645773	-0.343827	-3.3164174	1.658452717	0.000137631	5411	7	1046
CO645773	-0.7820695	-2.5137126	1.658452717	0.000103918	430	7	506
NM_003541	-0.2160295	-2.9573428	2.119668791	5.20E-06	5537	7	886
NM_003541	-0.2162674	-2.296184	2.119668791	1.29E-05	5574	7	372
NM_003541	-0.30289	-2.8300154	2.119668791	6.83E-05	5535	7	843
NM_003541	-0.7106951	-2.3545946	2.119668791	5.48E-08	443	7	561
XR_011133	-0.0740762	-1.2900083	1.604472829	0.001776582	4023	7	533
XR_011133	-0.2944177	-1.710288	1.604472829	0.000479243	439	7	97
NM_005726	-0.4566577	-2.4673264	2.292488777	0.000129989	5587	7	891
NM_005726	-0.526788	-2.1279089	2.292488777	0.000133256	5617	7	631
NM_000491	-0.6509516	-2.982758	2.02965279	0.00041983	5413	7	1024
CB549220	-0.0823324	-1.827182	2.061162779	3.69E-06	854	7	191
CB549220	-0.0979442	-1.834885	2.061162779	1.72E-05	795	7	154
NM_001005386	-0.0262303	-2.1092552	1.762491678	0.006227623	507	7	736
NM_001005386	-0.3888732	-1.5577696	1.762491678	0.009357179	505	7	720
NM_006353	-1.2873113	-1.9941272	2.366739291	0.036309264	5859	7	1203
NM_005561	0.49237342	-1.9494239	1.770528905	0.003234446	1003	7	811
NM_002661	-0.0479654	-1.3717075	1.603624199	0.000569786	4019	7	275
NM_002661	-0.3245783	-1.61871	1.603624199	0.002583948	690	7	311
NM_001289	0.33574813	-2.1997804	2.412153586	1.10E-05	5643	7	837
NM_001289	0.04981946	-1.9506869	2.412153586	3.57E-06	5646	7	674
NM_000418	-0.1746287	-1.9553201	1.939859605	1.28E-05	772	7	96
NM_000418	-0.1646647	-1.8692766	1.939859605	1.37E-05	773	7	86
NM_005874	-0.116188	-1.5611392	1.826053127	0.001471819	845	7	511
NM_005874	-0.1791079	-1.596781	1.826053127	0.000232076	843	7	184
NM_002598	-0.219578	-1.2882987	1.676533122	0.00036752	4018	7	303
NM_002598	-0.3628787	-1.3312368	1.676533122	0.000517937	4017	7	280
NM_004972	-0.5544541	-2.137389	2.071928709	0.001658076	5506	7	696

NM_004972	-0.1160961	-1.9757592	2.071928709	0.000385701	5622	7	410
NM_153811	-0.4523484	-1.8485343	1.976292673	0.000856491	5507	7	405
NM_004844	-0.0041027	-1.6298371	2.045386513	3.43E-05	933	7	448
NM_032331	-0.1516095	-1.20681	1.563743354	0.004543692	734	7	611
NM_004074	-0.4623192	-2.1491615	2.26417137	7.70E-05	5583	7	572
NM_004074	-0.6738282	-2.1329409	2.26417137	3.38E-06	5581	7	584
NM_152713	-0.0025146	-1.6035305	1.644836489	0.001649558	693	7	267
CN645487	-0.0357813	-1.816234	2.086561061	0.0002196	861	7	512
NM_000747	-0.7769382	-2.2390575	1.560333945	0.017378288	109	7	1019
NM_001693	-0.5634955	-2.0956166	2.303725384	0.000297623	5618	7	682
NM_002436	-0.1740662	-1.9738318	2.290468583	9.28E-06	857	7	466
NM_002436	0.02984546	-1.8354159	2.290468583	1.54E-05	858	7	502
NM_005564	-0.2163545	-1.809103	2.369376338	0.001413243	864	7	889
NM_002112	0.10240914	-3.0218877	1.854081371	0.003473372	5427	7	1110
NM_002112	0.30416587	-3.1048947	1.854081371	0.001397119	5453	7	1087
NM_001558	-0.1232803	-1.7126979	2.322005051	5.06E-06	856	7	501
NM_007315	-0.2512235	-1.8835419	2.143279024	0.000143174	5477	7	1056
NM_007315	0.33458821	-1.7691714	2.143279024	0.000184879	1006	7	657
NM_000597	-0.5562618	-1.6027481	1.122747806	0.016345447	1365	7	957
NM_000597	0.32400149	-1.9218648	1.122747806	0.007104753	1364	7	830
NM_002125	-0.2209826	-1.8577384	2.544378055	9.59E-05	860	7	878
NM_002125	-0.6700002	-2.0007997	2.544378055	2.40E-05	5480	7	1045
NM_016350	-0.8526231	-2.1922455	1.879051267	0.009894912	5504	7	1026
NM_005693	-0.1411141	-3.0003748	1.857781713	3.34E-05	5449	7	913
NM_005693	-0.3074622	-2.7482029	1.857781713	4.06E-05	5534	7	605
NM_001497	-0.0202733	-2.2028527	2.316986109	0.000141148	5634	7	626
NM_001497	-0.1024162	-2.2441534	2.316986109	0.000229492	5633	7	656
XR_013712	-0.4630687	-2.3907841	2.297151681	9.57E-05	5575	7	733
NM_002115	-0.3036531	-2.3045131	1.434346558	0.001760384	422	7	453
NM_002115	-0.0664532	-2.3974883	1.434346558	0.000839208	425	7	383
NM_000591	-0.2062714	-2.5475758	2.255046293	3.52E-06	5570	7	672
NM_015892	0.11820699	-1.8778327	2.25865923	0.000278648	5656	7	854
NM_001398	-0.6605967	-2.2745304	1.891158595	0.000207799	431	7	468
NM_001398	-0.7740803	-2.0711386	1.891158595	0.000174135	440	7	361
NM_012264	-0.5559339	-1.8540577	1.954972967	0.001140448	531	7	476
NM_012264	-0.6249402	-1.5208004	1.954972967	0.003053727	533	7	684
XR_012031	-1.4144275	-1.9333006	1.868883546	0.00619292	22	7	1219
XR_012031	-0.1906015	-2.0439154	1.868883546	0.015749268	5505	7	997
NM_080792	0.05489692	-1.1591793	1.463782343	0.004056985	4024	7	760
NM_080792	-3.7626743	-1.246354	1.463782343	0.004128117	4	7	1236
NM_001012456	0.75881652	-1.9958637	2.356191899	7.39E-06	5666	7	977
NM_005721	-0.6861456	-2.6342177	1.832777827	0.000693715	5359	7	893
NM_005721	-0.3039411	-2.1534581	1.832777827	0.000871361	497	7	478

NM_002631	-0.2858172	-2.1811945	2.451336715	1.67E-06	5627	7	649
NM_003764	-0.9971067	-2.1425106	2.117620961	1.06E-05	5556	7	708
NM_003764	-0.5315992	-2.0905281	2.117620961	0.000500654	5616	7	554
NM_017817	-1.0288123	-3.381384	1.920300106	9.08E-05	5432	7	1176
NM_017817	-1.3566532	-2.9927673	1.920300106	3.36E-05	5403	7	1118
NM_021970	-0.0736567	-2.1854777	2.003293503	0.004188623	508	7	892
NM_000101	-0.4696046	-2.0347142	2.415986181	3.61E-05	5585	7	673
NM_000101	-0.4135913	-1.9207698	2.415986181	1.67E-05	5586	7	615
NM_003946	-0.1579574	-1.4145429	1.195982845	0.019692166	644	7	785
NM_052871	0.20390679	-2.5311664	1.935097398	0.000103032	5608	7	669
NM_018845	-0.4378092	-1.4348739	1.269220891	0.013809332	4827	7	781
NM_018845	-0.1474156	-1.222301	1.269220891	0.014046192	4828	7	794
NM_152322	0.28563906	-2.1082254	2.30210361	0.000123228	5660	7	772
NM_152322	0.29224974	-2.5406989	2.30210361	3.93E-05	1015	7	1009
NM_005754	0.01959169	-1.7352005	1.931540841	0.000240681	830	7	266
NM_005313	-0.6167348	-3.1692973	1.273552512	0.003607246	5430	7	1106
NM_005313	-0.0582519	-2.3814795	1.273552512	0.004612144	421	7	751
NM_198282	-1.0455467	-2.7937353	1.655393231	0.006998847	5404	7	1132
NM_198282	-0.7200907	-3.1787079	1.655393231	0.001894708	5395	7	1138
NM_002801	-0.1411417	-2.7487232	2.134404117	1.09E-05	5538	7	764
NM_002801	-0.1933166	-2.7446422	2.134404117	0.000231864	5536	7	822
NM_004995	-0.6510896	-2.186441	1.67819072	0.001543507	1023	7	707
NM_004995	1.26154465	-1.5132566	1.67819072	0.002009906	1005	7	862
NM_000930	-0.9022147	-1.8051935	1.903155532	0.000246724	5377	7	859
NM_000930	-0.2154314	-1.5001857	1.903155532	0.000702016	1037	7	353
NM_001428	-0.2007223	-2.0276764	2.449188359	2.92E-06	5631	7	601
NM_001428	-0.2214115	-2.0929956	2.449188359	4.89E-06	5630	7	627
CO648453	0.10124194	-2.2055262	2.459941762	2.88E-06	5637	7	763
NM_016511	-0.1059348	-2.2108819	2.220111198	6.80E-05	5589	7	612
NM_016511	0.2603499	-2.2740431	2.220111198	5.95E-05	5610	7	713
U46661	-0.0275257	-2.9618617	1.067827714	0.009752976	5459	7	1116
U46661	-0.0351621	-2.9176384	1.067827714	0.005610422	5460	7	1052
CK230160	-1.8692517	-2.0272143	1.628155068	0.005789141	18	7	1207
CK230160	-0.5204577	-1.777567	1.628155068	0.001892795	1024	7	668
NM_001078	0.03826907	-1.8939505	2.332908721	0.003484681	1019	7	945
NM_015878	-0.0915514	-1.5079103	1.9295265	0.000790605	1044	7	434
NM_145725	-0.1049609	-2.296165	2.45817106	0.00621201	5348	7	1100
NM_016209	-0.2214462	-1.7178192	2.09712322	0.000210813	862	7	393
AK057584	-0.3462362	-1.376867	1.522497018	0.01909309	652	7	840
NM_031453	-0.2033076	-1.620429	1.716235278	0.008587718	653	7	670
NM_001975	-0.5192974	-2.4104475	2.306654511	0.000931851	5566	7	928
CN642065	-0.0618632	-1.4744335	1.337702709	0.016366254	1028	7	865
NM_017583	-0.4903364	-1.1409078	1.457891242	0.020420712	729	7	983

NM_002467	-0.2886805	-1.3192488	1.651265912	0.006636864	732	7	677
NM_002881	-0.8466622	-1.6017174	1.861208095	0.002803218	529	7	801
NM_005565	-0.3515803	-2.1749134	1.830891407	0.001973581	496	7	494
NM_005565	-0.4394449	-1.610597	1.830891407	0.002350918	532	7	458
NM_000593	0.19711232	-1.8885909	2.426719224	2.12E-05	5652	7	727
NM_000593	-0.0032068	-1.837994	2.426719224	4.32E-05	5651	7	644
NM_022555	-0.5862425	-2.1093082	2.441930794	9.90E-05	5582	7	812
NM_022555	-0.5485204	-2.1097916	2.441930794	2.27E-05	5584	7	728
NM_020169	-0.8600522	-1.4053785	1.890747411	0.001209444	1035	7	726
NM_054012	-0.0494422	-2.6597361	1.895689474	0.000609434	5450	7	990
NM_054012	1.04365356	-2.6120183	1.895689474	0.000363311	5102	7	1182
XR_010680	-0.0164937	-1.4856931	1.790098237	0.004690427	1030	7	769
XR_010680	-0.7676654	-2.5138133	1.790098237	0.007458736	108	7	1039
NM_003768	-1.5207528	-1.7090639	1.992752609	0.003242462	5378	7	1197
NM_003768	-1.2260197	-1.6548161	1.992752609	0.006453133	5385	7	1027
NM_001305	0.6840264	-1.7127684	2.040035493	0.04701346	5923	7	1188
NM_022170	-1.1156512	-1.1496245	1.437069367	0.036371998	5384	7	1088
NM_021983	-0.7153019	-1.9547896	2.530745111	3.23E-05	5578	7	851
NM_021983	-0.8188998	-2.0556016	2.530745111	1.16E-05	5577	7	874
NM_000934	-0.4845437	-2.4717418	2.310396426	0.000356066	5544	7	890
DV770912	-0.5322913	-1.67044	2.017579719	0.00213727	1032	7	701
NM_002104	-0.366045	-1.9645646	1.931792689	0.001943341	1058	7	522
NM_001572	-0.4218853	-2.1699649	2.334317258	0.000289493	5576	7	752
NM_001572	-0.0930802	-2.0437494	2.334317258	0.00014824	5648	7	609
NM_018326	0.09108242	-1.7525039	2.302135332	0.000455669	1067	7	806
NM_018326	0.3325991	-2.0481348	2.302135332	0.000298837	5649	7	807
NM_001003927	0.38118569	-2.420755	2.378808674	0.009431492	5856	7	1155
NM_012331	-0.0865851	-2.2138547	2.113849321	0.00077786	5647	7	604
NM_012331	0.26478262	-2.483464	2.113849321	0.001387352	5542	7	941
NM_000575	0.63584603	-1.970084	2.0309222	0.009026252	5104	7	1131
NM_005161	0.300142	-1.7406585	1.92452245	0.005174038	1063	7	881
NM_005161	0.0820051	-2.3673566	1.92452245	0.001491594	1057	7	743
NM_020370	0.12163605	-1.8548632	1.704567053	0.00667761	1061	7	749
NM_020370	-0.1399966	-1.858872	1.704567053	0.006572544	1060	7	695
NM_006627	-0.1740937	-2.115903	2.392410228	0.000748033	1059	7	842
NM_000310	-0.9815273	-2.00151	2.453324295	0.001548902	5382	7	1029
NM_024656	-0.1959735	-3.0478601	1.534348577	0.018756255	122	7	1180
CB229722	-0.2867521	-1.6690768	2.07849476	0.005122951	1034	7	793
NM_014918	0.13935266	-1.8416631	2.495871566	0.0003291	5650	7	883
A_01_P010985	-1.8862825	-2.5795985	1.069976993	0.031193191	11357	7	1213
NM_000595	0.81892982	-3.1397322	1.592593183	0.015463545	5465	7	1204
NM_000595	0.38620794	-2.6459276	1.592593183	0.00998621	5461	7	1101
NM_004567	0.64017885	-2.0861105	1.320396917	0.039097439	5921	7	1186

CN647493	-1.3711166	-2.3815145	2.350175779	0.00307348	23	7	1232
NM_021075	-0.8613024	-1.8865835	2.34813726	0.002564378	1031	7	1012
NM_014445	-0.2887465	-1.8466882	2.055723709	0.006332527	1062	7	901
NM_004729	0.38774713	-2.0195098	2.31287753	0.002184675	1070	7	994
NM_003355	-0.2207761	-2.7963023	1.739991385	0.014549365	5464	7	1150
NM_003355	-0.9780751	-2.5658833	1.739991385	0.018928134	5462	7	1162
NM_032192	1.01239761	-1.7992657	1.756876088	0.049292624	5922	7	1194
NM_002265	-1.0359072	-2.6112537	1.904997071	0.012935286	107	7	1178
NM_002265	-0.9933136	-1.5446777	1.904997071	0.016610652	5386	7	1074
NM_006140	-0.0180775	-2.0205091	1.956920025	0.007512356	1064	7	922
NM_006140	0.281717	-2.2378739	1.956920025	0.005579772	1065	7	975
NM_015545	-0.3841492	-2.713128	1.624602124	0.03185115	5466	7	1181
NM_015545	-1.1500007	-1.6484167	1.624602124	0.029663698	5383	7	1113
NM_004946	-0.2111838	-1.566465	2.045558034	0.009338987	1052	7	925
NM_004946	-0.0855905	-1.5907044	2.045558034	0.010748392	1054	7	980
NM_003608	-0.0459401	-2.8180147	1.93022699	0.005042767	5467	7	1073
NM_003608	0.34321179	-2.4764434	1.93022699	0.009778578	5468	7	1081
NM_022059	-0.0606678	-3.1754754	1.912304562	0.011305644	5463	7	1185
NM_022059	0.31005661	-1.9077286	1.912304562	0.01232351	1066	7	1035
NM_014002	-0.3321938	-1.479538	1.815337069	0.038474508	1050	7	1082
NM_003449	-0.2866157	-1.5421179	1.965004623	0.02110779	1051	7	1036
CO648296	-0.1045902	-2.5643325	1.302179433	0.045381441	5457	7	1179
CO648296	-0.329967	-2.9488641	1.302179433	0.033018881	5456	7	1189
NM_024648	0.02144104	-1.707421	2.002668577	0.025350342	5885	7	1125

Cluster 6

Gene Name	Av Normal	Av M (w12)	Av M (w4)	*P*	Hierarchical Clustering (order)	K-means clustering	K-means clustering (rank)
XM_375712	0.38579764	-1.3814609	1.880510925	0.038049199	1155	6	748
NM_014050	0.30587572	-0.640535	1.75624154	0.045822938	1269	6	601
NM_138636	-0.3444684	-1.4357309	2.034789629	0.025768526	1276	6	688
NM_003740	-0.3684674	-0.8665591	1.779720114	0.032180247	1230	6	548
NM_139265	-0.3290389	-1.2920402	3.294911076	0.048313624	5250	6	857
NM_139265	-1.8010079	-1.2662863	3.294911076	0.048673382	11326	6	871
NM_001516	-1.3038675	-0.6706767	1.776183435	0.024521054	71	6	779
NM_004887	0.45233128	-1.0770689	1.708268354	0.034747478	1158	6	684
NM_007199	0.32792817	-1.2093009	1.957923955	0.040463158	1171	6	747
NM_001205	-0.12932	-1.1265186	2.519256901	0.035916113	1279	6	745
NM_001205	-0.029895	-1.1740718	2.519256901	0.033590463	1278	6	740
XR_011471	0.12585072	-1.3048579	2.014252787	0.018895648	1272	6	646
NM_012329	0.39519939	-0.7181353	1.842628291	0.044048762	1270	6	694
NM_001006616	0.11686971	-1.1139271	1.577166266	0.012686225	1188	6	489
NM_001006616	0.01075597	-0.852366	1.577166266	0.014010902	1235	6	427
NM_018197	-0.0124601	-1.1679268	1.549394535	0.02307819	4650	6	610

NM_172341	-0.38491	-1.7128348	2.619369334	0.019148336	5487	6	786
AY635466	-3.2947492	-0.8812609	1.539545141	0.030151003	69	6	833
CN644911	-0.1624788	-0.7766612	1.56926896	0.045627036	4651	6	603
CN644911	-0.0250006	-0.8743828	1.56926896	0.030425971	4652	6	561
NM_006255	0.25007373	-0.9479334	1.791951498	0.037835553	1266	6	654
NM_020786	-0.0269157	-1.2311888	1.935291862	0.01718479	1156	6	549
NM_020786	-1.2679294	-0.6437697	1.935291862	0.031309583	70	6	855
CN802199	-0.5545002	-1.0803501	3.29772434	0.034880992	5248	6	846
CN802199	-0.7392591	-0.7085122	3.29772434	0.045608255	5249	6	850
NM_006902	-0.9872019	-0.9263439	1.936590108	0.041687156	1221	6	666
NM_005700	0.04529481	-1.0837054	1.562474306	0.018030274	1142	6	552
NM_005700	0.07204944	-1.1159398	1.562474306	0.019330646	1141	6	559
NM_005700	0.27742626	-1.0344055	1.562474306	0.018602338	1143	6	570
NM_004941	0.03620887	-0.5139323	1.748673184	0.034844575	5272	6	723
NM_002120	-1.8601279	-1.0034503	1.517534434	0.033153646	173	6	853
NM_002120	-0.4842829	-0.9183629	1.517534434	0.036688114	237	6	720
NM_005333	0.43262339	-1.2353585	1.608299153	0.004088181	1190	6	514
XM_371581	-0.0460908	-0.6463888	1.738870663	0.037740277	1159	6	638
NM_003879	0.21801833	-1.1179808	2.060117681	0.020576871	5271	6	830
NM_003879	-0.0375838	-0.9531672	2.060117681	0.017414074	1277	6	470
NM_000143	0.16191723	-0.3761731	2.364058197	0.02845837	5121	6	627
NM_020685	-0.137005	-0.6759632	1.685911425	0.012884198	1236	6	315
NM_002250	0.29383981	-1.7774607	2.643176774	0.02534101	11310	6	824
NM_152219	-0.2053489	-0.8045841	1.739296805	0.048152504	1218	6	631
CN646245	-0.0492746	0.09110327	2.369852663	0.048501076	5170	6	683
CN646245	-0.4483948	-0.4149105	2.369852663	0.035119688	5120	6	622
NM_002318	0.44319422	-1.2356193	2.002940636	0.006701254	1273	6	499
NM_000285	-0.1738323	-0.789119	1.708805166	0.018418371	1233	6	387
NM_000285	-0.0898946	-0.8813856	1.708805166	0.013186305	1234	6	345
NM_003833	0.38929395	-0.9103262	2.039912226	0.01472843	1157	6	501
NM_003833	-0.9475554	-0.7808078	2.039912226	0.017499041	4714	6	676
XR_014609	-0.1567947	-1.0514827	1.487397301	0.039124052	1217	6	636
NM_000772	0.00101476	-0.0629451	1.956875856	0.019585367	5168	6	521
CN644516	-0.4281013	-1.0482959	1.414757919	0.001893599	4107	6	357
NM_153236	2.95349659	-0.7965621	2.102771357	0.04121696	5870	6	845
NM_021137	-0.6381973	-0.9648993	1.486521683	0.004618749	1773	6	498
XR_013734	0.04748659	-0.9056667	1.726241978	0.02139424	1144	6	515
NM_006406	0.13052401	-0.7398783	3.857922381	0.044165863	5252	6	861
NM_001198	-0.0371685	-1.2030415	1.973002099	0.004510174	1135	6	306
NM_002413	0.19168986	-0.8223592	1.900906351	0.036205407	1267	6	580
NM_002413	0.21349725	-0.1789452	1.900906351	0.047332747	1258	6	582
CK230616	-0.8124529	-0.585766	1.590833888	0.031162743	238	6	759
NM_007348	-0.2973185	-0.9960264	1.662150429	0.007513052	1231	6	343

NM_003524	-0.2245502	-1.0752069	1.523129937	0.026149707	1138	6	609
NM_018368	-1.6289948	-0.2534371	1.844923073	0.04920825	235	6	802
NM_032732	-0.7447421	-0.9170185	1.486194613	0.004956179	3980	6	469
NM_032732	-0.1767343	-1.0250091	1.486194613	0.001903402	4114	6	286
CN647604	-1.6639543	-1.0029147	1.569373998	0.04569664	45	6	868
XM_031561	0.14964009	-0.1578474	1.909940829	0.030452793	1257	6	531
NM_004544	-1.3713107	-1.2720582	2.666566232	0.009689849	5115	6	734
NM_004544	-0.5810162	-0.7312192	2.666566232	0.015588938	5118	6	588
CO648465	-0.1869383	-0.9925128	1.604886264	0.003962069	4648	6	263
NM_006895	-0.9629693	-0.5485782	1.703707488	0.019186729	4739	6	563
NM_004906	-0.5981631	-1.8264586	2.629219828	0.036239992	11306	6	852
NM_004906	-0.585657	-1.5250691	2.629219828	0.043426155	11307	6	836
NM_030752	-0.2230902	-0.7221915	1.670757995	0.006281523	1232	6	247
XM_375853	-0.6352762	-0.0954197	1.88867262	0.023427207	4872	6	621
NM_016533	-0.5567213	-0.8427171	2.057583695	0.042181151	1275	6	658
NM_153374	-0.2190158	-1.166476	1.640951767	0.00295599	4647	6	292
NM_005192	-0.1956331	-0.4447853	3.354859521	0.033360309	5251	6	829
XR_013871	-0.0532767	-0.9217413	2.187686822	0.011905187	1173	6	422
XR_013871	-0.4438818	-0.7951005	2.187686822	0.006806414	4715	6	441
NM_031452	0.25404867	-1.0942617	1.589052932	0.001161188	945	6	331
NM_031452	-1.2897899	-0.8464224	1.589052932	0.026926885	43	6	791
NM_015341	0.02743304	-0.9518675	1.630156209	0.004990313	4053	6	457
CN641808	-0.3000862	-0.703638	1.692949468	0.038047608	1145	6	578
NM_005044	-0.6747437	-1.1869739	1.943024298	0.0032371	546	6	346
NM_005044	-0.3689795	-0.7012883	1.943024298	0.004601017	1238	6	138
NM_005044	-0.3805413	-1.0068905	1.943024298	0.003214624	1237	6	142
NM_005044	-0.2143211	-0.5784189	1.943024298	0.006046913	1239	6	147
NM_016817	1.02905174	-1.2339403	1.819703161	0.003646694	5110	6	717
NM_020158	-0.2037577	-1.1881339	1.624617718	0.008962778	1196	6	442
NM_020239	-0.7721809	-0.8378334	1.50014904	0.018291621	4731	6	581
NM_004560	-0.170011	-1.2753354	2.228678032	0.015652017	1172	6	596
NM_080655	-0.7950758	-0.4875421	2.177432603	0.026163387	5126	6	590
XM_375247	-0.0931643	-0.8453734	2.07905892	0.041916534	1154	6	661
NM_014570	-0.6848821	-1.0646129	1.549945375	0.001779315	641	6	420
NM_014570	0.09853785	-0.9999676	1.549945375	0.001846872	725	6	244
NM_007042	-0.4317064	-0.845627	1.656479657	0.002227596	1225	6	165
XR_011615	-0.8064616	-1.0029677	1.698105476	0.009101162	4707	6	509
NM_182919	-0.6093908	-1.3443461	1.996747685	0.012171738	1274	6	553
NM_182919	-0.1849548	-1.1710784	1.996747685	0.015220393	1210	6	494
NM_138451	0.04670067	-1.0796354	1.926170581	0.048204765	1324	6	702
NM_021227	-0.6691112	-0.4772434	1.768665568	0.021816328	5001	6	468
NM_021227	-0.1818597	-0.5106994	1.768665568	0.023907404	1248	6	388
NM_213720	0.11814928	-1.3618324	1.823038121	0.000633092	835	6	237

NM_024028	-0.094072	-0.4966967	1.903065691	0.036317933	1262	6	505
CN644462	-0.2520855	0.17851542	2.319556867	0.025663768	5169	6	591
NM_012176	-0.0644011	-1.0076673	1.62588897	0.000945558	943	6	179
NM_012176	-0.2219218	-0.7215652	1.62588897	0.004140681	1226	6	216
NM_003592	-0.4546488	-0.5273575	1.967955588	0.027470214	5123	6	504
NM_003592	-0.4099912	-0.5238415	1.967955588	0.027533284	5124	6	491
NM_006273	-0.145779	-1.3965516	3.290346891	0.01455769	5247	6	816
CO644768	-0.0034584	-1.1383564	2.101497425	0.046857287	1325	6	743
NM_003821	-0.3214198	-1.4593483	2.167833334	0.005749269	1127	6	506
NM_023937	-0.1715084	-1.3291539	2.233774015	0.005820743	1282	6	397
NM_023937	-0.2469517	-1.0583569	2.233774015	0.008635989	1283	6	367
NM_006808	-0.0931702	-1.3397814	1.780445565	0.00453813	1206	6	363
NM_006808	-0.1709874	-1.1655812	1.780445565	0.005652163	1207	6	323
XR_010283	-0.3208179	-0.8869807	1.516553263	0.000170731	4156	6	150
CN803456	-0.2185663	-1.0388013	1.847375777	0.004455904	4706	6	497
CN803456	0.40177457	-0.4710481	1.847375777	0.010517337	1160	6	532
CN803456	-0.844925	-0.6250013	1.847375777	0.007976532	4708	6	450
NM_205848	0.0448516	-1.4205244	2.451853959	0.047035182	1322	6	812
NM_205848	0.05880662	-1.3950111	2.451853959	0.047280803	1323	6	807
XR_012020	-1.403985	-0.8284951	1.713697866	0.000532122	4727	6	657
NM_015444	0.17318183	-1.2296697	2.362609137	0.006271968	1285	6	456
NM_022454	-0.0489817	-0.2479815	1.979631291	0.037848141	1265	6	539
NM_022454	-0.1537172	-0.0966675	1.979631291	0.043931566	1263	6	614
NM_003863	-0.6051094	-0.8856493	1.43599647	0.000463663	4142	6	329
NM_003863	-0.4303207	-0.9772323	1.43599647	0.000344642	4143	6	266
NM_016034	-0.2900346	-1.1547227	1.644570971	0.004705142	1195	6	424
XR_010624	-0.0394186	-0.9831109	1.909258001	0.001988961	876	6	95
NM_006928	0.02960313	-1.0899728	2.770207964	0.045657592	5190	6	814
NM_006963	0.04300886	-1.3285012	1.763403305	0.001572352	1208	6	261
NM_006963	-0.0702824	-1.082384	1.763403305	0.002789113	1209	6	238
NM_003486	0.25737661	-0.9584914	2.427810254	0.015454	1290	6	560
NM_006327	-0.7446499	-0.9134992	2.185039965	0.006519507	4713	6	373
NM_003091	-0.520684	-0.9865473	1.426775113	0.000246951	4141	6	295
NM_014822	-0.6136256	-1.2946548	2.350710317	0.003285719	547	6	517
CO579644	-0.6216398	-1.0418095	1.4085828	9.94E-06	662	6	352
NM_007178	-0.3786856	-0.9141926	1.651061692	0.000387886	4138	6	107
NM_007178	-0.3924243	-0.9296744	1.651061692	0.010254256	642	6	370
NM_017975	-0.0232956	-0.5968887	2.522222501	0.016398511	5160	6	571
A_01_P001492	-0.710438	-0.0038161	2.048285783	0.042779038	5002	6	600
AL831922	-0.0130887	-0.7064477	1.74861124	0.023185385	1247	6	412
NM_013237	-0.0248526	-1.2264374	1.769492255	0.00020813	963	6	101
NM_013237	0.02116237	-0.8461618	1.769492255	0.000390037	970	6	62
A_01_P007045	-0.1748762	-0.7619445	2.325951278	0.015047902	1289	6	415

A_01_P007045	-0.2597389	-0.8183789	2.325951278	0.013123492	1288	6	385
NM_006373	-0.4989986	-1.3574901	2.356077423	0.002860962	1133	6	400
NM_006373	-0.2991854	-0.9772613	2.356077423	0.003305859	1291	6	236
DR774547	-0.4690768	-1.1774268	1.738721119	0.004581664	1198	6	326
NM_005805	-0.0590386	-0.6317509	2.000711261	0.014236597	1147	6	436
NM_005805	-0.6722795	-1.3532395	2.000711261	0.007639798	4705	6	663
NM_013281	-0.6367002	-0.0955738	1.796832079	0.001685065	4875	6	440
NM_000311	-0.4886126	-0.8288889	2.748098591	0.021413275	5215	6	670
NM_000311	-0.7836845	-0.6036029	2.748098591	0.025383986	5214	6	705
NM_052849	0.65041818	-1.1737223	1.888897406	0.001581908	1169	6	471
NM_052849	0.76108351	-0.9853084	1.888897406	0.000600603	1170	6	448
XR_013105	-0.2460746	-0.343376	1.748022477	0.001656938	4874	6	408
NM_053045	0.22749044	-0.8927935	1.922019306	0.00585807	1151	6	287
NM_053045	0.2762176	-1.1702866	1.922019306	0.006297006	1150	6	428
NM_004053	-0.1129568	-1.2913291	2.069988579	0.008920963	1280	6	453
NM_001099	0.73362975	-0.3203084	2.079063691	0.015140286	5277	6	809
NM_001099	-0.0830611	-0.0220041	2.079063691	0.029705991	5166	6	530
NM_024580	0.07958149	-0.9239715	1.533997031	0.007626176	744	6	384
NM_018169	0.08546585	-0.39835	1.997984828	0.001851073	4520	6	160
CB310061	-0.1097217	-1.2262424	1.789629176	0.000754866	879	6	212
NM_002205	-0.3419597	-0.866126	1.685808102	0.013883967	4649	6	405
XR_010663	0.43422344	-1.2146484	1.71805644	0.002558145	728	6	386
NM_003136	-0.676428	-1.2451746	1.818826563	0.000861219	4709	6	360
NM_006817	-0.1423994	-1.2552988	1.739275193	0.000241695	962	6	118
NM_006817	-0.0432495	-0.8760982	1.739275193	0.00023647	968	6	48
NM_022488	-0.0027669	-1.0504626	1.663351717	0.003420433	710	6	289
CO646712	0.03408696	-1.353746	2.168450566	0.002028394	1136	6	358
NM_000579	0.50107245	-0.9102066	2.765429056	0.043127535	5194	6	804
NM_000579	1.13970656	-0.4597806	2.765429056	0.036247337	5278	6	851
NM_013995	0.16445839	-0.8097621	1.662595492	0.001820941	4310	6	192
NM_013995	0.5979857	-1.1144371	1.662595492	0.00568178	1335	6	555
NM_001933	-0.4924254	-0.7623817	1.727914814	0.033044673	1220	6	536
NM_001933	-1.203751	-0.9882387	1.727914814	0.032586017	5474	6	754
NM_004346	0.34493642	-1.2520733	1.638791337	0.005222298	1211	6	493
NM_004346	0.36766064	-1.224151	1.638791337	0.004173311	1212	6	478
NM_004642	-1.0539071	-1.2201204	1.776005541	0.002853073	4730	6	522
NM_004642	-0.9322595	-0.9721742	1.776005541	0.006200272	4762	6	495
NM_018482	0.09571773	-1.1256906	1.711243789	0.000600467	966	6	149
NM_006136	-0.1147316	-1.1700099	1.612961598	0.000692066	708	6	208
NM_004069	0.12884963	-1.3059883	1.714279064	0.000106402	895	6	202
NM_002388	0.01235999	-0.8536367	1.802024035	0.000165157	969	6	40
NM_002388	0.27826034	-1.0914483	1.802024035	0.003989365	1009	6	401
NM_002118	0.17697812	-1.116624	1.583684328	0.000239036	726	6	211

NM_002118	0.27049373	-1.1463707	1.583684328	0.002137816	727	6	349
NM_000165	-0.1462821	-0.7222795	1.838203117	0.000453248	4502	6	30
NM_000165	0.14083605	-0.740139	1.838203117	0.005776397	4519	6	225
XM_373742	-0.127327	-0.775844	1.737258097	0.000188818	967	6	43
XM_373742	0.05160211	-0.566635	1.737258097	0.000256265	4298	6	91
XR_010922	-1.1282661	0.27883462	2.024408889	0.049150623	5200	6	787
NM_018297	-1.2293652	-0.8611628	2.390633566	0.046099926	5862	6	795
XR_014008	-0.6122353	-0.3641013	1.721484145	7.21E-05	4796	6	204
XR_014008	-0.257472	-0.3548357	1.721484145	7.94E-05	4487	6	128
XR_014008	-0.5340895	-0.4748849	1.721484145	0.000276282	4795	6	140
NM_001539	-0.2434468	-0.6482936	1.778749032	0.000786111	4503	6	77
NM_001539	-0.1990736	-0.6378871	1.778749032	0.001084279	4500	6	75
CK230400	-0.9161274	-0.6895018	1.887856486	0.001727704	4763	6	291
CK230400	-1.0607295	-0.5034163	1.887856486	0.004401933	4764	6	462
CK230400	-0.6873652	-0.0246874	1.887856486	0.001123083	5005	6	410
CK230400	-0.8898804	0.05186204	1.887856486	0.002506128	5004	6	534
NM_004725	-0.7334861	-0.4798195	1.608724978	0.009260602	4742	6	425
NM_004725	-0.8828344	-0.4248976	1.608724978	0.005899954	4741	6	487
NM_014245	0.64149739	-1.1398812	1.893337629	0.002873119	1214	6	399
NM_014245	0.98296375	-1.1283338	1.893337629	0.003247515	1337	6	604
CN648808	0.11860484	-1.2375366	1.865601553	0.000619348	896	6	189
CN648808	0.20314366	-1.2288651	1.865601553	0.000351601	897	6	181
NM_013352	-0.3163404	-0.4399705	1.741940671	0.000716367	4794	6	172
NM_013352	0.00864875	-0.8077132	1.741940671	8.15E-05	4501	6	60
NM_007217	0.16349457	-0.9956785	1.565594529	0.004292855	746	6	330
NM_058217	0.97510887	-1.0129183	2.700555457	0.039037208	5198	6	826
CN802386	-0.1211043	-0.7661499	2.252924002	0.001040593	1292	6	29
CN802386	-2.6849749	-1.2189537	2.252924002	0.005383161	21	6	859
CN802386	-1.3707759	-1.0776398	2.252924002	0.009960212	5369	6	742
NM_012249	-0.0936439	-1.0911712	1.490199343	0.002599982	743	6	312
NM_002901	0.01653509	-1.0293793	1.830431335	0.000112449	965	6	32
NM_002901	-0.0111202	-1.0055021	1.830431335	0.00043801	964	6	42
NM_012112	-0.2293566	-1.1220808	1.515357047	0.000386041	3995	6	270
NM_002626	-0.22269	-1.2519822	1.847850388	0.002974937	1199	6	250
NM_002626	-0.2745465	-0.887602	1.847850388	0.003908872	1200	6	144
CO647278	-0.0242391	-1.2296701	1.698709002	1.45E-05	894	6	109
NM_003463	-0.7383608	-0.7241767	1.601731739	0.001830025	4740	6	313
NM_003463	-1.4217691	-0.5812315	1.601731739	0.018384636	233	6	797
NM_020640	-2.0945477	-0.7335017	1.555526403	0.028066873	183	6	825
NM_018145	-0.4311522	-0.0474969	1.8073414	0.00182979	5010	6	390
NM_000358	-0.0358419	-0.3236529	1.837947592	0.000177664	4505	6	110
NM_000358	-0.0747682	-0.5132755	1.837947592	0.001008315	4504	6	68
NM_030766	0.07227566	-1.4267544	3.193509546	0.007690577	5257	6	749

NM_030766	-0.0549894	-1.4016452	3.193509546	0.008633139	5256	6	751
NM_152911	-0.4738392	-0.9745332	1.998543519	0.001023838	4711	6	143
CB549988	-1.0278825	-1.5332501	2.821706	0.047322328	5861	6	856
NM_020457	-0.4909857	-0.9343308	1.956558304	0.001069498	4712	6	223
NM_020457	-0.4251046	-0.5836849	1.956558304	0.001083109	4800	6	153
NM_018445	0.06235944	-1.2965847	1.809546981	0.000372439	722	6	152
NM_004633	-0.1441531	-1.1560925	2.548693716	0.015577296	1281	6	626
NM_004633	-0.2961758	-1.5135517	2.548693716	0.010688583	1294	6	697
NM_006014	-0.1833793	-1.224552	2.450205942	0.001646153	1284	6	191
NM_006014	-0.0238272	-0.840585	2.450205942	0.002398935	1293	6	173
NM_014175	0.07619857	-1.0530017	1.534933177	0.000499887	739	6	429
XR_012647	-0.7544594	-0.7999253	2.386038237	0.008456788	5392	6	625
XR_012647	-0.2776895	-0.776182	2.386038237	0.005072468	5144	6	201
NM_033306	0.55790235	-0.9641836	1.637067011	2.50E-07	4322	6	325
NM_033306	0.6750653	-1.1886832	1.637067011	0.000434573	635	6	519
NM_015332	-0.578632	-0.8405122	1.85722289	0.015422131	5122	6	473
NM_015332	-0.1910529	-0.367045	1.85722289	0.027304936	5125	6	460
NM_013300	0.02003071	-0.8159448	1.835855617	0.020515438	1146	6	512
NM_001065	-0.1953027	-1.0744172	1.807695093	5.37E-06	880	6	65
NM_001065	-0.0025514	-0.8944902	1.807695093	1.62E-05	974	6	15
NM_000416	-0.3337082	-0.4354702	2.24427326	0.004493531	1264	6	184
NM_000416	-1.0402025	-0.2234048	2.24427326	0.005181258	5201	6	617
NM_153649	-0.9217788	-0.8257189	1.625770031	1.22E-05	4769	6	335
NM_153649	-0.6353811	-0.7341258	1.625770031	1.25E-05	4771	6	158
NM_003965	-0.5643853	-0.2147341	1.797143376	0.003467076	5003	6	319
NM_015161	-0.047235	-1.4126995	1.924361483	0.000527627	902	6	190
NM_004867	0.13471477	-0.2270557	2.313269955	0.003707109	5167	6	342
NM_004867	-0.9910741	0.16948607	2.313269955	0.014771643	74	6	841
NM_000194	-0.0898627	-0.958375	1.529711828	0.001126766	687	6	185
NM_002168	0.16453396	-1.1154915	1.719144554	2.86E-06	959	6	96
NM_002168	-0.1431146	-0.9553289	1.719144554	0.000632563	686	6	69
NM_001660	-0.3507336	-1.0958901	1.599638189	0.001475964	677	6	277
NM_001660	-0.3688195	-1.0763923	1.599638189	0.001943715	678	6	260
NM_002105	0.03115033	-1.1638794	1.77775592	4.07E-05	957	6	82
NM_007261	-0.2154493	-1.5490007	2.149207876	0.000915536	1129	6	353
NM_006049	-0.5796241	-0.819207	1.756318043	0.001259565	4807	6	252
NM_001416	-0.2651343	-1.1888174	1.601136991	4.81E-06	4012	6	131
NM_148979	0.02817019	-0.9275169	1.648921861	4.38E-06	954	6	70
NM_148979	0.01440547	-1.1030714	1.648921861	9.67E-06	949	6	93
NM_148979	0.08563642	-1.0613666	1.648921861	1.60E-05	958	6	103
NM_148979	-0.1646583	-0.9978529	1.648921861	7.31E-06	947	6	58
NM_003282	-0.1875189	-0.8619802	1.512686281	0.003371306	4044	6	280
NM_007022	-0.0604782	-1.0308652	1.874664528	0.000437439	946	6	76

NM_007022	0.2113933	-1.1288177	1.874664528	0.000692315	944	6	222
NM_025079	0.06207012	-1.0428311	1.646778633	1.05E-05	953	6	116
CO647754	-0.569077	-0.9787884	1.563333054	0.001067715	673	6	285
CO647754	-1.0227148	-0.7813701	1.563333054	0.007556103	4770	6	547
CN646039	-0.7347063	-1.13799	1.573816656	0.000490648	667	6	336
CN646039	-0.406905	-1.0400697	1.573816656	0.001120071	672	6	198
NM_018475	-2.4652729	0.02704921	2.120037781	0.005750774	67	6	873
NM_018475	-0.4487543	0.09171281	2.120037781	0.009527764	4997	6	472
NM_004553	-0.6475703	-1.0478818	2.076234779	0.002597968	4710	6	293
NM_016816	-0.0973287	-1.1355731	2.325216809	0.008225644	1286	6	407
NM_016816	-0.2685777	-0.8388848	2.325216809	0.011497232	1287	6	394
NM_014861	0.48228488	-0.5982623	2.333959097	0.044047046	1175	6	738
NM_014861	-0.3076808	-0.2750208	2.333959097	0.002982362	5164	6	229
NM_002488	0.00590065	-1.0271796	1.622756673	0.000154258	951	6	124
NM_014062	-0.1131528	-1.4691627	2.739648774	0.029761029	5188	6	815
NM_014062	-0.2281996	-1.116435	2.739648774	0.039127888	5189	6	794
NM_002615	-0.4290365	-0.9874301	1.765976545	8.11E-08	4776	6	31
NM_002615	-0.3328802	-0.805289	1.765976545	3.49E-05	4779	6	26
NM_052853	-1.2716567	-0.5827663	1.527966602	0.004326061	222	6	741
BM423313	0.07546282	-1.4997835	2.08889863	3.60E-05	912	6	163
BM423313	0.06924284	-1.3043639	2.08889863	6.41E-05	913	6	67
CN641646	-0.315302	-0.917649	1.515119077	0.000883552	4027	6	359
CN641646	-0.6748184	-0.9078151	1.515119077	0.000276108	3585	6	438
NM_001780	0.5244058	-0.333718	1.845251306	2.60E-05	4557	6	337
NM_001780	0.46917018	-0.3263142	1.845251306	1.14E-05	4556	6	284
CO774990	-0.4634272	-0.9381308	1.921096824	8.00E-05	865	6	12
CO774990	-0.3051268	-0.9242473	1.921096824	0.003065434	4804	6	136
NM_002560	0.09372572	-0.9827337	1.709936229	7.99E-06	955	6	63
NM_000308	-0.1050145	-1.0644812	1.684506671	2.15E-05	948	6	61
NM_032558	-0.5582543	-1.0044369	1.554508327	0.000391736	668	6	218
NM_032558	-0.5753483	-1.0563838	1.554508327	0.002056398	669	6	347
NM_005274	-0.3201024	-1.1830525	1.668041046	2.51E-06	4015	6	97
XR_011197	0.33931154	0.43812335	2.277398765	0.03601841	5173	6	725
XR_011110	0.23432808	-1.11208	1.687784197	1.30E-06	941	6	164
NM_007085	0.23570745	-0.4243254	1.754582705	0.000885809	4553	6	230
NM_007085	0.08691413	-0.4230386	1.754582705	3.04E-05	4489	6	115
NM_001288	-0.4132006	-0.9468325	1.740187094	1.49E-05	4777	6	46
NM_001288	-0.409898	-0.8413761	1.740187094	5.75E-05	4778	6	44
NM_002094	0.41341783	-1.2767452	1.677368262	0.000178786	939	6	355
NM_002094	0.24865313	-1.0947149	1.677368262	6.21E-05	961	6	177
NM_001778	-0.3441616	-1.0248831	2.034793633	0.000234932	875	6	19
NM_001778	-0.4185545	-1.0415548	2.034793633	0.000397923	874	6	27
NM_024829	-0.1006874	-0.2152005	1.828547094	0.000178553	4491	6	182

NM_024829	-0.3465674	-0.0351683	1.828547094	0.000715965	4468	6	303
NM_002946	-0.203719	-0.5215221	1.731781654	0.006105593	4808	6	364
NM_181836	-0.5504354	-0.8981274	1.508091027	0.005205404	1776	6	687
NM_181054	-0.2988183	-1.0349613	1.759421747	0.000329415	685	6	54
CO581027	-0.2799034	-1.4880241	2.206339269	0.00434182	1125	6	486
NM_001450	-0.5324075	-1.6135035	2.319185441	0.000894647	1130	6	393
NM_001892	-0.2871942	-0.8826211	1.514700371	0.000491199	684	6	195
NM_001892	-0.4627063	-1.0579238	1.514700371	0.002094457	671	6	279
NM_182513	0.41496588	-0.8996714	2.188834907	0.001442393	1174	6	322
NM_182513	-0.2873249	-1.2769505	2.188834907	0.001181864	877	6	141
NM_181512	0.04461727	-1.1990742	1.898672424	0.010524073	1022	6	414
NM_022154	-0.5425004	-0.4234646	1.83981903	0.004010156	5006	6	197
NM_022154	-0.3670331	-0.0767433	1.83981903	0.01319926	5007	6	398
NM_019111	-0.0971629	-1.4067068	1.9174185	2.96E-05	849	6	114
NM_024496	0.11012388	-0.4840483	1.870638463	0.000635437	4514	6	139
NM_014788	-0.0285799	-1.2962015	2.060438436	0.011993552	1148	6	524
NM_014788	0.33744685	-0.9443584	2.060438436	0.016358706	1153	6	546
NM_016613	-0.0709207	-1.3156914	2.450003848	0.018722613	1117	6	669
NM_021708	-0.2965093	-0.4522126	2.233623258	0.00212907	5162	6	127
NM_021708	-0.1337916	-0.2995924	2.233623258	0.003995103	5163	6	318
NM_004079	-0.5479653	-1.0481576	1.396737983	0.004590243	737	6	573
NM_002790	0.01329777	-0.724834	1.775012461	3.04E-05	4297	6	36
NM_002790	0.19074546	-0.7085119	1.775012461	5.62E-05	977	6	72
NM_001747	-0.2007235	-1.3950142	1.947140483	0.000383884	848	6	170
NM_001772	-2.5479976	-0.1767866	2.416646313	0.004878426	68	6	863
NM_002298	0.10037851	-0.774	1.975441684	2.66E-05	976	6	10
NM_002298	0.14135618	-0.9015386	1.975441684	1.96E-05	975	6	11
NM_006762	-0.4996072	-0.5641622	2.818477927	0.018489751	5218	6	674
NM_006762	-0.1714735	-1.0682202	2.818477927	0.009198047	1295	6	630
CK231384	-0.0367733	-1.0656195	1.631182678	0.000220162	950	6	122
CK231384	-0.0128203	-0.7350395	1.631182678	0.000123541	4296	6	104
XR_012275	-0.6305235	-0.6278545	1.686725531	0.00027256	4772	6	151
XR_012275	-0.4828061	-0.6922565	1.686725531	0.000831949	4773	6	120
NM_198552	-0.5094577	-1.4235741	2.931904509	0.00352351	5114	6	619
NM_018370	-0.0915761	-0.9769858	1.93818973	6.11E-06	973	6	3
NM_018370	-0.1977904	-1.0987734	1.93818973	2.20E-05	866	6	9
NM_005099	-0.1690598	-1.5147396	2.31750334	0.001594217	1126	6	389
XR_012149	-0.5560377	-0.3570607	2.323321614	0.008492132	5127	6	403
XR_012149	-0.6735733	-0.0876204	2.323321614	0.011566233	5128	6	516
NM_024946	3.3349558	-0.6661916	2.291757078	0.042412845	11262	6	867
NM_006372	-0.545404	-0.7374777	1.741986904	0.000160906	4759	6	356
NM_006372	-1.8754626	-1.1473938	1.741986904	0.00047814	38	6	840
NM_000677	0.67422843	-1.2299168	1.793594685	0.005218637	1168	6	641

NM_016097	0.10984531	-0.9011271	1.57252154	0.000206233	952	6	135
NM_016097	-1.1103706	-0.6590549	1.57252154	0.000384391	4722	6	411
NM_005408	0.00045917	-1.0563979	3.135424554	0.01003332	5195	6	722
NM_005408	0.07554827	-1.050765	3.135424554	0.00797945	5196	6	713
NM_212482	-0.7860417	-0.5940034	1.819044633	0.000726433	4785	6	200
NM_212482	-0.784813	-0.4956594	1.819044633	0.003380924	4786	6	294
AY966403	-0.0281445	-1.3157627	1.899100305	1.79E-07	907	6	56
AY966403	0.04188339	-1.2644339	1.899100305	6.26E-07	909	6	55
AY966403	-0.0537847	-1.2555224	1.899100305	3.34E-06	908	6	34
AY966403	-0.0972816	-1.2713365	1.899100305	5.02E-06	906	6	38
NM_000063	-0.6067825	-1.0647482	1.65021381	0.000118658	4774	6	196
NM_000963	-0.8242639	-0.6494405	1.651625992	0.000159946	1777	6	689
NM_000963	-0.1372301	-0.7472066	1.651625992	0.001253356	688	6	106
NM_014369	-0.8893996	-1.2346163	2.91896768	0.001302851	5116	6	579
NM_014369	-1.1028718	-0.8620578	2.91896768	0.00237492	5117	6	642
NM_006002	-1.1363395	-0.7427716	1.834851393	0.000351822	4728	6	597
NM_001063	0.45119653	-1.2643855	1.872291761	1.31E-05	942	6	262
NM_001063	0.21749362	-1.1682097	1.872291761	3.10E-06	960	6	86
NM_002491	0.42245222	-1.1522078	1.652376925	0.007476763	634	6	544
NM_080677	0.35708385	-1.1079366	1.520265549	0.000549927	980	6	381
NM_080677	-0.8661048	-0.6789777	1.520265549	0.001278955	4721	6	592
XM_290631	-0.1278539	-0.1391691	3.239307761	0.043967445	5222	6	820
NM_000577	0.24131751	-0.8067564	1.928599906	2.65E-06	979	6	41
NM_000577	0.24634732	-0.8363575	1.928599906	1.16E-05	978	6	33
NM_003864	-0.7870316	-1.5077752	2.896335629	0.003281056	5113	6	714
NM_001746	0.34630774	-0.3138561	1.877115762	0.002398909	4548	6	298
NM_001560	-0.4765839	-0.1892266	2.643167498	0.010685098	5217	6	510
NM_001560	-0.5022929	-0.533439	2.643167498	0.00904273	5216	6	437
NM_152703	-0.7192963	-1.1130825	1.815982614	0.00250518	4801	6	324
NM_152703	-0.5033191	-0.9465974	1.815982614	0.002205961	4802	6	145
NM_004310	-0.282598	-1.5428726	2.125391853	0.000120034	853	6	227
NM_004587	-0.2598053	-0.8694872	1.590064604	0.00102257	4028	6	215
NM_004587	-0.3623298	-0.9643315	1.590064604	0.000649728	683	6	174
NM_002755	0.26988815	-1.2422417	1.815815968	9.49E-05	982	6	175
NM_012456	-0.1405023	-1.2695798	2.176066714	0.003562943	1149	6	307
NM_012456	0.12299371	-0.8607525	2.176066714	0.005451501	1152	6	245
NM_080546	-0.0293442	-0.5661106	1.92676606	0.000285268	4516	6	28
NM_080546	0.23162787	-0.2208913	1.92676606	7.52E-05	5029	6	210
NM_053067	0.39922766	-0.2099772	2.096357296	0.008544924	5159	6	461
NM_053067	-1.8611349	-0.6506473	2.096357296	0.006036784	46	6	866
NM_003183	-0.921704	-0.7638663	1.601765719	0.000854228	4720	6	416
NM_003183	-0.3299024	-0.8211824	1.601765719	0.005331427	681	6	249
NM_018838	0.07391017	-0.2418261	1.970388799	0.001977544	5021	6	231

NM_018838	0.40756323	-0.5271598	1.970388799	0.002057759	1309	6	241
NM_017813	0.15288472	-0.5444191	2.113601182	0.013006147	5158	6	365
NM_001012754	-0.4336099	-1.034808	1.774406542	3.98E-05	4775	6	66
NM_000628	-0.0708798	-1.5758102	2.263525512	0.000461264	986	6	268
NM_000628	-0.0441809	-1.3084986	2.263525512	0.000240739	987	6	64
CK232102	-1.5687477	-0.226119	1.961028813	0.01381153	4812	6	788
CK232102	-0.7317412	-0.0374037	1.961028813	0.018153469	4995	6	564
NM_005461	0.9379077	-0.524253	2.088525979	0.002265827	5242	6	649
NM_021249	-0.5411014	-0.3686757	1.696499592	0.002107916	4790	6	273
NM_021249	-0.3011276	-0.4950406	1.696499592	0.001726368	5017	6	161
NM_020154	-0.3341296	-0.9686034	1.92636238	0.010637797	5134	6	340
NM_020154	-0.1205218	-0.6686602	1.92636238	0.00253235	4515	6	81
NM_000402	0.13626338	-1.3194643	2.569777726	0.004094716	5788	6	502
NM_000402	-0.2612649	-1.5274997	2.569777726	0.000934501	5747	6	391
XR_012338	-0.7291963	-0.3536659	1.92063487	0.000837628	4729	6	430
XR_012338	-0.6943037	-0.2918711	1.92063487	0.001574298	4815	6	566
CN648722	-0.1902021	-0.1240081	2.570211061	0.009926435	5184	6	620
XR_009709	-0.1087873	-1.10924	1.715037908	0.000918944	680	6	134
XR_009709	-0.0251395	-0.5419141	1.715037908	0.003420479	4507	6	162
NM_020548	0.24294652	-0.0899107	1.959554541	2.72E-05	5030	6	297
NM_006700	-0.1860273	-1.2917995	2.036706519	0.000173786	881	6	102
NM_006700	-0.2516763	-1.161762	2.036706519	0.000209839	883	6	35
NM_006597	-0.8165683	-0.8047516	2.269297065	4.10E-05	872	6	90
NM_006597	-0.5920676	-0.9989025	2.269297065	0.000456402	873	6	53
NM_013324	0.45432264	-1.3325655	1.824825061	0.002222767	1007	6	446
XM_498423	-0.302589	0.14128589	2.616443651	0.006006889	5171	6	565
XM_498423	-0.1095315	0.55567151	2.616443651	0.010538647	5172	6	706
NM_003031	-0.3938302	-0.5891677	1.905538737	0.017749442	5129	6	409
NM_003031	-0.5888486	-0.2845694	1.905538737	0.021742432	5130	6	513
NM_003031	-0.3735926	-0.2253543	1.905538737	0.027862648	5131	6	541
NM_003031	0.01333505	-0.2967103	1.905538737	0.034573273	5157	6	511
NM_022570	-0.0165218	-0.7337543	2.579161947	0.003659824	5142	6	274
NM_022570	-0.1611478	-0.8063668	2.579161947	0.003672213	5141	6	253
CN642567	-0.7291422	0.08376997	2.091365439	0.00519158	4996	6	535
CK231776	-0.5406187	-0.8775631	2.745458356	0.042232014	5213	6	768
NM_014395	-0.1241047	-1.5402372	2.231677606	0.003837579	1319	6	466
NM_017832	-0.2147418	-1.0831446	1.421546278	0.002730961	4022	6	375
NM_032208	0.09257507	-0.3137103	2.121750005	0.002184631	5020	6	154
NM_032208	0.29763276	0.2407899	2.121750005	0.003045296	5035	6	554
NM_006472	-0.9958228	-0.900798	1.70128646	0.000656767	4767	6	396
NM_006472	-1.0855746	-0.7697246	1.70128646	0.000305565	4768	6	426
NM_017709	-0.1331041	-0.4451939	2.056073276	0.000219596	5019	6	23
NM_017709	-0.4675941	-0.8092464	2.056073276	0.00543512	4806	6	234

NM_001219	-0.1853199	-1.2621604	1.966187705	5.76E-05	911	6	39
NM_001219	-0.4763071	-1.4174696	1.966187705	4.35E-05	540	6	133
NM_001324	-0.244987	-0.8530439	2.174146425	0.003712919	5136	6	171
NM_002797	0.10881627	-1.0295401	3.665128656	0.004373542	5259	6	783
NM_002797	-0.2584358	-1.0157605	3.665128656	0.0041679	5258	6	776
NM_004458	-0.571826	-0.2542969	1.916207085	0.000290445	4797	6	205
NM_004458	-0.6672329	-0.1093396	1.916207085	0.000467741	4798	6	332
NM_015544	-0.8519961	-0.0757688	2.234290805	0.012802549	5206	6	637
NM_015544	-1.1922147	0.11519629	2.234290805	0.005907582	5202	6	770
CN645399	0.06179341	-0.9696235	2.334357434	0.023203758	1327	6	585
NM_198232	-0.173239	-1.3622261	1.86804729	0.00070383	846	6	214
NM_198232	-0.0644329	-1.3274054	1.86804729	3.51E-05	910	6	98
NM_021159	-0.1875575	-0.9683744	1.895236213	0.000113964	4780	6	18
NM_021159	-1.0343157	-0.8997957	1.895236213	0.001837295	4726	6	543
NM_015488	-0.2113901	-1.7570599	2.491645446	0.004572404	1320	6	644
NM_015488	-0.135832	-1.7963919	2.491645446	0.002991816	1318	6	643
NM_014385	-0.6141364	-0.4089596	1.649823719	0.009913706	4761	6	574
NM_004897	-0.374858	-0.1659574	2.496328238	0.004798974	5161	6	545
NM_201612	-0.2829896	-0.5529635	1.892940173	0.005451625	1301	6	186
NM_024027	-0.289633	-1.144462	2.142631414	0.000217073	869	6	24
CK231327	-0.4513441	-1.3546077	2.372182392	1.69E-05	870	6	83
CK231327	-0.3416621	-1.2058007	2.372182392	1.10E-05	871	6	14
NM_000090	-0.1701412	-1.5599102	2.165693284	1.32E-06	914	6	130
NM_000090	-0.1486613	-1.3195481	2.165693284	8.73E-07	915	6	20
NM_016579	-0.0812791	-0.8513568	2.114837377	6.63E-05	998	6	13
NM_016579	-0.5246235	-0.6613278	2.114837377	0.000106689	4784	6	49
CB551618	-0.7687492	-1.4495679	2.236529175	0.000527626	4695	6	379
CB551618	-0.757395	-1.3723625	2.236529175	0.001155634	4696	6	368
NM_152673	-0.3919294	-0.7402013	1.865838412	0.000419535	4783	6	50
NM_152673	0.00997821	-0.8908622	1.865838412	0.000743311	996	6	45
CO647113	-0.985644	-1.1868534	2.241933083	0.000109204	4697	6	316
CO647113	-0.8705022	-1.2468106	2.241933083	3.00E-06	4698	6	228
NM_005319	0.11084733	-1.5647351	2.13426378	0.000905831	1115	6	404
NM_000560	-0.6824952	-1.5158045	2.744784076	0.00022571	5749	6	443
NM_000560	-0.403943	-1.2992169	2.744784076	0.000349166	5750	6	255
NM_000560	0.57676569	-1.1875379	2.744784076	0.001555364	5781	6	537
NM_000560	0.30947431	-0.7909499	2.744784076	0.001797606	5147	6	350
NM_005561	0.62374975	-1.2020264	1.770528905	0.015558074	1307	6	634
NM_001906	0.19041269	-1.3231752	2.230760319	0.000623223	991	6	209
NM_001906	0.23316282	-1.2724462	2.230760319	0.000522249	992	6	176
NM_000391	-0.0195726	-0.9655243	2.125102119	1.99E-06	972	6	1
NM_000391	-0.1359633	-1.0291774	2.125102119	5.07E-06	971	6	2
NM_003272	0.00861972	-1.1242179	2.054702447	0.000109501	882	6	52

NM_003272	0.0794741	-1.0211676	2.054702447	0.000207459	885	6	79
NM_000676	0.32469165	-1.3280667	1.912942839	5.35E-05	934	6	314
NM_002592	-0.081207	-1.1918827	2.383740263	0.000292086	989	6	85
NM_002592	-0.0712354	-1.3656191	2.383740263	0.00048755	988	6	132
NM_153811	-0.1814934	-1.3112336	1.976292673	0.001004947	697	6	156
NM_016545	-0.547247	-0.478139	1.874305319	0.001367893	4791	6	168
NM_016545	0.9515617	-0.342608	1.874305319	0.045025166	5074	6	781
NM_004844	0.2129109	-1.4465366	2.045386513	0.000303006	929	6	257
NM_004766	0.19440343	-1.0629445	1.808142936	0.000565333	984	6	126
NM_004766	0.14486231	-0.8596737	1.808142936	0.000330643	985	6	78
NM_005534	0.17542821	-1.6082348	2.201450458	3.73E-06	917	6	243
NM_005534	0.30716887	-1.5974126	2.201450458	5.40E-06	928	6	309
NM_001011722	0.29301375	-0.6633305	1.667585857	0.002369902	4539	6	267
NM_001011722	0.25584153	-0.6872968	1.667585857	0.002030428	4540	6	276
NM_152713	-0.5259525	-1.112913	1.644836489	0.001340078	670	6	235
CN645487	0.17008339	-1.2782572	2.086561061	0.000742377	990	6	203
CN645487	-0.1111984	-0.0119063	2.086561061	0.00018181	5022	6	321
NM_001694	-0.4417034	-1.0991592	2.120853402	9.80E-06	867	6	6
NM_001694	-0.3857189	-1.1145378	2.120853402	9.77E-06	868	6	5
NM_006667	-0.130838	-0.6884075	1.799828416	0.002234049	4893	6	125
NM_006667	-0.539339	-0.5108981	1.799828416	0.004255239	4799	6	369
XM_498527	-0.3835849	-1.2043277	1.677508828	0.000971244	674	6	240
NM_014752	0.51933275	-1.054039	2.22166261	2.97E-06	5776	6	159
NM_014752	-0.0722659	-0.6825691	2.22166261	1.52E-05	5015	6	16
NM_004987	0.08023087	-1.1572595	1.548783262	0.016024329	654	6	526
NM_018229	-0.9225383	-0.4582234	2.2141009	0.003381257	4732	6	488
NM_018229	-0.9091437	-0.6316044	2.2141009	0.007548291	5204	6	463
XR_012476	0.57241724	-1.3588038	3.108485883	0.002785084	5197	6	733
CN648055	0.02138165	-1.4134364	2.278031065	6.11E-07	918	6	74
CN648055	-0.1005077	-1.2161759	2.278031065	9.40E-05	920	6	17
NM_005564	-0.1760806	-1.7162621	2.369376338	1.65E-05	859	6	339
NM_001558	-0.320105	-1.6382782	2.322005051	1.46E-05	863	6	296
NM_052889	0.2955225	-1.2214887	2.436321811	1.16E-05	5773	6	113
NM_052889	0.18919464	-1.2982102	2.436321811	3.70E-06	5772	6	100
NM_018656	0.11402986	-0.6381273	1.888073502	0.002648611	4517	6	123
NM_018656	-1.377292	-0.1152944	1.888073502	0.004294336	4718	6	746
CD768052	-0.0602161	-0.3208034	1.898890207	0.000352522	5018	6	105
CD768052	-0.4306396	-0.8765106	1.898890207	0.00294042	4803	6	129
NM_002396	-0.8077459	-1.4743185	2.984895041	0.00322492	5391	6	726
NM_000097	-0.8789871	-0.5761867	3.101350837	0.025575064	5221	6	785
NM_016733	-0.0899546	-0.9291594	2.165218415	0.000303841	5138	6	25
NM_016733	0.04359564	-0.5333647	2.165218415	0.000272203	5016	6	21
NM_003152	-0.2957518	-0.7890253	2.792451347	0.019732484	5193	6	729

NM_003152	0.22338245	-1.3853134	2.792451347	0.005141873	5799	6	628
XR_014800	-0.5285593	-1.7253832	2.659449551	0.000271414	5746	6	520
NM_000618	0.00039658	-0.8962952	3.394062018	0.024208255	5191	6	832
NM_000618	0.14836511	-0.6085507	3.394062018	0.028467827	5192	6	827
NM_002791	0.14450228	-1.285569	2.144486899	7.94E-06	919	6	51
NM_002791	0.22580112	-1.3654665	2.144486899	0.000197245	930	6	155
NM_021033	0.04274622	-0.6131953	1.781510324	0.002167969	4542	6	180
NM_021033	-0.0544443	-0.57006	1.781510324	0.00132922	4894	6	121
NM_017554	0.20405733	-1.1835659	2.101542861	0.001400032	994	6	178
NM_017554	0.27605493	-0.9563544	2.101542861	0.000733261	995	6	87
NM_002794	0.24330526	-0.7762973	1.950422789	0.005319602	4545	6	281
NM_002794	0.38600438	-0.8940615	1.950422789	0.00117087	4546	6	221
NM_007126	-0.3497998	-0.8082155	1.996145241	8.18E-05	4782	6	4
NM_007126	-0.4939175	-0.8045268	1.996145241	7.80E-05	4781	6	22
NM_006713	-0.1555025	-0.6670859	1.744679492	0.009764549	750	6	278
NM_018950	-0.2028145	-1.6117176	2.296788998	3.48E-05	855	6	207
NM_018950	-0.1479093	-1.4291521	2.296788998	7.96E-06	916	6	73
NM_021970	-0.3755305	-1.3799253	2.003293503	0.000972838	4699	6	362
NM_002696	0.25834428	-0.8020422	2.349484041	0.002960037	1310	6	310
NM_002696	0.36167057	-0.662392	2.349484041	0.003395957	1311	6	269
NM_006753	0.13724461	-0.8124613	1.873536273	0.001164539	4541	6	117
NM_006753	0.24953173	-0.8201343	1.873536273	0.001252156	999	6	146
NM_002345	0.41635321	-1.5410787	2.12495715	0.001281732	1008	6	503
NM_002345	-0.3980491	-0.9164282	2.12495715	0.005305062	4805	6	169
NM_005776	-0.3884147	-0.846998	2.618506343	0.00159774	5145	6	188
NM_005776	-0.3869413	-0.5995804	2.618506343	0.001378105	5208	6	540
NM_014506	-0.6308605	-1.1703308	2.463117001	8.15E-05	5133	6	317
NM_014506	-0.5986617	-0.9677047	2.463117001	0.000314262	5137	6	71
CO646433	-0.596876	-0.4887709	2.02666991	0.000303879	4787	6	99
CO646433	-0.4083588	-0.5446597	2.02666991	0.001000142	4788	6	47
NM_004233	0.61444668	-0.8548729	2.22013954	0.000126691	5778	6	259
NM_004233	0.54294723	-1.147995	2.22013954	0.000635451	5775	6	302
CO647386	0.24680798	-0.3536039	4.037359278	0.040718613	11321	6	862
NM_002041	0.27419913	-0.8089118	2.513262119	0.003143686	5143	6	311
NM_002041	-0.0999179	-0.2121989	2.513262119	0.008575693	5273	6	849
NM_020314	0.06081476	-1.3605587	2.987367895	0.001402171	5765	6	551
NM_020314	-1.1597928	-0.1472555	2.987367895	0.00304027	5211	6	716
CO581416	-0.3121489	-0.8858154	1.708508198	0.002228099	675	6	187
CO581416	-0.2495553	-0.6064232	1.708508198	0.002977457	4341	6	148
CN804590	-0.1000507	-1.3786788	2.322800979	0.000417752	886	6	166
CN804590	-0.0729657	-1.2319767	2.322800979	0.000268425	887	6	94
XM_374879	-0.0616517	-0.1260086	2.35312919	0.000579566	5038	6	224
XM_374879	-0.3739507	-0.0393012	2.35312919	0.001605414	5039	6	304

XM_374879	-0.2390666	0.31978497	2.35312919	0.001433166	5041	6	529
XM_374879	-0.4659151	0.27989474	2.35312919	0.001436332	5040	6	518
CK230208	0.5078774	-1.1079849	2.470939887	3.15E-05	5777	6	220
CK230208	0.42803411	-0.678973	2.470939887	0.000209985	5779	6	157
NM_032311	-0.032627	-0.0459566	1.881328784	0.002743336	4914	6	465
NM_005754	0.18609684	-1.1640507	1.931540841	0.000313774	983	6	108
XR_012082	0.44800522	-1.3394621	2.239088642	2.87E-05	5393	6	730
XR_012082	-0.2521891	-1.1283454	2.239088642	6.74E-05	884	6	8
NM_015660	0.02051105	-1.4373779	2.713734759	5.46E-05	5751	6	246
NM_015660	-0.0718793	-0.7770753	2.713734759	0.000209807	5146	6	88
NM_015149	-0.8695304	-0.4323389	1.648503759	0.03544306	4814	6	640
NM_016466	-0.0941664	-1.0652916	1.490405567	0.012493004	1369	6	572
NM_016466	-0.0444088	-1.1003091	1.490405567	0.008747594	736	6	455
NM_152562	-0.03773	0.19956097	2.053736128	0.039456145	5037	6	665
NM_152295	-0.3758422	-0.7171911	2.54792954	0.000758776	5139	6	92
NM_152295	-0.4667454	-0.5054502	2.54792954	0.002757559	5140	6	265
NM_015393	-0.2651308	-1.6365322	2.784779633	0.000314491	5748	6	475
NM_002485	-1.1508122	-0.6846236	1.657063797	0.005235783	232	6	773
NM_002485	-0.6447623	-0.9191832	1.657063797	0.003799532	4724	6	595
CO048892	-0.2121415	-0.5452129	2.076034033	0.022727452	1305	6	445
NM_025019	0.59204777	0.57252969	2.355346942	0.000890891	5058	6	736
NM_005384	-0.9723206	-0.5749979	2.442412199	3.52E-05	5207	6	492
NM_005384	-0.2567392	-0.6858853	2.442412199	0.000254403	4810	6	111
CB312177	-0.4390762	0.38558018	2.346577148	0.019131102	4999	6	677
NM_004237	-0.1204659	-0.2292477	1.825111655	0.004897664	4905	6	282
NM_170672	-0.5446689	-0.2929805	2.300172111	0.027944053	5132	6	675
NM_004847	0.17556092	-0.1049167	2.622316165	0.000764311	5165	6	395
NM_004847	0.51399458	0.39558277	2.622316165	0.003163583	5057	6	711
NM_003039	-0.5059218	-0.6219681	2.224311064	0.001207083	4789	6	59
NM_003039	-0.0993429	-0.505001	2.224311064	0.002666513	4518	6	89
NM_002627	-0.1290578	-1.2209077	2.814666213	0.000650781	5789	6	327
NM_002627	0.20711092	-1.424348	2.814666213	0.000165579	5790	6	419
XR_000287	-0.435494	-1.8256663	2.684568906	6.96E-05	5365	6	693
NM_031943	-1.11278	-0.7115062	1.54052076	0.017108799	4744	6	647
NM_031943	-0.7818728	-0.546754	1.54052076	0.011692775	4746	6	485
NM_001629	-0.0568985	-1.0069489	2.127034061	0.000781942	993	6	37
NM_001629	-0.0962082	-0.7661397	2.127034061	0.000481555	997	6	7
NM_014399	-0.2917443	-0.9398525	1.553980913	0.015079083	735	6	435
NM_002408	-0.3084773	-0.7720889	2.315192582	0.00011406	4809	6	305
NM_002408	-0.0905328	-0.6092504	2.315192582	0.000494039	4811	6	57
NM_001078	0.52110812	-1.074048	2.332908721	0.004803099	1308	6	527
NM_080881	-0.2876312	-1.0493251	1.653914401	0.006694405	748	6	348
NM_080881	-0.0702141	-0.9074547	1.653914401	0.007325376	749	6	283

CO580437	-0.0847428	-0.9013971	1.691368712	0.006951332	689	6	256
CO580437	-0.1280161	-0.9342596	1.691368712	0.008878734	676	6	334
NM_014890	-0.5063413	-0.4491407	3.149138632	0.030923633	5219	6	790
NM_014890	-0.5445121	-0.2157981	3.149138632	0.036547213	5220	6	798
NM_145725	-0.0515354	-1.5558765	2.45817106	0.000256227	5740	6	589
NM_005165	0.41993208	-1.634525	3.068289799	0.001312756	5817	6	701
NM_005165	0.22135059	-1.5079667	3.068289799	0.000466209	5819	6	577
NM_016209	-0.4141506	-1.4660176	2.09712322	0.00042648	1036	6	242
AK057584	-0.4629256	-1.053694	1.522497018	0.009972151	731	6	458
NM_016448	1.00159127	-0.5124382	3.050598841	0.005172903	5246	6	757
NM_004969	-0.1096388	-0.5862886	2.208757881	0.010483899	5148	6	333
NM_004969	0.17280172	-0.4770695	2.208757881	0.011576956	5149	6	377
NM_031453	-0.2116174	-1.2138386	1.716235278	0.00864631	733	6	417
NM_176798	-0.1660936	-1.3119588	2.929139095	3.33E-05	5752	6	290
NM_006307	-0.4192927	0.0717907	2.383752908	0.003433259	4998	6	479
CN644511	0.26360348	0.09973539	2.490224421	7.55E-06	5036	6	433
CN644511	0.47738157	0.60355998	2.490224421	0.000126669	5059	6	735
XM_371236	-0.619726	0.01824083	3.131802785	0.003024892	5185	6	744
NM_001238	0.07332534	-1.4009627	2.830698628	0.000146266	5818	6	341
NM_004024	-0.3632308	-1.4461258	2.136388802	0.000114757	1043	6	137
NM_004024	-0.0790596	-1.5461223	2.136388802	0.000211642	1045	6	206
NM_031299	0.36586904	-1.407277	2.412489772	6.65E-05	5742	6	361
NM_031299	0.52769745	-1.1332917	2.412489772	0.001935884	1010	6	421
NM_013242	-1.1389759	-0.3071138	1.577413005	0.029261357	4813	6	695
NM_002346	-0.7953703	-1.7374822	2.433982596	0.006928239	5472	6	721
NM_002438	-0.8138712	-0.0486881	2.264726654	0.002293683	4991	6	482
NM_002438	0.09935225	0.1043635	2.264726654	0.004772123	5033	6	439
NM_002438	0.08544047	0.13071889	2.264726654	0.004769227	5034	6	476
NM_002438	-0.9282437	-0.0566442	2.264726654	0.027751559	5205	6	651
CB311553	0.2297134	-1.4493169	3.11658238	0.000506744	5800	6	598
XR_012394	-0.5632925	-1.5743813	2.889144224	0.003783113	1314	6	668
XR_012394	-0.1670171	-1.0225342	2.889144224	0.006409731	1328	6	587
CN648522	-0.4996173	-1.0496776	2.005224399	0.009599351	1298	6	382
CN648522	-0.248396	-1.2395549	2.005224399	0.014128915	1296	6	550
CO647336	-0.4929044	-0.9410317	2.417024092	0.000770283	1299	6	84
CO647336	0.21574424	-0.5423438	2.417024092	0.002304341	1306	6	248
NM_052837	-0.2081374	-0.7934585	2.867144803	0.041218362	1331	6	777
NM_052837	-0.1943612	-0.8962674	2.867144803	0.035201051	1330	6	763
NM_052837	-0.237112	-1.2187437	2.867144803	0.02617302	1329	6	766
NM_052837	-0.2404551	-0.6083066	2.867144803	0.042024841	1332	6	758
NM_006404	0.16419269	-1.5995826	2.909484805	0.001006421	5786	6	594
NM_001177	-0.2333057	0.34511574	2.349826095	0.025975607	5042	6	653
NM_018184	-0.4588866	-0.4843288	1.978046566	0.021579425	1300	6	413

NM_018184	-0.1655188	-0.4622037	1.978046566	0.024885656	1303	6	418
NM_002467	-0.0551337	-0.6462816	1.651265912	0.013450668	4891	6	374
NM_002881	-0.8387987	-1.1671228	1.861208095	0.004135709	530	6	432
XR_014613	-0.5069498	0.12895816	3.312093971	0.005491948	5212	6	756
NM_014452	1.43721794	-1.6998546	2.333409586	0.001545919	5741	6	673
NM_002350	-0.4939857	-1.7231682	2.561628875	2.42E-05	5753	6	383
NM_002350	-0.2012455	-1.3480783	2.561628875	5.49E-06	5759	6	80
NM_021972	-0.1249153	-0.2108866	1.896164041	0.015351483	4912	6	477
NM_021972	0.30654115	-0.6317598	1.896164041	0.005178928	1000	6	264
NM_025196	0.06846191	-0.8726316	1.727730172	0.009114461	4846	6	431
NM_020169	-0.2897922	-1.2493431	1.890747411	0.001571132	1039	6	183
NM_001005415	-1.2584092	-0.852196	1.740250899	0.012163252	4743	6	667
NM_001005415	-0.3527232	-0.9860207	1.740250899	0.00815408	4845	6	444
NM_001553	-0.7100649	-1.5090288	2.856225831	0.000317943	5481	6	732
NM_000641	0.53191374	-0.3021517	2.848167233	0.035445188	5180	6	762
NM_003198	0.16129676	-1.1400344	2.336404832	0.011761614	1326	6	523
NM_002852	-0.2952566	-0.8311513	2.427423756	0.00187188	5135	6	119
NM_014437	0.09163308	-1.3566017	2.836813387	0.000124993	5744	6	496
NM_003901	0.12191762	-1.3099008	2.53946117	0.000181086	5766	6	193
CR627373	-0.5245167	-1.2190513	2.228247537	0.029973173	5876	6	700
CR627373	-0.612409	-0.8630903	2.228247537	0.024940934	1297	6	576
NM_004355	-0.5364202	0.45161916	2.382715246	0.014346022	5000	6	680
NM_004355	-0.8528519	-0.0751439	2.382715246	0.000361065	4990	6	423
NM_005738	-1.0611517	0.16072202	3.073630459	0.003697859	5203	6	817
NM_005738	0.50046058	-0.2552675	3.073630459	0.000601233	5236	6	606
NM_024956	0.15048203	-0.2547394	2.753774796	0.018829144	5179	6	664
NM_024956	-0.1597395	-0.5081338	2.753774796	0.026827857	5178	6	678
NM_016139	-0.2962485	-1.4396853	2.06572616	0.000989117	1041	6	226
NM_016139	-0.3681523	-1.2944128	2.06572616	0.001790984	1042	6	194
NM_001305	0.57663591	-0.2870841	2.040035493	0.022732744	1312	6	608
NM_000859	0.27166162	-0.1664278	2.035014985	0.007041329	4913	6	449
NM_000859	-0.4772977	-0.0826184	2.035014985	0.011774623	5044	6	648
NM_005623	1.37026308	-0.5592615	2.450307412	0.003014039	5243	6	710
NM_005623	0.11307263	-0.5260268	2.450307412	0.000504896	1001	6	112
NM_145039	0.41797545	-0.4277582	1.943211826	0.022706537	4911	6	507
NM_022170	-0.6178254	-0.8947895	1.437069367	0.037035697	730	6	623
NM_002182	0.33996691	-1.2611322	2.844243818	0.000941836	5791	6	556
NM_002182	-0.236504	-1.5044764	2.844243818	0.000216688	5762	6	380
NM_021821	0.56426654	-0.0754326	3.036061152	0.007442315	5182	6	718
NM_021821	0.39782974	-0.1088264	3.036061152	0.008454953	5181	6	698
DV770912	-0.4795663	-1.4199688	2.017579719	0.001851138	1038	6	338
NM_003706	-0.2447795	-1.8001852	2.76169494	2.98E-06	5756	6	459
NM_003706	-0.2095513	-1.7384331	2.76169494	1.47E-06	5757	6	406

NM_003467	0.64075984	-1.3337834	2.705911947	1.16E-05	5774	6	447
NM_003467	0.07155771	-1.4002919	2.705911947	5.20E-05	5767	6	299
NM_003467	0.2767532	-1.2245102	2.705911947	4.30E-05	5769	6	219
NM_003467	0.7932353	-1.4429491	2.705911947	0.000533726	5780	6	618
A_01_P004730	0.18472905	-0.907506	2.457902476	0.001327195	5153	6	199
A_01_P004730	0.09343925	-0.4372819	2.457902476	0.007287719	5152	6	351
XR_014707	-0.1266373	-1.4323516	2.71909386	4.03E-05	5760	6	213
XR_014707	-0.0510931	-1.6078825	2.71909386	0.000118529	5758	6	378
NM_001003927	-0.0865061	-1.3571339	2.378808674	0.000618156	1046	6	167
A_01_P005239	-0.5649187	-1.4997737	2.554005116	6.95E-05	5755	6	275
A_01_P005239	-0.4603879	-1.5944101	2.554005116	9.85E-05	5754	6	344
NM_016281	-0.5402423	-0.7181767	2.037084118	0.00653983	4847	6	251
NM_016281	-1.2276047	-1.1551526	2.037084118	0.006392391	5387	6	632
BU680450	-0.342351	-0.4163821	2.005923622	0.00899636	4849	6	288
BU680450	-0.150533	-0.1587461	2.005923622	0.013299132	4850	6	372
NM_054114	-0.1643893	-0.5449168	2.500147801	0.003825053	5150	6	233
NM_054114	-0.187852	-0.4597583	2.500147801	0.007420917	5151	6	354
NM_016030	-0.1293783	-1.4577305	1.941289018	0.005497322	154	6	806
NM_016030	-0.1410535	-0.9463327	1.941289018	0.006481551	4836	6	217
NM_018410	0.13338286	-1.1417396	2.285435206	0.008527546	1302	6	392
NM_018410	-0.035367	-0.7728462	2.285435206	0.008874008	1304	6	328
NM_004701	1.29511749	-1.105461	2.43037746	0.001295427	5281	6	819
NM_004701	-0.9774636	-0.8306842	2.43037746	0.003485803	75	6	800
NM_014498	-0.1489097	0.42543943	2.227918263	0.020336643	5043	6	650
NM_001250	0.14613756	-1.306883	2.936583526	9.99E-06	5770	6	320
NM_001250	0.16218183	-0.9367469	2.936583526	3.24E-05	5771	6	239
NM_000310	-0.5995584	-1.6664577	2.453324295	0.000574415	1047	6	452
NM_004591	-0.2030895	-0.3108381	2.955656604	0.007315386	5228	6	568
NM_004591	-0.3648126	-0.1077105	2.955656604	0.036390144	5223	6	774
NM_000607	0.29865795	-0.2197444	3.8643475	0.000908452	5266	6	792
AK127395	-0.5319636	-1.1680765	2.959506303	8.37E-07	5764	6	300
AK127395	-0.1673554	-1.4811592	2.959506303	0.000238754	5763	6	454
NM_006526	-0.9586516	-1.3737519	2.170686675	0.014998905	5875	6	708
NM_016184	0.33070568	-0.2309953	2.120801349	0.009618576	5049	6	467
NM_016184	0.54845145	-0.0829478	2.120801349	0.012287235	5050	6	567
NM_005779	1.05965423	-1.3445964	2.846509337	0.000406376	5244	6	712
NM_005779	0.97411399	-1.0018717	2.846509337	0.001043858	5245	6	681
XR_014077	-0.7550451	-0.2801542	2.320002078	0.00380948	4989	6	376
XR_014077	-0.5608033	0.15898779	2.320002078	0.006792307	4993	6	528
XR_014077	-0.6641957	0.28592386	2.320002078	0.008429124	4994	6	607
XR_014077	-1.1624783	0.18262353	2.320002078	0.010026934	4992	6	692
CB229722	-0.2383586	-1.47801	2.07849476	0.002631851	1040	6	371
NM_021244	0.39312754	-0.527404	2.938661848	0.000845096	5227	6	709

NM_021244	-1.0043252	-0.1608114	2.938661848	0.000466681	5209	6	679
NM_001237	-0.7859557	0.06219444	2.957839402	0.03307601	5187	6	803
NM_015609	-0.0027852	-0.7569618	2.810678219	0.009238426	5155	6	575
NM_015609	0.16272661	-1.2146431	2.810678219	0.006502384	5174	6	615
NM_004504	0.4144067	0.00222031	3.356200918	0.031957389	5235	6	822
NM_004504	-0.5051467	-0.3249061	3.356200918	0.04334779	5224	6	838
NM_001771	-0.0128441	-0.6241913	1.749103936	0.040206179	4854	6	538
NM_182898	-0.4072341	-0.3750486	1.876753684	0.030217296	4848	6	474
NM_152716	0.32748376	-1.1295057	1.718975121	0.02860257	4843	6	671
NM_013372	-0.3583201	-0.5420132	2.264285424	0.00726592	4851	6	272
XR_011982	0.16640855	-0.4672565	2.385670235	0.003464231	5046	6	232
XR_011982	-0.1695477	-0.5032475	2.385670235	0.008337874	4860	6	271
NM_199186	0.99865342	-0.8288591	3.055850306	0.008705956	5183	6	769
NM_199186	-0.5773002	-0.7575427	3.055850306	0.006301979	5186	6	724
NM_199186	-0.6165226	-0.6989184	3.055850306	0.007766379	5177	6	656
NM_020199	-0.3296524	-0.7847407	3.322255349	0.000589208	5226	6	605
XR_011199	0.3137548	0.27387899	2.675583944	0.006224398	5055	6	662
XR_011199	0.18217634	0.63953012	2.675583944	0.016504785	5056	6	755
NM_000559	-0.4637488	0.12981012	2.627926998	0.00329088	5053	6	525
CB230042	-0.0453559	-0.9829566	2.855291961	0.000365617	5768	6	258
CB230042	-0.275295	-0.6970328	2.855291961	0.000712198	5154	6	254
NM_018004	-0.2601379	0.17008587	2.249971395	0.032086183	5052	6	672
NM_001549	-0.037351	-0.8270193	3.918548646	0.000751972	5263	6	778
NM_001549	-0.2899367	-0.8155912	3.918548646	0.00065916	5262	6	771
NM_198797	-0.0872314	-1.093423	1.89755736	0.014583366	4835	6	451
NM_198797	0.09742202	-1.0945552	1.89755736	0.016725322	4837	6	508
NM_006286	0.27294893	0.32778528	2.275580002	0.045739296	5054	6	707
NM_020240	0.12542778	-1.1906757	2.849674041	0.016700143	5175	6	737
NM_020240	0.14678118	-0.8072121	2.849674041	0.022983166	5176	6	731
NM_021075	-0.7247832	-1.5725194	2.34813726	0.003485238	1033	6	613
NM_014445	-0.4474732	-1.0193911	2.055723709	0.016048674	1055	6	490
NM_004172	0.37770992	0.1841721	3.35522574	0.002732495	5237	6	760
NM_004172	0.77632742	0.25278857	3.35522574	0.002893891	5238	6	801
NM_004729	0.32198642	-1.6785356	2.31287753	0.002110086	1071	6	569
NM_002309	0.09503869	-1.299579	1.78478835	0.019429309	4831	6	629
NM_002309	-1.8779717	-1.0006836	1.78478835	0.029810737	231	6	847
NM_182757	-0.7931275	-0.9678536	2.171318996	0.013420074	5878	6	558
NM_182757	-0.3226433	-0.8998914	2.171318996	0.037662589	4834	6	645
NM_032192	-0.0995093	-1.2107954	1.756876088	0.028262186	4830	6	616
NM_020772	0.73213208	-0.8495358	1.884932249	0.027240243	4844	6	682
NM_020772	0.36688768	-0.7395734	1.884932249	0.030319001	4839	6	584
A_01_P002411	-0.3419959	-0.893601	2.406038047	0.009319167	4858	6	366
A_01_P002411	-0.2797456	-0.7131096	2.406038047	0.009076024	4859	6	301

CO646750	-0.1272901	-0.352942	2.366087047	0.007743906	5045	6	308
CO646750	-0.0027291	-0.055524	2.366087047	0.01548632	5047	6	500
NM_014220	-0.2595943	-1.5766912	2.969236861	8.97E-05	5761	6	484
NM_173853	0.2696502	0.00941313	2.476470397	0.00831213	5048	6	557
NM_173853	0.2569075	-0.2396671	2.476470397	0.028424428	5051	6	624
NM_002865	-0.2090114	-0.6920336	2.122533766	0.018786949	4855	6	402
NM_033631	-0.1478276	-0.8061434	1.825427743	0.041689861	4838	6	593
NM_004289	0.0589655	-1.4761555	2.36131381	0.002993692	1073	6	464
NM_004289	-0.4395418	-1.0869185	2.36131381	0.004557246	1049	6	480
AK131204	-0.4076332	-0.653409	2.624191752	0.025408317	5895	6	685
CN646485	0.05676243	-0.1762067	2.837902901	0.006418818	5230	6	562
CN646485	-0.0146269	-0.3553313	2.837902901	0.004540572	5229	6	481
A_01_P007942	-0.6762211	-0.990984	2.785694493	0.005932902	5225	6	611
A_01_P007942	-1.1093245	0.56362453	2.785694493	0.042507563	5210	6	810
NM_006803	-0.0563385	0.00771796	2.825311869	0.005106665	5233	6	599
NM_002575	0.15980338	-1.2091112	3.240638024	0.001884376	5826	6	652
NM_030643	0.10547081	-1.7770174	2.481223264	0.001940914	1072	6	586
A_01_P012169	-0.6278453	-1.1841015	2.177012628	0.012840983	1056	6	639
NM_000552	0.12895874	-0.6328946	3.931298265	7.03E-05	5265	6	761
NM_000552	0.14370399	-0.762475	3.931298265	5.67E-06	5264	6	753
NM_022718	0.58961683	-0.7622752	2.435462971	0.02335789	5240	6	690
NM_022718	0.88667216	-0.1171443	2.435462971	0.033040968	5241	6	780
NM_000186	-0.6714689	-0.2796424	2.144984485	0.044378708	4852	6	686
XR_013717	-0.5301656	-1.4260161	2.580547944	0.003662611	1048	6	533
NM_003449	-0.2352151	-1.4537624	1.965004623	0.02694147	1053	6	704
NM_021105	-1.382108	-1.1915469	2.315142807	0.011370926	5881	6	752
NM_006670	-0.1226834	-0.4223539	2.237490324	0.036239816	4857	6	612
NM_001007538	-0.2433036	-0.0004026	5.302568014	0.004506926	11355	6	869
CN648627	-1.345519	-1.4530509	2.482997695	0.012200128	5877	6	789
CN648627	-0.4744123	-1.0735836	2.482997695	0.008216415	5879	6	483
NM_138373	-0.2573913	-0.1002724	3.141723913	0.013347383	5231	6	715
NM_138373	-0.1965775	-0.0005151	3.141723913	0.014567236	5232	6	727
NM_005516	-0.045022	-1.4232394	3.151676574	0.000917906	5825	6	602
NM_005516	-0.1543298	-1.3577563	3.151676574	0.000805314	5824	6	583
NM_152329	0.07526776	-0.8969341	2.328294265	0.019735663	4856	6	542
A_01_P005403	-0.3990849	-0.3913113	2.229508409	0.038169766	4853	6	635
NM_014317	-0.2276679	-0.7995771	2.11715729	0.046502558	4841	6	659
NM_014317	-0.4195721	-1.0958323	2.11715729	0.049268347	4833	6	739
NM_138957	-0.6871498	-0.9852182	2.716960486	0.008328491	5880	6	633
NM_138957	-0.0663892	-0.7155147	2.716960486	0.006482195	4861	6	434
NM_013282	0.29302108	-0.6792344	3.194723701	0.031779618	5903	6	799
NM_014291	-0.2097105	-0.989704	2.083783955	0.038013341	4840	6	655
NM_014291	0.05351975	-0.7736094	2.083783955	0.048126468	4842	6	660

XR_014652	0.42936672	-0.7504289	2.28449476	0.029271861	5887	6	703
XR_014652	-0.6162094	-0.3186462	2.28449476	0.045902464	5882	6	813
NM_004414	0.29573258	-1.3557514	3.075839342	0.00365732	5838	6	691
NM_000917	-0.2492184	-1.5586206	2.924325797	0.006049762	5897	6	699
NM_000917	0.74384348	-1.6266779	2.924325797	0.0057745	5900	6	782
XM_496386	-0.0005959	0.12802785	3.134354481	0.004230141	5234	6	696
NM_006290	0.19816378	-0.9026123	3.950890063	0.002785181	5847	6	821
NM_000361	-0.7149261	-6.06E-05	3.61712446	0.025376746	5914	6	842
XR_010074	-0.2087073	-0.9972904	3.248322925	0.014064618	5901	6	764
XR_010074	0.22806867	-1.1237236	3.248322925	0.013551331	5902	6	775
NM_199129	0.12800002	-1.5724034	2.495536861	0.031287979	5896	6	808
NM_199129	-0.6978936	-1.2290825	2.495536861	0.033240632	5894	6	793
NM_032239	-0.41896	-0.3552668	3.10337584	0.048702765	5199	6	837
NM_005724	0.24448351	-0.1364577	2.857679756	0.048675292	5893	6	811
XR_012582	-0.9737261	-0.4363412	2.651650935	0.035292378	5883	6	765
NM_203463	-0.1287732	-1.4682839	2.64388279	0.031453164	5898	6	796
NM_203463	-0.0359572	-1.0225418	2.64388279	0.043734265	5899	6	772
NM_020412	-0.1418561	-1.0908013	3.623000773	0.000953261	5915	6	719
NM_020412	0.0541461	-0.544152	3.623000773	0.001313352	5916	6	728
NM_015184	0.26683729	-0.6242637	2.937134306	0.025329126	5239	6	767
NM_015184	0.49523503	-0.7709499	2.937134306	0.019998403	5888	6	750
NM_002810	-0.8163639	-0.8013765	3.687034608	0.01056031	5910	6	835
NM_002810	-0.9351273	-0.2198662	3.687034608	0.017682675	5913	6	843
NM_002810	-0.9914771	-0.3325182	3.687034608	0.01508065	5912	6	844
NM_002810	-0.7175603	-0.6427673	3.687034608	0.01277351	5911	6	828
NM_002810	0.14920012	-0.6395156	3.687034608	0.012587046	5904	6	818
NM_080593	0.35016008	-1.2329165	3.483940199	0.013994869	5839	6	834
NM_020437	0.81473142	-1.3601136	2.743171834	0.039240637	5279	6	858
NM_020437	0.54184463	-1.6011624	2.743171834	0.028666611	5886	6	831
XR_010049	-0.3873568	-1.1833879	3.29318527	0.013371988	5889	6	805
XR_010049	-0.4141836	-0.7757479	3.29318527	0.017818508	5890	6	784
NM_001955	-0.5019779	-0.5869377	4.375792779	0.001298975	5917	6	854
NM_001955	-0.3039436	-0.5274538	4.375792779	0.001400074	5918	6	848
CN479040	0.15963019	-0.7927	3.396220027	0.025424612	5892	6	823
CN479040	0.11645534	-0.8140713	3.396220027	0.031849956	5891	6	839
NM_006596	-1.7978073	-0.2869294	4.364645633	0.013457926	5874	6	864
AF184160	-0.5117428	0.20024688	4.318305729	0.033294863	11375	6	865
AF184160	0.11873119	-0.0747904	4.318305729	0.016581273	5919	6	860
NM_025108	1.46198214	-0.5870343	4.19138272	0.043879021	11376	6	870
NM_006952	0.10685334	0.13274792	5.022734757	0.046985736	11377	6	872

Cluster 5

**Gene Name**	**Av Normal**	**Av M (w12)**	**Av M (w4)**	***P***	**Hierarchical Clustering (order)**	**K-means clustering**	**K-means clustering (rank)**

NM_000193	0.51756555	1.41823726	-2.972454595	0.022407804	5979	5	1210
NM_201402	1.06385382	1.44408497	-2.650209434	0.020175502	5981	5	1204
CN647126	-0.7724888	0.59350755	-3.436807043	0.006860438	5966	5	1217
NM_024980	1.0045178	1.2076352	-3.258914289	0.002395522	5978	5	1192
NM_024980	0.41954343	1.35621	-3.258914289	0.001653483	5977	5	1178
NM_003862	-1.233085	-1.8865166	-4.050452933	0.002103417	7	5	1246
AK097951	0.4805349	1.46210398	-3.040515809	0.004828375	5980	5	1193
NM_032545	-1.4828172	-1.8842647	-4.942628871	0.038858525	6	5	1248
NM_000023	0.17537183	1.00937253	-2.221069739	0.029001703	6081	5	1125
NM_005297	-0.4613143	0.18651526	-2.629117153	0.011816638	5969	5	1181
NM_012101	0.04744892	1.62004792	-2.788994826	0.001425337	10935	5	1119
XR_010759	-0.5031444	-0.9884746	-3.122365894	0.009824917	5932	5	1228
NM_031921	-0.2491805	-1.1400902	-3.320481699	0.001576034	5931	5	1229
NM_004742	-0.2929889	0.3917047	-3.154118887	0.0021791	5967	5	1189
NM_004742	-0.2529092	0.49427009	-3.154118887	0.002736019	5968	5	1191
A_01_P011025	0.53283876	1.23954838	-2.341793168	0.009559318	6080	5	993
A_01_P011025	0.49529923	1.08912162	-2.341793168	0.00692012	6079	5	946
NM_152378	0.62121847	1.61049996	-1.876404024	0.020569623	6097	5	1087
NM_006772	0.52645399	1.22959254	-2.755515829	0.010126834	6075	5	1126
NM_005491	0.92435384	1.31454621	-1.764162695	0.025917518	6096	5	1063
NM_000336	1.59127359	1.34465838	-3.252354459	4.74E-05	5995	5	1201
NM_000336	1.63326855	1.2532698	-3.252354459	2.71E-06	5994	5	1202
NM_003520	0.45527254	0.99116902	-1.642208426	0.039088679	6085	5	979
NM_182686	0.1987843	-0.6186889	-3.774637921	0.002407597	5935	5	1226
NM_182686	-0.1888032	0.16812204	-3.774637921	0.003442637	8	5	1238
NM_030655	0.78638186	1.42301586	-1.912811976	0.014289528	6095	5	1007
XR_013912	0.79271193	0.02999542	-2.340332955	0.036289947	6070	5	1150
XR_013912	0.65445698	0.7974042	-2.340332955	0.013227125	6078	5	968
NM_020061	0.49548833	0.82880043	-2.407100669	0.015934193	6077	5	1011
NM_020061	0.48506305	0.75736138	-2.407100669	0.011561392	6076	5	959
XR_013236	-0.0440759	-0.0863775	-3.639411062	0.000367118	5936	5	1216
NM_001445	-0.2811995	0.7599844	-2.29985002	0.013273427	6069	5	1112
XM_379766	0.11138976	0.96740343	-3.089116984	1.50E-06	9766	5	1098
XM_379766	0.23059062	1.35289848	-3.089116984	2.71E-05	9767	5	1120
NM_014351	-0.4481698	1.36106332	-2.341710102	0.002373516	9756	5	1080
NM_152465	0.17839312	0.74011063	-1.795714987	0.018801043	6082	5	766
NM_152465	0.45111598	0.93298638	-1.795714987	0.018562734	6084	5	895
NM_182614	0.87841169	1.75709947	-2.700527403	0.000520812	10516	5	1099
NM_147161	1.80653889	1.49219575	-3.075849943	1.05E-06	5996	5	1205
NM_145912	0.67193386	1.57621702	-2.404042076	0.001222065	10515	5	916
XM_498188	-0.6701162	0.87290704	-2.033407674	0.005544726	9740	5	1032
XM_498188	-0.9083866	1.47104777	-2.033407674	0.003715793	9741	5	1143
NM_005462	0.61174841	0.99177655	-1.537538549	0.035180832	6094	5	950

NM_001996	2.68056301	1.84897894	-2.766393041	3.10E-05	11247	5	1225
NM_005106	-0.5606839	1.46507003	-3.058353287	6.62E-06	5972	5	1176
NM_020039	-0.2986477	1.15298369	-2.477784882	0.003880766	9831	5	1090
NM_020039	-0.3838576	1.20210396	-2.477784882	0.01009874	9830	5	1164
NM_006766	0.26140327	1.64076934	-2.810701308	1.00E-05	10449	5	1035
NM_173587	0.23390305	0.99155295	-2.779286627	0.00022907	9768	5	996
NM_152479	1.11098725	1.68813918	-2.997088553	0.000156199	10457	5	1162
NM_178525	-0.1045422	-0.5166902	-2.552546352	0.017767477	5947	5	1194
NM_178525	-0.5485503	-0.1619132	-2.552546352	0.001891878	5946	5	1186
NM_152698	-0.4456989	-1.1661904	-2.584776798	0.020106285	5930	5	1220
NM_031294	0.61931454	1.75917447	-2.429085976	0.002918061	10521	5	1037
NM_031294	0.56728826	1.80835114	-2.429085976	0.000369845	10523	5	941
NM_001519	-0.3176537	-0.0587377	-2.95461556	3.93E-05	5941	5	1180
NM_001519	-0.3800124	0.387064	-2.95461556	3.17E-06	5942	5	1155
NM_003955	1.44839791	1.31346243	-2.433033219	0.009442516	10531	5	1159
NM_020353	-0.051871	0.70443724	-2.554979715	3.42E-05	9769	5	829
NM_020353	-0.0532999	0.84339247	-2.554979715	3.25E-05	9770	5	791
XM_496422	0.76892961	1.34063985	-2.461512583	0.004143081	10490	5	965
XM_496422	0.76784895	1.56078179	-2.461512583	0.001315438	10491	5	944
NM_004790	0.46523395	0.72729225	-2.001604175	0.007458852	6243	5	510
NM_004790	0.62482678	1.46389805	-2.001604175	0.015336619	11130	5	1002
NM_181710	-0.0788584	-0.9750583	-3.170271395	0.048160533	5934	5	1227
NM_181710	-0.3374713	-0.8174683	-3.170271395	0.013267446	5933	5	1219
NM_013271	0.87010152	0.81163387	-2.721342912	0.006961419	6074	5	1091
NM_013271	0.58076159	1.01008438	-2.721342912	0.004122198	6073	5	1026
NM_032326	0.86692599	1.45378497	-2.670663424	0.000596831	10494	5	1017
NM_032326	0.72248554	1.80618049	-2.670663424	0.000838343	10520	5	1079
NM_019593	-0.0127688	1.11417657	-2.652326883	0.000532619	9774	5	1004
NM_019593	-0.1001823	1.48673571	-2.652326883	4.35E-05	9777	5	997
NM_005586	0.38844748	0.87420762	-1.782621891	0.011105848	6246	5	405
NM_005586	0.41091721	0.92884287	-1.782621891	0.007962152	6086	5	381
NM_053017	-0.147635	1.0410398	-2.601788018	0.000786147	9773	5	1012
NM_000030	0.96550114	1.89254282	-2.280475878	0.002992359	10529	5	1086
NM_012309	0.33167791	1.44293224	-2.555206389	0.002947179	9836	5	1083
CN644322	0.14425135	1.44765294	-1.632530628	0.008940448	6104	5	689
CN644322	-0.1576913	1.71471422	-1.632530628	0.010012785	6103	5	1006
NM_032818	0.69150437	1.93547518	-1.964082445	0.000470055	10585	5	888
NM_006783	0.93759338	1.30181593	-1.88293888	0.036348148	6381	5	1127
NM_024671	-0.4939565	-1.5846523	-2.771631969	0.007967477	5929	5	1233
XM_376681	0.35599057	1.0952453	-2.173420385	0.006070359	6223	5	605
XM_376681	0.4381512	1.13184168	-2.173420385	0.00160043	6226	5	358
NM_138286	0.14198688	1.57035653	-2.102848374	0.039376293	6003	5	1179
NM_002147	-0.2380752	1.78695282	-1.98393035	0.00107345	9761	5	990

NM_005167	0.68480798	1.48704849	-2.235749536	0.000239134	10501	5	549
NM_033513	0.86536544	1.55044968	-2.388917266	0.002507957	10493	5	976
NM_033513	0.86308827	1.24384304	-2.388917266	0.000554894	10496	5	711
XR_011978	0.21250755	1.02114551	-2.418779964	0.00032203	9776	5	724
XR_011978	-0.1151623	1.13343393	-2.418779964	0.000411208	9775	5	936
XR_012542	0.3577885	1.12996898	-1.823176128	0.006117117	6087	5	395
NM_003253	2.96138799	0.79436658	-2.751292143	0.007564811	11241	5	1234
NM_003253	3.01310147	1.7886254	-2.751292143	0.002484601	11245	5	1239
A_01_P001826	0.8481234	0.66866766	-2.387418004	0.012116963	6071	5	984
A_01_P001826	1.25152174	1.32056113	-2.387418004	0.000458642	5992	5	1071
NM_003085	0.99971704	0.53968906	-1.742030972	0.011471945	6187	5	841
NM_003085	0.73321716	0.88870253	-1.742030972	0.010267092	6249	5	504
NM_001004067	1.06963414	1.57484433	-2.117946794	0.014826924	10513	5	1115
NM_001004067	1.07560997	2.02219348	-2.117946794	0.001168193	10530	5	1077
NM_173651	0.30571394	1.91479089	-1.667593047	0.001661369	10586	5	880
NM_017797	0.3826972	1.0372647	-2.515587055	0.004274057	6072	5	879
NM_017797	0.90092841	1.32484258	-2.515587055	0.000186577	10495	5	838
NM_004349	0.25017451	1.58921224	-2.093931878	0.00324185	9765	5	744
NM_004349	0.33231684	1.44991644	-2.093931878	0.004417125	9764	5	719
NM_033553	0.90532023	1.05905289	-2.352669883	0.003472321	5987	5	917
NM_033553	0.95101683	1.0309028	-2.352669883	0.004900815	5988	5	988
NM_030567	-0.5110843	-0.6015625	-2.352846524	0.004518706	5945	5	1200
NM_014476	-2.2244331	1.22353386	-1.882579236	0.001192145	5924	5	1242
NM_080861	0.0141027	1.70776231	-2.456785288	1.47E-06	9782	5	871
NM_080861	0.01877728	1.67052261	-2.456785288	1.12E-06	9781	5	862
NM_144616	0.77823146	0.3507253	-1.527189855	0.036729457	6179	5	940
NM_205545	0.99774846	0.54206018	-2.29718948	0.026069673	6130	5	1093
NM_205545	0.8709391	0.67743593	-2.29718948	0.023820665	6131	5	1055
NM_017877	0.82570322	1.15839588	-1.80303392	0.004339416	6250	5	535
NM_017877	0.38560527	1.25135237	-1.80303392	0.004179995	6248	5	260
NM_018996	1.02512158	0.72299523	-2.279194669	0.002438648	6136	5	846
NM_018996	0.59606978	0.83520366	-2.279194669	0.002472789	6222	5	532
NM_014380	-0.0345945	1.70671565	-1.83613151	0.002923391	9853	5	930
NM_024768	-0.4041051	-0.3966377	-2.861960036	0.000641291	5940	5	1207
NM_000207	0.67388755	1.48116173	-2.028574524	0.019054529	10510	5	1036
NM_000207	0.6178467	1.0573863	-2.028574524	0.007079952	5989	5	781
NM_001012270	0.26682423	0.73025638	-1.671288956	0.006286495	6083	5	361
NM_002751	0.38938829	0.30072939	-2.168882382	0.000252857	6682	5	824
NM_002751	-0.1079018	0.74794684	-2.168882382	0.000148774	6704	5	467
NM_002751	-0.1369564	0.83174869	-2.168882382	8.48E-05	6705	5	443
NM_004166	0.34190104	1.6921925	-2.033980943	0.008074915	10509	5	943
NM_004166	0.7583702	1.72050708	-2.033980943	0.001376524	10502	5	742
NM_033520	-0.2765847	1.51418661	-2.214253402	4.77E-05	9778	5	701

DQ148132	-0.1919102	0.91306812	-1.604869211	0.008943766	6741	5	732
NM_001286	2.73905166	1.57219385	-2.763350521	0.005223809	11244	5	1232
NM_001286	2.78656303	1.44738665	-2.763350521	0.011155692	11243	5	1237
NM_001286	2.62125882	1.55453174	-2.763350521	0.014325465	11242	5	1235
NM_012183	0.27852599	0.94677355	-1.742452745	0.003558922	6247	5	115
NM_001501	0.04571193	0.8940562	-1.857673501	0.003503681	6198	5	900
NM_000515	0.2305083	0.77882829	-1.715259624	0.030056081	6099	5	807
NM_003325	0.28532774	-0.0852608	-2.473007446	0.007456618	5959	5	1145
NM_003325	0.48704039	0.02071078	-2.473007446	0.007281369	5960	5	1124
XR_014263	1.45267172	1.51510724	-2.354808911	0.004745068	10532	5	1147
XR_014263	1.30039516	1.86686923	-2.354808911	0.004966399	10533	5	1165
NM_024552	-0.6962743	-1.6274314	-3.009939885	0.015460535	5928	5	1241
XR_012838	0.06820521	0.83663803	-2.130066742	0.003639085	6737	5	741
NM_000691	0.39423611	1.11025029	-2.244457141	0.000433094	6225	5	201
NM_000691	0.14901391	1.3038392	-2.244457141	0.000493553	6224	5	339
NM_152399	0.00941733	1.06329059	-1.961818129	0.00060872	6742	5	319
NM_152399	-0.0197224	0.792285	-1.961818129	0.00873431	6738	5	869
XR_011853	1.05653736	1.69921919	-2.411466792	0.000452541	10505	5	1025
XR_011853	1.0895713	1.61379314	-2.411466792	0.000272505	10504	5	989
NM_144645	1.15292562	0.03392228	-2.339741632	0.038842146	6127	5	1171
NM_144645	1.02604419	0.36678211	-2.339741632	0.006184346	6132	5	1031
NM_198537	0.70955412	1.1460496	-2.321310176	0.002887618	10489	5	681
NM_198537	0.95236531	1.84494751	-2.321310176	0.001235164	10528	5	1039
A_01_P016330	1.14184535	0.85357315	-2.190852485	0.024304986	6158	5	1100
NM_144622	0.65276082	0.9125005	-1.540745181	0.043126908	8682	5	897
NM_203471	0.17377066	0.66491046	-1.71282276	0.011618722	6244	5	391
NM_203471	0.59954466	1.21363392	-1.71282276	0.005319456	6275	5	297
NM_203471	0.78687504	2.03932249	-1.71282276	0.007593156	10596	5	1084
NM_203471	0.23694054	1.22522711	-1.71282276	0.001163784	6090	5	101
A_01_P010554	-0.2530856	-0.4718851	-2.118056506	0.006193495	5948	5	1167
A_01_P010554	-0.334868	-0.2970054	-2.118056506	0.00062321	5949	5	1148
NM_014370	-0.0084705	1.75092397	-2.31838146	0.008973112	6002	5	1134
NM_001106	0.45902606	1.56956415	-2.269341836	1.58E-05	9809	5	562
XR_012049	-0.1410036	1.74213067	-2.150952335	0.000102137	10134	5	858
NM_018646	-2.6601064	-0.1346	-4.488012281	0.030553905	5	5	1249
NM_021044	-1.0285942	-0.0583543	-2.535422752	0.011170427	5938	5	1206
NM_178829	0.86864896	1.18240674	-2.795635119	0.000238689	10488	5	1028
XR_011748	0.82378701	0.27441366	-1.631041546	0.032107499	6184	5	928
XR_011748	0.76256874	0.66705563	-1.631041546	0.019070461	6185	5	617
NM_198943	-0.4497165	1.29162543	-2.31626978	2.23E-06	9746	5	820
NM_198943	-0.5469181	0.96110851	-2.31626978	5.25E-05	9744	5	947
NM_198943	-0.3168345	1.31880802	-2.31626978	5.14E-08	9747	5	706
NM_198943	-0.4174235	1.25805287	-2.31626978	2.37E-05	9745	5	882

NM_152422	-0.0008486	0.36604486	-2.012942936	0.000305974	6698	5	593
NM_152422	-0.299795	0.71199816	-2.012942936	0.005695931	6402	5	932
BM423312	0.8397767	0.60304556	-2.139930689	0.010012312	6134	5	809
BM423312	0.97163617	0.6466177	-2.139930689	0.008989266	6135	5	866
NM_003949	0.57415055	1.70600657	-1.832827873	0.025708306	11131	5	1114
NM_003949	0.41506925	1.30620052	-1.832827873	0.000516187	6092	5	136
NM_198488	0.44818482	1.15874473	-2.186903896	7.40E-05	6228	5	112
NM_005269	-0.9440369	1.00210384	-1.993905012	0.001786769	6039	5	1173
NM_020926	0.36686238	1.67035696	-2.378906896	0.000107613	9806	5	854
NM_020926	0.47571826	1.60917123	-2.378906896	0.000241813	9807	5	878
NM_021211	-0.0002237	1.54824425	-2.059952584	0.000945949	9801	5	801
NM_173846	1.84249544	1.33916003	-2.304641821	0.00050808	6000	5	1187
NM_152492	1.14944955	0.37670234	-2.015609717	0.024682281	6133	5	1056
NM_152492	1.25518877	0.72294762	-2.015609717	0.006847609	6137	5	949
NM_001010972	0.51032343	0.72218041	-2.276286529	1.22E-05	6697	5	270
NM_001010972	0.22256406	0.62575199	-2.276286529	2.13E-06	6694	5	347
BC043401	-0.9599246	0.32228843	-1.828771922	0.001363055	6394	5	1130
BC043401	-0.7249464	0.3530768	-1.828771922	0.004860375	6395	5	1097
NM_000033	0.06694553	1.00874328	-1.923813222	0.000680033	6743	5	332
AF038185	0.82228566	1.40138546	-1.986535658	0.006756818	10511	5	762
AF038185	1.58549709	0.45029505	-1.986535658	0.000875273	10864	5	1213
AF038185	0.42798035	0.39080971	-1.986535658	0.000415281	6683	5	697
AF038185	0.76451763	1.32550939	-1.986535658	0.002527619	6221	5	683
NM_144565	0.96092118	1.93309646	-1.570256346	0.00199814	10594	5	987
NM_020982	0.74643355	1.44691118	-2.284249009	0.002747782	10492	5	799
NM_020982	0.75633577	1.46911012	-2.284249009	0.000228977	10499	5	587
NM_022467	0.2144954	0.81685883	-1.712864593	0.01110577	6245	5	280
NM_022467	-0.1218284	0.69545303	-1.712864593	0.003325674	6907	5	368
NM_001715	0.61333747	0.41785167	-1.301884538	0.042982388	6177	5	927
NM_033068	1.94241963	-0.1426297	-2.339826424	0.023774192	10860	5	1214
NM_033068	2.03546902	0.33353886	-2.339826424	0.004441671	10861	5	1199
XM_375633	0.86737349	0.36448926	-2.255148276	0.00440905	6126	5	1078
NM_173462	0.02120291	1.01970925	-1.709528623	0.000804323	6088	5	117
NM_005909	0.37188651	1.20548273	-2.285044035	6.84E-06	9805	5	406
NM_005909	0.44255929	1.57109657	-2.285044035	0.00043515	9808	5	800
NM_139205	-0.0631247	0.98549884	-1.529201709	0.003499252	6089	5	300
NM_139205	-0.0693287	1.9900375	-1.529201709	0.00571542	6105	5	1038
NM_015897	0.82642265	1.61334666	-1.822741017	0.031519126	6382	5	1144
NM_015897	0.99570052	1.70393978	-1.822741017	0.025774547	6384	5	1175
NM_145248	0.76564068	1.54215475	-3.527759505	0.023426941	11382	5	1230
DR771020	-0.5949431	0.87845674	-1.940621134	0.000303362	9749	5	805
DR771020	-0.5476165	0.8494564	-1.940621134	0.000185012	9750	5	793
NM_005354	0.26593112	0.57570711	-2.164025543	1.35E-05	6695	5	344

NM_005354	0.3010227	0.60584691	-2.164025543	1.83E-05	6696	5	305
NM_001040	-0.0346448	1.14817863	-2.175544901	0.008591961	9762	5	967
NM_001040	-1.3783244	1.51350556	-2.175544901	0.021348799	10883	5	1218
NM_001525	-0.7564958	0.47836892	-2.784760541	0.008570562	5939	5	1203
NM_001525	-0.3826725	-0.0899239	-2.784760541	0.005580533	5943	5	1198
NM_080825	0.03841475	0.1494528	-2.128518379	0.000826508	6684	5	877
NM_080825	-0.0292418	0.33592911	-2.128518379	0.000465717	6685	5	739
NM_006533	0.03404132	1.15528325	-2.071960968	0.000827881	6227	5	188
NM_006533	-0.1634017	1.34232824	-2.071960968	9.80E-06	9787	5	336
XR_014590	-0.1749339	1.42424731	-1.996821632	0.000188562	9790	5	454
XR_014590	-0.038963	1.22546398	-1.996821632	0.000145683	9789	5	218
NM_030806	0.49755544	1.72418509	-2.841804073	0.009338677	10442	5	1183
NM_021805	-0.0636962	1.5556306	-2.288100251	7.34E-07	9779	5	599
NM_021805	-0.0640327	1.55612226	-2.288100251	2.60E-07	9780	5	600
NM_020064	1.58953798	-0.4604348	-2.027743554	0.048833105	10859	5	1208
NM_020064	0.74141536	0.02605521	-2.027743554	0.012811021	6166	5	1065
NM_152891	0.90851636	1.61708419	-1.912796168	0.003042377	10512	5	786
NM_019057	-0.1673593	1.70459848	-2.235611009	0.001154117	9758	5	910
NM_014419	1.09334884	0.76417263	-2.464439471	0.006627967	6129	5	1049
NM_014419	1.06323225	1.40570284	-2.464439471	0.000491653	10503	5	957
NM_182553	0.8706431	0.83912361	-1.722535564	0.022111492	6290	5	746
NM_182553	0.9785946	0.96834407	-1.722535564	0.018073517	6291	5	794
NM_153368	0.89307784	1.13692483	-2.198650465	7.16E-05	10497	5	385
NM_153368	1.05154826	1.73258556	-2.198650465	5.16E-06	10506	5	892
NM_015726	-0.0451339	1.36313693	-2.076172166	4.72E-06	9788	5	254
NM_015726	-0.1099502	1.21320509	-2.076172166	2.78E-05	9785	5	292
NM_001702	0.76982973	1.76703946	-1.54919636	0.007078987	10553	5	982
NM_001702	0.55122211	1.81461228	-1.54919636	0.00687645	10552	5	971
NM_032389	-0.086967	0.76295542	-1.95961581	3.41E-05	6706	5	189
NM_032389	-0.1174597	0.89304289	-1.95961581	2.68E-05	6707	5	149
NM_017556	0.2357941	0.81833803	-1.068253112	0.041994841	6117	5	899
NM_020897	0.98232211	1.46995906	-1.874967835	0.02199158	11132	5	1096
NM_020897	0.78779362	0.93331117	-1.874967835	0.000834933	6220	5	581
NM_024757	0.33120992	1.73326733	-1.940997099	5.09E-05	9818	5	450
NM_147133	0.01720706	0.99490998	-2.425850495	6.61E-05	9771	5	564
NM_147133	0.12931801	1.16838391	-2.425850495	3.97E-05	9772	5	580
NM_000850	-0.6379256	1.08901659	-2.00812778	0.000180339	9752	5	818
NM_000850	-0.6341911	1.09658386	-2.00812778	8.21E-05	9753	5	804
XR_014158	0.97704613	0.24678404	-1.974505394	0.0113154	6139	5	958
NM_003812	-0.0531029	0.8308128	-2.926012323	0.015489122	5973	5	1184
NM_003812	0.30908215	1.23461771	-2.926012323	0.0071225	5976	5	1169
NM_032529	-0.1015328	0.55998557	-1.309816592	0.025126652	6909	5	857
NM_025158	0.18940741	0.97716224	-2.050907922	0.000546827	9798	5	293

NM_025158	0.09395237	1.32530191	-2.050907922	0.00015116	9802	5	343
NM_032207	-0.2225047	1.69523765	-1.881968136	0.000215494	6961	5	686
NM_032207	-0.2358279	1.47714827	-1.881968136	0.000130718	9791	5	550
NM_145733	0.48830976	1.18413619	-1.631925453	0.000576409	6091	5	21
NM_145733	0.92712714	1.69367572	-1.631925453	0.000760765	10591	5	813
NM_015065	-0.222949	1.2784621	-1.79789235	0.000476116	6955	5	489
NM_015065	-0.2740206	1.47355765	-1.79789235	0.011716915	9861	5	1092
U87259	0.22917226	1.5907006	-2.768624069	0.000249114	10444	5	1064
U87259	0.76658971	1.30022475	-2.768624069	0.000876539	5986	5	1123
NM_182528	0.37443733	1.10568961	-1.99885085	0.00358829	6202	5	597
NM_006615	-0.7399676	1.72257885	-1.681171928	0.002613241	9734	5	1101
NM_007056	-0.3165031	0.92773462	-2.004721465	1.69E-05	9751	5	448
NM_007056	0.00695605	0.84357628	-2.004721465	3.69E-05	6708	5	140
NM_004991	0.02617615	1.25784566	-2.043520437	2.94E-05	9792	5	155
NM_004626	1.48838534	0.71999448	-1.620010396	0.012663798	6157	5	1047
NM_014587	0.15531264	1.32407209	-1.608009495	0.002417166	10544	5	182
NM_014587	-0.1681108	0.94236569	-1.608009495	0.000638351	6886	5	230
NM_014619	0.85791743	0.07599664	-1.561274386	0.040728825	6181	5	1015
NM_014619	0.86656888	0.29458323	-1.561274386	0.008526726	6182	5	782
NM_016541	0.88054119	1.3576258	-2.083788749	0.001193731	10500	5	521
NM_016541	0.83970239	1.12378999	-2.083788749	0.000192499	10498	5	215
NM_213726	0.60870836	1.22204391	-2.193501246	0.001955391	6208	5	447
NM_213726	0.66130503	1.4537412	-2.193501246	0.001989017	6209	5	646
NM_170692	0.21251292	1.08420442	-1.793335876	0.001423486	6238	5	28
NM_170692	0.27343197	0.70771871	-1.793335876	0.003329084	6740	5	412
NM_006594	-0.7536698	0.2973098	-2.011009058	0.001856224	6397	5	1122
NM_006594	-0.6710129	0.38379251	-2.011009058	0.004106596	6401	5	1121
NM_031205	1.1476362	0.34784801	-1.336607312	0.022460374	6188	5	1020
NM_005608	0.57070368	0.84040394	-1.671545886	0.016408576	6266	5	465
NM_005608	0.86965322	2.03267968	-1.671545886	0.003935738	10598	5	1048
NM_014430	0.57080887	0.30368497	-1.342950632	0.02430741	6173	5	780
NM_014430	1.29787023	0.31117383	-1.342950632	0.024025729	6189	5	1089
NM_005232	0.24605166	1.75005019	-2.270621964	0.000170141	9828	5	860
A_01_P011265	0.65611903	1.44068247	-2.02458698	0.014051593	10550	5	998
A_01_P011265	0.8673534	1.4545675	-2.02458698	0.005092886	10551	5	918
NM_016569	-0.1499059	1.46231209	-2.192202714	0.000465905	9796	5	817
NM_152733	0.60233411	0.38922127	-1.998080974	0.00627331	6138	5	649
NM_152733	0.65493688	1.14770487	-1.998080974	0.008839875	6203	5	733
NM_207414	0.49252964	0.32058017	-1.547731405	0.011030829	8626	5	569
NM_207414	0.44582046	1.81041532	-1.547731405	0.001738194	10566	5	653
NM_004502	1.04437893	1.8442399	-1.715065287	0.004977984	10597	5	1018
NM_181353	0.25327352	1.0176739	-1.781629854	0.001979167	6744	5	307
NM_014884	-0.1734554	1.16505656	-2.176868311	0.002195042	9794	5	872

NM_014884	-0.2945778	1.23168753	-2.176868311	0.00132637	9795	5	922
NM_012345	0.02261108	-0.4317509	-1.917129283	0.005566625	5957	5	1137
NM_014801	0.13810662	0.44405796	-1.370261956	0.028994664	8619	5	709
NM_014801	0.45062201	0.44984497	-1.370261956	0.01618644	6178	5	704
NM_014555	0.07178504	1.42374489	-2.036200886	5.01E-05	9793	5	204
NM_014555	0.39249074	1.85430969	-2.036200886	2.17E-06	9815	5	584
NM_006688	0.40561398	0.92695343	-1.973876438	0.005019661	6231	5	213
NM_006688	0.35804993	1.15795762	-1.973876438	0.001326408	6237	5	54
NM_005581	-0.4149943	1.28106621	-1.304913318	0.044718765	6098	5	1104
NM_005581	0.30571093	1.72793314	-1.304913318	0.013147105	10546	5	964
NM_024307	0.07679608	1.74957183	-2.072214899	8.85E-06	9814	5	536
NM_001004450	0.47932648	0.20860023	-1.490593158	0.016602252	6172	5	753
NM_001004450	0.67468986	0.40351848	-1.490593158	0.01482244	7312	5	844
NM_021926	0.73133348	0.90215407	-1.609344877	0.01169507	6278	5	393
NM_021926	0.96460548	1.25726961	-1.609344877	0.00730364	6287	5	650
XR_009827	-0.267746	0.70412209	-1.932959187	0.000487423	6876	5	499
XR_009827	0.10783965	0.29825625	-1.932959187	0.001339324	6710	5	703
NM_001586	0.29885232	1.32129073	-1.363383069	0.021591182	10545	5	763
NM_001586	0.74136021	1.10914016	-1.363383069	0.00289028	6304	5	348
NM_176875	0.77511834	1.8541691	-2.042610581	0.000418316	10526	5	853
NM_176875	0.85560503	1.7990654	-2.042610581	1.95E-05	10589	5	792
NM_001007156	0.115572	0.52199741	-2.046928554	0.00018131	6693	5	354
NM_001007156	0.0328646	1.02546696	-2.046928554	0.00106028	9797	5	480
NM_005686	-1.1435779	1.0182771	-1.741609436	0.006954191	6007	5	1158
NM_005686	-0.7764892	0.65572539	-1.741609436	0.000222511	9748	5	995
NM_005654	-0.1055738	1.25818094	-1.95537165	6.01E-06	9786	5	178
NM_005654	-0.0163676	1.52968294	-1.95537165	3.89E-07	10142	5	267
NM_003922	-0.4959799	1.13011125	-1.711882505	0.002124543	6644	5	907
NM_003922	-0.536545	1.51709785	-1.711882505	0.001497325	9864	5	1005
NM_024007	1.24412874	1.06896836	-2.330559058	0.009566221	5993	5	1117
NM_182964	0.2343847	1.70347228	-1.845404008	0.000473078	9940	5	661
NM_007148	0.02626067	0.32056183	-1.82909843	0.011370651	6403	5	938
NM_007148	0.0454424	0.60961165	-1.82909843	0.009439494	6739	5	779
NM_015981	0.30530693	1.19533927	-1.657808497	0.000333938	6942	5	81
A_01_P007828	0.86762855	1.10045713	-1.740466445	0.000219866	6258	5	126
A_01_P007828	0.87940331	1.06050566	-1.740466445	0.000234728	6257	5	152
NM_025198	-0.1892423	1.69286081	-2.16354633	4.77E-05	9783	5	825
NM_000937	0.62303716	1.71555831	-2.330293715	0.007640739	10507	5	1076
NM_019003	-0.6528385	1.67532613	-1.780765562	0.000146466	9863	5	1033
XR_010104	0.92589026	1.23705248	-1.562693421	0.006235251	6283	5	539
XR_010104	0.75530856	1.18131302	-1.562693421	0.004101237	6282	5	251
NM_003877	0.50395509	1.50347503	-2.014484026	0.000222342	9811	5	444
NM_153488	0.60246313	0.69848367	-1.875512208	0.004578324	6230	5	227

NM_153488	0.35899056	0.63779327	-1.875512208	0.002722159	6229	5	195
NM_019858	0.20006135	1.73454364	-1.744287532	0.000121241	6965	5	362
NM_019858	-0.0348954	1.56872163	-1.744287532	2.49E-05	6964	5	247
NM_024682	0.40608901	0.92016041	-1.693033505	0.008747256	6267	5	141
NM_024682	0.50533798	1.26210423	-1.693033505	0.007543806	6269	5	291
NM_153832	-0.1871011	0.8013719	-1.538210321	0.00036652	6885	5	239
NM_153832	0.05207766	1.32245206	-1.538210321	0.00604408	6100	5	490
NM_024333	0.55573178	1.08756935	-1.619168733	0.00628585	6268	5	143
NM_024333	0.08555002	0.8893943	-1.619168733	0.002800935	6235	5	62
NM_145297	0.14093228	0.66361638	-2.188485774	0.002415711	6689	5	883
NM_173674	1.42376558	1.20366441	-2.507632389	0.007231645	5998	5	1168
NM_017451	0.19810581	1.52353432	-1.91068474	0.000146676	9817	5	320
NM_017451	0.03847809	1.84765676	-1.91068474	0.000262984	9827	5	934
CN645828	0.67044668	0.62633098	-1.283915218	0.048321761	8683	5	833
NM_005463	-0.3505931	0.03294539	-2.045603311	0.002274137	6398	5	1075
NM_005463	-0.1271135	-0.1071902	-2.045603311	0.02298159	5958	5	1139
NM_182538	0.72855892	0.16664881	-1.263038856	0.037225115	6174	5	966
NM_015308	-0.2964954	1.66669876	-1.844353914	7.98E-06	6962	5	699
NM_178835	0.06313951	0.78528603	-1.697554966	0.000294457	6894	5	71
NM_178835	-0.1355525	1.27070667	-1.697554966	0.000626694	7018	5	416
NM_002043	0.41927215	1.64801964	-2.01344783	0.000757028	9849	5	725
NM_002002	0.48932321	0.53760342	-1.5053759	0.00791654	6176	5	469
NM_002002	0.14855549	1.2484178	-1.5053759	0.002780059	7090	5	324
NM_013343	-0.621477	1.28753827	-2.208072971	0.00106344	9755	5	1069
NM_013343	0.6653461	1.2462256	-2.208072971	0.002259203	5990	5	845
XR_011927	0.19775002	1.52333685	-1.774087956	0.00110586	9932	5	551
XR_011927	-0.0462547	1.40387564	-1.774087956	0.006042239	10378	5	929
NM_058175	-0.4923157	1.07824991	-2.305758461	0.000434416	9743	5	885
NM_058175	-0.5670302	1.07056273	-2.305758461	0.000215176	9742	5	955
NM_178453	-0.0569716	0.93886593	-1.512434319	0.001400364	6905	5	295
NM_004177	-0.0122003	1.73479591	-1.981426372	3.03E-05	10144	5	639
NM_004177	-0.1240569	1.7094742	-1.981426372	8.13E-05	10143	5	720
NM_178448	0.41549334	1.9403734	-1.967673715	0.000319841	10475	5	728
NM_015528	0.10213803	0.44198522	-1.865456596	0.000374066	6701	5	466
NM_015528	0.08906402	0.55792149	-1.865456596	0.000360116	6702	5	349
NM_003456	-0.0283885	0.75978082	-1.920770174	5.56E-06	6709	5	133
NM_003456	-0.0567415	0.57548294	-1.920770174	0.000527217	6700	5	462
NM_145056	0.05522743	1.63847987	-2.068614981	0.000152042	9784	5	526
NM_145056	0.33494527	1.62996036	-2.068614981	0.000351928	9812	5	626
XM_370946	-0.1555439	1.50120697	-1.655429453	0.001829442	6957	5	685
XR_013836	0.51274493	1.25522046	-1.415763314	0.015559861	8674	5	548
XR_013836	0.47338609	1.16402609	-1.415763314	0.009302238	8672	5	272
NM_022553	1.04112182	1.01914689	-1.688697499	0.0001723	6259	5	287

NM_022553	1.36741372	1.0773907	-1.688697499	0.000225304	6264	5	790
NM_015417	0.65582382	0.98596299	-1.709675819	0.00286319	6271	5	83
NM_015417	0.57172437	1.05171768	-1.709675819	0.000725729	6270	5	11
NM_005455	0.22856397	1.56108117	-2.146302972	0.005681589	9837	5	1052
NM_033036	0.59545416	1.1708579	-1.792758211	0.000667108	6273	5	27
NM_033036	1.02437703	1.20873854	-1.792758211	0.00023008	6260	5	337
NM_004714	0.01008067	0.07925153	-1.568828878	0.002772937	6470	5	849
NM_004714	-0.2588425	0.32559618	-1.568828878	0.002145636	6468	5	710
NM_032019	0.66974918	1.45153135	-1.663005787	0.000145078	6312	5	183
NM_032019	0.89849142	1.71949762	-1.663005787	4.44E-05	10590	5	696
NM_021255	-0.5129032	1.37801741	-1.5567089	0.006222129	9732	5	1103
NM_021255	0.6058174	1.46016168	-1.5567089	0.000199469	6093	5	237
NM_015385	-0.1201931	1.25439597	-1.564007011	0.000174558	7086	5	386
NM_003573	-0.3649462	1.36343892	-1.796623934	0.001141894	6958	5	561
NM_003573	-0.3459684	1.69984444	-1.796623934	0.001073216	6959	5	803
NM_145283	0.871626	-0.3338141	-1.602339696	0.033712447	6167	5	1132
NM_145283	1.0764422	0.47295924	-1.602339696	0.008250255	6186	5	784
NM_175885	0.74394153	1.1045531	-1.49919906	0.010589921	6281	5	417
NM_175885	0.89535226	1.37418798	-1.49919906	0.003835557	6284	5	517
NM_002740	1.54774834	1.07254276	-1.349236396	0.01533829	6379	5	1088
NM_002740	1.47426842	1.49973682	-1.349236396	0.009889771	6380	5	1110
NM_021569	0.81364397	0.28204884	-1.828345513	0.025473831	6140	5	919
NM_021569	0.87392489	0.38720155	-1.828345513	0.026753414	6141	5	912
NM_021569	0.81306852	0.45476678	-1.828345513	0.012399491	6142	5	694
NM_021569	0.86876224	0.66745871	-1.828345513	0.014436014	6147	5	676
NM_021569	0.84934379	0.52320624	-1.828345513	0.011888483	6146	5	675
NM_021569	0.93454602	0.47355281	-1.828345513	0.004875408	6145	5	637
NM_033312	0.78633554	1.30722338	-2.815931511	0.002597476	5985	5	1156
NM_016437	0.95433088	1.62416568	-1.318398162	0.002281909	10593	5	835
NM_016437	0.7851841	1.74096682	-1.318398162	0.001162744	10592	5	837
NM_203402	0.60989063	0.22578527	-1.725410306	0.004575758	6180	5	659
NM_203402	0.74485846	1.13578721	-1.725410306	0.000713103	6274	5	68
NM_016484	0.24927623	0.70875778	-1.704094679	0.008660451	6199	5	693
NM_016484	0.59121784	1.05831954	-1.704094679	0.002090407	6272	5	88
XR_010179	0.01428699	0.59459427	-1.750331322	7.21E-05	6716	5	121
XR_010179	-0.1804985	0.69841874	-1.750331322	4.80E-05	6877	5	203
XR_010179	-0.0013955	0.80137949	-1.750331322	4.38E-05	6888	5	93
XR_010179	0.07236766	1.00818821	-1.750331322	3.14E-06	6893	5	15
NM_032752	0.55308661	1.23761775	-1.104427697	0.038128896	6123	5	1013
NM_005510	0.60769313	1.25065849	-1.547607341	0.004119293	6279	5	168
NM_005510	0.6356646	1.31667124	-1.547607341	0.000653541	6301	5	128
NM_152898	0.29880156	1.05069216	-1.128662999	0.029614362	6102	5	759
CB550080	0.22811133	0.13784657	-1.859649048	0.000350153	6527	5	902

NM_002319	-0.6186604	0.4748281	-1.686820712	0.000265205	6870	5	963
NM_002319	-0.2231189	0.86989707	-1.686820712	2.79E-05	6882	5	173
NM_153813	0.91595687	-0.1453931	-1.803780843	0.012022388	6168	5	1066
NM_014759	1.7459644	1.53461506	-1.924844425	0.000482148	6001	5	1154
NM_014759	1.16152687	1.24562561	-1.924844425	3.44E-05	6265	5	688
A_01_P013169	0.31323435	0.99738586	-1.75365996	0.013445711	6200	5	776
A_01_P013169	0.76705288	1.28942951	-1.75365996	0.003018144	6254	5	414
NM_000554	0.41974902	0.47642326	-1.193232065	0.038519169	8623	5	756
NM_000554	0.8338906	0.33258376	-1.193232065	0.024060775	6183	5	891
NM_020812	-0.1549351	1.86380671	-1.907432405	9.33E-05	10203	5	743
NM_003954	0.59308177	0.59233763	-1.513729363	0.00775351	8629	5	282
NM_003954	0.82392771	1.29336675	-1.513729363	0.004263975	6285	5	401
NM_080722	0.71276041	1.5309617	-1.37700879	0.016856326	6327	5	913
NM_080722	0.64573117	1.28116915	-1.37700879	0.007703998	6280	5	398
NM_023015	-0.0888458	1.80305056	-1.862644077	1.18E-05	10146	5	668
NM_023015	-0.1719925	1.60181459	-1.862644077	0.001910371	9867	5	923
NM_001002017	0.00362532	0.47313198	-1.670857458	0.001811081	6711	5	527
NM_001002017	0.26813278	0.61989991	-1.670857458	0.000601518	6729	5	113
A_01_P018326	0.46496704	0.75672645	-1.307720066	0.017726913	8659	5	501
A_01_P018326	0.45518952	0.72428008	-1.307720066	0.003485024	8660	5	153
NM_000540	0.20507645	0.4350134	-1.312758619	0.044845942	6108	5	915
NM_000540	0.39621305	0.28031075	-1.312758619	0.033111348	6171	5	764
NM_002449	1.20567697	1.1922666	-1.625439878	0.007062816	6289	5	834
NM_002449	1.15741149	1.31623576	-1.625439878	0.004041495	6288	5	749
NM_018207	-0.1932887	0.5533502	-1.508027217	0.01110913	6908	5	682
CN643965	0.34933987	0.00820806	-1.765210598	0.000597588	6457	5	948
CN643965	0.26448098	2.04705141	-1.765210598	0.011005563	10541	5	1116
CN643965	0.36544475	1.5337056	-1.765210598	0.00041748	6305	5	131
NM_006385	0.3619125	1.0790423	-1.881488567	0.000289704	7051	5	118
NM_006385	0.02502819	1.67557605	-1.881488567	0.000239875	10145	5	677
NM_014497	0.26687104	1.0373014	-1.721516204	0.000527512	7041	5	100
NM_014497	0.23816645	1.29837875	-1.721516204	0.002899398	9931	5	533
NM_152221	0.09099577	0.97987411	-1.924014466	0.001437868	9799	5	359
NM_152221	-0.1070972	1.26255374	-1.924014466	0.000999142	9800	5	542
A_01_P002414	0.2503801	0.5522474	-1.602438484	0.000253102	6730	5	107
A_01_P002414	0.31017039	0.97770456	-1.602438484	0.000154585	7040	5	6
NM_080283	-0.8581806	1.49851039	-1.798487329	0.006326096	6043	5	1157
NM_017679	0.43079523	-0.340344	-3.387816596	0.038598019	5937	5	1221
NM_015404	0.00021553	1.45466142	-1.719862234	0.000934838	6960	5	327
NM_015404	0.15246848	1.30125638	-1.719862234	4.72E-06	7058	5	36
NM_171997	0.8450926	1.40077552	-1.599747354	0.013216462	6286	5	785
NM_171997	0.57487187	1.21800043	-1.599747354	0.006571322	6277	5	268
NM_006732	0.60824158	0.72366323	-1.092797882	0.031955161	6115	5	843

XR_012313	0.58704084	0.76799992	-1.231671495	0.047454989	8684	5	772
XR_012313	0.735379	0.8688362	-1.231671495	0.026559584	8685	5	669
NM_012433	0.01020319	1.77442402	-1.700975318	0.000536328	9942	5	770
NM_024056	-0.5290303	0.98728268	-1.599966836	0.000866233	6649	5	795
NM_024056	-0.4238993	1.03893968	-1.599966836	0.006093658	6645	5	939
XR_009754	0.84120572	0.44515608	-1.400745969	0.001277958	8771	5	586
XR_009754	0.75176364	0.70471744	-1.400745969	0.001275385	8772	5	301
NM_000448	-0.098651	0.93144183	-2.131163829	0.004398475	6040	5	1149
NM_014224	0.5791665	0.41736083	-1.206234048	0.031614023	6175	5	827
NM_014580	0.43954961	0.86216485	-1.635600536	6.28E-05	7065	5	2
NM_014580	0.40836437	0.72703775	-1.635600536	0.000231233	6731	5	59
NM_182571	-0.3288613	0.26748117	-1.654992687	1.37E-05	6466	5	806
NM_182571	-0.4010286	0.15865133	-1.654992687	4.59E-05	6460	5	1003
XR_014568	0.91604545	0.88493231	-1.499825816	0.029783964	6294	5	819
XR_014568	1.20005649	1.08916472	-1.499825816	0.016691561	6369	5	953
A_01_P015849	0.08499158	0.58371755	-2.124311183	0.000117989	6692	5	360
A_01_P015849	-0.0673603	0.44038208	-2.124311183	0.000142966	6691	5	643
NM_030651	0.62202585	0.12088478	-1.30512967	0.038161718	8690	5	951
NM_017806	0.35678626	0.5977475	-1.440610199	0.013033913	8633	5	330
NM_017806	0.42966141	0.70077397	-1.440610199	0.009628158	8634	5	200
NM_005649	0.31260322	0.45023285	-1.428282256	0.005924688	6732	5	609
NM_203393	0.8480328	1.57251099	-1.9846643	0.002687734	10508	5	796
NM_203393	0.84446312	1.72833711	-1.9846643	0.000158468	10588	5	712
XR_014215	-0.5288964	0.75037947	-1.992207922	0.00364417	6030	5	1152
XR_014215	-0.1032157	1.13645241	-1.992207922	0.003293061	9763	5	541
NM_182947	0.33707118	1.78823086	-1.669870496	0.000493552	10559	5	513
NM_182947	0.18194779	1.8820633	-1.669870496	0.001633208	10560	5	747
NM_018607	-0.2493985	1.61305839	-1.647657566	4.59E-05	6963	5	560
NM_018607	-0.0610038	1.74643194	-1.647657566	3.30E-06	9981	5	509
NM_013292	1.0807891	0.01953959	-1.937773929	0.038534384	6128	5	1113
NM_002602	0.79290541	0.57253081	-1.742184531	0.009678192	6160	5	553
NM_002602	1.36757752	0.42398617	-1.742184531	0.007407597	6155	5	1030
NM_024042	0.0700409	-0.7423023	-2.211028326	0.021463093	5954	5	1195
NM_024042	-0.9128258	-0.4863948	-2.211028326	0.00319029	5944	5	1211
NM_000070	-0.0785637	1.6801577	-1.634670806	3.24E-06	9980	5	434
NM_000070	0.00420977	1.44727312	-1.634670806	9.26E-05	7054	5	238
XR_011108	-0.3004942	1.76067908	-1.841049853	0.000166891	10202	5	757
NM_006653	0.13564975	0.61926167	-1.778023167	0.000109459	6703	5	127
NM_006653	0.34281577	1.03692836	-1.778023167	3.57E-05	7038	5	9
NM_080823	1.75320135	0.34967687	-1.22981877	0.037087227	10854	5	1215
NM_080823	0.58946167	0.99510613	-1.22981877	0.010667757	8778	5	615
NM_019002	-0.1087179	1.82720891	-1.769633833	1.23E-05	9979	5	702
NM_001703	-0.2093502	0.45617801	-1.482099009	0.014379718	6906	5	745

NM_001703	0.23096199	0.63630691	-1.482099009	0.001966861	6923	5	96
NM_033419	-0.0288803	0.29896031	-1.587204692	7.23E-05	6471	5	588
NM_033419	0.12165733	0.48358844	-1.587204692	3.25E-05	6722	5	212
NM_024165	0.00720695	0.92077287	-1.653471799	1.69E-05	6892	5	32
NM_024165	0.17324584	1.24698105	-1.653471799	1.98E-05	7060	5	18
NM_005808	0.17860695	1.31515284	-1.818977426	0.000229717	9803	5	177
NM_005808	0.16374465	1.52574822	-1.818977426	0.000555543	9933	5	483
NM_016366	0.76697243	1.92632943	-1.39460556	0.005406323	10599	5	986
NM_016366	0.83707737	1.98485938	-1.39460556	0.002413213	10600	5	991
NM_002077	-0.0439151	1.83500994	-1.741818322	0.000357255	10205	5	645
NM_002077	0.03782861	1.07844967	-1.741818322	0.00010853	7037	5	85
NM_003315	0.44986347	0.76993063	-1.528718396	3.54E-05	7066	5	20
NM_003315	0.28805263	0.80909866	-1.528718396	5.88E-05	7062	5	16
NM_004473	0.85771407	1.40390269	-1.124660169	0.044763134	11133	5	1118
NM_004473	0.68306991	0.85355809	-1.124660169	0.008432537	6116	5	644
NM_020246	-0.199614	0.73084822	-1.594874017	6.66E-05	6881	5	186
NM_020246	-0.2152006	0.64540363	-1.594874017	4.15E-05	6880	5	308
NM_152600	-0.2171073	0.81709169	-1.704060721	1.85E-05	6879	5	187
NM_152600	-0.2205795	0.55508714	-1.704060721	0.000154565	6699	5	506
XR_013013	0.01323219	1.44496515	-1.662784475	9.19E-05	6940	5	477
NM_016408	0.08844267	1.5973799	-1.653953287	0.000378943	9936	5	503
NM_016408	0.10531035	1.56045706	-1.653953287	0.00054653	9935	5	495
NM_016408	0.23435168	1.88314519	-1.653953287	0.000179327	10018	5	722
NM_016408	0.03196805	1.8970169	-1.653953287	0.000534823	9943	5	894
NM_014827	-0.0409315	0.69674889	-1.691597821	0.002476465	6733	5	538
NM_014827	0.16743958	1.00815214	-1.691597821	0.000327052	7052	5	82
NM_018043	-0.3153248	0.31705912	-2.076828401	0.002335768	6399	5	1051
NM_014515	0.08945813	1.04441596	-1.496260482	0.001156624	7240	5	33
NM_014515	0.26975443	1.29781899	-1.496260482	0.000676841	7246	5	25
NM_198267	1.02458338	0.08550934	-1.229031224	0.018660421	10851	5	1177
NM_013353	0.4358877	1.57387173	-1.90378369	0.000103324	9821	5	458
NM_001665	1.46512037	-0.8036688	-1.463483399	0.011991309	10857	5	1212
NM_001665	1.73247975	-0.3448727	-1.463483399	0.00185526	10858	5	1197
NM_015148	0.01412846	0.7376589	-1.396336939	0.018713456	6611	5	890
NM_018010	0.54789657	1.74022267	-1.725549272	0.0044809	9844	5	1016
XR_012186	0.66151701	1.20971584	-1.81393032	0.004641452	6276	5	274
XR_012186	0.51724466	1.06864399	-1.81393032	0.000515137	6253	5	120
NM_001261	0.25130822	1.59927029	-1.639551368	0.000105522	9970	5	296
NM_001261	0.37259435	1.74035198	-1.639551368	3.82E-05	9971	5	437
NM_001835	-0.224093	0.62789658	-1.424578022	0.041861197	6106	5	901
A_01_P002846	-0.0507756	0.94289064	-1.571971837	3.98E-05	6884	5	38
A_01_P002846	0.41729852	0.97419748	-1.571971837	3.22E-05	7068	5	3
NM_002971	0.13956666	0.59054187	-1.98271058	0.012131016	5962	5	821

NM_033118	1.40043394	2.17103737	-1.502676682	0.00376445	10611	5	1172
NM_033118	1.16631993	1.91851237	-1.502676682	0.00043534	10595	5	1001
NM_198698	0.28657785	1.03759678	-1.665510939	0.001441112	6239	5	10
NM_198698	0.4159171	1.31456455	-1.665510939	0.000477226	6306	5	14
NM_005741	0.02251343	0.30504441	-1.593352619	0.000138379	6718	5	567
NM_002991	0.61169393	0.79214683	-1.154817658	0.013353238	8662	5	511
NM_019092	0.43703449	1.48649578	-1.501921814	9.46E-05	9976	5	166
NM_019092	0.53955391	1.48557189	-1.501921814	0.000210693	9977	5	253
NM_130434	0.23820366	0.98232177	-1.612115242	8.39E-05	7045	5	17
NM_130434	0.17669668	1.08680593	-1.612115242	0.000237162	7039	5	39
NM_017514	-0.0307726	1.42724913	-1.66085133	2.91E-05	6991	5	94
NM_017514	0.24929562	1.66589725	-1.66085133	1.77E-06	9964	5	197
A_01_P017040	-0.8552641	0.78319272	-1.788038817	0.038643231	6012	5	1209
NM_005657	-0.0704911	1.15727946	-1.659553953	0.001270543	7012	5	446
NM_005657	-0.0849759	0.97935627	-1.659553953	0.006656349	7010	5	716
NM_005825	0.01263442	0.80221788	-1.342762784	0.000427077	6938	5	233
NM_020927	0.60200708	1.45864868	-0.937606741	0.009225623	9646	5	911
NM_001481	-0.1466618	0.65279219	-1.534220862	0.000567644	6878	5	310
NM_001481	-0.3783228	0.78901148	-1.534220862	0.000230665	6875	5	505
NM_032442	0.16682	1.07387099	-1.498397932	2.77E-05	7049	5	13
NM_032442	0.14671987	0.93463848	-1.498397932	0.000609236	7044	5	108
NM_006093	0.987786	0.91042666	-1.957232991	0.018007665	6154	5	924
NM_006093	0.89040784	0.77921806	-1.957232991	0.00929722	6152	5	663
XR_010258	0.71056933	0.57198383	-1.418807341	0.034188556	6292	5	707
XR_010258	0.61484125	0.62144786	-1.418807341	0.005405501	8630	5	222
NM_018463	-0.3939398	1.81335805	-1.656990034	3.29E-06	9888	5	864
NM_012139	-0.3429069	1.08680696	-1.530910331	0.000304349	6898	5	500
NM_012139	-0.4811486	1.20982708	-1.530910331	0.001954768	6646	5	868
NM_014371	-0.5860304	0.4855132	-1.690265747	2.63E-06	6871	5	875
NM_014371	-0.4412008	0.63540096	-1.690265747	5.07E-06	6872	5	604
NM_138414	0.07287633	1.76742834	-1.438099542	8.46E-05	10002	5	475
NM_172171	-0.4220342	0.64214564	-1.701001184	0.004775035	6636	5	942
NM_145755	-0.45667	1.02530485	-1.677734133	0.000160439	6897	5	582
NM_145755	-0.3842211	0.94633128	-1.677734133	1.16E-05	6896	5	426
NM_032105	-0.8141984	1.10949585	-1.660407808	0.002567287	6640	5	1072
NM_032105	-0.9474366	1.39148759	-1.660407808	0.000863887	6643	5	1107
NM_153340	0.49842441	0.82462315	-1.337068565	0.006561333	8661	5	144
NM_153340	0.47667465	1.24665451	-1.337068565	0.004608294	8673	5	165
NM_014786	-0.3548117	1.62549299	-1.565310443	8.02E-05	6973	5	695
NM_145059	-0.4555948	1.45822019	-1.651486262	9.76E-06	6967	5	616
NM_145059	-0.4225315	1.56469313	-1.651486262	4.70E-07	6972	5	687
NM_004076	0.45365732	0.91191212	-1.425645299	0.000111378	7069	5	51
XR_010803	0.15152576	0.5438305	-1.859751166	0.009149375	6688	5	476

XR_010803	-0.0706445	0.49685685	-1.859751166	0.001967076	6686	5	494
NM_001989	0.94863012	0.23311558	-1.128416757	0.0471047	9629	5	1108
NM_003635	0.55877226	0.98324092	-1.336232228	0.002352306	8671	5	103
NM_003635	0.65700532	1.1525984	-1.336232228	0.000118222	6302	5	106
NM_148888	-0.1374971	1.34364515	-1.65501932	5.06E-05	6989	5	114
NM_148888	0.11508281	1.40441807	-1.65501932	6.16E-06	7056	5	76
NM_001567	0.34695835	0.42011864	-1.724065214	0.000102001	6727	5	285
NM_001567	0.49125512	0.58964914	-1.724065214	7.05E-05	6728	5	124
NM_020796	-0.0733527	1.68032686	-1.501630002	4.67E-05	9894	5	463
NM_014232	-0.618925	1.04451465	-1.663658555	0.000102201	9754	5	708
NM_014232	-0.4061369	1.40589468	-1.663658555	0.000398445	6966	5	570
NM_016368	0.0420452	0.63894037	-1.517876898	0.003808459	6922	5	175
NM_016368	0.12290672	1.00279449	-1.517876898	0.004844132	6236	5	90
NM_020684	-0.0400067	0.82306704	-1.579204577	3.09E-05	6895	5	42
NM_020684	-0.079166	1.2301027	-1.579204577	0.00029871	6988	5	55
NM_201994	0.12483277	0.95629999	-1.247970117	0.003660144	7089	5	338
NM_020899	0.25139316	1.9557204	-1.632036806	7.05E-05	10019	5	765
NM_016335	0.39225402	1.70151195	-1.939984392	0.001574825	6216	5	748
NM_018081	-0.0353492	0.85117001	-1.516300341	2.58E-05	6883	5	58
NM_018081	-0.1360195	1.06568571	-1.516300341	2.26E-05	6901	5	84
NM_015503	0.43279491	1.30131344	-1.320091961	0.001638046	7249	5	111
NM_015503	0.17641695	1.49054697	-1.320091961	0.001664601	7247	5	265
NM_004200	-0.6878641	0.51024274	-1.617017168	0.000214165	6873	5	814
NM_001631	0.06851135	1.3063034	-1.406775365	0.000265892	6993	5	67
XR_010913	-0.09067	0.7646547	-1.369160636	0.000326081	6913	5	174
XR_010913	-0.2668766	0.91630375	-1.369160636	6.13E-05	6887	5	313
NM_032432	0.40476005	1.90893365	-1.689675473	0.002361669	10561	5	808
NM_032432	-0.2352205	1.03036467	-1.689675473	0.000863453	6658	5	627
NM_015133	0.99047995	-0.1145879	-1.49668702	0.017080818	6169	5	1061
NM_001632	0.44959763	0.84478023	-1.026458755	0.045307394	8664	5	810
NM_145003	1.61036391	0.55583997	-1.609381113	0.037949094	6156	5	1146
NM_017784	0.50472091	1.77011944	-1.874682611	3.82E-05	9826	5	568
NM_017613	-0.1692256	1.21799043	-1.67226149	0.000719588	7023	5	555
NM_015037	-0.0638987	1.21174178	-1.533988961	2.44E-06	6903	5	65
NM_015037	-0.1935542	1.11894881	-1.533988961	1.28E-05	6902	5	171
NM_025099	-0.1341156	0.83975125	-1.539161683	0.007156173	7207	5	734
NM_025099	-0.1021123	1.22470117	-1.539161683	0.001570993	7021	5	498
NM_021827	0.13168284	-0.3519625	-2.218479117	0.006646536	5956	5	1153
NM_021827	0.08868271	-0.4770263	-2.218479117	0.00819279	5955	5	1170
NM_012182	1.06960718	1.08026002	-1.211785155	0.003327772	8779	5	754
NM_024874	-0.0137568	1.18934168	-1.630553455	0.002581776	7013	5	546
NM_024874	0.02936171	1.45003902	-1.630553455	0.000953742	9934	5	516
NM_017759	0.42812553	0.06259471	-1.663583459	0.003073434	6458	5	898

NM_017759	-0.0583781	0.10018575	-1.663583459	0.00068349	6448	5	914
NM_019605	0.57159014	2.02910476	-1.546853953	0.010327318	10554	5	1136
NM_019605	0.24626816	0.93277993	-1.546853953	0.041939989	10623	5	1094
NM_024895	0.13005754	1.22465959	-1.628853354	2.18E-05	7059	5	31
NM_024895	0.21301133	1.37487852	-1.628853354	1.79E-05	7057	5	48
XR_010160	0.44348738	1.89146254	-1.603567412	0.00013163	10567	5	611
XR_010160	0.26885644	1.81919728	-1.603567412	4.85E-06	9968	5	459
NM_144999	-0.1876845	0.60404795	-1.366585709	0.000394002	6912	5	404
NM_199002	-0.1847975	1.02451258	-1.323278459	0.001380495	7242	5	263
NM_199002	-0.0641999	1.50513034	-1.323278459	0.004419283	7003	5	574
DR771278	0.2643529	1.36267958	-1.540934933	8.93E-05	7061	5	61
DR771278	0.28016882	1.25868807	-1.540934933	0.000468439	9949	5	159
NM_017664	-0.3481672	1.7867852	-1.591012524	0.000333201	9892	5	830
NM_002528	-0.0495346	0.82788187	-1.687308394	2.50E-05	6891	5	87
NM_002528	0.03120259	0.82174651	-1.687308394	0.000145074	6890	5	99
NM_002075	-0.6251272	1.09680403	-1.395314916	0.000221581	6869	5	788
NM_032867	-0.0956937	0.76989896	-1.408606486	0.00281659	6657	5	737
NM_032867	-0.1195048	1.06447341	-1.408606486	0.000649262	7088	5	266
NM_001280	0.22330614	1.05950688	-1.560301047	9.31E-05	7046	5	24
NM_001280	0.19817308	1.18912183	-1.560301047	0.000136556	7047	5	44
XR_014577	0.91818115	0.35968125	-1.680702348	0.026083308	6143	5	896
XR_014577	0.80454297	0.48476651	-1.680702348	0.015495134	6144	5	664
NM_001001323	-0.1031019	0.7990029	-1.517496495	0.00198571	6802	5	452
NM_001001323	-0.0057739	0.7896696	-1.517496495	0.007676695	7208	5	654
CN801688	-0.0266673	0.80594069	-1.691293373	0.000294878	6889	5	211
CN801688	-0.0183266	0.91412234	-1.691293373	0.001623223	7011	5	372
NM_000537	1.08589826	0.77265757	-1.625976093	2.87E-05	6261	5	433
NM_000537	1.08896607	0.87950679	-1.625976093	5.04E-07	6262	5	415
NM_014716	0.46731929	0.24486602	-1.508127969	0.008756111	8627	5	591
NM_014716	0.57120937	0.39579557	-1.508127969	0.005048217	8628	5	390
NM_054031	0.42977046	1.48334821	-1.450661326	0.00859494	6326	5	573
NM_054031	0.59557476	1.31114251	-1.450661326	0.001259244	6303	5	130
NM_005660	0.42248055	0.90650926	-1.315634529	0.000847236	8667	5	34
NM_005660	0.37715068	1.01447428	-1.315634529	0.000902315	8668	5	35
NM_032687	-0.1264907	1.23857213	-1.585402377	0.000276358	7019	5	283
NM_032687	-0.2480023	1.47447756	-1.585402377	1.04E-05	6974	5	441
XR_010742	0.10759599	1.15567683	-1.531541376	0.000458412	7043	5	125
XR_010742	0.32303679	1.68478825	-1.531541376	0.002719935	9944	5	850
NM_017503	0.02955225	1.26285686	-1.634528992	3.02E-05	7055	5	64
NM_017503	0.10722933	1.16933239	-1.634528992	0.000208895	7053	5	80
NM_002824	0.08107626	0.48995155	-1.157334311	0.048303665	8621	5	798
NM_007029	0.7739259	1.33997197	-1.776214161	0.016397221	6328	5	863
NM_007029	1.49867449	1.55436477	-1.776214161	0.011802501	10534	5	1135

NM_022819	0.3322489	1.45732243	-1.479911494	0.007661459	6324	5	486
NM_022819	0.50815395	0.85947042	-1.479911494	0.000803261	8666	5	41
NM_203370	0.34666192	0.8893811	-1.612355032	7.42E-06	7071	5	5
NM_203370	0.54696301	1.00042829	-1.612355032	3.11E-06	7072	5	4
NM_015644	-0.1377895	1.05129667	-1.571700498	3.79E-05	6987	5	50
NM_015644	-0.0647925	1.29059499	-1.571700498	2.50E-06	6990	5	49
NM_007101	0.98945621	1.45232414	-1.100928242	0.028968482	9719	5	1067
NM_198541	0.48912	1.94755633	-1.464032634	0.000134926	10568	5	679
XR_011067	1.02887998	0.8915496	-1.013192555	0.044039651	6297	5	999
XR_011067	0.58849197	0.86011095	-1.013192555	0.021089017	8663	5	740
NM_000364	0.42377402	1.01584576	-1.288277546	0.002159311	8669	5	47
NM_000364	0.44861779	1.21086477	-1.288277546	0.001857732	7248	5	79
NM_178500	0.58574582	0.7945775	-1.328440038	0.03471359	6293	5	641
NM_178500	0.59636645	1.04822228	-1.328440038	0.010557342	8675	5	288
NM_025132	0.61732621	1.60028716	-1.469086765	0.000640017	6314	5	286
NM_025132	0.5198783	1.503099	-1.469086765	0.000153694	9978	5	371
NM_016291	0.24003374	1.22426449	-1.444215077	0.000628795	9950	5	145
NM_016291	0.02231286	1.30366905	-1.444215077	0.000424665	7022	5	261
NM_003899	0.32751241	1.87245889	-1.776677671	3.63E-05	9822	5	638
NM_003899	0.2761382	1.7130196	-1.776677671	0.000292415	9824	5	576
NM_001009598	2.79336288	1.5237927	-2.529736282	0.010225592	11239	5	1231
NM_033378	0.75505022	0.9174638	-1.09339309	0.006993392	8827	5	633
NM_003427	0.08917565	1.51031916	-1.629678594	0.00011393	9937	5	290
NM_003427	0.24643117	1.62102302	-1.629678594	6.25E-05	9963	5	275
NM_145645	0.27887913	1.65259206	-1.539558754	5.23E-05	9973	5	369
NM_145645	0.32911604	1.80193189	-1.539558754	5.87E-05	9972	5	571
NM_005231	0.05800262	1.75739719	-1.522552027	2.61E-05	9989	5	552
NM_002498	0.30793958	0.9973782	-1.171794662	0.028542306	10362	5	961
NM_173618	0.1855888	0.64502337	-1.522507244	1.43E-05	6723	5	43
NM_173618	0.35667101	0.88764455	-1.522507244	6.09E-06	7067	5	7
NM_145200	0.23508882	1.54423634	-1.21790672	0.042468337	10548	5	1102
NM_145200	0.5689714	1.38910231	-1.21790672	0.018853514	6387	5	822
NM_201523	-0.1471133	1.43213438	-1.48017937	1.18E-05	6975	5	277
NM_201523	-0.2817534	1.74393286	-1.48017937	0.000128699	9893	5	727
NM_152901	0.63052491	1.19715848	-0.767342515	0.026649927	9652	5	952
NM_145275	0.84444529	0.85894829	-1.75205491	0.013319206	6153	5	596
NM_145275	0.84977646	1.49226175	-1.75205491	0.000426618	6310	5	318
NM_015665	0.40876309	1.00717347	-1.784903566	0.010377934	6232	5	322
NM_015665	0.56678158	1.446257	-1.784903566	0.000274711	6309	5	78
NM_033113	0.34515507	0.38104058	-1.424231025	0.022735684	6612	5	768
NM_145729	0.37007715	1.40012722	-1.361937354	1.20E-05	7279	5	60
NM_145729	0.39244382	1.45708123	-1.361937354	6.54E-06	7280	5	74
NM_023004	0.40156178	0.9873797	-1.786674887	0.00793375	6201	5	484

NM_023004	0.547693	1.50146701	-1.786674887	0.00064823	9825	5	455
NM_174976	0.24476348	0.89874522	-1.252399724	0.001794851	6939	5	453
XR_012933	-0.0420923	0.39495582	-1.46903418	4.39E-07	6719	5	432
XR_012933	-0.050266	0.52602889	-1.46903418	0.000353092	6789	5	449
NM_138477	-0.1186317	0.16071119	-1.670004185	0.000128488	6465	5	769
NM_138477	-0.0072503	0.48028762	-1.670004185	0.000297083	6715	5	235
NM_172027	-0.3896927	0.38967744	-1.57918007	0.000366499	6467	5	729
NM_016423	0.20312567	1.20329835	-1.43803106	1.59E-05	7050	5	29
NM_016423	0.07706953	1.57947928	-1.43803106	2.04E-06	10003	5	245
NM_007322	0.2338649	0.29608382	-1.633284023	0.004644237	6521	5	655
NM_007322	0.02254162	0.39318649	-1.633284023	0.000356402	6528	5	612
NM_032269	0.54476201	1.56496671	-1.733291126	0.000574867	6311	5	269
NM_032269	0.52976567	2.06615877	-1.733291126	0.000746979	10575	5	945
NM_020991	0.15907768	1.90216389	-1.431738843	0.001909779	10565	5	823
NM_015015	0.73820337	0.98252608	-1.428400195	0.001183979	6300	5	530
NM_000229	0.10057161	1.47271959	-1.721479595	0.000942383	10555	5	170
NM_000229	-0.1991287	1.92765245	-1.721479595	0.000311805	10204	5	861
NM_024517	0.37799252	0.81005304	-1.415572334	0.00073888	7064	5	135
XM_496244	0.27893506	0.43460719	-1.34338875	0.000157419	6725	5	335
XM_496244	0.23675805	0.55709881	-1.34338875	0.000139904	6726	5	161
NM_145288	0.5277359	0.80916183	-1.736317763	0.00356161	6234	5	86
NM_145288	0.70200268	1.62739322	-1.736317763	0.0010967	6313	5	519
NM_012384	-0.1475006	0.85219889	-1.495153849	0.000491815	6803	5	316
NM_012384	0.10913476	0.79509237	-1.495153849	0.009270855	7209	5	647
XM_371614	-0.3377641	1.04293295	-1.405033927	0.001031399	6651	5	635
NM_018254	-0.2708913	1.39489788	-1.562512186	0.000590836	6956	5	608
NM_013957	0.66491222	0.80135054	-1.879221547	0.007850249	6233	5	422
NM_006271	0.69971469	1.0199483	-1.705119446	0.000444976	6298	5	30
NM_006271	0.51152361	1.38901595	-1.705119446	0.000218177	6308	5	40
NM_016340	0.53658973	1.18946131	-1.5426799	0.000137368	7077	5	56
NM_016340	0.45015167	1.75433047	-1.5426799	7.57E-05	9974	5	559
NM_153635	0.17759058	1.09642346	-1.266630444	0.011448098	7244	5	407
NM_153635	0.80848945	1.4144497	-1.266630444	0.000462781	6320	5	373
NM_001684	0.04976167	0.98520067	-1.390890106	0.009031217	7215	5	692
NM_000458	0.19809721	0.9946533	-1.508907167	0.000522346	7042	5	63
NM_021976	0.29453633	1.63750489	-1.585424297	1.82E-06	9967	5	148
NM_021976	0.22686661	1.65466516	-1.585424297	4.90E-06	9966	5	205
NM_175850	0.07989023	0.75148167	-1.664242756	0.002797181	6935	5	229
NM_175850	0.12227848	0.5829587	-1.664242756	0.001430487	6734	5	353
NM_053051	0.25638317	1.03043454	-1.540520456	1.27E-05	7070	5	1
NM_053051	0.26567962	1.40302842	-1.540520456	4.82E-05	6999	5	19
NM_173637	0.99915017	0.3326157	-1.044603638	0.041593741	6193	5	1014
NM_005341	-0.1070684	0.78657797	-1.238768467	0.002765397	6927	5	341

NM_006671	0.29898685	1.14001685	-1.72009047	0.006227576	6241	5	528
NM_006671	0.39650134	0.93618912	-1.72009047	0.013393189	6124	5	797
NM_003195	-0.0047969	1.11312157	-1.18378469	0.004891226	7243	5	350
NM_003195	0.18431293	1.32437024	-1.18378469	0.002145852	7245	5	306
XR_013358	0.23407351	1.01363295	-1.676496385	0.007202865	6240	5	179
XR_013358	1.18071994	1.14381185	-1.676496385	0.000309921	6263	5	634
NM_030818	-0.2200856	1.43951627	-1.489630368	0.000140936	6971	5	328
NM_030818	-0.4296159	1.44552301	-1.489630368	6.69E-06	6968	5	590
NM_021922	-0.0246233	1.22961516	-1.687663845	0.000298009	7015	5	196
NM_021922	-0.1059172	1.1941272	-1.687663845	0.000479729	7014	5	312
NM_005157	1.0275857	0.91539155	-1.246070594	0.027540851	6295	5	867
NM_005157	0.69516525	1.16695253	-1.246070594	0.013986869	8676	5	583
NM_016143	0.17187674	1.15858152	-1.206511196	0.001537981	7187	5	355
NM_021734	0.2474505	0.39229642	-1.413889334	0.016190762	8631	5	575
NM_021734	0.44619975	0.5596477	-1.413889334	0.014001318	8632	5	379
NM_001008568	0.14362712	0.53113977	-1.341804222	5.71E-05	6724	5	228
NM_001006607	0.71975276	1.13636716	-1.258179551	0.016446118	8677	5	585
NM_001006607	0.73295554	1.57657584	-1.258179551	0.003893482	6321	5	631
NM_000292	-0.0604958	1.20794692	-1.612406914	0.0001053	7024	5	276
NM_000292	0.48184364	1.54225442	-1.612406914	0.000176766	9975	5	299
NM_152856	0.08919479	0.94368115	-1.292775166	0.000689416	7182	5	194
NM_005011	0.00950792	0.41301308	-1.335988112	0.010957922	8620	5	610
NM_005011	0.52173121	0.24242635	-1.335988112	0.000600601	8692	5	607
NM_054016	0.48293558	1.32357139	-1.504528032	0.000966221	7078	5	294
NM_005220	0.59288876	1.39108323	-1.556528779	0.00808888	6325	5	529
NM_005220	0.80325838	1.21757843	-1.556528779	0.002076369	6315	5	180
CO646479	-0.0297895	1.07501902	-1.357807704	0.000500226	7169	5	236
CO646479	-0.03713	1.11933434	-1.357807704	0.00134053	7171	5	418
NM_000155	0.1905147	0.61678265	-1.326911329	0.001834293	6924	5	219
NM_144664	-0.0763502	1.56941213	-1.492583705	6.42E-06	7006	5	525
NM_144664	0.19511054	1.6295636	-1.492583705	0.005943261	9842	5	983
NM_177417	0.00265178	1.93412706	-1.712940687	0.000244562	10206	5	755
NM_015113	-0.0678109	0.51079476	-1.475206685	0.002963811	6807	5	640
NM_015113	-0.2695171	0.68159284	-1.475206685	0.00212389	6801	5	726
NM_001006623	-0.9805109	1.56400826	-1.794278444	0.001603193	9730	5	1161
NM_003479	2.9077143	1.16881659	-1.478807963	0.005170874	11249	5	1224
NM_003479	2.85733252	1.26823886	-1.478807963	0.005092759	11250	5	1222
NM_021628	-0.5336708	0.90833412	-1.483035037	0.008819039	6610	5	937
NM_207442	0.39849812	1.25251282	-1.310568879	0.000434835	7080	5	164
NM_207442	0.1297866	1.46725348	-1.310568879	0.00026015	7272	5	325
NM_019591	0.22562527	1.48640914	-1.853679939	0.000520259	9804	5	435
NM_019591	0.36532157	1.67804718	-1.853679939	0.000721696	9823	5	662
NM_006458	0.95805726	1.08006267	-1.54135538	0.000775353	6316	5	224

NM_006458	1.10225629	1.46827331	-1.54135538	0.00188419	6318	5	718
NM_145296	-0.1997588	0.63560645	-1.538471564	0.026006248	5963	5	1058
NM_144982	-0.1988788	1.40274019	-1.643782946	0.000441001	7026	5	540
NM_144982	-0.4338349	1.66825248	-1.643782946	0.001035202	9869	5	985
NM_016228	0.27086272	1.18874727	-1.668093244	5.88E-05	6941	5	129
NM_016228	-0.3188083	1.42302985	-1.668093244	0.000684703	9866	5	736
NM_021932	-0.1675286	1.16199977	-1.405711097	0.000358534	7167	5	394
NM_021932	-0.1292625	1.09496207	-1.405711097	0.00063271	7166	5	365
NM_003695	0.87753057	0.90282954	-1.765084784	0.000975327	6252	5	496
NM_003695	0.89883185	0.89772746	-1.765084784	0.014366154	5991	5	969
XR_010449	0.22829443	1.54141715	-1.96714086	0.002543017	6215	5	592
XR_010449	0.22077761	1.51956194	-1.96714086	0.001339079	6214	5	457
CO582642	0.0869648	1.84268701	-1.521045224	7.43E-06	9995	5	621
NM_033624	0.04536096	1.09266522	-1.168097765	0.0008575	7186	5	387
NM_022039	0.29327443	1.54540743	-1.536690758	3.53E-06	9965	5	109
NM_022039	0.34029384	1.75834206	-1.536690758	2.03E-06	9969	5	377
NM_030931	1.79336526	0.01905225	-1.400542719	0.016276856	10862	5	1182
NM_030931	2.05419399	0.30306406	-1.400542719	0.030022677	10863	5	1196
NM_014964	0.04393823	1.69095362	-1.629047883	4.60E-05	9983	5	440
NM_014964	-0.041456	1.7608286	-1.629047883	8.31E-05	9982	5	614
NM_005515	0.35787187	0.42216333	-1.572837715	0.021972332	8624	5	578
NM_005515	0.77201961	0.53624306	-1.572837715	0.018021616	6149	5	622
NM_021958	0.16121556	1.01024275	-1.276728329	0.000139188	7184	5	110
NM_021958	0.0663619	1.03339965	-1.276728329	0.000417935	7183	5	185
NM_199054	0.99725473	1.90467906	-1.216950245	0.00498704	10601	5	1043
NM_199054	1.22380565	2.23538261	-1.216950245	0.003967544	10602	5	1160
NM_198699	0.36948458	0.90651302	-1.089937203	0.002448239	7294	5	356
NM_021140	0.21278081	0.68724088	-1.484898814	0.041978242	7302	5	1034
NM_001945	-0.1452668	1.53185044	-1.229018815	0.002062566	7004	5	656
NM_033208	0.07122642	1.43515529	-1.86640707	0.004760496	9846	5	851
NM_033208	0.02848935	1.64529834	-1.86640707	0.003944854	9847	5	1045
NM_000954	-0.8166116	1.28271341	-1.263367592	0.000998093	6946	5	1023
NM_152657	0.7631319	0.67913038	-1.655536204	0.001369784	6251	5	278
NM_152657	0.80383872	0.72051919	-1.655536204	0.002651677	6161	5	380
NM_031293	0.90884608	1.48412889	-1.475628196	0.034908935	6329	5	1068
NM_031293	0.69979211	1.94136605	-1.475628196	0.001078108	10576	5	889
NM_002336	-0.4442149	1.28752464	-1.315396763	0.00030073	6944	5	731
NM_032512	0.26951241	0.69231244	-1.105103622	0.010754704	8638	5	413
NM_032512	0.38870726	1.12387398	-1.105103622	0.007840014	8679	5	340
NM_020817	-0.3868845	0.60405311	-1.565186647	0.000928463	6874	5	778
NM_005131	-0.2102599	0.98964587	-1.367551992	0.007119747	7033	5	816
NM_005131	-0.2333548	1.14353645	-1.367551992	0.007080982	7034	5	881
NM_018257	0.27356582	1.77331729	-1.998271906	0.000391752	9820	5	705

NM_018257	0.19274831	1.6659231	-1.998271906	0.000565398	9819	5	690
NM_018062	0.03889777	1.32521775	-1.544274617	0.020007558	10379	5	1053
NM_015526	0.19430442	0.60455442	-1.581663865	0.000485932	6713	5	209
NM_015526	0.31740227	1.26694223	-1.581663865	0.000872518	6943	5	45
NM_019071	0.59615085	0.65526551	-1.116965604	0.002130473	8783	5	630
NM_019071	0.37135046	0.9401	-1.116965604	0.000379697	7130	5	223
NM_015205	-0.0883782	1.41018809	-1.259952055	7.48E-06	6985	5	289
NM_002864	-0.0552208	0.99150292	-1.278241679	0.000415311	7168	5	317
NM_007078	0.13447379	1.12969861	-1.289659352	0.004270952	6101	5	262
NM_003631	0.53715341	0.95686341	-1.281530752	0.001198438	8670	5	53
NM_003631	0.79908526	1.38662235	-1.281530752	0.000115634	6319	5	311
NM_002230	0.90772908	0.95186633	-1.226279576	0.04482582	6296	5	933
NM_002230	1.29766683	1.34474865	-1.226279576	0.016093702	6373	5	1059
NM_002230	-0.0369813	0.85307385	-1.226279576	0.000274423	7105	5	273
NM_001118	0.90981121	0.82740684	-1.236135098	0.000699383	8773	5	399
NM_033450	-0.4000392	1.11580752	-1.437185757	1.65E-05	6899	5	544
NM_033450	-0.1921903	1.13723113	-1.437185757	0.000202678	6904	5	304
NM_025082	0.82337505	0.67597242	-1.138425681	0.013440509	8658	5	625
NM_025082	0.64807937	1.23165989	-1.138425681	0.005650418	8681	5	419
CN643612	0.67235298	-0.2763445	-1.368190861	0.003620539	10865	5	1190
NM_001920	0.26672094	1.61184902	-1.443499621	0.000254209	9955	5	431
NM_005859	0.0960143	1.27057475	-1.150497572	2.73E-05	7195	5	220
NM_080632	0.42815934	1.08539815	-1.454663657	0.000163234	7073	5	12
NM_080632	0.04008591	1.28465348	-1.454663657	4.59E-05	7005	5	323
NM_021079	0.00449907	0.74295654	-1.281059106	0.00107096	6928	5	162
NM_021079	0.24171226	1.16260978	-1.281059106	4.89E-05	7196	5	70
NM_198149	0.31063452	0.90705546	-1.389873371	2.57E-06	7063	5	23
NM_145178	1.18218842	0.81687537	-1.779682718	0.003026766	6256	5	758
NM_145178	1.27168753	0.90162051	-1.779682718	0.000832635	6165	5	856
XR_013072	0.01852832	0.61258694	-1.426453482	0.003120841	6522	5	670
XR_013072	0.61643713	1.01059047	-1.426453482	0.00018349	6299	5	57
NM_032488	0.13436191	1.0215159	-1.245712082	0.001047339	7257	5	92
NM_032488	0.24098858	1.14764804	-1.245712082	0.000745555	7258	5	73
NM_017991	0.15069967	1.46380302	-1.481040869	0.00010756	9953	5	208
NM_017991	0.12482921	1.40452779	-1.481040869	0.000322836	9952	5	243
NM_014376	0.18433102	1.19627558	-1.310623385	3.64E-05	7253	5	26
NM_014376	0.24284038	1.4367325	-1.310623385	6.73E-06	7281	5	89
CN803406	-0.3526563	1.41012775	-1.46900219	0.000115017	6970	5	482
CN803406	-0.3319789	1.3710123	-1.46900219	1.75E-05	6969	5	423
NM_178493	-0.1797848	0.21112252	-1.545898608	0.004789792	6464	5	839
NM_178493	-0.1173231	0.12789188	-1.545898608	0.002520617	6463	5	887
NM_006137	0.94017233	0.65405389	-2.307052969	0.009679854	6204	5	1021
NM_006137	0.65367247	0.97772053	-2.307052969	0.008047743	6205	5	905

NM_018467	-0.1380997	1.23230887	-1.298358698	0.000303951	6992	5	193
NM_144697	-0.2083936	1.40740928	-1.391235975	0.000177213	7027	5	512
NM_173596	0.81705161	0.95213208	-1.988024837	0.003638047	6211	5	556
NM_173596	0.59296358	1.39293601	-1.988024837	0.004831862	6210	5	620
NM_019012	0.03357171	1.56239827	-1.48694905	0.001099253	9947	5	652
NM_182905	0.09361677	1.72373275	-1.480561343	0.002156203	10557	5	632
NM_182905	0.1549812	1.74677567	-1.480561343	0.000481748	10556	5	531
NM_178569	0.0192083	1.4759967	-1.486490595	0.000195998	7007	5	326
NM_178569	0.08570035	1.45339098	-1.486490595	0.000419356	9946	5	363
NM_032179	-0.204177	1.12806097	-1.290192983	1.30E-06	7143	5	252
NM_032179	-0.0906697	1.13923247	-1.290192983	8.53E-05	7170	5	279
NM_152689	0.35214617	1.00128702	-1.621738098	0.009932201	6242	5	400
NM_152689	0.63727521	1.81317299	-1.621738098	0.000558606	9852	5	865
NM_152408	0.32564389	1.23963341	-1.220523418	0.000838128	7259	5	122
NM_152408	0.44947979	1.46805624	-1.220523418	0.000358402	7286	5	210
NM_021070	-0.4119383	1.00341961	-1.315493497	2.11E-05	7133	5	507
NM_139067	-0.1906386	1.46111467	-1.373129912	7.26E-06	6976	5	430
NM_017607	-0.0479291	0.94360271	-1.181224659	2.42E-05	7145	5	202
AK125739	-0.2172747	1.3051727	-1.335226713	0.000740236	7001	5	427
NM_018416	0.13628757	0.94802438	-1.191449026	2.63E-05	7250	5	123
NM_018416	0.19955376	1.02875726	-1.191449026	2.92E-05	7251	5	77
NM_178466	1.07596465	0.50267537	-1.020680733	0.004077709	6194	5	935
NM_000898	0.2769898	1.37399337	-1.194683511	0.001223645	9177	5	470
NM_007180	0.06312742	0.80498306	-1.256526249	0.009807323	6931	5	334
NM_007180	0.33109225	1.16824313	-1.256526249	0.00365961	7260	5	150
NM_006676	0.06146159	0.69742427	-1.251752077	0.005787928	6929	5	309
NM_006676	0.06317047	0.72319538	-1.251752077	0.004738646	6930	5	256
NM_006676	-0.0557644	0.73903158	-1.251752077	0.001594459	6925	5	255
NM_006676	-0.0182443	0.72558453	-1.251752077	0.000455237	6926	5	214
NM_001005368	0.64433412	1.62114733	-1.312086063	0.000991832	10374	5	691
XR_011047	0.09017496	1.33851095	-1.41393188	0.000143654	6998	5	69
XR_011047	-0.0148644	1.70409565	-1.41393188	7.22E-05	10000	5	456
XR_012685	-0.4904049	1.53361019	-1.833521472	0.001043442	9862	5	978
NM_012231	0.17763234	1.05115998	-1.38800917	4.13E-05	7048	5	37
NM_012231	0.40003931	1.53198097	-1.38800917	0.000232437	9961	5	346
NM_004313	0.10848569	1.50691171	-1.354810577	4.13E-06	9959	5	216
NM_004313	0.1534696	1.54269326	-1.354810577	1.79E-05	9960	5	264
NM_006454	-0.2023224	1.02308247	-1.27024407	1.12E-05	7141	5	240
NM_006454	-0.2115865	1.05459113	-1.27024407	2.81E-07	7142	5	271
NM_014972	0.1926343	1.35297126	-1.342516381	2.41E-05	9957	5	116
NM_014972	0.27123014	1.42083988	-1.342516381	2.91E-05	9958	5	134
NM_198207	0.747773	0.99937361	-1.033818376	0.030231396	6197	5	926
NM_014494	-0.3221992	0.71485348	-1.466608845	0.000198225	6800	5	547

NM_014057	0.08608492	0.97196063	-1.217182442	0.000254511	7185	5	181
NM_177401	0.43594601	0.87138609	-1.173476073	0.047521965	8686	5	815
NM_177401	0.6308012	0.86499343	-1.173476073	0.013098271	8687	5	479
NM_005777	0.3074048	1.48129615	-1.24770601	4.43E-06	7284	5	199
NM_032205	-0.1077593	1.71680949	-1.679293139	0.001057906	9868	5	842
XR_013922	0.19975557	0.97243906	-1.223140516	0.008417792	7216	5	657
XR_011854	1.06023507	0.40537383	-1.254001966	0.04102448	6192	5	977
NM_017633	-0.0563721	1.24442845	-1.131178995	0.000367276	8207	5	438
NM_001798	0.03063158	1.1501143	-1.138203514	3.79E-05	7194	5	258
NM_001798	0.45913964	1.26270835	-1.138203514	0.000124861	7277	5	403
NM_001346	0.2077509	-0.0555315	-1.741765373	0.003872144	6446	5	962
NM_032228	-0.0580952	1.36601169	-1.400642525	0.000418637	7020	5	409
NM_032228	0.24981782	1.4841601	-1.400642525	0.000175103	9954	5	246
NM_018261	0.17282176	0.8346973	-1.388830577	0.002338979	7213	5	367
NM_018261	0.17142973	0.84767066	-1.388830577	0.00728264	7214	5	598
NM_016153	0.67693536	0.39953189	-1.10259865	0.002565969	8694	5	698
NM_147193	0.45423774	0.60240298	-1.033676044	0.014309088	8637	5	594
NM_014683	-0.4905487	0.35487067	-1.816076292	0.002451463	6400	5	1009
NM_014683	-0.0193878	0.50735832	-1.816076292	0.00195426	6690	5	624
NM_018171	0.46601789	0.82085613	-1.11728226	0.009966636	8640	5	366
NM_018171	0.4475419	1.08641207	-1.11728226	0.009531382	8680	5	382
NM_144617	0.64181058	-0.42995	-1.481236509	0.002115284	6507	5	1131
NM_000116	-0.1992197	1.11010805	-1.46190442	3.50E-05	6900	5	169
NM_000116	-0.1535419	1.03465058	-1.46190442	0.00070685	7165	5	370
NM_001012984	0.82090178	1.22706977	-1.90605704	0.002944318	6212	5	491
NM_001012984	1.04315215	1.18782354	-1.90605704	0.000605697	6164	5	848
NM_001008695	0.39523954	1.01162711	-1.06532297	0.004075543	8678	5	259
NM_001008695	0.33110103	1.06688401	-1.06532297	0.000554759	7256	5	160
NM_005052	-0.249947	1.45639173	-1.307360335	0.001236781	7002	5	684
NM_052844	0.32120759	0.70420796	-1.318638635	0.000162059	7121	5	137
NM_052844	0.13484904	0.75457325	-1.318638635	0.000169862	7120	5	158
NM_003213	1.00435333	0.50691905	-1.087368477	0.001918201	8776	5	832
NM_003213	1.01192371	0.5729554	-1.087368477	0.000890354	8777	5	876
NM_018944	0.55421949	0.71893277	-1.138325238	0.000698296	7318	5	460
NM_005187	0.0876818	0.42076175	-1.822581233	0.00905264	6687	5	713
NM_019113	0.78053477	1.36937637	-1.314336025	0.046444044	6386	5	1057
NM_019113	0.55705113	1.05372506	-1.314336025	0.027408924	6114	5	840
NM_181489	0.53239921	1.07744026	-1.683498138	0.002430898	7075	5	383
NM_181489	0.31568961	1.58537715	-1.683498138	0.001566102	9939	5	658
NM_016633	0.56992112	0.81116502	-1.436553436	0.001549084	8665	5	66
NM_016633	0.64616763	0.82591171	-1.436553436	0.000212688	7074	5	207
NM_004484	-0.2607347	1.69343607	-1.502932972	3.30E-05	6978	5	678
NM_024558	0.01982726	1.21981777	-1.372696652	9.38E-05	7087	5	402

NM_024558	0.19767971	1.24177793	-1.372696652	0.000733515	9951	5	231
NM_022640	0.11901848	0.63323619	-1.346433066	0.00317003	6735	5	472
NM_004944	-0.6635047	0.63239081	-1.806140794	0.015973586	6037	5	1151
NM_012234	0.93475031	0.82137168	-1.272896552	1.91E-05	8774	5	442
NM_012234	1.00694805	0.80349542	-1.272896552	7.88E-05	8775	5	565
NM_014519	-0.0129853	1.13432198	-1.139699126	0.000207824	7193	5	436
NM_003673	-0.0703376	1.62880822	-1.323880445	1.50E-05	9999	5	451
NM_001002252	0.30529516	1.12335776	-1.622982298	0.00048381	6341	5	8
NM_001002252	0.382743	1.42824941	-1.622982298	0.00085628	6307	5	91
NM_032440	0.39704922	0.74066628	-1.243795746	0.000320118	7122	5	184
NM_032440	0.32690131	1.11311269	-1.243795746	0.004068693	7229	5	537
XR_014513	0.05257169	1.11454431	-1.119869846	0.000281419	7201	5	331
NM_002611	0.05116456	0.61933791	-1.28618608	0.018165166	6110	5	613
NM_016337	0.26070888	1.48477536	-1.218206159	1.03E-05	7282	5	176
NM_001190	0.12364338	1.74586752	-1.341819943	0.000131911	10001	5	485
NM_016828	0.44317193	1.12581673	-0.861399122	0.029069215	8889	5	836
NM_016828	0.44074587	1.11165227	-0.861399122	0.023999472	8888	5	783
NM_014155	-0.5943187	1.04121144	-1.509883158	0.000514319	9731	5	1095
NM_197960	-0.2644474	1.10462849	-1.260305186	6.18E-05	7144	5	473
NM_172006	0.83519827	0.51338612	-1.483713329	0.021650633	6151	5	715
NM_172006	0.78087149	0.601962	-1.483713329	0.016245511	6150	5	563
NM_015896	0.11331923	0.78097003	-1.123688678	0.007381548	6932	5	408
NM_149379	-0.2350952	1.24018403	-1.278052476	0.000291171	7179	5	619
NM_149379	0.15808155	1.38846094	-1.278052476	0.000150373	9956	5	241
NM_005474	0.21504243	1.00493088	-1.173170976	0.000717371	7254	5	98
NM_005474	0.12609175	1.07758154	-1.173170976	5.69E-05	7252	5	95
XR_014021	0.83827728	0.91947005	-1.026875674	0.000378997	8816	5	514
XR_014021	0.8057739	0.97778665	-1.026875674	0.000147197	8817	5	488
NM_024927	0.05582536	1.27136933	-1.47204051	0.000224931	6996	5	104
NM_024927	0.10785357	1.41798869	-1.47204051	0.000458798	7025	5	329
NM_004756	0.35693787	0.41803588	-1.628355718	0.005858317	6714	5	589
CO644967	0.26149888	0.88795239	-1.230967323	4.30E-05	7124	5	102
CO644967	0.19394841	0.96351721	-1.230967323	6.81E-05	7125	5	97
NM_022822	0.02803329	0.81384782	-1.257680533	5.09E-05	7107	5	132
NM_001012509	0.89008613	0.42777617	-1.022569003	0.034447871	6196	5	1022
NM_001012509	1.15036353	1.24434509	-1.022569003	0.010673988	10430	5	1060
NM_173527	0.18308766	1.54453486	-1.237592762	0.037516381	10547	5	1062
NM_030768	0.27707586	1.06574209	-1.095055291	0.000701937	7131	5	375
NM_022817	0.74745917	1.82747144	-1.255213699	4.46E-05	10414	5	773
NM_022817	0.64972914	1.17849433	-1.255213699	0.002305119	7081	5	508
CN642591	0.78926138	0.9260202	-1.333612481	0.004220249	6357	5	248
CN642591	1.37793658	1.17116155	-1.333612481	0.003438925	6376	5	954
NM_003913	-0.0533579	1.26925411	-1.607017286	0.001013948	7016	5	411

NM_003913	-0.0383566	1.24321089	-1.607017286	0.001332459	7017	5	420
NM_015270	0.52556294	0.75489049	-1.033863629	0.003610703	8837	5	723
XR_013832	0.066204	0.57787558	-1.551550099	0.006431143	6529	5	492
XR_013832	-0.0550194	1.10924272	-1.551550099	0.004137973	6337	5	244
NM_005858	-0.1454684	1.2546911	-1.302361519	0.00019934	7180	5	445
NM_005858	0.08954517	1.39683883	-1.302361519	0.003242838	7032	5	671
NM_181805	-0.0837756	1.00076658	-1.304982658	1.43E-05	7149	5	217
NM_181805	-0.1960527	1.07717534	-1.304982658	2.31E-05	7147	5	352
NM_032467	1.34404121	1.0393322	-1.14625929	0.022965287	6372	5	1070
NM_201997	0.20716607	1.27893279	-1.335369184	4.86E-05	7000	5	46
NM_201997	0.0305356	1.61703982	-1.335369184	2.22E-05	10006	5	428
NM_013366	0.2206546	0.55105171	-1.229351713	0.02066417	8625	5	523
NM_013366	-0.0131561	0.99150449	-1.229351713	0.003260835	7241	5	249
NM_003279	0.07518743	1.35463278	-1.075480252	0.024956472	6121	5	960
NM_031945	0.94069767	0.34013587	-1.187649529	0.020318013	6195	5	970
NM_031945	1.09573582	1.15299253	-1.187649529	0.010833569	6371	5	974
NM_018259	0.05523301	1.25709798	-1.540942927	0.000145959	6995	5	52
NM_018259	0.15675608	1.45546281	-1.540942927	0.000146942	6997	5	105
NM_016162	0.0064854	0.82955677	-1.227112748	2.00E-05	7096	5	147
NM_024335	1.27396547	0.8636574	-1.270852777	0.0032058	6374	5	852
NM_024335	1.54708824	1.26393719	-1.270852777	0.003171654	6377	5	1054
NM_030964	0.48166102	0.13015663	-1.380042805	0.016928377	10849	5	1174
NM_005361	0.62559002	0.75992652	-1.263011224	0.003528663	6332	5	342
NM_005361	0.53631399	1.89843132	-1.263011224	0.012453815	6330	5	1040
NM_176800	-0.0334609	1.51751175	-1.536247148	0.000585959	9945	5	522
NM_176800	0.1579167	1.47728946	-1.536247148	0.001407764	9938	5	534
NM_152426	0.99766249	1.63370972	-1.148501742	0.02071197	10409	5	1140
NM_207033	0.05492149	0.74273663	-1.396997029	0.030711233	6112	5	774
NM_207033	0.19435329	0.93856472	-1.396997029	0.015535174	6109	5	700
NM_207033	0.4901908	0.96519115	-1.396997029	0.030847617	10364	5	1000
NM_207033	0.15702912	1.04253755	-1.396997029	0.031136544	10363	5	973
NM_207033	0.66219654	1.17366794	-1.396997029	0.039649999	10629	5	1163
XR_011719	1.12778497	0.16057434	-1.087049756	0.003688692	6191	5	1082
XR_011719	0.79928035	0.36071293	-1.087049756	0.004510958	6190	5	980
NM_017570	0.3118529	1.83997796	-1.370845273	0.002016716	10569	5	735
NM_017570	0.48053574	1.88808649	-1.370845273	8.25E-05	10570	5	680
U06694	0.33002912	1.22817771	-1.185024083	0.002855782	10367	5	481
U06694	0.33002912	1.33581534	-1.185024083	0.004594715	10368	5	666
XR_010623	-0.0357798	0.91734951	-1.338108979	0.00126994	6936	5	172
XR_010623	0.01857642	0.96248487	-1.338108979	0.000640575	6937	5	163
NM_004787	-0.1225445	1.19549278	-1.365355839	0.002626566	7035	5	672
NM_032151	0.39374388	1.01349507	-1.156229937	0.001864836	7079	5	388
NM_182982	0.35122532	1.05035197	-1.468598046	0.000687247	6342	5	22

NM_182982	0.06123297	1.19843627	-1.468598046	0.000115918	6994	5	75
NM_006917	0.31114356	0.93721751	-1.374547353	0.006417191	6111	5	257
NM_194281	0.25261833	1.16387626	-1.209416962	0.001476896	7230	5	374
NM_180990	0.45806715	1.16698263	-1.383618316	0.044792163	6385	5	975
XR_010245	0.2121107	0.78052825	-1.398852822	0.000799577	7119	5	151
XR_010245	0.37636671	1.19442092	-1.398852822	0.001244735	7076	5	192
NM_020145	0.19028871	1.05436657	-1.089579008	0.001236022	7255	5	191
NM_020145	0.21134934	1.13002388	-1.089579008	0.001496175	7261	5	190
NM_001546	-0.2396765	1.52718573	-1.421149567	0.000208129	7008	5	886
NM_139248	0.48418848	1.20101816	-0.991055256	0.000171395	7276	5	461
NM_015401	0.56874766	1.2921432	-1.126693507	0.000377723	7082	5	421
NM_004984	1.43198133	0.9562553	-0.634037104	0.008065153	9715	5	1141
NM_005314	-0.1067562	0.29234771	-2.291748451	0.044337359	5961	5	1166
NM_000066	0.71448355	1.64276165	-1.776109642	0.012112914	6388	5	1024
NM_000066	-0.4937643	1.39840193	-1.776109642	0.013588622	10358	5	1105
NM_014578	0.63859665	0.68371776	-1.043447662	0.002382436	8652	5	493
NM_025074	0.02291549	0.42699008	-1.653005553	0.004821131	6712	5	606
NM_025074	-0.0126178	1.2520637	-1.653005553	0.024610237	10359	5	1074
NM_000638	0.61924344	0.77249046	-1.328344961	0.012199918	8644	5	364
NM_000638	0.70008643	1.09691537	-1.328344961	0.008178539	6359	5	345
NM_004783	0.1003172	1.07200755	-1.149505385	1.42E-05	7126	5	157
NM_004783	0.37301854	1.18616033	-1.149505385	3.69E-06	7275	5	138
NM_002762	0.5107751	0.74534802	-0.996669272	0.012366802	8649	5	543
NM_020219	0.46540811	0.70485995	-1.187362304	0.014989151	8642	5	464
NM_020219	0.44329535	0.87980917	-1.187362304	0.006041584	8647	5	198
CK231504	0.56547677	0.96550142	-2.003926102	0.003824745	6206	5	478
CK231504	0.59060471	1.05463365	-2.003926102	0.003492777	6207	5	520
NM_182554	0.73485595	0.48053081	-1.617159532	0.005302773	6159	5	674
NM_182554	0.84689848	1.241907	-1.617159532	0.000560123	6255	5	623
NM_021570	0.22893417	1.2439765	-1.157973307	0.023478549	6120	5	909
NM_006062	0.47231447	0.89574572	-0.954131548	0.018020397	8650	5	603
NM_001792	1.07205814	0.46814767	-1.152669844	0.030999951	7324	5	1106
NM_033130	1.46120585	0.81623372	-1.084914989	0.043360058	6378	5	1133
NM_033130	1.39147436	0.72987881	-1.084914989	0.004417715	6375	5	1029
NM_153221	0.48989545	0.44259693	-1.204475964	0.018457774	8617	5	618
NM_153221	0.42867087	0.85677222	-1.204475964	0.010392558	8643	5	351
XM_375485	0.03514212	1.08202463	-1.262668578	0.00050846	7190	5	281
NM_001012505	0.32947525	0.89956843	-1.17589907	0.02021025	7307	5	767
NM_004959	0.54871041	0.68224239	-1.515604582	0.015378285	6148	5	397
NM_004959	0.7895225	1.1253377	-1.515604582	0.010610519	6350	5	502
NM_020693	0.59850165	1.03678914	-1.326510298	0.004322621	6358	5	154
NM_020693	0.56015341	1.1464468	-1.326510298	0.00106694	6356	5	72
NM_145735	1.1597243	1.0429038	-0.983055131	0.005993016	11237	5	1243

NM_138360	0.33857663	1.21425677	-1.409914486	0.000979139	6334	5	142
NM_004506	0.42038752	1.25786922	-1.106382495	0.006908539	10369	5	714
NM_175569	0.36692807	1.62284367	-1.544157257	0.002857432	10375	5	750
NM_001011667	0.15301536	0.79008672	-1.271638609	0.003681806	7220	5	389
NM_003674	-0.1314472	1.16720549	-1.141415478	1.90E-05	7154	5	315
NM_003674	-0.1747684	1.28601289	-1.141415478	4.89E-05	7155	5	439
NM_004127	0.28533206	1.29859823	-1.070809506	0.00144574	7264	5	302
NM_004127	0.30390282	1.51319252	-1.070809506	0.00135506	7265	5	474
NM_013412	0.40613305	0.73572387	-1.162111016	0.02937864	8645	5	577
NM_013412	0.47810941	0.88798198	-1.162111016	0.011264937	8646	5	333
NM_024342	0.4959718	0.622048	-1.103080555	0.008296844	8651	5	471
NM_024342	0.61826326	0.78959922	-1.103080555	0.008179726	8656	5	424
XR_014033	0.19068231	1.20922192	-1.154659664	0.001159739	7262	5	146
XR_014033	0.16523738	1.27759608	-1.154659664	0.000842918	7263	5	206
NM_144674	0.41527451	1.26395475	-1.398776393	0.003632449	6344	5	167
NM_144674	0.14489949	1.84586845	-1.398776393	0.005115653	10612	5	921
NM_178837	0.35715466	0.90122417	-1.288882044	0.00708533	10366	5	595
NM_014226	0.46020335	1.1690008	-1.258703173	0.000763038	7274	5	156
NM_014226	0.46157761	1.36550566	-1.258703173	0.000139084	7287	5	119
NM_017767	2.37534926	1.05587027	-2.994729736	0.048367337	11238	5	1240
NM_000151	0.97877823	1.20857351	-1.35040014	0.001908768	6317	5	497
NM_000151	0.14510681	1.61705572	-1.35040014	0.003261267	10558	5	673
NM_032300	0.86315227	1.58549113	-1.127546111	0.000126493	10415	5	884
NM_207373	0.58663731	1.83367418	-1.402130532	0.005700562	10572	5	904
NM_003854	0.11748322	0.26418129	-1.730536555	0.016107813	6524	5	931
NM_004296	0.54376236	1.14227723	-1.633453038	0.008933426	6349	5	376
NM_001001936	0.14521245	1.23863975	-1.196105587	0.000835766	6338	5	384
NM_018052	0.57438876	0.65345942	-1.058124706	0.024384603	8654	5	648
NM_021954	0.42184323	0.22236848	-1.422147662	0.008630862	6530	5	870
NM_021954	0.41155475	0.48431018	-1.422147662	0.007199671	6525	5	515
NM_057162	-0.1805019	-0.1744008	-1.925786658	0.028275002	5952	5	1128
NM_057162	-0.1068751	-0.0646114	-1.925786658	0.026419717	5953	5	1111
NM_144621	0.13519874	1.21107479	-1.285676201	0.000412328	7192	5	221
NM_144621	0.29574303	1.30789667	-1.285676201	0.000540029	9962	5	234
NM_022551	0.35606243	1.0631718	-1.00428548	1.56E-05	8290	5	284
NM_022551	0.38026256	1.0788062	-1.00428548	7.40E-05	8291	5	321
NM_019052	-0.1034451	0.82435927	-1.289026679	0.000335434	7093	5	298
NM_152775	0.32096263	1.10391538	-1.059996201	7.95E-05	7203	5	378
CO579632	0.41636221	0.84613232	-1.161762239	0.000952589	7123	5	314
NM_003180	0.51617906	1.20936798	-1.127688387	0.001623322	10370	5	468
NM_002209	0.6823345	1.21502495	-0.774809405	0.008550482	9648	5	847
A_01_P000129	0.12706064	1.06854559	-1.612679796	0.010570981	6333	5	579
A_01_P000129	1.07297531	1.32807138	-1.612679796	0.007561555	5999	5	1085

NM_022066	0.94687117	1.01953041	-1.130256492	0.004323661	6363	5	636
NM_022066	0.87429681	1.27406454	-1.130256492	0.004062598	6361	5	665
A_01_P019537	3.56331091	1.13643255	-2.148621481	0.027601834	11251	5	1245
A_01_P019537	4.24558832	1.3472412	-2.148621481	0.032406118	11252	5	1247
NM_198534	0.97634275	1.21537843	-1.234576643	0.020844958	6370	5	908
NM_198534	0.86816742	0.93090033	-1.234576643	0.006091639	6362	5	518
NM_005185	0.35475736	1.44451097	-1.425742868	0.002613036	6345	5	303
NM_005185	0.13877365	1.19178249	-1.425742868	0.002519878	6343	5	226
NM_152283	0.70413685	0.88660096	-1.08240319	0.000942067	8789	5	554
NM_173525	0.96003471	1.98862011	-1.533447278	0.031755198	6389	5	1185
NM_001091	0.56373505	0.58946373	-1.336479875	0.033066409	6113	5	802
NM_032637	0.59761446	1.3824143	-1.071069464	4.19E-05	7278	5	487
NM_007351	0.34385596	1.6021137	-1.962061059	0.006078218	6217	5	874
NM_024527	0.8217302	1.03274816	-0.818152543	0.044801491	8769	5	981
NM_024527	0.69665084	0.98148706	-0.818152543	0.019388166	8768	5	811
NM_207429	0.61604527	1.0825086	-1.287412855	0.002130956	6365	5	410
XR_010253	-0.00061	1.14690041	-1.205521764	0.000390128	7189	5	225
XR_010253	-8.73E-05	1.08529667	-1.205521764	0.000290758	7188	5	232
NM_005251	-0.177506	1.43187715	-2.046503929	0.021662269	6004	5	1109
NM_005251	-0.0104915	0.56238535	-2.046503929	0.014434698	6523	5	994
NM_152572	0.6300674	0.76473877	-0.995268725	0.018105941	8655	5	642
NM_152572	0.69863542	0.85993938	-0.995268725	0.005837193	8657	5	545
NM_013290	0.10414924	1.01652283	-1.199521841	0.00185217	7191	5	425
BC108680	0.70994922	0.55042961	-1.032733536	0.007948913	8653	5	660
NM_019099	0.50817656	1.09940724	-1.278448822	0.002281647	6355	5	139
NM_019099	0.75288848	1.53055832	-1.278448822	0.001465212	6322	5	557
NM_080385	0.62554328	1.34916393	-1.621771817	0.003740247	6213	5	396
NM_080385	0.96392548	1.69422644	-1.621771817	0.00181228	10618	5	859
NM_080646	0.51354043	0.86740927	-1.102179475	0.002519112	8648	5	242
AF207834	0.77793141	0.45594775	-1.629775116	0.015843639	5965	5	831
AF207834	0.67642723	1.19099332	-1.629775116	0.003464376	9851	5	771
NM_006657	0.63809665	0.92389192	-1.724103943	0.034573966	6125	5	956
XR_014721	0.89884029	0.93000132	-1.507540614	0.021531193	6163	5	828
XR_014721	0.78196206	0.7743508	-1.507540614	0.020535633	6162	5	925
CK231784	1.56697179	1.35883117	-0.839876646	4.21E-05	10432	5	1129
CK231784	1.32336957	1.31734891	-0.839876646	0.000535063	10431	5	1073
NM_014994	0.27922767	0.50701929	-1.462681311	0.019014929	6532	5	730
NM_014994	-0.2241823	0.76522909	-1.462681311	0.005065453	6863	5	602
NM_014485	0.29865905	0.84774002	-1.105613549	0.00984988	8860	5	558
NM_005091	0.59283517	1.31866429	-1.717636979	0.009749025	6218	5	651
NM_005091	0.72188761	1.60834588	-1.717636979	0.008553989	6219	5	893
A_01_P013029	0.36679841	1.03977132	-1.529102737	0.00532191	6339	5	250
NM_022134	0.39165699	0.32699253	-1.902312322	0.040011998	5964	5	1081

NM_178160	0.64677923	0.7626175	-0.991614654	0.00270139	8802	5	524
NM_178160	0.76256404	1.0890371	-0.991614654	0.001103431	9691	5	760
XM_376007	0.70686893	0.98577953	-1.258727345	0.02432296	6367	5	826
NM_145652	0.67336773	1.10810035	-0.943404461	0.014235551	9693	5	721
NM_005750	1.07740292	0.45549564	-0.891344462	0.021412403	9630	5	1142
NM_198476	0.22653668	1.31395456	-1.046197554	0.012187756	8895	5	667
NM_198476	0.66838081	0.92257682	-1.046197554	0.005185964	6360	5	566
NM_145170	0.32679559	1.54994321	-1.418739267	0.007691527	10614	5	775
NM_020427	0.26236804	1.2950554	-0.990190844	0.00232306	8863	5	629
NM_001004745	0.72583603	1.00013319	-1.094228561	0.03919025	10365	5	1044
NM_057157	2.88173229	1.16811808	-2.006833253	0.047141921	11240	5	1236
NM_181646	0.92750968	1.43453334	-0.933194868	0.010477039	9689	5	1008
NM_181646	1.34406127	0.82547278	-0.933194868	0.001216467	9643	5	1027
NM_175923	0.87859538	1.00918693	-1.238049117	0.049261485	6368	5	1019
NM_030971	0.72836886	1.25369619	-1.234037491	0.020968712	6351	5	789
NM_030971	0.93020421	1.32532431	-1.234037491	0.009535194	6323	5	787
XR_010711	1.04536724	1.49217591	-1.472775767	0.018894377	10617	5	1042
XR_010711	1.391303	1.81902867	-1.472775767	0.002291078	10622	5	1138
XR_014054	0.26795677	0.91255918	-1.133643526	0.045705679	6614	5	972
NM_021224	0.36264892	1.03644215	-1.376249344	0.005390597	6340	5	357
NM_021224	0.44394885	1.74158109	-1.376249344	0.010756087	10613	5	992
NM_173659	0.26206382	0.90647682	-1.225392732	0.0118024	6348	5	429
NM_173659	0.12072169	0.93788617	-1.225392732	0.007467627	6347	5	392
NM_000119	0.37636119	1.01878677	-1.18207817	0.010673909	6336	5	572
NM_001003794	0.58204356	1.21116166	-0.922427358	0.004456504	9694	5	628
NM_001003794	1.07079915	1.22217734	-0.922427358	0.00282021	9697	5	903
NM_144705	0.47677325	0.63393025	-1.14273296	0.043929207	6364	5	906
NM_138697	0.61545423	0.82311095	-1.026190871	0.004269937	8780	5	751
NM_020813	0.21402518	0.29079134	-1.334232201	0.039525738	6526	5	920
NM_181449	0.41966391	0.53918426	-1.169658178	0.039072027	6331	5	855
NM_130900	0.2143346	0.80067816	-1.224869752	0.042763695	6346	5	812
NM_130900	0.50021912	1.11285407	-1.224869752	0.024147339	6352	5	717
NM_013351	0.54839008	0.96729561	-1.171984444	0.032518383	6353	5	738
NM_013351	0.48822771	1.1709032	-1.171984444	0.015013072	6354	5	601
NM_018231	0.92118637	0.97315917	-0.93302755	0.031558877	9695	5	1010
NM_018231	0.58267763	1.05085558	-0.93302755	0.009728417	6366	5	752
XR_010916	0.75893584	0.65640481	-1.010322141	0.016955049	8741	5	777
NM_001282	0.73613997	1.23689199	-0.986109926	0.007880831	10410	5	761
NM_025168	-0.1216414	1.04980222	-1.180616047	0.022143361	6537	5	873
NM_033310	1.13319129	1.59969905	-0.971826443	0.004824568	10619	5	1046
NM_002777	2.29087498	1.85975545	-0.949335069	0.015013466	10878	5	1223
NM_152731	-0.6021164	1.19130896	-1.769832195	0.041707372	6044	5	1188
XM_291277	1.03356216	1.07860853	-0.766426338	0.029975746	9696	5	1041

NM_182828	0.44984375	1.62318509	-1.246187396	0.028383113	10616	5	1050
NM_173815	2.48520018	1.45121973	-0.541888514	0.009742658	11260	5	1244

Cluster 4

Gene Name	Av Normal	Av M (w12)	Av M (w4)	*P*	Hierarchical Clustering (order)	K-means clustering	K-means clustering (rank)
NM_152390	-0.7805672	1.55556836	-1.27638708	0.007007491	6051	4	1111
NM_152390	0.41298825	1.46412065	-1.27638708	0.037060485	10625	4	1116
XR_013481	-0.9360774	1.54220351	-1.231266492	0.013853962	6050	4	1163
NM_004378	1.47427342	2.01589995	-0.877415705	0.027106408	9723	4	1199
NM_012467	1.10319246	1.87010863	-0.897026124	0.01728533	9722	4	1140
NM_152474	0.15197552	1.43779696	-1.110657018	0.012950127	8957	4	904
NM_021634	-0.215687	1.24755488	-0.736709695	0.039568376	8901	4	905
NM_001556	-0.1402756	1.39647528	-1.372071688	0.016864729	10382	4	1081
NM_033258	-0.3326108	1.82590027	-1.124380447	0.011700579	9737	4	1012
XM_371146	-0.486179	1.76048076	-1.229232652	0.003864656	9859	4	859
NM_006105	0.00930778	2.0437009	-1.333639076	0.002553756	9930	4	837
NM_006105	-0.0971277	1.91316467	-1.333639076	0.003035616	9929	4	806
NM_174923	-0.2482866	1.69153778	-1.170259938	0.000925247	9927	4	520
NM_174923	-0.1347382	1.79855908	-1.170259938	0.001541509	9928	4	536
NM_080283	-1.2310964	1.65070918	-1.798487329	0.021691559	6042	4	1213
NM_002641	-0.237734	1.70217837	-1.589155854	0.008771223	9876	4	1071
NM_014648	0.02377581	1.85129219	-1.379557093	0.030926718	10624	4	1150
NM_005588	0.27728885	1.96214277	-1.11500209	0.01261299	10549	4	1156
NM_005588	0.22692947	2.48047701	-1.11500209	0.006725451	11178	4	1126
NM_018069	-0.335013	1.83119534	-1.480796845	0.000647562	9895	4	846
NM_152643	0.29344765	2.35907668	-0.957770601	0.036767731	11179	4	1184
NM_001002036	0.34623537	1.32475605	-0.925551039	0.017772789	6119	4	901
NM_001002036	0.29352215	1.29838087	-0.925551039	0.015843396	6118	4	862
NM_017786	-0.5810288	3.33042635	-0.601650027	0.004181157	11188	4	1201
NM_001835	-0.4719184	2.31448466	-1.424578022	0.001631065	9857	4	1100
CO725485	0.14419355	1.82998703	-1.294536736	0.001474091	10564	4	726
CO725485	0.03920594	1.89165496	-1.294536736	0.001256972	10563	4	710
NM_001006655	-0.4146768	1.46392641	-1.173628515	0.010057413	6668	4	992
NM_020927	1.16499341	1.79058125	-0.937606741	0.003322274	9721	4	1075
NM_138414	-0.1061102	2.09523393	-1.438099542	1.95E-05	10052	4	686
NM_014786	-0.3406531	2.00574757	-1.565310443	1.88E-05	10213	4	872
A_01_P000370	-0.4868579	1.40730103	-1.251769125	0.000354784	6945	4	822
NM_020796	-0.4137915	1.64248384	-1.501630002	0.001827296	9865	4	949
NM_017434	0.30566215	1.51821023	-0.936100443	0.004427513	7301	4	570
NM_017434	0.65067594	1.77107705	-0.936100443	0.024878692	10433	4	1056
NM_201994	0.37572535	1.44427227	-1.247970117	0.027605178	10385	4	1064
NM_020847	1.00568047	1.96241082	-1.079059099	0.012721109	10434	4	1134
NM_001631	-0.0244208	1.92600095	-1.406775365	0.000933072	10562	4	811

NM_178502	-0.4504242	1.62420338	-1.391012913	4.65E-05	6980	4	774
NM_178502	-0.6029418	1.70109031	-1.391012913	5.44E-05	9885	4	860
NM_017664	-0.5574347	1.93271205	-1.591012524	8.68E-06	9886	4	968
NM_005399	-1.9465615	1.88020192	-1.381871613	0.000169104	6025	4	1229
NM_005399	-0.0472071	1.3148728	-1.381871613	0.028011048	10380	4	1091
NM_144679	-0.2996255	1.83912415	-1.406270626	0.000128102	9891	4	721
NM_144679	-0.3635391	1.92269433	-1.406270626	2.42E-05	9901	4	681
NM_024669	-0.3589128	1.31391642	-1.164079334	0.000850687	7156	4	658
NM_024669	-0.0523633	1.52523387	-1.164079334	0.00254514	9181	4	515
XR_011718	-0.3687566	2.0524564	-1.546381881	0.000630861	10207	4	1002
NM_002292	-0.3684235	1.97821654	-1.479134574	8.70E-06	9890	4	810
NM_002292	-0.3320341	1.8882942	-1.479134574	9.35E-07	9889	4	752
NM_005231	-0.2122456	1.81768854	-1.522552027	6.36E-05	9985	4	743
NM_005231	-0.1252541	1.85162788	-1.522552027	0.000166133	9986	4	747
NM_005231	0.05559154	1.95422836	-1.522552027	0.000151205	9993	4	757
NM_021005	0.13518354	1.41678082	-0.848270671	0.013241759	8967	4	538
NM_021005	0.02161368	1.30727215	-0.848270671	0.0315256	8966	4	844
NM_174976	-0.849586	1.72602373	-1.252399724	0.014823391	6047	4	1205
NM_032709	-0.353958	1.9324127	-1.314778282	7.21E-05	9902	4	574
NM_213674	-0.4299886	2.40278867	-1.123937202	0.000395224	10719	4	830
NM_213674	-0.3312246	2.63324803	-1.123937202	0.000304828	10720	4	937
NM_013360	-0.9154536	1.66986829	-1.171565349	0.000239082	9858	4	927
NM_000458	-0.3497343	1.48840483	-1.508907167	0.005764942	9874	4	1019
NM_031207	-0.3451018	1.50959364	-1.224039689	4.78E-05	6982	4	551
NM_031207	-0.3700806	1.54738681	-1.224039689	5.18E-05	6983	4	546
NM_001001974	0.37724039	1.18284085	-0.980997754	0.012073236	8963	4	783
NM_015115	-0.0991756	1.4863194	-0.995164404	0.020374998	10383	4	762
NM_006006	0.21003457	1.93543945	-1.361901949	0.000335407	10028	4	585
NM_006006	0.12279349	2.27979766	-1.361901949	0.000861394	10333	4	823
NM_016333	0.02815205	1.94112394	-1.247556551	2.30E-05	10012	4	390
NM_016333	0.00411606	1.77416083	-1.247556551	0.000217279	10061	4	377
NM_006647	-0.1974984	1.85365091	-1.254490805	9.40E-05	10010	4	466
NM_006647	-0.4918213	2.20943414	-1.254490805	0.0002089	9906	4	839
NM_030781	0.04391676	2.01598924	-1.505247482	8.19E-05	9994	4	724
NM_006696	-0.2398903	2.01865899	-1.42606692	4.61E-05	9903	4	668
NM_006696	-0.1410582	1.89388448	-1.42606692	0.000386512	9987	4	670
NM_004396	-0.6537676	1.57776995	-1.548186866	0.009285109	9873	4	1115
NM_004396	-0.4797836	1.94156203	-1.548186866	0.003669995	9871	4	1050
CO582642	0.0729865	1.87710904	-1.521045224	0.000218756	9992	4	737
NM_003319	-0.9328498	2.43465564	-1.11689532	0.003588374	10687	4	1149
CK231263	0.12843958	1.76593594	-1.020704039	0.000272413	9241	4	127
CK231263	0.08688177	1.62872223	-1.020704039	0.001267712	9182	4	204
CK231263	-0.0474845	1.9840526	-1.020704039	0.000541844	10075	4	217

CK231263	0.06359754	2.03757691	-1.020704039	0.000345563	10076	4	186
XR_014480	-0.3814351	1.4672246	-1.289588922	8.89E-06	6981	4	638
XR_014480	-0.4503104	1.86535381	-1.289588922	1.74E-06	9899	4	602
NM_002217	-0.4836626	1.9367348	-1.488619938	9.72E-06	9897	4	842
NM_032937	0.85041731	2.04011239	-0.716014786	0.009128318	10781	4	1080
NM_017822	0.35593244	2.03030338	-1.354057891	3.01E-06	10087	4	622
NM_017822	0.27800625	2.05331246	-1.354057891	4.48E-05	10085	4	595
NM_152449	0.10564163	1.67150844	-1.358043034	0.000253566	10014	4	562
NM_152449	0.17783934	1.95589879	-1.358043034	0.007592191	9843	4	951
NM_020672	0.19048485	1.55931371	-1.186753054	0.000166226	9183	4	331
NM_020672	0.32500874	1.57329642	-1.186753054	0.000899317	9184	4	474
NM_001945	-0.190587	1.48038795	-1.229018815	0.000287855	6984	4	561
NM_015330	-0.1257151	1.81733528	-1.448974957	3.41E-06	9984	4	641
NM_015330	-0.000379	1.93442314	-1.448974957	8.33E-06	9990	4	609
NM_000954	-0.8119435	1.41906839	-1.263367592	0.001030435	6947	4	1030
NM_002744	-0.0672179	2.08893176	-1.44118654	9.46E-06	10220	4	694
NM_002744	0.03295685	1.95835677	-1.44118654	2.01E-05	9991	4	615
NM_052902	-0.1781527	1.39898249	-1.226888095	0.000451056	7028	4	568
NM_052902	-0.0842674	1.40048044	-1.226888095	0.00033243	9179	4	485
NM_052897	-0.0028455	1.6257293	-1.057424153	0.000256589	9240	4	190
NM_003283	0.21009045	1.39982275	-0.930526165	0.000744331	7298	4	291
NM_033396	-0.3185551	1.30357705	-1.046458735	0.001430848	6952	4	642
NM_015363	1.18932651	2.5655584	-0.607229576	0.013118161	10835	4	1180
NM_015205	-0.0070193	1.69695829	-1.259952055	0.000979465	10015	4	500
NM_015205	-0.1004745	1.82135738	-1.259952055	0.001588665	10035	4	547
NM_015205	-0.070716	1.75448943	-1.259952055	0.002516739	10033	4	654
NM_020214	0.19287786	2.04568416	-1.330561022	8.82E-07	10083	4	509
NM_020214	0.21143126	2.05689387	-1.330561022	1.83E-05	10086	4	550
NM_007078	-0.1108952	1.87013477	-1.289659352	0.000152213	10062	4	472
NM_004424	-0.2730431	1.93018741	-0.948567683	0.000474852	9251	4	303
NM_004424	-0.4793572	1.79431483	-0.948567683	0.001293474	9923	4	483
NM_012240	-0.0308232	1.80252543	-1.215370134	0.000377659	10037	4	431
NM_004830	-0.1361738	1.99485589	-1.390631371	0.022799497	10387	4	1138
NM_006329	-0.3683631	2.50938898	-1.11720734	8.53E-05	10721	4	845
NM_006329	-0.424768	1.82987765	-1.11720734	0.000984738	10040	4	553
NM_001847	0.07853791	2.24379908	-0.899661613	0.014894788	10393	4	921
NM_014234	-0.160043	1.25911208	-1.04153134	0.000239441	7159	4	491
NM_014234	-0.1118928	1.31154015	-1.04153134	0.00015479	7162	4	372
CN643612	0.06447294	1.57801254	-1.368190861	0.000382189	9180	4	582
CN643612	0.08729606	1.91566113	-1.368190861	0.001997221	10036	4	722
NM_001920	0.24431762	2.045591	-1.443499621	5.82E-05	10031	4	692
NM_015541	0.2020654	2.04264036	-0.996855901	0.00029987	10077	4	202
NM_015541	0.02013914	2.7059493	-0.996855901	0.000293936	10330	4	849

NM_001009820	-0.3952872	1.29307423	-1.117053315	0.000530493	6951	4	675
NM_003019	-0.1657249	2.18246787	-1.280649049	5.90E-07	10054	4	531
NM_003019	-0.173082	2.08605086	-1.280649049	2.59E-05	10053	4	482
NM_004689	0.01060112	1.56425274	-1.270404607	1.16E-06	10004	4	385
NM_004689	-0.0686047	1.64671135	-1.270404607	8.16E-07	10005	4	357
NM_015949	-0.4835208	1.71491009	-1.284684373	0.000450564	9898	4	685
NM_015949	-0.2903386	2.10924169	-1.284684373	0.009146558	10388	4	1007
NM_002224	0.03358537	1.94114526	-1.420133404	2.33E-05	9996	4	577
NM_002224	0.16160213	1.95798416	-1.420133404	6.42E-05	9998	4	606
CR604926	-0.7564734	1.74431854	-0.71854621	0.003400335	9398	4	716
CR604926	-0.3433436	2.06496579	-0.71854621	0.002236376	10725	4	427
CR604926	-0.8678678	2.66683081	-0.71854621	0.001679367	10691	4	1109
CR604926	-0.2345839	2.45083895	-0.71854621	0.001385142	10726	4	635
NM_144697	-0.0911159	1.90206903	-1.391235975	0.000250867	9988	4	618
NM_019012	-0.3358577	1.80749628	-1.48694905	0.002234273	9872	4	916
NM_020870	-0.0721724	2.07887111	-1.135981047	0.008910054	10392	4	913
XR_011805	-0.1314724	1.74043188	-1.370133352	0.001265483	10034	4	652
XR_011805	-0.1837735	1.65823833	-1.370133352	0.00794286	9875	4	906
NM_032531	0.9094543	2.05046169	-0.761148409	0.012472649	9720	4	1070
NM_139067	-0.0167476	1.76895207	-1.373129912	1.67E-06	10008	4	495
NM_005407	-0.0912914	1.3874166	-1.200751563	0.001402046	7036	4	640
NM_000898	0.32403491	1.83766193	-1.194683511	0.01319883	10402	4	948
NM_020349	-0.0625307	1.45015245	-0.740331837	0.022873316	8873	4	809
NM_020349	0.15289704	1.41719851	-0.740331837	0.002499867	7300	4	383
NM_032870	-0.2784672	2.17955727	-1.086490896	0.000188738	10043	4	437
NM_032870	-0.3741532	2.27409614	-1.086490896	0.000232961	10042	4	597
NM_001005368	0.37662656	1.73622494	-1.312086063	0.000376233	10027	4	607
XR_013572	-0.4201473	2.00459348	-1.574941979	9.60E-05	9887	4	961
NM_003565	-0.0407834	2.01736793	-1.235007768	3.66E-05	10055	4	371
NM_003565	-0.0387761	2.09430668	-1.235007768	7.59E-05	10056	4	434
NM_012207	-0.1470385	1.26685901	-1.275059363	0.034698178	10381	4	1079
NM_001512	-0.0732757	1.93983786	-0.929516824	0.000478913	9252	4	211
NM_001512	0.23774217	2.21882543	-0.929516824	0.003300375	10712	4	678
NM_003250	0.07030858	1.25952859	-0.750193931	0.012680943	8870	4	662
NM_005777	0.34182379	1.76907085	-1.24770601	1.12E-05	10097	4	399
NM_020433	0.19964222	1.71457613	-0.744739621	0.010322261	9367	4	439
NM_020433	0.32146807	1.93760224	-0.744739621	0.008219874	9368	4	498
NM_017633	0.0997621	1.44910958	-1.131178995	0.001492973	7273	4	529
NM_002389	-0.3534608	1.90244134	-0.660139702	0.004654983	9860	4	416
NM_002389	-0.318399	2.61262901	-0.660139702	0.001548028	10745	4	865
NM_002660	0.11940916	1.48822217	-1.139448863	5.57E-06	7283	4	272
NM_002660	0.26584905	1.67812488	-1.139448863	7.17E-06	9246	4	223
NM_018025	0.15458472	1.36770205	-0.809651155	0.001309605	7299	4	235

NM_000309	0.20117647	1.86672366	-1.380830547	3.17E-05	9997	4	537
NM_000309	0.35267206	1.94083989	-1.380830547	3.44E-05	10084	4	625
NM_003611	-0.1230359	1.86380077	-1.236937937	0.00033755	10049	4	404
NM_003611	-0.0520202	1.9105668	-1.236937937	0.000197673	10063	4	363
NM_017791	0.27508229	2.47597404	-1.24732273	0.011178527	11194	4	1118
NM_030665	-0.0894172	1.8378938	-1.070258146	0.020764023	10662	4	1048
XR_012522	0.33135493	1.79761869	-1.363254727	0.000122032	10029	4	567
XR_012522	0.44095669	1.85276359	-1.363254727	0.000410357	10030	4	687
NM_025215	0.16426243	1.69644378	-1.024956261	0.00013912	9244	4	154
NM_025215	0.18426852	1.5196024	-1.024956261	0.000263231	9242	4	234
NM_006784	0.20840477	1.97981183	-1.288901907	1.53E-06	10082	4	457
NM_006784	0.06906791	1.97468871	-1.288901907	6.49E-06	10081	4	413
NM_153213	0.21130835	2.16140649	-0.96923811	7.29E-05	10092	4	252
NM_153213	0.16769176	2.37866297	-0.96923811	0.000101586	10117	4	435
NM_022640	-0.0757519	2.20555469	-1.346433066	0.013225875	10542	4	1148
BX649142	-1.792654	2.1945912	-1.286116328	0.016167598	6048	4	1230
NM_006760	0.64814726	2.60489825	-0.880652489	0.007614914	11224	4	1153
NM_003673	0.13803636	1.71781734	-1.323880445	4.11E-06	10007	4	432
NM_144994	-0.3168143	1.72316263	-1.229676041	3.71E-06	9900	4	418
NM_144994	0.04916662	2.08795272	-1.229676041	7.77E-06	10057	4	366
XR_014513	-0.1328707	1.48642348	-1.119869846	0.000396215	6986	4	481
NM_016337	0.0464635	1.86865904	-1.218206159	6.62E-08	10009	4	260
NM_017810	0.23154807	1.45100223	-0.849870078	0.003071688	8924	4	398
NM_001190	0.11876717	2.00434842	-1.341819943	6.26E-06	10080	4	535
NM_080625	-0.739667	2.07189021	-0.875671281	0.002015415	10670	4	924
NM_080625	-0.2667366	2.16678083	-0.875671281	0.001031153	10045	4	424
XR_014848	0.12540804	1.42612812	-1.13128969	5.76E-05	9198	4	317
XR_014848	0.12304816	1.49323637	-1.13128969	0.000198679	9200	4	266
NM_022118	-0.5864893	1.69682318	-1.317167554	0.00263574	9878	4	888
NM_022118	-0.7492659	1.68023728	-1.317167554	0.006076924	9877	4	1043
NM_003259	-0.3254869	1.50734858	-0.63833998	0.029055992	6536	4	884
NM_014270	0.0899157	2.13295058	-1.171106197	0.005425404	10039	4	796
NM_000906	0.13979337	2.08037543	-1.173558635	2.29E-06	10088	4	347
NM_000906	0.15456851	2.62666644	-1.173558635	2.03E-07	10345	4	819
NM_148916	0.80824536	1.26557664	-0.617586245	0.034627487	9653	4	1061
NM_000805	0.91732195	2.22420417	-1.338122286	0.000136311	10577	4	1073
NM_032951	0.2564127	2.07715589	-1.129920419	1.79E-06	10091	4	340
NM_032951	0.46284301	2.34690556	-1.129920419	3.35E-06	10123	4	653
XM_496500	-0.5116393	2.29048401	-0.536027004	0.005975084	10679	4	915
A_01_P018381	0.05202311	1.52628358	-1.09003799	0.003598034	9178	4	449
A_01_P018381	0.0583764	1.8108255	-1.09003799	0.011588576	10390	4	784
NM_182485	-0.1636347	1.7215699	-0.487256195	0.028442632	10673	4	740
NM_024315	-0.1528793	2.26374497	-1.167463287	5.73E-05	10058	4	506

NM_024315	-0.2065113	2.51594038	-1.167463287	0.001958867	10331	4	938
NM_016316	0.24301255	2.30725824	-1.334758555	8.18E-05	10342	4	755
NM_022760	-0.1696736	1.82347224	-1.148852676	2.56E-05	10011	4	245
NM_022760	-0.0824266	1.95302312	-1.148852676	7.88E-05	10064	4	242
NM_001967	0.50129149	2.23998625	-1.173673686	8.46E-05	10425	4	628
NM_001967	0.54413008	2.20584944	-1.173673686	0.000318331	10426	4	690
NM_018262	-0.0547069	1.37750639	-1.070057034	3.35E-06	7163	4	270
XR_010365	-0.1802358	1.26212632	-0.971473369	0.001641992	8208	4	503
XR_010365	-0.3349258	1.25687675	-0.971473369	0.015319719	8953	4	800
NM_003279	0.24382797	1.9075247	-1.075480252	0.017627329	10666	4	1031
NM_032328	-0.2342725	2.09633199	-0.938744183	0.003304253	10406	4	510
NM_003811	0.69747362	1.2424344	-0.564025854	0.02186134	9651	4	931
NM_020856	0.28652324	1.53202006	-1.143180107	0.000880717	7288	4	486
NM_020856	0.24154053	1.68804446	-1.143180107	0.001685947	10401	4	683
NM_199184	0.63170259	1.34581039	-0.568052345	0.017923555	9661	4	975
NM_014906	0.40807333	2.55712098	-0.693156503	0.036199653	10826	4	1182
NM_183353	0.42163147	2.21401816	-0.426080328	0.002806207	10782	4	708
NM_183353	0.238746	1.95365672	-0.426080328	0.00780547	9616	4	365
NM_183353	0.69772887	3.23327479	-0.426080328	0.026107839	11205	4	1210
NM_183353	0.33454558	3.03208106	-0.426080328	0.042185146	11204	4	1204
NM_144775	0.38680719	1.40352303	-0.533379421	0.0444687	9664	4	933
NM_013293	-0.1593592	1.46989977	-1.323245282	0.000439733	7029	4	608
NM_013293	-0.3079877	1.69388836	-1.323245282	0.001105203	9881	4	671
NM_014582	0.35569983	1.57941372	-0.078769053	0.049868764	9615	4	871
NM_003717	0.12200728	1.50901532	-0.917251775	0.000867791	7269	4	262
NM_003717	0.1848031	1.26428944	-0.917251775	0.000422303	8329	4	352
NM_003257	0.19950614	1.66866851	-0.988728474	0.001974897	9185	4	220
NM_003257	-0.2518973	1.99965772	-0.988728474	0.001811982	10041	4	455
NM_005244	0.40282478	1.83852403	-0.976210991	2.22E-05	10107	4	184
NM_005244	0.36685109	1.50070398	-0.976210991	0.001332431	9214	4	348
NM_181870	0.13129128	1.85405461	-1.070524199	5.84E-05	10013	4	156
NM_181870	0.10650229	1.69187353	-1.070524199	1.70E-05	9245	4	111
NM_181870	-0.027683	1.6588978	-1.070524199	0.000481608	9190	4	185
NM_181870	0.23620507	1.98337495	-1.070524199	4.32E-05	10102	4	172
NM_004787	0.15220936	1.62530475	-1.365355839	0.000569716	9948	4	659
NM_032151	1.66409016	2.42969586	-1.156229937	0.000481537	11212	4	1215
NM_021167	-0.2848019	1.58483345	-1.334383281	1.69E-05	6979	4	596
NM_021167	-0.0806628	1.60637896	-1.334383281	7.86E-05	6977	4	516
NM_000977	-0.0814901	2.187599	-1.044578452	7.58E-05	10059	4	250
NM_000977	-0.1439195	2.33708457	-1.044578452	3.20E-05	10060	4	426
NM_020166	-0.0970089	2.02592521	-1.228694091	0.000477584	10051	4	463
NM_020166	0.06001576	1.94097568	-1.228694091	0.00101156	10038	4	530
NM_182983	-0.1178949	1.89532338	-0.960987836	1.59E-05	10071	4	52

NM_182983	-0.044233	2.06751959	-0.960987836	6.36E-05	10073	4	91
NM_001011655	-0.3610809	1.59154148	-1.232124899	0.000767249	9917	4	623
NM_001011655	-0.248945	1.74404944	-1.232124899	0.014601867	10389	4	945
NM_000107	-0.0069518	1.43692473	-1.180590427	0.000507431	9188	4	411
NM_000107	0.01294197	1.61226083	-1.180590427	0.000153486	9189	4	254
NM_032726	-1.1873742	1.58706854	-1.135541545	0.015527298	6026	4	1222
NM_173474	0.20749751	1.50292381	-1.153778149	7.54E-07	7285	4	316
NM_173474	0.36312724	1.5779543	-1.153778149	1.17E-05	7290	4	346
NM_000507	-0.4090323	1.87207383	-1.167857858	3.35E-05	9907	4	501
NM_000507	-0.585364	2.14062763	-1.167857858	3.33E-05	9910	4	733
NM_020225	-0.1126537	1.44891556	-1.092883046	5.31E-05	7164	4	320
NM_020225	-0.2991246	1.97897888	-1.092883046	0.000289376	10050	4	344
XM_027074	0.80767377	1.68530957	-0.717197453	0.002791773	10423	4	695
NM_016084	-0.4694863	1.35740097	-0.985987444	0.00275666	6667	4	778
NM_139248	-0.0166626	2.53093431	-0.991055256	5.83E-05	10723	4	643
NM_004235	-0.6116579	2.63457515	-0.935485925	1.04E-05	10731	4	911
NM_004235	-0.4461421	2.73230459	-0.935485925	0.000336702	10733	4	947
NM_015401	0.51133151	1.49548613	-1.126693507	1.58E-05	7291	4	473
NM_004984	0.94049602	1.61435374	-0.634037104	0.012178161	9660	4	997
NM_006031	0.2402221	1.21473852	-0.502930134	0.027672538	8925	4	736
CN801728	-0.5872585	2.09988872	-1.155951359	8.55E-07	9909	4	646
CN801728	-0.4358385	2.00179789	-1.155951359	4.18E-06	9911	4	454
CN801728	-0.3697265	2.16770561	-1.155951359	5.65E-07	9912	4	502
CN801728	-0.4466953	2.29703562	-1.155951359	5.63E-07	9913	4	649
XR_014061	-0.5703612	1.49616979	-1.029667179	0.000170977	6954	4	599
XR_014061	-0.6137586	1.82327117	-1.029667179	6.27E-05	9922	4	522
NM_022821	0.37316171	1.92499933	-1.093600461	0.000109807	10101	4	290
NM_022821	0.34789194	1.96452956	-1.093600461	0.000410453	10105	4	354
NM_014583	0.07431225	2.14624236	-1.118906469	2.52E-05	10089	4	338
NM_014583	0.1429368	2.1696632	-1.118906469	0.000254807	10113	4	402
NM_133175	-0.2046083	1.98791698	-1.003473252	5.61E-05	10048	4	182
NM_133175	-0.3527872	1.8413359	-1.003473252	0.000327772	9926	4	251
NM_000122	-0.2250562	1.46572363	-0.64407629	0.004274329	8930	4	349
NM_000122	-0.189785	1.24074933	-0.64407629	0.001241434	9043	4	373
NM_144626	0.07960646	1.91607477	-1.136838378	7.06E-05	10066	4	188
NM_144626	-0.0605994	1.96296043	-1.136838378	0.000121089	10065	4	224
NM_014016	-0.5735493	1.48529558	-1.154845664	0.006506872	9879	4	854
NM_014016	-0.3866116	1.60797873	-1.154845664	0.008122544	9880	4	794
NM_032329	-0.3431203	1.24281312	-1.011490886	0.002031896	9003	4	593
NM_032329	-0.11655	1.87948212	-1.011490886	0.003807799	10405	4	408
NM_001263	0.45553339	2.21911393	-0.941030609	0.000373101	10124	4	528
NM_145015	-0.5350885	1.42190423	-0.325246102	0.044120583	9399	4	893
NM_145015	-0.3793557	2.16779171	-0.325246102	0.042882014	10630	4	1045

NM_006275	-0.617222	1.78739732	-1.139103945	0.000649943	9905	4	764
NM_006275	-0.6697905	1.75853999	-1.139103945	7.60E-05	9904	4	688
NM_207514	-0.084699	1.58719582	-0.935940559	3.34E-06	9229	4	64
NM_207514	0.03742431	2.06653875	-0.935940559	9.98E-05	9261	4	173
NM_025137	-0.4448323	1.50430142	-0.697525709	0.001308575	9406	4	397
NM_030753	-0.9712926	1.65617224	-0.608918944	0.041151319	6046	4	1200
NM_006697	-0.3817447	2.18843859	-1.005462595	0.005876156	10663	4	1020
NM_006697	0.71349255	2.00648845	-1.005462595	0.008996895	10876	4	1224
NM_006697	-0.2784032	2.36528758	-1.005462595	0.008040153	11190	4	1090
NM_207446	0.34512996	1.21339506	-0.870790892	0.000151858	8334	4	419
NM_002931	0.08805107	1.27869364	-0.9078186	0.000591732	7268	4	423
NM_020435	0.2824553	1.23639249	-0.841356222	0.00385823	8891	4	620
NM_013398	-0.7562506	2.88163598	-0.573035321	0.008925405	10690	4	1175
NM_001120	0.08717352	1.6211227	-0.983892918	4.30E-06	9233	4	67
NM_001120	-0.0901646	1.57447205	-0.983892918	2.99E-05	9230	4	102
NM_024330	-0.2914381	2.18973593	-0.986065339	0.00040817	10709	4	545
NM_024330	0.07081714	2.01192905	-0.986065339	0.000115259	10074	4	96
NM_015888	0.576935	1.66909032	-0.834911146	0.000181975	9265	4	332
NM_015888	0.57066648	1.74677215	-0.834911146	0.000217732	9266	4	325
NM_001010938	0.07001173	1.50842622	-1.018541242	4.01E-06	9232	4	175
NM_001010938	0.20583063	1.6984983	-1.018541242	1.35E-06	9247	4	95
NM_001338	0.7008395	2.05916474	-1.118480962	0.000336809	10427	4	739
NM_001338	0.79413891	2.2223258	-1.118480962	0.000256108	10428	4	850
NM_015964	-0.3103617	2.20830972	-1.24238857	0.000245689	9916	4	734
NM_015964	-0.4059466	2.09020547	-1.24238857	0.000135322	9915	4	667
NM_004199	0.28616883	1.71181034	-0.962059599	1.24E-05	9248	4	92
NM_004199	0.09966697	1.67869461	-0.962059599	3.29E-05	9249	4	62
NM_032118	-0.4195154	1.88308812	-0.999895582	1.09E-06	9924	4	237
NM_032118	-0.3415298	1.81627	-0.999895582	1.19E-05	9925	4	157
NM_014333	0.10477819	1.50983414	-0.931268675	0.030364216	10384	4	829
NM_024657	-0.1054505	1.88541995	-0.647320483	0.000387952	9255	4	37
NM_024657	-0.1469833	1.88589763	-0.647320483	0.000594219	10078	4	24
XR_013095	-0.1279625	2.4954438	-1.073083427	5.15E-05	10722	4	707
XR_013095	-0.2523077	2.15191384	-1.073083427	7.85E-07	9914	4	337
NM_003917	-0.2363033	1.25262311	-0.810589747	0.000344152	8254	4	382
NM_001277	-0.3150143	1.23785887	-0.897223323	8.46E-05	9014	4	429
NM_001277	-0.1150791	1.47944727	-0.897223323	0.000366176	9160	4	94
NM_014733	-0.0890668	1.58497162	-0.762813255	0.006921308	9364	4	327
NM_014733	-0.494015	1.46231065	-0.762813255	0.012461625	8954	4	672
NM_020690	0.18183086	1.58464598	-0.986833935	3.91E-05	9206	4	85
NM_020690	0.28712678	1.52941845	-0.986833935	6.29E-05	9208	4	135
XR_010398	-0.2227892	1.94086088	-1.1945174	8.11E-06	9908	4	386
XR_010398	-0.2214942	1.5585453	-1.1945174	0.00230096	9882	4	556

NM_018142	-0.2151576	1.28820808	-1.124546069	0.000269901	7181	4	512
NM_006209	-0.3055657	1.26548702	-0.663944347	0.006175426	9018	4	496
NM_018288	0.1232005	1.44386012	-0.889900971	0.000836292	9210	4	142
NM_018288	-0.019177	1.94607511	-0.889900971	0.000328784	10072	4	72
NM_014315	0.22095725	2.49753743	-1.198255419	0.000515576	10338	4	818
NM_001928	0.10989055	2.52059353	-1.176327379	2.52E-05	10335	4	725
NM_001928	0.0385849	2.39254796	-1.176327379	0.000101836	10334	4	610
NM_198587	1.04529708	2.15583631	-0.577960886	0.002708965	9396	4	973
NM_032300	0.80923934	1.63907671	-1.127546111	0.002198298	10418	4	851
NM_002628	0.10738953	1.70793061	-0.978440021	0.008706857	10395	4	564
NM_018282	0.23365127	1.93048946	-1.133671391	0.000146156	10100	4	279
NM_018282	-0.0221885	1.93047265	-1.133671391	0.000253806	10067	4	239
NM_004448	0.15631245	1.29747046	-1.039660919	3.41E-06	8325	4	328
NM_004448	0.15184576	1.55302485	-1.039660919	3.54E-06	9205	4	141
NM_001976	0.12965262	1.33582898	-0.950738685	0.000589934	8923	4	405
NM_001807	-1.0476851	1.44613459	-1.839635199	0.043144167	6041	4	1220
NM_005520	-0.2749987	2.19700714	-1.056006309	0.000585365	10044	4	484
NM_005520	-0.1474911	2.48614065	-1.056006309	0.001176598	10332	4	782
NM_015190	0.40335715	1.4122225	-0.831398838	0.001226852	9338	4	298
NM_015190	0.33644363	1.40783118	-0.831398838	0.003073001	9337	4	318
NM_000846	0.07422634	1.31501381	-0.745395417	0.020316913	6122	4	689
NM_000846	-0.0372284	1.3040153	-0.745395417	0.015670206	8952	4	548
NM_001449	-0.8212139	2.41398215	0.214202977	0.028496899	10631	4	1157
NM_000192	0.62911815	1.78143392	-1.091750464	0.000473375	10417	4	566
NM_000192	0.52534583	1.95068507	-1.091750464	0.000139015	10106	4	468
NM_004227	0.28190261	1.57807987	-0.97727315	0.000127374	7289	4	163
NM_004227	0.14503397	1.80636288	-0.97727315	3.58E-05	9250	4	73
NM_005688	0.16345768	1.70294934	-1.152866695	0.000357726	9193	4	255
NM_004747	0.29650843	1.96942133	-0.981553945	9.00E-06	10104	4	107
NM_004747	0.22655809	1.99378728	-0.981553945	0.000109685	10103	4	118
CN643290	0.24941312	1.4476206	-0.951642703	0.000104584	9243	4	199
CN643290	0.50290046	1.47770392	-0.951642703	8.16E-06	7292	4	323
NM_139021	1.53616917	1.18304388	-0.401008218	0.029190364	9718	4	1178
NM_016223	-0.1460477	2.04872824	-1.068671596	1.64E-05	10069	4	212
NM_016223	-0.1282997	2.14981118	-1.068671596	3.63E-05	10070	4	289
NM_006999	0.2418197	1.32136143	-0.942354258	0.000341937	9211	4	285
NM_006999	0.07078194	1.43873196	-0.942354258	8.76E-05	9202	4	104
NM_024638	0.11904508	1.40264995	-1.033874938	0.021010096	10376	4	820
AB209405	0.65134227	1.81812987	-0.764039224	0.000170154	10420	4	310
AB209405	0.50521886	1.95006213	-0.764039224	0.001546318	10419	4	299
NM_003388	0.32425399	2.91730082	-0.482147783	0.045951606	11186	4	1214
NM_018719	0.23200201	1.45787234	-0.931196959	1.40E-05	9207	4	129
NM_018719	-0.1701208	1.43188467	-0.931196959	0.000342707	9159	4	176

NM_145274	0.18603869	1.43043069	-1.074610126	7.52E-05	9199	4	274
NM_145274	0.22080565	1.31465997	-1.074610126	0.000889586	9196	4	476
NM_001291	-0.0139085	1.72239859	-1.098949565	1.65E-05	9195	4	126
NM_001291	0.18633222	1.58711766	-1.098949565	0.000199863	9201	4	200
NM_016302	0.30424025	1.24667206	-0.953796364	0.006345654	7234	4	575
NM_016302	0.49475132	1.50076999	-0.953796364	0.002039341	9215	4	441
NM_025267	0.31338742	1.29140782	-0.950650904	2.52E-05	8327	4	379
NM_025267	0.34451849	1.27058791	-0.950650904	7.44E-05	8333	4	370
NM_004884	0.58032436	1.7222044	-0.431026295	0.005541931	9676	4	576
NM_004884	0.90914806	3.27848261	-0.431026295	0.00082902	11208	4	1185
NM_018263	0.50265554	1.50401427	-1.095648505	0.000159752	7293	4	492
NM_152640	-0.0657155	1.37281669	-0.784774068	0.000204598	8318	4	125
XR_014196	-0.1392885	1.30453944	-1.244868649	0.002344952	7030	4	697
XR_014196	-0.1521635	1.81941848	-1.244868649	7.26E-05	10046	4	452
NM_021961	-0.0265338	1.60220729	-0.752845189	0.000203824	9239	4	41
NM_021961	-0.0231589	1.92009593	-0.752845189	0.000229155	10079	4	12
NM_015252	-0.4720146	1.50605504	-0.731736353	0.009768802	8929	4	705
NM_001748	0.12864296	2.05585366	-0.162973448	0.013243423	9617	4	612
NM_015626	0.06978896	2.03724376	-0.808704844	5.28E-06	9259	4	23
NM_015626	0.25097057	1.94260806	-0.808704844	3.96E-05	10108	4	36
NM_001001419	0.18290478	1.6034026	-0.91752907	0.000277238	9209	4	77
NM_001001419	0.32062234	1.37622238	-0.91752907	0.001102974	9213	4	305
NM_012103	0.10812469	1.44618036	-0.887178559	4.85E-05	9204	4	74
NM_145030	0.07850914	1.44068885	-0.96470587	1.84E-05	9203	4	120
NM_145030	0.03688404	1.65370424	-0.96470587	8.15E-06	9231	4	44
XR_014109	-0.0241873	1.21981603	-0.824220987	7.91E-06	8315	4	244
NM_032133	-0.3490921	1.49645093	-1.122132285	0.00449713	9738	4	909
NM_032133	0.08169292	2.54263937	-1.122132285	0.000887955	10336	4	835
NM_152775	-0.0821322	1.73366901	-1.059996201	6.98E-05	7009	4	508
NM_002948	-0.2609175	1.22398458	-0.841707717	0.000728473	9015	4	375
NM_004603	-0.1458122	1.7084447	-0.981028398	0.003787386	10661	4	637
NM_004603	-0.0475186	1.5775417	-0.981028398	0.000314837	9228	4	222
NM_022737	-0.536198	1.59106546	-0.307722741	0.040489812	10644	4	919
NM_000355	0.11930647	2.03025037	-0.838665713	7.15E-05	9260	4	61
NM_000355	-0.0574471	1.95134291	-0.838665713	2.20E-05	9258	4	19
NM_003597	-0.0367141	1.36959462	-1.217038152	0.002221792	7031	4	603
NM_003597	0.12567153	2.46641761	-1.217038152	0.001236272	10337	4	856
NM_020195	-0.2274795	1.33699476	-0.830798899	0.000136258	8257	4	297
NM_003180	0.47361319	1.58997955	-1.127688387	0.003922141	10373	4	664
NM_173554	0.92880069	1.21271769	-0.49569116	0.00831629	9654	4	1016
NM_173554	0.52761828	3.49230044	-0.49569116	0.017134218	11228	4	1223
NM_017922	-0.034271	2.25204303	-1.236921969	0.001393077	10710	4	848
NM_017922	-0.0550095	2.00785031	-1.236921969	0.000636877	10068	4	524

NM_004075	-0.3370111	1.21058787	-0.772488916	0.014986053	6670	4	838
NM_173670	0.32361469	1.56851722	-0.487172841	0.03771947	8926	4	908
NM_024953	-0.7367365	1.88455049	-1.263822563	0.04207192	6049	4	1186
NM_002742	0.14014103	2.4334757	-0.953824578	9.80E-05	10724	4	540
NM_031206	0.39937217	1.76260197	-1.003229863	2.98E-05	10094	4	219
NM_031206	0.34405533	1.78310862	-1.003229863	2.88E-05	10093	4	191
NM_005842	-0.2412002	1.3452174	-1.073530002	0.004238411	8944	4	631
NM_022105	0.29478652	1.61966695	-1.065217401	0.002751957	10372	4	433
NM_022105	0.2894042	1.42940551	-1.065217401	0.031650294	10386	4	972
NM_022105	0.25343527	1.98248139	-1.065217401	0.028178238	10391	4	1060
NM_001007560	0.67481849	1.33602547	-0.31693669	0.005928425	9670	4	676
XR_011954	0.39089497	1.77706333	-0.743774765	9.80E-05	9264	4	87
XR_011954	0.18169347	1.78753378	-0.743774765	0.000265278	9284	4	31
NM_153612	-0.040163	2.58018234	-1.154631762	0.03134842	11192	4	1181
NM_001606	-0.0533269	1.30546827	-0.828118177	0.020070786	8897	4	840
NM_001777	0.20861176	1.97300779	-0.725033254	0.000533906	9285	4	78
NM_016478	0.34164434	1.61425147	-0.517758342	0.000932652	9353	4	66
NM_016478	0.14250409	1.64850701	-0.517758342	0.00315489	9037	4	63
NM_032998	0.59033077	1.35502319	-0.797257365	0.007988492	8865	4	723
NM_032998	-0.1927851	2.50538244	-0.797257365	0.000560422	10727	4	887
NM_006392	0.42871132	1.74983504	-1.016179647	3.44E-05	10098	4	196
NM_006392	0.39532029	1.82480509	-1.016179647	2.01E-05	10099	4	162
NM_013279	-0.5347776	2.97342893	-0.9262038	0.020874879	11180	4	1211
NM_003110	0.32476296	2.02087164	-1.132430688	0.029488145	10665	4	1158
NM_138619	0.25788593	1.43247743	-0.961318466	0.000314422	9197	4	233
NM_138619	0.03425885	1.39352505	-0.961318466	0.000782617	9162	4	214
NM_019590	-0.0814792	1.71483467	-1.113491053	0.002688338	10127	4	555
A_01_P006584	-0.5031182	1.5867099	-0.857952471	0.000222832	9919	4	324
NM_003506	-0.3678553	2.11747974	-0.84282015	0.005787837	10671	4	785
NM_015631	0.15599547	1.33806718	-0.626257352	0.028699253	8921	4	777
CN641580	0.44831366	2.83256778	-0.960027717	3.85E-05	10356	4	980
CN641580	0.49549019	2.94001059	-0.960027717	0.000316449	10357	4	1074
NM_214462	0.10572945	1.32549961	-0.466609337	0.00313099	9106	4	258
XM_035527	0.19104118	2.13056618	-0.939913922	6.70E-06	10120	4	130
XM_035527	0.018505	2.12948861	-0.939913922	0.000516926	10114	4	187
XR_009781	-0.8715585	1.34571695	-1.220330875	0.018238603	6059	4	1124
NM_001643	0.81483122	1.74497096	-0.698323707	0.002210388	10424	4	767
CB549194	0.26763488	2.38063375	-0.855121623	0.000192148	10119	4	400
CB549194	0.24159407	2.33594635	-0.855121623	0.000520648	10118	4	392
NM_153331	-0.2079296	1.7738769	-0.279829461	0.047250718	8937	4	926
NM_153331	-0.2606632	1.7633936	-0.279829461	0.019215785	8936	4	630
XR_013017	0.22713246	1.95821808	-0.968497586	0.000115182	10096	4	192
XR_013017	0.30069355	1.93035569	-0.968497586	6.54E-06	10095	4	143

NM_006200	0.08855645	1.43139924	-0.721071825	0.018110062	8965	4	523
NM_014296	-0.2023359	1.2989548	-0.540915594	0.023376939	9039	4	560
NM_005139	0.00382401	2.15349901	-0.974644052	4.39E-06	10090	4	151
NM_020987	0.00339196	1.7181924	-0.882583152	0.000561824	9170	4	46
NM_016019	-0.1140282	1.39020716	-0.881899102	1.63E-05	8319	4	106
NM_016019	0.12690982	1.37589718	-0.881899102	0.000533982	9164	4	165
NM_022778	0.11553645	1.63264202	-0.308806538	0.005527923	9374	4	221
NM_022778	-0.0092825	1.50739697	-0.308806538	0.005470262	9109	4	193
NM_012229	0.0861222	1.64013563	-1.036833319	0.001382536	9192	4	213
NM_012229	0.07147193	1.61007956	-1.036833319	0.002103501	9191	4	257
NM_001012506	-0.1385221	1.89193551	-1.163011232	0.000283119	10047	4	381
NM_001012506	0.0006659	1.7091365	-1.163011232	0.008673929	10394	4	731
CN803652	-0.9072667	1.93574564	-0.845718891	0.00308701	6064	4	895
CN803652	-0.6459473	2.95962098	-0.845718891	0.001514324	10736	4	1133
NM_002281	0.46480756	1.30051541	-0.740670035	0.007097446	8893	4	702
NM_145291	0.40036314	1.24418638	-0.689247708	0.00078562	9335	4	389
NM_145291	0.49804077	1.58739227	-0.689247708	0.000875695	9339	4	209
BC067766	-0.3265652	1.76640307	-1.085198631	0.001217963	9918	4	470
BC067766	-0.2955071	2.59358606	-1.085198631	0.002499237	10735	4	1000
NM_018185	-1.6348707	1.66824821	-0.53731641	0.003008841	6055	4	1183
NM_015022	0.31278345	2.71694828	-0.543064166	0.009854808	10829	4	1113
NM_015022	0.15176594	2.58200823	-0.543064166	0.003903337	11200	4	843
NM_000380	-0.0461633	1.44898254	-0.455805113	0.003287944	9033	4	132
NM_013382	-0.7016626	1.42221522	-1.115743262	0.000769806	6561	4	1034
NM_016370	0.17202279	2.56498053	-0.416999219	0.024580794	10821	4	1141
NM_002013	0.4899974	1.35688358	-0.591193196	0.001667118	9336	4	360
NM_002746	-0.0138685	1.4395522	-0.704510058	3.71E-05	9237	4	55
NM_002746	0.04115351	1.57378376	-0.704510058	0.000149097	9238	4	35
NM_004496	-0.6364296	2.32247997	-0.391975624	0.01181696	10699	4	1066
NM_004657	-1.5022664	3.08211627	-0.697728289	0.009793955	11187	4	1227
NM_080731	0.02247463	1.45427888	-1.187189054	0.000526714	9186	4	436
NM_080731	0.08793737	1.63104775	-1.187189054	0.000720911	9187	4	380
XR_011842	0.02430328	1.30323279	-1.014202076	0.001076922	7270	4	543
NM_001826	0.36360246	1.57088932	-0.827837326	0.000394668	9219	4	113
NM_001826	0.41354734	1.48360311	-0.827837326	0.002424129	9216	4	296
NM_145047	0.56038026	1.66202007	-0.456001469	0.000504237	9675	4	312
NM_145047	0.44935543	3.20926936	-0.456001469	0.008324177	10830	4	1190
NM_015470	0.69819787	1.20209679	-0.400833412	0.048335688	9659	4	1027
NM_002686	-0.2303226	2.47102895	-0.496555845	0.000677336	10746	4	558
NM_002686	-0.5859502	2.60419825	-0.496555845	0.000407497	10744	4	883
NM_207310	-0.1408062	1.83607409	-1.405028257	0.0012668	9883	4	878
NM_016111	0.24503814	1.23888347	-0.979375775	5.56E-05	8332	4	391
CO725992	-0.0025779	1.42972135	-0.874056513	0.002111967	9166	4	180

CO725992	-0.1120997	1.49334262	-0.874056513	0.002911024	9165	4	201
NM_198129	-0.7601749	2.54563171	-0.927872531	0.000131716	10689	4	1010
NM_198129	-0.4522226	2.71166788	-0.927872531	0.000132992	10732	4	935
NM_021738	0.50129155	2.44854079	-0.724968551	0.000273867	10789	4	693
NM_003791	0.20425515	1.23496439	-0.753107084	3.77E-05	8331	4	247
NM_002344	0.04735448	1.91036338	-0.89391713	0.000501399	9267	4	119
NM_002344	-0.042219	2.21902219	-0.89391713	0.001004579	10711	4	499
NM_145914	0.06806195	1.61972856	-0.541409229	0.002396846	9366	4	117
NM_145914	0.31155204	1.73288158	-0.541409229	0.002382714	9369	4	93
NM_007013	-0.001609	1.21250582	-0.292207945	0.015327816	9048	4	513
NM_017805	-0.2388989	1.2897849	-0.579908285	0.009281747	9020	4	403
NM_000142	0.03465894	1.72385092	-0.900674251	8.31E-07	9234	4	27
NM_000142	-0.0036433	1.84147508	-0.900674251	1.96E-06	9256	4	34
NM_005144	0.52408259	1.94348453	-1.059315525	0.001008782	10571	4	592
NM_032012	0.23316142	1.30337653	-0.773966854	0.001176291	9212	4	215
NM_032012	0.27342592	1.56624459	-0.773966854	0.002123218	9225	4	134
NM_001001433	-0.2796162	1.25568878	-0.616424324	0.000473762	9013	4	263
NM_000304	-0.0005214	1.63502331	-0.698004378	0.001245456	9171	4	28
NM_003196	-0.2568653	1.30876875	-0.823988325	0.002658482	8255	4	511
NM_016604	0.33310336	1.68469123	-0.702208199	1.57E-05	9263	4	26
NM_016604	0.34608412	1.78983633	-0.702208199	0.000228887	9220	4	38
NM_000440	0.18579928	1.40117345	-0.883815431	0.007400165	10371	4	605
NM_003873	0.1431903	2.12526024	-0.874400891	0.00302383	10115	4	388
NM_003873	-0.339781	3.01124498	-0.874400891	0.00024771	10734	4	1076
NM_015296	0.3616274	2.19070044	-0.838894307	0.002072299	10116	4	446
NM_015296	0.59215665	2.47912616	-0.838894307	0.002047575	10429	4	861
NM_004510	-0.0415426	1.28046715	-0.805860103	0.00013111	8320	4	181
NM_004510	0.23029191	1.63246294	-0.805860103	0.000716765	9218	4	51
NM_205854	-0.0980121	1.92566638	-0.702747835	0.00018218	9254	4	56
NM_205854	-0.1081457	1.79447551	-0.702747835	4.66E-05	9253	4	13
NM_000542	0.23885665	1.74211488	-0.8693788	0.001376475	9222	4	108
NM_000542	0.25376211	1.57067679	-0.8693788	0.004622322	9221	4	288
NM_006545	0.00459642	1.33781603	-0.953122383	0.000808594	9161	4	241
NM_006545	-0.0250297	1.55137329	-0.953122383	0.00022338	9194	4	97
XR_013821	-0.0108304	2.02736557	-0.758006016	0.036993062	10667	4	1084
XR_013086	0.20080621	1.7557	-0.754971749	0.023981686	10397	4	679
XR_013086	0.38697707	2.6169833	-0.754971749	0.009054752	11201	4	1038
NM_173640	0.0095246	1.52666001	-0.486285692	0.014558596	8931	4	445
NM_173640	0.03110919	1.58981807	-0.486285692	0.006499491	9365	4	170
NM_145065	0.22518099	1.38114205	-0.669374902	0.000403512	9304	4	207
XM_376355	0.7602962	2.39132459	-0.518681247	6.44E-05	10796	4	765
XM_376148	0.05228283	1.6600531	-0.832800118	3.93E-05	9235	4	49
XM_376148	0.253971	2.03721777	-0.832800118	0.00026463	10110	4	90

NM_003951	0.29034503	1.33708655	-0.713407125	0.000398586	9332	4	169
NM_003940	-0.0290266	1.90532681	-1.212036554	0.003933521	10128	4	773
NM_003940	-0.0047214	1.87847585	-1.212036554	0.01090665	10126	4	930
NM_014550	-0.8195384	1.4381297	-0.750795713	0.000349914	9400	4	748
NM_006769	0.0209987	1.39578543	-0.849184686	0.001563386	9163	4	183
NM_006769	0.18712111	1.56425829	-0.849184686	0.000961663	9217	4	80
NM_022913	0.10767583	1.34418713	-0.777486098	0.000481924	9173	4	159
NM_020738	-0.1281393	1.42020502	-0.801735431	0.00423133	9153	4	248
NM_002235	0.49713066	1.64782014	-0.824990584	0.000291766	9262	4	430
CB229350	-0.1654564	1.50005197	-0.736136881	0.000442941	9226	4	210
NM_014485	0.67102307	1.89812857	-1.105613549	0.004913242	10573	4	1014
NM_019096	-0.4176968	1.28381452	-0.761770556	0.000433964	9007	4	394
NM_024841	0.2945407	2.28532519	-1.007482503	0.00573451	10713	4	963
NM_002393	0.20971557	1.55351472	-0.701873054	0.001791129	9223	4	103
XM_495839	0.81172052	1.43480114	-0.647141604	0.000934177	10421	4	619
XM_495839	0.83642093	1.55311437	-0.647141604	0.000628281	10422	4	583
NM_001390	0.18920342	1.44854273	-0.569838427	0.000505766	9341	4	47
NM_145260	-0.0283089	1.27759574	-0.525113777	0.00431081	9050	4	315
NM_145260	-0.3761523	2.52938615	-0.525113777	0.004977921	10700	4	998
NM_181526	-0.2986563	1.56333613	-0.915508194	1.33E-05	9921	4	133
NM_181526	-0.3322391	1.54174867	-0.915508194	4.94E-05	9920	4	164
NM_021089	-0.1957559	1.25321229	-0.931630448	0.014813272	9150	4	691
NM_021089	0.47315662	2.22163443	-0.931630448	0.037184006	11191	4	1194
NM_020776	1.77852468	2.51333884	-1.110772146	0.000190636	11217	4	1206
NM_019852	-0.0925293	2.03665999	-0.800453122	2.53E-05	9257	4	54
NM_019852	-0.9264269	2.02233411	-0.800453122	0.000370076	6056	4	1114
NM_000102	0.60813322	1.20336742	-0.542626263	0.033126364	9658	4	983
NM_032433	0.09940899	2.31650443	-0.771667858	0.005186373	10664	4	858
XM_498468	0.04551677	1.43692323	-0.698616881	1.06E-05	8322	4	43
XM_498468	0.13822459	1.72465152	-0.698616881	2.71E-06	9275	4	2
A_01_P016919	0.24784583	1.36275891	-1.139988745	0.002352511	8993	4	680
NM_031477	-0.0418559	1.4912423	-0.744027438	2.58E-05	9236	4	45
NM_001008701	-0.9123803	1.69376383	-0.807780231	0.007381168	10668	4	1008
NM_205853	-0.4788815	2.19632866	-0.380901861	0.042646562	10698	4	1122
NM_205853	-0.2167021	1.72443195	-0.380901861	0.006275968	9373	4	307
NM_145236	0.59235582	1.50352161	-0.40923311	0.043389316	9387	4	868
NM_006253	0.11221692	1.56421052	-0.499096458	0.001373269	9112	4	75
NM_052946	0.2486014	1.95133336	-0.671106745	0.000464606	9287	4	25
NM_052946	0.24861652	2.18894851	-0.671106745	0.000719777	10122	4	226
NM_005241	0.04639045	2.14393005	-0.514074386	2.40E-05	10752	4	121
NM_005241	0.35831409	2.74019068	-0.514074386	7.40E-05	10798	4	834
XR_014490	-0.1407989	1.60411963	-0.678804507	0.000395451	8932	4	58
NM_017954	0.28080744	1.99200518	-0.908499159	0.000162973	10109	4	101

NM_017954	0.41120914	2.21716659	-0.908499159	8.39E-05	10121	4	359
NM_139161	-0.1227994	1.34957292	-0.581404334	0.00044131	8243	4	139
NM_000587	-0.0986926	1.44962324	-0.612743235	0.005694264	9030	4	160
NM_000587	0.13264807	1.5426166	-0.612743235	0.006959675	9224	4	166
NM_080749	0.47287414	1.18344596	-0.180642208	0.010559728	9669	4	786
NM_014312	-0.138738	1.46578152	-0.48211011	0.00409804	8978	4	462
NM_012385	0.07996194	1.71131357	-0.701379044	0.000126766	9272	4	5
NM_012385	-0.0026156	1.78432694	-0.701379044	6.32E-05	9271	4	1
NM_024652	-0.0957476	1.55399342	-0.695760872	0.000921671	9169	4	33
NM_006505	0.17493592	1.82838802	-0.405532765	0.000209015	9115	4	32
NM_006505	-0.045861	1.59713829	-0.405532765	0.006213887	9036	4	131
A_01_P000240	0.3066207	2.04557453	-0.577877351	0.026198542	10824	4	1049
A_01_P000240	0.20576451	1.37710751	-0.577877351	8.12E-05	9340	4	84
XR_000285	-0.2901755	1.32872806	-0.777151806	0.000777917	9017	4	326
XR_000285	-0.1175785	1.40385549	-0.777151806	0.000738441	9167	4	110
XR_000285	-0.028541	1.53290312	-0.777151806	0.000272063	9168	4	20
NM_016589	-0.0139002	1.77995066	-0.513535662	0.005572142	10674	4	661
NM_016292	0.28040936	1.37466751	-0.510075386	5.17E-05	9345	4	124
NM_016292	0.40676881	1.39931951	-0.510075386	6.04E-05	9348	4	161
NM_002165	-0.2473159	1.5334635	-0.551768754	0.001780438	9031	4	88
NM_002165	-0.0621181	1.61900475	-0.551768754	0.001351279	9032	4	21
NM_006746	-0.5381216	1.36113821	-0.713400246	0.029602357	8928	4	934
NM_013260	0.02309181	1.21995527	-0.702802125	0.000972087	8179	4	269
NM_005486	0.06124271	1.37681109	-0.379939463	0.009275071	9053	4	343
NM_005486	-0.0125895	1.95398099	-0.379939463	0.018623206	8938	4	636
NM_130781	0.00836859	1.35618277	-0.895168315	0.000184492	8316	4	203
CN805335	0.1703486	1.60716833	-0.494442235	0.00095613	9278	4	14
CN805335	0.24255875	2.0022547	-0.494442235	0.000528932	9291	4	50
CN805335	0.34812451	1.92399468	-0.494442235	0.000634958	9289	4	59
CN805335	0.13728437	2.00506276	-0.494442235	0.0013962	9286	4	53
NM_002447	-0.7016309	1.32026696	-0.489486381	0.002798948	7509	4	790
NM_145170	0.07757843	1.54351785	-1.418739267	0.028067605	10360	4	1123
NM_022777	0.41322751	1.50553437	-0.397838589	0.032229017	8922	4	807
NM_018110	0.18745802	1.84901352	-0.548355326	0.000158815	9280	4	7
NM_018110	0.14667787	1.73671997	-0.548355326	0.000538996	9279	4	9
NM_006175	0.54233176	2.70430856	-0.374641893	0.000775121	10797	4	984
NM_006175	0.39829845	3.13615315	-0.374641893	0.000599176	11207	4	1121
NM_002616	0.25600735	1.31994619	-0.542340347	5.15E-05	9343	4	116
NM_002616	0.38561802	1.36915726	-0.542340347	0.000149502	9347	4	158
NM_006462	-0.003615	1.24035869	-0.403899788	0.004555507	9051	4	369
NM_006462	-0.0424422	1.28752694	-0.403899788	0.003997197	9052	4	319
NM_052970	0.00350043	1.89942624	-0.104383705	0.048389076	9382	4	890
NM_020427	0.37749037	1.45967293	-0.990190844	0.017530377	10377	4	852

NM_005799	-0.6720207	2.17541683	-0.211936643	0.049161755	10819	4	1146
NM_005799	-0.404742	2.53953841	-0.211936643	0.008629721	10750	4	1001
NM_022734	-0.1226368	1.2224199	-0.610472724	0.000759092	9049	4	293
NM_005630	-0.1561014	1.20984743	0.028416122	0.031655082	9512	4	832
NM_005630	0.23624603	2.0698742	0.028416122	0.006736704	9619	4	613
NM_007146	0.38534778	1.30921519	-0.744596989	5.90E-05	9333	4	240
NM_007146	-0.1391599	1.51806904	-0.744596989	8.62E-05	9157	4	69
CB548748	-0.2155023	1.27724364	-0.466519468	0.00424591	9021	4	284
CB548748	-0.4396092	1.3306158	-0.466519468	0.003863899	9024	4	417
CB548748	-0.3357335	1.54415197	-0.466519468	0.004176477	9028	4	195
NM_017612	0.06114363	1.64993625	-0.770276866	0.00266715	9227	4	277
NM_182507	0.38873539	3.60714571	-0.484346704	0.000817642	11231	4	1218
NM_014802	0.54231373	1.39586968	-0.788896544	3.55E-05	9329	4	387
NM_014802	0.6435663	1.32154611	-0.788896544	0.000213719	9328	4	591
NM_000224	0.0506587	1.59818378	-0.566726035	4.68E-06	9273	4	4
NM_000224	0.21220325	1.65190582	-0.566726035	6.43E-05	9277	4	8
NM_007018	-0.1978011	1.30658797	-0.437769527	0.004128638	8977	4	792
NM_007018	-0.3223027	1.46124309	-0.437769527	0.001117361	9417	4	259
NM_017636	0.23303507	1.33956263	-0.469174707	6.24E-05	9344	4	148
NM_017636	0.19174043	1.27980075	-0.469174707	5.14E-05	9342	4	144
NM_003060	-0.4755946	1.45346696	-0.913770935	0.003699941	6062	4	729
NM_003060	-0.2399548	2.06816708	-0.913770935	0.000937845	10407	4	273
NM_139275	0.33906123	1.3967541	-0.553984596	0.000712062	9346	4	128
NM_139275	0.28510936	1.57926517	-0.553984596	0.002737419	9349	4	79
NM_178818	0.84967107	1.87345199	-0.246170612	0.003637925	9393	4	771
NM_054013	0.12674155	1.25845682	-0.512510514	0.005689932	9176	4	287
NM_022474	0.54088323	2.57052384	-0.803369322	3.79E-05	10355	4	756
NM_014624	0.05495843	1.31253649	-0.628112828	0.000256997	9174	4	89
NM_022492	-0.8800867	1.50058984	-0.467236956	6.80E-05	9401	4	836
NM_198471	0.02012599	1.71756388	-0.654178697	3.57E-06	9274	4	3
NM_198471	0.01620585	1.57823412	-0.654178697	0.000140688	9270	4	10
NM_004181	-0.1255621	1.25272869	-0.492619745	0.007281201	9022	4	350
NM_004181	-0.189114	1.56443057	-0.492619745	0.010027518	9029	4	261
NM_033261	0.52385338	2.62561165	-0.803274326	0.000954283	10791	4	958
XR_010302	0.208765	1.43986585	-0.55786443	0.000455062	9308	4	114
NM_003260	-0.0971202	1.57276041	-0.367472274	0.009282209	9372	4	361
NM_003260	0.08384909	1.79226596	-0.367472274	0.001851064	9295	4	70
NM_148909	-0.0304221	1.54038494	-0.392050551	0.000353271	9035	4	16
NM_148909	-0.0433204	1.48958113	-0.392050551	0.000806558	9034	4	57
NM_006997	-0.1080598	2.24609463	-0.336852167	0.007370003	10751	4	559
NM_006997	-0.1590373	2.68535853	-0.336852167	0.00095737	10749	4	827
NM_004114	1.59655877	1.56505063	-0.374159636	0.006631979	10877	4	1219
NM_004469	0.11740539	1.3466416	-0.197060339	0.031664411	9371	4	730

NM_015158	0.49167729	1.99073169	-0.363098702	0.001292763	10783	4	407
NM_015158	0.26165255	1.48387678	-0.363098702	0.004355894	9107	4	256
CN644644	-0.4751411	1.38777653	-0.330438282	0.000155246	9408	4	401
CN644644	-0.4295865	1.37676922	-0.330438282	0.000570034	9026	4	339
NM_004594	-0.1587295	3.36582792	-0.551645501	0.014620939	11185	4	1207
NM_133476	0.15063895	1.77843001	-0.619922787	3.96E-06	9276	4	6
NM_133476	0.28335932	1.73854645	-0.619922787	3.71E-05	9281	4	11
NM_018357	0.53076905	1.21315418	-0.583789671	0.000358722	9326	4	541
NM_018357	-0.0336697	1.70347807	-0.583789671	0.00325939	10675	4	308
NM_012193	-0.0056312	1.33523172	-0.793160961	0.013939225	9151	4	507
NM_012193	-0.0949873	1.43618035	-0.793160961	0.008995977	9152	4	362
XR_009857	0.13853691	1.68733212	-0.722601687	0.001589351	9268	4	65
XR_009857	-0.1115825	1.76281908	-0.722601687	0.000654868	9269	4	15
NM_000014	0.21388348	1.97940068	-0.509239275	0.000750885	9288	4	29
NM_000014	0.20910355	2.1792948	-0.509239275	0.000310401	9290	4	81
NM_003280	-0.2753558	2.0814544	-1.350262771	0.001803941	9884	4	885
NM_003280	-0.3126942	2.40777355	-1.350262771	0.002497768	10437	4	1033
NM_018555	-0.8041548	1.8203219	-0.709550711	2.38E-05	9403	4	580
NM_015497	-0.3289514	1.26452795	-0.084714087	0.010386686	9462	4	763
NM_015497	-0.0051655	1.46203205	-0.084714087	0.015993838	9108	4	518
NM_015846	-0.1792166	1.21492505	-0.660926977	0.003923973	9155	4	393
NM_015846	0.08623178	1.33613602	-0.660926977	0.002882922	9172	4	174
NM_015846	0.18500546	1.18128097	-0.660926977	0.019439142	8990	4	621
NM_152312	0.08927913	1.87426101	-0.682473196	0.00259514	10706	4	282
NM_152312	0.25489702	2.3605595	-0.682473196	0.003067136	10714	4	795
NM_001762	0.36154901	1.17309701	-0.546421082	0.002178838	9318	4	410
NM_173617	0.78884092	2.23762726	-0.54372192	0.04453881	10825	4	1171
NM_002663	0.16351754	2.07161577	-0.412445132	0.036047732	10677	4	981
NM_018178	0.3117185	1.495451	0.154872716	0.028543356	9599	4	768
BC054816	-0.5175258	1.45968562	-0.634905892	0.005659847	6544	4	863
NM_003072	0.17949182	1.60265887	-0.414490814	0.00062604	9351	4	18
NM_003072	0.31955865	1.80585901	-0.414490814	0.000188817	9293	4	22
NM_003459	-0.1167424	1.25810548	-0.171312849	0.03255218	8986	4	759
NM_018181	-0.0177673	2.0642562	-0.512231019	0.003240249	10683	4	197
NM_018181	-0.3681316	3.26964035	-0.512231019	0.015547667	11182	4	1208
XM_371619	0.03228528	1.20749486	-0.330443391	0.001875537	9070	4	406
XM_371619	0.08749288	1.48633803	-0.330443391	0.030202123	9142	4	526
NM_004454	0.35662288	1.429373	-0.543776154	9.52E-05	9309	4	150
NM_004454	0.44976969	1.19956293	-0.543776154	0.000147266	9325	4	420
NM_000120	-0.2185779	1.61894345	-0.532651327	6.37E-05	9414	4	42
NM_000120	-0.3105869	1.58048788	-0.532651327	1.92E-05	9413	4	76
NM_003968	-0.0387735	1.54389318	-0.701013576	0.000522284	9158	4	40
NM_021627	-0.1563082	1.83591667	-0.332026064	0.000204798	9114	4	71

NM_021627	0.0802761	1.77711054	-0.332026064	0.000224686	9113	4	109
NM_153365	0.33785533	1.78840627	-0.447576095	4.24E-06	9292	4	39
NM_153365	-0.0185538	1.70715416	-0.447576095	0.001075479	9283	4	17
NM_152879	-0.175028	1.35967095	-0.73047565	0.000707101	9156	4	155
NM_002023	0.01071863	1.34924779	-0.767646647	0.000229697	8321	4	167
NM_002023	0.49261438	1.47277737	-0.767646647	0.000386611	9330	4	249
NM_032195	-0.2807628	1.23628619	-0.568484977	2.75E-05	8241	4	301
NM_032195	-0.5436409	1.7633513	-0.568484977	0.000477498	9405	4	243
NM_018296	-0.073376	1.70209585	-0.847598738	0.015077718	10396	4	565
AB209485	-0.7217171	2.01210178	-0.431192841	0.018394753	10692	4	1009
AB209485	-0.4883712	2.08441478	-0.431192841	0.016683895	10693	4	917
AB209485	-0.9229475	2.08065919	-0.431192841	0.012118139	10694	4	1022
AB209485	-0.8625367	2.47965788	-0.431192841	0.005283319	10695	4	1072
AB209485	-0.5023304	2.48572104	-0.431192841	0.005227899	10696	4	925
AB209485	-0.5600259	2.65597316	-0.431192841	0.004960634	10697	4	1054
NM_001010987	0.69313282	2.29698646	-0.809526137	0.018996366	10628	4	1093
CN646916	0.09373802	2.21644834	-0.869611683	0.00124903	10716	4	633
CN646916	0.38308133	3.00501919	-0.869611683	0.006266225	11202	4	1159
NM_032793	0.14264894	2.25775011	-0.59164648	0.000912877	10755	4	225
NM_032793	-0.1045858	2.32331576	-0.59164648	0.000786264	10754	4	314
NM_016175	-0.0607283	1.50678605	-0.700387688	0.005754327	8972	4	330
NM_016175	-0.1184496	1.67116236	-0.700387688	0.028954872	10398	4	753
NM_015690	-0.3343793	2.10265607	-0.645449389	0.002902747	10672	4	586
NM_015690	-0.0983877	1.60628623	-0.645449389	0.006816134	9154	4	194
NM_144668	0.61969824	1.48265202	-0.402257023	0.035684012	9665	4	956
NM_144668	0.61620717	1.73025049	-0.402257023	0.003230616	9674	4	589
NM_005012	0.40990664	1.70087461	-0.301398666	0.000975552	9299	4	189
NM_022443	0.17840366	2.23165325	-0.287430414	0.004905615	9383	4	579
NM_130761	-0.4403304	1.53006679	-0.844613291	0.006062849	6543	4	666
NM_007202	-0.8744224	1.64527604	-1.031205856	0.002224158	6061	4	942
CO647467	-0.3874584	1.48929067	-0.044581546	0.009372159	9510	4	594
CO647467	-0.1941751	1.8870433	-0.044581546	0.005662421	9575	4	444
NM_005575	0.31364311	1.49140478	-0.222936978	0.000418544	9671	4	341
NM_018430	0.18245276	2.02168189	-0.642355775	0.003398766	10708	4	479
NM_001424	-0.1579329	2.54567829	-0.276034477	1.28E-05	10747	4	584
NM_001424	-0.1804768	2.65435353	-0.276034477	5.16E-05	10748	4	711
NM_005243	0.23615218	1.23080283	-0.540746237	0.001650994	9316	4	265
NM_005243	0.10211571	1.33991057	-0.540746237	0.002059074	9175	4	138
NM_000407	-0.0676297	1.27285267	-0.62231551	0.010646132	8898	4	598
NM_004328	-0.3564098	1.2396628	-0.190266836	0.023962572	9436	4	673
NM_003979	-0.2725742	1.41326298	-0.255243105	0.003699321	9027	4	278
NM_003979	-0.1991009	1.60719489	-0.255243105	0.000817125	9563	4	82
NM_000366	1.12601003	1.48716707	-0.580842516	6.41E-05	10832	4	1135

CN646799	0.00069603	1.20801162	-0.430991439	0.000555936	8201	4	216
NM_145804	0.10941189	2.45574996	0.019368714	0.031643239	10822	4	1085
NM_004629	0.32009009	1.20506455	-0.712606253	0.002806472	9305	4	539
NM_004629	0.44066287	1.43138351	-0.712606253	0.001155797	9306	4	367
XM_095965	0.84288046	1.82943202	-1.094536823	0.002487187	10416	4	940
NM_003617	0.06084764	1.18085072	-0.270669323	0.014574768	9127	4	494
NM_024513	0.01915361	1.61452379	-0.657857409	0.026185513	10399	4	572
NM_014977	0.18002527	1.73139349	-0.275476907	0.006422459	9377	4	364
NM_014977	0.23802857	1.939569	-0.275476907	0.002067616	9378	4	271
NM_015705	-0.5827809	1.42812751	-0.412338727	1.20E-07	9407	4	414
NM_015705	-0.4610234	1.80986628	-0.412338727	1.51E-06	9415	4	145
NM_003243	0.09994757	2.05447156	-0.650977676	0.004719743	10408	4	264
NM_003243	0.01063257	2.36736574	-0.650977676	0.004512194	10718	4	660
NM_207348	0.26175793	2.51973283	-0.663879243	0.036838658	10827	4	1172
NM_207348	0.06818712	3.08930801	-0.663879243	0.009419784	10828	4	1188
NM_178123	0.10241464	1.91238695	0.111018625	0.014827287	9589	4	781
NM_178123	0.28330959	1.34389225	0.111018625	0.01116514	9597	4	750
NM_002905	-0.8676678	1.60812987	-0.513985347	0.000383104	9402	4	817
NM_052851	-0.3067257	1.90866188	-0.574942222	0.036091259	10404	4	877
NM_032638	0.4965632	1.87962819	-0.817178948	0.000629005	10111	4	246
NM_032638	0.49946444	2.02929825	-0.817178948	0.000219739	10112	4	267
NM_020698	-0.0194093	1.25067318	-0.253848842	0.012709884	9055	4	600
NM_005031	-0.0561376	1.71398754	-0.144008016	0.008927176	9729	4	1028
NM_003985	0.50419098	1.44834043	-0.456098352	0.002530075	9673	4	497
NM_007039	0.45118618	1.70881901	-0.318793967	0.01918234	9148	4	534
NM_007039	0.15586125	2.94303002	-0.318793967	0.002458915	11206	4	1103
NM_016608	-0.3695054	1.30396091	-0.416999979	0.001603523	9100	4	378
NM_016608	-0.3910553	1.30740011	-0.416999979	0.006424626	9025	4	440
NM_005103	0.34387097	1.42897355	0.073032817	0.013245473	9598	4	645
NM_004209	-0.2518524	1.62424849	-0.858938995	0.003096112	10705	4	471
A_01_P019886	0.35654357	1.32741113	-0.338643854	0.000333298	9358	4	227
A_01_P019886	0.3441279	1.58010779	-0.338643854	8.56E-05	9298	4	86
NM_004417	-0.687065	3.20922866	-0.480672793	7.10E-06	10772	4	1155
NM_004417	-0.50061	3.2740506	-0.480672793	4.14E-06	10773	4	1151
NM_005922	0.09247189	1.52102302	-0.237424754	0.001638067	9097	4	281
XR_013448	-0.0169825	1.57297591	-0.145374772	0.0246206	9375	4	650
XR_013448	0.12647724	1.61821371	-0.145374772	0.010911318	9376	4	488
XR_012616	-0.06833	1.46147046	-0.463008796	0.025244113	10400	4	587
XR_012616	0.20137021	2.5390989	-0.463008796	0.000898132	10794	4	699
NM_173050	-0.0672746	1.26863824	-0.832849984	0.010513092	8970	4	714
NM_006377	-0.6075009	1.38589246	-0.219072282	0.00330439	9410	4	719
NM_006377	-0.5173282	1.67007424	-0.219072282	0.000964139	9412	4	453
NM_001001995	0.26204285	2.0369007	-0.627334485	0.000241459	10717	4	112

XM_373419	-0.0832318	2.24874582	-0.318011802	3.86E-05	10756	4	153
XM_373419	0.02429506	2.30953194	-0.318011802	0.000178069	10757	4	228
NM_018170	0.87237373	1.1805017	-0.364533849	0.00079546	9709	4	847
NM_001006682	-0.1355185	2.00498976	-0.373120919	0.000786226	10676	4	177
NM_001006682	0.01704996	2.37205531	-0.373120919	0.000153017	10753	4	409
NM_001006682	-0.2023298	2.7783372	-0.373120919	0.012591061	11184	4	1164
NM_001006682	0.27972312	2.44288337	-0.373120919	0.001890985	10762	4	713
NM_198935	0.40121829	1.5395642	-0.614583921	0.000912771	9307	4	229
NM_015061	-0.3556867	1.33271574	-0.297141287	0.016615563	9116	4	588
NM_000825	0.03944233	1.81428462	-0.553550972	0.008443496	10707	4	611
NM_000825	-0.4376958	1.94741768	-0.553550972	0.019996112	8939	4	971
NM_020789	-0.7836532	1.61655438	-1.059098929	0.01143952	6533	4	1078
XM_371227	0.71679189	2.30800481	-0.713046848	0.000476641	10790	4	804
NM_016586	-0.3785783	2.00231636	-0.683082054	0.012529806	10403	4	655
NM_016586	-0.9279857	3.42167227	-0.683082054	8.48E-05	10771	4	1196
NM_019055	0.06465583	1.33979269	-0.134904898	0.000844957	9110	4	300
NM_019055	0.02685165	1.50225943	-0.134904898	0.0009753	9111	4	206
NM_003586	-0.1992847	1.73230956	-0.164870718	0.024016989	9370	4	788
NM_003586	-0.2890698	1.37431573	-0.164870718	0.018602394	9423	4	684
NM_015607	-0.0814282	1.35740236	-0.423082603	0.006111297	9023	4	205
NM_018119	-0.6204957	1.31917536	-0.816326687	0.007343459	6676	4	769
NM_145270	1.26955325	1.88832218	-0.996433016	0.003109377	10621	4	1130
NM_145270	1.11257503	1.80671275	-0.996433016	0.001532071	10620	4	1063
NM_031497	1.04953788	1.40049849	-0.534796396	0.023108292	9701	4	1083
NM_198484	0.31154431	1.67125463	-0.321938791	0.04761582	9384	4	955
CK231501	0.31897796	2.23460666	-0.681751985	0.029317817	10823	4	1125
CK231501	0.60377073	1.93763706	-0.681751985	0.000548579	10784	4	590
NM_052890	0.83809181	1.43366593	-0.651818462	0.014944178	9690	4	1086
NM_052890	0.48303144	1.18354235	-0.651818462	0.008652959	9692	4	754
CN645262	-0.6331108	1.48024339	0.006975612	0.048258849	9447	4	982
NM_003161	-0.3853007	1.3002492	-0.28925114	0.013067779	9419	4	648
NM_024830	-0.6064671	2.496976	-0.247134467	0.002290257	10742	4	897
NM_024830	-0.6596587	2.6103117	-0.247134467	0.00120629	10743	4	967
NM_020850	0.33420715	1.31900517	-0.259055965	0.037593203	9663	4	824
CN643169	0.55252388	2.12654932	-0.378522116	0.001831707	10787	4	614
CN643169	0.56498499	2.12976487	-0.378522116	9.02E-05	10788	4	464
NM_206927	0.02282524	1.57329383	-0.420203631	0.002061768	9059	4	100
NM_206927	-0.1412838	3.42906159	-0.420203631	0.001194814	10774	4	1174
NM_014328	0.1955116	1.38251908	-0.606109947	0.007823498	8899	4	493
NM_014328	0.40998406	1.48286301	-0.606109947	0.000461475	9310	4	231
NM_004695	-0.4578773	1.3704335	-0.280202196	0.00187292	9411	4	525
NM_052874	0.32196641	1.32561191	-0.285000224	0.000975234	9094	4	374
NM_052874	0.42835596	1.36259997	-0.285000224	3.31E-05	9362	4	334

NM_014879	0.72185507	1.96529871	-0.538363719	0.00149107	10792	4	571
NM_014879	0.61341159	2.39782068	-0.538363719	0.000990687	10793	4	698
NM_003357	0.04002794	1.92460965	0.039178419	0.00154326	9587	4	422
NM_003357	0.05117063	2.60321674	0.039178419	0.000798517	10809	4	894
XR_012355	-0.1004829	1.72253568	-0.383312613	0.049972599	8940	4	991
NM_032134	-1.4678159	1.77580683	-0.605065017	0.042620746	9739	4	1127
NM_014629	0.10306095	1.81060146	-0.22301065	0.000647476	9297	4	83
NM_014629	0.37534606	1.75359855	-0.22301065	0.005334874	9146	4	342
NM_079834	0.33621547	1.27994189	-0.346540825	0.031899149	9662	4	808
NM_007104	0.2896535	1.55639216	-0.331232652	0.000222481	9352	4	60
NM_007104	0.36022245	1.86518239	-0.331232652	2.83E-05	9294	4	48
NM_002395	-0.4225824	1.35808717	-0.880179292	0.00306276	8995	4	639
NM_032890	-0.6837516	1.75530724	-0.596476837	0.010926836	6065	4	826
NM_032890	-0.2131924	2.40448634	-0.596476837	0.001542754	10738	4	552
NM_032890	-0.1467534	2.82247231	-0.596476837	0.000547305	10739	4	957
NM_032890	-0.9788688	1.68423367	-0.596476837	0.027493358	6063	4	1057
NM_000159	-0.0905468	1.40635322	-0.655810451	0.024160201	8971	4	732
BU933087	-0.2045536	1.43714745	-0.682163609	0.008294522	9040	4	368
BU933087	-0.2112305	2.50529054	-0.682163609	0.001249376	10730	4	629
NM_006350	0.13965956	2.03410456	-0.21993494	0.040269885	10678	4	1052
NM_006350	0.45332275	2.4837539	-0.21993494	0.000330235	10795	4	797
NM_032255	0.05622028	1.34085439	-0.862504443	0.00943025	6541	4	798
NM_023011	-0.7641228	2.48116028	-0.66679133	0.000445072	10728	4	928
NM_023011	-0.5431613	2.55524992	-0.66679133	0.001418232	10729	4	864
NM_025176	-0.1067708	1.84154487	-0.559238386	0.001871685	8933	4	123
NM_025176	-0.7126545	1.73371182	-0.559238386	0.002585043	9404	4	617
NM_145306	0.38885931	1.2652356	-0.41885914	0.004027688	9363	4	459
NM_080574	0.62745175	1.94297763	-1.130968357	0.003453341	10574	4	902
NM_080574	0.62865434	2.2805989	-1.130968357	0.033426954	11222	4	1189
NM_003273	-0.4080342	1.38379149	-0.043527956	0.029308179	9102	4	741
NM_017512	0.27555917	1.83912445	0.187536092	0.017877314	9618	4	744
NM_017512	-0.3852704	2.01030599	0.187536092	0.019271697	10649	4	999
XR_012028	0.65131843	1.17181392	-0.655603806	0.023078466	9726	4	1117
NM_024855	-0.3096298	1.5440647	-0.387206336	0.017855866	6067	4	489
NM_018676	-0.1847076	1.29702832	-0.317157362	0.012113752	9463	4	569
NM_003881	0.21438587	1.41092421	-0.317031113	6.07E-07	9092	4	137
CN647114	-0.0422353	1.24540751	-0.3359752	0.000118447	9083	4	218
CN647114	-0.171705	1.86808321	-0.3359752	0.016960554	10682	4	527
NM_016339	-0.2956488	1.53481636	-0.589843718	0.001121294	9416	4	268
NM_003786	-0.7617521	1.28852427	-0.015037878	0.027334214	9437	4	987
NM_003786	-0.4678151	2.34732046	-0.015037878	0.002012801	9379	4	875
NM_004462	0.13107358	1.30134777	-0.208629821	0.007978042	9128	4	353
NM_004462	0.01135972	1.3497088	-0.208629821	0.024560039	9129	4	477

NM_005822	0.56041652	1.3459616	-0.717128786	0.003903204	9331	4	542
NM_000894	0.74654549	1.77190824	-0.590386718	0.007120297	10785	4	821
NM_000894	0.79431004	2.05247954	-0.590386718	0.001579245	10786	4	751
NM_139208	0.75282714	1.38120361	-0.68670197	0.002655042	10411	4	746
NM_004586	0.30028876	1.41927578	-0.341615887	0.000882609	9359	4	146
NM_181712	1.87588168	3.24848479	-0.571759582	0.002421342	11233	4	1228
XR_012588	-0.2372776	2.18209503	-1.100469371	0.003768671	10129	4	853
XR_012588	-0.3583511	1.60868607	-1.100469371	0.012437407	10125	4	873
NM_024574	0.1326793	1.43373083	-0.201673239	0.016868756	8935	4	505
NM_024574	0.16291015	1.34204254	-0.201673239	0.000661616	9093	4	286
NM_181784	1.09763209	1.42903925	-0.421028763	0.041678461	9702	4	1129
NM_004241	-0.8629247	1.30714711	-0.309359078	0.005137987	9425	4	910
NM_153335	-0.1096904	1.22464086	-0.351045402	0.002613524	9054	4	384
NM_153335	-0.4179294	1.39810769	-0.351045402	0.001529808	9056	4	356
NM_153335	-0.2053984	1.53664096	-0.351045402	0.001459451	9057	4	98
NM_153335	-0.1663493	1.54945819	-0.351045402	0.003371548	9058	4	149
NM_002167	0.40403914	1.51976185	-0.442151693	0.001565557	9350	4	147
NM_002167	0.17056315	1.73261264	-0.442151693	0.000648088	9282	4	30
NM_139247	0.04866352	1.63367604	-0.117570247	0.00066739	9139	4	171
NM_139247	0.17691357	1.79102266	-0.117570247	0.0071571	9389	4	358
NM_012258	0.34677942	1.66528055	-0.436522999	0.009457589	9390	4	294
NM_012258	0.92890511	2.36247845	-0.436522999	0.003286481	10834	4	1110
NM_014836	0.36857424	1.57079901	-0.292162841	0.006013843	9144	4	304
NM_006152	0.30024842	1.1979439	-0.44365956	0.000292363	9355	4	355
NM_006152	0.51722076	1.59663271	-0.44365956	0.000122248	9300	4	178
NM_005324	-0.1824595	1.2663835	-0.328037651	0.002040445	9117	4	280
NM_032924	-0.3516869	1.3364944	-0.610615797	0.033740425	6535	4	950
NM_032476	0.29979476	1.29127245	-0.191784324	0.000682966	8576	4	351
NM_032476	0.26314342	1.41478493	-0.191784324	0.001100316	9140	4	232
NM_016355	0.09655324	1.40775555	-0.343155336	9.55E-06	9091	4	122
NM_016355	0.20552408	1.27537233	-0.343155336	0.002684768	9090	4	253
NM_025265	-0.0644317	1.82428871	-0.811987264	0.001381853	10715	4	487
NM_145649	-0.0269138	1.29599546	-0.06947668	0.018857949	9103	4	634
NM_058241	-0.6902318	1.61709658	-0.383386382	0.01879321	6066	4	802
XR_010229	-0.258766	1.90962613	0.120536445	0.015827521	9380	4	779
NM_023928	0.34492349	1.56515362	-0.16924259	0.001905671	9672	4	425
NM_012137	0.00178113	1.91569264	-0.288118473	0.036522949	10684	4	793
NM_000048	-0.3047757	1.26916397	0.14685609	0.019911482	9511	4	831
NM_014970	0.75662383	1.29293621	0.110318632	0.012700069	9607	4	976
NM_017815	0.30066512	1.28201457	-0.283496698	0.000268697	9357	4	309
NM_017815	0.47608905	1.44797312	-0.283496698	0.000811112	9360	4	283
NM_207168	-0.4365344	1.58176923	0.041757892	0.034631638	10647	4	814
NM_024897	-0.2109874	1.41783117	-0.012574961	0.031498333	9465	4	881

NM_018044	0.21671419	1.60877739	-0.150122474	0.001348708	9141	4	136
NM_018044	0.34073857	1.58698425	-0.150122474	0.005059738	9145	4	292
A_01_P019871	1.18729726	1.31180677	-0.460016446	0.026292721	9703	4	1137
NM_002719	0.07690799	1.44168608	0.142820343	0.00775176	9105	4	601
A_01_P016929	-0.174475	3.01803901	0.110766335	0.002271803	10770	4	1142
NM_003297	-0.2436797	3.16729352	-0.781184728	0.039651805	11183	4	1225
NM_012255	-0.1257953	1.19319149	-0.214429017	0.011953928	9118	4	514
NM_144686	0.26697533	1.22976537	-0.242683464	0.001151405	9080	4	461
NM_144686	0.15255008	1.31903638	-0.242683464	0.00105017	9079	4	345
NM_025257	0.83863743	1.44231267	0.358570392	0.036373678	9608	4	1094
NM_025257	0.61783103	1.93485959	0.358570392	0.010495029	9614	4	1004
XM_496408	-0.2105788	1.4978793	-0.060458599	0.001815392	9564	4	329
NM_032468	-0.0508129	1.33962479	-0.145084558	0.012313027	9120	4	544
NM_032468	-0.2187997	1.82318636	-0.145084558	0.020161966	10646	4	738
NM_173558	0.07775315	1.29622402	-0.59108237	0.012946182	8900	4	627
NM_005240	0.07933959	3.25997317	-0.153833719	0.000362058	10814	4	1160
NM_006032	0.94109412	1.24763484	-0.050308178	0.015656884	9685	4	1055
CB550361	-0.0450913	1.20648534	-0.2202578	6.04E-05	9068	4	396
CB550361	0.05738782	1.38981784	-0.2202578	0.000868346	9098	4	238
NM_053025	0.10920844	1.71828039	-0.264090398	0.00053195	9296	4	68
NM_053025	-0.0333535	1.85800213	-0.264090398	0.016333568	9388	4	475
NM_003941	0.10976379	1.21652043	-0.290540763	0.03814694	9126	4	626
NM_030577	0.82508641	1.98476665	-0.285428053	0.01854149	10801	4	1046
NM_006664	-1.4150574	1.92746864	-0.919625016	0.013290211	5927	4	1233
NM_139057	0.38554553	2.06858682	-0.239843542	0.0009565	9392	4	302
NM_021003	0.47461184	1.26554384	-0.274184433	0.000580747	9361	4	521
NM_002777	0.69006517	2.01099113	-0.949335069	0.045244599	10627	4	1154
NM_006624	-0.0045879	1.23456204	-0.401087817	0.020070784	8980	4	715
NM_006624	0.47131679	1.44914754	-0.401087817	0.004678342	10412	4	451
NM_139168	-0.1034618	1.47702346	-0.378567712	0.0104657	9041	4	313
NM_198474	-0.6452565	1.70361864	0.246536994	0.00534355	9453	4	1006
NM_015234	0.26780776	2.09835057	-0.390850509	0.00010531	10759	4	99
NM_015234	0.14231498	2.24094316	-0.390850509	7.53E-05	10760	4	152
NM_020956	0.12366763	1.25109222	-0.078273175	1.34E-05	9075	4	421
NM_014608	-0.2355155	1.27528942	-0.179870849	0.006997945	9119	4	480
NM_014608	0.04691897	1.75688822	-0.179870849	0.002638203	9567	4	115
NM_198328	0.44011804	1.62765055	-0.308299815	0.019198532	9385	4	772
NM_000673	0.69890119	1.60693486	-0.647497739	0.007544479	10413	4	717
NM_005856	-0.0906517	2.73304629	-0.694203015	0.000280345	10740	4	833
NM_005856	0.03776298	2.82175401	-0.694203015	0.000349915	10741	4	899
NM_003458	1.28613135	1.49278892	-0.504719915	0.017578326	9704	4	1145
NM_003458	0.85828022	2.4961001	-0.504719915	0.042907565	11223	4	1192
NM_173158	1.25608348	1.47736268	-0.301317572	0.019197744	9705	4	1131

NM_005268	1.43054631	1.87212154	-0.40235157	0.019921652	9706	4	1168
NM_005268	1.70366742	3.28552611	-0.40235157	0.007475384	11234	4	1232
NM_031281	0.73010636	1.93039925	-0.803402265	0.046098418	10626	4	1143
NM_006994	-0.0897041	1.74582658	-0.024911578	3.44E-06	9571	4	179
NM_006994	0.02199427	1.84089467	-0.024911578	5.27E-05	9572	4	168
NM_018082	0.44453818	1.27503869	-0.28679223	0.026556815	9143	4	682
NM_018948	-0.0858479	1.3991973	-0.048948193	0.009726827	9104	4	465
NM_018948	0.21945552	1.185714	-0.048948193	0.033846712	9130	4	703
NM_025109	-0.5503811	1.50879293	-0.103532197	0.006256345	9422	4	704
CN647246	0.36441372	1.62572749	-0.18574962	0.002668738	9666	4	428
CN647246	0.27927819	1.46144593	-0.18574962	0.000463489	9099	4	295
NM_138409	-0.0580949	2.13772306	0.244859083	0.000814577	9588	4	718
NM_138409	-0.1989998	2.87206152	0.244859083	0.00495632	10813	4	1147
NM_018192	0.13879007	2.80010766	-0.270461477	0.000202382	10766	4	891
NM_018192	0.01579595	2.82229785	-0.270461477	0.000781664	10765	4	932
NM_031924	0.03157636	1.2159405	0.054087294	0.00010139	9514	4	700
XM_291054	0.54269584	2.21492627	-0.075542552	0.003554246	10833	4	962
CO581492	-0.1921712	1.33150024	-0.202518092	0.000299922	9085	4	322
CO581492	-0.0352744	1.44875796	-0.202518092	5.76E-05	9086	4	140
NM_012098	0.58079054	1.50375685	-1.089044104	0.038177987	10615	4	1102
CB554423	0.81587878	1.34136864	-0.016653261	0.034184799	9686	4	1018
CB554423	0.43890907	1.16142321	-0.016653261	0.000351275	8580	4	709
NM_006832	-0.0893806	1.74856753	-0.098804272	0.000415004	9573	4	276
NM_006832	-0.1206663	1.68172576	-0.098804272	0.001676296	9566	4	198
NM_014647	-0.2000712	1.25668395	0.019625744	0.015519764	9464	4	812
NM_153041	0.74483951	1.46199932	-0.346013936	0.009899966	9668	4	941
NM_153041	0.51854824	1.90780965	-0.346013936	0.000235677	9301	4	236
NM_005357	0.37246639	2.21484241	0.251731928	0.012649758	10804	4	1003
NM_005357	0.4312948	2.41754502	0.251731928	0.009983843	10805	4	1068
NM_015001	0.39550878	1.34151594	-0.173730833	0.000155455	9096	4	448
NM_015001	0.16737828	1.28863335	-0.173730833	8.00E-05	9095	4	335
NM_016122	0.08088811	2.7393832	-0.056801194	0.003477711	10810	4	1047
NM_016270	0.07542769	1.2554498	-0.226423033	0.00145588	9089	4	321
NM_016270	-0.0100443	1.37763202	-0.226423033	0.001272828	9084	4	208
NM_018652	-0.6352665	1.38656858	-0.138159758	0.041857692	9443	4	944
XR_013530	0.07755769	1.3686915	-0.779999392	0.007421186	8998	4	438
XR_013530	0.11836885	1.52182395	-0.779999392	0.007505379	8999	4	376
NM_021145	-0.9439086	2.10013832	-0.341672002	0.003513662	6068	4	946
CN801659	-0.1442815	2.26942873	0.0764435	0.006917846	9381	4	801
CN801659	-0.0441074	2.78511559	0.0764435	0.004107252	10812	4	1065
NM_005766	-0.6220068	1.81946745	0.027636561	0.000304783	9458	4	825
NM_005766	-0.1017771	1.87797722	0.027636561	0.002826667	9574	4	450
NM_031953	-1.0696244	2.38932966	0.25171115	0.031769296	10820	4	1177

NM_031953	-0.5861803	3.10421476	0.25171115	0.019452224	10840	4	1198
CN645773	-0.0711926	4.07875538	-0.098033042	0.046407553	11410	4	1239
NM_005502	0.08086298	1.19047897	-0.040969681	0.008478539	9132	4	573
NM_005502	0.13767566	1.28375584	-0.040969681	0.010117202	9134	4	519
CK231978	0.02357648	1.62840604	0.061783727	0.000229696	9570	4	333
CK231978	-0.187196	1.54727974	0.061783727	0.013640757	9565	4	549
NM_004105	0.08755272	1.4343502	-0.192974954	0.006628672	9137	4	306
NM_004105	-0.0239971	1.65061312	-0.192974954	0.00480778	9042	4	275
NM_000298	0.79740099	1.44663434	-0.260625188	0.013944421	9684	4	889
NM_015997	0.19910443	1.99217475	-0.153790307	0.000575364	9595	4	336
NM_015997	0.43072454	2.65413196	-0.153790307	0.000123877	10799	4	874
NM_001007258	-0.6784642	1.54626607	-0.104930948	0.023661827	9418	4	966
NM_032029	0.85181345	1.72313033	-0.349387188	0.049052751	9700	4	1112
NM_001698	0.11500246	2.03447968	-0.222633596	0.000532868	10758	4	105
NM_001010985	-0.3396232	1.6212526	0.460090556	0.03251932	9455	4	1058
NM_001010985	-0.4043658	1.68645729	0.460090556	0.019632486	9454	4	1040
NM_182532	0.17191256	2.22925636	0.47196042	0.049082941	10802	4	1139
NM_147150	-0.2246195	1.65972676	-0.06817327	0.000403463	10639	4	533
NM_147150	0.2515031	1.51404419	-0.06817327	0.011349468	9138	4	490
NM_005613	0.46994707	2.56672057	-0.955575531	0.004142609	10777	4	1069
NM_004910	-0.0276602	1.38199512	-0.05868296	0.011915182	8987	4	657
NM_004910	0.45158772	1.51042949	-0.05868296	0.013472636	9667	4	766
NM_015423	0.64654825	1.16130698	-0.103913624	0.009309394	9683	4	867
A_01_P016928	-0.2408922	2.82574175	-0.134500721	4.68E-05	10763	4	974
A_01_P016928	0.15362438	2.95859733	-0.134500721	7.23E-05	10767	4	1036
BM423206	-0.0954268	1.7430072	0.087022574	0.000240271	9582	4	415
BM423206	-0.1452872	1.70933819	0.087022574	0.000388282	9569	4	395
XR_010710	-0.6722957	1.39778762	0.095732059	0.000314701	9432	4	855
NM_183422	0.76035193	2.01228955	-0.023267925	0.003499957	9394	4	799
NM_183422	0.80702853	1.99553811	-0.023267925	0.004888661	9395	4	841
NM_198976	-0.1368835	1.22934888	0.180305847	0.00021719	9528	4	728
NM_001010983	-0.0166974	1.32581571	-0.279838154	0.001599743	9087	4	230
NM_001010983	0.00628084	1.31549629	-0.279838154	0.006425239	9088	4	311
NM_016593	-0.3893333	1.43586446	-0.30788474	0.006747886	9438	4	478
NM_003713	-0.2659641	1.75073681	0.102430618	3.30E-05	9568	4	442
NM_003713	-0.3751755	1.39167212	0.102430618	0.011459124	9124	4	706
XR_013303	-0.3220585	1.30806156	-0.723272455	0.011324837	8996	4	651
NM_021161	-1.8988623	1.45132038	-0.146164891	0.020644576	6027	4	1231
NM_024642	-0.6239556	1.47300289	0.172096245	0.004412333	9516	4	882
XM_930351	0.0931169	1.78296626	-0.143291951	0.010161533	10685	4	456
CN647387	-0.3813458	1.43119149	-0.165085311	0.01759249	9420	4	663
CN647387	-0.4738363	1.46842281	-0.165085311	0.001593858	9439	4	504
NM_022783	-0.5619105	1.8356729	0.203908996	0.006796638	9459	4	1015

NM_022783	-0.2989036	1.40487438	0.203908996	0.002071637	9515	4	805
NM_024519	0.06545343	1.22858433	0.27555435	0.04262695	9523	4	936
NM_000297	-0.682479	1.33350982	0.231682664	0.003488804	9433	4	970
NM_153343	0.20332554	1.31915678	-0.674395065	0.028571581	8994	4	761
NM_015462	0.51787503	1.37792242	0.497102974	0.028758957	9604	4	1042
NM_181519	0.31339675	1.46067568	0.10182336	2.15E-05	9592	4	532
NM_181519	0.25417039	1.48758173	0.10182336	4.69E-05	9557	4	554
XR_013643	0.230887	1.7304188	0.489727356	0.037017233	9600	4	1005
NM_000245	0.1158184	1.33485763	0.006121746	0.00821432	9133	4	517
NM_173469	-0.0836625	1.53390415	-0.439903308	0.046141818	10681	4	787
BC057815	0.20534363	1.18281215	0.215694393	0.000446534	9533	4	813
NM_033254	-0.0669651	2.35260867	-0.257904203	0.000507597	10761	4	412
NM_033254	-0.1329832	2.94946383	-0.257904203	0.0004023	10764	4	1029
NM_022753	0.3412701	1.47500789	-0.582036263	0.011460882	9002	4	447
NM_022753	0.09562815	1.38113638	-0.582036263	0.018181826	9000	4	467
NM_014341	0.08794549	1.18637871	0.136451313	0.015101851	9135	4	742
NM_014341	0.15076342	1.34549589	0.136451313	0.004138476	9136	4	604
NM_024514	0.11275116	1.4692928	-0.02019654	0.038304319	8988	4	776
NM_015194	-0.2002151	1.25738258	0.042625024	0.009089932	9122	4	632
NM_015194	-0.1569882	1.36738736	0.042625024	0.011215282	9123	4	563
XR_013772	0.47708626	1.36932572	0.505347776	0.014881187	9603	4	1032
XR_013772	0.40682827	1.68879776	0.505347776	0.004241419	9606	4	994
NM_174899	0.58702664	2.02110453	-0.163919826	0.023945992	10800	4	988
NM_022462	-0.0778551	1.87735319	0.291172967	0.004757053	9581	4	712
NM_022462	-0.0333352	2.65311289	0.291172967	0.008068943	10811	4	1107
NM_004624	-0.3314322	1.30474377	0.02176038	0.047237452	9421	4	914
NM_177967	0.17384894	1.34389302	-0.347693031	0.007923837	8984	4	460
XM_374765	-0.8871161	1.52504327	0.216058995	0.015242464	9444	4	1062
NM_002004	-0.225722	1.56086396	0.348826945	0.015477106	9579	4	828
NM_002004	-0.2447581	1.6259939	0.348826945	0.011682461	9580	4	815
NM_001008390	0.43439856	1.83729419	-0.322850837	0.017040218	9391	4	581
NM_002135	0.00935826	1.28142341	0.445745931	0.029564638	9546	4	990
NM_002135	-0.1778122	1.49107367	0.445745931	0.02046404	9456	4	964
NM_020455	-0.5478059	1.86911866	-0.337802313	0.021094347	6545	4	898
NM_024320	0.01548834	1.39793888	0.08755577	0.009167623	9125	4	578
NM_024320	0.29686638	1.50438066	0.08755577	0.023334077	9147	4	656
NM_003715	0.02406045	1.35778064	0.175011644	0.000158038	9531	4	616
NM_012067	-0.1983577	1.7486242	0.388319417	0.001479631	9457	4	857
NM_024807	0.92682055	1.19808699	-0.116766356	0.040001143	9687	4	1097
NM_024807	0.84647591	1.65718214	-0.116766356	0.013648962	9688	4	952
CO645930	0.21305812	1.44405337	-0.220991858	0.018910713	8985	4	624
NM_144689	-0.2096234	1.55328859	-0.775512177	0.035290855	10680	4	876
NM_023037	-0.9797731	2.46556856	0.484186082	0.042740771	10837	4	1202

NM_000210	0.39799518	1.30481199	0.31910667	0.002198576	9609	4	886
NM_024059	0.51271512	1.20131755	0.22922794	0.01309757	9680	4	965
XR_014794	-0.2106022	1.83756668	0.261513702	0.00198812	9585	4	735
XR_014794	-0.214025	1.90430322	0.261513702	0.000221655	9586	4	677
NM_012294	-0.8161533	1.65424148	0.136093599	0.000698065	9450	4	923
NM_012294	-0.5334442	2.06659173	0.136093599	0.039632683	10648	4	1035
NM_012294	-0.0537689	2.80974978	0.136093599	0.000971465	10768	4	1053
NM_012294	0.08884076	2.93947856	0.136093599	0.000365098	10769	4	1088
XR_014258	0.15704642	1.29812125	0.260004077	0.000286697	9535	4	760
XR_014258	-0.1526113	1.37035937	0.260004077	0.006378299	9537	4	745
NM_020423	-0.4301309	1.52975158	0.004523725	0.005482388	10632	4	954
NM_002871	0.5036413	1.62709546	0.069321312	0.045770198	9397	4	920
XR_012426	0.38488637	1.75190427	0.218909812	0.001116987	9594	4	701
XR_012426	0.49329984	2.11218072	0.218909812	0.003549136	10803	4	907
NM_021930	-0.5968353	1.86668424	-0.115262446	0.008699617	10645	4	922
NM_001086	-0.4518968	3.80185333	0.207857282	0.006985207	10841	4	1226
NM_175605	0.05173756	1.45917532	-0.671632194	0.018053997	9001	4	557
NM_175605	-0.1034724	1.62506485	-0.671632194	0.024225105	8997	4	644
NM_013961	-0.0761046	1.88991375	0.1029615	0.005346565	10633	4	996
NM_002337	0.02910858	1.38553513	0.285460142	1.85E-05	9532	4	727
NM_002337	0.16292443	1.24920186	0.285460142	0.000237381	9534	4	816
NM_032495	-0.6070857	2.02159427	0.249947745	0.002190198	10650	4	892
NM_032495	-0.5566105	2.00046881	0.249947745	0.008254026	10651	4	903
NM_004788	-0.1903743	1.28190431	0.330199689	0.009351699	9536	4	896
NM_147156	-1.1147834	2.8306373	0.536420997	0.036253414	10836	4	1216
NM_018032	-0.1848404	1.96881632	0.277911503	0.002059782	9577	4	696
NM_018032	-0.230793	2.26141213	0.277911503	0.004953098	10653	4	880
NM_004645	0.26036977	1.48632946	0.573038625	0.029663287	9601	4	1041
NM_006243	-0.3879554	1.33891661	0.301312557	0.002259738	9519	4	900
NM_006243	-0.3319571	1.35951764	0.301312557	0.001273746	9520	4	869
NM_017898	-0.6107412	3.12196973	0.181850411	0.027874156	10838	4	1209
NM_005411	-0.4250919	2.34432446	0.368435771	0.000341357	10655	4	1011
NM_005411	-0.4044322	2.43367286	0.368435771	0.000100175	10656	4	1024
NM_004684	-0.3767333	2.45869282	0.405934162	9.71E-05	10657	4	1025
NM_004684	-0.301932	3.196331	0.405934162	0.003286076	10839	4	1191
NM_005410	0.91853636	1.7474048	0.300012133	6.39E-05	9613	4	1017
NM_005410	0.94715225	1.66292495	0.300012133	0.001105785	9612	4	1044
NM_004925	-0.0321648	1.19501059	0.37668446	0.031492696	9538	4	943
NM_207395	0.2080864	1.20572577	0.109247982	0.001758721	9556	4	770
NM_003665	-0.0857973	1.71819538	0.206400062	0.003861666	9584	4	665
NM_003665	-0.1287455	1.57913126	0.206400062	0.001633189	9583	4	647
NM_001735	-0.6604745	2.12415476	0.31281424	0.011215331	10652	4	1037
NM_001735	-0.2758824	2.43339609	0.31281424	0.00394018	10654	4	993

NM_006471	0.61210161	1.39783986	0.386477363	0.003664332	9610	4	986
NM_006471	0.60931157	1.63571389	0.386477363	0.002745377	9611	4	929
NM_005134	0.13566089	1.6779409	0.097302172	0.001018181	9591	4	458
NM_005134	0.18724953	2.01733915	0.097302172	0.000325377	9596	4	469
NM_025208	0.1208318	2.46548551	0.370491906	0.020820396	10660	4	1089
NM_001300	-0.6330426	1.6504572	0.228065932	0.001145827	9451	4	866
NM_001300	-0.6369632	1.85294589	0.228065932	0.000369015	9452	4	870
NM_000419	-0.6501788	1.40397598	0.592484772	0.031470099	10634	4	1161
NM_152544	-0.3640543	1.29121752	-0.002874443	0.027086758	9441	4	803
NM_052845	1.20322877	2.32726543	-0.20704667	0.015288033	10806	4	1162
NM_003194	0.50819706	1.15025999	0.369758587	0.034615452	8613	4	1051
NM_007159	0.31262096	1.50858917	0.51983123	0.000367399	9602	4	959
NM_004851	-0.5700021	1.23627732	0.504875796	0.017077155	9518	4	1098
NM_032921	-0.0076412	1.38653574	0.445851237	0.017820683	9578	4	939
NM_152287	0.01895031	1.52162668	0.212494708	0.004496399	9590	4	669
NM_152287	-0.1761715	1.29159306	0.212494708	0.002308897	9530	4	749
NM_031431	-0.9359262	1.89528367	-0.329741092	0.046240668	10669	4	1119
NM_005647	-0.4623406	1.47770444	-0.039498991	0.008524188	9449	4	720
NM_000196	0.52851781	2.33779672	0.592734237	0.00028102	10817	4	1099
NM_000196	0.45347326	2.92538337	0.592734237	6.39E-06	10818	4	1166
CN803179	0.1331769	2.15819589	-0.794787862	0.009448591	10776	4	789
NM_201222	0.15746126	1.44954646	0.744401704	0.031682348	9554	4	1106
XM_496807	0.0007386	1.18103216	0.52293261	0.010436124	9540	4	1026
XM_496807	0.0298372	1.33246238	0.52293261	0.002309564	9541	4	978
NM_020467	-0.4797987	1.63066354	-0.27902009	0.019078648	10637	4	791
NM_012414	0.29791134	1.4468408	0.479755849	0.000466807	9553	4	960
NM_012414	-0.1289051	1.69716473	0.479755849	0.009016585	9576	4	918
CN802687	0.0200887	1.58792936	-0.04960961	0.011331166	9562	4	443
CN802687	0.01640428	2.01229075	-0.04960961	0.017851196	9386	4	674
NM_002537	0.35553528	1.21026591	0.103919181	0.021005224	9560	4	879
NM_002537	0.57843212	1.57149669	0.103919181	0.004036564	9593	4	758
NM_000617	0.09771854	1.29236793	0.489047813	0.000650494	9551	4	979
NM_012245	-0.2284105	1.23615553	0.458145805	0.000213158	9521	4	989
NM_003275	-0.698232	1.91868785	0.429477537	0.046774662	9620	4	1120
NM_002543	-1.012067	1.49276486	0.437572983	0.002366402	9448	4	1144
CN805323	0.10407272	1.2900237	0.486954105	0.008022487	9552	4	1013
NM_002392	3.00916319	1.47396649	0.10955255	0.026149051	11255	4	1237
NM_002392	3.47274578	1.31996594	0.10955255	0.011212602	11256	4	1241
NM_002392	3.00718039	1.33757484	0.10955255	0.017521902	11254	4	1236
NM_002392	2.8913846	2.79680634	0.10955255	0.000821894	11258	4	1240
NM_002392	2.95959935	2.41026688	0.10955255	0.012766658	11257	4	1238
NM_002392	3.57861396	2.9890876	0.10955255	0.019639449	11259	4	1242
NM_004683	0.22990179	2.65562911	0.202360385	0.000943565	10815	4	1023

NM_004683	0.61168318	2.59742997	0.202360385	0.002703347	10816	4	1067
NM_000907	-0.0243198	1.34421345	0.710791243	0.009619422	9542	4	1082
NM_000289	0.27874076	1.62781949	0.100539349	0.043846732	9149	4	780
NM_004206	-0.6441136	1.78486397	-0.140169454	0.008215384	10638	4	985
NM_205842	0.16145396	1.19947574	0.42142697	0.043169235	9545	4	995
NM_138762	0.48965375	1.22661178	-0.174053898	0.04141525	8989	4	953
NM_005308	-0.4749961	1.43789385	0.405352529	0.00418369	9517	4	969
NM_002130	0.49063611	1.42584718	0.795956829	0.001524838	9605	4	1132
NM_173471	0.87843777	2.27086074	-0.742439263	0.03154319	10775	4	1167
NM_014326	0.29921433	3.65952335	0.552447673	0.002216952	10844	4	1221
NM_002482	0.62942063	2.59992733	-0.319181947	0.008255126	10778	4	1077
NM_002482	0.71731065	2.61079858	-0.319181947	0.008273886	10779	4	1104
NM_001613	-0.1070826	1.23487722	0.692835504	0.012874363	9548	4	1105
NM_003919	0.26781729	1.76456711	-0.087506544	0.031249559	10686	4	775
NM_006257	-0.1337348	1.38126539	0.662858368	0.009342136	9547	4	1092
NM_006257	-0.2934847	1.53931032	0.662858368	0.001300441	9622	4	1059
CN802260	-0.1310221	1.37097813	0.685930052	0.036791391	9549	4	1095
NM_003129	-0.2997345	2.48113172	-0.094418764	0.016246428	10807	4	1087
NM_004557	0.02404927	2.31545908	0.365750692	0.032444773	10808	4	1108
NM_004557	-0.1955675	1.79256451	0.365750692	0.014106288	10642	4	912
DV769210	-1.4749168	1.84784367	-0.490664513	0.042063432	6054	4	1193
XR_013675	-0.193168	1.57831669	1.06345726	0.03734906	9625	4	1169
NM_000928	-0.1669038	1.68265546	0.790511168	0.001366184	9623	4	1096
NM_000928	-0.2471933	1.82897006	0.790511168	0.000294667	9624	4	1101
NM_022127	-0.3747805	1.24812485	0.915315108	0.027554887	9621	4	1165
NM_020453	-0.3761496	2.40409487	0.731062217	0.003542503	10658	4	1152
NM_018476	0.0378316	1.30656607	0.832354767	0.02634292	9550	4	1136
NM_001885	-0.6549745	1.86234881	0.196646253	0.024536335	10641	4	1021
NM_001885	-0.4024616	1.99350978	0.196646253	0.021598787	10643	4	977
NM_001010877	-0.6588192	1.7962114	-0.294017729	0.046306979	10640	4	1039
NM_001442	-0.1077497	3.18511843	0.573815594	0.004831968	10843	4	1195
NM_001442	-0.2717619	2.59747208	0.573815594	0.040923794	10842	4	1170
NM_000228	-0.0519638	1.43485738	1.169102601	0.017581219	9627	4	1179
NM_000228	-0.2264662	1.55546159	1.169102601	0.007221054	9626	4	1176
NM_005656	-0.2120526	2.46667644	0.519545748	0.012931872	10659	4	1128
NM_018346	-0.6200648	1.96839902	0.844976599	0.002134246	10635	4	1173
NM_005239	-0.0664933	1.50613952	1.203139826	0.019972363	9628	4	1187
NM_005239	0.29749662	2.63668363	1.203139826	0.001332638	10848	4	1203
NM_006770	-0.1434211	2.73675524	1.313027696	0.000795915	10845	4	1212
NM_006770	-0.0760267	2.91582783	1.313027696	3.66E-05	10846	4	1217
NM_001153	0.36126934	2.60060839	1.108800277	0.001751877	10847	4	1197
NM_006926	-1.0689097	2.57762518	2.135025499	0.001474786	11235	4	1234
NM_006926	-1.1447977	2.76509481	2.135025499	0.001765047	11236	4	1235

Cluster 3

Gene Name	Av Normal	Av M (w12)	Av M (w4)	*P*	Hierarchical Clustering (order)	K-means clustering	K-means clustering (rank)
NM_006564	-1.9459028	-2.7541924	-0.218880717	0.045746657	11267	3	1696
NM_145738	-2.7101724	-0.9502	-1.292425943	0.034732036	9	3	1699
NM_002229	-1.0451102	-1.7309015	-1.00188941	0.037557567	138	3	1658
NM_002229	-2.060413	-1.6646283	-1.00188941	0.037824978	134	3	1679
NM_024509	-0.3860861	-1.3818355	-0.687006705	0.017989204	1478	3	1563
NM_024509	0.13934982	-1.3174902	-0.687006705	0.032781124	1476	3	1591
NM_214710	-0.4323752	-2.0619221	-0.757489733	0.000118831	140	3	1644
NM_214710	-0.3313151	-1.4982504	-0.757489733	0.001735526	1477	3	1585
NM_025182	-0.7379411	-1.3198046	-0.603946907	0.014006709	1506	3	1556
NM_015399	-0.8390486	-1.257873	-0.17968222	0.009825707	1508	3	1392
NM_015399	-0.7900048	-1.2536805	-0.17968222	0.010490393	1509	3	1305
NM_033102	-1.2923307	-2.0856216	-0.386359976	0.002075284	136	3	1670
NM_033102	-0.3928425	-1.7698922	-0.386359976	0.000569612	1479	3	1539
XR_012342	0.09619911	-0.7359526	-0.384735077	0.019220498	1853	3	1367
XR_012342	0.00792526	-0.7331033	-0.384735077	0.043560876	1852	3	1322
NM_032797	-0.0305916	-1.309738	-0.211297349	0.010850709	1486	3	1217
NM_032797	-0.0118215	-1.2487607	-0.211297349	0.010999738	1819	3	1215
XR_012747	0.003379	-0.9042832	-0.274852142	0.029516166	1849	3	1164
NM_007069	-0.4274284	-1.8593337	0.167779556	0.012581635	1469	3	1509
NM_007069	-0.2678648	-1.5445051	0.167779556	0.046379569	1470	3	1463
NM_000206	-0.7017358	-2.2024907	-0.383639325	0.0037989	139	3	1629
NM_000206	-1.7125629	-1.9833221	-0.383639325	0.000223642	135	3	1680
NM_145867	0.20575342	-1.0707612	-0.44526243	0.013655661	1824	3	1462
NM_014798	-0.9052598	-1.6443087	-0.517658585	0.001651344	1502	3	1582
NM_014798	-0.8075755	-1.6340447	-0.517658585	0.017461597	1503	3	1580
NM_032251	-0.1333881	-1.0384649	-0.395221555	0.0025076	1821	3	1248
NM_032251	-0.0955345	-0.9411467	-0.395221555	0.032673198	1822	3	1273
NM_001009813	-0.5141913	-1.4832314	-0.339758387	0.0213002	1507	3	1485
NM_016539	-0.447675	-1.1410924	-0.266346075	0.003028292	1492	3	1143
NM_016539	-0.7224206	-1.7245892	-0.266346075	0.009947084	1504	3	1544
NM_138401	-0.5905116	-0.5496084	-0.350714604	0.015744349	1457	3	1358
CO579252	-1.6484193	-0.9295227	-0.063593531	0.015008947	24	3	1688
NM_032306	-0.3442659	-0.9256899	-0.270702309	0.033595646	1494	3	1109
NM_032306	-0.0082011	-0.7311736	-0.270702309	0.04156018	1854	3	1200
NM_138300	-0.4519966	-1.2648412	-0.34787646	0.01390766	1491	3	1324
NM_138300	-0.3589722	-1.1298863	-0.34787646	0.037882838	1493	3	1360
XR_014163	-0.6470845	-0.7404232	-0.037092934	0.019476511	1459	3	884
XR_014163	-0.7486677	-1.3637522	-0.037092934	0.023974183	1517	3	1349
NM_032478	-1.0919943	-0.9972958	0.090208864	0.017492029	1462	3	1192
NM_032478	-0.6032821	-1.0470762	0.090208864	0.012334934	1510	3	618

NM_002517	-1.2117917	-1.2661283	-0.020685115	0.030312858	186	3	1586
NM_053056	0.03685519	-0.6921485	-0.205429478	0.00760025	1855	3	1101
NM_053056	0.05033358	-0.7989896	-0.205429478	0.015778814	1850	3	1072
XR_010591	0.28304597	-1.0181635	-0.036211531	0.006384483	1630	3	1117
XR_010591	0.22710278	-1.0675914	-0.036211531	0.004265723	1629	3	1074
NM_003074	-0.0833581	-1.2651662	-0.427108959	0.004172891	1820	3	1368
NM_003074	0.06701066	-1.028177	-0.427108959	0.029774582	1823	3	1384
NM_003969	0.18265273	-0.9035417	-0.238994157	0.011420096	1856	3	1258
NM_004860	0.29265167	-0.6592796	-0.180283786	0.037285748	1859	3	1315
XR_013413	0.15717754	-1.0621921	-0.266054068	0.004946275	1825	3	1243
NM_183360	0.08591076	-0.5160515	-0.270926922	0.026269256	1865	3	1325
NM_183360	-0.0083893	-0.5007206	-0.270926922	0.03001698	1864	3	1298
NM_173547	0.14639346	-1.5128709	-0.334120873	0.003108755	1484	3	1493
NM_173547	0.01226989	-1.2403366	-0.334120873	0.031523314	1485	3	1459
NM_032415	0.39471083	-1.2289254	-0.181535914	0.005211024	1626	3	1440
NM_032415	0.3015569	-1.2090445	-0.181535914	0.001898711	1605	3	1361
NM_002541	-0.3651893	-0.8629728	-0.175847563	0.013992198	1834	3	877
NM_002541	-0.4450597	-0.855385	-0.175847563	0.013448979	1460	3	1152
NM_002455	-0.0292195	-0.5172491	-0.05474853	0.04617621	1869	3	1005
NM_016459	-0.0257175	-2.4065958	0.010422596	0.003210493	142	3	1652
NM_019112	-0.3932039	-0.9945884	-0.230506453	0.000608564	1833	3	880
NM_019112	-0.2565704	-1.1862866	-0.230506453	0.00767498	1829	3	1014
NM_182488	0.23804205	-0.6321529	-0.203208478	0.032668714	1874	3	1464
NM_003465	-0.2456185	-1.4549353	-0.258878712	0.013157307	1488	3	1370
NM_003465	-0.3211229	-1.5474526	-0.258878712	0.003706075	1487	3	1379
NM_031289	0.90259698	-2.0367077	0.005073508	0.025310535	145	3	1667
NM_003815	0.06265674	-0.9332213	0.001532462	0.012744669	2012	3	726
NM_003815	0.10588764	-0.8515728	0.001532462	0.015488286	2014	3	786
NM_138410	-0.1010503	-1.6818366	-0.071908985	0.00072823	1592	3	1434
NM_138410	0.04427096	-1.0865255	-0.071908985	0.006675108	1628	3	842
NM_020680	-0.2940568	-0.5962464	-0.081066693	0.018215059	1954	3	840
NM_006595	-0.011279	-1.0001634	0.028969039	0.040333173	1499	3	898
NM_006595	0.00162234	-0.7541045	0.028969039	0.040658679	2015	3	790
NM_174921	0.05301469	-1.4293045	-0.26450623	0.030811804	1483	3	1447
CN644776	-0.3706973	-1.6056694	0.78781683	0.043779216	1177	3	1577
NM_173457	-0.0900124	-0.5314259	-0.050306792	0.038891588	1870	3	931
NM_021807	0.0314655	-1.5394669	-0.202649906	0.002131378	1817	3	1369
NM_021807	0.18920674	-1.4740226	-0.202649906	0.002869859	1818	3	1403
A_01_P019559	-1.0793178	-1.0727565	0.175071948	0.044942425	1781	3	1433
NM_031892	0.30197668	-0.8010902	0.370658871	0.016361068	2035	3	933
NM_031892	-0.7930337	-0.7021861	0.370658871	0.02189761	1411	3	1614
NM_015956	0.20381989	-0.7007971	-0.218473829	7.23E-06	1857	3	1249
NM_015956	0.39086047	-0.4720949	-0.218473829	0.001635407	2237	3	1474

NM_138764	0.05321659	-1.6589889	0.014094122	0.007339607	1597	3	1413
NM_138764	-0.3122299	-1.1062257	0.014094122	0.006662247	1496	3	558
NM_138764	0.1089952	-0.9431049	0.014094122	0.023126102	2013	3	947
NM_138764	-0.3010976	-1.7699465	0.014094122	0.027283291	1662	3	1581
NM_003470	-0.2742563	-0.3879574	-0.218424257	0.044413218	2142	3	1334
NM_006289	-0.42485	-0.7670929	-0.096713555	0.020821831	1942	3	804
NM_006289	-0.305886	-0.7570735	-0.096713555	0.001130083	1946	3	602
XM_495935	-0.3201104	-0.6536907	0.22159807	0.032121941	2006	3	367
NM_032850	-0.6760701	-1.4513552	-0.087368536	0.000938253	1525	3	1210
NM_032850	-0.4665139	-1.6161766	-0.087368536	0.008188133	1480	3	1362
NM_001003897	-0.5176897	-1.3025692	0.516905548	0.041577515	1176	3	1406
CK231493	-0.1450933	-0.5196467	-0.095307328	0.049350036	2151	3	1039
NM_007278	0.02214201	-0.7631317	-0.15039731	0.020834145	1851	3	962
NM_016170	-0.2300649	-1.3499939	-0.079953101	0.010181158	1489	3	1125
NM_016170	0.06642298	-1.1748432	-0.079953101	0.006782937	1498	3	960
NM_016170	0.23920622	-1.3522421	-0.079953101	0.006866219	1625	3	1329
NM_205767	-0.2711146	-0.8490881	0.046803509	0.011937734	1943	3	416
NM_205767	-0.3286739	-0.8298018	0.046803509	0.001697167	1947	3	257
NM_019897	-0.3704004	-0.6855613	-0.299381013	0.025187133	1932	3	1201
BX648935	-0.8456148	-0.57173	0.442498049	0.048852957	1467	3	1140
NM_133259	0.4286366	-0.5332248	-0.11753588	0.011001122	2238	3	1443
NM_002688	-0.0570878	-0.5658333	-0.136397423	0.031585897	1863	3	1078
NM_002688	-0.1750289	-0.8066088	-0.136397423	0.021249306	1881	3	1012
NM_006230	-1.1028865	-0.3741251	-0.115693889	0.043600371	1452	3	1525
NM_020440	0.06751836	-0.6015547	0.213313928	0.036335769	2209	3	1120
NM_006712	-0.5779657	-1.2049643	-0.21666486	0.001884204	1513	3	1113
NM_006712	-0.4434477	-0.9038169	-0.21666486	0.001873647	1827	3	934
NM_003721	-0.1128961	-0.4860237	-0.088504482	0.016672627	2145	3	1030
NM_003721	0.01419231	-0.3675031	-0.088504482	0.039058237	2159	3	1233
NM_005652	-0.2039206	-1.0597569	-0.113181437	0.017598349	1830	3	780
NM_014632	-1.5606986	-0.4632524	0.213362916	0.037652275	157	3	1638
NM_001864	-0.1847479	-0.6798792	0.063996509	0.003475048	1951	3	354
NM_001864	-0.2822192	-0.6644409	0.063996509	0.003306485	1950	3	365
NM_033018	-0.0629869	-0.9095326	-0.111313962	0.001164018	1845	3	727
NM_006649	1.11779677	-1.2820943	0.251859768	0.002065743	10868	3	1622
XR_012284	-0.4257603	-0.287047	-0.0645696	0.011542454	2498	3	1223
NM_002809	-0.0364132	-0.506292	-0.014338896	0.003041068	1871	3	864
NM_032706	-0.6906893	-0.9190223	0.703988772	0.036750956	1421	3	1431
NM_022898	0.94432267	-1.0837692	-0.351234766	0.035952796	10867	3	1647
XR_013058	-0.3258922	-0.7482008	0.003435903	0.016770178	1944	3	563
XR_013058	-0.2224801	-0.5188378	0.003435903	0.036582551	2152	3	822
NM_001750	-0.6173579	-0.932336	-0.106169801	0.046224576	1549	3	1033
NM_004739	0.05335874	-0.552506	0.24403349	0.036918852	2024	3	859

NM_012461	-0.0470674	-0.6457415	-0.103711455	0.010760366	1847	3	887
NM_005254	0.25429067	-0.6728651	-0.066848477	0.015715323	1862	3	1234
NM_005254	0.382389	-0.747869	-0.066848477	0.004824402	1877	3	1377
NM_182557	-0.1438901	-0.9126282	0.304114697	0.024556185	1912	3	399
NM_024832	-0.3938261	-0.6851836	0.105937061	0.002361251	1953	3	400
NM_024832	-0.3010935	-0.3532734	0.105937061	0.021040283	2500	3	883
NM_004419	0.0696098	-1.7842472	0.028687751	0.031423161	1472	3	1550
NM_006042	0.24837197	-1.7339726	0.239254086	0.000505561	1596	3	1422
NM_006042	0.12001272	-1.6714121	0.239254086	0.000401994	1595	3	1229
NM_016732	-0.4796549	-0.9163529	0.101756524	0.030102181	1897	3	586
NM_016732	-0.4334101	-0.7319274	0.101756524	0.033364926	1898	3	536
NM_021131	-0.3220411	-0.9157625	0.085552258	0.048397229	1911	3	711
NM_021131	-0.2456312	-0.6907035	0.085552258	0.013423175	1945	3	436
NM_005453	0.00733634	-0.6940941	0.095294731	0.004108985	1788	3	1307
NM_004037	-0.1342478	-0.8856155	-0.203948657	0.013355558	1840	3	938
NM_004037	0.09333112	-0.5256605	-0.203948657	0.00487115	1866	3	1306
NM_003164	-0.501859	-0.9086425	0.239786543	0.0136987	1908	3	305
NM_015560	-0.1693457	-0.3405445	0.233305722	0.048893455	2272	3	1356
NM_178830	-0.3539911	-1.210509	-0.244296443	0.006911452	1826	3	1090
NM_178830	0.00605635	-0.8918974	-0.244296443	0.035346451	1838	3	1151
NM_025058	-0.0479168	-0.5901138	-0.090292194	0.001750425	1867	3	991
NM_007342	-0.2633417	-0.4077334	0.168968808	0.013279391	1888	3	1093
CN645176	-1.4770444	-0.8328485	0.630170797	0.022495845	159	3	1604
NM_002953	-0.0686845	-1.035171	-0.065705874	1.22E-05	1836	3	542
NM_002953	0.13080559	-0.6391257	-0.065705874	4.08E-05	1858	3	973
NM_007363	-0.4874229	-0.421308	-0.079138199	0.018120504	1955	3	1103
AK090447	-0.3161686	-0.7047514	-0.070037267	0.01742955	1882	3	964
NM_005081	-0.7010414	-0.689132	0.327850936	0.035230893	2459	3	1198
XR_013902	-0.2096192	-0.6414022	-0.17899508	0.033069429	1941	3	1020
XR_013902	-0.5698947	-0.6540227	-0.17899508	0.003391501	1458	3	1123
NM_152264	-0.16533	-0.6013066	0.069769722	0.046086679	2135	3	606
NM_014661	-0.1558433	-0.7939867	-0.010596091	0.011815564	1841	3	487
NM_014661	-0.0151677	-0.502608	-0.010596091	0.010203581	2146	3	968
NM_004327	0.0232672	-0.5353135	0.073266715	0.041138719	1873	3	878
NM_004327	-0.6379629	-0.5565328	0.073266715	0.024850171	1878	3	1097
NM_001005752	0.10630514	-1.3991457	0.515465739	0.010506151	1635	3	1196
NM_001005752	-0.0653877	-1.145937	0.515465739	0.006737515	3634	3	455
CN643645	-0.0544859	-0.3962687	-0.061617282	0.024190391	2153	3	1142
NM_002815	0.02386832	-1.2598234	0.065074926	0.00016592	1627	3	549
NM_002815	0.03092724	-1.1205885	0.065074926	0.037850889	2254	3	919
NM_001155	-0.0259892	-0.3156624	-0.020206859	0.006792253	2160	3	1190
NM_182612	0.02124957	-0.693155	-0.116283809	0.005493106	1846	3	940
XR_012910	-0.18827	-0.3818088	0.422394516	0.049697264	2219	3	871

XM_496306	-0.5785497	-1.1414429	0.791719813	0.018376234	1420	3	1402
NM_145064	-0.3415293	-1.4017341	0.283910744	0.000599703	1539	3	385
NM_145064	0.00557761	-1.1736066	0.283910744	0.001189189	1620	3	206
NM_006331	-0.0721204	-1.0983388	0.236325867	0.001629867	1619	3	150
NM_006331	-0.1699631	-0.8409932	0.236325867	0.006896005	2004	3	99
NM_014795	-0.2741843	-1.5066137	0.358606646	0.019660768	1471	3	1182
NM_014795	-0.5718578	-0.9382589	0.358606646	0.022974241	3614	3	510
NM_024044	-0.2674368	-0.9332766	-0.030648589	0.000531423	1835	3	402
NM_024044	-0.3120901	-0.7570527	-0.030648589	0.00179954	1952	3	545
NM_031434	-0.2893443	-0.6402234	0.244819633	0.004673055	2007	3	147
NM_031434	-0.2079321	-0.6133923	0.244819633	0.009945091	2008	3	248
NM_004895	0.20017208	-1.1970612	0.737007912	0.012916569	4614	3	1167
NM_005224	0.11283513	-1.0296176	0.505732047	0.04819568	4609	3	1236
NM_005224	0.14948818	-0.9874062	0.505732047	0.03262523	4611	3	994
NM_156036	-0.7778052	-1.2250187	-0.067888184	0.023901853	1511	3	1224
NM_156036	-0.5644446	-1.0632711	-0.067888184	0.019183728	1519	3	1178
NM_156036	-0.4349348	-1.3085977	-0.067888184	0.042766956	1546	3	1189
NM_005234	-0.476763	-1.4020931	0.200502024	0.003033604	1386	3	1388
NM_005234	-0.4185993	-0.7871417	0.200502024	0.047633683	1784	3	1487
NM_015179	0.15956453	-0.5420821	0.20735995	0.018261715	2222	3	1079
NM_015179	0.36490999	-0.4100015	0.20735995	0.03765388	3343	3	1290
XR_014380	-0.0972801	-1.2914928	-0.012354956	0.002483012	1497	3	921
XR_014380	-0.4653632	-1.2393475	-0.012354956	0.003696939	1783	3	1411
NM_005442	-1.2646941	-2.1222235	-0.190917969	0.0092051	137	3	1674
NM_005442	-0.7106504	-1.5916245	-0.190917969	0.021613772	1501	3	1552
NM_021183	-0.0383294	-0.7706521	0.271116997	0.025492735	2038	3	449
NM_032627	-0.8721024	-0.9783292	0.169354087	0.0009739	1463	3	792
NM_032627	-0.7520191	-0.8642314	0.169354087	0.003038988	1464	3	588
NM_173593	0.2361294	-0.8145567	0.368963067	0.02624545	2023	3	972
NM_016538	-0.5325347	-0.8025108	0.877759875	0.023255698	1711	3	1075
NM_016538	-0.6878816	-0.7279935	0.877759875	0.029998272	1710	3	1253
XR_012912	-0.2461026	-1.3154523	0.390147496	0.002375487	1541	3	268
XR_012912	-0.3334139	-1.2424694	0.390147496	0.001819493	1540	3	138
NM_014463	-0.1433741	-0.7518309	0.315579241	0.043380656	2218	3	492
NM_019085	0.00595687	-0.854707	0.040732349	0.001233666	1987	3	468
NM_019085	0.12741771	-0.698265	0.040732349	0.005592835	1861	3	807
NM_006349	-0.3895228	-0.4626808	0.039212577	0.033743253	1956	3	860
NM_006349	-0.3535536	-0.5274548	0.039212577	0.006116235	1957	3	683
NM_005026	0.3468229	-0.8328075	0.194853867	0.015025814	2231	3	992
NM_005026	0.61367938	-0.5894302	0.194853867	0.041574221	2234	3	1454
NM_006742	-0.3088046	-0.6267062	0.110621204	0.00988414	1883	3	509
NM_018090	-0.3648539	-1.2694413	-0.02351254	0.001841262	1514	3	725
NM_018090	-0.2199818	-1.1243734	-0.02351254	0.005494605	1831	3	609

NM_001350	-0.2447434	-0.8524039	0.014327605	0.002181033	1948	3	300
NM_001350	-0.2570948	-0.7643751	0.014327605	0.002722315	1949	3	386
NM_004637	-0.1685976	-0.5247205	0.152462561	0.042382295	2136	3	625
NM_000051	0.03003124	-0.8646461	0.248817951	0.009899828	1787	3	1110
NM_000051	-0.4358107	-1.0764929	0.248817951	0.01304289	1520	3	496
NM_016049	-0.2846892	-0.7747023	0.445222415	0.010879434	2575	3	303
NM_016049	-0.1250821	-0.8772463	0.445222415	0.015557163	2011	3	190
NM_020962	-0.938913	-1.0202118	0.124748849	0.017688454	25	3	1664
NM_057089	0.25267762	-1.9734392	0.14208512	5.00E-06	1593	3	1530
NM_057089	-0.0070516	-1.9127458	0.14208512	1.96E-05	1594	3	1446
NM_199360	-0.5293768	-1.1101608	0.334026902	0.004479198	3613	3	209
NM_199360	-0.5354675	-1.2747297	0.334026902	0.012398187	1518	3	827
NM_032377	-0.4784883	-0.7834619	0.465259461	0.023152029	1891	3	590
NM_032377	-0.3589454	-0.7937436	0.465259461	0.008130928	2568	3	128
NM_173174	-0.3342522	-1.2704067	-0.099364507	0.023392224	1490	3	1211
NM_173174	-0.3758512	-1.1420643	-0.099364507	0.047457423	1495	3	1147
NM_023007	-1.0229838	-1.3054428	0.593509024	0.019314577	1373	3	1536
NM_023007	-0.088421	-1.1575685	0.593509024	0.013297193	4610	3	742
NM_003960	0.21136523	-0.8570284	-0.185390366	0.049198841	1839	3	1288
NM_145052	-0.6054111	-0.9963789	0.219081836	0.020972381	1785	3	937
XR_011434	-0.1519467	-0.7017767	0.243228594	0.017831114	1913	3	437
XR_011434	0.07964733	-0.6278547	0.243228594	0.005408032	2184	3	521
BC078669	0.54321578	-1.8514833	0.086873428	0.000327929	1600	3	1566
BC078669	0.38440175	-1.6345533	0.086873428	0.000276095	1599	3	1430
NM_002444	0.11285125	-0.6453554	-0.076770293	0.03851426	1848	3	1055
NM_178867	0.06652887	-1.5059511	0.086593649	0.002436255	1608	3	1122
NM_178867	0.0408154	-1.5781823	0.086593649	0.001901126	1598	3	1180
NM_178867	0.10181119	-1.09305	0.086593649	0.000370607	2016	3	579
NM_178867	0.00316461	-1.383904	0.086593649	0.008126944	1610	3	952
CN801616	-0.1798319	-0.883427	0.180815591	0.0035464	1992	3	141
NM_015099	-1.1611528	-1.3549508	-0.198098727	0.006709873	185	3	1660
NM_015099	-0.8779635	-0.7640708	-0.198098727	0.028523724	1455	3	1354
NM_175738	-0.1589528	-1.1432778	0.337900583	0.004002254	2283	3	722
NM_175738	-0.2248845	-1.0313599	0.337900583	0.005187286	2324	3	92
NM_175738	0.14981086	-0.9116869	0.337900583	0.013602413	2028	3	565
NM_175738	0.03030351	-0.7862166	0.337900583	0.011916333	2022	3	577
NM_016360	-0.0248418	-0.975969	0.23864219	0.011656635	2245	3	414
CN642941	-0.3870358	-0.6740064	0.2327668	0.01480279	1969	3	299
CN642941	-0.3775983	-0.6533118	0.2327668	0.012657651	1970	3	320
NM_175061	-0.5294392	-0.6474817	0.391333333	0.041745094	2460	3	1021
NM_001646	-0.8885818	-1.3737815	0.348420829	0.005475025	1428	3	1226
NM_001646	-0.5030905	-1.6851727	0.348420829	0.006780804	1468	3	1287
NM_001039690	-0.0510224	-1.0874935	0.027675907	0.006177192	2243	3	528

NM_001039690	-0.0754937	-0.8169791	0.027675907	0.03338856	1843	3	578
NM_020385	-0.990017	-0.3266086	0.520107872	0.047010595	2492	3	1445
NM_005339	-0.7183668	-1.1330713	0.512067687	0.015993709	190	3	1625
NM_004187	-0.743106	-0.6943278	0.192502336	0.034473107	1902	3	1026
NM_019023	-0.3549348	-0.6147512	0.029111773	0.035196454	1982	3	704
NM_003689	0.20737827	-1.2772685	0.233154793	0.00060486	1612	3	702
NM_003689	0.0791123	-1.401909	0.233154793	0.003179743	1611	3	783
NM_013443	-0.3749102	-0.5954377	0.48110733	0.009181194	2574	3	333
NM_003926	-0.1011265	-0.9020964	0.166301886	0.000246095	2000	3	83
NM_003926	-0.2008027	-0.9505702	0.166301886	0.000733126	1995	3	54
NM_004779	-0.4636113	-0.8231133	-0.035025776	0.031813407	1791	3	1520
NM_004779	-0.3251106	-0.8847826	-0.035025776	0.041802895	1832	3	650
NM_006729	-0.0482797	-0.6372219	0.05467892	0.000239273	2143	3	659
NM_006729	0.39798508	-0.5666123	0.05467892	0.027625201	2230	3	1312
NM_016839	-0.3466593	-0.532193	0.075579789	0.026516379	1983	3	875
NM_002085	-0.5961911	-1.2739929	0.149373178	0.000123951	1526	3	427
NM_002085	-0.5538068	-1.194077	0.149373178	2.60E-05	1527	3	241
NM_152244	-0.0968755	-0.6599875	0.221389586	0.002771198	2183	3	216
NM_152244	-0.2706498	-0.2813108	0.221389586	0.027428595	3086	3	943
NM_175622	0.07393706	-1.5506337	0.476758059	0.011230622	1634	3	1291
NM_152374	-0.9898916	-0.6137828	0.313910144	0.017268343	1399	3	1505
NM_152374	-0.275943	-0.802861	0.313910144	0.005573945	2466	3	102
NM_017825	0.22337999	-0.218039	0.069163732	0.044799643	2241	3	1387
NM_001571	-0.6726377	-0.8394819	0.210723793	0.017827398	1904	3	670
NM_007203	-0.0601179	-0.6692088	0.144628319	0.036881961	2078	3	672
NM_024681	-0.1185371	-0.843809	0.076931547	0.001012374	1986	3	371
NM_024681	-0.0808263	-0.9146572	0.076931547	0.000982486	1842	3	236
NM_023018	-0.5620959	-1.1199805	0.163907289	0.000960574	1532	3	202
NM_023018	-0.7832516	-1.0976965	0.163907289	0.016246389	1554	3	935
NM_015045	-0.397005	-0.6720149	-0.157413019	0.021905223	1461	3	1176
NM_015045	-0.2748665	-0.7184432	-0.157413019	0.028616454	1933	3	1115
NM_016023	-0.7858207	-0.5156193	0.201881166	0.001705954	1880	3	927
NM_004730	0.3280559	-0.6749987	0.27850143	0.024699679	2232	3	1013
NM_180976	-0.0192035	-0.6052453	0.045113783	0.004360222	1872	3	615
NM_180976	-0.3273746	-0.3045772	0.045113783	0.018179917	2499	3	1049
NM_033386	-0.2247297	-0.8511293	0.147351541	0.000162991	1998	3	67
NM_033386	-0.2019347	-0.6658815	0.147351541	0.001053134	1979	3	473
NM_006311	-1.1568595	-0.6100776	0.214190521	0.003245311	1398	3	1516
NM_006311	-0.2461988	-0.4041402	0.214190521	0.041696277	2477	3	1068
NM_152260	0.04124727	-1.5530135	0.294649218	0.000674105	1609	3	890
NM_152260	-0.0397553	-1.21915	0.294649218	0.000286371	1615	3	490
NM_006703	0.29041293	-1.1956188	0.619286706	0.015713215	4612	3	1256
NM_006703	0.51290111	-1.0886017	0.619286706	0.014742899	4613	3	1381

NM_007371	-1.0373256	-1.8792187	0.026472727	0.046674016	89	3	1617
NM_007371	-0.9538857	-0.7734878	0.026472727	0.022373846	1456	3	1266
NM_170744	-0.5381046	-1.4168691	0.640308809	0.027184769	3886	3	1382
NM_178422	0.6349877	-0.4516102	0.077106168	0.000102217	2239	3	1510
NM_004350	0.40133572	-0.754086	0.431830829	0.005851352	2036	3	989
NM_004350	0.15768734	-0.8294928	0.431830829	0.010587708	2034	3	531
NM_032730	-0.0703738	-0.7964924	0.643606238	0.032017806	3906	3	765
NM_001487	-0.1690451	-0.763122	0.26117766	0.004707411	2005	3	88
NM_018056	-0.9176506	-1.0088267	-0.166560384	0.031642332	1426	3	1511
NM_014225	-0.3005385	-1.1147868	0.643249717	0.016424945	2280	3	756
NM_000546	-0.0453544	-1.860096	-0.332837498	0.010140548	141	3	1570
NM_013365	-0.6959389	-0.9001745	0.00139996	0.002681251	1550	3	690
NM_013365	-0.5663305	-0.8661976	0.00139996	0.012442176	1552	3	559
NM_004935	-0.0638461	-0.3738448	0.1411594	0.00303858	2158	3	881
NM_058169	-0.3867111	-1.5520042	0.801026132	0.010154582	4601	3	1529
NM_058169	-0.0146892	-1.5172783	0.801026132	0.020269885	1102	3	1500
NM_199044	0.026683	-0.508032	0.000604308	0.008474971	1868	3	1035
NM_016491	0.20696831	-0.768469	0.301373534	0.004668471	2352	3	682
XR_010584	-0.4278586	-0.5258859	0.102891279	0.001367545	1939	3	653
XR_010584	-0.5549369	-0.2799268	0.102891279	0.006702117	2497	3	1131
NM_000074	0.52062295	-1.4774504	0.120549144	0.002291769	1602	3	1478
NM_007074	-0.0364131	-0.3925805	0.080510291	0.010299057	2157	3	982
NM_000344	0.28830683	-1.039603	0.118681997	0.024220276	2253	3	1227
NM_001242	-0.1628037	-1.2853125	-0.104831985	0.001338432	1482	3	957
NM_001242	-0.2080619	-0.862101	-0.104831985	0.024259948	1940	3	744
NM_031450	0.1579628	-0.7906362	-0.001046061	0.006797578	1860	3	897
NM_212535	-0.3452745	-1.2179839	0.597655961	0.003397189	3651	3	222
NM_212535	-1.0833416	-0.7988941	0.597655961	0.010606565	1381	3	1598
NM_012478	-0.815694	-1.3406866	-0.107189189	0.00107863	1512	3	1299
NM_012478	-0.9732665	-1.5452233	-0.107189189	0.004808762	1505	3	1501
NM_020935	0.25494002	-1.0663287	0.095167212	0.004589092	1606	3	911
NM_020935	0.07137926	-0.8705608	0.095167212	0.004338399	2017	3	677
NM_032932	0.09590028	-0.8500602	0.508575597	0.010460434	2353	3	470
NM_032932	-1.670208	-0.4846616	0.508575597	0.037050876	27	3	1695
NM_000626	0.50825465	-1.1262819	-0.067089324	0.036599791	1607	3	1481
NM_000626	0.07982395	-1.2958385	-0.067089324	0.024714542	1604	3	1292
NM_145715	-1.3735414	-0.2982676	0.143735354	0.00386162	1794	3	1404
NM_005018	-0.5399111	-0.4403557	0.203115436	0.004118851	1980	3	941
NM_014601	-0.8719413	-1.1825438	0.569237615	0.008021218	1443	3	1106
NM_014601	-0.9194232	-0.8417718	0.569237615	0.005551749	2409	3	930
NM_015871	0.24458861	-0.811105	0.332791288	0.000831747	2029	3	543
NM_015871	0.23148843	-0.9990208	0.332791288	0.000913236	2027	3	401
NM_022377	-0.1379292	-0.8232031	0.311896495	0.019359637	2354	3	1136

NM_022377	-0.6679311	-1.5035327	0.311896495	0.005302404	1384	3	1342
NM_022377	-0.3765054	-0.696929	0.311896495	0.020071255	2467	3	466
NM_022377	-0.9938666	-0.8357145	0.311896495	0.006299937	1466	3	1254
NM_015921	-0.2146279	-0.5336489	0.246115708	0.005145404	3079	3	412
NM_015921	-0.0645255	-0.3417522	0.246115708	0.010659324	2173	3	889
NM_138422	-0.387126	-1.0740945	0.139261789	0.042234696	1896	3	975
NM_002461	-0.3082379	-0.5003418	0.007405256	0.032815412	2134	3	766
NM_015655	-0.6925602	-1.5286797	-0.315509368	0.012267527	1500	3	1504
NM_015655	-0.3658601	-1.3787799	-0.315509368	0.022834164	1481	3	1410
XR_011186	-0.0426749	-1.0648845	0.215104938	0.003355979	1991	3	173
NM_006521	-0.3691045	-0.6037332	0.31109253	0.02923221	2462	3	428
NM_033518	-0.0858993	-1.0063688	0.32435814	0.000372277	1997	3	16
NM_033518	-0.0968962	-1.009756	0.32435814	0.000544121	1996	3	19
NM_199183	-0.1934822	-0.1169915	0.168795974	0.009928415	3095	3	1274
NM_005188	-0.3267593	-1.2216979	0.974684262	0.0175554	4602	3	1450
NM_032822	-0.0269838	-0.7839073	0.784126524	0.016044953	3908	3	852
NM_032822	0.0847296	-0.8805046	0.784126524	0.023686673	3909	3	1158
XR_013427	-0.4135863	-0.7267075	0.322355833	0.012298947	1968	3	231
XR_013427	-0.5559862	-0.5322904	0.322355833	0.014508506	1971	3	557
CB554865	-0.5091454	-0.7254662	0.702754717	0.038817353	2452	3	912
NM_018091	-0.1460294	-0.9539327	0.270504515	0.001116851	2009	3	175
NM_018091	-0.1656189	-1.000302	0.270504515	0.005834919	2244	3	461
NM_080794	-0.593785	-0.6150597	0.556684713	0.020839522	1714	3	1037
NM_002596	0.28918472	-1.9531639	-0.121348637	0.015959357	1638	3	1601
NM_004095	0.46679246	-0.7900894	0.502240713	0.020457154	2404	3	1311
NM_020186	0.12678515	-0.6011441	0.422807803	0.018604372	2395	3	666
XR_010951	-0.2851505	-0.2882354	0.260528419	0.001325117	3082	3	826
XR_010951	-0.3074871	-0.2925852	0.260528419	0.001190658	3081	3	813
NM_000733	-0.2413624	-1.5090164	0.146217757	0.003397997	1531	3	928
NM_000733	-0.5959889	-1.3868948	0.146217757	0.00294495	1385	3	1250
NM_080391	-0.1272786	-1.0604861	0.183747514	0.001825356	1990	3	153
NM_080391	-0.3260334	-0.8467329	0.183747514	0.000935381	1993	3	38
NM_015722	0.07694418	-0.9725048	0.320928259	0.010290006	2351	3	540
NM_001322	0.22176551	-0.4137318	0.221290759	0.049936266	2182	3	1222
NM_018188	-0.5479962	-0.7297376	0.035503921	0.006883575	1879	3	893
NM_018188	-0.2336181	-0.5326102	0.035503921	0.009179925	1966	3	656
AB037781	-0.3616766	-0.412206	0.53022362	0.032398329	2463	3	768
NM_006705	0.15432806	-0.5615977	0.077902888	0.01605346	2226	3	939
NM_172230	-1.7278448	-1.8337688	0.128878903	0.036171268	33	3	1691
NM_012445	-0.3652967	-1.0915584	0.143753436	0.001660408	1566	3	265
NM_012445	0.18691589	-1.0783539	0.143753436	0.006008628	2257	3	681
NM_020832	0.50920976	-0.9359498	0.118322755	2.86E-05	1633	3	1204
NM_020832	0.16828013	-0.5995272	0.118322755	0.002199205	2180	3	838

NM_133336	0.07609305	-0.5696581	0.308516782	0.000667701	2185	3	469
NM_133336	0.14050314	-0.553255	0.308516782	0.007106813	2213	3	720
NM_148179	-0.0167523	-0.6981235	0.140121294	0.007534635	1844	3	342
NM_148179	-0.0628046	-0.5116555	0.140121294	0.049319611	2137	3	716
NM_032520	-0.8791019	-0.8154466	0.653836324	0.023608482	1709	3	1155
NM_032520	-1.7246716	-0.5557116	0.653836324	0.042515104	160	3	1651
NM_183239	0.10614394	-1.9337353	0.314182271	0.035015103	1663	3	1611
NM_022157	0.5503877	-0.6174881	0.108535886	0.038942352	2233	3	1414
NM_022157	0.27946928	-0.5209811	0.108535886	0.030275718	2228	3	1169
XM_495889	-0.153309	-0.4310359	0.243404105	0.000561589	2166	3	505
NM_030914	0.09216159	-0.432906	0.137523192	0.0007276	2165	3	909
NM_030914	0.08185573	-0.5170413	0.137523192	0.002135567	2164	3	733
NM_138431	-0.0501648	-0.4491793	-0.004481143	0.010080634	2154	3	983
NM_017520	-0.3146326	-0.3004378	0.381498976	0.016402353	3088	3	858
NM_003111	-1.4120143	-0.4763854	0.344271002	0.015650423	158	3	1639
NM_003111	-0.1459159	-0.7589602	0.344271002	0.038064093	2458	3	1195
NM_000884	0.57938102	-1.0632862	0.481460363	0.002916543	2403	3	1301
NM_000884	-1.4466532	-0.7309789	0.481460363	0.0016897	218	3	1649
NM_006816	-0.2272026	-0.2546724	0.20716964	0.012629013	3087	3	1016
NM_013354	-0.236858	-0.392745	0.136205059	0.020975229	1795	3	1126
NM_013333	-0.4782044	-1.4090237	0.191536025	0.000763272	1528	3	595
NM_013333	-0.3342891	-1.3480405	0.191536025	0.000192143	1529	3	281
NM_003334	-0.5407942	-0.9962483	0.249957593	0.006843717	1906	3	316
NM_003334	-0.4323319	-0.5287448	0.249957593	0.044671838	1972	3	694
NM_016643	0.53251344	-1.6736265	0.317837999	0.041101388	147	3	1633
NM_004160	0.09600799	-1.7213683	0.562744514	0.007375673	1353	3	1470
NM_004160	0.18591437	-1.4536291	0.562744514	0.013425058	1354	3	1337
NM_007097	-0.6418146	-1.004573	0.401014484	0.016691543	1890	3	833
NM_203351	-0.628318	-0.4841414	-0.014985664	0.011198939	2495	3	1028
XM_379968	-0.1244414	-0.8310846	0.229882285	1.10E-05	1999	3	29
XM_379968	-0.0035145	-0.4214221	0.229882285	0.00489563	2163	3	700
NM_145214	0.08928394	-0.5140138	0.200860908	0.001955023	2174	3	735
NM_170606	-1.456566	-0.5336615	0.017166002	0.04173516	1451	3	1602
NM_032339	-0.9746116	-1.4717606	0.744138236	0.031589428	208	3	1631
NM_032339	-0.3886467	-1.3458947	0.744138236	0.010622486	3916	3	1056
XR_013052	-0.5829979	-1.0801501	0.792059275	0.017070387	3889	3	1050
NM_016256	-0.5397827	-0.7119926	0.743085136	0.019282711	2453	3	737
NM_016256	-0.642457	-0.5466114	0.743085136	0.021115842	2454	3	980
NM_002022	-0.137436	-0.3152056	0.21565727	0.011187675	3085	3	907
NM_006412	0.19141368	-1.7535325	0.19558479	0.006791647	189	3	1594
NM_006412	-0.3359096	-0.5344189	0.19558479	0.020892123	1977	3	706
NM_015691	-2.076559	-1.0822956	0.288440664	0.012970238	35	3	1677
NM_015691	-1.0765665	-1.1056246	0.288440664	0.037416145	1664	3	1405

NM_012204	0.45054704	-0.6005252	0.30167442	0.000317817	3055	3	1395
NM_012204	0.43372907	-0.5752499	0.30167442	0.001506511	2274	3	1319
NM_018645	-0.4103134	-0.7024348	0.486639761	0.009495174	1412	3	1495
NM_153367	-0.0183766	-0.4385561	0.294957774	0.023528956	2211	3	775
NM_152267	-0.2549829	-0.4375839	0.109348756	0.029983544	1978	3	892
NM_152267	-0.1803288	-0.4762143	0.109348756	0.00023355	2156	3	622
XM_370738	-0.5903508	-1.242043	-0.022927553	0.00148216	1523	3	778
XM_370738	-0.5560799	-1.3372808	-0.022927553	0.006156329	1522	3	990
NM_004749	-0.2363081	-0.5464086	0.022890076	0.017126714	2147	3	849
NM_004749	0.12937635	-0.3580029	0.022890076	0.048066844	2161	3	1228
NM_018166	0.22080115	-1.3526195	0.223420595	2.25E-05	1616	3	971
NM_018166	0.02161062	-1.1749958	0.223420595	0.000132433	1617	3	204
NM_024881	0	-1.3595413	0.633535772	0.001673478	3633	3	572
NM_015983	-0.0426734	-0.7315094	0.266855896	0.000383134	2003	3	114
NM_015983	-0.6972476	-0.0553813	0.266855896	0.039875634	2489	3	1460
NM_003731	-0.1477965	-0.3124003	0.033171063	0.042142601	2155	3	1096
AK124768	-0.1565772	-0.4108405	0.330902172	0.001662153	2167	3	553
NM_004068	-0.4989686	-1.0190784	0.306591942	0.007154539	1907	3	264
NM_004068	-0.3376311	-0.839157	0.306591942	0.003463989	1917	3	65
NM_024328	-0.6301047	-0.8008669	0.621753635	0.020801677	4604	3	1040
CN646224	0.26738203	-0.2993631	0.056185317	0.034404407	2240	3	1344
NM_014172	-0.3052125	-0.2971941	0.236423466	0.016509182	3084	3	920
NM_030662	-0.4352623	-0.6865771	0.216010154	0.002311086	1959	3	181
NM_030662	-0.4738231	-0.5409944	0.216010154	0.001943771	1960	3	382
NM_005632	-0.2908543	-0.5564695	0.203013968	0.009967604	1963	3	404
NM_005632	-0.2879274	-0.4028033	0.203013968	0.048177604	1964	3	825
NM_001003703	-0.3272113	-0.6176267	0.281500173	0.022093713	1984	3	420
XR_012618	0.00879725	-0.3740438	0.176745576	0.014153039	2150	3	1095
NM_015957	-0.3799199	-0.4554539	0.342149061	0.037434189	2430	3	750
NM_024570	-0.3882016	-0.7759693	0.215061715	0.024300753	1792	3	1022
NM_032636	0.05564644	-0.9403671	0.83824797	0.004874824	2933	3	630
NM_004937	-0.489174	-0.9539096	-0.035954591	0.009432529	1828	3	584
NM_004937	-0.5780261	-0.696719	-0.035954591	0.021765281	1935	3	923
NM_006254	-1.064983	-0.7782811	0.803238256	0.00930124	219	3	1632
NM_014077	-0.36899	-1.4449656	0.226561741	0.013616034	1547	3	946
NM_014077	-0.529334	-1.023177	0.226561741	0.010949383	1557	3	292
NM_003650	-0.3014903	-1.0437547	0.247628418	0.000601211	1914	3	52
NM_003650	-0.2196509	-0.7695611	0.247628418	0.001340387	1919	3	113
NM_198334	0.12736557	-0.8134957	0.129007599	0.001727767	2020	3	457
NM_198334	0.13449705	-0.816991	0.129007599	0.001772401	2021	3	532
XR_012642	-0.5986462	-1.8846849	0.465102099	0.000480725	348	3	1391
NM_005745	-0.0744649	-0.8191899	0.307439131	0.000298124	2002	3	35
NM_005745	-0.0910754	-0.8887257	0.307439131	0.00068857	2001	3	18

NM_000487	-0.1300093	-0.8545393	0.409778678	0.005074433	1885	3	119
NM_000487	0.13580686	-0.700663	0.409778678	0.004587238	2188	3	379
NM_152348	-0.1584461	-0.9074758	0.402266424	0.000801971	2010	3	22
NM_152348	-0.3638086	-0.788753	0.402266424	0.000778016	2569	3	23
CN641959	-2.4334213	-1.0764198	0.799718398	0.009607277	1418	3	1583
NM_203495	-0.0821631	-0.9084035	0.389358241	0.007225835	2249	3	98
NM_203495	0.02962653	-0.7502645	0.389358241	0.010530584	2117	3	285
NM_080821	-0.1068099	-0.5850509	0.472245535	0.028006973	2976	3	450
NM_017823	-0.0400238	-0.9402551	0.274303917	0.0004362	1988	3	108
NM_017823	0.08257359	-0.911887	0.274303917	0.002028403	1989	3	237
NM_198319	0.39969104	-0.9247395	0.367725702	0.031265022	2319	3	1376
NM_198319	0.04865372	-0.8525419	0.367725702	0.022555684	2350	3	575
A_01_P016008	-0.3769955	-1.76682	0.595031846	0.001112661	1584	3	1296
NM_144609	-1.4332324	-1.5259021	0.148738903	0.008063429	91	3	1600
NM_144609	0.39624507	-1.0869216	0.148738903	0.008280592	1631	3	1174
NM_032310	-0.1287128	-0.9479913	0.105948684	0.000891509	1985	3	253
NM_013299	0.03573213	-1.1372515	0.270201445	0.001045787	1623	3	229
NM_013299	-0.3362249	-0.903888	0.270201445	0.002823759	1895	3	71
NM_015106	-0.1846642	-0.9293585	0.453200944	0.020020949	2252	3	391
NM_015106	-0.2682322	-0.6619783	0.453200944	0.001321936	2570	3	43
NM_002268	0.22497452	-0.6316301	0.237894596	0.034304371	2224	3	1031
NM_181708	-0.2158076	-0.5771091	0.572225785	0.00360923	2688	3	661
NM_003501	0.01367656	-1.0945328	-0.090463683	0.007605735	1837	3	891
NM_001284	-0.2394352	-0.7346017	0.300997187	0.005424925	2086	3	59
NM_001284	0.1280098	-0.7760226	0.300997187	0.009443544	1887	3	651
NM_024295	-0.0455894	-0.782035	0.776220694	0.013256592	3907	3	663
NM_032344	-0.0353357	-0.5200806	0.003471128	0.048621755	2149	3	1067
NM_024122	0.40095169	-1.0582729	0.735197629	0.002104313	4081	3	1064
A_01_P017143	-0.4689096	-0.8047247	0.502826708	0.003963302	2566	3	186
NM_018250	-0.0767505	-0.609227	0.042841247	0.027390158	2144	3	803
NM_002014	-0.4567648	-0.8352709	0.254082125	0.006351294	1909	3	160
NM_002014	-0.5368052	-1.040633	0.254082125	0.003019893	1894	3	157
NM_002028	0.01764039	-0.9232778	-0.027514758	0.011413339	1875	3	1008
NM_181484	0.08870871	-0.5855865	0.26607591	0.022144918	2189	3	686
NM_181484	-0.1240126	-0.6744645	0.26607591	0.020730633	2037	3	311
NM_181484	0.09184095	-0.4376673	0.26607591	0.043117878	2190	3	959
NM_032178	0.22817655	-1.0543605	0.436389079	0.002955022	3763	3	484
NM_032178	-0.2322202	-1.1090949	0.436389079	0.013602628	3890	3	411
NM_024662	-0.237032	-0.3794397	0.346783305	0.017055236	2141	3	633
NM_016272	-0.705295	-1.0920527	0.166570278	0.033859786	1555	3	932
CN646732	-0.6497165	-0.1846208	0.255894164	0.02237403	2502	3	1262
CN646732	-0.62765	-0.0450654	0.255894164	0.026446234	2503	3	1386
NM_024061	-0.382318	-0.8924759	0.25085244	0.01486821	1390	3	1238

NM_005610	-0.0252693	-0.6205581	0.499367711	0.006001991	2118	3	276
NM_006761	-1.1922006	-1.1948634	0.357575003	0.000387298	1441	3	1321
NM_006761	-1.0843826	-0.7906881	0.357575003	0.000562232	1465	3	1132
NM_012399	-0.6706882	-0.5207656	0.27598693	0.00206689	2424	3	646
NM_012399	-0.0498054	-0.7814935	0.27598693	0.046153823	2042	3	555
NM_021204	-0.5078698	-0.6331957	0.510140679	0.004456047	1712	3	1421
CN641640	-0.8994254	-0.9058655	0.577678509	0.009104074	2434	3	870
CN641640	-0.9297489	-0.722463	0.577678509	0.011396653	2436	3	997
NM_000516	-0.3802825	-0.1632633	0.205474878	0.04538527	2505	3	1177
NM_005529	0.00339698	-0.3376242	0.25451383	0.033725867	2179	3	1129
NM_005702	-0.3851466	-0.9825307	0.131407944	0.033958434	1892	3	717
NM_152609	-0.3178068	-0.6740701	0.737524801	0.042754782	2461	3	1088
NM_001383	-0.7482404	-1.1435938	0.565122207	0.016532994	1431	3	967
NM_001383	-1.0965644	-0.5578758	0.565122207	0.040048915	1704	3	1526
NM_001383	-0.6083644	-0.9310119	0.565122207	0.045879408	3885	3	1048
NM_006555	-0.2413672	-1.2634429	0.367059507	6.59E-05	1536	3	40
NM_006555	-0.2776917	-1.1117118	0.367059507	0.00021876	1537	3	6
NM_032366	-0.0395645	-0.5016175	0.485182894	0.006038662	2977	3	426
NM_021260	-0.4357625	-1.1770793	0.739425196	0.004674943	3632	3	508
NM_021260	-0.7745627	-1.2387421	0.739425196	0.001171049	1419	3	1157
NM_020892	0.16838703	-0.532452	0.105854791	0.024819644	2227	3	1034
NM_032334	-0.4012352	-0.5632704	0.121710112	0.028220399	1938	3	599
XM_371010	-0.5005411	-0.7268157	0.32674258	0.037853577	1899	3	634
NM_207121	0.10731559	-0.7459123	0.407464624	0.017877923	2392	3	645
NM_207121	0.08818532	-1.067955	0.407464624	0.001482388	1624	3	203
NM_030930	-0.0626088	-1.002603	0.249103039	0.032102261	2347	3	713
NM_030930	0.03475898	-0.8213266	0.249103039	0.009438999	2349	3	362
XR_011099	-0.620294	-1.1466698	0.532396112	0.014736955	2277	3	808
XR_011099	-0.544248	-1.0656573	0.532396112	0.015769497	2278	3	767
XR_011099	-0.8336672	-1.0671898	0.532396112	0.001881426	1444	3	643
XR_011099	-0.8034603	-1.0882032	0.532396112	0.000797475	3615	3	424
XR_011099	-0.4821985	-0.5406726	0.532396112	0.007271327	2602	3	393
XR_011099	-0.4094665	-0.7277544	0.532396112	0.001736379	2571	3	37
NM_152649	-1.3548539	-0.6126071	0.750179491	0.032726387	4733	3	1567
NM_004154	0.33457526	-0.603084	0.256798292	0.007730496	2181	3	657
NM_004154	0.22837477	-0.5278544	0.256798292	0.027581049	3329	3	1044
NM_025203	0.2156943	-0.8483486	0.453620146	0.001645906	2031	3	395
NM_025203	-0.3786378	-0.6215325	0.453620146	0.003596552	1797	3	814
NM_014268	0.10341273	-0.5341617	0.30108509	0.013949552	2212	3	869
NM_014268	0.01841492	-0.4002298	0.30108509	0.002454564	2168	3	770
NM_130782	-2.549047	-0.1978277	0.978890564	0.048347769	170	3	1675
NM_138773	-0.3846764	-0.8434933	0.797478904	0.033969176	3901	3	985
XR_011614	0.00383401	-0.5837083	0.452208537	0.029034871	2690	3	693

XR_011614	0.04988373	-0.8008644	0.452208537	0.002317903	2895	3	287
NM_003333	-0.0556941	-0.400941	0.246839766	0.000133876	2169	3	712
NM_003860	-0.5628887	-0.9927321	0.287982109	0.006527522	1905	3	262
NM_003860	-0.6892246	-0.6780283	0.287982109	0.01284495	1903	3	660
NR_001444	0.1734773	-0.7314959	0.037289916	0.019173728	1876	3	1225
NM_014235	0.25458199	-1.8626818	0.307467715	0.035225196	144	3	1656
NM_014403	0.08760658	-0.5432371	0.319746375	0.000833299	2102	3	516
NM_012426	-0.1905723	-0.5520019	0.248542257	0.00652203	2139	3	464
NM_012426	0.11381426	-0.4566731	0.248542257	0.006007183	2223	3	1124
NM_001004105	0.85156966	-0.7698231	0.221567414	0.047316648	10873	3	1630
NM_002947	-0.8023235	-1.197937	1.069439379	0.009806115	1417	3	1562
NM_031219	-0.0047086	-1.0100772	0.403363409	0.004082719	2325	3	261
NM_031219	-0.6089473	-0.3772921	0.403363409	0.017264366	2488	3	1208
NM_003073	-0.2674495	-1.2658037	0.260408104	0.002982757	1567	3	448
NM_003073	0.03925246	-0.8654579	0.260408104	0.013170032	2043	3	363
XM_376350	-0.6890635	-1.2695696	0.980743056	0.008694082	206	3	1569
NM_194259	0.03217504	-0.8370903	0.436932765	0.000290438	2186	3	55
NM_194259	-0.0344328	-0.7533484	0.436932765	0.001141619	2187	3	72
XM_043118	-0.2562629	-0.353884	0.375369196	0.038888943	1889	3	1193
NM_016399	-0.8693456	-1.0539633	0.7572921	0.002951967	1705	3	1374
NM_024104	0.23009172	-0.5863367	0.273767709	0.026798839	2225	3	1018
XM_059972	-0.2800583	-1.2701177	0.503067639	0.002690948	3631	3	319
XM_059972	-0.3894855	-0.785068	0.503067639	0.001974761	2572	3	34
NM_018641	-0.2468898	-0.6601429	0.582368576	0.022280559	2643	3	538
NM_030571	-0.7166785	-0.913596	0.515988609	0.005409622	3616	3	348
NM_030571	-0.4339465	-1.0245592	0.515988609	0.014739099	2440	3	456
NM_004870	0.13098963	-0.8867636	0.418224509	0.000409336	2112	3	193
NM_004870	0.1105014	-0.7861487	0.418224509	0.000807496	2113	3	207
NM_024784	-0.0539272	-1.3444056	0.264152222	0.000748565	1591	3	537
NM_024784	-0.8970487	-1.1580525	0.264152222	0.000577709	1429	3	956
NM_002412	-0.3153721	-0.5184491	0.389903556	0.000375703	3080	3	249
NM_002412	-0.0317696	-0.4183765	0.389903556	0.019284811	2121	3	818
NM_025238	-0.378761	-1.1903919	0.424426841	0.000719728	1535	3	57
NM_025238	-0.1878486	-0.9315689	0.424426841	0.008485439	2248	3	95
NM_032840	-0.0278465	-1.0174154	0.27560011	5.34E-06	2335	3	42
NM_032840	-0.2072466	-0.9588838	0.27560011	9.98E-05	1994	3	9
NM_173608	-0.1590605	-1.4303293	0.397373995	0.010199754	2282	3	978
NM_173608	-0.4638871	-1.239064	0.397373995	0.006397296	2276	3	603
NM_022078	0.01461856	-1.4260525	0.21822996	8.77E-05	1613	3	564
NM_022078	-0.0657249	-1.6016999	0.21822996	0.000935257	1576	3	1045
NM_001064	0.13003218	-0.8459429	0.298958642	0.013197777	2083	3	421
NM_001064	0.06540886	-0.6628505	0.298958642	0.036604735	2085	3	587
NM_004360	0.08121861	-0.4327367	0.238993987	0.038373743	2220	3	1006

NM_005439	-0.1511999	-0.5777744	0.131071689	0.018580789	2148	3	662
NM_005439	-0.2830459	-0.494235	0.131071689	0.02889236	1962	3	703
NM_002817	0.25755344	-0.4540307	0.324185448	0.000283836	2201	3	954
NM_002817	0.18186955	-0.5706963	0.324185448	7.58E-05	2200	3	596
NM_019056	0.06167533	-0.3885974	0.304094426	0.000447818	2170	3	798
NM_019107	0.21931715	-1.4031227	0.210878108	0.00067135	1621	3	953
NM_019107	0.10282766	-1.1756697	0.210878108	0.000502023	1622	3	372
NM_015986	-0.2821001	-1.1195426	0.403303607	0.002916236	1545	3	121
NM_017907	-0.344147	-1.6583578	0.52423944	0.001338066	1586	3	1029
NM_017907	-0.3835152	-1.5307294	0.52423944	0.001352792	1585	3	793
NM_000478	0.24633217	-1.2697176	0.873617187	0.003198846	4093	3	1213
NM_024029	-0.3466765	-1.060469	0.379932986	0.000817109	1544	3	79
NM_024029	-0.3022453	-1.0172811	0.379932986	0.000731586	1786	3	743
NM_001253	-1.6986796	-0.3326125	0.510911458	0.012553584	162	3	1663
NM_014169	-0.1316288	-0.7105248	0.631054017	0.004408264	2644	3	270
XR_013588	-0.8469015	-0.8935518	0.414873516	0.012569517	1432	3	791
XR_013588	-0.4799319	-0.4891651	0.414873516	0.029874919	2482	3	810
NM_020655	0	-0.5857797	0.430703414	0.016275008	2026	3	458
NM_015511	0.19980684	-1.1300912	0.218561518	0.000161773	1618	3	583
NM_015511	-0.0797328	-0.8358744	0.218561518	0.000562067	2018	3	143
XR_014545	-0.6238825	-0.6962399	0.164921401	0.019508251	1893	3	669
NM_016062	-0.2502208	-0.473575	0.253832709	0.019166102	2138	3	476
NM_013345	0.03559567	-1.1894547	0.533269669	0.0005468	2323	3	760
NM_013345	-0.2574216	-0.8899173	0.533269669	0.007589199	2577	3	164
NM_016407	-0.3388515	-0.5499078	0.203387376	0.002047713	1961	3	322
NM_016407	-0.3522528	-0.2940214	0.203387376	0.047831824	2501	3	979
NM_145752	-0.2690384	-0.4234846	0.421265512	0.016953506	1974	3	605
NM_006354	-0.3671847	-0.3161625	0.330290744	0.013657854	2507	3	855
NM_021134	-0.1457282	-0.7945041	0.350277162	0.000189304	2090	3	13
NM_021134	-0.0801143	-0.5910291	0.350277162	5.53E-05	2093	3	159
NM_024667	0.09189569	-0.8298397	0.272975167	0.001525314	2019	3	345
NM_024667	0.01844075	-0.4789374	0.272975167	0.019011473	2104	3	695
XR_012771	-0.0761116	-0.8071706	0.458786943	0.006528416	2025	3	161
XR_012771	-1.7318025	-0.4865071	0.458786943	0.022010213	26	3	1689
NM_032280	-0.4445696	-0.2467122	0.451535982	0.041540414	2514	3	1154
NM_024943	0.03396147	-0.3834537	0.316524271	0.026269852	2221	3	1042
A_01_P007547	0.4280425	-0.4152373	0.107732561	0.029199575	2242	3	1400
BU533041	-0.5388267	-1.114159	0.205761204	0.013529505	1556	3	407
BU533041	-0.6152435	-0.8619497	0.205761204	0.017614716	1553	3	396
NM_000932	-0.4677775	-0.9350657	0.557126671	0.032416574	187	3	1616
NM_000932	-0.2972488	-1.0677004	0.557126671	0.006874125	2284	3	453
NM_004145	-0.4488402	-0.5812902	0.18785969	0.00625229	1958	3	475
NM_004145	-0.3044641	-0.5646136	0.18785969	0.002936309	1967	3	306

NM_194326	-0.1103684	-0.6038866	0.380024273	9.66E-05	2094	3	120
NM_194326	-0.0912765	-0.6517288	0.380024273	0.001524558	2091	3	101
NM_033502	0.02389508	-1.463127	0.476973345	0.01750532	1691	3	1251
NM_003211	-0.0387553	-0.4767448	0.183082927	0.004651435	2162	3	613
NM_003211	-0.3853278	-0.9596174	0.183082927	0.04097779	1558	3	503
NM_052875	0.1742083	-0.7636186	0.587371484	0.000809188	2903	3	358
NM_052875	0.19243584	-0.6824859	0.587371484	0.001343242	2896	3	533
NM_030912	-0.4091683	-0.6129248	0.556307247	0.049553374	2431	3	773
NM_015150	0.06762363	-0.8482187	0.458609512	0.000251191	2030	3	136
NM_015150	0.26038506	-0.8593554	0.458609512	0.000803577	2032	3	524
NM_012105	-0.1617566	-0.5999433	0.349874709	0.000176157	2092	3	107
CK232088	-0.0187798	-0.8985102	0.25553417	0.012650634	2348	3	405
CK232088	-0.2103113	-0.7270198	0.25553417	0.001612171	1921	3	105
NM_176863	-1.0664712	-1.620875	0.406523685	0.033303705	1641	3	1588
NM_176863	-0.6723968	-1.4468772	0.406523685	0.037685992	1647	3	1475
NM_022833	-0.4986497	-0.5828931	0.141482386	0.004251044	1936	3	554
NM_022833	-0.6545579	-0.490805	0.141482386	0.036052257	2496	3	885
NM_006442	-0.3075793	-1.0409144	0.291990147	0.002711273	1915	3	68
NM_006442	-0.1533599	-0.9147552	0.291990147	0.003309527	1916	3	93
NM_032525	-0.0291508	-0.5645813	0.421916077	0.035254468	2119	3	612
NM_016029	-1.0282451	-1.1541808	0.875048033	0.025326009	177	3	1568
NM_001658	-0.1954536	-0.5585173	0.373131778	0.004315188	2095	3	176
NM_001658	-0.1768628	-0.5594621	0.373131778	0.009015764	2096	3	230
XR_012190	-0.5509964	-0.7961818	0.511040762	0.000695805	2567	3	53
XR_012190	-0.6736649	-0.7275013	0.511040762	0.000399919	2411	3	293
NM_018310	-0.3698021	-0.7119663	0.019249249	0.0178646	1934	3	654
NM_006355	-0.1390914	-1.6451392	0.442509768	0.001672686	1587	3	1130
NM_006355	-0.2751878	-1.3911466	0.442509768	0.002447449	1590	3	774
DR770061	-0.4631774	-1.1590909	0.816766636	0.005049378	3888	3	548
DR770061	-0.379959	-0.8578821	0.816766636	0.007947006	2755	3	394
XM_926382	-0.3022922	-0.6724903	0.334770681	0.016057354	2133	3	296
XM_926382	-0.0025658	-0.7816135	0.334770681	0.044313632	2079	3	639
NM_005873	0.03166094	-1.0143182	0.503505035	0.005171251	2333	3	314
NM_005873	-0.7027488	-1.4871822	0.503505035	0.000216297	3598	3	787
NM_005873	-0.6190669	-1.3338535	0.503505035	0.000632784	3599	3	403
NM_022473	-1.1340552	-0.5499485	0.136769306	0.034024322	212	3	1610
XM_926525	-0.5344716	-1.6862165	0.185740232	0.002485648	1515	3	1310
NM_002185	-0.3347696	-1.1135292	0.814427005	0.007079721	4102	3	747
NM_021978	-0.3179916	-1.2775942	0.600773166	0.000125192	3640	3	174
NM_021978	-0.3392189	-1.0937581	0.600773166	0.000213531	3652	3	27
NM_025139	0.05498916	-1.023201	0.598883805	0.00720467	2318	3	1098
NM_025139	0.27808736	-1.2401039	0.598883805	0.028994331	557	3	1355
NM_031918	0.21377592	-1.1317256	0.548795526	0.031164307	2360	3	1278

NM_004034	0.29832325	-0.5387018	0.314025259	0.019993194	2273	3	1398
XR_011395	-0.7621308	-0.9315177	0.133703019	0.013700933	1551	3	708
NM_016428	0.08322864	-1.0847639	0.281613714	0.039033309	1650	3	1091
NM_016580	0.45323155	-0.5812834	0.066163771	0.03270126	2229	3	1332
A_01_P018422	0.14814884	-0.9268846	0.491838331	0.003018897	2033	3	594
A_01_P018422	0.35433438	-1.0376204	0.491838331	0.007663785	3054	3	1121
NM_020365	-0.4436255	-0.2899321	0.48702958	0.016157875	2508	3	976
NM_012392	-0.3562548	-1.074513	0.174448283	0.007528679	1568	3	326
NM_012392	-0.1694411	-0.7489729	0.174448283	0.021348499	1884	3	460
NM_033161	-0.3951147	-1.5980633	0.632315557	0.000146913	349	3	1148
NM_024093	-0.1041247	-0.8078646	0.61959369	0.002535074	2638	3	148
NM_015535	-1.1168684	-1.1702333	0.571510074	0.008875686	1667	3	1397
NM_015535	-0.8164892	-0.9056814	0.571510074	0.014221755	2435	3	797
NM_001009566	0.00628682	-0.6144039	0.533097883	0.002589394	2640	3	444
NM_001498	-0.115832	-0.7707563	0.374406052	0.029118716	2039	3	472
NM_022047	-0.413286	-1.4838587	0.126355745	0.001505364	1530	3	846
NM_022047	-0.3868631	-1.2712607	0.126355745	0.001400904	1524	3	398
NM_014462	0.01424948	-1.1184697	0.651780749	0.004694568	3745	3	324
NM_021626	-0.3998224	-0.8370851	0.763943471	0.001599854	2581	3	180
NM_014012	-1.6202663	-0.7036014	1.104390799	0.00855203	169	3	1668
NM_145266	-0.4421177	-1.2128364	1.155338752	0.005985326	4103	3	1317
NM_014696	-0.7251137	-1.4524818	0.502410688	0.000129465	3597	3	828
NM_014696	-0.4141382	-1.4186639	0.502410688	2.18E-05	3629	3	644
NM_006167	-0.3312464	-1.0250432	0.47771465	0.002061676	2355	3	217
NM_004368	-0.6233135	-1.2791295	0.376900611	0.000425956	1533	3	258
NM_004368	-0.6527184	-1.2174871	0.376900611	0.00093519	1534	3	277
NM_021009	-0.7690303	-0.5675273	0.419703609	0.000487351	2423	3	581
NM_021009	-0.8310429	-0.6382567	0.419703609	0.001874024	2422	3	647
NM_021934	-0.3415319	-1.0522007	0.583798915	0.001470102	3664	3	33
NM_021934	-0.245528	-0.8262469	0.583798915	0.001894877	2637	3	103
CN646119	-0.4263792	-1.0702424	0.426250594	0.005163121	1543	3	196
CN646119	-0.2319449	-0.8815328	0.426250594	0.008774209	1925	3	137
NM_134421	-0.1251528	-0.5919746	0.357571897	0.002533219	2099	3	185
NM_134421	-0.043759	-0.4413159	0.357571897	0.0157176	2097	3	608
NM_138793	-0.3543687	-1.1205723	0.682985576	0.002849374	3653	3	187
NM_138793	-0.2333974	-0.8188293	0.682985576	0.001859496	2639	3	96
NM_018471	-0.5169225	-0.4803258	0.663443371	0.030946862	2456	3	821
NM_005470	-0.0846051	-1.4848028	0.515955413	0.000190388	3716	3	576
NM_004146	-0.3033655	-0.9030932	0.343754151	0.008843253	1918	3	139
NM_004146	-0.288963	-0.7632123	0.343754151	0.001759331	1920	3	58
NM_203505	-0.6393294	-0.9252846	0.463592435	0.000117624	1394	3	1108
NM_203505	0.18772772	-0.6916482	0.463592435	0.001401885	2114	3	381
NM_053050	-0.1834088	-0.6682559	0.494774548	0.02285802	2060	3	278

NM_053050	-0.4333632	-0.8052876	0.494774548	0.043903601	2445	3	568
XM_371380	-0.4149905	-0.1515399	0.303028825	0.010180536	2522	3	1170
XM_371380	-0.1806741	-0.4077977	0.303028825	0.010754889	3134	3	562
XM_376724	0.25651297	-1.0543138	0.77534552	0.001341373	4080	3	759
NM_030929	0.14630595	-0.9808688	0.564832413	0.025942782	2334	3	1025
NM_014149	-0.6898776	-1.5773124	0.40090935	0.008541598	1516	3	1269
NM_014207	-0.1073753	-1.2100791	0.33174472	0.000170031	3630	3	547
NM_014207	-1.0335159	-0.6378228	0.33174472	0.013102284	1401	3	1534
CB230918	0.09940006	-0.6423748	0.525865598	0.011012386	2393	3	628
NM_004332	-0.6218472	-0.7544983	0.21332748	0.014767327	1901	3	570
NM_005176	-0.1866422	-0.7211757	0.30626903	0.001459275	2088	3	46
NM_005176	-0.2235844	-0.7247257	0.30626903	0.011455337	2087	3	118
NM_005028	-0.6945384	-1.0214853	0.601106419	0.001031476	1410	3	1646
NM_024613	-0.066702	-1.6713303	0.4543767	0.00195643	1577	3	1202
NM_024613	-0.0006381	-1.7674943	0.4543767	0.012029881	1581	3	1469
NM_080603	-0.4220558	-1.173527	0.789727994	0.000865103	3622	3	433
NM_080603	0.13751171	-1.3947932	0.789727994	0.01321745	1692	3	1333
NM_054033	0.93521546	-1.0171739	0.476138221	0.004864842	148	3	1620
NM_054033	-1.9285139	-1.7270994	0.476138221	0.007949593	34	3	1676
NM_054033	-0.6826352	-1.2086006	0.476138221	0.024727731	1677	3	1023
NM_080549	-0.4391118	-0.9881295	0.792359543	0.005069108	2579	3	432
NM_006572	0.22825779	-0.9953387	0.598524471	0.001912233	2854	3	517
NM_006572	0.34261109	-1.0509185	0.598524471	0.005847534	3765	3	986
NM_016495	-0.105557	-0.699042	0.290878536	0.003974697	1886	3	551
NM_016495	0.09511352	-0.8741782	0.290878536	0.042332815	2264	3	734
NM_015540	-0.6756125	-0.3042351	0.469730986	0.001511379	1742	3	1345
NM_001001716	-0.459279	-1.1940452	0.822158794	0.0237641	3919	3	1313
NM_001001716	0.27412573	-0.9854584	0.822158794	0.021474695	4640	3	1393
NM_025083	-0.4241778	-0.79431	0.394173817	0.015396202	1910	3	228
NM_025083	-0.4180637	-0.879158	0.394173817	0.012480879	2465	3	219
NM_139201	0.66318224	-0.9161921	0.673320539	0.003754606	2857	3	1230
XR_011668	-0.1168078	-0.9897774	0.366171456	4.84E-05	2336	3	7
XR_011668	-0.1471924	-0.7190257	0.366171456	0.001193383	1924	3	77
NM_004180	-0.9401968	-0.9432203	0.759351345	0.005640955	1706	3	1107
NM_004180	-0.5745372	-1.1343021	0.759351345	0.012077224	3887	3	801
NM_020820	-0.0983427	-1.0548505	0.308685675	0.003405288	1569	3	165
NM_020820	-0.0751795	-0.9096482	0.308685675	0.00556879	2045	3	89
NM_006356	-0.1662231	-0.7874679	0.34394168	0.022551745	2081	3	214
NM_006356	0.01724856	-0.7747406	0.34394168	0.049266063	2082	3	621
NM_018194	1.10631071	-1.4117561	0.770019373	0.023233167	1357	3	1621
NM_018194	0.21063527	-1.1667447	0.770019373	0.030947437	4615	3	1451
NM_004623	-0.6844899	-1.2817499	0.470145215	0.004396082	1782	3	1455
NM_004623	-0.0531819	-1.0611713	0.470145215	2.93E-05	2337	3	12

NM_004542	-0.2336303	-0.6695966	0.443980236	0.009244211	1927	3	243
NM_004542	-0.2308057	-0.6780945	0.443980236	0.00013519	2590	3	26
NM_024536	-0.1381125	-0.7652698	0.368736004	0.008103422	1923	3	168
NM_024536	-0.2713417	-0.7204906	0.368736004	0.007758194	1922	3	155
NM_022156	-0.4243728	-0.4391798	0.397117061	0.02827648	1973	3	698
NM_022156	-0.3367878	-0.3639029	0.397117061	0.00013239	3083	3	511
NM_139280	-0.5047159	-1.0069611	1.034639245	0.040776699	1178	3	1508
NM_024100	0.10155427	-0.4635703	0.284912087	0.002434206	2175	3	739
NM_024100	0.02778109	-0.6060254	0.284912087	0.006419571	2098	3	392
NM_024419	-0.5031083	-0.2343529	0.240571391	0.021630791	2504	3	1089
NM_198324	-0.158924	-0.554746	0.632918616	0.003005484	2645	3	289
NM_003959	-0.2582702	-0.6560933	0.194158397	0.010336685	1976	3	338
NM_003959	-0.1723953	-0.5390577	0.194158397	0.023084853	2177	3	560
NM_024718	-0.4669528	-1.2150465	0.288059089	0.005482026	2296	3	441
NM_024718	-0.4875519	-1.2090557	0.288059089	0.002962926	2295	3	295
NM_000701	-0.1405073	-0.993806	0.471817825	0.014081144	2247	3	191
NM_000701	-0.1296455	-0.4828576	0.471817825	0.017656878	2120	3	465
NM_207336	0.45881808	-0.9203489	0.667339262	0.016959907	3048	3	1270
NM_006628	-2.1626763	-1.2272196	1.081212835	0.01083407	171	3	1683
NM_006628	-0.691253	-0.7293439	1.081212835	0.0163878	4115	3	1427
XR_013652	-0.5509953	-1.0126876	1.159492832	0.010265305	3975	3	1375
NM_032630	-1.1360494	-1.1561209	0.608120787	0.003579742	1442	3	1394
NM_032630	-0.8141753	-1.3970873	0.608120787	0.000359646	1439	3	1179
NM_002950	0.10440137	-0.704436	0.634943919	0.000527414	2899	3	247
NM_002950	0.0138933	-0.7780538	0.634943919	0.000845847	2898	3	111
NM_203290	-0.1663515	-1.7717371	0.484388307	0.001551274	1575	3	1221
NM_203290	-1.0980064	-1.4921363	0.484388307	0.015196998	1375	3	1642
NM_016319	-0.5852994	-1.0655122	0.81482587	0.001860153	3617	3	353
NM_016319	-0.6124631	-0.655494	0.81482587	0.005334282	2455	3	611
XR_012894	-0.2481117	-1.0132955	0.837818556	0.005786463	2754	3	591
XR_012894	-1.3920867	-1.0695109	0.837818556	0.010808652	199	3	1606
NM_002918	-0.4318411	-0.4949494	0.124664745	0.024783218	1965	3	728
NM_002918	-0.4657514	-0.4818877	0.124664745	0.040175503	1937	3	809
CO583502	-0.0609555	-1.1304292	0.966916611	0.003452355	4078	3	872
CB549630	0.02383666	-0.4520265	0.373295449	0.000508244	2172	3	546
NM_016230	0.13386595	-0.5686806	0.487834999	0.001998065	2207	3	620
XR_013472	-0.1393936	-1.3934497	0.449227833	0.003119913	3731	3	489
XR_013472	0.09146828	-1.2672004	0.449227833	0.004151479	2251	3	649
NM_024111	-0.0893267	-1.3703077	0.674676818	0.002788111	14	3	1692
NM_024111	0.18295825	-0.9865635	0.674676818	0.005209092	3766	3	329
NM_006427	-0.1837317	-0.7996907	0.790969084	0.019026433	2861	3	779
CN648668	-0.2587068	-1.6660419	0.715848337	0.012470196	1351	3	1484
CN648668	-0.445119	-1.4002307	0.715848337	0.002881316	3635	3	839

NM_016479	-0.0224088	-0.8146592	0.379653069	0.000822486	2105	3	32
NM_016479	0.05537572	-1.0472939	0.379653069	0.006441206	2259	3	330
CN805014	-1.1689982	-1.4501573	0.677601915	0.000139966	1436	3	1458
CN805014	-0.7062317	-1.683384	0.677601915	0.000859233	3596	3	1297
NM_002939	-0.2547609	-0.7424623	0.574959383	0.002709576	2573	3	82
NM_002939	-0.3361491	-0.6885812	0.574959383	0.000651681	2615	3	44
NM_020151	0.29537855	-1.0664267	0.315673938	0.021904304	2262	3	1009
NM_020151	0.25949326	-0.9274873	0.315673938	0.025932315	2265	3	963
NM_024736	-0.4030326	-0.7883422	0.750309215	0.008640235	2580	3	452
NM_024736	-0.4252849	-0.9804552	0.750309215	0.001257981	2578	3	132
NM_198679	-0.0403601	-1.0867108	0.628105935	0.002449224	2331	3	210
NM_198679	-0.3215855	-1.1755478	0.628105935	0.000343974	3641	3	39
NM_014366	-0.7684633	-0.7197086	0.523216427	0.026292253	2437	3	998
NM_014366	-0.9867472	-0.71934	0.523216427	0.044632615	2432	3	1341
NM_197977	-0.2931329	-0.5612813	0.499971276	0.020453929	1798	3	1340
NM_005644	-0.17335	-0.5424346	0.372828088	0.000454121	2140	3	332
NM_005644	0.00368444	-0.575538	0.372828088	0.002928762	2101	3	331
NM_025146	0.19203638	-1.0984369	0.674649602	0.004259513	3764	3	631
NM_004715	0.20113696	-1.1729674	0.523848335	0.003904969	2849	3	816
NM_004715	-0.4662514	-0.9844106	0.523848335	0.003379758	3891	3	240
NM_007170	-0.2454152	-0.4037691	0.37800934	0.029162938	2210	3	781
NM_001001349	-0.0219361	-1.0333997	0.329805012	0.005611112	2044	3	156
NM_001001349	-0.0059577	-0.7874701	0.329805012	0.017031861	2084	3	269
NM_025191	-0.2404379	-1.3322093	1.043096238	0.023593575	1103	3	1506
NM_025191	1.30780569	-1.3193875	1.043096238	0.04093394	571	3	1619
NM_001421	-1.6784627	-1.3470048	1.078017634	0.014344306	5349	3	1654
NM_017841	0.16132296	-1.119409	0.836714143	0.000661496	2932	3	627
NM_017841	-0.0448605	-1.0117412	0.836714143	0.000971148	2930	3	569
NM_030574	-1.1607374	-1.2296048	0.830280235	0.007040401	200	3	1554
NM_032557	0.06700327	-1.6035166	0.763937376	0.029298097	1352	3	1555
NM_032557	-2.5723768	-0.8223752	0.763937376	0.018116141	39	3	1678
NM_002375	-0.7744501	-0.9937808	1.300254664	0.011842969	207	3	1605
NM_144658	-0.1460577	-0.5307326	0.54697694	4.80E-05	2204	3	442
NM_002578	0.10119645	-1.0826081	0.489022128	0.000574745	2340	3	259
NM_002578	-0.3237742	-1.8586649	0.489022128	0.010439524	188	3	1574
CB310020	-0.077895	-1.5153572	0.660782456	0.00109668	1415	3	1537
CB310020	-0.3205999	-1.1029794	0.660782456	0.005554708	1413	3	1584
CN646657	-1.0426479	-1.1839468	0.841803595	0.002617788	1446	3	1323
CN646657	-1.0391729	-1.0923189	0.841803595	0.002874071	1449	3	1304
NM_016570	-0.6097647	-0.877974	0.73531645	0.003098159	2446	3	463
NM_016570	-0.4401788	-1.3549273	0.73531645	0.021197514	1671	3	1435
NM_014282	-0.9712239	-0.8913372	0.082477904	0.046173982	1453	3	1267
CN806518	-0.0781247	-0.8637195	0.885673415	0.007838382	3913	3	951

CN806518	-0.1533894	-1.114524	0.885673415	0.01021767	3897	3	863
NM_014063	-0.2774676	-0.7581682	0.494892092	0.005598975	1926	3	184
NM_032370	0.1579103	-0.988147	0.465500038	0.00908285	2327	3	709
NM_032370	-0.2641892	-0.5756058	0.465500038	0.034046216	2636	3	619
NM_005153	-0.4807709	-0.853367	0.891409428	0.003314425	2730	3	585
NM_005153	-1.022853	-0.7673694	0.891409428	0.002290919	1707	3	1347
NM_018982	-0.1993118	-1.4028645	0.900568383	0.00134116	3709	3	788
NM_006623	-0.916954	-0.4515293	0.236040873	0.017562553	2493	3	1252
NM_030762	0.25846116	-1.7648519	0.555084329	0.045323021	1360	3	1637
NM_030762	-0.150925	-1.0698512	0.555084329	0.008911387	2326	3	498
NM_138346	-0.2712986	-1.2216921	0.628616137	0.000275048	3655	3	51
NM_138346	-0.0004974	-1.1408877	0.628616137	0.000715742	3744	3	115
NM_032648	0.22322594	-0.9490537	0.559015475	0.00371099	2357	3	853
NM_032648	0.07376864	-0.8503678	0.559015475	0.00118962	2346	3	195
NM_080927	-0.2916957	-0.9050333	0.5389352	0.000123481	1395	3	183
NM_080927	0.11222484	-1.0992436	0.5389352	0.001111704	191	3	1597
NM_002878	-0.1101583	-0.8944051	0.297630428	0.004817815	2046	3	125
NM_016050	-0.2744532	-0.5014843	0.530463524	0.000770608	2591	3	238
NM_017838	-0.2430902	-0.7938798	0.566089246	0.001242749	3663	3	10
NM_017838	-0.0964642	-0.9078269	0.566089246	0.00395174	2073	3	41
NM_001968	-0.6934283	-0.6192842	0.805309188	0.030926748	228	3	1648
NM_212472	0.42594541	-0.6554724	0.373299153	0.037332585	2236	3	1264
NM_001010856	-1.0247069	-0.7882188	0.885240783	0.002372183	1713	3	1144
NM_006591	-0.2054736	-1.0019723	0.614088346	0.001693289	3669	3	75
NM_138387	-0.6261616	-1.4620086	0.593277066	9.82E-05	3600	3	589
NM_138387	-0.4556031	-1.7200102	0.593277066	0.000517868	3605	3	1146
CK231716	-0.4208537	-0.6961763	0.644928147	0.001407588	1396	3	777
CK231716	-0.6128852	-0.8723212	0.644928147	0.048275133	2444	3	1082
NM_015463	-0.1999499	-0.9194243	0.952819367	0.015770261	4624	3	1081
NM_019070	-0.1498122	-1.0046944	0.595134535	0.000534303	2339	3	60
NM_019070	-0.2343142	-1.0374282	0.595134535	4.21E-05	3665	3	2
NM_016639	-0.4121923	-1.3624539	0.796370319	0.002564035	3917	3	926
NM_018109	-0.4130262	-1.0133805	0.682529663	0.001167118	3892	3	369
NM_018109	-0.028203	-0.728844	0.682529663	0.026602493	2131	3	751
NM_145051	-0.0692879	-1.0194609	0.473176751	0.006645961	2332	3	223
NM_015079	0.04217366	-1.09165	0.866106008	0.000586792	2931	3	387
NM_015926	-0.5981465	-1.2976681	0.81644692	0.001367269	3636	3	714
NM_015926	-0.3636231	-1.2063375	0.81644692	0.002234112	3828	3	341
NM_006763	-1.1273721	-0.1785253	0.374868128	0.014511543	1753	3	1523
NM_018009	-0.418359	-0.995028	0.591753332	0.001026103	2576	3	134
NM_012254	-0.1111671	-0.7454946	0.671064679	0.01695323	2386	3	665
NM_031314	-0.0772812	-0.7134934	0.536380107	0.003131125	2076	3	129
NM_031314	-0.1810104	-0.6497293	0.536380107	0.027074199	2061	3	359

NM_005135	-0.7246212	-0.2788501	0.514922604	0.017594473	2490	3	1348
NM_015426	-0.4330559	-0.8935937	0.99157691	0.046262758	1409	3	1573
NM_022496	-0.0642774	-0.5433889	0.376331766	0.016795951	2103	3	439
NM_018247	0.07422158	-1.068421	0.37509219	0.009162866	2258	3	430
NM_018247	0.0846675	-0.6018731	0.37509219	0.038620595	2122	3	776
NM_005488	0.01612508	-1.2428991	0.86707312	0.003746642	2285	3	1153
NM_004550	-0.6029312	-0.987666	0.49119735	0.007369644	2439	3	297
NM_004550	-0.4200888	-0.6868888	0.49119735	0.004326776	2471	3	171
NM_005998	-0.3628332	-0.9432608	0.659378834	7.94E-05	3661	3	4
NM_005998	-0.3325969	-0.903324	0.659378834	0.00015797	3662	3	5
NM_024839	0.1097989	-0.6619587	0.566536834	0.001076697	2996	3	422
NM_018320	-1.369482	-0.9642693	0.695810783	0.025774263	40	3	1669
NM_018320	-0.3478608	-0.6951657	0.695810783	0.043031751	4606	3	1024
NM_032160	-0.4098228	-1.4724171	0.206316976	0.037729428	1474	3	1489
NM_032160	0.11639253	-1.1251064	0.206316976	0.016718833	2260	3	740
NM_001500	0.33604399	-1.0549315	0.674455824	0.000480031	2855	3	918
NM_001500	0.459622	-1.6107981	0.674455824	0.019603807	556	3	1564
XR_010332	-0.0648963	-0.8020255	0.47418484	0.020992651	2124	3	522
NM_032855	-0.2128756	-0.7520762	0.72551027	0.001585988	2657	3	226
NM_007373	-0.0636472	-0.5716128	0.45028917	0.022237103	2126	3	924
XM_496288	-0.2634327	-0.6811825	0.733956938	0.005636589	2617	3	328
XM_496288	-0.4078936	-0.7338806	0.733956938	0.000942877	2616	3	140
XM_496288	-0.2626108	-0.6671798	0.733956938	0.001118306	2618	3	163
NM_001779	-0.4572572	-0.6412427	0.370084953	0.016146999	2426	3	254
CN806478	0.05323843	-1.2902214	0.873385795	0.011012339	1356	3	1276
CN806478	-0.1407199	-0.9006401	0.873385795	0.008860568	4607	3	1166
NM_133328	-0.7667042	-1.3194987	0.510649432	0.003496077	1438	3	958
NM_133328	-0.7141754	-1.1516148	0.510649432	0.0027506	1393	3	1043
NM_005973	-1.0386239	-0.7204247	0.342156837	0.030439607	1780	3	1589
NM_005973	-0.3573102	-1.0423994	0.342156837	0.00171406	2299	3	144
NM_004563	-0.8094997	-0.5939647	0.983595433	0.035183361	4381	3	1436
NM_024069	-0.8385617	-1.669122	0.660902282	0.003053636	1673	3	1424
NM_024069	-0.6024408	-1.4146352	0.660902282	0.00246614	1680	3	899
NM_018590	-1.5285598	-1.6023152	0.765706336	0.002093957	1435	3	1615
NM_018590	-0.621494	-0.5404412	0.765706336	0.025405905	1408	3	1503
NM_005078	-0.4533045	-0.4779784	0.657285347	0.001393901	2610	3	680
NM_005078	-0.9030848	-0.3000488	0.657285347	0.032399888	1754	3	1453
NM_005827	-0.7586198	-0.5466877	0.637138152	0.030140496	2438	3	1092
NM_004759	0.05449269	-0.8072403	0.479583131	0.000107968	2107	3	69
NM_004759	0.00858383	-0.8046937	0.479583131	0.001823469	2106	3	76
NM_013388	0.1538159	-1.0044852	0.404345331	0.008154042	2050	3	552
NM_003186	-0.4859482	-0.5641097	0.613874983	0.000173502	2604	3	172
NM_003186	-0.4862934	-0.7511889	0.613874983	0.002912766	2751	3	104

NM_014941	0.16488642	-0.6865833	0.48665222	0.000201216	2115	3	313
NM_145113	0.43998036	-1.2904249	0.718367098	7.80E-05	2852	3	1160
NM_145113	0.30740375	-1.3903238	0.718367098	0.00015842	3761	3	1060
NM_015331	0.24391092	-0.7262003	0.443872132	0.012213513	2389	3	882
NM_006139	0.19187886	-1.297023	0.31872963	0.027370311	2255	3	1133
NM_002484	-1.0089369	-1.1884206	0.656084709	0.007815188	1430	3	1335
NM_002484	-0.9823614	-1.209432	0.656084709	0.008016231	1668	3	1303
NM_007046	-0.151563	-1.1620176	0.67247077	0.002746333	2330	3	273
NM_007046	0.12630443	-0.8583715	0.67247077	0.012603376	2380	3	784
NM_018639	-0.3321729	-0.5752224	0.595105209	0.038974996	2760	3	655
NM_018639	-0.2099135	-0.60372	0.595105209	0.046617318	2761	3	749
NM_003819	-0.6966025	-0.7214784	0.646946702	5.86E-05	2410	3	325
NM_003819	-0.6220683	-0.3694642	0.646946702	0.000548143	2603	3	824
NM_175852	-0.0580445	-0.9613587	0.560871003	0.001670135	3670	3	81
NM_175852	0.2279464	-0.9915134	0.560871003	0.00093142	3767	3	397
NM_017918	-0.0968122	-0.923748	0.713714938	0.002334171	3029	3	213
NM_145869	-0.1047879	-0.7006049	0.366741403	0.00377193	2089	3	94
NM_145869	-0.0215318	-0.5530492	0.366741403	0.003913206	2100	3	307
CO649104	0.34556717	-1.3581063	0.736997517	0.001634527	2850	3	1168
CO649104	0.41314794	-1.2868633	0.736997517	0.00145664	2851	3	1246
NM_006871	-0.7832554	-1.1676834	0.78558863	0.001737765	1445	3	830
NM_006871	-1.2084069	-1.5164547	0.78558863	0.000253783	1437	3	1502
NM_175744	-0.460789	-1.1109184	0.491654827	0.00139587	1542	3	73
NM_175744	-0.3148621	-1.1859193	0.491654827	0.000166333	1538	3	8
NM_001893	-0.0732619	-1.306016	0.702149417	0.00304337	3733	3	480
NM_005381	-0.2459575	-1.195781	0.146630546	0.016974815	1564	3	675
NM_005381	-0.357359	-1.3436035	0.146630546	0.016182432	1562	3	862
NM_014046	-0.2278257	-0.3615683	0.478190677	0.000161337	2516	3	632
NM_015173	-0.4573665	-1.4412572	0.374730148	0.021499543	1548	3	1137
NM_019034	0.32186038	-0.8607986	0.201971002	0.014303457	1632	3	1032
NM_020642	0.03220702	-0.7453619	0.632473635	0.002474625	2129	3	256
XR_014722	-0.7029235	-0.4431713	0.757475256	0.015028302	2718	3	1065
NM_175866	-0.1725738	-1.3182693	0.61460172	0.001568626	3732	3	218
NM_175866	-0.2003763	-1.1243397	0.61460172	0.008652878	2246	3	288
NM_003827	-0.2144754	-0.865418	0.964557218	0.022863833	4630	3	1235
NM_003827	-0.2232419	-1.1431705	0.964557218	0.005144961	3922	3	904
NM_004279	-0.3685421	-0.5231581	0.436947791	0.0406781	2058	3	556
NM_145271	-0.4234431	-1.1696451	0.150176687	0.042136019	1521	3	1071
NM_145271	-1.220065	-0.9260515	0.150176687	0.027072462	1779	3	1541
NM_002985	-0.1451112	-1.601504	0.503532956	2.66E-05	371	3	691
NM_002985	-0.2687907	-1.603187	0.503532956	3.36E-05	369	3	648
A_01_P007044	-1.9496741	-1.7872021	0.563815448	1.31E-05	59	3	1681
NM_031210	0.38525092	-1.2105334	0.490060326	0.009340653	2263	3	1197

NM_031210	0.34722567	-0.9315736	0.490060326	0.017654112	2268	3	1070
NM_001007254	-0.2125817	-1.138277	0.690192923	0.000973692	3654	3	151
NM_001007254	-0.0269304	-1.2914627	0.690192923	0.001277235	3710	3	544
NM_017724	0.03486107	-1.1262252	0.396420856	0.003727629	2256	3	380
NM_017724	0.25109991	-0.7012858	0.396420856	0.007349302	3326	3	707
NM_004046	-0.3995749	-0.7175531	0.50356762	0.024047731	2057	3	266
NM_004046	-0.538704	-0.6455264	0.50356762	0.030305685	2056	3	574
NM_006640	0.05564153	-1.120906	0.569505382	0.025400489	2361	3	1094
NM_006640	-0.1449486	-0.8476308	0.569505382	0.00699926	1929	3	194
NM_012396	-0.1594198	-1.0591604	0.441589883	0.006012515	2328	3	267
NM_012396	-0.0179986	-1.0240693	0.441589883	0.000136105	2338	3	85
NM_024098	-0.5554142	-1.0311795	0.886934891	0.002941145	3623	3	908
NM_006777	-0.1192949	-1.1448683	0.61158504	0.000224667	3666	3	28
CN805072	0.07591157	-0.6733322	0.466206961	0.002107333	2109	3	271
CN805072	0.00276296	-0.6360664	0.466206961	0.004054273	2108	3	239
AL833264	-1.2689215	-0.1347191	0.534489999	0.024888347	213	3	1643
NM_207340	-0.1973369	-1.4170728	0.67532283	0.000248887	3701	3	520
NM_207340	-0.1210246	-1.4857915	0.67532283	3.71E-05	3717	3	527
XR_013439	-0.0508471	-1.1566834	0.95872697	0.001286123	3696	3	719
NM_005027	0.20156629	-1.1791935	0.383479512	0.012514727	2321	3	1015
NM_005027	0.02061097	-0.9278949	0.383479512	0.021663203	2356	3	836
NM_018103	0.08612829	-1.546516	0.3712227	0.000539281	1614	3	936
NM_018103	-0.1888036	-1.9880456	0.3712227	0.007088892	1573	3	1535
NM_032227	-0.4436226	-0.9025621	0.833268569	0.002370566	2752	3	255
NM_032227	-1.0234956	-0.6862841	0.833268569	0.009659107	2433	3	1352
XR_010199	0.10080869	-1.2990466	0.763819007	0.000398127	3759	3	550
XR_010199	0.03959823	-1.1806379	0.763819007	0.000252386	3746	3	335
NM_005981	-0.1126689	-0.8552041	0.381186701	0.008070953	2047	3	91
CO647711	-0.1902206	-1.1112722	1.080089692	0.007780742	3923	3	1220
NM_003011	0.24360605	-1.0214	0.60001693	0.018762475	2267	3	961
NM_002860	-0.5172861	-0.6693457	0.891036014	0.004584591	2451	3	834
NM_007221	0.0800268	-1.5988341	0.634051208	0.009681831	1355	3	1419
NM_002005	-0.1307551	-0.9272265	0.401243295	0.000913897	1570	3	25
NM_002005	-0.177543	-0.6706144	0.401243295	0.001457284	2587	3	86
NM_000234	0.15896701	-0.5891021	0.345546358	0.003156239	2192	3	738
NM_000158	0.19452643	-0.8570348	0.772175228	0.003117481	3038	3	676
NM_032355	-0.4779879	-1.2836949	0.526225532	0.003009177	3610	3	327
NM_032355	-0.4815596	-0.8294619	0.526225532	0.001405995	2412	3	84
NM_007266	-0.1217697	-0.8448838	0.807953672	0.000780378	2658	3	252
NM_080686	-0.3925555	-1.5259936	0.338468094	0.00592755	2275	3	1408
NM_080686	-0.4182388	-1.5037649	0.338468094	0.004285364	2294	3	929
NM_017758	0.07452758	-0.7760858	0.759535674	0.001007325	2921	3	346
NM_023930	-0.0785932	-1.1850963	0.516515689	0.001092604	2329	3	188

NM_024591	0.0684836	-0.7134493	0.357814619	0.003264049	2191	3	294
NM_024591	-0.145786	-0.4372043	0.357814619	0.007705238	2178	3	593
NM_005904	-0.0731513	-0.6696016	0.60540093	0.000127243	2662	3	434
NM_005904	0.04786557	-0.8919756	0.60540093	0.00874199	2075	3	351
NM_002859	0.13008489	-1.0874096	0.47770659	0.036635735	1800	3	1479
XR_014319	-0.6129311	-0.7437335	0.401658243	0.014435221	2425	3	357
NM_004413	-0.2573018	-1.5307253	0.470765505	0.000356677	370	3	580
CO579741	-0.3915149	-0.8147314	0.636340777	0.000459788	2612	3	30
CO579741	-0.3291816	-0.7399955	0.636340777	0.00084084	2613	3	45
NM_014161	0.03579367	-0.6348107	0.584281763	9.12E-06	2205	3	340
NM_017789	-0.0009811	-1.5391886	0.863669696	0.000540404	3713	3	1218
NM_017789	-0.1795936	-1.4306514	0.863669696	0.000659378	3708	3	915
NM_020070	-0.4216576	-0.8325351	0.632003471	0.019951413	1930	3	573
NM_020070	-0.4439224	-0.6725215	0.632003471	0.008857894	1931	3	376
NM_019008	-0.4213343	-1.357243	0.657899553	0.045227781	101	3	1540
NM_001003891	-0.449501	-0.6412374	0.351110249	0.003524235	1981	3	488
NM_152361	-0.0212699	-0.9298066	0.546653676	0.000831383	1801	3	1549
NM_152361	0.38705126	-0.8371907	0.546653676	0.000185841	3328	3	805
NM_003932	-0.3423588	-0.494308	0.406784381	0.006365395	2476	3	506
NM_003932	-0.5303791	-1.0572455	0.406784381	0.030152329	1559	3	617
NM_000138	-0.2277533	-0.7036445	0.115194145	0.047103288	1975	3	640
CB549360	-1.2444599	-1.89281	0.125276166	0.003775214	184	3	1655
CB549360	-0.5901067	-2.0982909	0.125276166	0.021990574	1658	3	1612
NM_144641	0.1943525	-0.6953443	0.524097939	0.003061526	2116	3	478
NM_172101	0.05541386	-0.9856273	0.983527489	0.038304683	4626	3	1496
NM_016009	-0.324268	-1.3638481	0.946625686	0.004561496	204	3	1560
NM_024708	-0.3606737	-0.3031267	0.372523389	0.037778733	1744	3	1293
NM_000484	0.10722497	-1.0325738	0.729491791	0.016108491	3028	3	1001
CK230065	-0.0081987	-0.7830093	0.648967401	0.000113117	2897	3	62
NM_144582	-0.707169	-0.1845956	0.518788787	0.012869015	2518	3	1328
NM_144582	-1.0468799	-0.1229175	0.518788787	0.012246139	1755	3	1527
NM_001774	-1.0957003	-1.4274243	0.392897466	0.017943436	1642	3	1524
NM_001774	-0.3310512	-1.6724783	0.392897466	0.000667139	368	3	987
XR_012565	-0.4424608	-1.1495286	0.199907592	0.010743144	1563	3	474
XR_012565	-0.4739618	-0.6949158	0.199907592	0.047502355	1561	3	541
NM_003204	-0.5350028	-0.7722975	0.745748111	0.003842883	2450	3	723
NM_003204	-0.6139134	-0.5620358	0.745748111	0.003093438	2448	3	815
NM_013979	0.09402535	-1.2165934	0.798427465	0.000158838	3760	3	471
NM_013979	0.18254062	-0.9958968	0.798427465	8.76E-05	2934	3	485
NM_178508	-0.1800568	-1.2615635	0.305238561	0.001531961	1565	3	250
NM_178508	-0.3767782	-1.031213	0.305238561	0.00696758	1560	3	130
NM_152766	0.05579492	-1.3504466	0.439768016	0.000317669	3720	3	417
NM_152766	0.04003299	-1.2086797	0.439768016	0.000168405	3721	3	167

NM_007169	-0.2925449	-1.014689	0.559170451	0.016172984	1392	3	812
NM_007169	-0.0501754	-0.8070926	0.559170451	0.011975295	2387	3	410
NM_013338	-0.3582077	-1.0885451	1.238695768	0.046807832	1099	3	1579
NM_181468	-0.3673016	-0.6506038	0.838185476	0.003756229	2619	3	501
NM_181468	-0.4070001	-0.8825327	0.838185476	0.000346576	2582	3	178
CB312367	-0.2610436	-1.6201576	0.293043887	0.016283504	1648	3	1380
CB312367	0.02747582	-1.3375014	0.293043887	0.026274783	2320	3	1241
NM_017909	-0.1911921	-0.9469872	0.818904524	0.015249628	1422	3	1461
NM_017909	0.09081635	-1.0653991	0.818904524	0.011933472	4061	3	1283
CN646662	-0.5125382	-0.9926286	0.457408835	0.011282301	2288	3	1135
CN646662	-0.3725915	-1.0978797	0.457408835	0.003073671	2300	3	220
NM_006380	-0.6961567	-0.560873	0.187781764	0.034179525	2494	3	879
NM_004595	-0.5295547	-0.8160347	0.683817921	0.010302577	2750	3	339
NM_004595	-0.7483885	-0.8046994	0.683817921	0.032785496	2443	3	1087
NM_207012	0.36563151	-0.8683404	0.695836393	0.001221141	2407	3	917
NM_015916	0.12339459	-0.78925	0.397274816	0.002631623	2049	3	298
NM_015916	0.21239784	-0.6214632	0.397274816	0.001868404	2215	3	692
NM_002258	-1.125116	-0.719189	0.352753077	0.004035827	1380	3	1635
NM_017947	-0.6546051	-0.6190075	0.766180927	0.001170261	2447	3	530
NM_152862	-0.1498473	-0.6972522	0.330090677	0.025245314	2080	3	438
NM_145203	-0.9318934	-1.1918923	0.723705815	0.011347181	203	3	1547
NM_005911	-0.7893943	-0.6734286	0.42275472	0.006255295	2420	3	600
NM_005911	-0.8284055	-0.5432409	0.42275472	0.025479262	2421	3	1000
NM_001767	0.12063618	-1.0027644	0.492344824	0.00020899	2341	3	169
NM_001767	0.2195545	-1.0941701	0.492344824	8.12E-05	2342	3	291
NM_014188	-0.4989251	-0.4716401	0.68829572	0.010141358	2609	3	769
NM_152678	-0.0076875	-0.7371742	0.54925561	0.010444176	2041	3	406
NM_152678	-0.3350534	-0.6245916	0.54925561	0.01696293	2483	3	626
NM_024845	-0.2638466	-0.8913546	0.481653201	0.002866251	1928	3	78
NM_024845	-0.0514639	-0.8306442	0.481653201	0.002123278	2048	3	47
NM_003389	-1.1407039	-0.5739822	0.851232277	0.023762048	1407	3	1533
NM_003389	0.82402176	-1.4771617	0.851232277	0.000338696	152	3	1665
NM_006831	-0.2208855	-0.7702061	0.816470423	0.001404726	2659	3	201
NM_003093	-0.197826	-1.4595622	0.816988696	6.00E-05	3706	3	502
NM_003093	-0.2980464	-1.3004766	0.816988696	4.85E-05	3647	3	197
NM_138705	-0.3279388	-1.6635465	0.299120486	0.005780923	1473	3	1363
NM_145904	-0.1844414	-1.5334733	0.865151676	2.72E-05	3705	3	771
NM_005662	0.03169654	-0.8547462	0.559530387	0.004041809	2074	3	162
NM_003893	-0.4917334	-0.3430989	0.438873613	0.041289057	2650	3	1017
NM_016113	0.08956347	-0.6357916	0.580576692	0.000536749	2193	3	375
NM_001607	-0.5801131	-0.845542	0.649004852	0.003793148	3893	3	352
NM_001607	-0.2836968	-0.8040631	0.649004852	0.006862289	2759	3	274
NM_178820	0.22035366	-1.4625707	0.298989843	0.020612882	146	3	1596

XM_372262	0.14822559	-0.5842127	0.509402975	0.000192799	2194	3	446
NM_006698	0.14329157	-1.202698	0.545909239	0.002119852	3747	3	415
NM_006698	-0.0383115	-1.031045	0.545909239	0.001038376	2051	3	106
NM_181441	-0.2652111	-0.9269209	0.590559622	0.001588904	3668	3	14
NM_181441	-0.2106452	-0.5999876	0.590559622	0.002081338	2592	3	154
NM_002419	-0.4032735	-0.5663649	0.744657787	0.000102121	2606	3	225
NM_002419	-0.4512742	-0.5943541	0.744657787	1.89E-05	2605	3	192
NM_004436	-0.7444804	-1.3590738	0.981699275	0.002215422	3586	3	1272
NM_006423	-0.35095	-0.5329144	0.698953668	0.004320143	2611	3	507
NM_032038	-0.2649454	-1.8104016	0.638847161	0.000298353	366	3	1268
NM_021074	-0.256041	-1.1214271	1.090932825	0.003154987	3878	3	950
NM_021074	-0.0414677	-1.1644432	1.090932825	0.004636513	3879	3	1141
NM_012179	-0.5143272	-1.4715216	0.922984806	0.000173795	3604	3	916
NM_012179	-0.8375142	-1.3566073	0.922984806	0.000234449	3603	3	1149
NM_020191	-0.0724761	-1.0598862	0.935427506	0.003092387	3675	3	636
XR_009909	-0.9114461	-1.4884789	0.584902453	0.014737868	1672	3	1444
XR_009909	-0.6063685	-1.5751552	0.584902453	0.030291766	1670	3	1521
NM_006747	-0.303844	-1.2155104	0.956069805	0.007090604	3921	3	1083
NM_006747	-0.169021	-0.8894295	0.956069805	0.024305111	4631	3	1240
NM_016118	-0.0582407	-1.5965151	0.524593285	0.001107995	1582	3	874
NM_016118	-0.0245933	-1.5246406	0.524593285	0.005353643	1583	3	981
NM_080430	-0.157764	-0.9323756	0.955876847	0.032013651	4629	3	1346
CN648602	-0.529404	-0.668873	0.554351007	0.006467415	2428	3	388
NM_001066	-0.0927787	-1.0286917	0.704237517	3.18E-06	2879	3	11
NM_001066	-0.0036584	-0.8819312	0.704237517	2.16E-05	2880	3	48
XR_011066	-1.1767215	-1.6526388	0.283072687	0.049209082	90	3	1609
NM_201434	0.05304102	-1.7054603	0.57581684	1.47E-05	1579	3	1099
NM_201434	-0.1667828	-1.64641	0.57581684	0.000140832	1578	3	996
NM_024664	-1.2697966	-0.6005615	0.826380056	0.002223596	165	3	1578
NM_024664	-0.4973609	-0.7463986	0.826380056	0.002858773	2753	3	493
NM_173507	-0.5260245	-0.5364164	0.774216931	0.004177971	2620	3	671
NM_173507	-0.1719097	-1.1264472	0.774216931	0.000464501	3656	3	100
NM_021170	0.43743363	-0.8831098	0.492558606	0.005419647	2405	3	1237
A_01_P003274	-0.6316591	-0.5384804	0.81582497	0.000380806	1815	3	1399
NM_000819	-0.6417933	-1.4205606	0.615854823	0.022908105	1676	3	1320
NM_000819	-0.2258376	-0.8523084	0.615854823	0.006839938	2764	3	131
CO579800	-0.4329349	-0.5942055	0.826932132	0.002240731	2621	3	497
NM_018389	-0.1580407	-0.8216958	0.883637536	0.000386335	2666	3	233
CB228901	0.23911803	-0.8736601	0.671159155	1.53E-06	2905	3	356
NM_025201	0.3142188	-1.2554613	0.781334336	0.000196766	3756	3	866
NM_025201	0.34516868	-1.0954911	0.781334336	0.000409072	3757	3	837
NM_001697	-0.1390438	-0.8160374	0.519772963	0.001937853	2067	3	31
NM_001697	-0.1200088	-0.8395802	0.519772963	0.002121696	2066	3	21

NM_002342	-0.4958865	-0.8796332	0.665244894	0.00264387	2468	3	482
NM_033085	0.61770637	-1.0414872	0.50835883	0.043349832	1362	3	1645
NM_152318	-0.5282295	-1.3526949	0.653797041	0.000665008	3619	3	350
NM_152318	-0.3416581	-1.143502	0.653797041	0.001087338	3657	3	56
NM_005932	0.0496912	-1.0331203	0.835892605	0.023739229	3898	3	1259
NM_003751	0.13403294	-1.562272	0.71029223	0.010187498	1343	3	1490
XR_013497	0.06396396	-0.7138812	0.555169014	0.00501452	2128	3	286
XR_013497	0.27788876	-0.8661994	0.555169014	0.018909537	2269	3	995
NM_021732	-0.1132299	-0.434428	0.409364354	0.010484619	1796	3	1181
NM_022766	-0.4881685	-1.5349001	0.635317604	0.00323805	3608	3	1047
NM_006114	-0.3664742	-1.1625945	0.742133973	0.01886833	2281	3	1294
NM_006114	-0.0874373	-0.9649295	0.742133973	0.019882923	2359	3	1063
NM_001838	-0.2874119	-0.7984692	0.63294888	0.006940707	1397	3	1409
XR_011940	0.31181868	-0.9791697	0.848005657	0.000474893	2892	3	856
NM_152829	-0.66008	-0.7047089	0.899497417	0.001820144	1735	3	1385
NM_031903	-0.1736618	-0.8438642	0.675039505	0.010199877	2558	3	279
NM_031903	-0.4374653	-1.0245503	0.675039505	0.031335548	2441	3	888
NM_020182	-1.763869	-1.7714282	0.528839935	0.002312345	60	3	1685
NM_020182	-0.1911812	-1.4766681	0.528839935	0.007407036	1589	3	974
NM_030926	-0.3535948	-0.8473949	0.896070684	0.001892014	2756	3	423
NM_000904	-0.1029273	-0.9873897	0.884626726	0.000179765	3699	3	182
NM_000904	0.10246628	-0.9267723	0.884626726	0.00019867	2935	3	425
NM_000112	0.02695166	-1.2184234	0.807632613	0.003252626	4067	3	794
NM_000112	0.10276428	-0.9709568	0.807632613	0.00344456	3031	3	715
NM_018460	0.54819684	-1.1592797	0.793417747	0.003832456	2853	3	1372
NM_032477	-0.2096782	-1.3864325	1.003344547	0.000130486	3672	3	753
NM_032477	-0.1235727	-1.0539535	1.003344547	0.000372918	3697	3	512
NM_022831	-0.2069166	-0.8621029	0.986643779	0.01318707	3904	3	1080
NM_006613	-0.3053486	-0.6739528	0.492717058	0.001520969	2586	3	63
NM_012100	-0.357866	-0.9902464	0.583956398	0.000214434	3659	3	3
NM_012100	-0.3147481	-0.9968516	0.583956398	0.000252598	3660	3	1
NM_000152	-0.1422372	-0.9888075	0.607110329	0.003476993	2555	3	447
NM_000152	-0.5508836	-0.9459359	0.607110329	0.004633233	4603	3	642
NM_018340	-0.0661771	-1.2357166	1.098687402	0.019443449	4617	3	1497
NM_018340	-2.4558602	-1.180382	1.098687402	0.019519214	172	3	1672
NM_015999	-1.0269856	-1.1430861	0.222227667	0.032690231	1643	3	1412
NM_015999	-0.6283198	-0.9110419	0.222227667	0.016373261	1900	3	673
NM_016068	-0.4710566	-0.4513871	0.689230566	0.000896323	2608	3	523
NM_016068	-0.4283484	-0.5483467	0.689230566	0.000188835	2607	3	221
NM_006634	-0.2934412	-1.1756911	0.82706422	1.20E-05	3648	3	109
NM_006634	-0.1079753	-1.1508783	0.82706422	1.93E-05	3650	3	133
NM_000693	-0.3075268	-1.5981098	0.124693212	0.010316939	1387	3	1365
NM_000693	0.21711572	-1.3876987	0.124693212	0.012127067	1603	3	1326

NM_000259	0.05805998	-1.0443098	0.351182845	0.029508813	2261	3	806
NM_015358	-1.0479099	-0.7376052	1.202455884	0.013028818	1770	3	1575
NM_022779	-0.3876976	-0.871626	0.479032461	0.011493563	2469	3	179
NM_022779	-0.0204886	-1.2466977	0.479032461	0.020220701	2250	3	1185
NM_006291	-0.3084127	-1.2570035	0.72959108	0.000305408	3643	3	290
NM_006291	-0.1978416	-1.1891089	0.72959108	9.75E-05	3649	3	87
NM_170711	-1.2292348	-0.7905253	0.4113619	0.019466704	1427	3	1491
NM_018067	-0.0186859	-0.9005041	0.642103819	0.000278559	2344	3	61
NM_018067	0.09100144	-0.7612327	0.642103819	8.13E-06	2906	3	152
NM_006612	0.02938761	-0.9730137	0.384345745	0.025298378	2554	3	699
NM_152456	0.40265253	-1.8776842	0.131452006	0.015020581	1639	3	1592
NM_152456	0.42995708	-1.5528696	0.131452006	0.006351937	1601	3	1517
CN647305	-0.3908123	-1.5285435	0.611009728	0.00190354	3606	3	752
CN647305	-0.4707613	-1.3990388	0.611009728	0.002260686	3611	3	561
NM_033101	0.13350012	-0.8743009	0.881733835	0.024910995	4627	3	1279
XM_291085	-0.0414949	-1.0883039	0.654179692	0.000187479	2343	3	110
XM_291085	0.01603287	-0.6674949	0.654179692	0.003613301	2391	3	486
NM_003900	-0.9374887	-0.6655284	0.696099182	0.001161621	1403	3	1389
CB550154	-0.252852	-0.7319389	0.501788292	0.015801031	2064	3	166
CB550154	-0.2037758	-0.5732826	0.501788292	0.047341695	2063	3	624
NM_006582	-0.7194856	-0.9863303	1.109316173	0.014363738	4605	3	1482
XR_010016	-0.4596366	-0.753187	0.711895234	0.035218179	2758	3	817
NM_138792	-1.1815491	-0.7356053	0.221157836	0.044548482	1454	3	1498
XM_376178	-0.4751311	-0.7868831	0.616110211	0.002775147	2472	3	198
XM_376178	-0.2088052	-0.5949298	0.616110211	0.018041629	2062	3	368
NM_032508	-0.1117018	-0.6091817	0.650019007	0.020690158	2560	3	755
NM_032508	-0.8928793	-0.5943621	0.650019007	0.006052349	1405	3	1209
CB308778	0.08568319	-1.001677	0.746161578	0.000345135	2345	3	242
CB308778	0.03918963	-1.2175277	0.746161578	2.05E-05	3725	3	215
NM_004786	-0.547646	-0.6501511	0.552891273	0.020348259	2429	3	629
CN644859	-0.9308523	-0.633632	0.785214139	0.000194995	1404	3	1457
NM_007234	-1.006816	-1.251422	0.925920242	0.001327787	1447	3	1359
NM_007234	-1.0269924	-1.1564624	0.925920242	0.00091783	1448	3	1331
NM_003375	-1.2570334	-1.6958351	0.53015132	0.006851629	92	3	1599
NM_003375	-0.7509145	-1.3556448	0.53015132	0.012655178	1669	3	1214
XR_014116	-0.5108112	-0.7247419	0.766033912	0.004977267	1814	3	1423
XR_014116	0.09185727	-0.9067664	0.766033912	0.008952166	2127	3	1051
NM_005957	-0.247728	-1.0881945	0.493113316	0.041940376	1654	3	1343
NM_000784	-0.2570713	-1.4730877	0.684215787	3.43E-06	3645	3	334
NM_000784	-0.0983144	-1.359577	0.684215787	1.42E-05	3646	3	211
NM_004343	0.21605112	-1.0935608	0.763453428	0.025106494	2362	3	1378
NM_004343	0.18747698	-1.0458626	0.763453428	0.017934888	2364	3	1205
NM_016526	-0.1051385	-1.0785417	0.630199649	0.001593602	3735	3	49

NM_016526	-0.0935509	-0.7126475	0.630199649	0.001769985	2071	3	146
NM_006937	-0.8604893	-0.5060681	0.751914905	0.023159138	2720	3	1244
XR_013467	-0.2663065	-1.3429223	1.023266887	0.002016256	3895	3	1171
NM_007108	-0.1794104	-1.251848	0.128552956	0.043033508	1649	3	1187
NM_080392	0.04903993	-1.3937768	0.901363826	0.000463234	3712	3	820
NM_080392	0.02024881	-1.4941072	0.901363826	0.000934242	3711	3	1038
NM_145690	-0.8566837	-1.1107413	1.093417297	0.007075263	1708	3	1515
NM_001127	0.06157195	-1.3405291	1.014167903	0.000998796	4077	3	1172
NM_001127	-0.2472138	-1.2145565	1.014167903	0.000834644	3829	3	697
NM_001970	0.19503711	-1.1452706	0.787513953	0.017361145	2363	3	1285
NM_001970	0.05510322	-1.1051631	0.787513953	0.012572739	1361	3	1590
NM_017921	-0.1919799	-0.8750739	0.60069199	0.004058423	2040	3	117
NM_024407	-0.2771994	-0.8139152	0.711960161	0.000142315	2595	3	20
NM_024407	-0.2947475	-0.7857866	0.711960161	0.000508956	2614	3	64
NM_199193	-0.393782	-1.1642647	0.409367723	0.019128875	2297	3	684
NM_199193	-0.1579788	-1.135427	0.409367723	0.003812186	2312	3	135
XR_011928	-0.2886666	-0.7011121	0.709947647	0.004710528	2765	3	251
NM_001711	-0.7018046	-0.6931847	0.863880461	0.004646663	1720	3	902
NM_001711	-0.9993703	-0.839136	0.863880461	0.002060099	1719	3	1206
CK230137	0.02328363	-0.8925603	0.700664083	0.027773327	2381	3	1019
CK230137	0.12721478	-0.7692215	0.700664083	0.018281212	2382	3	965
NM_016075	-0.2847701	-0.5049599	0.33080653	0.037229987	2475	3	900
NM_016075	-1.5760147	-0.5844732	0.33080653	0.026822668	1400	3	1572
NM_005574	-0.7138953	-0.58779	0.739872768	0.016378505	2721	3	1066
NM_000093	-0.4400612	-1.2671602	0.614466245	0.008819494	2279	3	782
NM_000093	-0.6391124	-0.9823665	0.614466245	0.018792143	2290	3	948
NM_153437	-0.212676	-0.7153142	0.555362824	0.016064413	2069	3	275
NM_004901	0.24054862	-1.2145856	1.020005992	0.02123992	1181	3	1522
NM_020458	-0.0081672	-1.3827773	0.363590999	0.02177145	1359	3	1456
NM_020458	-0.5988798	-1.8419726	0.363590999	0.034172064	55	3	1686
CO649191	-0.1347483	-1.0319205	1.007136849	0.000117651	3694	3	389
CO649191	-0.1581273	-0.989195	1.007136849	0.000342797	3695	3	479
NM_004793	-0.4991101	-0.8418742	0.888237448	3.42E-05	2449	3	454
NM_006586	0.18770978	-1.2551164	0.558680536	0.005711296	2322	3	913
NM_006586	0.37362053	-0.8932032	0.558680536	0.013460232	2406	3	1139
NM_130446	-0.2178964	-0.9225856	0.971271408	0.01598106	3903	3	1127
NM_012335	0.01525809	-1.6741908	0.744918568	7.11E-05	3718	3	1134
NM_017567	-0.9624997	-1.3691846	0.611696908	0.017049892	1665	3	1425
NM_017567	-0.8101625	-0.798594	0.611696908	0.030767159	2442	3	1052
NM_018138	0.04351854	-0.8384821	0.497594987	0.044568433	2317	3	1289
NM_018138	-1.1920715	-0.777865	0.497594987	0.004288848	57	3	1694
NM_002730	-0.1083324	-1.1566063	0.640834395	0.000678197	3734	3	66
NM_002730	-0.0644352	-0.8834526	0.640834395	0.00043211	2878	3	36

NM_203500	-0.1565482	-1.5227346	0.413326611	0.004824156	1580	3	1007
NM_203500	-0.3305538	-1.1686641	0.413326611	0.001809171	2298	3	337
CN648445	-1.8963845	-0.2228836	0.864764119	0.026091691	166	3	1661
NM_003366	-0.2968457	-0.6240297	0.7413765	0.035676009	2762	3	886
NM_006370	-0.3855749	-1.0278273	0.731494288	1.32E-05	3658	3	15
NM_006370	-0.647193	-1.1398007	0.731494288	0.001257302	3620	3	408
NM_019557	-0.2924598	-0.7434427	0.656593419	0.001663251	2473	3	235
NM_018841	-0.4779133	-0.9279044	0.33224772	0.045878475	1571	3	831
NM_002430	-0.5288717	-1.3023464	0.546097256	0.000187249	3609	3	323
NM_021975	-0.1991655	-1.2029406	1.066580515	0.038044281	1180	3	1545
NM_021975	-0.2431086	-1.2956646	1.066580515	0.029656133	1179	3	1538
NM_021158	-2.2617317	-0.7937416	1.240211765	0.006012972	28	3	1698
NM_033452	-0.3700127	-1.1544567	0.660968475	0.000751913	3642	3	142
NM_033452	-0.285506	-0.6898821	0.660968475	0.016398369	2585	3	582
NM_017453	-0.155323	-0.8286971	0.538928641	0.009205159	2065	3	116
NM_017453	-0.4845858	-0.706616	0.538928641	0.014214075	2427	3	246
NM_005826	-0.3860209	-0.6172044	0.660108361	0.014771094	2059	3	377
NM_004450	0.16745663	-1.2795123	0.827412978	0.001035781	3752	3	799
NM_022074	-0.3030508	-1.4434564	0.756028557	0.000549937	1681	3	848
NM_022074	0.06152257	-1.2060007	0.756028557	0.000576817	3751	3	360
NM_014623	-0.19442	-0.6309041	0.687075047	0.000167563	2593	3	127
NM_003374	0.00366258	-1.1464699	0.824180733	0.005054909	3739	3	623
NM_031910	0.21240764	-1.4516678	0.653700711	0.017501745	1636	3	1448
NM_014026	-0.4055487	-0.7806865	0.564734169	0.007422349	2470	3	158
NM_014026	-0.1158708	-0.7749681	0.564734169	0.019399262	2068	3	310
BC094693	0.06603646	-0.6763328	0.661574946	0.000191508	2206	3	598
NM_020143	-0.6547207	-0.7075533	0.274994963	0.039952485	1793	3	1261
CN641561	0.05850697	-0.896755	0.885475188	0.000422758	2887	3	390
CN641561	0.1217322	-0.9356143	0.885475188	0.00101051	2886	3	514
NM_014501	0.0406074	-1.3832185	0.626856665	0.000123812	3722	3	499
NM_014501	0.1218975	-0.8149012	0.626856665	0.001272082	2390	3	366
NM_007282	-0.0647379	-0.9392827	0.956086361	0.012282576	2774	3	955
XR_010444	0.29667397	-0.8291116	0.56613751	0.004628103	2055	3	762
NM_015683	-0.7879533	-0.6066453	0.519186412	0.024955526	1402	3	1559
NM_015683	0.27789785	-0.7488926	0.519186412	0.002535287	3327	3	668
NM_000873	-0.3711539	-0.9824746	1.070269888	0.000647149	4119	3	901
NM_000873	-0.2374138	-1.2008745	1.070269888	0.001014535	3674	3	851
NM_182917	-0.3188259	-1.2083237	0.850873008	0.000390626	3830	3	283
NM_182917	-0.3468373	-0.9727746	0.850873008	0.000165412	3685	3	124
NM_001916	0.23861594	-0.8495211	0.716475342	0.000566125	2910	3	601
NM_016146	0.21472692	-1.0537588	0.802408623	8.61E-05	2891	3	495
NM_016146	0.20113079	-0.9092389	0.802408623	0.000265707	2911	3	467
XR_010166	-0.1852374	-1.3317235	0.678341202	0.006599147	1588	3	942

XR_010166	-0.2027637	-0.8519542	0.678341202	0.018662034	1789	3	844
NM_014145	-0.4356884	-0.8571797	1.008525367	0.012553136	3593	3	1111
NM_006457	-0.0044797	-1.0621835	0.967864369	0.001468763	2372	3	857
NM_172373	0.18559601	-0.9283955	0.631767815	0.001286953	2889	3	481
NM_172373	0.1026295	-1.2048991	0.631767815	0.019853848	1693	3	1191
NM_000629	0.15727502	-0.752065	0.370448818	0.032848514	2266	3	658
NM_016293	-0.1537102	-1.1891586	0.860639826	0.002689668	3738	3	462
NM_016293	-0.0228259	-1.2514482	0.860639826	0.004906306	3737	3	847
NM_000599	0.11995118	-1.1030683	0.844108815	0.002470925	3743	3	761
NM_000599	-0.2660778	-0.9857355	0.844108815	0.003687318	3894	3	567
NM_001949	0.17476606	-1.4560365	0.760141066	0.003431128	3729	3	1173
NM_032350	-0.6002316	-1.1823077	0.982954389	4.56E-05	3953	3	616
NM_032350	-0.4805341	-1.1642562	0.982954389	0.000132926	3831	3	440
NM_003025	-0.7586579	-0.9628673	0.375543439	0.026291156	2286	3	914
NM_003025	-0.1160345	-0.8142746	0.375543439	0.023827305	2316	3	525
NM_152307	0.46836165	-1.2357681	0.908323771	0.021568664	4580	3	1548
NM_152307	-0.1284875	-1.0696799	0.908323771	0.003151403	4034	3	736
NM_004612	-0.7695306	-1.2932889	0.851882366	0.001000317	3939	3	1203
NM_004612	0.11976355	-0.9661924	0.851882366	0.000401735	2885	3	318
NM_006148	0.08911953	-1.0564298	0.71988786	0.000302445	2888	3	302
NM_006148	0.02725298	-1.0498653	0.71988786	0.003197865	3740	3	280
NM_016372	-0.2205196	-0.5718185	0.584225866	0.00235148	2589	3	199
NM_002793	0.25612456	-1.1572442	0.752731533	0.000759109	3754	3	724
NM_002793	0.11648893	-1.1577764	0.752731533	0.000753206	3753	3	443
NM_006428	-0.2942782	-0.9894074	0.967979763	0.000895797	3686	3	445
NM_006428	-0.3701013	-0.8349128	0.967979763	0.000413502	3687	3	336
NM_020179	-0.0854986	-1.0661401	0.827890171	0.003440108	3741	3	344
NM_014008	-0.2327674	-0.7694075	0.540990592	0.004765288	2583	3	200
NM_014008	-0.5726646	-0.9603545	0.540990592	0.00140448	2415	3	90
NM_032364	-0.0615808	-1.1997106	1.068262162	0.000589613	3880	3	802
CK231337	-0.5270852	-1.101889	0.856450192	0.002791048	3638	3	592
CK231337	-0.5605899	-1.209888	0.856450192	0.000517726	3637	3	535
NM_000414	-0.6355919	-0.4858429	0.703060001	0.006626617	2722	3	832
NM_006389	-0.33936	-1.0977802	1.259528828	0.001974133	4104	3	1260
NM_183233	-0.1377971	-1.0385343	0.969280602	0.000366168	3698	3	431
NM_003404	-0.117049	-1.1426742	0.686384563	0.001727442	3736	3	149
NM_003404	-0.0237478	-0.6740877	0.686384563	0.004417905	2072	3	343
NM_174925	-0.3458197	-1.4206113	0.634310102	0.00123018	3612	3	500
NM_174925	-0.1696828	-0.9861804	0.634310102	0.001422658	2313	3	70
NM_025147	-0.4700946	-1.4637848	0.927986777	2.59E-06	3602	3	966
NM_025147	0.23542835	-1.2394681	0.927986777	2.96E-05	3762	3	1059
XR_012381	-0.3558126	-0.6473168	0.811100255	0.002081584	2767	3	364
CO581798	-0.6618272	-1.6806778	0.587977237	0.020438675	195	3	1607

CO649006	-0.3468948	-1.4338933	0.968360823	0.008033123	1683	3	1437
CN648211	-0.5427653	-1.092398	0.863190913	0.000644527	1804	3	1011
XM_375261	0.13111569	-1.2558105	0.859877507	3.19E-05	3726	3	638
XM_375261	0.24265638	-1.2774818	0.859877507	0.004776721	3758	3	1175
NM_017712	0.34786312	-1.4620399	0.638754244	0.002111835	1637	3	1353
NM_017712	0.5000729	-1.4300776	0.638754244	0.001161087	3715	3	1207
NM_058229	0.10345005	-1.2547025	0.827113717	0.000646752	3750	3	718
NM_058229	-0.2438741	-1.5773323	0.827113717	0.011300167	1682	3	1492
NM_006360	0.03952622	-0.8224213	0.750747173	0.003600404	2869	3	349
NM_014595	-0.0221838	-0.5480421	0.572064694	0.033035499	2973	3	731
NM_003998	-0.2921037	-0.676414	0.484394819	0.006498827	2588	3	112
NM_016016	0.45413296	-0.7142571	0.493729109	0.009042123	3334	3	1184
NM_078629	0.04746803	-0.7914777	0.589584165	0.006384598	2054	3	308
NM_078629	-0.1144227	-0.5411525	0.589584165	0.033136526	2972	3	635
NM_006368	0.33405157	-1.2164666	1.088133154	0.000312694	4086	3	1281
NM_001545	-0.1498034	-0.6276856	0.664737372	0.025990151	2070	3	641
NM_005429	-0.6462049	-1.0876953	0.714132901	0.004519423	1802	3	850
CO647394	-0.5492378	-1.0050991	1.274355645	0.00113312	3976	3	1247
NM_018049	-0.3974417	-1.0020751	0.712376276	0.000200094	3667	3	17
NM_018049	-0.2069211	-0.8461288	0.712376276	0.000431591	2597	3	24
NM_017949	-0.2107355	-0.8850335	0.806229517	0.000598243	2596	3	122
NM_017949	-0.0719501	-0.933953	0.806229517	5.10E-05	2598	3	97
NM_003581	0.42240032	-0.8393716	0.631017075	0.009257597	3056	3	1162
NM_006638	0.04836579	-0.6479888	0.363712503	0.046452298	2214	3	1114
NM_006238	-0.6907845	-0.9824559	0.555174881	0.004113441	2287	3	483
NM_006238	-0.6913793	-0.935951	0.555174881	0.002011039	2414	3	272
NM_017955	0.12794331	-0.9535001	0.645702522	0.00143466	2367	3	383
NM_017955	0.10589756	-1.1817155	0.645702522	0.000730289	3748	3	355
NM_002275	0.10777912	-1.215402	1.190669849	0.000213191	4087	3	1188
NM_181861	-0.0401778	-0.847895	0.637248926	0.029943576	2125	3	841
NM_006010	-0.0170836	-1.3027038	0.854787413	0.000197775	3719	3	705
NM_006010	0.00861677	-1.1133551	0.854787413	0.003801641	3742	3	614
NM_013397	-0.1949577	-1.0446433	0.965829424	7.60E-05	3692	3	260
NM_013397	-0.1812855	-1.0069777	0.965829424	6.43E-05	3693	3	244
NM_004737	-0.2801231	-1.3797965	0.720789104	0.031738176	1689	3	1383
NM_001018067	-0.0411943	-1.1761682	0.798310039	0.040472815	4056	3	1439
NM_016576	-0.628506	-0.6907722	0.763940092	0.00106831	2417	3	429
NM_016576	-1.0109797	-0.9858506	0.763940092	0.029017956	1666	3	1426
NM_013392	-0.2153328	-1.3885978	0.748088266	0.002149721	3627	3	687
NM_013392	-0.6747382	-1.218575	0.748088266	0.002605394	3618	3	701
A_01_P007358	-1.3320467	-1.0086331	1.365606155	0.007784017	178	3	1628
XR_010902	-1.7414379	-0.4512216	0.887456565	0.000195964	1750	3	1627
XR_010902	-0.7713558	-1.199845	0.887456565	0.031871591	1675	3	1473

XM_374431	-0.72668	-1.1968281	0.098261745	0.038211541	1778	3	1512
NM_000108	-1.6486733	-0.661999	1.023228358	0.000711237	163	3	1641
NM_002803	-0.1694247	-1.2743551	1.074145853	0.000117898	3673	3	746
NM_002803	-0.4331208	-1.2330723	1.074145853	0.00023098	3825	3	868
XR_010958	-0.2709746	-1.2175058	0.757943407	0.006958598	3768	3	823
NM_001983	-0.6013385	-0.9937747	0.597883881	0.020576163	2291	3	894
NM_001983	-0.6705111	-0.858902	0.597883881	0.009247475	2292	3	757
NM_013400	-0.5162204	-1.002725	1.013640912	0.015335689	3590	3	1477
NM_138392	-0.9774574	-1.6460942	0.763024908	0.003190011	1434	3	1519
NM_021625	-0.4375278	-1.2964568	0.917222437	0.004890937	3589	3	1112
NM_002708	0.06197627	-1.019067	0.844739441	1.19E-05	2881	3	232
NM_002708	0.03253119	-0.860588	0.844739441	0.000143095	2883	3	301
NM_018291	-0.0627282	-1.1694291	0.764810684	0.016198417	1696	3	1004
NM_021222	-0.944533	-0.4296588	0.808154735	0.005556529	1759	3	1514
XR_011945	-0.1180136	-0.8175176	0.796700562	0.000271319	2599	3	177
XR_011945	-0.2215535	-0.7484953	0.796700562	0.000313815	2584	3	170
NM_194285	-0.0839839	-1.4833644	0.387667885	0.049038831	1475	3	1468
XR_010915	-0.3920987	-0.9922761	1.217807873	0.001442514	4105	3	1116
NM_024815	0.30204451	-1.5148359	0.879313412	0.000261426	605	3	1282
NM_024815	0.29582939	-1.2483211	0.879313412	7.92E-06	4168	3	969
NM_144563	-0.2322723	-1.5386823	0.332137279	0.009127918	2301	3	1277
NM_144563	-0.4030331	-1.6917247	0.332137279	0.002843165	1388	3	1417
NM_006852	-0.3834974	-0.5339252	0.498518542	0.019700536	2562	3	1069
NM_024678	-1.3225034	-0.2479157	0.828619602	0.005534411	224	3	1653
NM_004892	0.17670538	-1.1477245	1.027078205	4.80E-05	4169	3	1057
NM_198939	-0.2122359	-1.3757876	1.048196038	0.007757338	4029	3	1371
NM_002134	-0.1688287	-1.0437564	0.684940778	0.00336402	2365	3	282
NM_002134	0.00155634	-1.2056081	0.684940778	0.000709557	3723	3	378
NM_033416	-0.6131777	-1.6336875	0.748109409	6.40E-05	3601	3	1118
NM_033416	-0.3560277	-0.870934	0.748109409	0.00066695	2594	3	74
NM_016065	-0.45825	-0.9012118	0.718859954	0.000771705	2053	3	123
NM_016065	0	-0.7069016	0.718859954	0.004261321	2388	3	370
NM_015477	-0.3381938	-1.254705	0.436674698	0.013382451	1391	3	1265
NM_014187	0.07511884	-1.0476616	1.056074947	0.000146794	4241	3	729
NM_014812	-0.0681405	-1.1379281	0.720501653	0.000275445	2556	3	384
NM_014812	-0.4883064	-1.0950302	0.720501653	0.001450227	3621	3	205
NM_014117	-0.0357752	-1.314096	0.796641787	0.001078995	3749	3	504
XR_012050	-0.3801683	-1.0337866	0.601821995	0.037070226	1694	3	876
NM_033389	-0.0366138	-1.3828524	0.918446167	6.10E-05	3724	3	678
NM_004547	-0.2940231	-1.1344695	0.826125359	0.014985299	1695	3	896
NM_004547	-0.2835893	-0.8118873	0.826125359	0.005414811	2766	3	312
NM_178812	0.13599406	-0.983304	0.86436869	0.020900104	4059	3	1186
NM_198446	-0.7330759	-1.0339161	1.140643028	1.88E-05	3955	3	1003

NM_198446	-0.6030138	-0.8222159	1.140643028	6.29E-05	2731	3	903
NM_014819	-0.3263387	-0.5509521	0.638403723	0.035863718	2563	3	845
NM_144593	0.45541902	-1.0627728	0.588716781	0.03908708	4579	3	1488
NM_033209	-0.333677	-1.1894645	0.531912986	0.004249785	2305	3	227
NM_033209	-0.392519	-1.1178767	0.531912986	0.004538655	2306	3	189
NM_033209	-0.364319	-0.9735516	0.531912986	0.002958318	2309	3	50
NM_033209	-0.2853282	-1.1137806	0.531912986	0.003243542	2308	3	80
NM_015140	0.18732129	-1.4756	0.944162194	0.001821309	3774	3	1314
NM_005475	-0.5685634	-0.8210334	1.070000755	0.001145656	1722	3	843
NM_014742	-0.8006367	-1.0845097	0.592139645	0.001711178	2413	3	571
NM_014742	-0.459094	-1.0133974	0.592139645	0.008824104	1572	3	374
NM_003271	-0.2275512	-1.0162813	0.949501882	5.87E-05	3690	3	208
NM_003271	-0.2592992	-0.8500835	0.949501882	0.000108542	3691	3	224
NM_201280	-0.1593906	-0.8277412	0.883017959	0.000122655	4172	3	494
NM_145049	-0.5708575	-0.770893	0.746633435	0.002369198	2416	3	304
NM_004890	-0.2069888	-0.7924679	0.75406513	0.003069432	2559	3	321
NM_030969	-0.6089627	-0.6653674	0.956963451	4.44E-06	2738	3	745
NM_017829	-0.1000383	-1.0066574	0.753088832	0.00102046	2052	3	145
NM_017829	0.16094533	-0.940123	0.753088832	0.00135492	2890	3	435
XM_495908	-0.7200231	-1.1735829	1.059109753	0.045633146	1700	3	1593
NM_018217	-0.3707793	-1.172396	1.027355766	2.27E-05	1807	3	1232
NM_015412	0.07581648	-1.252062	1.075428251	0.001694134	4085	3	1163
NM_003778	0.34160895	-0.9920323	0.789805937	0.000179197	4171	3	970
NM_003778	0.02142518	-1.3308221	0.789805937	0.001986781	3728	3	829
NM_005533	-0.3073837	-1.3938775	1.014010483	2.18E-07	3820	3	689
NM_004635	-0.0217274	-1.0336044	0.865664529	0.000906412	2368	3	419
NM_004635	-0.2123247	-1.0463545	0.865664529	0.000358147	3837	3	126
NM_022917	-0.4688619	-0.9492315	0.864417694	0.000805416	1805	3	1339
NM_080422	-0.0256141	-1.1280902	0.878747822	0.004016294	3730	3	748
NM_080422	0.00904649	-0.9288906	0.878747822	0.000497564	2884	3	317
NM_080422	0.18626729	-0.950296	0.878747822	0.0036349	2945	3	811
NM_004418	0.48447508	-1.3719484	1.008397554	9.54E-05	606	3	1428
NM_004418	-0.0860224	-1.0812055	1.008397554	2.76E-05	3700	3	518
NM_031286	-0.7762123	-1.1745289	1.058540721	0.000165813	3954	3	1061
NM_000314	-0.0298293	-1.2484072	0.999839873	0.002269877	4030	3	1138
NM_078483	-0.6720825	-1.0821676	1.135418183	0.001857068	267	3	1571
CO582842	-0.4368992	-1.2094746	1.005989866	0.000194895	3832	3	519
CO582842	-0.387331	-0.9616773	1.005989866	0.000338414	3682	3	347
NM_003549	-0.1718728	-0.9116216	0.634647487	0.00963327	2557	3	263
NM_003549	-0.3835098	-0.8106469	0.634647487	0.018679912	2474	3	730
NM_003365	-0.5767128	-0.7582497	0.937140333	0.001471229	1806	3	1418
NM_005744	-0.1265998	-0.6926136	0.622619572	0.014331765	2478	3	685
NM_003358	-0.3428922	-1.1338462	1.245496485	0.004092995	700	3	1396

NM_001688	-0.4148805	-0.6272503	0.810388084	0.020828488	2727	3	835
NM_002689	-0.4302534	-0.6923973	0.913372347	0.000406608	2734	3	413
NM_000883	-0.3289195	-1.4307913	0.898908773	0.000312256	3639	3	772
NM_000883	-0.3264127	-1.1873869	0.898908773	0.000190354	3833	3	212
NM_014481	0	-1.1308295	1.168957856	0.004149899	4032	3	1284
NM_006347	-0.0542073	-0.8463582	0.850650091	0.003437687	2369	3	539
NM_006347	0.0221689	-1.4432794	0.850650091	0.000106331	3727	3	796
NM_015074	-0.6517049	-0.644255	0.623500904	0.011540303	1406	3	1239
NM_002807	-0.3895529	-1.3856021	0.857908357	0.011030282	3588	3	1280
NM_014521	-1.3896069	-0.3045934	0.923145165	0.00076147	167	3	1618
NM_014521	-1.5565257	-0.2665764	0.923145165	0.001178055	164	3	1640
NM_000079	0.11791135	-1.0927359	0.679089642	0.00326652	2366	3	534
NM_172089	0.17353456	-1.1720088	0.92798986	0.000682295	3755	3	795
NM_172089	0.09407736	-0.9418162	0.92798986	0.000445817	2871	3	652
NM_019088	-0.0227685	-1.0172667	0.714246684	0.017181306	2358	3	925
NM_024959	-0.8394708	-1.3013185	0.81555972	8.31E-05	1440	3	1046
NM_024959	-0.3191745	-1.0874997	0.81555972	0.000450436	3644	3	245
XR_012243	-0.9641882	-0.5766885	1.025822595	0.013441996	4384	3	1452
NM_001514	-0.1766329	-0.7874165	0.460971382	0.02755069	2549	3	315
NM_144566	-0.0057371	-1.5284593	0.81122944	0.002090702	3772	3	1156
NM_144566	0.06752113	-0.8903693	0.81122944	0.002826818	2370	3	688
NM_017899	-0.2831934	-1.3012405	0.510374797	0.006711782	2303	3	764
NM_017899	-0.047393	-1.1902438	0.510374797	0.008458149	2304	3	732
NM_032505	0.5070897	-1.3225049	0.737144354	0.023482929	5087	3	1623
NM_032505	0.20219295	-0.9755018	0.737144354	0.017849417	4162	3	1128
NM_032309	-0.5318753	-1.0954503	0.510682991	0.007060195	1389	3	1199
NM_032309	-0.3643674	-0.9185664	0.510682991	0.007451501	2315	3	409
NM_013238	-0.7403477	-1.1548282	0.982600884	3.66E-05	3951	3	741
NM_013238	-0.6033258	-1.1620497	0.982600884	0.000503162	3824	3	873
XR_014011	-0.1551559	-0.8408213	0.876225901	0.004400045	2768	3	361
NM_015009	-0.1358199	-0.8097024	0.53900495	0.022487782	2552	3	459
NM_138440	0.06283318	-1.3249736	1.014835994	6.22E-05	4167	3	905
NM_138440	0.07518045	-1.0814535	1.014835994	0.000126204	4174	3	867
NM_013248	0.35415988	-1.2437067	1.115011914	0.000719445	620	3	1557
NM_052850	-0.1759824	-1.1505145	1.084218019	0.003687344	4039	3	1102
NM_004309	-0.6922584	-1.4866671	0.939796317	5.60E-05	3941	3	1073
NM_004309	-0.7114613	-1.4556923	0.939796317	5.03E-05	3942	3	1053
CN801916	-0.1607867	-1.152556	1.210826885	0.001036593	3874	3	1084
NM_001102	-0.2210835	-1.3384134	0.882531907	0.000125822	3769	3	607
NM_001102	0.17596728	-1.3253242	0.882531907	0.000158789	4166	3	865
NM_152994	-0.4029432	-1.0384871	1.032923023	0.022467333	3899	3	1327
NM_152994	-1.9561747	-0.746126	1.032923023	0.021924567	155	3	1666
NM_021173	-0.3949382	-1.0997607	1.063643736	6.71E-06	3688	3	513

NM_021173	-0.3490387	-1.204469	1.063643736	1.36E-05	3835	3	566
NM_004281	-0.213158	-1.1925705	1.173713752	0.003599625	699	3	1263
NM_014764	-0.5768621	-0.7372027	0.984575363	0.00111146	1721	3	667
NM_014764	-0.6294725	-0.8183118	0.984575363	0.00602852	3591	3	1183
NM_000373	-0.4968161	-0.5709109	0.803105967	0.000915134	2419	3	418
NM_000373	-1.2232709	-0.2266999	0.803105967	0.004578554	161	3	1636
NM_130459	-0.7469642	-0.9079164	1.087404959	0.00361895	3583	3	1336
NM_130459	-0.511728	-0.8254858	1.087404959	0.004651886	3584	3	1086
XM_290820	-0.7253317	-1.384091	0.433753827	0.029683892	1644	3	1420
XM_290820	-0.296851	-1.8043612	0.433753827	0.011099206	1574	3	1480
CK231506	-0.2456594	-1.0188307	1.037304328	0.00010916	3689	3	515
NM_145654	0	-0.9055589	0.915970885	0.004357851	2371	3	710
AI913343	-0.3998805	-0.8594728	1.067037532	0.009591163	1813	3	1245
AI913343	-0.2703306	-1.307318	1.067037532	0.000334354	3834	3	819
NM_152444	-0.0530488	-1.6329507	0.591041768	0.02276411	1687	3	1483
NM_152444	-0.324501	-0.990351	0.591041768	0.041950043	2548	3	949
NM_005619	-0.5801105	-0.6519746	0.78972807	0.025677773	1803	3	1441
NM_020362	-0.79454	-0.6069242	0.860696951	0.006176432	215	3	1595
NM_013314	-0.3995092	-1.046426	1.192126199	0.004629073	1423	3	1576
NM_003977	-0.3946905	-0.7714229	0.654449481	0.029329401	3471	3	758
NM_003977	-0.3768187	-0.9639754	0.654449481	0.011242407	2314	3	477
NM_002156	0.1002766	-1.1596116	1.018424438	0.007595708	4058	3	1216
NM_002156	-0.0537778	-1.1483767	1.018424438	0.02028316	4057	3	1338
NM_002577	-0.159516	-1.2999169	0.73724233	0.00093206	3770	3	491
NM_016021	-0.2935389	-1.2978297	1.124424253	0.000901377	3781	3	1161
NM_020706	-0.4267355	-0.6778934	0.904605603	0.0021971	1737	3	861
NM_020706	-0.8547034	-0.531826	0.904605603	0.01037596	4734	3	1438
NM_144564	-0.4579117	-1.095236	0.885079801	0.00335798	3628	3	763
NM_024747	-0.051624	-1.1480186	0.987314416	7.59E-05	3679	3	597
NM_024747	0.07805109	-1.1497612	0.987314416	0.000805972	4068	3	910
XM_934556	-1.0234341	-1.1988384	0.819718739	0.004030981	3576	3	1467
XR_012665	-0.5180076	-1.1909978	1.138538028	1.46E-07	3956	3	789
XR_012665	-0.4206698	-0.9371076	1.138538028	0.000172987	3683	3	679
NM_006196	-0.7230101	-0.9992585	1.068288189	0.00017858	3952	3	906
NM_006196	-0.7909737	-1.3476009	1.068288189	0.001754935	3587	3	1366
NM_001659	-0.0026007	-1.0162382	1.034902881	0.000304684	4230	3	721
XR_013529	0.19707987	-1.1690388	1.110720268	2.59E-05	4232	3	1054
NM_178819	-0.1611739	-0.9091938	0.997880075	0.001887061	3910	3	800
NM_178819	-0.8750502	-0.7621469	0.997880075	0.008935145	1718	3	1295
NM_014244	-0.3910781	-1.0445958	1.024269	0.000219968	3681	3	526
CK232214	-0.7334472	-1.1140016	1.207778827	0.002397485	3960	3	1357
NM_175063	-0.5732101	-0.6527882	0.792048508	0.003555525	2418	3	637
NM_175063	-0.9665596	-0.4862156	0.792048508	0.047969004	1752	3	1513

NM_004526	0.28453884	-0.9712592	0.765092979	0.025738477	5088	3	1494
DQ266251	-1.1550059	-0.9354218	1.212536107	0.005153243	266	3	1613
NM_052965	-0.0660541	-0.879478	0.613667806	0.021339789	2551	3	604
NM_152759	0.15154787	-1.6078612	0.372479433	0.015011087	1640	3	1465
NM_152759	-0.2191377	-1.3448495	0.372479433	0.021773706	2302	3	1159
NM_003000	-0.192655	-1.050286	1.08273054	9.90E-05	4229	3	610
NM_015942	-1.1093703	-1.5119153	0.788262347	0.006894569	1376	3	1587
NM_003902	0.14509304	-1.0656231	0.939622835	0.000268421	2882	3	664
NM_009587	-0.4783649	-1.4733678	0.932019478	0.000287174	3625	3	922
NM_009587	-0.5974739	-1.4827501	0.932019478	0.000703652	3624	3	1077
NM_009587	-0.4744263	-1.1090806	0.932019478	0.00037513	3677	3	451
NM_009587	-0.3486745	-1.0061311	0.932019478	0.000468297	3678	3	234
NM_003023	0.01142104	-1.2043777	1.144766531	0.009450278	1698	3	1449
CN646695	-0.439632	-0.9292691	1.189190617	0.001035846	4325	3	1041
CN646695	-0.8672816	-0.917039	1.189190617	0.000691294	3580	3	1401
NM_080588	-0.1364334	-1.0111764	1.149509742	0.000869783	4042	3	1002
NM_080588	-0.3835023	-1.2498533	1.149509742	0.000136802	3823	3	944
NM_004270	-1.6801059	-0.4590374	0.666057637	0.044351481	56	3	1697
CN648711	-0.4763156	-0.8828426	1.050018614	0.001924499	4123	3	754
NM_175748	-0.4248405	-0.9013324	1.077569481	0.00149931	1736	3	1219
XM_375359	-0.3351581	-1.0832856	1.146360683	0.003637669	3594	3	1275
CN804946	-0.5770502	-1.0982801	1.163472229	0.000490663	3971	3	1257
NM_014604	-0.5941352	-1.1301173	1.232899517	5.03E-05	3957	3	1100
AK128435	-0.4428453	-0.5507235	0.781659842	0.041575236	1731	3	1309
NM_001320	-0.3659593	-0.9722705	0.625568508	0.00981606	2310	3	284
NM_001320	-0.2813593	-0.9347718	0.625568508	0.012225845	2311	3	309
NM_018683	-0.3702953	-1.1661807	1.24445774	0.000848935	701	3	1145
NM_007033	-0.2943052	-1.1581455	1.226168372	0.003814024	3896	3	1318
CN641451	-2.7411714	-0.0497324	1.181235627	0.022078331	32	3	1693
NM_001005353	0.27548509	-0.933287	0.560544806	0.025019809	4573	3	1300
NM_001005353	0.29304032	-1.0684137	0.560544806	0.0135535	4572	3	1058
NM_000222	0.49927241	-1.602067	0.7426528	0.00308669	5105	3	1608
NM_000222	0.23669883	-1.2577988	0.7426528	0.003502082	4574	3	1432
NM_001731	-0.2891556	-1.1991463	1.153991727	0.000206923	3836	3	999
NM_001731	-0.2720368	-1.093847	1.153991727	0.002749718	702	3	1036
NM_003775	-0.1499351	-1.4446932	0.957774285	0.003209461	3790	3	1212
NM_003775	-0.1150566	-1.3401794	0.957774285	0.001572652	3791	3	945
XR_014799	-0.2856036	-1.1480202	1.053140957	0.001122063	3838	3	696
CN647420	0.15979959	-1.0321765	0.989839745	0.002304445	4183	3	1085
NM_138412	-0.3304623	-1.3528896	0.683313667	0.009036635	1651	3	1308
NM_138412	0.06744439	-0.9516177	0.683313667	0.034315663	1372	3	1551
NM_005472	-0.0629056	-1.5150229	0.812276122	0.004231925	3771	3	1255
NM_031466	-0.1514956	-0.9396697	0.993419621	0.000335138	3680	3	529

NM_005956	-0.3326592	-1.321318	1.070765347	0.001505557	3826	3	988
NM_020895	-0.1010376	-1.1705458	1.148369609	0.001427994	4041	3	1150
NM_174869	-0.3752796	-0.7889113	0.826223328	0.045035138	3472	3	1242
NM_174869	-0.7554498	-1.0942661	0.826223328	0.007027803	2289	3	1076
NM_174928	-0.0396939	-1.0816947	1.095102077	0.00198639	4025	3	1165
NM_174928	-0.9552134	-0.7602587	1.095102077	0.001462698	221	3	1603
XR_011100	-0.9262249	-0.7508634	0.876975437	0.016784281	1717	3	1351
XR_011100	-1.3152955	-1.0584867	0.876975437	0.011131912	1450	3	1561
A_01_P018736	-1.9330044	-0.7417675	1.173367188	0.04425524	41	3	1684
NM_017542	-0.1570584	-1.2389093	1.04348962	0.003268134	3671	3	1027
NM_017542	-0.396498	-0.9175048	1.04348962	0.006116486	3595	3	993
NM_000442	-0.3726494	-1.0900082	1.244914972	0.000237577	4121	3	1104
NM_000442	-0.542992	-1.0289937	1.244914972	0.000464921	3958	3	1105
NM_003077	-1.2871953	-1.0483074	0.579438078	0.034005011	1379	3	1659
NM_031208	-0.6575977	-0.9169917	0.862976616	0.019479103	2293	3	1231
NM_080612	-0.2656139	-0.9299011	0.987237199	0.035232787	4653	3	1416
NM_080820	0.00369009	-1.3094303	0.777279011	0.004774931	3714	3	895
NM_080820	0.17063683	-1.0124736	0.777279011	0.006190464	4170	3	977
NM_022748	-0.7148038	-0.4184883	0.833983021	0.015341583	1766	3	1486
NM_032343	-0.8142944	-1.2082015	1.121974732	0.000372896	3577	3	1507
NM_014350	-1.2363547	-1.0678548	1.189235461	0.002499857	220	3	1657
NM_006979	0.05314428	-1.039024	0.918501224	0.004927276	4158	3	854
NM_007263	-0.2371209	-1.0763852	0.826704009	0.003469972	2307	3	373
NM_007263	0.00456739	-0.7966954	0.826704009	0.006826919	4179	3	674
NM_183373	-1.0359873	-0.4468503	0.931705121	0.011449672	1733	3	1532
NM_183373	-0.0816523	-1.0229372	0.931705121	0.042943959	1697	3	1429
XM_928586	-0.0089637	-1.2705924	1.051374668	0.003056356	4037	3	1286
NM_006066	-0.3460533	-1.0496461	1.259203626	4.63E-05	4200	3	984
NM_031311	-0.6822415	-1.0356231	1.360137547	0.000616279	663	3	1364
NM_015603	-0.3746531	-1.3256343	1.007361862	0.01656672	1655	3	1518
NM_006384	-0.1922191	-1.1808425	1.163496462	0.001500531	4040	3	1119
XM_051271	-1.1293258	-0.5791007	0.915748784	0.02892635	214	3	1624
NM_001002234	-0.1345688	-0.9596475	0.982118596	0.004504495	4036	3	1010
NM_001773	-0.1880707	-0.820823	0.725047376	0.028125149	2550	3	785
NM_152905	-2.0943373	-1.0673212	0.708725178	0.034229052	61	3	1687
NM_020979	-0.5708321	-0.9024739	1.227716496	0.000899242	3973	3	1194
NM_018238	-1.7743473	-1.0218768	1.233797118	0.004432115	156	3	1673
NM_004359	-0.0350942	-1.6868196	0.724456319	0.017654102	1363	3	1553
NM_000377	-0.7465423	-1.5608459	0.646811486	0.025943657	1645	3	1542
NM_000377	-0.4326148	-1.5572876	0.646811486	0.012202312	1646	3	1316
NM_024611	-0.8855392	-0.7539341	1.010803174	0.046051647	47	3	1682
NM_020421	-0.1902973	-1.4144774	0.906134113	0.02176361	1652	3	1546
NM_020421	-0.5677029	-0.9444027	0.906134113	0.008871504	1811	3	1062

NM_002015	-0.0010839	-0.9618397	0.754941248	0.049616705	2553	3	1330
NM_012425	-0.0689459	-1.0294917	0.632225318	0.047967883	1371	3	1390
NM_002081	0.35635619	-1.2447886	1.110936995	0.00231442	651	3	1415
NM_002139	-1.5194326	-0.5733454	1.296116183	0.009803193	1751	3	1634
CB228320	-1.6466865	-0.4757539	1.336334852	0.005408627	168	3	1662
NM_001335	-0.6565796	-1.2188108	1.007970558	0.022355535	1367	3	1528
NM_001335	-0.6697835	-1.0734569	1.007970558	0.020705866	1808	3	1466
CN801997	-0.6640092	-1.171669	1.220768067	0.002401924	3581	3	1350
NM_003659	-0.6824239	-0.9167373	1.113185525	0.015096277	1810	3	1531
NM_005782	0.28269714	-1.3092368	0.762105079	0.016922163	1653	3	1471
NM_005782	0.70978428	-1.4816319	0.762105079	0.014026623	149	3	1650
NM_030579	-0.1840816	-1.0828361	1.083018008	0.01154416	1657	3	1472
NM_030579	-0.0060423	-1.3154319	1.083018008	0.004767109	3792	3	1302
NM_005219	-0.5328462	-0.8999471	1.188867775	0.002883024	3974	3	1271
NM_002638	0.24152065	-1.4364485	0.895324733	0.024777979	1656	3	1565
NM_031300	-0.0814392	-1.133267	1.224639521	0.024222096	3988	3	1543
XM_496355	-0.424213	-0.8400828	0.993246102	0.038431889	4819	3	1373
XM_496355	-0.7618428	-0.7960199	0.993246102	0.043148064	4816	3	1499
XR_012444	-0.5640653	-0.6582522	0.944884077	0.048021047	4818	3	1407
XR_012444	-0.4075744	-0.9648286	0.944884077	0.048079509	4817	3	1442
NM_018024	-0.2392234	-1.2184486	1.1205123	0.014295943	643	3	1476
NM_007207	-1.4980712	-1.0582788	1.058732752	0.028963595	1382	3	1671
NM_001001710	-0.4639798	-1.3992145	0.918927503	0.038971216	1370	3	1626
NM_003946	-0.2179418	-1.0092366	1.195982845	0.027206749	1368	3	1558
NM_001634	-2.284582	-0.6616352	1.280768143	0.049440299	36	3	1690

Cluster 2

Gene Name	Av Normal	Av M (w12)	Av M (w4)	*P*	Hierarchical Clustering (order)	K-means clustering	K-means clustering (rank)
XM_496306	0.17448229	-0.6756747	0.791719813	0.033584411	4361	2	1075
NM_004895	0.21631708	-0.712758	0.737007912	0.030499819	4369	2	1177
NM_020384	0	-0.306223	0.341989384	0.044281324	2979	2	1009
NM_004265	-0.2005715	-0.2217287	0.347884263	0.02352912	3090	2	1064
NM_000075	0.16894671	-0.4740377	0.51097132	0.044522226	2396	2	1046
NM_000075	0.66541407	-0.3448736	0.51097132	0.020514066	3353	2	1358
NM_016047	-0.0200933	-0.4726091	0.523883814	0.030080975	2978	2	740
NM_178422	0.56081246	-0.0980546	0.077106168	0.041312118	8913	2	1665
CN806498	0.10728253	-0.4340797	0.862128391	0.038429941	4366	2	577
NM_024510	1.43558112	-0.6057917	0.239073363	0.016082723	10874	2	1768
NM_004121	0.55664136	-1.0845047	0.939140938	0.03765647	1183	2	1707
NM_001191	0.51554572	-0.9904445	1.331778136	0.037915375	1187	2	1725
NM_001191	0.36406456	-1.0587417	1.331778136	0.045551028	1189	2	1729
XR_011953	-0.0775206	-0.0869465	0.316837503	0.04737296	3103	2	1200
NM_006170	-0.2660834	-0.2878404	0.464747051	0.011320011	3089	2	765

NM_005188	-0.1078098	-0.8979374	0.974684262	0.027532873	4618	2	1512
NM_001458	0.26583656	-0.2518273	0.970526311	0.047624861	4376	2	697
NM_002213	0.12530471	-0.188325	0.271040011	0.039837035	3149	2	1245
NM_015721	-0.0826009	-0.1976658	0.348291892	0.037294103	3110	2	1071
NM_020186	0.17920788	-0.4539064	0.422807803	0.006331458	3341	2	997
NM_020183	-0.114332	-0.5779577	0.889598056	0.032232652	4358	2	1050
NM_020183	-0.1077511	-0.277456	0.889598056	0.041005456	4360	2	690
NM_014216	-0.1050453	0.04694231	0.29071323	0.04819872	3105	2	1318
AB037781	-0.0081114	-0.3066528	0.53022362	0.013175657	3112	2	527
NM_003123	-0.0481336	0.02327323	0.176470462	0.001062295	3104	2	1476
NM_006816	0.00197847	-0.1539533	0.20716964	0.01429901	3101	2	1307
NM_012117	-0.4033656	-0.5606853	0.842821552	0.030189101	1716	2	1638
NM_031262	0.39766902	-0.5120682	0.576494514	0.010289039	3342	2	882
NM_031262	0.47496596	-0.1512269	0.576494514	0.043614512	3355	2	1055
NM_145214	0.27144707	-0.2624655	0.200860908	0.006924294	3150	2	1381
NM_001348	-0.1087892	-0.147189	0.688198913	0.043344941	3187	2	626
NM_181985	1.78563174	-0.6810607	0.399393933	0.008381982	10870	2	1783
NM_014230	0.052894	-0.020365	0.362532166	0.043669908	3106	2	1160
AK124768	0.4162427	-0.1690329	0.330902172	0.021318247	3346	2	1344
NM_004551	0.17368318	-0.3765058	0.345132107	0.006444698	2176	2	1022
NM_004551	0.47727056	-0.4003844	0.345132107	0.005079008	3349	2	1295
NM_003530	1.41146927	-0.7751416	0.150751058	0.007303002	10869	2	1777
NM_033423	0.24615526	-0.2977248	0.385573242	0.020675816	3140	2	1063
NM_033423	0.20754603	-0.2136639	0.385573242	0.016092264	3141	2	952
NM_032340	-0.0128773	-0.424837	0.720375183	0.015635394	2684	2	333
NM_032340	-0.3446658	-0.4896915	0.720375183	0.011789402	2680	2	505
XR_012618	0.11870457	-0.2098659	0.176745576	0.000208892	3102	2	1337
NM_032636	0.20633433	-0.9031484	0.83824797	0.005111755	4083	2	1067
NM_145212	0.06541977	-1.0474246	1.3112801	0.035579104	4619	2	1711
NM_006254	-0.1458299	-0.6075985	0.803238256	0.031970394	4621	2	1147
NM_007096	0.31508559	0.02888056	0.277167915	0.005557792	3180	2	1403
NM_080821	-0.0885189	-0.3189381	0.472245535	0.049867065	3071	2	988
NM_006819	-0.0282743	-0.4941414	0.870088483	0.017654194	4362	2	294
NM_006819	0.15974583	-0.1576888	0.870088483	0.044646859	3323	2	498
XR_013954	0.13701453	0.1114304	0.428609972	0.027700418	3176	2	1199
NM_019555	0.07644108	-0.3382368	0.478477815	0.01302298	3138	2	650
NM_004322	-0.3295908	-0.2835521	0.552758996	0.038106933	2512	2	921
NM_004322	-0.1290578	-0.2148904	0.552758996	0.019099018	3111	2	564
NM_005985	0.35818039	-0.4376489	0.34207124	0.001564863	3338	2	1331
NM_182943	-0.2018271	-0.2523368	0.502418539	0.022008733	3093	2	676
NM_182943	0.46601533	-0.0816863	0.502418539	0.035828059	3375	2	1466
NM_000956	0.46788796	-0.1376649	0.723797083	0.038778389	3324	2	878
NM_001848	-0.378931	-0.0362931	0.432106756	0.020792436	2525	2	1208

NM_017593	-0.3395568	-0.6321275	0.885075999	0.020064255	229	2	1776
NM_005610	0.08288393	-0.5217705	0.499367711	0.03613237	2974	2	906
NM_001262	-0.1421073	-0.6691037	0.80027583	0.009759	2863	2	534
NM_000516	-0.4128336	-0.00061	0.205474878	0.025172057	2506	2	1521
NM_024515	0.30549719	-0.3292266	0.445829133	0.001566037	3158	2	857
NM_024515	0.55988749	-0.3287718	0.445829133	0.003646334	3350	2	1286
NM_015629	0.33980695	-0.2351959	0.521238194	0.028780636	3166	2	849
NM_015629	0.31611729	-0.2566312	0.521238194	0.013245735	3165	2	718
NM_016053	-0.0448165	-0.1734865	0.64208335	0.033847443	3227	2	369
NM_032366	-0.0563872	-0.390511	0.485182894	0.037365079	2397	2	916
XR_009684	1.21203392	-0.1316228	0.267465735	0.044140599	10855	2	1790
NM_002414	0.21492967	-0.0585331	0.398027824	0.004877267	3173	2	1037
NM_014999	-0.2468613	-0.2877167	0.578004289	0.048364027	2689	2	1121
NM_024551	0.50690112	-0.4427747	0.455833775	0.011538377	3348	2	1166
NM_024551	0.38201737	-0.2292167	0.455833775	0.005061489	3161	2	933
NM_130782	-0.2261729	-0.4597517	0.978890564	0.017304929	4359	2	488
NM_003333	-0.1050292	-0.1610457	0.246839766	0.00124263	3099	2	1235
NM_139286	0.27928227	-0.3920022	0.340633185	0.006014262	3344	2	1109
NM_139286	0.278199	-0.2546768	0.340633185	0.027983155	3345	2	1129
XM_045423	-0.3058804	-0.3967654	0.967129885	0.028037849	4622	2	1148
NM_002717	-0.2272695	-0.1808082	0.521565917	0.016646199	3094	2	619
NM_014403	0.13160389	-0.3896345	0.319746375	0.014629954	3151	2	1032
NM_014403	-0.0968495	-0.2198075	0.319746375	0.006148636	3098	2	1001
NM_014403	0.0670323	-0.221439	0.319746375	0.007937282	3156	2	1040
NM_052848	0.81985542	-0.1781692	0.234816353	0.044752248	8914	2	1681
NM_016399	0.27973716	-0.8495864	0.7572921	0.043382222	4568	2	1499
NM_018641	0.07168525	-0.3194446	0.582368576	0.023141088	2398	2	557
NM_017971	0.63584948	-0.5217275	0.574424152	0.002090108	3352	2	1223
NM_017971	0.43250283	-0.4777844	0.574424152	0.004647823	3351	2	798
NM_007080	0.395258	-0.22857	0.555998954	0.047172276	3356	2	1181
NM_000431	0.15319086	-0.0447757	0.518119273	0.009773746	3175	2	746
NM_000431	0.32523389	-0.0327273	0.518119273	0.013650519	3374	2	1161
NM_019056	-0.0060345	-0.2858646	0.304094426	0.000141801	2171	2	1005
NM_015004	0.32084729	-0.3314576	0.332635104	0.016822641	3152	2	1070
NM_030628	0.41036955	-0.6459932	0.552908397	0.008893932	2408	2	1269
NM_030628	0.40183125	-0.5824657	0.552908397	0.000831194	2995	2	794
NM_025195	0.16484614	-0.3680396	0.399979651	0.038656964	2975	2	971
NM_014169	-0.1707716	-0.4849437	0.631054017	0.007625642	2646	2	377
NM_178507	-0.4256139	-0.6937941	0.951072406	0.041748174	4635	2	1361
CO647725	-0.9082586	-0.3517326	1.610573804	0.044365688	5119	2	1714
CO648602	0.112569	-0.8622292	1.058653658	0.020320035	4616	2	1543
CO648602	0.58672756	-0.343646	1.058653658	0.037899127	5075	2	1668
NM_012105	-0.0335146	-0.3035833	0.349874709	0.022838303	3135	2	948

CN642651	-0.1296686	-0.1148618	0.684758437	0.013687099	3212	2	361
CN642651	-0.2508894	-0.025222	0.684758437	0.020830914	3210	2	554
DV769745	-0.1338515	-0.1295373	0.301604623	0.001390323	3100	2	1143
NM_017694	-0.6506833	-0.101794	0.489688522	0.042945688	1743	2	1656
NM_005371	0.45071095	-0.6172772	1.261190025	0.016226947	4380	2	1412
NM_005873	0.16420856	-0.5663389	0.503505035	0.0077607	2208	2	960
NM_016196	0.43291585	-0.8987585	0.908334822	0.029306374	1184	2	1568
NM_002185	0.22696541	-0.6293393	0.814427005	0.01873168	3040	2	755
XR_010574	-0.0340304	-1.0386825	1.236107016	0.01309837	4100	2	1577
NM_004492	0.21041318	-0.4795735	0.657509052	0.040228182	3567	2	1564
NM_004492	0.01133696	-0.3571938	0.657509052	0.03502091	3321	2	530
NM_030809	-0.15322	-0.1001424	0.793065285	0.022880619	3530	2	307
NM_016129	0.13484414	-0.7469114	0.980493713	0.0325131	3034	2	1263
NM_020365	0.12215309	-0.1770999	0.48702958	0.019313904	3074	2	814
NM_001009566	-0.0660885	-0.3527757	0.533097883	0.008291768	3186	2	661
NM_004381	0.15562137	-0.5065127	0.709741866	0.002918186	2866	2	600
NM_004381	0.15050899	-0.3257316	0.709741866	0.009430498	3010	2	133
NM_014462	0.24925992	-0.5159758	0.651780749	0.017782286	3017	2	578
NM_021626	-0.2052331	-0.4704552	0.763943471	0.004678382	2708	2	266
NM_014012	-0.4151274	-0.0721178	1.104390799	0.038095228	3554	2	1261
NM_025029	0.23779679	-0.0909489	0.385422554	0.001129456	3172	2	1024
NM_025029	0.22919933	0.04977622	0.385422554	0.015404002	3177	2	1226
NM_005739	-0.1696213	-0.5197134	0.848910385	0.033957878	1746	2	1627
NM_016947	-0.3888967	-0.2848464	0.50160887	0.002346471	2509	2	927
NM_006281	-0.2826918	-0.2870744	0.619574819	0.01135064	2487	2	748
NM_006281	-0.7329159	-0.2127416	0.619574819	0.021906025	2491	2	1482
CK231237	-0.3184001	-0.1220853	0.417345607	0.036521284	2527	2	1153
CK231237	-0.3569147	-0.012848	0.417345607	0.001304685	2526	2	1176
NM_182764	-0.2371532	0.12094311	0.573364262	0.01357286	3211	2	963
NM_012154	-0.3469536	-0.3037943	0.591820354	0.003161891	1799	2	1224
CO647692	0.56988895	-0.5349162	0.324205605	0.013293039	2235	2	1457
CO647692	0.23471575	-0.2639389	0.324205605	0.048211586	3153	2	1157
NM_005614	0.08752562	-0.2009668	0.544900053	0.0107043	3157	2	566
NM_005375	-0.0244242	-0.4174468	0.62817846	0.028043076	2983	2	424
NM_005375	0	-0.141761	0.62817846	0.03408059	3228	2	421
XM_376724	0.14024124	-0.7442127	0.77534552	0.002095636	2904	2	368
CB230918	-0.0273441	-0.4700408	0.525865598	0.049311127	2394	2	1042
CO725888	0.03440038	-0.6459723	0.966878392	0.017259491	4363	2	747
CO725888	0.04450926	-0.3626899	0.966878392	0.04039769	4364	2	790
NM_018077	0.47325081	-0.8791704	1.073595245	0.047713137	4642	2	1715
NM_080549	-0.2036832	-0.453499	0.792359543	0.009302309	2626	2	253
NM_003528	-0.3107993	-0.6148015	0.940147852	0.00694795	2681	2	480
NM_003528	-0.5459278	-0.5385035	0.940147852	0.013052624	1715	2	1262

NM_139201	-0.0325914	-0.6206033	0.673320539	0.029670138	1790	2	1038
NM_004147	0.12261216	-0.4570919	0.72571466	0.003432374	3009	2	80
NM_004147	0.24743177	-0.2879395	0.72571466	0.003262604	3012	2	139
NM_013334	1.13126057	-1.3002841	1.263284126	0.019118107	5095	2	1763
NM_013334	1.05643782	-0.7903095	1.263284126	0.036285767	5096	2	1737
NM_002431	-0.0848382	0.06594143	0.604932478	0.02871561	3222	2	750
NM_032361	-0.3858714	-0.6711261	1.462925805	0.026442571	1229	2	1591
NM_032361	-0.3034525	-0.6444409	1.462925805	0.042283946	1228	2	1645
NM_198324	-0.1027095	-0.3724671	0.632918616	0.004853442	2647	2	178
NM_001904	0.14973465	0.0116986	0.620368561	0.022696329	3250	2	722
NM_001904	-0.0758078	-0.281688	0.620368561	0.038917819	2985	2	392
XR_013652	0.26892896	-0.5459551	1.159492832	0.0141703	4368	2	1025
NM_019018	-0.0318901	-0.4869377	1.269489526	0.023892073	4528	2	1231
NM_000060	0.35598866	0.04888425	0.964371145	0.04521663	3390	2	1214
CO583502	0.27057015	-0.9005241	0.966916611	0.007676275	4062	2	1251
CK231625	0.18271518	-0.1617446	0.486509158	0.000191776	3170	2	616
CK231625	0.32969505	-0.0899386	0.486509158	0.000125591	3164	2	901
CB549498	0.22861976	-0.1661881	0.551987881	0.001657317	3174	2	501
CB549498	0.18086193	0.00876871	0.551987881	0.005592344	3179	2	719
CB549630	-0.3107772	-0.0855762	0.373295449	0.007868089	2523	2	1187
NM_002997	-0.3127323	-0.2488761	0.533115885	0.017897254	2513	2	941
NM_002997	-0.5398863	-0.0541244	0.533115885	0.004157497	230	2	1759
NM_002796	0.47540039	-0.2866697	0.563984654	0.016352382	3354	2	934
NM_021008	-0.1779968	-0.5364762	0.638436626	0.004482461	2641	2	523
NM_021008	0.06441446	-0.2509935	0.638436626	0.007648986	3198	2	347
NM_015139	0.20524457	-0.075909	0.395403529	0.030474615	3075	2	1230
NM_006427	0.04691653	-0.7520143	0.790969084	0.001179715	2663	2	493
NM_006513	-0.4701522	0.08577783	0.499071943	0.010403739	2535	2	1290
BX648585	-0.1250883	-0.2663943	0.340937641	0.036128638	3136	2	1081
NM_002882	0.21420185	-0.1626968	0.338082347	0.007400833	3167	2	1142
NM_007253	0.30344846	-0.0344534	0.367771958	0.015478969	3181	2	1255
NM_181336	-0.161467	-0.2945624	0.550481139	0.002378326	3092	2	401
NM_181336	-0.0459745	-0.2255636	0.550481139	0.000603442	3124	2	278
CN648534	0.34585088	-0.5288181	0.486498732	0.002674118	2994	2	833
CN648534	0.24357172	-0.5248588	0.486498732	0.008874588	2993	2	749
NM_144721	-0.7037958	0.17930016	0.484356667	0.026013698	1745	2	1700
NM_024544	0.20761447	-0.4809919	0.466663953	0.004112104	2992	2	668
NM_024544	0.14332614	-0.2508616	0.466663953	0.013133489	3139	2	841
XR_013508	-0.3146589	-0.2346067	0.798134156	0.029978651	2710	2	705
NM_025146	0.14475334	-0.66457	0.674649602	0.002929622	2915	2	316
NM_006278	0.16177899	-0.7148635	0.708496515	0.001337773	2913	2	336
NM_006278	0.04816303	-0.4793777	0.708496515	0.0101903	3004	2	143
NM_144498	-0.8062035	-0.2269392	0.931105778	0.049563199	3493	2	1535

NM_002714	-0.0188803	-0.3928407	0.493002119	0.00351891	3114	2	537
NM_002714	-0.0322615	-0.2594505	0.493002119	0.010504095	3115	2	594
NM_022121	-0.0175004	-0.0177991	0.480430713	0.019847928	3107	2	843
NM_022121	-0.0558511	-0.2513331	0.480430713	0.003235458	3116	2	529
XR_012175	-0.1836574	-0.2379674	0.597815799	0.035551134	2464	2	648
NM_015200	0.03223224	-0.882245	0.890316184	0.007827551	3030	2	1034
NM_003457	0.24122398	-0.0743503	0.749538823	0.024536283	3268	2	865
NM_017840	0.35908482	-0.2017404	0.783659004	0.011103767	3277	2	397
NM_002375	-0.0533189	-1.0958065	1.300254664	0.016716749	1205	2	1621
NM_153211	1.98158246	-0.8746532	1.425415972	0.03275298	5275	2	1785
NM_003801	-0.3069058	-0.0208569	0.560326914	0.022534429	2528	2	964
NM_003801	-0.4640086	-0.1004209	0.560326914	0.000659013	2533	2	926
NM_003321	0.01662702	-0.0783964	0.647161451	0.008574192	3229	2	288
NM_003321	-0.0564242	0.09421867	0.647161451	0.028161954	3215	2	658
NM_012321	-0.0064127	-0.3499746	0.50001588	0.003331373	3118	2	458
NM_012321	-0.0328777	-0.3157053	0.50001588	0.002298778	3119	2	474
NM_014453	0.09623656	-0.0958935	0.460631463	0.000378473	3169	2	680
NM_014453	0.14697876	-0.2612548	0.460631463	0.023381286	3144	2	681
XR_009776	-0.000773	0.11541571	0.680252293	0.022624189	3217	2	657
NM_014878	0.0844475	0.07679435	0.554239034	0.024007405	4683	2	1294
A_01_P016989	-0.2430771	-0.3867495	0.763417976	0.013672723	2683	2	407
NM_014063	0.04096683	-0.2462671	0.494892092	0.03392619	3109	2	903
NM_005113	-0.0849114	-0.2991327	0.622927799	0.008013909	2865	2	977
NM_207327	-0.1050538	-0.5421717	0.590989821	0.033492523	2485	2	1085
NM_004552	0.45213942	-0.3583763	0.413514749	0.007317176	3347	2	1122
NM_004552	0.34797422	-0.0866564	0.413514749	0.013309129	3182	2	1103
NM_002539	0.40916092	-0.8445988	0.768910228	0.005599122	2858	2	1225
NM_002539	0.5807513	-0.8079959	0.768910228	0.023851545	3060	2	1465
NM_013321	0.34765748	-0.8599201	0.978969763	0.010818784	4641	2	1364
NM_012111	0.22714104	-0.2432009	0.474960731	0.00836873	3155	2	716
NM_012111	0.08128322	-0.1812358	0.474960731	0.011574514	3145	2	589
NM_017923	-0.2789974	-0.6554443	0.762715285	0.007613412	2642	2	526
NM_016050	-0.3101706	-0.2651998	0.530463524	0.001492572	3091	2	581
CN642478	0.15277479	-0.3393176	0.714640854	0.03542334	3016	2	457
NM_001968	-0.668401	-0.4068186	0.805309188	0.043094515	1729	2	1605
A_01_P018842	0.01819529	-0.903778	1.169688735	0.039174222	4639	2	1646
XR_011934	-0.1745404	-0.2238206	0.668568453	0.014269013	3129	2	330
XR_011934	-0.1021637	0.07925231	0.668568453	0.019091427	3221	2	689
NM_212472	0.17683721	-0.425149	0.373299153	0.043445814	2123	2	1100
XR_011873	0.08545886	-0.4294494	0.696402852	0.038876511	3337	2	759
NM_006591	0.36887193	-0.6665813	0.614088346	0.026866331	2271	2	1156
NM_018321	0.20170815	-0.8039326	0.810123536	0.025237173	3047	2	1502
NM_138801	-0.1003327	-0.1576005	0.499105151	0.024374952	3142	2	691

NM_032830	0.17343572	-0.0869905	0.69031086	0.005570202	3248	2	386
NM_032830	0.22580364	-0.0561362	0.69031086	0.007913817	3249	2	372
XM_372124	0.67557776	-0.0606606	0.510768549	0.004286437	3370	2	1391
XM_372124	0.49670094	-0.1127443	0.510768549	0.02787595	3365	2	1167
NM_005829	0.28677786	-0.7634094	0.65564737	0.01974638	3027	2	1426
NM_003849	-0.0136881	-0.1434904	0.668213955	0.015041046	3231	2	264
NM_003849	-0.0303509	0.16463957	0.668213955	0.041330131	3216	2	817
NM_015463	0.61890062	-1.0010267	0.952819367	0.012715862	4063	2	1496
NM_014239	-0.1264258	-0.7075472	1.113150766	0.022671017	1424	2	1510
NM_005880	0.44937494	-0.6158905	0.686252763	0.000883108	3051	2	1312
NM_015294	0.02686488	-0.3161843	0.675538132	0.014740691	3322	2	570
NM_145051	-0.0342691	-0.3659585	0.473176751	0.041591676	3108	2	1096
NM_015079	0.1877364	-0.8587151	0.866106008	0.00124591	4084	2	640
NM_004247	0.16814557	-0.0516638	0.363981133	0.025018069	3168	2	1124
NM_015600	0.34921332	-0.3916101	0.617367414	0.012756834	3018	2	667
NM_003669	-0.0467242	-0.3406629	0.419402541	0.033725832	3113	2	979
NM_018009	-0.1817006	-0.3750165	0.591753332	0.04106737	2691	2	890
NM_006493	-0.2826659	-0.7339686	1.0480738	0.036299783	4620	2	1597
NM_012254	0.0222978	-0.5724358	0.671064679	0.002080197	2982	2	241
AK055375	0.05028389	-0.1796842	0.30924737	0.0457661	3077	2	1296
NM_015426	0.33383938	-0.7533266	0.99157691	0.004064181	4370	2	729
NM_019896	0.03316291	-0.2192989	0.528920071	0.0451825	3143	2	593
NM_016548	-0.1893267	-0.1091539	0.714698664	0.048828583	3063	2	776
NM_024839	0.08212534	-0.2349305	0.566536834	0.000454279	3125	2	292
NM_003454	-1.0305088	-0.1509424	0.668459625	0.046546054	48	2	1794
NM_003454	0.91446184	-0.5210628	0.668459625	0.007920153	4597	2	1524
NM_018466	0.05143772	-0.3441182	0.654243419	0.043021467	5089	2	1702
NM_032855	0.17214045	-0.6429978	0.72551027	0.000639158	2664	2	610
NM_006135	-0.0620206	-0.1679831	0.410009649	0.024229213	3137	2	844
NM_006135	-0.2363106	-0.0329186	0.410009649	0.045967829	2542	2	1087
NM_015646	-0.267902	-0.6207776	0.87104696	0.008309663	2862	2	580
NM_015646	0.12528591	-0.4233724	0.87104696	0.007376454	3011	2	43
CN644199	-0.3751762	-0.2507925	0.643909004	0.024616539	2486	2	876
NM_003379	-0.0098717	0.27355877	0.51525617	0.028057071	3224	2	1274
NM_003379	-0.0464305	0.07872764	0.51525617	0.043818648	2543	2	912
NM_014571	0.2067705	-0.6480694	0.777393382	0.004173515	2924	2	317
NM_014571	-0.142529	-0.5804551	0.777393382	0.006978844	2661	2	450
NM_018197	0.05520292	-0.2382978	1.549394535	0.04972836	240	2	1723
XM_496288	-0.2590705	-0.4920144	0.733956938	0.00057446	2627	2	137
XR_010115	-0.706873	-0.0735384	0.472538491	0.0083542	2519	2	1470
NM_013277	-0.3293385	-0.2448124	1.017359987	0.014595178	3452	2	152
NM_017906	0.74767108	-0.4035957	1.475941055	0.02119655	5082	2	1590
NM_017906	-1.3945284	-0.2941231	1.475941055	0.043648021	236	2	1770

NM_013393	-0.1294282	-0.417841	0.72666429	0.006542001	3062	2	390
NM_013393	0.27094561	-0.1339302	0.72666429	0.007063026	3280	2	331
NM_004563	-0.363499	-0.4200827	0.983595433	0.007541976	2709	2	325
NM_000016	0.62225446	-0.1549388	0.799141132	0.011138415	3367	2	1060
NM_000016	0.38251506	-0.2728916	0.799141132	0.016031929	3019	2	443
NM_002080	0.3526342	-0.3501228	0.550078823	0.003716721	3160	2	643
NM_002080	0.11609946	0.11706892	0.550078823	0.001943685	3225	2	936
NM_019095	-0.1532967	-0.0268496	0.577908284	0.02974992	3220	2	806
NM_019095	-0.3105374	-0.4193747	0.577908284	0.047932388	2763	2	874
NM_002765	0.05858213	-0.5581007	0.790651149	0.003042945	2867	2	587
NM_002765	-0.4748281	-0.2951863	0.790651149	0.009317655	2699	2	623
NM_153812	0.07285152	-0.3903493	0.820909777	0.00964279	2695	2	122
NM_153812	-0.305843	-0.4949591	0.820909777	0.009180615	2707	2	454
NM_030981	0.11344627	-0.4805202	0.757881025	0.015096378	2400	2	417
NM_030981	0.07676128	-0.4822254	0.757881025	0.00956646	2399	2	280
NM_032901	0.00553211	-0.4007944	0.649239618	0.001408813	2981	2	89
NM_032901	0.02535039	-0.0653066	0.649239618	0.014238467	3240	2	300
NM_024901	-0.0689616	-0.4453888	0.70960087	0.009791119	3003	2	165
NM_024901	-0.2176558	-0.0202543	0.70960087	0.017669167	3213	2	447
NM_016183	0.09351944	-0.9832051	1.330559213	0.006964648	4097	2	1445
NM_003653	-0.0100619	-0.3330095	0.714812105	0.012191036	3072	2	481
NM_015055	-0.5563747	-0.1977828	1.335176781	0.022931497	4870	2	1530
NM_015055	-1.0021925	-0.134637	1.335176781	0.029932455	4676	2	1716
CO580490	0.37862207	-0.2778552	0.562004232	1.29E-06	3162	2	598
CO580490	0.32103853	-0.1915849	0.562004232	1.17E-05	3163	2	582
NM_006559	0.17484433	-0.0028039	0.685040271	0.017603979	3251	2	520
NM_014941	0.11936562	-0.3086403	0.48665222	0.000293818	3122	2	470
NM_015331	0.31722454	-0.5725303	0.443872132	0.002060684	2202	2	980
NM_001001521	0.29101599	-0.1627982	0.632416749	0.001227099	3279	2	539
XR_014082	0.13692408	-0.6442897	0.729215607	0.000254307	2901	2	146
XR_014082	0.29668236	-0.1511575	0.729215607	0.00167447	3267	2	634
NM_033542	-0.1580877	0.01086497	1.097498319	0.033264103	3533	2	931
NM_017491	0.75268894	-0.4787489	0.563487198	0.000330845	3359	2	1383
NM_017491	0.5329967	-0.0181767	0.563487198	5.87E-05	3369	2	1175
NM_017918	0.3748895	-0.607516	0.713714938	0.007241789	3021	2	823
NM_206918	-0.1459011	-0.4558856	0.766823378	0.012207833	2692	2	289
NM_005334	-0.0083066	-0.5463324	0.782738284	0.001517225	3005	2	47
NM_005334	0.01718103	-0.4901633	0.782738284	0.001406837	3006	2	27
NM_052813	-0.4495931	-0.305079	1.080162886	0.040383321	4422	2	1057
NM_052813	-0.329849	-0.2397117	1.080162886	0.049665954	4423	2	1048
NM_032182	-0.0502112	-0.209287	1.567350788	0.026809493	4453	2	1404
NM_032182	-0.0692947	-0.1408316	1.567350788	0.040986658	4456	2	1467
NM_001893	0.28749475	-0.577854	0.702149417	0.002334846	3022	2	398

NM_014046	-0.3080811	-0.1464439	0.478190677	0.040371513	2540	2	984
NM_177983	0.36164916	0.18559478	0.671943165	0.029670878	3292	2	1196
NM_177983	0.30723387	0.10206874	0.671943165	0.005559849	3297	2	913
NM_014699	-0.0415019	-0.6915682	1.406524123	0.024581611	1242	2	1588
NM_004846	-0.0263207	-0.546384	0.831010869	0.004117976	3007	2	68
NM_004846	0.0743501	-0.5251115	0.831010869	0.004181461	3008	2	54
NM_019862	-0.0443223	-0.4075409	0.91792293	0.045633459	4351	2	1053
NM_019862	0.15391381	-0.9627567	0.91792293	0.002795423	4082	2	1132
NM_005770	0.11418863	-0.4863521	0.471880279	0.001062004	2196	2	602
NM_005770	0.14205632	-0.5301845	0.471880279	0.000219	2195	2	585
NM_020642	0.22955756	-0.731483	0.632473635	0.001949084	2914	2	567
XR_014722	-0.511111	-0.5081488	0.757475256	0.016301022	2457	2	889
NM_016301	0.21136391	-0.4019577	0.901839983	0.002394274	4367	2	156
NM_016301	0.11278178	-0.8613645	0.901839983	0.007360183	2873	2	1078
NM_018158	-0.4417871	-0.260828	0.905550328	0.017968843	3507	2	753
NM_032883	-0.1565424	-0.0234794	0.799591898	0.004729412	3066	2	571
NM_032883	0.41170756	-0.1592724	0.799591898	0.018859137	3243	2	223
NM_006811	-0.0256309	-0.1791855	0.817027813	0.018480381	2694	2	196
NM_033112	-0.2888536	0.01831221	0.882339766	0.022764571	3531	2	519
NM_033112	-0.1457191	-0.3015924	0.882339766	0.035285415	2825	2	314
NM_014940	0.26179567	-0.4040412	0.598309123	0.036209333	3000	2	685
NM_012460	-0.4716597	-0.5561338	1.269255194	0.011147158	4116	2	1128
NM_012460	-0.1632846	-0.7301547	1.269255194	0.028528937	4399	2	1369
NM_001257	0.08724963	-0.2548031	0.713961454	0.004557732	3068	2	628
NM_175629	0.08393795	-0.3907273	0.484381847	0.000334401	3117	2	542
NM_175629	0.18778712	-0.3442281	0.484381847	0.001975349	3154	2	673
NM_153206	0.00716272	-0.7245751	1.128949259	0.013591841	4636	2	1047
NM_153206	-0.2183015	-0.9455953	1.128949259	0.006579968	4108	2	1155
XR_013517	-0.471628	-0.5077152	0.763713599	0.003494455	2624	2	636
XR_013517	-0.0602588	-0.2785919	0.763713599	0.005250752	3226	2	83
NM_018325	0.38468423	-0.1634141	0.819464489	0.010611742	3382	2	466
NM_018325	-0.4025392	-0.1653137	0.819464489	0.017839391	4679	2	1491
NM_205834	-0.1902097	-0.5965057	1.139062056	0.042295602	239	2	1606
NM_205834	0.51228821	-1.0050588	1.139062056	0.006040473	4092	2	1575
NM_004604	-0.1320186	-0.455841	0.546137982	0.002729038	2980	2	353
NM_004604	0.0735145	-0.217938	0.546137982	0.006228008	3127	2	431
XR_013996	-0.6748514	-0.6875937	1.395992827	0.011410169	3981	2	1567
XR_013996	-0.3958659	-0.6661987	1.395992827	0.007885676	4117	2	1221
NM_004168	0.11480651	-0.468924	0.571865471	0.000321513	2199	2	226
NM_004168	0.02343473	-0.4812955	0.571865471	0.000336532	2198	2	194
NM_014383	-0.1267821	-0.6311144	1.473772234	0.018355166	4531	2	1402
NM_014383	0.35065799	-0.3052916	1.473772234	0.025488944	4672	2	1533
NM_177533	-0.3408104	-0.1130943	0.540523312	0.034183615	2529	2	991

NM_177533	-0.358487	0.05939945	0.540523312	0.021681004	2532	2	1240
NM_006936	-0.1760388	-0.7466904	0.903780274	0.003835868	2665	2	611
NM_006936	0.21080565	-0.5449435	0.903780274	0.006891163	3050	2	590
NM_032360	0.24699286	-0.477037	0.659843665	0.004804994	2999	2	349
NM_032360	0.16449729	-0.3668421	0.659843665	0.030468277	3013	2	504
XR_012860	-0.0421227	-0.5175597	0.964825956	0.005219277	2634	2	129
XR_012860	-0.277905	-0.506807	0.964825956	0.003755227	2682	2	170
NM_003601	-0.2335809	-0.5565533	0.904535434	0.001722519	2864	2	123
NM_003601	0.37777097	-0.5413414	0.904535434	0.003421046	2929	2	261
NM_024334	-0.6227438	-0.5448804	1.116768456	0.009772243	2702	2	1058
NM_024334	-0.6012138	-0.44772	1.116768456	0.026934079	4382	2	1281
NM_032501	0.94441475	0.01042712	0.607061565	0.011560294	10856	2	1771
NM_005981	0.24914204	-0.5056366	0.381186701	0.011299049	3330	2	1052
CO647711	0.03029028	-0.9642337	1.080089692	0.017107146	4632	2	1486
CO647711	0.04579137	-0.7725812	1.080089692	0.02741814	4633	2	1366
CO647711	0.04284545	-0.7622851	1.080089692	0.027563824	4634	2	1349
NM_003011	0.01178306	-0.5795871	0.60001693	0.000362722	2111	2	209
NM_000234	0.39322128	-0.4178668	0.345546358	0.005151351	2203	2	1248
NM_000158	0.46249816	-0.9365434	0.772175228	0.025290187	4064	2	1508
XR_014323	-0.3920442	0.1549282	0.656412134	0.010148838	2537	2	1106
NM_025246	0.6224651	-0.7261274	0.668118378	0.002053971	3049	2	802
NM_024541	0.27873773	-0.4919878	0.692455443	0.002834933	3001	2	258
NM_024541	0.04371336	-0.105253	0.692455443	0.001165765	3236	2	149
NM_007266	0.16067684	-0.8699358	0.807953672	0.003289869	2874	2	816
NM_016134	0.02617323	-0.3099163	0.771780867	0.000913949	3188	2	168
NM_016134	0.01475822	-0.4744926	0.771780867	0.009352953	2868	2	700
NM_030815	0.27513724	-0.3281637	0.505935344	0.001153747	3159	2	656
NM_030815	0.21036121	-0.015787	0.505935344	0.01957616	3178	2	835
NM_001001890	-0.3271392	-0.8910436	1.337341755	0.042311733	1215	2	1731
NM_001001890	-0.1664388	-1.0163952	1.337341755	0.034399004	1216	2	1697
NM_017758	0.01318842	-0.6898626	0.759535674	0.000186046	2900	2	158
NM_003227	0.17176362	-0.3800519	1.355660429	0.033097379	4532	2	1254
NM_003227	1.31144917	-0.3509707	1.355660429	0.033070079	5276	2	1752
NM_002938	-0.376204	-0.0324988	0.641556329	0.015714203	2531	2	875
NM_002938	-0.484021	-0.0769642	0.641556329	0.001087009	2534	2	880
NM_030978	0.23314465	-0.7241407	0.919122364	0.003235548	2927	2	318
NM_030978	0.25255198	-0.5510512	0.919122364	0.004199218	2928	2	118
NM_016494	0.03041959	-0.2914611	0.500981174	0.003967234	3121	2	512
NM_016494	0.01630048	-0.1716853	0.500981174	0.004667752	3123	2	573
NM_004447	0.16976646	-0.624586	1.035854716	0.004100156	3053	2	630
NM_004447	0.62143554	-0.5231164	1.035854716	0.006057024	4570	2	1260
XR_010798	0.24034051	-0.4767308	1.104571779	0.011448374	4365	2	463
CN804671	-0.3066731	-0.0948267	1.047274703	0.01900786	2711	2	808

NM_014161	-0.1713938	-0.42272	0.584281763	0.007966126	2651	2	671
XM_370665	-0.1122845	-0.0903149	0.770809289	0.014789809	3214	2	180
NM_020683	0.11141493	-0.7606053	1.188438528	0.020350632	4637	2	1327
NM_019008	0.09465716	-0.3476897	0.657899553	0.047145737	3391	2	560
NM_004597	0.23427812	-0.1361668	0.50858553	0.025177501	3184	2	751
NM_005101	0.35596949	0.37192779	0.807960648	0.04357524	3307	2	1328
NM_016274	0.21048612	-0.7030315	0.749213055	9.17E-05	2902	2	224
NM_016274	0.16815293	-0.8073106	0.749213055	0.000242506	2912	2	362
NM_144641	0.16730098	-0.0777701	0.524097939	0.00176091	3171	2	662
NM_006496	-0.0152314	-0.4054306	1.012936889	0.011935802	4526	2	464
NM_000484	0.30628607	-0.7901122	0.729491791	0.013849985	3039	2	1112
CK230065	0.11341389	-0.6613113	0.648967401	0.000644002	2916	2	268
NM_015387	0.62472169	-0.2703	1.587440232	0.020754959	4674	2	1589
NM_015387	0.36425015	0.0834503	1.587440232	0.037647679	4673	2	1570
NM_003090	-0.3453192	-0.0483089	0.744034198	0.031206895	2545	2	614
NM_003584	0.15817336	-0.7824524	0.871021518	0.006267493	3032	2	767
NM_001436	0.10534107	-0.1733908	0.71072944	0.019582492	3241	2	192
NM_001436	0.11357503	-0.1340757	0.71072944	0.021070624	3242	2	230
NM_005828	0.50756348	0.12317109	0.939505925	0.039520067	3465	2	1232
NM_005828	0.471792	0.02653188	0.939505925	0.031063534	3466	2	1091
NM_005828	0.35919634	0.11941628	0.939505925	0.037093153	3464	2	957
NM_004842	-0.042394	-0.5848383	1.218198741	0.021059887	4638	2	1149
NM_207012	0.53992831	-0.6954232	0.695836393	0.000367376	2860	2	1019
NM_016602	0.45297385	-1.0535281	0.909157162	0.031669264	4643	2	1684
NM_138740	0.19049938	-0.4017143	0.646502702	0.016122741	3392	2	383
NM_138740	0.20456817	-0.2731352	0.646502702	0.007492038	3380	2	303
NM_138740	-0.0665678	-0.1308264	0.646502702	0.012259518	3147	2	492
NM_138740	0.34801176	-0.1409322	0.646502702	0.013769652	3381	2	632
NM_017947	-0.7887584	-0.3276906	0.766180927	0.003198226	2719	2	1303
NM_003858	-0.1763343	-0.2392091	0.684128184	0.004531624	3070	2	575
CN802495	-0.3557629	0.11146608	0.505348999	0.005447904	4681	2	1650
NM_003545	1.13803606	-0.8974706	1.335705399	0.006072475	5111	2	1761
NM_014188	-0.0995728	-0.178679	0.68829572	0.001510085	3131	2	203
NM_013254	-0.1675764	-0.3525368	0.824085634	0.004840314	2815	2	39
CN644731	0.2239884	0.07566262	0.855608755	0.007796732	3294	2	525
CN644731	-0.1050293	0.08494568	0.855608755	0.007202788	3219	2	452
NM_031266	0.33974264	-0.2897924	0.707415989	0.021467576	3325	2	1126
NM_031266	0.82199795	-0.7264455	0.707415989	0.009458696	3061	2	1598
NM_012342	0.30219814	-0.7511087	1.033633477	0.001569172	2942	2	487
NM_012342	-0.0324616	-0.7607037	1.033633477	0.006360092	2778	2	462
NM_003096	0.05089782	-0.4263529	0.701199851	0.027106356	2984	2	320
XR_012252	0.13684341	-0.0088059	0.884069197	0.006515889	3289	2	546
XR_012252	0.30785999	-0.1739913	0.884069197	0.027162384	3389	2	1164

NM_139279	-0.7525217	-0.4036557	1.071609131	0.012674729	4736	2	1338
NM_052862	0.78966424	-0.8384343	0.760778767	0.014127928	4596	2	1616
NM_052862	-0.1602057	-0.6726694	0.760778767	0.015134069	2772	2	664
NM_005662	0.06749574	-0.5740565	0.559530387	0.002172581	2110	2	356
NM_014674	-0.1222303	-0.2330705	0.707506763	0.000163967	3130	2	56
CN802671	-0.7041088	-0.5945901	1.40806922	0.026156543	1774	2	1743
CN802671	0.15954271	-0.2098259	1.40806922	0.013365812	4671	2	1238
CN802671	-0.3983036	0.12876244	1.40806922	0.03391672	4871	2	1635
NM_016113	0.19254004	-0.5565467	0.580576692	4.23E-05	2197	2	310
CN802860	-0.1275259	-0.4366123	1.048244282	0.011629622	4354	2	961
CN802860	-0.6492792	-0.1248269	1.048244282	0.022225741	3523	2	1293
XM_372262	0.04049752	-0.3517167	0.509402975	0.000621625	3120	2	399
NM_016077	-0.2224806	-0.3344955	0.63914758	0.002482455	2649	2	412
NM_016077	0.07196259	-0.6508147	0.63914758	0.007870253	2997	2	532
XR_014043	0.21181669	-0.7093119	0.771445217	0.003411561	2922	2	428
XR_014043	0.0705846	-0.618007	0.771445217	0.007637204	2991	2	271
NM_003310	-0.1572955	-0.7595753	1.00368184	0.029382075	3914	2	1347
NM_003310	-0.2063278	-0.8277054	1.00368184	0.01740175	3905	2	1236
NM_007024	-0.166769	-0.5585236	0.829679422	0.002124076	2632	2	101
NM_007024	-0.2496254	-0.407494	0.829679422	0.004645792	2630	2	162
NM_015694	-0.0735761	-0.7080017	1.362536202	0.043891895	1240	2	1654
BX648613	-0.2961452	-0.739697	1.262013765	0.031890357	4112	2	1525
NM_006423	-0.2899656	-0.4042238	0.698953668	0.010537738	2652	2	514
NM_003100	0.16396922	-0.0474348	0.663711096	0.041700658	3377	2	973
NM_005762	-0.5891749	-0.3372763	0.763902776	0.014116093	2696	2	1076
NM_003491	-0.6091297	-0.1614715	0.660676498	0.01083539	2520	2	1151
NM_003491	-0.5450547	0.1294833	0.660676498	0.021884589	2536	2	1304
NM_003792	-0.1716308	-0.4533609	0.771017532	0.000848167	2628	2	60
NM_003792	-0.1709455	-0.4103173	0.771017532	0.001333952	2629	2	62
NM_020191	0.10840465	-0.732095	0.935427506	0.002234668	2923	2	324
NM_014567	-0.0136003	-0.8388713	0.866630094	0.000331558	2667	2	410
NM_014567	-0.0668881	-0.6023207	0.866630094	0.001036241	2673	2	67
CN646864	-0.214886	-0.2309461	0.442208297	0.014926255	2511	2	861
NM_139005	0.48616733	-0.2341396	1.092633357	0.03835956	4667	2	1352
CN648602	-0.3779925	-0.3241957	0.554351007	0.019543939	2510	2	859
XR_012086	-0.1476664	-0.9257348	1.2010308	0.00864226	3915	2	1356
NM_032889	0.3162124	-0.9645322	0.86034676	0.001307839	2875	2	1077
NM_032889	0.26063933	-0.9529738	0.86034676	0.001373093	2944	2	937
NM_152277	0.01395204	-0.288175	0.632926585	0.013439904	3230	2	211
NM_014078	0.2711206	-0.3255134	0.83471968	0.019798385	3020	2	547
NM_014078	0.33823728	-0.034473	0.83471968	0.026822697	3266	2	762
XR_011531	-0.1751554	-0.6255827	0.936560076	0.003414842	2715	2	193
XR_011531	0.00231661	-0.4376566	0.936560076	0.001037469	2635	2	24

BX648005	0.59504518	-0.1363764	0.702630619	0.000709906	3368	2	1010
BX648005	0.6585853	0.08491556	0.702630619	0.002365101	3371	2	1330
NM_021177	0.21730101	-0.6675488	0.885213193	0.001081624	2925	2	138
NM_021177	0.24986523	-0.5031149	0.885213193	0.001893358	2926	2	76
NM_012394	0.59517862	-0.361672	0.623558632	0.005125483	3364	2	1179
NM_012394	0.7399018	0.08611616	0.623558632	0.000718617	3372	2	1484
CK231551	0.07279904	-0.2799645	0.431012013	0.02951732	3133	2	870
NM_017816	-0.3698634	-0.6864063	1.183911636	0.018985024	1223	2	1373
NM_017816	-0.0852573	-0.2886204	1.183911636	0.021708489	3453	2	409
A_01_P003274	-0.2616705	-0.2371556	0.81582497	0.002111204	3527	2	478
NM_020383	-0.3495268	-0.1402699	0.550397197	0.004155352	2524	2	867
NM_153705	-0.2470367	-0.3044378	0.89362986	0.00037848	2631	2	26
NM_153705	-0.326667	-0.1129291	0.89362986	0.000922542	3529	2	237
NM_022356	-0.6227547	-0.1496387	0.583504033	0.009753264	2521	2	1271
NM_130469	0.48830508	0.33226801	0.723685259	0.002125639	3308	2	1396
CO579800	-0.2989073	-0.5651995	0.826932132	0.002069933	2622	2	281
NM_005415	0.1091586	-0.8653534	0.966867908	0.000721782	2938	2	419
NM_005415	-0.0047923	-0.8288052	0.966867908	0.006439855	2777	2	592
NM_018389	0.12740321	-0.850643	0.883637536	0.000156993	2936	2	440
CB228901	0.15809348	-0.6926909	0.671159155	3.54E-06	2907	2	275
NM_005932	0.14774918	-0.6625172	0.835892605	0.012090078	2876	2	757
NM_021732	0.27842123	-0.4147171	0.409364354	0.025969815	3331	2	1182
NM_001696	0.07156023	-0.4995966	0.690129408	0.000442295	3199	2	164
NM_001696	0.04119521	-0.6726551	0.690129408	0.003801231	2077	2	305
NM_032312	-0.2501598	-0.6341519	1.038286805	0.015958394	3902	2	773
NM_032312	0.81803726	-0.281881	1.038286805	0.027712209	4666	2	1401
NM_001838	0.65085581	-0.7481109	0.63294888	0.002789329	2859	2	1432
XR_011940	0.54067032	-0.7208868	0.848005657	0.002187243	2956	2	982
AL137502	0.00310509	-0.2947404	0.595239966	0.01243653	3128	2	375
NM_152829	0.1397	-0.0222683	0.899497417	0.031294261	3456	2	379
NM_002437	0.46358514	-0.6875031	1.108764955	0.00769799	4371	2	1222
NM_002437	0.35260722	-0.3568242	1.108764955	0.008991241	4374	2	473
NM_030926	-0.01818	-0.6897382	0.896070684	0.000133082	2669	2	100
NM_006527	-0.4306003	-0.7157884	1.27946545	0.01106674	1224	2	1244
NM_018460	0.62226886	-0.9850853	0.793417747	0.000117078	2953	2	1409
XM_372816	-0.084225	-0.0491963	0.629561651	0.01741824	3067	2	1306
XM_372816	0.31243138	-0.1871453	0.629561651	0.037937548	3393	2	687
NM_016085	0.34463082	0.33419674	0.685173761	0.001015396	3306	2	1375
NM_032482	0.46886407	-0.3992699	0.585141302	0.00312549	3363	2	943
NM_032482	0.47356843	-0.3714601	0.585141302	0.002043579	3360	2	873
NM_012250	-0.1674672	-0.2473099	0.845021113	0.009268977	3064	2	374
NM_022831	-0.163416	-0.6606501	0.986643779	0.022306634	3912	2	904
A_01_P018380	-0.2746243	-0.4981914	0.698818325	0.00324038	2625	2	311

A_01_P018380	-0.4804963	-0.287259	0.698818325	0.027927289	3069	2	1020
NM_016039	0.21478501	-0.8407807	0.759171334	0.003137172	2943	2	683
NM_016039	0.20437747	-0.5816209	0.759171334	0.000833681	2917	2	98
NM_172127	0.29839391	-0.5045813	0.692973526	0.000953166	3023	2	242
NM_172127	0.52208745	-0.9231941	0.692973526	0.011037419	3057	2	1463
NM_006613	0.18564997	-0.5368241	0.492717058	0.00135185	2216	2	795
NM_031444	0.05803164	0.07483437	0.73255539	0.017545844	3218	2	536
NM_031444	0.01063976	-0.0971426	0.73255539	0.025038498	3232	2	249
NM_032828	0.17579553	-0.421751	0.62067509	0.002267494	3197	2	298
NM_033247	-0.4107624	-0.4381829	0.911237239	0.003085007	2623	2	338
NM_033247	-0.445829	-0.2489576	0.911237239	0.009538897	2698	2	604
NM_058246	-0.4879011	-0.2202984	0.966751028	0.043801328	2701	2	1138
NM_003675	-0.3758339	-0.2028701	1.062284999	0.007209243	4424	2	800
BC110642	0.0492285	-0.1972923	0.704345517	4.71E-06	3204	2	81
BC110642	0.06297424	-0.6606352	0.704345517	0.02462809	2132	2	708
NM_004186	-0.026417	-0.1134516	0.644563258	0.005237529	3132	2	351
NM_004186	-0.0488588	0.1122529	0.644563258	0.015391824	3223	2	793
NM_022341	0.13403346	-0.3454397	0.751157711	0.000272388	3203	2	38
NM_022341	0.12872272	-0.4370013	0.751157711	0.001166466	3200	2	71
NM_002712	0.03574191	-0.0550479	0.526892773	0.011910155	3148	2	725
NM_002712	0.09706565	-0.1771695	0.526892773	0.024392222	3146	2	584
NM_016059	0.61405548	-1.2303076	1.122205436	0.007697359	631	2	1675
NM_006295	0.34684714	-0.3022412	0.874485232	0.031360741	2402	2	834
NM_024700	0.27421655	-0.1873916	0.399802551	0.041975289	3183	2	1089
CN802634	-0.4747995	-0.2846693	1.162900657	0.014415729	3495	2	735
CN802634	0.10693472	-0.3100581	1.162900657	0.011996141	4527	2	371
CN802634	0.58494498	-0.4947974	1.162900657	0.009999735	4571	2	1538
CN802634	0.39270173	-0.3218466	1.162900657	0.049814174	4670	2	1639
NM_020371	0.1045519	-0.4381465	1.248954035	0.017612327	1243	2	1287
NM_019895	-1.4065637	0.34189656	0.870246414	0.02136282	1758	2	1754
NM_019895	-0.3050082	0.38394885	0.870246414	0.034173396	3539	2	1346
NM_032490	0.11745191	-0.9349126	1.191989548	0.001737002	4079	2	1013
NM_006681	0.12297903	0.05648173	0.711693757	0.004459141	3272	2	471
NM_006110	0.00404718	-0.7864894	0.91868086	0.000727823	2668	2	235
NM_006110	-0.0206703	-0.6706398	0.91868086	0.00123232	2920	2	82
NM_003844	-0.24267	-0.6710232	1.324824544	0.020466086	1227	2	1277
XR_012925	-0.1297651	-0.0731313	0.635207164	0.001736264	3208	2	340
NM_152464	-0.40149	-0.3169758	1.453203066	0.015351943	4454	2	1134
NM_152464	-0.3356047	-0.1891641	1.453203066	0.044067259	4455	2	1439
NM_004613	0.16568166	-0.0609927	0.702723605	0.000576091	3238	2	222
NM_004613	0.17416554	-0.1199343	0.702723605	0.003314999	3237	2	177
NM_000404	-0.3864509	-0.1661253	0.762904573	0.049285049	3508	2	868
NM_000485	-0.4886004	0.1285322	1.229185603	0.03211002	3553	2	1322

NM_002405	0.21070823	-0.4094135	0.793975792	0.003704984	2401	2	179
NM_002405	0.27713406	-0.0314822	0.793975792	0.001436524	3285	2	342
NM_016006	-0.3098643	-0.3472579	1.446018703	0.037483299	4522	2	1471
NM_003313	-0.5860626	-0.2721035	0.718748467	0.020624636	2697	2	1150
NM_003313	-0.4473746	-0.1525343	0.718748467	0.021014735	2530	2	877
NM_006582	0.12751717	-0.7051048	1.109316173	0.016569737	3041	2	928
NM_152268	0.30902239	-0.2873551	0.557848247	0.047038228	3376	2	1215
NM_004786	-0.0221469	-0.4961207	0.552891273	0.020408141	2988	2	612
NM_012145	0.33082517	-0.4011296	0.677665759	0.000910699	3339	2	531
NM_012145	0.59159721	-0.4950158	0.677665759	0.000677659	3361	2	987
CN644859	-0.0060538	-0.5178069	0.785214139	0.001035822	3052	2	737
NM_182491	0.46491055	-0.4202954	0.662233006	0.002478942	3025	2	721
NM_182491	0.60625242	-0.2286176	0.662233006	0.001794497	3366	2	1018
NM_000532	0.23140757	-0.6459423	0.970959018	0.009404965	3043	2	556
NM_000532	-0.3626516	-0.2251613	0.970959018	0.041499593	3494	2	1377
NM_152594	0.42887224	-0.5504457	1.08187722	0.033797469	4569	2	1291
NM_152594	0.21028542	-0.9467057	1.08187722	0.02336765	4065	2	1474
NM_018180	-0.0440216	-0.1717979	0.743782076	0.01032407	3195	2	227
XR_012251	-0.3330726	-0.2251085	1.203793129	0.043771119	4404	2	1041
NM_000449	-0.1484169	-0.3143768	1.007656649	0.030419759	3451	2	511
NM_000449	-0.4857582	-0.28651	1.007656649	0.039481245	4421	2	1372
NM_152485	0.12818041	-0.2529576	0.74516789	0.009604275	3233	2	111
NM_004401	0.28166393	-0.6939547	0.783545886	0.028166583	2383	2	1297
NM_004401	0.46408726	-0.920547	0.783545886	0.00030015	2856	2	1159
NM_004236	0.17423106	-0.2776135	0.690652081	0.008240397	3378	2	451
NM_033296	0.1583913	-0.3483039	0.824804177	0.016003126	3015	2	121
NM_033296	0.0122377	-0.1071105	0.824804177	0.013067213	3244	2	108
NM_017802	-0.0209573	-0.1063701	0.612247848	0.003733053	3076	2	944
NM_006407	0.21871567	-0.3912396	0.760495832	0.005851957	3002	2	134
NM_006407	0.24974316	-0.6199613	0.760495832	0.015359274	2998	2	622
NM_000639	0.57629921	-0.9098911	1.45212075	0.004749147	1334	2	1671
NM_006280	0.10491322	-0.8648115	0.987173431	0.004068418	2375	2	824
NM_006280	-0.0279728	-0.8579797	0.987173431	0.002983631	2374	2	712
NM_005764	-0.1608203	-0.1073285	1.006376168	0.001589801	3540	2	63
NM_005764	-0.0186582	-0.1240294	1.006376168	0.041203976	3454	2	350
NM_001166	-0.1494257	-0.3434664	0.906145441	0.016136193	2810	2	366
NM_001166	-0.2152396	-0.3017598	0.906145441	0.029097619	2824	2	276
NM_001111	-0.2767654	-0.2669322	0.785929985	0.003388429	2814	2	109
NM_001111	-0.3581488	-0.3118608	0.785929985	0.002279772	2813	2	175
NM_145690	-0.5388245	-0.6655815	1.093417297	0.029268057	3979	2	1526
NM_014281	-0.4551106	-0.0626534	0.843495515	0.000426081	2820	2	482
NM_014281	-0.5408092	0.19464718	0.843495515	0.001111869	2539	2	1201
NM_000281	-0.5356295	-0.3427407	1.142836816	0.003093003	2703	2	670

NM_000281	-0.6333938	-0.6318075	1.142836816	0.001087743	2732	2	993
NM_001961	-1.0041638	0.19166398	0.747837492	0.013482752	1756	2	1676
NM_001961	-0.6786361	0.21794504	0.747837492	0.00452036	2538	2	1456
NM_005255	0.15068447	0.02674652	0.819310765	0.001871428	3290	2	346
NM_005255	0.23590063	0.10348718	0.819310765	0.002445237	3291	2	655
NM_001804	0	-0.2978958	0.89771648	0.022119074	2693	2	283
NM_138807	-0.3150948	-0.1577754	0.939684162	0.026569144	3509	2	455
NM_014756	0.05124672	0.08251615	0.679330766	0.028544222	3271	2	821
NM_001903	-0.1432695	-0.0515573	1.04735902	0.012230457	3532	2	229
NM_001903	-0.5151243	0.35228467	1.04735902	0.016453535	4919	2	1386
NM_016100	0.25499645	-0.0047323	0.996538473	0.022320928	3463	2	550
NM_016100	0.19548687	0.05516838	0.996538473	0.040169556	3467	2	693
NM_021228	0.37009626	-0.8659909	0.990500029	0.049935043	4644	2	1647
CO581496	-0.1671847	0.06067802	0.566752466	0.03153527	3207	2	939
CO581496	-0.2626252	0.08673888	0.566752466	0.024868566	3206	2	978
NM_017750	0.04140739	-0.2713236	1.714070919	0.031832439	4533	2	1600
NM_017750	-0.4443489	-0.1977911	1.714070919	0.033932103	4873	2	1720
NM_153437	0.23246565	-0.5392698	0.555362824	0.008734579	2989	2	588
XM_497184	0.40427771	-0.3247219	1.01145869	0.005401069	3401	2	416
XM_497184	0.35346292	-0.3814523	1.01145869	0.038923963	3420	2	805
NM_004901	0.24195149	-1.0612636	1.020005992	0.027171242	1182	2	1617
CN643259	0.44002152	-0.5088944	0.863808479	2.15E-05	3362	2	252
CN643259	0.35208617	-0.145318	0.863808479	1.64E-05	3286	2	155
NM_004793	-0.3020963	-0.6337732	0.888237448	0.001067614	2736	2	376
NM_005402	0.0444633	-0.5383399	0.728659161	0.00600583	2986	2	151
NM_005402	0.01676038	-0.2583802	0.728659161	0.011335944	3239	2	107
A_01_P005263	-0.0778877	-0.1425903	0.681168037	0.015390214	3065	2	513
XR_011087	-0.2598691	-0.2492159	0.85193818	0.036441675	2828	2	538
NM_002293	0.36455602	0.61200473	0.980046633	0.035958346	4983	2	1582
NM_003746	0.34931778	0.12375806	0.764617756	0.002193303	3301	2	778
NM_003746	0.39758805	-0.0012567	0.764617756	0.023239569	3383	2	732
NM_006875	0.56545241	-1.2916947	1.129094476	0.002928849	608	2	1663
AB062478	-0.3516149	0.0506429	0.784899397	0.042284091	3496	2	1114
XR_009969	0.1389336	-0.857604	1.082751183	0.001417858	4242	2	420
XR_009969	0.14603548	-0.4715276	1.082751183	0.000553555	4275	2	7
NM_152531	-0.6236696	0.04157428	1.086622227	0.023598744	4868	2	1701
CN648445	-0.1286626	-0.4151202	0.864764119	0.018333328	3484	2	427
NM_014814	-0.0907919	-0.529784	1.181086718	0.001813954	4417	2	91
NM_014814	-0.2232454	-0.3318027	1.181086718	0.004553289	4405	2	141
NM_015917	-0.4027102	-0.3635641	0.881867669	0.014335591	2700	2	627
NM_015917	-0.2765501	-0.4836889	0.881867669	0.002216824	2671	2	161
NM_014112	0.7977156	-0.0460559	0.885969697	0.036948496	5078	2	1637
NM_004087	-0.1082159	-0.2376	1.109929035	0.005777706	4432	2	535

NM_181740	0.39263403	-0.3993141	0.826622362	0.022372305	3394	2	781
NM_019557	-0.1838909	-0.4747175	0.656593419	0.001089004	2648	2	160
NM_016630	0.27634579	0.01211816	0.581318261	0.032085872	3185	2	899
NM_005531	-0.3458324	-0.115347	0.606976186	0.035702086	2541	2	858
NM_173824	-0.0540987	-0.2905701	1.354950055	0.005735067	4529	2	1273
NM_000098	0.83633582	-1.0472098	1.044753998	0.00231105	4581	2	1561
NM_000098	-0.0318588	-0.5910639	1.044753998	0.004235982	2675	2	220
CK231052	0.05423862	0.19490823	0.887805194	0.018287424	3259	2	620
CK231052	0.06742155	0.1956877	0.887805194	0.046106307	3260	2	715
NM_012138	0.25739252	-0.1696476	0.938409981	0.004105468	3278	2	153
NM_012138	0.15939988	-0.3226931	0.938409981	0.000349053	2843	2	17
NM_017592	-0.3488285	0.3715336	0.920601679	0.011031384	4682	2	1704
NM_018457	0.25976652	-0.6606006	0.708106945	0.003301468	2918	2	403
NM_018457	0.39157363	-0.4350348	0.708106945	0.003525929	3024	2	426
CN643353	-0.2983274	-0.6516163	0.888746768	0.012997727	2660	2	731
NM_006007	-1.0100162	0.26870868	0.855830543	0.036397438	1757	2	1683
NM_005826	-0.336643	-0.4521097	0.660108361	0.00056463	2515	2	533
NM_001096	0.17673903	-0.273877	1.188270511	0.025850328	3461	2	659
NM_001096	0.27819324	-0.0831262	1.188270511	0.037101509	3462	2	947
NM_004450	0.45913654	-0.9657019	0.827412978	0.000993941	2947	2	1216
NM_004048	0.34035159	-0.0657089	0.805999205	0.003110389	3283	2	363
NM_004048	0.40113498	-0.0776171	0.805999205	0.004175987	3284	2	469
NM_004631	0.37752091	-1.0500497	0.999786511	0.003514245	4195	2	1427
DV769814	-0.2349567	0.28182845	1.011650039	0.007396327	4920	2	908
DV769814	-0.3377048	0.42338777	1.011650039	0.031912542	4921	2	1343
NM_014623	0.03794575	-0.3468011	0.687075047	0.009997633	3191	2	328
NM_015172	-0.0438505	-0.08451	0.50768785	0.043859273	3073	2	1008
XM_374526	-0.5068453	-0.1793221	0.788962524	0.035815185	2544	2	848
NM_003374	-0.0034023	-0.6655516	0.824180733	0.001705344	2919	2	105
NM_018406	0.27184567	-0.5905628	1.381283548	0.004076023	4377	2	742
NM_018406	-0.2463888	-0.440169	1.381283548	0.006833892	4433	2	660
NM_173823	-0.553204	-0.0506724	1.138710832	0.00863894	3524	2	995
NM_173823	-0.0333777	0.19428708	1.138710832	0.03498901	3458	2	932
NM_002721	-0.0008486	-0.6360172	0.99714734	0.003406519	2802	2	145
NM_002721	-0.231065	-0.613963	0.99714734	0.00322402	2807	2	251
BC094693	0.17510905	-0.6835357	0.661574946	0.000118347	2908	2	326
NM_012305	-0.4052543	-0.3207701	0.666369918	0.005854812	2517	2	665
NM_012305	-0.1396618	-0.0704661	0.666369918	0.009302303	3209	2	381
NM_003341	0.23944847	-0.2865344	0.877311931	0.017988602	3385	2	378
NM_003341	0.26930139	-0.1498608	0.877311931	0.020466918	3314	2	267
NM_016645	-0.1735591	-0.1692194	0.814928338	0.002891413	2819	2	55
NM_016645	-0.138135	0.0211158	0.814928338	0.003634286	3253	2	240
NM_003099	-0.0884326	-0.7348435	0.912421356	0.000892091	2771	2	144

NM_003099	0.20983126	-0.6565402	0.912421356	0.000853072	2961	2	84
NM_207043	0.04191673	-0.6833649	1.361205622	0.029344568	1244	2	1501
CN804935	0.24761982	-1.0416864	1.210427668	0.001970161	4090	2	1321
CN804935	0.03969395	-1.1377887	1.210427668	0.000285362	4088	2	1289
NM_004589	0.12749238	-0.2077191	0.675838772	0.043314076	3379	2	436
NM_002473	0.37538322	0.11118817	0.867794153	0.000755356	3302	2	739
NM_002473	0.2132851	0.11811837	0.867794153	0.00045222	3300	2	441
NM_017647	-0.4298902	-0.3764474	1.150471873	0.004539589	2704	2	484
NM_017647	-0.766107	-0.524703	1.150471873	0.002988018	1725	2	1443
NM_206817	0.03855109	-0.2207357	0.940041925	0.020114996	3488	2	327
NM_206817	0.03242374	-0.2958624	0.940041925	0.006072265	3196	2	94
NM_206817	0.04897875	0.23747573	0.940041925	0.048650678	3275	2	1217
NM_206817	0.09315448	0.20171264	0.940041925	0.020266386	3274	2	918
NM_022457	0.10541437	-0.6404549	1.04214222	0.000292448	2941	2	69
NM_022457	0.16450621	-0.4503726	1.04214222	0.001175532	4274	2	22
NM_017526	-0.1174902	-0.5908466	1.197302379	0.041454832	4660	2	1487
NM_002642	0.0405394	-0.3526218	0.721245235	0.000342602	3201	2	181
NM_002642	-0.139419	-0.535995	0.721245235	0.004187769	2481	2	651
NM_007282	-0.2688147	-0.7904941	0.956086361	0.000612475	2757	2	341
XR_010444	0.43011848	-0.5258099	0.56613751	0.002760128	3357	2	864
NM_144669	1.33075349	-0.1881479	0.914926628	0.008926114	5091	2	1719
AK125512	-1.0355322	0.40319625	1.588274447	0.049466176	4876	2	1748
NM_018686	-0.2473317	-0.3655509	1.392671854	0.025227464	4413	2	1105
NM_001916	0.23966871	-0.7820229	0.716475342	0.000115228	2909	2	499
NM_012453	0.12742165	-0.8807643	1.410357568	0.003085657	4309	2	1123
NM_012453	-0.154381	-0.6297578	1.410357568	0.004033277	4118	2	1178
CN805417	0.70698337	-0.098322	1.540615355	0.034174959	5085	2	1738
CN805417	0.82997101	-0.1708562	1.540615355	0.022430072	5084	2	1706
NM_014145	0	-0.5442377	1.008525367	0.011433627	3037	2	332
NM_001829	-0.9155917	-0.0465416	1.529709068	0.035886521	73	2	1767
NM_003299	0.36033149	0.2006925	0.878931647	0.000139341	3304	2	896
NM_003299	0.36300869	0.45482714	0.878931647	0.024776107	4982	2	1455
NM_006457	0.14584012	-0.7925749	0.967864369	1.34E-05	2939	2	112
NM_018648	0.4213437	-0.7938958	1.352372283	0.002081376	4378	2	1197
NM_018648	0.36285444	-0.4235058	1.352372283	0.002877845	4280	2	609
NM_012434	-0.4131452	-0.3938293	1.097604521	0.000862808	2685	2	596
NM_012434	-0.6315154	0.10609264	1.097604521	0.021805744	3522	2	1418
NM_152359	0.25515768	-0.7975628	1.011933975	0.00232932	2877	2	618
CO645096	1.00312044	-0.1320372	0.772338032	0.003078834	5076	2	1746
CN802953	-0.1367532	-0.4703808	1.240619678	0.018575912	4403	2	601
CN802953	-0.229713	-0.5835722	1.240619678	0.03004654	4401	2	1194
NM_003945	0.49615195	-0.7412596	0.900809254	0.000410963	2955	2	672
NM_003945	0.42480501	-0.8505216	0.900809254	0.003386933	2950	2	949

NM_007213	-0.1021775	-0.7184393	1.039252838	0.000103277	2716	2	270
NM_007213	0.19371626	-0.7331481	1.039252838	5.15E-05	2940	2	128
NM_033546	0.08613014	-0.079961	1.500916471	0.043913183	1254	2	1419
NM_033546	0.04334798	-0.0659924	1.500916471	0.046868013	1253	2	1431
NM_080659	-0.3518526	0.13620425	0.949747436	0.01179751	3555	2	1079
NM_002608	0.52404849	-0.8459918	1.283892643	0.023204144	4646	2	1642
NM_002608	0.36246145	-0.7798983	1.283892643	0.027018148	4645	2	1592
NM_016372	0.07499731	-0.2633237	0.584225866	0.004599211	3126	2	286
CO725491	-0.6438478	-0.6800343	1.287978622	0.011028504	1740	2	1652
CO725491	0.2149377	-0.3571822	1.287978622	0.028186917	4669	2	1385
NM_002979	0.0587371	0.25222125	0.778991229	0.043679063	3258	2	935
NM_138779	0.00060724	-0.2888674	0.758900708	0.002087477	3194	2	57
NM_052905	0.30192466	-0.8625998	1.249847496	0.002209007	4198	2	1188
NM_052905	0.23892072	-0.6622662	1.249847496	0.00165102	4199	2	569
NM_001117	-0.0885929	-0.6818079	0.835883805	0.01675902	2773	2	607
NM_001239	0.25193571	-0.5349004	1.335799484	0.004955737	4375	2	562
NM_001239	-0.6837744	-0.5349675	1.335799484	0.005996916	1775	2	1713
BC104664	-0.0198925	0.08035991	0.887694896	0.000695721	3256	2	215
BC104664	0.00769299	0.19343487	0.887694896	0.000385183	3257	2	502
NM_016647	-0.1482834	-0.0220457	0.940198133	0.007599692	3541	2	277
NM_016647	-0.5496863	-0.4388186	0.940198133	0.005023942	2741	2	847
NM_020179	0.05586596	-0.5144365	0.827890171	0.000116305	2679	2	13
NM_005110	0.11076929	-0.509626	1.479375535	0.003489063	4450	2	807
NM_014045	-0.5327471	0.15824569	0.734627381	0.029758202	2546	2	1259
NM_014045	-0.4143018	0.05164391	0.734627381	0.038091451	2547	2	920
NM_002966	-0.2493288	-0.204503	0.787145357	0.001901768	3192	2	148
NM_002966	-0.1667857	-0.2132062	0.787145357	0.000364237	3193	2	61
NM_032364	-0.0767885	-0.1173431	1.068262162	0.011523466	3455	2	115
NM_004251	-0.2401618	-0.5512162	1.2429834	0.002393945	4416	2	682
NM_004251	-0.1431601	-0.3992749	1.2429834	0.001450218	4418	2	41
NM_001007544	-0.0529025	-0.31688	1.057645913	0.002038518	2687	2	64
NM_006389	-0.2493914	-0.8477596	1.259528828	0.005039127	4109	2	992
NM_183233	-0.1219418	-0.6490776	0.969280602	0.000347558	2674	2	49
NM_033055	0.09512897	0.07761553	0.928566566	0.006338936	3293	2	373
NM_012479	0.2707917	-0.6560439	0.941579477	0.001020542	2962	2	110
NM_012479	0.27341264	-0.8575714	0.941579477	0.004327948	2946	2	754
XR_013359	-0.0096775	-0.3386176	0.949792803	0.007557738	2829	2	30
XR_013359	0.15577955	-0.1491937	0.949792803	0.024103768	3407	2	285
XR_011375	-0.3961985	-0.3045341	0.928117016	0.011994753	2726	2	360
XR_011375	-0.0025602	-0.5044707	0.928117016	0.046963727	4054	2	1027
XR_012381	-0.1412711	-0.5882476	0.811100255	0.001843536	2770	2	72
NM_020857	0.2683438	-0.4183238	0.758013524	0.012350612	3340	2	595
NM_020857	-0.0830042	-0.6154555	0.758013524	0.013365927	2561	2	666

CO649006	-0.1647431	-0.5979472	0.968360823	0.001898378	2633	2	65
CO649006	0.77959553	-0.2720804	0.968360823	0.011758173	4668	2	1460
CO649006	0.30359853	-0.2407602	0.968360823	0.019581848	3444	2	319
NM_032390	-0.097551	-0.2419106	1.030468946	0.019509327	3573	2	786
NM_032390	-0.0886818	-0.1396907	1.030468946	0.014076325	3556	2	703
CB231257	0.21670734	0.1223788	0.82417971	0.003320286	3298	2	515
CB231257	0.31312994	0.06517175	0.82417971	0.007567113	3299	2	541
CN648211	-0.5442003	-0.2755075	0.863190913	0.008455207	3498	2	1021
CN648211	0.04383993	-0.1328771	0.863190913	0.004900377	3263	2	132
CN648211	0.33660673	-0.0502044	0.863190913	0.007872088	3315	2	406
NM_130468	0.47198039	-0.0572223	1.253532175	0.038502017	4961	2	1363
XR_000132	-0.9187228	-0.4367708	1.081602117	0.012895613	1724	2	1607
NM_006814	0.11073288	-0.5521503	0.599344652	0.012424623	2480	2	1146
NM_006814	-0.2389446	-0.4495048	0.599344652	0.031399705	2564	2	1169
NM_005648	0.26547115	-0.1322845	1.538369205	0.03570643	1256	2	1488
NM_005648	0.52532691	-0.1661107	1.538369205	0.034375393	5083	2	1651
NM_138391	-0.4142449	-0.6627365	0.940627385	0.000228779	2739	2	370
NM_138391	-0.3642092	-0.4759338	0.940627385	0.000738785	2740	2	114
NM_013943	-0.2168933	-0.5666236	0.918017466	0.008779747	2806	2	257
NM_013943	-0.1306789	-0.3535538	0.918017466	0.049236674	2809	2	638
NM_006360	0.0396148	-0.7134134	0.750747173	0.011663279	2130	2	652
NM_078481	-0.1311922	-0.295879	0.872367196	0.001657909	2818	2	10
NM_078481	-0.0510226	-0.0996319	0.872367196	0.016413156	3245	2	102
NM_002124	0.05600053	-0.1053257	0.770507012	0.001138155	3235	2	73
NM_002124	0.25270162	0.34026618	0.770507012	0.006235956	3305	2	1219
NM_194071	-0.1180445	-0.3405568	1.236678917	0.005984213	4406	2	117
NM_016016	0.55754581	-0.5698075	0.493729109	0.028466018	3336	2	1472
NM_006368	0.00678455	-0.886677	1.088133154	0.000333934	2937	2	354
NM_006815	0.52385851	-0.2554671	0.947118312	0.011537019	3425	2	688
NM_006815	0.33995136	-0.1903642	0.947118312	0.020434893	3410	2	408
NM_006216	-0.3413745	-0.3873176	0.91776677	0.006728023	2705	2	692
NM_006216	0.17555333	-0.7421041	0.91776677	0.000206876	2670	2	208
NM_016310	0.63506289	0.5371322	1.241507628	0.035325116	4984	2	1657
NM_006682	-0.4510129	0.19893807	1.183749598	0.007699979	4869	2	1669
NM_006682	0.29595871	0.10159144	1.183749598	0.021441167	3468	2	1090
NM_001545	-0.0076013	-0.6076564	0.664737372	0.023089312	2479	2	1250
NM_178229	0.61117092	0.04865813	1.156076779	0.033260905	4978	2	1406
NM_178229	1.0579509	0.27849149	1.156076779	0.017313158	5079	2	1726
NM_003581	0.61247506	-0.4207382	0.631017075	0.005390323	3358	2	1118
NM_014180	0.2600934	-0.4529286	1.178538487	0.003450097	4273	2	174
NM_014180	0.48319996	-0.2289716	1.178538487	0.018826244	3449	2	1116
NM_014713	0.04084825	-0.4078171	0.845357288	0.017430898	3014	2	142
NM_014713	0.00396905	-0.0849733	0.845357288	0.022401766	3247	2	206

NM_002906	0.16036346	-0.4175985	1.212580085	0.002355688	4373	2	106
NM_002906	0.01325209	-0.398576	1.212580085	0.004875688	4419	2	78
NM_014064	0.22116939	-0.2823932	0.763910337	0.000178246	3205	2	93
NM_014064	0.29244945	-0.4118346	0.763910337	0.003732991	3026	2	402
NM_017955	1.94331949	-0.2432723	0.645702522	0.017679545	10875	2	1778
NM_002492	0.25286969	-0.0722592	0.829453361	0.001308298	3282	2	228
NM_002492	-0.2696821	-0.0896568	0.829453361	0.020485475	3571	2	819
CN805309	-0.9051413	-0.3248621	1.156560001	0.025314512	4675	2	1735
CN805309	-1.2857428	-0.2249267	1.156560001	0.024039707	225	2	1756
NM_001905	0.74181862	-0.1274533	1.265763543	0.047385482	5081	2	1608
NM_016258	-0.4595411	-0.4767457	0.997416397	0.001863741	2725	2	355
NM_198098	-0.0178596	-0.3224907	0.974322842	3.12E-05	2841	2	1
NM_198098	0.04044897	-0.202845	0.974322842	0.000107454	2842	2	3
NM_002512	-0.0933428	-0.5707266	0.8411465	0.000138208	2676	2	25
NM_002512	-0.1198125	-0.331255	0.8411465	0.000738013	2817	2	11
XM_376589	1.20551646	-1.1826571	1.430110505	0.010137894	5112	2	1797
NM_014500	-0.3050152	-0.6105224	0.913069725	0.008006337	2779	2	545
NM_014500	-0.2605341	-0.5203389	0.913069725	0.007802241	2782	2	190
NM_005917	-0.1433035	-0.7715876	0.890863345	0.015790795	2775	2	785
NM_005917	-0.1057387	-0.5460891	0.890863345	0.030460192	2808	2	591
NM_005860	0.09683638	-0.2803227	1.154656693	0.0321728	3489	2	839
NM_005860	0.02602015	-0.4015895	1.154656693	0.002397984	4372	2	48
NM_000382	-0.2303241	-0.4470481	1.172338462	0.000665747	2686	2	157
NM_000382	-0.5693145	-0.2988034	1.172338462	0.005042185	4390	2	804
NM_031298	-0.0546592	-1.0677439	1.17568097	0.00080832	3882	2	1054
NM_031298	-0.0245803	-1.0688816	1.17568097	0.000762978	3881	2	1108
NM_005138	-0.0157999	0.05681204	1.414043805	0.006581288	4470	2	724
NM_005138	-0.0775031	0.38391305	1.414043805	0.023008603	4474	2	1410
NM_024598	-0.0810124	-1.0309196	1.206376282	0.001948943	3676	2	1190
NM_024598	-0.17783	-0.7647977	1.206376282	0.001162513	4110	2	395
NM_032319	0.03256665	-0.630685	0.985490232	0.00722045	3036	2	393
NM_032319	0.11101074	-0.6520996	0.985490232	0.005196326	3042	2	315
XR_014069	0.14388288	-1.0068316	1.265872498	0.004560522	4101	2	1451
XR_014069	0.29966083	-0.7969573	1.265872498	0.002408339	4091	2	956
NM_015474	0.04101418	-0.0625683	1.044207278	0.032621984	3460	2	615
NM_015474	0.08974839	-0.2216098	1.044207278	0.009518616	3459	2	127
NM_001018067	0.48731485	-0.1541328	0.798310039	0.008090721	3384	2	838
NM_002494	0.19770165	-0.7482627	0.96555178	0.010784388	3035	2	958
NM_002494	-0.141416	-0.7636981	0.96555178	0.010772381	4608	2	1444
NM_000260	-0.9325973	-0.432862	1.074463535	0.044905278	4735	2	1661
XR_011890	0.39598808	-1.0122796	0.94063994	0.000849918	2948	2	1136
XR_011890	0.42446163	-0.9371421	0.94063994	0.002622763	2949	2	1098
NM_024097	0.34470036	-0.2810024	0.835401156	0.006269441	3276	2	302

NM_000108	-0.5321832	-0.6362931	1.023228358	0.004279655	2733	2	1094
CO578964	-1.2207267	-0.0217774	1.199468931	0.021230015	4677	2	1690
CO578964	-0.4448521	-0.1188091	1.199468931	0.001607678	3525	2	818
NM_020123	0.21465224	-0.1613086	0.891273193	0.018595887	3409	2	185
NM_024615	-0.4187791	-0.6464254	0.900964835	0.002264625	2735	2	760
NM_013400	-0.3317808	-0.6107064	1.013640912	0.011428493	3476	2	799
NM_014472	0.16525269	-0.0193835	0.81346385	0.016763685	3264	2	559
NM_001494	0.04168696	-0.3429942	1.120263897	0.007542312	3402	2	36
NM_001494	0.11178197	-0.2962087	1.120263897	0.015897918	3403	2	212
NM_004832	0.17886066	-0.4183098	0.916666702	0.000162444	3202	2	46
NM_004832	-0.0304425	-0.5056448	0.916666702	0.001805375	2833	2	14
NM_014214	-0.3465145	0.62037975	0.96883653	0.008863031	4922	2	1581
NM_003407	-0.4473326	-0.2724654	0.948018226	0.000607684	2821	2	188
NM_003407	-0.3713562	-0.1690213	0.948018226	0.001047992	2823	2	131
CN648772	0.27203548	-0.4157145	0.955029858	0.010999973	3046	2	345
CN648772	0.12300332	-0.0376063	0.955029858	0.018624676	3313	2	260
NM_016071	0.4216309	-0.7557219	0.971716528	0.002991033	4196	2	811
NM_016071	-0.0561214	-0.5220717	0.971716528	0.008615354	2830	2	103
XR_010210	-0.6651882	-0.2671923	1.20584526	0.002383227	4737	2	1310
XR_010210	-0.421074	-0.0809057	1.20584526	0.00520501	3511	2	548
NM_021222	0.14827141	-0.4730597	0.808154735	0.011849964	2987	2	198
XR_010915	-0.2793454	-0.966396	1.217807873	0.002427846	4120	2	1229
NM_012110	0.12176109	-0.3107281	1.093758157	0.007128741	3574	2	1158
NM_018955	-0.2162196	-0.3768123	0.933547298	0.000428335	2816	2	8
NM_018955	-0.3114691	-0.5914371	0.933547298	0.00483432	2781	2	247
NM_000955	0.26540598	-0.1398804	0.816045436	0.044417306	4594	2	1308
NM_000955	0.89437767	-0.359754	0.816045436	0.002282595	4598	2	1649
NM_032777	-0.5659638	-0.5478053	0.835296853	0.002682684	2565	2	981
NM_004824	-0.8748239	-0.0837743	1.389037176	0.006784576	1747	2	1693
NM_004824	-0.2066053	-0.7002197	1.389037176	0.005563474	4398	2	959
NM_024678	0.00084324	-0.3632721	0.828619602	0.005858554	4662	2	1518
NM_005318	0.41424156	-1.1590164	1.385379637	0.016489118	1186	2	1710
NM_005918	0.55349039	-0.3672277	1.009496936	0.000354945	3422	2	400
NM_005918	0.39893292	-0.275085	1.009496936	0.006678626	3421	2	272
CN644332	-0.5324551	-0.1874134	1.61880944	0.022800393	4523	2	1586
CN644332	-0.7091136	0.06910521	1.61880944	0.038501935	4524	2	1678
NM_182728	0.57265194	-0.745462	0.670283714	0.01138142	3058	2	1428
NM_182728	0.64290778	-0.5839772	0.670283714	0.030091135	3059	2	1430
NM_014725	-1.9125966	-0.1846131	0.889950288	0.000907194	216	2	1747
NM_001001	0.07269607	-0.2649237	1.40600966	0.008423079	4483	2	707
NM_001001	0.16939672	-0.3025274	1.40600966	0.012855583	4485	2	815
NM_017945	-0.2414394	0.3203131	1.709527539	0.028025928	4475	2	1619
A_01_P016459	0.4459306	-0.7503488	0.920105957	0.014710872	4587	2	1279

A_01_P016459	0.48435219	-0.6496036	0.920105957	0.012725014	4588	2	1191
NM_014300	0.09639789	-0.5489137	1.3859383	0.002600886	4420	2	646
NM_014300	-0.1839642	-0.136461	1.3859383	0.00245626	4459	2	352
NM_015471	0.39690919	-0.2491045	1.148658343	0.00047807	3443	2	309
NM_015471	0.30727282	-0.1988307	1.148658343	0.005404447	3442	2	521
NM_015969	0.11822934	-0.878557	1.166505978	0.000260003	4245	2	475
XM_046861	0.45356999	-0.0864068	0.92530935	0.008242317	3287	2	645
NM_024821	-0.1718506	-1.0375205	1.202262736	0.028651857	4658	2	1686
NM_024821	-0.0052363	-0.9752149	1.202262736	0.024090031	4659	2	1573
CK230616	0.01576114	-0.5948133	1.590833888	0.003807903	4451	2	1043
NM_014412	-0.1883173	-0.5949363	0.980414263	0.038792509	4048	2	1407
NM_014412	0.0075709	-0.3523275	0.980414263	0.016559551	2847	2	184
NM_033389	0.29319804	-0.9094157	0.918446167	4.99E-05	2893	2	663
NM_006082	0.30170174	-0.4382737	0.891176276	0.007322165	3404	2	182
NM_006082	0.32063214	-0.4053508	0.891176276	0.009170981	3405	2	197
NM_014763	-0.0851373	0.03803929	0.94515871	0.00964132	3265	2	549
NM_003524	0.20119877	-0.7619052	1.523129937	0.042235746	4661	2	1692
NM_000578	0.16058306	-0.3607552	0.600000437	0.030711102	2217	2	791
NM_000578	-0.1243242	-0.2610674	0.600000437	0.032375323	3078	2	704
NM_031901	0.28512687	-0.1382829	0.776078241	0.005832583	3281	2	313
NM_000073	0.04720518	-0.3521443	0.683066695	0.048236841	2990	2	555
NM_012383	-0.3788404	-0.9665843	1.249467733	0.00341784	4106	2	1353
NM_012383	-0.0796214	-0.6732796	1.249467733	0.011538985	4402	2	727
NM_004182	0.10565479	0.06739074	0.823856331	0.029452214	3273	2	810
NM_004182	0.09708965	0.23721636	0.823856331	0.027125853	3309	2	909
NM_016045	0.07937741	-0.3176128	1.656742485	0.026717621	1268	2	1688
NM_144593	0.43090174	-0.6947358	0.588716781	0.014925272	3335	2	1268
NM_001379	-0.1798016	-0.3945325	1.120174837	0.000703832	2832	2	12
NM_001379	-0.2749343	0.00644353	1.120174837	0.000771661	3550	2	214
NM_002456	-0.443329	-0.4279579	1.138241869	0.007290656	4353	2	1062
NM_002456	-0.2666525	-0.204113	1.138241869	0.009075678	3450	2	273
CN647604	2.15958138	-1.2451579	1.569373998	0.00188835	5274	2	1798
NM_000616	-0.1103542	-0.505346	0.853888404	0.032486817	3485	2	893
NM_000616	-0.1125238	-0.3251549	0.853888404	0.019898992	3486	2	430
NM_145342	0.00737765	0.05985829	0.848408218	0.017236177	3254	2	343
NM_005475	-0.4533397	-0.3681474	1.070000755	0.004247065	2706	2	842
CN644296	-0.1904308	-0.7162582	0.972562274	0.000522184	2791	2	113
CN644296	-0.0523517	-0.1160867	0.972562274	0.009310676	3246	2	53
CN646285	-0.2116792	-0.5468342	1.251752562	0.007246675	4400	2	423
CN646285	-0.4854985	-0.4101322	1.251752562	0.006298098	3510	2	775
NM_201280	0.48239953	-0.8335094	0.883017959	0.000247634	2954	2	846
NM_003755	-0.4511964	0.02765706	0.97578082	0.026666757	3535	2	1014
NM_003755	-0.4259045	-0.0874785	0.97578082	0.007116229	3534	2	599

NM_003755	-0.317282	0.25100971	0.97578082	0.035594951	3538	2	1173
NM_145049	-0.0987515	-0.6901295	0.746633435	0.039864045	2787	2	1068
NM_000441	0	-0.1215872	0.765500496	0.009269402	3234	2	125
NM_004890	-0.3358282	-0.3303445	0.75406513	0.006845077	3499	2	713
NM_005154	0.20209658	-0.551393	1.10708663	0.005941807	3045	2	304
NM_005154	0.2425711	-0.6505498	1.10708663	0.00508019	3044	2	508
CO648465	-0.2184033	-0.339438	1.604886264	0.004167424	4452	2	1184
CO649194	-0.0673098	-0.5502671	1.227307401	0.000441835	4355	2	77
CO649194	-0.1882545	-0.5942015	1.227307401	0.00035427	2717	2	205
NM_018217	0.00962196	-0.7425885	1.027355766	0.00050571	2378	2	262
NM_017653	-0.0053464	-0.7135555	1.375133894	0.002420108	4282	2	563
NM_017653	0.18938129	-0.618691	1.375133894	0.00931819	1249	2	887
NM_003981	1.42363594	-1.2510391	0.944673359	0.001632178	5108	2	1788
NM_005803	-0.3298541	-0.6190569	1.362975222	0.00423551	1241	2	1340
NM_006818	0.21624478	-0.2430282	1.399125881	0.016823242	4357	2	1393
NM_022917	0.12637724	-0.7663795	0.864417694	0.008024122	2384	2	869
NM_022917	-0.1878359	-0.519207	0.864417694	0.000793906	2672	2	45
NM_080422	0.12147924	-0.8598949	0.878747822	0.003057318	2872	2	686
NM_005041	0.037274	-1.0358141	1.368288176	0.000427336	4089	2	1195
NM_005041	-0.227208	-0.8830957	1.368288176	0.000487285	4155	2	856
XM_114685	0.14976007	-0.8715337	0.938485656	0.025957815	4628	2	1416
XM_114685	0.17576892	-0.860863	0.938485656	0.009694939	3033	2	1111
NM_003769	-0.1848435	0.20287303	1.209109601	0.007973491	4924	2	820
NM_003769	-0.0483179	0.32993795	1.209109601	0.00963714	4925	2	1035
NM_007273	-0.192709	-0.615104	0.791609755	0.00221072	2601	2	217
NM_007273	-0.0220847	-0.3940323	0.791609755	0.001272911	3190	2	58
NM_030752	-0.1935547	-0.2481387	1.670757995	0.010427653	4438	2	1380
NM_003365	-0.0407191	-0.7030712	0.937140333	0.001607492	2377	2	225
NM_002573	-0.2057431	-0.091589	0.931495654	0.049504772	3491	2	907
NM_002573	0.21299752	-0.1816651	0.931495654	0.004663965	3295	2	172
NM_013375	-0.4627693	-0.759882	1.106965384	0.009324348	2712	2	1422
NM_020150	-0.2843159	-0.3895361	0.892709583	0.02542867	2788	2	446
NM_006111	-0.438083	-0.5932915	0.920935713	0.021346692	2780	2	925
NM_006111	-0.2287073	-0.5287136	0.920935713	0.039219813	2783	2	840
NM_001688	0.19065238	-0.5203731	0.810388084	0.016646662	4663	2	1417
NM_002689	-0.5401325	-0.4050952	0.913372347	0.003604076	3497	2	702
NM_000942	-0.1651992	-0.9413421	1.088017426	7.03E-07	4202	2	479
NM_000942	-0.160856	-0.8469838	1.088017426	8.13E-06	4203	2	216
CO645097	-0.1895859	-0.0680986	1.000927961	0.029071724	2812	2	485
CO645097	-0.263816	-0.1850313	1.000927961	0.036985256	2811	2	551
NM_014481	-0.2600488	-0.8593592	1.168957856	0.002575553	4134	2	989
NM_015904	0.2194493	-0.3296403	1.255830338	0.036150881	4959	2	1241
NM_032747	0.43558077	-0.7877667	1.264979507	0.000107623	4323	2	967

NM_032747	0.54882578	-0.6833515	1.264979507	0.000426467	4559	2	836
NM_021237	0.35593947	-0.2027608	1.022390474	0.013947623	3411	2	312
NM_021237	0.54719451	-0.142248	1.022390474	0.020586773	3426	2	879
NM_000666	0.16383802	-0.8280154	0.982680726	0.002342041	2373	2	1110
NM_000666	0.30230029	0.02709052	0.982680726	0.012928142	3296	2	669
NM_016289	0.21367188	-1.0494254	1.058869611	0.011799466	4060	2	1438
NM_016289	-0.1518481	-0.6312011	1.058869611	0.008170292	2797	2	453
CN804369	-0.3173854	-0.9784831	1.310102085	0.002165428	3978	2	1309
CN804369	-1.0243178	-0.5981813	1.310102085	0.011268213	1771	2	1733
NM_004219	-0.3108365	-0.2341015	1.125229037	0.003658328	4426	2	914
NM_004219	0.84730376	-0.1739945	1.125229037	0.003492252	5077	2	1724
XR_013363	0.19935314	-0.953205	1.132377907	0.00282008	4069	2	1183
XR_013363	0.32285271	-0.8071775	1.132377907	0.00598021	4066	2	883
NM_016320	-0.5828161	0.02380764	1.087273815	0.018873585	1749	2	1625
NM_016320	-0.7267141	0.13297014	1.087273815	0.015734299	3521	2	1464
NM_015074	0.29908772	-0.7193936	0.623500904	0.023566733	2270	2	1137
NM_002807	-0.0496287	-0.7817952	0.857908357	0.006254741	2870	2	509
NM_003907	-0.5608379	-0.4181339	0.859151031	0.002014389	2724	2	621
NM_003907	-0.7455217	-0.4003455	0.859151031	0.003456574	2723	2	1162
NM_006284	0.35278337	-0.5848757	1.303749194	0.010191033	4589	2	1228
NM_006284	0.24850948	-0.5659726	1.303749194	0.002058083	4379	2	698
NM_000079	0.56513845	-0.8452565	0.679089642	0.016870621	4578	2	1601
NM_005348	-0.1365391	-0.5429196	1.224485415	0.00678513	3428	2	248
NM_005348	0.06169018	-0.5042071	1.224485415	0.00871175	3429	2	232
DB566244	0.49717398	-0.2892711	1.054571606	0.009447959	3423	2	710
DB566244	0.37681365	-0.283067	1.054571606	0.01906866	3424	2	486
NM_019006	-0.3270334	-0.2804531	0.786712435	0.029071617	3570	2	910
NM_019006	-0.8624233	-0.157904	0.786712435	0.025252703	1762	2	1520
NM_014041	-0.7685823	-0.3934346	1.379902644	0.001670068	4389	2	1326
NM_014041	-0.4098988	-0.524048	1.379902644	0.00591796	4397	2	871
NM_002811	0.38615725	-0.0072356	0.917382525	0.007846185	3319	2	761
NM_002811	0.55632421	0.12002625	0.917382525	0.007807841	3320	2	1220
CO725904	0.03386842	-0.3776055	1.245553258	0.004654324	4356	2	434
CO725904	0.07442597	-0.5865237	1.245553258	0.002383405	4272	2	201
NM_020404	-0.2148005	-0.8350375	1.003312122	0.001515713	4049	2	1210
NM_020404	-0.1909257	-0.6179763	1.003312122	0.003809073	2713	2	495
NM_024734	0.46645286	-1.0985862	1.304705128	0.000214991	4315	2	1511
NM_001003938	-1.2341925	0.39149446	1.427627972	0.012818368	72	2	1791
NM_194247	-0.4691088	-0.7442421	1.402203208	0.0015412	4133	2	1270
NM_001090	-0.3295144	-0.0042181	1.194331435	0.004421831	3543	2	629
NM_001090	-0.1962018	-0.1198113	1.194331435	0.00047808	3548	2	75
NM_019088	-0.1298139	-0.5597961	0.714246684	0.011855624	2655	2	540
NM_024805	0.36748711	-0.1992327	1.230875701	0.002344123	3448	2	329

NM_024805	0.52420954	0.09680839	1.230875701	0.001983821	4973	2	1080
XR_012243	-0.7455584	-0.5034612	1.025822595	0.020231662	4385	2	1374
NM_199203	0.21036443	-0.3265368	1.595674183	0.018419635	1259	2	1469
NM_199203	0.06792919	-0.2127942	1.595674183	0.022492679	1255	2	1376
NM_199203	-0.0162267	-0.5138225	1.595674183	0.029328542	1250	2	1536
NM_199203	-0.0936046	-0.2192575	1.595674183	0.0303624	1252	2	1453
NM_145040	-0.2215877	-0.4362957	0.675502924	0.017920546	2654	2	613
NM_145040	-0.263424	-0.4078468	0.675502924	0.01240747	2653	2	528
NM_006866	-0.1566762	-0.5488039	1.425015458	0.009684523	4435	2	1051
NM_006866	-0.1930173	-0.3287152	1.425015458	0.010905105	4436	2	888
NM_001862	0.30980101	-0.6744772	0.956155615	4.54E-05	2964	2	163
NM_001862	0.35097175	-0.6556711	0.956155615	6.27E-05	2965	2	176
CN804276	0.3770882	-0.6190083	0.953088972	0.003313941	2963	2	365
CN804276	0.36035214	-0.4689699	0.953088972	0.015987563	3400	2	766
NM_002798	-0.0728189	-0.533213	1.450985264	0.000501966	4448	2	244
NM_002798	-0.109127	-0.4286031	1.450985264	0.00078694	4449	2	291
XR_014011	-0.252207	-0.6978745	0.876225901	0.00141433	2769	2	231
XM_208423	-0.3683509	-0.0071756	1.033439298	0.001286876	3536	2	435
XM_208423	-0.3180215	0.1594801	1.033439298	4.29E-05	3551	2	625
NM_001006617	0.50289895	-0.0136999	0.97346999	0.001121267	4971	2	769
NM_001006617	0.32445761	0.19532608	0.97346999	0.000538191	3311	2	894
NM_015341	1.11016066	-0.9999779	1.630156209	0.002598744	5109	2	1793
NM_013248	-0.0586527	-0.9784138	1.115011914	0.00018259	4240	2	677
NM_032509	-0.2322858	-0.7819802	1.004982177	0.014360078	3911	2	1174
NM_003875	0.0131021	-0.1230023	0.777831914	0.010643067	3262	2	472
A_01_P014153	0.36111586	0.10622461	0.89373692	0.002761851	3303	2	752
CN801916	0.24867212	-0.6803745	1.210826885	0.001358686	4268	2	358
CN801916	0.41119456	-0.3061078	1.210826885	0.002765265	3447	2	339
NM_001795	-0.3207118	-0.2111642	1.261284948	0.017127491	3557	2	1002
NM_001795	0.00806559	0.04322726	1.261284948	0.040804057	3558	2	1104
NM_013232	-0.4185228	-0.5339608	1.071136157	0.016610235	2805	2	827
NM_013232	-0.5350663	-0.4513714	1.071136157	0.015924036	2804	2	969
NM_031420	-0.4518998	-0.5728265	1.018581694	0.000683071	3474	2	456
NM_031420	-0.1666347	-0.6831448	1.018581694	0.000969714	2798	2	85
NM_004559	-0.2426867	-0.3984964	0.976395554	4.82E-05	2839	2	15
NM_004559	-0.3707261	-0.1988376	0.976395554	0.000799231	2822	2	140
A_01_P007665	-3.520927	0.06763875	1.69764417	0.029527079	44	2	1787
NM_001007231	0.20812702	-0.7615648	1.193240845	5.83E-05	4265	2	204
NM_001007231	0.207098	-0.434939	1.193240845	0.000143468	4276	2	31
XM_497144	-0.3751501	-0.4513655	1.033639287	0.000470386	2743	2	135
XM_497144	-0.5196683	-0.4479846	1.033639287	0.000470618	2742	2	442
NM_015948	-0.6831691	0.39419974	1.071101062	0.044311958	4863	2	1609
NM_005642	-0.0280213	0.15089776	1.175430993	0.012283532	3457	2	898

NM_005642	-0.0633361	0.02980434	1.175430993	0.022585221	3563	2	711
NM_138425	0.04399752	-0.6072511	0.868914099	0.000256241	2677	2	35
NM_138425	0.04328471	-0.5517663	0.868914099	0.000252886	2678	2	23
NM_052884	0.53517192	-0.5879738	1.177167733	0.00034202	4324	2	678
NM_052884	0.55930163	-0.6844856	1.177167733	0.002268021	4558	2	1023
CB311167	0.20095621	-0.2967339	0.91860988	0.041578597	3398	2	489
XR_014331	0.36266226	-0.404894	0.912169271	0.005985755	3386	2	777
XR_014331	0.15739911	-0.2466441	0.912169271	0.039312724	3399	2	606
NM_004281	-0.115785	-0.8943068	1.173713752	0.000457291	2792	2	483
NM_014939	0.01851096	0.36119854	1.206602921	0.002803189	4928	2	1252
NM_022768	0.32537663	-0.882335	0.822409775	0.014697257	2385	2	1378
XR_011441	-0.5229423	-0.13634	0.821978293	0.0263167	4678	2	1544
XR_011441	-0.1607939	0.14631781	0.821978293	0.040884464	3252	2	830
XR_011441	-0.0942327	0.37429218	0.821978293	0.042283581	4664	2	1546
NM_016306	0.56872874	-0.2790863	0.997512534	0.008470659	3427	2	828
NM_014342	-0.5136168	-0.0095168	1.72909924	0.022920165	5009	2	1664
NM_080733	-0.2518359	-0.1183181	1.112705001	0.008456114	3544	2	364
NM_080733	-0.2112273	0.00353223	1.112705001	0.002658976	3546	2	290
NM_080733	-0.2169584	0.10701333	1.112705001	0.006411948	3547	2	608
NM_080733	-0.2980172	0.03812623	1.112705001	0.000959698	3542	2	387
NM_020239	-0.4441278	-0.7756632	1.50014904	0.005931823	1222	2	1550
CK231506	-0.2172164	-0.8473584	1.037304328	0.003186974	2376	2	829
NM_000594	0.26683129	-0.9798772	1.164547046	0.000695585	4313	2	1017
NM_000594	0.34237174	-1.1025703	1.164547046	0.00017386	4312	2	1315
NM_003314	-0.6950741	-0.4224373	0.837199096	0.0190117	1730	2	1473
NM_003314	-0.1299549	0.0272596	0.837199096	0.035834618	3269	2	653
NM_005328	-0.1590486	-0.5053307	1.257095187	0.047126177	4055	2	1341
NM_014952	-0.9508056	-0.0423297	1.133464341	0.011280231	1761	2	1566
XR_013749	-0.1674303	-0.8494543	1.383629711	0.000296299	4135	2	1066
XR_013749	0.08000768	-1.0533004	1.383629711	0.001103931	4197	2	1437
NM_006324	-0.1156288	-0.7458931	0.895954086	0.012514788	2784	2	831
NM_006324	-0.2633125	-0.3621551	0.895954086	0.015180111	2826	2	200
NM_006796	-0.0161188	-0.4991317	1.077231352	0.00040311	2834	2	2
NM_006796	-0.0885091	-0.595209	1.077231352	0.00094843	2794	2	33
NM_145654	0.49527832	-1.0614331	0.915970885	0.002573001	4582	2	1548
NM_021814	0.11661961	-0.2707365	0.985337605	0.007490442	3414	2	28
NM_021814	0.08280505	-0.1522651	0.985337605	0.022868283	3413	2	166
NM_032299	-0.0175213	-0.320983	0.989783218	0.002037431	2837	2	5
NM_032299	0.11315153	0.00180335	0.989783218	0.021952224	4684	2	996
NM_000860	-0.6460198	0.01769229	1.69826276	0.025125679	4525	2	1659
NR_000030	-0.320859	-0.6254872	1.088486374	4.31E-05	2744	2	74
NR_000030	-0.3656465	-0.4709888	1.088486374	8.78E-05	2745	2	79
NM_012180	-0.156281	-0.496002	1.239274114	0.008927008	4395	2	415

NM_012180	-0.4566976	-0.1996234	1.239274114	0.006701126	4391	2	756
NM_014142	0.23481513	-0.7803645	0.885987835	0.000382819	2894	2	385
NM_014142	0.23916279	-0.6452101	0.885987835	0.000820608	2958	2	195
NM_005619	0.21058472	-0.6600941	0.78972807	0.01892444	4576	2	1458
NM_003776	-0.1041856	-0.4254677	0.979397081	0.000123264	2840	2	6
NM_003776	0.00064537	-0.466058	0.979397081	0.002365493	2836	2	9
NM_020362	-0.483037	-0.3287811	0.860696951	0.006908597	3503	2	568
NM_022085	0.52424134	-0.2379932	1.223838113	0.000217491	4562	2	796
NM_022085	0.36629782	-0.3555611	1.223838113	0.00127701	3387	2	506
NM_002101	0.57957549	-1.1012782	1.299959577	8.83E-05	4316	2	1503
NM_017414	0.55022773	-0.6538847	0.962871649	0.001114231	2957	2	850
NM_017414	0.37228639	-0.3201564	0.962871649	0.001149847	3388	2	597
NM_030799	-0.1130182	-0.9989347	1.171496473	0.02615818	4071	2	1579
CB555416	-0.1860687	-0.298661	1.542387827	0.004325956	4437	2	965
CB555416	-0.083402	-0.3646157	1.542387827	0.01275203	1251	2	1133
NM_018017	-0.675622	-0.4846797	0.994177074	0.022934545	3492	2	1425
NM_018017	-0.2915403	-0.2322153	0.994177074	0.047633648	4427	2	1097
CK231580	-0.0697815	0.41355676	1.184967945	0.002855035	4926	2	1203
CK231580	-0.0936383	0.60450291	1.184967945	0.003595813	4930	2	1522
NM_020243	-0.1962184	-0.6184849	0.78264623	0.003958218	2600	2	413
NM_020243	-0.0137701	-0.4538982	0.78264623	0.003933427	3189	2	99
NM_020474	0.18905871	-0.7621753	1.425900157	0.001078683	4311	2	1082
NM_020474	0.12103894	-1.0416991	1.425900157	0.000407572	4308	2	1233
NM_012108	-0.1788571	-0.4566669	1.126179847	0.008200787	4476	2	269
CN642847	-0.2457262	-0.1926746	1.517884614	0.006128005	4464	2	826
CN642847	-0.2636109	0.10963826	1.517884614	0.007212485	4465	2	1180
CN642258	-0.9191178	-0.3360244	0.933869718	0.009219388	1760	2	1532
CN642258	-0.2829628	-0.5527892	0.933869718	0.03356982	2786	2	862
NM_004231	0.06723593	-0.9604607	1.273546371	9.17E-05	4209	2	745
NM_004231	-0.1062791	-0.8169045	1.273546371	1.74E-05	4210	2	259
NM_004394	-0.0256829	-0.5396263	1.071586963	0.008560359	3487	2	572
NM_004394	-0.3560839	-0.4680758	1.071586963	7.29E-05	4425	2	970
NM_006000	-0.0961913	-0.7312676	1.10147337	0.002879264	2790	2	265
NM_006000	-0.1484859	-0.5438875	1.10147337	0.00152818	2799	2	37
NM_024531	0.21365717	-0.5937206	0.515912001	0.044483776	3332	2	1311
NM_024531	0.38098852	-0.5077833	0.515912001	0.038299973	3333	2	1258
NM_024647	0.78487758	-1.1033211	1.208447415	0.003442011	1336	2	1739
BM991538	0.52812013	-0.6024263	1.064199601	0.000121863	2969	2	558
BM991538	0.24380408	-0.2469554	1.064199601	0.031336904	3396	2	624
NM_001070	-0.1493152	-0.3081081	1.071178902	0.009103733	3517	2	159
NM_001070	0.11811107	0.06520156	1.071178902	0.009534538	3261	2	388
XM_496348	0.22563186	-0.4555893	1.04803321	0.010062499	3406	2	221
XM_496348	0.16630768	-0.2296634	1.04803321	0.005135574	3408	2	40

NM_152690	-0.0396518	-0.9884701	1.278759671	0.002229144	4033	2	1272
NM_152690	-0.1594407	-0.8331333	1.278759671	0.001434961	4224	2	809
NM_144564	-0.3505807	-0.6284994	0.885079801	0.010795236	3473	2	813
XR_014443	-0.1576771	-0.6751065	0.976118462	0.0013868	2800	2	97
CK231268	0.05615174	-0.2908266	0.936881876	0.037492097	3395	2	422
CK231268	-0.1306046	0.09008811	0.936881876	0.009506551	3255	2	438
NM_002225	0.15701048	-0.8503962	1.024769324	0.000517809	4212	2	460
NM_002225	0.44451882	-0.5093506	1.024769324	0.000183725	2968	2	295
NM_001659	-0.1272773	-0.7791052	1.034902881	0.023117966	2776	2	1083
XR_013529	0.03847803	-0.8269831	1.110720268	3.27E-05	4213	2	186
NM_015895	0.1395059	-0.6132128	1.084472836	7.10E-06	4215	2	19
NM_015523	0.41857876	0.22110019	1.296801831	0.001355697	4974	2	1140
NM_015523	0.61402299	0.31585401	1.296801831	0.007069253	4975	2	1507
NM_019001	0.09314039	-0.7572249	1.123604763	0.000211274	4246	2	187
NM_019001	-0.0761477	-0.8216142	1.123604763	0.006488563	2789	2	723
NM_014184	0.41695078	-0.6403957	0.942266334	0.00077774	2966	2	396
NM_014184	0.80895175	-0.2033835	0.942266334	0.003179972	3373	2	1336
NM_182920	0.00319426	0.42297023	1.247493848	0.011335077	4935	2	1392
NM_182920	-0.0796702	0.5679889	1.247493848	0.019377057	4936	2	1572
NM_000786	1.44802475	-0.2799638	0.847050324	0.032795999	5090	2	1766
CN802406	-0.6666386	0.26425447	1.015118932	0.045232372	4862	2	1529
NM_007042	-0.4593369	0.00955881	1.656479657	0.030172316	5008	2	1584
CN647252	-0.0564856	-1.0074865	1.335905085	0.000517428	4250	2	976
NM_014244	0.0492313	-0.8797157	1.024269	6.80E-05	4176	2	561
CK232214	-0.4119232	-0.8994611	1.207778827	0.000479881	4125	2	1000
CK232214	-0.3517441	-0.6825996	1.207778827	0.000839833	4126	2	494
NM_153699	-0.4656104	-0.4182707	0.811582526	0.03439767	2484	2	1163
NM_153699	0.0011355	-0.0153351	0.811582526	0.026404174	3270	2	405
NM_005548	0.30986323	-0.6720639	1.065201866	0.001168074	2967	2	263
NM_005548	0.43916517	-0.9989105	1.065201866	0.007393498	2952	2	1399
NM_198477	-0.5075799	0.113218	1.244445763	0.000235197	3526	2	1257
NM_198477	0.04384177	0.62534201	1.244445763	0.011219416	4939	2	1549
CB309570	-0.0019646	-0.936188	1.390778545	2.54E-05	4211	2	720
CB309570	0.06641238	-0.673062	1.390778545	8.19E-05	4286	2	236
NM_198075	-0.2395549	-0.4436058	0.830836269	0.004791883	2656	2	490
NM_198075	-0.1719934	-0.0790632	0.830836269	0.042646121	3528	2	897
NM_002584	0.59419545	-0.8995599	0.934133235	0.00258913	4584	2	1462
NM_002584	0.63357559	-0.9439954	0.934133235	0.001416428	4585	2	1435
NM_032940	-0.569348	-0.6120796	1.080110833	0.03436362	1727	2	1421
NM_032940	-0.8142068	-0.1013493	1.080110833	0.00157176	4680	2	1354
NM_000633	-0.3696627	-0.806773	1.095742909	0.022792466	1816	2	1670
NM_000633	0.72925077	-0.6462733	1.095742909	0.027064238	4599	2	1611
NM_178858	-0.3673337	-0.7520384	1.453461961	0.000267425	4137	2	1154

NM_178858	0.00786215	-1.0790341	1.453461961	0.001708769	709	2	1547
NM_031458	-0.8212699	-0.2334026	1.083948047	0.016996699	1764	2	1540
NM_031458	-0.377099	-0.0724343	1.083948047	0.012982357	3515	2	679
NM_003859	-0.2295695	-0.1520599	1.442817857	0.015433098	4482	2	1045
NM_003859	-0.1250446	0.16324827	1.442817857	0.028523478	4953	2	1357
NM_004104	-0.1711887	0.07294764	1.796166827	0.016007368	5011	2	1585
NM_004104	-0.3887474	0.36553931	1.796166827	0.013330737	4957	2	1682
BM918631	0.2002809	-0.5033966	1.167704794	0.013764284	3430	2	439
BM918631	0.05707857	-0.2030616	1.167704794	0.009066606	3416	2	88
CB231011	0.00485815	-0.8543026	1.182126225	0.000733684	4243	2	384
CB231011	0.06470586	-0.809108	1.182126225	0.00057415	4244	2	245
DQ148160	0.26520066	-0.3799066	1.128842575	0.007225431	3441	2	544
DQ148160	-0.2251557	-0.2892617	1.128842575	0.008961142	4407	2	124
NM_001418	-0.1756708	-0.4008923	1.010842275	0.005103641	2835	2	44
NM_001418	-0.2493899	-0.20573	1.010842275	0.017680628	2827	2	210
NM_012097	0.19268302	-0.9250721	1.086322565	0.000762569	4231	2	872
NM_012097	0.41732303	-0.4620493	1.086322565	0.002039862	3445	2	344
NM_016078	-0.479144	0.02168171	0.963757813	0.006864774	3514	2	905
NM_016078	-0.7471452	-0.1346603	0.963757813	0.020389533	1763	2	1395
NM_019043	0.44606866	-0.7304021	1.301258099	7.08E-05	4271	2	783
NM_019043	0.32843898	-1.0017307	1.301258099	0.000234352	4317	2	1113
XR_012719	-0.0996375	-0.6028269	1.354665125	0.004351867	4228	2	633
XR_012719	-0.3226761	-0.7258803	1.354665125	0.008013181	4111	2	1145
NM_003902	0.18979199	-0.6207083	0.939622835	0.001668773	2959	2	90
CB230657	-0.0323332	-0.2955606	1.068586613	0.003769652	2838	2	16
CB230657	0.18970545	-0.1449289	1.068586613	0.008196432	3415	2	95
CB230657	0.06142286	-0.0447545	1.068586613	0.017103386	3417	2	219
CB230657	0.21749724	0.03428813	1.068586613	0.015488476	3419	2	432
CN642474	0.0424625	-0.2018211	1.433184006	0.011187633	4484	2	733
NM_018322	-0.8919171	0.02342135	0.891995317	0.012826122	1767	2	1653
NM_003023	0.08675529	-0.6436757	1.144766531	0.012805694	4352	2	1084
NM_015379	0.16049834	-0.6984021	1.176414325	7.83E-05	4247	2	70
NM_015379	0.20524363	-0.6571826	1.176414325	0.000678235	4248	2	96
NM_004161	-0.3531824	-0.5186819	0.975276535	0.002214813	2728	2	234
NM_004161	0.32154207	0.02423712	0.975276535	0.011626094	3288	2	579
NM_020300	-0.1010739	0.66684769	1.082890727	0.032345662	4929	2	1596
CN647507	0.12114598	-1.0159766	1.276463337	3.00E-05	4239	2	895
CN647507	0.31600459	-1.0616618	1.276463337	0.000613097	4237	2	1206
XR_009960	-0.1927915	0.02469581	1.200817786	0.017280761	4867	2	1565
NM_020529	0.34898558	-0.0534175	1.287105319	0.013827722	4979	2	768
NM_020529	0.50522605	0.31754266	1.287105319	0.047676733	4980	2	1500
XM_373301	-0.1774052	-0.0478849	1.162649128	0.004441546	3545	2	297
XM_373301	-0.1176517	-0.1119326	1.162649128	0.000518298	3549	2	66

XR_010456	-0.4758592	-0.7246436	1.328300234	6.23E-05	4145	2	780
XR_010456	-0.4554274	-0.6735221	1.328300234	1.50E-05	4146	2	576
XR_014546	0.26567192	0.19220549	1.348441669	0.036523599	3470	2	1398
XR_014546	0.12296954	0.10691241	1.348441669	0.03143659	3469	2	1218
NM_144646	-0.3935606	-0.9958321	1.227991348	0.002347103	666	2	1305
NM_144646	-0.315367	-1.0148812	1.227991348	0.005579065	703	2	1389
NM_006118	-0.2737581	-0.5070199	1.230613769	0.001624265	4151	2	238
NM_006118	-0.3429653	-0.4374356	1.230613769	0.001159392	4152	2	334
NM_022763	-0.1697304	-0.8482782	1.059490048	0.015623845	4070	2	1253
NM_152330	0.09800725	-0.1868125	1.108034811	0.0002078	3559	2	674
CN648711	-0.5104911	-0.6881107	1.050018614	0.003671038	4124	2	955
NM_178191	0.16221597	-0.0351188	1.563535416	0.002586379	4471	2	852
NM_178191	-0.3843926	0.11776688	1.563535416	0.008056466	4956	2	1477
NM_178191	-0.2077805	0.43503604	1.563535416	0.006004537	4954	2	1556
NM_178191	0.16541475	0.0819678	1.563535416	0.021166729	4472	2	1325
NM_178191	0.20383512	0.5918725	1.563535416	0.010359723	4942	2	1633
NM_178191	0.13002938	0.1878786	1.563535416	0.031256326	4473	2	1441
NM_030961	0.04944309	-0.2375875	1.060951066	0.024157077	4687	2	1658
NM_006079	-1.3072779	-0.4482402	1.401477681	0.011498907	4738	2	1736
NM_006079	-2.0446528	-0.1662451	1.401477681	0.033331169	234	2	1792
NM_002647	0.35567538	-0.7278369	1.183497361	0.006969699	4262	2	942
NM_002647	-0.1727068	-0.4372068	1.183497361	0.003020769	4431	2	465
NM_175748	0.17454004	-0.6583192	1.077569481	0.000438545	4178	2	296
NM_024863	-0.2254443	0.31263685	1.344460411	0.038265643	4927	2	1379
NM_016481	-0.0615008	-0.7457632	1.36258403	9.29E-05	4281	2	255
NM_020676	0.33592074	-0.1706881	0.960744514	0.003324315	3316	2	246
NM_018115	0.34039132	0.06793206	0.79565608	0.03633308	4665	2	1388
NM_013448	0.43683717	-0.1955619	1.224212804	0.003365077	4595	2	1446
NM_013448	0.5409445	-0.0064596	1.224212804	0.002833455	4972	2	1056
NM_078468	-0.3886215	-0.7677417	1.12665152	0.000174118	4129	2	553
NM_000043	-0.8069084	-0.4817849	1.302848973	0.000545669	1741	2	1454
NM_000043	-0.1863275	-0.6840785	1.302848973	0.000813777	4136	2	516
NM_000043	-0.5756608	-0.8354489	1.302848973	0.004455983	3592	2	1449
NM_000043	0.06463797	-0.5118485	1.302848973	0.00242869	4429	2	476
NM_000732	-0.1281263	-0.8462955	1.163413562	0.009080792	4625	2	1242
XR_014700	-0.9465672	-0.7284341	1.278671387	0.008725084	4383	2	1636
XR_014700	-0.8694019	-0.2222743	1.278671387	0.022316029	4388	2	1560
NM_005891	-0.3427617	-0.0669801	0.926738555	0.006757452	29	2	1796
NM_005891	-0.1187393	0.38275036	0.926738555	0.04157023	4865	2	1626
NM_032737	-0.3861641	-0.6474942	1.089068059	3.15E-05	2737	2	543
NM_032737	-0.0180742	-0.6348258	1.089068059	0.0002539	2803	2	59
NM_014604	-0.5209683	-0.8091078	1.232899517	5.25E-05	4128	2	884
AK128435	0.21218552	-0.6815689	0.781659842	0.023927561	4163	2	1092

AK128435	-0.0205174	-0.2933623	0.781659842	0.048375425	3569	2	1028
NM_002128	0.24606238	-0.0899033	1.003807725	0.020293352	3418	2	337
NM_002128	0.4762083	0.07336726	1.003807725	0.0242094	3318	2	1033
XR_011517	-0.4605515	-0.8454763	1.228118168	0.001070889	4127	2	968
NM_001686	-0.3851564	-0.6930045	1.242373721	1.59E-05	4147	2	308
NM_001686	-0.1587263	-0.4892827	1.242373721	0.000333689	4333	2	20
NM_005675	-0.2275769	-0.5142649	0.971449849	0.015740537	2714	2	784
NM_005675	-0.1809902	-0.6520897	0.971449849	0.00888377	2729	2	517
NM_016594	-0.1748876	-0.7436047	1.332487176	0.001544448	4255	2	461
NM_016594	-0.0699659	-0.8265834	1.332487176	0.002840878	4256	2	741
NM_016507	-0.2963206	-0.8577437	1.057407739	0.000969456	3684	2	605
NM_032704	-0.0811194	-0.933535	1.209475363	5.66E-06	4205	2	468
NM_032704	-0.0458126	-0.9831239	1.209475363	2.00E-05	4204	2	603
NM_000889	-0.1470213	-0.8771694	1.06601608	0.006249935	4035	2	1072
NM_000889	0.44336272	-0.340529	1.06601608	0.034076302	4960	2	1202
NM_030938	-0.4880057	-0.7553335	1.254114674	0.021419671	1425	2	1760
NM_005002	0.06630398	-0.6258549	1.153551761	8.78E-05	4214	2	52
NM_005002	0.25958845	-0.4717785	1.153551761	6.65E-05	4277	2	34
XR_012012	-0.7628704	-0.6425832	1.163208829	0.000549756	1723	2	1314
XR_012012	-0.6356702	-0.5216093	1.163208829	0.000214829	2746	2	779
NM_000062	-0.2090624	-1.0672109	1.244727483	8.91E-06	4201	2	1030
NM_000062	-0.2306804	-0.7771579	1.244727483	1.27E-06	4206	2	213
NM_004853	0.09770566	-0.7426747	1.429744299	8.88E-05	4287	2	524
NM_004853	0.16468196	-0.7241306	1.429744299	7.26E-05	4288	2	459
NM_030782	-0.2731653	-0.1902398	1.652459373	0.003337672	4439	2	1205
NM_030782	0.27120153	0.00215989	1.652459373	0.004092024	1261	2	1368
NM_013336	0.18328109	-1.0881569	1.281630272	1.24E-05	4238	2	1074
NM_022337	0.75584231	-0.1947118	0.957363963	0.032083553	4600	2	1662
NM_024119	-0.9127538	0.47898481	1.311705121	0.004571129	4879	2	1712
NM_024119	-0.6592967	0.66687751	1.311705121	0.015996744	4880	2	1703
NM_176805	-0.1663394	-0.4639484	1.412276234	0.000793551	4441	2	279
NM_176805	-0.1347868	-0.4605955	1.412276234	0.000506928	4442	2	293
NM_176805	-0.1992387	-0.4431724	1.412276234	0.000939724	4434	2	617
NM_176805	0.16638703	-0.4243816	1.412276234	0.000465538	4279	2	250
NM_007033	-0.5274502	-0.8806934	1.226168372	0.00086727	664	2	1243
NM_016127	-0.1787663	-0.2941649	1.349119473	0.014413313	4408	2	565
NM_016127	-0.1125009	-0.3638074	1.349119473	0.02582942	4414	2	922
CN641451	-1.0411356	-0.5345422	1.181235627	0.013883382	1772	2	1679
XR_014668	-0.2584215	-0.3919115	0.98677809	0.001597029	3572	2	1015
XR_014668	0.19327566	0.3076559	0.98677809	0.032231222	3310	2	1141
NM_006335	-0.9333212	-0.1720451	1.28015611	0.003364992	223	2	1750
NM_006335	0.03973612	-0.4693662	1.28015611	0.002265192	3433	2	104
NM_152255	0.38741095	-0.9258029	1.34374925	8.22E-06	4269	2	990

NM_152255	0.27411108	-0.8115962	1.34374925	4.56E-05	4270	2	644
NM_002087	0.25017152	-0.6702084	1.267530081	9.20E-06	4267	2	150
NM_002087	0.24722475	-0.4284383	1.267530081	1.33E-05	4278	2	32
XR_010283	-0.3140497	-0.6971819	1.516553263	0.000426223	4157	2	972
XR_009898	0.05499396	0.12603718	1.208447038	0.006448501	4963	2	999
NM_014402	0.25416967	-1.06353	1.097671837	0.002353098	2951	2	1237
NM_014402	0.12040098	-0.8124616	1.097671837	0.001651866	4258	2	359
XR_010705	-0.4304136	0.0085958	0.876649026	0.042485216	3506	2	919
NM_031412	0.28718787	0.25587373	1.087533764	0.005609771	3312	2	1192
NM_031412	-0.2511825	0.40576079	1.087533764	0.029636741	4923	2	1301
NM_000386	-0.5421266	-0.4708832	1.220771123	0.008798295	4387	2	945
NM_000386	-0.7437368	-0.5137235	1.220771123	0.015281698	4386	2	1468
NM_018101	0.02752929	-0.8031324	1.386766353	0.002616379	4227	2	962
XR_014799	0.15067241	-0.6083778	1.053140957	0.001097901	2960	2	42
XR_014799	0.21690091	-0.5509659	1.053140957	0.002611532	2970	2	92
NM_000417	-0.092218	-0.6312984	1.406760446	0.019718851	4657	2	1370
CN647420	0.35601761	-1.0131684	0.989839745	0.001897883	4184	2	1335
NM_001376	-0.0416405	-0.318913	1.175055705	5.19E-05	2844	2	4
NM_001376	-0.0317087	-0.1538564	1.175055705	6.62E-05	2845	2	21
CK230740	0.13873441	-1.005449	1.331831709	0.000291892	4253	2	998
CK230740	0.03100422	-0.949758	1.331831709	0.001469104	4251	2	924
A_01_P006157	0.28209189	-0.8126737	1.162395783	0.001789572	4259	2	574
A_01_P006157	-0.0554596	-0.6447097	1.162395783	0.00221047	2795	2	136
NM_006542	0.24015116	-0.3819931	0.923872949	0.044473217	3397	2	860
A_01_P007352	0.0461589	-0.2058651	1.118309451	0.003043274	4347	2	116
A_01_P007352	-1.4917305	-0.4081158	1.118309451	0.008534139	226	2	1789
A_01_P003292	-0.0394216	-0.4939928	1.48281546	0.003979428	4530	2	1003
NM_080614	0.2192379	-0.1472949	1.542310354	0.00125737	4307	2	911
NM_080614	0.16178792	-0.0491403	1.542310354	0.002569519	1260	2	1061
NM_006145	0.14836494	-1.0156378	1.29438274	6.17E-05	4235	2	930
NM_001001486	-0.0884618	-0.3263046	1.19010083	0.007586031	4428	2	287
NM_001001486	0.05904847	-0.33562	1.19010083	0.013893612	3431	2	274
XR_013248	-0.1826109	-0.0477981	1.356097588	0.000206125	4460	2	218
XR_013248	-0.0376086	-0.0945194	1.356097588	0.000520821	4462	2	130
NM_001001795	-0.6251454	-0.5229044	1.40162358	0.021836039	1219	2	1602
NM_002788	0.42810395	-1.1532314	1.373348164	0.000762327	629	2	1513
NM_002788	0.81086418	-1.2046588	1.373348164	0.003200651	633	2	1705
NM_006988	-0.5409635	-0.3637762	1.375695624	0.000249186	2747	2	728
NM_006988	0.13335377	0.19571642	1.375695624	0.000220891	4937	2	953
NM_002106	-0.0252871	-0.4393229	1.546011965	0.000907723	4486	2	510
NM_002106	-0.0552341	-0.2079128	1.546011965	0.010043531	4415	2	966
NM_005730	-0.4021574	-0.2356657	0.931441044	0.028361771	3501	2	764
NM_005730	-0.5142852	-0.0854032	0.931441044	0.039397391	3502	2	1171

NM_031466	-0.1723068	-0.8087357	0.993419621	0.000461342	4173	2	437
CN806583	-0.5015428	0.23185705	1.127723712	0.037711075	3537	2	1411
CN806583	-0.292114	0.11857387	1.127723712	0.001939886	3552	2	635
NM_004618	-0.2977022	-1.0490309	1.33657716	1.43E-05	4122	2	1239
NM_014206	0.15005462	-0.3732497	1.445541095	6.45E-05	4305	2	171
NM_014206	-0.107227	-0.1533126	1.445541095	0.000227478	4463	2	233
NM_198507	0.18844501	-0.3196547	1.610519377	0.005714815	4497	2	1011
NM_001212	-0.1363819	-0.7660181	1.149552357	0.000426919	2796	2	382
NM_001212	-0.1884085	-0.6650613	1.149552357	0.00247511	2793	2	243
NM_005956	-0.1353233	-0.856703	1.070765347	0.00307486	4177	2	717
NM_198194	0.36066891	-1.0616074	1.366636675	0.00016252	4314	2	1350
NM_198194	0.37017102	-0.8710563	1.366636675	4.64E-05	4320	2	938
NM_003347	0.19257422	-0.775901	1.245380207	0.000330959	4266	2	321
NM_003347	0.35056502	-0.4699481	1.245380207	0.001726339	3446	2	256
NM_181789	-0.104993	-0.5117357	1.089517854	0.003618473	2801	2	120
NM_020895	0.09653406	-0.7817946	1.148369609	0.002753803	2379	2	649
NM_003091	-0.4609128	-0.6631944	1.426775113	0.002999645	3480	2	1256
NM_003091	-0.2451209	-0.6258221	1.426775113	0.002865374	3481	2	951
NM_018344	-0.0824768	-0.4855136	1.030660298	0.006685651	2831	2	87
NM_018344	0.07387873	-0.3024746	1.030660298	0.006333826	3412	2	50
XR_011417	-0.3102602	-0.6639733	1.063066673	0.011798506	2785	2	788
XR_011417	0.04201571	-0.2798893	1.063066673	0.020044273	2848	2	202
NM_023003	-0.1676303	-0.0350235	1.27561182	0.049591301	3575	2	1495
NM_033274	0.39242897	-0.9569028	1.504004829	0.000247425	4319	2	1324
NM_033274	-0.0752275	-0.843686	1.504004829	0.000201643	4139	2	886
NM_052928	0.3433633	-0.036741	1.016582627	0.008481451	3317	2	448
NM_019045	-1.5854441	-0.0237737	1.314795928	0.015437527	217	2	1769
NM_005389	0.86453093	0.39379415	1.064120283	0.048527543	5080	2	1698
NM_006768	0.0561568	-0.8419884	1.201395905	9.41E-05	4223	2	586
NM_006768	0.00853114	-0.7269082	1.201395905	0.000298363	4216	2	154
CK231677	-0.7140283	0.82472802	1.520157384	0.017982816	4881	2	1740
NM_020701	0.41590633	-1.154588	1.188431536	0.000291271	621	2	1555
NM_007236	0.07813739	-1.0263172	1.204118499	8.10E-05	4233	2	797
NM_007236	0.10517358	-1.0508477	1.204118499	0.000565918	4234	2	1012
NM_004365	-0.5490166	-0.3758352	1.266217429	8.25E-05	1748	2	1610
XR_010293	-0.3862291	-0.1562092	1.421739005	0.0001368	4457	2	654
XR_010293	-0.5964836	-0.0874837	1.421739005	0.00208844	4392	2	1249
NM_030627	-0.0488905	-0.5932294	1.204433682	0.005722746	4430	2	1144
NM_080612	-0.1803484	-0.7545894	0.987237199	0.046268003	4654	2	1479
NM_212552	-0.2701773	-0.4566031	1.030263746	0.025335526	3477	2	743
NM_181782	-0.3059733	-0.3471097	0.9455959	0.033157995	3500	2	837
NM_001004196	0.04848882	0.00972632	1.27419931	0.01575632	4685	2	929
NM_006317	0.41973306	-0.6771738	1.564999705	0.000911518	4549	2	1276

NM_006317	0.50472047	-0.5377805	1.564999705	0.000633092	4550	2	1198
NM_018064	0.17425636	-0.7409134	1.511589755	0.000342927	4511	2	923
NM_018064	-0.3844666	-0.6881055	1.511589755	0.001821793	4393	2	1086
NM_016041	0.54057042	-0.6092807	1.537322213	0.003546769	4560	2	1348
NM_007277	-0.3045891	-0.6547504	1.317412084	0.017972283	3478	2	1387
NM_007277	-0.4178737	-0.5640494	1.317412084	0.005184334	3479	2	1016
XM_496854	-0.3069965	-0.2594519	1.443920279	0.005479097	4412	2	641
XM_496854	-0.372178	-0.3366304	1.443920279	0.013641545	4410	2	1036
NM_022748	0.26242986	-0.7649348	0.833983021	0.0067259	4577	2	1139
NM_032343	0.05306529	-0.9742047	1.121974732	0.000575283	4175	2	822
NM_006979	-0.5509497	-0.5318203	0.918501224	0.010069258	1732	2	1434
AL831922	0.46519311	-0.0644297	1.74861124	0.046838042	5267	2	1757
NM_015367	-0.8551244	-0.5120826	1.42242789	0.013248145	1726	2	1622
NM_015367	-0.4310287	-0.498615	1.42242789	0.013104681	4394	2	1267
NM_174905	-0.3680047	-0.1489032	1.107732851	0.029988075	3513	2	974
XR_010481	-0.3662022	-0.6112302	1.510487119	0.00098507	4132	2	881
XR_010481	-0.3042186	-0.3211137	1.510487119	0.00791701	4411	2	917
XM_371835	0.06860147	-0.3759503	1.079332877	0.007760268	4346	2	507
A_01_P020061	-0.3858519	0.50925304	1.49729074	0.010933803	4948	2	1620
NM_024873	0.12802164	-0.3747321	1.283704924	0.002970175	3434	2	169
NM_005443	0.63830648	-0.9260999	1.185853242	0.000563434	4586	2	1397
NM_017728	0.24097198	-0.6348873	0.786809652	0.014191738	4181	2	782
NM_015954	0.41080909	-0.2347568	1.236735828	0.005911334	3440	2	522
NM_015954	0.19599465	-0.1139732	1.236735828	0.0466492	3432	2	940
NM_003810	-0.547293	-0.2511381	0.932486631	0.041579589	3504	2	1211
NM_032837	0.63649651	-0.5249138	1.313355647	0.008908543	4563	2	1332
NM_032837	0.64569101	-0.240596	1.313355647	0.010620753	4564	2	1320
NM_013402	-0.7763959	-0.0687603	1.006256302	0.020686931	3505	2	1400
XR_014670	-0.3023517	-0.1808344	1.766757233	0.001271806	4466	2	1493
XR_014670	-0.2149305	0.09190127	1.766757233	0.001605365	4469	2	1450
NM_013281	-0.8401885	0.36458971	1.796832079	0.002042293	4878	2	1732
NM_014060	0.10375779	-0.8324582	1.494719862	3.31E-05	4285	2	787
NM_014060	0.05501687	-0.7388745	1.494719862	8.43E-05	4284	2	583
CB550069	0.21053762	-0.2937925	1.290650531	0.001042852	4965	2	173
CB550069	0.36884266	-0.0758087	1.290650531	0.000509348	4967	2	497
NM_022842	0.28290638	-0.4518374	1.012269792	0.002192126	2971	2	147
XR_013163	-0.2834296	-0.9760246	1.351825252	0.00023205	3993	2	1189
XR_013163	-0.5089844	-0.5498528	1.351825252	0.000670742	4149	2	866
NM_013446	-0.645278	0.31746838	1.498039542	0.016462547	4882	2	1630
NM_013446	-0.3905	-0.1600483	1.498039542	0.0011021	4458	2	891
CK230426	-0.0983351	-0.5599064	1.285999777	1.32E-05	4220	2	29
CK230426	0.00053644	-0.4911991	1.285999777	2.67E-05	4221	2	18
NM_019027	-0.473117	-0.8544778	1.247236428	0.005971981	3900	2	1313

NM_001655	0.06444566	0.00713011	1.252778043	0.004638218	4962	2	444
NM_176783	-0.0375949	-0.6016631	1.214875798	0.000243365	4219	2	51
NM_176783	-0.2019939	-0.7803823	1.214875798	7.49E-05	4207	2	254
XR_013105	0.21968089	-0.2506584	1.748022477	0.001141897	4521	2	1247
NM_014038	0.3030535	-0.3258842	1.276723464	0.001365907	4561	2	418
NM_014038	0.37691806	-0.285638	1.276723464	0.003548656	3439	2	414
NM_006092	-0.5224196	-0.4048378	1.614955809	0.003211092	4793	2	1365
NM_006092	-0.0440646	-0.2747459	1.614955809	0.000125891	4490	2	736
NM_003403	0.02481652	-0.9580036	1.407016739	0.001100007	4050	2	1275
NM_003576	-0.2105792	0.34884168	1.321174916	0.00581028	4949	2	1299
NM_003576	-0.209227	0.78475383	1.321174916	0.009476704	4931	2	1680
NM_024580	0.50101545	-0.9774115	1.533997031	0.003447117	1213	2	1603
NM_004092	-0.095284	-0.3470292	1.466186157	0.000287552	4444	2	322
NM_004092	-0.1029555	-0.3442838	1.466186157	0.00016444	4443	2	284
NM_014791	0	-0.2552748	1.143378683	0.023769503	3490	2	637
NM_138338	-0.6687651	-0.6040414	1.258230839	0.011056928	4719	2	1623
NM_138338	-0.0703607	-0.2974494	1.258230839	0.020348893	4479	2	832
A_01_P016657	-0.0979897	-0.6306408	1.575212855	0.022602844	1245	2	1563
A_01_P016657	-0.1270511	-0.5877574	1.575212855	0.02675733	1246	2	1552
NM_018075	0.55748893	-1.1956277	1.355392937	0.000576492	4583	2	1629
NM_004052	0.33453826	-0.3136938	1.425638955	0.000362732	4494	2	425
NM_004052	0.14974252	-0.215515	1.425638955	0.001127997	4492	2	282
NM_004823	-0.0381923	-0.7163841	1.293376132	0.001393826	4217	2	445
NM_004823	0.00320459	-0.5676496	1.293376132	0.002871379	4478	2	380
NM_015995	0.1196297	-0.3212512	1.059819791	0.008934982	3519	2	183
NM_015995	-0.1257384	-0.4232554	1.059819791	0.004731791	3518	2	126
NM_032906	-0.4038773	-0.2161162	1.310761194	0.010777976	4409	2	771
NM_032906	-0.3760677	-0.101954	1.310761194	0.043875113	4866	2	1554
NM_002587	0.30677042	-0.684899	0.909314824	0.016055072	4182	2	1095
NM_002587	0.14803864	-0.7585885	0.909314824	0.010781537	4575	2	1152
NM_003217	-0.395278	-0.3663308	1.289855807	0.000727991	2748	2	299
NM_003217	-0.367792	-0.5136709	1.289855807	0.001124181	4330	2	449
NM_170784	-0.2036762	-0.4441606	1.522016281	0.008773563	4396	2	1212
NM_170784	-0.0851544	0.45665649	1.522016281	0.009828869	4932	2	1599
NM_006066	-0.1620222	-0.7375314	1.259203626	8.54E-05	4208	2	199
NM_031311	-0.4229129	-0.7508111	1.360137547	9.03E-05	4148	2	744
NM_031311	-0.472848	-1.0198264	1.360137547	0.000877884	665	2	1408
NM_031311	-0.3269721	-0.5438433	1.360137547	0.000583487	4332	2	323
NM_020190	-0.0368833	-0.5424692	1.333250896	0.00067521	4222	2	189
NM_020190	-0.1152495	-0.7854538	1.333250896	0.00626188	4254	2	950
NM_002093	0.14980338	-0.6242856	1.115369162	0.007555807	4075	2	500
NM_002093	0.31899169	-1.1459856	1.115369162	0.027046418	564	2	1674
NM_031455	-0.4004222	-0.1002189	0.999601007	0.017302259	4686	2	1734

NM_145045	0.15157539	-1.1308627	1.394376737	0.005184312	4046	2	1595
NM_145045	0.39185168	-0.9211944	1.394376737	0.002044103	4047	2	1384
XR_012757	-0.4299387	0.14509679	1.053580694	0.040107538	3516	2	1213
XR_012757	-0.2107648	0.07015226	1.053580694	0.032957483	3561	2	855
NM_003808	0.11759761	-0.6172522	1.513697273	1.28E-06	4291	2	348
NM_003808	0.00925332	-0.8163508	1.513697273	3.15E-05	4283	2	695
NM_003808	0.19265313	-0.5600901	1.513697273	2.20E-06	4292	2	335
NM_003808	0.32952099	-0.5741843	1.513697273	4.72E-06	4294	2	552
NM_006384	-0.013027	-1.0121618	1.163496462	0.001172544	4186	2	1135
NM_012310	0.82022444	-1.0004607	1.54755089	0.003890375	1167	2	1667
NM_007117	0.74418465	0.61828362	1.666237548	0.040848879	4988	2	1741
NM_007117	0.67977515	1.27006771	1.666237548	0.048934745	5069	2	1773
NM_005340	-0.0066695	-0.3340341	1.511269262	9.85E-05	4488	2	207
NM_005340	0.09519573	-0.1460433	1.511269262	0.000108609	4493	2	411
XR_011344	-0.09219	-0.8625899	1.419236189	0.000250878	4257	2	684
XR_011344	0.00959037	-1.0824491	1.419236189	0.001307474	4252	2	1333
NM_001002234	0.31026855	-0.9759982	0.982118596	0.003485409	4185	2	1204
NM_003255	-1.1120138	-0.0130826	1.319486558	0.027965864	1765	2	1708
XR_014283	0.00509661	-0.6648322	1.363156032	0.017343413	4045	2	1359
XR_014283	-0.2850596	-0.8739442	1.363156032	0.00044492	4144	2	983
CB550573	-0.0946395	-0.6091361	1.314615509	0.000917829	4334	2	191
CB550573	-0.0601487	-0.3763108	1.314615509	0.00125793	4301	2	86
XR_010580	-0.1289129	-0.8488555	1.471114662	0.026888261	5072	2	1722
NM_012216	0.99803477	-0.5459684	1.527905712	0.004122945	4566	2	1687
NM_012216	0.62920663	-0.3555869	1.527905712	0.0043417	4551	2	1481
NM_003872	0.04019272	-1.0399245	1.342688258	0.003365265	4249	2	1382
NM_170705	0.23998723	-0.5205873	1.380517289	0.000997112	4299	2	477
NM_170705	0.05938569	-0.3229316	1.380517289	0.001197424	4302	2	167
NM_001069	-0.1666571	-1.0153689	1.381880291	0.000757159	4026	2	1264
NM_001069	-0.0914054	-0.8937364	1.381880291	0.000581769	4225	2	946
NM_001069	-0.0390882	-0.8565168	1.381880291	0.000485127	4226	2	851
NM_001069	-0.0261453	-0.6985958	1.381880291	0.000372952	4218	2	389
XR_013400	0.10522743	-0.4166035	1.53846941	0.000469632	4510	2	433
XR_013400	0.07921314	-0.4567034	1.53846941	0.005285877	4509	2	774
NM_024298	-0.1497758	-0.9016356	1.225133154	0.009225877	738	2	1569
NM_024298	-0.1343264	-1.0406781	1.225133154	0.006909332	740	2	1442
NM_002556	0.0518377	-0.5180813	0.963962709	0.029986585	2846	2	825
NM_002556	-0.1102202	-0.0929784	0.963962709	0.046220625	3520	2	647
NM_003492	-0.0493132	-0.5364789	1.243519228	0.007840434	4477	2	639
NM_003492	-0.5577085	-0.75913	1.243519228	0.00482552	4130	2	1334
NM_032936	-0.1662291	0.19850743	1.355287447	0.016491895	4944	2	1186
NM_032936	-0.0324653	-0.091334	1.355287447	0.007600296	3564	2	642
NM_001768	-0.224179	-0.0781769	1.472001877	0.000757407	4461	2	503

XR_013476	-0.3117945	-0.7585042	1.44592893	8.67E-05	4131	2	770
NM_015053	0.1613384	0.06404855	1.08048721	0.026923486	4903	2	726
CB550386	0.26430993	-0.8378316	1.355775146	0.004146143	4261	2	1073
NM_004915	-0.1213528	-0.1899904	1.27851102	0.034915374	4480	2	1007
NM_004915	0.27301991	-0.1454362	1.27851102	0.040638589	4481	2	1165
AK094316	-0.2090188	-0.6803702	1.146899966	0.006115096	3475	2	714
NM_033281	-0.4672569	0.03705638	1.070772065	0.040002055	4864	2	1452
NM_024523	-0.4210216	-0.5268791	0.981520294	0.009041279	1738	2	1119
NM_152302	-0.4546508	-0.5266894	0.980073641	0.013187063	1739	2	1342
XR_014008	-0.4971411	0.03218286	1.721484145	0.000288992	4467	2	1436
NM_015645	0.36523811	-0.4795624	0.93123476	0.045352202	4592	2	1317
CN643443	-0.7419601	0.46224718	1.763106369	0.008733188	4883	2	1728
CN643443	-0.9104368	0.36919398	1.763106369	0.005796818	4877	2	1742
NM_000282	-0.0255789	0.10232997	1.253026103	0.013019874	3565	2	699
NM_018999	-0.7085109	-0.2022084	1.105428432	0.013419808	1734	2	1539
NM_004725	0.23264832	-0.4305865	1.608724978	0.002150535	4498	2	1026
NM_004725	0.23657999	-0.1659005	1.608724978	0.003945947	4499	2	1065
NM_033027	0.00191248	-0.3154194	1.506282792	9.21E-05	4304	2	301
NM_033027	0.20139058	-0.2837127	1.506282792	0.000172769	4306	2	394
NM_003187	0.68790744	0.25458279	1.371078793	0.002982333	4977	2	1551
NM_003187	0.69483275	0.53386671	1.371078793	0.040533685	4986	2	1689
NM_003003	-0.2342254	-0.8672942	1.452653954	5.68E-05	4154	2	1039
NM_003003	-0.2112628	-0.620975	1.452653954	4.27E-05	4150	2	404
NM_007217	-0.0508525	-0.7583433	1.565594529	0.002947594	747	2	1207
NM_002764	0.80736859	-1.1334415	1.150093188	0.003362018	5100	2	1749
NM_024612	-0.4557639	-0.7442834	1.221162263	0.001280386	1812	2	1193
NM_000166	0.49601173	0.23080894	1.35385838	0.026459315	4976	2	1515
NM_000166	0.14224281	-0.0456128	1.35385838	0.000352711	4968	2	306
NM_020387	-0.5170738	-0.4598752	1.274626642	0.01267935	3482	2	1300
NM_020387	-0.6695611	-0.2635159	1.274626642	0.030458614	4757	2	1528
NM_005554	-0.1081576	-0.8854349	1.099450061	0.009990809	4074	2	1265
NM_033087	0.49684862	0.39092164	1.512120395	0.000512639	4985	2	1643
NM_016052	0.45531057	-0.4769491	1.571764872	0.0336755	5073	2	1745
NM_015450	-0.2575239	-0.7679315	1.16065234	0.009374054	4326	2	1246
NM_005627	0.49626098	-1.0392559	1.439279183	6.90E-05	4318	2	1433
NM_002095	0.28585738	0.39362921	1.442848741	0.001902681	4941	2	1420
NM_002095	0.03984207	0.49314143	1.442848741	0.001674905	4940	2	1494
CN641426	0.19801043	-0.3371396	1.515683993	0.006289703	4547	2	975
CN641426	0.260731	-0.596144	1.515683993	0.004473894	4264	2	1029
CN641426	0.30341885	-0.5107223	1.515683993	0.006338048	4535	2	1355
NM_018145	-0.1560879	0.49986979	1.8073414	0.021468192	4958	2	1696
NM_004060	-0.4616857	-0.7002502	1.493799035	0.001036414	4153	2	1285
NM_024611	0.43570262	-0.3279509	1.010803174	0.032949612	4593	2	1302

NM_004099	0.22636765	-0.8390988	1.574559093	0.000130709	956	2	1127
NM_004099	0.47115916	-0.9742311	1.574559093	2.15E-06	4321	2	1415
NM_014547	-0.0892931	0.40786611	1.199524401	0.037963184	4933	2	1371
NM_014547	-0.0417075	0.51035859	1.199524401	0.049064014	4934	2	1537
NM_005428	0.15258433	-0.6222591	1.579226837	4.31E-05	4293	2	631
NM_005428	0.0060976	-0.6513668	1.579226837	9.68E-05	4289	2	709
XR_014059	-0.4388125	-0.9253679	1.36201154	0.000550368	4723	2	1695
NM_017917	0.12131996	-0.0242597	1.347531776	0.012665827	4964	2	738
NM_001007279	0.47582935	-0.3336473	1.243761802	0.003280335	4966	2	801
NM_001007279	-0.0840638	-0.3329272	1.243761802	0.002170823	4338	2	119
NM_001007279	0.39405127	-0.3716724	1.243761802	0.002699011	3437	2	467
NM_001007279	0.3133026	-0.3481901	1.243761802	0.010275305	3436	2	496
NM_016144	0.36510794	-0.6921061	0.904978835	0.027426536	4161	2	1292
NM_024324	0.50441737	-0.5471473	1.269316266	0.019107359	4590	2	1498
NM_024324	0.52915336	-0.5139816	1.269316266	0.021131089	4591	2	1489
NM_024324	0.21391276	0.01349761	1.269316266	0.04928492	4970	2	1283
NM_024324	0.50237558	-0.4709027	1.269316266	0.006712888	4300	2	1131
NM_007256	0.26399605	-0.5475144	1.674581346	8.69E-06	4295	2	885
NM_007256	0.38342571	-0.3999561	1.674581346	3.45E-05	4554	2	1101
XM_377713	-0.0234657	-0.1406037	1.356701041	0.009473301	3560	2	1004
NM_003930	0.30082269	-1.1366938	1.168899341	0.011887602	565	2	1618
NM_000572	-0.3559644	-0.6409643	0.819553102	0.034807852	4821	2	1069
NM_000194	0.36910046	-0.7666509	1.529711828	0.002754358	4263	2	1280
NM_152828	0.79428065	-0.327087	1.658771141	1.07E-06	4567	2	1634
NM_152828	0.37654367	-0.373273	1.658771141	8.10E-05	4555	2	1006
NM_203298	-0.1024842	-0.4657216	1.442330207	0.001331683	4345	2	357
NM_203298	-0.1561361	-0.4534925	1.442330207	0.003163401	4331	2	853
NM_004261	0.07993013	-0.6356008	1.497121824	0.00218822	4506	2	675
NM_004261	-0.1567207	-0.8866158	1.497121824	0.012395502	682	2	1527
NM_017994	0.12756708	-0.2854756	1.571744989	0.002003727	4495	2	772
NM_017994	0.49543655	0.06989876	1.571744989	0.006127739	4981	2	1423
NM_005746	-0.4921607	-0.507612	1.351478821	0.023101476	1728	2	1447
NM_018149	0.22582345	-0.3100878	1.465380586	0.005808216	3435	2	701
CN802384	-0.0188071	-0.5305319	1.659941904	0.003271351	4508	2	1099
CN802384	-0.0716832	0.01977614	1.659941904	0.019411933	5014	2	1631
NM_004663	-0.9059344	-0.7449318	1.41952138	0.00525806	42	2	1780
NM_004663	0.30456746	-0.419014	1.41952138	0.006996689	3438	2	915
NM_006405	0.32936973	-0.3584014	1.498839613	0.000453033	4552	2	792
NM_006405	0.20169959	0.048871	1.498839613	0.000469438	4969	2	854
NM_002081	0.10001659	-1.0327872	1.110936995	0.002460129	4187	2	1266
NM_032315	-0.241862	0.31588736	1.310642255	0.031411888	3566	2	1461
NM_172220	1.7826086	0.75402678	1.912098779	0.033303886	5268	2	1795
NM_172220	0.38530307	0.92966541	1.912098779	0.025490508	5067	2	1755

NM_172220	0.33583801	1.148692	1.912098779	0.023435172	5068	2	1765
NM_001615	-0.3078757	-0.1399683	1.519410692	0.000277644	4446	2	900
NM_001615	-0.2272934	-0.1584285	1.519410692	7.14E-05	4447	2	518
NM_173647	-0.0095407	0.78634042	1.951044675	0.022843345	5060	2	1744
NM_173647	-0.0997993	0.9853611	1.951044675	0.020377472	5061	2	1753
NM_003908	0.10016498	-0.7941962	1.333697124	0.002390715	4260	2	696
NM_003908	0.08335615	-0.4114155	1.333697124	0.002005518	4348	2	239
NM_031937	0.02463171	-1.0020126	1.243332096	0.004715484	4038	2	1405
NM_002139	-1.0310024	-0.0217547	1.296116183	0.006190525	1768	2	1730
NM_002835	0.0076755	-0.5885263	1.598047436	0.013659458	5156	2	1429
NM_004475	-0.7807652	0.01785483	1.172649188	0.045543764	4756	2	1583
NM_004475	-0.4834779	-0.1628882	1.172649188	0.014320597	3512	2	985
NM_032869	0.10882979	-0.9436631	1.068559469	0.014691285	4159	2	1367
NM_032869	0.09446509	-0.1846271	1.068559469	0.03720693	3562	2	986
CB228320	-0.4776155	-0.3540585	1.336334852	0.01587689	3483	2	1394
NM_005216	-0.1926381	0.43349152	1.659596085	5.90E-05	4950	2	1558
NM_005216	0.2058437	0.26680726	1.659596085	0.003879725	4938	2	1490
NM_005216	-0.037302	0.66027158	1.659596085	0.000779367	4951	2	1673
NM_005216	0.2118569	0.62181998	1.659596085	0.000940162	4943	2	1660
NM_021242	-0.2842873	-0.3208277	1.444577702	0.00252423	4440	2	812
NM_021242	-0.4788678	-0.4213444	1.444577702	0.000409449	2749	2	803
NM_002965	-0.0168727	1.19724725	2.115115917	0.012143255	5063	2	1775
NM_002965	-0.1123351	1.17550605	2.115115917	0.011810203	5062	2	1772
XR_011062	0.03631198	-0.3675356	1.626831358	0.000251302	4445	2	730
XR_011062	0.11800148	-0.5763434	1.626831358	4.56E-05	4290	2	694
CN801997	0.10470227	-0.8320889	1.220768067	0.011594179	4165	2	1319
NM_001790	0	-0.6186055	0.988777524	0.022558832	4180	2	902
NM_024760	0.45003338	-0.8334654	1.270291892	0.002441836	4191	2	1125
NM_002729	-0.1644072	-0.680695	0.930810196	0.037187409	4160	2	1234
NM_004280	0.00477037	-0.8007413	1.347249857	0.012209504	4076	2	1284
NM_052853	0.01015333	-0.4563312	1.527966602	0.002199888	4303	2	789
NM_018135	-0.4903754	-0.4102479	1.234805426	0.009360833	4328	2	1031
NM_080631	-0.4525313	-0.5945803	1.166281066	0.018097863	4329	2	1049
NM_003659	-0.3260127	-0.795439	1.113185525	0.00529403	4051	2	1545
NM_198066	-0.0140791	-0.570661	1.303069861	0.00371976	4335	2	429
NM_004705	-0.6020273	-0.7131552	1.150655348	0.008744438	4327	2	1360
NM_004705	-0.1243446	-0.4074382	1.150655348	0.015000619	4336	2	367
XR_013392	-0.0304734	-0.8861916	1.065922681	0.014033062	4164	2	1362
XR_013392	-0.0927731	-0.5321852	1.065922681	0.039616708	3568	2	1339
NM_006029	-0.8892986	-0.7690262	1.403705411	0.001061941	4725	2	1718
NM_006029	-0.250919	-0.3494497	1.403705411	0.001643562	4337	2	391
NM_001677	0.11886757	-0.033147	1.819686742	0.002087067	5025	2	1459
NM_001677	0.19077172	0.12367603	1.819686742	0.001385676	5027	2	1485

NM_001677	0.14518705	-0.0817991	1.819686742	0.015074741	5024	2	1576
NM_001677	0.16980083	0.15517133	1.819686742	0.007586003	5028	2	1557
XR_013560	-1.0077933	-0.2263779	1.380833148	0.011912929	4752	2	1666
XR_013560	-0.9552857	-0.1915473	1.380833148	0.012274361	4753	2	1648
XR_013560	-0.7866425	-0.2428162	1.380833148	0.006447636	4755	2	1514
XR_013560	-0.8509265	-0.1244913	1.380833148	0.011220587	4754	2	1587
XR_013663	0.06144069	1.57311823	1.842958027	0.000365173	5065	2	1782
XR_013663	0.17118024	1.62527913	1.842958027	0.004887511	5066	2	1784
NM_002946	-0.0603098	0.21531669	1.731781654	0.005951835	5026	2	1504
NM_181836	0.08661507	-0.8074832	1.508091027	0.004668445	745	2	1278
NM_005765	0.12218187	-0.5801394	1.639246023	0.000252852	4512	2	954
NM_005765	0.15717229	-0.5856169	1.639246023	0.001211392	4513	2	1088
NM_024099	0.24765303	-1.0493582	1.369538881	0.000699726	4190	2	1329
NM_024099	0.02034766	-0.9705741	1.369538881	0.00083322	4188	2	1130
NM_006855	0.65568037	-0.5458931	1.609509779	0.000786597	4565	2	1475
NM_024006	0.03553346	-0.8277801	1.412622071	0.015982152	4656	2	1534
NM_024006	-0.157356	-0.770429	1.412622071	0.014616917	4655	2	1480
NM_002468	-0.0309007	-0.6634668	1.433986773	0.003563935	4339	2	763
NM_002468	-0.0476068	-0.5718011	1.433986773	0.001803398	4342	2	491
NM_002984	0.07842848	-0.8141614	1.284682822	0.002856008	4189	2	863
NM_002984	0.34703834	-0.8092472	1.284682822	0.002659173	4194	2	1172
NM_007198	-0.0007771	-0.6206135	1.152915374	0.014109342	4822	2	845
NM_007198	-0.1547554	-0.3609859	1.152915374	0.018486954	4052	2	1414
NM_000887	0.34993667	-0.4431529	1.502795996	0.009631782	4544	2	1316
NM_000887	0.02712806	-0.2791951	1.502795996	0.008992635	4543	2	1102
NM_024018	-0.1368918	0.25450377	1.459895243	0.044198319	4945	2	1505
NM_031300	-0.5646451	-0.8211124	1.224639521	0.02307009	1809	2	1655
NM_170725	0.04982796	-0.0594602	1.342657529	0.017589743	4901	2	1093
NM_005168	0.22887082	-0.6752346	1.552165133	0.006676199	4534	2	1424
NM_005168	-1.2201401	-0.2648146	1.552165133	0.016494601	227	2	1781
XR_013023	-0.3136492	-0.7125254	0.892880054	0.049056198	4820	2	1492
NM_015465	-0.2140757	0.43203683	1.620445845	0.032507212	4955	2	1640
NM_001001410	-0.0503333	-0.3130222	1.17391014	0.02248089	4898	2	706
NM_001001410	0.04204016	-0.0738053	1.17391014	0.046265392	4902	2	1044
CN647374	-0.4029546	-0.4926463	1.585765927	0.003725543	4792	2	1351
CN647374	0.05980203	-0.0870159	1.585765927	0.00619684	4496	2	1115
CN647374	-0.3893718	-0.323977	1.585765927	0.007700528	4888	2	1298
CN647374	-0.1648473	-0.1637649	1.585765927	0.00804184	4889	2	1107
NM_022343	-0.0035169	-0.5882815	1.431760303	0.004333093	4343	2	734
NM_022343	0.02894186	-0.1934892	1.431760303	0.004244653	4349	2	758
NM_002819	-0.6492246	-0.7394476	1.320909224	0.004707156	4747	2	1478
NM_002819	-0.4770584	-0.6139783	1.320909224	0.004792921	4748	2	1117
NM_001746	0.58115616	-0.0866642	1.877115762	0.00033102	5032	2	1594

DR767858	-0.4774774	-0.4898543	1.31468949	0.013959005	4749	2	1288
DR767858	-0.2532688	-0.4296024	1.31468949	0.016815878	4750	2	1120
NM_032342	-1.3683575	-0.2670465	1.42272222	0.005171007	4716	2	1758
NM_032342	-0.3050097	0.42292245	1.42272222	0.042865212	4947	2	1562
NM_017625	0.0453365	1.3940094	1.988324662	0.005054988	5064	2	1779
NM_019026	-0.3416987	-0.210453	1.687667265	0.003176151	5013	2	1448
NM_019026	0.30379203	0.06103836	1.687667265	0.001559284	5023	2	1519
NM_007167	0.47400967	0.05001869	1.817217506	0.005542956	4915	2	1641
NM_007167	0.24491522	0.36389294	1.817217506	0.018830175	4987	2	1699
XM_087254	-0.4814335	-0.0532513	1.589249646	0.008630027	5012	2	1541
XM_087254	-0.4487607	0.52848173	1.589249646	0.038376113	4952	2	1685
NM_020548	0.47068339	0.02163828	1.959554541	1.48E-05	5031	2	1604
NM_005793	-0.1032371	-0.9345296	1.127577797	0.029895641	4072	2	1578
NM_005793	-0.0431718	-0.8043149	1.127577797	0.036456248	4073	2	1531
NM_017832	-0.3873402	-0.9106954	1.421546278	0.00591002	4021	2	1523
NM_001562	0.26248485	-0.5963363	1.172720741	0.021988879	4895	2	1168
NM_004859	-0.0626634	-0.3778803	1.296094003	0.02717072	4899	2	1170
NM_004859	0.25140652	-0.0936065	1.296094003	0.046094646	4908	2	1345
NM_006805	-0.2553262	-0.0486831	1.591918186	0.019342236	4916	2	1574
NM_006805	0.20444613	-0.3229551	1.591918186	0.011016208	4537	2	1413
NM_004568	-0.4498137	0.44984704	1.526106171	0.025840568	4946	2	1644
NM_030800	0.04319913	-0.0608571	1.489411393	0.017199388	4906	2	1227
NM_030800	-0.0124497	0.30280408	1.489411393	0.025389481	4918	2	1559
A_01_P013097	-0.7038646	-0.1812895	1.516383664	0.00865961	1769	2	1672
NM_014385	0.0407049	-0.2350783	1.649823719	0.03671543	4536	2	1571
NM_138459	0.10659251	-0.6159191	1.46145047	0.006014062	4344	2	994
NM_138459	-0.2355784	-0.2307889	1.46145047	0.012230318	4350	2	892
NM_017413	0.21558688	-0.1553081	1.214799197	0.04959741	4900	2	1185
XR_013672	-0.5017156	-0.934635	1.387755055	0.008971003	1383	2	1764
XR_013672	0.00786382	-0.8007681	1.387755055	0.009446791	4193	2	1282
NM_201515	-0.3674535	-0.6069845	1.148792311	0.037292467	4824	2	1390
NM_003141	-0.2715285	-0.9138306	1.255675064	0.014003497	4020	2	1542
NM_003141	-0.8109786	-0.4078294	1.255675064	0.033989324	4758	2	1691
CK230576	0	-0.3135347	1.515592845	0.014288882	4904	2	1209
CK230576	-0.7152191	-0.3378232	1.515592845	0.01020305	4760	2	1677
NM_138417	0.13632988	-0.9048884	1.241037142	0.012574744	4192	2	1440
NM_138417	-0.7341176	-0.7320697	1.241037142	0.016245988	4745	2	1613
NM_138358	0.17875965	-0.6709779	1.527270974	0.005571349	4538	2	1323
NM_138358	-0.2220209	-0.5402486	1.527270974	0.004793444	4340	2	1059
NM_000747	-0.5355755	-0.208919	1.560333945	0.02184407	4885	2	1580
NM_004987	0.06678532	-0.3966879	1.548783262	0.014402719	4907	2	1517
NM_203391	-1.2825298	-0.3372189	1.524123505	0.018107765	4717	2	1751
NM_203391	-0.2147206	-0.1979831	1.524123505	0.026995789	4890	2	1483

NM_005214	1.02184634	-0.3033129	1.556628632	0.021429304	5071	2	1727
NM_153690	0.15440058	-0.0881374	1.487485965	0.042224986	5070	2	1717
NM_032311	-0.3672988	0.26451271	1.881328784	0.006544873	4917	2	1709
NM_015187	-0.0243502	-0.6259129	1.437645865	0.022290648	4823	2	1506
NM_015187	0.49597797	-0.3597636	1.437645865	0.032544233	4910	2	1614
NM_014399	0.10028745	-0.5064286	1.553980913	0.02368384	4892	2	1516
NM_012225	-0.070467	-0.9660765	1.1309223	0.049563755	4829	2	1694
XR_010577	-0.4138668	-0.4820534	1.502726067	0.025655858	4884	2	1624
XR_010577	-0.778965	-0.3251309	1.502726067	0.028933053	4751	2	1628
CN642065	0.35372482	-1.0739576	1.337702709	0.021618618	4832	2	1721
CN642065	0.04792581	-0.5978508	1.337702709	0.037131	4896	2	1497
CN642065	-0.1740428	-0.7697369	1.337702709	0.032369749	4825	2	1593
CN642065	0.27873751	-0.4543206	1.337702709	0.047091476	4909	2	1553
CN642065	-0.1065855	-0.5527098	1.337702709	0.0432868	4826	2	1509
CN806006	-0.5122313	-0.0361961	1.671173788	0.024492495	4887	2	1632
NM_013242	-0.49776	-0.2575231	1.577413005	0.029162362	4886	2	1612
NM_000452	1.09449496	-0.1905365	1.62674274	0.03014343	5092	2	1774
CK232451	1.08277335	-1.0719979	1.582908322	0.015640829	5101	2	1762
AY369856	0.14716859	-0.3454152	1.618253308	0.039828487	4897	2	1615
NM_152716	1.1362315	-0.8455567	1.718975121	0.047154883	5280	2	1786

Cluster 1

Gene Name	Av Normal	Av M (w12)	Av M (w4)	*P*	Hierarchical Clustering (order)	K-means clustering	K-means clustering (rank)
XM_208097	-0.6302376	-0.0519317	-1.569315353	0.009397583	6396	1	2129
XM_208097	-0.7567766	0.71254514	-1.569315353	0.004071999	6621	1	2110
NM_152474	0.08169561	1.18817129	-1.110657018	0.017798931	8956	1	1987
NM_178453	-0.4923529	0.50697678	-1.512434319	0.000942827	6864	1	2004
NM_001556	-0.8676133	0.53299678	-1.372071688	0.024705872	6019	1	2157
NM_024884	-0.3977009	0.60144944	-1.054856888	0.028158731	6392	1	2018
NM_033258	-0.6995001	1.17440255	-1.124380447	0.024051657	9736	1	2133
BX647378	-0.2676204	0.42806361	-1.414908454	0.040246784	6637	1	2067
NM_025045	-0.4092316	0.40434646	-1.040023003	0.040036616	7348	1	1779
NM_025045	-0.8981712	0.94107822	-1.040023003	0.025340221	8915	1	2100
NM_018418	-0.7014452	0.62263901	-1.571045142	0.044082462	6014	1	2184
NM_133474	-0.122684	-0.0325104	-1.34510028	0.027979663	6445	1	2016
NM_000553	-0.9473327	0.7197897	-1.385828853	0.003919017	6622	1	2117
NM_001692	-0.4572914	0.61044255	-1.38274721	0.006783643	6865	1	1918
NM_001692	-0.3927035	0.36030695	-1.38274721	0.003114335	6486	1	1852
NM_014224	0.40783317	0.16808908	-1.206234048	0.030827341	6170	1	1992
NM_030802	-0.554681	0.48815851	-1.405560073	0.013351397	6635	1	2006
NM_030802	-0.4620024	0.78622777	-1.405560073	0.006299045	6647	1	1926
NM_005649	-0.7235748	0.45202691	-1.428282256	0.004112433	6623	1	2119
NM_005919	-0.0178238	-0.2609866	-1.605851985	0.002237976	6449	1	2130

NM_005919	-0.512699	-0.4117437	-1.605851985	0.004645259	6407	1	2149
NM_152643	-0.2155565	1.14943773	-0.957770601	0.020235099	8869	1	1916
NM_178026	0.10093999	0.6556873	-1.005529493	0.029801794	6933	1	1574
NM_178026	0.17323587	0.38710382	-1.005529493	0.03554386	7327	1	1487
NM_032876	-0.4893616	0.23883339	-1.36180702	0.046754016	6633	1	2058
NM_032876	-0.4458856	0.88164763	-1.36180702	0.008229095	6648	1	1968
NM_207453	0.43702297	0.592843	-1.05697023	0.013250603	8797	1	1594
NM_005741	-0.3084918	0.08728845	-1.593352619	0.001715692	6461	1	2028
NM_178556	0.28608874	0.85343957	-0.872325982	0.045468129	8955	1	1814
NM_000756	-0.6447438	0.4468987	-1.036607964	0.017638808	6625	1	1545
NM_015343	-0.2431268	0.00807947	-1.493482776	0.001830375	6462	1	2005
NM_015343	-0.1241692	0.19066217	-1.493482776	0.000673132	6717	1	1875
NM_001671	0.01368334	-0.4479772	-1.607023853	0.02574663	6444	1	2151
NM_004076	-0.3804324	0.45584135	-1.425645299	0.002516636	6520	1	2036
A_01_P000370	-0.256552	0.79412145	-1.251769125	0.0045871	6769	1	1521
NM_001989	-0.9261506	0.31035314	-1.128416757	0.034909567	6022	1	2167
XR_012677	0.58274488	0.32274633	-0.909719871	0.039478679	7333	1	1790
NM_175733	0.17539415	0.87097169	-0.676793198	0.038865703	8902	1	1710
NM_004647	0.03153288	0.57605396	-0.991952798	0.016679428	7328	1	1538
NM_020847	-0.0099835	0.78733521	-1.079059099	0.037636029	8866	1	1891
NM_001632	0.0502668	0.74539439	-1.026458755	0.01195839	6934	1	1213
NM_002507	0.14499258	0.3874422	-1.027772176	0.010915327	7329	1	1104
NM_002507	-0.0072812	0.38592475	-1.027772176	0.010598567	7640	1	956
NM_144999	-0.1889327	0.55031125	-1.366585709	0.000252283	6791	1	1357
NM_198401	-0.0431398	0.82904492	-1.082679312	0.026426933	8959	1	1715
NM_198401	0.02949696	1.00235629	-1.082679312	0.017246993	8958	1	1934
NM_002075	-0.5786516	0.71977065	-1.395314916	0.000335566	6866	1	1860
NM_021933	-0.4785215	0.14461551	-1.254520584	0.000960414	6480	1	1727
NM_021933	-0.2677337	0.35679401	-1.254520584	0.000891355	6482	1	1305
NM_002824	0.17752735	0.430794	-1.157334311	0.020446707	8622	1	1657
NM_025141	-0.1225804	0.74503735	-0.837059513	0.049335136	8947	1	1507
NM_207477	0.36447342	0.17697813	-0.965590371	0.026512187	7330	1	1575
NM_181532	-0.2729968	0.49493819	-1.176803614	0.011968352	6021	1	2144
NM_024663	-0.467025	0.26265605	-1.209860259	0.002935347	6473	1	1640
NM_024663	-0.9982585	0.5573516	-1.209860259	0.005087126	6619	1	2068
NM_014743	0.43652756	1.13353811	-0.858278834	0.005738491	8830	1	1740
NM_172027	-0.5435709	4.92E-06	-1.57918007	0.000967325	6459	1	2095
NM_198464	-0.1660041	0.111924	-1.126676375	0.03223322	6493	1	1747
NM_020944	-0.0876971	0.81611025	-1.174654313	0.000592892	6919	1	969
NM_020944	-0.1528744	1.11271333	-1.174654313	0.000997742	7085	1	1739
BM423165	-0.4691401	0.29428353	-1.191340555	0.003106049	6624	1	1874
NM_013360	-0.5195485	0.88036324	-1.171565349	0.001802566	6766	1	1595
NM_024517	-0.1552362	0.48870915	-1.415572334	0.000598827	6806	1	1760

XM_371614	-0.540402	0.45462043	-1.405033927	0.000119631	6750	1	1771
XM_371614	-0.5354773	0.63122748	-1.405033927	0.000334719	6754	1	1789
XM_371614	-0.3713477	0.90032976	-1.405033927	0.00259368	6650	1	1838
NM_152777	0.34799736	0.82432873	-0.739484677	0.044907395	8852	1	1726
NM_001005492	0.11554651	-0.2794042	-1.309526985	0.007284741	6450	1	2054
NM_001005492	0.1366146	0.05300684	-1.309526985	0.00304206	6456	1	1770
NM_001684	0.09971784	0.62151426	-1.390890106	0.013257361	7210	1	1865
XM_498462	-0.5955567	1.0295168	-1.210623339	0.003456084	6770	1	1876
XM_498462	-0.4831762	0.97869992	-1.210623339	0.01020836	6654	1	1907
NM_005341	-0.2646136	0.63009276	-1.238768467	0.001280609	6914	1	1275
NM_032874	0.40391428	-0.5250739	-1.465750182	0.024921789	6506	1	2153
NM_032874	0.2871885	-0.5094749	-1.465750182	0.025075598	6505	1	2150
NM_016143	-0.2706566	0.94095174	-1.206511196	0.000434353	7084	1	1571
NM_001008568	-0.1972518	0.35909585	-1.341804222	0.000285903	6469	1	1460
NM_152856	-0.0535174	0.69508095	-1.292775166	0.003012113	6804	1	1309
NM_017916	0.04850209	0.31939779	-1.415783132	8.80E-05	6720	1	1586
NM_017916	0.07477451	0.26386522	-1.415783132	0.000829833	6721	1	1703
NM_152375	0.4866393	0.86168965	-0.860502637	0.006719921	8828	1	1334
NM_152375	0.34968956	0.95719142	-0.860502637	0.037716737	8962	1	1850
NM_000155	-0.3525482	0.30858661	-1.326911329	0.000202603	6481	1	1576
NM_001001974	0.10217974	1.01479172	-0.980997754	0.023107532	8961	1	1848
NM_015115	0.06434981	1.14390675	-0.995164404	0.002538455	7205	1	1481
NM_030915	-0.4817571	0.5140873	-1.362062855	0.002550808	6752	1	1752
NM_030915	-0.5565211	0.6896532	-1.362062855	0.000561836	6755	1	1735
NM_174891	-0.3733902	0.63228589	-0.680269254	0.039236468	7514	1	1441
NM_145296	-0.1437776	-0.226022	-1.538471564	0.005175309	6447	1	2118
NM_021944	-0.550965	0.58356898	-0.917362075	0.034373734	6035	1	2168
NM_018836	-0.6957304	0.6010635	-1.487856612	0.002228522	6020	1	2143
NM_033624	-0.2656972	1.05716892	-1.168097765	0.001470319	6774	1	1532
NM_015885	-0.3397764	1.07714761	-1.160483161	0.016148977	8950	1	1960
NM_016044	0.08885658	0.43955743	-1.068295505	0.001556719	7666	1	609
NM_016044	0.23025952	0.69706683	-1.068295505	0.000747032	7685	1	681
NM_017777	0.46854404	0.25111051	-1.235607602	0.01671244	7310	1	1859
NM_002773	-0.206015	0.13945187	-1.310854413	0.000567271	6476	1	1613
NM_002773	-0.1488319	0.58857066	-1.310854413	8.39E-05	6792	1	1091
NM_198699	0.11456189	0.85627529	-1.089937203	0.000853695	8258	1	985
NM_021140	-2.1159383	0.38103931	-1.484898814	0.008096572	6032	1	2158
NM_003436	0.07420081	0.51216813	-1.16317373	0.035168145	6613	1	1808
NM_003436	-1.1431814	0.99867425	-1.16317373	0.026138493	6045	1	2174
NM_005183	-0.4171251	0.66442302	-1.091254664	0.043345261	6107	1	1956
NM_005938	-0.1495486	0.63943688	-1.136585713	0.002284316	6917	1	931
NM_005938	-0.3192006	0.93405877	-1.136585713	0.000542367	6773	1	1055
NM_006001	-0.5899316	-0.6396471	-2.000226321	0.01829067	5950	1	2181

NM_006001	-0.3935643	-0.722794	-2.000226321	0.022686673	5951	1	2182
NM_002336	-0.8274592	0.76380426	-1.315396763	0.001518756	6660	1	2078
NM_003453	0.6670857	0.58228258	-0.931284246	0.007829757	8396	1	1695
NM_003453	0.00208892	0.56024611	-0.931284246	0.00923479	7342	1	1283
NM_020817	-0.3377796	0.32768779	-1.565186647	0.002027599	6518	1	2085
NM_052897	-0.1147213	1.10843812	-1.057424153	0.000639409	7204	1	1120
NM_003283	0.11676169	0.96185158	-0.930526165	0.001316353	7206	1	943
XR_010727	-0.1844098	0.86365966	-1.129339523	0.000168365	6918	1	800
XR_010727	0.11089644	1.02858807	-1.129339523	0.002423713	7198	1	1402
NM_033396	-0.3134288	1.02186725	-1.046458735	0.004014765	6949	1	1536
NM_018140	-0.7347419	0.91327947	-1.526225064	0.001603257	6641	1	2073
NM_002599	-0.0784466	0.43711878	-1.2790063	0.000595707	6793	1	1088
NM_002599	-0.1737077	0.52737047	-1.2790063	0.000486522	6794	1	1056
NM_007147	-0.3976538	0.19088494	-0.948407652	0.037147575	6391	1	2136
NM_007147	-1.3186748	0.22886142	-0.948407652	0.024289543	6029	1	2178
NM_015363	0.97316807	1.09885479	-0.607229576	0.013766201	9656	1	2089
NM_002864	-0.1490209	0.58705858	-1.278241679	0.000432357	6795	1	1030
NM_002230	-0.0287519	0.82858095	-1.226279576	0.000203002	7097	1	909
NM_015023	0.00430754	-0.0775863	-1.539572543	0.000286571	6452	1	2034
NM_015023	0.09889761	-0.1444021	-1.539572543	0.000253523	6453	1	2061
NM_173156	0.23122835	-0.2056027	-1.49256881	7.01E-05	6454	1	2074
NM_173156	0.04409019	-0.1401751	-1.49256881	0.000128779	6451	1	2066
NM_004830	-0.5655846	1.13405717	-1.390631371	0.013164426	6652	1	2128
NM_030792	-0.425173	-0.2704722	-1.222689959	0.000418059	6418	1	2017
NM_030792	-0.156224	0.22588818	-1.222689959	9.04E-05	6477	1	1282
NM_005859	-0.0237104	1.01844545	-1.150497572	5.60E-05	7146	1	995
NM_015051	-0.0440692	0.79561176	-1.220979811	0.026461141	10361	1	2086
NM_170707	-0.2116364	0.10044514	-1.310097738	0.00560941	6491	1	1822
NM_170707	-0.2774586	0.25431245	-1.310097738	0.005284549	6492	1	1756
NM_198149	-0.0958428	0.54429317	-1.389873371	0.000107368	6790	1	1376
NM_022727	-0.0967517	1.16741962	-1.048039639	0.00021235	7160	1	1245
NM_022727	-0.0100733	1.20839319	-1.048039639	0.000190187	7161	1	1278
NM_002073	0.39517589	0.49585688	-1.055652972	0.000853764	7684	1	1114
NM_002073	0.22648005	0.66536381	-1.055652972	0.005138809	7750	1	907
NM_001009820	-0.312298	1.11299763	-1.117053315	0.000664981	6950	1	1515
XM_088566	-0.5900585	0.81742726	-0.678339248	0.037354818	6669	1	1642
NM_175055	0.72148064	-0.0202866	-0.938162604	0.020588346	8904	1	2106
NM_024848	-0.5843574	0.782238	-0.515270653	0.042018475	7516	1	1561
NM_024848	0.32872077	0.80729517	-0.515270653	0.04421135	8903	1	1690
NM_015493	-1.0915321	0.58194774	-1.457707815	0.025012413	6616	1	2155
NM_015493	-0.7593569	1.09695808	-1.457707815	0.00087599	6642	1	2077
NM_018467	-0.5734819	0.87914835	-1.298358698	0.00128518	6867	1	1845
NM_145245	0.16898263	0.80571176	-1.003360945	0.009961732	8639	1	1281

NM_145245	0.08276904	0.84711897	-1.003360945	0.001408397	6921	1	655
NM_175918	0.3563265	-0.0145465	-1.266990758	0.003857668	7308	1	1885
NM_175918	0.67811672	0.01215897	-1.266990758	0.023352933	7309	1	2071
NM_033305	-0.4277183	0.85392804	-1.371657368	0.042346217	6033	1	2162
NM_201649	-0.2033774	1.07358682	-0.725053837	0.010202735	7524	1	1268
NM_021070	-0.5209301	0.88398333	-1.315493497	0.000713834	6868	1	1797
NM_017607	-0.0518275	0.73781691	-1.181224659	1.99E-05	7099	1	638
NM_002805	-0.0989048	0.98579641	-1.197623535	0.001262767	7172	1	1251
NM_002805	0.04739053	1.03504845	-1.197623535	0.002490806	7173	1	1489
NM_005407	-0.3745333	0.89702468	-1.200751563	0.000598466	6772	1	1336
NM_013326	-0.1213075	-0.0702239	-1.192725393	0.00449807	6455	1	1846
NM_013326	0.18135505	0.34908827	-1.192725393	0.009950008	7326	1	1546
NM_007139	-0.4833931	0.45028627	-1.325492353	0.030438733	6634	1	1983
NM_001783	0.4843783	0.32755845	-1.14213814	0.010639007	8710	1	1898
NM_016174	-0.3947271	0.56546259	-1.212815325	0.000811628	6818	1	1328
NM_016174	-0.2675012	0.64816794	-1.212815325	0.000137339	6797	1	910
NM_003250	-0.4027131	0.57250125	-0.750193931	0.041618565	6023	1	2027
NM_014494	-0.3144947	0.47801227	-1.466608845	0.011134275	6775	1	1932
NM_054020	-1.8540632	0.34638968	-1.302312542	0.009131847	6008	1	2185
NM_006782	0.20854293	0.78918588	-0.834274877	0.007925362	8849	1	762
NM_006782	0.34078809	1.11737192	-0.834274877	0.001920258	8306	1	1326
NM_017732	-0.2823478	0.67864402	-1.129607831	0.001717369	6916	1	1009
NM_017732	-0.6253962	0.86002014	-1.129607831	0.000813405	6828	1	1655
NM_017732	-0.3245514	1.08247498	-1.129607831	0.001245665	6948	1	1582
NM_017732	-0.5281287	0.78508584	-1.129607831	0.000513176	6765	1	1285
NM_002335	-0.6138528	1.01306557	-1.165014498	0.000837232	6771	1	1731
NM_002335	-0.9696928	1.07725634	-1.165014498	0.004902615	6659	1	2104
XR_013922	-0.3483325	0.48137603	-1.223140516	0.008598599	6777	1	1468
NM_194288	0.15817389	-0.0540297	-1.023772041	0.007679187	7689	1	1569
NM_194288	-0.1439869	0.23644437	-1.023772041	0.002138843	7639	1	1021
NM_018025	0.26754001	0.94135934	-0.809651155	0.007524827	7295	1	1002
NM_005072	-0.0119023	0.50521302	-1.057137932	0.000264995	7665	1	353
NM_005072	-0.0359942	0.80744968	-1.057137932	0.001157328	6920	1	651
NM_016153	0.24362232	0.19518057	-1.10259865	0.001541232	7325	1	1453
NM_147193	0.37575544	0.46915289	-1.033676044	0.000682752	7317	1	1477
NM_001003792	0.44141443	0.59728041	-1.170880074	0.023667759	7314	1	2011
NM_001003792	0.68054795	0.66122982	-1.170880074	0.028999128	7316	1	2051
XR_011876	-0.2269856	0.97144334	-0.610360255	0.021291323	7513	1	1607
NM_014780	-0.2525816	0.64543756	-1.22939494	0.000164043	6798	1	917
NM_014780	-0.2562385	0.70855498	-1.22939494	0.000799509	6768	1	1057
NM_139053	0.68740302	0.47890375	-0.618757305	0.040689947	7334	1	1683
NM_005052	-0.4054861	0.67555208	-1.307360335	0.001252933	6910	1	1658
NM_030665	-0.3197982	0.79357033	-1.070258146	0.005461972	6655	1	1588

NM_152743	-0.4832071	0.3012119	-1.123040452	0.00036623	6483	1	1290
NM_152743	-0.2723193	0.5911306	-1.123040452	0.000523885	6915	1	758
NM_014577	-0.0097597	0.56968806	-1.061221068	0.001895173	7110	1	475
NM_014577	0.03452716	0.76315813	-1.061221068	0.000222216	7111	1	551
NM_006240	-0.5280197	0.55929874	-0.984366591	0.021097904	6631	1	1713
NM_018944	0.66851661	0.39916445	-1.138325238	0.007249308	7311	1	1892
NM_012256	-0.4905242	0.56028174	-1.06699406	0.009136262	6762	1	1412
NM_194302	0.30312586	0.55911954	-0.968922674	0.012629557	8636	1	1288
NM_007182	-0.1388136	0.73261708	-1.228015196	2.30E-06	7094	1	745
NM_007182	-0.0226771	0.86318751	-1.228015196	0.000164475	7098	1	932
NM_005379	0.0946838	-0.0356603	-1.148750156	0.048095361	6494	1	1946
NM_022484	0.10793365	1.21251036	-0.833818441	0.024692019	8919	1	1951
NM_022484	0.2304054	0.99851275	-0.833818441	0.03788906	8960	1	1817
XR_010226	-0.9364862	-0.1742286	-1.297549317	0.010400932	6408	1	2134
XR_010226	-0.7370797	-0.0643024	-1.297549317	0.023101177	6409	1	2088
NM_021201	-1.4530311	0.64998077	-0.867255585	0.003130853	6390	1	2083
NM_021201	0.27549589	0.87250735	-0.867255585	0.007481637	7346	1	1911
NM_014519	0.08943658	0.70339195	-1.139699126	4.19E-05	7109	1	700
NM_006760	0.13072519	0.49736975	-0.880652489	0.007739106	8836	1	896
NM_003298	-0.2654481	0.36732397	-1.377043442	0.010420223	6747	1	1815
NM_021830	0.04961979	0.95449287	-1.091284159	0.000994761	7197	1	1369
NM_022447	0.01232104	0.49026476	-1.180846812	7.17E-06	7664	1	639
NM_022447	0.0531387	0.67287485	-1.180846812	0.000166944	7100	1	712
NM_022834	0.76142762	0.08021606	-1.066844009	0.031752457	8691	1	2053
NM_002526	-0.6665654	1.0532248	-1.24605014	0.006618873	6661	1	2064
NM_013255	-1.1338029	0.50989522	-1.159049104	0.011127497	6617	1	2102
NM_013255	-0.5987413	0.7223273	-1.159049104	0.044486184	6653	1	2025
NM_012469	-0.3277207	1.00100223	-1.215951131	5.70E-07	7136	1	1236
NM_012469	-0.2317956	0.93106855	-1.215951131	2.24E-05	7137	1	1033
NM_001009996	-0.643109	0.64239216	-1.295915535	0.000543989	6753	1	1759
NM_001009996	-0.7846019	0.72787939	-1.295915535	0.000410905	6749	1	1920
NM_031438	-0.3729685	0.34110342	-1.261789394	0.020647321	6034	1	2180
NM_031438	-0.0057389	0.57918621	-1.261789394	0.023858455	7221	1	1769
NM_002611	-0.5281893	0.55994135	-1.28618608	0.011465947	6519	1	1937
NM_006885	-0.5504388	0.11689759	-0.85396542	0.010543344	6438	1	1580
NM_006885	-0.5740957	0.60475213	-0.85396542	0.001853369	6826	1	890
NM_017810	-0.1176052	0.67316233	-0.849870078	0.016008489	7340	1	1095
NM_016828	0.04405457	0.83442279	-0.861399122	0.033843421	8867	1	1602
NM_016828	0.289605	1.13617608	-0.861399122	0.006131748	7297	1	1670
NM_014155	-0.4160638	0.72791519	-1.509883158	0.006393235	6656	1	2021
NM_016609	-0.484075	-0.2111204	-1.068057059	0.001496048	6419	1	1933
NM_016609	-0.3430937	0.47482353	-1.068057059	0.001255346	6487	1	1294
NM_016609	0.10145472	0.1815534	-1.068057059	0.001144986	7551	1	945

NM_016609	-0.2070272	0.58466659	-1.068057059	0.000136963	6836	1	550
NM_004370	-0.8005548	0.67943586	-0.941796392	0.001713214	6811	1	1732
NM_004370	-0.5781278	0.79174202	-0.941796392	0.000964513	6831	1	1047
NM_197960	-0.390667	0.80546767	-1.260305186	0.000587073	6767	1	1383
NM_005920	-0.1565251	0.40186029	-1.318108432	0.004421006	6779	1	1505
NM_005920	-0.2261526	0.43666054	-1.318108432	0.029526282	6639	1	1947
NM_015896	-0.2068575	0.68525947	-1.123688678	0.012011124	6911	1	1600
NM_018113	-0.5234057	0.44948276	-1.226502406	0.000266673	6751	1	1421
NM_018113	-0.4327819	0.91876857	-1.226502406	3.23E-05	7132	1	1348
NM_001609	0.13862507	0.88333042	-0.519021916	0.027566725	8404	1	1106
NM_004756	-0.8444872	0.40286584	-1.628355718	0.025150981	6013	1	2186
NM_020728	0.33135443	0.24642871	-0.719912276	0.0358107	7332	1	1159
NM_001009956	-0.3070486	0.82049982	-0.867557924	0.026076873	8946	1	1379
NM_003259	-0.0804447	1.18098501	-0.63833998	0.044423671	8920	1	1964
NM_006276	-0.162063	1.01393978	-0.826951468	0.020229297	8948	1	1364
NM_006276	0.05024883	1.0275837	-0.826951468	0.019300877	8949	1	1395
NM_017888	-1.3386767	0.42777958	-1.098746174	0.00410706	6009	1	2146
NM_017888	-0.8495853	0.45121361	-1.098746174	0.005955142	6620	1	1931
NM_000383	-0.4604416	1.01017787	-0.587586026	0.042075452	8917	1	1784
NM_022822	-0.1285408	0.76853432	-1.257680533	8.32E-06	7095	1	920
NM_003725	-0.0131201	0.02955395	-0.909777834	0.037985692	7548	1	1320
NM_173527	0.43716809	0.23068878	-1.237592762	0.002587216	8693	1	1766
NM_030768	0.25101275	0.94660633	-1.095055291	0.000621964	7128	1	1003
NM_004216	-0.0790138	-0.0702998	-0.873438335	0.015735367	7569	1	1492
NM_004216	-0.548728	0.21994661	-0.873438335	0.004078387	7365	1	1199
XR_013280	-0.3562583	1.20435033	-0.702036191	0.010211288	8871	1	1717
XR_013280	-0.4671877	0.76443699	-0.702036191	0.006541824	7517	1	723
NM_012222	0.22061344	1.20117731	-0.754782506	0.007314605	8366	1	1622
NM_012222	0.18578542	1.12529742	-0.754782506	0.004577158	8365	1	1229
NM_015270	-0.1395446	1.1157104	-1.033863629	0.001791469	8206	1	1485
NM_138342	0.27045433	0.95102723	-0.850256945	0.001430862	8303	1	721
NM_030645	0.15256055	0.65738463	-1.128824195	0.000340097	7116	1	676
NM_030645	0.04823153	0.70007022	-1.128824195	0.000436436	7115	1	612
NM_152360	-0.4692776	0.96106591	-0.461564274	0.049132747	7515	1	1780
NM_018262	-0.1511051	1.21981032	-1.070057034	1.95E-06	7158	1	1340
NM_152383	0.03113852	0.39394914	-0.976345341	0.000735239	7673	1	437
NM_152383	-0.0488978	0.53508125	-0.976345341	0.000746041	7674	1	273
NM_001012626	0.49690744	0.70767258	-0.828926599	0.005299353	8798	1	1148
NM_001012626	0.32116596	1.16169882	-0.828926599	0.003812029	7296	1	1518
NM_152271	0.05301916	0.67746236	-1.304889472	0.004771208	7218	1	1567
NM_152271	-0.0480636	0.81440212	-1.304889472	0.002733669	6805	1	1483
NM_000287	-0.4693708	0.6662251	-1.18826188	4.34E-05	6820	1	1187
NM_000287	-0.4235226	1.0056284	-1.18826188	3.01E-07	7134	1	1407

NM_018182	0.21857064	0.89505274	-0.870904822	0.001303024	8289	1	591
NM_016162	-0.1308971	0.99181889	-1.227112748	4.11E-05	7148	1	1166
NM_032328	-0.1896712	1.00384711	-0.938744183	0.013314854	8951	1	1396
NM_000136	-0.2502798	0.76692144	-0.563024137	0.01509817	7518	1	635
NM_005650	-0.1994024	0.41221504	-1.28852983	0.010540339	6778	1	1581
NM_005650	0.04298514	0.66927736	-1.28852983	0.002206322	6809	1	1243
NM_014779	-0.0409162	0.8384525	-1.294046139	0.017341143	7211	1	1882
NM_017519	-0.1379467	0.17624922	-1.392659062	0.049558925	6638	1	2032
NM_017519	-0.0478602	0.62270546	-1.392659062	0.005620565	6808	1	1682
NM_199184	0.63172027	1.15572184	-0.568052345	0.024463086	9657	1	2059
NM_014906	0.8144686	0.46198219	-0.693156503	0.008638437	10853	1	2115
NM_016115	-0.3987532	0.42057194	-1.048742302	0.001727097	6484	1	845
NM_016115	-0.6378265	1.20083475	-1.048742302	0.009884223	8927	1	2030
NM_014343	0.19646655	0.86275762	-1.085152463	3.66E-05	8259	1	741
NM_014343	-0.1064423	1.17385374	-1.085152463	1.15E-05	7157	1	1263
NM_153265	-0.7211027	-0.1941139	-1.079146774	0.00067064	6424	1	2026
NM_153265	-0.7909091	0.08498669	-1.079146774	7.68E-05	6425	1	1903
XR_011016	0.02851176	0.68858086	-0.865105776	0.032923625	8841	1	1168
NM_001038	-0.4403868	1.15113083	-0.351748491	0.038971782	8918	1	1887
NM_006340	-0.4312011	0.77644258	-1.017706213	0.000574409	6829	1	871
NM_006340	-0.5832624	0.72477805	-1.017706213	7.19E-05	6824	1	1031
NM_144698	-0.3441206	-0.3959886	-1.154711609	0.00081693	6417	1	2040
NM_144698	-0.5113768	-0.0594923	-1.154711609	0.033525773	6412	1	1972
NM_001256	-0.1611744	0.71058471	-0.948485204	0.016998831	7358	1	1237
NM_001256	-0.3923478	0.20024221	-0.948485204	0.044799937	6549	1	1922
NM_003040	0.34710055	0.76406565	-0.754401128	0.045509848	8879	1	1693
NM_016166	-0.2737561	0.11914789	-1.204533451	0.000230954	6475	1	1500
NM_016166	-0.2377624	0.52164786	-1.204533451	0.000136685	6796	1	827
NM_001680	0.65142609	1.03113431	-0.517120301	0.030467266	9649	1	1959
NM_145798	-0.5027431	0.22073772	-1.123064474	3.72E-05	6474	1	1502
NM_145798	-0.3937417	-0.0235065	-1.123064474	0.016511323	6414	1	1804
NM_004058	-0.3457656	0.17259828	-1.181314668	0.000957041	6478	1	1446
NM_138700	0.286785	0.16629729	-0.656975513	0.044921442	7331	1	1150
NM_012284	-0.509486	0.39685038	-0.843660819	0.008222387	7349	1	1558
NM_012284	-0.1512088	1.01697486	-0.843660819	0.008238768	8868	1	1291
NM_024561	0.14651445	1.05816814	-0.576434555	0.005046791	8362	1	834
NM_024561	0.15739821	0.82297541	-0.576434555	0.028360342	8403	1	832
NM_006702	-0.4889036	0.51046948	-0.852890022	0.041607746	7354	1	1625
NM_194281	0.0246565	0.93827962	-1.209416962	0.002207877	7224	1	1443
NM_145738	-0.2714716	-0.5916111	-1.292425943	0.012694828	6508	1	2139
XM_092995	-0.4424303	0.51866279	-0.924703243	0.01586447	6632	1	1788
XM_092995	0.19716505	0.36142023	-0.924703243	0.036995663	7313	1	1478
NM_006225	-0.2000651	0.89579706	-1.104905519	3.47E-06	7139	1	603

NM_006225	-0.200517	1.16802756	-1.104905519	1.19E-05	7153	1	1347
NM_138769	-0.3103368	0.94583512	-1.1120925	4.39E-05	7138	1	872
NM_138769	-0.1608631	0.99485832	-1.1120925	0.000129027	7102	1	915
NM_001006636	-0.0614633	0.78352856	-1.019286556	0.007650793	8158	1	744
NM_005550	-0.4609426	0.56140922	-1.064921553	0.0001166	6819	1	881
NM_005550	-0.0344713	0.73334565	-1.064921553	1.14E-05	7106	1	650
NM_002518	-0.0581653	0.86630912	-1.074501574	2.83E-05	7104	1	589
NM_002518	-0.0977517	0.90899997	-1.074501574	0.000608793	7113	1	708
NM_032726	-0.9408001	0.80185111	-1.135541545	7.44E-05	6662	1	2013
NM_004757	0.32871058	-0.0025806	-0.865282058	0.030498678	7686	1	1677
NM_152403	0.07386397	0.60520528	-1.036839855	0.002260076	8835	1	1038
NM_152403	0.04548343	0.82414927	-1.036839855	0.019861104	7223	1	1428
NM_003564	0.2645724	0.48725737	-1.112964728	0.000155606	7748	1	693
NM_003564	0.32871083	0.58552847	-1.112964728	0.000154862	7749	1	769
NM_020974	0.77048653	1.07938971	-0.811348289	0.000321599	8818	1	1869
NM_020974	0.45275297	0.92651347	-0.811348289	0.000754615	8829	1	967
NM_001001691	0.90063734	0.97072712	-0.58215392	0.044260767	9655	1	2112
NM_016084	-0.3903957	0.89469736	-0.985987444	0.014039694	7303	1	1639
DA237913	-0.0331332	1.0248197	-0.874913228	0.027491832	7305	1	1668
XR_011163	-0.1573285	0.47086469	-0.969763104	0.002545716	7531	1	540
XR_011163	-0.1955554	0.71044243	-0.969763104	6.86E-05	6844	1	143
NM_032905	-0.189099	1.0793221	-1.12890044	0.001475184	7174	1	1437
NM_032905	-0.2999731	1.12117528	-1.12890044	0.023181466	6678	1	2015
NM_025204	0.18664811	0.3437354	-1.084434661	0.000211735	7745	1	749
NM_025204	0.21462679	0.40976046	-1.084434661	0.000719777	7746	1	669
NM_012326	0.16360801	0.62807234	-0.813807275	0.044478967	8842	1	1486
NM_012326	-0.4982248	1.13081784	-0.813807275	0.041519036	6057	1	2122
NM_014223	0.01661241	0.21407734	-0.895967229	0.01472423	7641	1	1044
NM_014223	-0.2226635	0.48062385	-0.895967229	0.000724621	7642	1	430
XR_010838	-0.484034	-0.0277925	-0.915245203	0.011782224	6437	1	1721
XR_010838	-0.2541746	0.10228379	-0.915245203	0.010255651	6439	1	1265
NM_021168	-0.2170366	0.58330265	-1.129894427	0.000710479	6799	1	621
NM_021168	-0.0491558	0.87148246	-1.129894427	0.000520611	7101	1	751
NM_002762	0.38121069	0.47276058	-0.996669272	0.013985557	8635	1	1433
NM_025128	-0.479594	0.83078817	-0.970082543	0.000953189	6832	1	802
NM_025128	-0.520717	0.77368744	-0.970082543	0.007405388	6761	1	1286
NM_001176	0.15622109	0.97045662	-0.970496902	0.002004947	8260	1	1061
NM_005089	0.19132587	0.81739932	-0.983562103	0.000315378	8288	1	372
NM_005089	0.09935462	1.03536784	-0.983562103	6.23E-05	8287	1	632
NM_001441	-0.5269077	0.97203098	-0.910792739	0.001339059	6830	1	1440
NM_001441	-0.4938155	1.2203909	-0.910792739	0.000974572	6953	1	1704
NM_013974	-0.3817475	0.20237593	-0.78472843	0.006244702	7566	1	759
NM_013974	-0.2418271	0.25014237	-0.78472843	0.008132113	7568	1	431

NM_005149	0.36581715	0.77810626	-0.652585452	0.024069279	8397	1	1193
NM_005149	0.22651631	0.99398835	-0.652585452	0.006102491	8358	1	906
XR_010071<br>671962232							
NM_017707	-0.0841808	0.20316931	-0.940025898	0.005132032	7530	1	770
NM_012432	0.01488358	0.54245886	-0.974147187	3.35E-05	7670	1	158
NM_012432	0.02061658	0.3272271	-0.974147187	0.002745257	7650	1	434
NM_014615	0.04539419	0.81115514	-0.551019858	0.004839934	8357	1	256
NM_014615	0.03913687	0.93146087	-0.551019858	0.004023616	8361	1	338
NM_005605	-0.3740871	0.19482144	-1.029532025	0.001993939	7539	1	1220
NM_005605	-0.0180758	0.12899815	-1.029532025	0.011741146	7550	1	1101
NM_013313	0.33963085	0.84591752	-1.053238051	0.00018527	7127	1	905
NM_013313	0.21481609	1.01101117	-1.053238051	0.000139186	7129	1	1022
NM_005688	-0.1320441	1.08987619	-1.152866695	0.000134196	7199	1	1368
XR_012668	-0.1866548	0.18820515	-1.115232517	0.000496095	6479	1	1048
NM_032127	-0.8313556	0.33624288	-1.121960131	0.005362643	6626	1	2019
XM_371246	-0.1484076	0.52189067	-0.981261117	8.54E-05	7654	1	125
XM_371246	-0.1543891	0.52749675	-0.981261117	0.000258256	7653	1	168
NM_004192	-0.3422844	0.59363765	-1.050427633	0.000225147	6835	1	733
NM_004192	-0.3363755	1.16279432	-1.050427633	1.13E-05	7151	1	1330
NM_005427	0.37979494	0.60359241	-0.657709049	0.04856523	8843	1	1534
XR_013101	-0.4677791	0.61064818	-0.541616395	0.01103998	7519	1	610
XR_013101	-0.4929536	0.86944483	-0.541616395	0.008045059	7522	1	1043
XR_014146	-0.0232772	0.53151734	-0.691964839	0.002262694	8057	1	31
XR_014146	0.1403692	0.56884633	-0.691964839	0.00109942	8273	1	65
NM_017999	-0.0560574	0.51951154	-1.055925555	3.63E-05	7668	1	276
NM_017999	-0.2029042	0.72487353	-1.055925555	0.000112797	7103	1	432
NM_017854	-0.2778495	-0.0249719	-0.770135141	0.004485091	7552	1	1207
NM_017854	-0.1605003	0.18095859	-0.770135141	0.000787637	7573	1	409
NM_145029	0.22511137	0.8648745	-0.860521854	0.000210104	8300	1	322
NM_145029	0.23791578	0.81899673	-0.860521854	2.64E-05	8301	1	241
NM_001520	0.1450966	-0.5570982	-1.068187199	0.00940723	6510	1	2103
NM_001520	-0.2714325	-0.7773141	-1.068187199	0.034949317	6509	1	2142
NM_014593	-0.39139	0.8119655	-0.845698106	2.94E-05	6846	1	259
NM_014593	-0.2639465	0.94304169	-0.845698106	0.000132773	8224	1	307
NM_080732	0.21082166	0.25023806	-0.839792347	0.000705563	7703	1	397
NM_080732	0.20731171	0.50095782	-0.839792347	0.000383146	7680	1	93
NM_032087	0.5874853	0.46110682	-0.948865028	0.009610043	8746	1	1587
NM_024638	-0.218997	0.92008298	-1.033874938	0.008594723	8941	1	1584
NM_001010871	-0.5728601	0.32995202	-1.048654178	0.036976806	6627	1	1894
NM_007271	0.33179662	0.13275543	-0.988987234	0.000156774	7696	1	1239
NM_007271	0.36910661	0.21267189	-0.988987234	0.00173966	7700	1	1077
NM_022165	0.05315178	0.69510105	-0.45277361	0.04661444	8848	1	780

NM_005928	0.07859451	0.59944911	-0.928206619	0.000995658	7671	1	201
NM_005928	-0.0691812	0.57986858	-0.928206619	0.000510851	7669	1	140
NM_021045	0.21964468	0.8248065	-1.033270074	0.005582712	7226	1	984
NM_021045	0.14087079	1.08777769	-1.033270074	0.005485799	7235	1	1530
NM_004517	-0.2496635	0.99753439	-0.894236855	0.000282657	8225	1	570
NM_004517	-0.2937835	1.15236753	-0.894236855	2.19E-05	7152	1	941
NM_004258	-0.4718402	0.41422775	-0.561814408	0.010676843	7362	1	1127
NM_014701	-0.0719026	0.01984746	-1.096987484	0.003537276	7549	1	1411
NM_014701	0.309234	0.271834	-1.096987484	0.026256144	7742	1	1577
NM_014701	0.2777226	0.59126855	-1.096987484	0.027771164	7315	1	1646
NM_003906	-0.0042457	0.18678452	-0.666255575	0.044042413	7579	1	697
NM_000123	-0.3870531	0.41374721	-1.170799955	0.027635912	6784	1	1712
NM_000123	-0.4803463	0.70642524	-1.170799955	0.010150724	6780	1	1624
NM_033121	-0.3462686	0.25857173	-0.831970164	0.015182966	7565	1	690
NM_033121	-0.3921672	0.50843561	-0.831970164	0.00574417	6850	1	448
NM_018442	-0.4471799	0.76583947	-0.826901991	0.001613419	6764	1	587
NM_018442	-0.1618645	0.83707306	-0.826901991	0.032769491	8151	1	1224
NM_002145	-0.7745976	0.99603196	-0.99632799	0.000188927	6812	1	1818
NM_002145	-0.1757325	0.79110905	-0.99632799	0.002776818	8156	1	529
NM_005855	-0.5429106	0.90172818	-0.905933051	0.000195254	6834	1	1027
NM_005855	-0.5452597	1.05322535	-0.905933051	7.72E-06	8218	1	1163
NM_015555	-0.3960336	0.14033331	-1.093781086	0.006579699	6415	1	1630
NM_015555	-0.1680563	0.24263048	-1.093781086	0.014768929	6787	1	1211
NM_018263	0.27343944	1.10129157	-1.095648505	0.005273323	7231	1	1667
NM_152640	-0.0990501	1.09563047	-0.784774068	0.000971565	8371	1	779
AB075502	0.30308842	1.09347665	-1.036805096	0.001290556	7233	1	1378
AB075502	0.20354343	1.1126783	-1.036805096	0.003932146	7232	1	1496
NM_018502	0.07424708	0.24136031	-1.018740547	0.013442876	8695	1	1189
NM_018502	0.04109737	0.30060651	-1.018740547	0.013825056	8696	1	1124
AY669488	0.40258301	0.18678598	-0.981725603	0.006718397	8744	1	1470
NM_022067	-0.1323635	0.06127009	-0.595160456	0.036013448	7575	1	925
NM_022067	-0.4975279	0.32525173	-0.595160456	0.010449586	7360	1	826
NM_005624	-0.5353535	0.16830084	-0.955416887	0.019512071	6472	1	1687
NM_005299	0.10060979	0.31542249	-0.941264857	6.31E-05	7645	1	425
NM_006924	-0.1716398	0.72782466	-0.937985533	0.010002205	8154	1	582
NM_006924	-0.0233075	0.81437848	-0.937985533	0.005052145	8157	1	450
NM_022911	-0.0905438	-0.1192104	-0.827141403	0.006269322	7544	1	1424
NM_022911	-0.1939555	0.09767938	-0.827141403	0.019027291	7545	1	870
NM_012103	-0.0407379	1.21119485	-0.887178559	2.86E-05	8312	1	889
NM_033446	-0.5385964	0.09858197	-0.611036001	0.020193375	6433	1	1292
NM_033446	-0.4976211	0.3526482	-0.611036001	0.005992451	7367	1	607
NM_054028	0.29731348	0.35483595	-1.048352988	8.15E-05	7683	1	1062
NM_014153	-0.6491632	0.47564067	-1.03360673	0.004987975	6629	1	1590

NM_014153	-0.5258588	0.7419182	-1.03360673	0.001746006	6763	1	1284
NM_152299	-0.117649	0.60394768	-1.124941967	8.94E-05	7667	1	458
NM_152299	-0.1905288	0.67133619	-1.124941967	0.000325592	6855	1	620
NM_000106	0.08562271	0.92048241	-0.649966459	0.008721043	8876	1	682
NM_000106	0.17816665	0.96989434	-0.649966459	0.000951301	8363	1	352
XR_014187	-0.2873094	1.09734754	-0.890352961	0.002804036	8943	1	1085
XR_014187	-0.1932147	0.97713602	-0.890352961	0.018492793	8152	1	1408
NM_006233	0.65021933	0.67241784	-0.66213458	0.0480215	8764	1	1867
XR_014109	0.01138456	1.06694533	-0.824220987	2.84E-05	8313	1	383
NM_002585	0.22769384	1.17246986	-0.858904754	7.69E-05	8292	1	1026
NM_002585	0.18702165	1.1086248	-0.858904754	8.81E-05	8330	1	699
NM_019052	-0.2243874	0.85914933	-1.289026679	0.000383136	7092	1	1363
NM_033661	-0.3411743	0.58967372	-0.64047768	0.034086132	8029	1	773
NM_006440	-0.2103188	0.90930858	-0.484935456	0.007580732	7523	1	527
NM_006440	-0.1237812	1.05376678	-0.484935456	0.003196235	8360	1	633
NM_002948	-0.6057401	1.1029308	-0.841707717	0.003373867	9005	1	1589
XR_011148	-0.3718725	0.63953278	-0.727517281	0.014261903	7355	1	765
XR_013719	-0.253686	0.8238789	-0.64724376	0.021565676	8211	1	1024
NM_020195	-0.6921475	0.75181575	-0.830798899	0.000997969	6825	1	1228
CO579632	-1.1214769	0.34465511	-1.161762239	0.000408272	6016	1	2171
NM_000964	-0.605215	0.0710647	-0.77378969	0.000416627	6431	1	1475
NM_000964	-0.6377672	0.28904141	-0.77378969	0.000677616	7366	1	1219
NM_033487	0.42886823	0.44508094	-0.897655998	0.002955963	7756	1	739
NM_033487	0.3375353	0.55569951	-0.897655998	0.002774274	7757	1	517
DV768384	-0.3409566	0.68042222	-0.550592993	0.025517086	8032	1	783
NM_018031	-0.3972327	0.54687437	-0.878571697	1.15E-05	6840	1	223
NM_018031	-0.3360903	0.61517736	-0.878571697	6.02E-07	6842	1	117
NM_017807	-0.0831471	0.66747797	-1.0673245	0.002726192	7341	1	1300
NM_017807	0.2029056	0.90144682	-1.0673245	0.000225984	7118	1	1094
NM_173607	0.02585103	0.66040679	-1.181778412	0.025678123	7217	1	1793
NM_173607	0.04000645	0.99384623	-1.181778412	0.010544183	7212	1	1809
XR_011421	0.62279599	0.89475123	-0.553497727	0.024048023	9647	1	1864
XR_011421	0.61491066	0.89914375	-0.553497727	0.044796353	7321	1	1982
NM_018183	0.46806643	0.67416943	-0.800337832	0.036565235	8757	1	1415
NM_152528	-0.3223664	0.16288476	-0.731575148	0.01218373	7567	1	719
NM_021784	0.33643431	0.89231533	-0.987177684	0.006481293	7227	1	1401
NM_033388	0.351034	0.63427034	-0.900410953	0.008225196	7752	1	768
NM_002209	0.4292398	0.78293358	-0.774809405	0.01500823	8881	1	1425
NM_004075	0.19357658	1.17252588	-0.772488916	7.39E-05	8324	1	927
NM_000903	0.31319383	-0.1584729	-0.860253019	0.02121113	7690	1	1708
NM_000903	0.24879862	0.24118591	-0.860253019	0.002608153	7706	1	743
NM_005842	-0.1441003	0.54540315	-1.073530002	0.01709186	6786	1	1171
NM_014359	-0.5292871	0.14035695	-1.61835202	0.016968537	6031	1	2165

NM_022105	-0.0043461	1.06929745	-1.065217401	0.002631549	7225	1	1304
NM_005332	0.31937225	0.32481244	-0.934143395	0.030692376	8700	1	1495
NM_005332	0.28207356	0.29664888	-0.934143395	0.02368779	8699	1	1367
NM_017887	-0.2218283	0.79120184	-0.864270153	0.01225302	8149	1	675
NM_017887	-0.029372	1.15203003	-0.864270153	0.002028839	8178	1	994
NM_002547	-0.590421	0.71107539	-1.341520419	0.002416376	6756	1	1832
NM_006369	-0.2135397	0.0397281	-0.746649701	0.004582842	7555	1	1007
NM_006369	-0.1246629	0.09621553	-0.746649701	0.000555999	7572	1	644
NM_017490	0.2154415	0.42921512	-0.954979581	0.000367605	7747	1	297
NM_017490	0.22915141	0.67420301	-0.954979581	0.000175569	7761	1	239
NM_005096	-0.0169014	0.57589093	-0.955566395	0.000310169	7657	1	109
NM_005096	-0.0842449	0.59303054	-0.955566395	8.14E-05	7660	1	122
NM_001606	-0.5767488	0.94388006	-0.828118177	0.02866771	6534	1	1952
NM_144990	0.06892005	0.73232288	-0.764376116	0.031033618	8874	1	1167
NM_144990	0.13598998	0.9612992	-0.764376116	0.008058104	8875	1	989
NM_013279	-0.0741293	1.06912172	-0.9262038	0.041328242	6615	1	1996
NM_003110	0.33697524	0.22818734	-1.132430688	0.008436635	6531	1	1900
NM_019590	0.22736512	1.14047057	-1.113491053	0.018252516	7306	1	2001
NM_005104	0.21189964	0.1070553	-0.813312159	5.28E-05	7702	1	728
NM_005104	0.30614998	0.2431768	-0.813312159	2.19E-05	7704	1	504
XR_014604	0.53847097	0.64494587	-0.548932494	0.011097368	8399	1	1051
A_01_P006584	-0.1832549	1.24620399	-0.857952471	0.013711763	6538	1	1942
NM_003506	-0.1562805	1.03902901	-0.84282015	0.004238966	8209	1	981
NM_004669	0.57983056	0.49971648	-0.77291134	0.030026085	8725	1	1644
NM_004669	0.68418318	0.75233514	-0.77291134	0.034720312	8767	1	1938
NM_183415	0.43554402	0.63984383	-0.723471185	0.000244974	8810	1	418
NM_183415	0.0975033	0.63238254	-0.723471185	0.001586139	7681	1	30
NM_006558	-0.2387305	-0.1132999	-0.881705924	0.02385012	7543	1	1568
NM_152283	0.36967541	0.70647739	-1.08240319	0.00421793	7319	1	1681
NM_014233	0.12959718	0.28261153	-1.022109274	0.044447134	8697	1	1645
NM_014233	0.40136586	0.23906881	-1.022109274	0.017650202	8705	1	1628
NM_199336	0.0406898	0.53332847	-0.80037577	0.000503413	7676	1	44
NM_199336	0.0311868	0.68102251	-0.80037577	0.00021115	7677	1	29
NM_021248	0.12577666	0.8782876	-0.561625677	0.038879407	8877	1	1391
NM_021248	0.18410794	0.91648085	-0.561625677	0.017871388	8878	1	1058
NM_007073	-0.0099098	1.18416662	-0.522636041	0.022121545	8976	1	1868
NM_201627	-0.146132	0.44519504	-0.888026602	0.006951373	7649	1	234
NM_201627	-0.0665397	0.65380863	-0.888026602	0.004899265	8155	1	185
XR_009973	-1.9477788	0.01309034	-0.639532734	0.01130332	31	1	2193
XR_009973	0.42598894	0.04681118	-0.639532734	0.046132796	7337	1	1840
NM_002333	-0.4943474	-0.0563684	-0.996865798	0.007175014	6435	1	1767
NM_002333	-0.4721963	0.07337033	-0.996865798	0.002156213	6436	1	1559
NM_015570	0.24428182	0.47242775	-0.911654112	0.021915461	7751	1	959

NM_015570	0.27387586	0.99789898	-0.911654112	0.000485459	7320	1	1210
NM_006200	-0.0481683	1.03242214	-0.721071825	0.032775657	8964	1	1654
NM_000156	-0.1289579	0.53872481	-0.77886949	0.00030559	7675	1	39
NM_000156	-0.1792017	0.79305916	-0.77886949	0.001366154	8223	1	215
NM_014296	-0.62475	1.01570191	-0.540915594	0.041883762	6058	1	1940
NM_018093	0.00222023	0.39678981	-0.819608206	0.000110393	7605	1	63
NM_018093	0.02981935	0.804841	-0.819608206	0.001785445	8264	1	269
NM_004305	0.10756527	0.28954334	-0.757883328	0.000153791	7606	1	159
NM_004305	0.06070379	0.34351014	-0.757883328	0.00017102	7607	1	101
NM_001353	-0.1465342	0.46651648	-0.742492095	0.049290303	8004	1	757
NM_001079	-0.1725976	0.32138592	-0.760220365	0.000258908	7582	1	89
NM_001079	-0.110488	0.5776125	-0.760220365	0.000295794	8056	1	13
CB548564	-0.1174666	0.76898199	-0.784923348	0.021046361	7304	1	1388
NM_007317	-0.2433083	0.60492879	-0.52206281	0.034467968	7521	1	704
NM_178121	-0.6672143	0.10012103	-0.633870921	0.021645267	7359	1	1603
NM_016099	0.00623592	0.23061157	-0.734799012	0.016380195	7603	1	416
NM_016099	0.12700199	0.29597377	-0.734799012	0.02002734	7998	1	516
NM_006336	-0.4006743	-0.2817964	-0.789461733	0.027728586	6421	1	1880
NM_006336	-0.5529335	-0.3021757	-0.789461733	0.007643224	6420	1	1965
NM_203446	0.48306793	0.73791719	-0.482878034	0.029738291	8400	1	1191
NM_153342	0.11296705	0.89555216	-0.89201317	4.05E-05	8299	1	179
NM_153342	0.14253465	1.2060037	-0.89201317	6.39E-06	8326	1	1019
NM_003304	-0.2936999	1.13676757	-0.60684122	0.001315659	8373	1	1233
CB549641	0.42009078	0.3698045	-0.786765953	0.018887081	7754	1	929
CB549641	0.40639024	0.34431096	-0.786765953	0.024336988	7753	1	975
NM_024031	-0.4921676	0.53569082	-1.037341734	0.003005533	6817	1	1102
NM_024031	-0.6441898	0.75821336	-1.037341734	0.001091603	6821	1	1438
NM_002281	0.32880923	1.07845179	-0.740670035	0.032135643	8892	1	1914
NM_003130	0.31596982	1.09396611	-0.797264896	0.035789243	7239	1	1905
NM_206839	0.07158301	0.28578614	-0.904496541	0.016012658	7741	1	674
NM_004596	-0.1652202	0.00361047	-0.611312954	0.013072223	7574	1	887
NM_004596	0.083494	0.09162956	-0.611312954	0.00730195	7577	1	580
NM_144709	-1.0778203	0.11283963	-1.436444608	0.017105076	6036	1	2163
NM_174922	-0.4515972	0.38368229	-1.189511015	0.003272138	6815	1	1563
NM_174922	-0.4044577	0.49512377	-1.189511015	0.00103421	6816	1	1208
NM_052887	0.39639792	0.702822	-0.643514875	0.04535826	8760	1	1653
NM_052887	0.75753392	0.57456112	-0.643514875	0.002113848	8813	1	1570
NM_207346	-0.580484	1.03504335	-0.412355595	0.005010306	7510	1	1464
NM_207346	-0.5846891	1.14105221	-0.412355595	0.004801482	7511	1	1629
NM_007171	-0.1240899	0.5809668	-0.868315245	0.00018168	8038	1	91
NM_007171	-0.2809241	1.08319109	-0.868315245	0.001066016	9008	1	880
XR_010388	0.158483	1.05824346	-0.954373823	0.000584566	7202	1	864
NM_013262	-0.3235848	0.85097294	-1.184685685	0.000519295	6853	1	1506

NM_013262	-0.6269555	1.01809479	-1.184685685	0.000682833	6663	1	1873
NM_005053	-0.0333005	0.27648334	-0.695281254	0.00547848	7596	1	251
NM_005053	-0.1859846	0.35347604	-0.695281254	0.000536788	7585	1	104
NM_001002880	0.43341241	0.82123395	-0.680802742	0.000124121	8819	1	487
NM_001002880	0.29313504	0.63084199	-0.680802742	0.018610516	8401	1	914
NM_021117	0.05048781	0.75969884	-0.950132966	1.21E-05	8285	1	116
NM_021117	-0.0429517	0.90848838	-0.950132966	0.000639749	7114	1	311
NM_013382	-0.5114647	0.54365634	-1.115743262	0.01049948	6748	1	1598
NM_152930	0.02113803	0.60468213	-0.934573466	0.000798509	7672	1	166
NM_152930	0.04539457	0.95258377	-0.934573466	0.000469661	8267	1	542
NM_002013	0.38083114	1.13076274	-0.591193196	0.001763959	9334	1	1117
NM_005456	0.01889819	0.12579166	-0.967369038	0.045088374	7534	1	1722
NM_001003801	-0.3768751	0.47369388	-0.94929871	0.000106778	6837	1	411
NM_001003801	-0.0676625	0.50061539	-0.94929871	0.001142914	7656	1	288
A_01_P005022	-0.6110797	0.63048642	-0.549694329	0.003574306	7363	1	1183
A_01_P005022	-0.1325573	0.56985203	-0.549694329	0.003965645	8059	1	19
XR_014066	0.86780866	1.13442943	-0.304114948	0.009542072	9711	1	2096
NM_004496	-0.2901874	1.16774056	-0.391975624	0.034209392	9038	1	1763
NM_152286	-0.550329	-0.0772519	-0.789385641	0.01280649	6428	1	1725
NM_152286	-0.579154	0.124031	-0.789385641	0.012267909	6429	1	1539
NM_017432	-0.2812956	0.86842779	-0.711385935	0.002106883	8226	1	362
NM_017432	-0.2864087	0.97045315	-0.711385935	0.001778093	8227	1	554
NM_022485	-0.4673427	0.50693404	-0.787894972	3.68E-06	6838	1	300
NM_022485	-0.3655714	0.57335261	-0.787894972	0.000380483	6843	1	136
NM_004584	-0.5580667	0.79337455	-1.072300587	0.000780282	6822	1	1318
NM_004584	-0.4437957	0.95055754	-1.072300587	9.24E-05	7135	1	1109
NM_015017	0.63585484	0.66543801	-0.819081938	0.00152737	8787	1	1327
NM_015017	0.31049575	0.94588848	-0.819081938	0.00651232	7228	1	1028
NM_005834	0.27209334	0.18661935	-0.679224083	0.013750734	8717	1	875
NM_005834	0.35541515	0.35068559	-0.679224083	0.019571871	8719	1	902
NM_001927	0.20137981	0.14981128	-0.643105894	0.015219829	7873	1	760
NM_001927	0.26833668	0.40024503	-0.643105894	0.015210175	8718	1	578
NM_002270	0.40683987	0.16483367	-0.65513782	0.012365787	7733	1	974
NM_000328	-0.688036	0.94050524	-1.001665552	0.021298231	6666	1	1988
XR_012428	0.80164261	0.75871289	-0.655327409	0.034760284	8765	1	1981
XR_012428	0.44129901	1.03368271	-0.655327409	0.011847637	8883	1	1638
NM_005530	0.5174218	0.21588523	-0.737983044	0.00420979	7734	1	1274
NM_005530	0.07880276	0.23644621	-0.737983044	0.001988733	7608	1	265
XR_012827	-0.1890983	0.67916565	-0.932505841	0.013573308	8022	1	844
XR_012827	-0.0392769	1.02085001	-0.932505841	0.00202712	7178	1	755
NM_005007	0.40788093	0.67068244	-0.904540453	0.002926261	8800	1	882
NM_005007	0.51585894	0.63627839	-0.904540453	0.002436797	8801	1	1130
NM_006048	0.60401397	0.2171336	-1.136366224	0.02320744	8707	1	1984

NM_014634	0.0034043	0.56309448	-0.746561306	0.000850551	8041	1	45
NM_014634	0.1132913	0.80370805	-0.746561306	0.001144473	8173	1	72
NM_052840	-0.3755993	0.46306263	-0.994798491	0.047134565	7525	1	1888
NM_052840	-0.6785885	1.15557265	-0.994798491	0.045119364	6060	1	2125
XR_011842	0.15942125	0.76872533	-1.014202076	0.000891789	7117	1	558
NM_000187	0.01358778	1.05685307	-0.876501682	0.001541941	8268	1	1073
NM_000187	-0.3763242	1.20517701	-0.876501682	0.000695951	9006	1	1362
NM_003558	-1.3259855	0.66244951	-0.990204518	0.003221805	6618	1	2127
NM_003558	-0.732868	0.95821372	-0.990204518	0.007308718	6555	1	1847
NM_014147	-0.1379061	1.01377031	-0.525648672	0.020275988	8872	1	1323
NM_014147	0.28968986	0.87466403	-0.525648672	0.014340987	8853	1	1067
NM_024882	-0.378857	0.07675701	-1.353092915	0.023860794	6406	1	2087
NM_003643	0.13451075	0.91592846	-0.89932419	0.002573671	7344	1	1032
XR_010004	0.15371703	0.97511218	-0.80421904	0.00048644	8293	1	361
XR_010004	0.20759268	1.02948157	-0.80421904	0.000846899	8294	1	548
NM_178431	0.25843827	1.1532912	-0.423167377	0.00673948	9727	1	2145
NM_015470	1.11897815	0.8497356	-0.400833412	0.037211277	9716	1	2124
NM_148175	-0.1353639	0.17775608	-0.971309209	0.001457342	7646	1	703
NM_148175	-0.1259489	0.32363319	-0.971309209	0.003421806	7647	1	420
NM_016111	0.11212603	0.95078189	-0.979375775	9.43E-05	8286	1	441
NM_020249	-1.0795528	1.218741	-0.465029955	0.039430073	6542	1	2140
NM_013290	0.15252567	0.64387633	-1.199521841	0.001444474	7091	1	1138
NM_003791	0.31907787	1.02385197	-0.753107084	8.10E-05	8297	1	596
NM_003791	-0.0263398	1.05459238	-0.753107084	1.85E-05	8314	1	235
NM_003791	0.23020569	1.03988682	-0.753107084	0.001220153	8295	1	614
NM_144699	0.30319247	-0.0540452	-0.873202611	0.039685097	8712	1	1824
NM_024623	-0.2359311	0.36965359	-0.514497775	0.015861542	7793	1	298
NM_020919	-0.2685976	0.39212557	-0.699227121	0.007996892	7611	1	212
NM_138802	-0.2101247	0.70926874	-0.912725439	0.000211718	6845	1	139
NM_138802	-0.3813778	0.60673587	-0.912725439	2.45E-05	6841	1	221
NM_033271	0.29628194	0.75981541	-0.818378761	0.000894783	7762	1	242
NM_033271	0.13839534	0.90983299	-0.818378761	0.000361237	8176	1	178
NM_007013	-0.204105	1.10725877	-0.292207945	0.007996755	9045	1	1235
NM_004760	0.23422734	0.44739875	-0.895205815	0.006172393	9724	1	2132
NM_004760	0.2999539	1.07818661	-0.895205815	0.016101662	8840	1	1927
BC108680	0.71460684	0.55702059	-1.032733536	0.000529556	8785	1	1662
NM_017805	-0.1926469	1.12256168	-0.579908285	0.005200026	9019	1	846
NM_000028	0.00911432	0.83120361	-0.860329097	0.015747141	7237	1	972
NM_003682	-0.1382989	0.11986513	-0.79139647	0.000882878	7547	1	478
NM_003682	-0.1523355	0.29707938	-0.79139647	1.35E-05	7581	1	129
NM_022465	0.20284793	0.78465308	-0.826215916	0.000929739	8269	1	292
NM_022465	0.09520346	0.93254414	-0.826215916	0.000856802	8270	1	795
NM_001007071	-0.1270909	0.23052012	-0.6012523	0.001705178	7584	1	237

NM_001007071	-0.1750755	0.38964228	-0.6012523	0.001900714	7792	1	84
NM_004284	-0.2568388	0.31946281	-0.615346578	0.019164069	8027	1	533
NM_004284	-0.0053357	0.77142698	-0.615346578	0.000894236	8093	1	25
AL122084	-0.6572782	0.52617443	-1.083781445	0.00888316	6758	1	1643
NM_022762	-0.2633063	0.75862922	-0.702905093	0.002135978	7520	1	278
NM_022762	-0.3297637	1.104488	-0.702905093	0.006584918	8250	1	1359
CN647337	-0.4256443	0.48018007	-1.25860888	0.003753318	6745	1	1649
CN647337	-0.3637796	0.46054943	-1.25860888	0.014716787	6746	1	1819
NM_012235	0.11258664	0.50331118	-0.700728846	0.000231083	7678	1	58
NM_012235	0.20305889	0.81210579	-0.700728846	0.00010868	8302	1	74
NM_181746	-0.6060034	0.24378666	-0.772325794	0.017153943	6550	1	1877
NM_001001433	0.02340412	1.13661493	-0.616424324	9.37E-05	8372	1	634
NM_080646	0.18431272	0.65212351	-1.102179475	0.022103735	8618	1	1714
NM_000304	-0.0607435	0.99210699	-0.698004378	0.000262794	8335	1	227
NM_003726	0.31756775	0.09488598	-0.800835911	0.000263273	7698	1	996
NM_003726	-0.0128639	0.1407229	-0.800835911	0.000488506	7602	1	435
NM_003478	0.24615069	0.22417639	-0.762133693	0.00307557	7709	1	454
NM_003478	0.53997662	0.32168153	-0.762133693	0.003911103	7735	1	1158
NM_178832	-0.0161457	0.18201367	-0.636351265	0.002412576	7801	1	501
NM_032454	-0.1039273	0.53827063	-0.845108185	0.000160788	7658	1	37
NM_032454	-0.0519689	0.76026837	-0.845108185	0.000362771	8163	1	46
NM_006293	0.45675915	0.15061605	-0.530042386	0.016614541	7730	1	1266
NM_153831	-0.1515307	1.18367823	-0.447635527	0.000791206	9044	1	1093
NM_153831	-0.1717283	0.85605964	-0.447635527	0.012651093	8095	1	334
NM_022574	0.00518139	0.87334168	-0.346283247	0.014538705	8479	1	714
NM_022574	0.19183361	1.11196552	-0.346283247	0.006359196	8480	1	1234
NM_017901	0.29520086	0.44530613	-0.783343422	0.014607904	8721	1	891
NM_024729	-0.3642423	0.79442687	-0.839928828	3.06E-06	6847	1	189
NM_024729	-0.4579689	0.96192269	-0.839928828	5.09E-05	8219	1	709
NM_022731	0.47679043	0.52504855	-0.585457647	0.000391141	8811	1	521
NM_022731	0.55948132	0.83559809	-0.585457647	0.00034954	8821	1	964
NM_017523	0.28066174	0.51702989	-0.848056661	0.004458554	8720	1	599
NM_017523	0.58241862	0.57693974	-0.848056661	0.000720631	8747	1	1123
NM_033010	0.31990265	0.50007238	-0.532087597	0.039049378	8846	1	848
NM_033010	0.15271626	0.52822417	-0.532087597	0.022269206	8844	1	514
NM_033010	-0.1042177	0.42767162	-0.532087597	0.023352322	7856	1	238
NM_033010	0.15814559	0.69014809	-0.532087597	0.014727691	8845	1	341
NM_001616	0.81768665	0.85544724	-0.304453044	0.036870045	9712	1	2044
NM_203456	-0.8075597	1.0259863	-0.959658258	0.003162037	6560	1	2047
NM_203456	-0.2139884	0.93199806	-0.959658258	0.001036712	6860	1	885
NM_032682	-0.2862335	0.08565557	-1.161323847	0.042005229	6497	1	1853
NM_032682	-0.4472826	0.5274575	-1.161323847	0.025875165	6785	1	1765
NM_018086	0.70780842	0.98163409	-0.559178299	0.000415204	8826	1	1691

NM_007110	0.11234312	0.35841023	-0.64839272	0.004772018	7601	1	170
NM_007110	0.39220666	0.94841065	-0.64839272	0.018552839	8833	1	1673
NM_025197	0.06079298	0.39126413	-0.790458609	0.000355057	7609	1	80
NM_025197	-0.0095447	0.64508485	-0.790458609	0.000323709	7661	1	22
NM_007358	0.39108446	-0.024839	-0.8098708	0.000537461	7699	1	1512
NM_007358	0.36665796	0.21405987	-0.8098708	0.004940192	7701	1	884
NM_001006	0.67058332	0.87797934	-0.835976587	0.004721148	8792	1	1617
NM_001006	0.58224309	1.11179888	-0.835976587	0.000709013	9321	1	1606
NM_006835	-0.0119727	0.5003618	-0.619406643	0.024011327	8050	1	138
NM_006835	0.01641176	0.65151985	-0.619406643	0.019857212	8055	1	187
NM_015853	-0.1191903	0.37320186	-0.602617281	0.003503701	7587	1	90
NM_015853	-0.1967502	0.34910116	-0.602617281	0.002955304	7586	1	141
NM_017711	0.22304257	-0.1706188	-0.918837114	0.018460537	6515	1	1754
NM_017711	0.71818701	0.58871242	-0.918837114	0.003685607	8749	1	1800
NM_003718	-0.0025125	0.69802453	-0.714453799	0.001278219	8043	1	167
NM_152468	0.31957076	0.38931116	-0.901099291	0.016353326	8701	1	1216
NM_153012	0.23842208	0.29422498	-0.806467124	0.000595178	7705	1	440
NM_153012	0.46903784	0.5498472	-0.806467124	7.77E-05	8809	1	581
NM_033222	-0.7738667	0.51534319	-0.998855062	0.013785954	6757	1	1791
NM_003079	-0.3457341	0.52039213	-0.960257507	0.004204788	8015	1	512
NM_003079	-0.5152295	0.69204547	-0.960257507	0.011513483	6760	1	1252
NM_145065	0.17571124	0.97865262	-0.669374902	0.00781986	8885	1	939
NM_022765	-0.068147	1.03793287	-0.641295767	4.01E-05	8338	1	195
NM_022765	-0.0484812	0.96067244	-0.641295767	0.000123351	8337	1	92
XM_042066	0.78797766	0.8134861	-0.753822051	0.001911642	8795	1	1672
XM_042066	0.50098461	1.05499413	-0.753822051	0.002962862	9320	1	1382
NM_080414	0.22357387	0.32640689	-0.745694903	0.007728743	7710	1	306
NM_080414	0.06637574	0.74783608	-0.745694903	0.000124847	8172	1	16
NM_014488	0.55937424	0.58434628	-0.611284586	0.001807208	8812	1	806
NM_002228	-0.1395638	0.34890375	-0.602633767	0.014977823	7612	1	231
XR_010243	0.29758117	0.20693574	-0.694113599	0.004251498	7726	1	643
XR_010243	0.19094768	0.35616786	-0.694113599	0.001089183	7716	1	208
NM_003951	-0.251803	0.86114398	-0.713407125	0.011394194	8146	1	707
NM_012332	0.30161814	0.69298358	-1.051667199	0.008709124	8756	1	1578
NM_012332	0.13677937	1.0018327	-1.051667199	0.001601603	6335	1	1430
NM_018292	0.0429965	0.22134502	-0.492332937	0.03120092	7578	1	556
NM_182501	0.2247873	1.0680842	-0.318779286	0.010354737	8380	1	1778
NM_014550	-0.503651	1.02746403	-0.750795713	1.53E-05	8220	1	977
NM_031436	-0.3169503	0.38260101	-0.526445738	0.0261623	8028	1	332
NM_173506	0.15612971	0.16136073	-0.669815194	0.022666348	7335	1	1434
NM_173506	0.45273155	0.4577386	-0.669815194	0.004361281	8807	1	952
NM_015254	-0.0418916	0.47931065	-0.561680353	0.028382065	8051	1	165
NM_014678	-0.4994093	-0.2611735	-0.81989678	0.011771695	6422	1	1899

NM_014678	-0.3555998	0.1335641	-0.81989678	0.000874312	7540	1	973
NM_022913	-0.0484276	1.00274562	-0.777486098	0.001905889	8170	1	351
NM_020738	-0.2498047	1.16904422	-0.801735431	0.006453824	9004	1	1361
NM_032204	-0.6365117	-0.331692	-0.640566805	0.048813471	6440	1	2039
NM_032204	-0.2316038	0.01992298	-0.640566805	0.001229741	7570	1	1065
XR_011624	0.53151994	0.63903994	-0.867420356	0.011333458	8799	1	1461
XR_011624	-0.1549591	1.14608317	-0.867420356	0.003018226	8210	1	1253
NM_002235	0.22546952	1.07852882	-0.824990584	0.002169295	7271	1	1273
NM_174933	0.3133475	0.07040309	-0.892675185	0.014824723	8703	1	1444
NM_174933	-0.1240175	-0.0779133	-0.892675185	0.001479634	6516	1	1394
CB229350	-0.207479	0.90936115	-0.736136881	8.26E-05	8010	1	275
NM_024108	0.13991899	-0.4605294	-0.863982159	0.00496033	6512	1	2009
NM_001930	0.33628528	0.29341905	-0.767477359	0.045513227	8702	1	1385
NM_001930	0.45431939	0.6331896	-0.767477359	0.023012383	8726	1	1413
NM_033200	-0.0348724	0.60132062	-0.640889054	0.000481925	8058	1	11
NM_033200	-0.0793017	0.59028922	-0.640889054	0.000142605	8070	1	1
NM_018486	0.67652074	0.96732982	-0.706445059	2.61E-05	8822	1	1467
NM_018486	0.54967947	0.95575478	-0.706445059	4.47E-06	8820	1	1100
NM_019096	-0.4400769	1.19443386	-0.761770556	0.000565165	9009	1	1227
NM_032854	0.13246405	0.81251171	-0.848043518	0.024148149	8861	1	1719
NM_032854	0.4639135	0.64117483	-0.848043518	0.010641367	8724	1	1466
NM_020315	0.39790044	0.23917518	-0.741115431	0.035888679	8713	1	1389
NM_052925	-0.0850897	0.77199189	-0.800286728	0.027811518	8838	1	1339
NM_024841	-0.0137067	1.0464789	-1.007482503	0.005389517	7238	1	1314
NM_052956	0.68909863	0.06895972	-0.794596949	0.009215626	8733	1	1896
NM_052956	1.13221907	0.57702794	-0.794596949	0.001408563	9642	1	2070
NM_178314	0.26896376	1.01549723	-0.781740823	0.000166533	8296	1	508
NM_178314	0.13194379	1.12830771	-0.781740823	0.000204078	8298	1	750
NM_002393	0.04219917	1.10357915	-0.701873054	0.021338507	8945	1	1555
NM_000398	-0.0759255	0.85441978	-0.758645663	0.001865996	8167	1	86
NM_000398	0.00825895	0.8723485	-0.758645663	0.002082619	8168	1	124
NM_031283	0.11089687	0.10680575	-0.844217637	0.027881768	7532	1	1452
NM_031283	-0.2163273	0.23774663	-0.844217637	0.026311081	7529	1	1190
DR773623	0.10201802	0.93987605	-0.677413119	0.001736737	8266	1	376
DR773623	-0.058143	0.92231264	-0.677413119	0.001409627	8263	1	224
NM_001390	0.29956409	1.12906663	-0.569838427	0.000371961	8356	1	1049
NM_032292	-0.1671956	0.68864184	-0.810480003	0.00057828	7659	1	34
NM_032292	-0.2913219	0.92796213	-0.810480003	0.002349134	8162	1	423
NM_014723	0.298546	0.50455835	-0.572798984	0.016192026	8001	1	1128
NM_014723	-0.3146108	1.15247608	-0.572798984	0.015183614	6671	1	1647
NM_013417	-0.7130287	0.4541167	-0.79843128	0.040490554	6630	1	1776
NM_024726	-0.4268074	0.53995982	-1.637953842	0.02734744	6038	1	2152
NM_001456	-0.8410884	-0.102573	-0.770187313	4.87E-05	6426	1	1969

NM_001456	-0.9143607	-0.0045443	-0.770187313	4.91E-05	6427	1	1961
NM_005595	-0.2574278	0.41079347	-0.8113475	0.017077904	8002	1	477
NM_000102	0.29501834	1.02736283	-0.542626263	0.011406542	8882	1	1306
NM_004643	-0.1478529	1.13195896	-0.256508865	0.009196169	9046	1	1352
NM_004643	0.01516971	1.0660896	-0.256508865	0.007496167	9047	1	1053
NM_178176	0.25970655	0.25637038	-0.846354243	0.008501143	7533	1	1307
NM_003793	-0.1944225	0.85806483	-0.851226133	0.000428569	8009	1	246
NM_003793	0.03849877	1.02893917	-0.851226133	0.000270609	8309	1	349
NM_032433	0.55914043	0.49390136	-0.771667858	0.023803587	8803	1	1550
NM_018963	0.41002168	0.61705003	-0.679091791	0.020220082	8738	1	1116
NM_031477	-0.2284896	1.21916539	-0.744027438	0.000789109	8253	1	1226
NM_000438	0.70514546	0.74496323	-0.513541034	0.009723661	8834	1	1733
NM_022453	-0.5107142	-0.1426436	-0.789995523	0.001104845	6423	1	1709
NM_001008701	-0.9934928	0.26258817	-0.807780231	0.023162224	6628	1	1995
XR_012519	0.42617815	0.97925898	-0.449727811	0.001144475	8570	1	979
XR_012519	0.37497419	1.05453155	-0.449727811	0.007181824	8566	1	1175
NM_002892	-0.1821663	0.95926332	-0.527712215	0.007165963	8212	1	811
NM_002892	-0.9300112	0.73089283	-0.527712215	0.029172768	6590	1	1954
NM_145236	0.37836169	0.56694781	-0.40923311	0.010055489	8381	1	1329
NM_017869	-0.1679194	0.80826871	-0.369020491	0.009951397	8096	1	277
XR_014317	0.02520502	0.06030212	-1.11960827	0.01777001	6499	1	1599
XR_014317	-0.4559116	0.14214325	-1.11960827	0.015125879	6495	1	1724
XR_014317	-0.1843757	0.06714091	-1.11960827	0.025308124	6498	1	1636
XR_014317	-0.1308737	0.31776105	-1.11960827	0.010301317	6500	1	1134
NM_006253	0.28869253	1.17037399	-0.499096458	0.001812205	8364	1	1125
NM_006482	0.5518508	0.37186647	-0.963709014	0.002870489	8784	1	1351
NM_006482	0.66735813	0.81257621	-0.963709014	0.001181786	8790	1	1585
NM_031212	-0.2985269	0.90444923	-0.580737616	0.000370235	8228	1	225
NM_031212	-0.7061057	0.92411111	-0.580737616	6.98E-05	7504	1	1256
NM_002687	-0.4897919	0.68501202	-1.038894594	0.012695371	6759	1	1432
NM_002687	-0.408157	0.86317774	-1.038894594	0.047924068	6677	1	1962
NM_005238	0.60864445	0.33310836	-0.589935847	0.009558375	7738	1	1255
NM_005238	0.50180303	0.42324283	-0.589935847	0.023870194	7737	1	1010
NM_018702	0.64860107	0.34269458	-0.979245067	0.009846067	8745	1	1729
NM_006804	0.04937456	-0.0133814	-0.582676023	0.028534212	7560	1	1064
NM_006804	-0.0345145	0.28856872	-0.582676023	0.005671422	7598	1	210
NM_005884	0.55730574	0.24416555	-0.825343179	0.019383994	8708	1	1605
NM_032552	-0.3251477	1.1190582	-0.673074371	0.000750807	9012	1	705
NM_032552	-0.4037384	1.10747534	-0.673074371	0.001395823	9011	1	879
NM_022336	0.38222968	0.62488717	-0.624561896	0.022010305	8727	1	1042
NM_001459	-0.2372291	0.32851364	-0.73040871	0.042778514	7350	1	960
NM_014563	-0.1560012	0.26516592	-0.495546849	0.049609186	7613	1	670
NM_003624	-0.2901163	0.74495499	-0.876981999	0.000889241	8159	1	192

NM_003624	-0.2656403	0.80700306	-0.876981999	0.000695709	8160	1	171
NM_022124	0.03575487	0.34082534	-0.582251185	0.004891417	7580	1	198
XR_014490	-0.0533327	1.19122088	-0.678804507	0.000325237	8317	1	720
NM_014329	0.08578023	-0.379332	-0.839992799	0.045657364	6513	1	1944
NM_006279	-0.4588745	0.57836953	-0.957530686	0.003318377	6823	1	1013
NM_006279	-0.6202541	0.63505349	-0.957530686	0.001202595	6813	1	1450
NM_139161	0.0154068	1.03679625	-0.581404334	0.001505544	8247	1	461
NM_139161	-0.3383431	1.06979594	-0.581404334	0.000772216	8236	1	742
NM_139161	0.02718022	0.87492035	-0.581404334	0.004250928	8094	1	190
NM_023926	-0.7060819	0.89906589	-0.568364904	0.002625443	7503	1	1448
NM_023926	-0.736903	0.98948401	-0.568364904	0.002620595	7506	1	1542
NM_015375	-0.5393485	0.11093313	-0.670376961	0.00261456	6566	1	1728
NM_021964	-0.1692943	0.7460509	-0.757911818	0.000139652	8277	1	60
NM_021964	0.21924161	0.54932959	-0.757911818	0.001779055	7682	1	105
NM_006802	0.18867693	-0.4657174	-0.854529685	0.041195768	6511	1	2033
NM_000081	0.16168978	0.80321591	-0.532131911	0.01684218	8407	1	446
NM_176801	-0.0532223	0.48067029	-0.588769039	0.000371046	8062	1	9
NM_176801	-0.0164463	0.61896059	-0.588769039	0.001062057	8071	1	2
NM_176801	-0.0195682	0.59137414	-0.588769039	0.00372585	8069	1	23
NM_176801	0.03677678	0.74219784	-0.588769039	0.002907272	8099	1	33
NM_004818	0.16800421	-0.0498195	-0.708651601	0.013240726	7693	1	1177
NM_004818	0.01384063	0.14715525	-0.708651601	0.0446562	7623	1	680
NM_033086	-1.119405	0.64317578	-0.559345246	0.015901394	6575	1	2035
NM_033086	-0.7592431	0.79201573	-0.559345246	0.001614956	7502	1	1333
NM_003017	-0.5206576	0.53250564	-0.614859434	0.003425109	6849	1	480
NM_017739	-0.2763089	0.90787209	-0.606635924	0.000695629	8235	1	173
NM_017739	-0.1853351	1.04276962	-0.606635924	0.002119282	8191	1	489
NM_018431	0.51621526	0.90705419	-0.727172206	0.006583313	8751	1	1514
NM_018431	0.58590995	0.82830649	-0.727172206	0.00464046	8750	1	1554
NM_004193	-0.1933745	0.66144105	-0.943027129	0.001183021	6856	1	203
NM_004193	-0.4968228	0.50755635	-0.943027129	0.001828446	8014	1	791
XM_290831	0.09642569	1.06344752	-0.936674414	0.023000756	8862	1	1948
NM_014174	0.42167739	0.46906658	-0.763651731	0.01183021	7758	1	849
NM_014174	0.32238685	0.58780258	-0.763651731	0.006024002	7759	1	375
NM_014312	-0.710897	1.0596926	-0.48211011	0.00110431	6579	1	1908
NM_001217	-0.3732226	0.71278884	-0.717724436	0.003140614	6852	1	282
NM_001217	-0.3409397	0.6172829	-0.717724436	0.00106474	8003	1	303
NM_032620	-0.0179278	0.3220033	-0.623301804	0.002419445	8060	1	56
NM_032620	0.14421614	0.19466965	-0.623301804	0.021954375	7629	1	600
NM_004036	0.1482851	0.60085972	-0.489577859	0.000842442	8089	1	62
NM_004036	0.42919586	0.80269534	-0.489577859	0.003808732	8435	1	986
NM_138473	-0.8434036	-0.2795122	-0.563590751	0.034388713	6441	1	2069
NM_138499	0.49952927	0.33546968	-0.507557213	0.004327531	7732	1	961

NM_138499	0.67215659	0.37491469	-0.507557213	0.002772125	7740	1	1429
NM_001699	0.14109574	-0.0493994	-0.710058753	0.001475703	7692	1	1143
NM_001699	0.31151135	0.3401978	-0.710058753	3.88E-05	7727	1	263
NM_015246	-0.0540735	0.41255436	-0.635065113	0.001967236	8061	1	24
NM_015246	-0.2182824	0.42705834	-0.635065113	0.00279918	7637	1	240
NM_024652	-0.2973577	0.94033728	-0.695760872	9.10E-06	8011	1	903
A_01_P008101	0.05865855	1.1297106	-0.817366336	2.74E-05	8311	1	535
A_01_P008101	-0.0153097	1.12398029	-0.817366336	0.000127491	8310	1	507
NM_007079	-0.681475	0.49109107	-0.690325193	4.44E-05	7373	1	926
NM_007079	-0.7744982	0.39950304	-0.690325193	5.35E-06	7372	1	1417
NM_022372	0.3141522	0.84775089	-0.735606907	0.019311291	8880	1	1353
NM_022372	0.2331997	0.65512628	-0.735606907	0.011461032	8722	1	738
XR_011558	0.03411684	0.39739676	-0.38798267	0.029241053	7493	1	455
XR_011558	-0.299091	0.81371979	-0.38798267	0.003528506	8117	1	445
NM_015382	0.40706816	0.8868055	-0.375642437	0.002985675	8569	1	892
NM_015382	0.5828404	0.98489003	-0.375642437	0.001714511	8571	1	1410
NM_024077	0.07414703	1.01794515	-0.885710634	0.002116277	8171	1	667
NM_024077	-0.6907977	1.02389698	-0.885710634	0.003997645	6665	1	1913
XR_000285	-0.1621771	1.17171632	-0.777151806	0.00110714	9016	1	823
XR_010309	-0.1136084	0.79115656	-0.584312716	2.26E-05	8097	1	5
XR_010309	0.08332404	0.91100777	-0.584312716	0.000192566	8175	1	55
NM_000840	0.13194115	0.71833626	-0.69094818	0.011610641	8411	1	343
NM_001005335	0.385572	0.78174802	-0.616381988	0.00334257	8422	1	426
NM_001005335	0.51069858	0.86764618	-0.616381988	0.002844316	8423	1	936
NM_000181	0.06284257	0.92695888	-0.71966218	0.002240206	8169	1	191
NM_000413	0.17644918	-0.1803755	-0.841832091	0.005799696	6517	1	1633
NM_000413	0.20574591	0.05043096	-0.841832091	0.001256759	7697	1	978
NM_006746	-0.4673972	0.83323588	-0.713400246	0.020363913	6598	1	1198
NM_031472	-0.591285	0.27347878	-0.435607894	0.026167438	7361	1	1523
NM_031472	-0.1370069	0.56953071	-0.435607894	0.010790278	7906	1	95
NM_000494	-0.1926043	0.33976248	-0.619157521	0.028165594	7790	1	427
NM_145252	-0.0550024	-0.1621862	-0.549803473	0.010385948	7859	1	1455
NM_145252	0.25404502	0.62222475	-0.549803473	0.015786294	8728	1	518
NM_005750	0.49549242	0.64228265	-0.891344462	0.004429781	8758	1	1552
NM_015448	0.17902639	0.30291782	-0.567929996	0.004629199	7720	1	211
NM_000851	-0.4388598	1.20643781	-0.532900249	0.005685579	8251	1	1616
NM_000851	-0.2998761	1.11788792	-0.532900249	0.003430362	8252	1	1072
NM_013260	-0.3097431	1.0472368	-0.702802125	0.001227181	9010	1	536
CN643015	-0.5220022	-0.1098957	-0.578415638	0.049945543	6442	1	1697
XM_497821	0.23403646	0.77086288	-0.813021035	0.023885416	8839	1	1823
NM_130781	-0.0526925	1.1303621	-0.895168315	0.000499072	8307	1	772
NM_003086	0.18014645	1.01946928	-0.477386734	0.002223544	8354	1	637
NM_003086	0.31827801	1.09602316	-0.477386734	0.000780703	8355	1	951

NM_015414	0.00449528	0.16628079	-0.607659703	0.00205362	7599	1	346
NM_015414	0.06405408	0.32707829	-0.607659703	0.000152632	7600	1	64
NM_006659	0.08373145	0.59477948	-0.543111789	0.000485267	8072	1	8
NM_006659	0.07902003	0.5257883	-0.543111789	0.01067311	8054	1	61
NM_002447	-0.7531921	1.17146147	-0.489486381	0.004506109	7508	1	1883
NM_052868	-0.5264847	0.00200642	-0.579468582	0.005849757	6432	1	1497
XR_013847	-0.0258136	0.15314558	-0.423848461	0.010077191	7817	1	470
NM_001329	-0.4729645	0.69673806	-0.562882224	0.006485942	8033	1	482
NM_001329	-0.3149185	0.82243899	-0.562882224	0.002253318	8234	1	163
NM_015266	-0.6417036	-0.2354227	-0.643601669	0.028920746	6565	1	2065
NM_015266	0.28312701	0.0578965	-0.643601669	0.021470681	7866	1	1110
NM_144588	-0.168113	0.35106788	-0.833528336	0.009416102	8016	1	327
NM_144588	-0.1980592	0.36550215	-0.833528336	0.012947518	8018	1	368
NM_024684	0.37850131	0.3796065	-0.64929972	0.000963595	7728	1	466
NM_024684	0.402998	0.39523112	-0.64929972	0.001693142	7729	1	557
NM_005452	0.02331559	-0.28425	-0.783832337	0.016295792	6514	1	1746
NM_014726	-0.1154648	0.08842602	-1.05664687	0.006220178	6501	1	1261
NM_014726	0.01345476	0.30614514	-1.05664687	0.003027438	6502	1	822
NM_019005	-0.0348601	0.19686721	-0.580367282	0.000781283	7803	1	294
NM_019104	0.08004017	0.59060944	-0.609979872	0.00249156	8274	1	47
NM_182744	-0.0668325	-0.2404845	-0.668677971	0.001642731	7557	1	1615
NM_182744	-0.1841951	0.02076107	-0.668677971	0.001014123	7554	1	767
NM_016434	-0.2805687	0.44765028	-0.488834972	0.007449927	7413	1	214
NM_016434	-0.4620179	0.49573413	-0.488834972	0.004361208	7424	1	1012
NM_006736	-0.1757538	0.26561653	-0.302804647	0.03748493	7807	1	541
NM_006736	-0.2889726	0.27158903	-0.302804647	0.032777577	7806	1	659
NM_001311	-0.3165055	0.28310858	-0.561415899	0.021459358	7411	1	673
NM_001311	-0.3680914	0.50117728	-0.561415899	0.00760509	7412	1	373
NM_006844	-0.6570683	0.04688257	-0.661147754	0.038217467	6430	1	1723
XR_013480	0.42676234	0.59743283	-0.779716273	0.010003062	7760	1	732
XR_013480	0.11428331	0.53230949	-0.779716273	0.047726971	7765	1	888
NM_002038	0.13139588	1.12355734	-0.610956024	1.51E-07	8349	1	428
NM_002038	0.05944947	1.17138524	-0.610956024	4.41E-06	8348	1	519
NM_001003722	0.30392022	0.19777913	-0.561756002	0.008705644	7721	1	590
NM_001003722	0.26908402	0.36048859	-0.561756002	0.01343273	7723	1	342
NM_022046	-0.0665251	-0.1498504	-0.615095731	0.004756359	7558	1	1427
NM_015049	0.22147358	0.7270143	-0.6668656	0.002078283	8412	1	82
NM_015049	0.23315004	0.90664716	-0.6668656	0.003454598	8414	1	344
NM_007186	-0.807415	0.47003847	-0.530865611	0.009959967	7370	1	1632
NM_007186	-1.0559016	0.49205836	-0.530865611	0.000522286	7369	1	1904
XR_012262	0.14444894	0.31846287	-0.576462432	0.001106244	7719	1	148
XR_012262	0.37207253	0.32540152	-0.576462432	0.001079634	7718	1	688
NM_005275	0.28821553	0.24476396	-0.672923818	0.0196826	8716	1	1152

NM_019592	0.2441743	0.98280772	-0.670114928	0.001491488	9314	1	611
NM_019592	0.53925545	1.02157166	-0.670114928	0.002666052	9322	1	1293
NM_002733	0.24681834	0.36142877	-0.73637279	0.006174704	7717	1	388
NM_002733	-0.0381809	0.70766622	-0.73637279	0.000955636	8164	1	35
NM_022734	-4.97E-05	1.01280108	-0.610472724	0.00336686	8340	1	424
NM_015230	-0.0681449	0.36487993	-0.732999104	0.018824405	7636	1	451
NM_015230	-0.0180221	0.55126652	-0.732999104	0.004556943	7663	1	40
NM_006185	-0.276961	0.16412981	-0.793563283	0.002004526	7562	1	567
NM_006185	-0.335144	0.52317021	-0.793563283	0.000853839	6851	1	177
NM_004699	-0.2759885	0.50174467	-0.309735512	0.018521821	7482	1	486
NM_004699	0.09421343	0.73620734	-0.309735512	0.020244393	8478	1	624
NM_004699	0.05806438	0.59942049	-0.309735512	0.026929047	7496	1	419
NM_001031	-0.098841	0.82120695	-0.831125247	0.001404704	6861	1	160
NM_001031	-0.1003322	0.52416817	-0.831125247	0.003868909	7662	1	88
NM_005641	-0.0729996	0.20411138	-0.66817108	0.000209441	7583	1	188
NM_005641	-0.1392261	0.17058581	-0.66817108	0.036190121	7620	1	601
NM_031215	-0.1823443	0.05206101	-0.456104736	0.026700867	7800	1	1186
NM_023039	-0.0630617	0.66670163	-0.556808922	0.004466311	8092	1	42
NM_023039	0.15106911	0.90454051	-0.556808922	1.27E-05	8458	1	152
NM_021138	0.26481268	0.39580821	-0.674344308	0.015009294	7715	1	360
NM_021138	0.09508555	0.79961578	-0.674344308	0.000316318	8174	1	20
NM_021038	0.57927986	0.33517122	-0.706667616	0.013542153	7736	1	1280
NM_021038	0.4199055	0.46534712	-0.706667616	0.031179275	7755	1	950
NM_014310	0.41692045	1.07991301	-0.817567801	0.027082705	8864	1	2008
XR_010261	0.14636626	0.30270136	-0.52765502	0.02112927	7857	1	408
NM_017566	0.1686152	0.20004712	-0.755221651	0.003043252	7708	1	401
NM_017566	-0.0335629	0.13062646	-0.755221651	0.014079273	7622	1	641
NM_144568	0.0643671	0.48341902	-0.557488053	0.000958098	8272	1	85
NM_144568	-0.3777818	0.61342571	-0.557488053	5.84E-05	7415	1	118
NM_031899	-0.3960512	0.2386117	-0.347171348	0.011273111	7805	1	847
NM_032528	0.49148442	-0.1756915	-0.562509253	0.016113426	8909	1	1843
NM_006313	0.43724232	0.83621148	-0.285789198	0.014929774	8485	1	1230
NM_020202	-0.477537	-0.192006	-0.474631652	0.041447106	6443	1	1825
NM_001331	-0.2218859	0.76928054	-0.635084667	0.008173422	8147	1	199
NM_001331	-0.063638	1.05792462	-0.635084667	0.001472557	8193	1	365
NM_018273	-0.2688133	0.40491527	-0.501661118	0.006630137	7410	1	228
NM_018273	0.14405291	0.54918601	-0.501661118	0.004093424	8275	1	155
CB548748	-0.3412775	1.05022746	-0.466519468	0.007740505	6604	1	953
NM_017612	0.66761776	1.12744884	-0.770276866	0.043912546	8992	1	2082
NM_004435	0.30346193	0.3419535	-0.737408181	0.000604977	7707	1	391
NM_004435	0.53097769	0.15937793	-0.737408181	0.044217707	7687	1	1608
CK231666	-0.1708513	0.98540005	-0.514888459	0.000244993	8336	1	186
CK231666	-0.2614746	0.83131932	-0.514888459	0.006595453	8148	1	249

NM_024070	0.31725352	0.17049698	-0.817230387	0.018641159	8688	1	1705
NM_024070	0.07711721	0.45556593	-0.817230387	0.024455874	7764	1	498
NM_182507	0.6113414	0.23096315	-0.484346704	0.01469905	8735	1	1543
XR_013622	-1.1574678	0.33681195	-0.901320035	0.007484699	6017	1	2175
NM_014790	-0.8198849	0.47012437	-0.863463281	0.018658392	6571	1	1935
NM_016200	-0.214887	0.66492947	-0.464280762	0.028050088	6606	1	1195
NM_001675	0.090693	0.43100426	-0.601645686	0.031143603	8052	1	229
NM_001675	0.11134528	0.55198227	-0.601645686	0.018722922	8053	1	134
XR_012155	0.79165258	0.76051518	-0.58970302	0.03706275	8766	1	1989
NM_001985	0.1141054	1.10828518	-0.540728569	2.26E-05	8350	1	413
NM_001985	0.22638429	1.12507697	-0.540728569	0.000110143	8351	1	608
NM_016563	-3.0304578	0.5579934	-0.601923683	0.022921054	12	1	2192
NM_016563	-0.0411773	0.61293205	-0.601923683	0.010572959	8048	1	206
NM_144598	-0.2606539	-0.0287173	-0.626009637	8.89E-05	7556	1	1145
NM_144598	0.02282078	0.01666958	-0.626009637	0.001600591	7559	1	899
NM_018668	-0.0430628	0.21187501	-0.506865816	0.037868505	7597	1	569
NM_018668	0.01511058	-0.0475818	-0.506865816	0.018010984	7860	1	1206
NM_054013	0.2295743	0.87708673	-0.512510514	0.000483104	8552	1	153
NM_012292	-0.1178631	0.28591294	-0.55425678	3.70E-05	7593	1	73
NM_012292	-0.1587277	0.11502941	-0.55425678	0.013813018	7621	1	511
NM_153618	-0.899881	1.12248964	-0.103112506	0.035203849	6053	1	2154
NM_153618	-1.0104522	1.19765023	-0.103112506	0.018234132	6052	1	2164
NM_014864	0.27988806	0.65700652	-0.652673627	0.0120946	8405	1	393
NM_022167	-0.0199767	0.31170016	-0.44583092	0.002303601	7829	1	169
NM_022167	-0.0457916	0.3078184	-0.44583092	0.000647841	7831	1	111
NM_014624	-0.0838457	1.09810504	-0.628112828	0.000860411	8194	1	407
NM_183057	-0.6496777	0.36518988	-0.454265288	0.001553198	7392	1	1029
NM_183057	-0.7737738	0.50986514	-0.454265288	0.001428004	7393	1	1377
NM_183057	-0.6298893	0.47780033	-0.454265288	0.001309302	7394	1	904
NM_183057	-0.8310442	0.50165344	-0.454265288	0.001788644	7391	1	1528
NM_005740	0.40846265	0.07614578	-0.491580617	0.007039128	7868	1	1381
NM_004819	0.37788276	0.54439989	-0.728722212	0.03643333	8759	1	1548
NM_004819	0.10476563	0.53942595	-0.728722212	0.002653652	8271	1	371
AY952296	-1.1208888	0.24111325	-0.487904393	0.044496009	6567	1	2107
AY952296	-0.528194	0.19484576	-0.487904393	0.014408545	7382	1	1089
NM_003869	0.06402383	0.5131523	-0.802996733	0.00187371	8040	1	252
NM_003869	0.23288566	0.62929931	-0.802996733	0.013911311	7766	1	722
NM_015461	0.33822049	0.71274842	-0.484186711	0.012541194	8482	1	766
NM_004840	0.18080474	1.1676042	-0.525407426	3.49E-06	8352	1	717
NM_004840	0.30636486	1.11223734	-0.525407426	4.29E-06	8353	1	789
NM_005966	-0.0544294	0.40249169	-0.359253419	0.008984263	7907	1	270
NM_022492	-0.9332525	0.70489611	-0.467236956	0.017083088	6595	1	1826
NM_005968	-0.0786405	0.46721519	-0.585044145	0.00586195	8067	1	28

NM_005968	-0.0451097	0.53685101	-0.585044145	0.004237117	8068	1	15
XR_011061	0.26533899	0.77526164	-0.692126078	0.010527736	8413	1	443
XR_011061	0.25875667	0.99600141	-0.692126078	0.005079733	9315	1	736
NM_013274	-0.8158041	0.57153913	-0.966977721	0.047016161	6564	1	1999
NM_005716	-0.2061257	0.22680297	-0.656604489	0.00340848	7615	1	220
NM_005716	-0.2869532	0.39076151	-0.656604489	0.001975775	7591	1	96
NM_001001998	-0.0029142	0.2297608	-0.492090663	0.033989669	7794	1	544
NM_001001998	-0.1636298	0.26710672	-0.492090663	0.007941189	7791	1	386
NM_014745	0.11714352	0.68662015	-0.376842371	0.002624599	8461	1	156
NM_014745	0.14337596	1.10452427	-0.376842371	0.000670068	9077	1	821
NM_005468	0.01609059	0.24909295	-0.977514898	0.010702674	7643	1	971
NM_005468	0.39039623	0.62675656	-0.977514898	0.00624618	8739	1	1192
NM_025258	0.55504761	0.73502698	-0.759435999	0.001257671	8748	1	997
NM_017730	0.40602204	1.05316503	-0.786895461	0.000576469	9311	1	1034
NM_017730	0.47174471	1.12875685	-0.786895461	0.003731121	9319	1	1533
XR_010302	0.05828444	1.19211952	-0.55786443	0.000624402	8346	1	869
NM_001467	-0.1088142	0.58375387	-0.835482911	0.00129518	8039	1	147
NM_001467	-0.0080979	0.75827228	-0.835482911	0.003616279	8165	1	174
CN642091	-0.0287696	0.69100673	-0.380695471	0.001602612	8100	1	32
CN642091	0.10524783	0.77398171	-0.380695471	0.000654574	8101	1	70
NM_004082	0.29994616	-0.0188221	-0.482565527	0.008968365	7867	1	1279
NM_144997	0.13777236	1.0914055	-0.495204527	0.004824856	8856	1	1393
NM_144997	-0.0984966	1.19392009	-0.495204527	0.00310976	8374	1	1564
NM_032827	0.17145236	0.97668093	-0.608449292	0.000615376	8344	1	325
NM_032827	0.21430315	1.05833009	-0.608449292	0.000232217	8347	1	456
NM_018276	-0.1495482	0.6524737	-0.716992366	0.000168347	8075	1	3
NM_018276	-0.3928639	0.51783506	-0.716992366	0.008609633	8021	1	396
NM_018276	-0.3000849	0.43792843	-0.716992366	0.014672715	8019	1	284
NM_018276	-0.3612923	0.72584178	-0.716992366	0.003260824	8144	1	219
NM_000982	-0.2713259	0.19753849	-0.473598673	0.023896262	7839	1	474
NM_000982	-0.2335638	0.3466351	-0.473598673	0.013291735	7840	1	164
NM_001493	-0.03129	0.51398794	-0.333002258	0.047999508	7495	1	543
NM_006157	0.0106281	0.30014598	-0.506839523	0.02348799	7336	1	1244
A_01_P005857	0.1746721	0.76519778	-0.295023418	0.009312379	8463	1	444
NM_138614	-0.0510641	0.41286231	-0.45670389	0.010216099	7846	1	83
NM_138614	0.15578616	0.71466564	-0.45670389	0.00634044	8409	1	108
NM_004644	0.11476881	1.01502406	-0.305465216	0.038184916	8979	1	1648
NM_023944	-0.1781426	1.11430216	-0.877717088	0.0019423	8256	1	1178
NM_023944	-0.0290912	1.14922684	-0.877717088	0.000601277	8308	1	861
NM_024712	-0.4929513	0.46369297	-0.56312744	2.17E-05	7405	1	369
NM_024712	-0.4104608	0.69190465	-0.56312744	0.004156248	8034	1	324
AB209633	0.30779498	0.6493534	-0.631274481	0.001600362	8808	1	333
AB209633	0.2403196	0.59090073	-0.631274481	0.001981495	8080	1	75

NM_019030	-1.601081	0.05037135	-0.681371728	0.029804271	6028	1	2187
XR_014633	-0.1104586	1.05760026	-0.62495194	0.000514901	8244	1	389
XR_014633	-0.221415	1.20026006	-0.62495194	0.000212416	8240	1	843
NM_198083	-0.1542724	0.32062538	-0.815604731	0.012688581	8017	1	525
NM_198083	0.12820459	0.69107253	-0.815604731	0.002047838	8166	1	103
NM_198083	0.40969588	0.72821293	-0.815604731	0.001565774	7763	1	594
NM_198083	0.21293845	0.64644176	-0.815604731	0.031257468	7767	1	1036
NM_000990	0.29994389	0.22556746	-0.551077222	0.000428887	7722	1	447
NM_000990	0.33639602	0.30880313	-0.551077222	0.037573119	7724	1	678
NM_013450	-0.2666442	0.77650112	-0.494755287	0.002263104	8012	1	588
NM_015517	0.11974079	0.52153265	-0.537463814	0.005262485	8083	1	52
NM_015517	0.23413865	0.44882787	-0.537463814	0.013403585	7725	1	290
NM_002690	0.03564488	0.25215614	-0.653867668	0.00873655	7628	1	250
NM_025129	0.41923034	0.43167308	-0.497067878	0.02690879	8729	1	1066
NM_032886	-0.6296908	1.00867352	-0.847708922	0.014666533	6675	1	1807
NM_032886	-0.3671747	0.89808425	-0.847708922	0.046316024	6679	1	1816
CN644267	0.46251317	0.4885036	-0.451308956	0.008626699	8398	1	764
CN644267	0.70092878	0.65009995	-0.451308956	0.007911182	8446	1	1499
NM_175854	-0.2323446	0.83171995	-0.820007255	0.015673049	8150	1	810
NM_015112	0.27661624	0.35694538	-0.265634797	0.020012568	7903	1	754
NM_006625	-0.307121	1.22261024	-0.502665567	4.62E-05	8242	1	966
NM_006625	-0.3934853	0.89359142	-0.502665567	0.005856566	8180	1	696
NM_006390	-0.0854537	0.8058454	-0.481015502	0.013263821	8184	1	197
NM_001915	-0.2674925	0.8711831	-0.4570056	0.041896951	8916	1	1549
NM_017658	0.29697413	0.55361553	-0.341395202	0.011305421	8436	1	404
NM_017658	0.41595399	0.64339055	-0.341395202	0.030200684	8434	1	988
NM_032412	-0.0792585	0.92038711	-0.505949026	9.94E-05	8245	1	79
NM_032412	-0.1421977	1.05133597	-0.505949026	0.000218579	8192	1	285
NM_182499	-0.3320063	0.99474957	-0.26249671	0.035969191	6393	1	2090
XM_042698	-0.4589508	0.09107971	-0.97754361	0.040185138	6416	1	1660
XR_012890	0.91626632	0.73883611	-0.770822215	0.026232175	8770	1	2043
CB230125	0.63419469	0.70185053	-0.659675974	0.00789879	8794	1	1277
CB230125	0.70024045	1.14500629	-0.659675974	0.001036967	9323	1	1758
NM_030785	-0.4308446	0.52929496	-0.820378942	0.034715356	6573	1	1537
NM_020801	0.48535669	0.71289189	-0.335982526	0.030657646	8438	1	1151
NM_020801	0.5113442	1.0981807	-0.335982526	0.002200348	8573	1	1480
NM_018555	-1.0791245	1.15781757	-0.709550711	0.001121335	6559	1	2062
NM_015846	-0.0404729	0.9516101	-0.660926977	0.004552533	8177	1	308
NM_001762	0.4209151	1.07643414	-0.546421082	0.002226262	9317	1	1071
NM_173617	0.5294815	1.04839431	-0.54372192	0.000397804	8824	1	1398
NM_014569	-0.1037742	0.15959525	-0.651741467	0.013068404	7564	1	503
NM_014569	-0.0828467	0.36582315	-0.651741467	0.004244112	7633	1	71
NM_032222	0.0583402	0.39752403	-0.395242023	0.030777015	7910	1	316

NM_032222	0.10581094	0.92499607	-0.395242023	0.007186751	8359	1	616
NM_004581	-0.6488205	0.31311441	-0.36966927	0.009476302	7384	1	1262
NM_004581	-0.5624844	0.44701405	-0.36966927	0.006434903	7385	1	1006
NM_004380	0.20903846	0.23325707	-0.420992511	0.002346408	7905	1	658
NM_004380	0.15664359	0.4937087	-0.420992511	0.048955383	7912	1	500
NM_002067	0.17845319	0.18227594	-0.487003159	0.024602417	7874	1	687
CK231382	0.0152937	0.16802827	-0.629738251	0.007256557	7626	1	381
CK231382	0.0671764	0.35208385	-0.629738251	0.003062193	7711	1	59
NM_001004055	-0.2836606	0.80409173	-1.084625419	0.00465884	6854	1	1144
NM_031209	0.30009546	0.6587062	-0.104000252	0.046663305	8496	1	1269
CO581794	-0.2787399	0.18746849	-0.554634765	5.27E-06	7590	1	374
CO581794	-0.3733489	0.34282437	-0.554634765	0.00015012	7404	1	182
NM_004259	-0.0399664	0.26926367	-0.313757945	0.042175836	7826	1	559
NM_004259	-0.114842	0.52437151	-0.313757945	0.007057059	7483	1	354
NM_032349	0.10693997	0.7412376	-0.369295496	0.001136132	8464	1	130
NM_032349	0.09159917	0.9096461	-0.369295496	0.000850548	8465	1	226
NM_003968	0.60462115	1.02206239	-0.701013576	0.001315343	8825	1	1562
NM_172390	-0.3091106	0.77216119	-0.680606912	0.012424929	8007	1	593
NM_172390	-0.0316534	0.6498503	-0.680606912	0.029606972	8008	1	865
CN647132	0.3962169	1.02729642	0.013635198	0.023862496	8616	1	1978
NM_206920	-0.6071573	0.30382177	-0.705324665	0.005958509	7374	1	1254
NM_206920	-0.2594444	0.20797658	-0.705324665	0.000917097	7588	1	348
NM_013266	-0.0807312	0.32656706	-0.474681598	0.009909113	7788	1	568
NM_001013	-0.013176	0.27684593	-0.413243248	0.001848143	7830	1	196
NM_001013	0.02446961	0.35944902	-0.413243248	0.000702759	7833	1	68
NM_007065	-0.1237132	0.50604882	-0.440465284	0.001962327	7848	1	27
NM_007065	-0.1046173	0.85249527	-0.440465284	0.000293089	8098	1	38
NM_052842	0.05394671	0.96320204	-0.61304981	0.007280665	8968	1	656
NM_052842	0.08015059	1.19008827	-0.61304981	2.66E-05	8345	1	689
NM_001009905	-0.0850914	0.27711977	-0.697889434	0.00866418	7627	1	200
NM_013275	-0.550859	0.93418313	-0.418211403	0.000814328	7505	1	912
NM_024725	-0.7944577	0.81249315	-0.353642345	0.048225298	6591	1	1924
NM_024725	-0.4888367	0.85624946	-0.353642345	0.041086899	6599	1	1387
NM_005418	0.0301569	1.01602874	-0.444172697	0.001668693	8248	1	513
NM_005418	-0.1281144	0.97125516	-0.444172697	0.000291162	8246	1	253
NM_178557	0.41054488	0.68022946	-0.858219809	0.010866409	8804	1	1209
NM_178557	0.53147366	0.73951854	-0.858219809	0.00673209	8805	1	1344
XM_371715	-0.0908058	0.42682514	-0.561181841	0.049853024	8049	1	321
NM_152879	-0.2508966	0.82552733	-0.73047565	0.00197412	8161	1	133
NM_001328	0.53576942	0.43713111	-0.55785728	0.006991821	7739	1	851
NM_001328	0.49271318	0.71578359	-0.55785728	0.000556056	8424	1	562
NM_025247	-0.5969828	0.43434957	-0.665920036	0.030089727	6551	1	1551
NM_025247	-0.3196862	0.54777869	-0.665920036	0.031291717	8145	1	506

NM_015125	-0.147559	0.15217097	-0.423898067	0.011425493	7802	1	573
NM_002402	-0.2158771	0.51309335	-0.788898859	0.014504442	7527	1	825
NM_001763	0.135775	0.69193963	-0.526707999	0.000203805	8408	1	51
XR_014641	-0.1244609	0.32176226	-0.338356423	0.025405222	7825	1	405
NM_080599	0.67099117	0.64350085	-0.348229769	0.004462307	8445	1	1374
NM_080599	0.39344447	0.6122415	-0.348229769	0.01107735	8437	1	572
NM_021185	-1.0874721	0.65580227	-0.898960636	0.044729307	6554	1	2137
NM_021185	0.25638823	1.10966742	-0.898960636	0.015723157	8969	1	1812
XR_012927	-0.509	0.08506724	-0.530704878	0.000745174	6434	1	1146
XR_012927	-0.4290731	0.330177	-0.530704878	0.006066085	7408	1	597
NM_001273	0.26253164	0.10877878	-0.484986061	0.006630998	7875	1	852
NM_001273	0.13757221	0.25418761	-0.484986061	0.000300736	7876	1	280
NM_004470	0.05703642	0.44248229	-0.680158184	0.029173513	7535	1	1370
NM_004470	-0.0581491	0.55403186	-0.680158184	0.029504435	9725	1	2148
NM_018338	-0.1279458	-0.0491094	-0.672032697	0.01366553	7780	1	1631
NM_002757	0.06807876	0.13964495	-0.306767861	0.030507808	7818	1	778
NM_002757	0.11080081	0.23044905	-0.306767861	0.039635971	7920	1	598
NM_002757	0.1544596	0.34947466	-0.306767861	0.007451866	7922	1	309
NM_002757	-0.0389397	0.50481362	-0.306767861	0.003361508	7838	1	102
NM_018253	-0.2119629	0.0736292	-0.31387737	0.025115224	7809	1	934
NM_032872	-0.3470799	0.69656645	-0.705031972	0.020468326	8214	1	958
NM_032872	-0.1571046	0.93296363	-0.705031972	0.012289138	8216	1	1082
NM_001010987	-0.4244481	0.80041782	-0.809526137	0.01628786	8213	1	1456
NM_005331	-0.4418535	1.02787511	-0.993986926	0.007259729	6862	1	1686
NM_005331	-0.8648728	0.94236353	-0.993986926	0.003187844	6556	1	1923
NM_000044	0.05425413	-0.0861409	-0.362796847	0.021291349	7861	1	1435
NM_033375	0.23764828	0.23427868	-0.577421488	0.040919704	8715	1	911
NM_177441	-0.03769	0.11794373	-0.652822365	0.005332297	7624	1	463
NM_177441	-0.036955	0.35117613	-0.652822365	0.000403774	7592	1	49
NM_177441	-0.005788	0.47810957	-0.652822365	0.000226953	8073	1	14
NM_177441	0.0229036	0.56004944	-0.652822365	0.000186689	8074	1	7
NM_006009	-0.2399307	0.73929886	-0.431947879	0.001635576	7443	1	57
NM_006009	-0.3396296	0.79190122	-0.431947879	0.006261085	7442	1	279
NM_014603	0.61944503	0.5672239	-0.265653057	0.029469528	9635	1	1786
NM_015407	-0.2591443	0.72931304	-0.451598936	0.008920203	7462	1	399
NM_015407	-0.2444384	0.70180872	-0.451598936	0.00023302	7444	1	26
NM_018956	0.01825753	0.86129416	-0.082998223	0.040329399	8508	1	1205
NM_001004	0.19190027	0.30433702	-0.598195584	0.005741932	7712	1	184
NM_001004	0.20288814	0.42766403	-0.598195584	0.0081169	7713	1	127
XR_012331	0.18453069	0.46231564	-0.508068905	0.00169982	8276	1	98
XR_012331	0.20925036	0.6524898	-0.508068905	0.008773644	8406	1	175
NM_024618	-0.1077375	0.64171836	-0.438188469	0.002009107	7488	1	54
NM_024618	-0.3772179	0.70176711	-0.438188469	0.001730271	7447	1	310

NM_032289	0.31856862	0.17888459	-0.515647742	0.038029643	8905	1	1757
NM_015710	-0.0428274	0.23961436	-0.365702978	0.000618084	7832	1	247
NM_015710	-0.0675572	0.45256651	-0.365702978	0.000285517	7837	1	41
NM_003935	0.37714052	0.30572189	-0.435605616	0.001086294	7731	1	630
NM_003935	-0.0995859	0.72177103	-0.435605616	0.001275718	8118	1	161
NM_015429	-0.5042541	0.65046582	-0.68166782	0.006859106	8143	1	579
NM_175058	-0.9823393	0.87066754	-0.713222688	0.004326323	6558	1	2024
NM_015392	-0.4672052	0.40594707	-0.528056066	0.001595499	7414	1	538
NM_015392	-0.4985886	0.29877352	-0.528056066	0.000231743	7403	1	584
CO725391	-0.403412	0.32621413	-0.676392949	0.001952446	7402	1	358
CO725391	-0.5862595	0.30917296	-0.676392949	0.001577511	7401	1	976
NM_002899	0.27854264	0.28360732	-0.342626011	0.036858109	7902	1	798
NM_002899	-0.0676703	0.40838279	-0.342626011	0.006224234	7836	1	181
NM_003324	0.38935827	0.81826923	-0.707734468	0.013519287	8884	1	1172
NM_003324	0.25050398	1.06314837	-0.707734468	0.004971471	9302	1	1426
NM_022750	-0.2161232	0.29856584	-0.349633807	0.006300946	7843	1	272
NM_022750	-0.2281542	0.45694939	-0.349633807	0.009454449	7844	1	162
NM_153225	-0.36766	0.78578853	-0.279083023	0.02042161	9460	1	1651
NM_007121	-0.2427788	0.07845314	-0.516927828	0.0264142	7775	1	1118
NM_172231	0.16486688	1.11645122	-0.593479108	0.009082757	8886	1	1371
NM_172231	0.37655886	1.10503161	-0.593479108	0.008742107	8887	1	1557
NM_130761	-0.910711	1.12229015	-0.844613291	0.005089119	6557	1	2049
NM_033515	-0.1949087	1.13392365	-0.629262962	0.007143815	8370	1	1054
NM_033515	-0.215531	0.89564643	-0.629262962	0.036969059	8153	1	1257
NM_024491	-0.3841671	0.33330984	-0.794918067	0.01138389	6572	1	1503
NM_020232	-0.1940381	-0.0303608	-0.649919267	0.027802334	7553	1	1078
NM_020232	-0.2826815	0.23089864	-0.649919267	0.023934204	7610	1	684
NM_007202	-0.7314257	1.15159623	-1.031205856	0.012047469	6673	1	2050
NM_015345	-0.5847903	0.16367245	-0.510908426	0.02014072	7381	1	1343
NM_015345	-0.7325596	0.37561718	-0.510908426	0.006483403	7383	1	1338
NM_032819	0.23087531	0.79541964	-0.360393016	0.008372834	8487	1	545
NM_032819	0.17161068	0.93498136	-0.360393016	0.001048211	8553	1	364
NM_139015	-0.2636703	0.46749719	-0.345969646	0.018366265	8035	1	492
NM_139015	-0.3290336	0.81410257	-0.345969646	0.001504332	7450	1	336
NM_006929	-0.0659349	0.52106141	-0.562563109	0.000179644	8066	1	6
NM_006929	-0.0598256	0.386085	-0.562563109	0.002287066	8063	1	36
NM_144976	-2.6206688	0.26121175	-0.250359925	0.030637601	30	1	2189
NM_003301	0.10593126	0.15108801	-0.26907234	0.015684411	7889	1	878
NM_133452	0.37126565	0.78873522	-0.401970588	0.003609542	8471	1	807
NM_005575	0.29181317	0.88291265	-0.222936978	0.013597581	8484	1	1129
NM_003683	-0.6638595	0.21989312	-0.712132858	0.00644778	7371	1	1459
NM_003683	-0.6102898	0.38761736	-0.712132858	0.001958561	7377	1	965
NM_032221	-0.4405186	0.56412348	-0.468626342	0.004497273	8031	1	908

XR_013014	0.06494301	0.4311218	-0.523800083	0.000206099	8065	1	17
XR_013014	-0.0268039	0.4562665	-0.523800083	0.000847795	8064	1	10
XR_014612	-1.4029854	0.08553291	-0.373188036	0.027866507	6568	1	2091
NM_006337	0.05087405	0.76394565	-0.633572296	0.003022592	8282	1	128
NM_006337	-0.0130037	0.70573283	-0.633572296	0.001692221	8278	1	320
NM_000407	-0.0697427	0.81917908	-0.62231551	0.021164856	8215	1	831
NM_022751	0.06868726	0.97384643	-0.666324484	0.005658926	8369	1	876
NM_004328	-0.4766239	1.19692601	-0.190266836	0.030049588	9435	1	1884
NM_198046	0.06806718	0.21547087	-0.425425344	0.004555066	7804	1	505
NM_198046	-0.0785054	0.56808814	-0.425425344	0.0001799	7487	1	18
NM_152599	0.73248965	0.28072315	-0.515989855	0.003492512	8907	1	2080
NM_152599	-1.8426169	0.34534223	-0.515989855	0.005406656	11	1	2191
NM_000366	0.60569651	0.926366	-0.580842516	0.003101185	8823	1	1508
NM_181684	0.99126059	0.07238876	-0.442640823	0.000411944	9634	1	2098
NM_181684	-0.403109	0.34086035	-0.442640823	0.012421683	7797	1	370
CN646799	0.06683083	1.06400685	-0.430991439	0.002254029	8199	1	522
NM_006090	0.31158947	0.09615139	-0.761046629	0.015512045	7695	1	1097
NM_182640	0.32296019	0.97755338	-0.400034381	9.36E-05	8554	1	577
NM_182640	0.51605663	1.07170641	-0.400034381	0.00018355	8572	1	1271
NM_018453	0.61058073	0.21331516	-0.456348587	0.02352826	8736	1	1634
NM_018453	0.72874259	0.58143947	-0.456348587	0.039102301	8763	1	1799
XR_012294	0.52209194	0.4650791	-0.489489646	0.043830619	8730	1	1406
NM_024537	-0.3420724	1.03040253	-0.672190176	0.000869364	8233	1	628
NM_024537	-0.372834	0.93912446	-0.672190176	0.000264674	8231	1	363
NM_013994	0.13518142	0.8254732	-0.393920544	0.001229135	8102	1	149
NM_013994	0.23410734	0.87860264	-0.393920544	0.009748205	8419	1	502
NM_007320	-0.1320557	0.17883573	-0.25925261	0.021518701	7813	1	794
NM_000866	-0.4089232	0.09200504	-0.33194926	0.027417074	7785	1	1423
NM_003617	0.17861149	0.83577435	-0.270669323	0.000855851	8467	1	384
NM_033301	0.08199953	0.59048395	-0.605959692	0.00172443	8077	1	12
NM_033301	-0.0635289	0.66894922	-0.605959692	0.001218332	8076	1	4
NM_003135	-0.1469388	0.31946561	-0.323592665	0.007352881	7824	1	314
NM_014585	-0.0593443	0.70305901	-0.604946805	0.00928509	8108	1	132
NM_181659	0.23227589	0.53160696	-0.550286268	0.002997306	8084	1	78
NM_181659	0.14468093	0.83084987	-0.550286268	0.024184219	8415	1	665
NM_006510	0.07552341	0.48010801	-0.488878845	0.001076881	8081	1	50
NM_006510	0.15945017	0.41714618	-0.488878845	0.000304044	8082	1	66
CN643352	0.54595998	-0.2090299	-0.412098769	0.04974722	8910	1	1919
CN643352	0.56485863	0.04130704	-0.412098769	0.022247502	8911	1	1621
NM_022068	-0.2704218	0.58424529	-0.597579535	0.00285207	8030	1	232
NM_022068	-0.0342525	0.42284151	-0.597579535	0.031218921	7638	1	350
NM_001009997	-0.0399441	0.3290605	-0.284408768	0.009242215	8375	1	1397
NM_002905	-0.2156146	1.08688928	-0.513985347	0.000242257	8239	1	560

NM_052851	0.02590691	0.91153369	-0.574942222	0.00535023	8339	1	315
CN648203	0.08732323	0.12838636	-0.349859156	0.002077994	7819	1	818
CN648203	0.13329406	0.1351664	-0.349859156	0.020975804	7882	1	771
NM_006494	0.85182084	0.52309431	-0.905815603	0.019752877	8742	1	2012
NM_138697	-0.004872	0.33766719	-1.026190871	0.011461879	6503	1	983
NM_138697	0.49848063	0.40502907	-1.026190871	0.049633158	7322	1	1936
XR_011234	0.06850921	0.66293173	-0.639313496	0.022648392	7768	1	299
NM_003609	-0.4502752	0.86052017	-0.251897882	0.009686963	7468	1	1069
NM_014003	-0.0173116	1.0692366	-0.484828576	0.001069935	8198	1	429
NM_014003	0.04851137	1.16243311	-0.484828576	0.000448034	8200	1	629
NM_005031	-0.5930514	1.20410085	-0.144008016	0.014130453	9446	1	1955
NM_000975	0.41221863	0.78418841	-0.535214282	0.002918808	8421	1	490
NM_000975	0.31706651	0.78117188	-0.535214282	0.004272126	8420	1	296
NM_003312	0.15399274	0.58055551	-0.303105216	0.000972027	7983	1	183
NM_003312	-0.0500952	0.52821726	-0.303105216	0.009490556	7851	1	151
NM_003331	-0.4784841	-0.0247339	-0.303770123	0.033843915	7769	1	1596
NM_030803	-0.0405586	0.621603	-0.546404538	0.004969972	8078	1	21
NM_030803	-0.8039053	0.70092011	-0.546404538	0.017741046	6553	1	2099
NM_152493	0.25417876	0.39252192	-0.693375079	0.020601166	7714	1	481
NM_003985	-0.0419416	1.1473742	-0.456098352	0.008914296	8249	1	1272
NM_199350	0.28586484	0.84882675	-0.460913093	0.005242366	8470	1	796
NM_006666	0.42688467	0.68006798	-0.264534464	0.002873655	8493	1	836
NM_006666	0.52017771	0.77620414	-0.264534464	0.001186819	8494	1	1141
NM_003639	-0.6172034	0.69987747	-0.393357467	0.009823201	7461	1	1142
NM_003639	-0.6848996	0.44522728	-0.393357467	0.002536583	7395	1	1200
XM_371470	0.26774384	0.75004461	-0.470698079	0.014692727	8417	1	436
XM_371470	0.20350712	0.75074275	-0.470698079	0.021021299	8416	1	394
NM_015832	0.66397528	0.49031032	-0.448844366	0.022913827	8814	1	1565
NM_015832	0.66238967	0.71282483	-0.448844366	0.015561413	8455	1	1609
A_01_P002494	-0.1516891	0.80018993	-0.478455689	0.003067766	8367	1	202
A_01_P002494	-0.0718702	0.64929146	-0.478455689	0.013059447	8079	1	100
NM_005922	0.30502181	1.13627229	-0.237424754	0.00102464	8568	1	1298
NM_022894	0.11643738	0.37499152	-0.638444047	0.036228218	8714	1	734
NM_022894	-0.0091054	0.56328214	-0.638444047	0.005992181	8280	1	97
NM_014680	0.66088275	0.51837683	-0.413250853	0.001540844	8441	1	1296
NM_014680	0.63814043	0.59611141	-0.413250853	0.002875647	8442	1	1179
NM_016334	0.36576341	0.93629248	0.048337624	0.040206859	8614	1	1745
NM_174887	0.32297771	0.46219079	-0.312692519	0.004205835	7941	1	439
NM_174887	0.12537423	0.50854624	-0.312692519	0.025423021	7927	1	274
NM_016492	0.230667	0.87495153	-0.517198306	0.012035821	8973	1	1155
NM_016492	0.51950896	1.07478241	-0.517198306	0.001484183	9324	1	1325
NM_018121	-0.0102343	0.48167361	-0.145498747	0.00874245	8376	1	893
NM_018121	-0.0933505	0.55124544	-0.145498747	0.024510449	7909	1	605

NM_173050	-0.2356515	0.27530598	-0.832849984	0.035973858	7526	1	1113
NM_006283	-0.1093467	0.79588522	-0.151809451	0.004093715	9493	1	483
NM_006283	-0.2512329	0.92918132	-0.151809451	0.002263293	9492	1	790
XM_371279	-0.0787038	-0.0500817	-0.464572648	0.005131392	7776	1	1516
XM_371279	-0.0793592	0.26818287	-0.464572648	0.001676999	7594	1	146
NM_025209	0.16881211	0.30478975	-1.16422157	0.025782592	6504	1	1744
NM_018174	0.71731495	0.63415391	-0.553754184	0.048672959	8762	1	1866
NM_018174	0.70160988	0.63383073	-0.553754184	0.038323474	8761	1	1810
NM_022041	-0.0280645	0.405232	-0.509877295	0.012675312	7480	1	260
NM_021126	0.35244712	0.12161489	-0.289426529	0.025958264	7899	1	1232
NM_181461	0.11151868	-0.082526	-0.448051281	0.024217207	7864	1	1360
NM_005077	0.01402266	0.95031248	-0.569308659	0.004681584	7347	1	1392
NM_005077	0.38743561	0.91002428	-0.569308659	0.006947965	8425	1	933
XR_013196	-0.4104529	0.81223958	-0.318847823	0.001949236	7463	1	622
XR_013196	-0.3437757	0.75802207	-0.318847823	0.00010772	7449	1	248
NM_015242	-0.1464671	0.31368675	-0.423614427	0.021500128	7822	1	387
NM_015242	-0.1065683	0.35026326	-0.423614427	0.00329488	7823	1	137
NM_014886	-0.2333033	0.90212665	-0.517285005	0.027358767	8182	1	863
NM_014886	-0.2782415	1.05822185	-0.517285005	0.027588281	8183	1	1365
NM_018170	0.23716955	1.14820339	-0.364533849	0.00147889	9354	1	1083
NM_015928	-0.0292918	0.5452243	-0.41533205	0.006874885	7908	1	244
NM_018036	-0.0340001	0.8992299	-0.92491031	0.004789465	8013	1	1040
NM_032409	0.0445062	0.36498178	-0.353727897	0.002867503	7835	1	176
NM_032409	-0.0391573	0.39322568	-0.353727897	0.000864469	7834	1	120
NM_000985	-0.1602117	0.09178673	-0.333912146	0.034764727	7810	1	814
NM_000985	0.00653115	0.54598252	-0.333912146	0.004873368	7852	1	67
NM_000985	0.61492781	0.58420049	-0.333912146	0.003017628	8443	1	1184
NM_000985	0.55702322	0.70229727	-0.333912146	0.007709691	8444	1	1139
NM_020533	-0.0689791	0.12032088	-0.553324643	0.032930127	7625	1	563
NM_020533	-0.1784872	0.27212962	-0.553324643	0.032827829	7616	1	347
NM_198935	0.46715256	0.83421652	-0.614583921	0.00433353	8452	1	1122
NM_198935	0.87754858	0.89812909	-0.614583921	0.015619714	8796	1	1958
NM_015061	-0.8118982	0.75100511	-0.297141287	0.023657577	6593	1	1753
NM_001003	0.2205897	0.17315628	-0.909093634	0.046413405	7743	1	1312
NM_003373	-0.3491039	0.92432729	-0.282468186	0.02430429	6605	1	1074
NM_080836	-0.3574828	0.10503015	-0.435793388	0.032455865	7796	1	1164
NM_020789	-0.8762642	0.82481308	-1.059098929	0.029691489	6672	1	2075
NM_013442	0.28331443	0.13086557	-0.219376578	0.0172315	7900	1	1169
NM_013433	0.20040993	0.04610239	-0.573088597	0.007035663	7694	1	947
NM_013433	-0.0211329	0.20731536	-0.573088597	0.010907234	7617	1	289
NM_144966	-0.274052	-0.0445768	-0.638323351	0.047304623	7561	1	1322
XM_290517	0.01025161	0.01454863	-0.609030492	0.007329165	7869	1	803
XM_290517	-0.2052915	0.27644249	-0.609030492	0.0045796	7589	1	216

NM_000294	-0.2898949	0.58226994	-0.472093662	0.001513683	7440	1	81
NM_000294	-0.2725343	0.69445642	-0.472093662	0.000296837	7441	1	53
NM_005283	0.70635485	0.21118813	-1.205616821	0.035667512	10850	1	2135
NM_138815	-0.7114328	0.4768116	-0.40144827	0.001395353	7390	1	1335
CN644139	-0.2417271	0.68050785	-0.814253084	0.012333641	6858	1	735
CN644139	-0.4338828	0.75132671	-0.814253084	0.006922971	6857	1	894
CN644139	-0.6893638	0.59098211	-0.814253084	0.004990457	7378	1	1390
CN644139	-0.3991275	0.66804444	-0.814253084	0.004490162	6814	1	496
NM_005883	1.24083643	0.87701443	-0.074848515	0.042941652	9717	1	2147
NM_006098	0.38413561	1.08437241	-0.704387084	0.003886636	9312	1	1181
NM_006098	0.36524329	1.16104731	-0.704387084	0.003776916	9313	1	1354
NM_005800	0.23120389	0.82994877	-0.323547656	0.049855199	8428	1	1111
XR_012112	0.21318708	-0.1812736	-0.397706905	0.014787111	7862	1	1627
XR_012112	0.1793495	-0.1294859	-0.397706905	0.004490486	7863	1	1484
NM_152455	-0.266349	0.93213656	-0.096090206	0.021654911	9482	1	1218
NM_002718	0.02710622	0.76670234	-0.290014667	0.003258102	8462	1	398
NM_002718	0.22401875	0.54027127	-0.290014667	0.009930265	7931	1	382
NM_153486	-0.2208495	0.79732536	-0.373361541	0.027331556	7467	1	948
NM_021226	0.14842402	0.18179359	-0.383990812	0.027999563	7904	1	1131
NM_032569	-0.3206784	-0.0494741	-0.318249771	0.024053127	7773	1	1311
NM_018119	-0.4708845	1.12400558	-0.816326687	0.025148437	6680	1	1897
NM_031497	-0.0831777	0.63368298	-0.534796396	0.010019285	6024	1	2063
NM_198484	-0.121891	1.12851279	-0.321938791	0.022611468	8975	1	1743
NM_173479	1.48605369	0.34617753	-0.544047482	0.02009167	9641	1	2159
NM_032302	0.35762329	0.456578	-0.324787754	0.03733086	8731	1	957
NM_032302	0.51280894	0.58588491	-0.324787754	0.025172228	8732	1	1249
NM_002313	0.13327057	0.85567623	-0.469354304	0.000858357	8459	1	268
NM_002313	0.44198581	0.76466834	-0.469354304	0.009028215	8432	1	835
CB309129	0.18424857	0.96129112	-0.369897346	4.25E-05	8555	1	301
CB309129	0.04556201	0.92267949	-0.369897346	0.017969759	8186	1	583
NM_001202	-0.3468595	0.48068551	-0.448618048	0.001202175	7416	1	180
NM_145294	-0.4230888	0.95833691	-0.358902494	0.002618597	7472	1	776
NM_145294	-0.421209	1.02559244	-0.358902494	0.000304326	7474	1	1035
NM_015275	0.26172719	0.73542974	-0.391264646	0.012702867	8418	1	392
NM_015275	0.31016192	0.90295294	-0.391264646	0.034155967	8431	1	1276
NM_145714	-0.688988	0.41093341	-0.27344792	0.005787026	7398	1	1386
NM_145714	-0.6040955	0.40515845	-0.27344792	0.002792721	7399	1	1099
NM_145714	-0.7380165	0.1659389	-0.27344792	0.041563049	7380	1	1707
NM_145714	-0.5783418	0.35214887	-0.27344792	0.001393177	7397	1	1041
NM_145714	-0.6697488	0.41006189	-0.27344792	0.002237026	7396	1	1221
NM_005015	-0.1431677	1.03586285	-0.223833462	0.000313543	9066	1	816
NM_005015	-0.0374714	1.11245452	-0.223833462	0.000409156	9069	1	987
NM_003744	0.07517496	0.10802395	-0.225283082	0.034974383	7888	1	992

NM_025264	0.10411809	0.67282336	-0.534851824	0.000211991	8279	1	69
NM_025264	0.14960337	0.70151217	-0.534851824	0.000251165	8045	1	838
NM_003730	-0.0889041	0.73901915	-0.304638885	0.018088736	8137	1	261
NM_003730	-0.226849	0.88528234	-0.304638885	0.020024683	8188	1	660
NM_003161	-0.0655405	0.92130359	-0.28925114	0.031574787	8507	1	1039
NM_080662	0.05579806	0.28282992	-0.653863869	0.019907304	7632	1	328
NM_152756	-0.9377482	0.28201719	-0.696749107	0.032429253	6562	1	2022
NM_152756	-1.2701812	0.36445944	-0.696749107	0.029003618	6546	1	2123
NM_014424	0.2728969	0.93961342	-0.272968799	0.036629968	8481	1	1614
NM_053023	0.47297919	0.79864869	-0.294306828	0.009634973	8439	1	1075
NM_053023	0.01505685	0.91346731	-0.294306828	0.003888467	8128	1	459
NM_015601	-0.5112065	0.56151262	-0.742809324	0.036347007	8020	1	1238
NM_173485	-0.3223362	0.06729121	-0.582269263	0.014770882	7774	1	1308
CN646216	0.19742324	0.88633621	-0.424403305	0.02750792	8854	1	944
NM_024023	1.26217389	0.12760111	-0.302928867	0.036485692	10866	1	2173
NM_145043	0.06329612	0.47894512	-0.31423125	0.048854373	7926	1	357
NM_145043	0.18624328	0.52039736	-0.31423125	0.041138875	7913	1	442
NM_006893	0.27821823	0.07740978	-0.467883855	0.043111152	8906	1	1764
NM_001765	-0.1431379	0.1707157	-0.68189058	0.020531036	7563	1	618
NM_004750	-0.2587738	0.87208568	-1.021470941	0.006580453	6859	1	1246
NM_004750	-0.5248679	0.99277643	-1.021470941	0.018765283	6674	1	1886
NM_139315	-0.2318899	0.16593776	-0.562082901	0.043163699	7614	1	652
NM_139315	-0.1868441	0.34408004	-0.562082901	0.006885046	7618	1	119
NM_004695	-0.5679044	0.97010282	-0.280202196	0.000616383	7470	1	1201
NM_015635	-0.0415644	0.18231585	-0.547234664	0.005563687	7782	1	672
NM_033285	0.76593179	0.72728491	-0.099096577	0.036359226	8586	1	1906
NM_024319	-0.3092271	1.12322426	-0.460597611	0.000259175	8238	1	793
NM_024319	-0.4020783	1.06086771	-0.460597611	6.96E-05	8237	1	857
NM_182800	-0.3765588	0.29976413	-0.383938829	0.02041269	7409	1	706
NM_182800	-0.4881658	0.39920526	-0.383938829	0.020549378	7407	1	938
NM_182800	-0.6558286	0.39000131	-0.383938829	0.006074721	7387	1	1259
NM_000363	0.17061297	1.10602267	-0.66130266	0.039433786	8896	1	1974
CO579435	0.26625935	0.4731481	-0.419818763	0.000122842	8087	1	144
CO579435	0.23231821	0.54398784	-0.419818763	0.002272734	8088	1	99
NM_006401	0.07151327	0.28312082	-0.479838846	0.039306051	7631	1	323
NM_006401	0.20697041	0.6493796	-0.479838846	0.006844201	8085	1	121
NM_152866	0.63612072	0.18301661	-0.250983806	0.009244199	9633	1	2002
NM_025207	0.34146956	0.31190845	-0.209848188	0.04639964	7939	1	1014
NM_025207	0.22742977	0.37061727	-0.209848188	0.043073743	7940	1	711
NM_000455	-0.4059813	0.30456444	-0.685650232	0.010105847	6548	1	1240
NM_000455	0.13081075	0.36603785	-0.685650232	0.017071011	8037	1	782
NM_032134	0.30327995	0.59956114	-0.605065017	0.042538327	8450	1	855
NM_003137	0.52046944	0.64394836	-0.239812413	0.001562968	8489	1	1153

NM_003137	-0.0910147	0.67676073	-0.239812413	0.016971062	8138	1	302
NM_012306	0.63082497	0.18233996	-0.380146654	0.004199753	9632	1	1971
NM_004732	0.16100037	0.96063011	-0.482221976	0.005337217	8460	1	686
NM_022827	-0.5770574	0.76129943	0.038603325	0.023486976	6584	1	1755
NM_003637	-1.0059155	0.71757892	-0.241007545	0.046814168	6577	1	2116
NM_012317	-0.0114342	0.97200063	-0.430359385	0.001171179	8196	1	267
NM_012317	0.04285208	0.87484349	-0.430359385	0.008984219	8187	1	286
A_01_P015997	0.57129027	0.3558278	-0.460856249	0.017321351	8737	1	1400
NM_033046	-0.1679305	0.63753205	-0.335683816	0.00678742	8129	1	113
NM_033046	0.2140203	1.17001575	-0.335683816	0.001057025	9356	1	1242
NM_005137	-0.0330787	0.2029032	-0.31152434	0.001825742	7886	1	460
NM_005137	-0.0964864	0.45102954	-0.31152434	0.003888869	7850	1	94
NM_004064	0.34062513	1.12206547	-0.138414419	0.001215121	8577	1	1520
NM_004064	0.21962729	1.15116557	-0.138414419	0.00351973	8575	1	1422
XR_009800	-2.6838166	0.13563763	-0.8704546	0.025267984	10	1	2188
NM_017904	0.06005744	0.1645287	-0.228388869	0.045667104	7827	1	867
XR_013802	-0.1823488	0.89682346	-0.605609416	0.001049125	8232	1	135
XR_013802	0.09548346	0.93607064	-0.605609416	0.001068474	8343	1	213
NM_015134	0.31934332	0.77450674	-0.248965226	0.001671431	8473	1	666
NM_015134	0.25125665	0.54655267	-0.248965226	0.003813588	7944	1	400
NM_032890	-0.8596491	0.70413729	-0.596476837	0.024965511	6589	1	1879
NM_000159	-0.2727268	0.85991905	-0.655810451	0.002544091	8230	1	414
NM_000983	0.10694091	0.42346624	-0.789418665	0.016923718	8036	1	472
NM_032036	0.36443838	0.20555924	-0.304843377	0.033006208	7936	1	1017
NM_032036	0.44401941	0.41674398	-0.304843377	0.00539233	7942	1	777
NM_006350	1.01822458	1.01556589	-0.21993494	0.00065769	9713	1	2111
NM_000387	0.04216751	0.4554125	-0.237829401	0.001495625	7915	1	345
NM_000387	0.15368748	0.34580191	-0.237829401	0.006534982	7911	1	619
NM_001306	0.01398817	0.46202732	-0.243248197	0.00277522	7494	1	293
NM_001306	0.15272083	0.71301471	-0.243248197	0.000787924	7987	1	317
NM_001535	-0.0966505	0.38529107	-0.343931008	0.002043419	7847	1	150
NM_001535	-0.1621505	0.54442602	-0.343931008	0.003662361	7849	1	76
NM_001005273	0.07046094	0.36888765	-0.653557761	0.004705685	7635	1	114
NM_001005273	0.02180629	0.33353293	-0.653557761	0.007326333	7634	1	112
NM_024589	-0.3493801	0.51052977	-0.319863923	0.001203383	7432	1	257
NM_024589	-0.2401955	0.6437223	-0.319863923	5.06E-05	7445	1	77
NM_012271	0.06093529	0.51277986	-0.325755981	0.000193151	7952	1	194
NM_025133	-0.1425302	0.66929974	-0.260624519	0.001325111	7446	1	126
NM_025133	-0.0399214	0.68578599	-0.260624519	0.007264559	8125	1	266
NM_031370	0.44605239	1.08622125	-0.300222841	0.001014884	8574	1	1321
NM_031370	0.3133648	1.10658552	-0.300222841	0.001025454	8567	1	1076
XR_010916	0.48094437	0.62331961	-1.010322141	0.02316959	8740	1	1773
NM_144720	0.4786073	0.63722704	-0.393232771	0.013526713	9707	1	1991

NM_017948	-0.2927679	0.55505915	-0.356564848	0.002838175	7426	1	313
NM_017948	-0.0757366	0.79165922	-0.356564848	0.005399244	8130	1	157
NM_003126	0.5204459	0.69436485	-0.242234905	0.010925799	8495	1	1264
NM_003126	-0.1778773	0.95899252	-0.242234905	0.020098423	8390	1	1316
NM_002486	0.02530412	0.36696848	-0.474307792	0.035234526	8046	1	415
NM_002486	0.01695126	0.72741961	-0.474307792	0.006839214	8109	1	142
NM_006810	-0.3786654	0.41607008	-0.276936911	5.48E-05	7430	1	422
NM_006810	-0.1071328	0.42037881	-0.276936911	0.012955369	7845	1	218
NM_153233	0.19203283	0.25231752	-0.395487168	0.01362678	7877	1	671
XM_048462	0.27826423	0.57480469	-0.606056534	0.019510008	8723	1	935
XM_048462	-0.0883384	0.99175733	-0.606056534	0.006511932	8217	1	877
NM_020312	0.37724394	0.75496455	-0.348200281	0.004103579	8483	1	604
NM_020312	0.31586503	0.89164375	-0.348200281	0.001112786	8557	1	539
NM_016390	0.07904236	0.6620262	-0.300878622	0.002470696	8131	1	131
NM_016390	0.10089351	1.06535034	-0.300878622	0.000130247	8556	1	571
NM_014663	0.21538431	0.6870439	-0.513370052	0.020174516	8451	1	694
NM_024698	3.13772722	-0.0107882	-0.512559713	0.026293789	10872	1	2179
NM_024293	-0.2167262	0.24858096	-0.329915659	0.021905112	7842	1	438
NM_024293	-0.2390593	0.26166722	-0.329915659	0.046918884	7841	1	523
NM_024855	0.15782698	1.04300051	-0.387206336	0.004128881	8197	1	664
NM_006709	0.39338331	0.8729188	-0.304343912	0.005034953	8472	1	1070
NM_003779	0.09655817	0.62547045	-0.580498661	0.002731839	8281	1	43
NM_003779	-0.217626	0.4669193	-0.580498661	0.003834565	7619	1	48
NM_153273	0.23404999	0.11677912	-0.275926038	0.032836805	7898	1	1081
NM_003881	0.16575751	1.11948899	-0.317031113	1.32E-05	8514	1	1096
NM_016339	-0.7980399	0.52433266	-0.589843718	0.017912232	6563	1	1957
CN647878	-0.838942	0.81251434	-0.521992743	0.007101279	6594	1	1698
CN647878	-0.0343687	1.15674524	-0.521992743	0.003181365	8195	1	928
NM_005822	0.1831641	0.98575855	-0.717128786	0.006141345	8202	1	695
NM_001009921	0.08014008	0.31939196	-0.202589284	0.025636505	7921	1	555
NM_001009921	0.13852579	0.50066468	-0.202589284	0.020494299	7929	1	465
NM_198317	-0.0666284	0.3731541	-0.37556236	0.001296007	7783	1	491
NM_139208	0.65329851	0.69571999	-0.68670197	0.024101433	8754	1	1716
NM_014824	0.16505586	0.09993634	-0.309246068	0.018209481	7884	1	998
NM_014824	0.68454063	0.21050714	-0.309246068	0.040343784	8912	1	1700
DR772617	-0.8107725	0.91174473	-0.406505353	0.020323135	6596	1	1777
DR772617	-0.6616725	0.8904381	-0.406505353	0.048248362	6597	1	1749
NM_004586	0.25990461	0.88528569	-0.341615887	0.033128873	8430	1	1149
NM_001008712	-0.4230218	0.73184202	-0.385125963	0.000855845	7448	1	356
NM_001008712	-0.4334788	1.01241109	-0.385125963	0.000374433	7473	1	740
NM_152511	0.49751084	0.69779192	-0.46797324	0.001415722	8440	1	813
NM_001010886	0.98891403	0.51290099	-0.631047738	0.038615409	8755	1	2045
NM_000238	-0.0626602	0.43430607	-0.548142974	0.04434366	7478	1	553

NM_015556	-0.0125887	1.19585241	-0.211985147	0.003876725	9071	1	1418
NM_015556	0.02917722	0.84784204	-0.211985147	0.000349764	8466	1	403
NM_078487	-0.1056402	0.83703077	-0.16635191	0.011198895	9486	1	636
NM_181523	0.39757945	0.42234496	-0.209409448	0.009467739	7943	1	886
XR_012301	-0.0940097	0.32044878	-0.418650743	0.021291039	7479	1	340
XR_012301	-0.0065174	0.1061709	-0.418650743	0.009525854	7870	1	647
NM_004593	0.43819343	0.48800962	-0.271175538	0.001601463	8488	1	841
NM_015513	-0.0327887	0.78966663	-0.289791208	0.002813218	9064	1	312
NM_015513	0.09390219	1.03415688	-0.289791208	0.003386316	9076	1	859
NM_005663	0.02112162	0.66696363	-0.575084956	0.006409385	8044	1	412
NM_005663	0.05982112	1.00654419	-0.575084956	0.001439555	8342	1	499
NM_005484	0.4570474	0.91739533	0.102686065	0.020627873	8615	1	1836
NM_173832	0.10671722	0.32103071	-0.46240758	0.01310631	7858	1	236
NM_004858	0.25078543	0.09543485	-0.400055625	0.028740382	7872	1	797
XM_378181	-0.0629994	0.58006046	-0.707266215	0.020576075	8110	1	258
NM_002316	0.8592605	0.36495278	-0.515225674	0.018190967	9639	1	1953
NM_002316	0.3708888	0.64189	-0.515225674	0.007154137	8385	1	895
CN802772	-0.4538616	0.686502	-0.19141511	0.000651367	7451	1	685
CN802772	-0.2726529	0.66048129	-0.19141511	0.013663475	8122	1	602
CN802772	-0.224816	1.16884694	-0.19141511	0.005607916	9101	1	1404
CN802772	-0.2850541	0.73093349	-0.19141511	0.026897159	8181	1	785
NM_181602	1.1590067	0.59299837	-0.668821029	0.011906709	9640	1	2109
NM_004990	-0.2322205	0.70651762	-0.461122346	0.008361875	8106	1	154
NM_002501	0.12252955	0.97131407	-0.294973291	0.011336045	8858	1	937
NM_021638	0.2037951	0.60061028	-0.069064351	0.00723691	7982	1	840
NM_021638	-0.1565802	0.74868145	-0.069064351	0.003066376	7970	1	784
NM_014836	0.60289965	1.14930942	-0.292162841	0.003450858	9327	1	1803
NM_032332	-0.3819773	0.38926347	-0.279896075	0.003213315	7427	1	547
XR_013549	1.63640321	0.47814748	-0.676442459	0.008830825	9644	1	2170
XR_013549	1.49625021	0.78368111	-0.676442459	0.003889261	9645	1	2161
NM_003169	-0.1738893	0.19020039	-0.315514434	0.003389208	7820	1	537
NM_003169	-0.1775591	0.28496216	-0.315514434	0.003996761	7821	1	378
NM_004922	0.12652054	-0.0595024	-0.303678393	0.04636562	7865	1	1442
NM_004922	0.20258832	0.06218801	-0.303678393	0.007841293	7896	1	1086
NM_138371	0.50229268	0.31235595	-0.565437266	0.030107855	8734	1	1313
NM_021253	0.11356918	0.20104265	-0.537668029	0.023513575	7630	1	493
NM_012189	0.74558117	0.96307742	-0.129249593	0.025485327	9708	1	2000
NM_005324	-0.0061488	0.91919788	-0.328037651	0.03049746	8185	1	829
NM_015677	-1.6130572	0.4616822	-0.609861457	0.008279974	6010	1	2166
XM_058581	-0.1854971	0.25117328	-0.321119351	0.002865866	7816	1	520
XM_058581	-0.276533	0.29840095	-0.321119351	0.005832381	7779	1	828
NM_002296	0.5560263	0.85494095	-0.133487556	0.003127773	8584	1	1501
NM_002296	0.66875506	0.7755083	-0.133487556	0.033019529	8585	1	1775

NM_033082	0.8269414	0.76011643	-0.35885529	0.000373435	8449	1	1737
NM_033082	0.74090853	0.72992624	-0.35885529	0.003030066	8448	1	1591
XR_013742	0.01833988	0.9880042	-0.56456466	0.005132651	8341	1	476
XR_013742	-0.0597802	0.86178124	-0.56456466	0.021081278	8368	1	585
NM_000191	0.09760146	0.17976594	-0.190747382	0.018770252	7887	1	858
NM_002650	-0.551731	0.91265983	-0.248840778	3.82E-05	7469	1	1063
NM_002650	-0.3374602	1.21685733	-0.248840778	9.51E-06	9409	1	1303
NM_032924	0.32649782	0.90194692	-0.610615797	0.009026249	8782	1	1260
NM_080284	0.33862365	0.74475799	-0.232821201	0.012559387	8454	1	1270
NM_018671	-0.0952786	0.35553945	-0.24155625	0.009966705	7828	1	417
NM_018671	-0.1208507	0.14426431	-0.24155625	0.010258788	7811	1	799
NM_016938	-0.8037068	0.38495889	-0.605408898	0.017241559	7375	1	1652
NM_016938	-0.6761683	0.42294687	-0.605408898	0.00556276	7376	1	1132
XR_010415	0.44114095	0.85934151	0.066558833	0.036985393	8498	1	1783
XR_010415	0.22862419	1.15534573	0.066558833	0.006881869	8578	1	1768
NM_144600	-0.1774028	0.84443579	0.047070745	0.028385182	9484	1	1527
NM_025265	-0.0869014	1.18193197	-0.811987264	0.025212698	6681	1	1854
NM_145290	-0.6335287	0.41617276	-0.597228231	0.023408134	6569	1	2046
NM_145290	0.20390553	1.15356143	-0.597228231	0.001602321	9303	1	1157
NM_145649	-0.3997192	1.04989972	-0.06947668	0.015095656	9428	1	1674
NM_130901	0.88431746	0.12118535	-0.462991884	0.038409383	10852	1	2126
NM_005886	0.12425898	0.57137082	-0.302056081	0.005683324	7953	1	395
NM_005886	-0.3969217	0.58491157	-0.302056081	0.012381488	7499	1	1016
NM_058241	-0.3258902	0.65955529	-0.383386382	0.03721592	6600	1	833
NM_174856	-0.280076	0.70620407	-0.266075246	0.017794738	7466	1	715
NM_174856	-0.3523438	0.8344289	-0.266075246	0.009433819	7464	1	809
NM_198227	0.13935146	0.32970494	-0.413198179	0.028745839	7878	1	473
NM_152557	0.19612864	0.88121522	-0.183883942	0.000447325	7988	1	663
NM_152557	-0.012274	1.10676146	-0.183883942	0.000280181	9073	1	901
NM_138432	0.05738194	0.6034479	-0.461967561	0.021068395	7481	1	526
NM_138432	0.1440973	0.81523347	-0.461967561	0.008343917	8469	1	468
NM_004970	0.79469967	0.56914332	-0.726359707	0.023910738	8753	1	1878
NM_017865	-0.2264502	0.54585859	-0.294430367	0.003914468	7484	1	262
NM_017865	-0.5276192	0.68563302	-0.294430367	0.004766976	7425	1	1182
NM_024643	0.51097816	0.58253412	-0.349297375	0.000798188	8382	1	1806
NM_170722	-0.1220031	0.60415231	-0.345063394	0.010637415	7465	1	379
NM_170722	-0.1280013	0.8809201	-0.345063394	0.000581651	9061	1	207
NM_015191	0.36579926	0.57583972	-0.316396707	0.010081817	7955	1	820
NM_023928	0.54713964	0.96654993	-0.16924259	0.031155543	9677	1	1861
NM_014260	0.22660833	0.12068017	-0.340733585	0.021482114	7885	1	866
NM_002669	-0.3734681	0.3401904	-0.209708069	0.000460649	7428	1	724
NM_002669	-0.2765713	0.5374669	-0.209708069	0.005762884	7436	1	402
NM_014592	0.28995428	0.92202129	-0.197212408	0.025151808	8388	1	1855

NM_020207	-0.695644	1.05482722	-0.095847037	0.034086954	9434	1	1925
NM_005861	0.23816719	0.64568552	-0.258249957	0.00102483	7985	1	337
NM_005861	0.29590546	0.59502402	-0.258249957	0.000498444	7986	1	385
NM_001008697	0.32323686	0.26286357	-0.161221156	0.024803134	7938	1	1052
NM_012286	-0.018504	0.51770398	-0.265796207	0.034175294	8135	1	355
NM_000048	-0.2213246	1.18133691	0.14685609	0.035636351	9513	1	1973
XR_013144	-0.36918	0.09362164	-0.228448963	0.000606327	7771	1	1174
NM_203473	-0.2869131	0.03361312	-0.213201181	0.03994811	7772	1	1405
NM_032124	-0.3986932	0.82630806	-0.380198118	0.024883532	6603	1	923
NM_017765	0.57706508	0.76489618	-0.547982882	0.009153045	8806	1	1355
NM_000535	-0.0433515	0.99006026	-0.086949967	0.002743951	9072	1	963
NM_014885	-0.1723737	0.33203344	-0.359479594	0.026240149	7795	1	613
NM_000712	-0.1010104	0.38609044	-0.085022784	0.040134473	7919	1	747
NM_000712	0.09916411	0.55917967	-0.085022784	0.011230988	7932	1	631
NM_007355	0.05639496	0.36220611	-0.235108224	0.009890841	7923	1	329
NM_007355	0.11725735	0.31998939	-0.235108224	0.037010261	7924	1	495
NM_018237	-0.2298215	0.68999103	-0.698979936	0.029458069	8025	1	646
XR_012617	-0.4808986	0.47007314	-0.469006302	0.011655261	7406	1	606
NM_024897	0.1804633	0.88577515	-0.012574961	0.04965564	8477	1	1572
NM_015959	0.37857924	0.34349227	-0.180114985	0.010023591	7947	1	1173
NM_015959	0.11499125	0.40579086	-0.180114985	0.006360335	7977	1	561
NM_018044	-0.4744111	0.67610269	-0.150122474	0.004498548	7460	1	991
NM_018044	-0.490176	0.55632897	-0.150122474	0.002042156	7429	1	942
NM_018044	-0.8389137	1.02096983	-0.150122474	0.022417255	9424	1	2007
NM_001003684	0.42289575	0.63453483	-0.736474505	0.010909923	9636	1	1774
NM_020216	-0.2549762	0.5987315	-0.280871517	0.019551833	7485	1	510
NM_020216	-0.2955332	0.66511217	-0.280871517	0.012949689	7486	1	546
NM_016653	0.25682158	0.62784386	-0.174622702	0.02386402	7976	1	788
NM_016653	0.19277698	0.93585553	-0.174622702	0.000206292	8468	1	756
NM_015282	-0.9428144	0.49322088	-0.551279114	0.011131962	7368	1	1831
NM_015282	-1.8608635	0.80555801	-0.551279114	0.027181669	6011	1	2176
CN643589	-0.380642	-0.128076	-0.233489404	0.036312905	7770	1	1718
NM_002536	-0.2681779	0.31268222	-0.327955977	0.006055848	7815	1	479
NM_181711	0.42032454	0.78166635	-0.312288408	0.010078031	8456	1	919
NM_181711	0.50016068	0.84431709	-0.312288408	0.008163535	8457	1	1140
NM_018035	-0.1193011	0.54398881	-0.17594642	0.000955586	7965	1	283
NM_018035	-0.4214132	0.74933051	-0.17594642	0.00066544	7453	1	731
CB548548	-0.4787394	0.71273941	-0.531600643	0.003363098	7418	1	727
NM_139137	0.9331414	0.76046127	-0.35753141	0.036134733	9637	1	1863
NM_015380	0.12292653	0.16962676	-0.302784776	0.007244213	7883	1	713
NM_012255	-0.0884564	0.99643156	-0.214429017	0.018377275	8189	1	962
NM_014427	0.24507274	0.88551612	-0.224520421	0.023350901	8475	1	1204
NM_002475	-0.0833723	0.208943	-0.180439607	0.006826789	7814	1	819

NM_002475	-0.0261364	0.3878151	-0.180439607	0.010972903	7918	1	449
NM_006312	0.20961118	0.24928487	-0.053336546	0.041729638	7901	1	1247
NM_006312	0.05543303	0.55848822	-0.053336546	0.00182766	7980	1	661
NM_207497	0.34011934	0.28039048	-0.863000945	0.037733999	8689	1	1676
NM_005326	-0.0325744	0.76159203	-0.06875835	0.004479453	8141	1	701
NM_005326	-0.0204354	0.80156064	-0.06875835	0.015102268	8142	1	781
NM_031304	0.29824146	0.04516975	-0.275403623	0.026106719	7897	1	1319
NM_022830	0.300105	0.30895164	-0.203640955	0.019092276	7937	1	924
NM_006165	-0.2573386	0.79679528	0.058270959	0.030869055	9491	1	1509
NM_006165	-0.762866	0.79526091	0.058270959	0.02379201	6583	1	1985
NM_014686	0.10858088	0.73757983	-0.09974304	0.027707967	8140	1	856
XM_496408	-0.1153052	0.92917321	-0.060458599	0.010015271	9487	1	1008
NM_012257	-0.069303	0.54853722	-0.287800264	0.000929569	7489	1	110
NM_012257	-0.1065356	0.64869054	-0.287800264	0.01658228	8123	1	304
CN646981	-0.6344454	0.95144313	-0.638472415	0.00441821	7507	1	1317
NM_032213	-0.1606478	0.46264322	-0.719356286	0.034734792	7536	1	668
NM_032213	-0.1056076	1.11962244	-0.719356286	0.015047907	6540	1	1583
NM_032016	0.21125408	0.95429819	-0.227806358	0.0382198	8389	1	2037
NM_201628	0.23768025	0.40646542	-0.666496276	0.034987586	7537	1	746
NM_002862	-0.2626084	0.13680111	-0.317588624	0.027955157	7808	1	792
NM_198057	0.26855125	0.44568635	-0.247591566	0.032557525	7928	1	586
NM_001321	0.17818575	0.95536752	-0.01223441	0.029271221	8509	1	1482
NM_002018	0.24156199	0.75163933	-0.210105926	0.004200961	8531	1	509
NM_002018	0.31820389	0.97634422	-0.210105926	0.000459582	8563	1	922
XM_051699	-0.1745262	0.18485657	-0.482818119	0.017625448	7781	1	837
NM_016119	0.0689514	1.08361734	-0.115505868	0.003463244	9074	1	1225
NM_016119	0.2508175	1.13091757	-0.115505868	0.001055954	9081	1	1465
NM_032148	0.08491936	0.23917907	-0.280561242	0.003888984	7892	1	471
NM_004094	0.09305015	0.7136836	-0.370549713	0.013816529	8410	1	295
NM_004094	-0.4520871	0.75163599	-0.370549713	0.012368273	6608	1	1474
NM_000177	-0.3381723	0.54458096	-0.320497413	0.000882871	7434	1	204
NM_000177	-0.1821805	0.96592244	-0.320497413	0.000911772	9062	1	467
NM_000177	-0.3714698	0.56408179	-0.320497413	0.001945318	7433	1	264
NM_000177	-0.1524917	0.97432214	-0.320497413	0.000256484	9063	1	359
NM_004628	-0.2050878	0.77495149	-0.762038544	0.0176755	8006	1	617
NM_004628	-0.2574255	0.77998861	-0.762038544	0.025171554	8005	1	954
NM_000252	0.33449362	0.98950414	-0.36269499	0.009473887	8859	1	1180
NM_000252	-0.078142	1.05721935	-0.36269499	0.00909956	8857	1	1170
NM_173074	0.17880409	0.50839533	-0.213941592	0.032972652	8393	1	1301
NM_000988	0.80921105	0.53334162	-0.220095331	0.028525602	8447	1	1827
NM_013373	0.20500773	0.25471484	-0.430833813	0.010766477	7881	1	531
NM_013373	0.38070098	0.20672944	-0.430833813	0.014271053	7934	1	980
CB550361	0.1200896	1.07750014	-0.2202578	0.000159542	9078	1	1000

CB550361	0.30943996	0.91179128	-0.2202578	0.002395219	8394	1	1341
NM_019897	0.21362474	0.65959694	-0.299381013	0.030947454	8386	1	949
NM_030577	-0.0441776	1.18044201	-0.285428053	0.013342803	8981	1	1663
NM_002656	-2.7165828	0.58535932	-0.307101586	0.012554074	13	1	2190
NM_018173	-0.2003468	0.5151142	-0.376238243	0.03407815	7789	1	595
NM_005180	-0.0038008	0.61712043	-0.294577721	0.022190112	8133	1	245
NM_005180	0.00841994	0.80252202	-0.294577721	0.003114089	8126	1	281
CN802553	-0.0468247	0.64137321	-0.237862379	0.002234421	7491	1	205
XR_014094	-0.8042148	0.20810283	-0.19906374	0.01766639	7386	1	1830
XR_014094	-0.798418	0.36249278	-0.19906374	0.01684767	7388	1	1762
NM_001003656	0.40950574	0.65155169	-0.247746004	0.046644692	8433	1	1299
NM_001003656	-0.0332028	1.16882612	-0.247746004	0.015353603	8934	1	1535
NM_021729	-0.146547	0.5457676	-0.092464909	0.000234691	7966	1	484
NM_007254	-0.7352015	0.5468541	-0.355496473	0.005153844	7400	1	1349
NM_021003	0.37956366	1.09130194	-0.274184433	0.001192818	8564	1	1196
NM_006306	0.31689368	0.80643541	-0.402909864	0.000915429	8453	1	653
NM_004861	0.20906215	0.30141566	-0.461661188	0.04116725	7880	1	692
NM_145806	0.36157097	0.68810554	-0.121844329	0.031564622	8474	1	1310
NM_001151	-0.3223001	0.96794157	-0.30239268	0.000768103	7475	1	691
NM_001151	-0.0489551	0.91209701	-0.30239268	0.001897341	8127	1	330
NM_016074	0.02154789	0.22082124	-0.08535032	0.031317754	7917	1	993
CN803418	0.12966065	0.4146539	-0.21101055	0.009860352	7925	1	390
CN803418	0.16352859	0.54832216	-0.21101055	0.014581602	7930	1	452
NM_003748	-0.2643312	0.38036218	-0.264408783	0.003039192	7431	1	377
NM_003748	-0.106175	0.3171994	-0.264408783	0.017105465	7853	1	433
NM_003748	-0.3846693	0.70544381	-0.264408783	0.001990564	7452	1	406
NM_014753	-0.1141475	0.51035928	-0.392976046	0.029522891	8104	1	193
NM_006117	-0.2741633	0.70935074	-0.731491491	0.015853247	8024	1	615
NM_001365	0.45142802	0.8096052	-0.011403682	0.037547986	8497	1	1592
NM_015346	0.24865438	0.89702315	-0.171226953	0.028637418	8392	1	1399
NM_015346	0.23511345	0.92139558	-0.171226953	0.023462847	8429	1	1154
NM_006244	-0.5738709	0.36344016	-0.219167982	0.024455253	7389	1	1297
NM_001022	-0.1126651	0.58535554	-0.021289048	0.002592666	7967	1	716
NM_207332	-1.0710897	0.78131133	-0.388237051	0.009763644	6592	1	2010
NM_020689	0.03083828	1.05768571	-0.194259149	0.016755771	8476	1	1529
CN802766	0	0.1685438	-0.424441454	0.044881689	7871	1	654
XR_012406	-0.6655282	0.6771928	-0.126077545	0.022428879	7498	1	1893
XR_012406	-0.121228	0.79429568	-0.126077545	0.035966434	8391	1	1188
NM_025233	0.07878823	0.19649393	-0.288780186	0.01301337	7891	1	592
NM_139168	0.11340763	0.80962326	-0.378567712	0.027471537	8427	1	786
NM_015953	0.0175461	0.1762972	-0.116312186	0.01746803	7894	1	999
NM_001431	-0.3567422	0.93925405	0.045234476	0.008829673	9483	1	1541
NM_015902	0.05476777	0.8733919	-0.48929817	0.003542224	8283	1	230

NM_015902	0.33529334	1.03963272	-0.48929817	0.001105259	8513	1	1092
NM_003131	0.10606187	0.76038191	-0.338747262	0.007320422	8132	1	217
NM_016086	-0.3044529	0.48280097	-0.132519938	0.03668998	6609	1	1851
NM_016086	-0.2340154	0.67216289	-0.132519938	0.044359913	8379	1	1675
NM_001146	0.38236928	0.65997452	-0.081944324	0.012774004	8490	1	1156
NM_001146	0.53319785	1.04818085	-0.081944324	0.0213796	9678	1	1909
XR_009835	-0.0761442	0.78101249	-0.33275458	0.004658453	9060	1	209
XR_009835	-0.2039167	1.17100342	-0.33275458	0.001066023	9082	1	1108
NM_017864	-0.283073	0.27156862	-0.441322978	0.032620645	7786	1	718
NM_018356	0.24580809	0.69573562	-0.394986013	0.017288305	8426	1	335
NM_020187	0.05478887	0.09657804	-0.16201381	0.044352215	7893	1	1136
NM_020187	0.3935661	0.4611863	-0.16201381	0.000453337	7946	1	940
NM_138349	0.20415901	0.3493661	-0.353931108	0.016190109	7879	1	464
NM_006590	0.0673826	0.58947373	-0.075150591	0.036241277	7933	1	726
NM_006590	-0.0136929	0.65148711	-0.075150591	0.032841346	8139	1	860
NM_000673	-0.8660136	0.85177814	-0.647497739	0.020036806	6018	1	2177
NM_005735	-0.2664281	0.45999849	-0.081097888	0.001015489	7438	1	730
NM_005735	-0.3377381	0.48406056	-0.081097888	0.006629731	7437	1	808
NM_015687	-0.0332294	1.08271676	-0.268495991	0.000693317	9067	1	839
XR_014091	0.87850679	0.09983573	-0.483689323	0.048907282	10871	1	2172
NM_003185	-0.9705086	0.55176808	-0.252403172	0.00268378	7497	1	1611
NM_004926	0.102495	1.1085872	-0.662748195	0.023228368	8894	1	1738
NM_004926	-0.1475001	1.06123369	-0.662748195	0.028790114	6539	1	1669
NM_003799	-0.4946937	0.64800341	-0.384122515	0.001720679	7419	1	565
NM_016618	-0.2257266	0.58871396	-0.385427823	0.020984686	8107	1	287
NM_016618	-0.253451	0.7230163	-0.385427823	0.038001309	6601	1	623
NM_004667	0.11824333	0.77751931	-0.140325154	0.001534794	8520	1	648
NM_004667	0.2886104	0.58882317	-0.140325154	0.016576473	8384	1	1241
NM_024843	-0.3082432	0.460948	-0.346321098	0.042415858	8103	1	453
CN644249	-0.1387938	0.52825022	-0.01955431	0.045015488	7963	1	968
CN644249	0.16377743	0.63308472	-0.01955431	0.040058174	7989	1	1046
NM_025057	-0.1049149	0.58932009	-0.912055997	0.031996802	7338	1	1112
NM_032641	-0.3724387	0.1596776	-0.262672999	0.014730867	7778	1	1194
NM_032641	-0.0503311	0.32837045	-0.262672999	0.001388224	7914	1	752
NM_052880	0.11438896	0.70267734	-0.239631049	0.029795595	8134	1	625
XM_208361	0.17391787	0.99041883	-0.196531403	0.001051784	8559	1	761
XM_208361	0.17091575	1.04543098	-0.196531403	0.001280621	8560	1	883
AY369845	-0.1930394	0.86889985	0.10302497	0.007684101	9494	1	1457
NM_000979	0.17439943	0.54176871	-0.207637022	0.000833567	7984	1	318
NM_000979	0.24031194	0.85714986	-0.207637022	0.000258885	8562	1	530
NM_001001336	0.35161322	0.74790761	-0.582447693	0.010504811	8284	1	787
NM_144676	0.66892014	0.34568638	-0.441538968	0.023634658	8908	1	1912
XM_208213	0.6166084	0.68218165	-0.711418804	0.027717658	8752	1	1834

NM_003983	-1.1234077	1.04363765	-0.541893282	0.003374026	6578	1	2094
NM_004712	0.37220601	0.23228144	-0.357633336	0.021143531	7935	1	897
NM_004712	0.24171525	0.45268279	-0.357633336	0.004554853	8086	1	243
NM_003153	-0.3628505	0.43912971	-0.11640031	0.040958082	7458	1	1059
NM_018082	0.40606935	1.03368219	-0.28679223	0.004911231	8486	1	1679
NM_013234	0.23142164	0.71708269	-0.044821659	0.001400887	8533	1	862
NM_024045	-0.354704	0.7489616	-0.062663243	0.012164713	9480	1	1001
NM_024045	-0.1792007	0.79309433	-0.062663243	0.04783359	9485	1	1090
XR_012074	-0.2739893	0.43835559	-0.232421205	0.011756587	7435	1	469
XR_012074	-0.3106411	0.74101065	-0.232421205	0.003500705	8119	1	524
NM_138998	0.08538249	0.50165851	-0.079558883	0.001545184	7979	1	642
NM_138998	0.1604079	0.62316173	-0.079558883	6.96E-05	7981	1	640
NM_001036	-0.0227549	0.03356616	-0.236724933	0.028913862	7812	1	1161
NM_014765	0.03531361	0.59930117	-0.281815104	0.015245709	8136	1	255
NM_025109	-1.0390411	1.15472968	-0.103532197	0.007693478	6585	1	2131
NM_016598	0.26371912	0.6140384	0.013749867	0.025897924	7949	1	1222
NM_016598	0.31616705	0.85694809	0.013749867	0.001071695	8539	1	1324
NM_001402	0.16212125	0.82592593	-0.194708928	0.005426489	8558	1	532
NM_001402	0.24799859	0.94395959	-0.194708928	0.009305949	8561	1	970
NM_024935	0.34155995	0.51139176	-0.236919431	0.006029609	7954	1	497
NM_005435	-0.2147068	0.38224262	-0.299850516	0.013574285	7854	1	367
NM_153646	0.08830126	0.58713588	-0.079291149	0.045938028	7978	1	1005
NM_054108	1.13559721	0.9173983	-0.333424229	0.02179177	9714	1	2120
NM_024619	-0.5874024	0.6626556	-0.2424719	0.008657834	7454	1	1103
NM_024619	-0.2851572	0.86515901	-0.2424719	0.000189144	8120	1	575
NM_003370	0.40554859	0.69742033	-0.119519528	0.004212079	8491	1	1203
NM_006083	-0.3804136	0.90766932	-0.013609783	0.005881351	9481	1	1337
NM_006083	-0.2188509	1.01968072	-0.013609783	0.017240566	9490	1	1463
NM_006083	-0.2543862	1.05518013	-0.013609783	0.01774253	9489	1	1560
NM_006083	-0.3098813	1.07294639	-0.013609783	0.018340865	9488	1	1620
NM_014757	0.19141981	0.22894995	-0.155530417	0.047077136	7890	1	1004
NM_003848	0.09165202	0.47383799	-0.491832604	0.023362681	8113	1	172
NM_000240	-0.9184734	0.81348342	0.095955392	0.023170414	6582	1	2052
NM_000240	-0.9983801	1.23368071	0.095955392	0.023780289	9445	1	2138
NM_153002	0.77848787	1.01667941	-0.323106867	0.028033324	9728	1	2141
NM_031924	0.00477562	1.09339183	0.054087294	0.007920549	9498	1	1706
NM_024692	-0.0049003	0.56540446	-0.962280947	0.044472976	7339	1	1665
NM_000986	0.51614685	0.6303114	0.035745145	0.043737137	8581	1	1661
NM_000986	0.46154618	0.81277957	0.035745145	0.027163676	8583	1	1656
XR_014351	-0.2095794	0.33037024	-0.114440303	0.013594075	7799	1	1087
NM_000919	0.31406182	0.5030491	-0.059523103	0.035201616	7948	1	1105
CB552954	0.51097553	0.65074648	-0.040900352	0.026138412	8506	1	1666
NM_003992	0.10802658	0.61402257	-0.425544452	0.008330231	8114	1	123

NM_003992	-0.287551	0.64186467	-0.425544452	0.013642141	8105	1	233
NM_004495	-0.4183855	0.98960183	-0.341056657	0.039373692	6602	1	1601
NM_014883	-0.7652736	1.17644361	-0.163542642	0.00820747	9426	1	1990
NM_153269	0.59741527	0.54119062	-0.325016625	0.030566979	8815	1	1458
CN648623	-0.4160633	0.73336492	-0.503153781	0.0127143	7422	1	729
CN648623	-0.6014414	0.99629283	-0.503153781	0.008287421	7421	1	1479
XR_012187	0.04845961	0.80361772	0.153992501	0.003112699	8594	1	1573
XR_012187	0.23079532	0.86512441	0.153992501	0.002526743	8595	1	1685
NM_173197	0.87632478	0.80932944	-0.122624522	0.013240553	8500	1	1997
XR_014644	-0.1066892	0.76395286	-0.194812309	0.029403579	7492	1	812
XR_014644	-0.0936529	0.58378939	-0.194812309	0.028071946	7490	1	576
A_01_P011652	-0.8040444	0.36389171	-0.440585231	0.025478944	6547	1	1820
CN647342	-0.4801214	0.52155716	-0.172681775	0.022659683	7459	1	1137
NM_012476	0.31439984	0.86967256	0.20274353	0.025434596	8609	1	1839
NM_012476	0.47386748	1.09312886	0.20274353	0.027132101	9679	1	2023
NM_001017	0.28430298	0.46504443	0.127133172	0.015538878	7994	1	1547
NM_014033	-0.1626254	1.14387691	-0.046810424	0.017731222	9121	1	1623
XR_012158	0.14559716	0.12081742	-0.127473794	0.037088721	7895	1	1217
NM_016096	0.27366499	0.9100949	-0.030478502	0.016403714	8543	1	1332
NM_016096	0.4978271	0.82257553	-0.030478502	0.041253718	8587	1	1781
NM_016474	0.14690608	0.50925129	-0.169199599	0.049846319	7916	1	1212
NM_022476	0.14654558	1.07917891	-0.521433833	0.022886192	8855	1	1604
NM_000978	0.41294181	0.85643673	-0.139577487	0.005934115	8547	1	1165
NM_000978	0.45192654	0.84525239	-0.139577487	0.034138963	8546	1	1488
NM_015484	0.29803418	0.91450341	-0.2524042	0.001745911	8565	1	817
NM_015484	0.27220086	0.77393519	-0.2524042	0.002620453	8510	1	662
NM_017850	-0.5134716	1.18246777	0.227201566	0.032724112	9429	1	2048
NM_017881	0.06686774	0.76875449	-0.018352661	0.00317228	8524	1	946
NM_017881	0.23511467	0.73722552	-0.018352661	0.000784864	8525	1	1135
NM_014236	0.18537692	0.91837873	0.019086245	0.001030768	8523	1	1267
NM_014236	0.24785924	0.81011935	0.019086245	0.006659016	8535	1	1215
NM_144726	0.21134802	0.81006415	-0.038033174	0.032699358	8542	1	1214
CO647185	-0.1325976	1.11014839	-0.14131109	0.006871507	9065	1	1346
CO647185	-0.4783169	1.08768071	-0.14131109	0.003411829	9440	1	1556
NM_001461	-0.5494238	0.99601195	0.106758385	0.032684956	9461	1	2003
NM_145716	-0.4966274	0.25799131	-0.428291958	0.03975094	7777	1	1115
NM_145716	-0.1563228	0.36352146	-0.428291958	0.025032182	7787	1	488
NM_002954	0.48182469	0.78466003	-0.009399963	0.031976833	8582	1	1619
NM_003903	0.2522684	0.85107586	-0.272707631	0.025273409	8517	1	1079
NM_003903	-0.1358147	1.12565191	-0.272707631	0.029134627	8190	1	1494
NM_002126	-0.527741	0.48121404	-0.623832052	0.026196465	7379	1	1015
NM_024900	0.47007026	0.73823847	-0.518444688	0.024597846	9631	1	1841
NM_024900	-0.3538149	0.91065058	-0.518444688	0.044820658	6607	1	1833

NM_032895	-0.0348574	0.50468064	-0.440409244	0.019068673	7538	1	380
NM_172387	-0.6726862	0.72495556	-0.203503367	0.026176349	7500	1	1553
NM_014345	0.14800394	0.80899358	-0.377479806	0.013041368	8204	1	552
NM_014345	0.12492179	0.9404558	-0.377479806	0.017300812	8205	1	815
NM_176792	0.34725659	0.29318326	-0.032189583	0.005206826	8383	1	1821
NM_001444	0.24579471	0.46145682	0.031872176	0.002101032	7993	1	1160
NM_002868	0.58787377	0.41783546	-0.033225676	0.002093398	8492	1	1689
NM_014572	-0.4899749	0.63967936	-0.166662081	0.009994594	7455	1	1050
NM_014572	-0.5300107	0.96791057	-0.166662081	0.000482448	7471	1	1302
CK231978	-0.4257223	1.19757142	0.061783727	0.004775604	9431	1	1858
NM_003654	0.04009715	0.97764665	-0.515324141	0.015231924	8203	1	775
NM_198843	-0.5230776	1.12720057	0.132192488	0.026818202	9430	1	1975
DR770587	-0.1744023	0.31884592	-0.184119115	0.036620557	7798	1	753
NM_001759	-0.1189805	0.6632499	-0.48209231	0.030276886	8112	1	366
NM_001007	0.19914837	0.80203945	0.07234691	0.015479594	8534	1	1366
NM_001007	0.183482	0.9144121	0.07234691	0.005348454	8536	1	1419
NM_002434	-0.1802896	0.38014476	-0.016422637	0.017762865	7958	1	1060
NM_002434	-0.103918	0.46097292	-0.016422637	0.011801306	7960	1	921
NM_014640	-0.134607	0.7063372	0.128061044	0.000606159	9499	1	1618
NM_001007258	0.062349	0.4267843	-0.104930948	0.038935303	7973	1	737
XM_928905	-0.1000036	0.46143992	0.079095574	0.031352483	7964	1	1162
NM_002045	1.03759704	0.83358482	-0.256493623	0.016010272	9710	1	2121
NM_030963	0.41819054	0.94016636	-0.070502602	0.007805566	8548	1	1498
NM_032029	0.95353005	0.99470685	-0.349387188	0.032242191	9699	1	2081
NM_002749	0.50363247	1.12453349	-0.166559321	0.043158993	9681	1	1977
NM_007062	0.26339387	0.89426682	0.030810146	0.00110065	8537	1	1373
NM_007062	0.0569756	1.04137114	0.030810146	0.00676306	8530	1	1526
NM_017790	-0.0809984	0.65340822	-0.054035015	0.001440075	7969	1	645
NM_017790	-0.1084405	0.6763248	-0.054035015	0.001298982	7968	1	677
NM_000725	0.55445882	0.64542748	-0.726969923	0.045100454	8743	1	1939
NM_018312	-0.1431093	0.71487229	-0.289349747	0.024192907	8124	1	574
NM_019110	-0.8065923	0.55800931	-0.3457189	0.04352728	6570	1	2114
NM_020444	0.26096348	0.60669485	-0.066072738	0.004615073	8532	1	854
CN804793	-0.6029828	0.88439787	-0.431864337	0.015369842	7420	1	1414
CN804793	-0.1272442	0.51909178	-0.431864337	0.041241308	8111	1	462
NM_020400	0	0.32292256	-0.019083938	0.022658355	7959	1	1020
NM_001347	0.2517658	0.84477467	-0.272518237	0.004513564	8511	1	657
NM_001347	0.00893562	1.02067706	-0.272518237	0.03718224	8974	1	1493
CN642722	-0.0890088	0.35174795	0.134508388	0.0306526	7961	1	1540
CN642722	-0.0882185	0.4920863	0.134508388	0.012446205	7962	1	1469
NM_001015	-0.1393675	0.89368176	0.135682222	0.00094954	9495	1	1513
NM_001015	-0.0242419	0.87366961	0.135682222	0.000647941	9496	1	1445
NM_012184	0.0308162	0.41764507	-0.275279552	0.022737385	7784	1	1451

NM_172251	-0.5617537	0.81738208	-0.515806739	0.048533011	7417	1	1684
NM_004430	1.0378331	0.9650701	-0.439051515	0.03048677	9698	1	2108
NM_014288	0.38713278	0.36927372	-0.090254986	0.023929021	7956	1	1491
NM_138341	0.10709078	0.82374561	-0.161942686	0.002807677	8521	1	649
NM_014749	0.15404647	1.06748043	0.269140059	0.01648538	8601	1	1930
NM_015423	0.72687304	1.03702316	-0.103913624	0.048321801	9682	1	2042
XR_014073	-0.3688515	0.51396479	-0.163128119	0.010883026	7439	1	710
NM_015292	0.12059925	0.34143378	0.057939551	0.003901373	7991	1	1295
NM_007048	-0.536598	0.5510857	0.19863795	0.041364811	9466	1	1829
NM_007048	-0.2988144	0.83616055	0.19863795	0.033473089	9497	1	1792
NM_003925	0.17397477	0.81535829	0.25564007	0.029035569	8600	1	1794
NM_006432	0.23138049	0.91514281	0.25025985	0.004281403	8599	1	1811
NM_006432	0.15793712	0.81057899	0.25025985	0.038804213	8598	1	1748
NM_153337	0.84612048	0.67108165	-0.481388082	0.027090341	9638	1	2055
NM_012156	-0.0978229	0.51901848	-0.009351595	0.025976463	8377	1	1287
NM_175609	-0.2427885	-0.0361781	0.076535939	0.037057594	3096	1	1849
NM_017751	-0.5979688	0.65680309	-0.01160228	0.004016284	7456	1	1525
XR_013569	-0.1684199	0.41935614	-0.30230361	0.048265501	7476	1	564
NM_021969	-0.3034206	0.8511126	-0.153399066	0.019693336	8121	1	1018
NM_031469	0.18299301	1.1447601	-0.162047281	0.004892907	8395	1	1659
NM_000921	0.31603004	0.54368162	0.069720258	0.010038957	7995	1	1579
XR_010710	-0.6777926	1.04127697	0.095732059	0.005454651	9427	1	1979
NM_198976	-0.1729942	1.09930014	0.180305847	5.61E-05	9527	1	1761
NM_030877	0.27331094	0.29549334	-0.145863845	0.038127709	7945	1	1023
NM_030877	0.24615266	0.5641717	-0.145863845	0.030766676	7975	1	850
NM_014455	0.45325918	0.49380352	-0.131507311	0.015121087	7957	1	1331
NM_014455	0.50197992	0.53314887	-0.131507311	0.034302898	7950	1	1372
XR_011365	-0.5065632	0.87932309	-0.282851217	0.009281878	7423	1	1231
NM_000976	0.28998545	0.8054605	-0.014223017	0.001816818	8538	1	1147
NM_000976	0.29375939	0.88012019	-0.014223017	0.03522251	8544	1	1472
CO725791	0.19764961	0.9862963	-0.434256336	0.040302684	8991	1	1420
NM_002160	0.23958027	0.89070934	0.155587332	0.000862856	8551	1	1688
NM_002160	0.06876073	0.78120778	0.155587332	0.008719709	8550	1	1519
CN648422	0.12471397	1.15214321	0.167549104	0.024591105	9544	1	1928
CN648422	0.09352078	1.1645094	0.167549104	0.031980413	9543	1	1945
NM_020679	0.59062109	0.85711805	0.243090007	0.006036901	8592	1	1998
BE568701	0.16528344	0.97198044	0.17151881	0.006203132	8608	1	1734
BE568701	0.13665167	1.09947006	0.17151881	0.049608042	9131	1	1901
NM_002027	0.39151215	1.09536971	0.043865157	0.000961524	8579	1	1796
NM_002027	0.34927639	1.00103612	0.043865157	0.010372851	8545	1	1678
NM_021161	-0.5966881	0.90772437	-0.146164891	0.014735068	7501	1	1696
NM_130897	-0.3816511	0.75642423	-0.502030097	0.022118679	8026	1	725
XM_371151	-0.0361405	0.65144999	-0.085808966	0.010847098	7971	1	566

NM_152758	0.09352335	0.45991889	-0.092110859	0.021380342	8378	1	1436
DarkCorner	-0.2668412	0.50277849	-0.194336087	0.048747711	7477	1	990
DarkCorner	-0.1766372	0.39397004	-0.194336087	0.043033332	7855	1	549
DarkCorner	0.10855347	0.40046052	-0.194336087	0.044411795	7951	1	748
NM_024519	-0.1566981	1.14470237	0.27555435	0.049740876	9522	1	2020
NM_005094	-0.8656114	0.64027978	-0.111786405	0.030657029	6580	1	1895
NM_014631	0.12410033	0.37887429	-0.101622079	0.022518283	7990	1	804
XR_010010	0.23816484	0.3404163	0.197810203	0.020729076	7992	1	1699
NM_000245	0.16363722	0.98755305	0.006121746	0.005138905	8541	1	1375
XR_011569	0.50180994	1.12918029	-0.15944024	0.006901478	8519	1	1872
BC057815	0.38893641	0.85535287	0.215694393	0.000378031	8589	1	1828
NM_007265	0.15863168	0.73170387	0.07844559	0.002054496	7996	1	1358
NM_007265	0.08993475	0.91359866	0.07844559	0.00188532	8527	1	1544
NM_001330	0.46304253	0.95993864	-0.763441721	0.024966811	8781	1	1742
NM_003496	-0.1182542	1.1166452	0.022213515	0.038947746	9503	1	1842
NM_003496	-0.2951356	0.93056089	0.022213515	0.046123327	9502	1	1694
NM_003274	-0.6462693	1.04427992	0.015163822	0.010890642	6587	1	1993
NM_003274	0.05891446	0.98085111	0.015163822	0.013568903	8529	1	1380
NM_130769	0.15537887	1.08388067	-0.237833184	0.028980743	8982	1	1641
NM_020175	0.15097668	0.5507229	-0.055720341	0.025523797	7974	1	868
NM_000984	-0.3791692	0.57021771	0.153699534	0.024841776	9467	1	1566
NM_000984	-0.4324833	0.76553074	0.153699534	0.004492879	9469	1	1635
XM_496304	0.08268861	0.83022276	-0.010554558	0.041664844	8528	1	1197
NM_004661	-0.1546592	-0.0339464	0.111356105	0.04357521	3097	1	1844
NM_177967	0.54306937	1.01720641	-0.347693031	0.040250483	8387	1	1943
NM_006537	0.21359432	0.7135838	0.058836066	0.03849371	8549	1	1473
NM_006400	-0.0166084	0.75854337	-0.096448795	0.010631619	7972	1	683
NM_006400	0.26057989	0.91948984	-0.096448795	0.007552663	8540	1	1121
NM_001008390	0.35538719	0.83953708	-0.322850837	0.049013378	8516	1	1342
XM_497217	0.03113995	0.57114207	-0.338755607	0.046121337	8115	1	421
XM_497217	0.00459758	0.67350562	-0.338755607	0.033321678	8116	1	410
NM_015253	0.5836918	0.71505255	0.147517987	0.029114358	8591	1	1890
NM_138931	0.42641764	1.06856084	0.196579417	0.000166693	8590	1	1917
NM_138931	0.46440628	0.87698284	0.196579417	0.03666499	8588	1	1910
NM_000821	-0.386894	0.65612186	-0.109227369	0.011332033	7457	1	1025
NM_145044	0.59048679	0.65110157	-0.250008379	0.020625259	8501	1	1524
NM_003715	-0.0649736	1.16557689	0.175011644	0.008399432	9529	1	1856
NM_003450	-0.9502978	0.83281906	-0.029665823	0.016308359	6581	1	2092
NM_133373	-0.2249874	0.62317395	0.217600025	0.032057535	9471	1	1626
NM_012067	-0.2113476	1.12226394	0.388319417	0.030698695	9524	1	2079
CO645930	0.15232114	1.15502212	-0.220991858	0.006846095	8983	1	1462
NM_024059	0.38992632	0.82618	0.22922794	0.008921432	8610	1	1871
DR773628	-0.1819031	0.63049247	0.126207586	0.036626041	9470	1	1454

NM_005088	0.50597508	0.88790029	-0.17139857	0.012316521	8503	1	1610
NM_005088	-0.1657572	1.01897995	-0.17139857	0.008564931	9504	1	1202
NM_001009	-0.2946603	0.7901019	0.323748894	0.024344467	9478	1	1881
NM_020782	-0.2141415	0.70484751	0.209303571	0.011408458	9500	1	1782
NM_152247	0.48969954	0.67267726	-0.320741032	0.048078371	8502	1	1471
NM_145023	0.76933156	0.79028147	-0.054838179	0.030013315	8499	1	1970
CO579725	0.40976204	0.60907563	-0.014334607	0.012514819	8504	1	1476
NM_020865	-0.0984718	0.91101661	0.03067903	0.008476213	9506	1	1384
NM_021104	-0.3407956	0.55495678	0.29232803	0.000168427	9468	1	1801
NM_021104	-0.1810499	0.77450567	0.29232803	0.001280354	9474	1	1751
NM_182547	0.2081963	0.96938528	-0.037974384	0.010834704	8522	1	1403
NM_021930	0.13563338	0.96586129	-0.115262446	0.011883217	8512	1	1356
NM_006357	-0.1156319	0.77825699	0.248311492	0.020798608	9475	1	1730
NM_006838	-0.1269754	0.67547481	0.152281583	0.04164972	9472	1	1439
NM_020194	-0.0278296	0.89319637	-0.043552409	0.026831595	9505	1	1258
XM_376420	0.09452449	0.74653855	0.281223039	0.008560713	8596	1	1720
XM_376420	0.10173784	0.82504052	0.281223039	0.048533562	8597	1	1798
NM_006299	0.07549524	1.05119823	0.205927705	0.043045116	9525	1	1929
XR_011734	-0.3750826	0.91091927	0.309757888	0.004101069	9479	1	1915
XR_011734	-0.5346201	0.77030101	0.309757888	0.000595071	9476	1	1949
NM_015238	0.12174748	0.65484252	0.278246327	0.044908534	8604	1	1787
NM_017645	0.39040668	1.01512403	-0.152113317	0.024843339	8518	1	1664
NM_207395	-0.6896321	0.70056421	0.109247982	0.043095236	6586	1	2097
NM_003928	-0.1144028	0.57418546	0.288328197	0.034329354	9473	1	1702
NM_002060	0.09654908	0.93085695	0.493764766	0.018743321	8602	1	2029
XR_012561	0.43984419	1.07128255	0.19817186	0.00494391	8611	1	1967
XR_012561	0.16219189	1.05491844	0.19817186	0.004387494	9526	1	1837
NM_016231	0.36610443	0.77009503	0.18221817	0.038762508	8606	1	1802
NM_016231	0.65872402	1.1110462	0.18221817	0.012526021	8593	1	2056
NM_022494	0.32516941	0.83945234	0.246343348	0.010070149	8607	1	1857
NM_022494	0.20819037	1.01930649	0.246343348	0.008364491	9558	1	1941
NM_002695	0.17747651	0.68632515	0.219254229	0.027828889	8526	1	1680
NM_052845	0.29777559	1.12759696	-0.20704667	0.015110841	8515	1	1671
NM_007159	0.43560422	1.02648857	0.51983123	0.012603585	8612	1	2105
NM_000854	-0.1446511	0.87740316	0.273784964	0.041436762	9508	1	1976
NM_025165	-0.1373629	1.09676639	0.125782854	0.047935264	9507	1	1966
NM_016282	0.4329157	0.60284659	0.325289581	0.039595528	8605	1	1963
NM_001752	-0.397606	0.93813854	0.408946137	0.00876983	9477	1	2014
NM_032603	0.27268228	0.74437399	0.152168402	0.027244329	8505	1	1692
NM_001010986	0.04232665	1.07388886	0.126700301	0.014670503	9555	1	1862
NM_203459	0.40725577	1.04727362	0.132177761	0.048338143	9561	1	1994
NM_020467	-0.6751105	1.19895798	-0.27902009	0.033558309	10636	1	2038
XR_009954	-0.1912246	1.08543703	0.078753594	0.038616832	9442	1	1805

NM_006726	-0.0544354	0.79067012	0.236357148	0.038072336	9501	1	1813
NM_006726	-0.5970168	1.0637183	0.236357148	0.011055638	6588	1	2041
NM_000617	0.13436795	1.11505014	0.489047813	0.025283321	9539	1	2084
NM_015578	0.24133568	0.87628891	0.539059293	0.000793529	8603	1	2060
NM_024116	-0.3416619	0.9292771	0.411111636	0.026142168	9509	1	2076
NM_017990	0.30407713	1.00566048	0.424006342	0.032293333	9559	1	2072
