# Supplementary material for: Transcriptional Reprogramming in Nonhuman Primate (Rhesus Macaque) Tuberculosis Granulomas
Source: PLoS One. 2010 Aug 31;5(8):e12266. doi: 10.1371/journal.pone.0012266 (PMC2930844; doi:10.1371/journal.pone.0012266)
Supplement: Table S10 — This table contains genes with a higher expression in both early and late lesions (i.e. the overlapping genes in Fig. 4A). (0.05 MB PDF) [file pone.0012266.s010.pdf]

GeneName	Description	Symbol
NM_002988	chemokine C-C motif ligand 18 pulmonary and activation-regulated	CCL18
NM_006926	surfactant, pulmonary-associated protein A2	SFTPA2
CN644462		YES1
NM_005063	stearoyl-CoA desaturase delta-9-desaturase	SCD
NM_002965	S100 calcium binding protein A9 calgranulin B	S100A9
NM_001002026	claudin 18	CLDN18 2
CO726190		CD36
NM_001153	annexin A4	ANXA4
XR_013663	Lymphocyte G0 /G1 switch protein 2 Small inducible cytokine A3 like 1/ C-C motif chemokine 3-like 1	SICA3
NM_033051	thymic stromal co-transporter	TSCOT
NM_152999	six transmembrane epithelial antigen of the prostate 2	STEAP2
XR_013675	Glutathione peroxidase 3 precursor GSHPx-3 GPx-3 Plasma glutathione peroxidase GSHPx-P Extracellular glutathione peroxidase GPx-P	GPX3
NM_006770	macrophage receptor with collagenous structure	MARCO
NM_017625	intelectin 1	ITLN1
NM_000228	laminin, beta 3	LAMB3
NM_144595	hypothetical protein FLJ30046	FLJ30046
NM_020415	resistin	RETN
NM_007117	thyrotropin-releasing hormone	TRH
NM_025232	chromosome 8 open reading frame 20	C8orf20
NM_005239	v-ets erythroblastosis virus E26 oncogene homolog 2	ETS2
