# Supplementary material for: Transcriptional Reprogramming in Nonhuman Primate (Rhesus Macaque) Tuberculosis Granulomas
Source: PLoS One. 2010 Aug 31;5(8):e12266. doi: 10.1371/journal.pone.0012266 (PMC2930844; doi:10.1371/journal.pone.0012266)
Supplement: Table S11 — This table contains genes with a lower expression in both early and late lesions (i.e. the overlapping genes in Fig. 4B). (0.05 MB PDF) [file pone.0012266.s011.pdf]

Gene Name	Description	Symbol
NM_012418	fascin homolog 2, actin-bundling protein, retinal (Strongylocentrotus purpuratus) (FSCN2).	FSCN2
NM_024552	LAG1 longevity assurance homolog 4 (S. cerevisiae) (LASS4).	LASS4
NM_024671	hypothetical protein FLJ23436 (FLJ23436).	FLJ23436
NM_138418	hypothetical protein MGC15416 (MGC15416)	MGC15416
