# Supplementary material for: Transcriptional Reprogramming in Nonhuman Primate (Rhesus Macaque) Tuberculosis Granulomas
Source: PLoS One. 2010 Aug 31;5(8):e12266. doi: 10.1371/journal.pone.0012266 (PMC2930844; doi:10.1371/journal.pone.0012266)
Supplement: Table S12 — This table contains genes with a higher expression in early but a lower expression in late lesions (i.e. the overlapping genes in Fig. 4C). (0.13 MB PDF) [file pone.0012266.s012.pdf]

Gene Name	Description	Symbol
NM_013375	activator of basal transcription 1 (ABT1).	ABT1
NM_014049	acyl-Coenzyme A dehydrogenase family, member 9 (ACAD9).	ACAD9
NM_001610	acid phosphatase 2, lysosomal (ACP2).	ACP2
NM_001611	acid phosphatase 5, tartrate resistant (ACP5).	ACP5
NM_005721	ARP3 actin-related protein 3 homolog (yeast) (ACTR3)	ACTR3
NM_001109	a disintegrin and metalloproteinase domain 8 (ADAM8).	ADAM8
NM_014479	ADAM-like, decysin 1 (ADAMDEC1).	ADAMDEC1
NM_001122	adipose differentiation-related protein (ADFP)	ADFP
NM_001124	adrenomedullin (ADM)	ADM
NM_000676	adenosine A2b receptor (ADORA2B).	ADORA2B
NM_020350	angiotensin II receptor-associated protein (AGTRAP).	AGTRAP
NM_005161	angiotensin II receptor-like 1 (AGTRL1)	AGTRL1
NM_000687	S-adenosylhomocysteine hydrolase (AHCY)	AHCY
NM_004847	allograft inflammatory factor 1 (AIF1), transcript variant 2.	AIF1
NM_001628	aldo-keto reductase family 1, member B1 (aldose reductase) (AKR1B1)	AKR1B1
NM_000689	aldehyde dehydrogenase 1 family, member A1 (ALDH1A1)	ALDH1A1
NM_000034	aldolase A, fructose-bisphosphate (ALDOA), transcript variant 1	ALDOA
NM_005165	aldolase C, fructose-bisphosphate (ALDOC)	ALDOC
NM_152326	ankyrin repeat domain 9 (ANKRD9).	ANKRD9
NM_001637	acyloxyacyl hydrolase (neutrophil) (AOAH).	AOAH
NM_001127	adaptor-related protein complex 1, beta 1 subunit (AP1B1), transcript variant 1	AP1B1
NM_004069	adaptor-related protein complex 2, sigma 1 subunit (AP2S1), transcript variant AP17.	AP2S1
XR_013467	Macaca mulatta adaptor-related protein complex 3, beta 1 subunit (AP3B1)	AP3B1
NM_022488	APG3 autophagy 3-like (S. cerevisiae) (APG3L).	APG3L
NM_001645	apolipoprotein C-I (APOC1).	APOC1
NM_003661	apolipoprotein L, 1 (APOL1), transcript variant 1.	APOL1
NM_030643	apolipoprotein L, 4 (APOL4), transcript variant a	APOL4
NM_020979	adaptor protein with pleckstrin homology and src homology 2 domains (APS)	APS
NM_004309	Rho GDP dissociation inhibitor (GDI) alpha (ARHGDIA).	ARHGDIA
NM_015161	ADP-ribosylation factor-like 6 interacting protein (ARL6IP).	ARL6IP
NM_005720	actin related protein 2/3 complex, subunit 1B, 41kDa (ARPC1B)	ARPC1B
NM_183376	arrestin domain containing 4 (ARRDC4)	ARRDC4
NM_005170	achaete-scute complex-like 2 (Drosophila) (ASCL2)	ASCL2

NM_025080	asparaginase like 1 (ASRGL1)	ASRGL1
NM_004024	activating transcription factor 3 (ATF3)	ATF3
NM_006886	ATP synthase, H+ transporting, mitochondrial F1 complex, epsilon subunit (ATP5E), nuclear gene encoding mitochondrial protein	ATP5E
NM_004691	ATPase, H+ transporting, lysosomal 38kDa, V0 subunit d isoform 1 (ATP6V0D1).	ATP6V0D1
NM_001693	ATPase, H+ transporting, lysosomal 56/58kDa, V1 subunit B, isoform 2 (ATP6V1B2).	ATP6V1B2
NM_001497	UDP-Gal:betaGlcNAc beta 1,4- galactosyltransferase, polypeptide 1 (B4GALT1).	B4GALT1
NM_001188	BCL2-antagonist/killer 1 (BAK1).	BAK1
NM_006399	basic leucine zipper transcription factor, ATF-like (BATF)	BATF
NM_030766	BCL2-like 14 (apoptosis facilitator) (BCL2L14), transcript variant 2	BCL2L14
NM_001710	B-factor, properdin (BF)	BF
NM_001165	baculoviral IAP repeat-containing 3 (BIRC3), transcript variant 1	BIRC3
NM_004335	bone marrow stromal cell antigen 2 (BST2).	BST2
NM_152322	BTB (POZ) domain containing 11 (BTBD11), transcript variant 1.	BTBD11
NM_007311	benzodiazapine receptor (peripheral) (BZRP), transcript variant PBR-S	BZRP
NM_022338	chromosome 11 open reading frame 24 (C11orf24).	C11orf24
NM_023933	chromosome 16 open reading frame 24 (C16orf24).	C16orf24
NM_174896	chromosome 1 open reading frame 162 (C1orf162).	C1orf162
NM_015991	complement component 1, q subcomponent, alpha polypeptide (C1QA)	C1QA
NM_000491	complement component 1, q subcomponent, beta polypeptide (C1QB)	C1QB
NM_198594	C1q and tumor necrosis factor related protein 1 (C1QTNF1)	C1QTNF1
NM_001734	complement component 1, s subcomponent (C1S), transcript variant 1	C1S
NM_000063	complement component 2 (C2)	C2
NM_080757	chromosome 20 open reading frame 127 (C20orf127).	C20orf127
NM_213720	chromosome 22 open reading frame 16 (C22orf16)	C22orf16
NM_024053	chromosome 22 open reading frame 18 (C22orf18)	C22orf18
NM_138408	chromosome 6 open reading frame 51 (C6orf51)	C6orf51
NM_001001790	chromosome 9 open reading frame 105 (C9orf105).	C9orf105
NM_001218	carbonic anhydrase XII (CA12), transcript variant 1.	CA12
NM_001216	carbonic anhydrase IX (CA9)	CA9
NM_001219	calumenin (CALU).	CALU
NM_006367	CAP, adenylate cyclase-associated protein 1 (yeast) (CAP1)	CAP1
NM_001747	capping protein (actin filament), gelsolin-like (CAPG).	CAPG

NM_002986	chemokine (C-C motif) ligand 11 (CCL11)	CCL11
NM_006274	chemokine (C-C motif) ligand 19 (CCL19).	CCL19
NM_001238	cyclin E1 (CCNE1), transcript variant 1	CCNE1
NM_001761	cyclin F (CCNF).	CCNF
NM_001295	chemokine (C-C motif) receptor 1 (CCR1).	CCR1
NM_000591	CD14 antigen (CD14).	CD14
XR_010680	Macaca mulatta CD180 antigen (CD180)	CD180
NM_014143	CD274 antigen (CD274).	CD274
NM_007261	CD300A antigen (CD300A).	CD300A
NM_001250	CD40 antigen (TNF receptor superfamily member 5) (CD40), transcript variant 1.	CD40
NM_000560	CD53 antigen (CD53)	CD53
NM_001251	CD68 antigen (CD68).	CD68
NM_006889	CD86 antigen (CD28 antigen ligand 2, B7-2 antigen) (CD86), transcript variant 2	CD86
NM_001785	cytidine deaminase (CDA)	CDA
NM_031299	cell division cycle associated 3 (CDCA3)	CDCA3
NM_080668	cell division cycle associated 5 (CDCA5)	CDCA5
NM_000389	cyclin-dependent kinase inhibitor 1A (p21, Cip1) (CDKN1A), transcript variant 1.	CDKN1A
NM_001803	CDW52 antigen (CAMPATH-1 antigen) (CDW52)	CDW52
NM_005194	CCAAT/enhancer binding protein (C/EBP), beta (CEBPB)	CEBPB
NM_005195	CCAAT/enhancer binding protein (C/EBP), delta (CEBPD)	CEBPD
NM_018404	centaurin, alpha 2 (CENTA2).	CENTA2
NM_005507	cofilin 1 (non-muscle) (CFL1)	CFL1
NM_015703	CGI-96 protein (CGI-96).	CGI-96
NM_016139	coiled-coil-helix-coiled-coil-helix domain containing 2 (CHCHD2)	CHCHD2
XR_010663	Macaca mulatta Mitochondrial intermembrane space import and assembly protein 40 (Coiled-coil-helix-coiled-coil-helix domain-containing protein 4) (CHCHD4)	CHCHD4
NM_176812	chromatin modifying protein 4B (CHMP4B).	CHMP4B
NM_018413	carbohydrate (chondroitin 4) sulfotransferase 11 (CHST11)	CHST11
NM_014918	carbohydrate (chondroitin) synthase 1 (CHSY1)	CHSY1
NM_133467	Cbp/p300-interacting transactivator, with Glu/Asp-rich carboxy-terminal domain, 4 (CITED4)	CITED4
NM_020990	creatine kinase, mitochondrial 1 (ubiquitous) (CKMT1), nuclear gene encoding mitochondrial protein	CKMT1
NM_001827	CDC28 protein kinase regulatory subunit 2 (CKS2)	CKS2
NM_016511	C-type lectin-like receptor-1 (CLEC1)	CLEC1
NM_001289	chloride intracellular channel 2 (CLIC2)	CLIC2

NM_198390	c-Maf-inducing protein (CMIP), transcript variant C-mip	CMIP
NM_018235	CNDP dipeptidase 2 (metallopeptidase M20 family) (CNDP2)	CNDP2
NM_000088	collagen, type I, alpha 1 (COL1A1)	COL1A1
NM_000089	collagen, type I, alpha 2 (COL1A2).	COL1A2
NM_000090	collagen, type III, alpha 1 (Ehlers-Danlos syndrome type IV, autosomal dominant) (COL3A1).	COL3A1
NM_004369	collagen, type VI, alpha 3 (COL6A3), transcript variant 1	COL6A3
NM_024027	collectin sub-family member 11 (COLEC11), transcript variant 1.	COLEC11
NM_052889	CARD only protein (COPl), transcript variant 2.	COPl
NM_021149	coactosin-like 1 (Dictyostelium) (COTL1).	COTL1
NM_004255	cytochrome c oxidase subunit Va (COX5A), nuclear gene encoding mitochondrial protein	COX5A
NM_004074	cytochrome c oxidase subunit 8A (ubiquitous) (COX8A).	COX8A
NM_003652	carboxypeptidase Z (CPZ)	CPZ
NM_003851	cellular repressor of E1A-stimulated genes (CREG)	CREG
NM_031476	cysteine-rich secretory protein LCCL domain containing 2 (CRISPLD2).	CRISPLD2
NM_005211	colony stimulating factor 1 receptor, formerly McDonough feline sarcoma viral (v-fms) oncogene homolog (CSF1R).	CSF1R
NM_004385	chondroitin sulfate proteoglycan 2 (versican) (CSPG2)	CSPG2
NM_000100	cystatin B (stefin B) (CSTB).	CSTB
XR_009750	Macaca mulatta Cathepsin Z precursor (Cathepsin X) (Cathepsin P) (LOC694157)	CTPP
NM_001906	chymotrypsinogen B1 (CTRB1).	CTRB1
NM_147780	cathepsin B (CTSB), transcript variant 2	CTSB
NM_001814	cathepsin C (CTSC), transcript variant 1	CTSC
NM_148170	cathepsin C (CTSC), transcript variant 2.	CTSC
NM_004079	cathepsin S (CTSS)	CTSS
NM_001565	chemokine (C-X-C motif) ligand 10 (CXCL10)	CXCL10
NM_000609	chemokine (C-X-C motif) ligand 12 (stromal cell-derived factor 1) (CXCL12).	CXCL12
NM_022059	chemokine (C-X-C motif) ligand 16 (CXCL16).	CXCL16
NM_002090	chemokine (C-X-C motif) ligand 3 (CXCL3).	CXCL3
NM_002993	chemokine (C-X-C motif) ligand 6 (granulocyte chemotactic protein 2) (CXCL6).	CXCL6
NM_002416	chemokine (C-X-C motif) ligand 9 (CXCL9)	CXCL9
NM_003467	chemokine (C-X-C motif) receptor 4 (CXCR4)	CXCR4
NM_000101	cytochrome b-245, alpha polypeptide (CYBA).	CYBA
NM_000397	cytochrome b-245, beta polypeptide (chronic granulomatous disease) (CYBB).	CYBB

NM_018947	cytochrome c, somatic (CYCS), nuclear gene encoding mitochondrial protein.	CYCS
NM_000104	cytochrome P450, family 1, subfamily B, polypeptide 1 (CYP1B1).	CYP1B1
NM_014395	dual adaptor of phosphotyrosine and 3-phosphoinositides (DAPP1).	DAPP1
NM_001919	dodecenoyl-Coenzyme A delta isomerase (3,2 trans-enoyl-Coenzyme A isomerase) (DCI), nuclear gene encoding mitochondrial protein.	DCI
NM_004753	dehydrogenase/reductase (SDR family) member 3 (DHRS3).	DHRS3
NM_015393	DKFZP564O0823 protein (DKFZP564O0823).	DKFZP564O0823
NM_006145	DnaJ (Hsp40) homolog, subfamily B, member 1 (DNAJB1)	DNAJB1
NM_006260	DnaJ (Hsp40) homolog, subfamily C, member 3 (DNAJC3)	DNAJC3
NM_001005360	dynamin 2 (DNM2), transcript variant 1.	DNM2
NM_004946	dedicator of cytokinesis 2 (DOCK2)	DOCK2
NM_018973	dolichyl-phosphate mannosyltransferase polypeptide 3 (DPM3), transcript variant 1	DPM3
NM_000110	dihydropyrimidine dehydrogenase (DPYD).	DPYD
NM_004416	deltex homolog 1 (Drosophila) (DTX1)	DTX1
NM_004418	dual specificity phosphatase 2 (DUSP2).	DUSP2
NM_006014	DNA segment on chromosome X (unique) 9879 expressed sequence (DXS9879E)	DXS9879E
NM_001953	endothelial cell growth factor 1 (platelet-derived) (ECGF1)	ECGF1
NM_001398	enoyl Coenzyme A hydratase 1, peroxisomal (ECH1).	ECH1
NM_024329	EF hand domain containing 2 (EFHD2)	EFHD2
NM_003757	eukaryotic translation initiation factor 3, subunit 2 beta, 36kDa (EIF3S2)	EIF3S2
NM_001416	eukaryotic translation initiation factor 4A, isoform 1 (EIF4A1)	EIF4A1
NM_004096	eukaryotic translation initiation factor 4E binding protein 2 (EIF4EBP2)	EIF4EBP2
NM_012081	elongation factor, RNA polymerase II, 2 (ELL2)	ELL2
NM_001428	enolase 1, (alpha) (ENO1)	ENO1
NM_001975	enolase 2 (gamma, neuronal) (ENO2)	ENO2
NM_016135	ets variant gene 7 (TEL2 oncogene) (ETV7).	ETV7
NM_020158	exosome component 5 (EXOSC5).	EXOSC5
NM_020223	family with sequence similarity 20, member C (FAM20C)	FAM20C
NM_004460	fibroblast activation protein, alpha (FAP).	FAP
NM_001004019	fibulin 2 (FBLN2), transcript variant 1.	FBLN2
NM_018438	F-box only protein 6 (FBXO6)	FBXO6
NM_012179	F-box protein 7 (FBXO7).	FBXO7

NM_000569	Fc fragment of IgG, low affinity IIIa, receptor for (CD16) (FCGR3A)	FCGR3A
NM_000570	Fc fragment of IgG, low affinity IIIb, receptor (CD16b) (FCGR3B).	FCGR3B
NM_005248	Gardner-Rasheed feline sarcoma viral (v-fgr) oncogene homolog (FGR).	FGR
NM_004468	four and a half LIM domains 3 (FHL3)	FHL3
NM_021939	FK506 binding protein 10, 65 kDa (FKBP10).	FKBP10
NM_054033	FK506 binding protein 1B, 12.6 kDa (FKBP1B), transcript variant 2.	FKBP1B
NM_004117	FK506 binding protein 5 (FKBP5).	FKBP5
NM_018295	hypothetical protein FLJ11000 (FLJ11000).	FLJ11000
NM_025147	hypothetical protein FLJ13448 (FLJ13448)	FLJ13448
NM_024660	hypothetical protein FLJ22573 (FLJ22573)	FLJ22573
NM_024579	hypothetical protein FLJ23221 (FLJ23221)	FLJ23221
NM_198446	FLJ45459 protein (FLJ45459)	FLJ45459
NM_000803	folate receptor 2 (fetal) (FOLR2)	FOLR2
NM_000804	folate receptor 3 (gamma) (FOLR3)	FOLR3
NM_002029	formyl peptide receptor 1 (FPR1)	FPR1
NM_002030	formyl peptide receptor-like 2 (FPRL2)	FPRL2
AL832403	mRNA; cDNA DKFZp667B1913 (from clone DKFZp667B1913)	from clone DKFZp667B19
CR936794	mRNA; cDNA DKFZp781I14186 (from clone DKFZp781I14186)	from clone DKFZp781I14
NM_001465	FYN binding protein (FYB-120/130) (FYB)	FYB
NM_005754	Ras-GTPase-activating protein SH3-domain-binding protein (G3BP), transcript variant 1	G3BP
NM_000402	glucose-6-phosphate dehydrogenase (G6PD), nuclear gene encoding mitochondrial protein	G6PD
NM_015892	B cell RAG associated protein (GALNAC4S-6ST).	GALNAC4S-6ST
NM_002046	glyceraldehyde-3-phosphate dehydrogenase (GAPDH).	GAPDH
NM_152237	growth arrest-specific 2 like 1 (GAS2L1), transcript variant 3	GAS2L1
NM_002053	guanylate binding protein 1, interferon-inducible, 67kDa (GBP1)	GBP1
NM_198460	guanylate binding protein family, member 6 (GBP6).	GBP6
NM_000161	GTP cyclohydrolase 1 (dopa-responsive dystonia) (GCH1)	GCH1
NM_004864	growth differentiation factor 15 (GDF15)	GDF15
NM_015044	golgi associated, gamma adaptin ear containing, ARF binding protein 2 (GGA2), transcript variant 1	GGA2
NM_130759	GTPase, IMAP family member 1 (GIMAP1).	GIMAP1
NM_015660	GTPase, IMAP family member 2 (GIMAP2).	GIMAP2
NM_018326	GTPase, IMAP family member 4 (GIMAP4).	GIMAP4
NM_000169	galactosidase, alpha (GLA)	GLA

NM_002065	glutamate-ammonia ligase (glutamine synthase) (GLUL).	GLUL
XR_010040	Macaca mulatta Glycyl-tRNA synthetase (Glycine--tRNA ligase) (GlyRS) (LOC696505)	GlyRS
NM_000405	GM2 ganglioside activator (GM2A).	GM2A
NM_013334	GDP-mannose pyrophosphorylase B (GMPPB), transcript variant 1	GMPPB
NM_002068	guanine nucleotide binding protein (G protein), alpha 15 (Gq class) (GNA15).	GNA15
NM_002074	guanine nucleotide binding protein (G protein), beta polypeptide 1 (GNB1)	GNB1
NM_005274	guanine nucleotide binding protein (G protein), gamma 5 (GNG5)	GNG5
NM_000175	glucose phosphate isomerase (GPI)	GPI
NM_001005340	glycoprotein (transmembrane) nmb (GPNMB), transcript variant 1.	GPNMB
NM_177551	G protein-coupled receptor 109A (GPR109A)	GPR109A
NM_020370	G protein-coupled receptor 84 (GPR84)	GPR84
AY966403	Callithrix jacchus cytosolic glutathione peroxidase (Gpx-1) mRNA, complete cds	Gpx-1 mRNA
NM_002086	growth factor receptor-bound protein 2 (GRB2), transcript variant 1	GRB2
XR_013405	Macaca mulatta Stress-70 protein, mitochondrial precursor (75 kDa glucose-regulated protein) (GRP 75) (Peptide-binding protein 74) (PBP74) (Mortalin) (MOT) (LOC705066)	GRP 75
NM_002094	G1 to S phase transition 1 (GSPT1)	GSPT1
NM_004130	glycogenin (GYG).	GYG
NM_002101	glycophorin C (Gerbich blood group) (GYPC), transcript variant 1	GYPC
NM_177925	H2A histone family, member J (H2AFJ), transcript variant 2	H2AFJ
NM_002105	H2A histone family, member X (H2AFX)	H2AFX
NM_178232	hyaluronan and proteoglycan link protein 3 (HAPLN3).	HAPLN3
NM_005333	holocytochrome c synthase (cytochrome c heme-lyase) (HCCS)	HCCS
NM_005335	hematopoietic cell-specific Lyn substrate 1 (HCLS1)	HCLS1
NM_014266	hematopoietic cell signal transducer (HCST)	HCST
NM_002112	histidine decarboxylase (HDC).	HDC
NM_004494	hepatoma-derived growth factor (high-mobility group protein 1-like) (HDGF).	HDGF
NM_024711	human immune associated nucleotide 2 (hIAN2)	hIAN2
NM_014056	likely ortholog of mouse hypoxia induced gene 1 (HIG1).	HIG1
NM_138720	histone 1, H2bd (HIST1H2BD), transcript variant 2	HIST1H2BD
NM_003547	histone 1, H4g (HIST1H4G).	HIST1H4G
NM_003541	histone 1, H4k (HIST1H4K).	HIST1H4K

NM_003546	histone 1, H4l (HIST1H4L).	HIST1H4L
NM_000188	hexokinase 1 (HK1), nuclear gene encoding mitochondrial protein, transcript variant 1.	HK1
NM_000189	hexokinase 2 (HK2).	HK2
NM_002115	hexokinase 3 (white cell) (HK3), nuclear gene encoding mitochondrial protein.	HK3
NM_002116	major histocompatibility complex, class I, A (HLA-A).	HLA-A
NM_006120	major histocompatibility complex, class II, DM alpha (HLA-DMA)	HLA-DMA
NM_033554	major histocompatibility complex, class II, DP alpha 1 (HLA-DPA1).	HLA-DPA1
NM_019111	major histocompatibility complex, class II, DR alpha (HLA-DRA).	HLA-DRA
NM_022555	major histocompatibility complex, class II, DR beta 3 (HLA-DRB3)	HLA-DRB3
NM_021983	major histocompatibility complex, class II, DR beta 4 (HLA-DRB4).	HLA-DRB4
NM_002125	major histocompatibility complex, class II, DR beta 5 (HLA-DRB5).	HLA-DRB5
NM_018950	major histocompatibility complex, class I, F (HLA-F)	HLA-F
NM_004838	homer homolog 3 (Drosophila) (HOMER3).	HOMER3
CO646675	ILLUMIGEN_MCQ_39493 Katze_MMPB2 Macaca mulatta cDNA clone IBIUW:23645 5' Bases 32 to 976 highly human CXCL11 (Hs.103982) sequence	Hs103982
CN648055	ILLUMIGEN_MCQ_29639 Katze_MMPB Macaca mulatta cDNA clone IBIUW:6975 5' Bases 1 to 889 highly human GZMB (Hs.1051) sequence	Hs1051
CN801572	ILLUMIGEN_MCQ_35741 Katze_MMPL1 Macaca mulatta cDNA clone IBIUW:14415 5' Bases 1 to 342 highly human HN1 (Hs.109706) sequence	Hs109706
CO644910	ILLUMIGEN_MCQ_43164 Katze_MMJJ Macaca mulatta cDNA clone IBIUW:23286 5' Bases 7 to 752 highly human NMES1 (Hs.112242) sequence	Hs112242
DR771539	ILLUMIGEN_MCQ_66662 Katze_MMTE Macaca mulatta cDNA clone IBIUW:35638 5' Bases 6 to 389 highly human ATOX1 (Hs.125213) sequence	Hs125213
CN644776	ILLUMIGEN_MCQ_10841 Katze_MMPL2 Macaca mulatta cDNA clone IBIUW:9592 5' Bases 1 to 695 highly human PP2135 (Hs.132569) sequence	Hs132569
CN647492	ILLUMIGEN_MCQ_28598 Katze_MMPB Macaca mulatta cDNA clone IBIUW:7532 5' Bases 1 to 534 highly human SPI1 (Hs.157441) sequence	Hs157441
CN648569	ILLUMIGEN_MCQ_30855 Katze_MMPB Macaca mulatta cDNA clone IBIUW:6483 5' Bases 1 to 1036 highly human BHLHB2 (Hs.171825) sequence	Hs171825

CO645773	ILLUMIGEN_MCQ_30118 Katze_MMPB Macaca mulatta cDNA clone IBIUW:22572 5' Bases 1 to 42 highly human RARRES3 (Hs.17466) sequence	Hs17466
CN642140	ILLUMIGEN_MCQ_5952 Katze_MMPL2 Macaca mulatta cDNA clone IBIUW:5117 5' Bases 1 to 669 highly human LGMN (Hs.18069) sequence	Hs18069
CN644277	ILLUMIGEN_MCQ_10044 Katze_MMPL2 Macaca mulatta cDNA clone IBIUW:9084 5' Bases 7 to 588 highly human TFRC (Hs.185726) sequence	Hs185726
CO580929	ILLUMIGEN_MCQ_48009 Katze_MMLV Macaca mulatta cDNA clone IBIUW:18847 5' Bases 166 to 669 highly human SAA2 (Hs.1955) sequence	Hs1955
CN647641	ILLUMIGEN_MCQ_28871 Katze_MMPB Macaca mulatta cDNA clone IBIUW:7393 5' Bases 1 to 845 highly human BZRP (Hs.202) sequence	Hs202
CO581027	ILLUMIGEN_MCQ_47873 Katze_MMOV Macaca mulatta cDNA clone IBIUW:17465 5' Bases 3 to 593 highly human MGC49942 (Hs.206824) sequence	Hs206824
CN643020	ILLUMIGEN_MCQ_7125 Katze_MMPL2 Macaca mulatta cDNA clone IBIUW:4136 5' Bases 1 to 1118 highly human LYZ (Hs.234734) sequence	Hs234734
CO647278	ILLUMIGEN_MCQ_40268 Katze_MMPB2 Macaca mulatta cDNA clone IBIUW:23292 5' Bases 70 to 562 highly human COMT (Hs.240013) sequence	Hs240013
CO647377	ILLUMIGEN_MCQ_40406 Katze_MMPB2 Macaca mulatta cDNA clone IBIUW:21576 5' Bases 2 to 949 highly human APOL3 (Hs.241535) sequence	Hs241535
DR774547	ILLUMIGEN_MCQ_58787 Katze_MMTE Macaca mulatta cDNA clone IBIUW:32927 5' Bases 5 to 768 highly human SNX17 (Hs.278569) sequence	Hs278569
CN647236	ILLUMIGEN_MCQ_28050 Katze_MMBR Macaca mulatta cDNA clone IBIUW:7788 5' Bases 196 to 727 highly human TTYH2 (Hs.27935) sequence	Hs27935
CK230409	ILLUMIGEN_MCQ_731 Katze_MMPL2 Macaca mulatta cDNA 5' human GLRX (Hs.28988) sequence	Hs28988
CO646399	ILLUMIGEN_MCQ_39129 Katze_MMPB2 Macaca mulatta cDNA clone IBIUW:21744 5' Bases 393 to 753 highly human CTSD (Hs.343475) sequence	Hs343475
CO725352	ILLUMIGEN_MCQ_32983 Katze_MMBR Macaca mulatta cDNA clone IBIUW:27860 5' Bases 1 to 613 highly human LSM10 (Hs.3496) sequence	Hs3496
CO648453	ILLUMIGEN_MCQ_41830 Katze_MMPB2 Macaca mulatta cDNA clone IBIUW:25190 5' Bases 185 to 962 highly human C6orf175 (Hs.356224) sequence	Hs356224
CN801994	ILLUMIGEN_MCQ_37433 Katze_MMBR Macaca mulatta cDNA clone IBIUW:16004 5' Bases 1 to 410 highly human MT1X (Hs.374950) sequence	Hs374950

CO648815	ILLUMIGEN_MCQ_42308 Katze_MMPB2 Macaca mulatta cDNA clone IBIUW:25858 5' Bases 5 to 765 highly human SYT11 (Hs.380439) sequence	Hs380439
CO646894	ILLUMIGEN_MCQ_39773 Katze_MMPB2 Macaca mulatta cDNA clone IBIUW:25891 5' Bases 5 to 696 highly human SLC16A3 (Hs.386678) sequence	Hs386678
CO581942	ILLUMIGEN_MCQ_46474 Katze_MMJJ Macaca mulatta cDNA clone IBIUW:19887 5' Bases 82 to 1012 highly human HLA-DQA1 (Hs.387679) sequence	Hs387679
CO579438	ILLUMIGEN_MCQ_50129 Katze_MMOV Macaca mulatta cDNA clone IBIUW:19046 5' Bases 4 to 953 highly human PSAP (Hs.406455) sequence	Hs406455
CN641710	ILLUMIGEN_MCQ_5231 Katze_MMBR Macaca mulatta cDNA clone IBIUW:6025 5' Bases 11 to 158 highly human RPN2 (Hs.406532) sequence	Hs406532
CO646712	ILLUMIGEN_MCQ_39536 Katze_MMPB2 Macaca mulatta cDNA clone IBIUW:23033 5' Bases 5 to 869 highly human IAN4L1 (Hs.412331) sequence	Hs412331
CN645487	ILLUMIGEN_MCQ_24069 Katze_MMSP Macaca mulatta cDNA clone IBIUW:10303 5' Bases 523 to 999 highly human IGHG1 (Hs.413826) sequence	Hs413826
CK231337	ILLUMIGEN_MCQ_1868 Katze_MMLG Macaca mulatta cDNA 5' human RPL7 (Hs.421257) sequence	Hs421257
CO725743	ILLUMIGEN_MCQ_38118 Katze_MMBR Macaca mulatta cDNA clone IBIUW:27611 5' Bases 356 to 881 highly human GNG10 (Hs.433898) sequence	Hs433898
CN644408	ILLUMIGEN_MCQ_10328 Katze_MMPL2 Macaca mulatta cDNA clone IBIUW:9224 5' Bases 70 to 978 highly human COL4A1 (Hs.437173) sequence	Hs437173
CK231327	ILLUMIGEN_MCQ_1845 Katze_MMLG Macaca mulatta cDNA 5' human TFPI2 (Hs.438231) sequence	Hs438231
CK232222	ILLUMIGEN_MCQ_3441 Katze_MMPL2 Macaca mulatta cDNA 5' human TFPI2 (Hs.438231) sequence	Hs438231
DV768600	ILLUMIGEN_MCQ_62274 Katze_MMTE Macaca mulatta cDNA clone IBIUW:40854 5' Bases 5 to 527 highly human IGFBP3 (Hs.450230) sequence	Hs450230
CK232488	ILLUMIGEN_MCQ_4157 Katze_MMPL2 Macaca mulatta cDNA 5' human PSG4 (Hs.458318) sequence	Hs458318
DV770912	ILLUMIGEN_MCQ_70634 Katze_MMTE Macaca mulatta cDNA clone IBIUW:40195 5' Bases 4 to 751 highly human HLA-DRB1 (Hs.520049) sequence	Hs520049
CN804935	ILLUMIGEN_MCQ_35237 Katze_MMPL1 Macaca mulatta cDNA clone IBIUW:12572 5' Bases 1 to 786 highly human LOXL1 (Hs.65436) sequence	Hs65436
CN806576	ILLUMIGEN_MCQ_35604 Katze_MMPL1 Macaca mulatta cDNA clone IBIUW:15312 5' Bases 1 to 980 highly human LITAF (Hs.76507) sequence	Hs76507

CN648306	ILLUMIGEN_MCQ_30203 Katze_MMPB Macaca mulatta cDNA clone IBIUW:6746 5' Bases 1 to 857 highly human GZMA (Hs.90708) sequence	Hs90708
CO580739	ILLUMIGEN_MCQ_48269 Katze_MMTE Macaca mulatta cDNA clone IBIUW:20526 5' Bases 460 to 845 highly human C1QG (Hs.94953) sequence	Hs94953
NM_181755	hydroxysteroid (11-beta) dehydrogenase 1 (HSD11B1), transcript variant 2	HSD11B1
NM_017510	gp25L2 protein (HSGP25L2G)	HSGP25L2G
NM_014187	HSPC171 protein (HSPC171).	HSPC171
NM_016209	hematopoietic stem/progenitor cells 176 (HSPC176).	HSPC176
NM_153341	IBR domain containing 3 (IBRDC3).	IBRDC3
NM_000201	intercellular adhesion molecule 1 (CD54), human rhinovirus receptor (ICAM1).	ICAM1
NM_002168	isocitrate dehydrogenase 2 (NADP+), mitochondrial (IDH2)	IDH2
NM_052815	immediate early response 3 (IER3), transcript variant long	IER3
NM_006332	interferon, gamma-inducible protein 30 (IFI30)	IFI30
NM_005533	interferon-induced protein 35 (IFI35).	IFI35
NM_001547	interferon-induced protein with tetratricopeptide repeats 2 (IFIT2).	IFIT2
NM_005534	interferon gamma receptor 2 (interferon gamma transducer 1) (IFNGR2).	IFNGR2
NM_001552	insulin-like growth factor binding protein 4 (IGFBP4)	IGFBP4
NM_001558	interleukin 10 receptor, alpha (IL10RA)	IL10RA
NM_000628	interleukin 10 receptor, beta (IL10RB).	IL10RB
NM_172200	interleukin 15 receptor, alpha (IL15RA), transcript variant 2	IL15RA
NM_004843	interleukin 27 receptor, alpha (IL27RA).	IL27RA
NM_172374	interleukin 4 induced 1 (IL4I1), transcript variant 2	IL4I1
NM_000418	interleukin 4 receptor (IL4R), transcript variant 1.	IL4R
NM_002164	indoleamine-pyrrole 2,3 dioxygenase (INDO).	INDO
NM_002198	interferon regulatory factor 1 (IRF1).	IRF1
NM_001572	interferon regulatory factor 7 (IRF7), transcript variant a.	IRF7
NM_002163	interferon regulatory factor 8 (IRF8)	IRF8
NM_002201	interferon stimulated gene 20kDa (ISG20)	ISG20
NM_002205	integrin, alpha 5 (fibronectin receptor, alpha polypeptide) (ITGA5)	ITGA5
XR_012415	Macaca mulatta integrin, beta 2 (ITGB2)	ITGB2
NM_152713	integral membrane protein 1 (ITM1).	ITM1
NM_004972	Janus kinase 2 (a protein tyrosine kinase) (JAK2).	JAK2
NM_080671	potassium voltage-gated channel, Isk-related family, member 4 (KCNE4).	KCNE4
NM_002247	potassium large conductance calcium-activated channel, subfamily M, alpha member 1 (KCNMA1)	KCNMA1

NM_018992	potassium channel tetramerisation domain containing 5 (KCTD5)	KCTD5
XM_093895	KIAA0882 protein (KIAA0882)	KIAA0882
XM_375553	KIAA0963 (KIAA0963)	KIAA0963
NM_020792	KIAA1363 protein (KIAA1363)	KIAA1363
NM_021035	KIAA1404 protein (KIAA1404)	KIAA1404
NM_002275	keratin 15 (KRT15).	KRT15
NM_032857	lactamase, beta (LACTB), nuclear gene encoding mitochondrial protein, transcript variant 1	LACTB
NM_002286	lymphocyte-activation gene 3 (LAG3)	LAG3
NM_005561	lysosomal-associated membrane protein 1 (LAMP1)	LAMP1
NM_015907	leucine aminopeptidase 3 (LAP3)	LAP3
NM_203463	LAG1 longevity assurance homolog 6 (S. cerevisiae) (LASS6).	LASS6
NM_005564	lipocalin 2 (oncogene 24p3) (LCN2).	LCN2
NM_005565	lymphocyte cytosolic protein 2 (SH2 domain containing leukocyte protein of 76kDa) (LCP2).	LCP2
NM_005566	lactate dehydrogenase A (LDHA).	LDHA
NM_002306	lectin, galactoside-binding, soluble, 3 (galectin 3) (LGALS3).	LGALS3
NM_009587	lectin, galactoside-binding, soluble, 9 (galectin 9) (LGALS9), transcript variant long	LGALS9
NM_005779	lipoma HMGIC fusion partner-like 2 (LHFPL2)	LHFPL2
NM_005874	leukocyte immunoglobulin-like receptor, subfamily B (with TM and ITIM domains), member 2 (LILRB2).	LILRB2
NM_002314	LIM domain kinase 1 (LIMK1), transcript variant 1	LIMK1
NM_004862	lipopolysaccharide-induced TNF factor (LITAF)	LITAF
NM_001002836	hypothetical protein LOC126208 (LOC126208).	LOC126208
NM_145266	RIKEN cDNA 2700047N05 (LOC134492)	LOC134492
NM_207322	hypothetical LOC145741 (LOC145741)	LOC145741
NM_001012754	RIKEN cDNA 8030451K01 (LOC387921), transcript variant 1.	LOC387921
NM_203434	RIKEN cDNA 2610524G09 (LOC389792)	LOC389792
XM_375224	cervical cancer suppressor-1 (LOC400410)	LOC400410
XM_376876	coiled-coil-helix-coiled-coil-helix domain containing 2; 16.7kD protein; chromosome 7 open reading frame 17 (LOC401531)	LOC401531
XM_496386	Fc gamma receptor type I (LOC440607)	LOC440607
XM_496823	RIKEN cDNA A630077B13 gene; RIKEN cDNA 2810048G17 (LOC441168)	LOC441168
NM_022733	hypothetical protein AL133206 (LOC64744)	LOC64744
XR_010082	Macaca mulatta neutrophil cytosolic factor 4 (40kD) isoform 1 (LOC695612)	LOC695612
XR_011279	Macaca mulatta epithelial stromal interaction 1 isoform 1 (LOC700208)	LOC700208
XR_011133	Macaca mulatta hypothetical protein LOC700625 (LOC700625)	LOC700625

XR_012351	Macaca mulatta riboflavin kinase (LOC704540)	LOC704540
XR_012726	Macaca mulatta ubiquitin-conjugating enzyme E2L 6 isoform 1 (LOC705561)	LOC705561
XR_012665	Macaca mulatta cytoplasmic beta-actin (LOC705671)	LOC705671
XR_013774	Macaca mulatta hematopoietic protein 1 (LOC705782)	LOC705782
XR_013529	Macaca mulatta golgi apparatus protein 1 (LOC710037)	LOC710037
XR_012394	Macaca mulatta putative small membrane protein NID67 (LOC711300)	LOC711300
XR_012476	Macaca mulatta hypothetical protein LOC711693 (LOC711693)	LOC711693
XR_012634	Macaca mulatta hypothetical protein LOC712466 (LOC712466)	LOC712466
XR_014226	Macaca mulatta lactotransferrin (LOC713115)	LOC713115
XR_013476	Macaca mulatta procollagen-lysine, 2-oxoglutarate 5-dioxygenase 3 precursor (LOC714283)	LOC714283
XR_013743	Macaca mulatta SH2 containing inositol phosphatase isoform b (LOC717832)	LOC717832
XR_014088	Macaca mulatta glucocerebrosidase precursor (LOC719103)	LOC719103
XR_014443	Macaca mulatta hydroxysteroid (17-beta) dehydrogenase 7 (LOC720399)	LOC720399
XR_014707	Macaca mulatta Complement C1r subcomponent precursor (Complement component 1, r subcomponent) (LOC722131)	LOC722131
XR_014800	Macaca mulatta lysyl hydroxylase precursor (LOC722763)	LOC722763
NM_002318	lysyl oxidase-like 2 (LOXL2)	LOXL2
NM_004811	leupaxin (LPXN).	LPXN
NM_130830	leucine rich repeat containing 15 (LRRC15).	LRRC15
NM_145256	leucine rich repeat containing 25 (LRRC25).	LRRC25
NM_002341	lymphotoxin beta (TNF superfamily, member 3) (LTB), transcript variant 1	LTB
NM_002345	lumican (LUM)	LUM
NM_020169	latexin (LXN)	LXN
NM_002346	lymphocyte antigen 6 complex, locus E (LY6E).	LY6E
NM_004271	lymphocyte antigen 86 (LY86)	LY86
NM_015364	lymphocyte antigen 96 (LY96)	LY96
NM_002350	v-yes-1 Yamaguchi sarcoma viral related oncogene homolog (LYN).	LYN
NM_002755	mitogen-activated protein kinase kinase 1 (MAP2K1).	MAP2K1
NM_021970	mitogen-activated protein kinase kinase 1 interacting protein 1 (MAP2K1IP1)	MAP2K1IP1
NM_002745	mitogen-activated protein kinase 1 (MAPK1), transcript variant 1.	MAPK1
NM_002356	myristoylated alanine-rich protein kinase C substrate (MARCKS)	MARCKS

NM_182796	methionine adenosyltransferase II, beta (MAT2B), transcript variant 2	MAT2B
XR_014169	Macaca mulatta minichromosome maintenance deficient protein 5 (MCM5)	MCM5
NM_001004431	meteorin, glial cell differentiation regulator-like (METRNL).	METRNL
NM_002403	microfibrillar-associated protein 2 (MFAP2), transcript variant 2.	MFAP2
NM_022736	major facilitator superfamily domain containing 1 (MFSD1).	MFSD1
NM_002406	mannosyl (alpha-1,3-)-glycoprotein beta-1,2-N-acetylglucosaminyltransferase (MGAT1).	MGAT1
NM_032350	hypothetical protein MGC11257 (MGC11257).	MGC11257
NM_001001437	chemokine (C-C motif) ligand 3-like, centromeric (MGC12815)	MGC12815
NM_031465	hypothetical protein MGC13204 (MGC13204).	MGC13204
NM_032369	hypothetical protein MGC15619 (MGC15619).	MGC15619
NM_182565	hypothetical protein MGC29814 (MGC29814)	MGC29814
NM_144580	kidney predominant protein NCU-G1 (MGC31963)	MGC31963
NM_152346	hypothetical protein MGC34680 (MGC34680)	MGC34680
NM_016466	hypothetical protein MGC41816 (MGC41816)	MGC41816
NM_052871	hypothetical protein MGC4677 (MGC4677)	MGC4677
NM_145058	hypothetical protein MGC7036 (MGC7036)	MGC7036
NM_023009	MARCKS-like protein (MLP)	MLP
NM_002421	matrix metalloproteinase 1 (interstitial collagenase) (MMP1).	MMP1
NM_004530	matrix metalloproteinase 2 (gelatinase A, 72kDa gelatinase, 72kDa type IV collagenase) (MMP2)	MMP2
NM_022468	matrix metalloproteinase 25 (MMP25), transcript variant 1	MMP25
NM_004994	matrix metalloproteinase 9 (gelatinase B, 92kDa gelatinase, 92kDa type IV collagenase) (MMP9).	MMP9
NM_002436	membrane protein, palmitoylated 1, 55kDa (MPP1)	MPP1
NM_015488	myofibrillogenesis regulator 1 (MR-1)	MR-1
NM_002949	mitochondrial ribosomal protein L12 (MRPL12), nuclear gene encoding mitochondrial protein	MRPL12
NM_023937	mitochondrial ribosomal protein L34 (MRPL34), nuclear gene encoding mitochondrial protein.	MRPL34
NM_032477	mitochondrial ribosomal protein L41 (MRPL41), nuclear gene encoding mitochondrial protein	MRPL41
NM_018141	mitochondrial ribosomal protein S10 (MRPS10), nuclear gene encoding mitochondrial protein.	MRPS10
NM_021107	mitochondrial ribosomal protein S12 (MRPS12), nuclear gene encoding mitochondrial protein, transcript variant 1	MRPS12
NM_015971	mitochondrial ribosomal protein S7 (MRPS7), nuclear gene encoding mitochondrial protein.	MRPS7

NM_152851	membrane-spanning 4-domains, subfamily A, member 6A (MS4A6A), transcript variant 3.	MS4A6A
NM_012331	methionine sulfoxide reductase A (MSRA)	MSRA
NM_175617	metallothionein 1E (functional) (MT1E)	MT1E
NM_005949	metallothionein 1F (functional) (MT1F)	MT1F
NM_032935	metallothionein IV (MT4).	MT4
NM_015440	methylenetetrahydrofolate dehydrogenase (NADP+ dependent) 1-like (MTHFD1L).	MTHFD1L
NM_006636	methylenetetrahydrofolate dehydrogenase (NADP+ dependent) 2, methenyltetrahydrofolate cyclohydrolase (MTHFD2), nuclear gene encoding mitochondrial protein.	MTHFD2
NM_016498	mitochondrial protein 18 kDa (MTP18)	MTP18
NM_017458	major vault protein (MVP), transcript variant 1	MVP
NM_002466	v-myb myeloblastosis viral oncogene homolog (avian)-like 2 (MYBL2).	MYBL2
NM_018946	N-acetylneuraminic acid synthase (sialic acid synthase) (NANS)	NANS
NM_005437	nuclear receptor coactivator 4 (NCOA4).	NCOA4
NM_002488	NADH dehydrogenase (ubiquinone) 1 alpha subcomplex, 2, 8kDa (NDUFA2)	NDUFA2
NM_021075	NADH dehydrogenase (ubiquinone) flavoprotein 3, 10kDa (NDUFV3)	NDUFV3
NM_006164	nuclear factor (erythroid-derived 2)-like 2 (NFE2L2).	NFE2L2
NM_004289	nuclear factor (erythroid-derived 2)-like 3 (NFE2L3).	NFE2L3
NM_004556	nuclear factor of kappa light polypeptide gene enhancer in B-cells inhibitor, epsilon (NFKBIE).	NFKBIE
NM_004148	ninjurin 1 (NINJ1).	NINJ1
NM_001008860	non imprinted in Prader-Willi/Angelman syndrome 2 (NIPA2), transcript variant 2.	NIPA2
XR_013712	Macaca mulatta Alpha-enolase (2-phospho-D-glycerate hydro-lyase) (Non-neural enolase) (NNE) (Enolase 1) (LOC717728)	NNE
NM_014062	nin one binding protein (NOB1P).	NOB1P
NM_004741	nucleolar and coiled-body phosphoprotein 1 (NOLC1)	NOLC1
NM_005693	nuclear receptor subfamily 1, group H, member 3 (NR1H3)	NR1H3
NM_016816	2',5'-oligoadenylate synthetase 1, 40/46kDa (OAS1), transcript variant E18.	OAS1
NM_015878	ornithine decarboxylase antizyme inhibitor (OAZIN), transcript variant 1	OAZIN
NM_013370	pregnancy-induced growth inhibitor (OKL38)	OKL38
NM_148962	oxoeicosanoid (OXE) receptor 1 (OXER1)	OXER1
NM_000632	integrin, alpha M (complement component receptor 3, alpha; also known as CD11b (p170), macrophage antigen alpha polypeptide) (ITGAM).	p170

NM_002560	purinergic receptor P2X, ligand-gated ion channel, 4 (P2RX4), transcript variant 1.	P2RX4
NM_002561	purinergic receptor P2X, ligand-gated ion channel, 5 (P2RX5), transcript variant 1.	P2RX5
NM_176798	pyrimidinergic receptor P2Y, G-protein coupled, 6 (P2RY6), transcript variant 2	P2RY6
NM_000917	procollagen-proline, 2-oxoglutarate 4-dioxygenase (proline 4-hydroxylase), alpha polypeptide I (P4HA1)	P4HA1
NM_002568	poly(A) binding protein, cytoplasmic 1 (PABPC1)	PABPC1
NM_152911	polyamine oxidase (exo-N4-amino) (PAOX), transcript variant 1.	PAOX
NM_001003828	parvin, beta (PARVB), transcript variant 1.	PARVB
NM_022141	parvin, gamma (PARVG)	PARVG
NM_002593	procollagen C-endopeptidase enhancer (PCOLCE)	PCOLCE
NM_002598	programmed cell death 2 (PDCD2), transcript variant 1	PDCD2
NM_213636	PDZ and LIM domain 7 (enigma) (PDLIM7), transcript variant 4	PDLIM7
NM_002620	platelet factor 4 variant 1 (PF4V1).	PF4V1
NM_002627	phosphofructokinase, platelet (PFKP).	PFKP
NM_002629	phosphoglycerate mutase 1 (brain) (PGAM1).	PGAM1
NM_002631	phosphogluconate dehydrogenase (PGD).	PGD
NM_012088	6-phosphogluconolactonase (PGLS)	PGLS
NM_013439	paired immunoglobin-like type 2 receptor alpha (PILRA), transcript variant 1.	PILRA
NM_015900	phospholipase A1 member A (PLA1A).	PLA1A
NM_003706	phospholipase A2, group IVC (cytosolic, calcium-independent) (PLA2G4C).	PLA2G4C
NM_000930	plasminogen activator, tissue (PLAT), transcript variant 1.	PLAT
NM_002658	plasminogen activator, urokinase (PLAU).	PLAU
NM_002661	phospholipase C, gamma 2 (phosphatidylinositol-specific) (PLCG2).	PLCG2
NM_002664	pleckstrin (PLEK).	PLEK
NM_017958	pleckstrin homology domain containing, family B (evectins) member 2 (PLEKHB2)	PLEKHB2
NM_000303	phosphomannomutase 2 (PMM2).	PMM2
NM_021173	polymerase (DNA-directed), delta 4 (POLD4)	POLD4
NM_006627	processing of precursor 4, ribonuclease P/MRP subunit (S. cerevisiae) (POP4).	POP4
NM_006475	periostin, osteoblast specific factor (POSTN).	POSTN
NM_006235	POU domain, class 2, associating factor 1 (POU2AF1).	POU2AF1
NM_021129	pyrophosphatase (inorganic) (PP)	PP
NM_005729	peptidylprolyl isomerase F (cyclophilin F) (PPIF), nuclear gene encoding mitochondrial protein	PPIF
NM_000310	palmitoyl-protein thioesterase 1 (ceroid-lipofuscinosis, neuronal 1, infantile) (PPT1).	PPT1

NM_199418	prolylcarboxypeptidase (angiotensinase C) (PRCP), transcript variant 2	PRCP
NM_001198	PR domain containing 1, with ZNF domain (PRDM1), transcript variant 1.	PRDM1
NM_006793	peroxiredoxin 3 (PRDX3), nuclear gene encoding mitochondrial protein, transcript variant 1.	PRDX3
NM_002727	proteoglycan 1, secretory granule (PRG1).	PRG1
NM_006404	protein C receptor, endothelial (EPCR) (PROCR).	PROCR
NM_016307	paired related homeobox 2 (PRRX2)	PRRX2
NM_002775	protease, serine, 11 (IGF binding) (PRSS11)	PRSS11
NM_148976	proteasome (prosome, macropain) subunit, alpha type, 1 (PSMA1), transcript variant 1	PSMA1
NM_002791	proteasome (prosome, macropain) subunit, alpha type, 6 (PSMA6)	PSMA6
NM_002801	proteasome (prosome, macropain) subunit, beta type, 10 (PSMB10).	PSMB10
NM_004159	proteasome (prosome, macropain) subunit, beta type, 8 (large multifunctional protease 7) (PSMB8), transcript variant 1	PSMB8
NM_002800	proteasome (prosome, macropain) subunit, beta type, 9 (large multifunctional protease 2) (PSMB9), transcript variant 1	PSMB9
NM_002803	proteasome (prosome, macropain) 26S subunit, ATPase, 2 (PSMC2)	PSMC2
NM_000958	prostaglandin E receptor 4 (subtype EP4) (PTGER4).	PTGER4
NM_007284	PTK9L protein tyrosine kinase 9-like (A6-related protein) (PTK9L)	PTK9L
NM_080588	protein tyrosine phosphatase, non-receptor type 7 (PTPN7), transcript variant 2	PTPN7
NM_080792	protein tyrosine phosphatase, non-receptor type substrate 1 (PTPNS1).	PTPNS1
NM_013237	px19-like protein (PX19)	PX19
NM_013258	PYD and CARD domain containing (PYCARD), transcript variant 1.	PYCARD
NM_017817	RAB20, member RAS oncogene family (RAB20).	RAB20
NM_006868	RAB31, member RAS oncogene family (RAB31).	RAB31
NM_006834	RAB32, member RAS oncogene family (RAB32).	RAB32
NM_032023	Ras association (RalGDS/AF-6) domain family 4 (RASSF4), transcript variant 1.	RASSF4
NM_006509	v-rel reticuloendotheliosis viral oncogene homolog B, nuclear factor of kappa light polypeptide gene enhancer in B-cells 3 (avian) (RELB)	RELB
NM_002910	renin binding protein (RENBP)	RENBP
NM_002922	regulator of G-protein signalling 1 (RGS1).	RGS1
NM_002928	regulator of G-protein signalling 16 (RGS16)	RGS16
NM_005873	regulator of G-protein signalling 19 (RGS19)	RGS19

NM_001005498	rhomboid, veinlet-like 6 (Drosophila) (RHBDL6), transcript variant 2.	RHBDL6
NM_024599	rhomboid, veinlet-like 6 (Drosophila) (RHBDL6)	RHBDL6
NM_001664	ras homolog gene family, member A (RHOA)	RHOA
NM_004310	ras homolog gene family, member H (RHOH).	RHOH
NM_021205	ras homolog gene family, member U (RHOU).	RHOU
NM_003821	receptor-interacting serine-threonine kinase 2 (RIPK2).	RIPK2
NM_198232	ribonuclease, RNase A family, 1 (pancreatic) (RNASE1), transcript variant 3	RNASE1
NM_002935	ribonuclease, RNase A family, 3 (eosinophil cationic protein) (RNASE3).	RNASE3
NM_005615	ribonuclease, RNase A family, k6 (RNASE6).	RNASE6
NM_018434	ring finger protein 130 (RNF130)	RNF130
NM_003942	ribosomal protein S6 kinase, 90kDa, polypeptide 4 (RPS6KA4)	RPS6KA4
NM_080388	S100 calcium binding protein A16 (S100A16).	S100A16
NM_005980	S100 calcium binding protein P (S100P).	S100P
NM_000331	serum amyloid A1 (SAA1), transcript variant 1.	SAA1
NM_003864	sin3-associated polypeptide, 30kDa (SAP30)	SAP30
NM_054023	secretoglobin, family 3A, member 2 (SCGB3A2).	SCGB3A2
NM_002975	stem cell growth factor; lymphocyte secreted C-type lectin (SCGF)	SCGF
NM_001037	sodium channel, voltage-gated, type I, beta (SCN1B), transcript variant a	SCN1B
NM_001007067	syndecan binding protein (syntenin) (SDCBP), transcript variant 2.	SDCBP
NM_022044	stromal cell-derived factor 2-like 1 (SDF2L1)	SDF2L1
NM_003002	succinate dehydrogenase complex, subunit D, integral membrane protein (SDHD), nuclear gene encoding mitochondrial protein.	SDHD
NM_004892	SEC22 vesicle trafficking protein-like 1 (S. cerevisiae) (SEC22L1)	SEC22L1
NM_013336	Sec61 alpha 1 subunit (S. cerevisiae) (SEC61A1)	SEC61A1
NM_006808	Sec61 beta subunit (SEC61B)	SEC61B
NM_001012456	Sec61 gamma subunit (SEC61G), transcript variant 2.	SEC61G
NM_003004	secreted and transmembrane 1 (SECTM1).	SECTM1
NM_003006	selectin P ligand (SELPLG).	SELPLG
NM_018445	selenoprotein S (SELS), transcript variant 2	SELS
NM_022367	sema domain, immunoglobulin domain (Ig), transmembrane domain (TM) and short cytoplasmic domain, (semaphorin) 4A (SEMA4A).	SEMA4A
NM_017789	sema domain, immunoglobulin domain (Ig), transmembrane domain (TM) and short cytoplasmic domain, (semaphorin) 4C (SEMA4C).	SEMA4C

NM_006378	sema domain, immunoglobulin domain (Ig), transmembrane domain (TM) and short cytoplasmic domain, (semaphorin) 4D (SEMA4D)	SEMA4D
NM_000295	serine (or cysteine) proteinase inhibitor, clade A (alpha-1 antiproteinase, antitrypsin), member 1 (SERPINA1), transcript variant 1.	SERPINA1
NM_001085	serine (or cysteine) proteinase inhibitor, clade A (alpha-1 antiproteinase, antitrypsin), member 3 (SERPINA3).	SERPINA3
NM_030666	serine (or cysteine) proteinase inhibitor, clade B (ovalbumin), member 1 (SERPINB1).	SERPINB1
NM_000602	serine (or cysteine) proteinase inhibitor, clade E (nexin, plasminogen activator inhibitor type 1), member 1 (SERPINE1)	SERPINE1
NM_000934	serine (or cysteine) proteinase inhibitor, clade F (alpha-2 antiplasmin, pigment epithelium derived factor), member 2 (SERPINF2)	SERPINF2
NM_001235	serine (or cysteine) proteinase inhibitor, clade H (heat shock protein 47), member 1, (collagen binding protein 1) (SERPINH1).	SERPINH1
NM_013376	SERTA domain containing 1 (SERTAD1).	SERTAD1
NM_003901	sphingosine-1-phosphate lyase 1 (SGPL1)	SGPL1
NM_031286	SH3 domain binding glutamic acid-rich protein like 3 (SH3BGRL3)	SH3BGRL3
NM_004844	SH3-domain binding protein 5 (BTK-associated) (SH3BP5)	SH3BP5
NM_005067	seven in absentia homolog 2 (Drosophila) (SIAH2)	SIAH2
NM_003037	signaling lymphocytic activation molecule family member 1 (SLAMF1)	SLAMF1
NM_020125	SLAM family member 8 (SLAMF8).	SLAMF8
NM_016582	solute carrier family 15, member 3 (SLC15A3)	SLC15A3
NM_017585	solute carrier family 2 (facilitated glucose transporter), member 6 (SLC2A6)	SLC2A6
NM_153811	solute carrier family 38, member 6 (SLC38A6)	SLC38A6
NM_014096	solute carrier family 43, member 3 (SLC43A3).	SLC43A3
NM_003486	solute carrier family 7 (cationic amino acid transporter, y+ system), member 5 (SLC7A5).	SLC7A5
NM_016354	solute carrier organic anion transporter family, member 4A1 (SLCO4A1).	SLCO4A1
NM_138440	slit-like 2 (Drosophila) (SLITL2).	SLITL2
NM_000543	sphingomyelin phosphodiesterase 1, acid lysosomal (acid sphingomyelinase) (SMPD1), transcript variant 1.	SMPD1
NM_014390	staphylococcal nuclease domain containing 1 (SND1)	SND1
NM_013322	sorting nexin 10 (SNX10).	SNX10
NM_003745	suppressor of cytokine signaling 1 (SOCS1).	SOCS1

NM_000582	secreted phosphoprotein 1 (osteopontin, bone sialoprotein I, early T-lymphocyte activation 1) (SPP1)	SPP1
NM_021199	sulfide quinone reductase-like (yeast) (SQRDL)	SQRDL
NM_021203	signal recognition particle receptor, B subunit (SRPRB)	SRPRB
NM_016305	synovial sarcoma translocation gene on chromosome 18-like 2 (SS18L2)	SS18L2
NM_006396	Sjogren's syndrome/scleroderma autoantigen 1 (SSSCA1)	SSSCA1
NM_139266	signal transducer and activator of transcription 1, 91kDa (STAT1), transcript variant beta	STAT1
NM_003764	syntaxin 11 (STX11).	STX11
NM_177424	syntaxin 12 (STX12).	STX12
NM_033050	succinate receptor 1 (SUCNR1).	SUCNR1
NM_033161	surfeit 4 (SURF4)	SURF4
NM_003177	spleen tyrosine kinase (SYK)	SYK
NM_205848	synaptotagmin VI (SYT6)	SYT6
NM_006755	transaldolase 1 (TALDO1)	TALDO1
NM_000593	transporter 1, ATP-binding cassette, sub-family B (MDR/TAP) (TAP1).	TAP1
NM_014604	Tax1 (human T-cell leukemia virus type I) binding protein 3 (TAX1BP3)	TAX1BP3
NM_006019	T-cell, immune regulator 1, ATPase, H+ transporting, lysosomal V0 protein a isoform 3 (TCIRG1), transcript variant 1	TCIRG1
NM_001063	transferrin (TF)	TF
NM_012252	transcription factor EC (TFEC), transcript variant 1.	TFEC
NM_000660	transforming growth factor, beta 1 (Camurati-Engelmann disease) (TGFB1)	TGFB1
XR_009902	Macaca mulatta Trans-Golgi network integral membrane protein 2 precursor (Trans-Golgi network protein TGN51) (TGN46) (TGN48) (TGN38 homolog) (LOC694942)	TGN46
NM_003246	thrombospondin 1 (THBS1).	THBS1
NM_012456	translocase of inner mitochondrial membrane 10 homolog (yeast) (TIMM10)	TIMM10
NM_003254	tissue inhibitor of metalloproteinase 1 (erythroid potentiating activity, collagenase inhibitor) (TIMP1)	TIMP1
NM_003258	thymidine kinase 1, soluble (TK1).	TK1
NM_014220	transmembrane 4 L six family member 1 (TM4SF1).	TM4SF1
NM_003272	transmembrane 7 superfamily member 1 (upregulated in kidney) (TM7SF1).	TM7SF1
NM_014313	transmembrane protein 50A (TMEM50A).	TMEM50A
NM_018022	transmembrane protein 51 (TMEM51).	TMEM51
XR_000287	Tmp21-II , transcribed pseudogene (Tmp21-II), misc RNA	Tmp21-II

NM_001065	tumor necrosis factor receptor superfamily, member 1A (TNFRSF1A).	TNFRSF1A
NM_003327	tumor necrosis factor receptor superfamily, member 4 (TNFRSF4)	TNFRSF4
NM_001243	tumor necrosis factor receptor superfamily, member 8 (TNFRSF8), transcript variant 1	TNFRSF8
NM_006573	tumor necrosis factor (ligand) superfamily, member 13b (TNFSF13B).	TNFSF13B
NM_003282	troponin I, skeletal, fast (TNNI2)	TNNI2
XR_011552	Macaca mulatta Triosephosphate isomerase (TIM) (Triose-phosphate isomerase) (TPI1)	TPI1
NM_012112	TPX2, microtubule-associated, homolog (Xenopus laevis) (TPX2).	TPX2
NM_006700	TRAF-type zinc finger domain containing 1 (TRAFD1).	TRAFD1
NM_018965	triggering receptor expressed on myeloid cells 2 (TREM2).	TREM2
NM_016381	three prime repair exonuclease 1 (TREX1), transcript variant 1	TREX1
NM_014788	tripartite motif-containing 14 (TRIM14), transcript variant 1.	TRIM14
NM_001001188	transient receptor potential cation channel, subfamily M, member 2 (TRPM2), transcript variant S.	TRPM2
NM_005726	Ts translation elongation factor, mitochondrial (TSFM)	TSFM
NM_003330	thioredoxin reductase 1 (TXNRD1), transcript variant 1	TXNRD1
NM_006398	ubiquitin D (UBD).	UBD
NM_181803	ubiquitin-conjugating enzyme E2C (UBE2C), transcript variant 6	UBE2C
NM_152653	ubiquitin-conjugating enzyme E2E 2 (UBC4/5 homolog, yeast) (UBE2E2)	UBE2E2
NM_003359	UDP-glucose dehydrogenase (UGDH).	UGDH
NM_006830	ubiquinol-cytochrome c reductase, 6.4kDa subunit (UQCR).	UQCR
NM_178443	UNC-112 related protein 2 (URP2), transcript variant URP2LF.	URP2
NM_001078	vascular cell adhesion molecule 1 (VCAM1), transcript variant 1.	VCAM1
NM_000376	vitamin D (1,25- dihydroxyvitamin D3) receptor (VDR), transcript variant 1.	VDR
NM_016226	vacuolar protein sorting 29 (yeast) (VPS29), transcript variant 1.	VPS29
NM_007268	V-set and immunoglobulin domain containing 4 (VSIG4).	VSIG4
NM_004184	tryptophanyl-tRNA synthetase (WARS), transcript variant 1	WARS
NM_032463	Williams-Beuren syndrome chromosome region 5 (WBSCR5), transcript variant 2	WBSCR5

NM_152858	Wilms tumor 1 associated protein (WTAP), transcript variant 3	WTAP
NM_024096	XTP3-transactivated protein A (XTP3TPA).	XTP3TPA
NM_003405	tyrosine 3-monooxygenase/tryptophan 5-monooxygenase activation protein, eta polypeptide (YWHAH)	YWHAH
NM_004729	zinc finger, BED-type containing 1 (ZBED1).	ZBED1
NM_025079	zinc finger CCCH-type containing 12A (ZC3H12A).	ZC3H12A
NM_032283	zinc finger, DHHC domain containing 18 (ZDHHC18)	ZDHHC18
XR_011110	Macaca mulatta zinc finger, DHHC domain containing 9 (ZDHHC9)	ZDHHC9
NM_006963	zinc finger protein 22 (KOX 15) (ZNF22)	ZNF22
CB229722	AGENCOURT_11500965 NICHD_Rh_Ov1 Macaca mulatta cDNA clone IMAGE:6882443 5' sequence	
CB311553	AGENCOURT_11823216 NICHD_Rh_Ov1 Macaca mulatta cDNA clone IMAGE:6912989 5' sequence	
CB310604	AGENCOURT_11830389 NICHD_Rh_Ov1 Macaca mulatta cDNA clone IMAGE:6916324 5' sequence	
CB310061	AGENCOURT_11876913 NICHD_Rh_Ov1 Macaca mulatta cDNA clone IMAGE:6913844 5' sequence	
AK127395	cDNA FLJ45486 fis, clone BRTHA2002726	
CK230655	ILLUMIGEN_MCQ_1009 Katze_MMPL2 Macaca mulatta cDNA 5' human Unigene Hs.500464 sequence	
CN645791	ILLUMIGEN_MCQ_24884 Katze_MMBR Macaca mulatta cDNA clone IBIUW:10607 5' Bases 254 to 768 highly human Unigene Hs.25892 sequence	
CN648004	ILLUMIGEN_MCQ_29540 Katze_MMPB Macaca mulatta cDNA clone IBIUW:7020 5' Bases 17 to 588 highly human Unigene Hs.435390 sequence	
CN801916	ILLUMIGEN_MCQ_37292 Katze_MMBR Macaca mulatta cDNA clone IBIUW:14420 5' Bases 166 to 772 highly human Unigene Hs.518521 sequence	
CO647394	ILLUMIGEN_MCQ_40429 Katze_MMPB2 Macaca mulatta cDNA clone IBIUW:24175 5' Bases 238 to 766 highly human Unigene Hs.517602 sequence	
CO580643	ILLUMIGEN_MCQ_48393 Katze_MMLV Macaca mulatta cDNA clone IBIUW:18409 5' Bases 755 to 975 highly human Unigene Hs.515465 sequence	
DR774422	ILLUMIGEN_MCQ_58593 Katze_MMLV Macaca mulatta cDNA clone IBIUW:34887 5' Bases 9 to 306 highly human Unigene Hs.529672 sequence	
CO645122	ILLUMIGEN_MCQ_8291 Katze_MMBR Macaca mulatta cDNA clone IBIUW:23018 5' Bases 1 to 553 highly human Unigene Hs.203697 sequence	
XR_010624	Macaca mulatta actin related protein 2	
DQ155428	Macaca mulatta LILRAb mRNA, complete cds	

CB549220	MMPL0027_F12 MMPL Macaca mulatta cDNA sequence
CB553994	MMSP0053_A12 MMSP Macaca mulatta cDNA sequence
CB551618	MMSP0057_A06 MMSP Macaca mulatta cDNA sequence
BM423313	PLATE4_C07 Rhesus Macaca mulatta cDNA sequence
CO646475	TAPBP
A_01_P012330	Unknown
A_01_P005239	Unknown
A_01_P018128	Unknown
NM_138397	Unknown
A_01_P000822	Unknown
AI913343	wa11b04.x1 NCI_CGAP_Kid11 cDNA clone IMAGE:2297743 3' contains Alu repetitive element; sequence

)13

186
