# Supplementary material for: Transcriptional Reprogramming in Nonhuman Primate (Rhesus Macaque) Tuberculosis Granulomas
Source: PLoS One. 2010 Aug 31;5(8):e12266. doi: 10.1371/journal.pone.0012266 (PMC2930844; doi:10.1371/journal.pone.0012266)
Supplement: Table S13 — This table lists genes with a lower expression in early but a higher expression in late genes (i.e. the overlapping genes in Fig. 4D). (0.11 MB PDF) [file pone.0012266.s013.pdf]

| Gene Name | Desccription | Symbol |
|---|---|---|
| NM_181806 | 2-aminoadipic 6-semialdehyde dehydrogenase | NRPS998 |
| NM_004670 | 3'-phosphoadenosine 5'-phosphosulfate synthase 2 | PAPSS2 |
| NM_020201 | 5',3'-nucleotidase, mitochondrial | NT5M |
| NM_203326 | 5-azacytidine induced 2 | AZI2 |
| NM_012229 | 5'-nucleotidase, cytosolic II | NT5C2 |
| NM_017570 | 5-oxoprolinase | OPLAH |
| NM_007037 | a disintegrin-like and metalloprotease | ADAMTS8 |
| NM_139025 | a disintegrin-like and metalloprotease | ADAMTS13 |
| NM_005858 | A kinase | AKAP8 |
| NM_004192 | acetylserotonin O-methyltransferase-like | ASMTL |
| XR_014079 | acid sphingomyelinase-like phosphodiesterase 3B isoform 1 | SMPDL3B |
| NM_006107 | acid-inducible phosphoprotein | OA48-18 |
| NM_032432 | actin binding LIM protein family, member 2 | ABLIM2 |
| NM_014945 | actin binding LIM protein family, member 3 | ABLIM3 |
| NM_004924 | actinin, alpha 4 | ACTN4 |
| NM_001106 | activin A receptor, type IIB | ACVR2B |
| NM_003500 | acyl-Coenzyme A oxidase 2, branched chain | ACOX2 |
| NM_018263 | additional sex combs like 2 | ASXL2 |
| NM_000022 | adenosine deaminase | ADA |
| NM_015833 | adenosine deaminase, RNA-specific, B1 | ADARB1 |
| NM_001136 | advanced glycosylation end product-specific receptor | AGER |
| NM_024060 | AHNAK nucleoprotein | AHNAK |
| NM_000667 | alcohol dehydrogenase 1A | ADH1A |
| NM_000669 | alcohol dehydrogenase 1C | ADH1C |
| NM_144650 | alcohol dehydrogenase, iron containing, 1 | ADHFE1 |
| NM_001354 | aldo-keto reductase family 1, member C2 | AKR1C2 |
| NM_001631 | alkaline phosphatase, intestinal | ALPI |
| NM_024083 | alveolar soft part sarcoma chromosome region, candidate 1 | ASPSCR1 |
| NM_016228 | aminoadipate aminotransferase | AADAT |
| NM_000481 | aminomethyltransferase | AMT |
| NM_001008218 | amylase, alpha 1B; salivary | AMY1B |
| NM_000699 | amylase, alpha 2A; pancreatic | AMY2A |
| NM_020978 | amylase, alpha 2B; pancreatic | AMY2B |
| NM_133175 | amyloid beta | APBB3 |
| NM_013367 | anaphase promoting complex subunit 4 | ANAPC4 |
| XR_011510 | anaphase-promoting complex subunit 5 | ANAPC5 |
| NM_139290 | angiopoietin 1 | ANGPT1 |
| NM_020987 | ankyrin 3, node of Ranvier | ankyrin G |
| NM_015245 | ankyrin repeat and sterile alpha motif domain containing 1 | ANKS1 |
| XR_012049 | ankyrin repeat and sterile alpha motif domain containing 3 | ANKS3 |
| NM_017664 | ankyrin repeat domain 10 | ANKRD10 |

| | | |
|---|---|---|
| NM_020349 | ankyrin repeat domain 2 | ANKRD2 |
| NM_144994 | ankyrin repeat domain 23 | ANKRD23 |
| NM_022096 | ankyrin repeat domain 5 | ANKRD5 |
| NM_014942 | ankyrin repeat domain 6 | ANKRD6 |
| NM_001630 | annexin A8 | ANXA8 |
| NM_005876 | aortic preferentially expressed protein 1 | APEG1 |
| CN646438 | APBB1 | APBB1 |
| NM_014977 | apoptotic chromatin condensation inducer 1 | ACIN1 |
| NM_006321 | ariadne homolog 2 | ARIH2 |
| NM_004313 | arrestin, beta 2 | ARRB2 |
| NM_057091 | artemin | ARTN |
| NM_015205 | ATPase, Class VI, type 11A | ATP11A |
| NM_144583 | ATPase, H+ transporting, lysosomal 42kDa, V1 subunit C isoform 2 | ATP6V1C2 |
| NM_007168 | ATP-binding cassette, sub-family A | ABC1 |
| NM_000352 | ATP-binding cassette, sub-family C | CFTR/MRP |
| NM_005688 | ATP-binding cassette, sub-family C | CFTR/MRP |
| NM_022161 | baculoviral IAP repeat-containing 7 | BIRC7 |
| NM_017451 | BAI1-associated protein 2 | BAIAP2 |
| NM_020926 | BCL6 co-repressor | BCOR |
| NM_001195 | beaded filament structural protein 1, filensin | BFSP1 |
| CN803179 | BEX2 | BEX2 |
| NM_015964 | brain specific protein | CGI-38 |
| NM_001190 | branched chain aminotransferase 2, mitochondrial | BCAT2 |
| NM_001519 | BRF1 homolog, subunit of RNA polymerase III transcription initiation factor IIIB | BRF1 |
| NM_018688 | bridging integrator 3 | BIN3 |
| NM_006696 | bromodomain containing 8 | BRD8 |
| XR_010742 | BTAF1 RNA polymerase II, B-TFIID transcription factor-associated, 170kDa | BTAF1 |
| NM_032156 | C1q domain containing 1 | C1QDC1 |
| NM_018584 | calcium/calmodulin-dependent protein kinase II | CaMKIINalpha |
| NM_000070 | calpain 3, | CAPN3 |
| NM_006615 | calpain 9 | CAPN9 |
| NM_032607 | cAMP responsive element binding protein 3-like 3 | CREB3L3 |
| NM_001266 | carboxylesterase 1 | CES1 |
| NM_080385 | carboxypeptidase A5 | CPA5 |
| NM_016382 | CD244 natural killer cell receptor 2B4 | CD244 |
| NM_001777 | CD47 antigen | CD47 |
| NM_134445 | CD99 antigen-like 2 | CD99L2 |
| NM_004071 | CDC-like kinase 1 | CLK1 |
| NM_001291 | CDC-like kinase 2 | CLK2 |
| NM_016408 | CDK5 regulatory subunit associated protein 1 | CDK5RAP1 |
| AK125739 | cDNA FLJ43751 fis, clone TESTI2034953, moderately 88-kDa Golgi protein | GM88 |
| NM_001263 | CDP-diacylglycerol synthase | CDS1 |
| NM_178569 | CEI protein | CEI |

| | | |
|---|---|---|
| NM_001812 | centromere protein C 1 | CENPC1 |
| NM_018069 | centrosomal protein 192 kDa | Cep192 |
| NM_032205 | CGI-72 protein | CGI-72 |
| NM_148888 | chemokine | C-C motif ligand 25 |
| NM_176875 | cholecystokinin B receptor | CCKBR |
| NM_001277 | choline kinase alpha | CHKA |
| NM_020991 | chorionic somatomammotropin hormone 2 | CSH2 |
| NM_175709 | chromobox homolog 7 | CBX7 |
| NM_017673 | chromosome 1 open reading frame 26 | C1orf26 |
| NM_032709 | chromosome 10 open reading frame 33 | C10orf33 |
| NM_017791 | chromosome 14 open reading frame 58 | C14orf58 |
| NM_032140 | chromosome 16 open reading frame 48 | C16orf48 |
| NM_033520 | chromosome 19 open reading frame 33 | C19orf33 |
| NM_018257 | chromosome 20 open reading frame 36 | C20orf36 |
| NM_022760 | chromosome 20 open reading frame 81 | C20orf81 |
| NM_016589 | chromosome 3 open reading frame 1 | C3orf1 |
| NM_032870 | chromosome 6 open reading frame 111 | C6orf111 |
| NM_025257 | chromosome 6 open reading frame 29 | C6orf29 |
| NM_017633 | chromosome 6 open reading frame 37 | C6orf37 |
| NM_024581 | chromosome 6 open reading frame 60 | C6orf60 |
| NM_015949 | chromosome 7 open reading frame 20 | C7orf20 |
| NM_024315 | chromosome 7 open reading frame 23 | C7orf23 |
| NM_178829 | chromosome 7 open reading frame 34 | C7orf34 |
| NM_032818 | chromosome 9 open reading frame 100 | C9orf100 |
| NM_178448 | chromosome 9 open reading frame 140 | C9orf140 |
| NM_004816 | chromosome 9 open reading frame 61 | C9orf61 |
| NM_020770 | cingulin | CGN |
| NM_001835 | clathrin, heavy polypeptide-like 1 | CLTCL1 |
| NM_014343 | claudin 15 | CLDN15 |
| XR_011108 | cleavage and polyadenylation specific factor 1, 160kDa | CPSF1 |
| NM_032179 | cleavage and polyadenylation specific factor 3-like | CPSF3L |
| NM_016438 | CLST 11240 protein | CLST11240 |
| NM_025103 | coiled-coil domain containing 2 | CCDC2 |
| NM_001844 | collagen, type II, alpha 1 | COL2A1 |
| NM_000092 | collagen, type IV, alpha 4 | COL4A4 |
| NM_000495 | collagen, type IV, alpha 5 | COL4A5 |
| NM_001847 | collagen, type IV, alpha 6 | COL4A6 |
| NM_058175 | collagen, type VI, alpha 2 | COL6A2 |
| NM_030781 | collectin sub-family member 12 | COLEC12 |
| NM_000758 | colony stimulating factor 2 | CSF2 |
| NM_182528 | complement component 1, q subcomponent-like 2 | C1QL2 |
| XR_013572 | Connector enhancer of kinase suppressor of ras 1 | hCNK1 |
| NM_153368 | connexin401 | CX401 |
| NM_153634 | copine VIII | CPNE8 |
| NM_015198 | cordon-bleu homolog | COBL |
| NM_005231 | cortactin | CTTN |

| | | |
|---|---|---|
| NM_001882 | corticotropin releasing hormone binding protein | CRHBP |
| NM_001338 | coxsackie virus and adenovirus receptor | CXADR |
| NM_005808 | CTD | carboxy-terminal domain |
| NM_182905 | CXYorf1-related protein | DKFZp434K1323 |
| NM_198943 | CXYorf1-related protein | MGC52000 |
| NM_020307 | cyclin L1 | CCNL1 |
| XR_013257 | cyclin M1 | CCNM1 |
| NM_003674 | cyclin-dependent kinase | CDK10 |
| NM_001798 | cyclin-dependent kinase 2 | CDK2 |
| NM_001261 | cyclin-dependent kinase 9 | CDK9 |
| NM_001323 | cystatin E/M | CST6 |
| NM_032687 | cysteine and histidine rich 1 | CYHR1 |
| NM_001554 | cysteine-rich, angiogenic inducer, 61 | CYR61 |
| NM_000778 | cytochrome P450, family 4, subfamily A, polypeptide 11 | CYP4A11 |
| NM_000779 | cytochrome P450, family 4, subfamily B, polypeptide 1 | CYP4B1 |
| NM_023944 | cytochrome P450, family 4, subfamily F, polypeptide 12 | CYP4F12 |
| NM_014376 | cytoplasmic FMR1 interacting protein 2 | CYFIP2 |
| NM_001928 | D component of complement | DF |
| NM_000107 | damage-specific DNA binding protein 2, 48kDa | DDB2 |
| NM_004396 | DEAD | DDX5 |
| NM_006386 | DEAD | DDX17 |
| NM_015404 | deafness, autosomal recessive 31 | DFNB31 |
| NM_022105 | death associated transcription factor 1 | DATF1 |
| NM_001920 | decorin | DCN |
| NM_020812 | dedicator of cytokinesis 6 | DOCK6 |
| NM_015296 | dedicator of cytokinesis 9 | DOCK9 |
| NM_005106 | deleted in lung and esophageal cancer 1 | DLEC1 |
| NM_178502 | deltex 3 homolog | DTX3 |
| NM_001937 | dermatopontin | DPT |
| NM_001943 | desmoglein 2 | DSG2 |
| NM_015881 | dickkopf homolog 3 | DKK3 |
| NM_181870 | dishevelled, dsh homolog 1 | DVL1 |
| NM_032890 | dispatched homolog 1 | DISP1 |
| NM_198489 | DLNB14 | DLNB14 |
| NM_022160 | DMRT-like family A1 | DMRTA1 |
| NM_015190 | DnaJ | DNAJC9 |
| NM_033105 | DnaJ | DNAJC5B |
| NM_017613 | downstream neighbor of SON | DONSON |
| CN646916 | DSCR6 | DSCR6 |
| NM_024025 | dual specificity phosphatase 26 | DUSP26 |
| NM_015548 | dystonin | DST |
| NM_183380 | dystonin | DST |
| NM_004424 | E4F transcription factor 1 | E4F1 |
| NM_201446 | EGF-like-domain, multiple 7 | EGFL7 |

| | | |
|---|---|---|
| NM_022821 | elongation of very long chain fatty acids | ELOVL1 |
| NM_016337 | Enah/Vasp-like | EVL |
| XM_370946 | endoplasmic reticulum | LOC388226 |
| NM_004826 | endothelin converting enzyme-like 1 | ECEL1 |
| NM_001991 | enhancer of zeste homolog 1 | EZH1 |
| NM_001976 | enolase 3, | ENO3 |
| CO582652 | ENPEP | ENPEP |
| NM_005232 | EphA1 | EPHA1 |
| NM_004445 | EphB6 | EPHB6 |
| NM_004428 | ephrin-A1 | EFNA1 |
| NM_001979 | epoxide hydrolase 2, cytoplasmic | EPHX2 |
| NM_133180 | EPS8-like 1 | EPS8L1 |
| NM_014964 | epsin 2 | EPN2 |
| NM_019002 | ETAA16 protein | ETAA16 |
| NM_004454 | ets variant gene 5 | ETV5 |
| NM_024757 | euchromatic histone methyltransferase 1 | EHMT1 |
| NM_001967 | eukaryotic translation initiation factor 4A, isoform 2 | EIF4A2 |
| XR_013017 | exosome component 7 | EXOSC7 |
| NM_005244 | eyes absent homolog 2 | EYA2 |
| NM_145249 | family with sequence similarity 14, member B | FAM14B |
| NM_024898 | family with sequence similarity 31, member C | FAM31C |
| NM_021727 | fatty acid desaturase 3 | FADS3 |
| NM_031456 | F-box and WD-40 domain protein 10 | FBXW10 |
| XR_013084 | F-box and WD-40 domain protein 7, archipelago homolog | FBXW7 |
| CN802687 | FBXW7 | FBXW7 |
| NM_032843 | fibrinogen C domain containing 1 | FIBCD1 |
| NM_023028 | fibroblast growth factor receptor 2 | FGFR2 |
| NM_213647 | fibroblast growth factor receptor 4 | FGFR4 |
| NM_173651 | fibrous sheath interacting protein 2 | FSIP2 |
| NM_001996 | fibulin 1 | FBLN1 |
| NM_006329 | fibulin 5 | FBLN5 |
| DR771278 | FLJ14525 | FLJ14525 |
| NM_147195 | FLJ35740 protein | FLJ35740 |
| NM_207442 | FLJ39779 protein | FLJ39779 |
| NM_207414 | FLJ43860 protein | FLJ43860 |
| NM_207458 | FLJ46026 protein | FLJ46026 |
| NM_001454 | forkhead box J1 | FOXJ1 |
| NM_015308 | formin binding protein 4 | FNBP4 |
| NM_013241 | formin homology 2 domain containing 1 | FHOD1 |
| NM_000507 | fructose-1,6-bisphosphatase 1 | FBP1 |
| NM_145059 | fucokinase | FUK |
| CR604926 | full-length cDNA clone CS0DF038YH05 of Fetal brain of | full-length cDNA clone CS0DF038Y |
| NM_006625 | FUS interacting protein | FUSIP1 |
| NM_025211 | G kinase anchoring protein 1 | GKAP1 |
| NM_018025 | G patch domain containing 1 | GPATC1 |

| | | |
|---|---|---|
| NM_032442 | G protein pathway suppressor 2 | GPS2 |
| NM_024980 | G protein-coupled receptor 157 | GPR157 |
| NM_182982 | G protein-coupled receptor kinase 4 | GRK4 |
| NM_022036 | G protein-coupled receptor, family C, group 5, member C | GPRC5C |
| NM_001470 | gamma-aminobutyric acid | GABBR1 |
| NM_002043 | gamma-aminobutyric acid | GABRR2 |
| NM_021990 | gamma-aminobutyric acid | GABRE |
| NM_006783 | gap junction protein, beta 6 | GJB6 |
| NM_000805 | gastrin | GAST |
| NM_032638 | GATA binding protein 2 | GATA2 |
| NM_021167 | GATA zinc finger domain containing 1 | GATAD1 |
| AB038463 | GC36 mRNA, complete cds | GC36 |
| NM_021078 | GCN5 general control of amino-acid synthesis 5-like 2 | GCN5L2 |
| NM_019858 | gene rich cluster, A gene | GRCA |
| XR_000285 | general transcription factor II, i, pseudogene 1 | GTF2IP1 |
| NM_014905 | glutaminase | GLS |
| NM_000847 | glutathione S-transferase A3 | GSTA3 |
| NM_000853 | glutathione S-transferase theta 1 | GSTT1 |
| NM_024307 | glycerophosphodiester phosphodiesterase domain containing 3 | GDPD3 |
| NM_001001995 | glycoprotein M6B | GPM6B |
| NM_152312 | glycosyltransferase-like 1B | GYLTL1B |
| NM_004484 | glypican 3 | GPC3 |
| NM_138619 | golgi associated, gamma adaptin ear containing, ARF binding protein 3 | GGA3 |
| NM_002077 | golgi autoantigen, golgin subfamily a, 1 | GOLGA1 |
| NM_001501 | gonadotropin-releasing hormone 2 | GNRH2 |
| NM_032293 | GTPase activating Rap/RanGAP domain-like 3 | GARNL3 |
| NM_007102 | guanylate cyclase activator 2B | GUCA2B |
| NM_015726 | H326 | H326 |
| CN801728 | HBA2 | HBA2 |
| NM_016173 | HemK methyltransferase family member 1 | HEMK1 |
| NM_000518 | hemoglobin, beta | HBB |
| NM_001945 | heparin-binding EGF-like growth factor | HBEGF |
| NM_182983 | hepsin | HPN |
| NM_005520 | heterogeneous nuclear ribonucleoprotein H1 | HNRPH1 |
| XR_010398 | histidyl-tRNA synthetase-like | HARSL |
| NM_032019 | histone deacetylase 10 | HDAC10 |
| NM_015401 | histone deacetylase 7A | HDAC7A |
| NM_002147 | homeo box B5 | HOXB5 |
| XR_011538 | Homeobox protein Hox-D10 | Hox-4D |
| NM_144565 | homolog of Drosophila Numb-interacting protein | NIP |
| NM_080731 | HOM-TES-103 tumor antigen-like | HOM-TES-103 |
| CK231263 | Hs296141 | Hs296141 |
| CO582642 | Hs521442 | Hs521442 |

| | | |
|---|---|---|
| NM_003949 | huntingtin-associated protein 1 | HAP1 |
| NM_014234 | hydroxysteroid | HSD17B8 |
| NM_052818 | hypothetical gene CG018 | CG018 |
| NM_139016 | hypothetical gene LOC128439 | LOC128439 |
| XM_498519 | hypothetical gene supported by AK124252 | LOC440049 |
| NM_203393 | hypothetical gene supported by BC031661 | LOC389458 |
| NM_138414 | hypothetical protein BC011981 | LOC112869 |
| NM_144697 | hypothetical protein BC017397 | LOC148523 |
| NM_207310 | hypothetical protein DKFZp434E2321 | DKFZp434E2321 |
| NM_017559 | hypothetical protein DKFZp434H2215 | DKFZp434H2215 |
| NM_032269 | hypothetical protein DKFZp434I099 | DKFZp434I099 |
| NM_017991 | hypothetical protein FLJ10081 | FLJ10081 |
| NM_018011 | hypothetical protein FLJ10154 | FLJ10154 |
| NM_019057 | hypothetical protein FLJ10404 | FLJ10404 |
| NM_018089 | hypothetical protein FLJ10415 | FLJ10415 |
| NM_018142 | hypothetical protein FLJ10569 | FLJ10569 |
| NM_018281 | hypothetical protein FLJ10948 | FLJ10948 |
| XM_035527 | hypothetical protein FLJ10980 | FLJ10980 |
| NM_024669 | hypothetical protein FLJ11795 | FLJ11795 |
| NM_031206 | hypothetical protein FLJ12525 | FLJ12525 |
| NM_022753 | hypothetical protein FLJ12903 | FLJ12903 |
| NM_032118 | hypothetical protein FLJ12953 Mus musculus D3Mm3e | FLJ12953 |
| NM_024841 | hypothetical protein FLJ14213 | FLJ14213 |
| NM_207514 | hypothetical protein FLJ20186 | FLJ20186 |
| NM_017820 | hypothetical protein FLJ20433 | FLJ20433 |
| NM_017822 | hypothetical protein FLJ20436 | FLJ20436 |
| NM_024927 | hypothetical protein FLJ21019 | FLJ21019 |
| NM_032207 | hypothetical protein FLJ21742 | FLJ21742 |
| NM_023015 | hypothetical protein FLJ21919 | FLJ21919 |
| NM_024790 | hypothetical protein FLJ22490 | FLJ22490 |
| NM_025092 | hypothetical protein FLJ22635 | FLJ22635 |
| NM_024730 | hypothetical protein FLJ22655 | FLJ22655 |
| NM_024631 | hypothetical protein FLJ23342 | FLJ23342 |
| NM_152343 | hypothetical protein FLJ25414 | FLJ25414 |
| NM_152748 | hypothetical protein FLJ31340 | FLJ31340 |
| NM_144679 | hypothetical protein FLJ31528 | FLJ31528 |
| NM_152509 | hypothetical protein FLJ31568 | FLJ31568 |
| NM_144674 | hypothetical protein FLJ32871 | FLJ32871 |
| NM_152449 | hypothetical protein FLJ33008 | FLJ33008 |
| NM_152683 | hypothetical protein FLJ33167 | FLJ33167 |
| NM_152408 | hypothetical protein FLJ35779 | FLJ35779 |
| NM_020214 | hypothetical protein from EUROIMAGE 1977056 | LOC56965 |
| NM_031207 | hypothetical protein HT036 | HT036 |
| NM_019092 | hypothetical protein KIAA1164 | KIAA1164 |
| NM_019593 | hypothetical protein KIAA1434 | KIAA1434 |
| NM_207324 | hypothetical protein LOC147650 | LOC147650 |

| | | |
|---|---|---|
| XM_113796 | hypothetical protein LOC196996 | LOC196996 |
| NM_198278 | hypothetical protein LOC255743 | LOC255743 |
| NM_182774 | hypothetical protein LOC259173 | FLJ36525 |
| NM_174940 | hypothetical protein LOC283232 | LOC283232 |
| NM_001012506 | hypothetical protein LOC285331 | LOC285331 |
| NM_198284 | hypothetical protein LOC349114 | LOC349114 |
| XR_012042 | hypothetical protein LOC709438 | LOC709438 |
| XR_014590 | hypothetical protein LOC721251 | LOC721251 |
| XR_014848 | hypothetical protein LOC723278 | LOC723278 |
| NM_030818 | hypothetical protein MGC10471 | MGC10471 |
| NM_032328 | hypothetical protein MGC12458 | MGC12458 |
| NM_144626 | hypothetical protein MGC17299 | MGC17299 |
| NM_152421 | hypothetical protein MGC20262 | MGC20262 |
| NM_182614 | hypothetical protein MGC20579 | MGC20579 |
| NM_145274 | hypothetical protein MGC21518 | MGC21518 |
| NM_144982 | hypothetical protein MGC23401 | MGC23401 |
| NM_144664 | hypothetical protein MGC33371 | MGC33371 |
| NM_152479 | hypothetical protein MGC33962 | MGC33962 |
| NM_173525 | hypothetical protein MGC34805 | MGC34805 |
| NM_152314 | hypothetical protein MGC34830 | MGC34830 |
| NM_178565 | hypothetical protein MGC35555 | MGC35555 |
| NM_147189 | hypothetical protein MGC39325 | MGC39325 |
| NM_203306 | hypothetical protein MGC39606 | MGC39606 |
| NM_153361 | hypothetical protein MGC42105 | MGC42105 |
| NM_152459 | hypothetical protein MGC45438 | MGC45438 |
| NM_152689 | hypothetical protein MGC9712 | MGC9712 |
| NM_018607 | hypothetical protein PRO1853 | PRO1853 |
| CN803406 | IGFBP6 | IGFBP6 |
| NM_178822 | immunoglobulin superfamily, member 10 | IGSF10 |
| NM_176878 | InaD-like | INADL |
| NM_181353 | inhibitor of DNA binding 1, dominant negative helix-loop-helix protein | ID1 |
| NM_001546 | inhibitor of DNA binding 4, dominant negative helix-loop-helix protein | ID4 |
| NM_032329 | inhibitor of growth family, member 5 | ING5 |
| NM_002224 | inositol 1,4,5-triphosphate receptor, type 3 | ITPR3 |
| NM_001005912 | inositol hexaphosphate kinase 2 | IHPK2 |
| NM_016291 | inositol hexaphosphate kinase 2 | IHPK2 |
| NM_198541 | insulin growth factor-like family member 1 | IGFL1 |
| XR_014528 | insulin receptor-related receptor | INSRR |
| NM_003638 | integrin, alpha 8 | ITGA8 |
| NM_002217 | inter-alpha | ITIH3 |
| NM_007059 | kaptin | KPTN |
| NM_014315 | kelch domain containing 2 | KLHDC2 |
| NM_000526 | keratin 14 | KRT14 |
| NM_000422 | keratin 17 | KRT17 |
| NM_015342 | KIAA0073 protein | KIAA0073 |

| | | |
|---|---|---|
| NM_015115 | KIAA0276 protein | KIAA0276 |
| NM_024874 | KIAA0319-like | KIAA0319L |
| NM_015330 | KIAA0376 protein | KIAA0376 |
| NM_014859 | KIAA0672 gene product | KIAA0672 |
| NM_016111 | KIAA0683 gene product | KIAA0683 |
| NM_015037 | KIAA0913 | KIAA0913 |
| NM_025176 | KIAA0980 protein | KIAA0980 |
| NM_014972 | KIAA1049 protein | KIAA1049 |
| XM_044461 | KIAA1102 protein | KIAA1102 |
| NM_019590 | KIAA1217 | KIAA1217 |
| NM_020734 | KIAA1238 protein | KIAA1238 |
| NM_015689 | KIAA1277 | KIAA1277 |
| NM_007054 | kinesin family member 3A | KIF3A |
| NM_177417 | kinesin light chain 2-like | KLC2L |
| NM_201523 | kinesin-like 8 | KNSL8 |
| NM_152775 | KM-HN-1 protein | KM-HN-1 |
| NM_198129 | laminin, alpha 3 | LAMA3 |
| NM_005560 | laminin, alpha 5 | LAMA5 |
| NM_002292 | laminin, beta 2 | LAMB2 |
| NM_018697 | LanC lantibiotic synthetase component C-like 2 | LANCL2 |
| NM_178354 | late cornified envelope 1F | LCE1F |
| CN641580 | LDLR | LDLR |
| NM_000229 | lecithin-cholesterol acyltransferase | LCAT |
| NM_203471 | lectin, galactoside-binding, soluble, 14 | LGALS14 |
| NM_001003679 | leptin receptor | LEPR |
| NM_004524 | lethal giant larvae homolog 2 | LLGL2 |
| XR_011718 | leucine rich repeat and sterile alpha motif containing 1 | LRSAM1 |
| NM_031294 | leucine rich repeat containing 48 | LRRC48 |
| NM_002344 | leukocyte tyrosine kinase | LTK |
| NM_014583 | LIM and cysteine-rich domains 1 | LMCD1 |
| NM_017980 | LIM and senescent cell antigen-like domains 2 | LIMS2 |
| NM_007078 | LIM domain binding 3 | LDB3 |
| NM_018032 | LUC7-like | LUC7L |
| NM_000894 | luteinizing hormone beta polypeptide | LHB |
| NM_053051 | LYST-interacting protein LIP8 | LIP8 |
| DQ159933 | Macaca fascicularis GluR5 | GRIK1 |
| BC067766 | MAD, mothers against decapentaplegic homolog 9 | MADH9 |
| NM_032228 | male sterility domain containing 2 | MLSTD2 |
| NM_002395 | malic enzyme 1, NADP | ME1 |
| NM_006715 | mannosidase, alpha, class 2C, member 1 | MAN2C1 |
| NM_020690 | MASK-4E-BP3 alternate reading frame gene | MASK-BP3 |
| NM_006454 | MAX dimerization protein 4 | MXD4 |
| NM_002398 | Meis1, myeloid ecotropic viral integration site 1 homolog | MEIS1 |
| NM_138703 | melanoma antigen family E, 2 | MAGEE2 |
| NM_006533 | melanoma inhibitory activity | MIA |

| | | |
|---|---|---|
| NM_024101 | melanophilin | MLPH |
| NM_001932 | membrane protein, palmitoylated 3 | MPP3 |
| XR_013910 | meningioma expressed antigen 5 | LOC712370 |
| NM_004689 | metastasis associated 1 | MTA1 |
| NM_052897 | methyl-CpG binding domain protein 6 | MBD6 |
| NM_020166 | methylcrotonoyl-Coenzyme A carboxylase 1 | alpha |
| NM_002404 | microfibrillar-associated protein 4 | MFAP4 |
| NM_033044 | microtubule-actin crosslinking factor 1 | MACF1 |
| NM_145729 | mitochondrial ribosomal protein L24 | MRPL24 |
| XR_011805 | mitochondrial tumor suppressor 1 isoform 1 | MTUS1 |
| NM_004672 | mitogen-activated protein kinase kinase kinase 6 | MAP3K6 |
| CB550393 | MMPL0003_B02 MMPL cDNA sequence | |
| CB548968 | MMPL0018_H05 MMPL cDNA sequence | |
| D86962 | mRNA for KIAA0207 gene, partial cds | mRNA for KIAA0207 gene, partial |
| AB209405 | mRNA for RYK receptor-like tyrosine kinase isoform 1 variant protein | RYKRLTK |
| AB209485 | mRNA for vascular endothelial growth factor variant protein | VEGF |
| NM_021924 | mucin and cadherin-like | MUCDHL |
| NM_031264 | mucin and cadherin-like | MUCDHL |
| NM_007351 | multimerin 1 | MMRN1 |
| NM_032133 | MYCBP associated protein | MYCBPAP |
| NM_004991 | myelodysplasia syndrome 1 | MDS1 |
| NM_001009569 | myeloid/lymphoid or mixed-lineage leukemia | MLLT10 |
| NM_003970 | myomesin | MYOM2 |
| XR_010160 | myomesin 1 | MYOM1 |
| NM_005964 | myosin, heavy polypeptide 10, non-muscle | MYH10 |
| NM_022844 | myosin, heavy polypeptide 11, smooth muscle | MYH11 |
| XR_012588 | myosin, heavy polypeptide 7B, cardiac muscle, beta | MYH7 |
| NM_181526 | myosin, light polypeptide 9, regulatory | MYL9 |
| NM_006766 | MYST histone acetyltransferase | MYST3 |
| NM_006647 | NADPH oxidase activator 1 | NOXA1 |
| NM_000906 | natriuretic peptide receptor A/guanylate cyclase A | NPR1 |
| NM_005385 | natural killer-tumor recognition sequence | NKTR |
| NM_003635 | N-deacetylase/N-sulfotransferase | NDST2 |
| NM_016250 | NDRG family member 2 | NDRG2 |
| NM_006175 | nebulin-related anchoring protein | NRAP |
| NM_024608 | nei endonuclease VIII-like 1 | NEIL1 |
| NM_014380 | nerve growth factor receptor | NGFRAP1 |
| NM_020795 | neuroligin 2 | NLGN2 |
| NM_182964 | neuron navigator 2 | NAV2 |
| NM_003717 | neuropeptide FF-amide peptide precursor | NPFF |
| NM_003873 | neuropilin 1 | NRP1 |
| NM_145912 | NFAT activating protein with ITAM motif 1 | NFAM1 |
| NM_002498 | NIMA | NEK3 |
| NM_007184 | nischarin | NISCH |
| NM_145285 | NK2 transcription factor related, locus 3 | NKX2-3 |

| | | |
|---|---|---|
| NM_002513 | non-metastatic cells 3, protein expressed in | NME3 |
| NM_020317 | NPD014 protein | NPD014 |
| NM_173474 | N-terminal asparagine amidase | NTAN1 |
| NM_005654 | nuclear receptor subfamily 2, group F, member 1 | NR2F1 |
| NM_006362 | nuclear RNA export factor 1 | NXF1 |
| NM_006392 | nucleolar protein 5A | NOL5A |
| NM_145260 | odd-skipped homolog | ODD |
| NM_001004713 | olfactory receptor, family 1, subfamily I, member 1 | OR1I1 |
| XR_014601 | olfactory receptor, family 7, subfamily D, member 4 | ORID2 |
| NM_003605 | O-linked N-acetylglucosamine | OGT |
| NM_007346 | opioid growth factor receptor | OGFR |
| NM_003611 | oral-facial-digital syndrome 1 | OFD1 |
| U87259 | oviductal glycoprotein mRNA, complete cds | oviductal glycoprotein mRNA, cor |
| NM_017784 | oxysterol binding protein-like 10 | OSBPL10 |
| NM_138934 | palmitoyl-protein thioesterase 2 | PPT2 |
| NM_001034171 | Pan troglodytes centromere protein J | CENPJ |
| NM_173462 | papilin, proteoglycan-like sulfated glycoprotein | PAPLN |
| NM_000940 | paraoxonase 3 | PON3 |
| NM_018282 | paraspeckle component 1 | PSPC1 |
| NM_000316 | parathyroid hormone receptor 1 | PTHR1 |
| NM_000264 | patched homolog | PTCH |
| NM_024895 | PDZ domain containing 7 | PDZK7 |
| NM_022817 | period homolog 2 | PER2 |
| NM_000304 | peripheral myelin protein 22 | PMP22 |
| NM_000466 | peroxisome biogenesis factor 1 | PEX1 |
| NM_024165 | PHD finger protein 1 | PHF1 |
| XR_013095 | phosphatidylethanolamine-binding protein 4 | PEBP4 |
| NM_002641 | phosphatidylinositol glycan, class A | PIGA |
| XR_010253 | phosphatidylserine synthase 2 | PSS2 |
| NM_005090 | phospholipase A2, group IVB | PLA2G4B |
| XR_011250 | phospholipase A2, group VI isoform a | PLA2G6 |
| NM_006225 | phospholipase C, delta 1 | PLCD1 |
| NM_002660 | phospholipase C, gamma 1 | PLCG1 |
| NM_006226 | phospholipase C-like 1 | PLCL1 |
| NM_002766 | phosphoribosyl pyrophosphate synthetase-associated protein 1 | PRPSAP1 |
| NM_000292 | phosphorylase kinase, alpha 2 | PHKA2 |
| NM_001012973 | placenta-specific 9 | PLAC9 |
| NM_003628 | plakophilin 4 | PKP4 |
| NM_019012 | pleckstrin homology domain containing, family A member 5 | PLEKHA5 |
| NM_014935 | pleckstrin homology domain containing, family A member 6 | PLEKHA6 |
| XR_013919 | pleckstrin homology domain containing, family H | LOC713488 |
| NM_145307 | pleckstrin homology domain containing, family K member 1 | PLEKHK1 |
| NM_017934 | pleckstrin homology domain interacting protein | PHIP |

| | | |
|---|---|---|
| XR_014196 | pleckstrin homology domain interacting protein | PHDIP |
| NM_004227 | pleckstrin homology, Sec7 and coiled-coil domains 3 | PSCD3 |
| NM_145753 | pleckstrin homology-like domain, family B, member 2 | PHLDB2 |
| NM_025179 | plexin A2 | PLXNA2 |
| NM_017514 | plexin A3 | PLXNA3 |
| NM_002673 | plexin B1 | PLXNB1 |
| NM_015100 | pogo transposable element with ZNF domain | POGZ |
| NM_003631 | poly | PARG |
| NM_031293 | polyamine modulated factor 1 binding protein 1 | PMFBP1 |
| NM_000937 | polymerase | RNA II |
| NM_016611 | potassium channel, subfamily K, member 4 | KCNK4 |
| NM_133497 | potassium channel, subfamily V, member 2 | KCNV2 |
| NM_002232 | potassium voltage-gated channel, shaker-related subfamily, member 3 | KCNA3 |
| NM_012285 | potassium voltage-gated channel, subfamily H | KCNH4 |
| CO725485 | PPP1R114A | PPP1R114A |
| NM_012231 | PR domain containing 2, with ZNF domain | PRDM2 |
| CN804253 | PRO0659 | PRO0659 |
| NM_002630 | progastricsin | PGC |
| NM_016335 | proline dehydrogenase | PRODH |
| NM_016223 | protein kinase C and casein kinase substrate in neurons 3 | PACSIN3 |
| NM_002744 | protein kinase C, zeta | PRKCZ |
| NM_005399 | protein kinase, AMP-activated, beta 2 non-catalytic subunit | PRKAB2 |
| NM_032105 | protein phosphatase 1, regulatory | PPP1R12B |
| NM_177951 | protein phosphatase 1A | PPM1A |
| NM_005167 | protein phosphatase 1J | PPM1J |
| XR_013214 | protein phosphatase 2, regulatory subunit B, beta isoform 1 | PPP2R2B |
| NM_000309 | protoporphyrinogen oxidase | PPOX |
| NM_017922 | PRP39 pre-mRNA processing factor 39 homolog | PRPF39 |
| NM_176800 | PRP4 pre-mRNA processing factor 4 homolog B | PRPF4B |
| NM_025215 | pseudouridylate synthase 1 | PUS1 |
| NM_005859 | purine-rich element binding protein A | PURA |
| NM_005049 | PWP2 periodic tryptophan protein homolog | PWP2H |
| NM_130781 | RAB24, member RAS oncogene family | RAB24 |
| NM_002866 | RAB3A, member RAS oncogene family | RAB3A |
| NM_182947 | RAC/CDC42 exchange factor | GEFT |
| NM_004761 | ral guanine nucleotide dissociation stimulator-like 2 | RGL2 |
| NM_006105 | Rap guanine nucleotide exchange factor | GEF 3 |
| NM_016340 | Rap guanine nucleotide exchange factor | GEF 6 |
| XR_013530 | RAR-related orphan receptor C isoform a | RORC |
| NM_152573 | RAS and EF hand domain containing | RASEF |
| NM_025252 | Ras association | RAPH1 |
| NM_052949 | RAS guanyl releasing protein 4 | RASGRP4 |

| | | |
|---|---|---|
| NM_016290 | receptor associated protein 80 | RAP80 |
| NM_020639 | receptor-interacting serine-threonine kinase 4 | RIPK4 |
| NM_005613 | regulator of G-protein signalling 4 | RGS4 |
| XR_014513 | regulator of G-protein signalling 9 | RGS9 |
| NM_014226 | renal tumor antigen | RAGE |
| NM_173587 | REST corepressor 2 | RCOR2 |
| NM_018254 | REST corepressor 3 | RCOR3 |
| NM_031429 | retbindin | RTBDN |
| NM_023004 | reticulon 4 receptor | RTN4R |
| NM_021976 | retinoid X receptor, beta | RXRB |
| NM_016316 | REV1-like | REV1L |
| NM_014433 | rhabdoid tumor deletion region gene 1 | RTDR1 |
| J04697 | Rhesus monkey plasminogen mRNA, complete cds | PLN |
| NM_020824 | Rho GTPase activating protein 21 | ARHGAP21 |
| NM_001174 | Rho GTPase activating protein 6 | ARHGAP6 |
| NM_003899 | Rho guanine nucleotide exchange factor | GEF 7 |
| NM_014786 | Rho guanine nucleotide exchange factor | GEF 17 |
| NM_022450 | rhomboid family 1 | RHBDF1 |
| NM_000977 | ribosomal protein L13 | RPL13 |
| NM_024557 | RIC3 protein | RIC3 |
| XM_376148 | RIKEN cDNA 5830415L20 | LOC401015 |
| NM_183353 | ring finger protein 12 | RNF12 |
| XM_027330 | RNA binding motif protein 25 | RBM25 |
| NM_005778 | RNA binding motif protein 5 | RBM5 |
| NM_005777 | RNA binding motif protein 6 | RBM6 |
| NM_001008710 | RNA binding protein with multiple splicing | RBPMS |
| NM_184244 | RNA-binding region | RNP1 |
| NM_173640 | roof plate-specific spondin | RSPONDIN |
| XR_011047 | RP11-506B151 protein isoform 1 | LOC702661 |
| NM_025158 | RUN and FYVE domain containing 1 | RUFY1 |
| NM_014328 | RUN and SH3 domain containing 1 | RUSC1 |
| NM_020672 | S100 calcium binding protein A14 | S100A14 |
| NM_014624 | S100 calcium binding protein A6 | calcyclin |
| NM_005407 | sal-like 2 | SALL2 |
| NM_015490 | SEC31-like 2 | SEC31L2 |
| NM_138355 | secernin 2 | SCRN2 |
| NM_015662 | selective LIM binding factor, rat homolog | SLB |
| NM_004636 | sema domain, immunoglobulin domain | semaphorin 3B |
| NM_020796 | sema domain, transmembrane domain | TM |
| NM_145733 | septin 3 | 3-Sep |
| NM_006843 | serine dehydratase | SDS |
| NM_016333 | serine/arginine repetitive matrix 2 | SRRM2 |
| NM_052902 | serine/threonine kinase 11 interacting protein | STK11IP |
| NM_014370 | serine/threonine kinase 23 | STK23 |
| NM_005490 | SH2 domain containing 3A | SH2D3A |
| NM_012309 | SH3 and multiple ankyrin repeat domains 2 | SHANK2 |
| NM_005413 | sine oculis homeobox homolog 3 | SIX3 |

| | | |
|---|---|---|
| NM_021805 | single Ig IL-1R-related molecule | SIGIRR |
| NM_012240 | sirtuin | SIRT4 |
| NM_015065 | SLAC2-B | SLAC2-B |
| NM_033438 | SLAM family member 9 | SLAMF9 |
| NM_004787 | slit homolog 2 | SLIT2 |
| NM_000336 | sodium channel, nonvoltage-gated 1, beta | SCNN1B |
| NM_004277 | solute carrier family 25, member 27 | SLC25A27 |
| NM_024330 | solute carrier family 27 | SLC27A3 |
| NM_018964 | solute carrier family 37 | SLC37A1 |
| NM_173854 | solute carrier family 41, member 1 | SLC41A1 |
| NM_014270 | solute carrier family 7 | cationic amino acid transporter |
| NM_003103 | SON DNA binding protein | SON |
| NM_058183 | SON DNA binding protein | SON |
| NM_005633 | son of sevenless homolog 1 | SOS1 |
| NM_004510 | SP110 nuclear body protein | SP110 |
| NM_002971 | special AT-rich sequence binding protein 1 | SATB1 |
| XR_012522 | spectrin repeat containing, nuclear envelope 2 isoform e | SYNE2 |
| XR_012522 | spectrin repeat containing, nuclear envelope 2 isoform e | |
| NM_032637 | S-phase kinase-associated protein 2 | SKP2 |
| NM_019003 | spindlin family, member 2 | SPIN2 |
| NM_201997 | splicing factor 1 | SF1 |
| NM_012433 | splicing factor 3b, subunit 1, 155kDa | SF3B1 |
| NM_004768 | splicing factor, arginine/serine-rich 11 | SFRS11 |
| NM_006925 | splicing factor, arginine/serine-rich 5 | SFRS5 |
| NM_006275 | splicing factor, arginine/serine-rich 6 | SFRS6 |
| NM_022039 | split hand/foot malformation | SHFM3 |
| NM_080861 | SPRY domain-containing SOCS box protein SSB-3 | SSB3 |
| NM_020225 | storkhead box 2 | STOX2 |
| NM_014351 | sulfotransferase family 4A, member 1 | SULT4A1 |
| NM_177529 | sulfotransferase family, cytosolic, 1A, phenol-preferring, member 1 | SULT1A1 |
| NM_177528 | sulfotransferase family, cytosolic, 1A, phenol-preferring, member 2 | SULT1A2 |
| NM_003166 | sulfotransferase family, cytosolic, 1A, phenol-preferring, member 3 | SULT1A3 |
| NM_003877 | suppressor of cytokine signaling 2 | SOCS2 |
| NM_003955 | suppressor of cytokine signaling 3 | SOCS3 |
| NM_003019 | surfactant, pulmonary-associated protein D | SFTPD |
| NM_017503 | surfeit 2 | SURF2 |
| NM_019601 | sushi domain containing 2 | SUSD2 |
| NM_139067 | SWI/SNF related, matrix associated, actin dependent regulator of chromatin, subfamily c, member 2 | SMARCC2 |
| NM_004209 | synaptogyrin 3 | SYNGR3 |
| NM_024875 | synaptopodin 2-like | SYNPO2L |
| NM_003180 | synaptotagmin V | SYT5 |

| | | |
|---|---|---|
| NM_004603 | syntaxin 1A | STX1A |
| NM_004177 | syntaxin 3A | STX3A |
| NM_019020 | TBC1 domain family, member 16 | TBC1D16 |
| NM_005994 | T-box 2 | TBX2 |
| NM_016569 | T-box 3 | TBX3 |
| NM_000192 | T-box 5 | TBX5 |
| CN645262 | TCERG1 | TCERG1 |
| XR_014213 | tenascin XB isoform 1 | TNXB |
| NM_015319 | tensin like C1 domain containing phosphatase | TENC1 |
| NM_175605 | tetratricopeptide repeat domain 10 | TTC10 |
| XR_011624 | tetratricopeptide repeat domain 16 | TTC16 |
| NM_018259 | tetratricopeptide repeat domain 17 | TTC17 |
| NM_145170 | tetratricopeptide repeat domain 18 | TTC18 |
| NM_003597 | TGFB inducible early growth response 2 | TIEG2 |
| NM_004783 | thousand and one amino acid protein kinase | TAO1 |
| NM_007112 | thrombospondin 3 | THBS3 |
| NM_025008 | thrombospondin repeat containing 1 | TSRC1 |
| NM_145056 | thymus expressed gene 3-like | MGC15476 |
| NM_004240 | thyroid hormone receptor interactor 10 | TRIP10 |
| NM_003317 | thyroid transcription factor 1 | TITF1 |
| NM_003216 | thyrotrophic embryonic factor | TEF |
| NM_033208 | tigger transposable element derived 7 | TIGD7 |
| NM_004817 | tight junction protein 2 | TJP2 |
| NM_000362 | tissue inhibitor of metalloproteinase 3 | TIMP3 |
| NM_003673 | titin-cap | TCAP |
| NM_000355 | transcobalamin II; macrocytic anemia | TCN2 |
| NM_018719 | transcription factor RAM2 | RAM2 |
| NM_025198 | transcription termination factor-like protein | LOC80298 |
| NM_003260 | transducin-like enhancer of split 2 | TLE2 |
| NM_013293 | transformer-2 alpha | TRA2A |
| NM_003243 | transforming growth factor, beta receptor III | TGFBR3 |
| NM_014555 | transient receptor potential cation channel, subfamily M, member 5 | TRPM5 |
| NM_032405 | transmembrane protease, serine 3 | TMPRSS3 |
| NM_032780 | transmembrane protein 25 | TMEM25 |
| NM_001011655 | transmembrane protein 44 | TMEM44 |
| NM_012101 | tripartite motif-containing 29 | TRIM29 |
| NM_033342 | tripartite motif-containing 7 | TRIM7 |
| NM_203293 | tripartite motif-containing 7 | TRIM7 |
| NM_013353 | tropomodulin 4 | muscle |
| NM_213674 | tropomyosin 2 | TPM2 |
| NM_003279 | troponin C2, fast | TNNC2 |
| NM_003283 | troponin T1, skeletal, slow | TNNT1 |
| NM_003320 | tubby homolog | TUB |
| NM_015644 | tubulin tyrosine ligase-like family, member 3 | TTLL3 |
| NM_016437 | tubulin, gamma 2 | TUBG2 |
| XR_014480 | tubulin, gamma complex associated protein 6 | TUBGCP6 |

| | | |
|---|---|---|
| NM_003193 | tubulin-specific chaperone e | TBCE |
| NM_006545 | tumor suppressor candidate 4 | TUSC4 |
| XR_011976 | tumor suppressor candidate 5 | TUSC5 |
| NM_001010938 | tyrosine kinase, non-receptor, 2 | TNK2 |
| NM_017619 | U11/U12 snRNP 65K protein | FLJ25070 |
| NM_024954 | ubiquitin domain containing 1 | UBTD1 |
| NM_003940 | ubiquitin specific protease 13 | USP13 |
| NM_018218 | ubiquitin specific protease 40 | USP40 |
| NM_014871 | ubiquitin specific protease 52 | USP52 |
| AW014767 | UI-H-BI0-aae-f-12-0-UIs1 NCI_CGAP_Sub1 cDNA clone IMAGE:2709262 3' sequence | |
| NM_199242 | unc-13 homolog D | UNC13D |
| NM_003565 | unc-51-like kinase 1 | ULK1 |
| XM_379766 | unc-84 homolog A | UNC84A |
| NM_018463 | uncharacterized hematopoietic stem/progenitor cells protein MDS028 | MDS028 |
| NM_139205 | Unknown | Unknown |
| CN643806 | USP32 | USP32 |
| NM_004448 | v-erb-b2 erythroblastic leukemia viral oncogene homolog 2, neuro/glioblastoma derived oncogene homolog | ERBB2 |
| NM_005252 | v-fos FBJ murine osteosarcoma viral oncogene homolog | FOS |
| XR_014061 | WAP four-disulfide core domain 1 precursor | WFDC1 |
| NM_015626 | WD repeat and SOCS box-containing 1 | WSB1 |
| NM_018262 | WD repeat domain 10 | WDR10 |
| NM_025132 | WD repeat domain 19 | WDR19 |
| NM_006784 | WD repeat domain 3 | WDR3 |
| NM_032951 | Williams Beuren syndrome chromosome region 14 | WBSCR14 |
| NM_149379 | Williams Beuren syndrome chromosome region 20C | WBSCR20C |
| NM_145645 | Williams-Beuren Syndrome critical region protein 20 copy B | WBSCR20B |
| NM_012477 | WW domain binding protein 1 | WBP1 |
| NM_020231 | x 010 protein | MDS010 |
| NM_031477 | yippee-like 3 | YPEL3 |
| NM_006006 | zinc finger and BTB domain containing 16 | ZBTB16 |
| NM_015642 | zinc finger and BTB domain containing 20 | ZBTB20 |
| NM_020899 | zinc finger and BTB domain containing 4 | ZBTB4 |
| NM_144621 | zinc finger and BTB domain containing 8 | ZBTB8 |
| XR_013013 | Zinc finger CW-type PWWP domain protein 1 homolog | ZCWPW2 |
| XM_290835 | zinc finger protein 181 | HHZ181 |
| NM_006385 | zinc finger protein 211 | ZNF211 |
| NM_016423 | zinc finger protein 219 | ZNF219 |
| XR_012685 | zinc finger protein 221 | ZNF221 |
| NM_019591 | zinc finger protein 26 | KOX 20 |
| NM_005455 | zinc finger protein 265 | ZNF265 |

| | | |
|---|---|---|
| NM_001005368 | zinc finger protein 32 | KOX 30 |
| NM_022095 | zinc finger protein 335 | ZNF335 |
| NM_017810 | zinc finger protein 434 | ZNF434 |
| NM_181489 | zinc finger protein 445 | ZNF445 |
| NM_145291 | zinc finger protein 509 | ZNF509 |
| NM_152520 | zinc finger protein 533 | ZNF533 |
| NM_020747 | zinc finger protein 608 | ZNF608 |
| XM_171060 | zinc finger protein 620 | ZNF620 |
| NM_003416 | zinc finger protein 7 | KOX 4 |
| NM_003427 | zinc finger protein 76 | ZNF76 |
| XM_930351 | zinc finger protein HIT-40 | zinc finger protein HIT-40 |
| NM_024657 | zinc finger, CW-type with coiled-coil domain 2 | ZCWCC2 |
| NM_013304 | zinc finger, DHHC-type containing 1 | ZDHHC1 |
| XR_013613 | ZXD family zinc finger C isoform 2 | ZXDC |

YH05 of Fetal brain of

cds

nplete cds
