# Supplementary figures and images for: Transcriptional Reprogramming in Nonhuman Primate (Rhesus Macaque) Tuberculosis Granulomas
Source: PLoS One. 2010 Aug 31;5(8):e12266. doi: 10.1371/journal.pone.0012266 (PMC2930844; doi:10.1371/journal.pone.0012266)

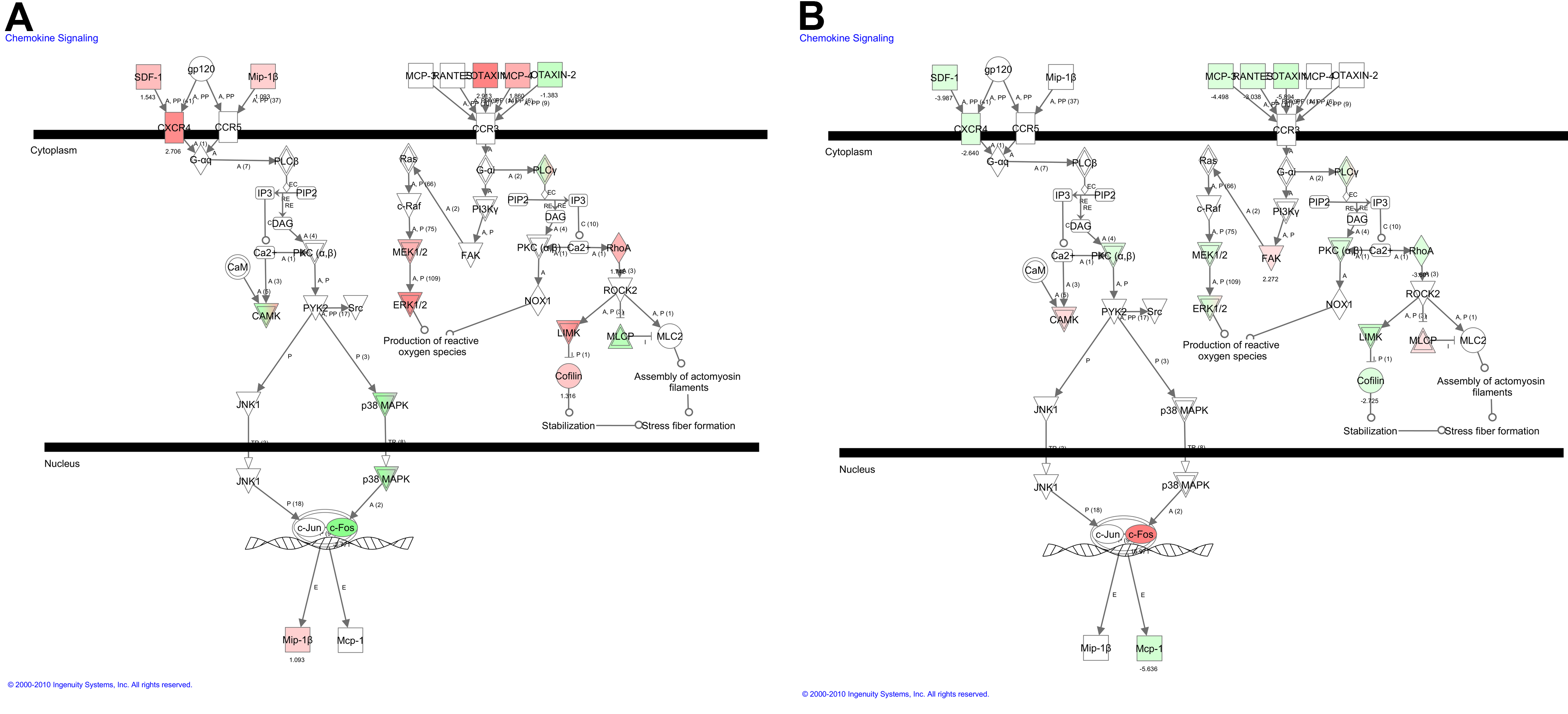

Supplement: Figure S1 — Comparison of the chemokine signaling pathway in early and late TB granulomas. Canonical pathways contained within the IPA algorithm were queried with the list of genes significantly up (red) or down (green) regulated in week 4 or week 13 lesions. Pathway illustrations are shown for week 4 (A) and week 13 (B) lesions. (0.75 MB TIF) [file pone.0012266.s014.tif]

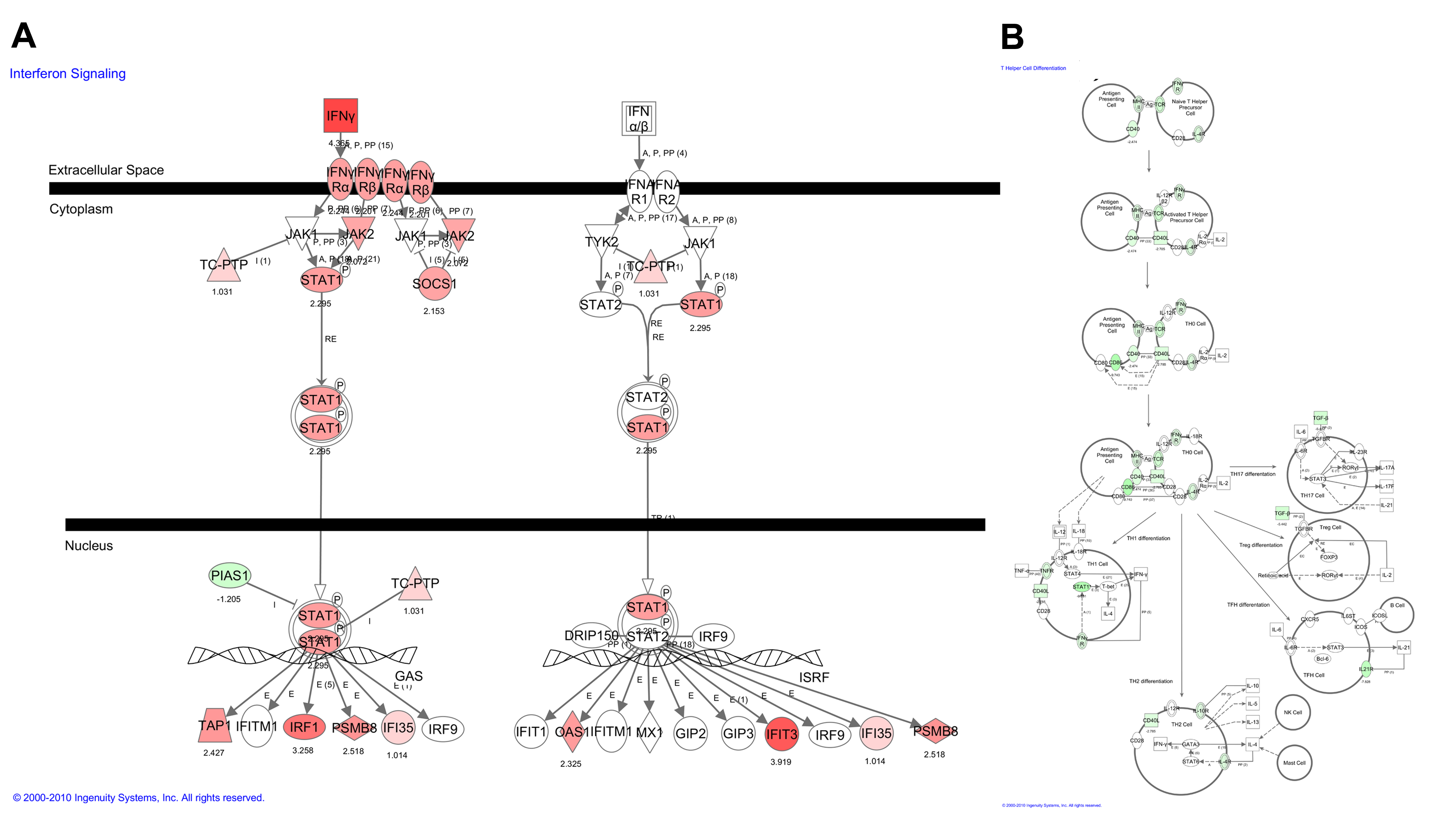

Supplement: Figure S2 — Comparison of the interferon signaling pathway in early and late TB granulomas. Canonical pathways contained within the IPA algorithm were queried with the list of genes significantly up (red) or down (green) regulated in week 4 or week 13 lesions. Pathway illustrations are shown for week 4 (A) and week 13 (B) lesions. (1.43 MB TIF) [file pone.0012266.s015.tif]

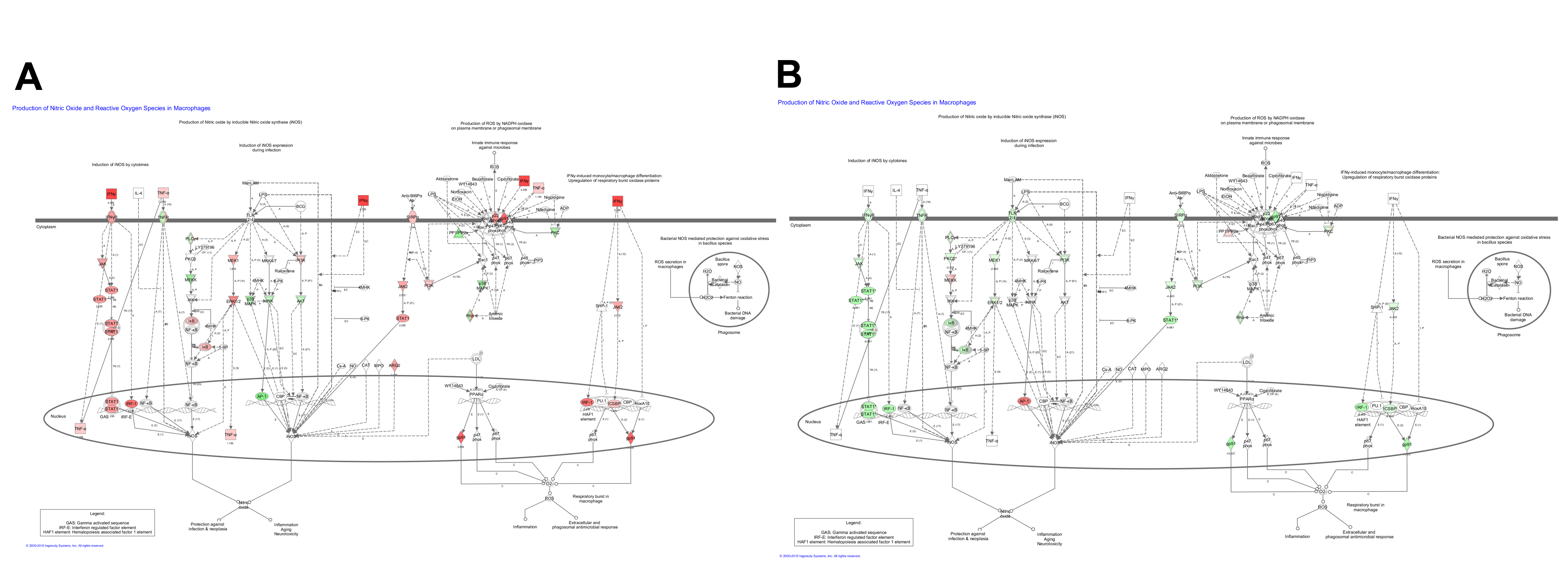

Supplement: Figure S3 — Comparison of the nitric oxide production pathway in early and late TB granulomas. Canonical pathways contained within the IPA algorithm were queried with the list of genes significantly up (red) or down (green) regulated in week 4 or week 13 lesions. Pathway illustrations are shown for week 4 (A) and week 13 (B) lesions. (0.96 MB TIF) [file pone.0012266.s016.tif]

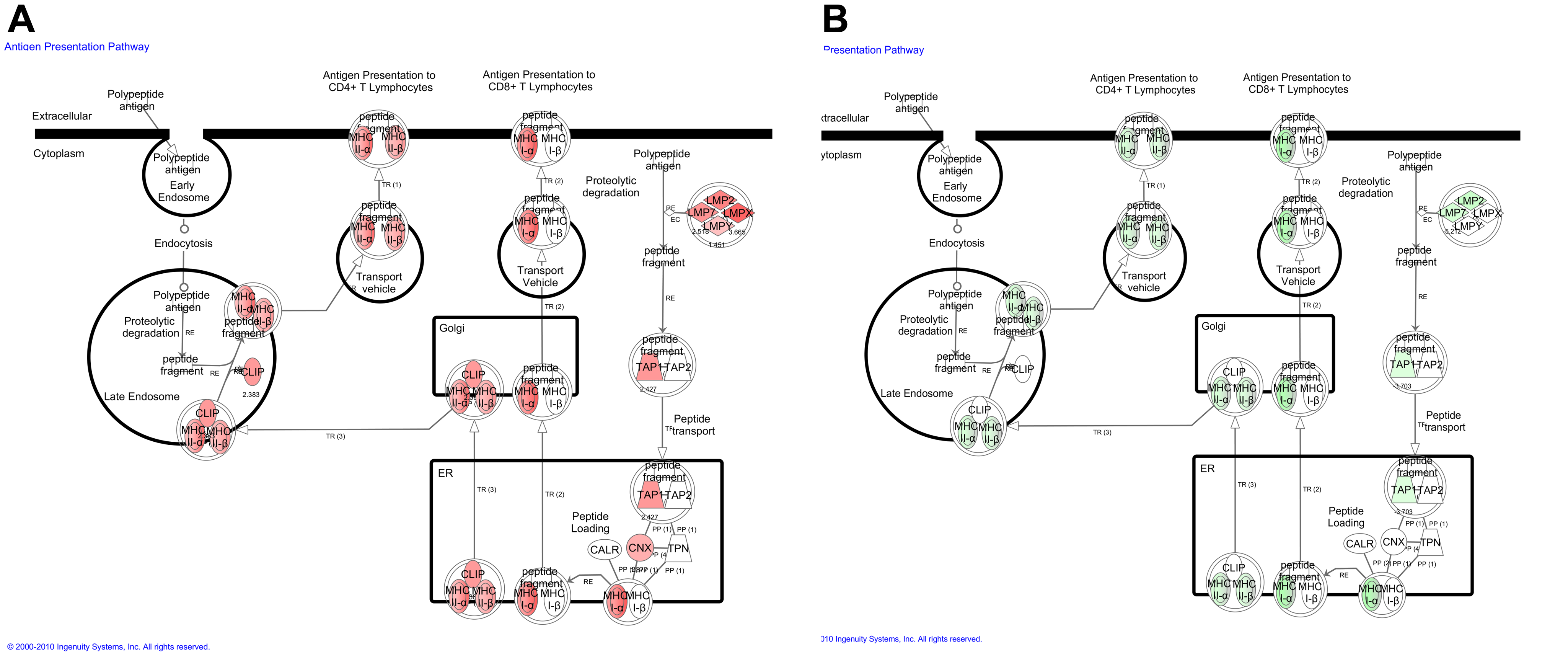

Supplement: Figure S4 — Comparison of the MHC antigen presentation pathway in early and late TB granulomas. Canonical pathways contained within the IPA algorithm were queried with the list of genes significantly up (red) or down (green) regulated in week 4 or week 13 lesions. Pathway illustrations are shown for week 4 (A) and week 13 (B) lesions. (1.40 MB TIF) [file pone.0012266.s017.tif]

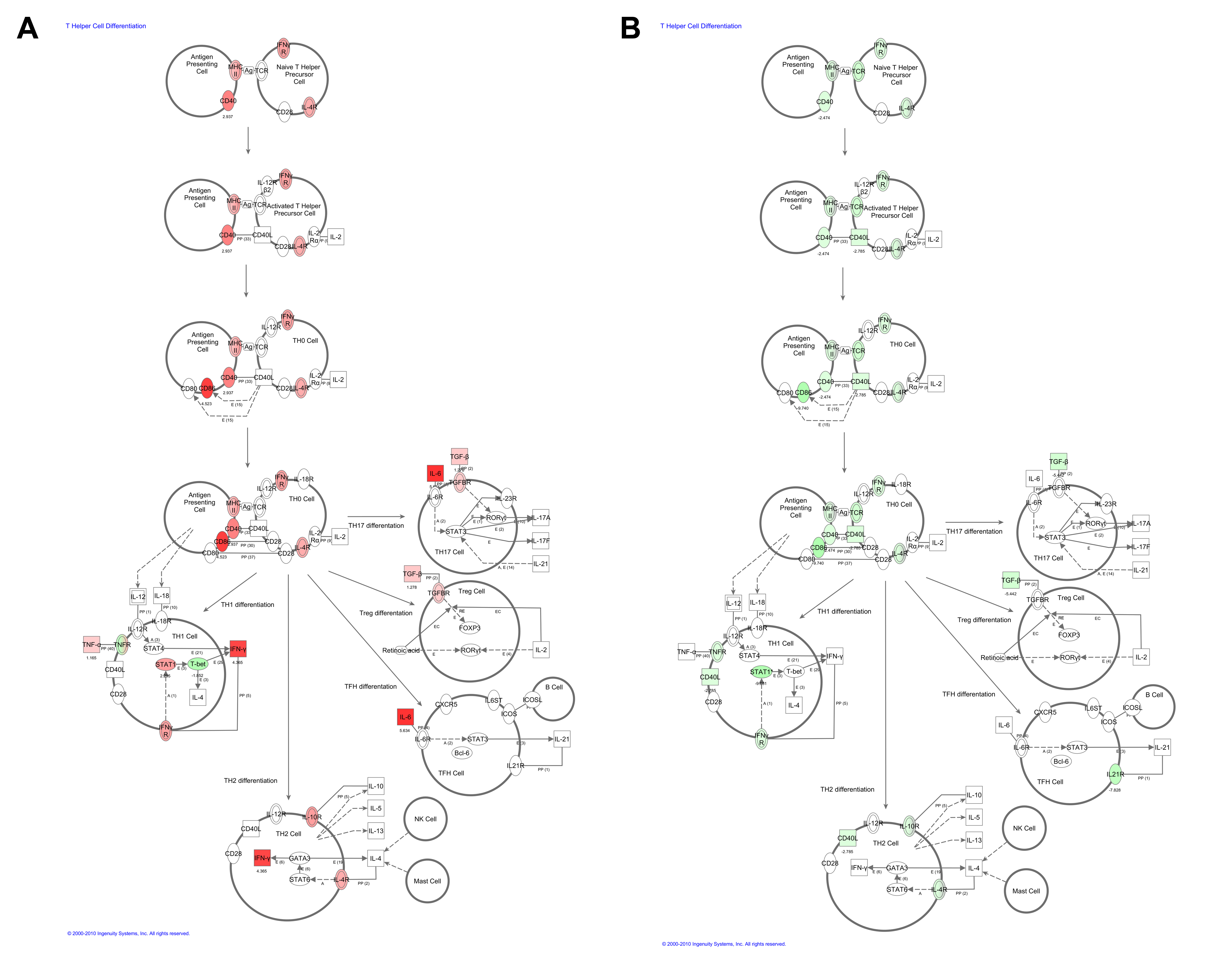

Supplement: Figure S5 — Comparison of the T-helper cell differentiation pathway in early and late TB granulomas. Canonical pathways contained within the IPA algorithm were queried with the list of genes significantly up (red) or down (green) regulated in week 4 or week 13 lesions. Pathway illustrations are shown for week 4 (A) and week 13 (B) lesions. (1.46 MB TIF) [file pone.0012266.s018.tif]

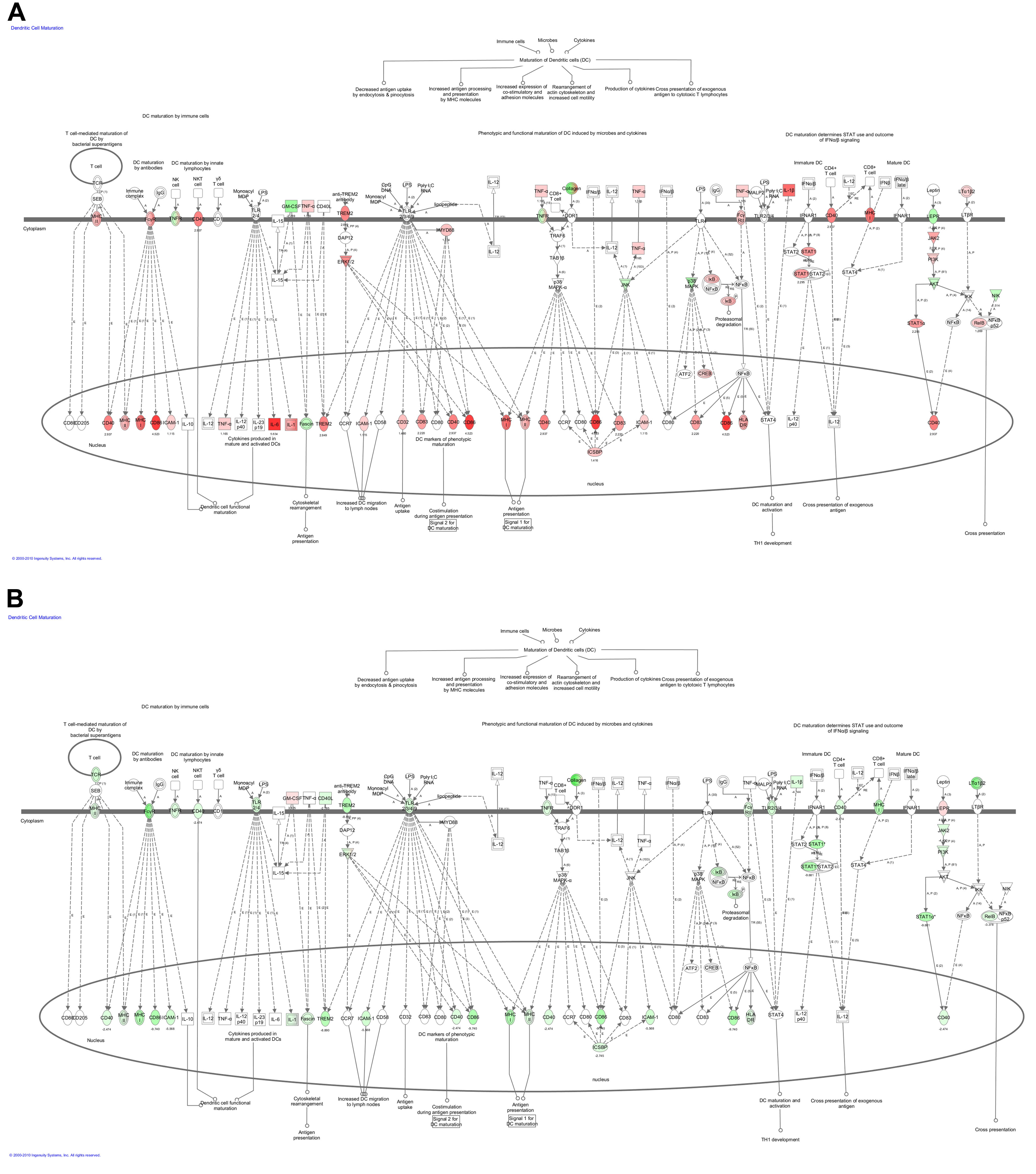

Supplement: Figure S6 — Comparison of the DC maturation pathway in early and late TB granulomas. Canonical pathways contained within the IPA algorithm were queried with the list of genes significantly up (red) or down (green) regulated in week 4 or week 13 lesions. Pathway illustrations are shown for week 4 (A) and week 13 (B) lesions. (3.22 MB TIF) [file pone.0012266.s019.tif]
